# Supplementary material for: Extensive monolayer formation after transplantation depends on an RPE subpopulation derived from human iPSCs
Source: iScience. 2026 Oct 7;29(11):117750. doi: 10.1016/j.isci.2026.117750 (PMC13676745; doi:10.1016/j.isci.2026.117750)
Supplement: Supplementary file 2 [file mmc3.pdf]

Date:2024-06-14

Operator: Sebastian Knoebel

Version:2.13.0+54.g95abf0f4

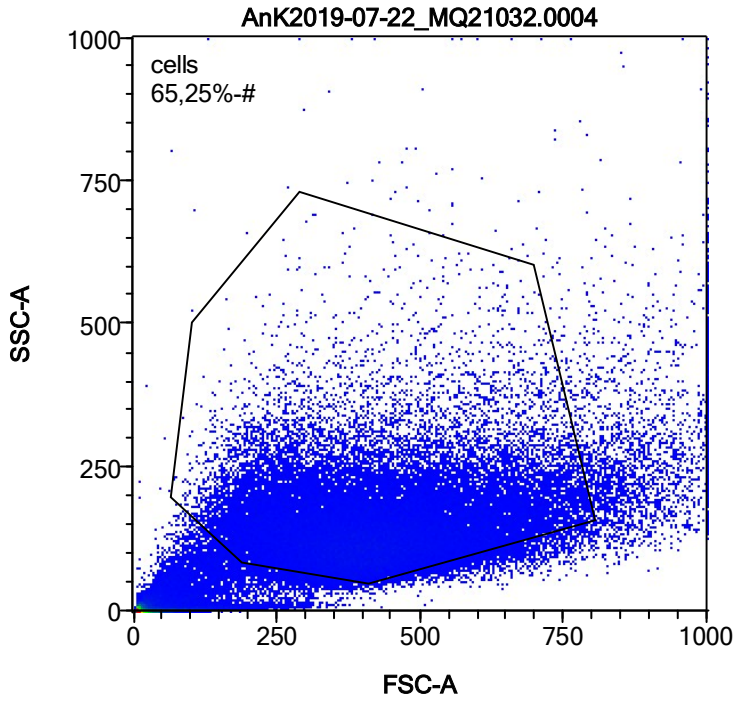

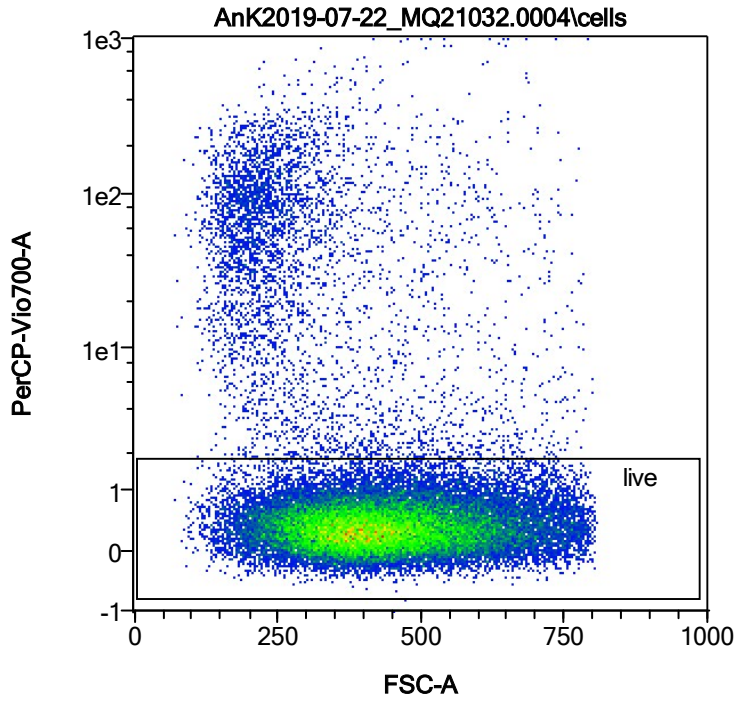

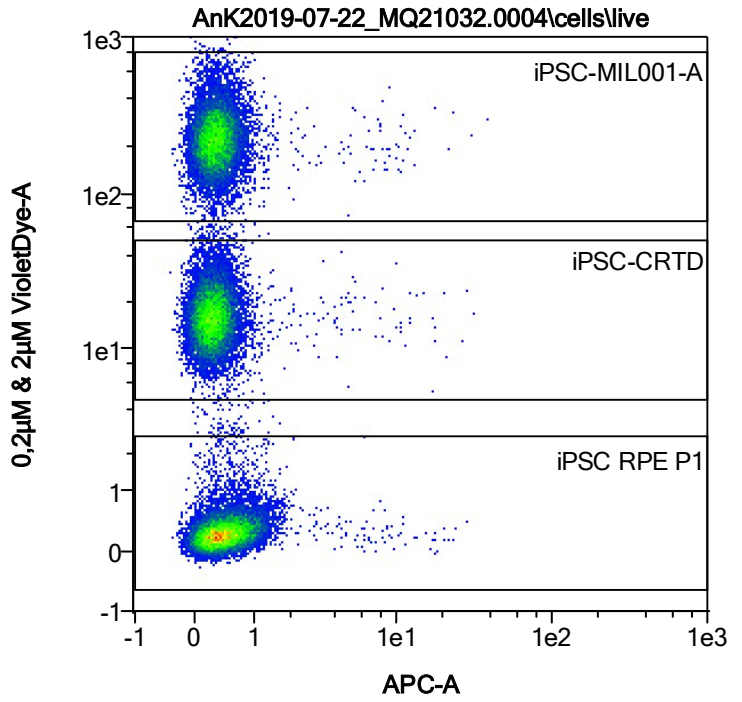

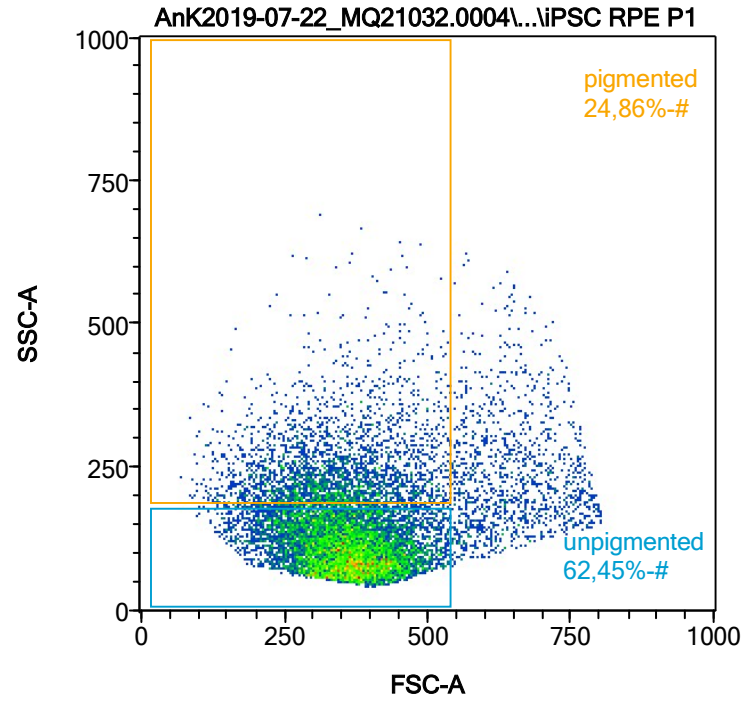

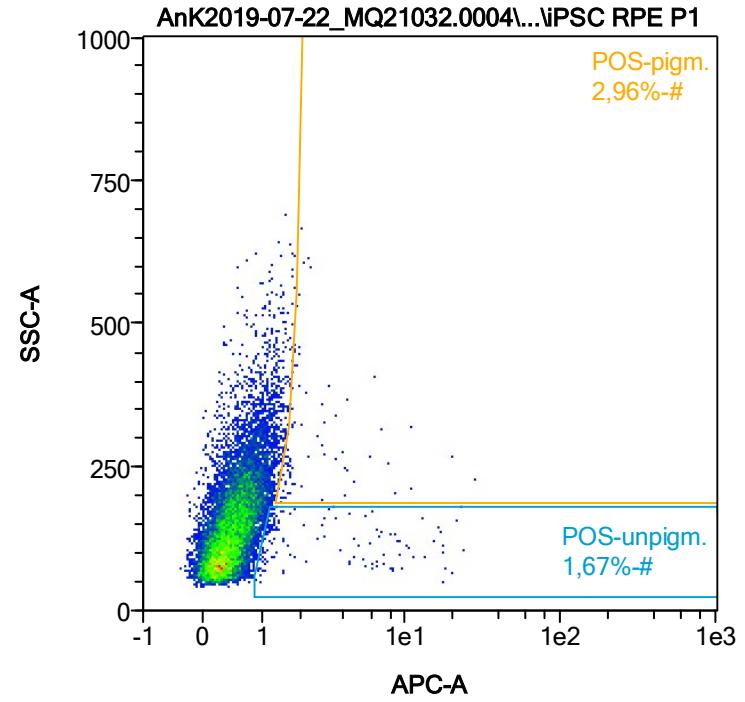

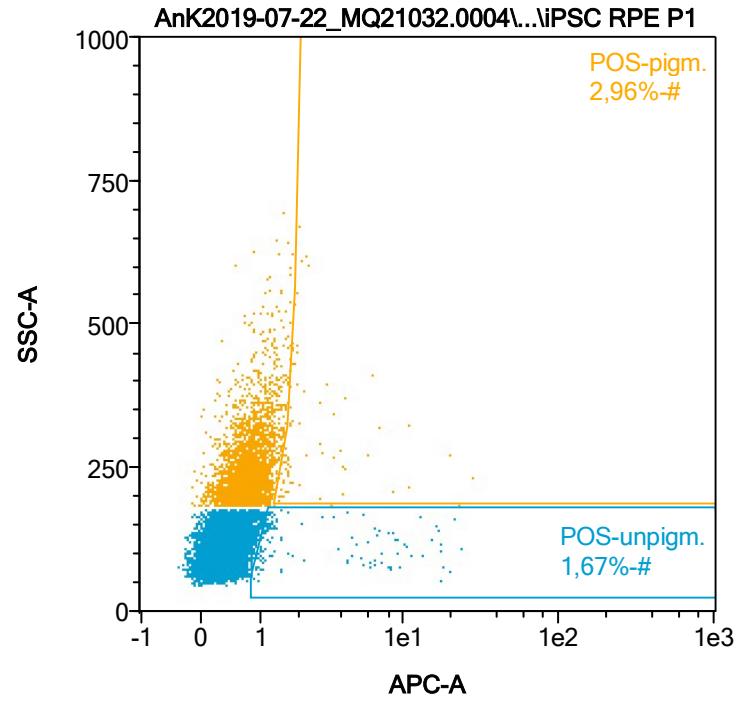

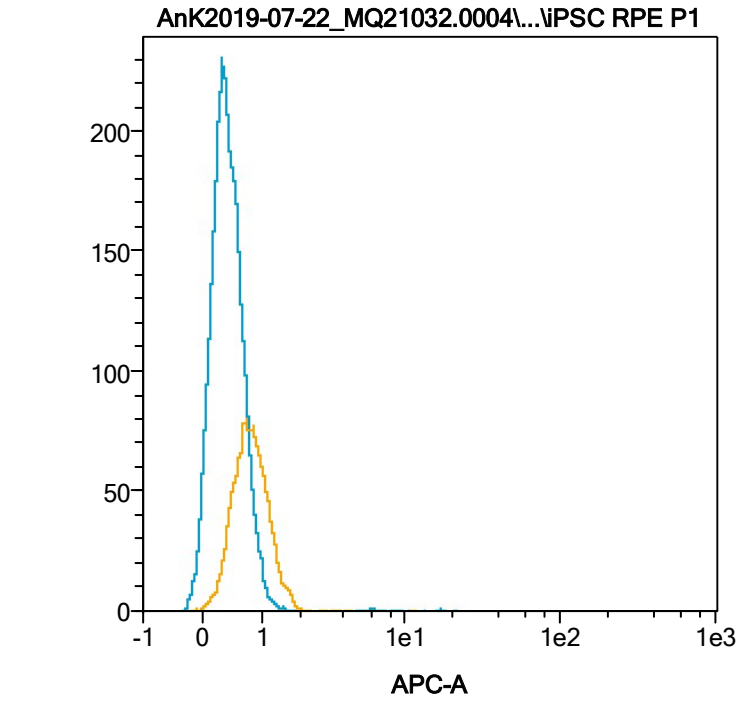

Name	%-#	APC-A Mean	APC-A Median
POS-unpigm.	1,67	4,39	1,34
POS-pigm.	2,96	3,20	1,78

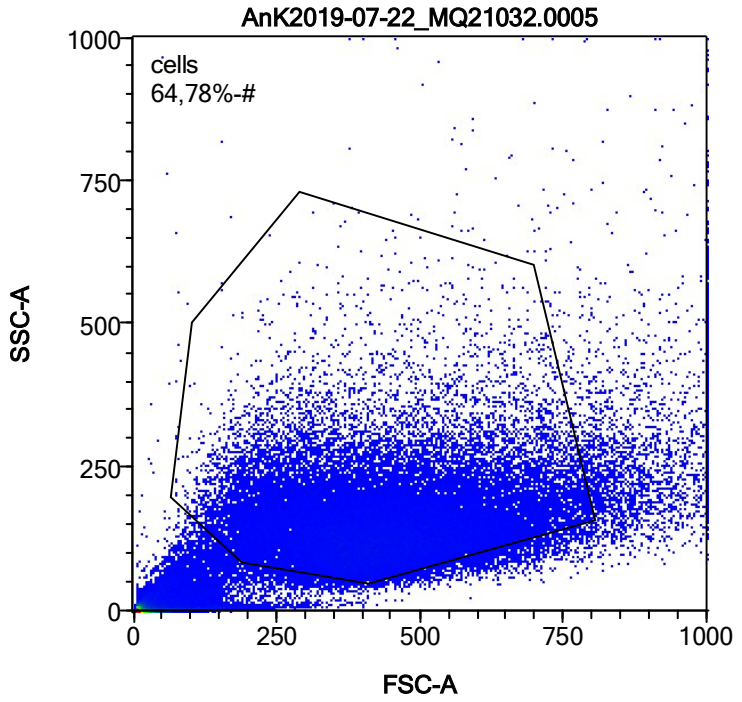

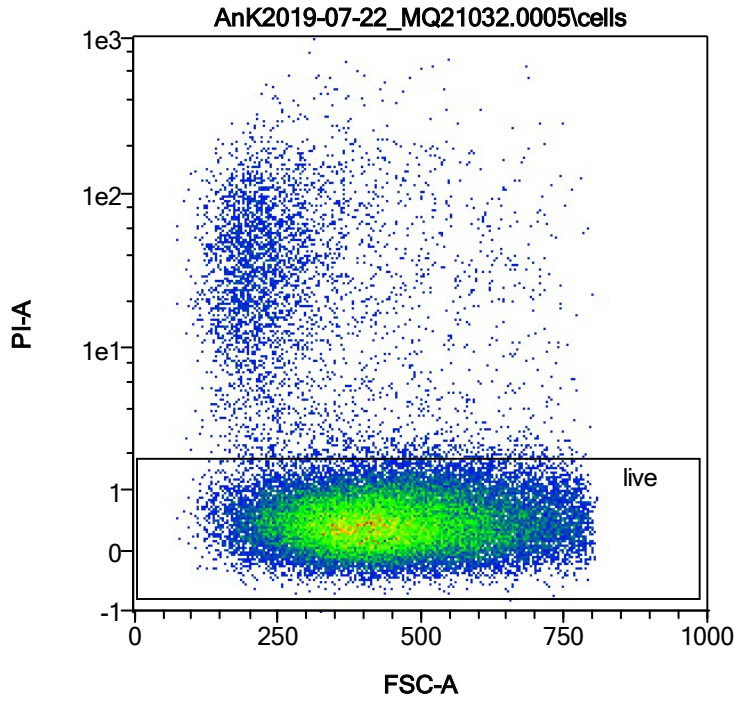

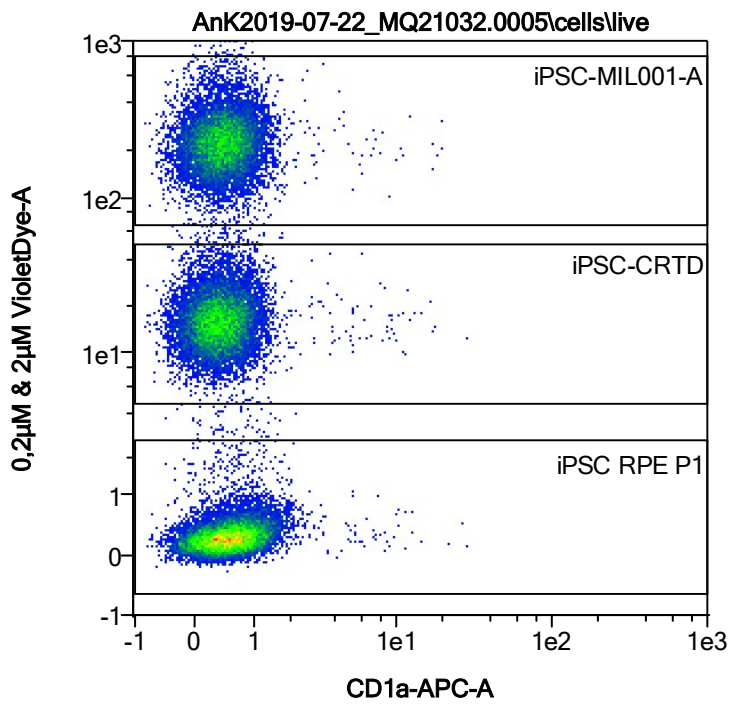

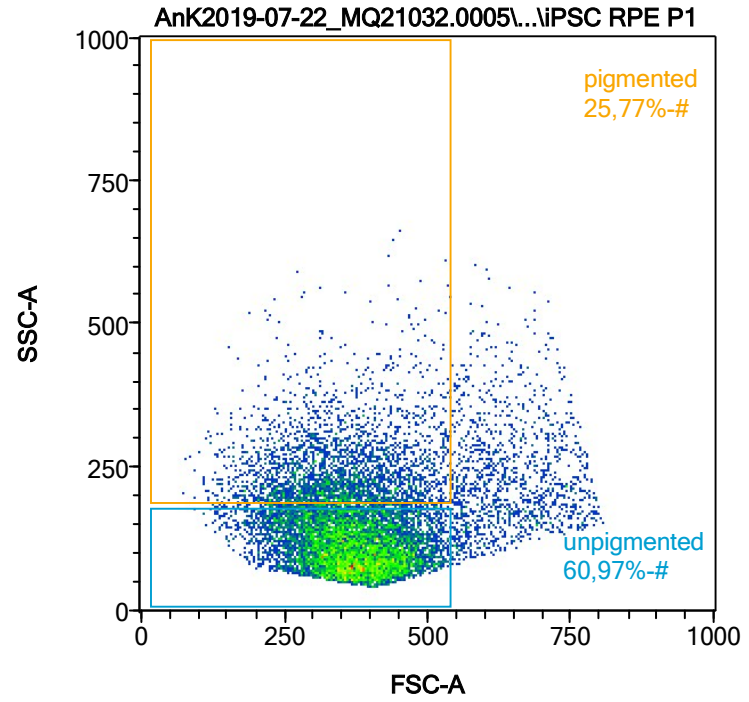

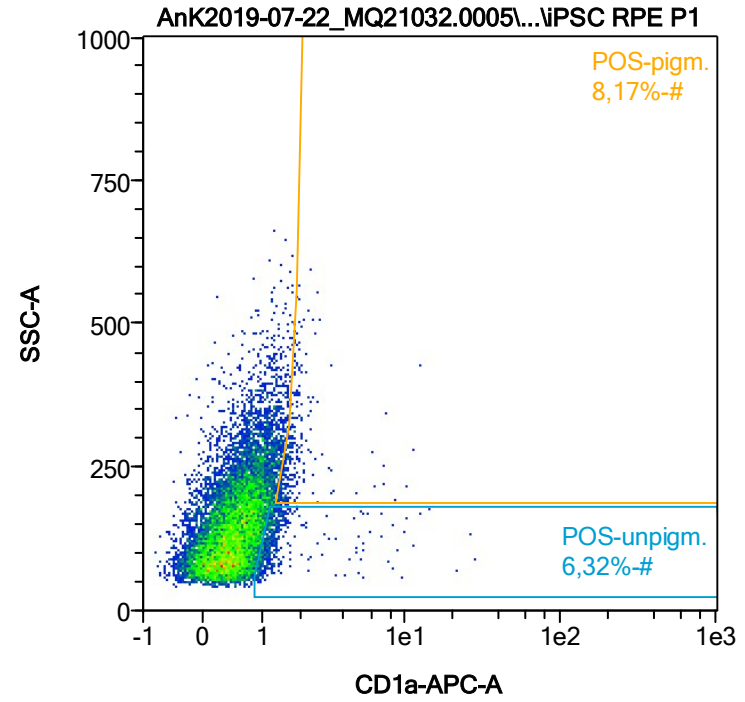

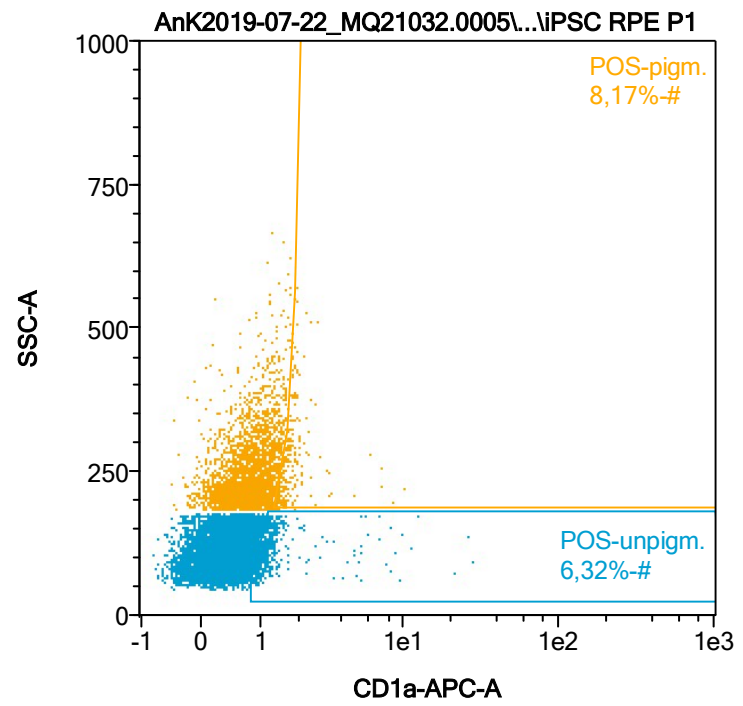

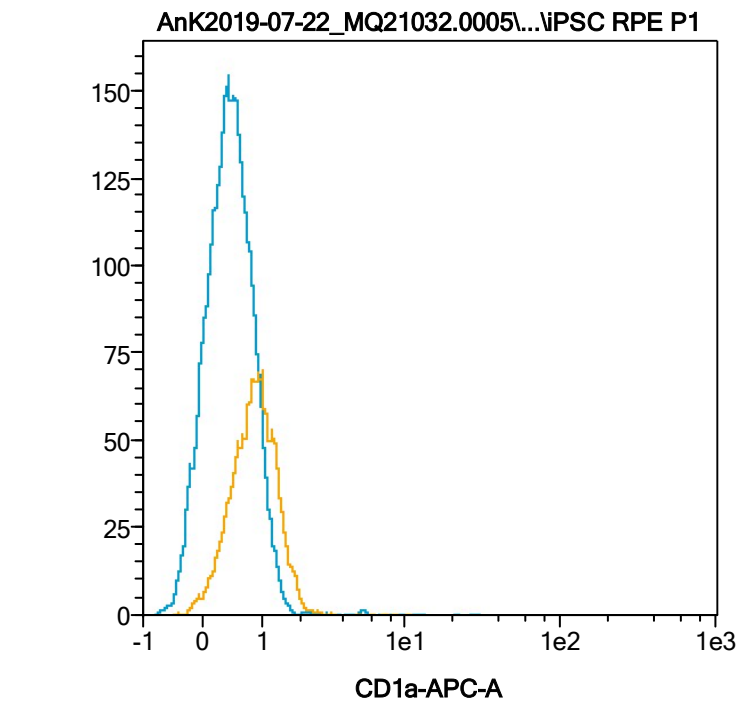

Name	%-#	CD1a-APC-A Mean	CD1a-APC-A Median
POS-unpigm.	6,32	1,64	1,17
POS-pigm.	8,17	1,87	1,68

Date:2024-06-14
Operator: Sebastian Knoebel
Version:2.13.0+54.g95abf0f4

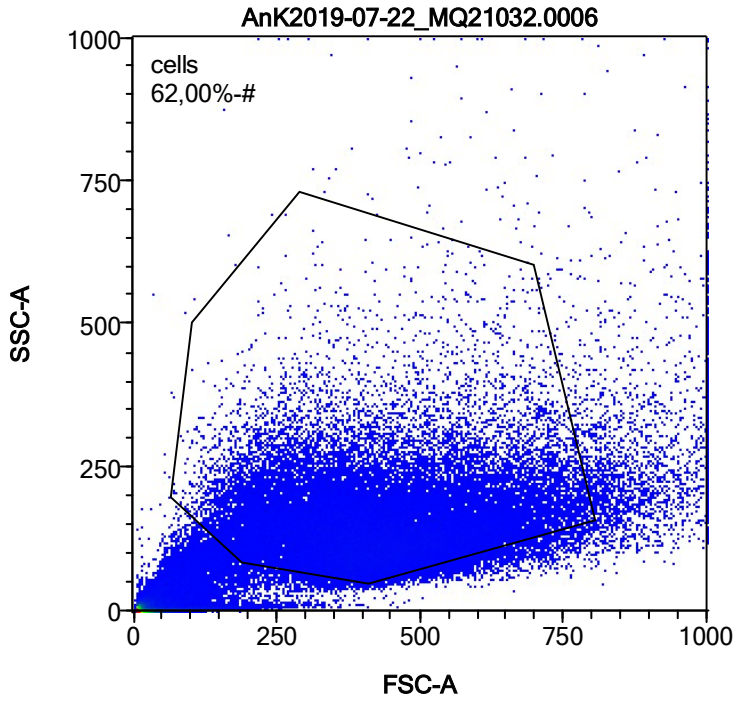

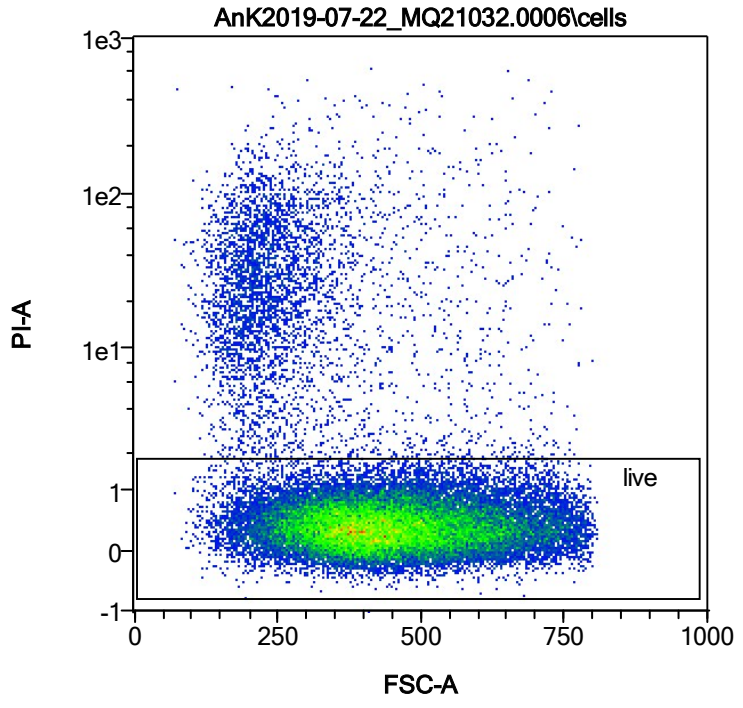

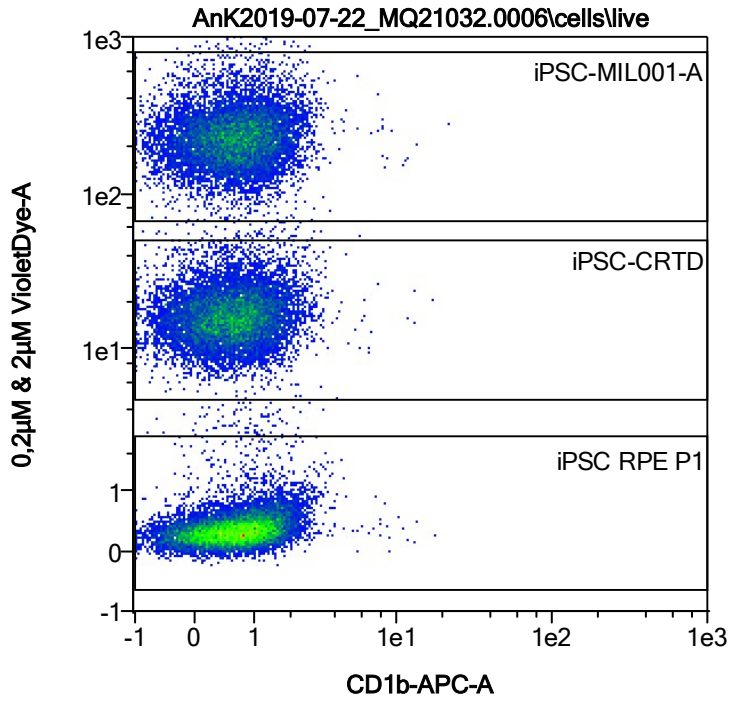

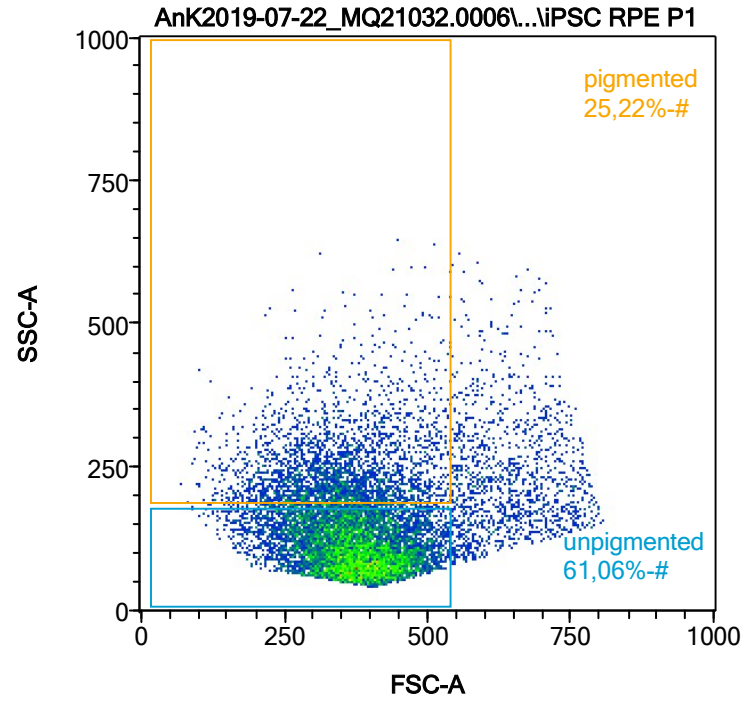

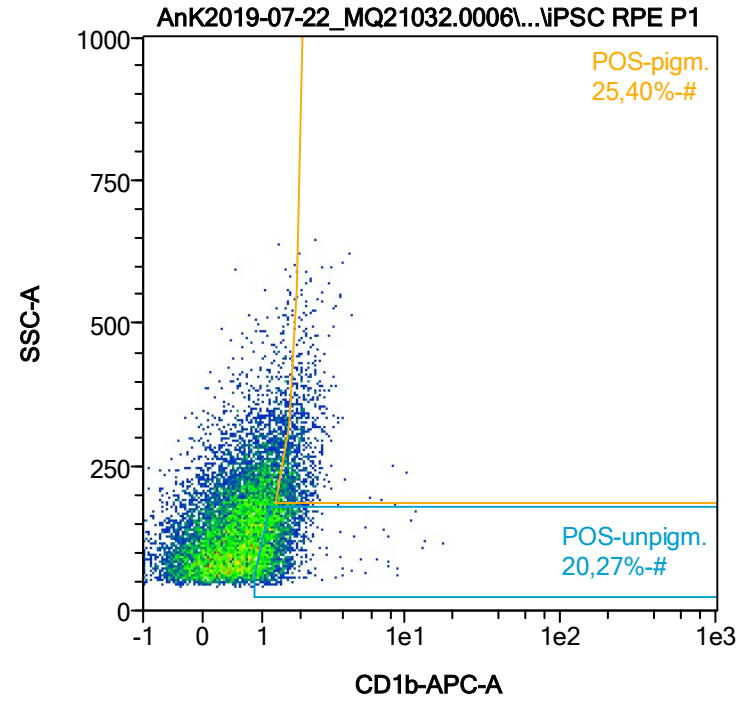

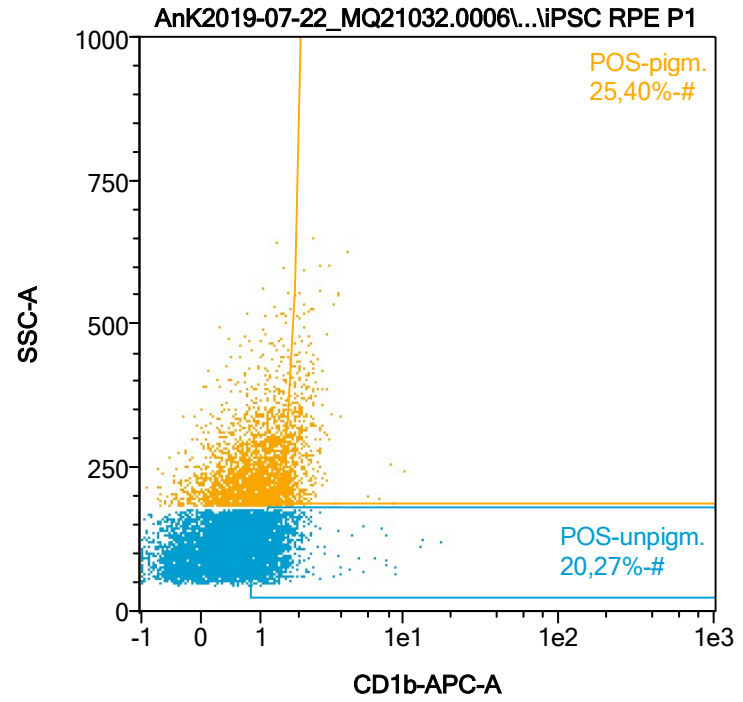

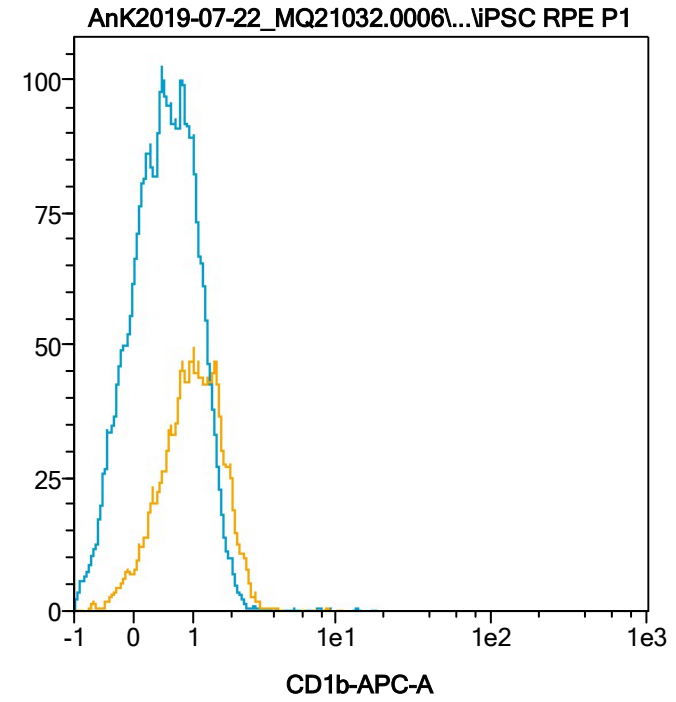

Name	%-#	CD1b-APC-A Mean	CD1b-APC-A Median
POS-unpigm.	20,27	1,40	1,25
POS-pigm.	25,40	1,92	1,78

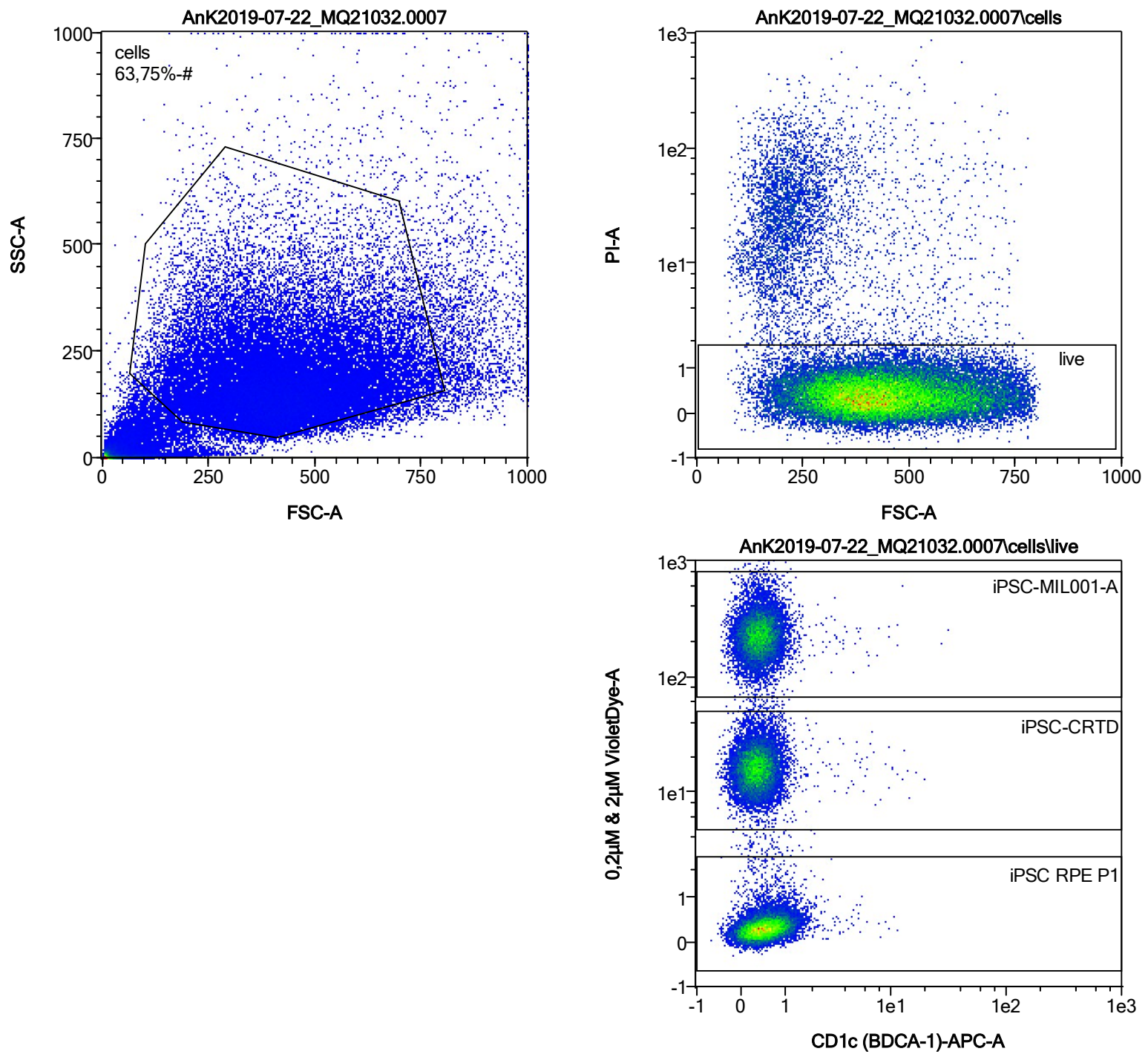

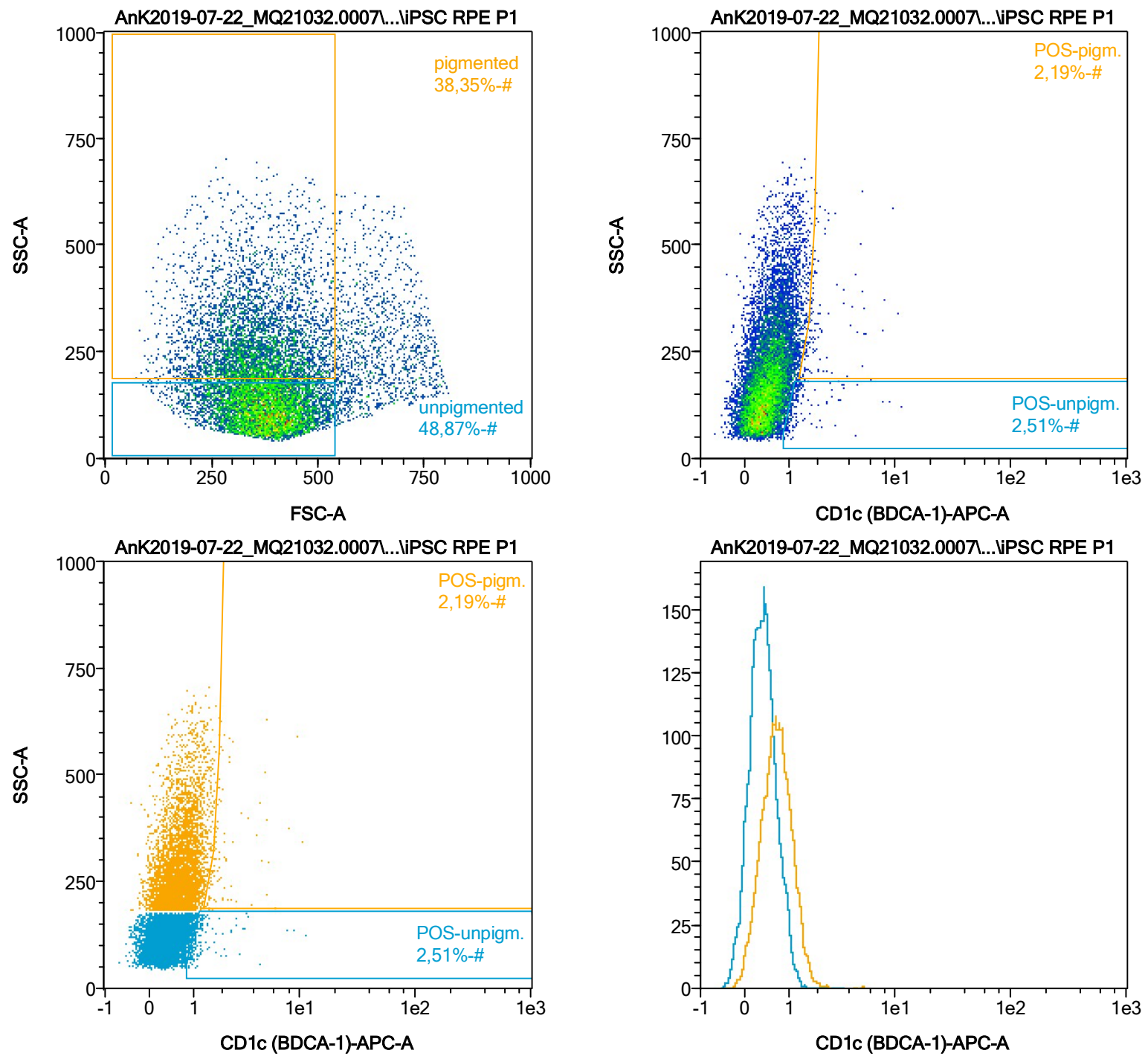

Name	%-#	CD1c (BDCA-1)-APC-A Mean	CD1c (BDCA-1)-APC-A Median
POS-unpigm.	2,51	1,46	1,12
POS-pigm.	2,19	2,37	1,83

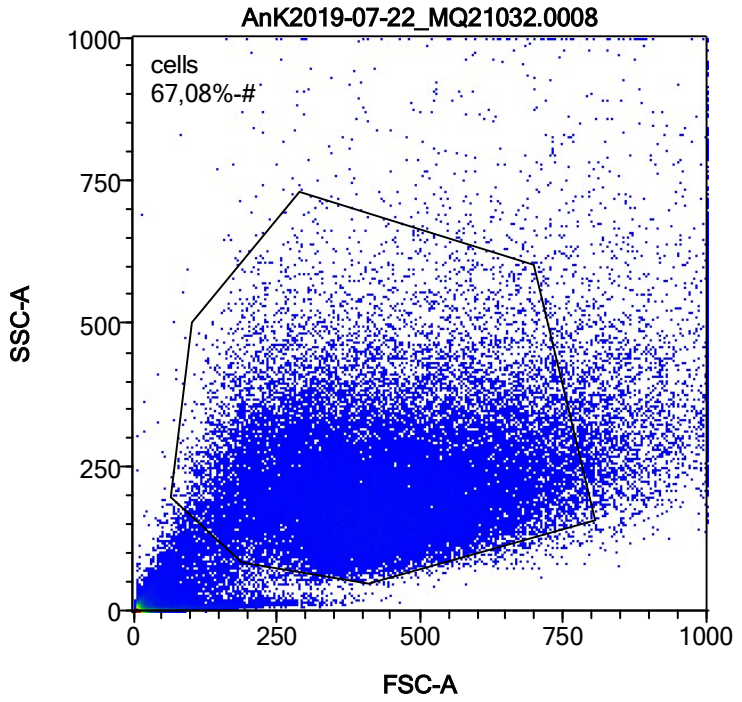

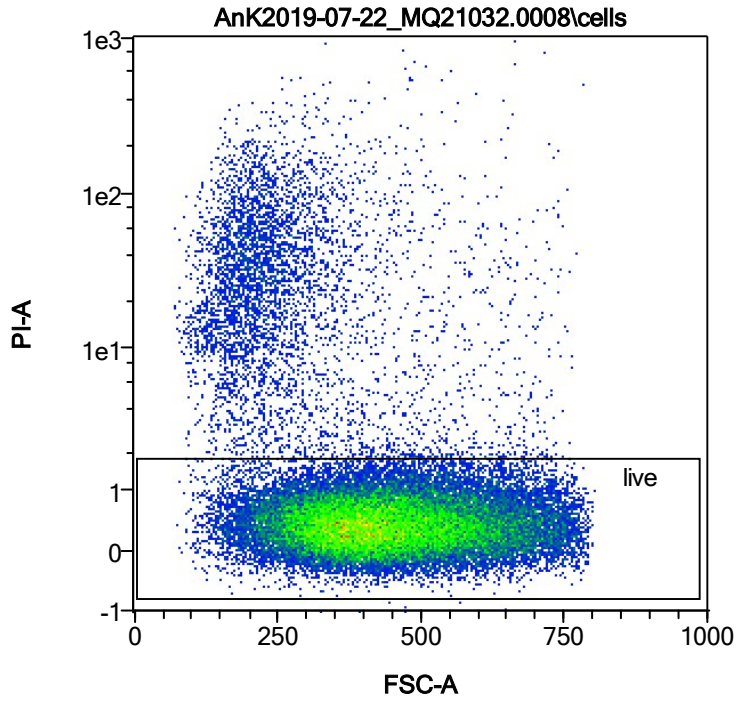

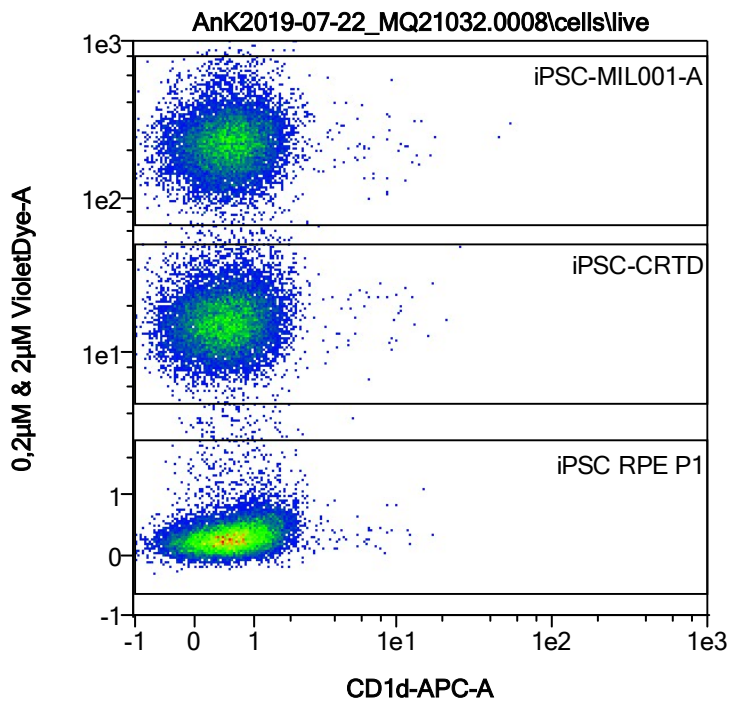

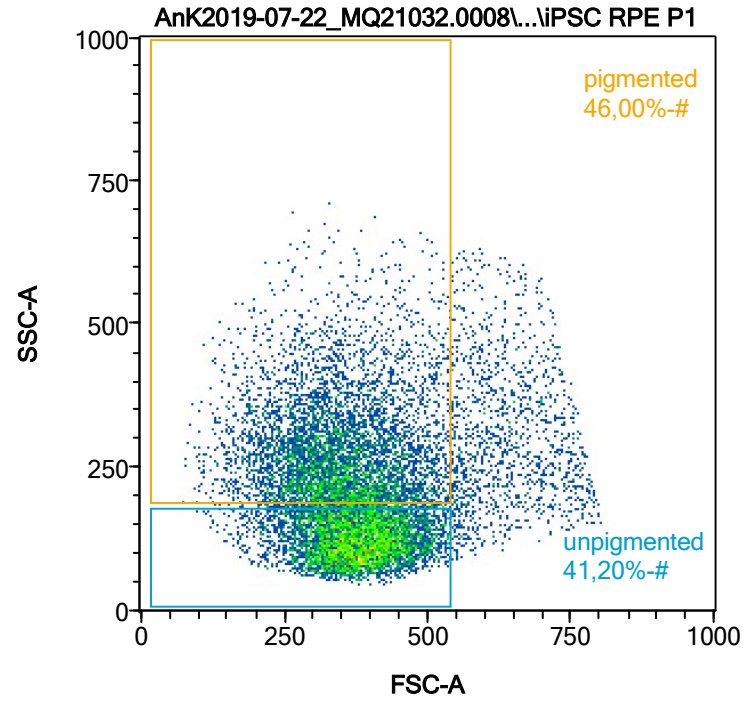

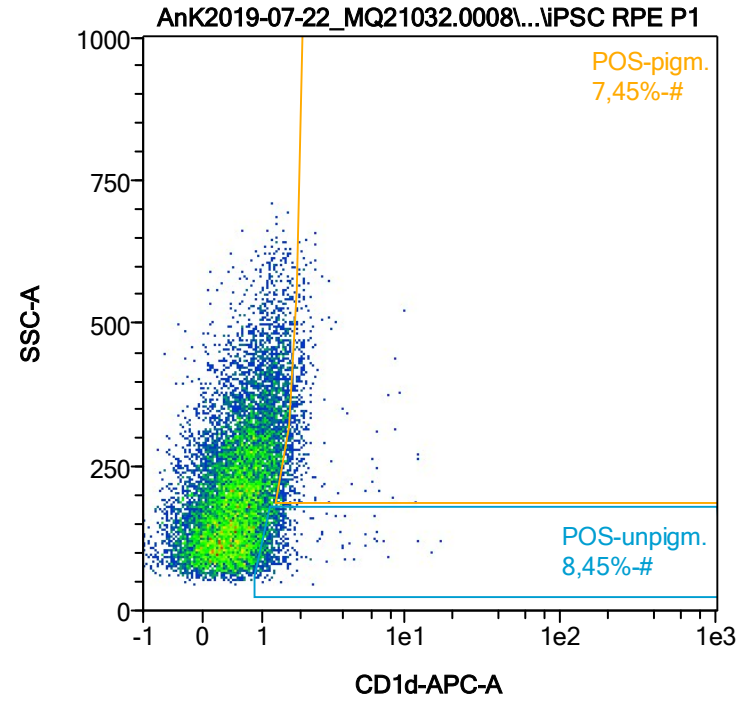

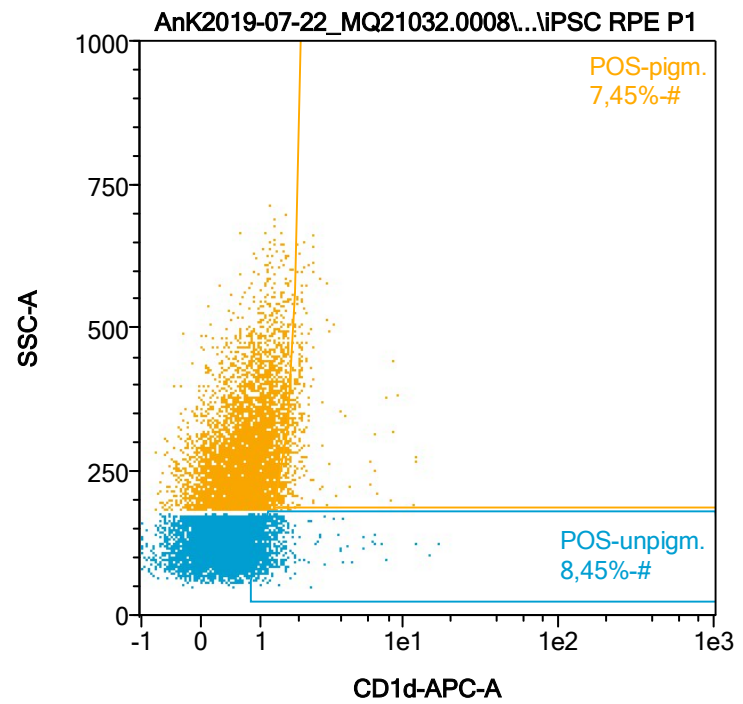

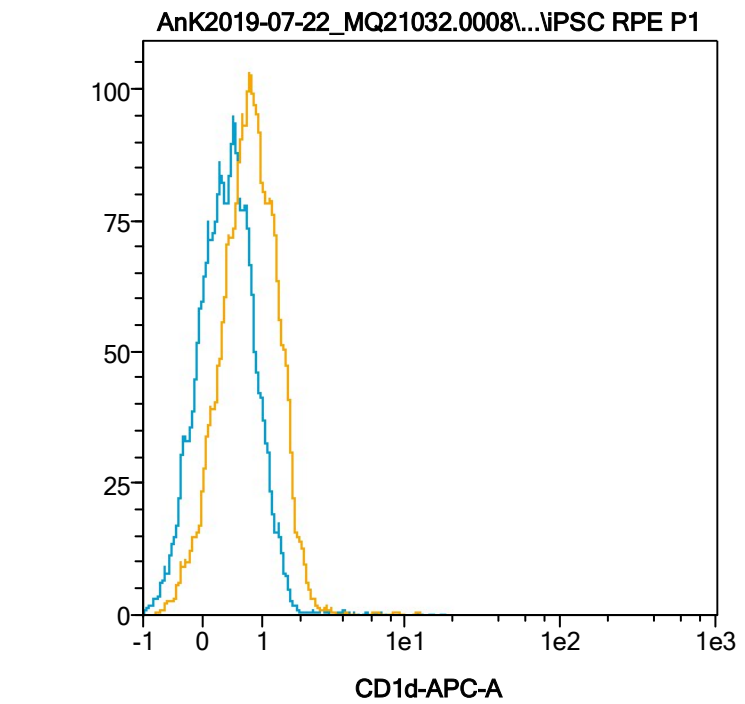

Name	%-#	CD1d-APC-A Mean	CD1d-APC-A Median
POS-unpigm.	8,45	1,52	1,21
POS-pigm.	7,45	2,06	1,79

Date:2024-06-14

Operator: Sebastian Knoebel

Version:2.13.0+54.g95abf0f4

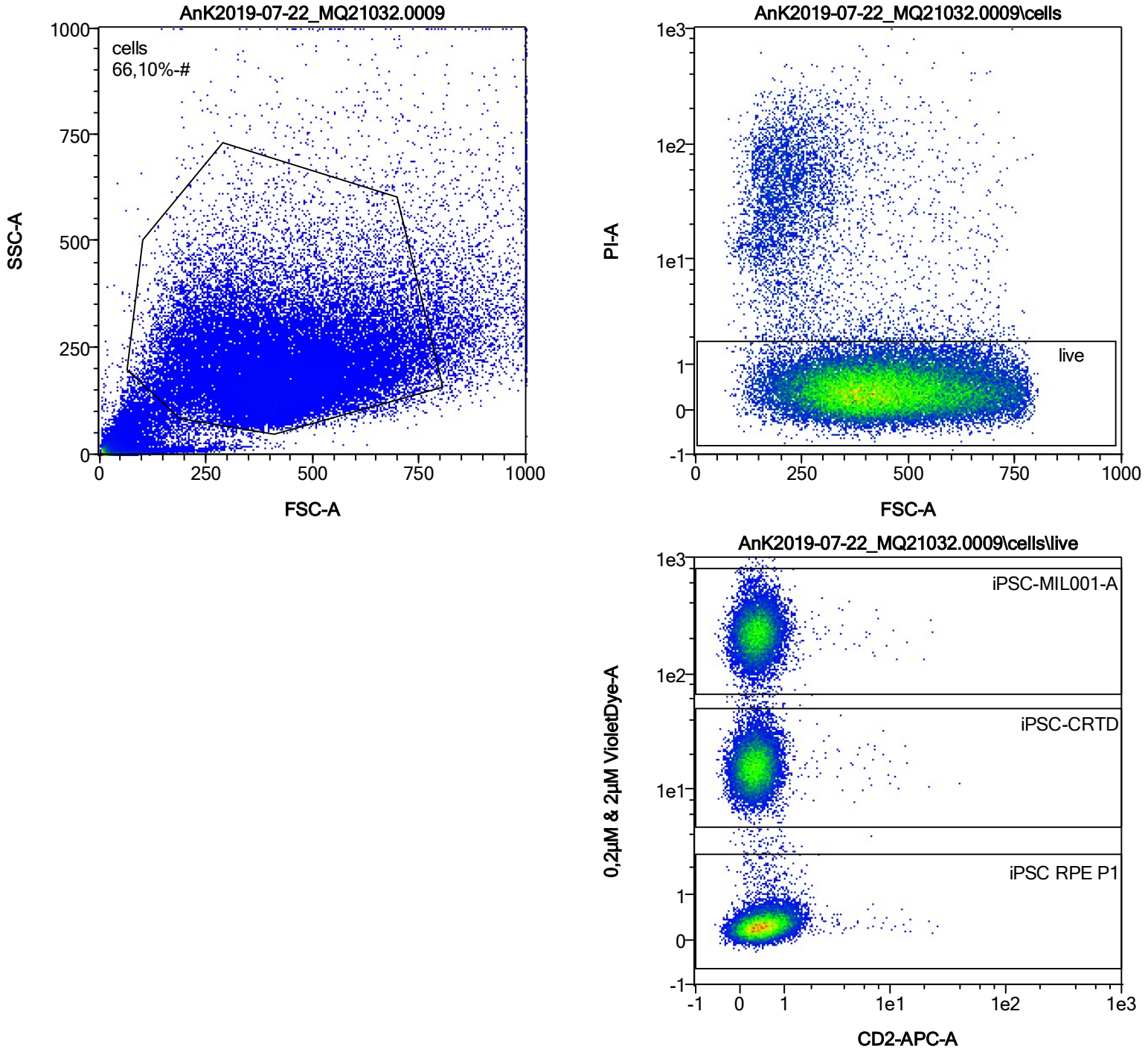

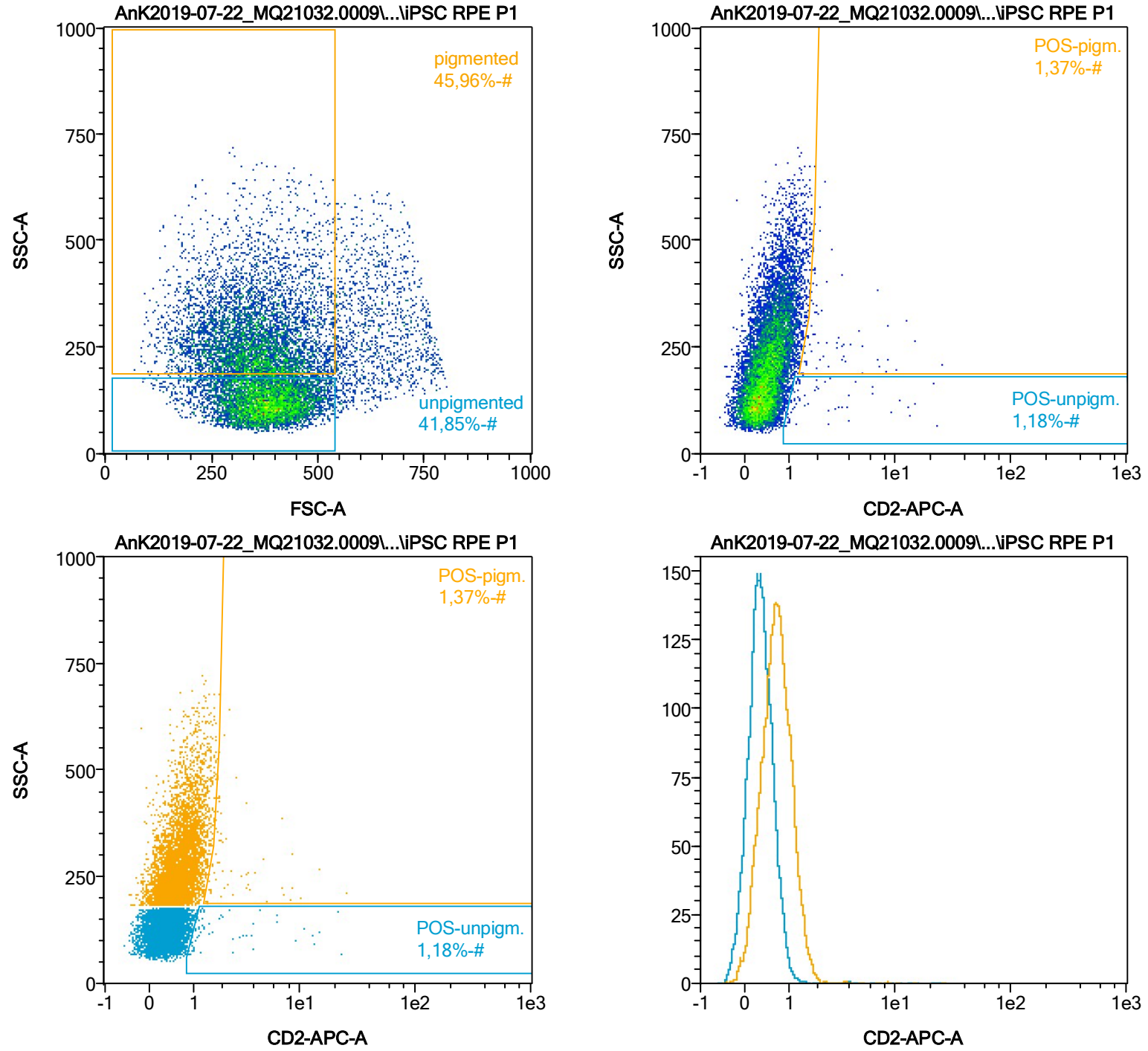

Name	%-#	CD2-APC-A Mean	CD2-APC-A Median
POS-unpigm.	1,18	3,33	1,28
POS-pigm.	1,37	3,10	1,91

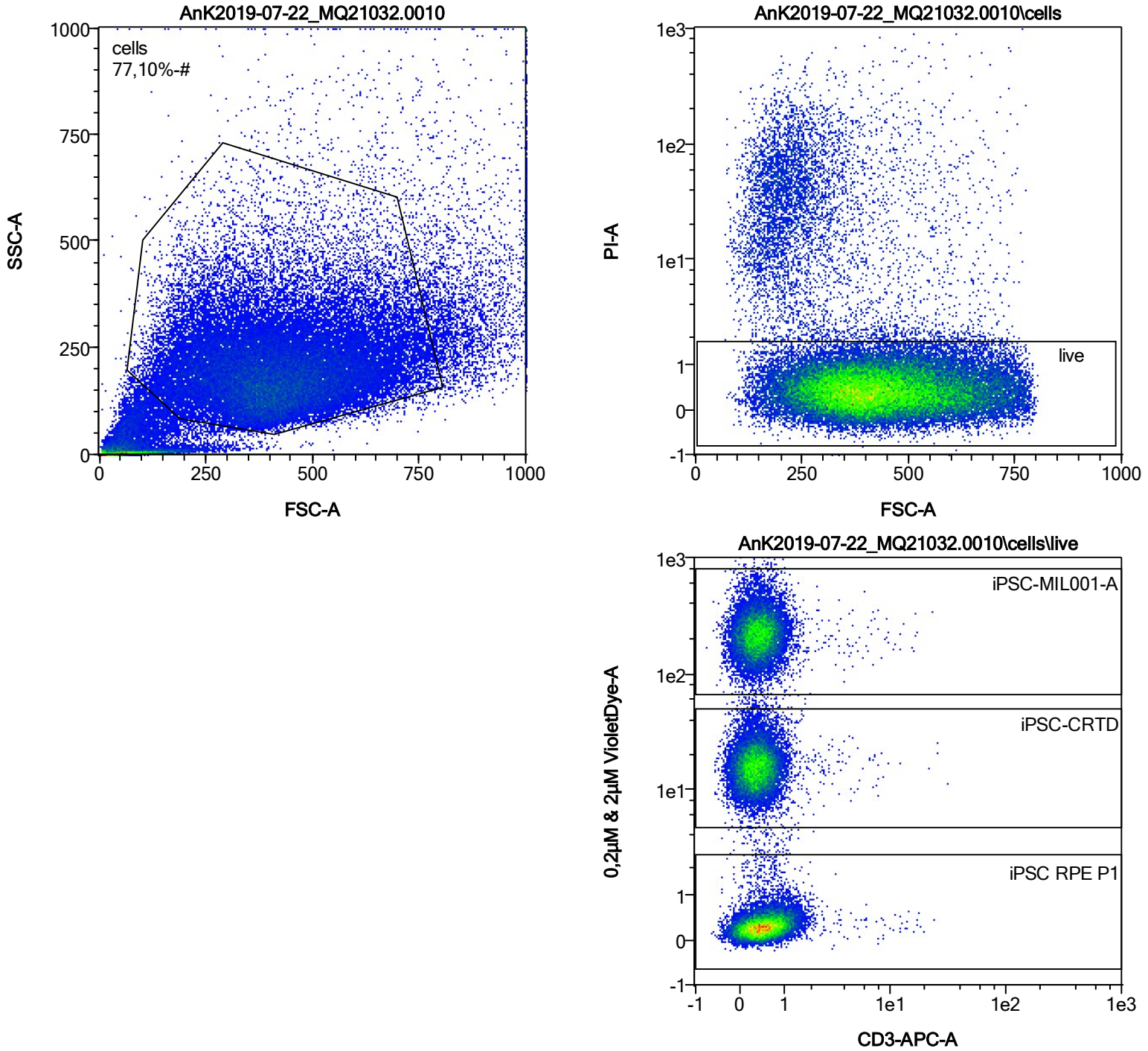

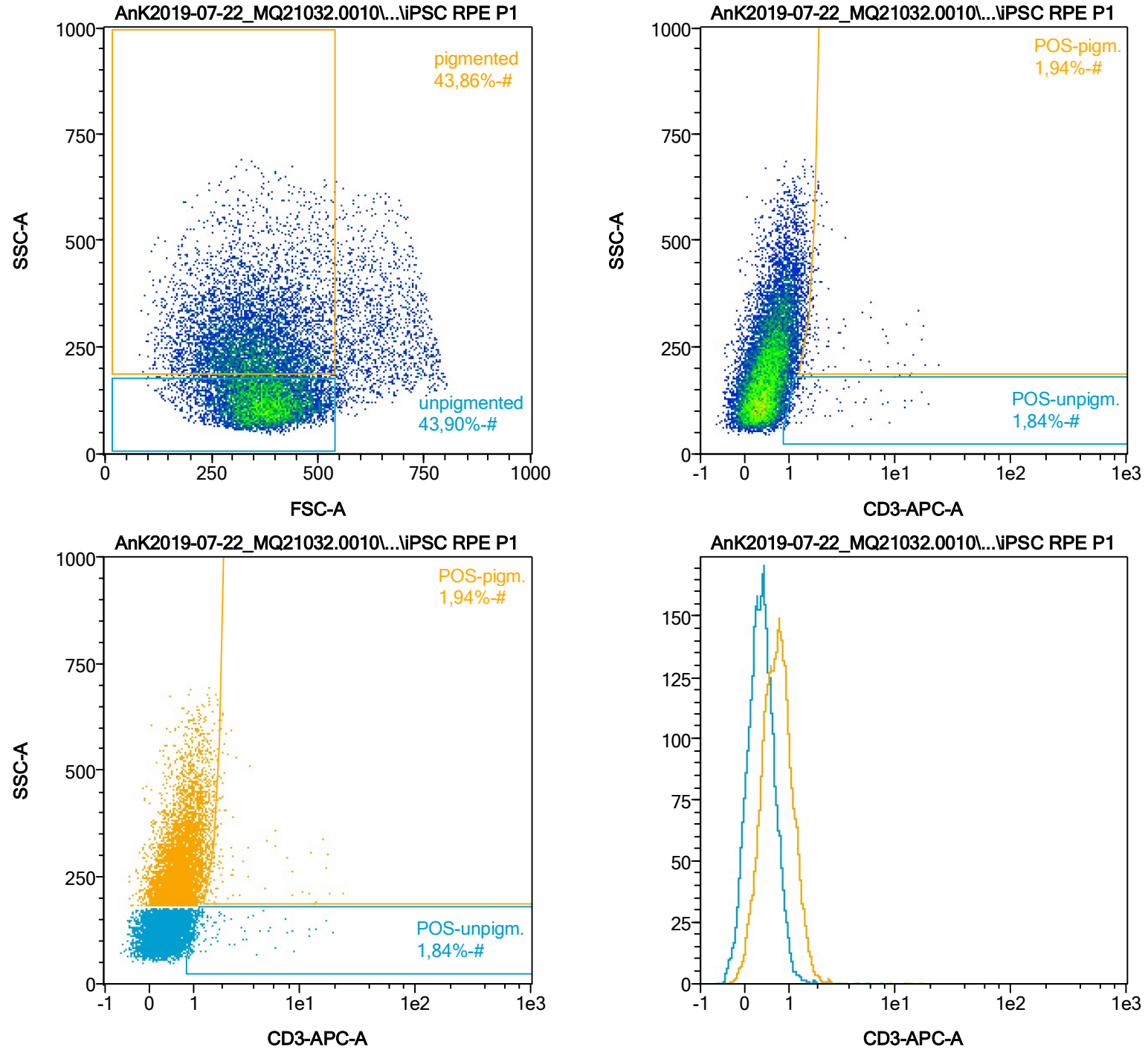

Name	%-#	CD3-APC-A Mean	CD3-APC-A Median
POS-unpigm.	1,84	2,44	1,27
POS-pigm.	1,94	3,33	1,88

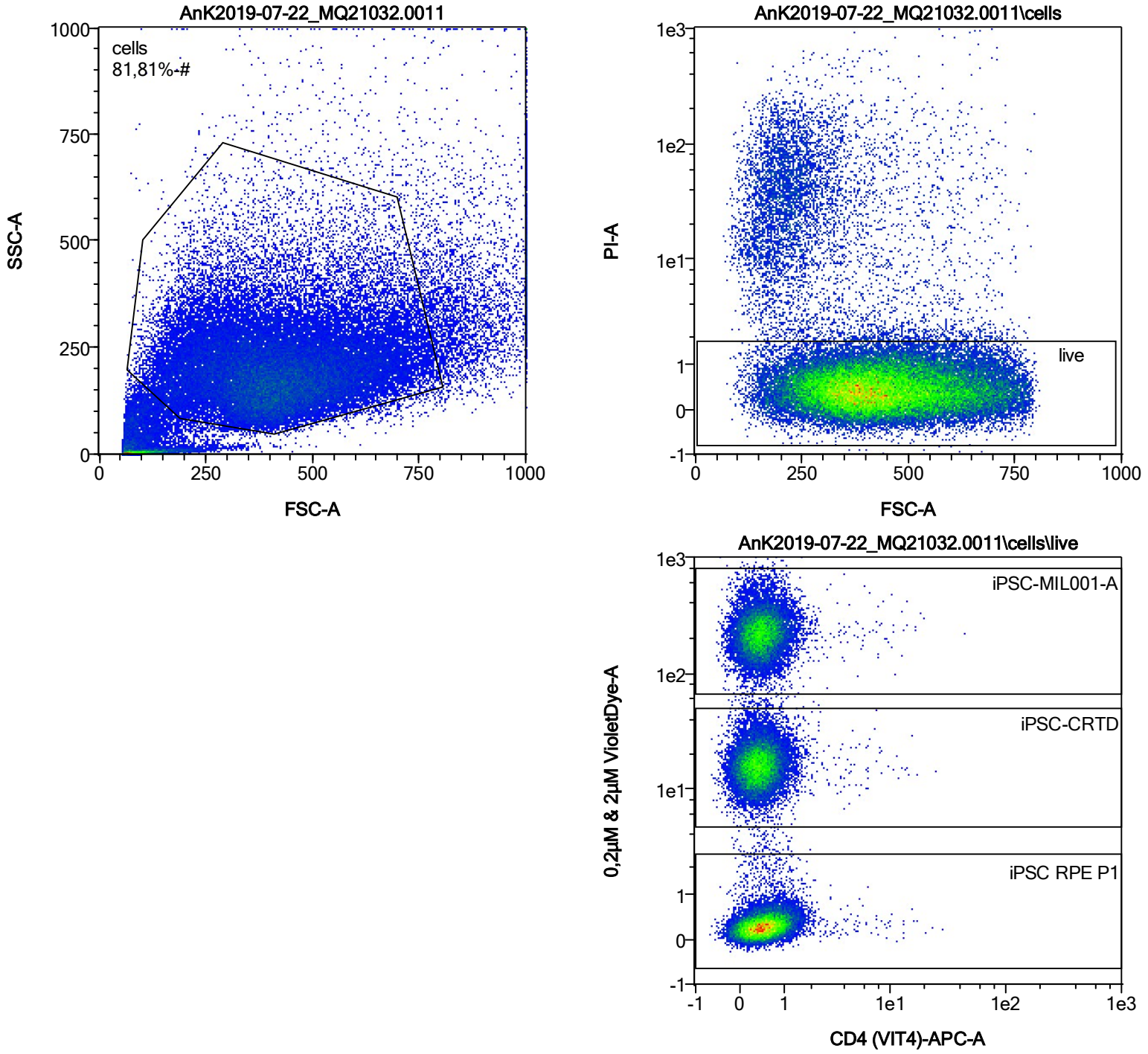

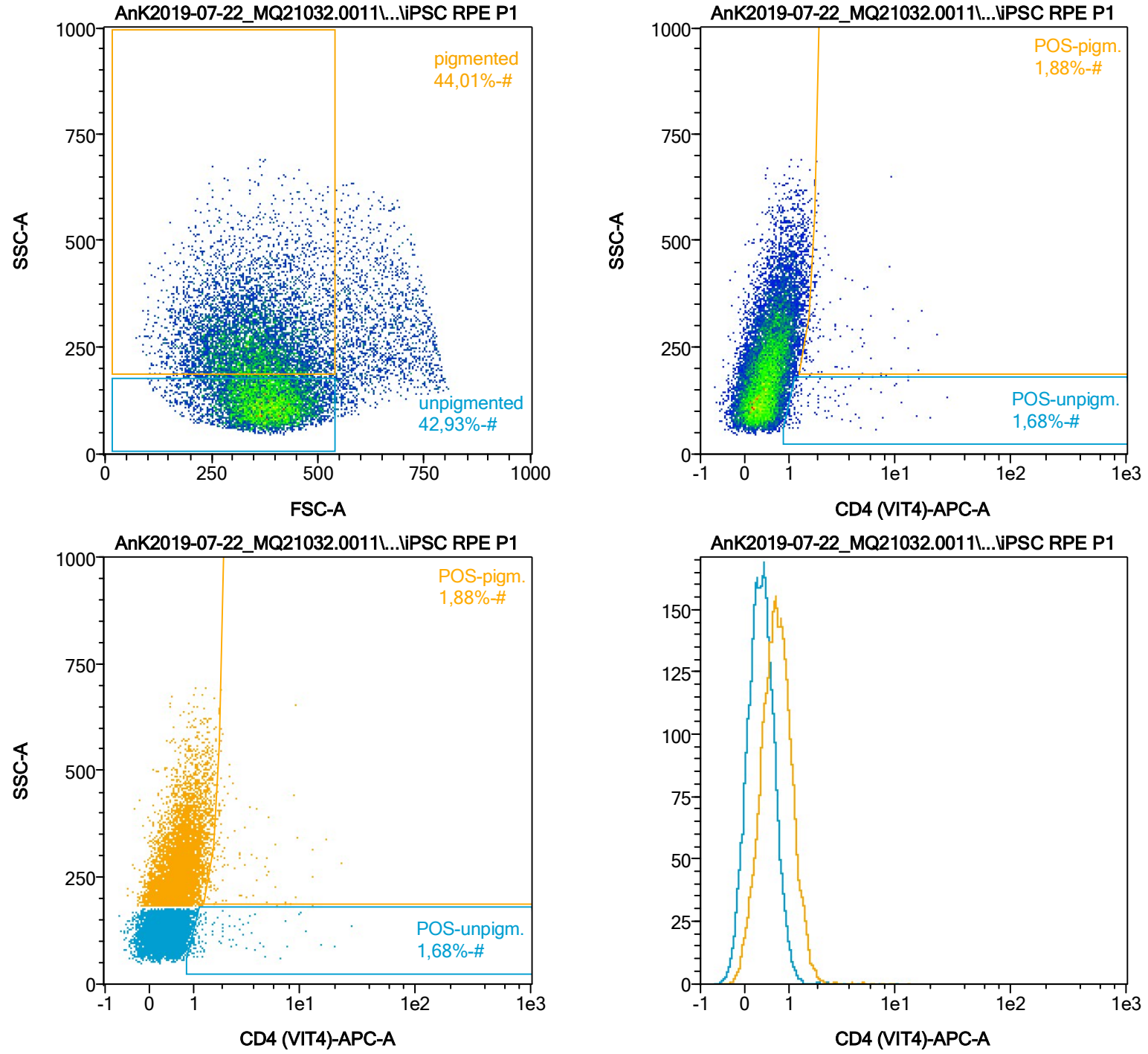

Name	%-#	CD4 (VIT4)-APC-A Mean	CD4 (VIT4)-APC-A Median
POS-unpigm.	1,68	2,59	1,25
POS-pigm.	1,88	3,25	1,97

Date:2024-06-14

Operator: Sebastian Knoebel

Version:2.13.0+54.g95abf0f4

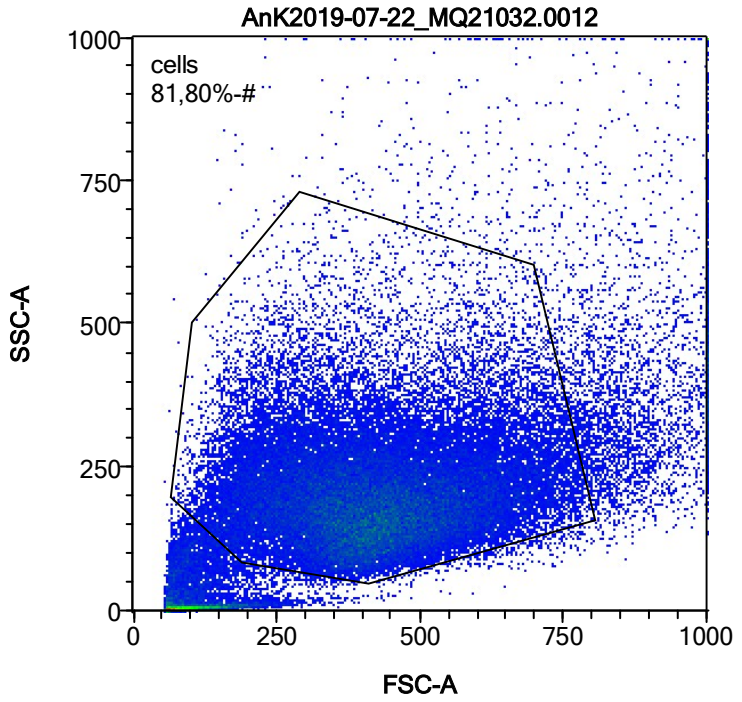

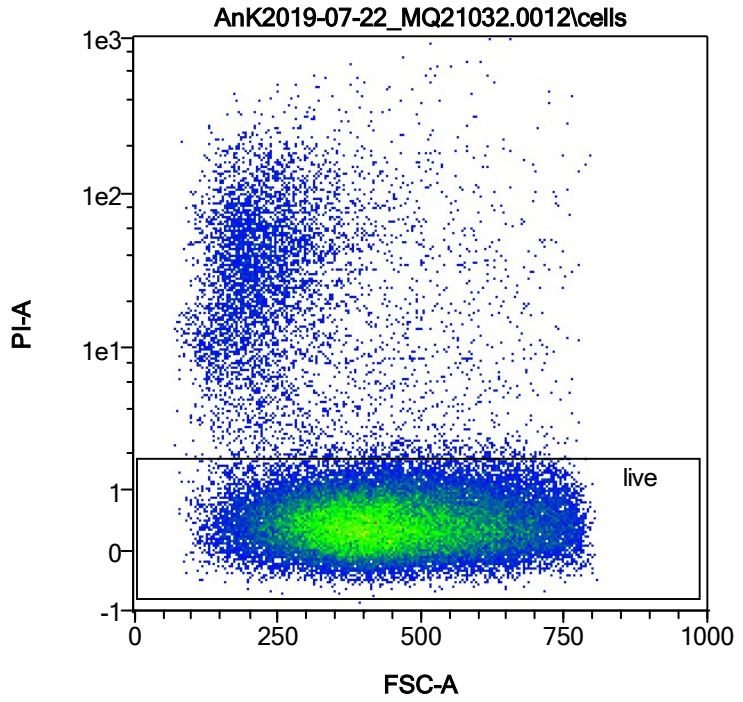

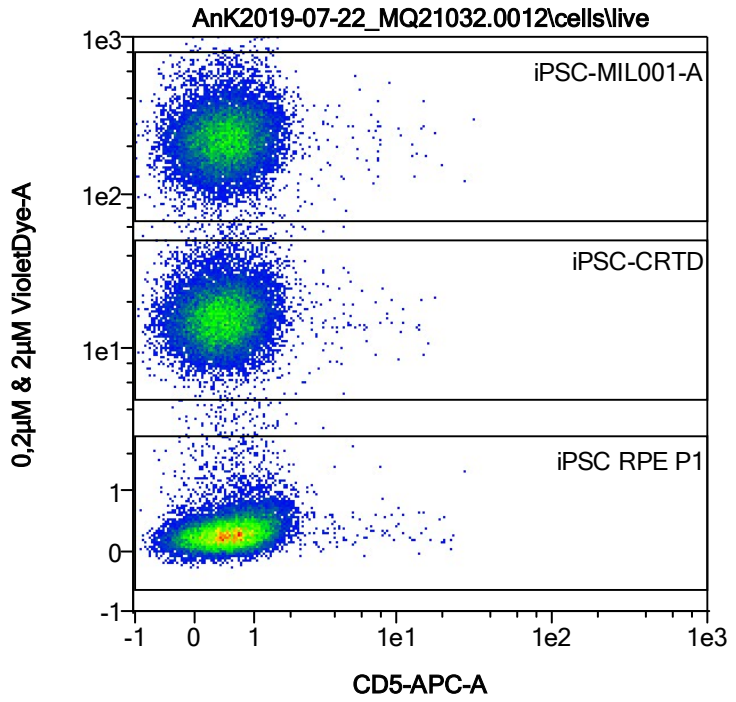

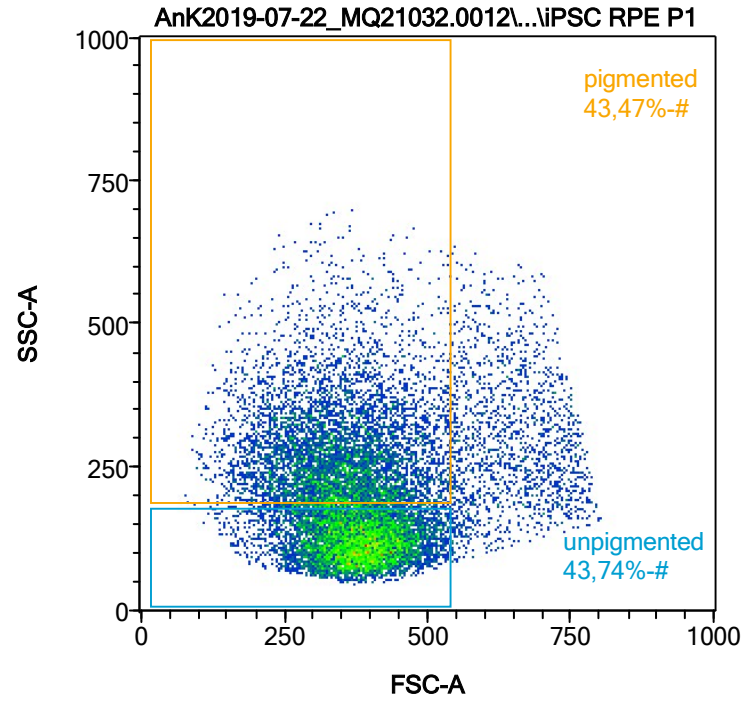

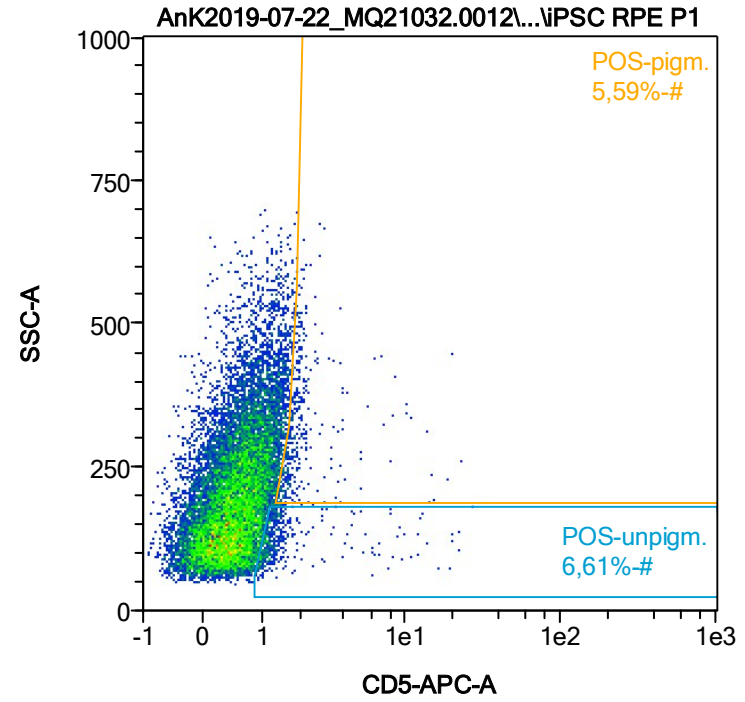

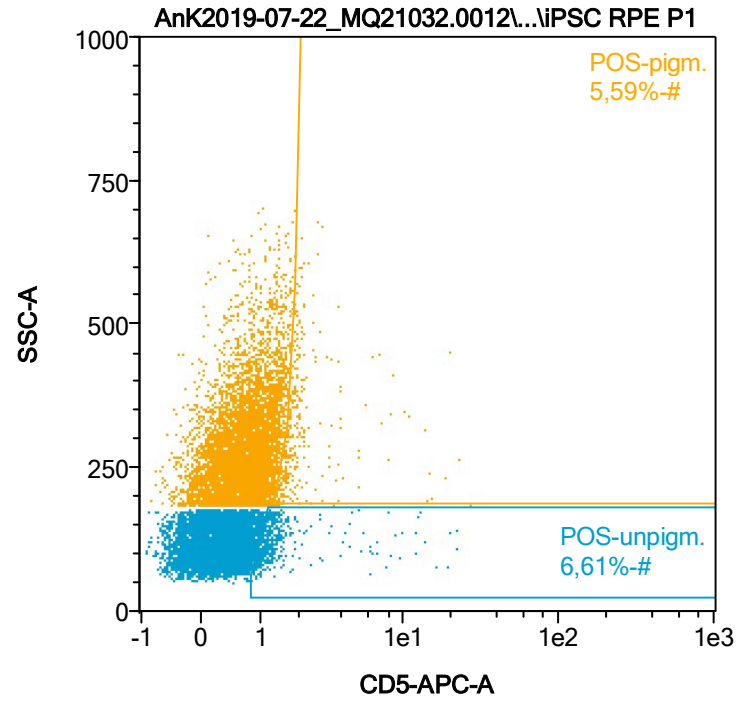

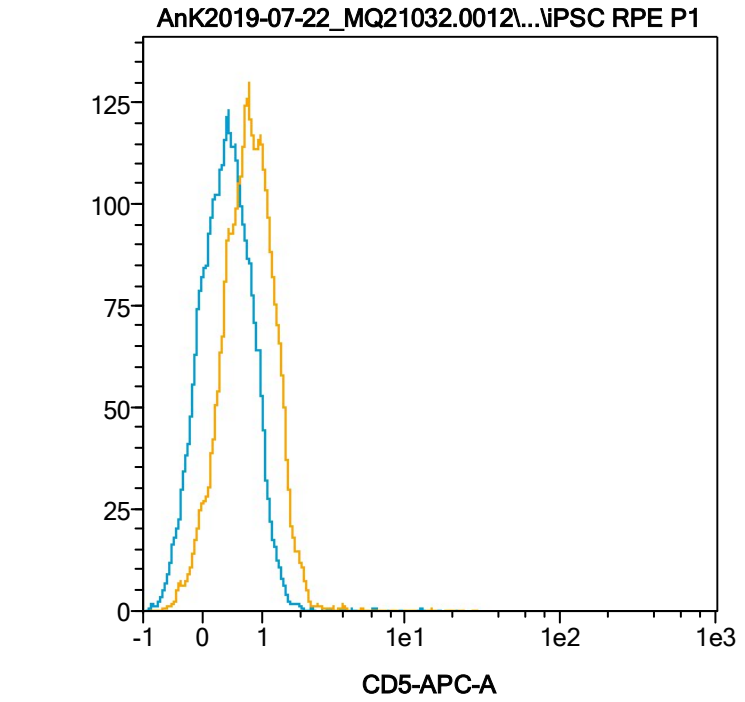

Name	%-#	CD5-APC-A Mean	CD5-APC-A Median
POS-unpigm.	6,61	1,84	1,19
POS-pigm.	5,59	2,42	1,78

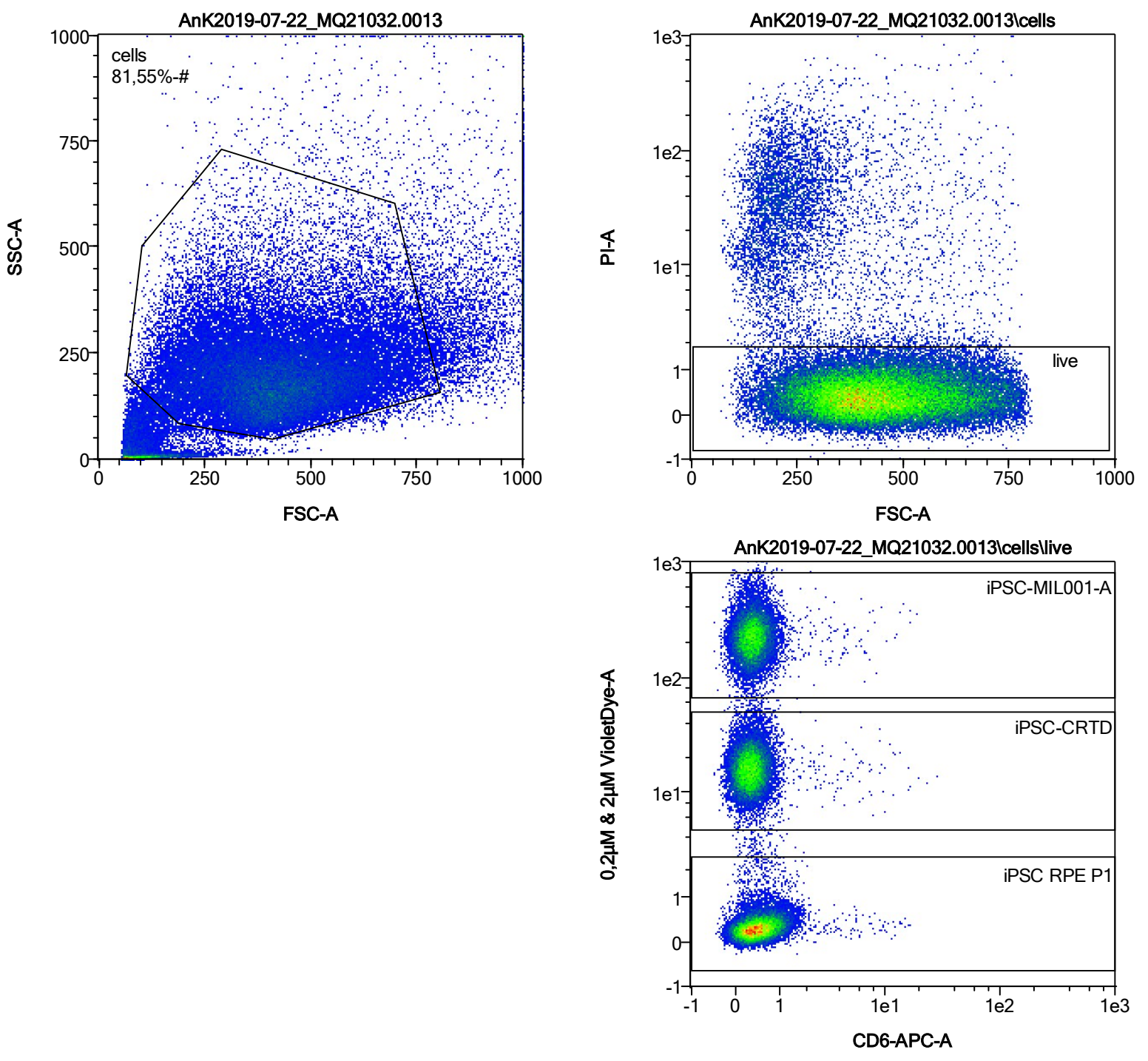

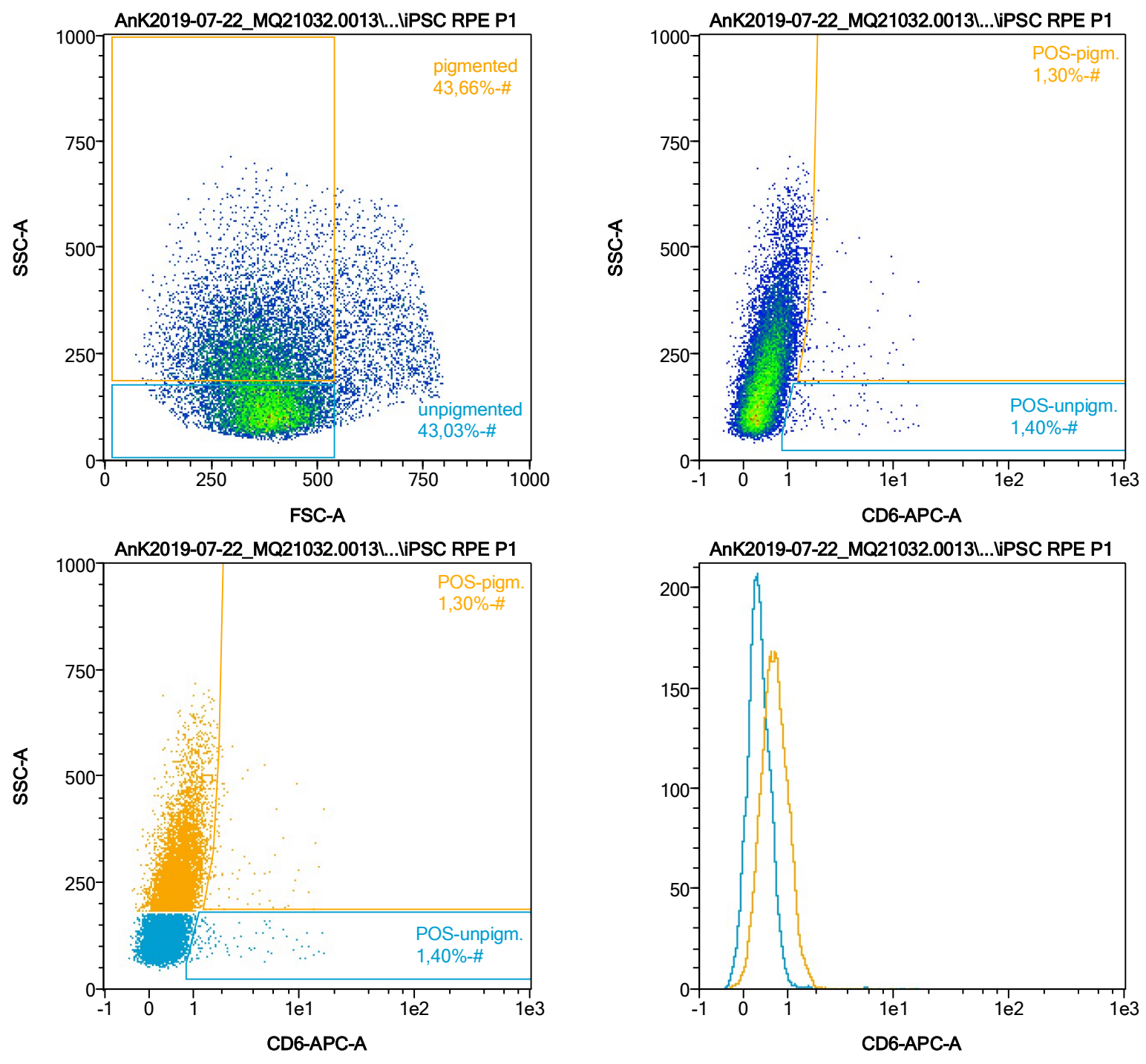

Name	%-#	CD6-APC-A Mean	CD6-APC-A Median
POS-unpigm.	1,40	3,46	1,59
POS-pigm.	1,30	3,70	2,08

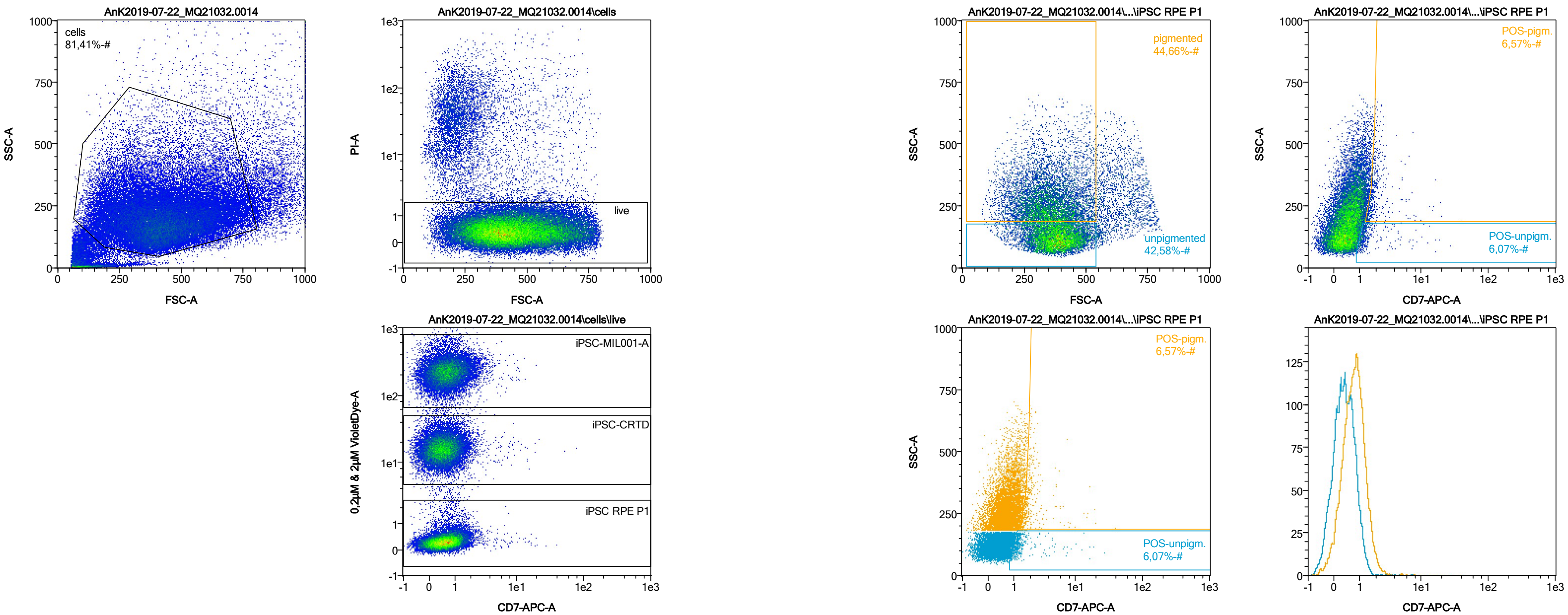

Name	%-#	CD7-APC-A Mean	CD7-APC-A Median
POS-unpigm.	6,07	1,74	1,20
POS-pigm.	6,57	2,34	1,79

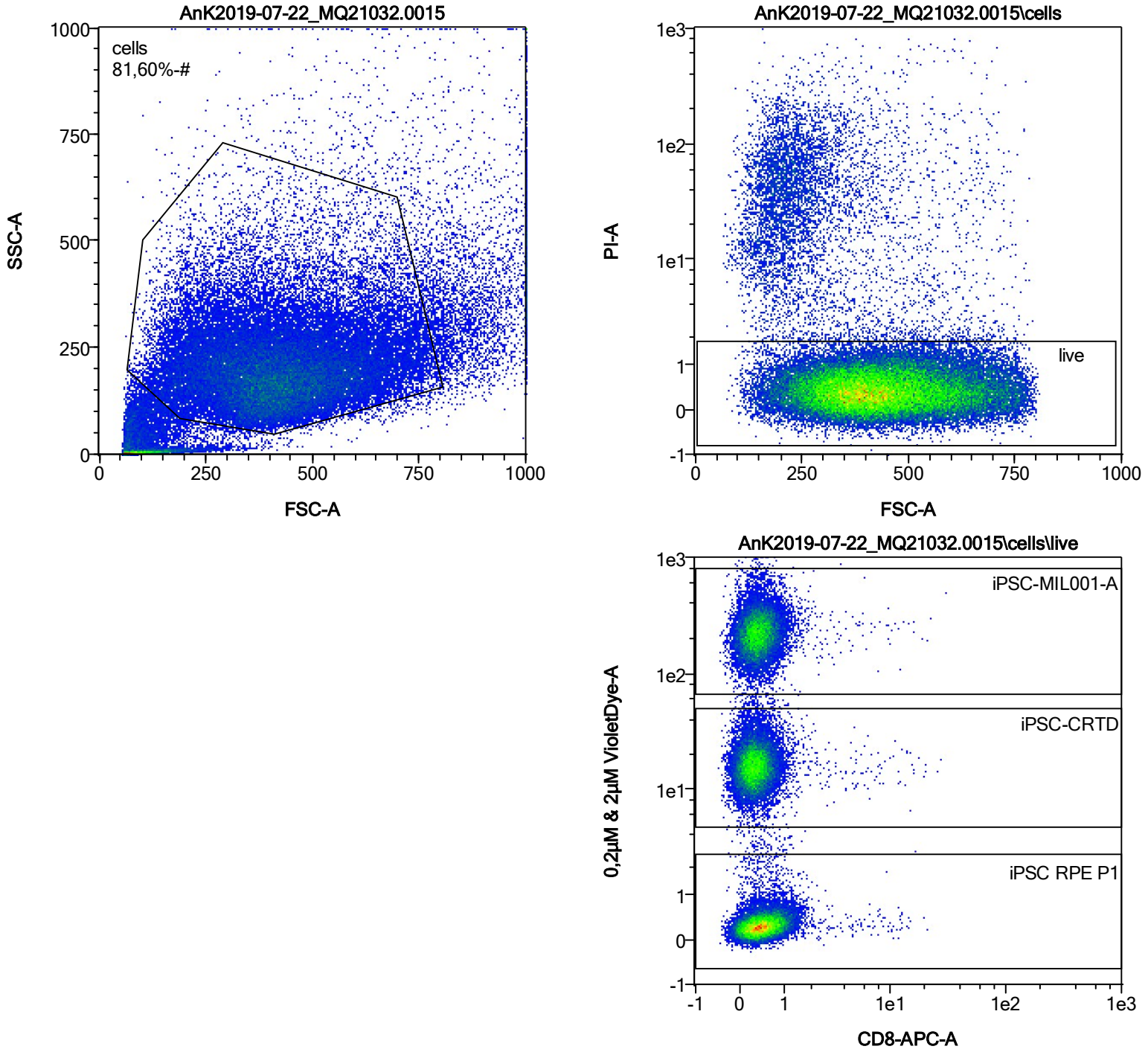

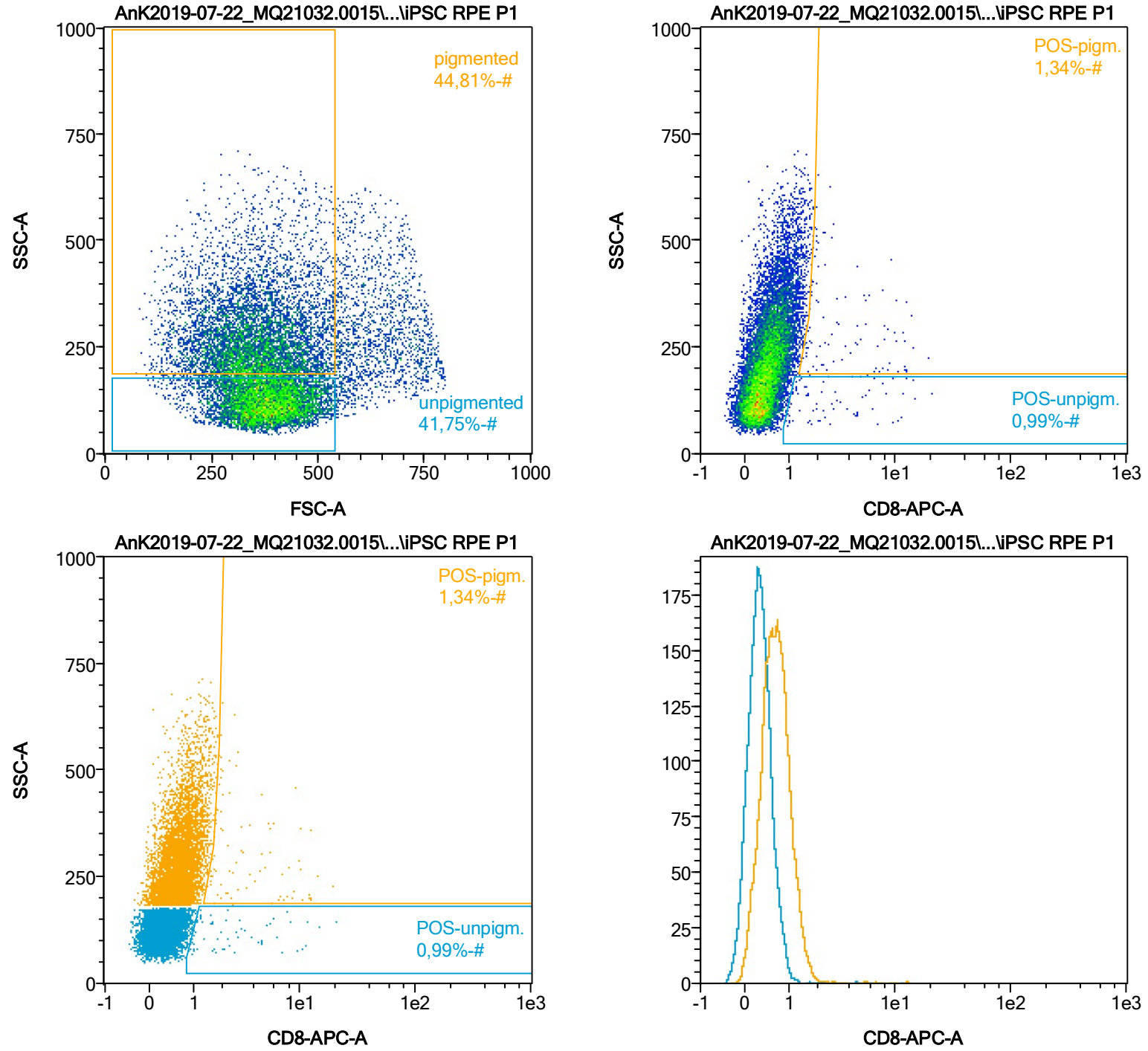

Name	%-#	CD8-APC-A Mean	CD8-APC-A Median
POS-unpigm.	0,99	3,52	1,96
POS-pigm.	1,34	4,32	2,43

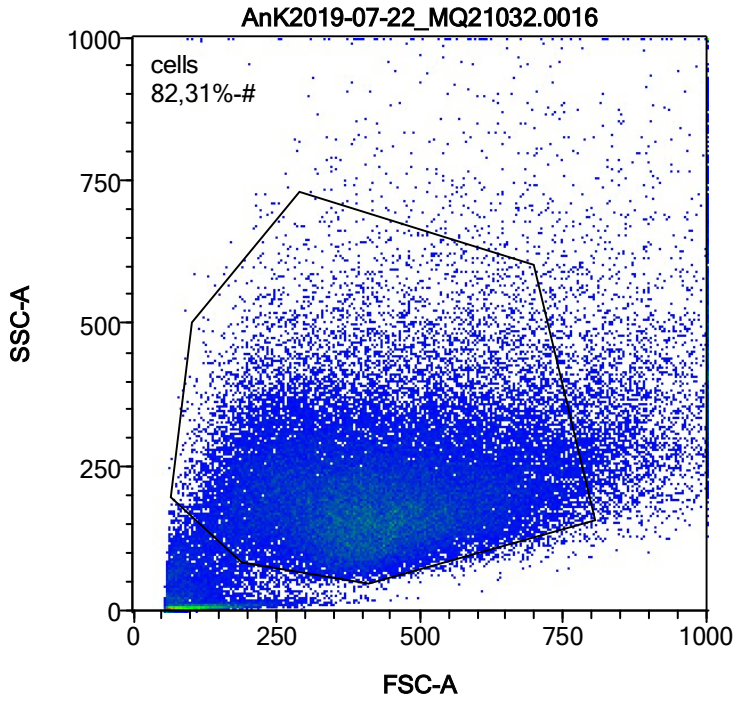

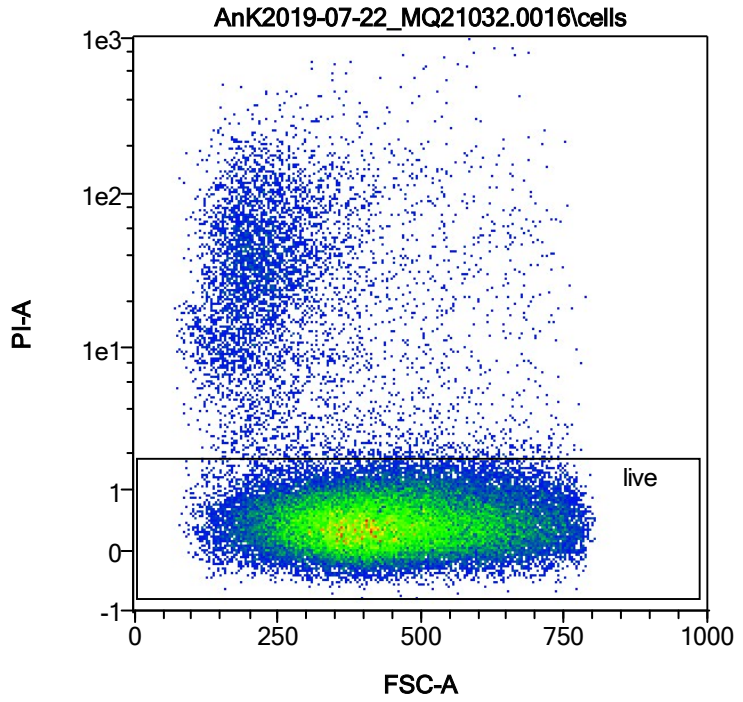

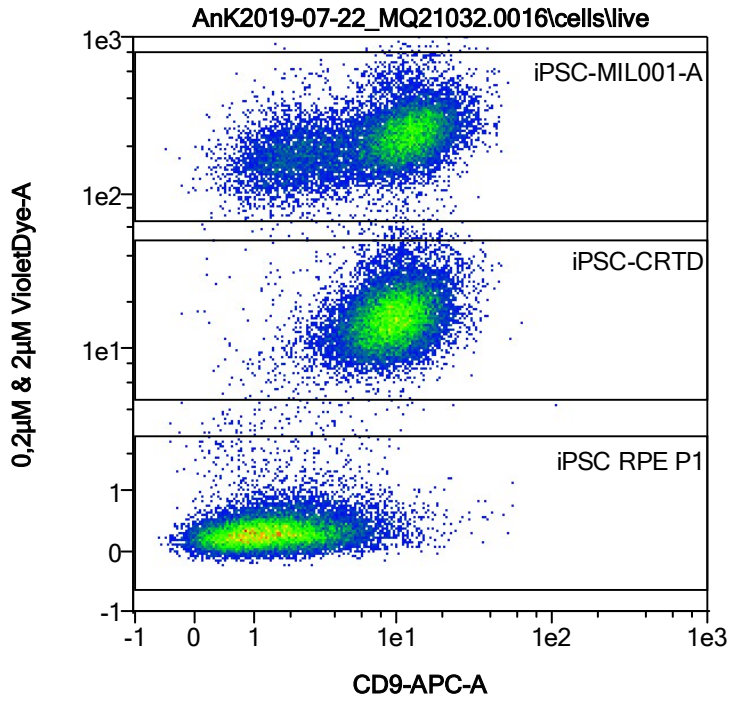

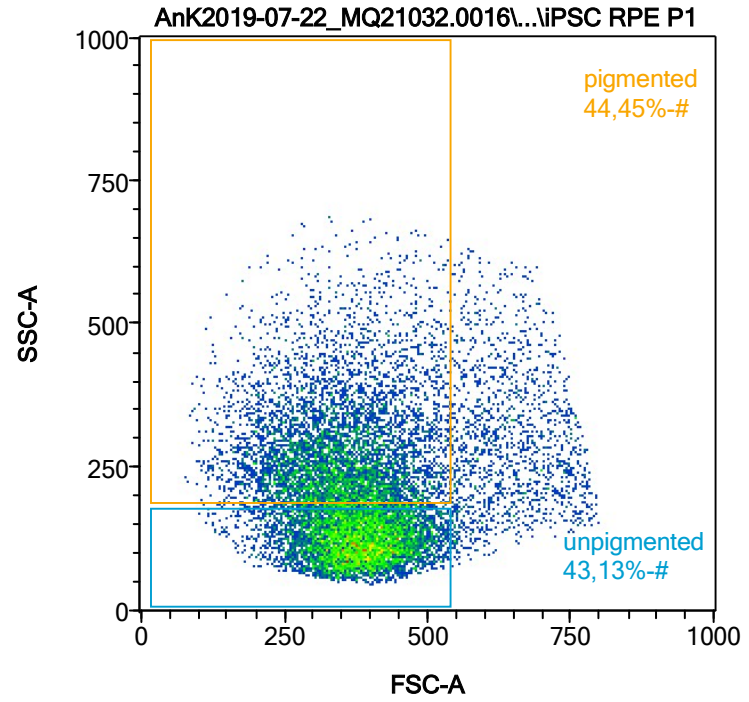

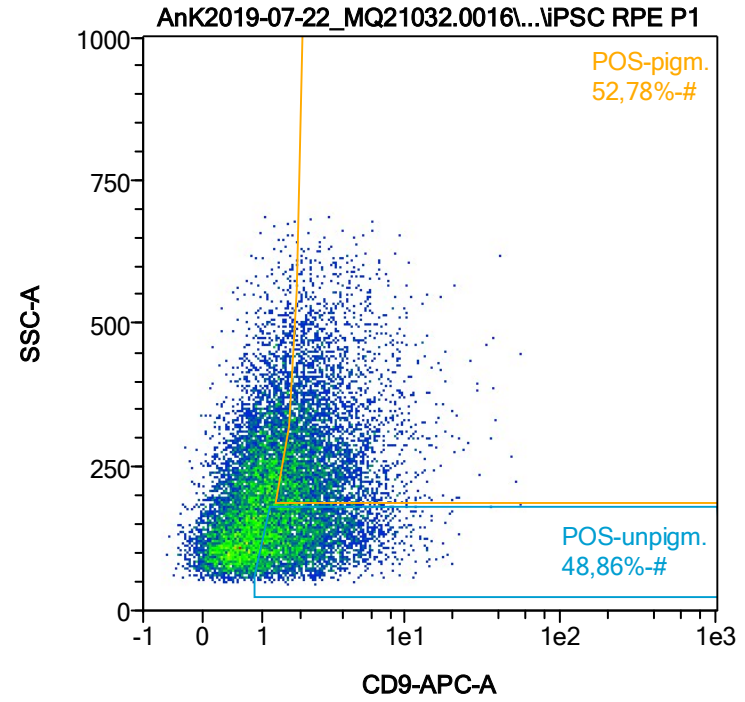

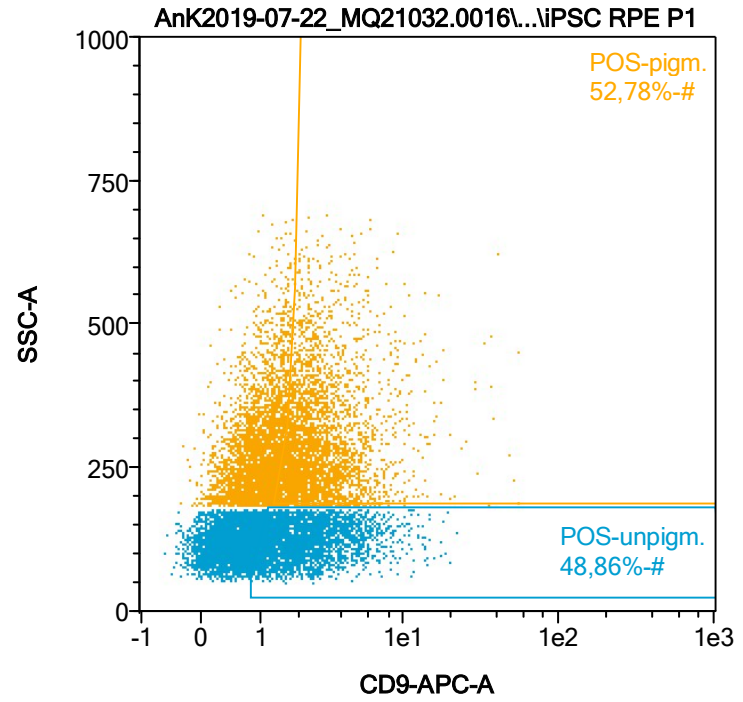

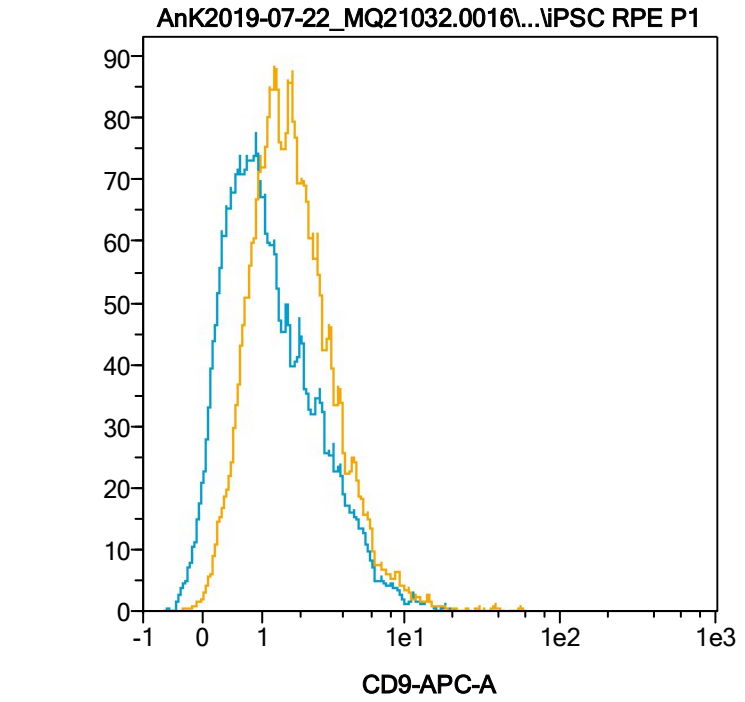

Name	%-#	CD9-APC-A Mean	CD9-APC-A Median
POS-unpigm.	48,86	2,60	1,98
POS-pigm.	52,78	3,28	2,50

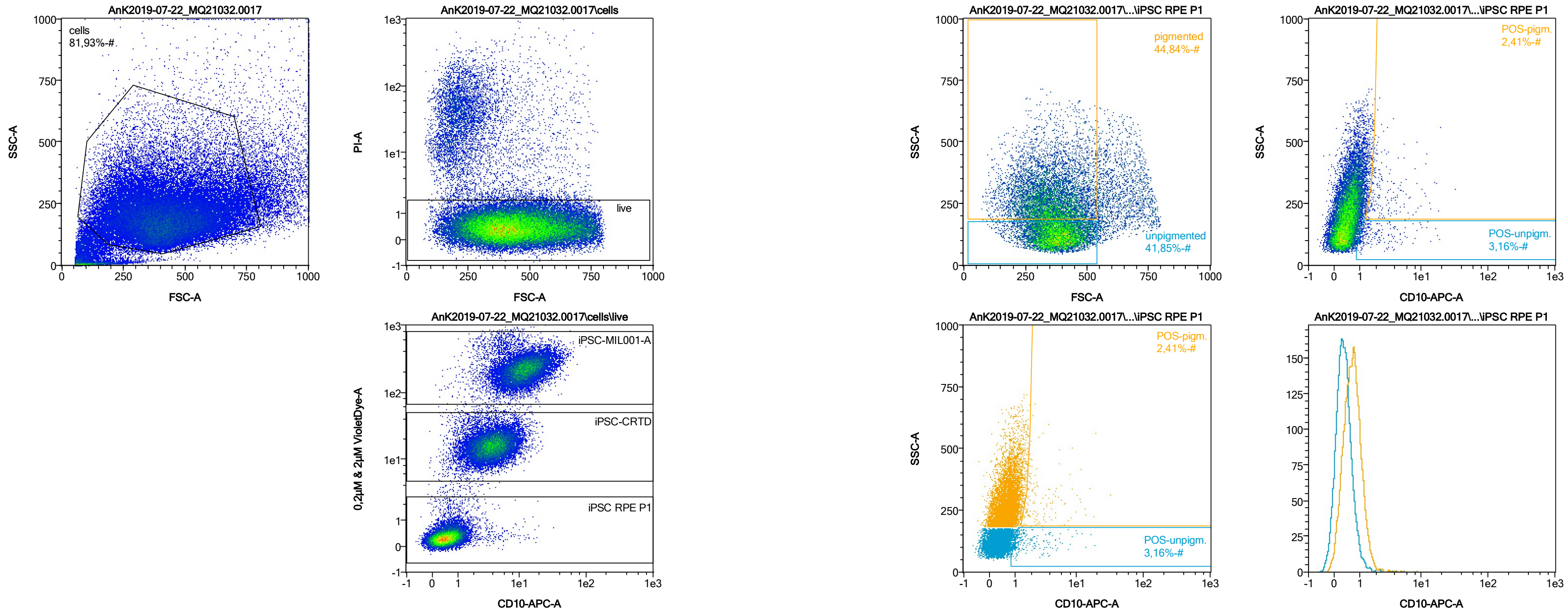

Name	%-#	CD10-APC-A Mean	CD10-APC-A Median
POS-unpigm.	3,16	2,40	1,41
POS-pigm.	2,41	3,58	2,14

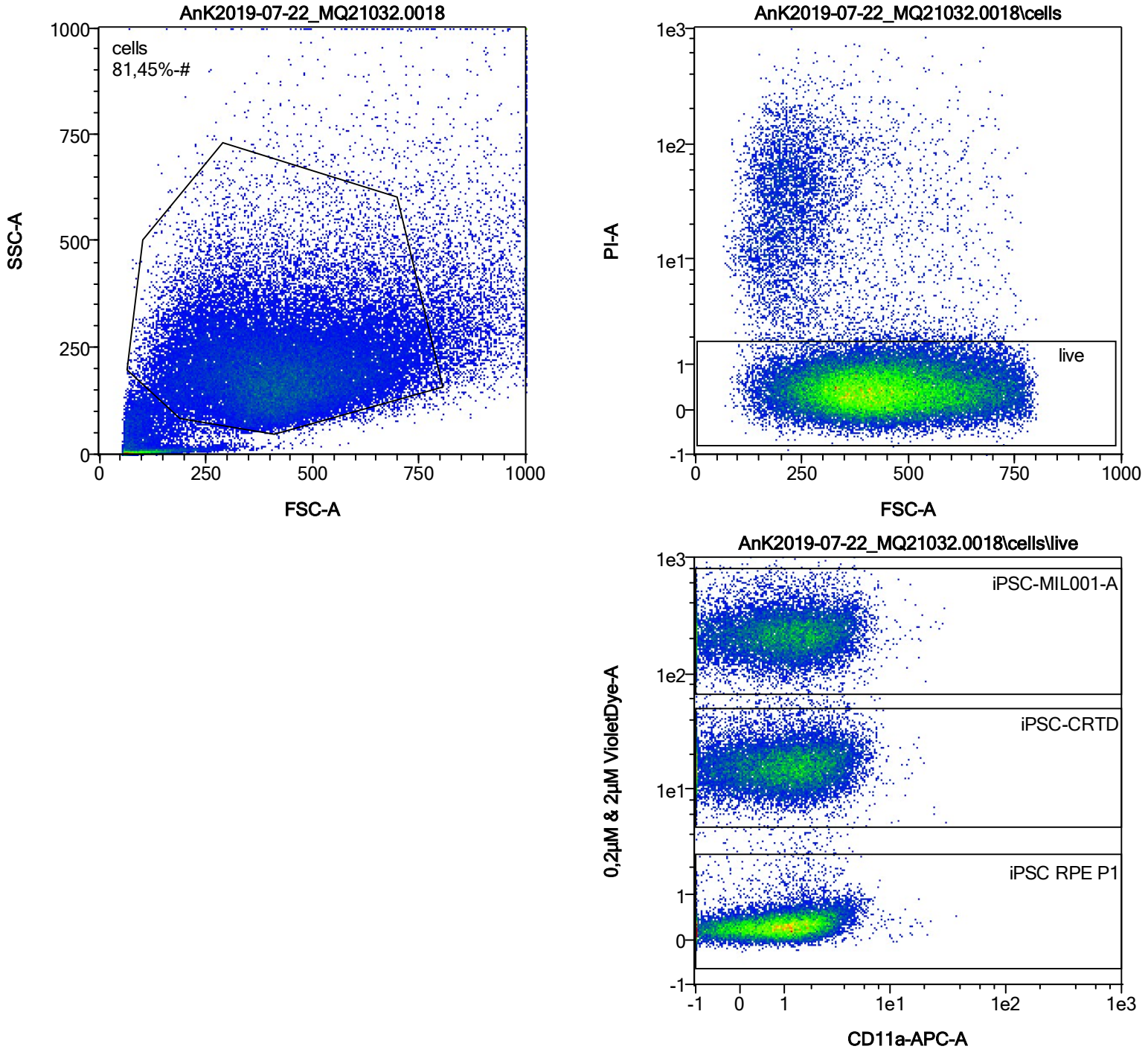

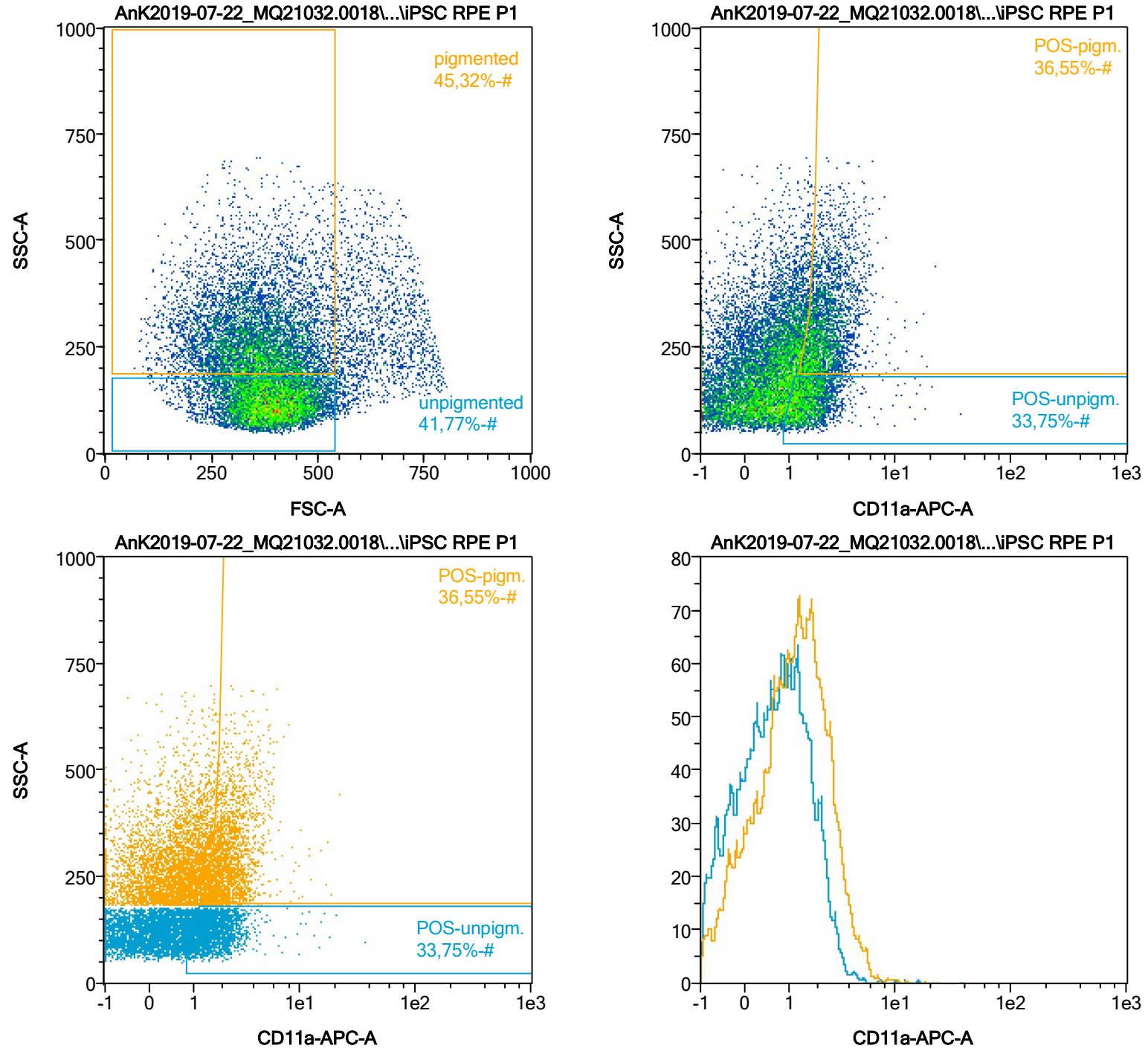

Name	%-#	CD11a-APC-A Mean	CD11a-APC-A Median
POS-unpigm.	33,75	1,81	1,55
POS-pigm.	36,55	2,53	2,23

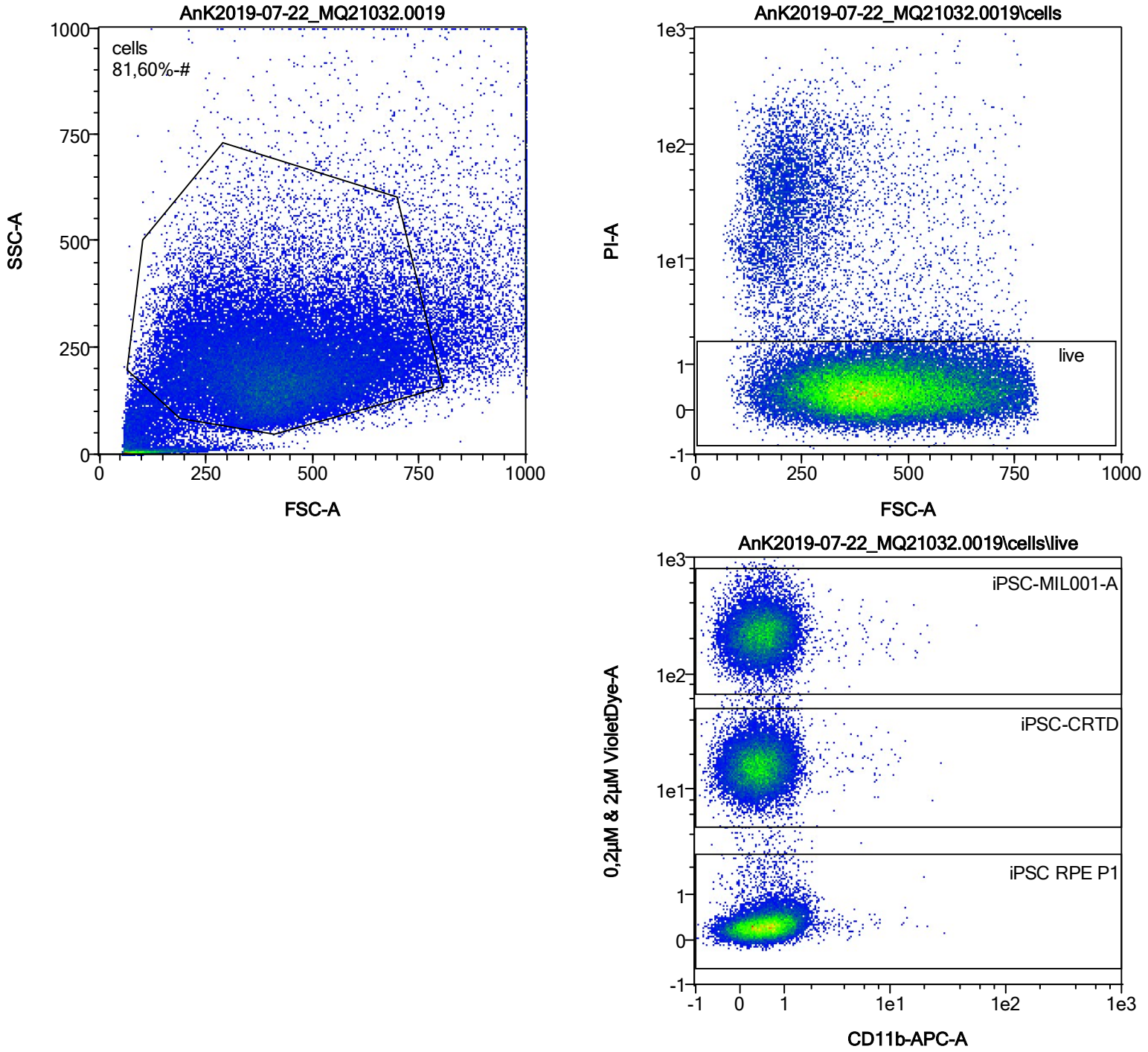

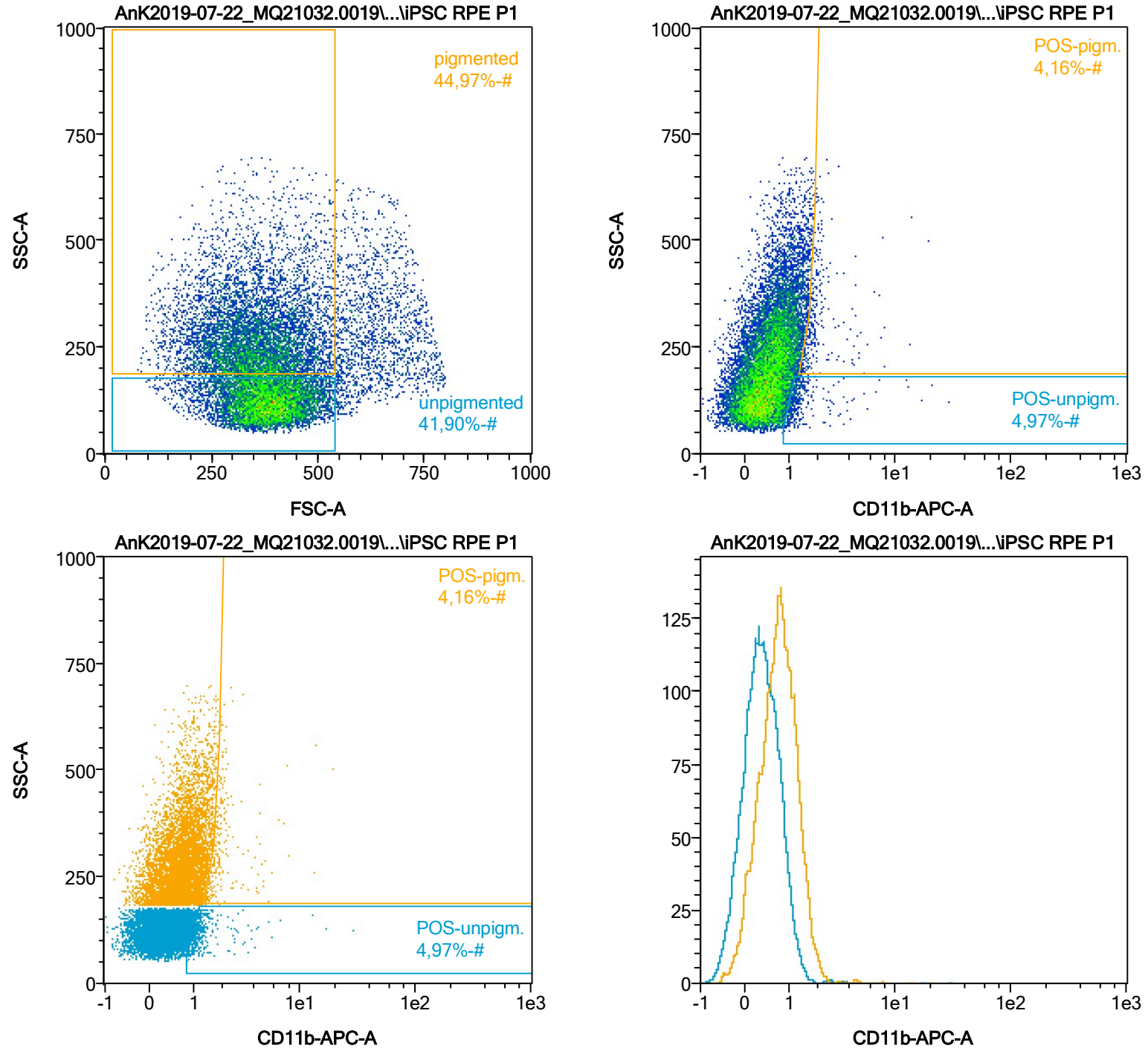

Name	%-#	CD11b-APC-A Mean	CD11b-APC-A Median
POS-unpigm.	4,97	1,58	1,20
POS-pigm.	4,16	2,23	1,82

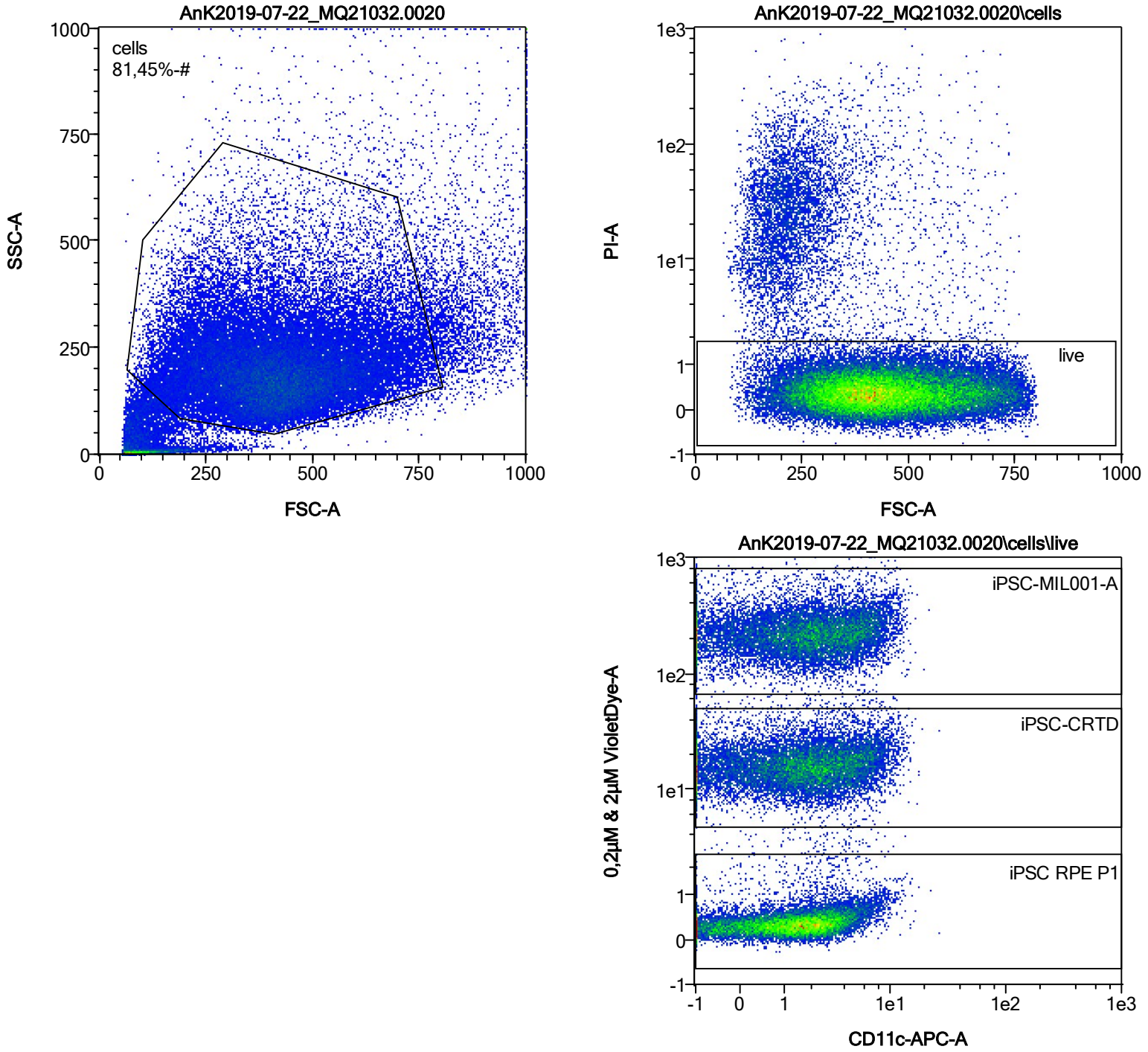

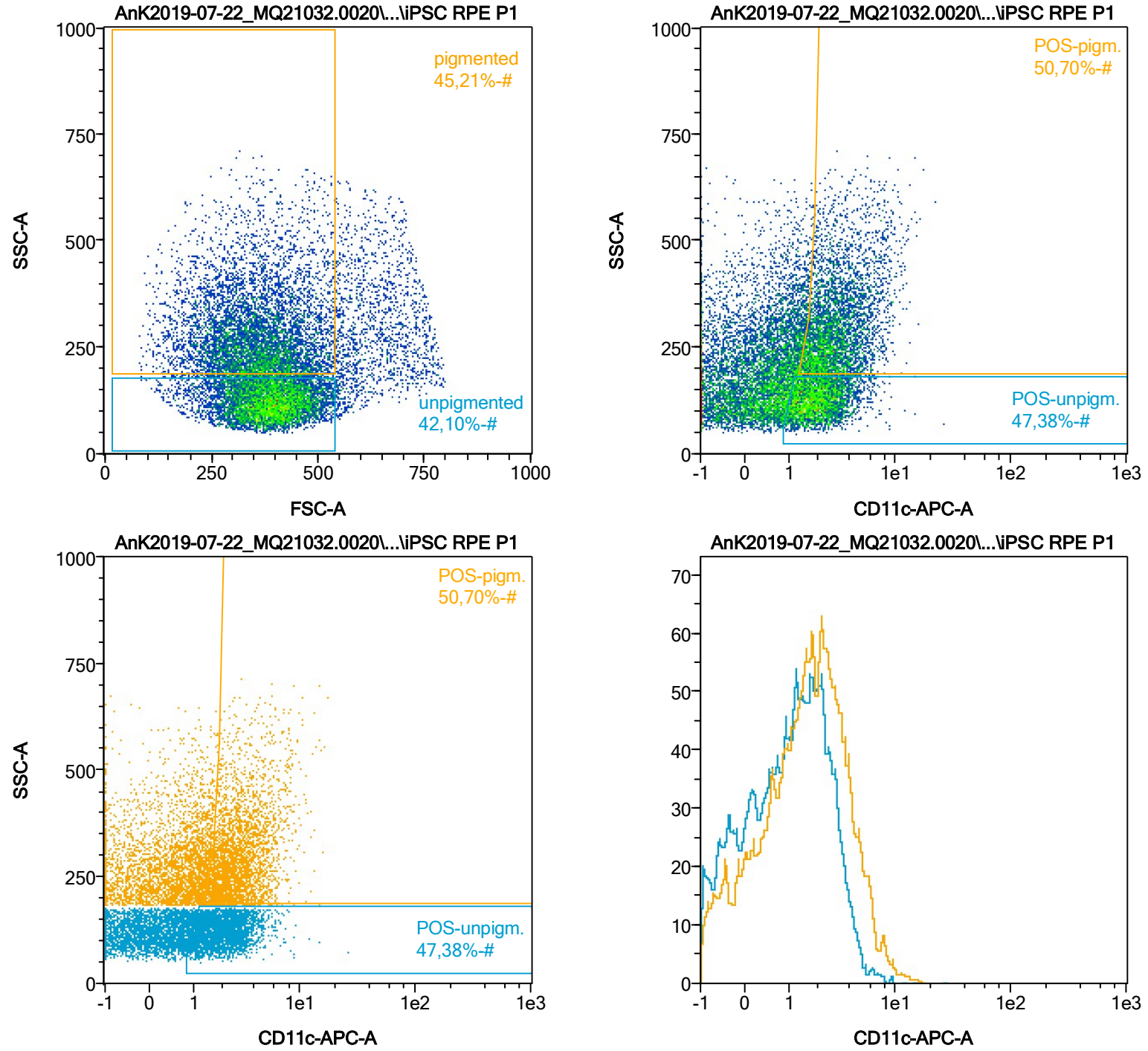

Name	%-#	CD11c-APC-A Mean	CD11c-APC-A Median
POS-unpigm.	47,38	2,20	1,94
POS-pigm.	50,70	3,21	2,70

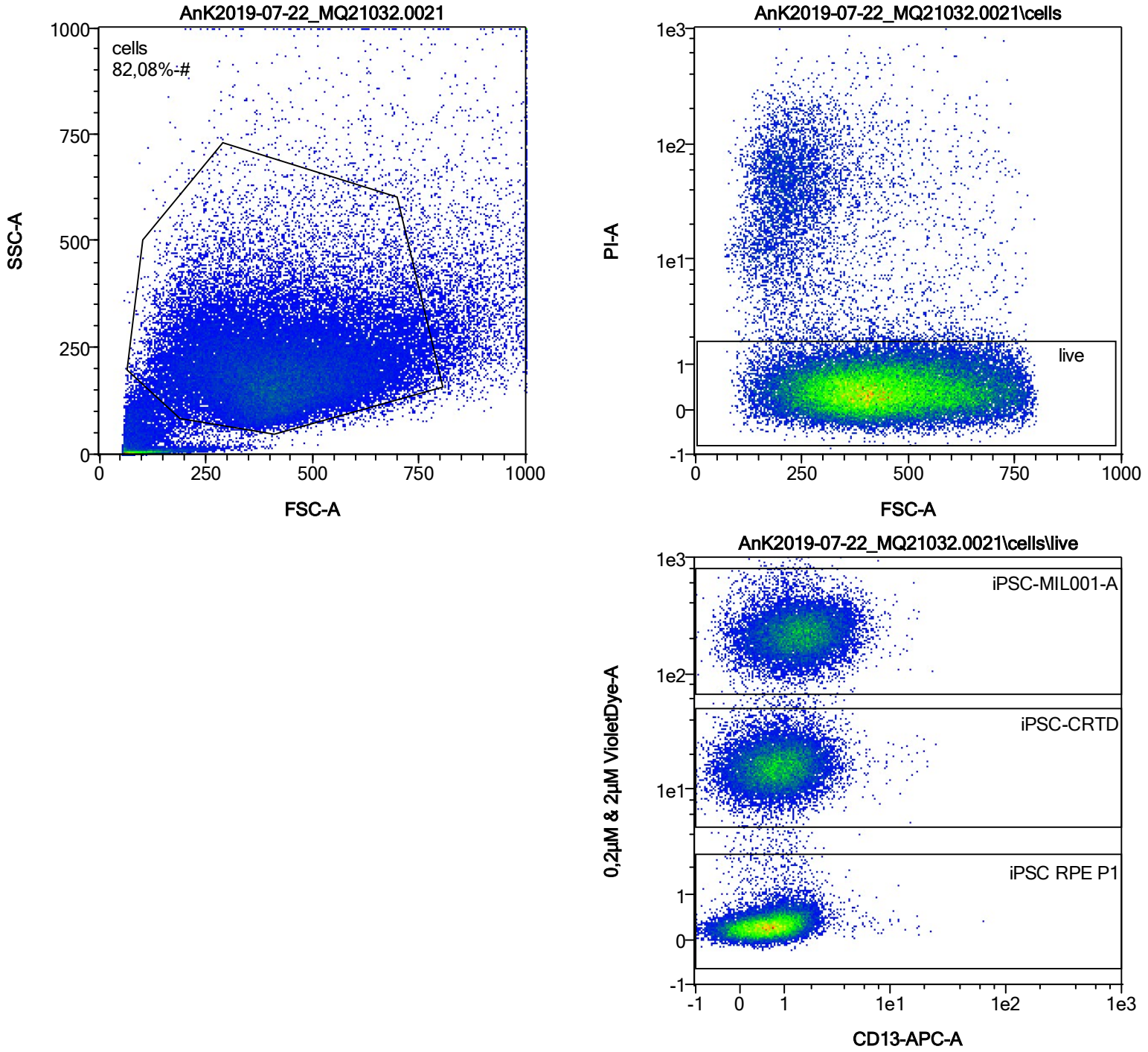

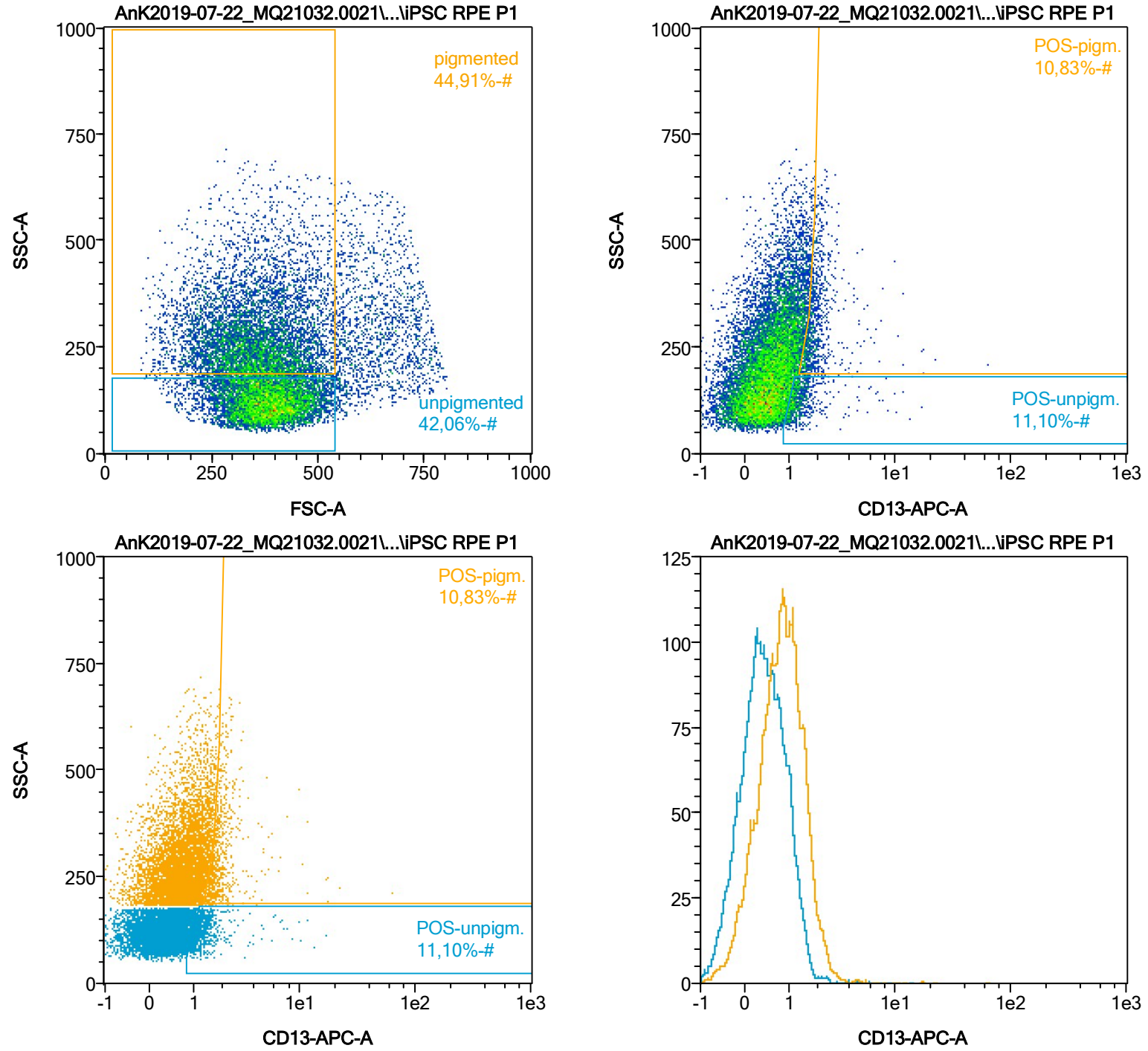

Name	%-#	CD13-APC-A Mean	CD13-APC-A Median
POS-unpigm.	11,10	1,47	1,23
POS-pigm.	10,83	2,20	1,83

Date:2024-06-14

Operator: Sebastian Knoebel

Version:2.13.0+54.g95abf0f4

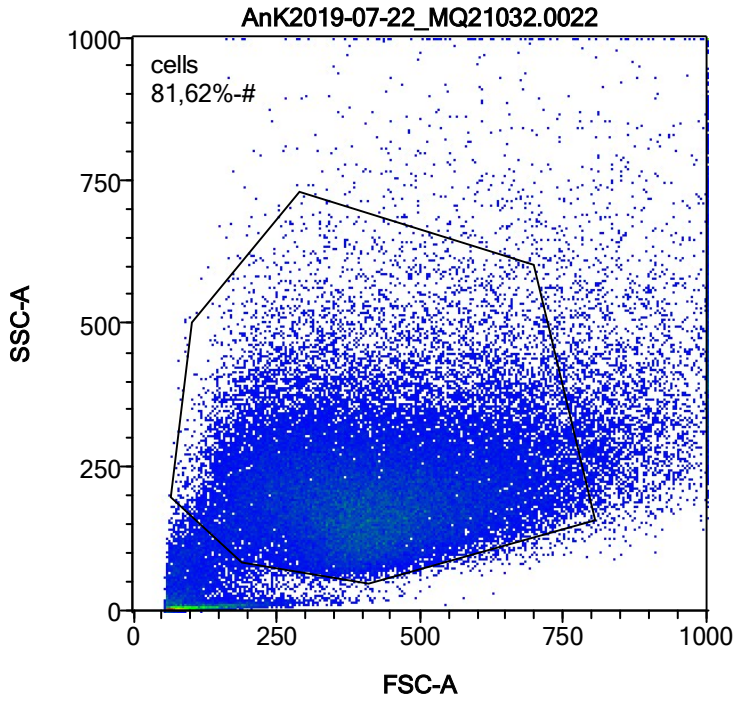

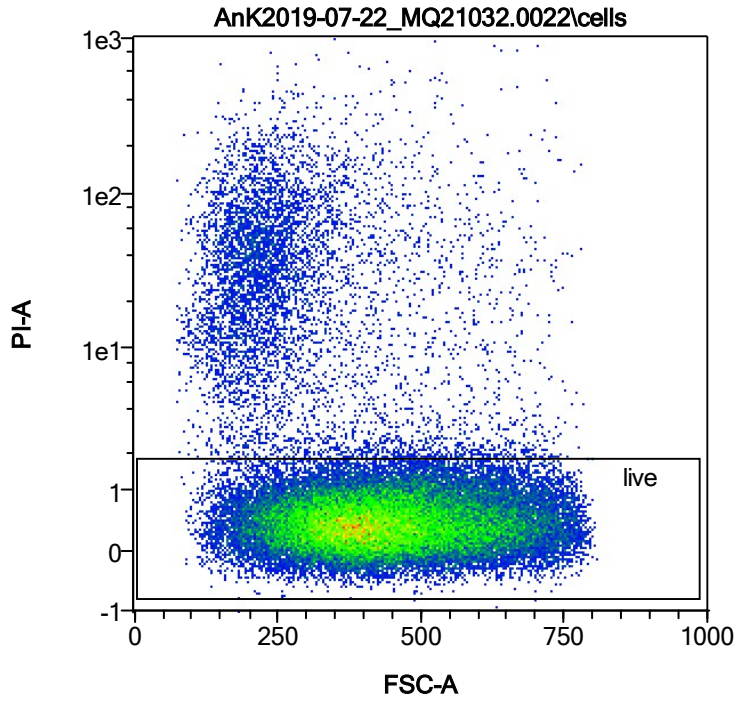

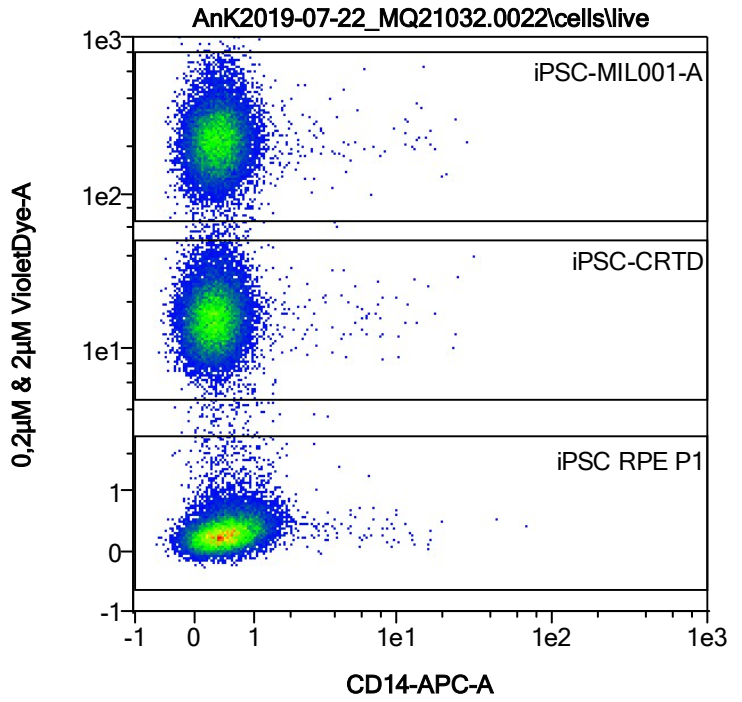

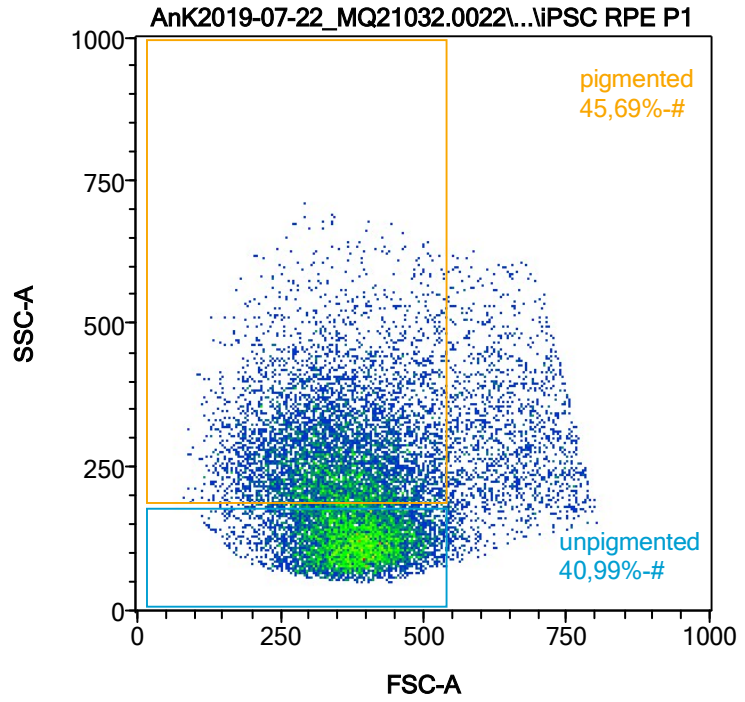

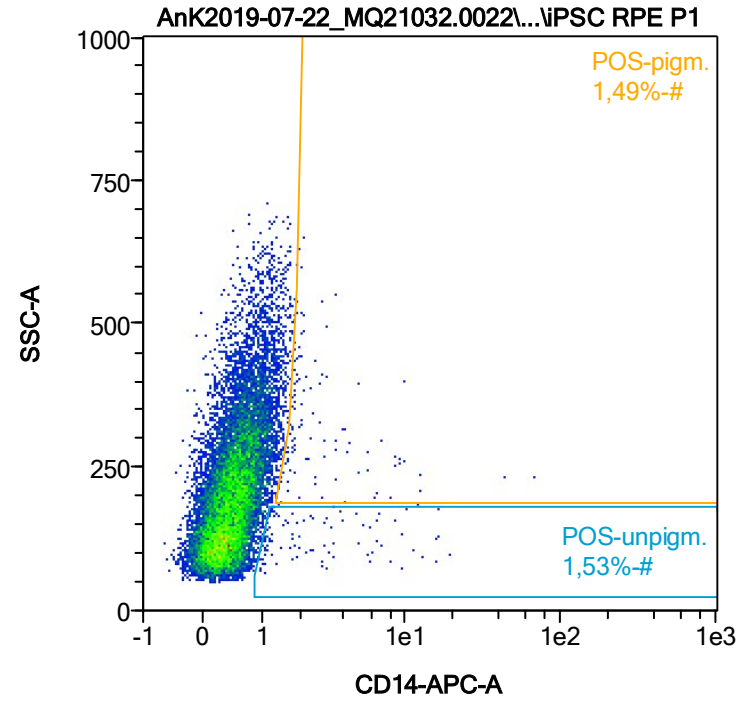

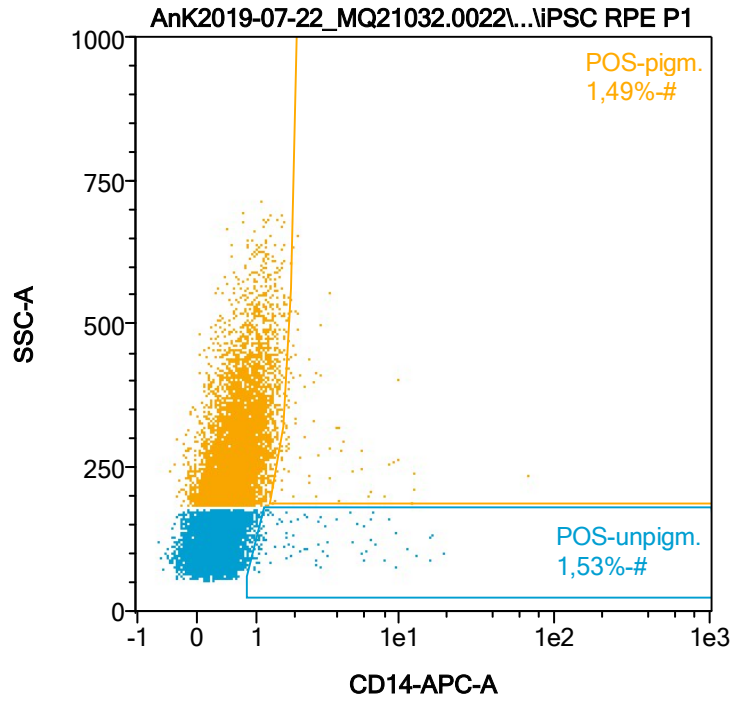

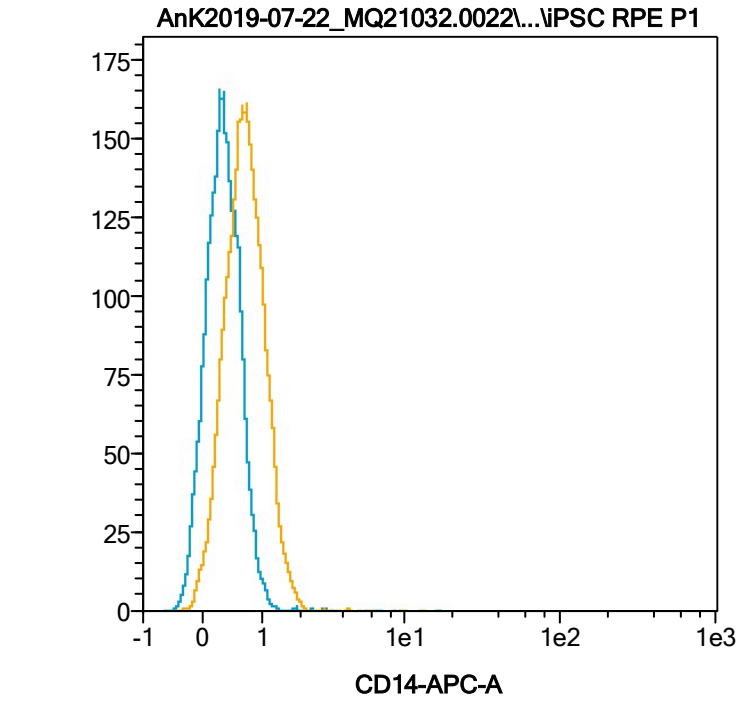

Name	%-#	CD14-APC-A Mean	CD14-APC-A Median
POS-unpigm.	1,53	3,41	1,79
POS-pigm.	1,49	3,64	1,93

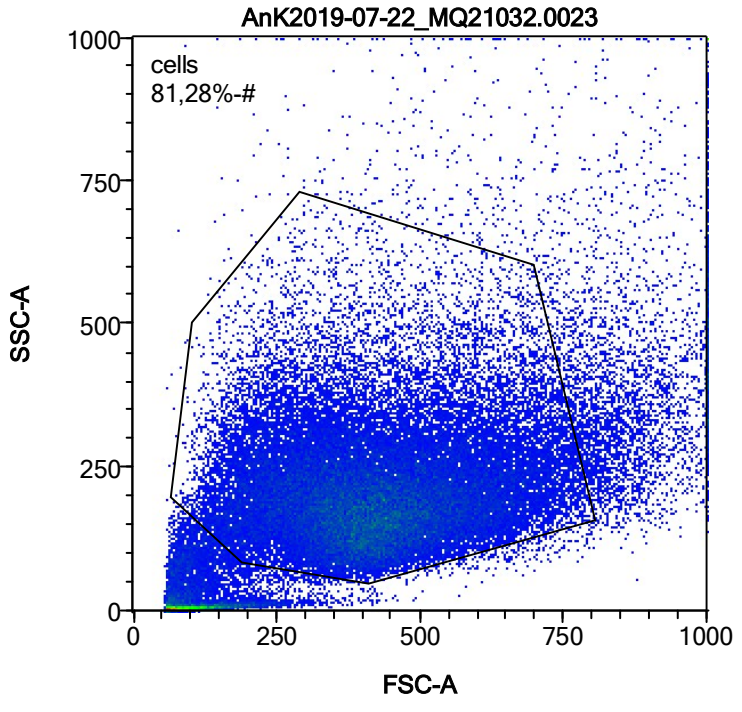

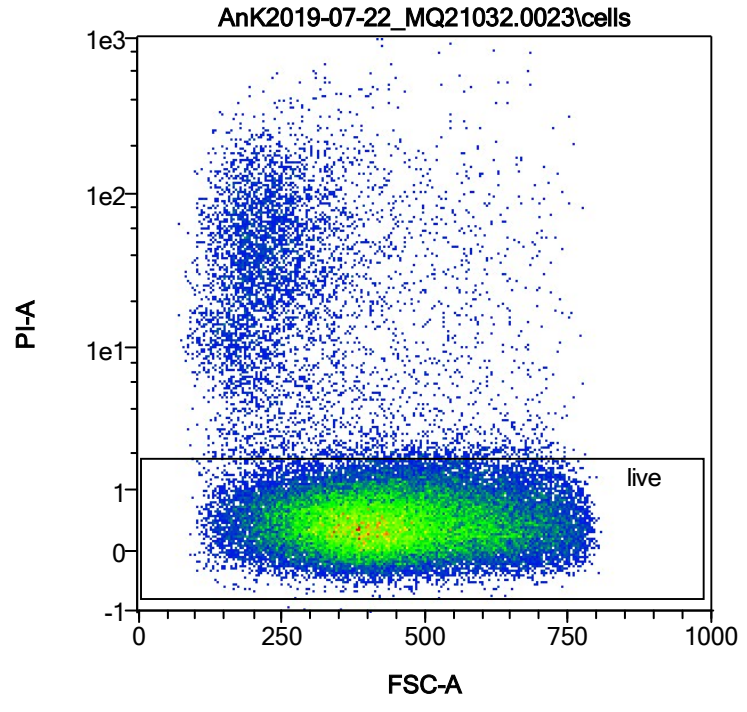

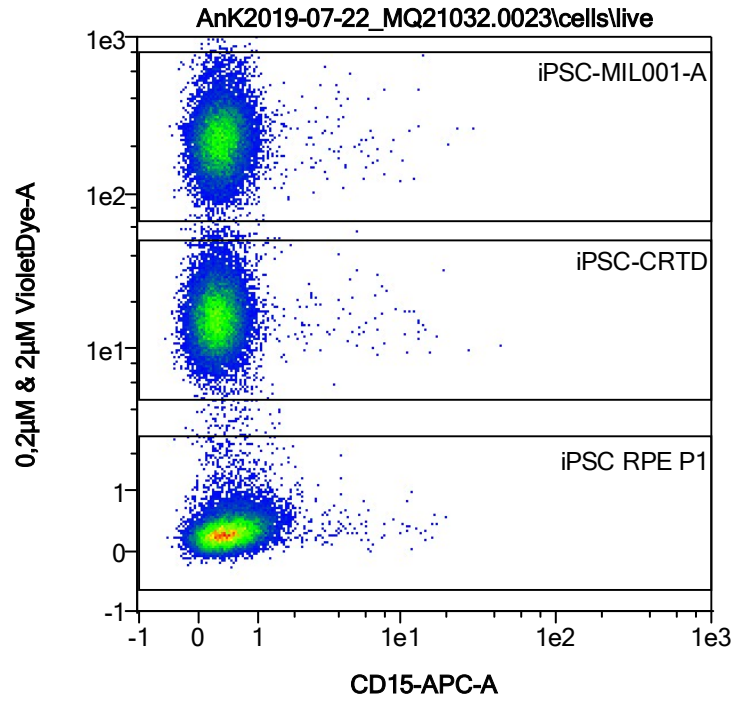

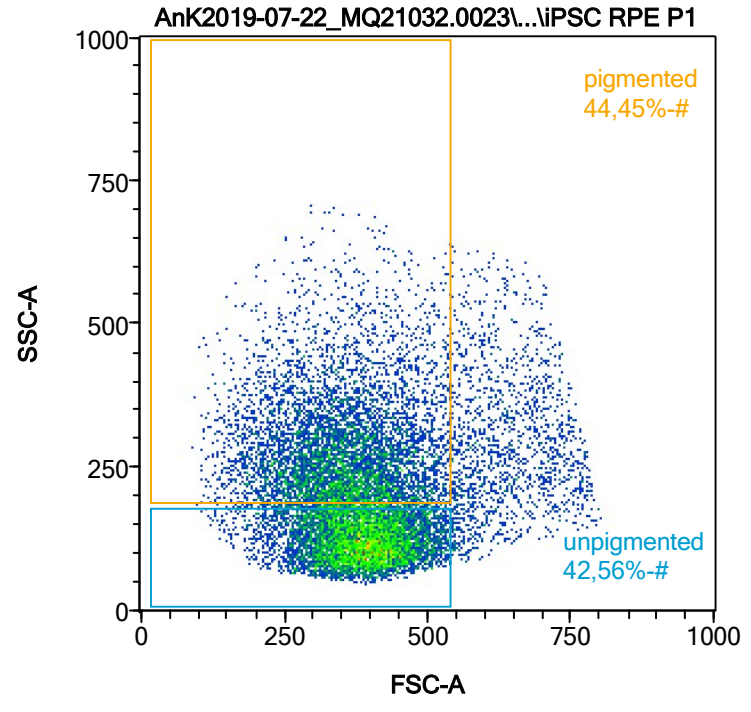

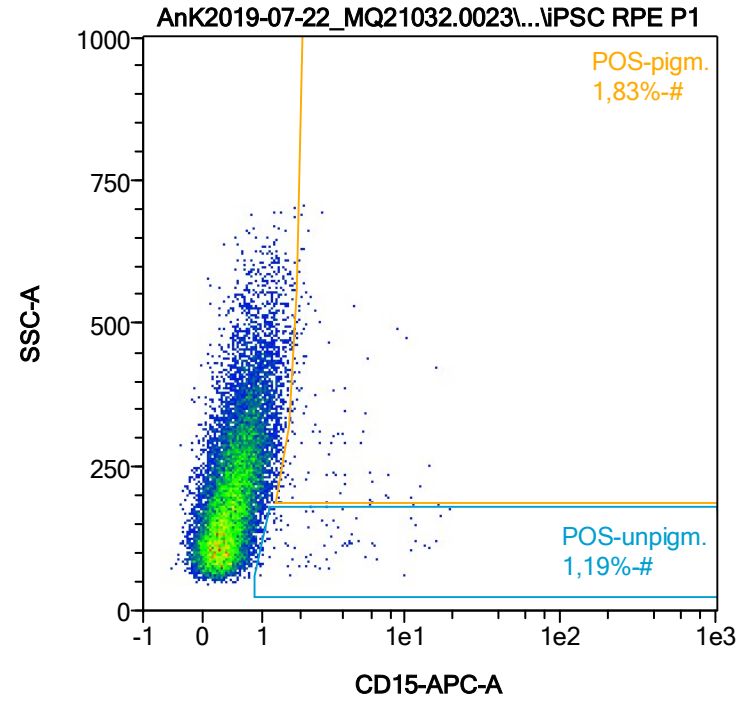

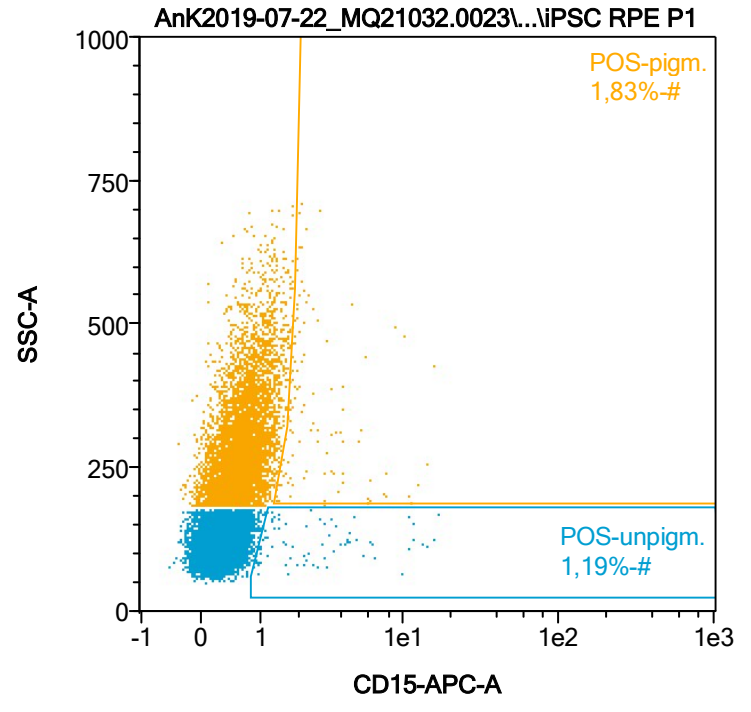

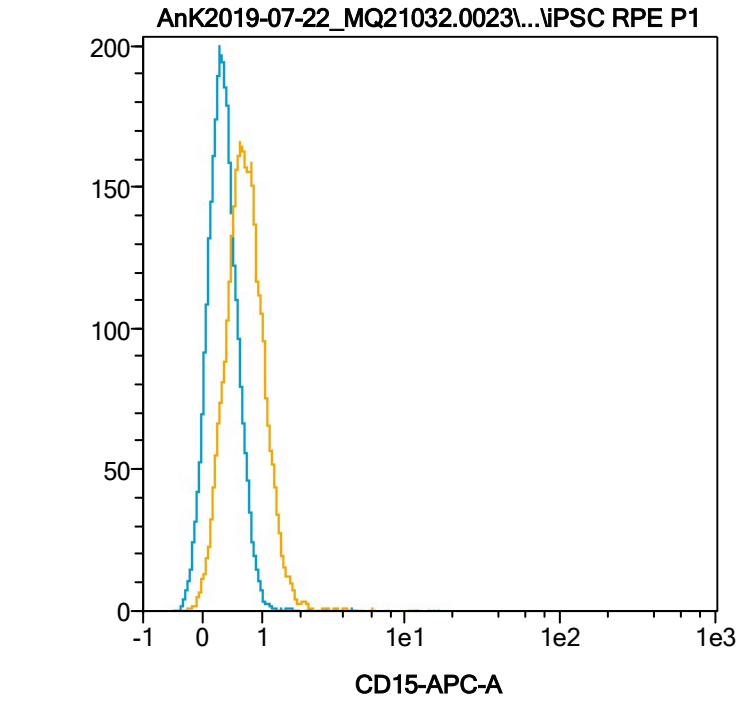

Name	%-#	CD15-APC-A Mean	CD15-APC-A Median
POS-unpigm.	1,19	3,62	2,18
POS-pigm.	1,83	3,15	2,08

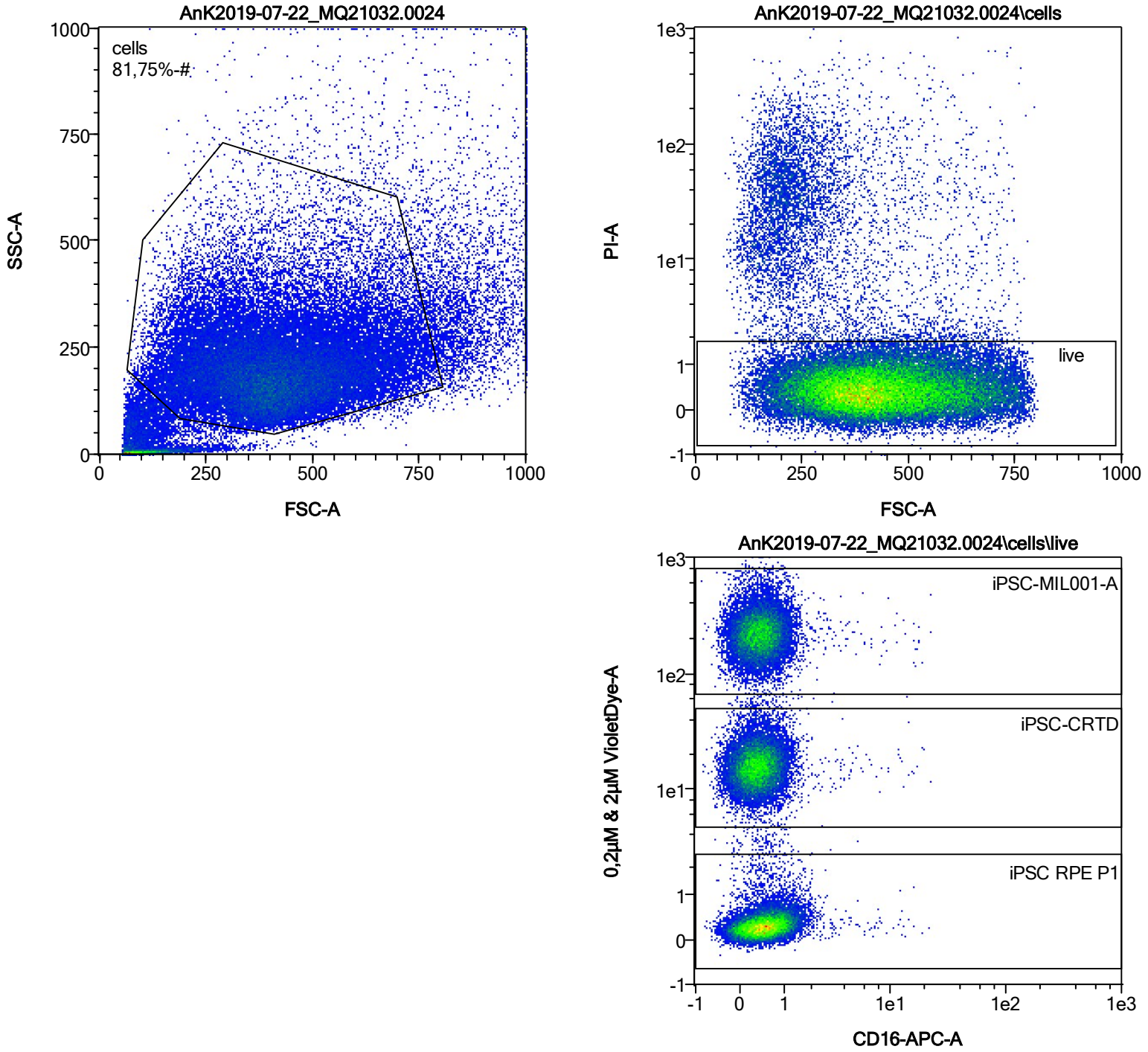

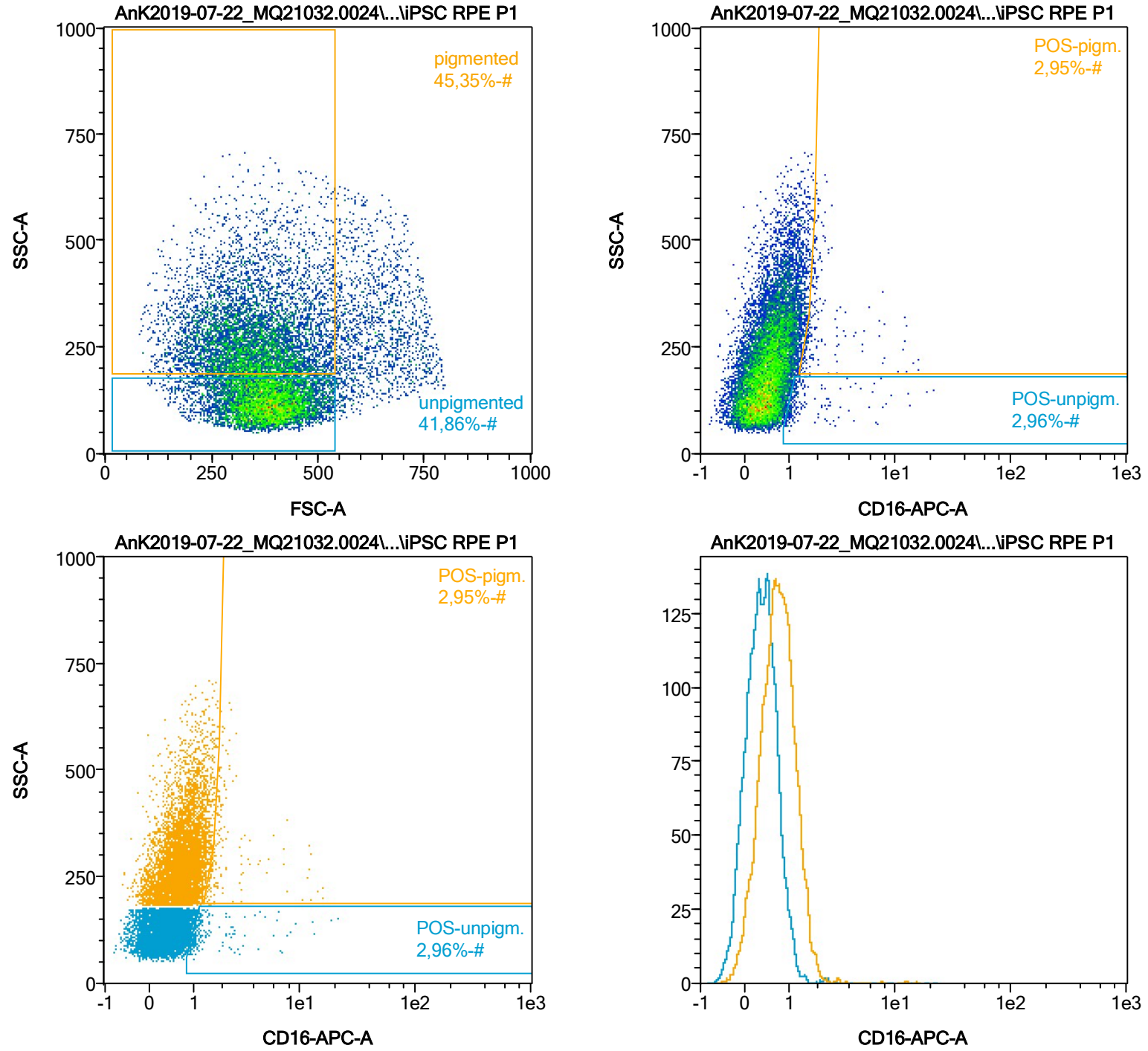

Name	%-#	CD16-APC-A Mean	CD16-APC-A Median
POS-unpigm.	2,96	2,00	1,17
POS-pigm.	2,95	2,59	1,82

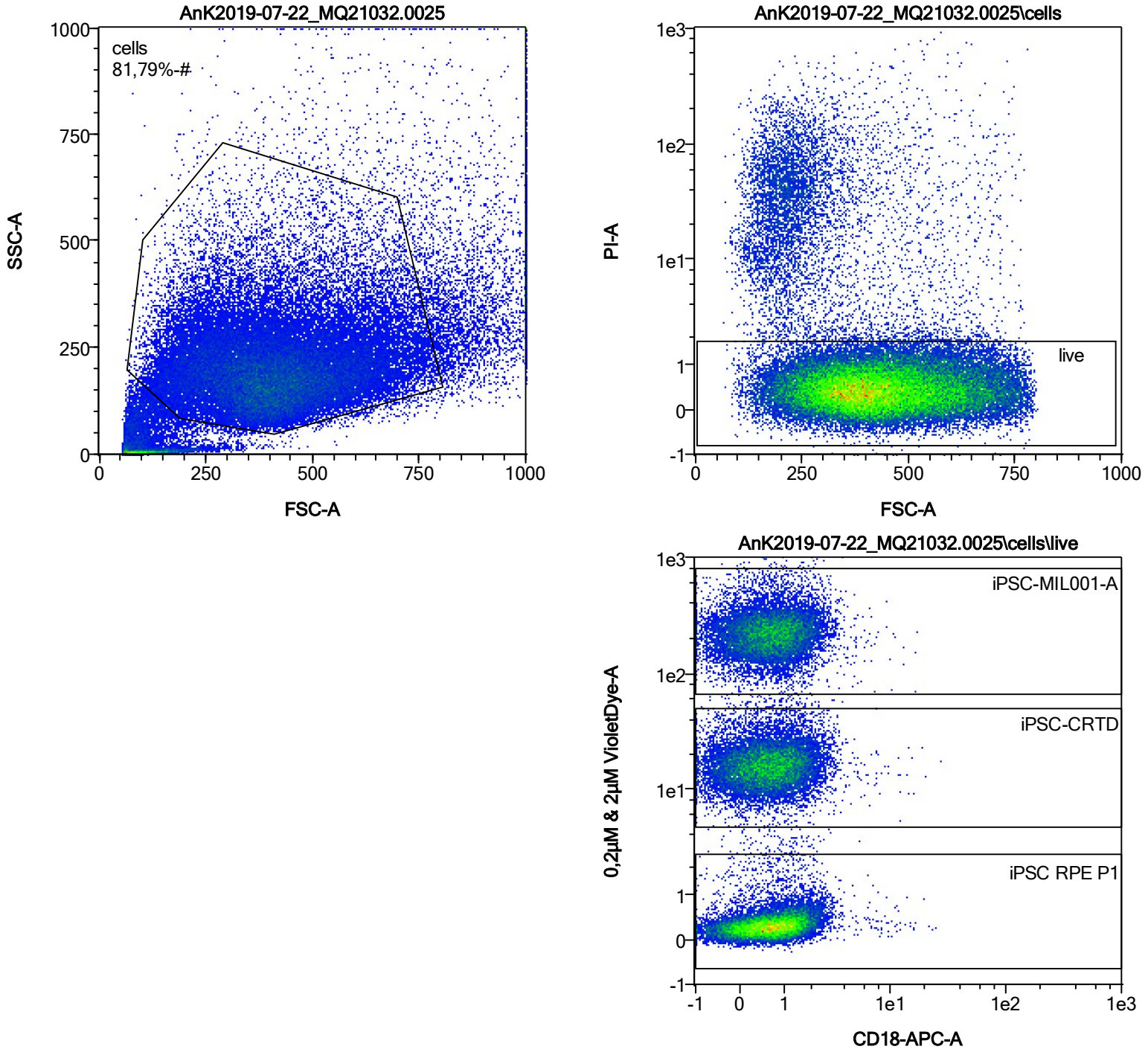

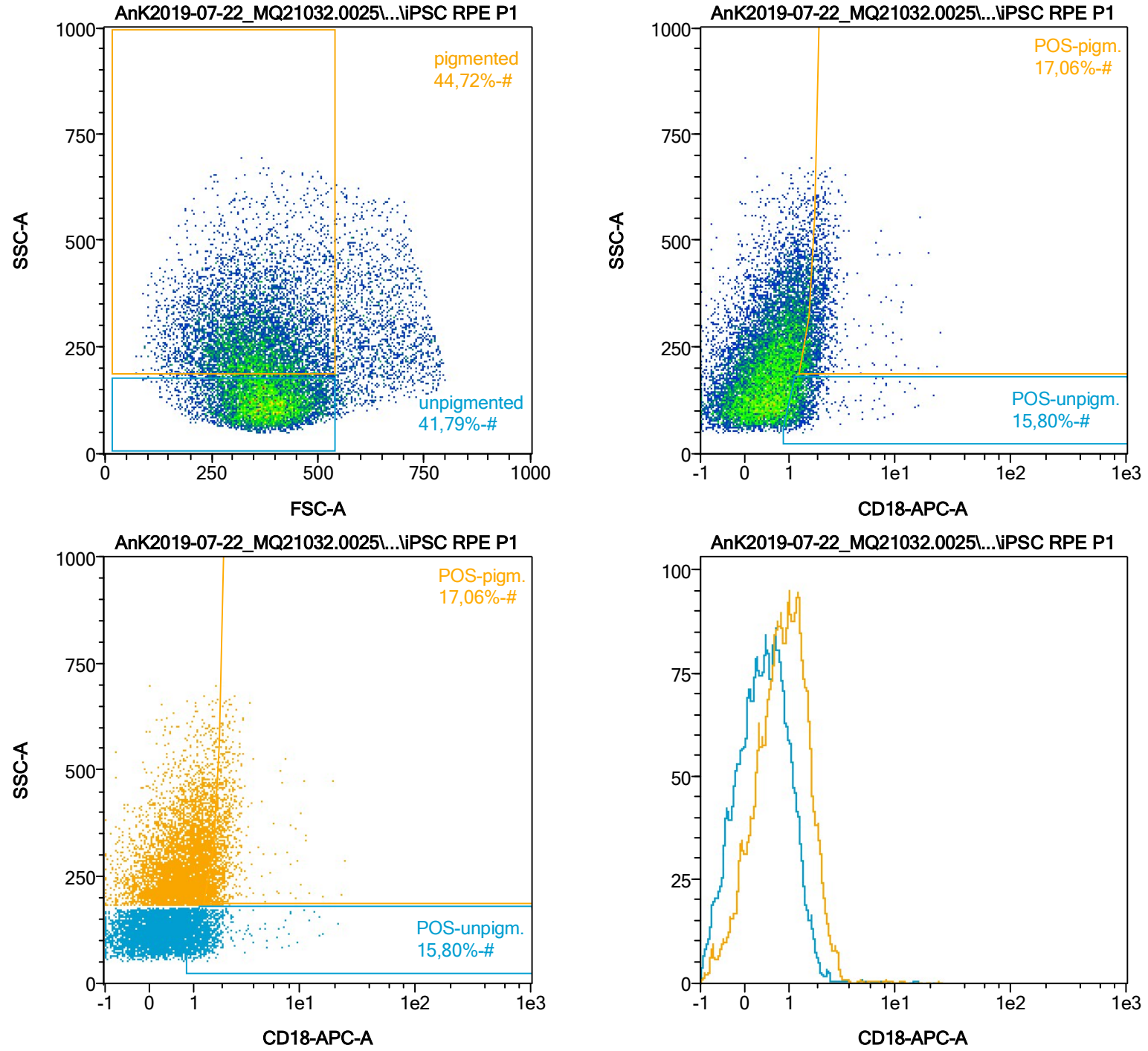

Name	%-#	CD18-APC-A Mean	CD18-APC-A Median
POS-unpigm.	15,80	1,55	1,28
POS-pigm.	17,06	2,16	1,89

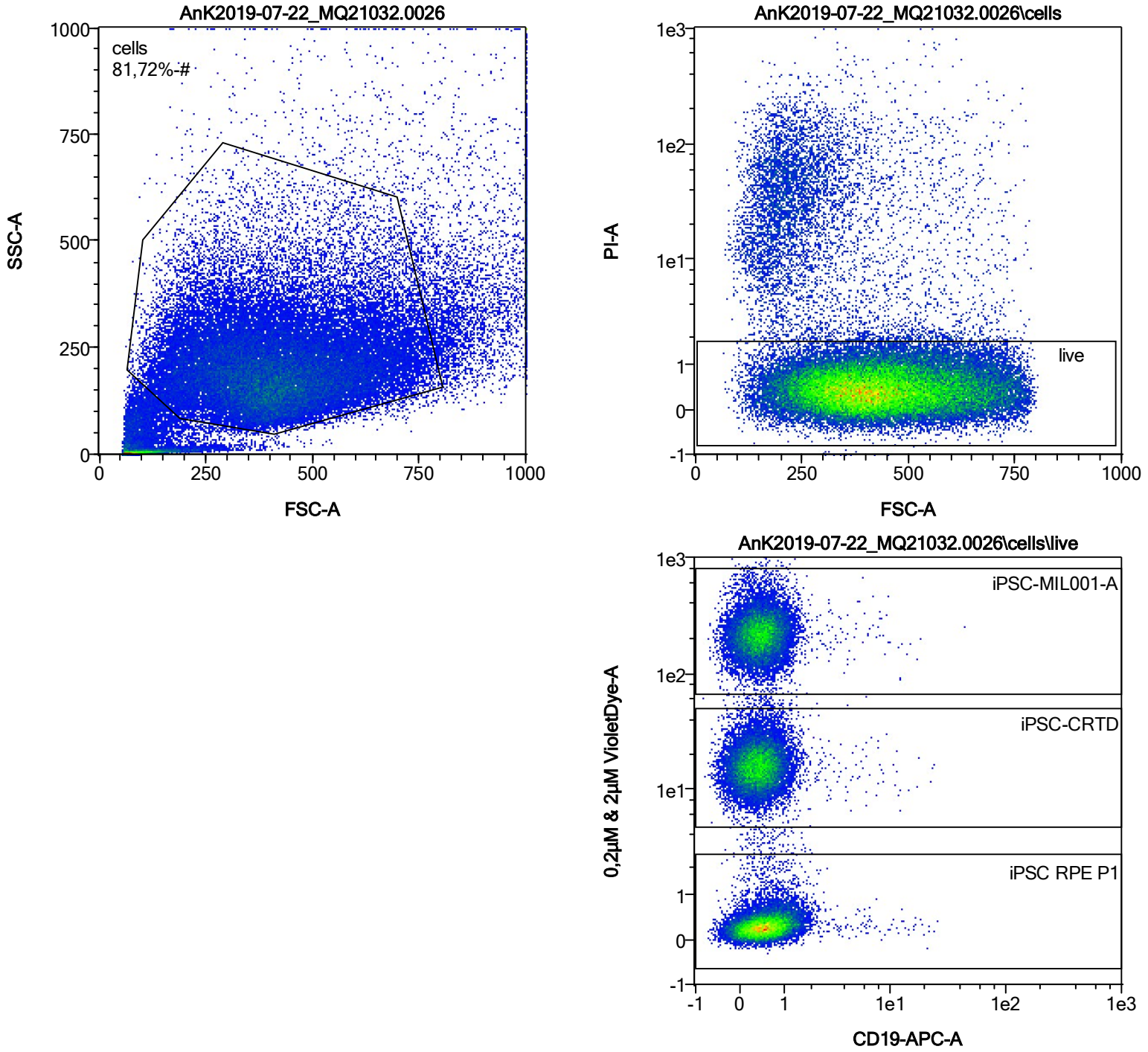

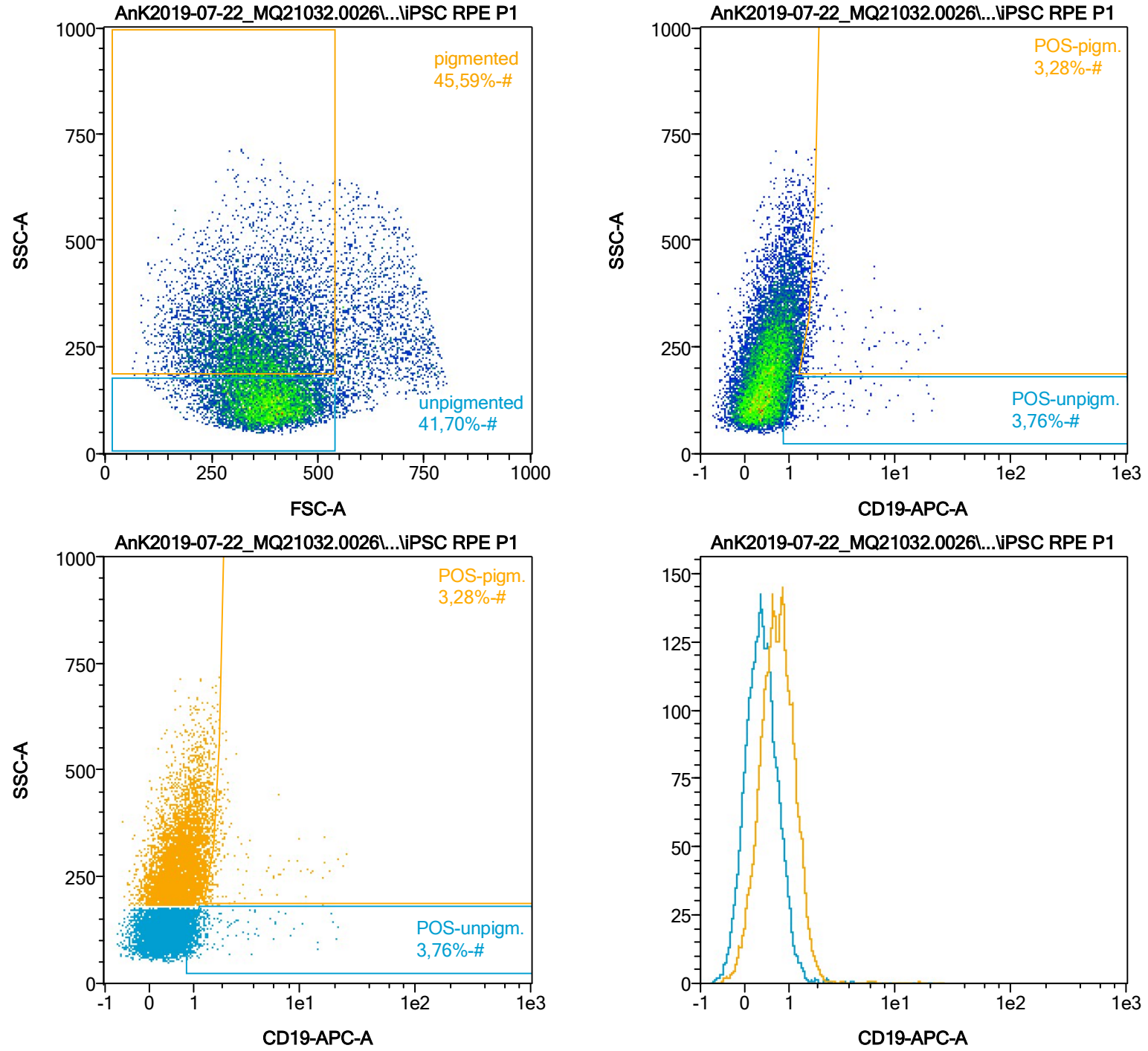

Name	%-#	CD19-APC-A Mean	CD19-APC-A Median
POS-unpigm.	3,76	2,06	1,20
POS-pigm.	3,28	3,01	1,81

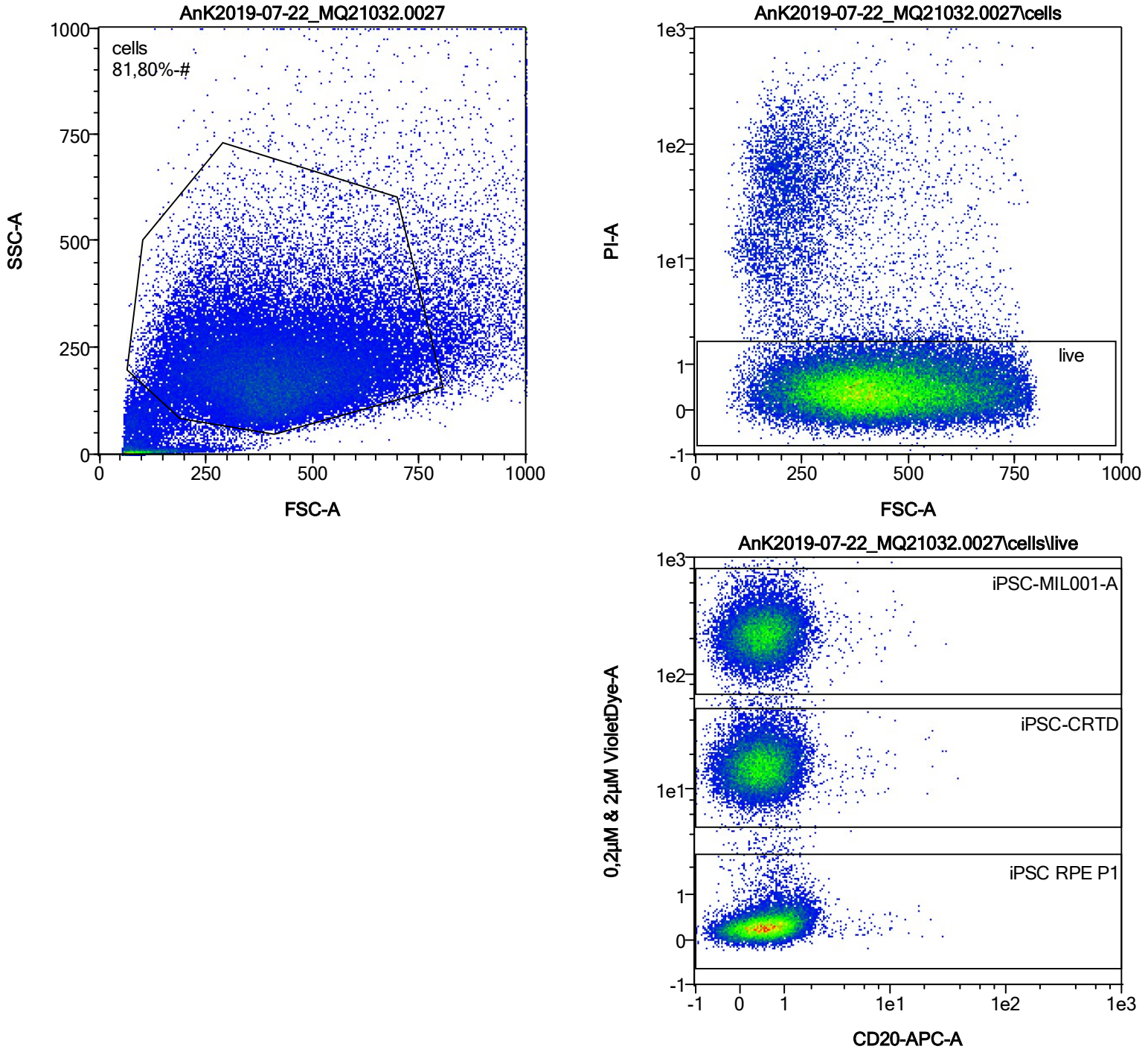

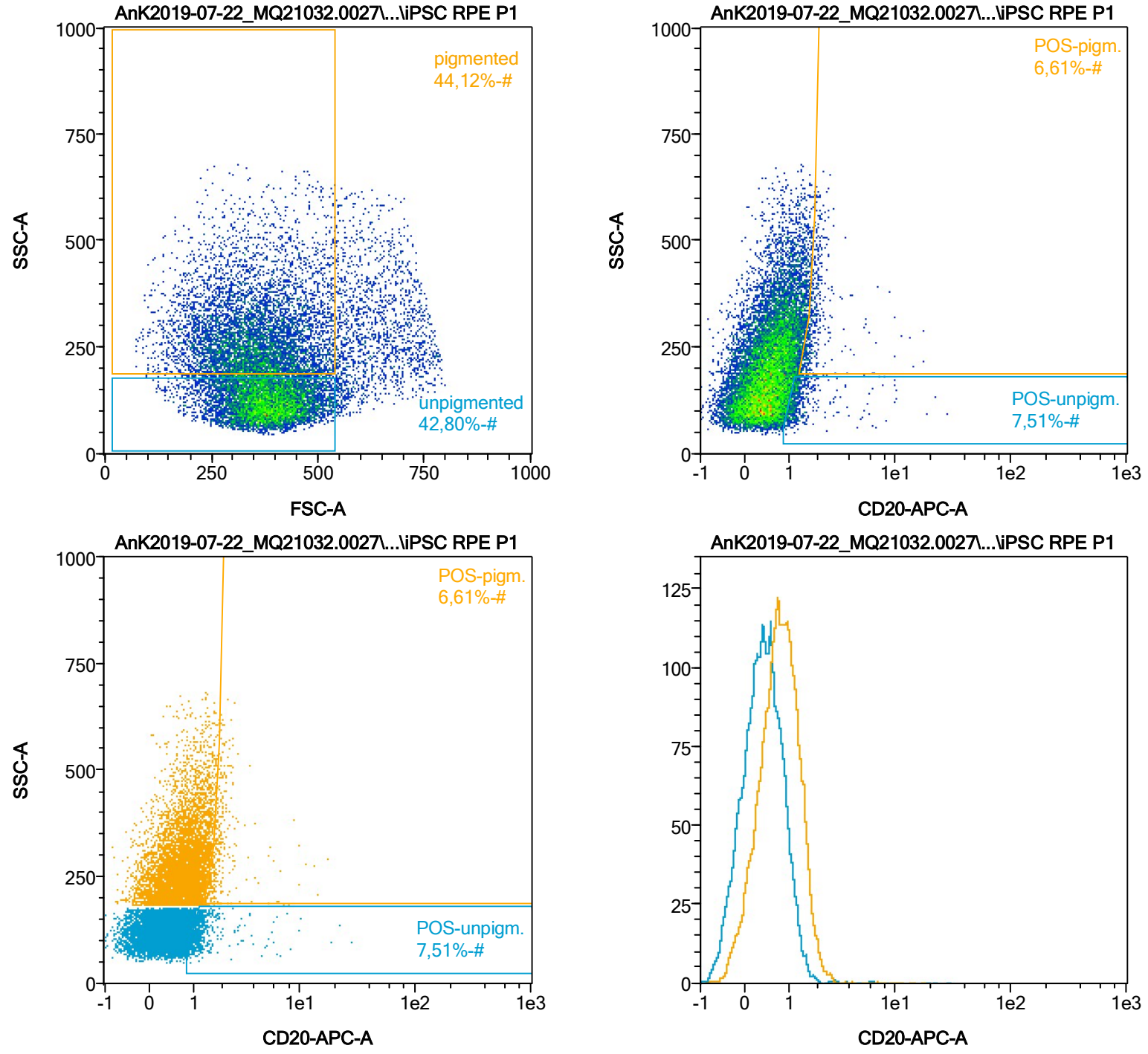

Name	%-#	CD20-APC-A Mean	CD20-APC-A Median
POS-unpigm.	7,51	1,59	1,20
POS-pigm.	6,61	2,11	1,78

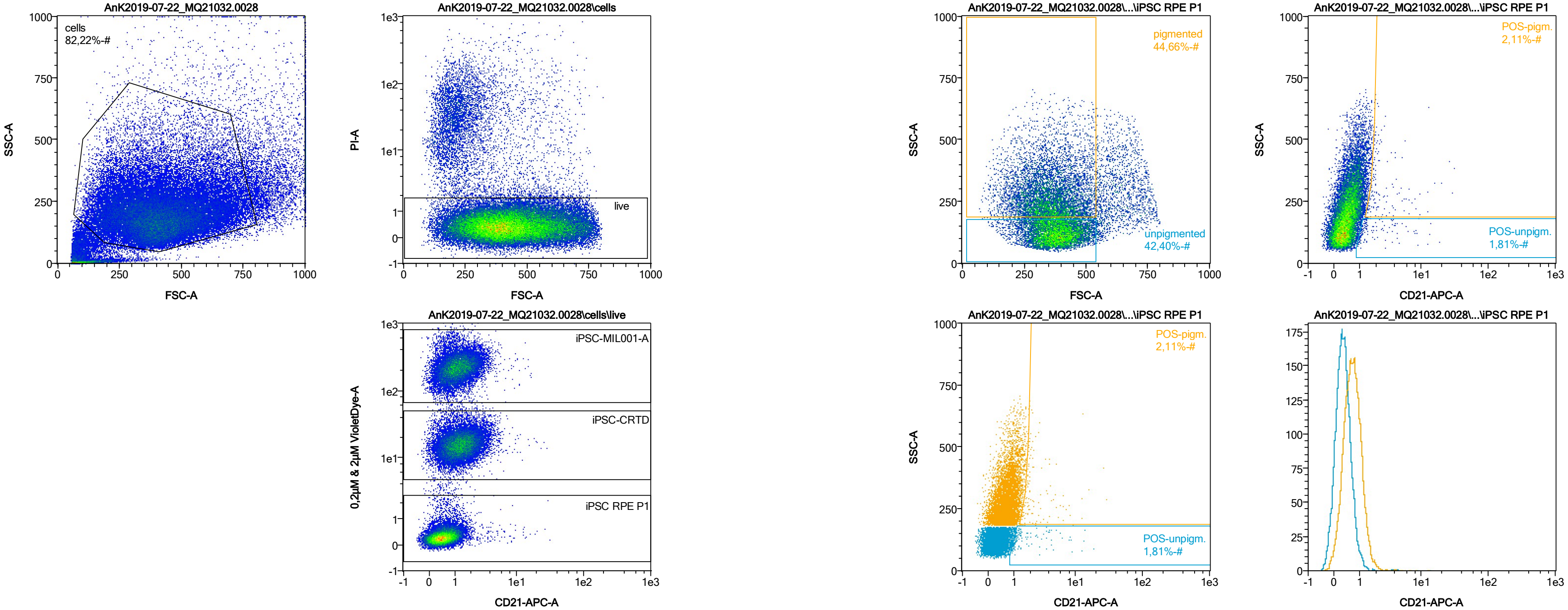

Name	%-#	CD21-APC-A Mean	CD21-APC-A Median
POS-unpigm.	1,81	2,42	1,25
POS-pigm.	2,11	3,13	1,94

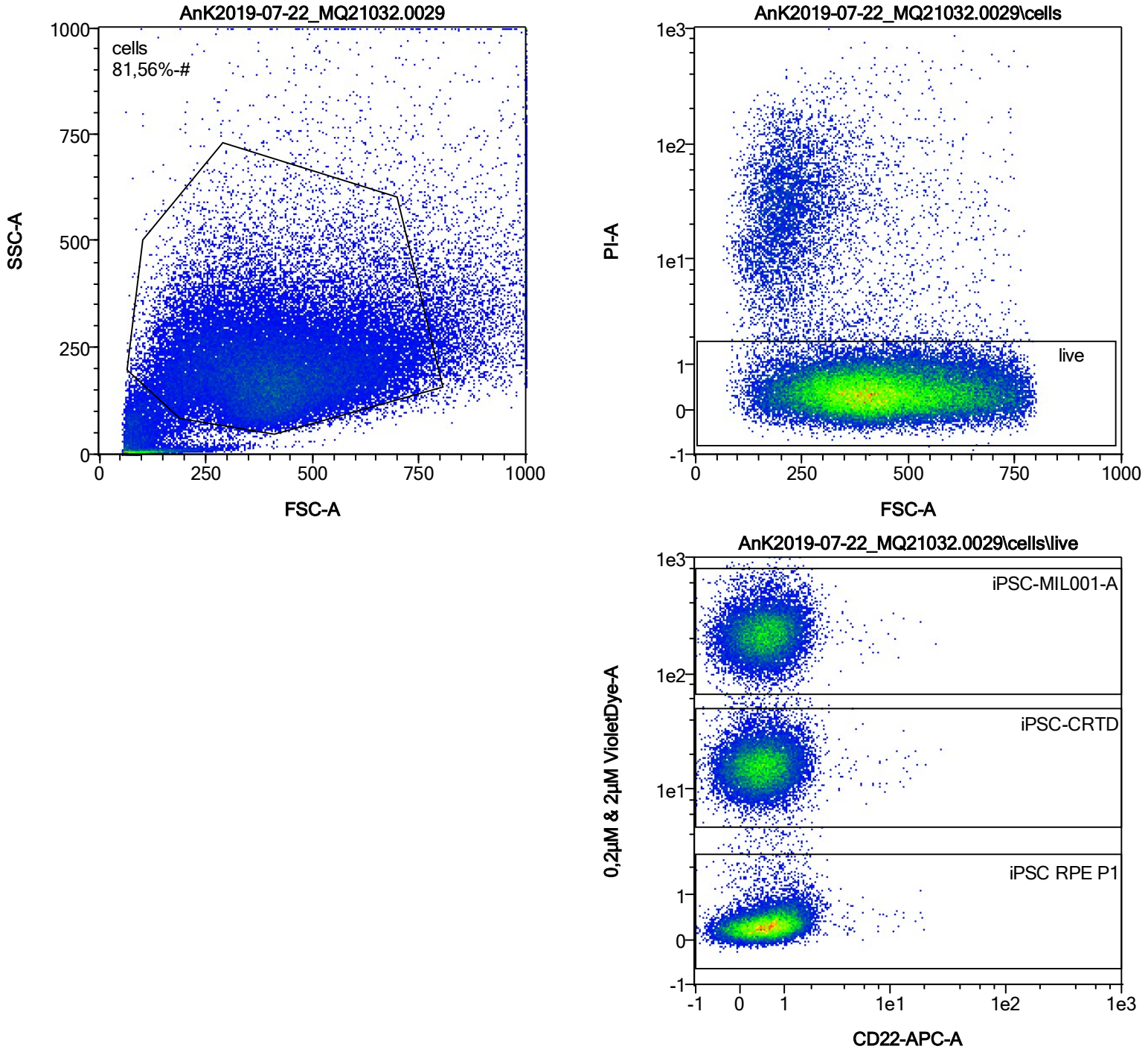

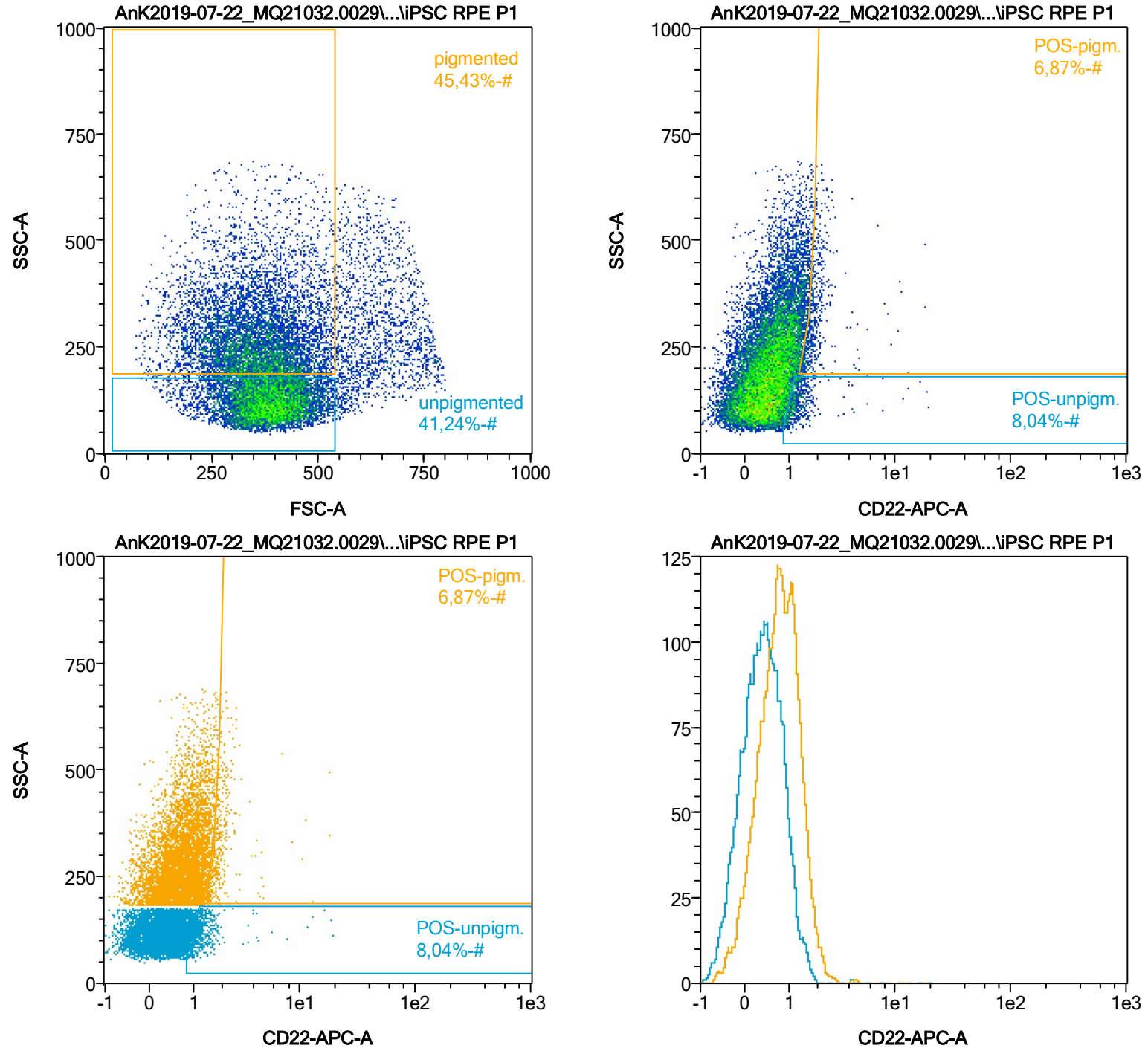

Name	%-#	CD22-APC-A Mean	CD22-APC-A Median
POS-unpigm.	8,04	1,45	1,18
POS-pigm.	6,87	2,04	1,80

Date:2024-06-14

Operator: Sebastian Knoebel

Version:2.13.0+54.g95abf0f4

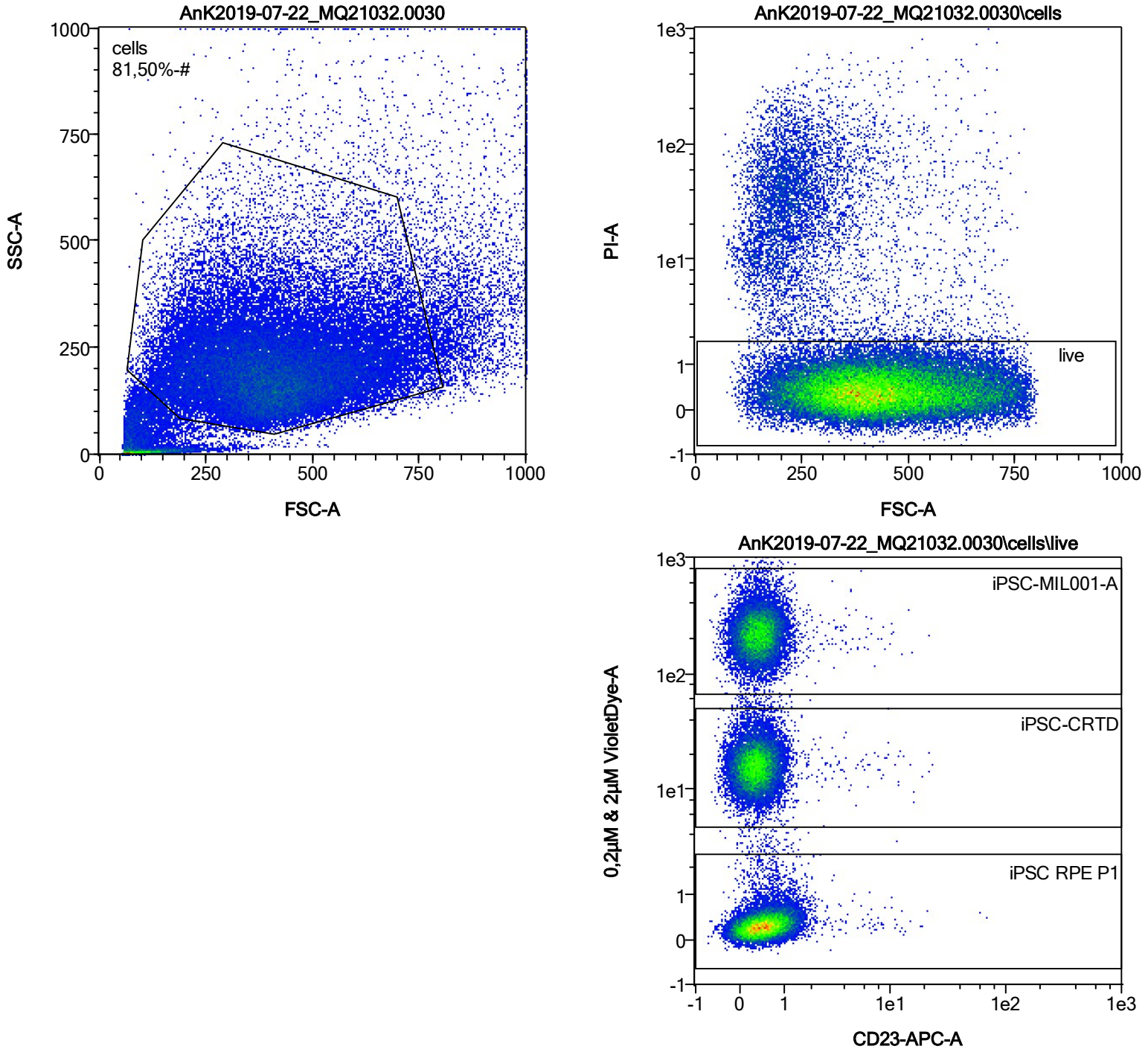

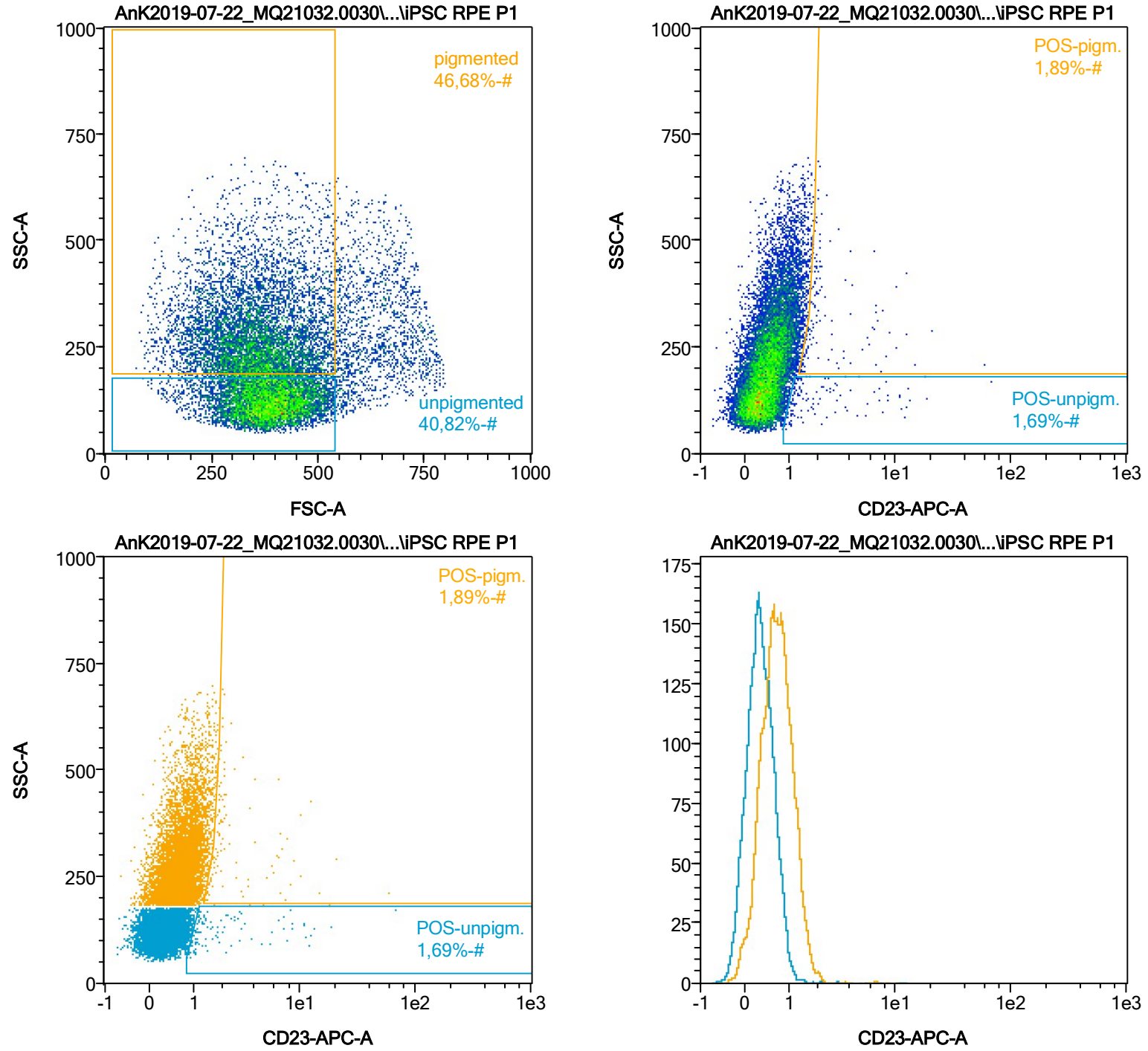

Name	%-#	CD23-APC-A Mean	CD23-APC-A Median
POS-unpigm.	1,69	3,41	1,34
POS-pigm.	1,89	3,44	1,92

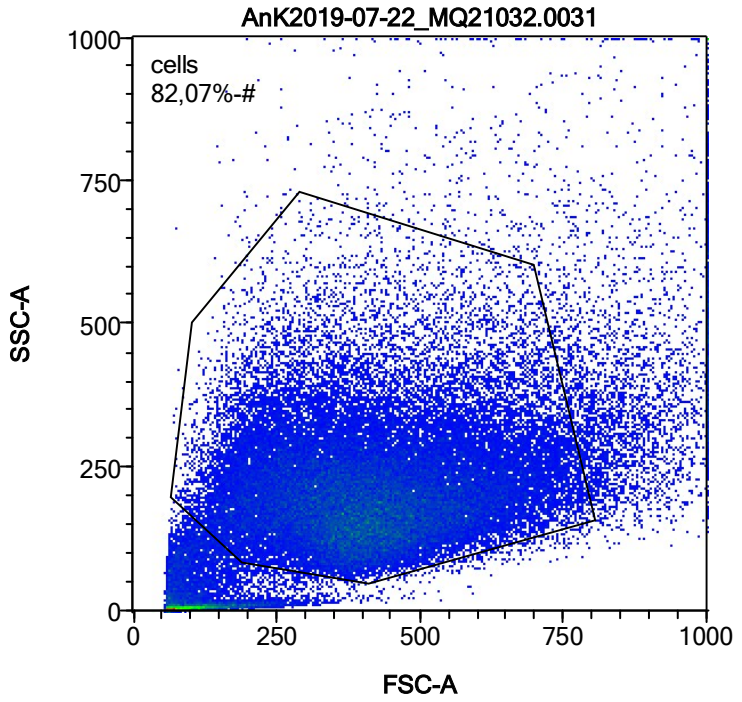

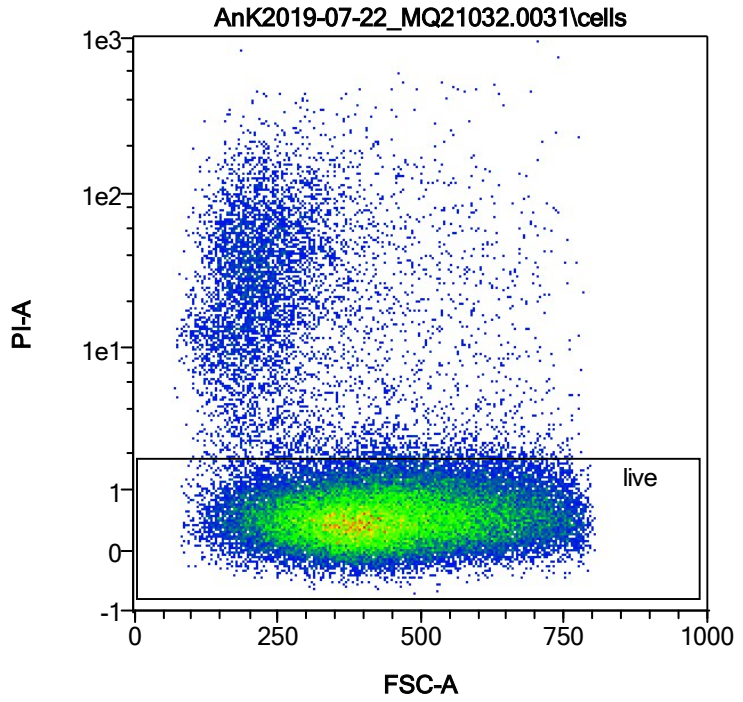

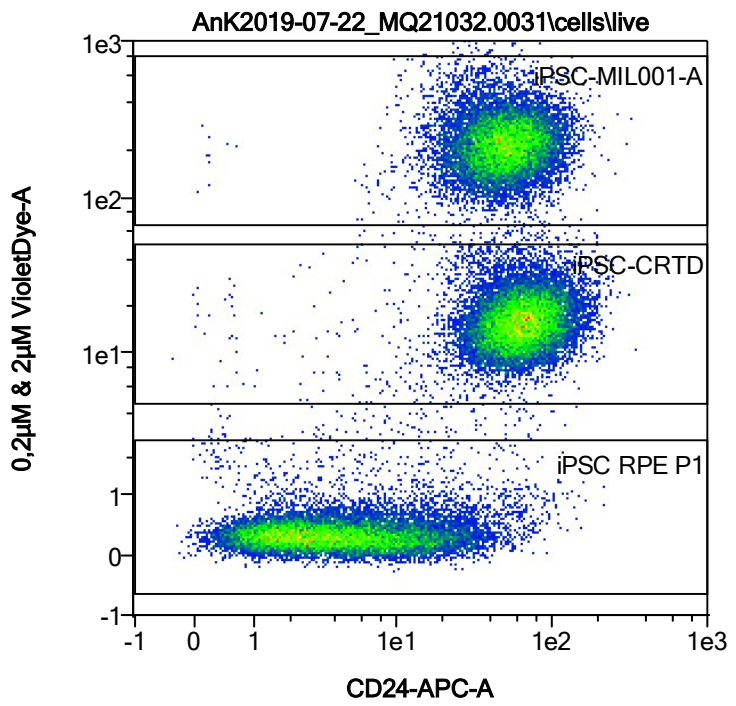

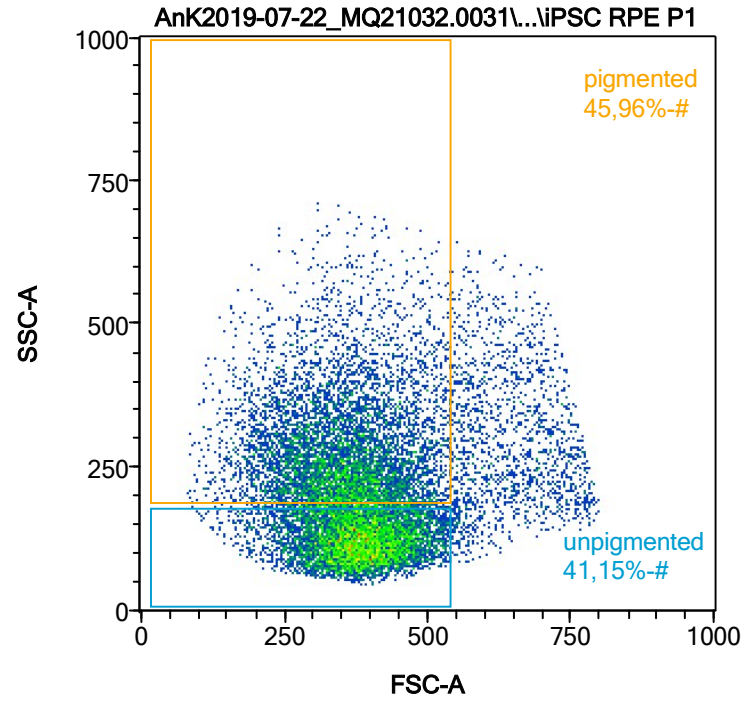

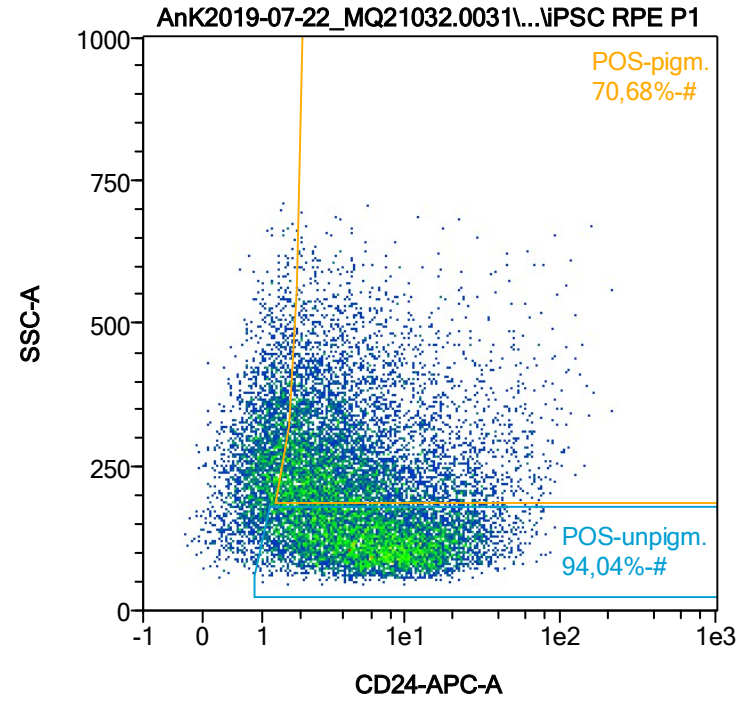

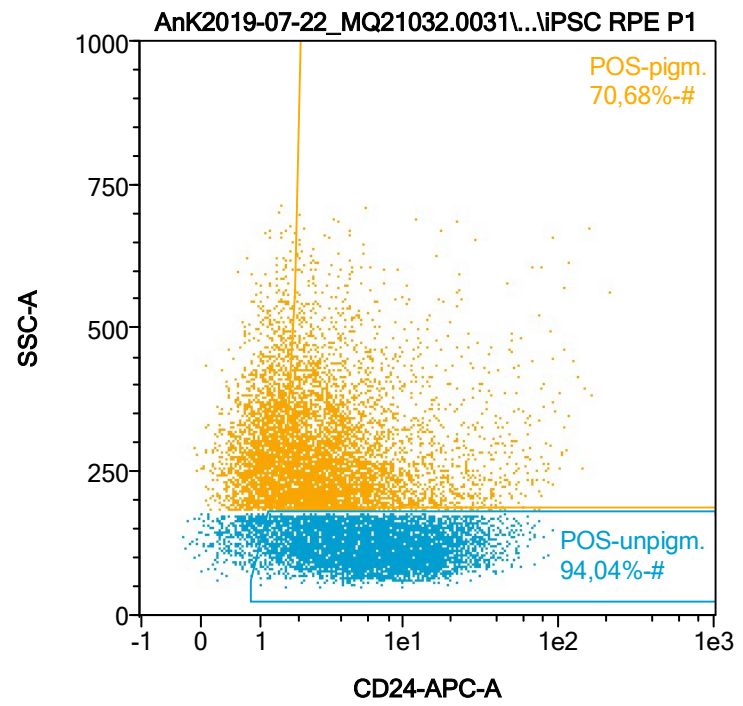

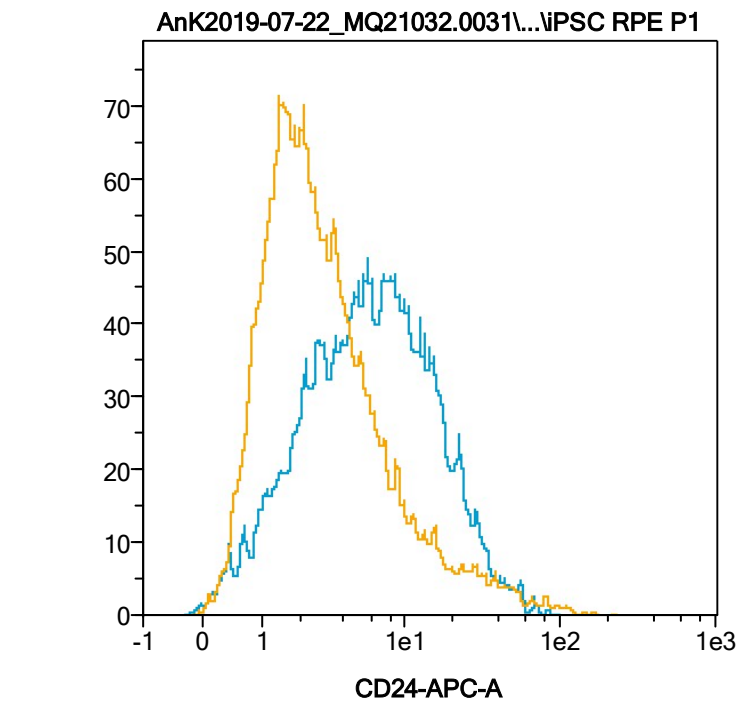

Name	%-#	CD24-APC-A Mean	CD24-APC-A Median
POS-unpigm.	94,04	9,18	6,36
POS-pigm.	70,68	7,13	3,40

Date:2024-06-14

Operator: Sebastian Knoebel

Version:2.13.0+54.g95abf0f4

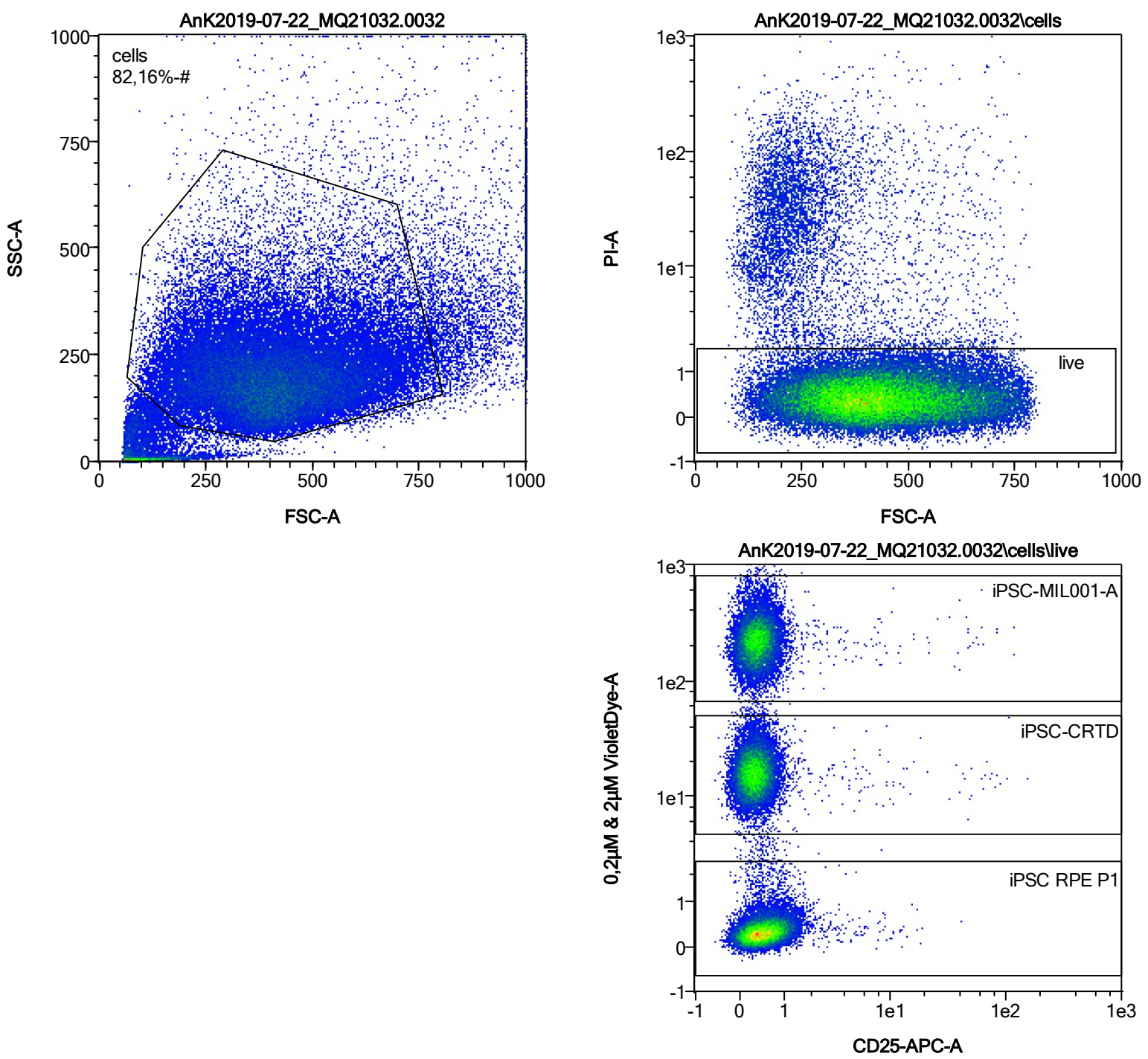

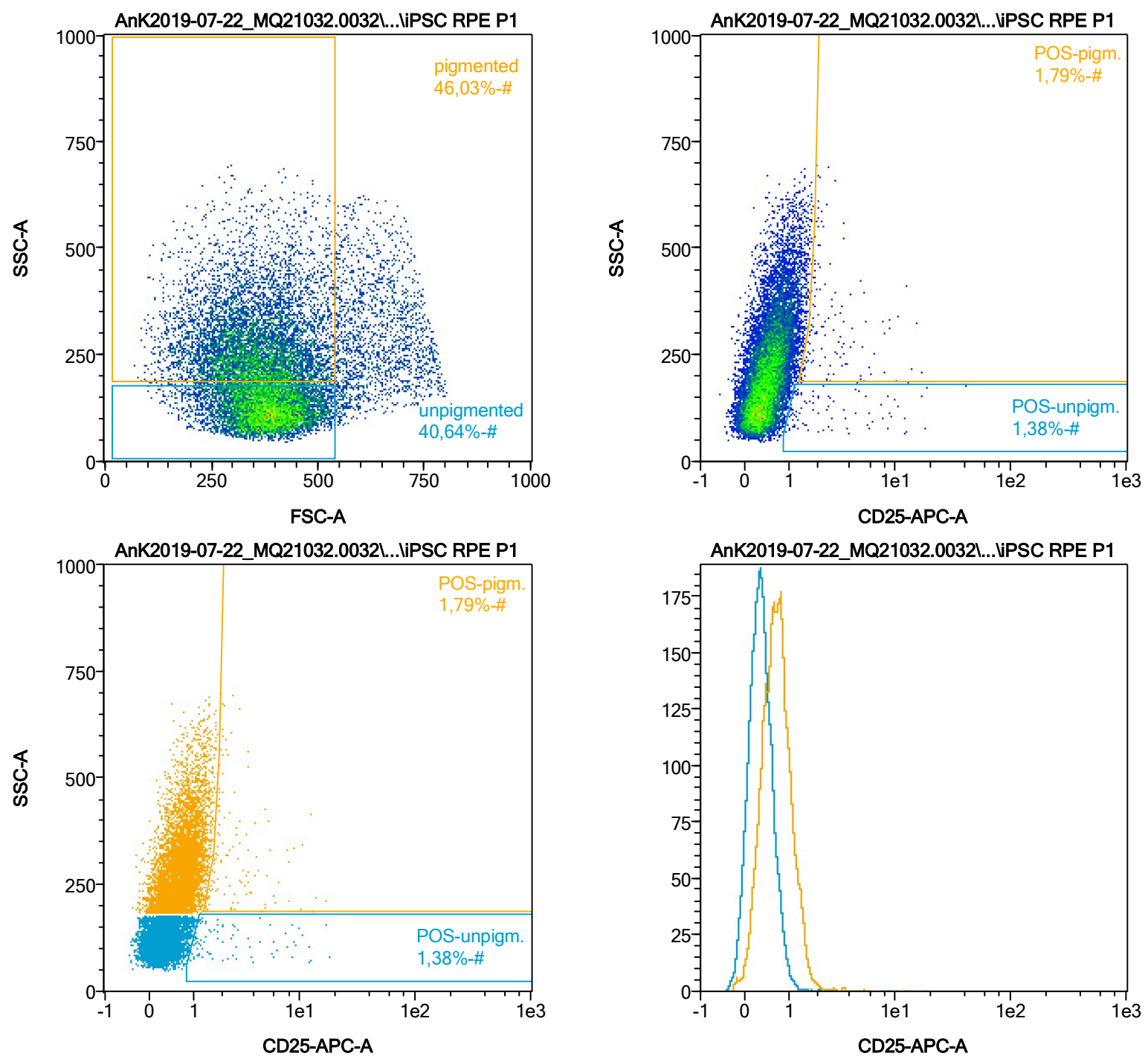

Name	%-#	CD25-APC-A Mean	CD25-APC-A Median
POS-unpigm.	1,38	3,38	1,66
POS-pigm.	1,79	3,18	2,17

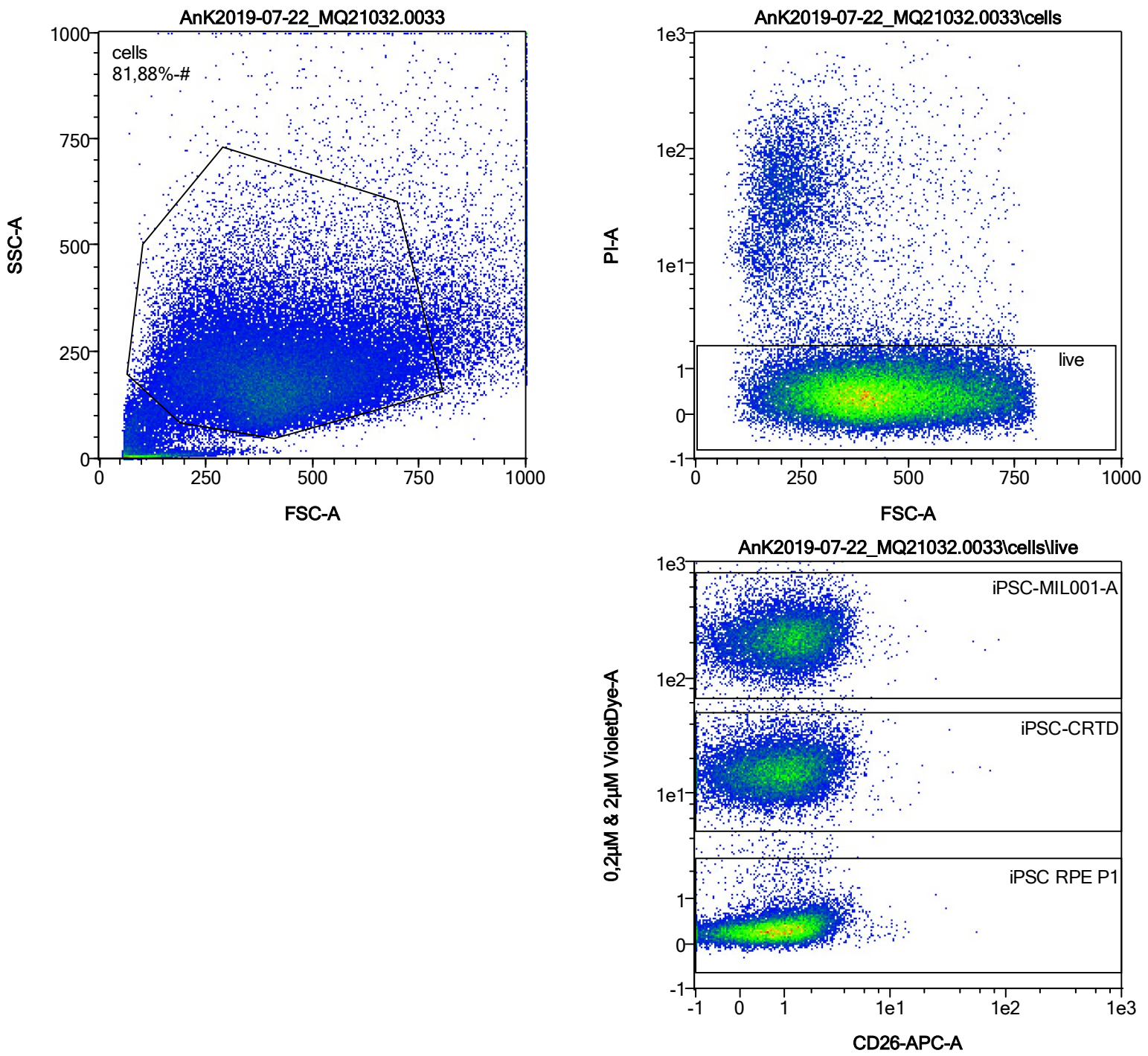

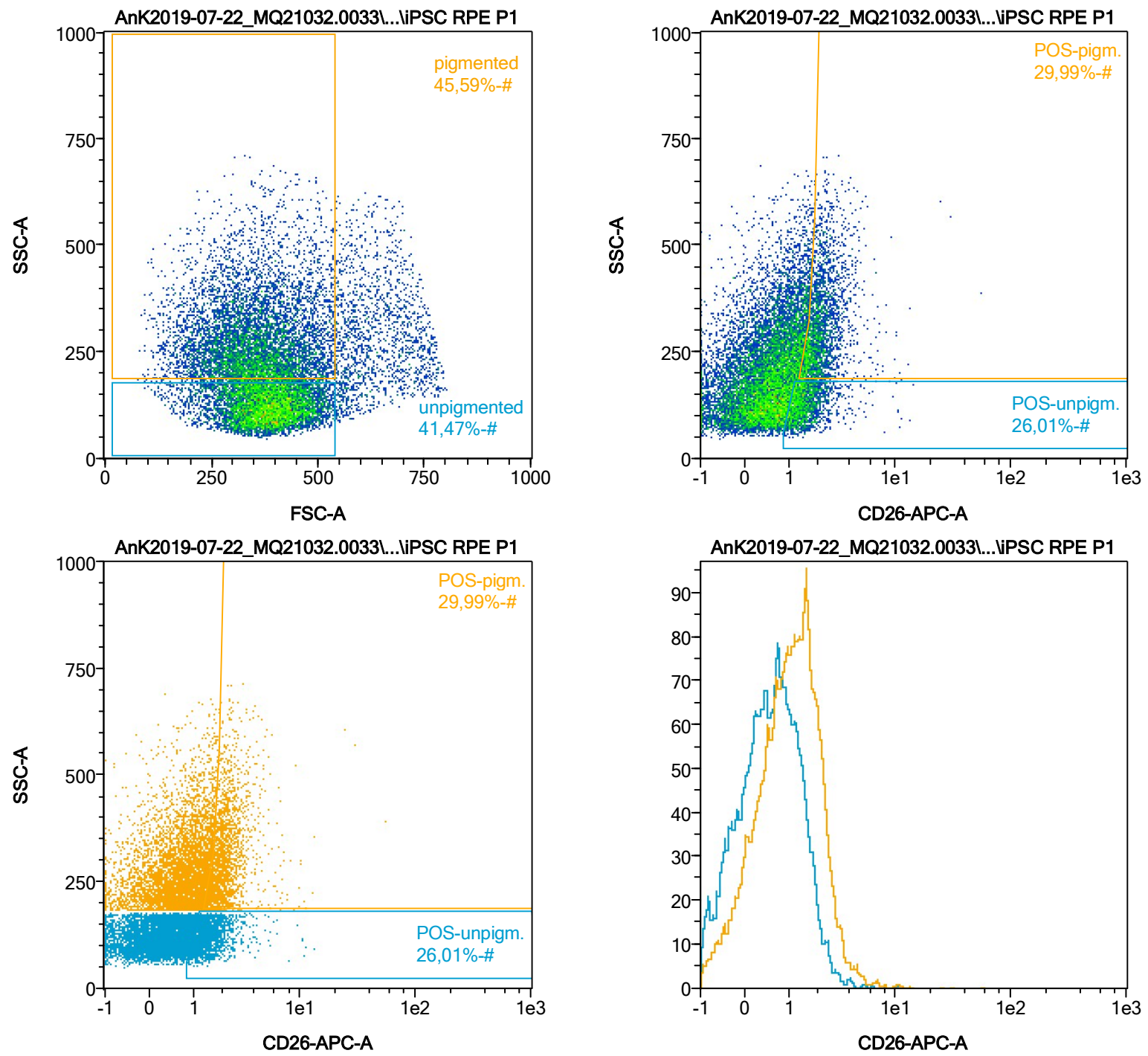

Name	%-#	CD26-APC-A Mean	CD26-APC-A Median
POS-unpigm.	26,01	1,60	1,41
POS-pigm.	29,99	2,32	2,03

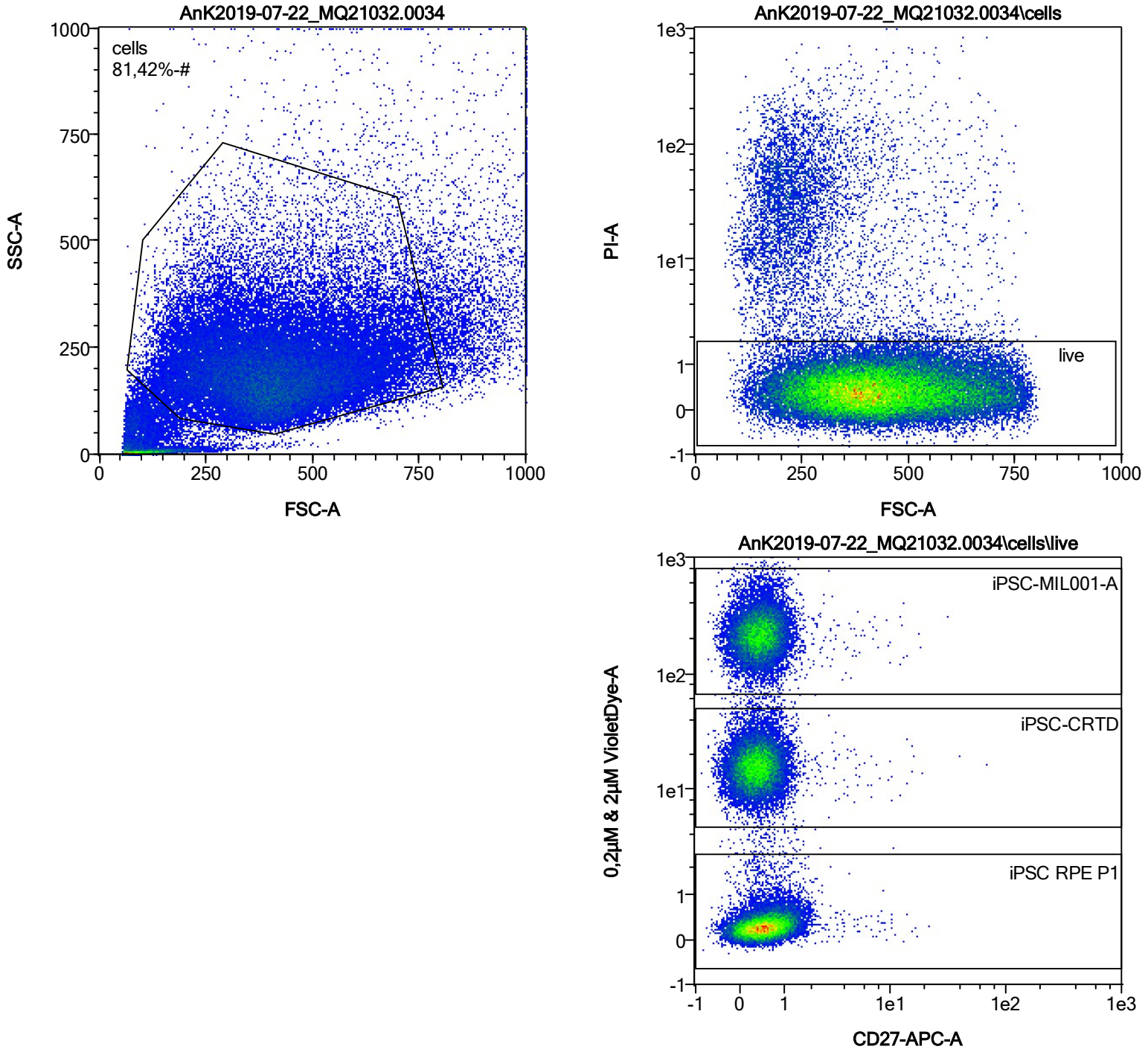

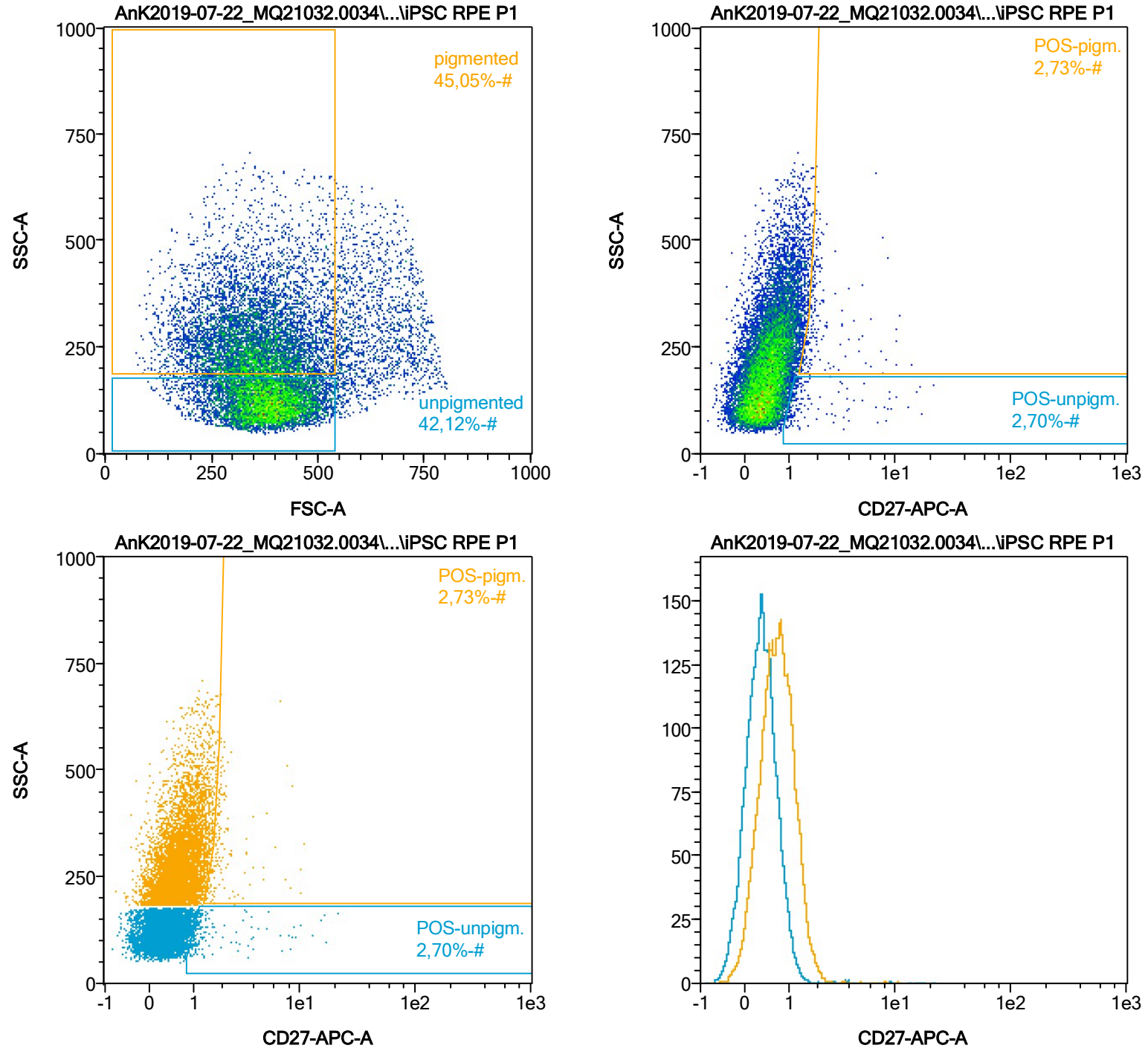

Name	%-#	CD27-APC-A Mean	CD27-APC-A Median
POS-unpigm.	2,70	2,22	1,18
POS-pigm.	2,73	2,27	1,85

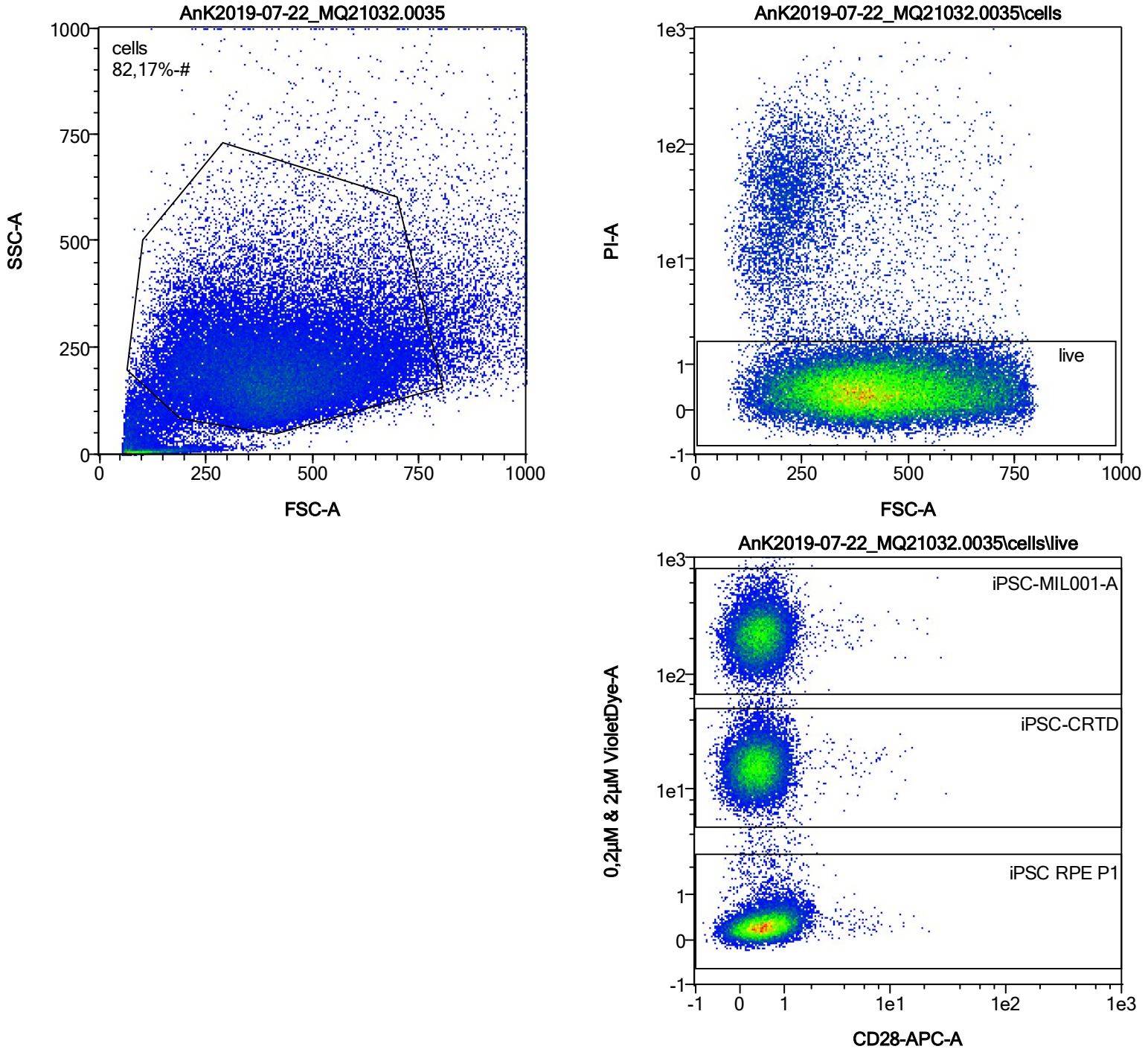

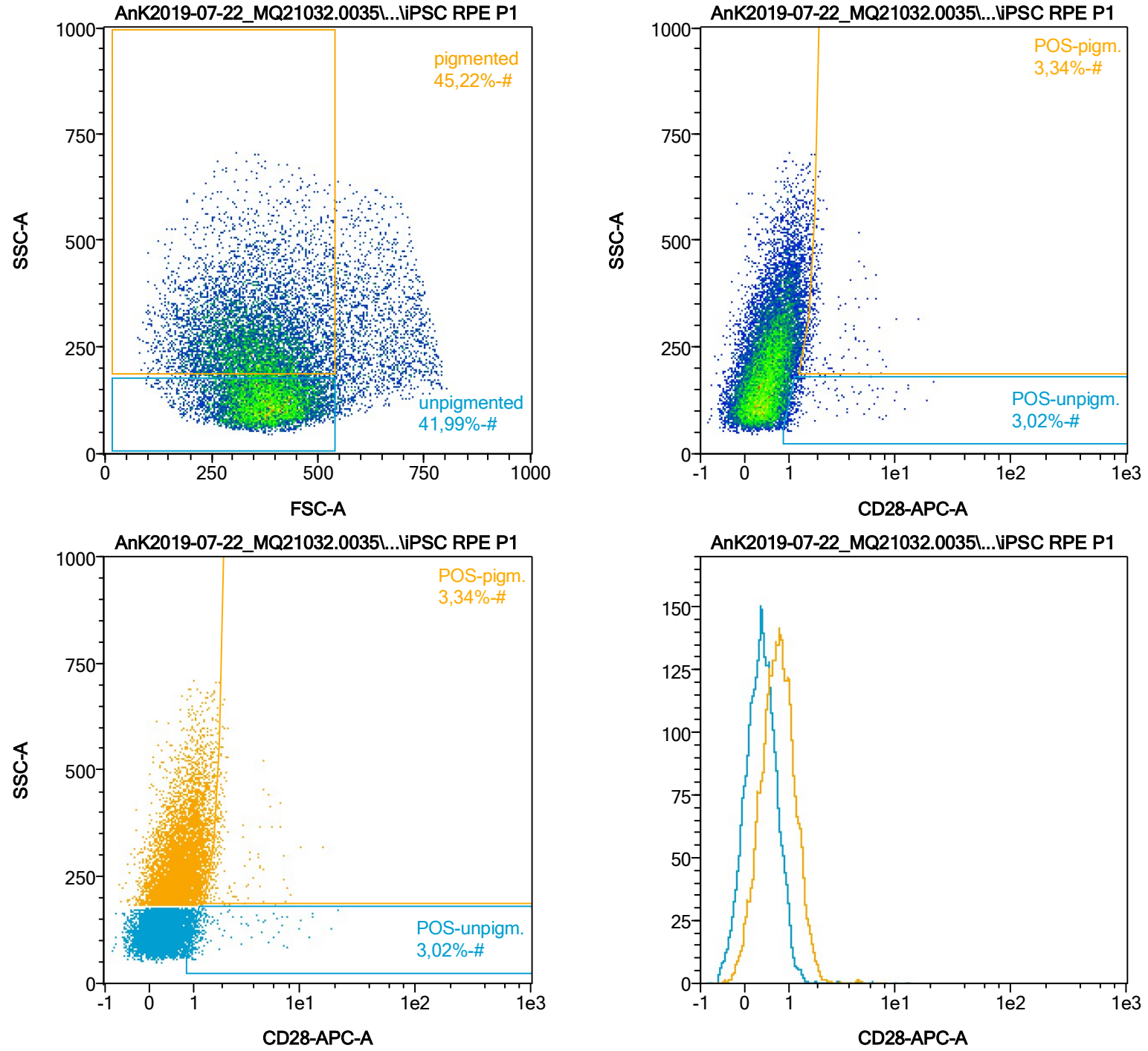

Name	%-#	CD28-APC-A Mean	CD28-APC-A Median
POS-unpigm.	3,02	2,14	1,19
POS-pigm.	3,34	2,37	1,85

Date:2024-06-14

Operator: Sebastian Knoebel

Version:2.13.0+54.g95abf0f4

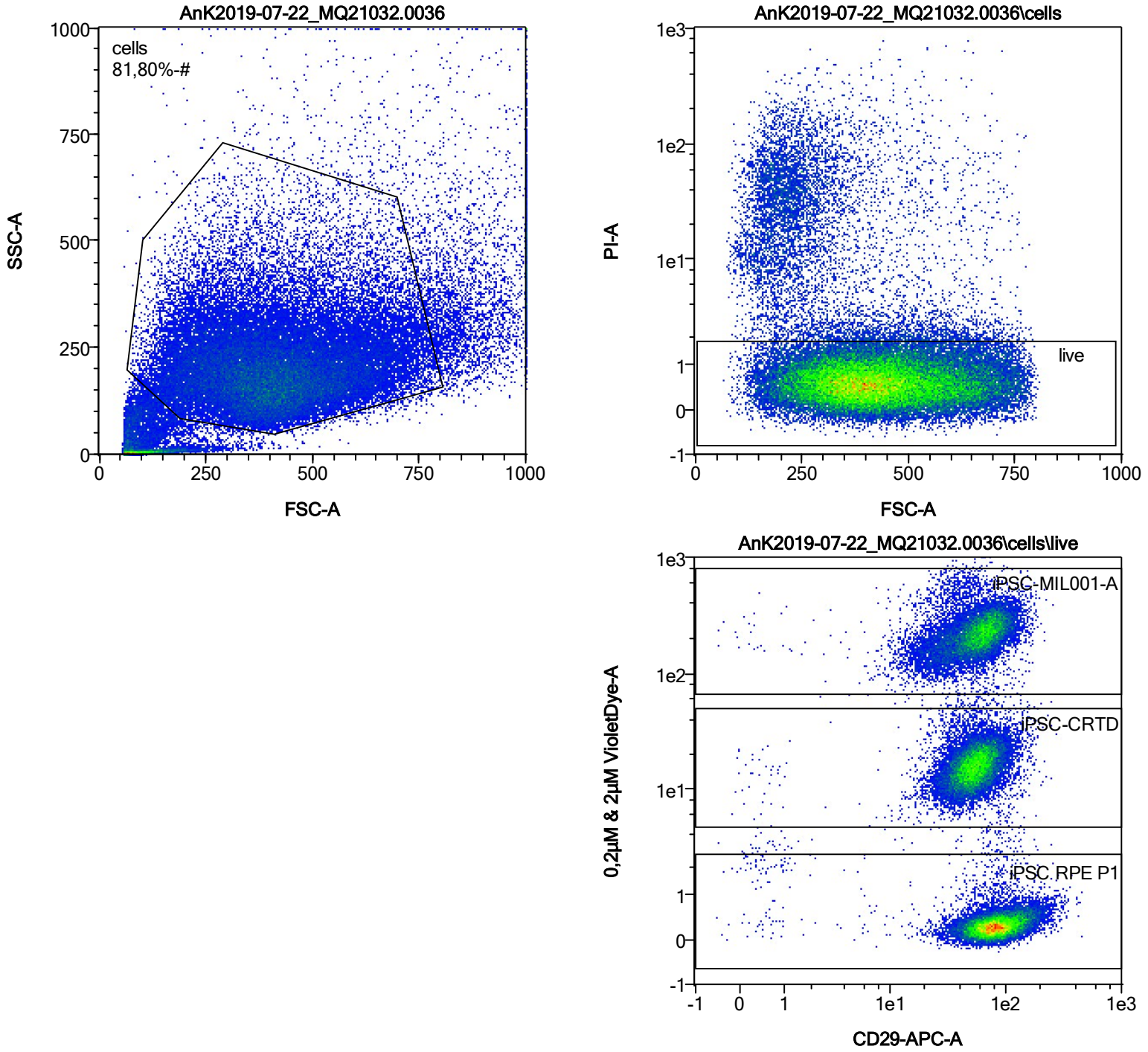

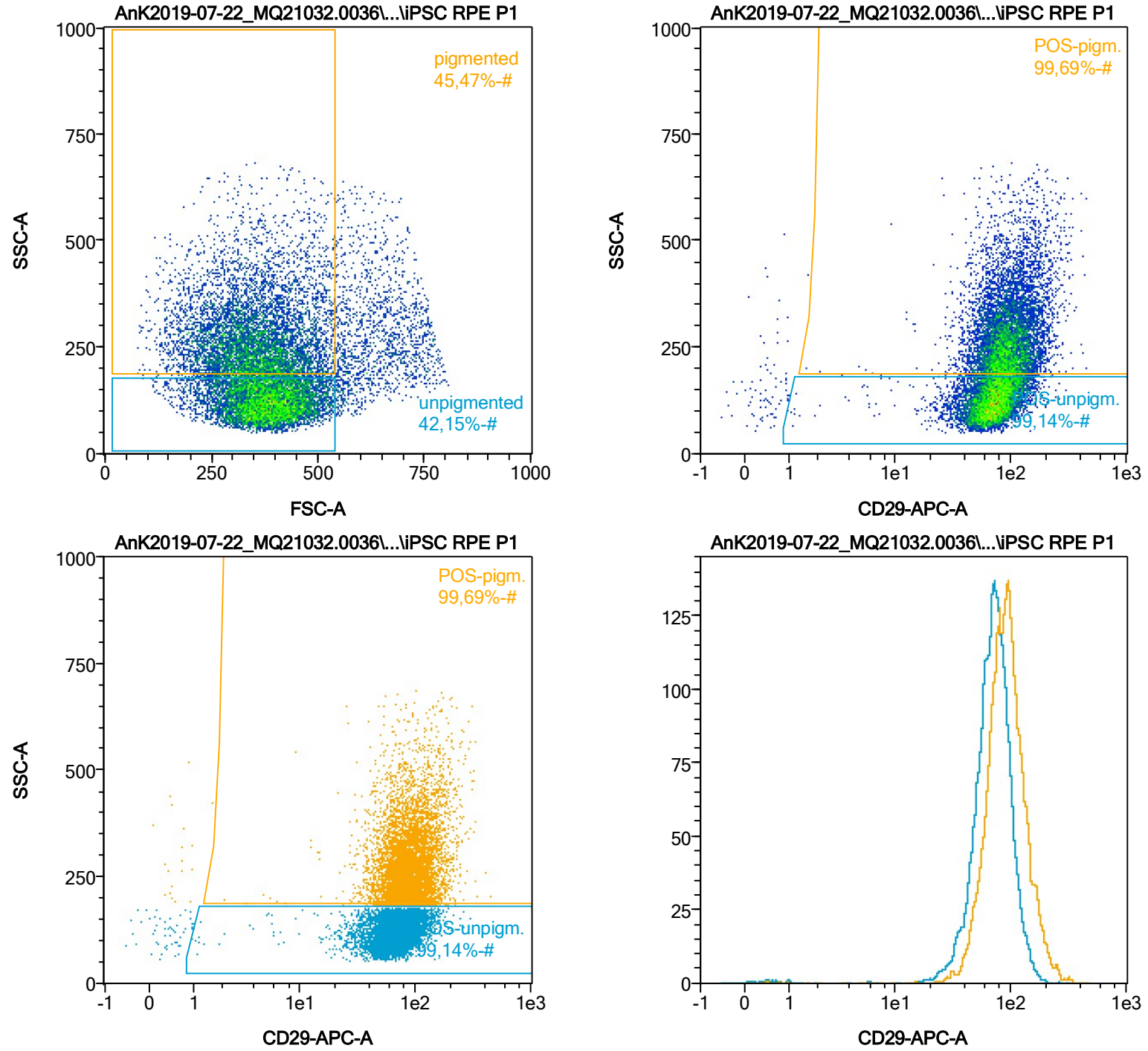

Name	%-#	CD29-APC-A Mean	CD29-APC-A Median
POS-unpigm.	99,14	72,40	69,94
POS-pigm.	99,69	96,14	89,02

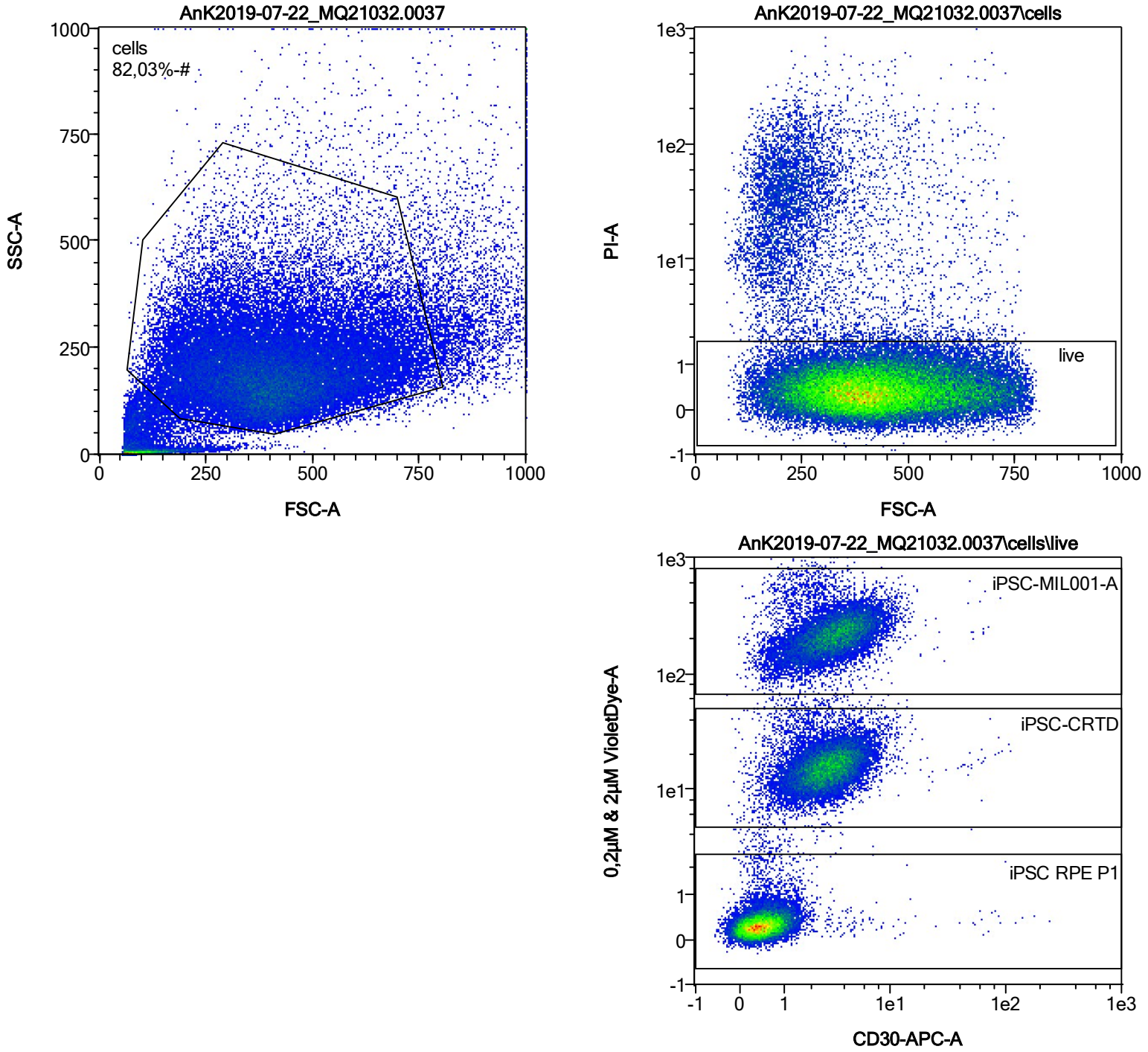

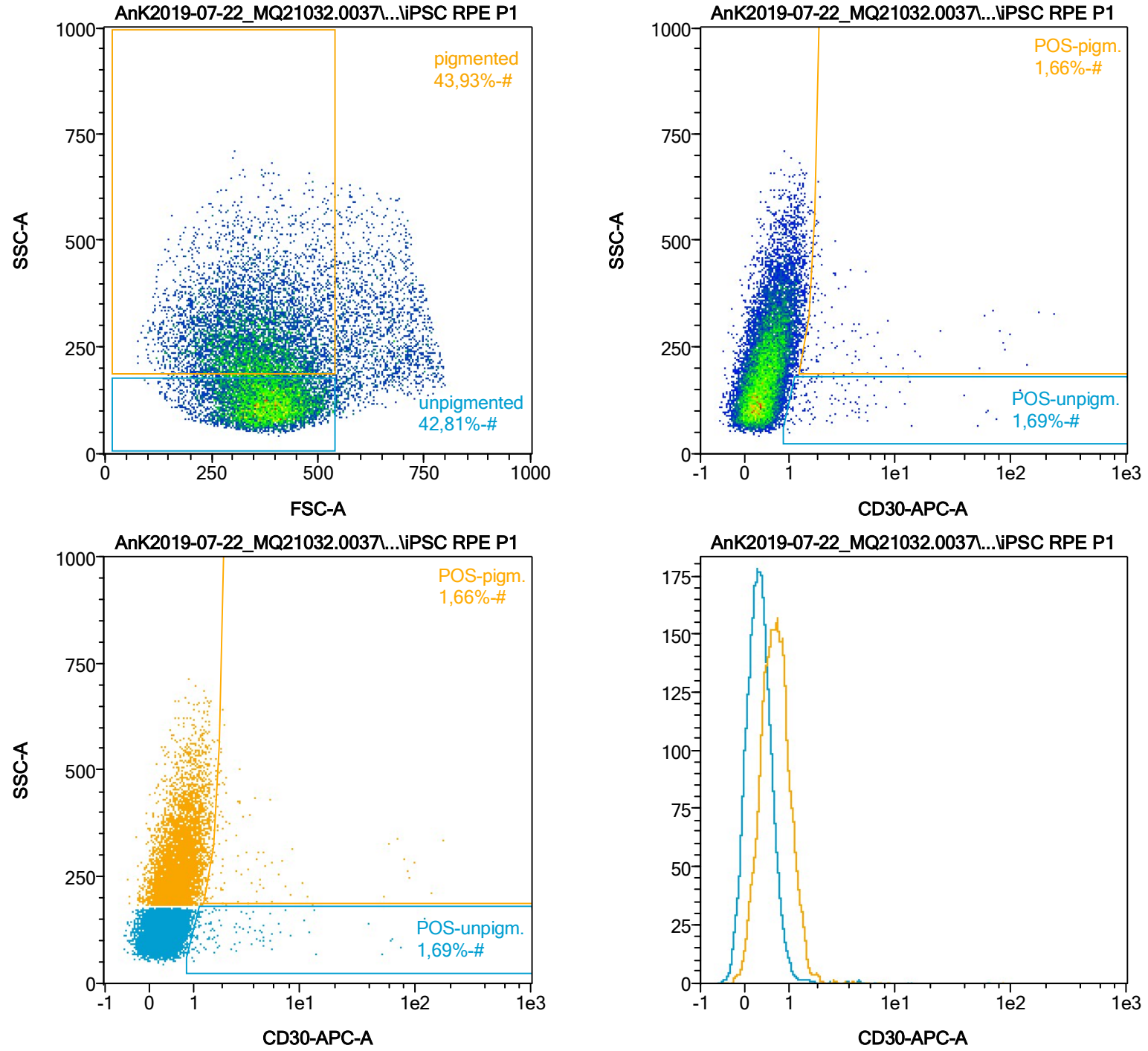

Name	%-#	CD30-APC-A Mean	CD30-APC-A Median
POS-unpigm.	1,69	6,30	1,69
POS-pigm.	1,66	10,29	2,04

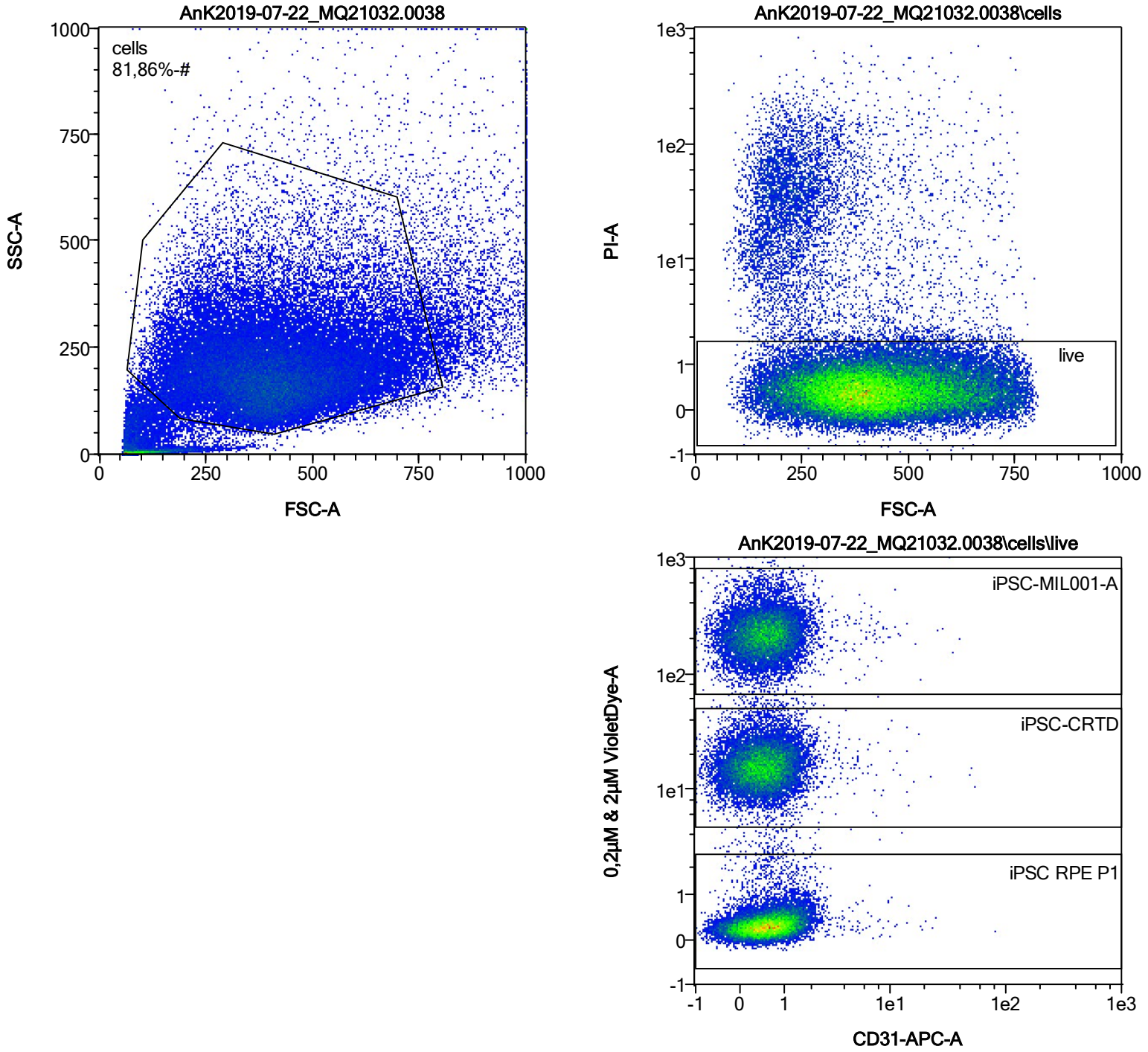

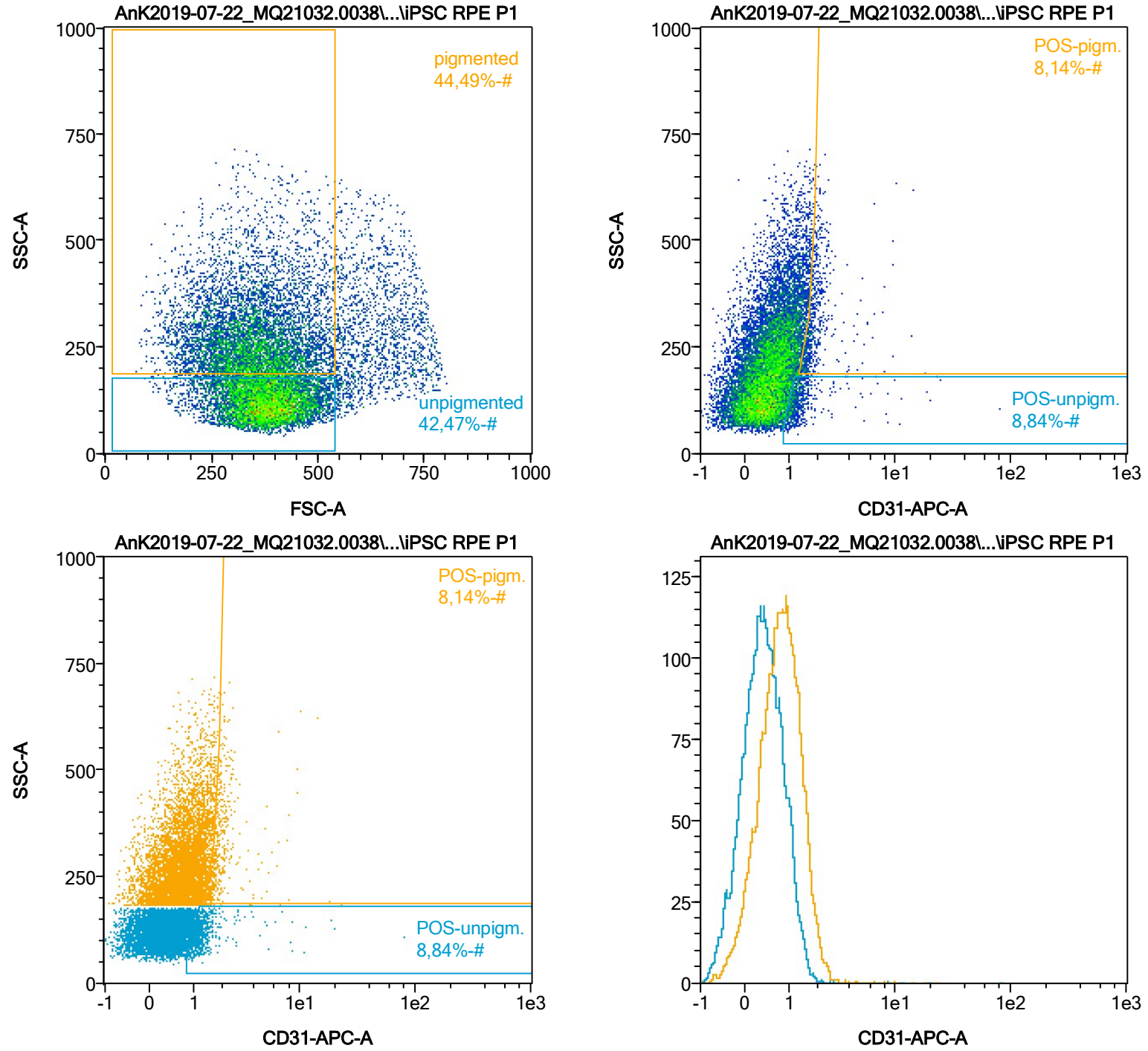

Name	%-#	CD31-APC-A Mean	CD31-APC-A Median
POS-unpigm.	8,84	1,59	1,18
POS-pigm.	8,14	2,09	1,79

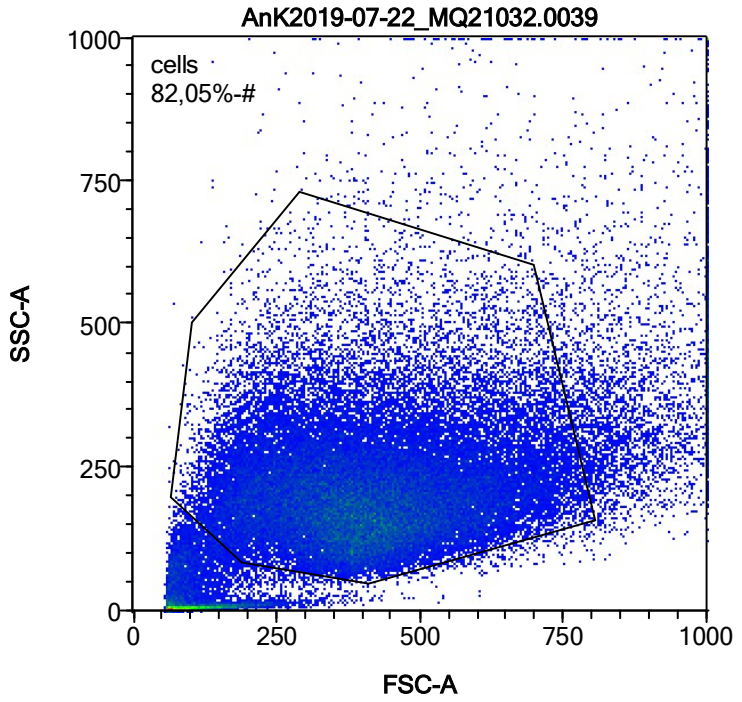

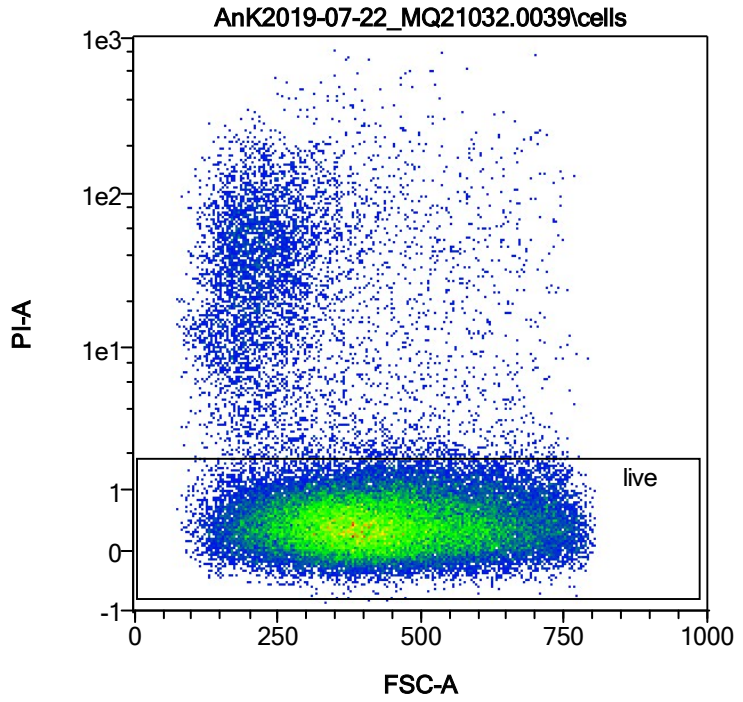

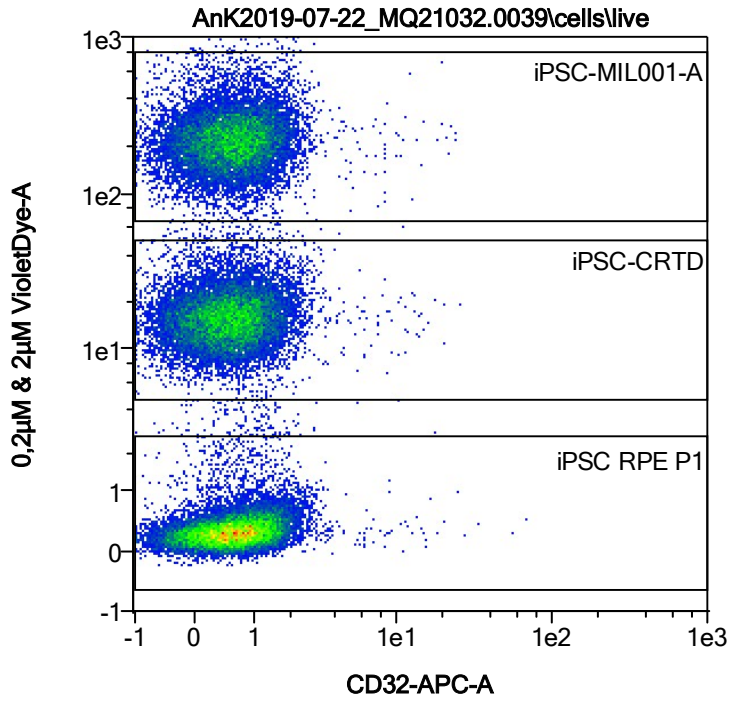

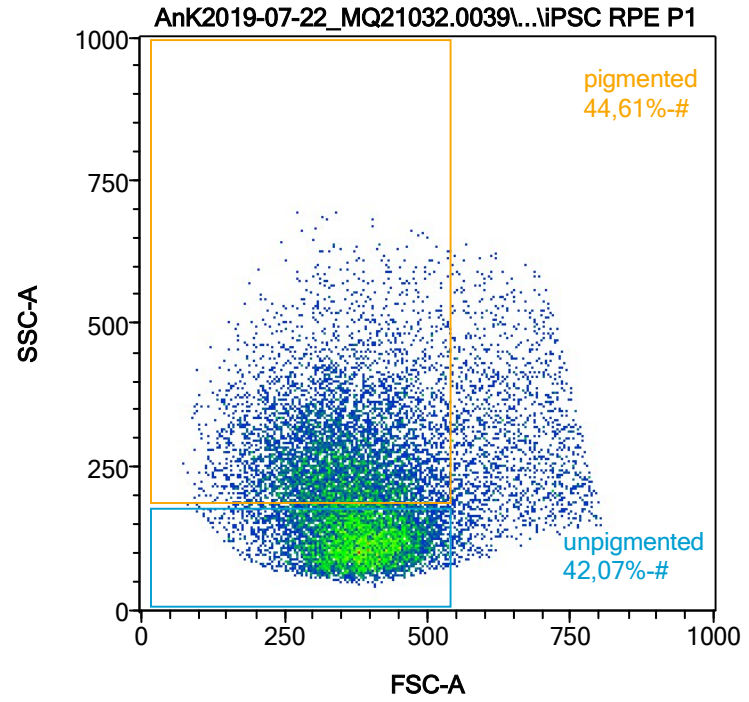

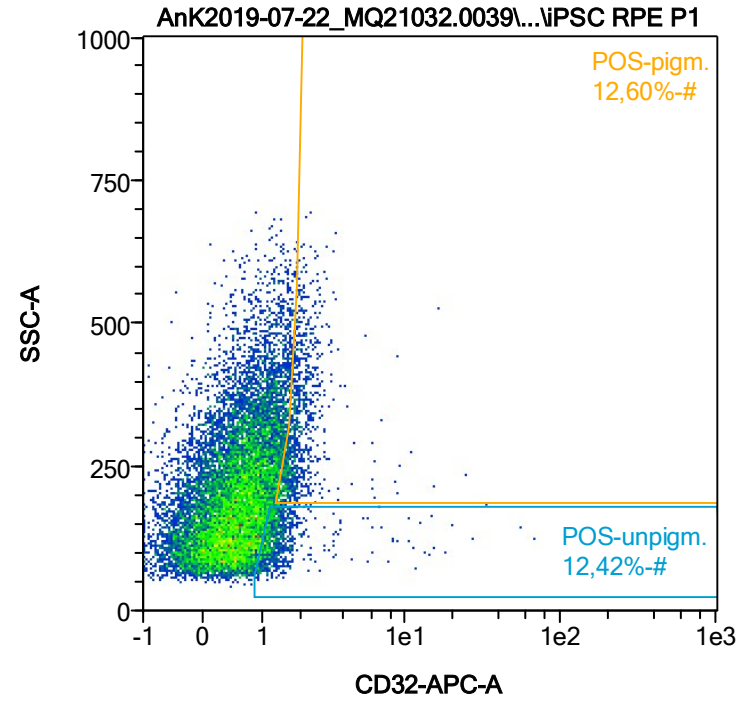

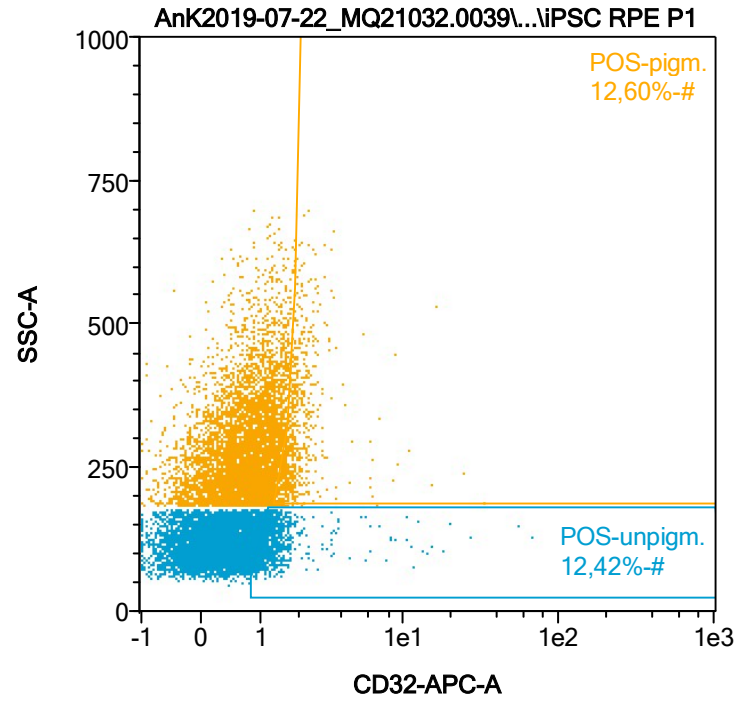

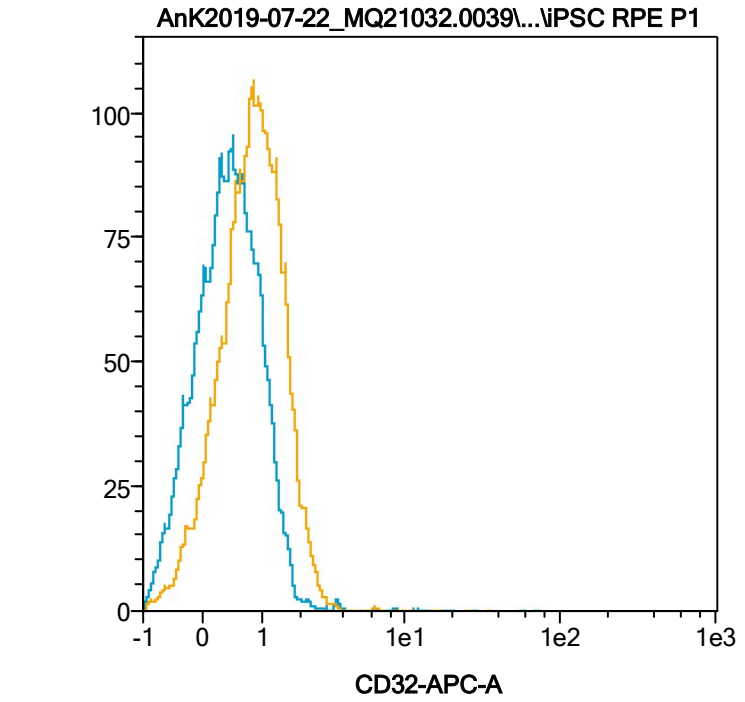

Name	%-#	CD32-APC-A Mean	CD32-APC-A Median
POS-unpigm.	12,42	1,77	1,25
POS-pigm.	12,60	2,08	1,82

Date:2024-06-14

Operator: Sebastian Knoebel

Version:2.13.0+54.g95abf0f4

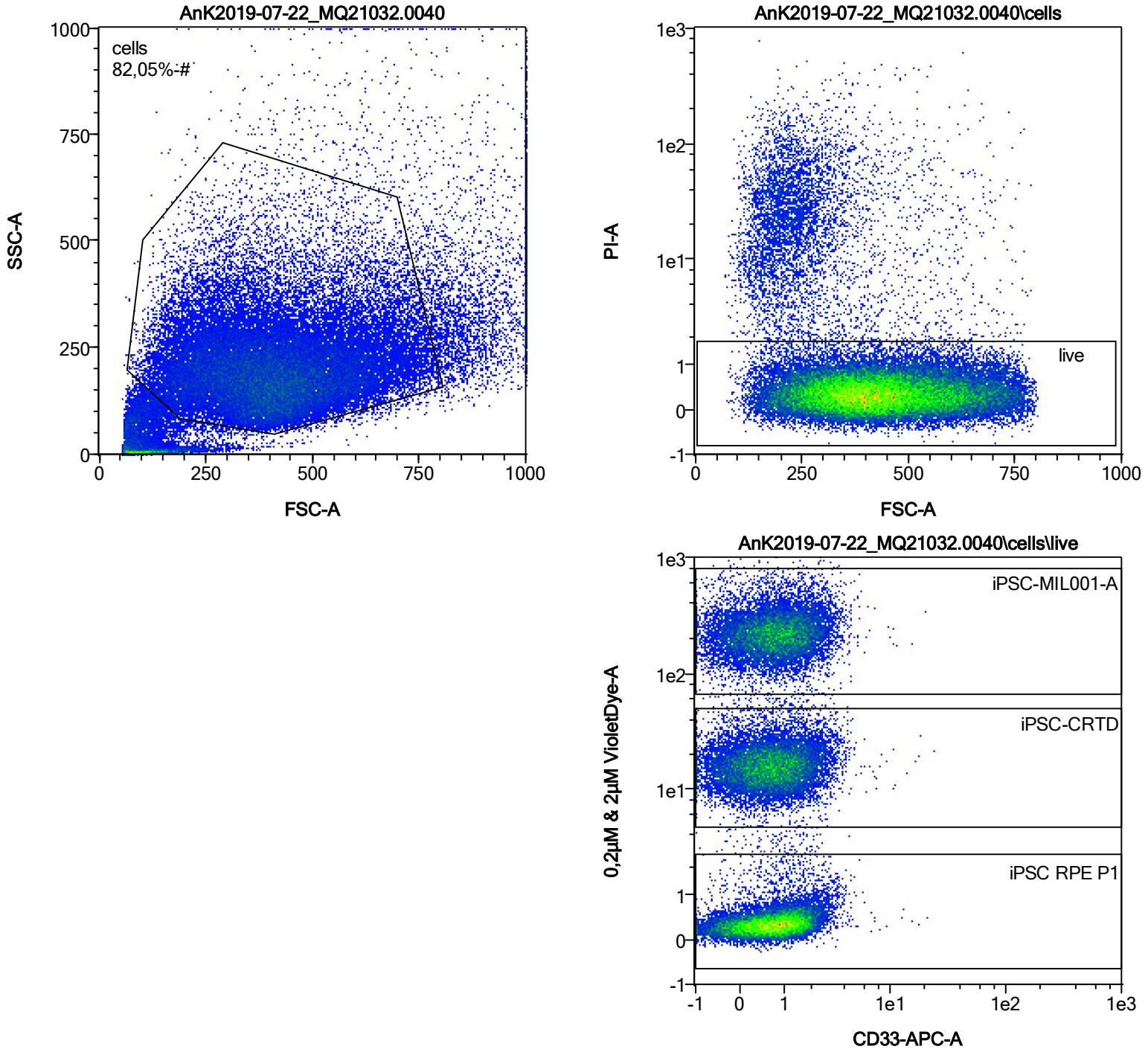

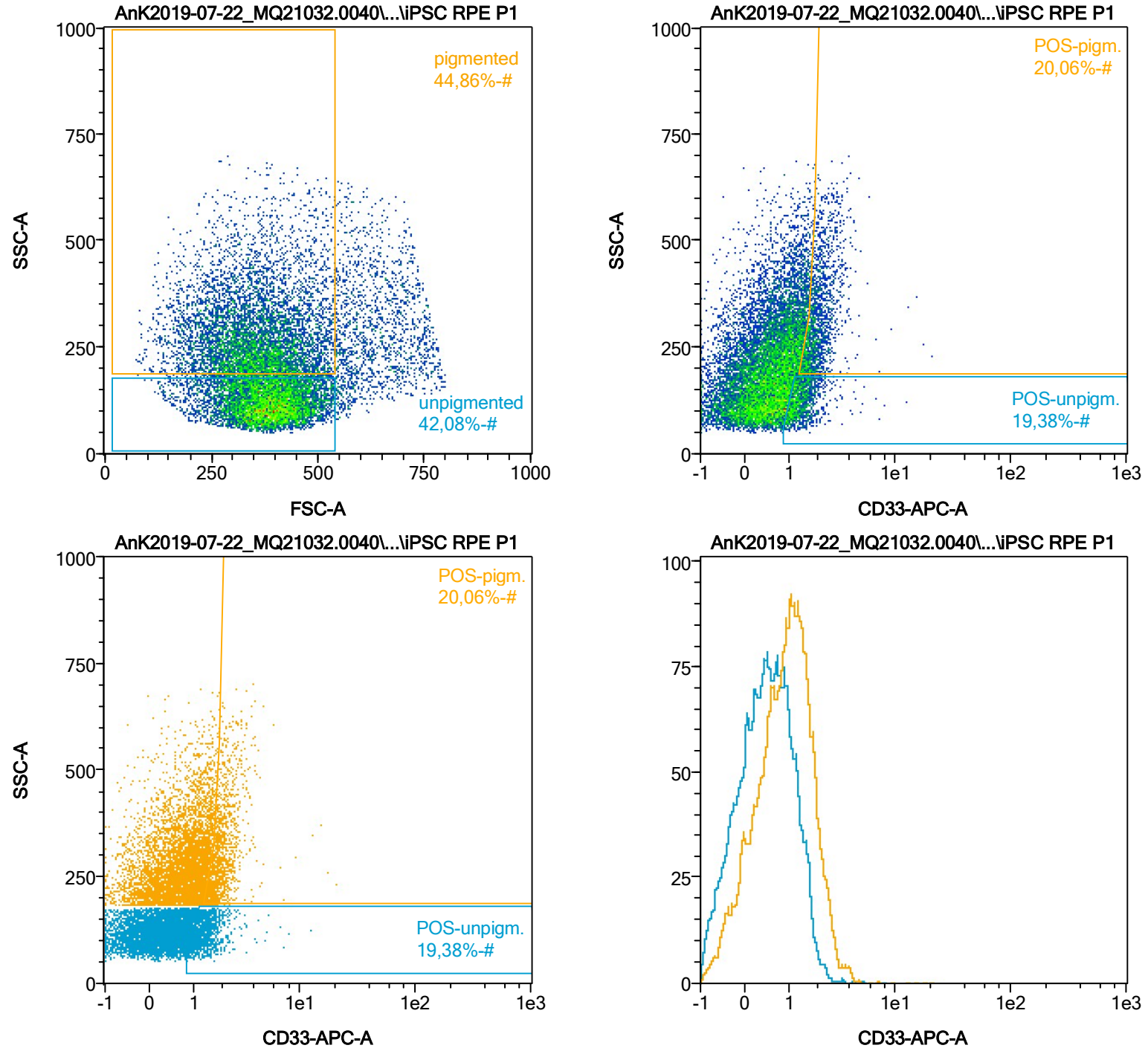

Name	%-#	CD33-APC-A Mean	CD33-APC-A Median
POS-unpigm.	19,38	1,43	1,30
POS-pigm.	20,06	2,10	1,90

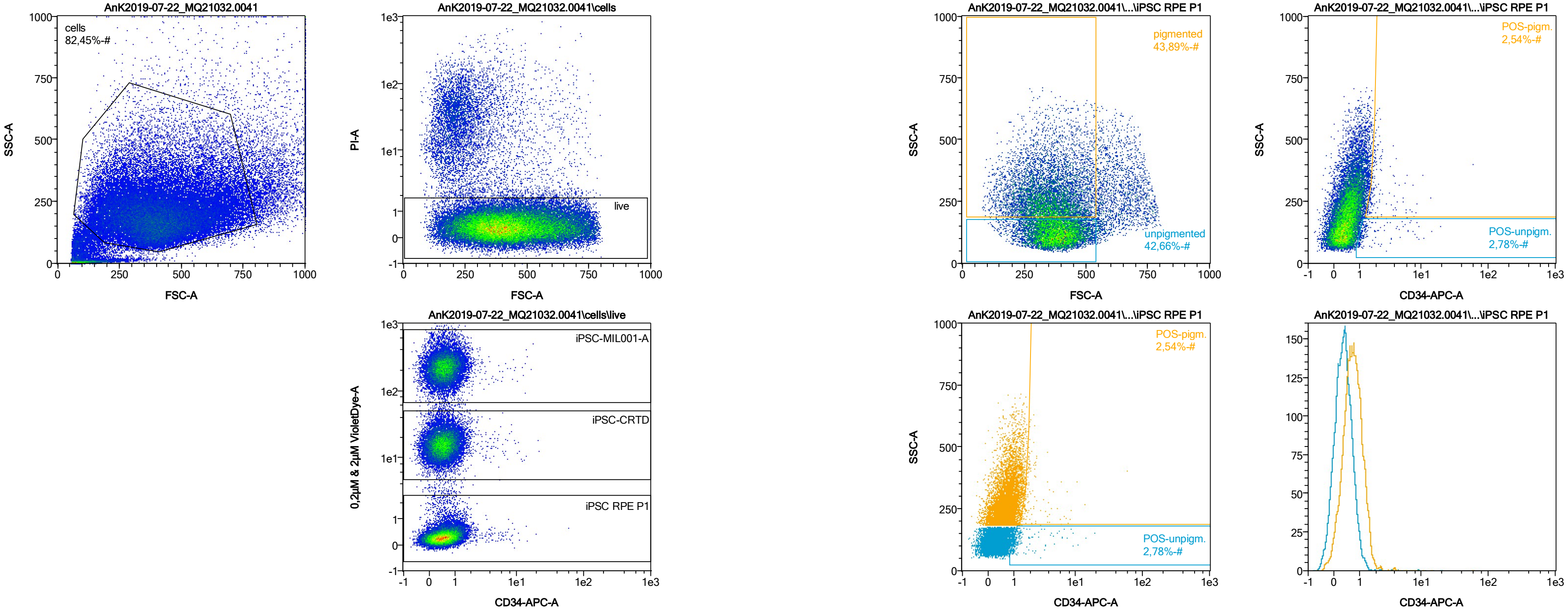

Name	%-#	CD34-APC-A Mean	CD34-APC-A Median
POS-unpigm.	2,78	1,91	1,18
POS-pigm.	2,54	2,69	1,80

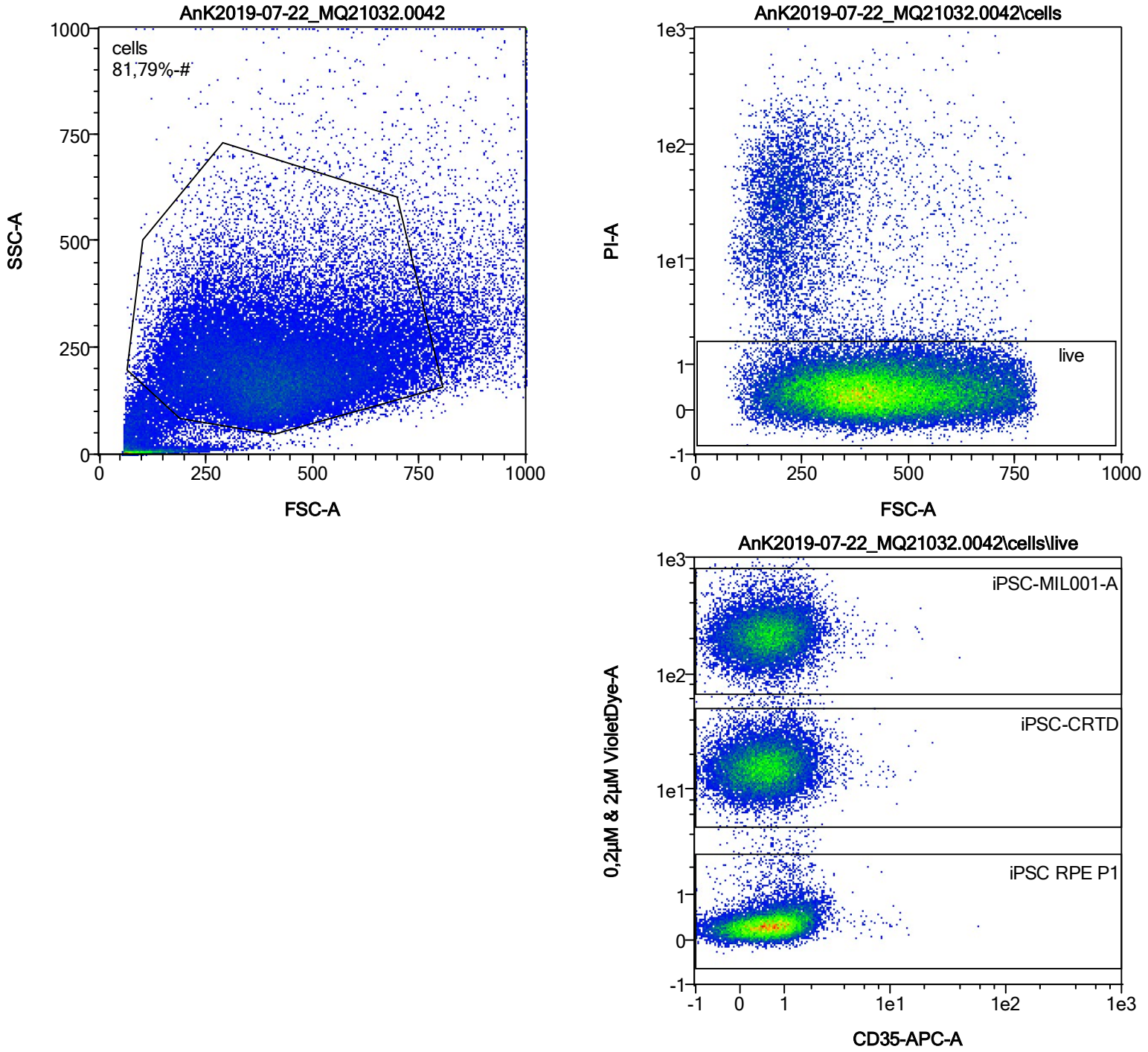

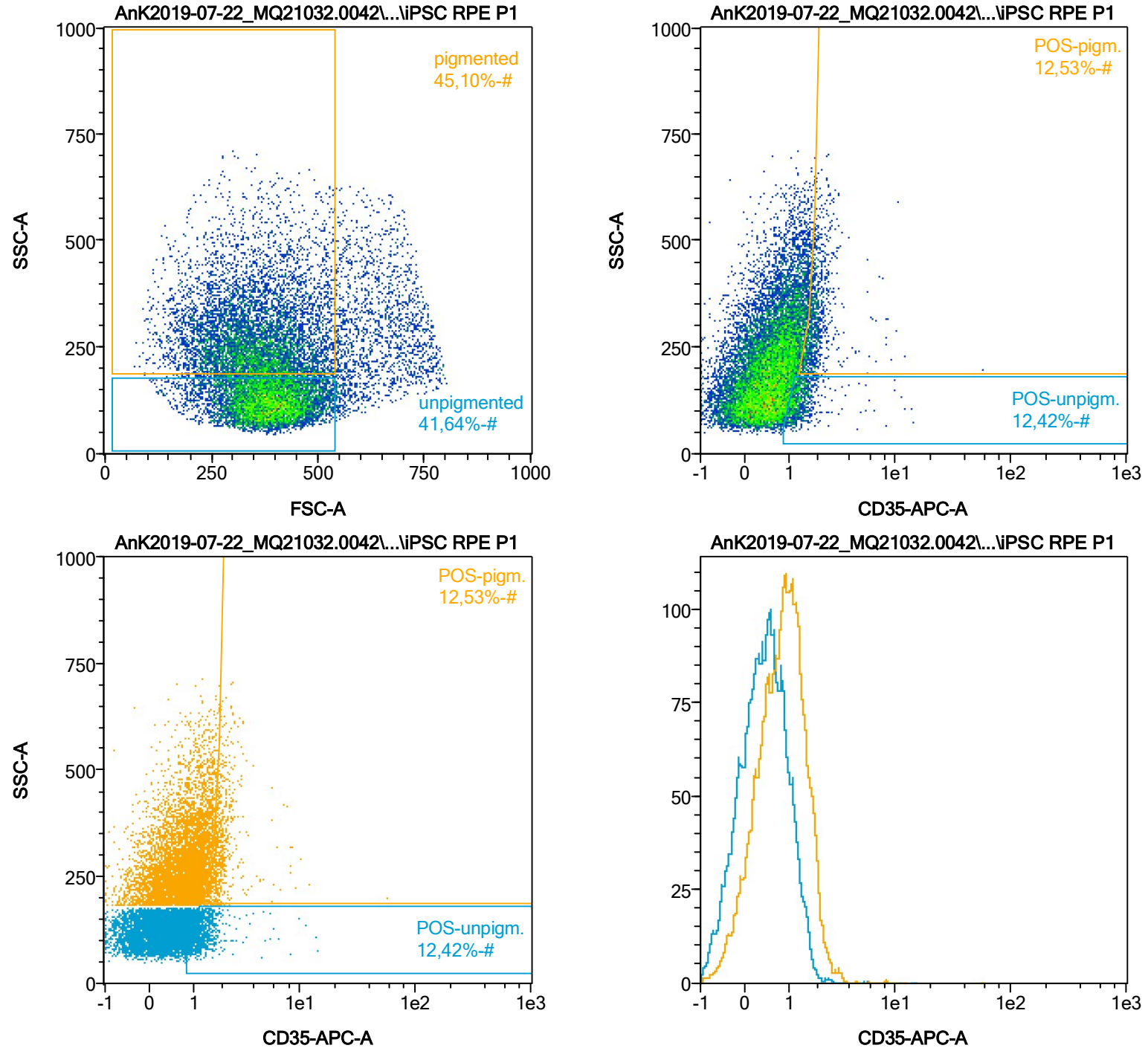

Name	%-#	CD35-APC-A Mean	CD35-APC-A Median
POS-unpigm.	12,42	1,42	1,25
POS-pigm.	12,53	2,09	1,85

Date:2024-06-14

Operator: Sebastian Knoebel

Version:2.13.0+54.g95abf0f4

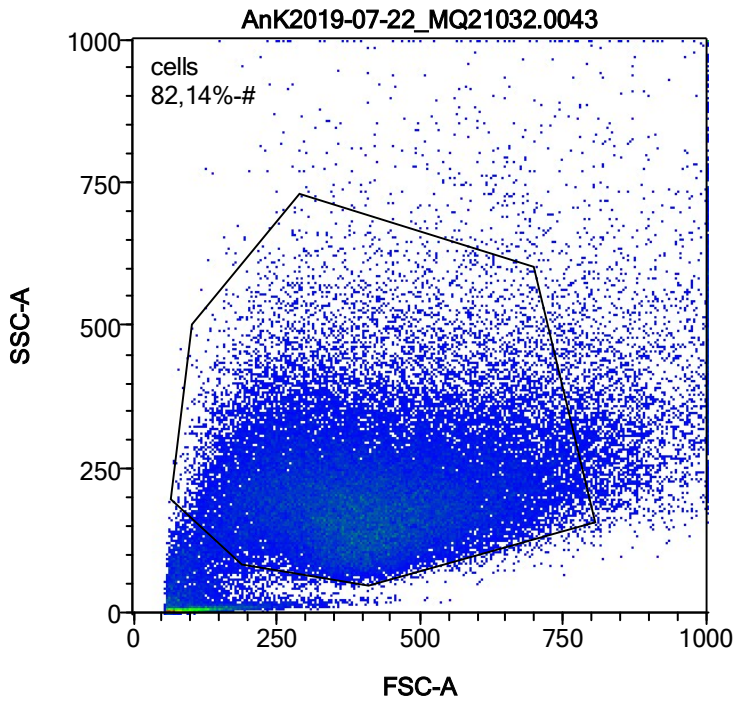

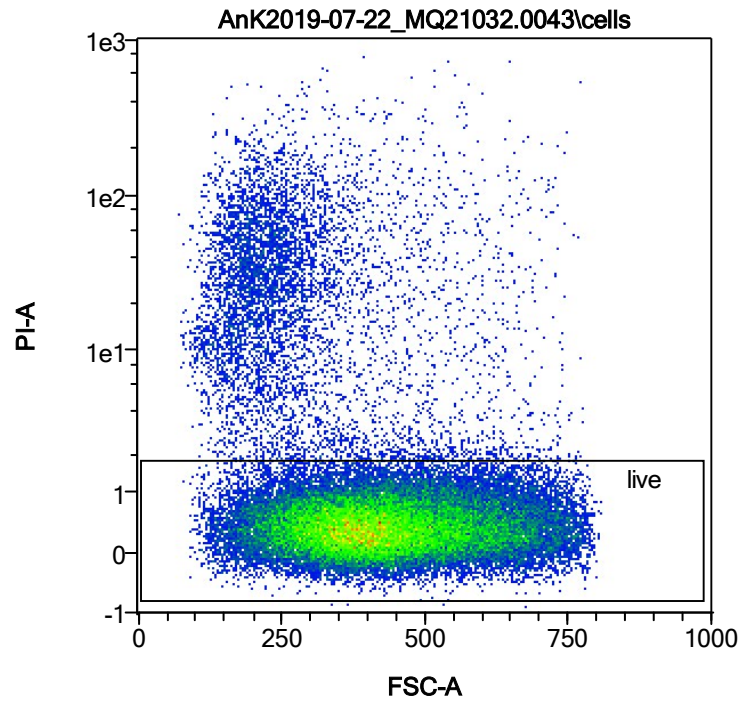

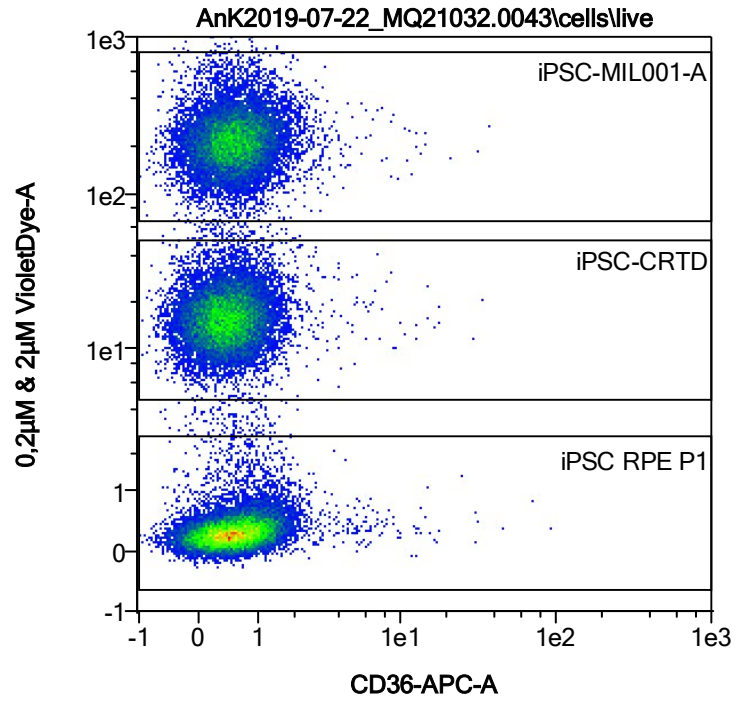

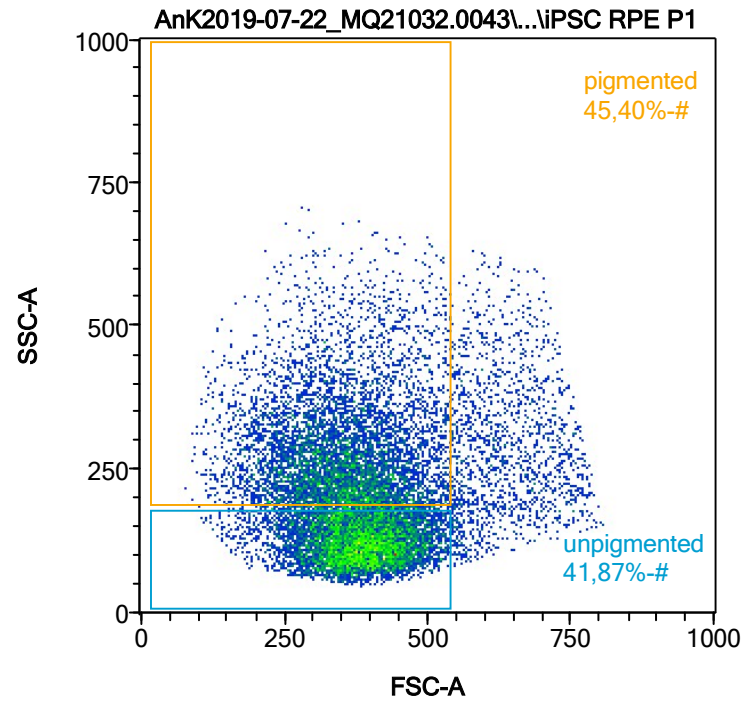

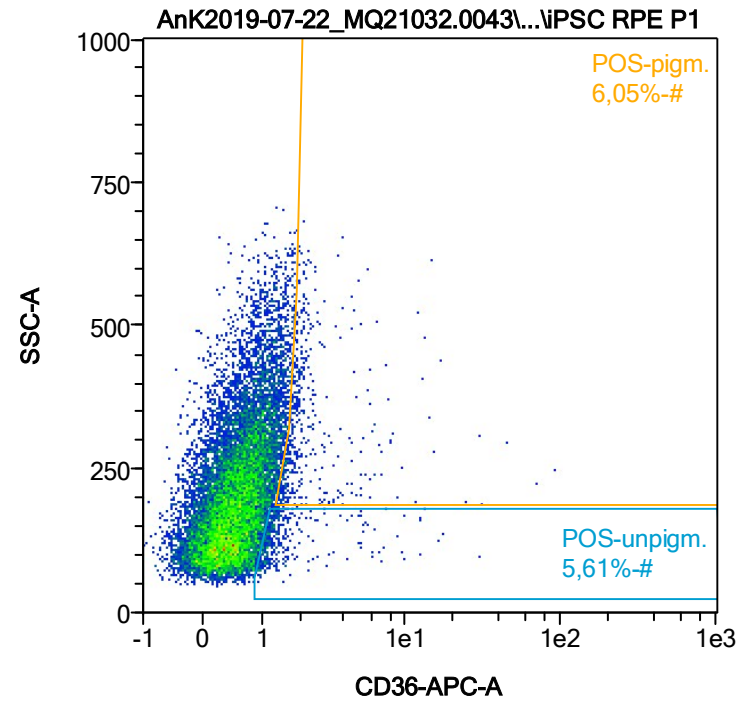

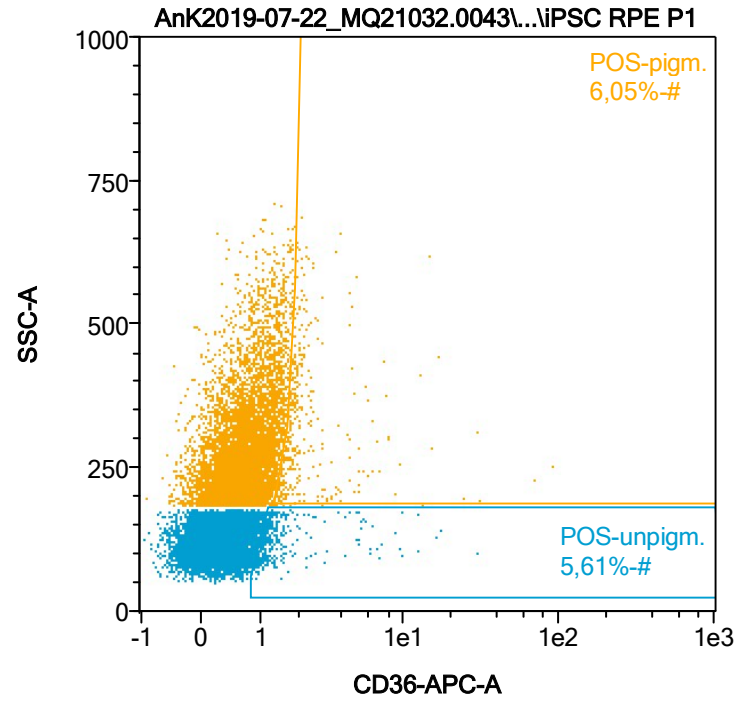

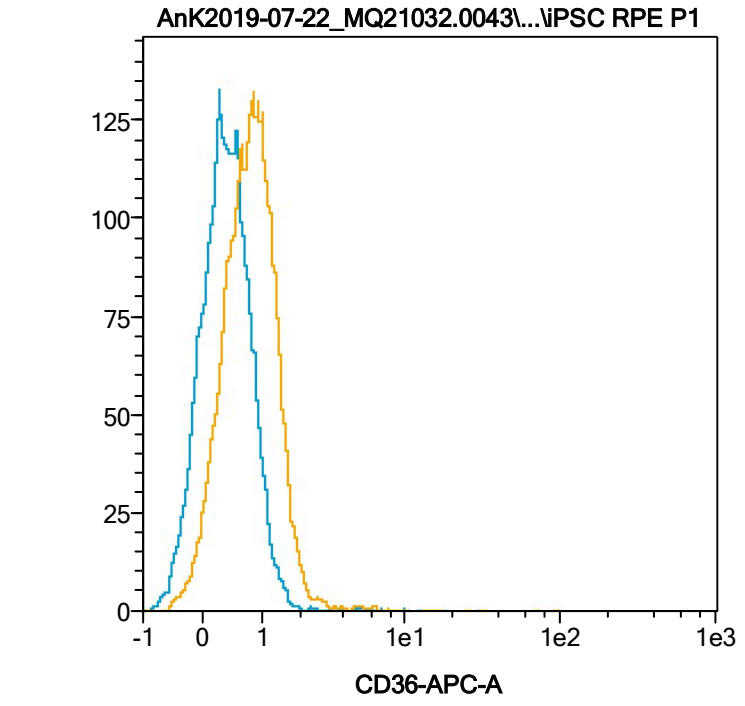

Name	%-#	CD36-APC-A Mean	CD36-APC-A Median
POS-unpigm.	5,61	1,81	1,20
POS-pigm.	6,05	2,93	1,86

Date:2024-06-14

Operator: Sebastian Knoebel

Version:2.13.0+54.g95abf0f4

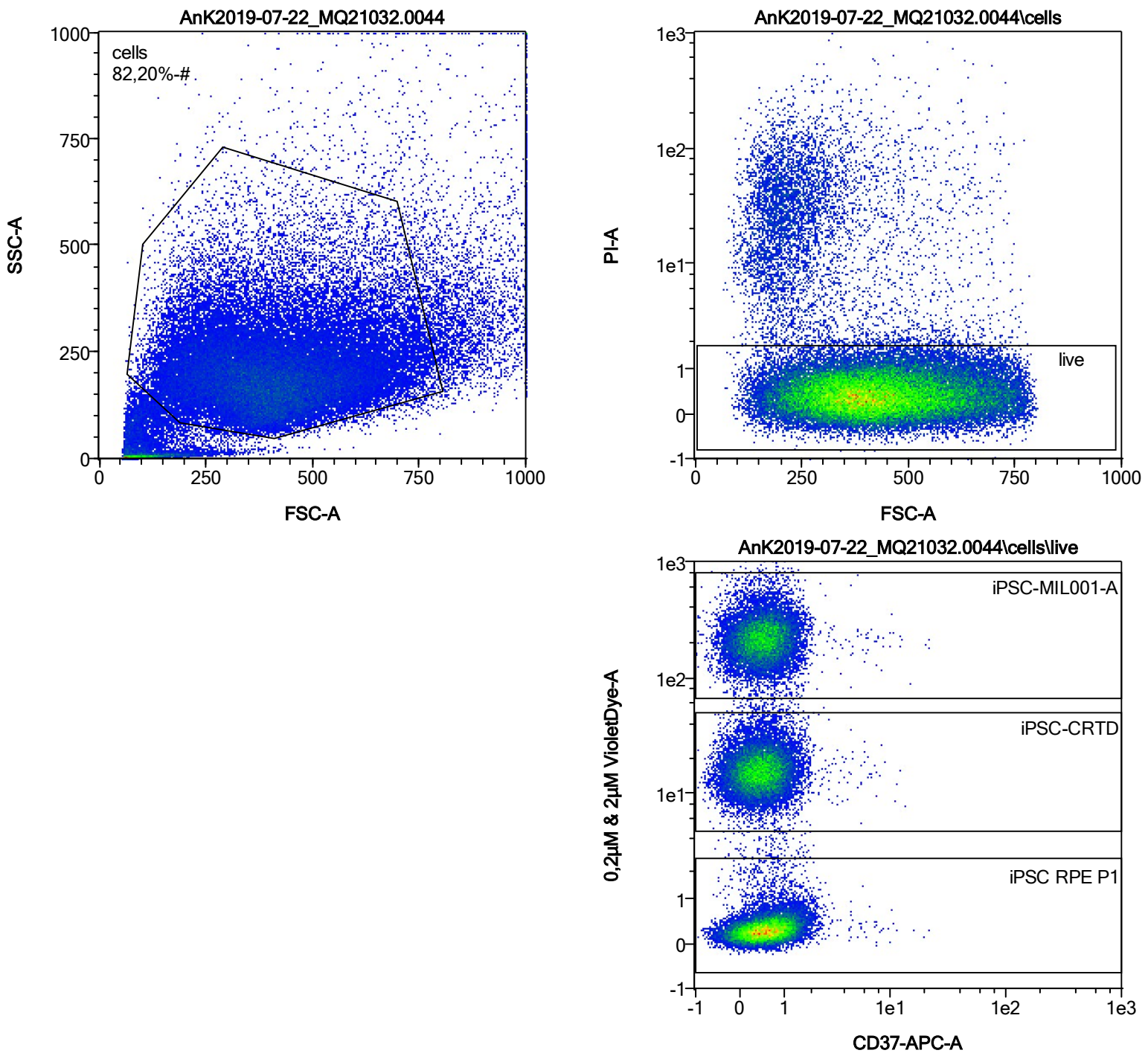

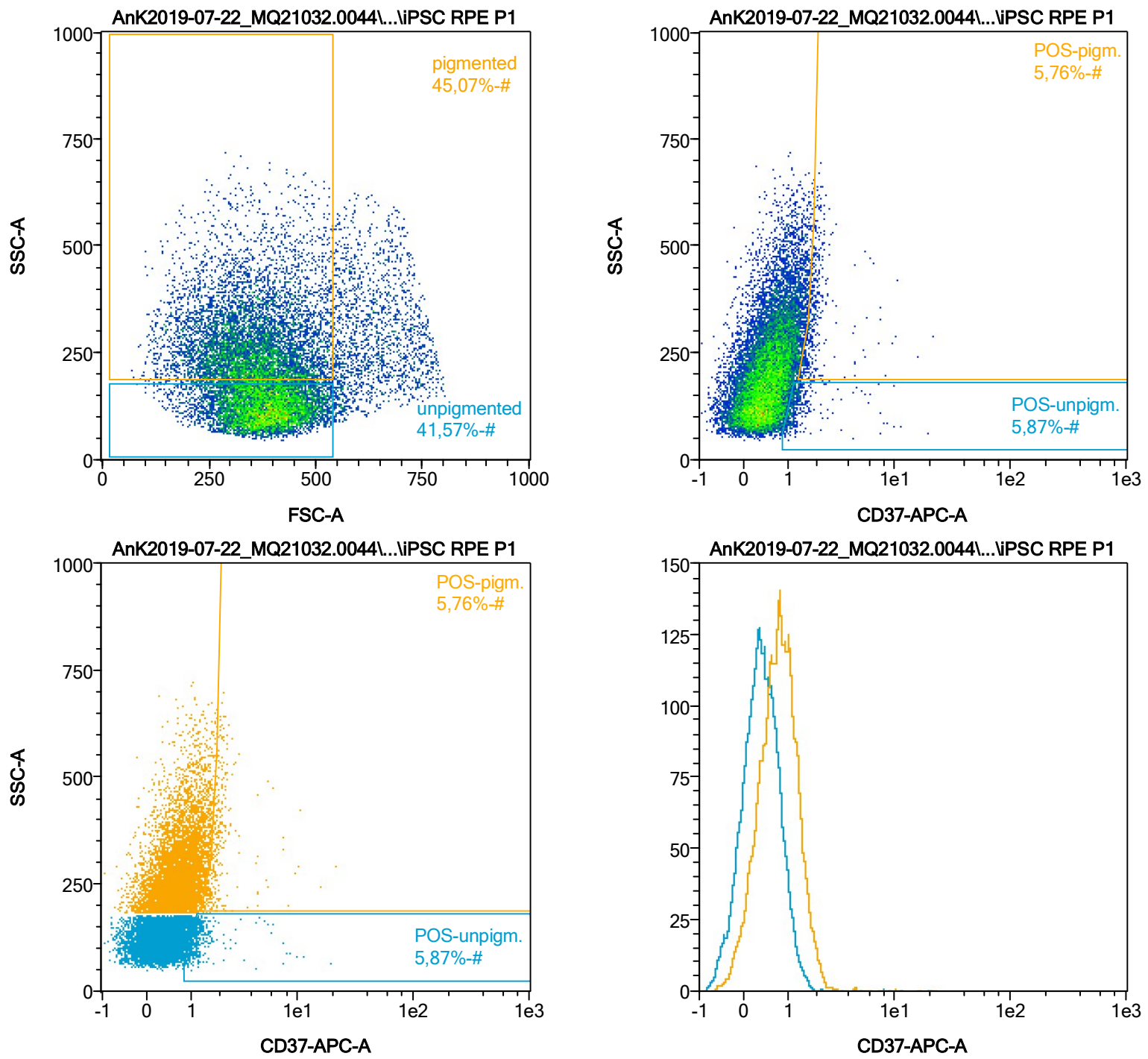

Name	%-#	CD37-APC-A Mean	CD37-APC-A Median
POS-unpigm.	5,87	1,48	1,17
POS-pigm.	5,76	2,12	1,84

Date:2024-06-14

Operator: Sebastian Knoebel

Version:2.13.0+54.g95abf0f4

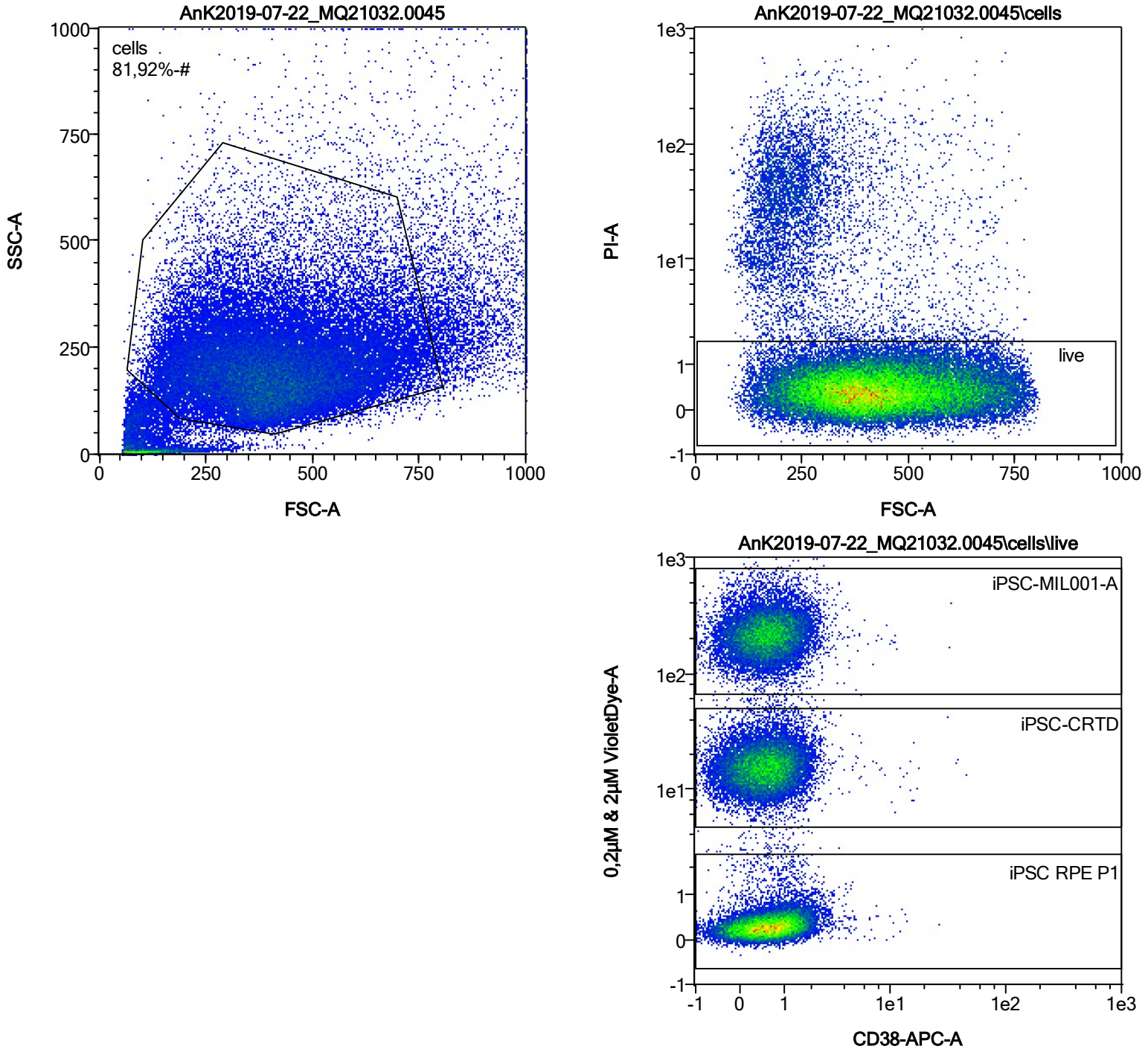

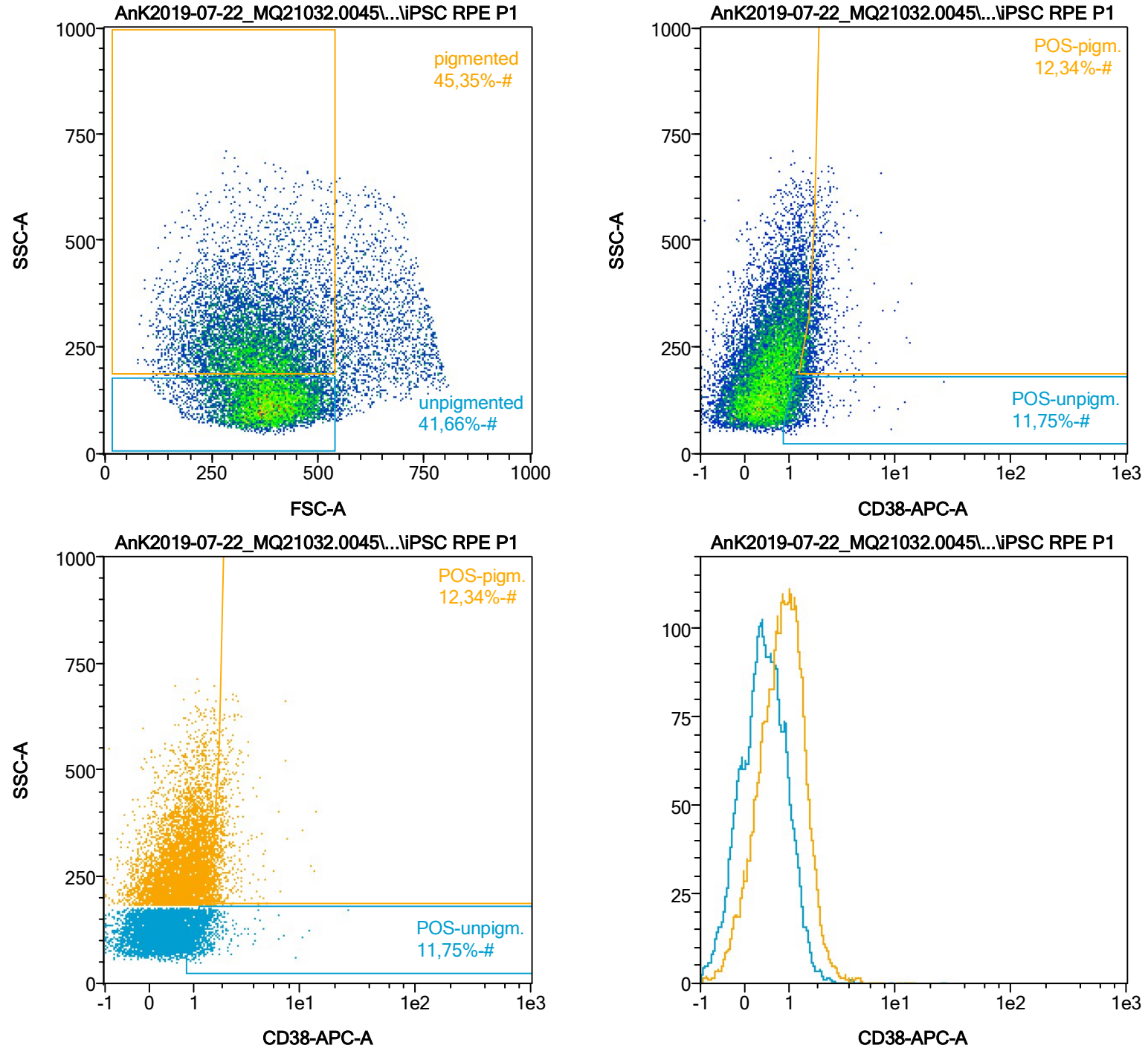

Name	%-#	CD38-APC-A Mean	CD38-APC-A Median
POS-unpigm.	11,75	1,45	1,24
POS-pigm.	12,34	2,06	1,83

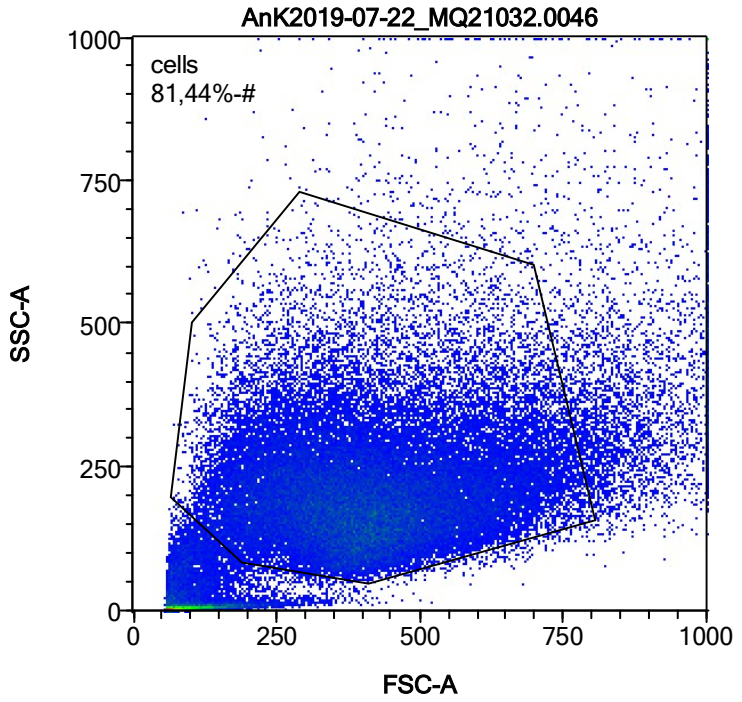

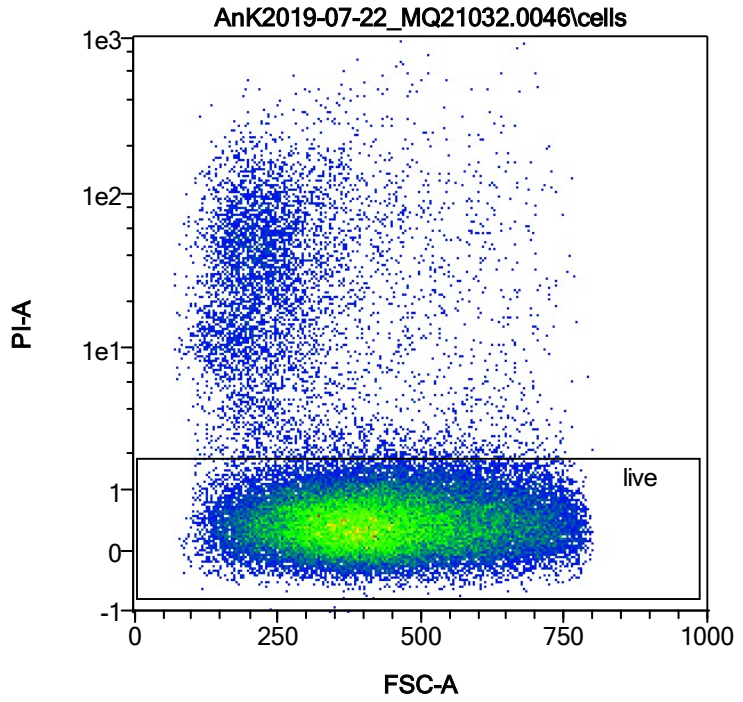

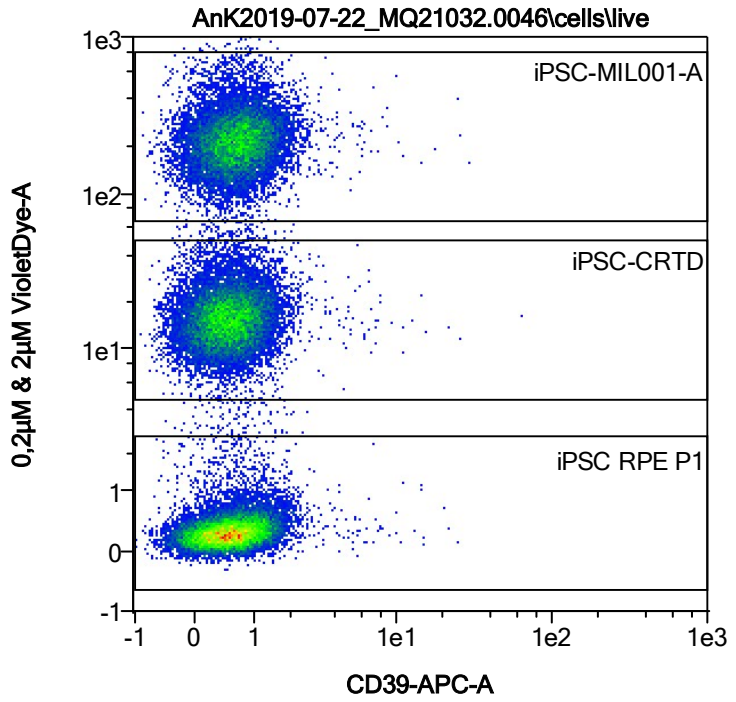

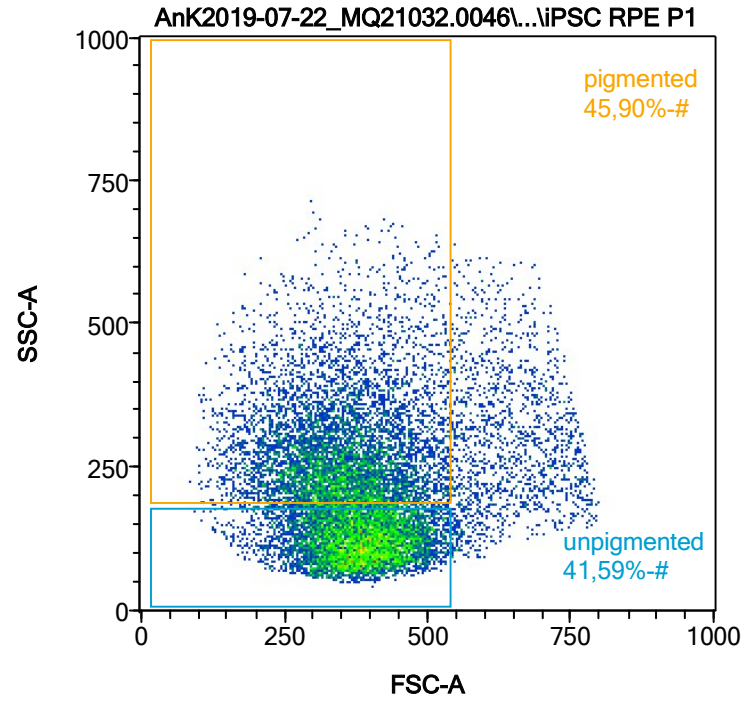

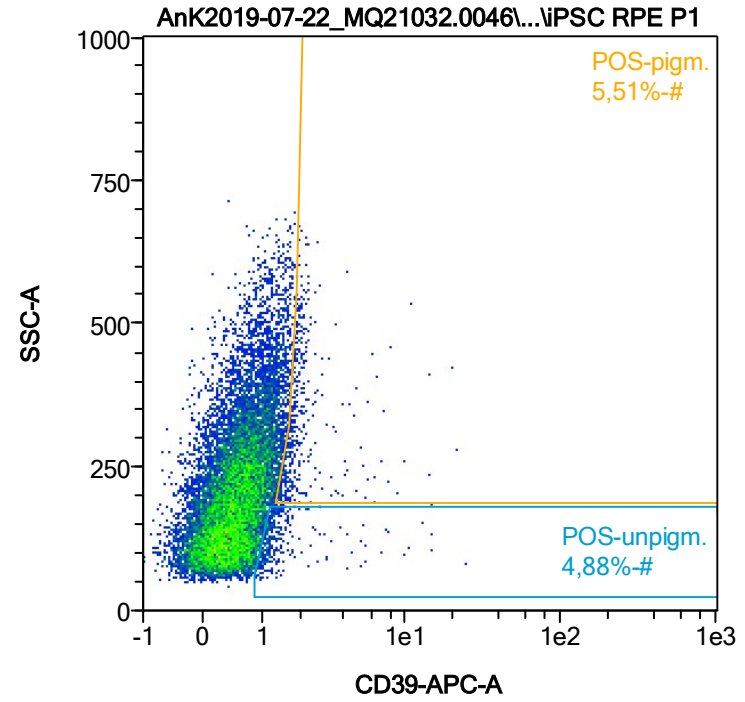

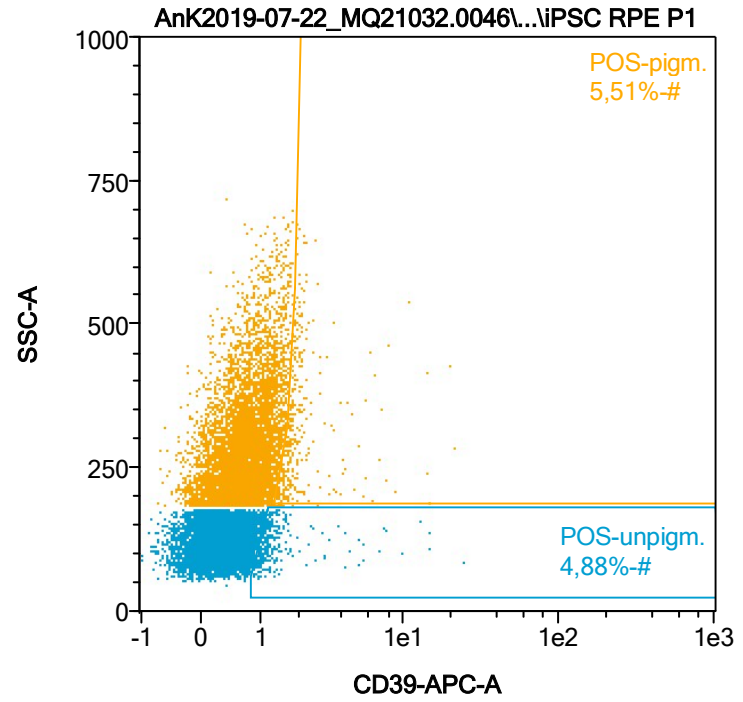

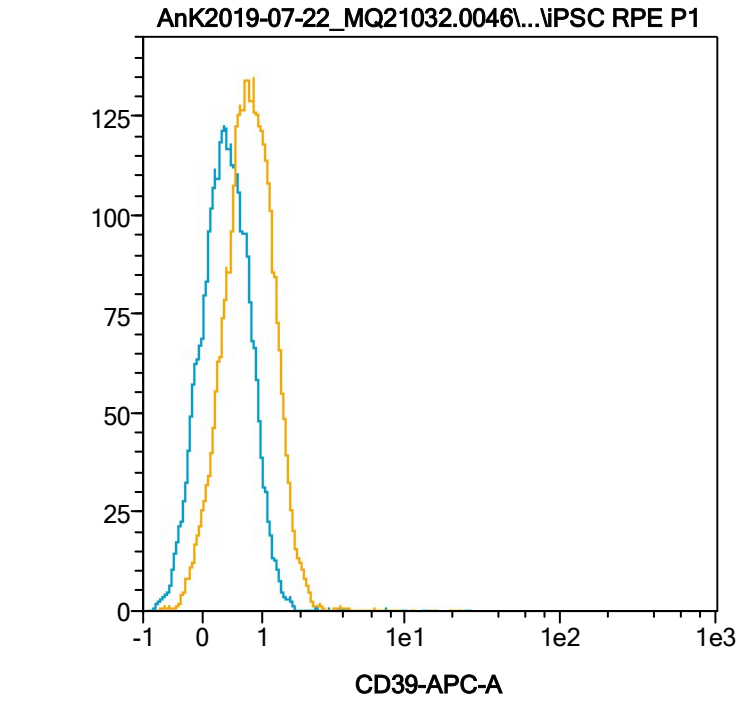

Name	%-#	CD39-APC-A Mean	CD39-APC-A Median
POS-unpigm.	4,88	1,62	1,17
POS-pigm.	5,51	2,26	1,80

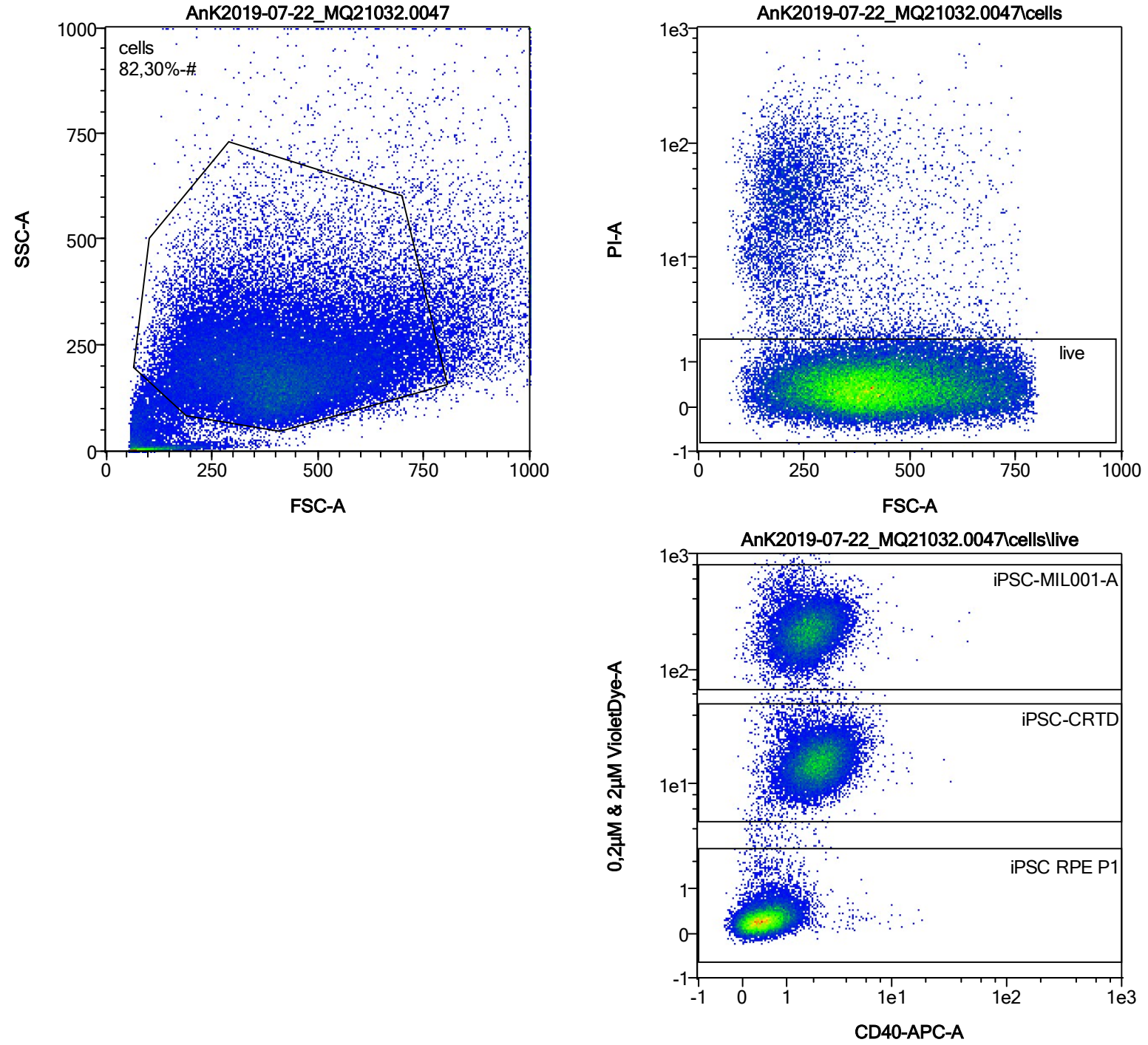

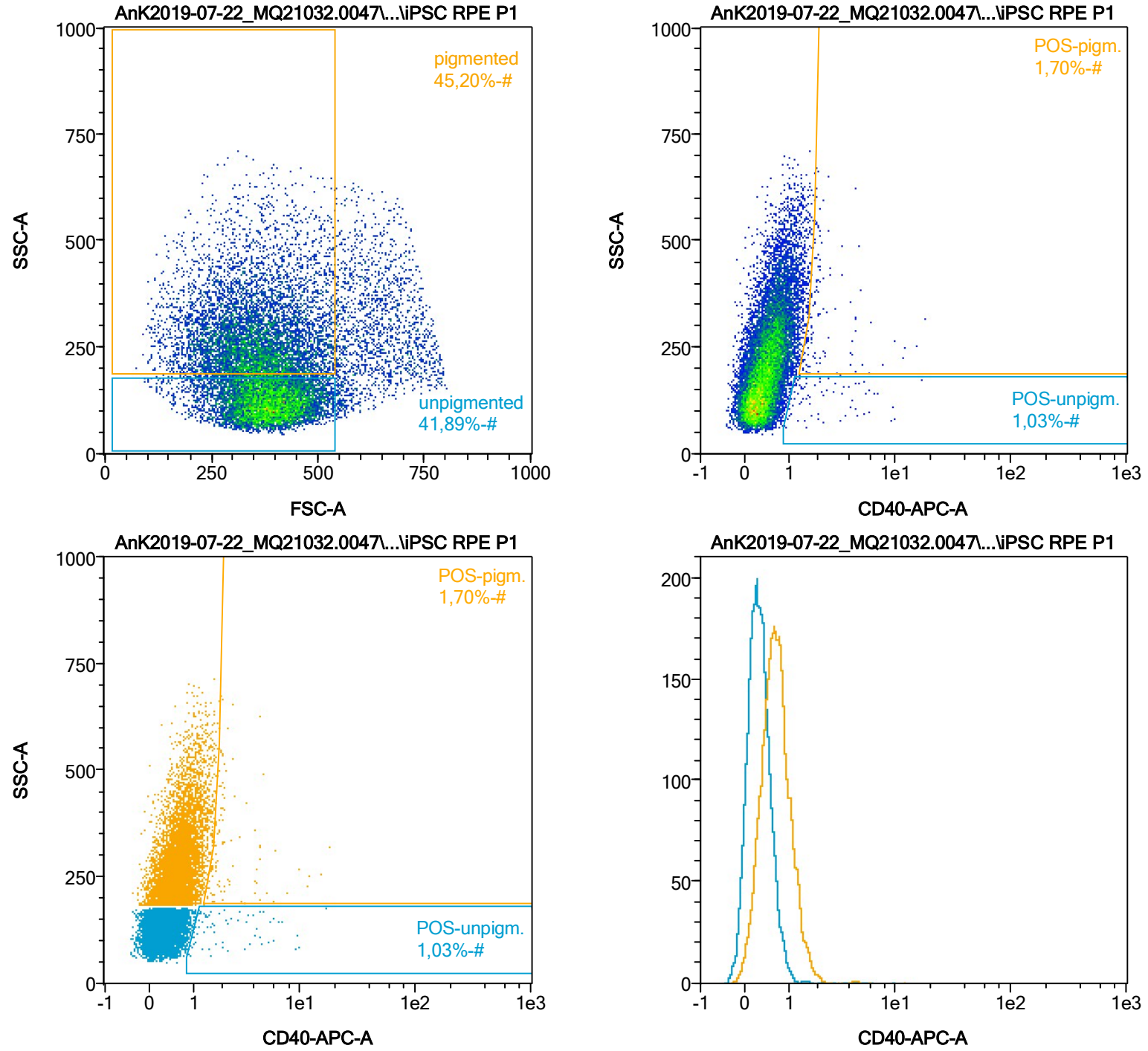

Name	%-#	CD40-APC-A Mean	CD40-APC-A Median
POS-unpigm.	1,03	2,92	1,71
POS-pigm.	1,70	2,77	1,93

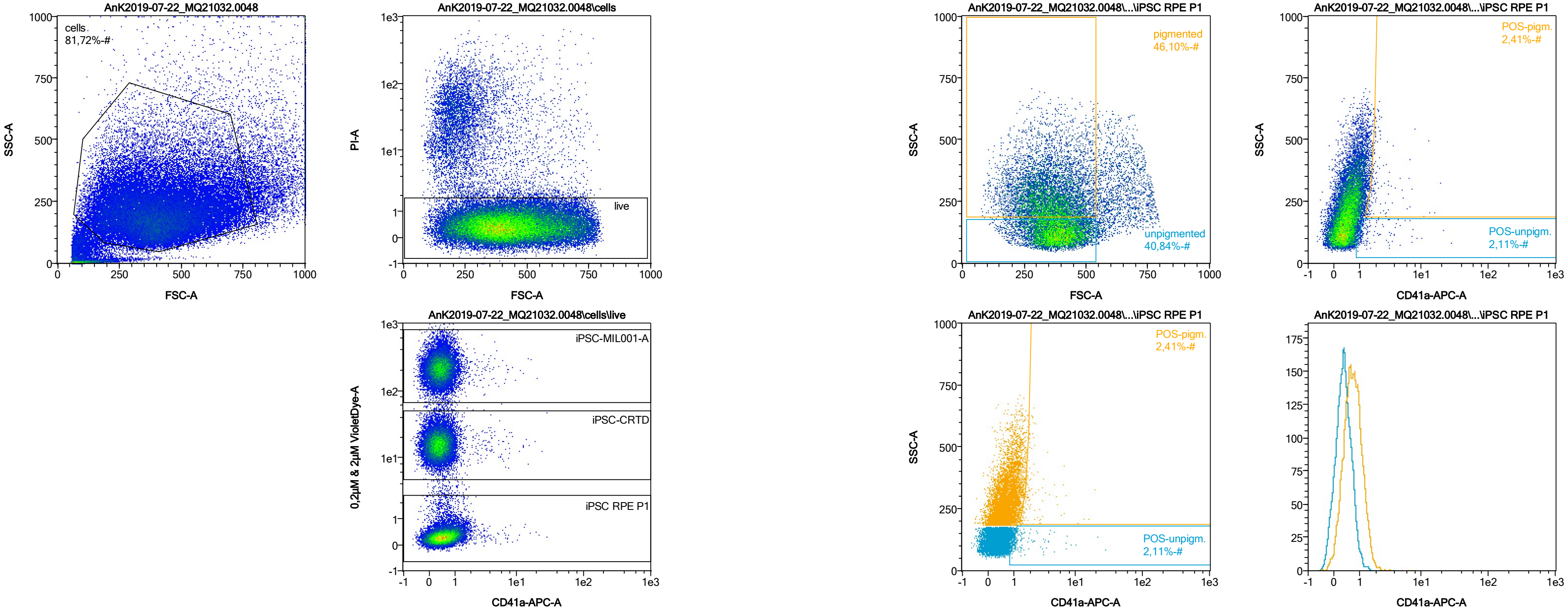

Name	%-#	CD41a-APC-A Mean	CD41a-APC-A Median
POS-unpigm.	2,11	2,16	1,21
POS-pigm.	2,41	2,40	1,80

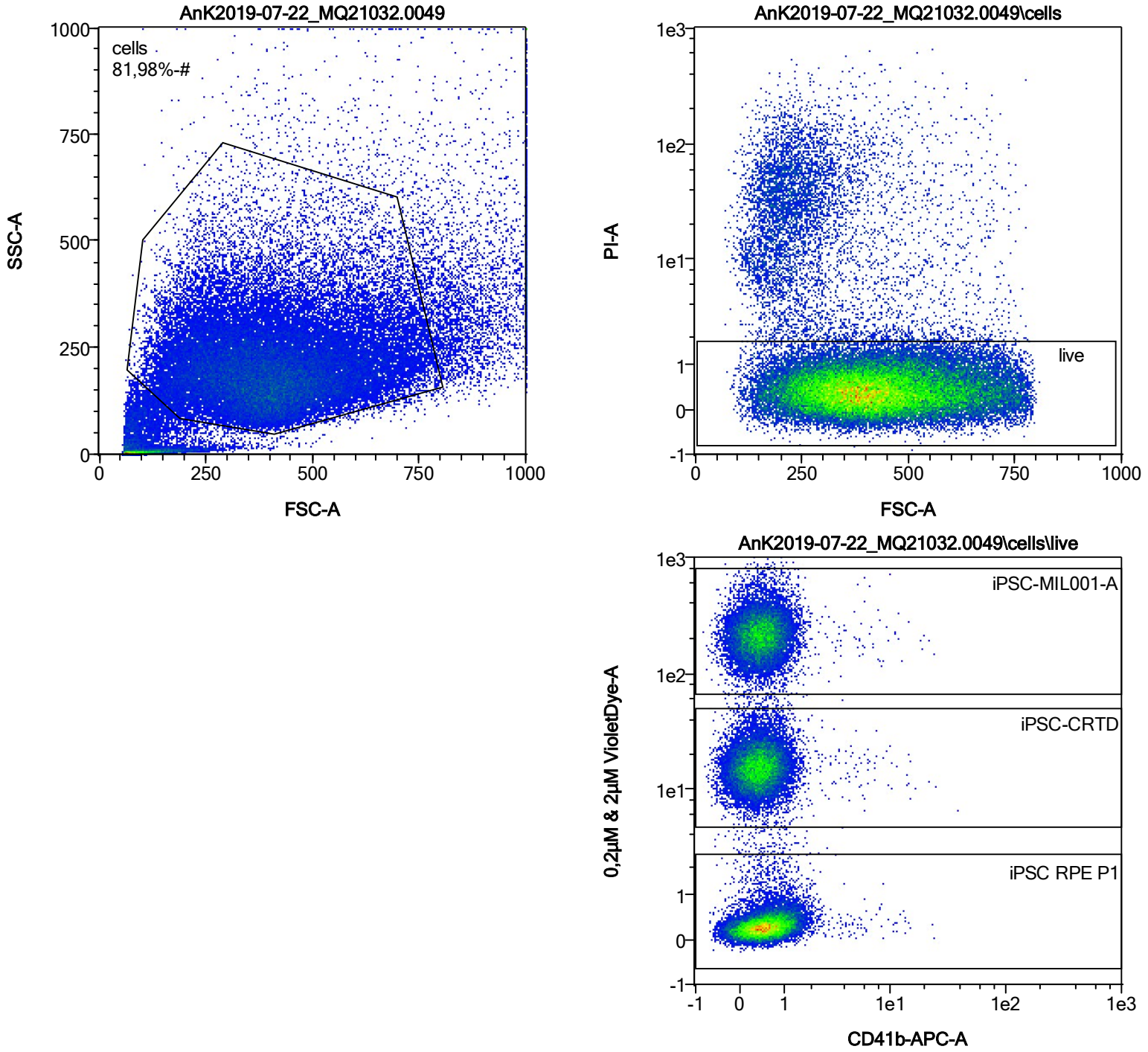

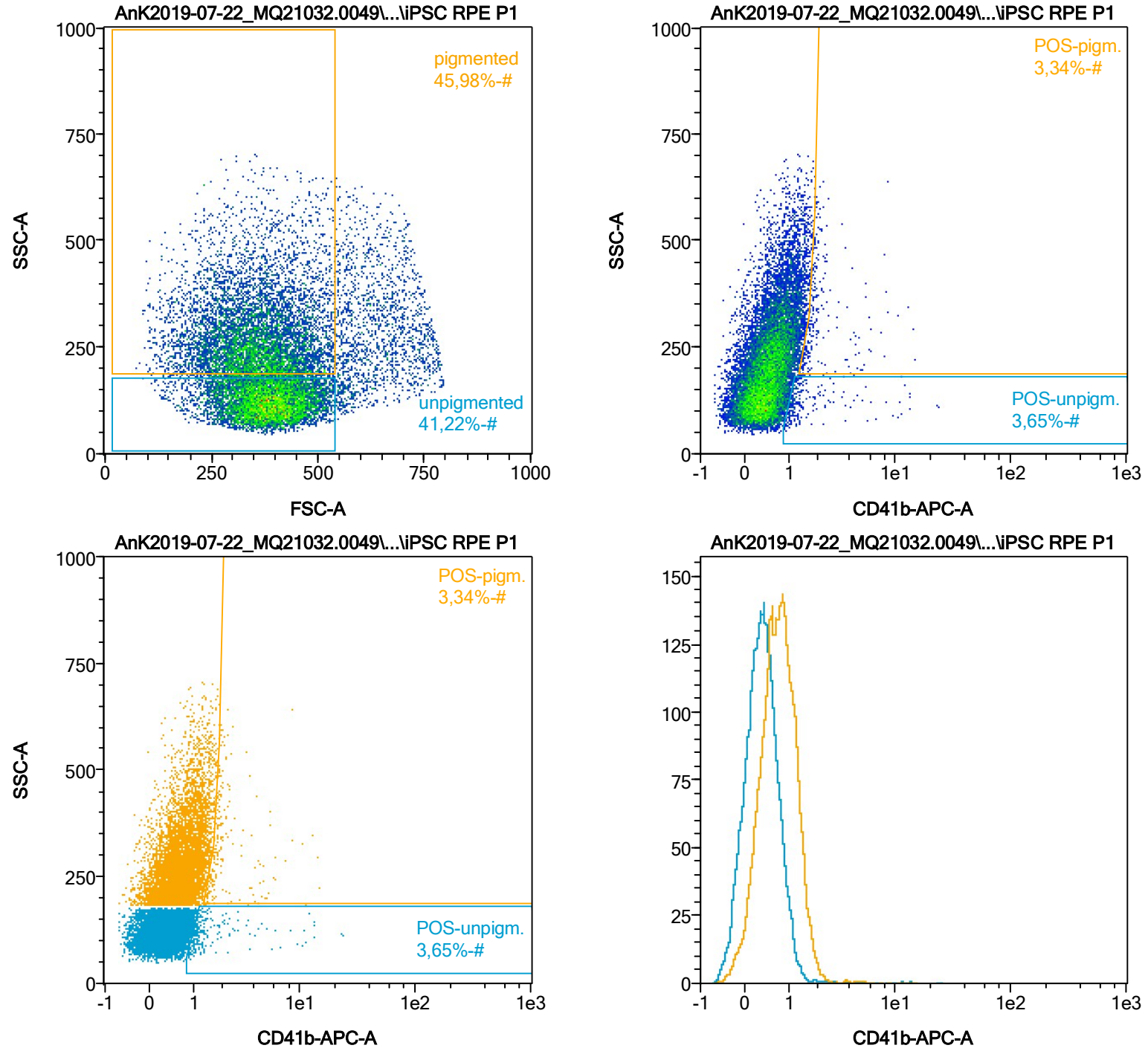

Name	%-#	CD41b-APC-A Mean	CD41b-APC-A Median
POS-unpigm.	3,65	2,03	1,19
POS-pigm.	3,34	2,43	1,85

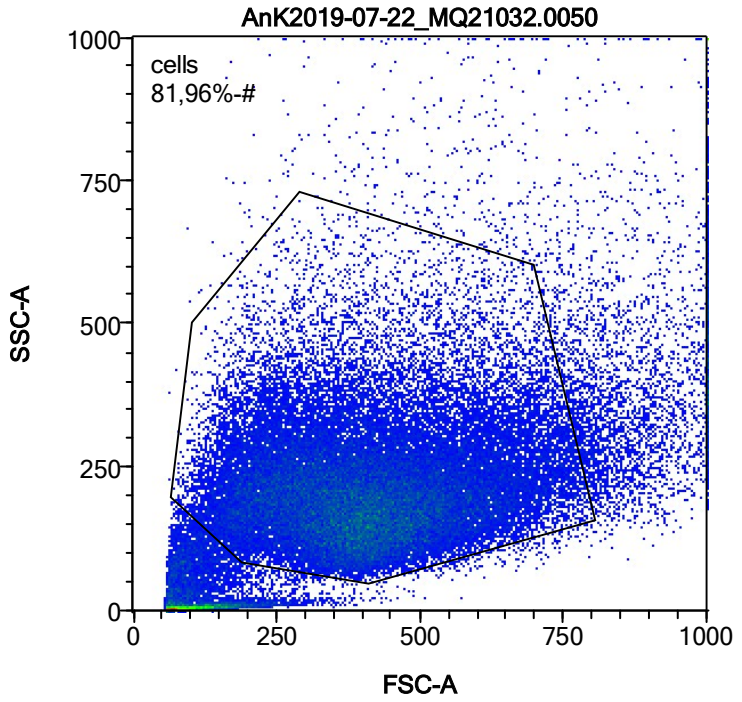

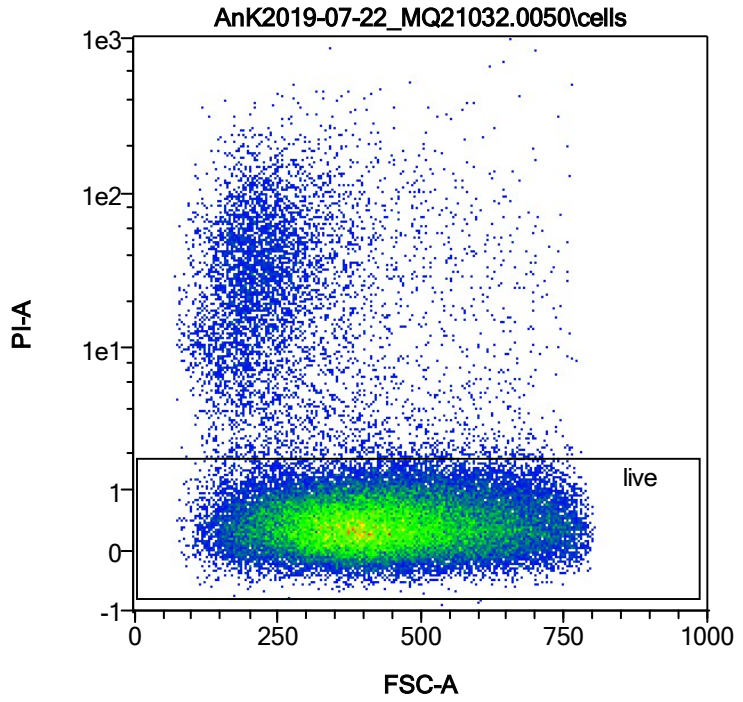

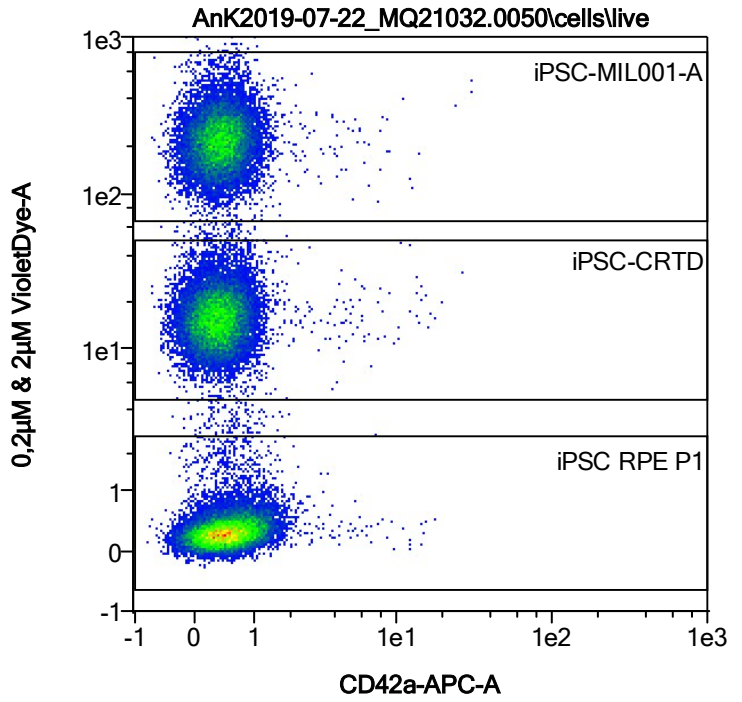

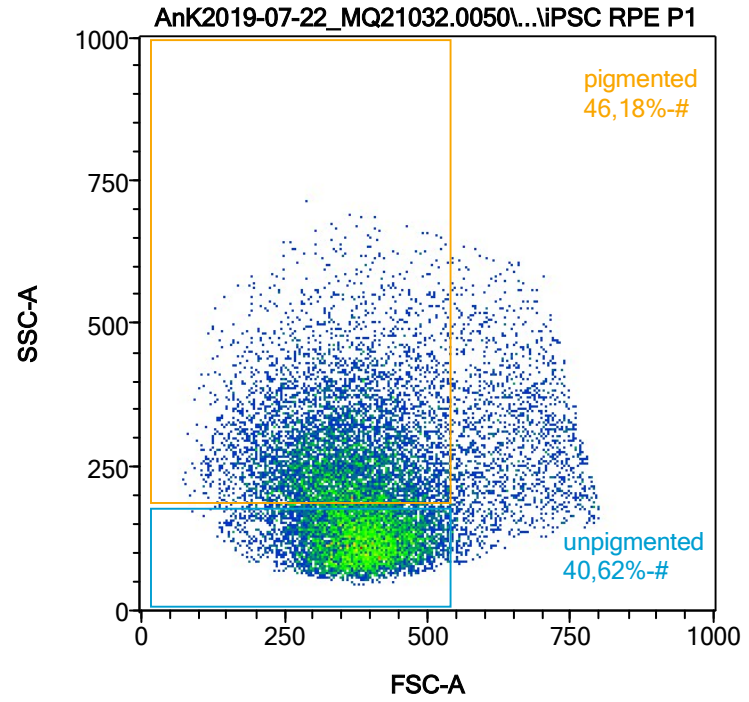

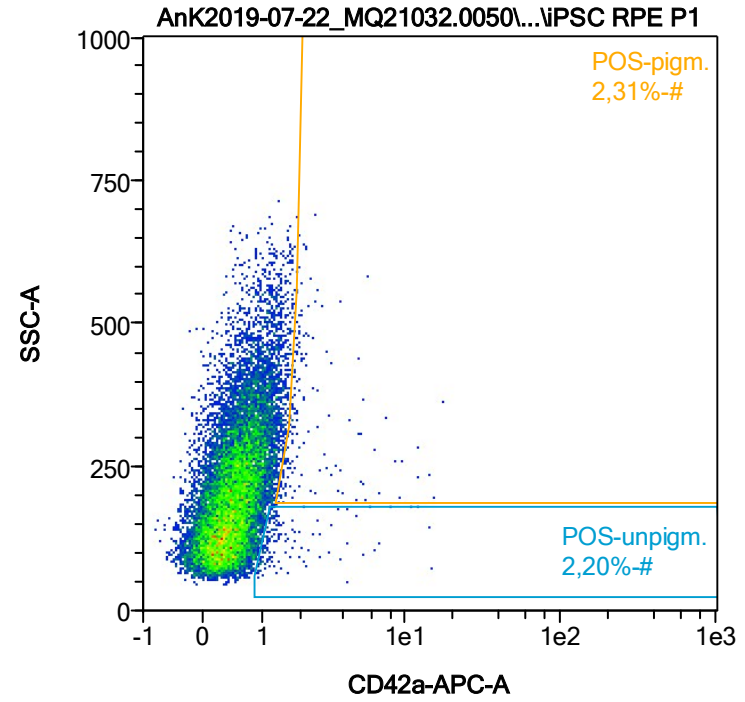

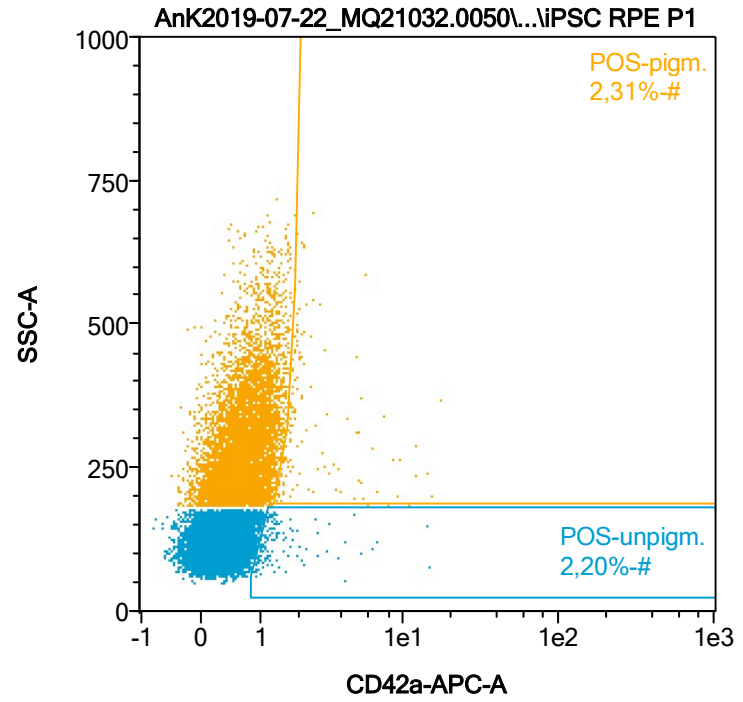

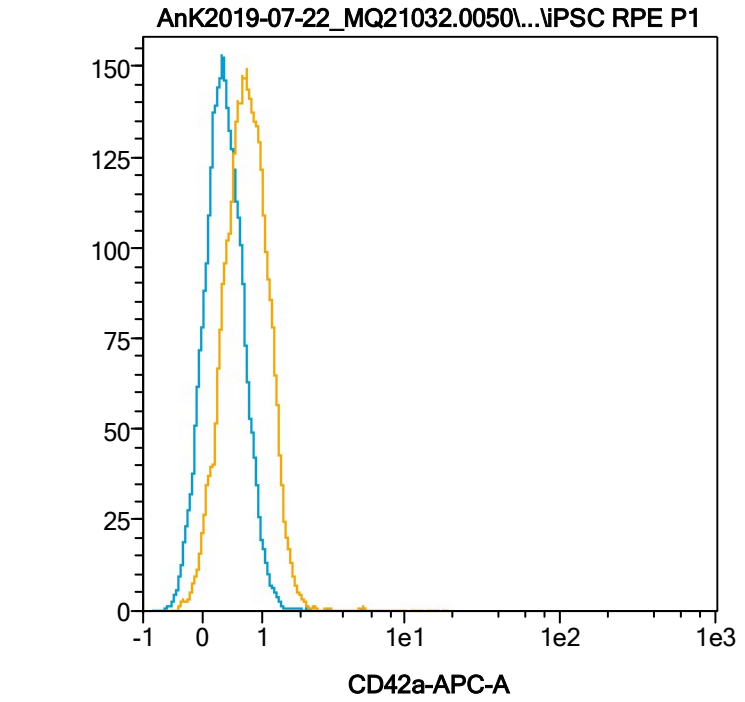

Name	%-#	CD42a-APC-A Mean	CD42a-APC-A Median
POS-unpigm.	2,20	1,70	1,21
POS-pigm.	2,31	2,85	1,87

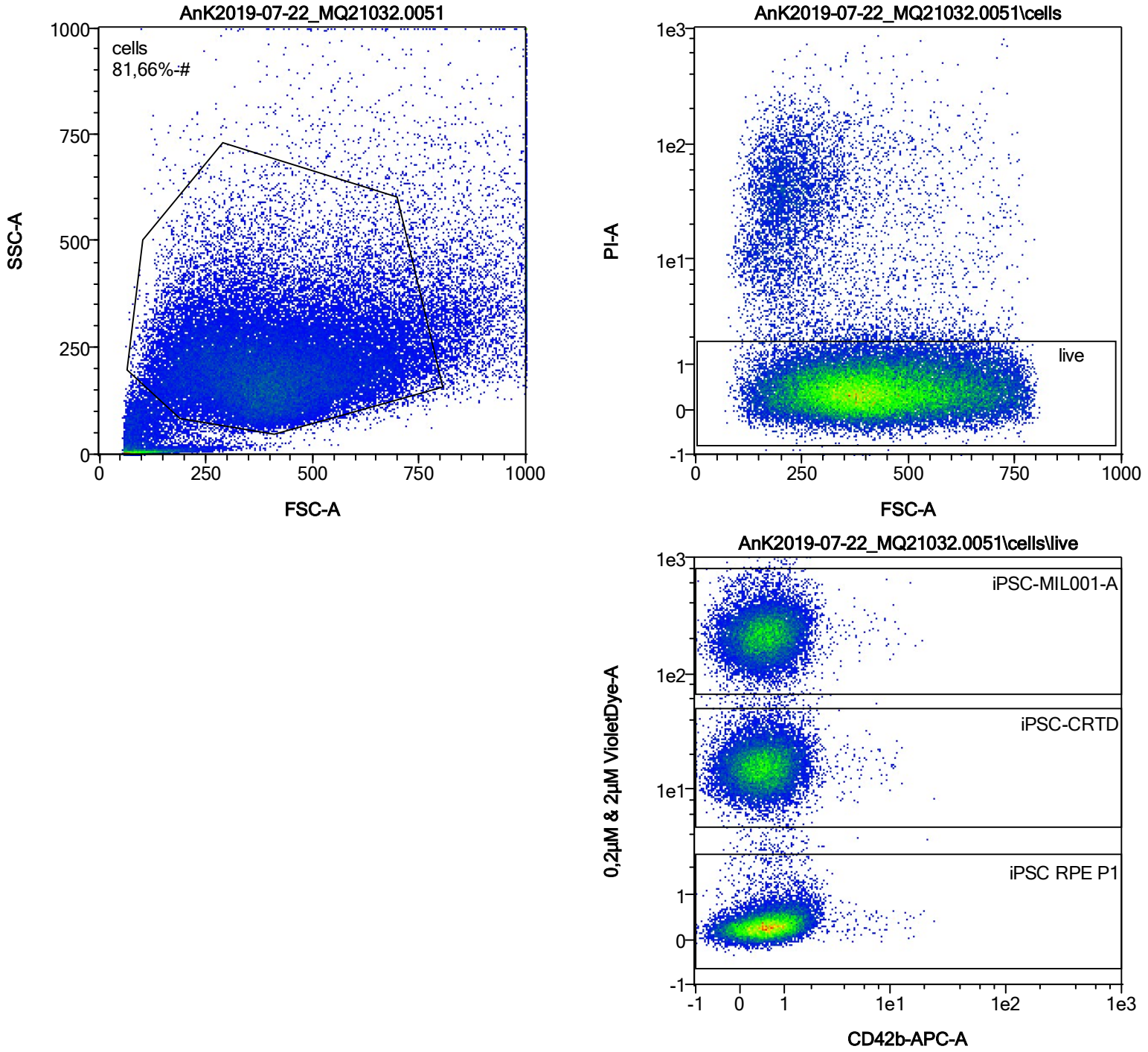

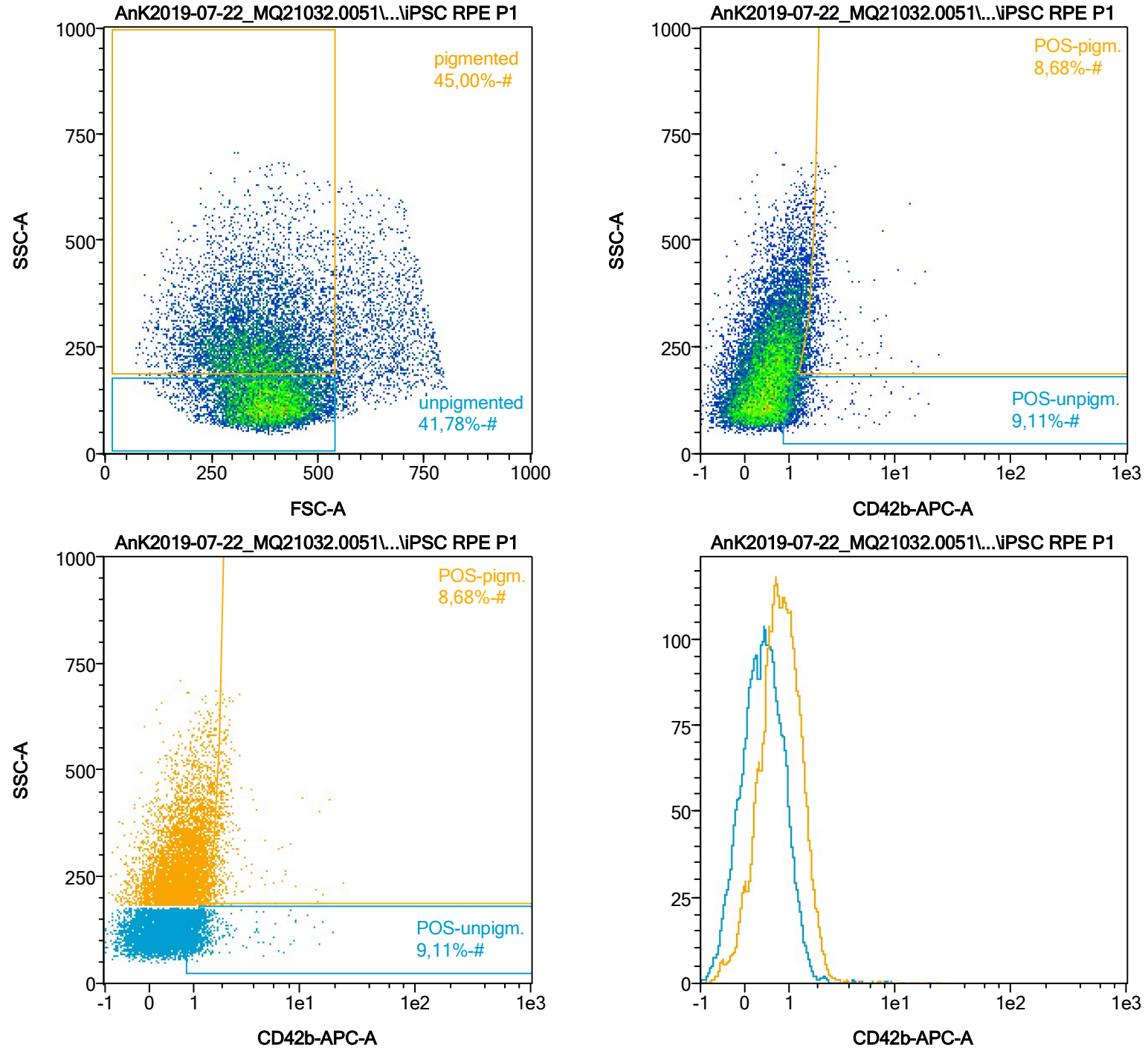

Name	%-#	CD42b-APC-A Mean	CD42b-APC-A Median
POS-unpigm.	9,11	1,59	1,22
POS-pigm.	8,68	2,17	1,82

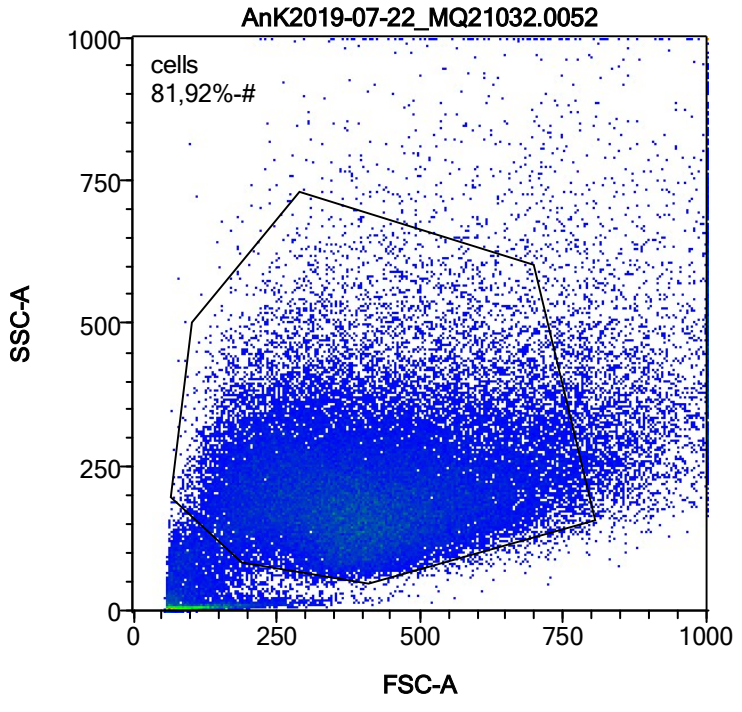

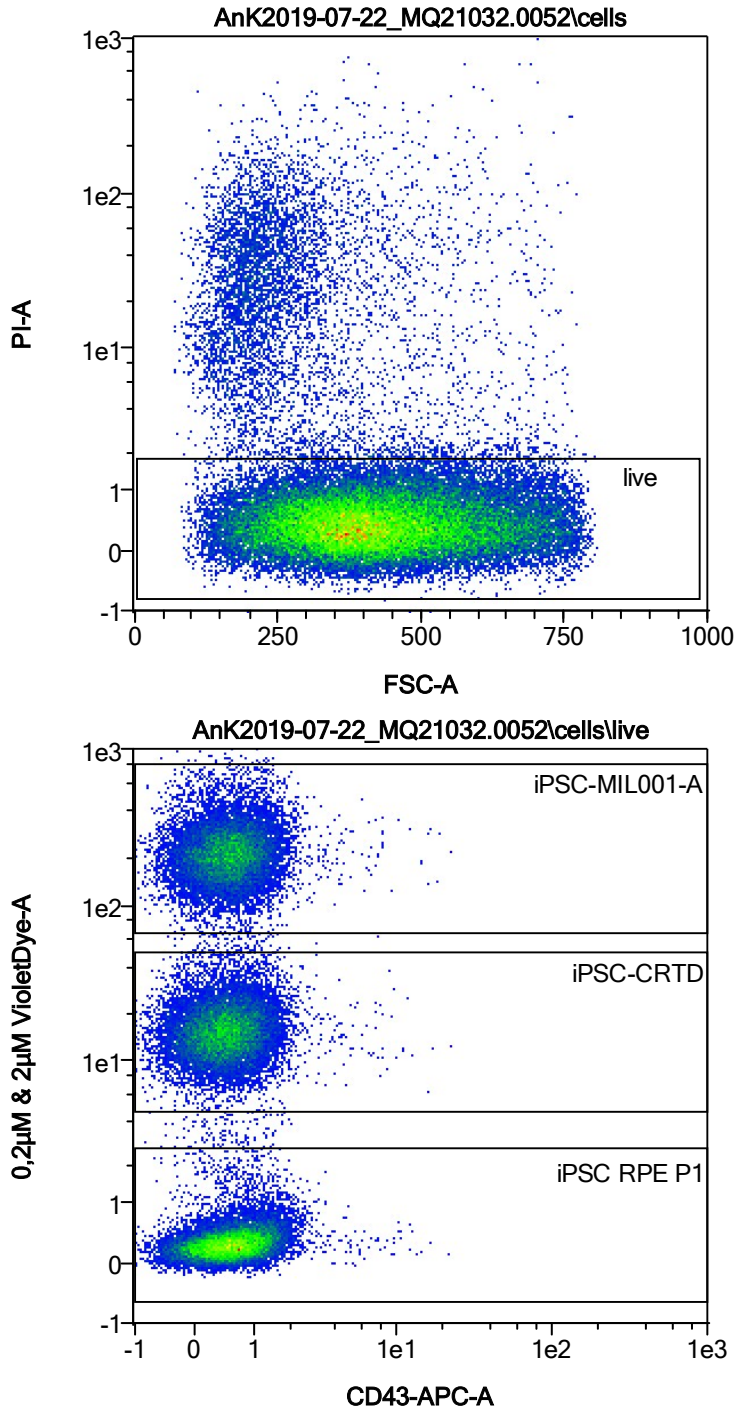

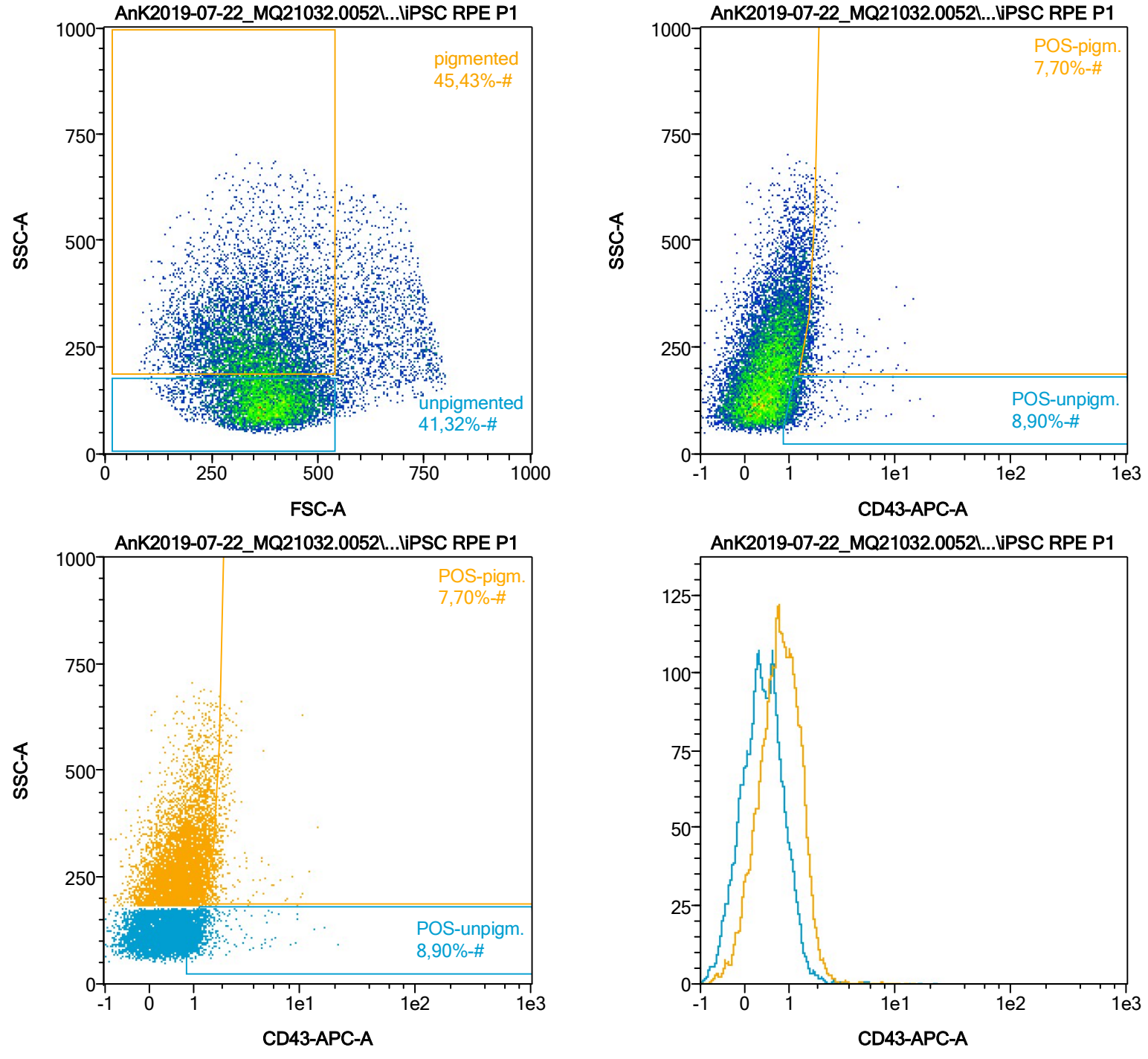

Name	%-#	CD43-APC-A Mean	CD43-APC-A Median
POS-unpigm.	8,90	1,56	1,22
POS-pigm.	7,70	2,08	1,78

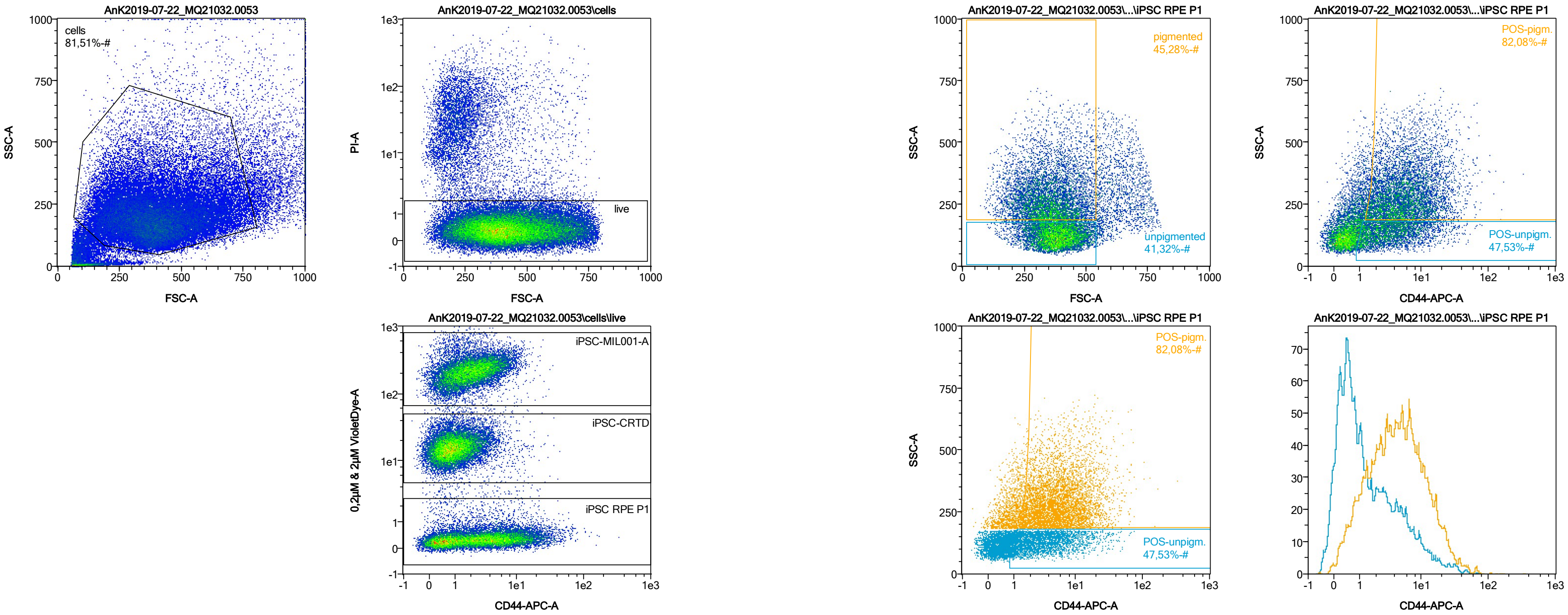

Name	%-#	CD44-APC-A Mean	CD44-APC-A Median
POS-unpigm.	47,53	4,98	2,94
POS-pigm.	82,08	8,01	5,31

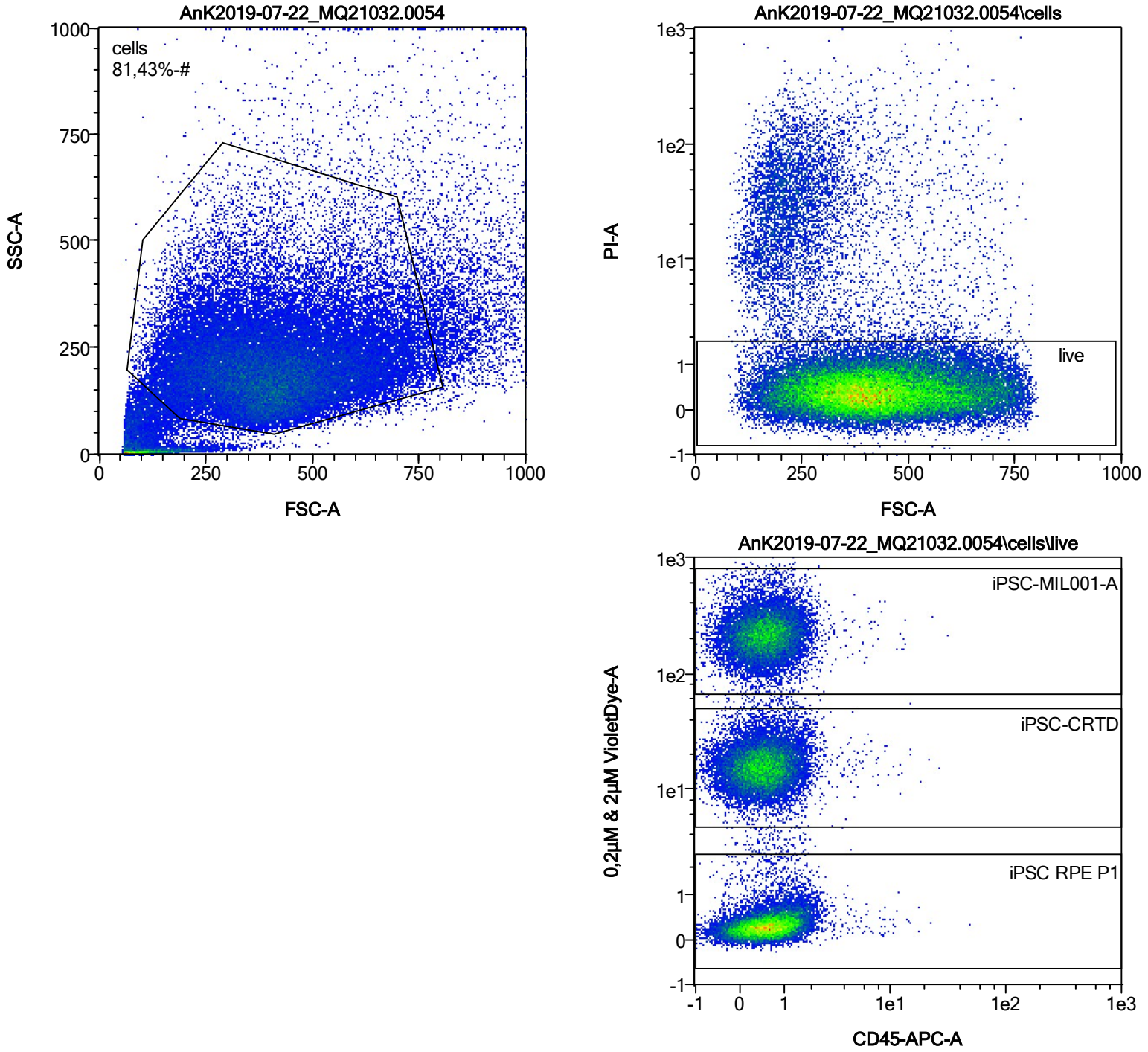

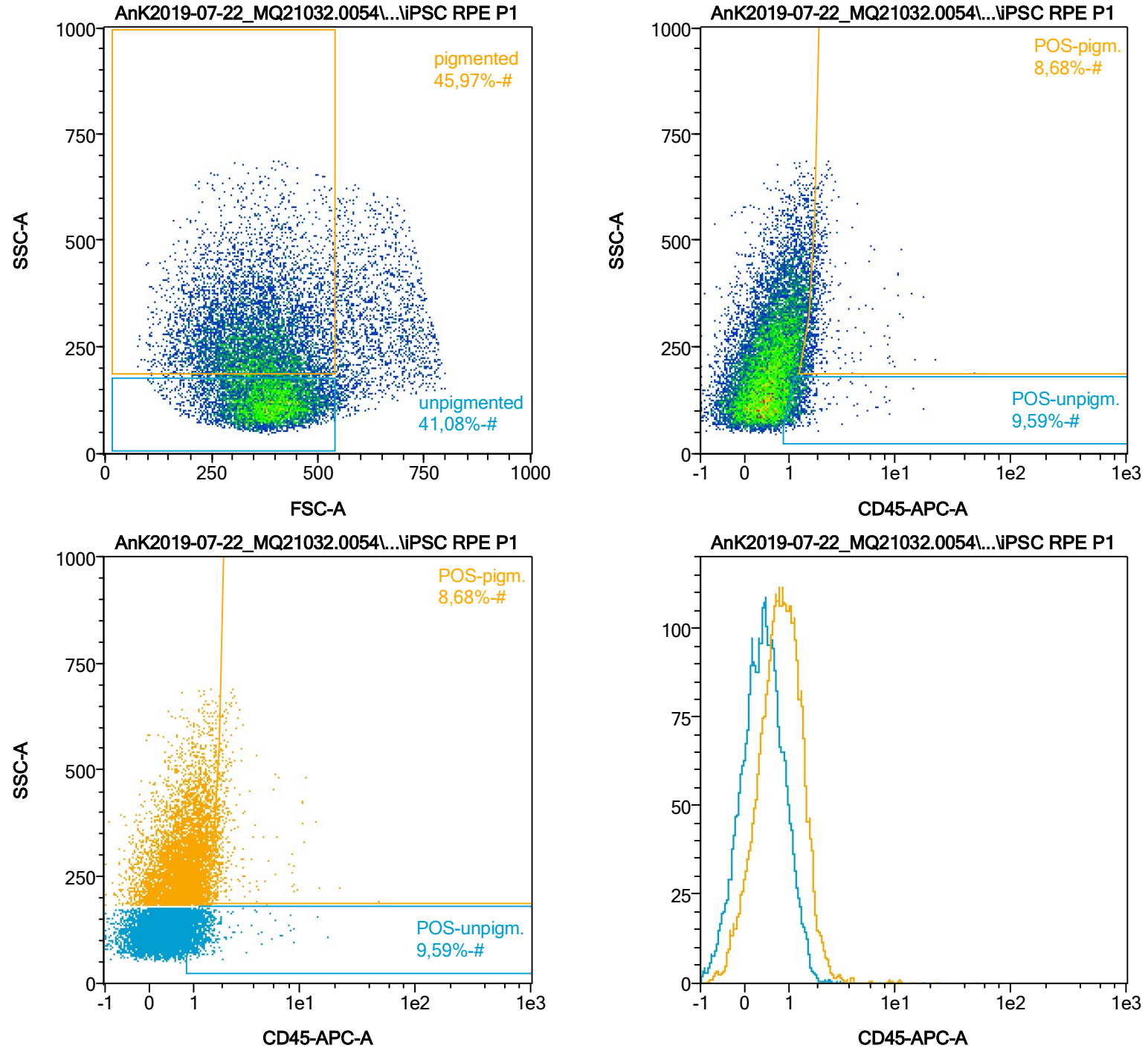

Name	%-#	CD45-APC-A Mean	CD45-APC-A Median
POS-unpigm.	9,59	1,48	1,23
POS-pigm.	8,68	2,31	1,84

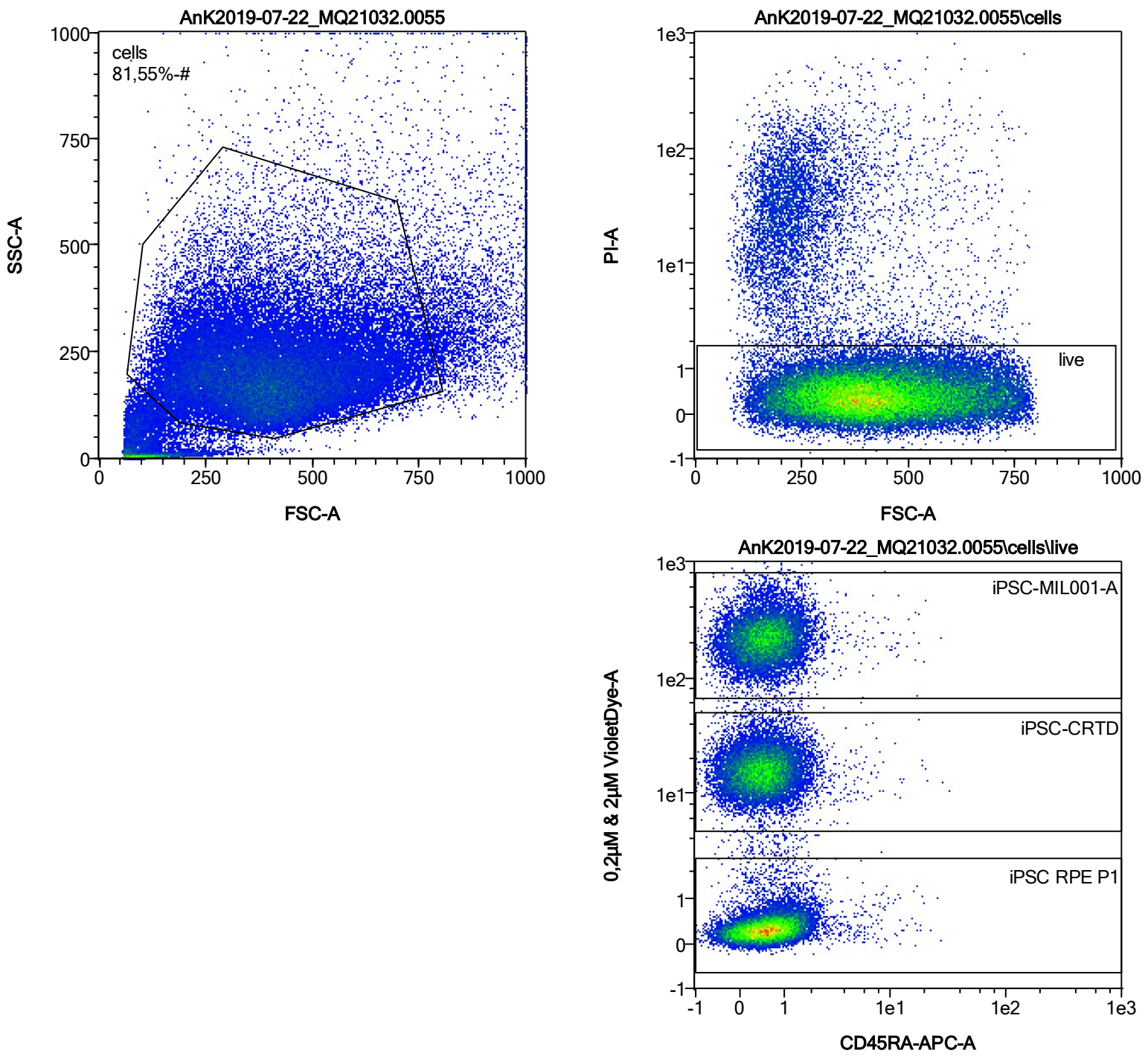

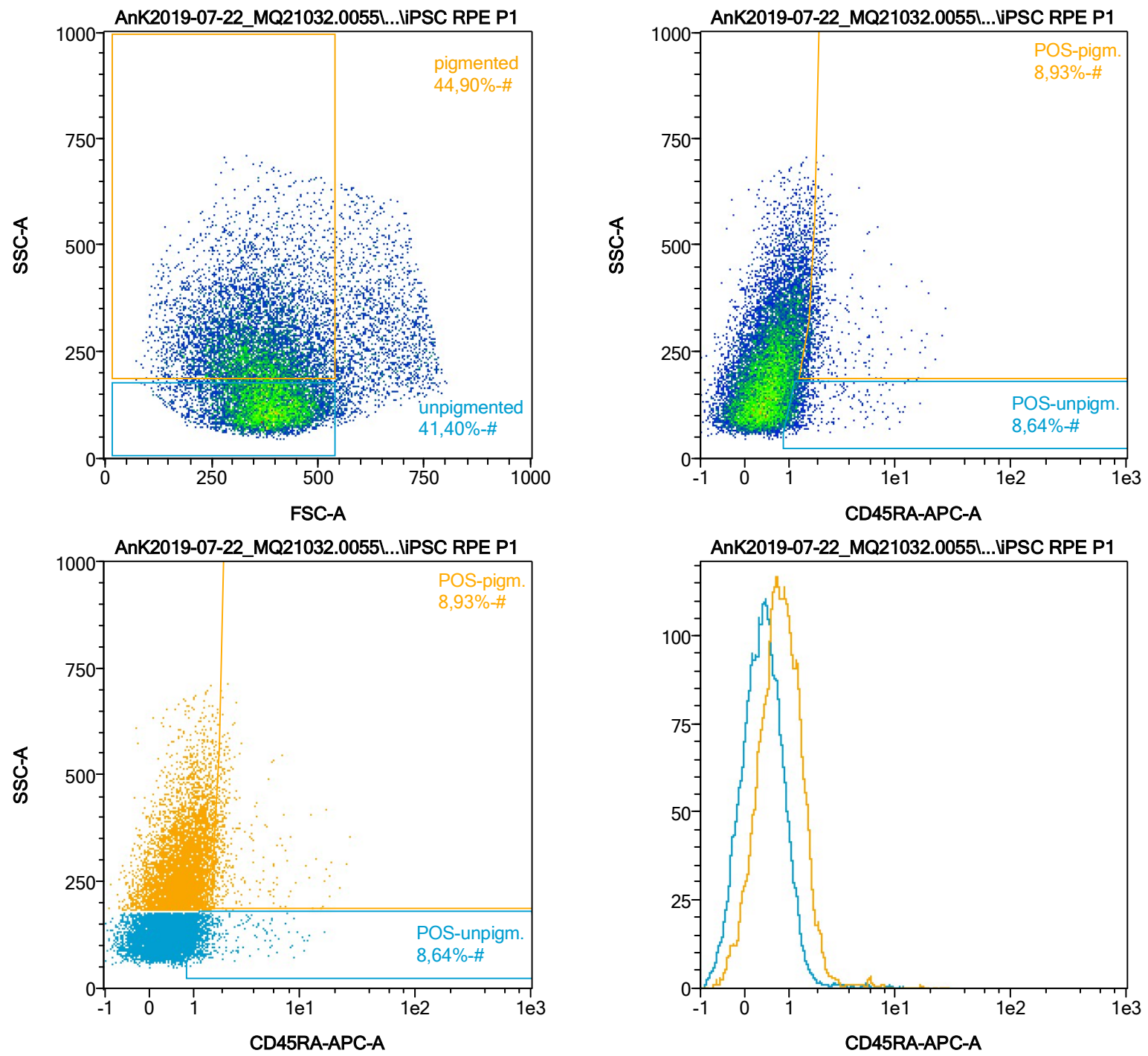

Name	%-#	CD45RA-APC-A Mean	CD45RA-APC-A Median
POS-unpigm.	8,64	1,77	1,25
POS-pigm.	8,93	2,61	1,85

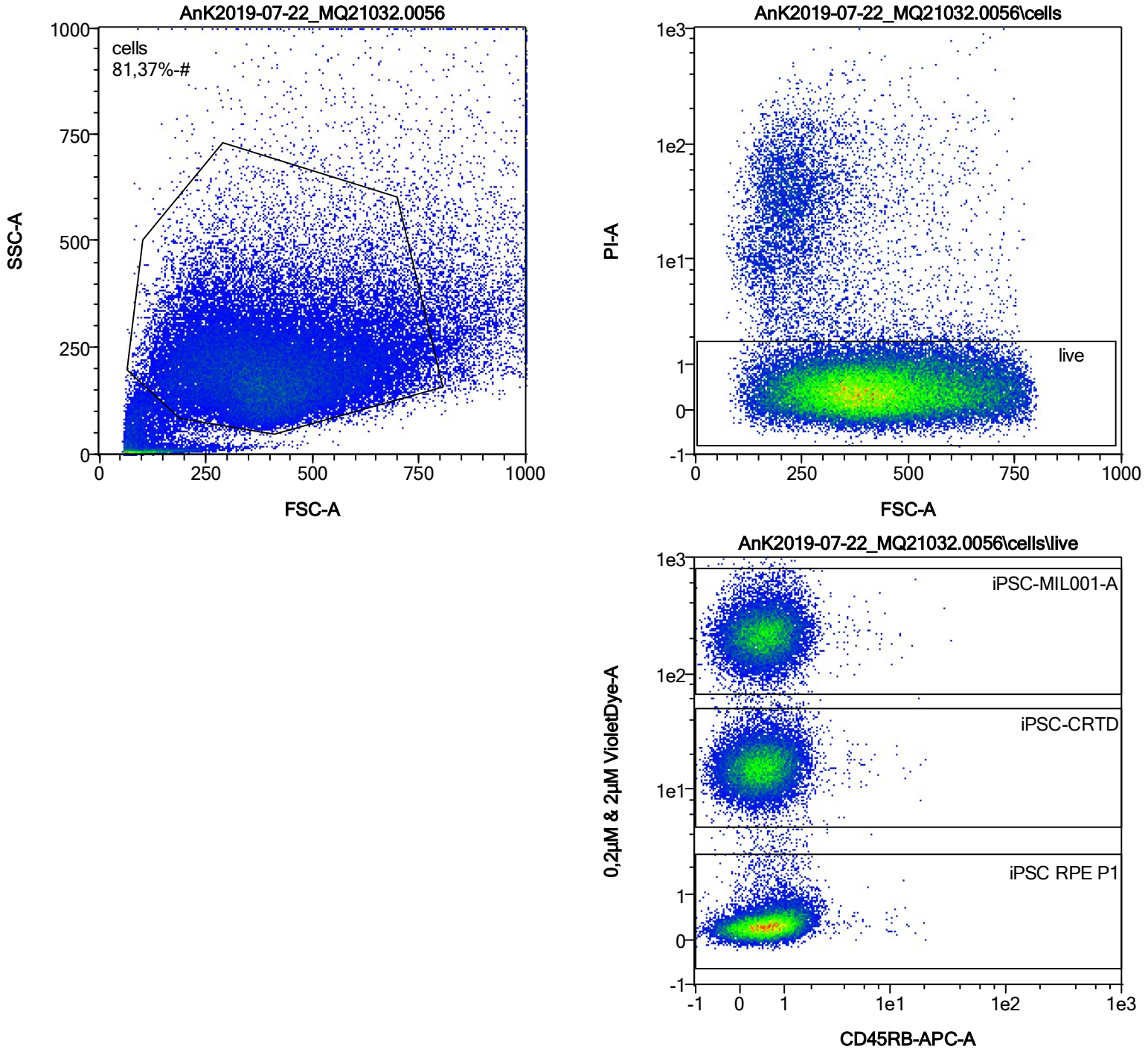

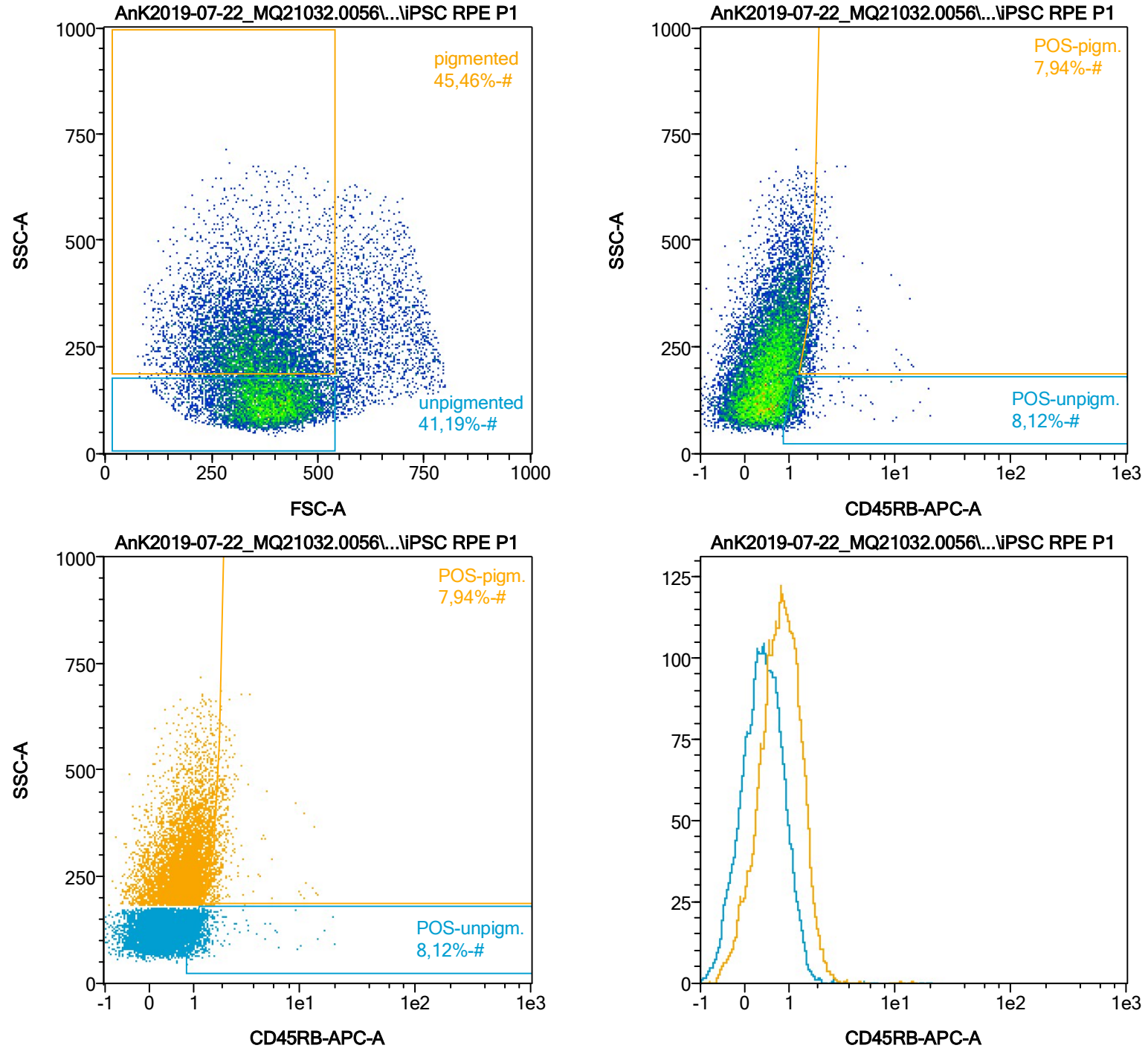

Name	%-#	CD45RB-APC-A Mean	CD45RB-APC-A Median
POS-unpigm.	8,12	1,54	1,23
POS-pigm.	7,94	2,08	1,80

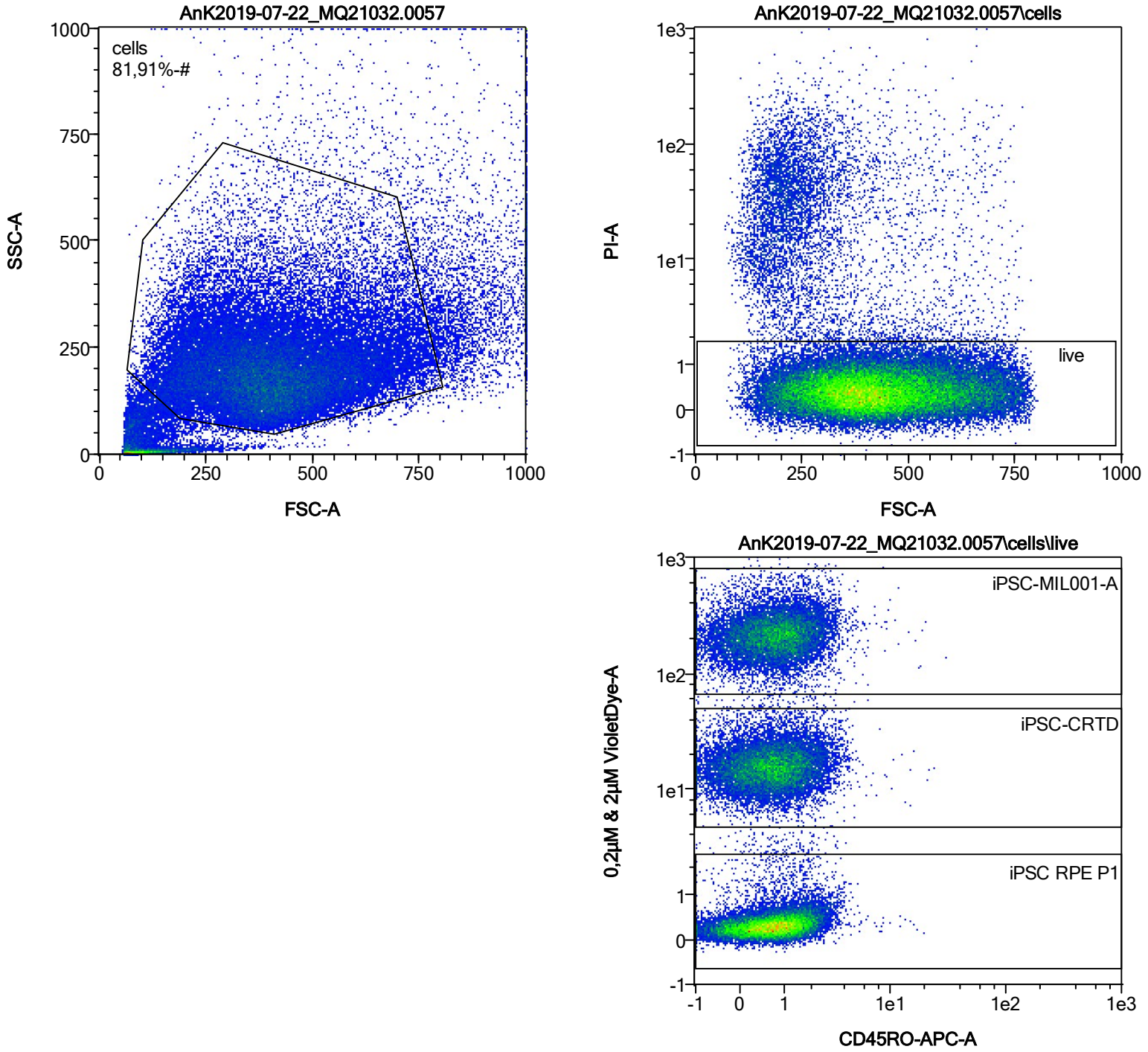

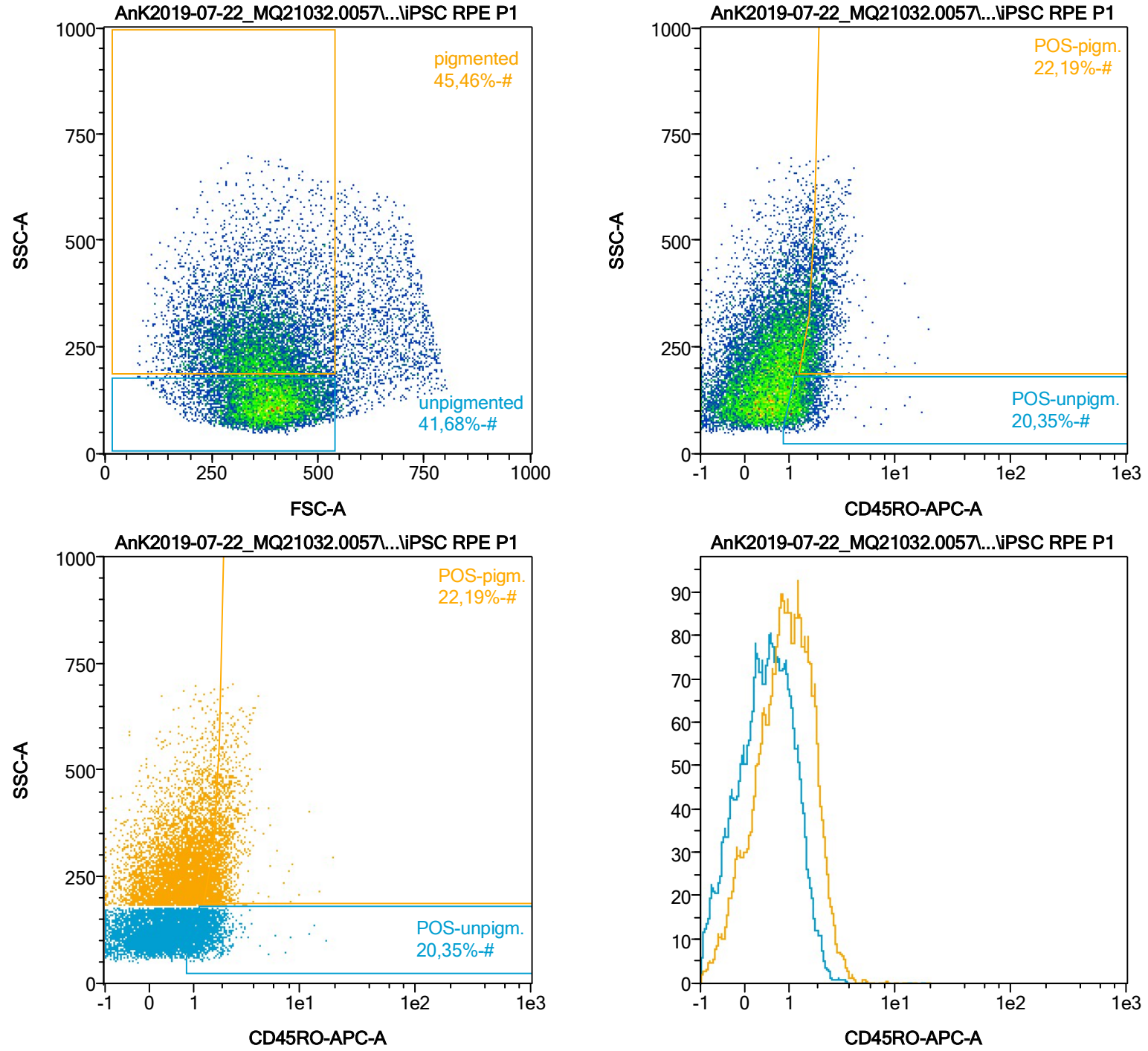

Name	%-#	CD45RO-APC-A Mean	CD45RO-APC-A Median
POS-unpigm.	20,35	1,47	1,32
POS-pigm.	22,19	2,10	1,93

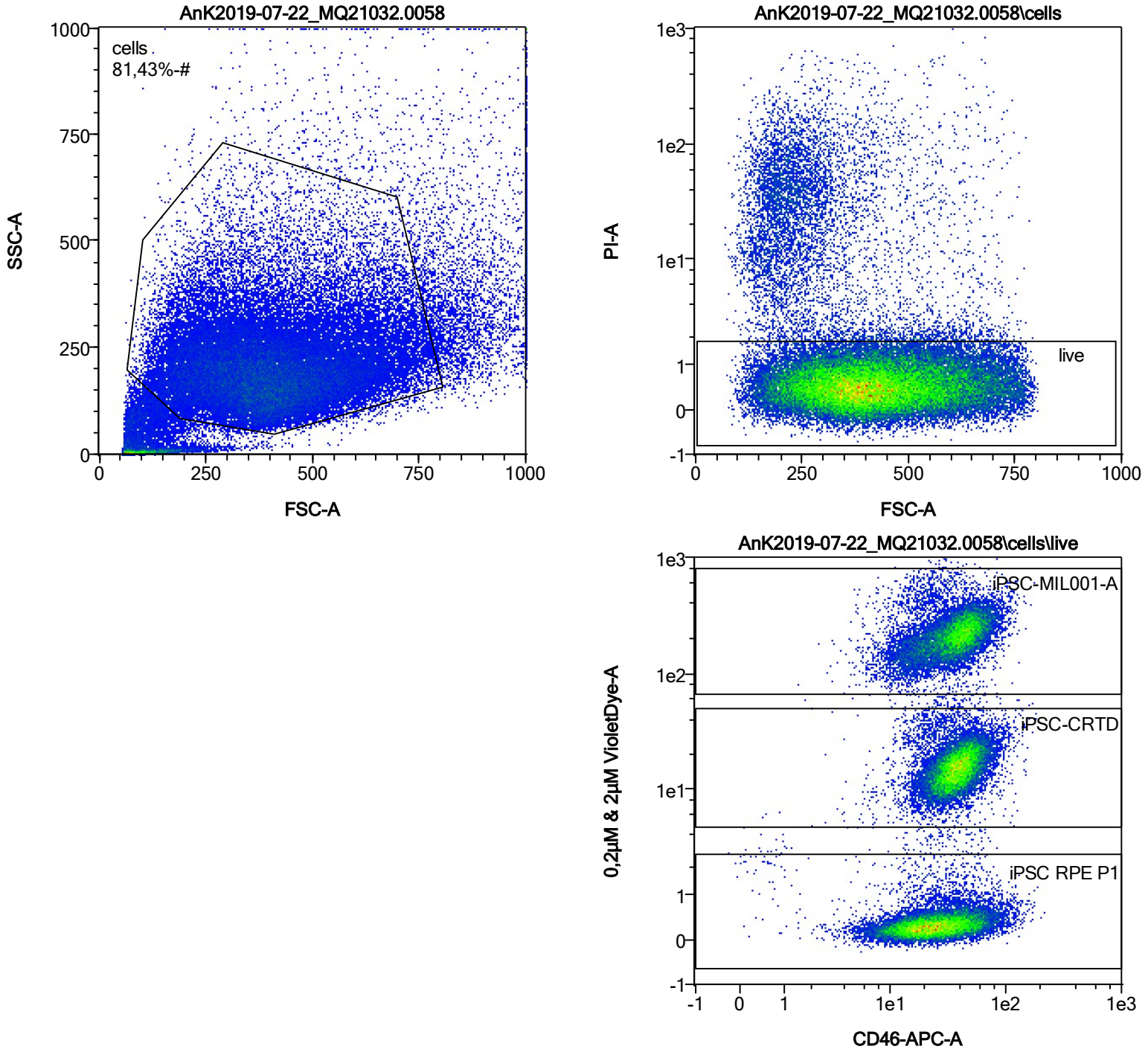

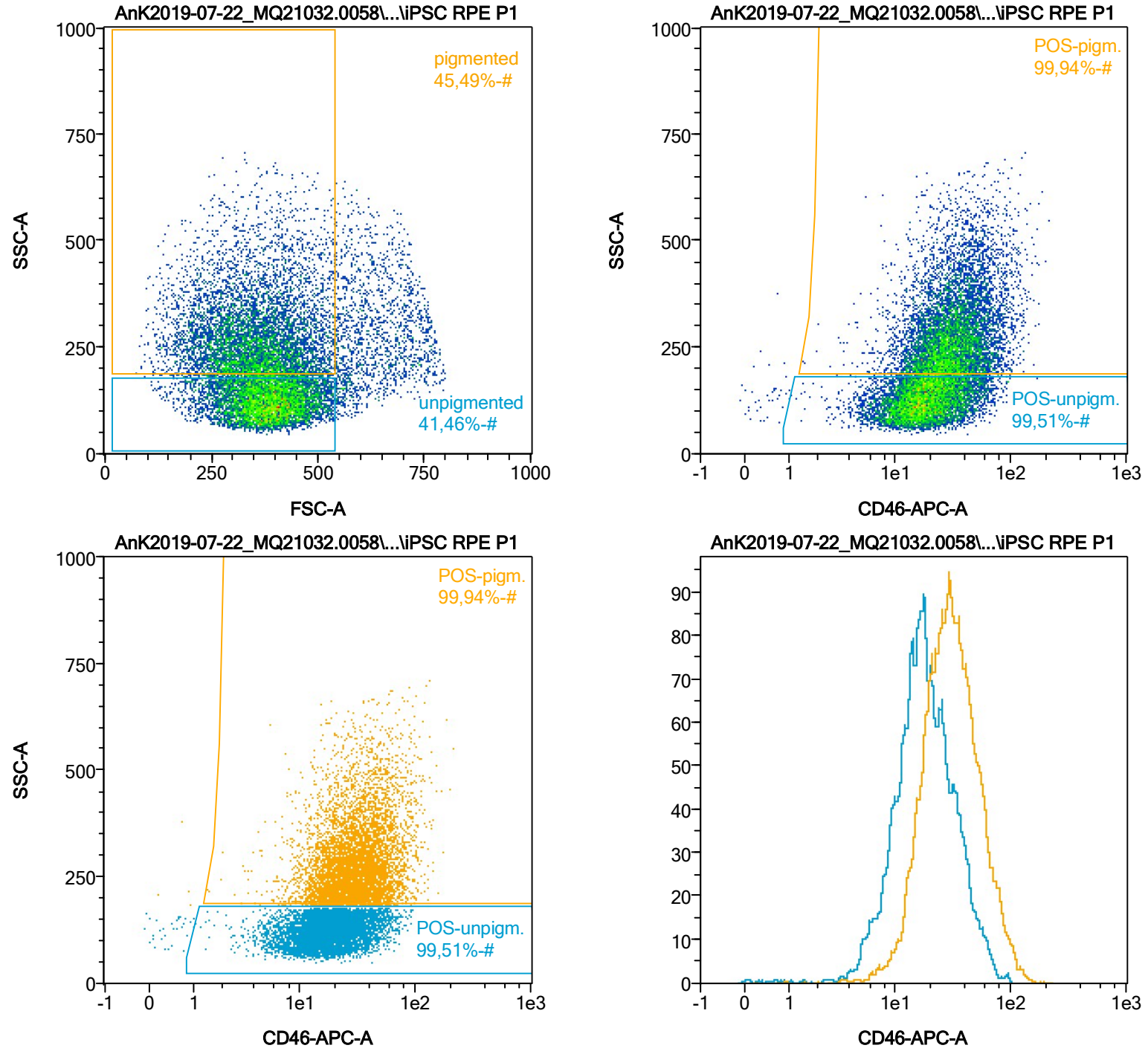

Name	%-#	CD46-APC-A Mean	CD46-APC-A Median
POS-unpigm.	99,51	20,96	17,66
POS-pigm.	99,94	34,77	29,71

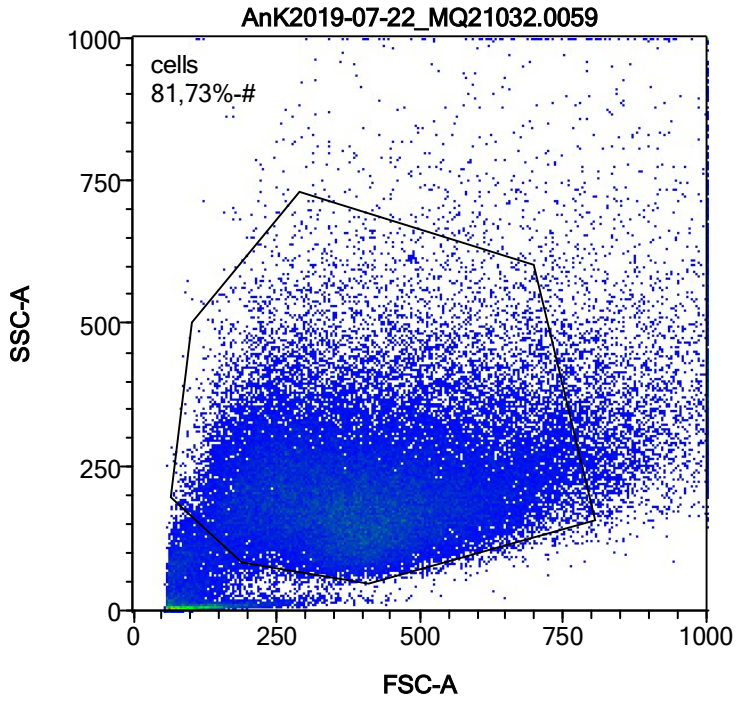

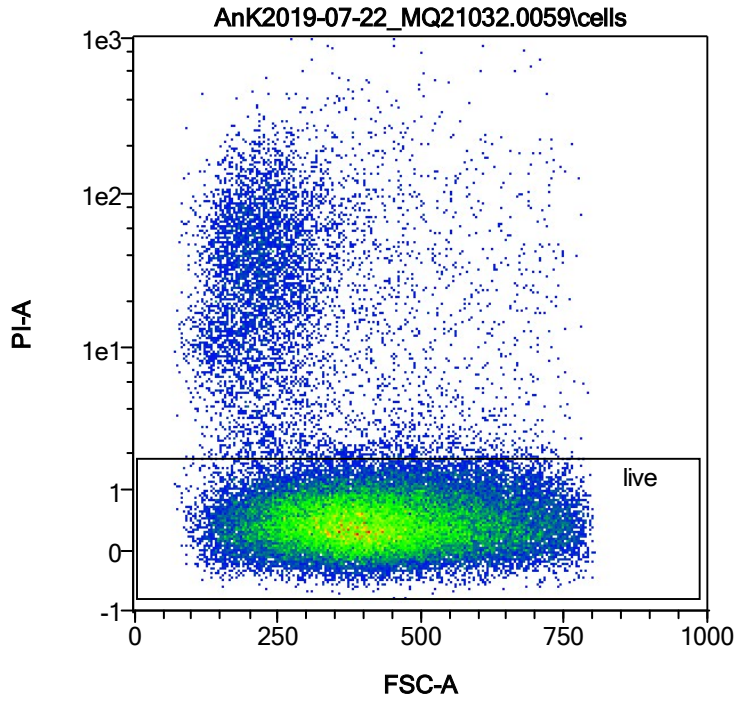

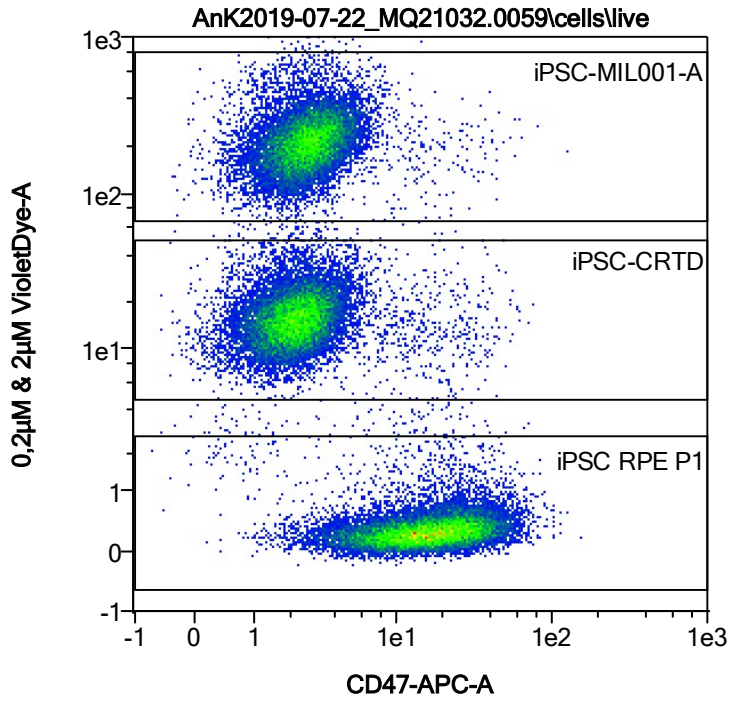

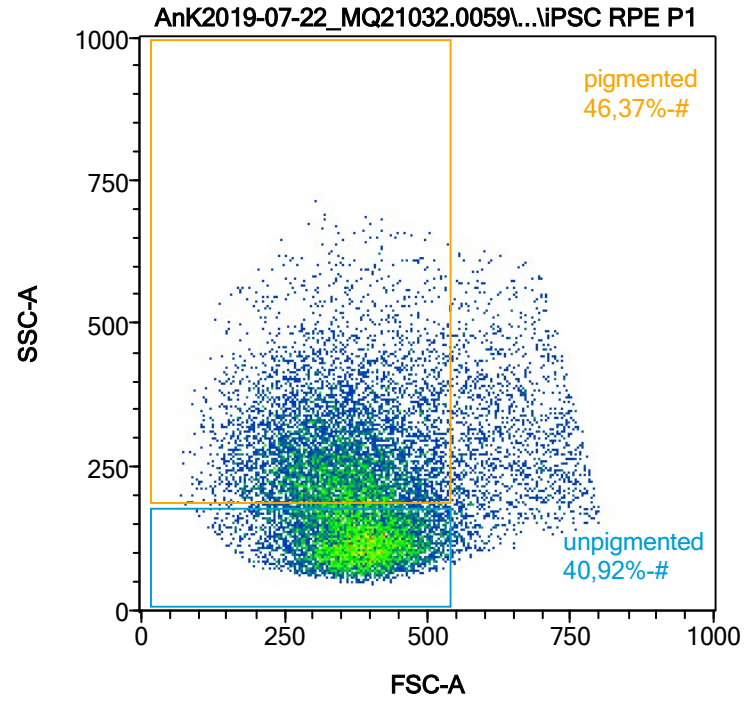

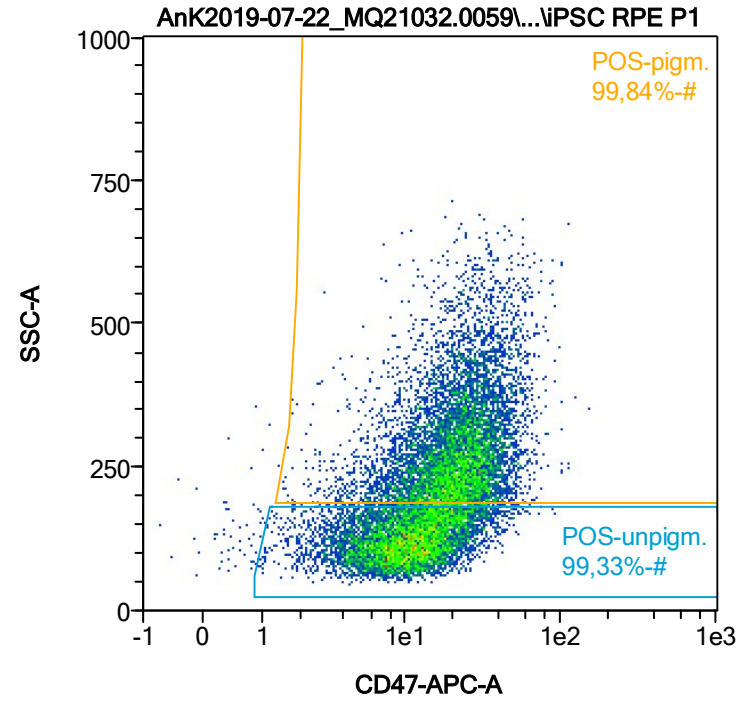

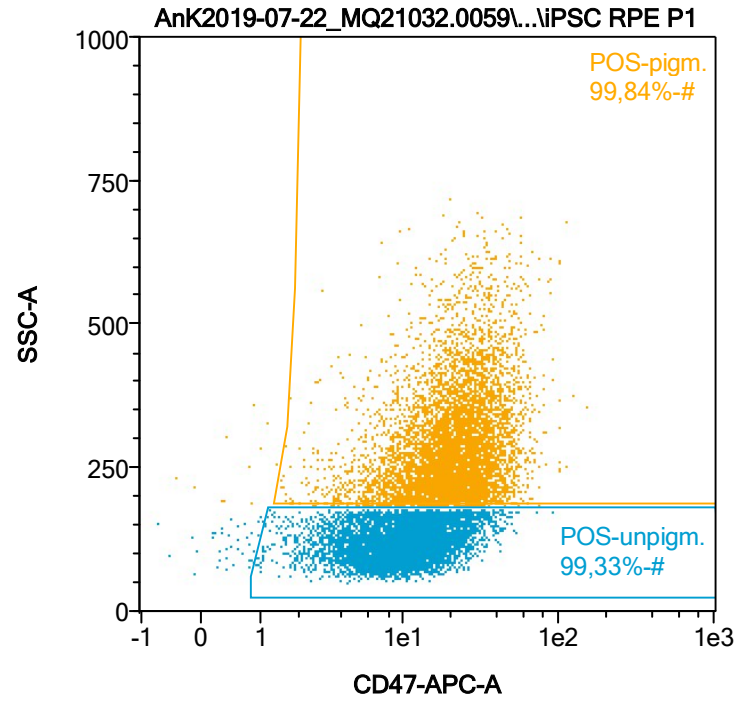

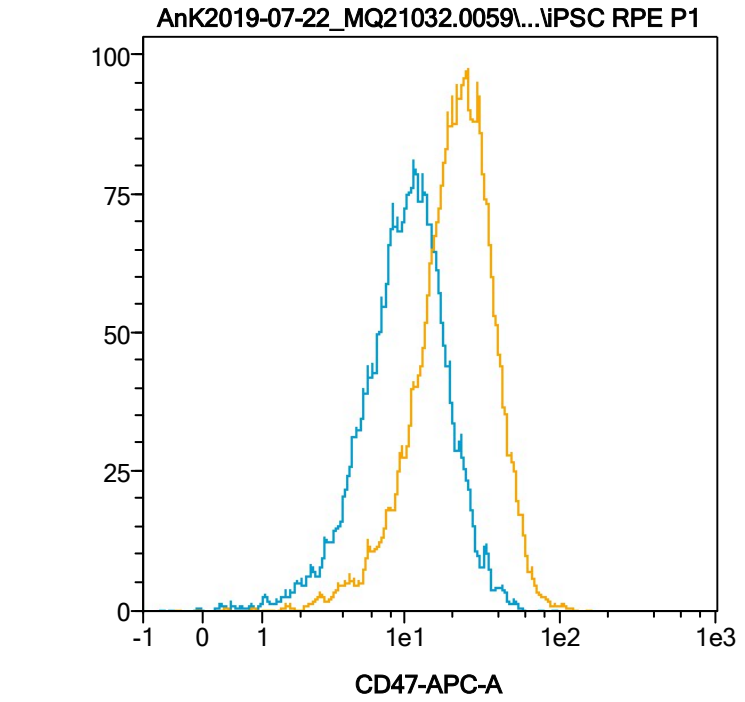

Name	%-#	CD47-APC-A Mean	CD47-APC-A Median
POS-unpigm.	99,33	11,73	10,30
POS-pigm.	99,84	23,80	21,78

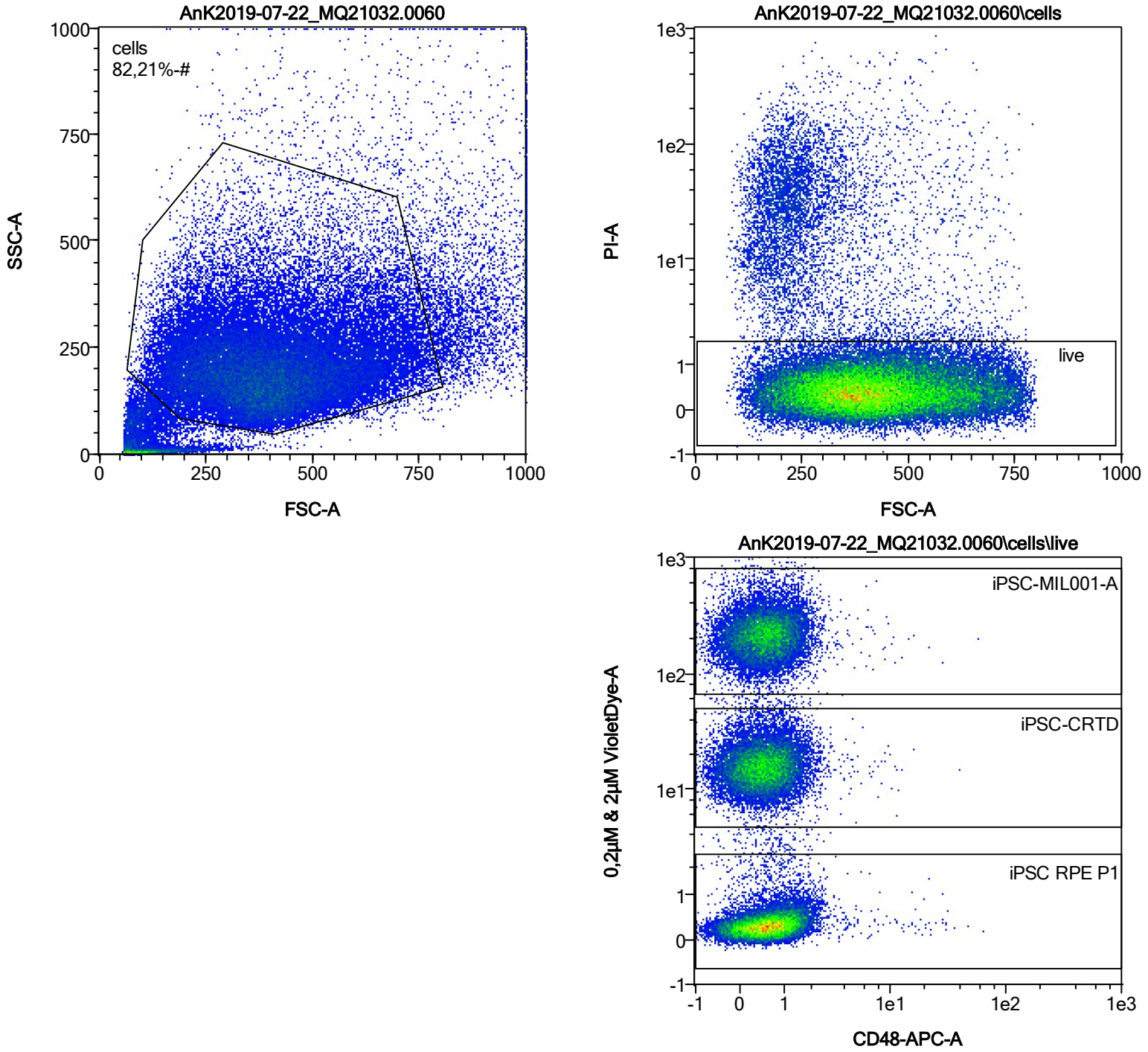

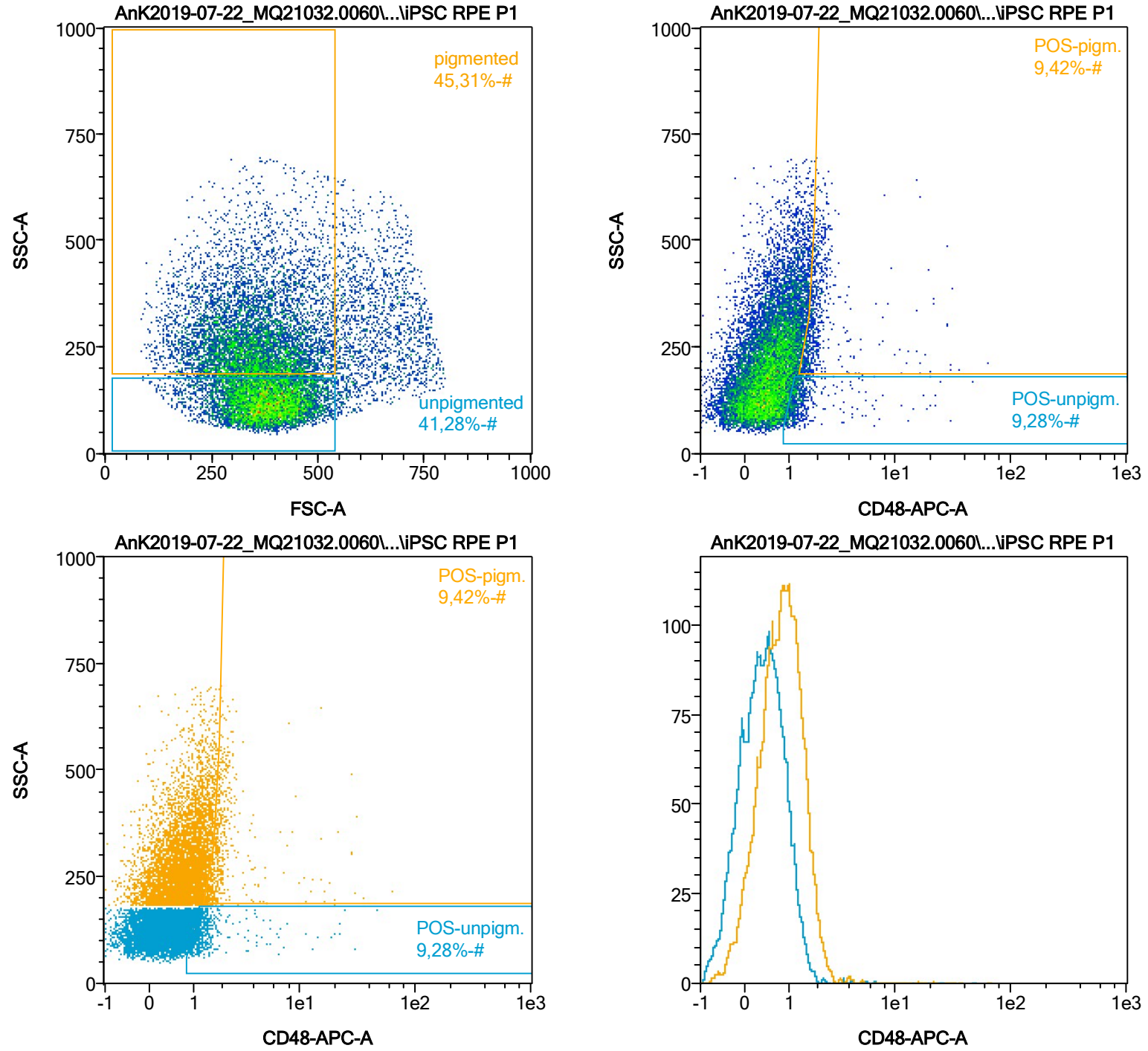

Name	%-#	CD48-APC-A Mean	CD48-APC-A Median
POS-unpigm.	9,28	1,79	1,22
POS-pigm.	9,42	2,65	1,85

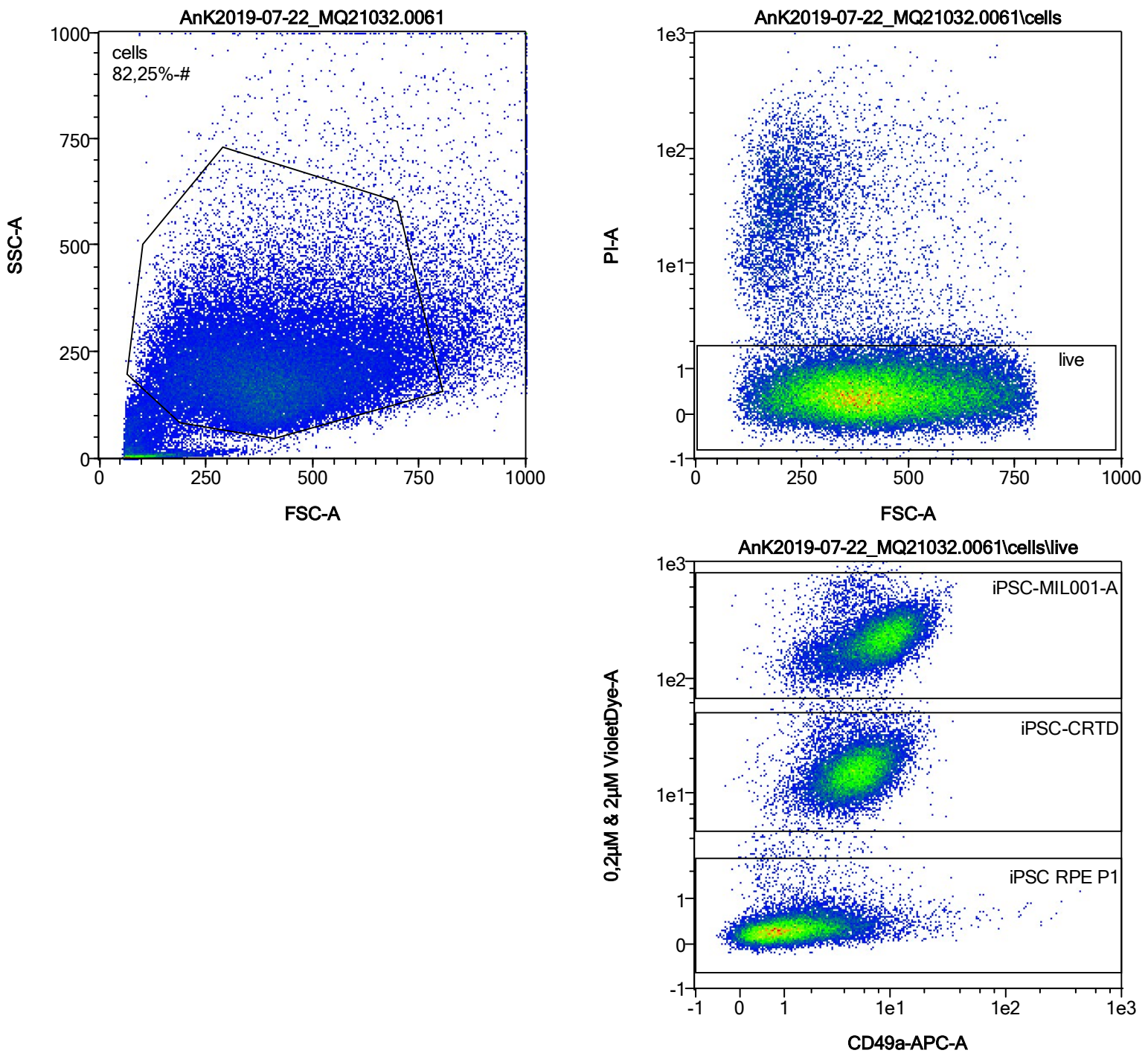

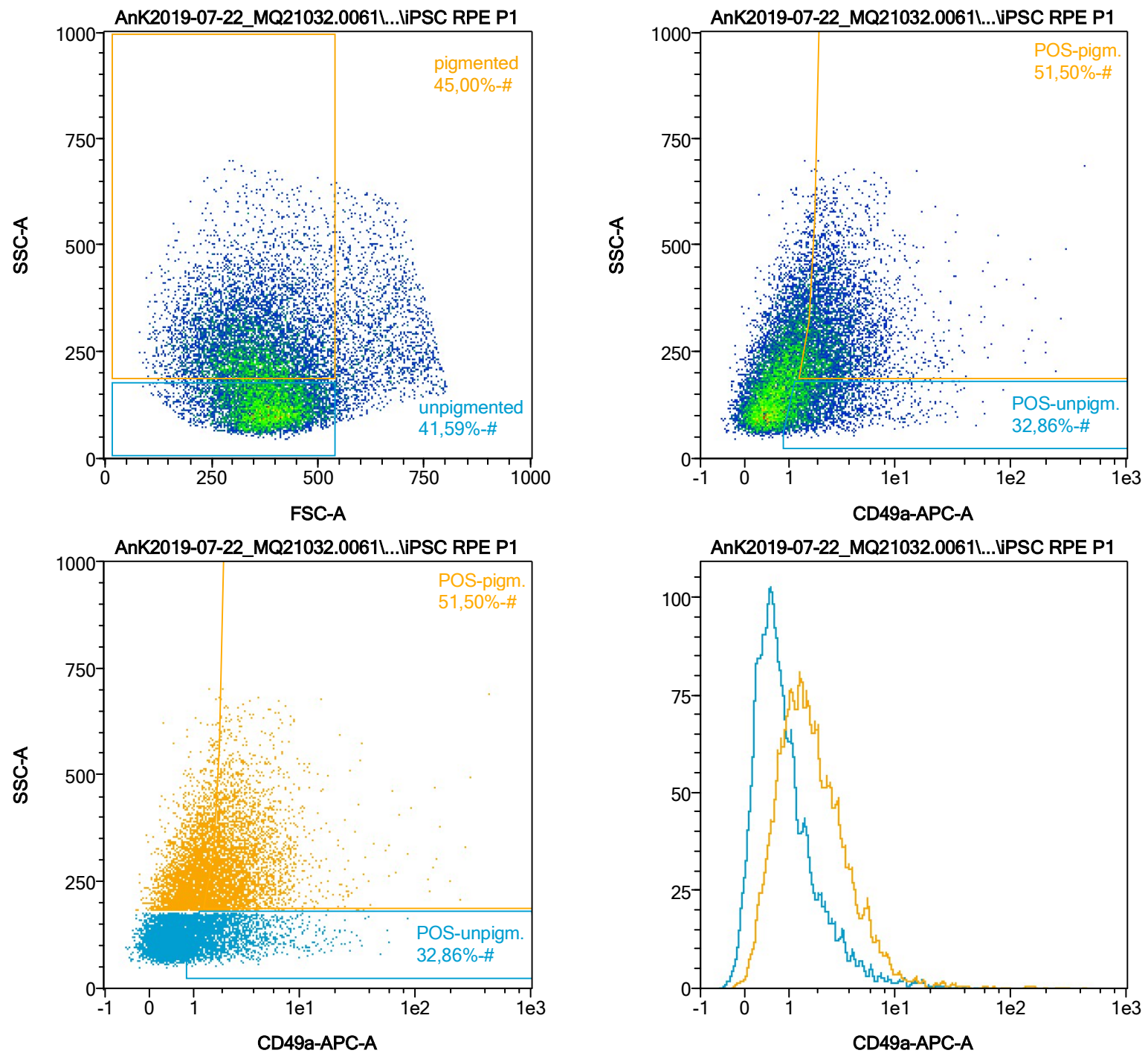

Name	%-#	CD49a-APC-A Mean	CD49a-APC-A Median
POS-unpigm.	32,86	2,81	1,67
POS-pigm.	51,50	4,41	2,65

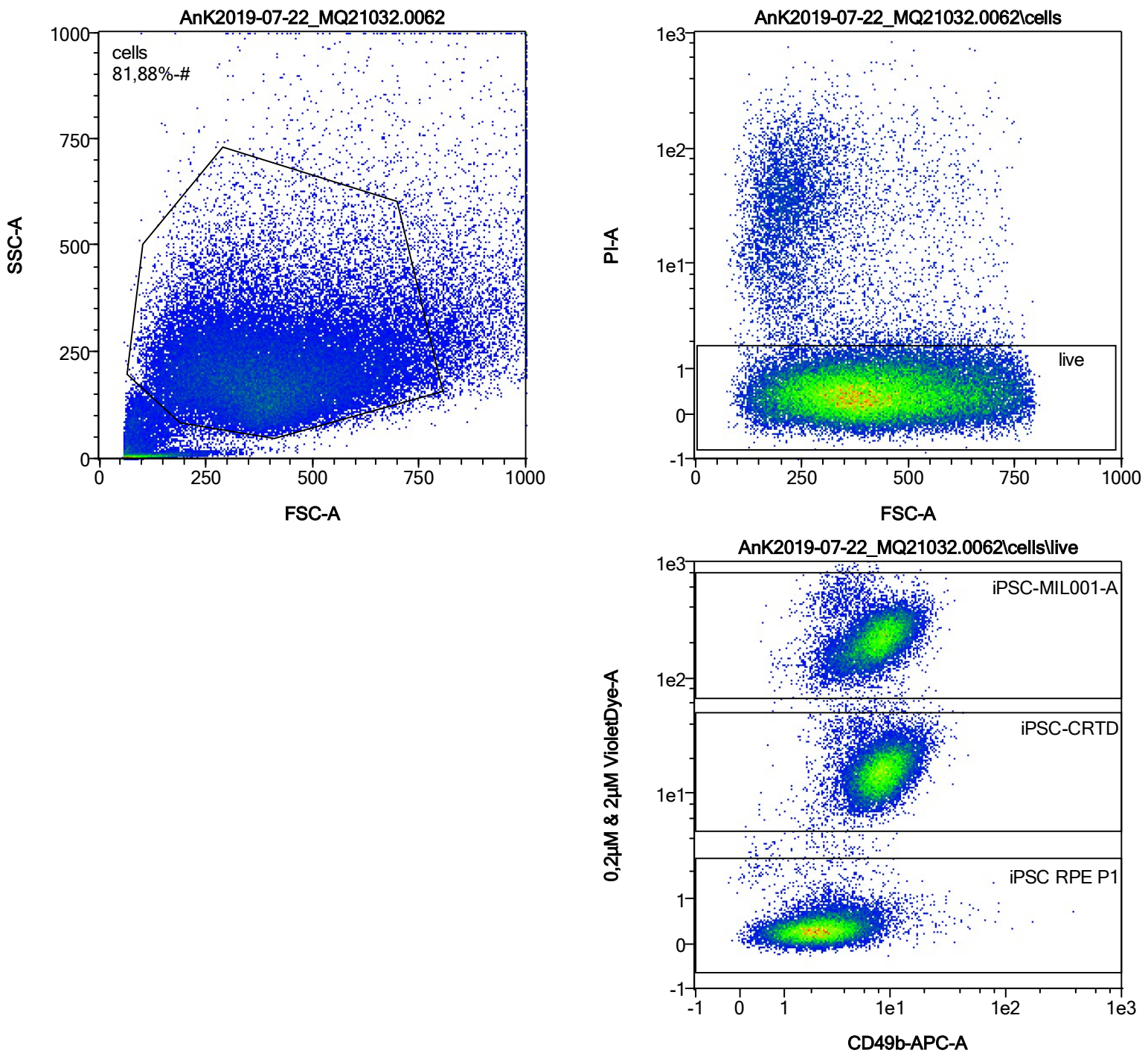

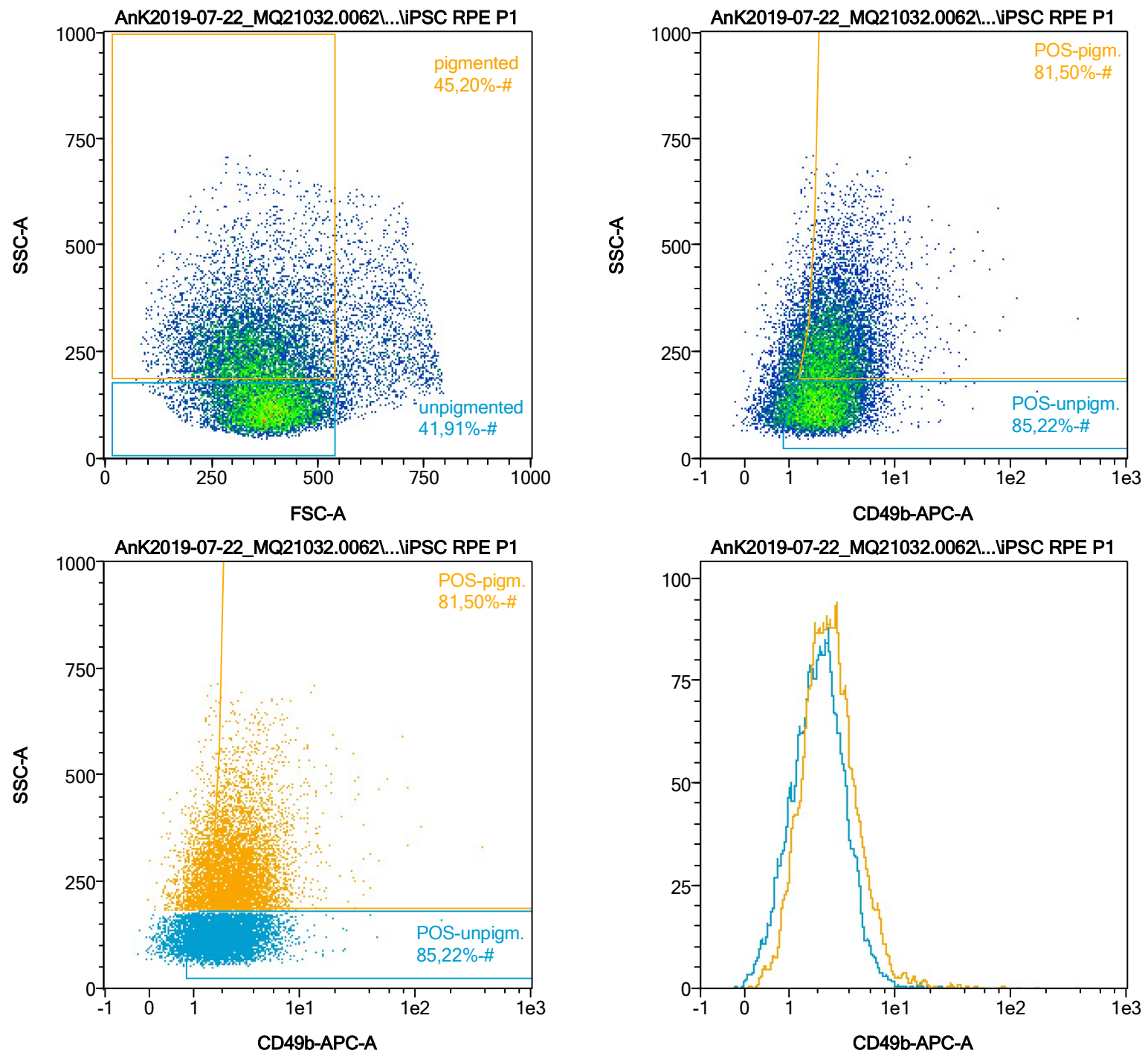

Name	%-#	CD49b-APC-A Mean	CD49b-APC-A Median
POS-unpigm.	85,22	2,66	2,26
POS-pigm.	81,50	3,62	2,84

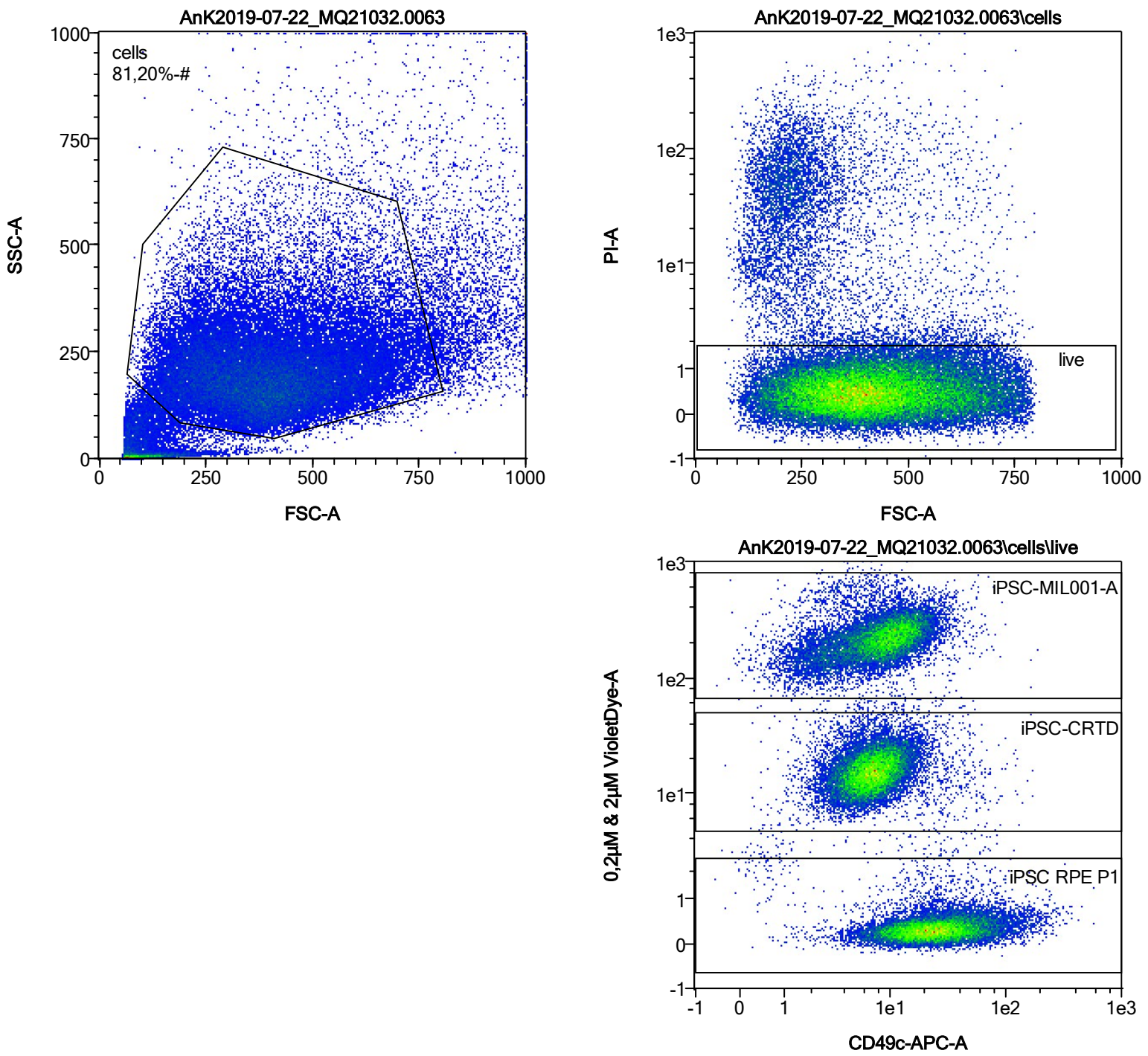

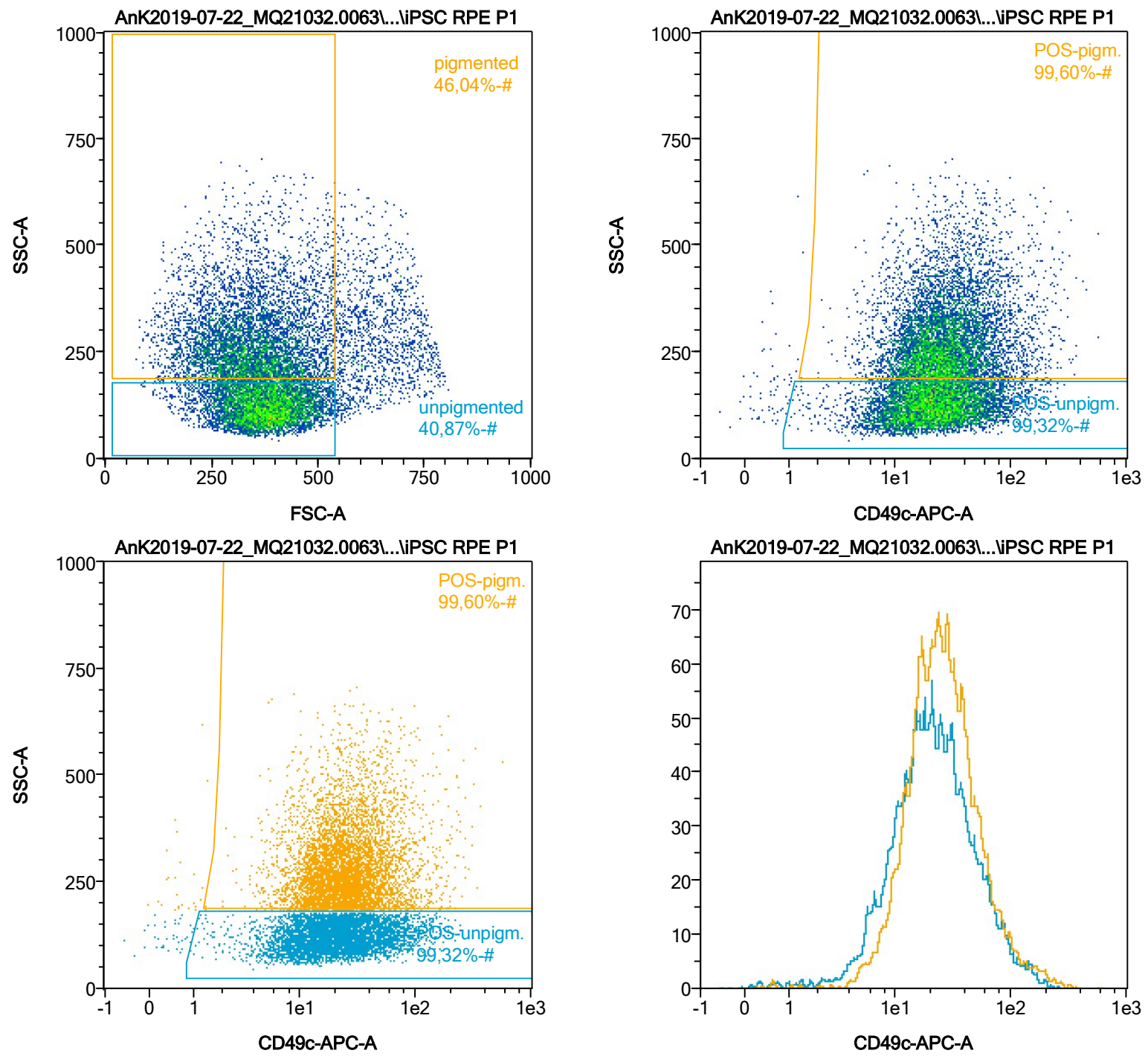

Name	%-#	CD49c-APC-A Mean	CD49c-APC-A Median
POS-unpigm.	99,32	29,08	21,12
POS-pigm.	99,60	32,83	24,43

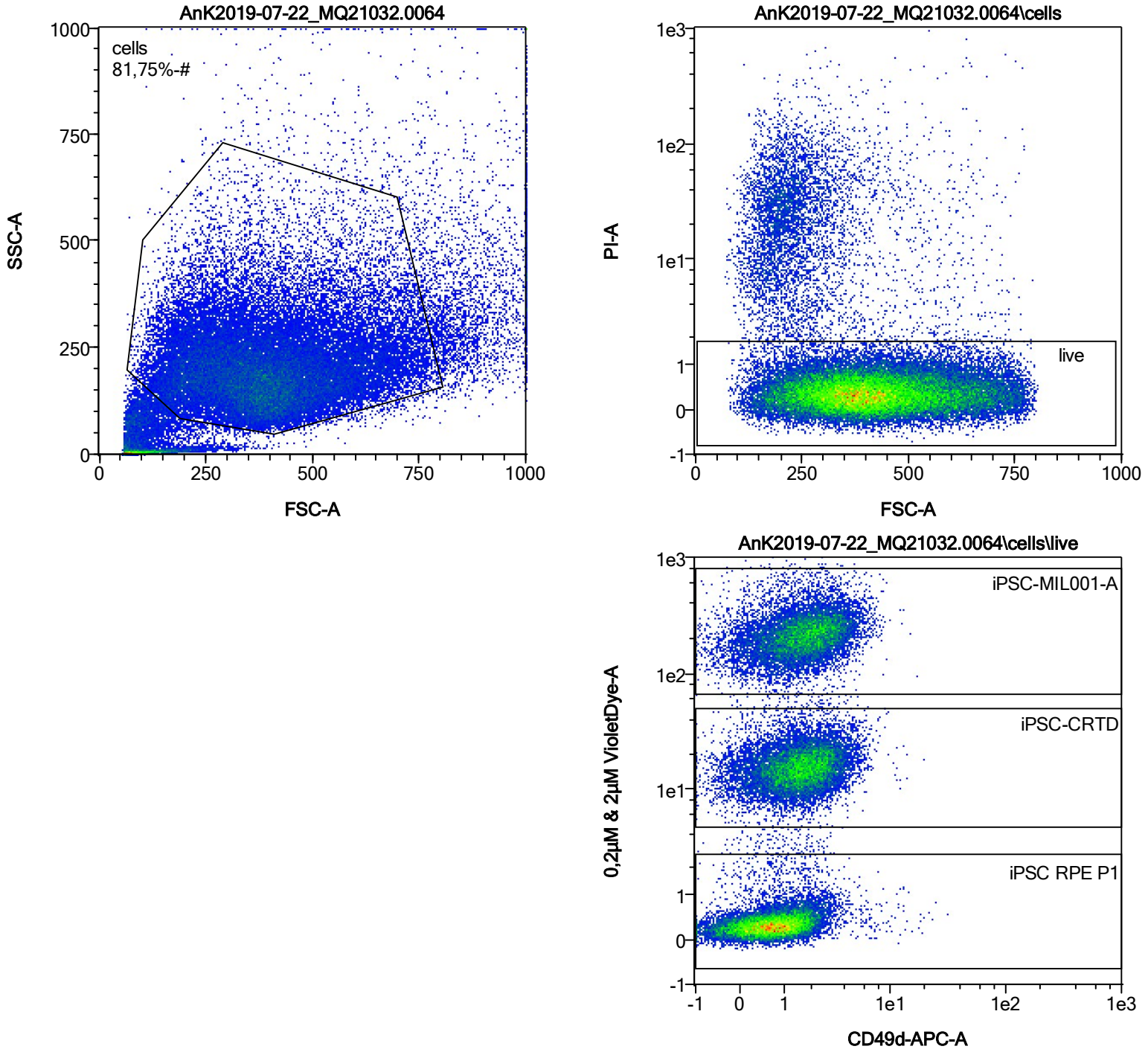

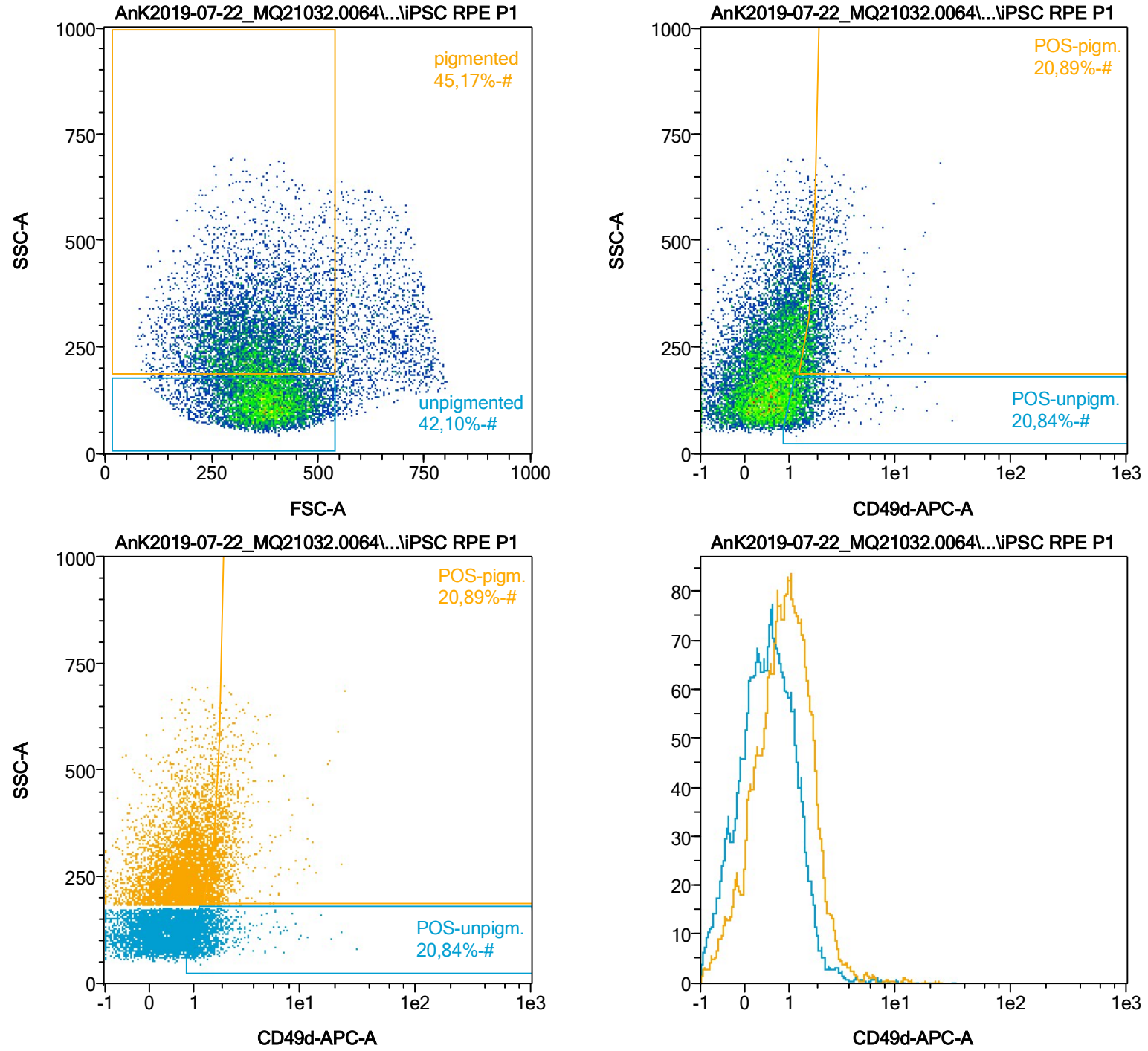

Name	%-#	CD49d-APC-A Mean	CD49d-APC-A Median
POS-unpigm.	20,84	1,63	1,33
POS-pigm.	20,89	2,29	1,92

Date:2024-06-14
Operator: Sebastian Knoebel
Version:2.13.0+54.g95abf0f4

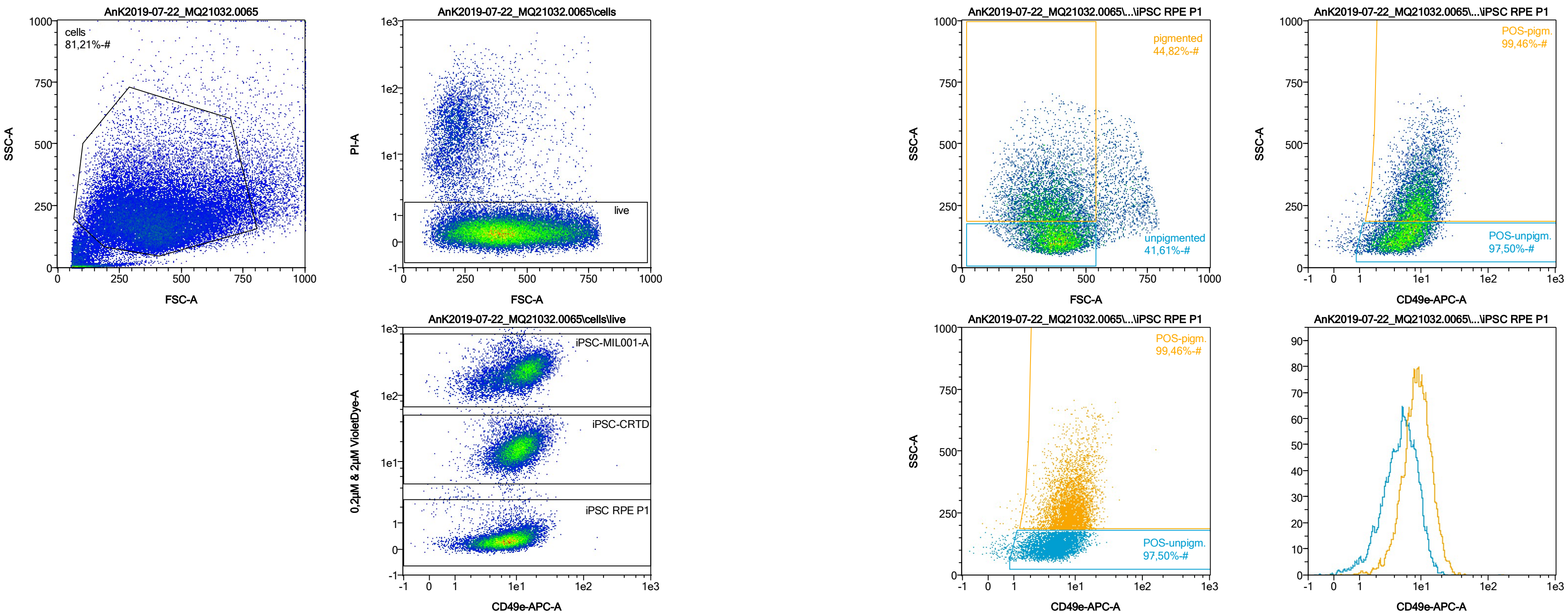

Name	%-#	CD49e-APC-A Mean	CD49e-APC-A Median
POS-unpigm.	97,50	5,64	5,08
POS-pigm.	99,46	9,50	8,67

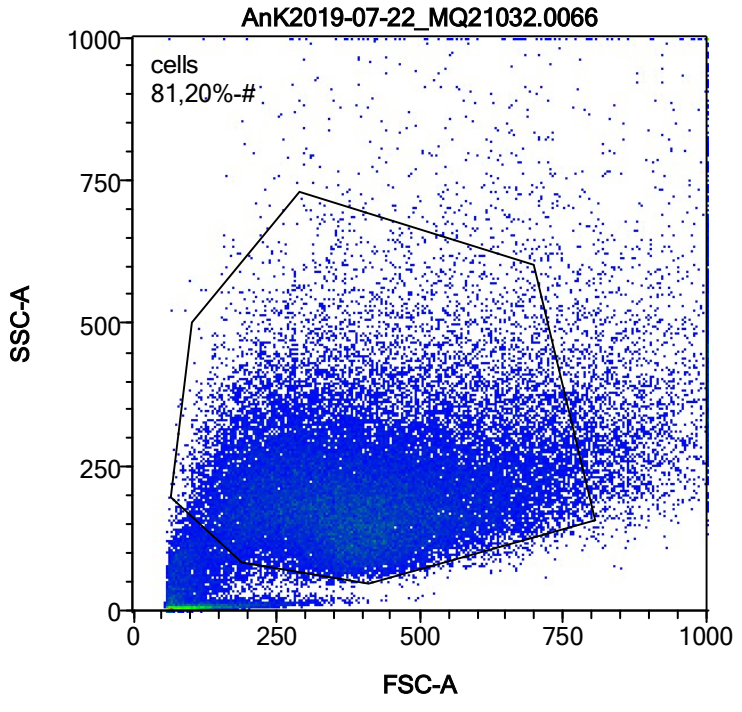

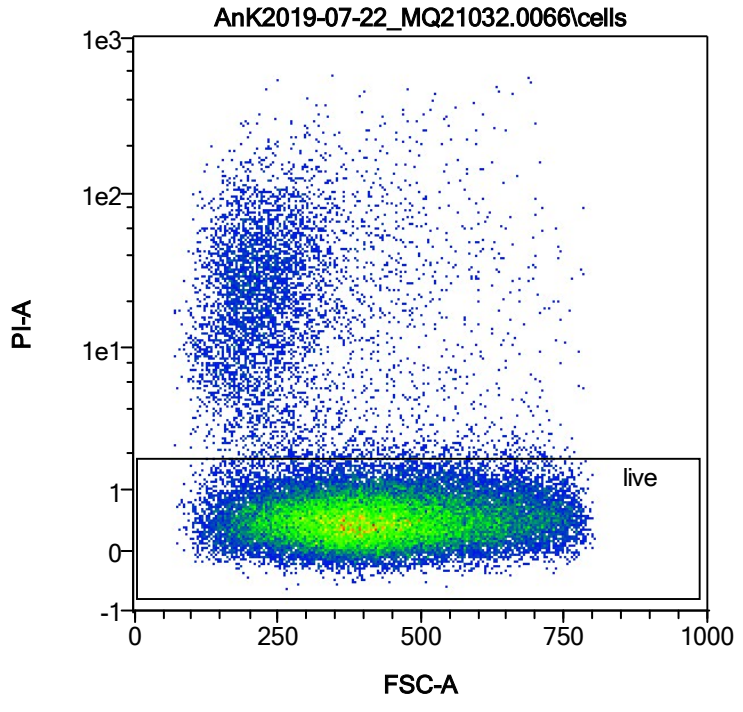

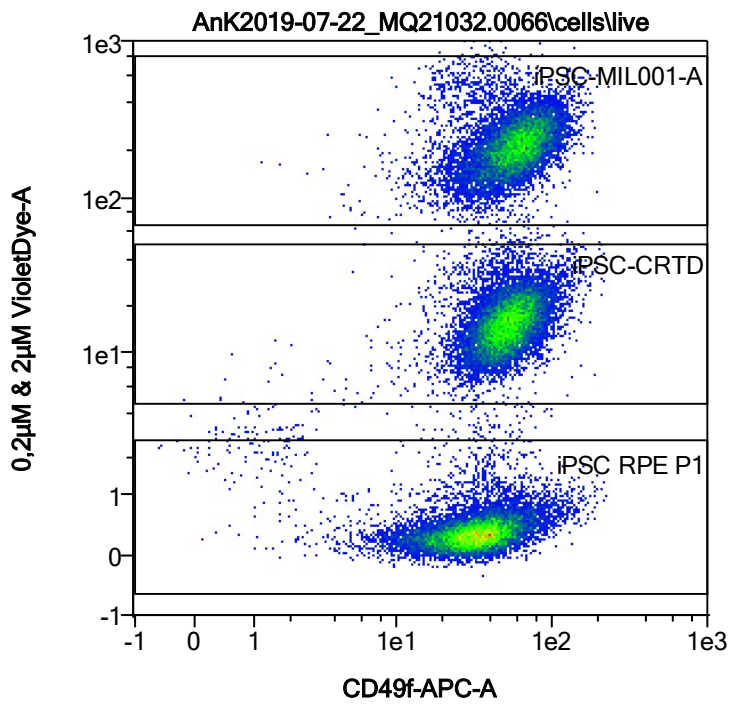

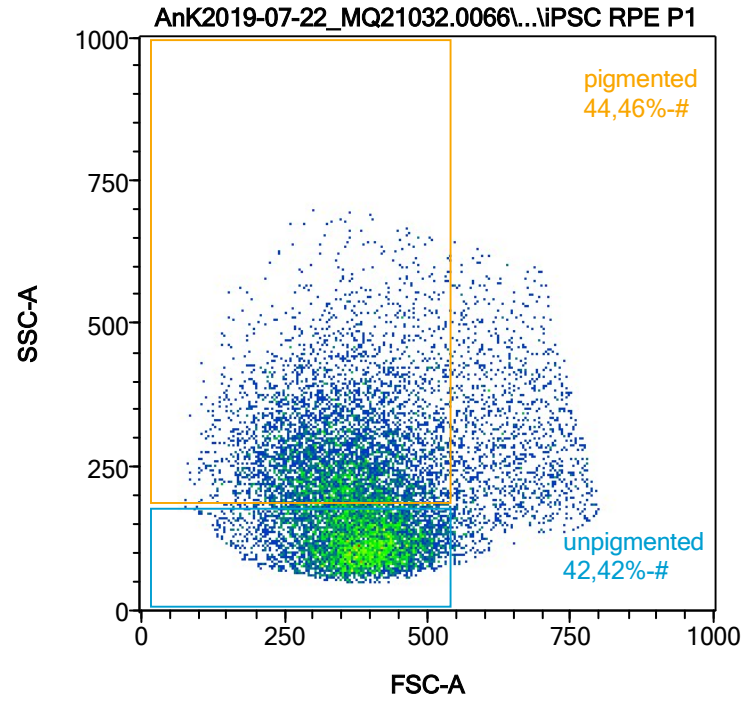

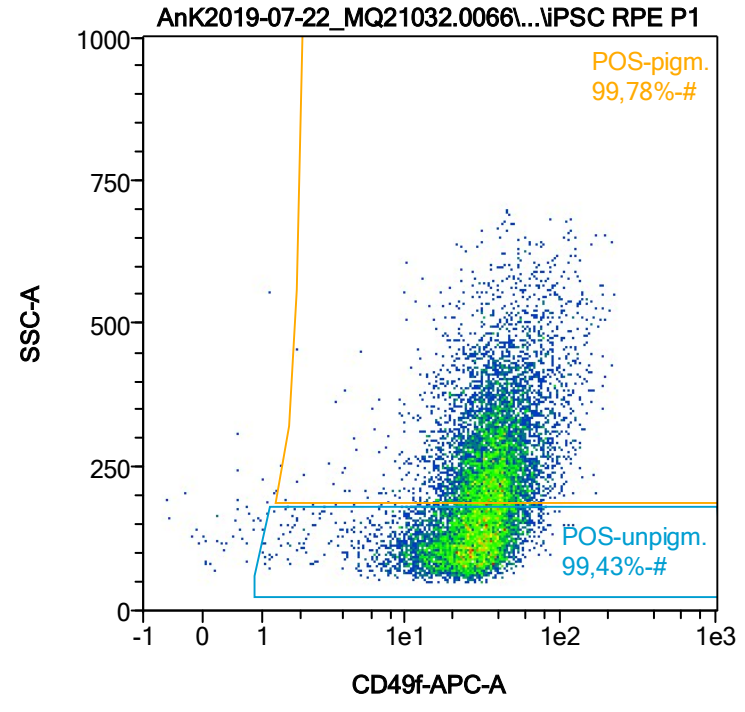

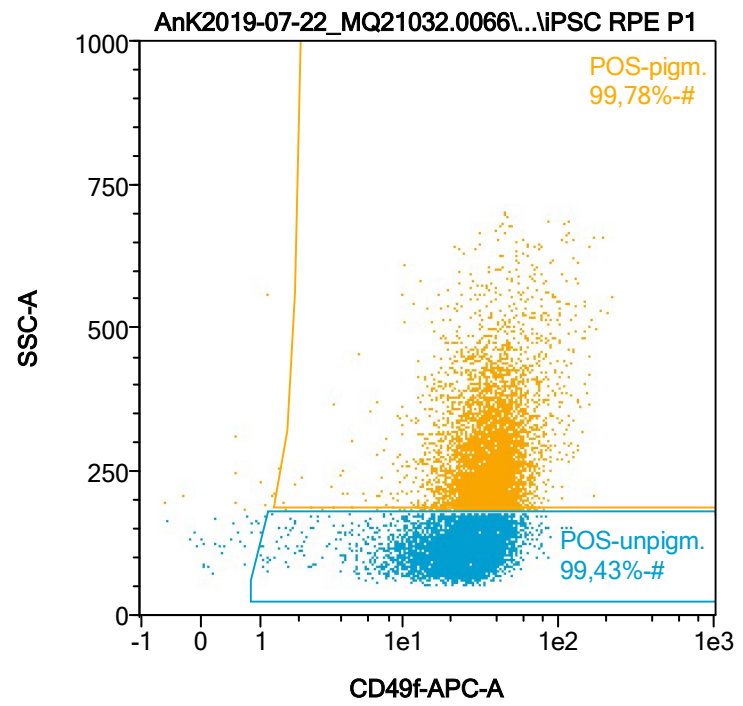

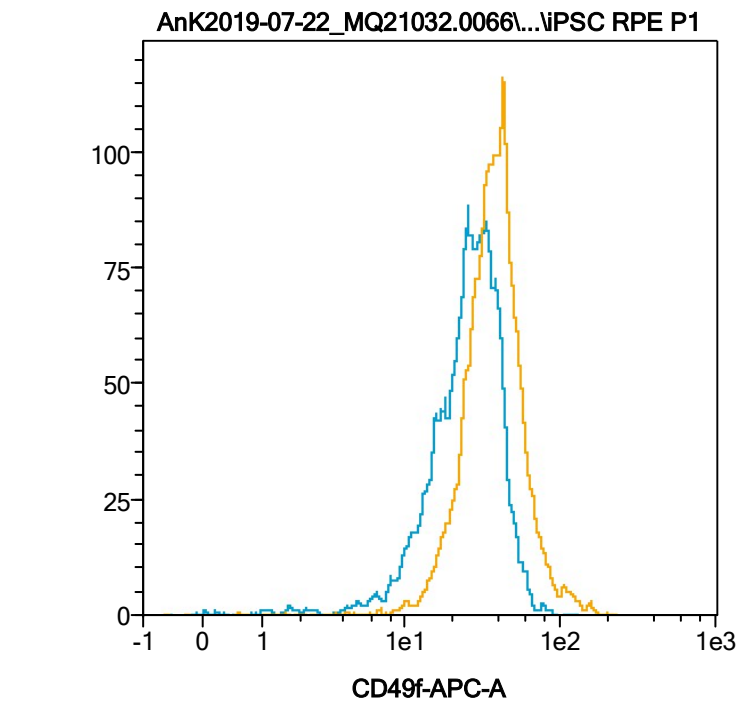

Name	%-#	CD49f-APC-A Mean	CD49f-APC-A Median
POS-unpigm.	99,43	27,05	26,22
POS-pigm.	99,78	39,64	36,92

Date:2024-06-14

Operator: Sebastian Knoebel

Version:2.13.0+54.g95abf0f4

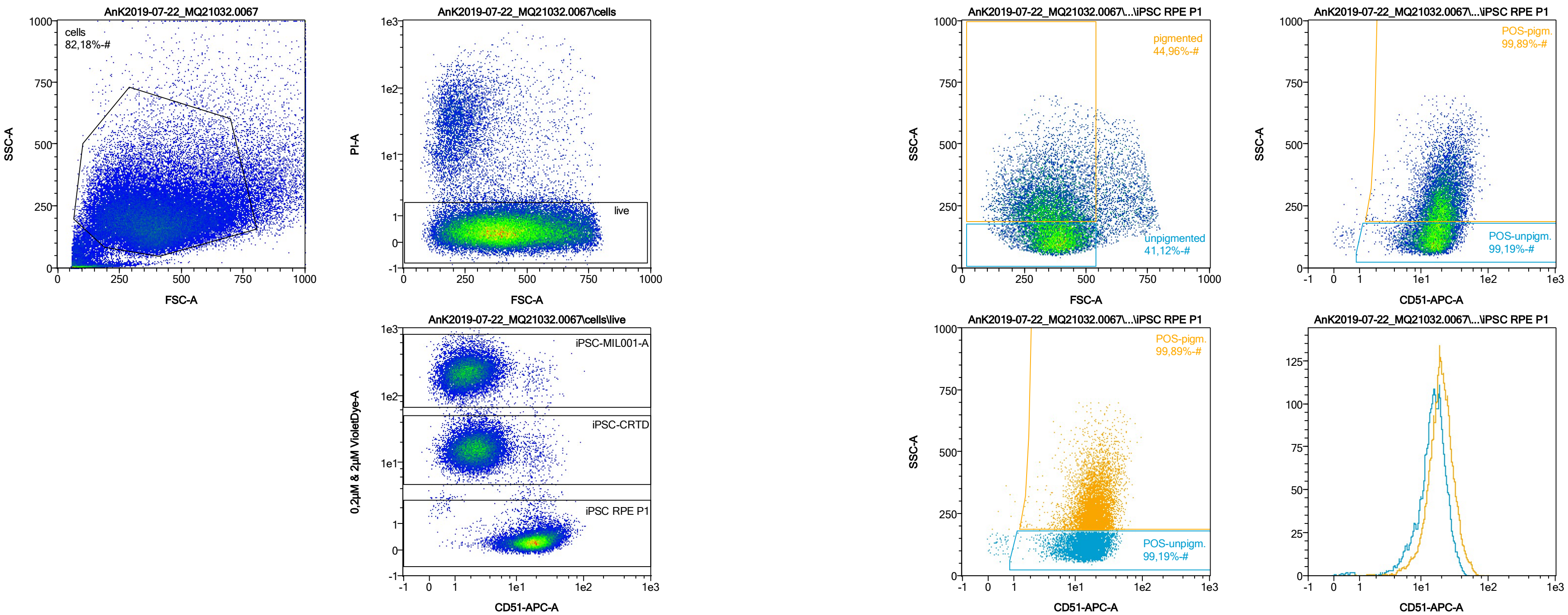

Name	%-#	CD51-APC-A Mean	CD51-APC-A Median
POS-unpigm.	99,19	15,99	15,32
POS-pigm.	99,89	20,87	19,43

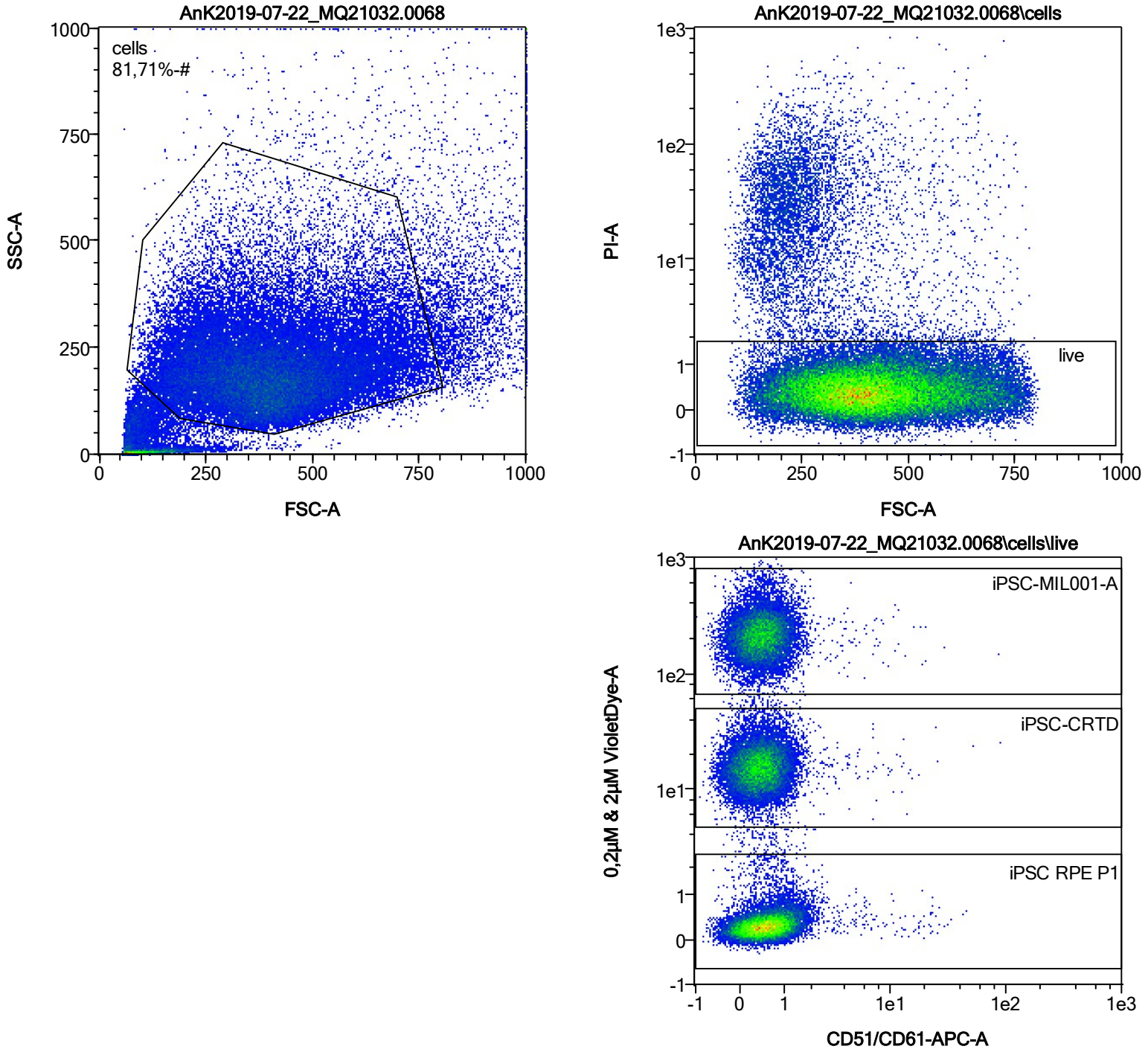

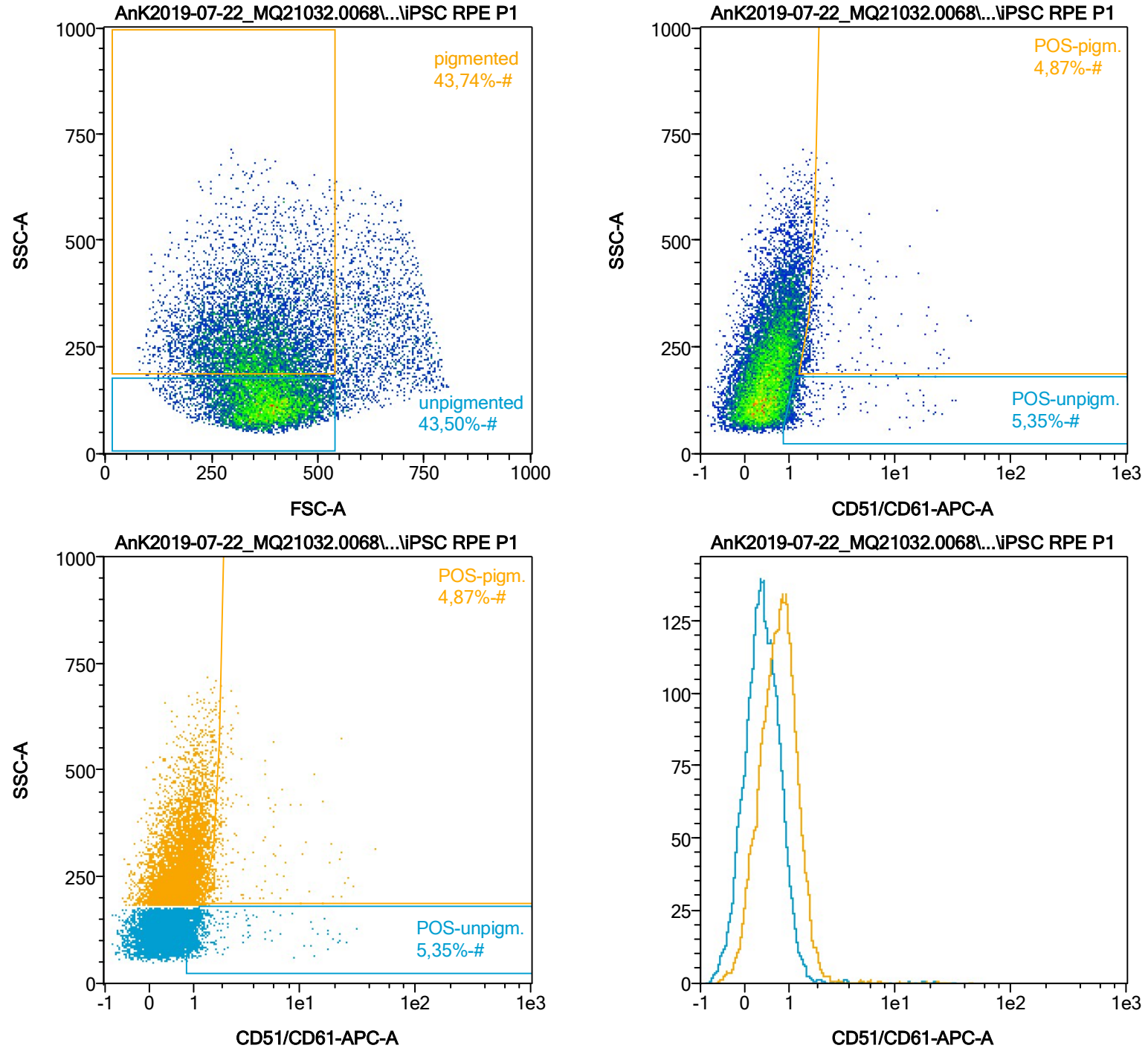

Name	%-#	CD51/CD61-APC-A Mean	CD51/CD61-APC-A Median
POS-unpigm.	5,35	2,25	1,18
POS-pigm.	4,87	3,21	1,85

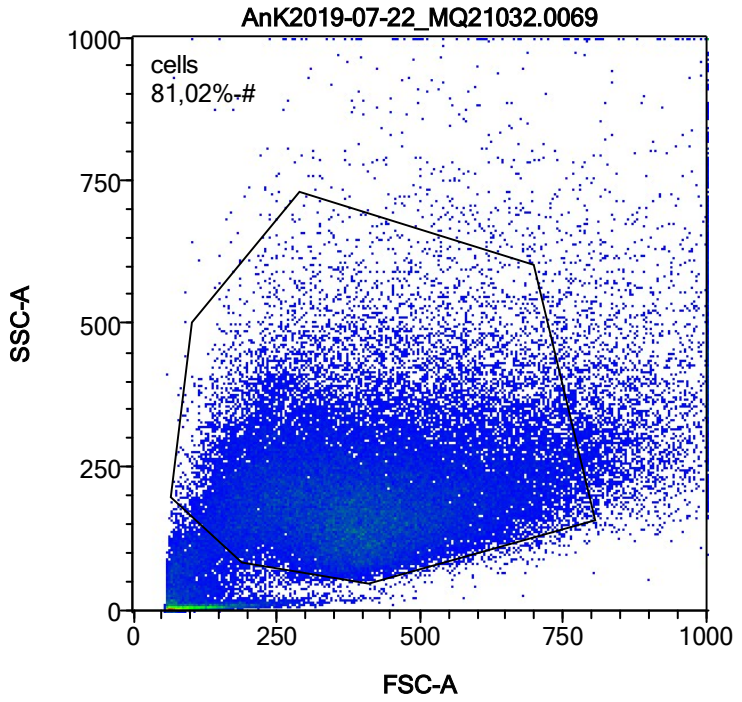

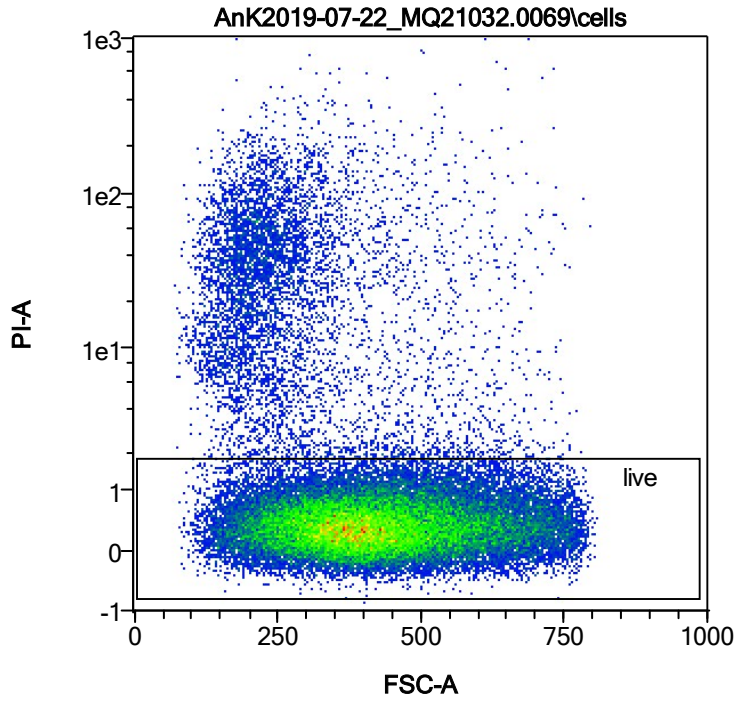

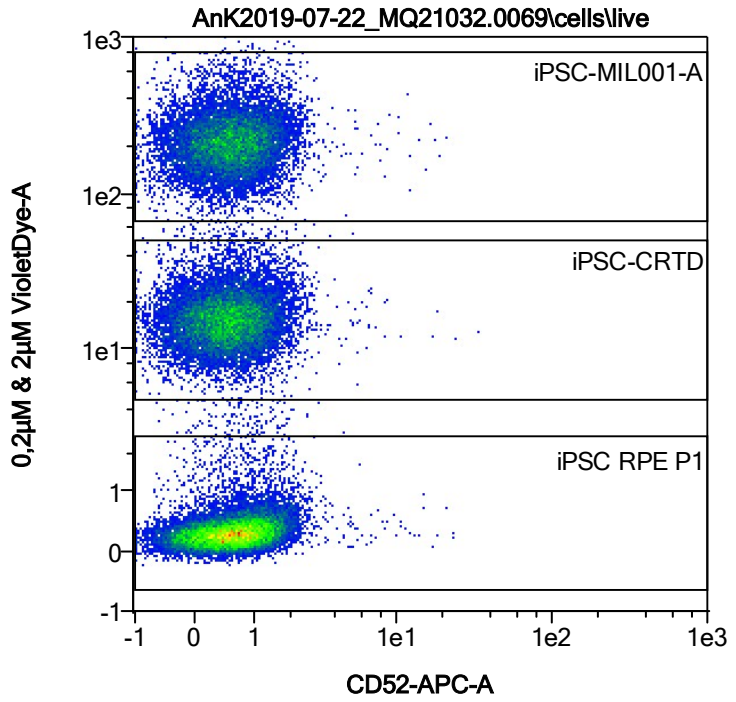

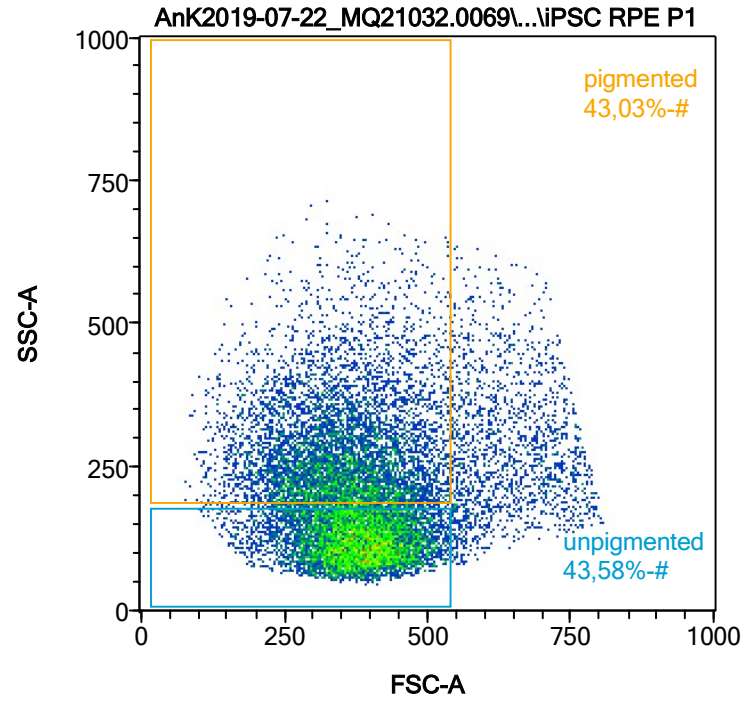

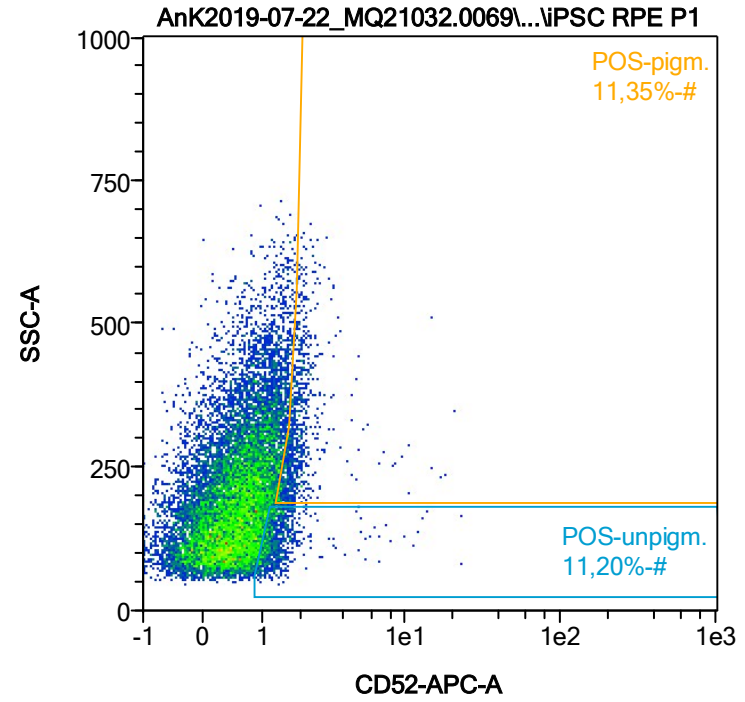

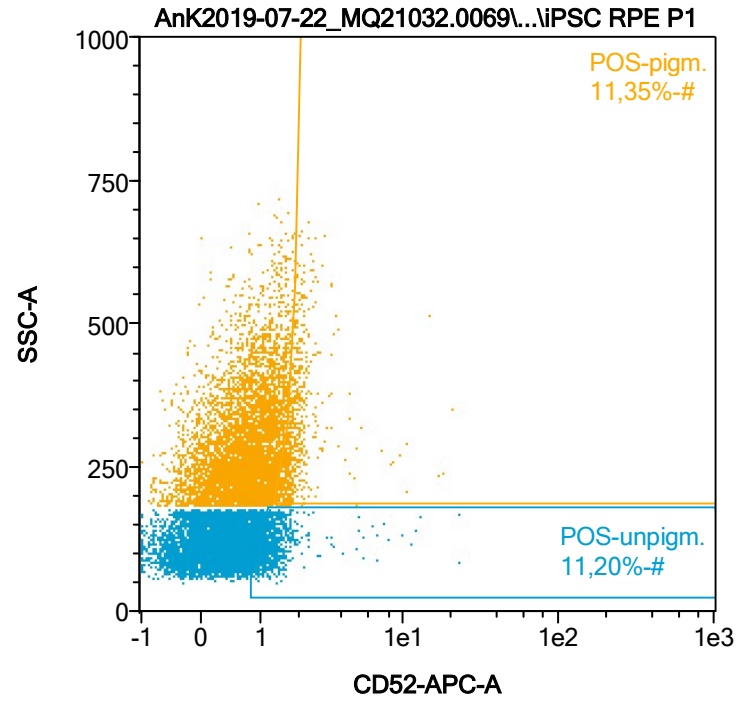

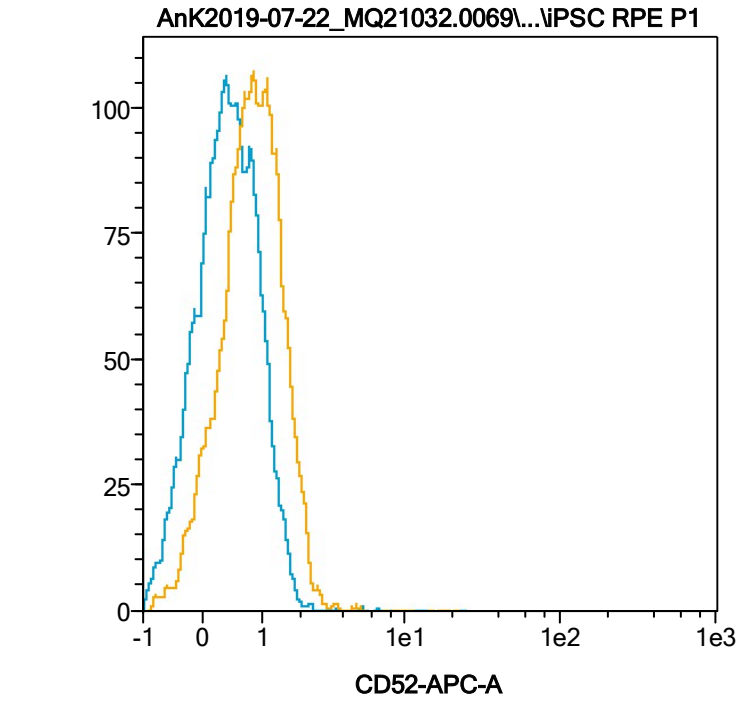

Name	%-#	CD52-APC-A Mean	CD52-APC-A Median
POS-unpigm.	11,20	1,47	1,23
POS-pigm.	11,35	2,03	1,82

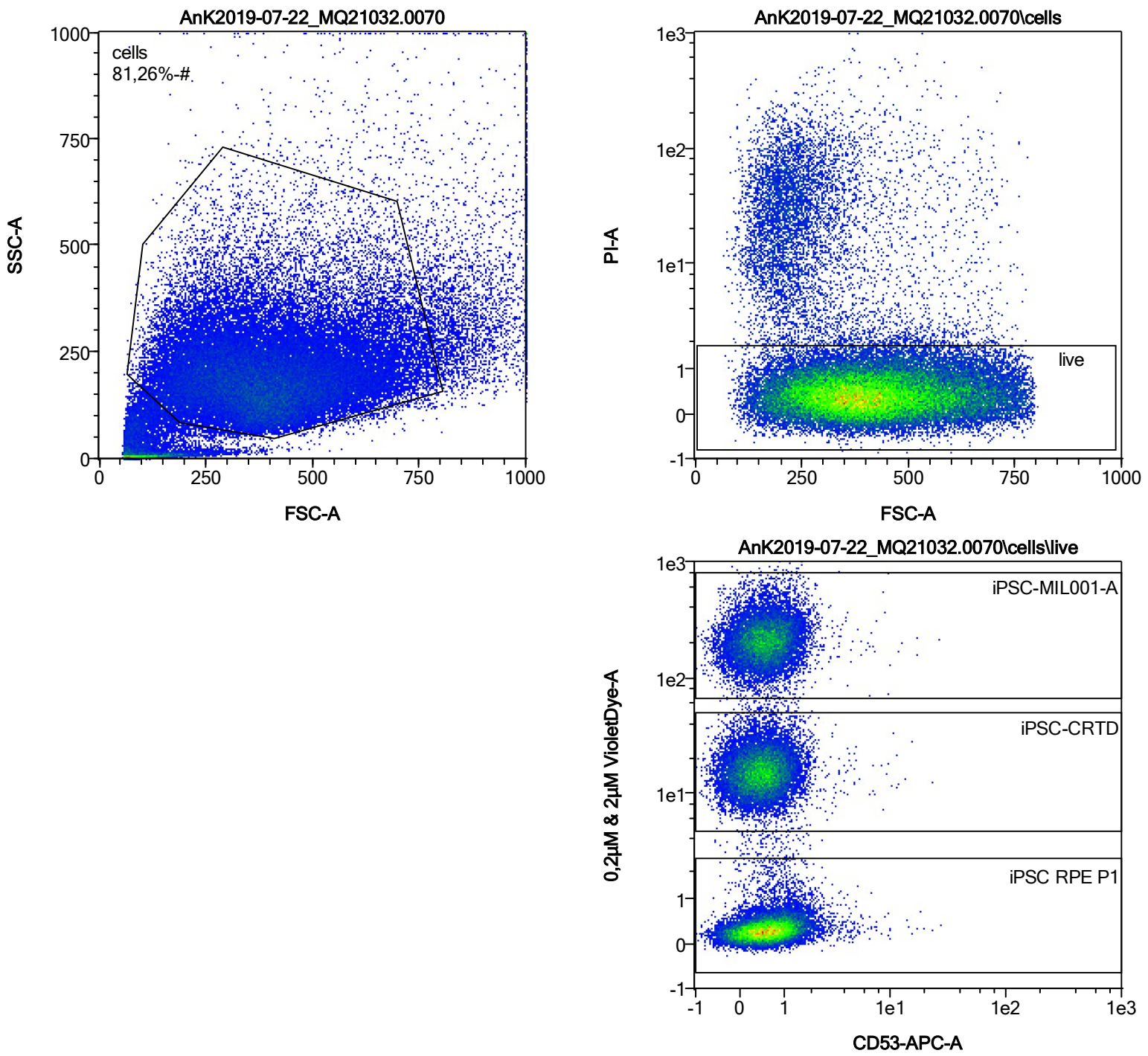

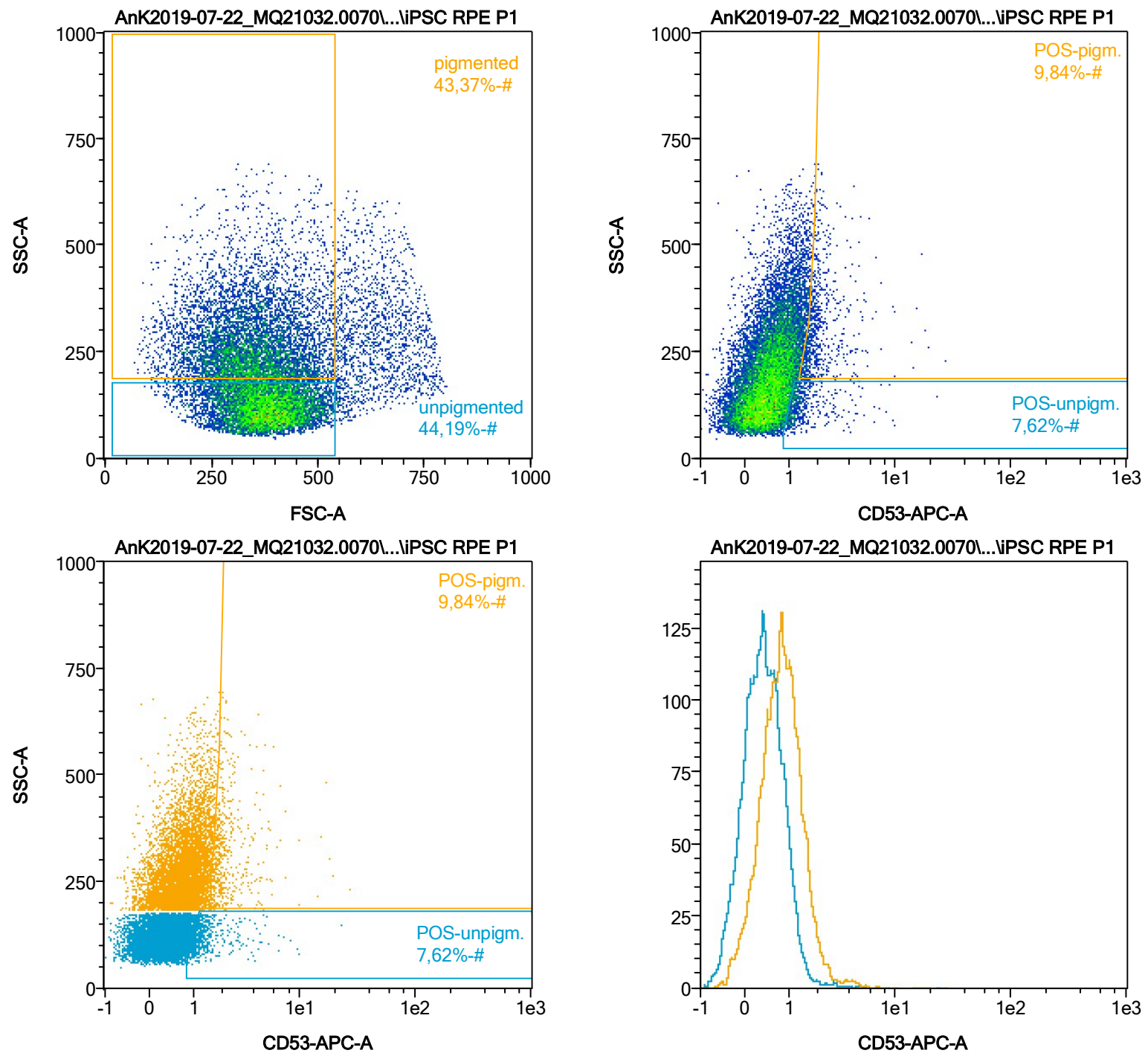

Name	%-#	CD53-APC-A Mean	CD53-APC-A Median
POS-unpigm.	7,62	1,56	1,22
POS-pigm.	9,84	2,32	1,88

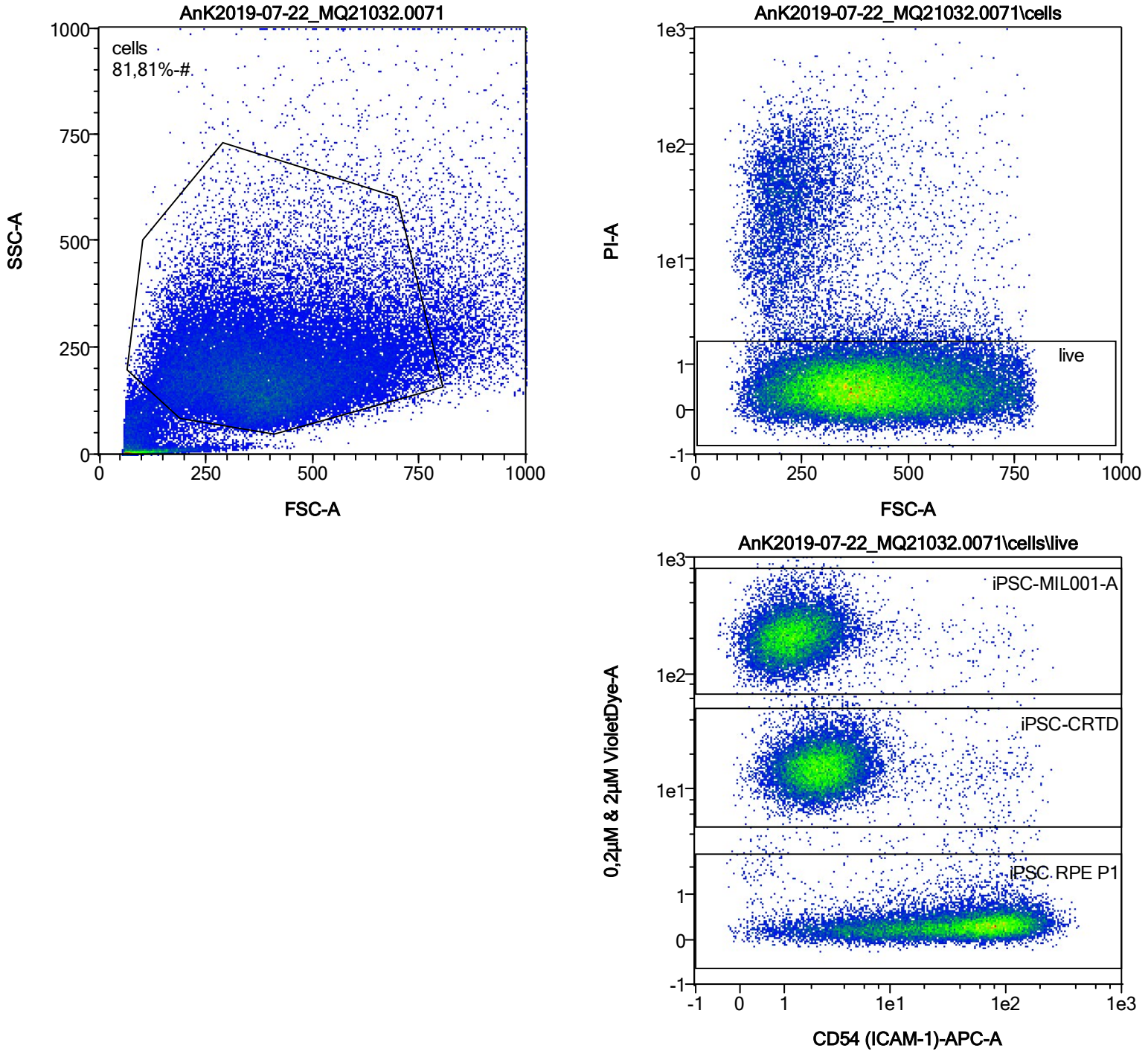

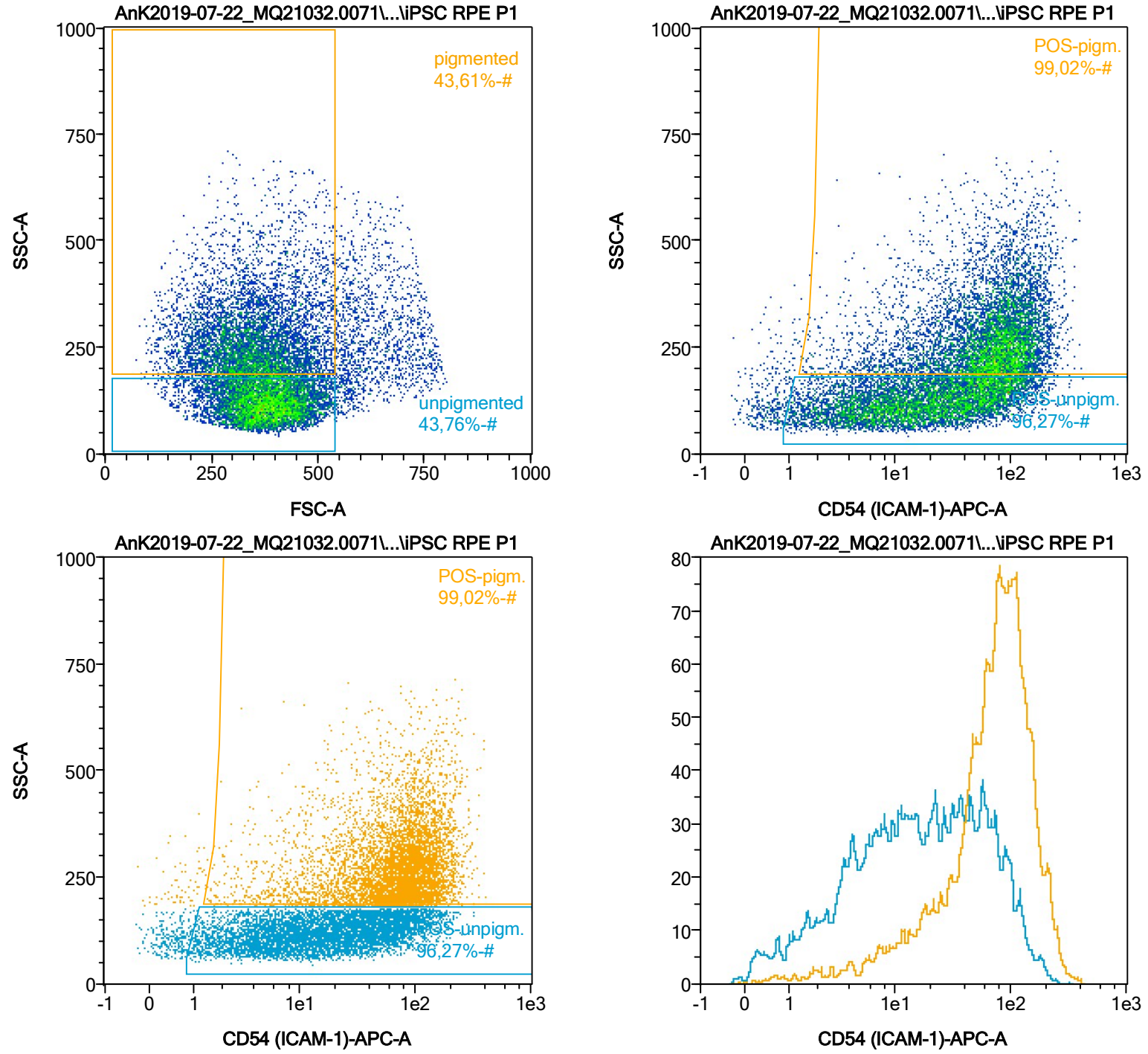

Name	%-#	CD54 (ICAM-1)-APC-A Mean	CD54 (ICAM-1)-APC-A Median
POS-unpigm.	96,27	32,02	18,14
POS-pigm.	99,02	84,34	76,68

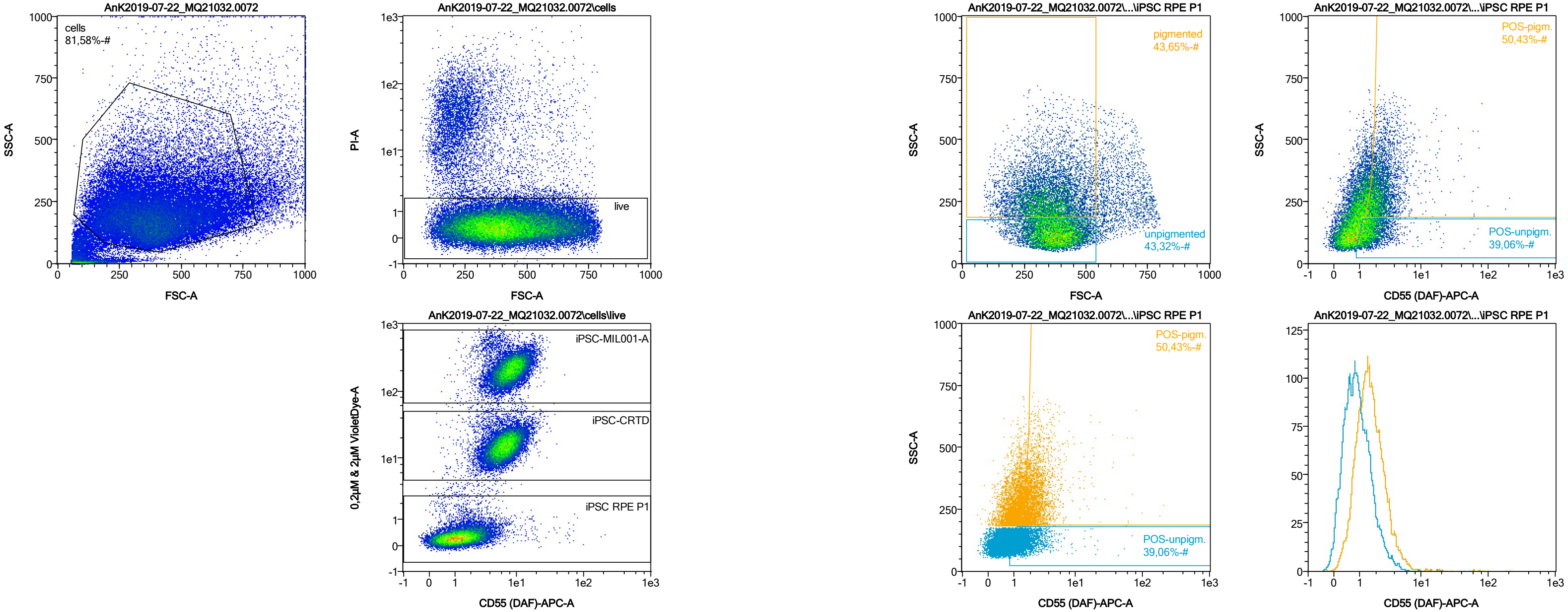

Name	%-#	CD55 (DAF)-APC-A Mean	CD55 (DAF)-APC-A Median
POS-unpigm.	39,06	1,85	1,49
POS-pigm.	50,43	2,92	2,18

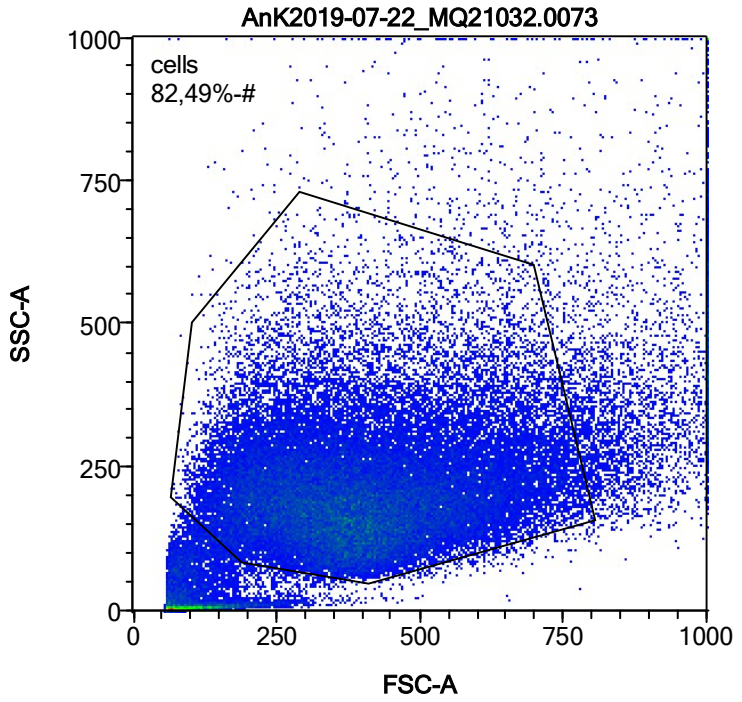

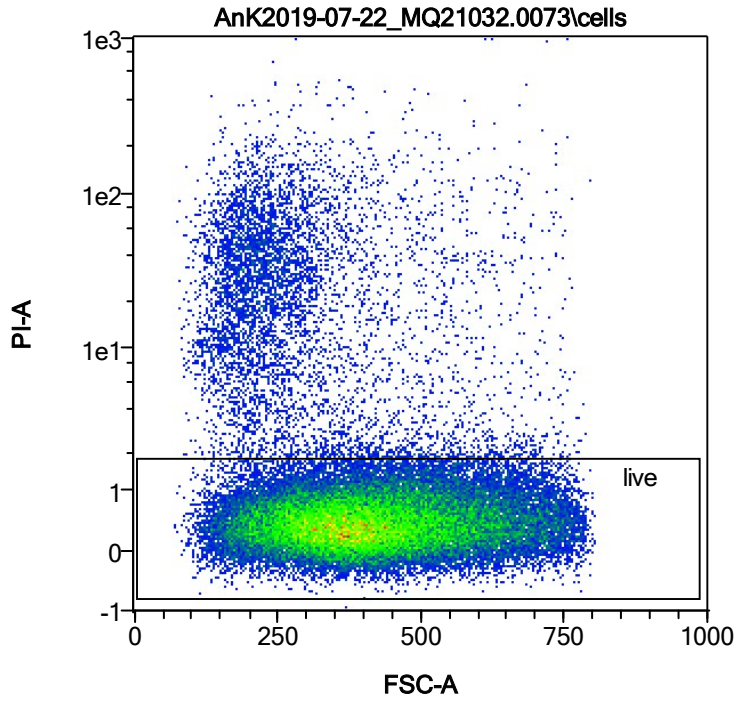

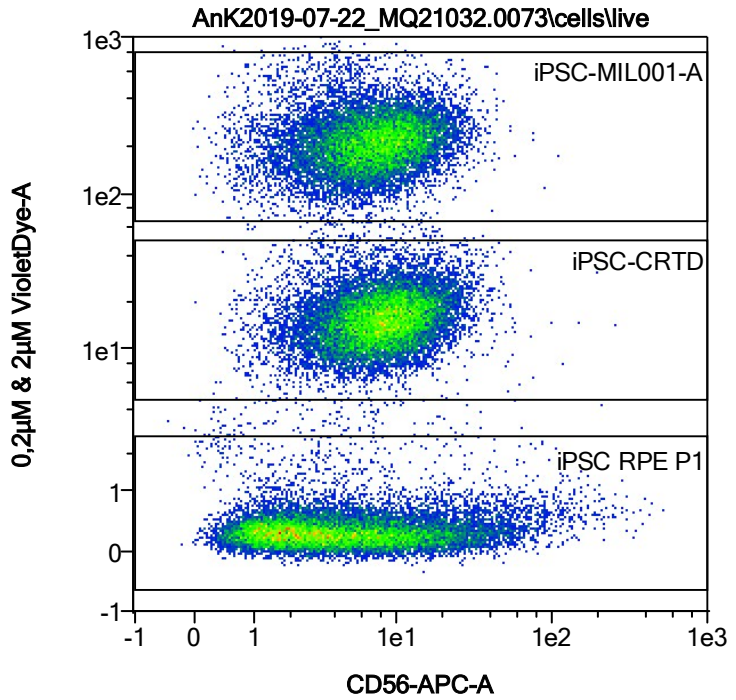

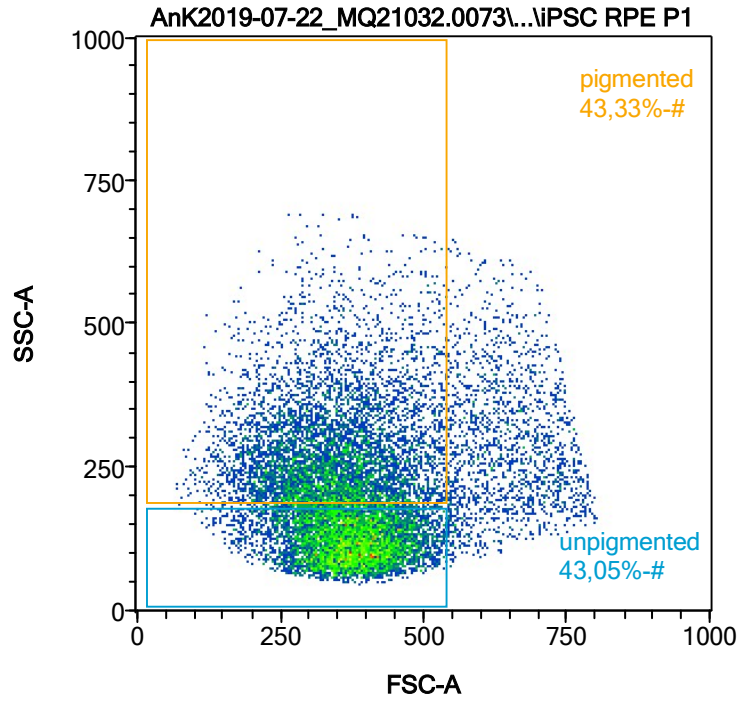

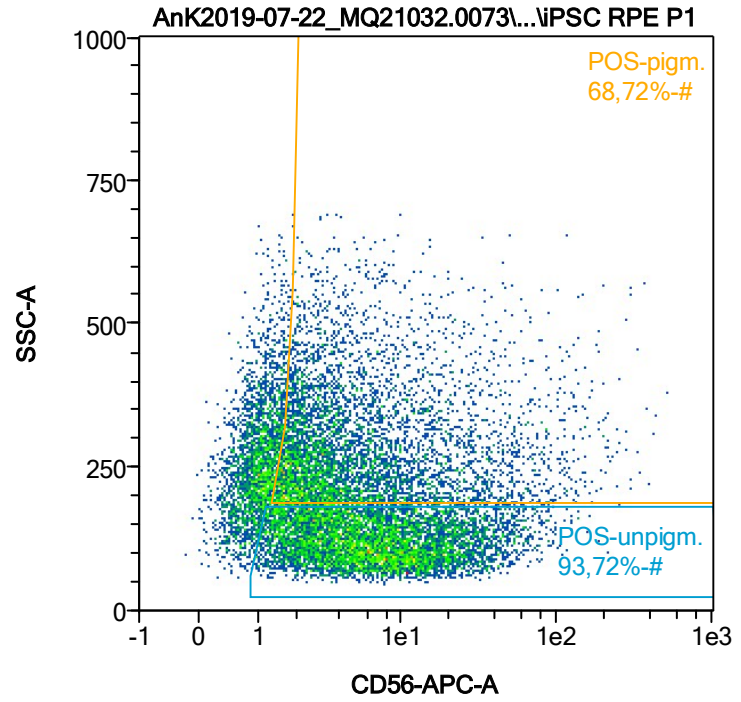

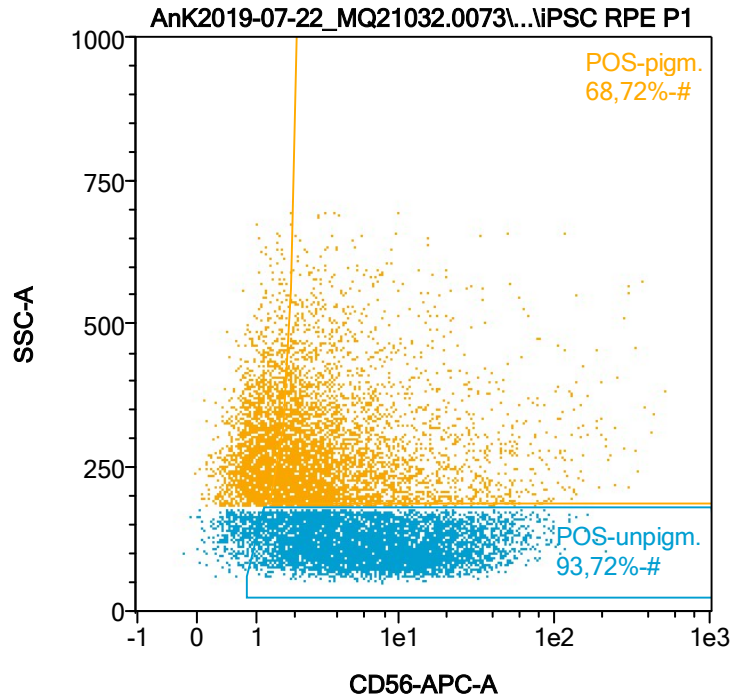

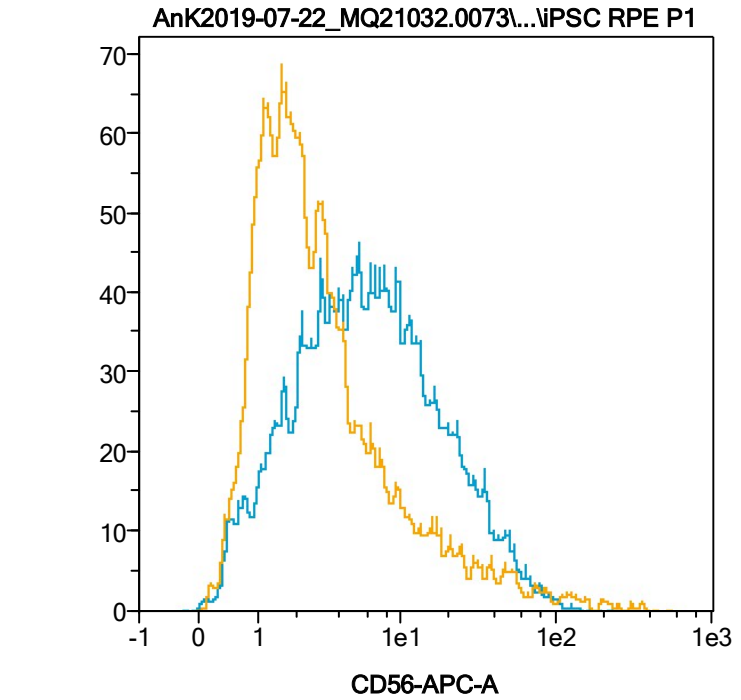

Name	%-#	CD56-APC-A Mean	CD56-APC-A Median
POS-unpigm.	93,72	11,08	6,44
POS-pigm.	68,72	10,32	3,34

Date:2024-06-14

Operator: Sebastian Knoebel

Version:2.13.0+54.g95abf0f4

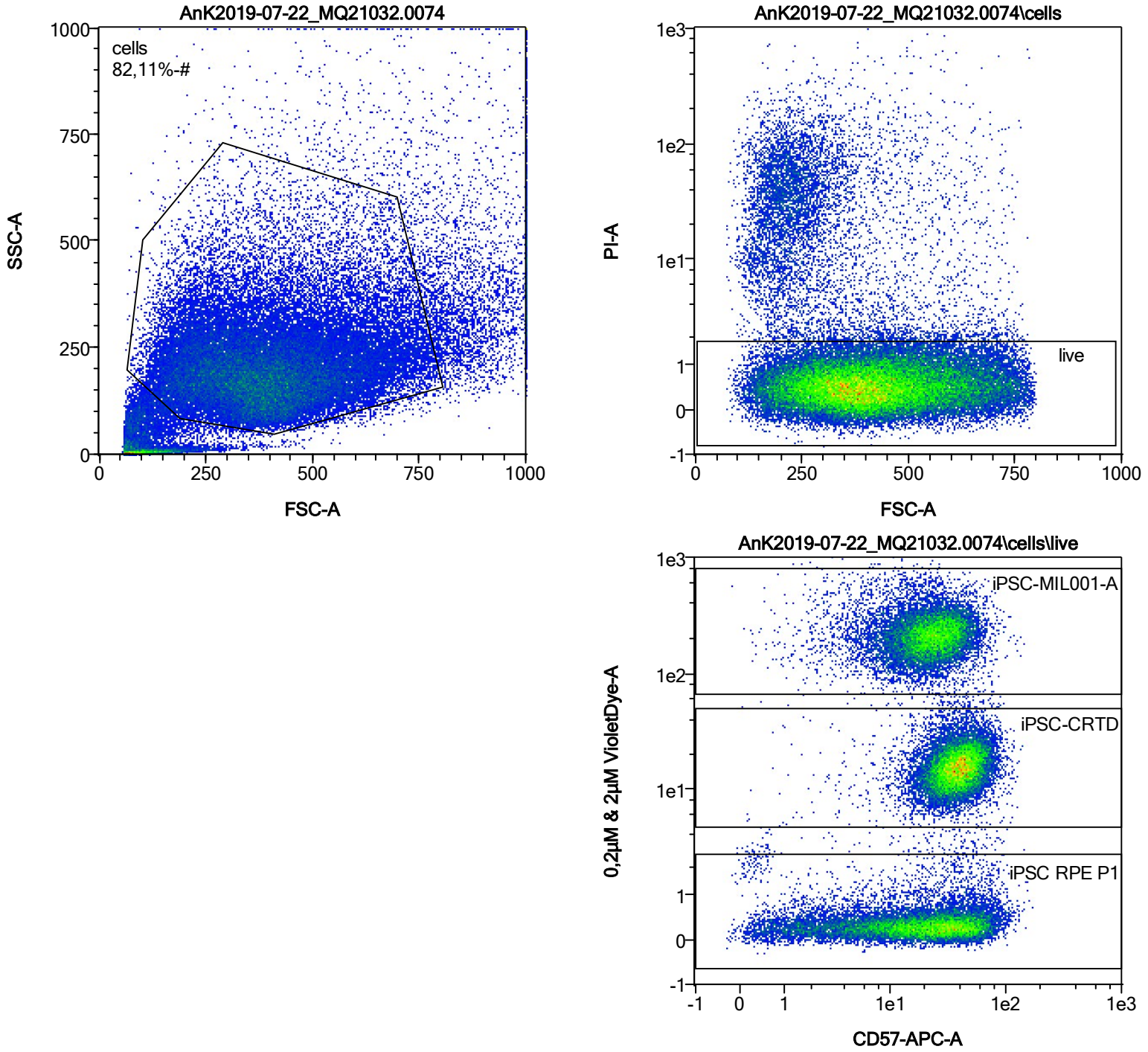

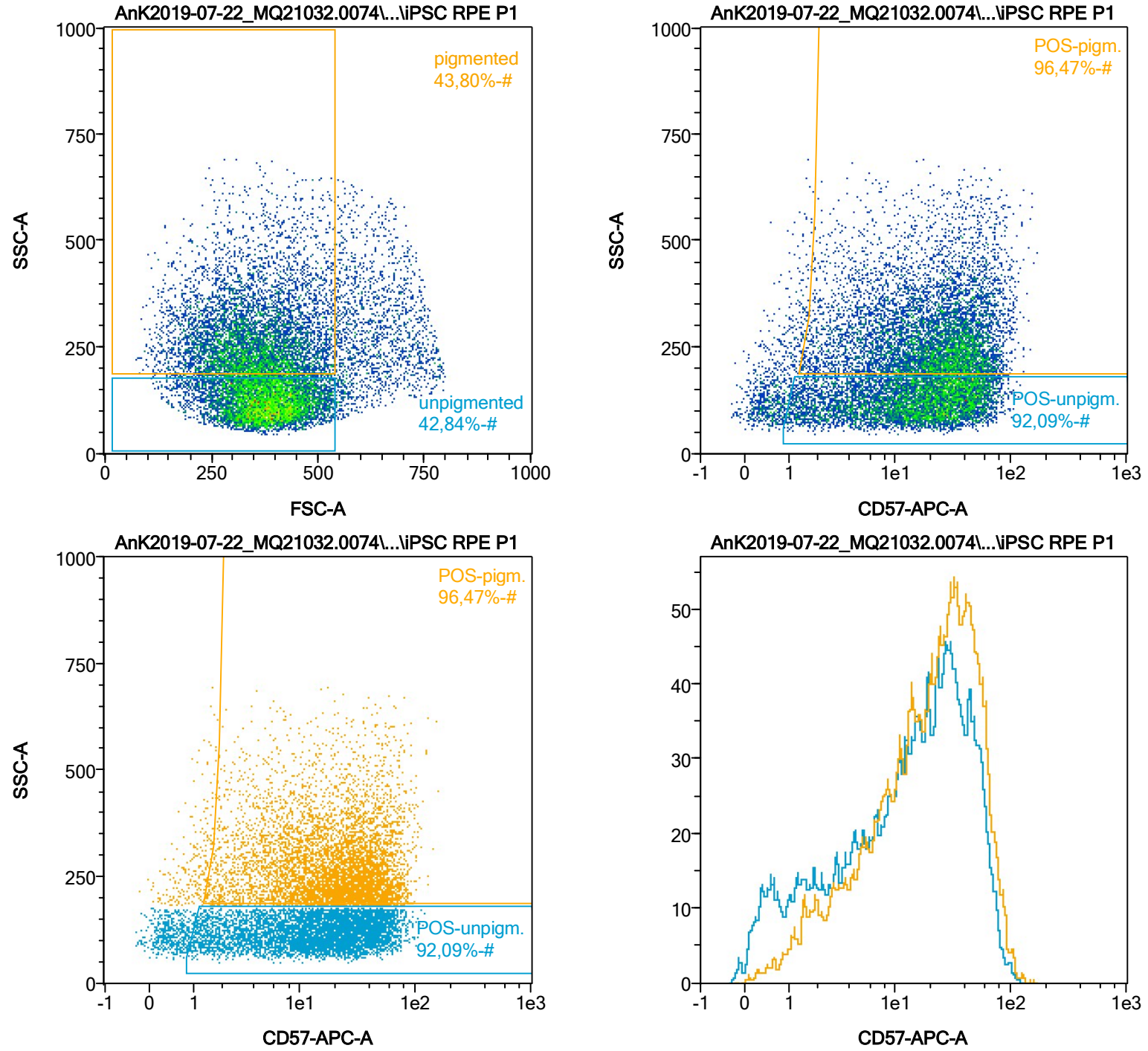

Name	%-#	CD57-APC-A Mean	CD57-APC-A Median
POS-unpigm.	92,09	22,09	17,03
POS-pigm.	96,47	26,70	21,99

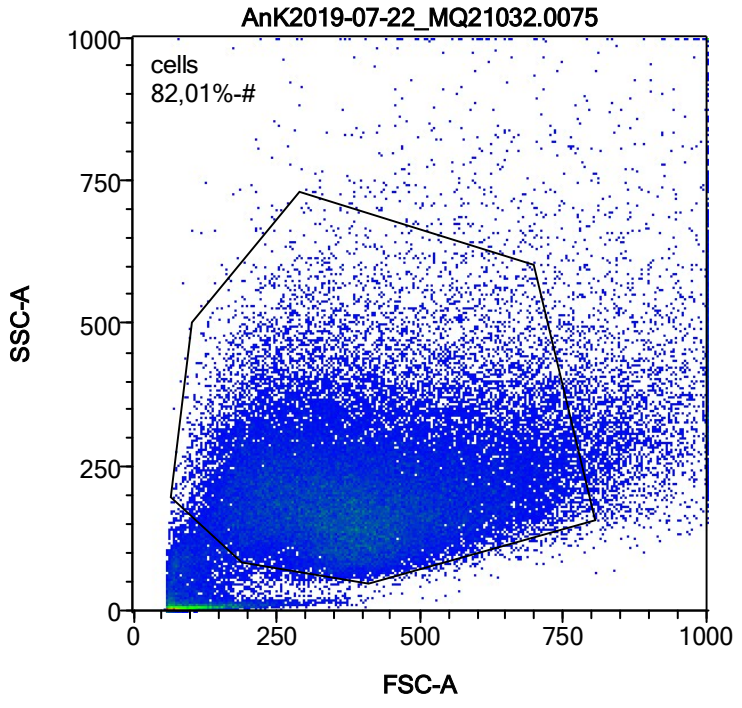

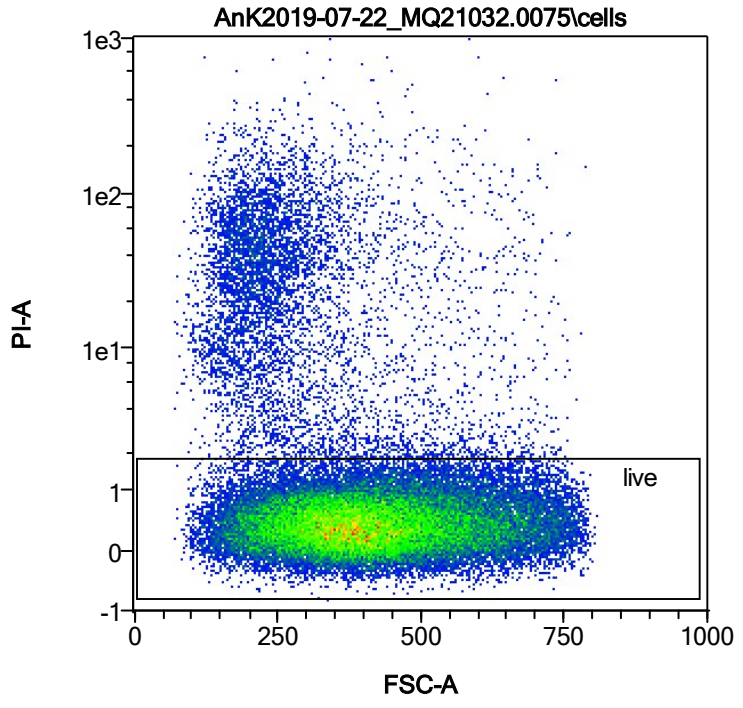

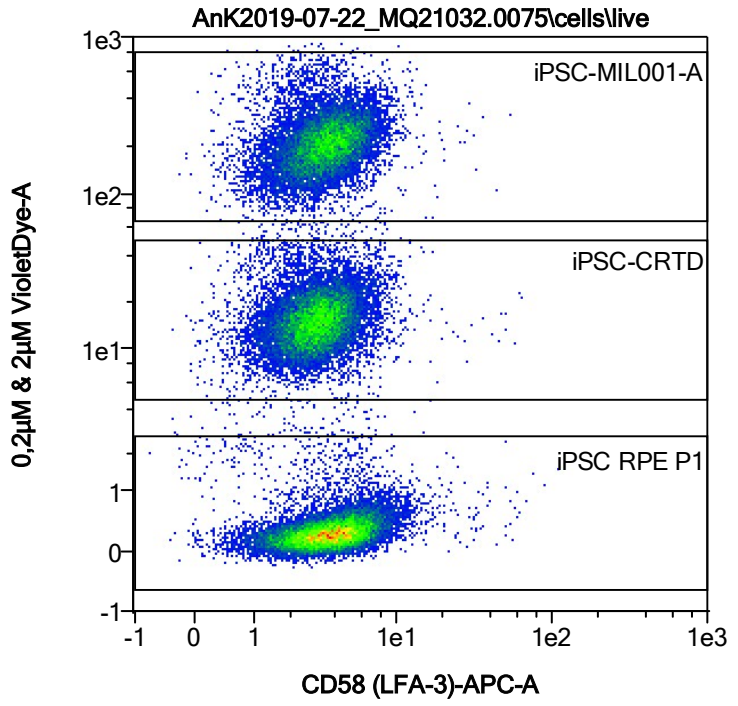

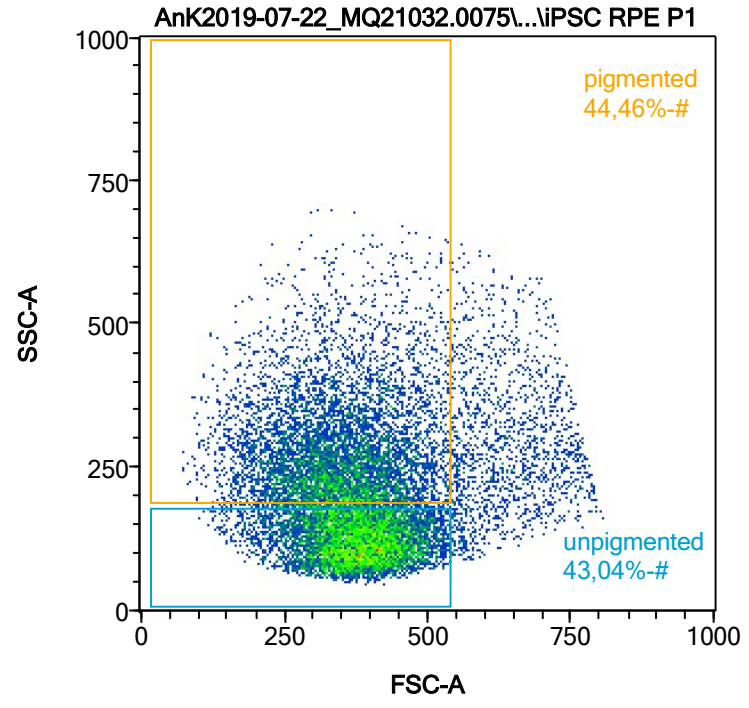

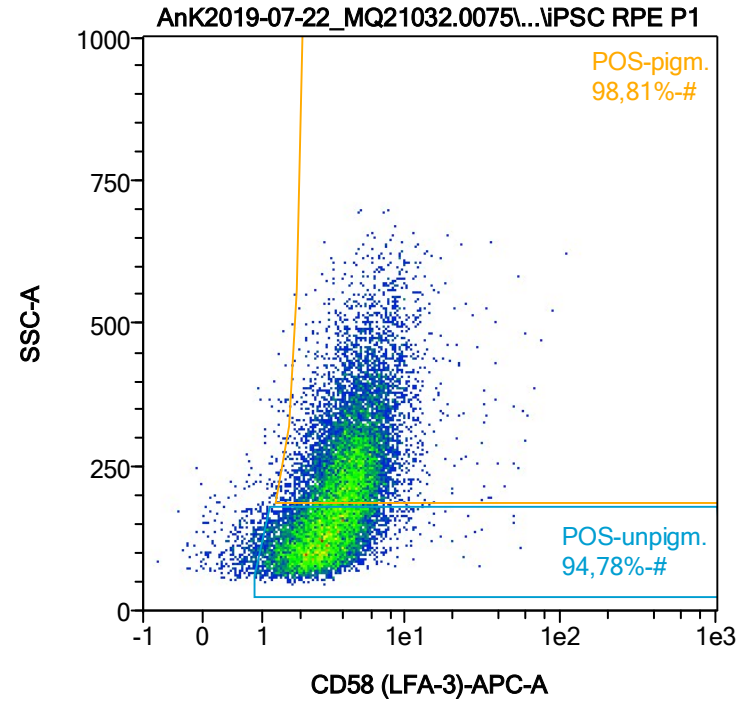

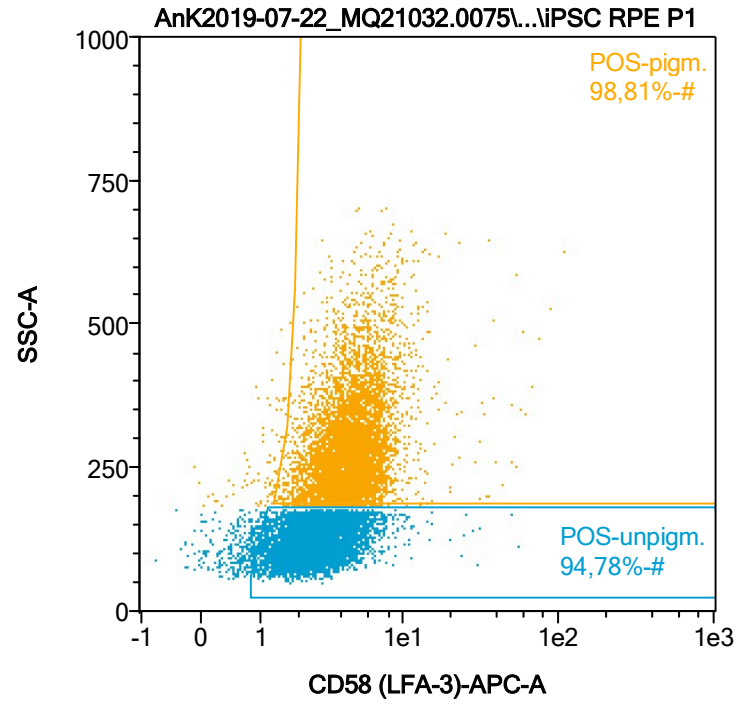

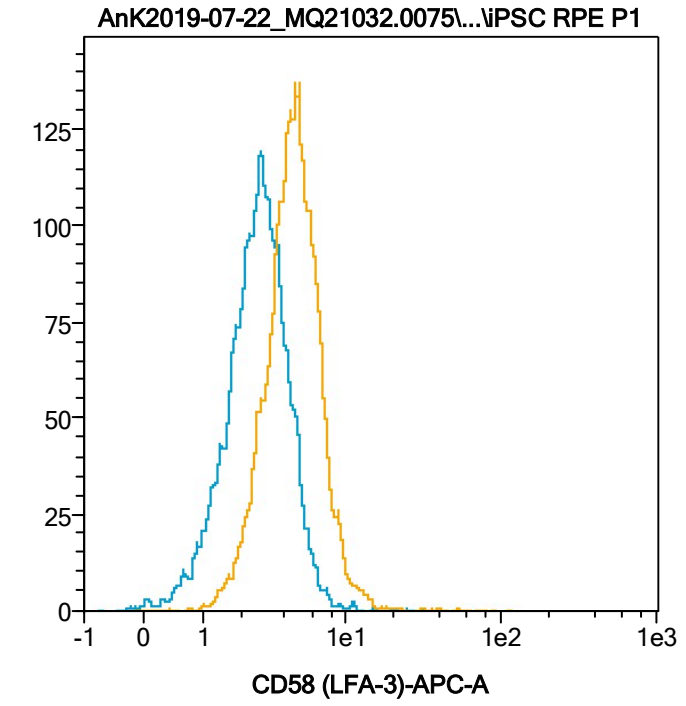

Name	%-#	CD58 (LFA-3)-APC-A Mean	CD58 (LFA-3)-APC-A Median
POS-unpigm.	94,78	2,99	2,72
POS-pigm.	98,81	4,98	4,53

Date:2024-06-14

Operator: Sebastian Knoebel

Version:2.13.0+54.g95abf0f4

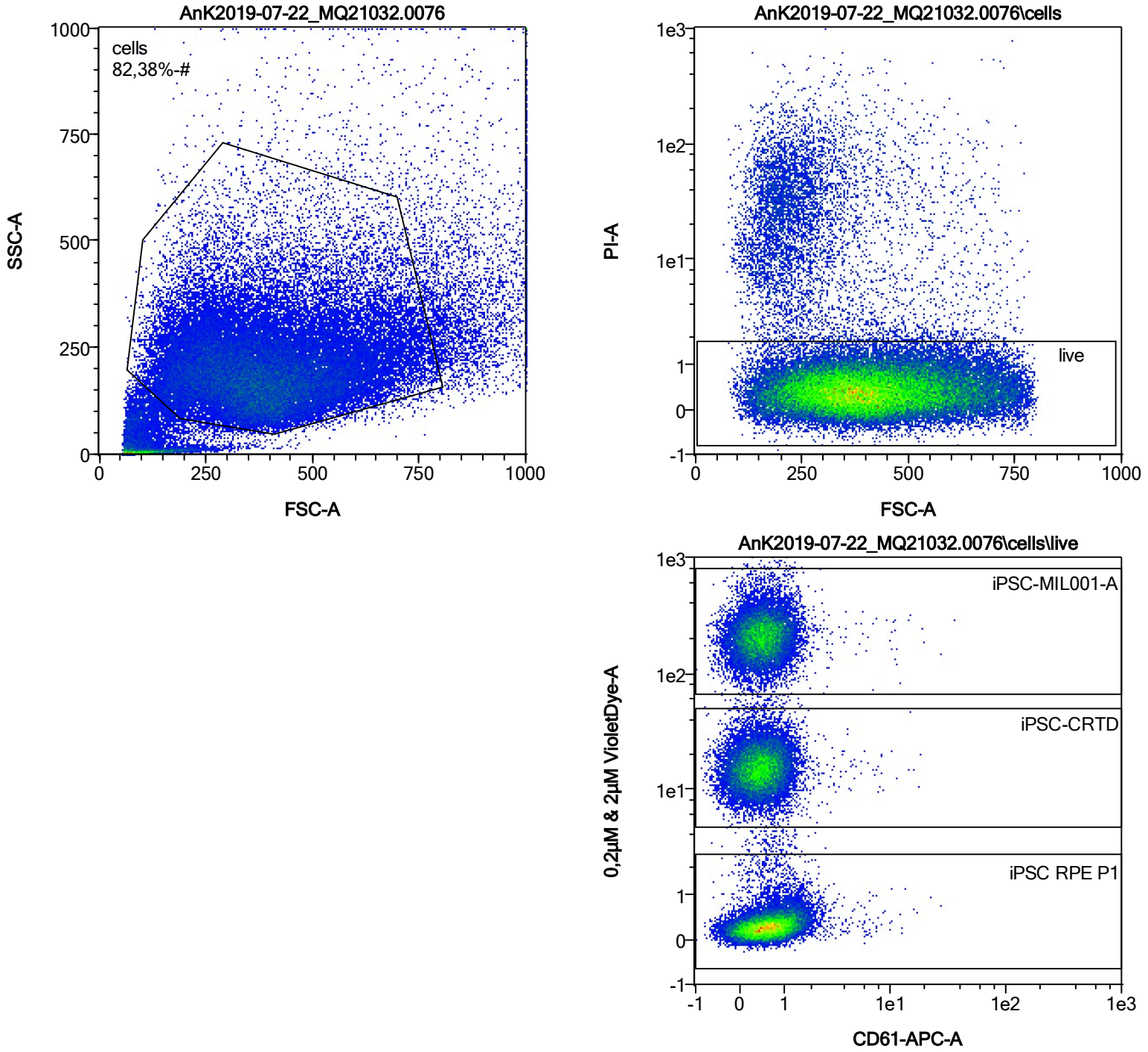

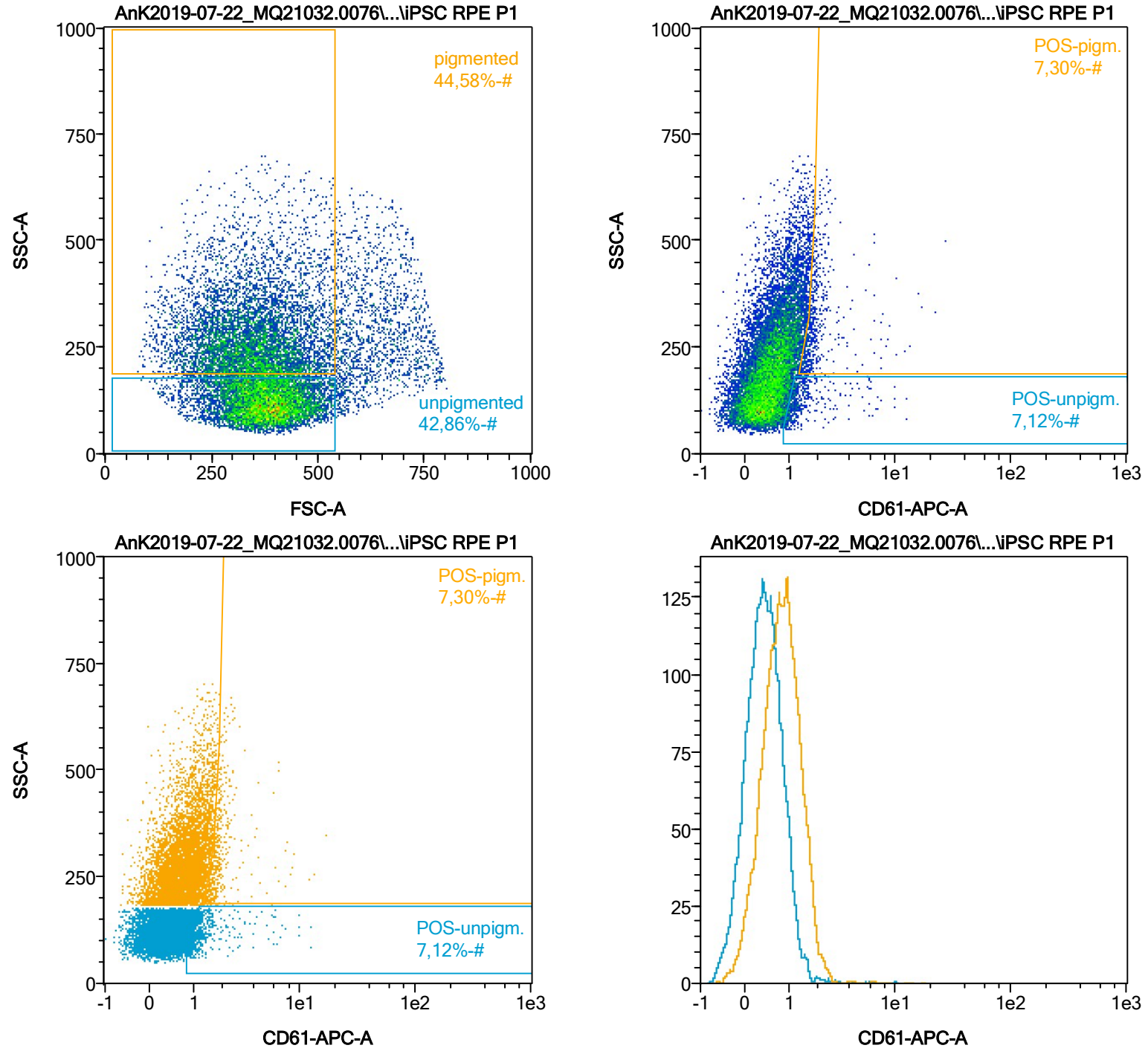

Name	%-#	CD61-APC-A Mean	CD61-APC-A Median
POS-unpigm.	7,12	1,56	1,19
POS-pigm.	7,30	2,08	1,78

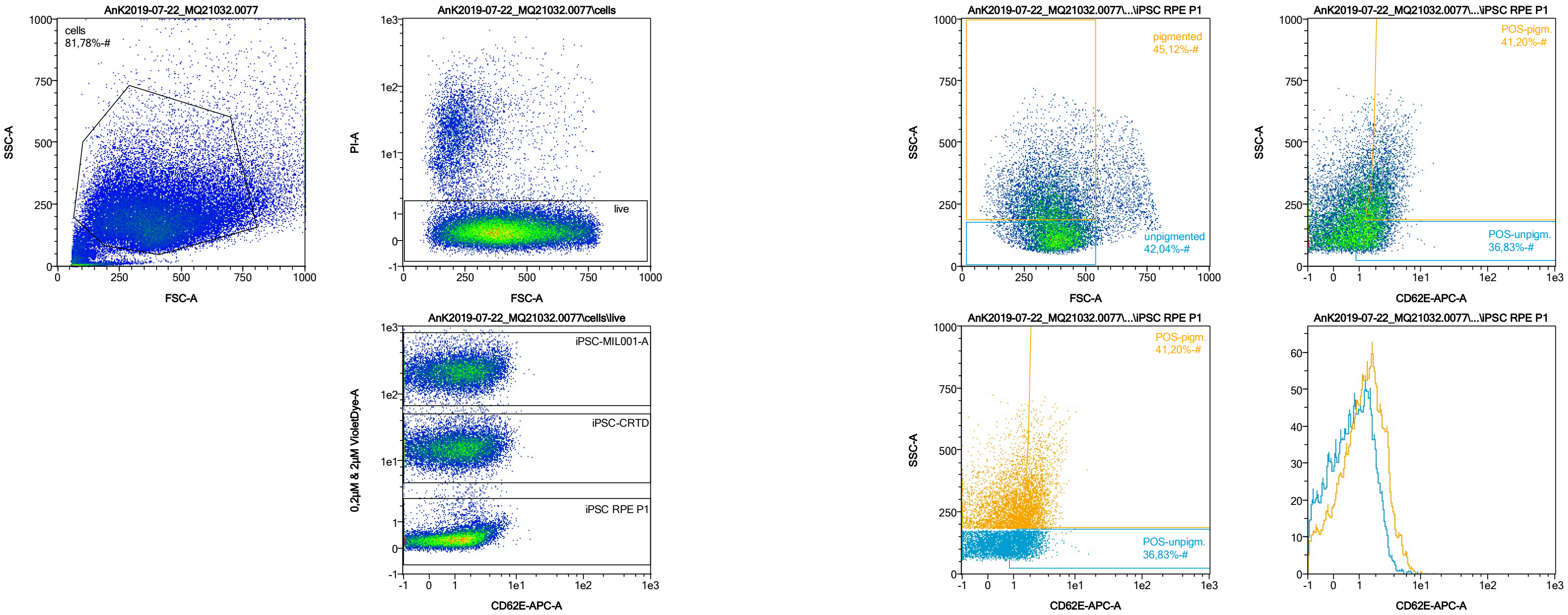

Name	%-#	CD62E-APC-A Mean	CD62E-APC-A Median
POS-unpigm.	36,83	1,82	1,62
POS-pigm.	41,20	2,64	2,36

Date:2024-06-14
Operator: Sebastian Knoebel
Version:2.13.0+54.g95abf0f4

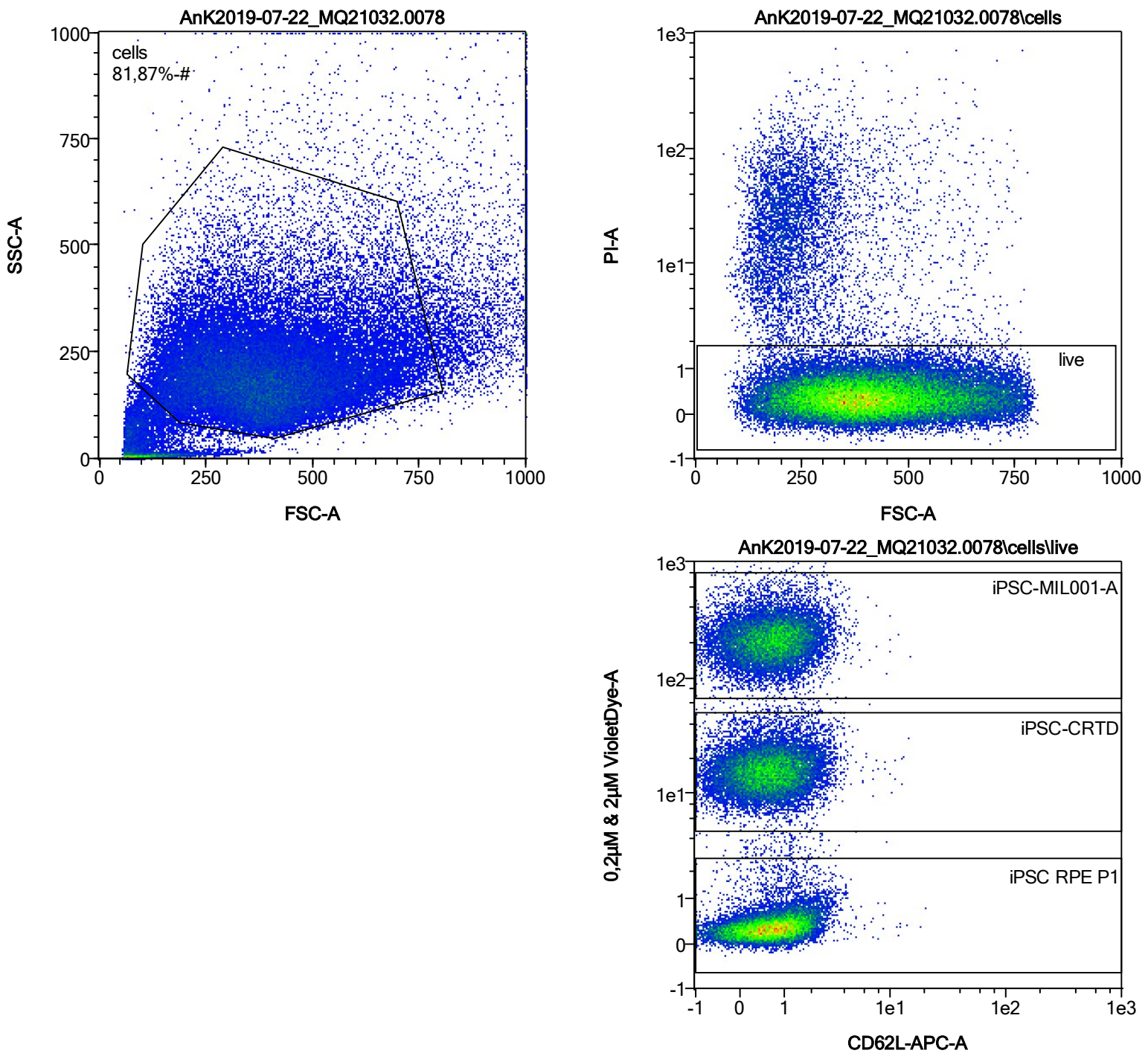

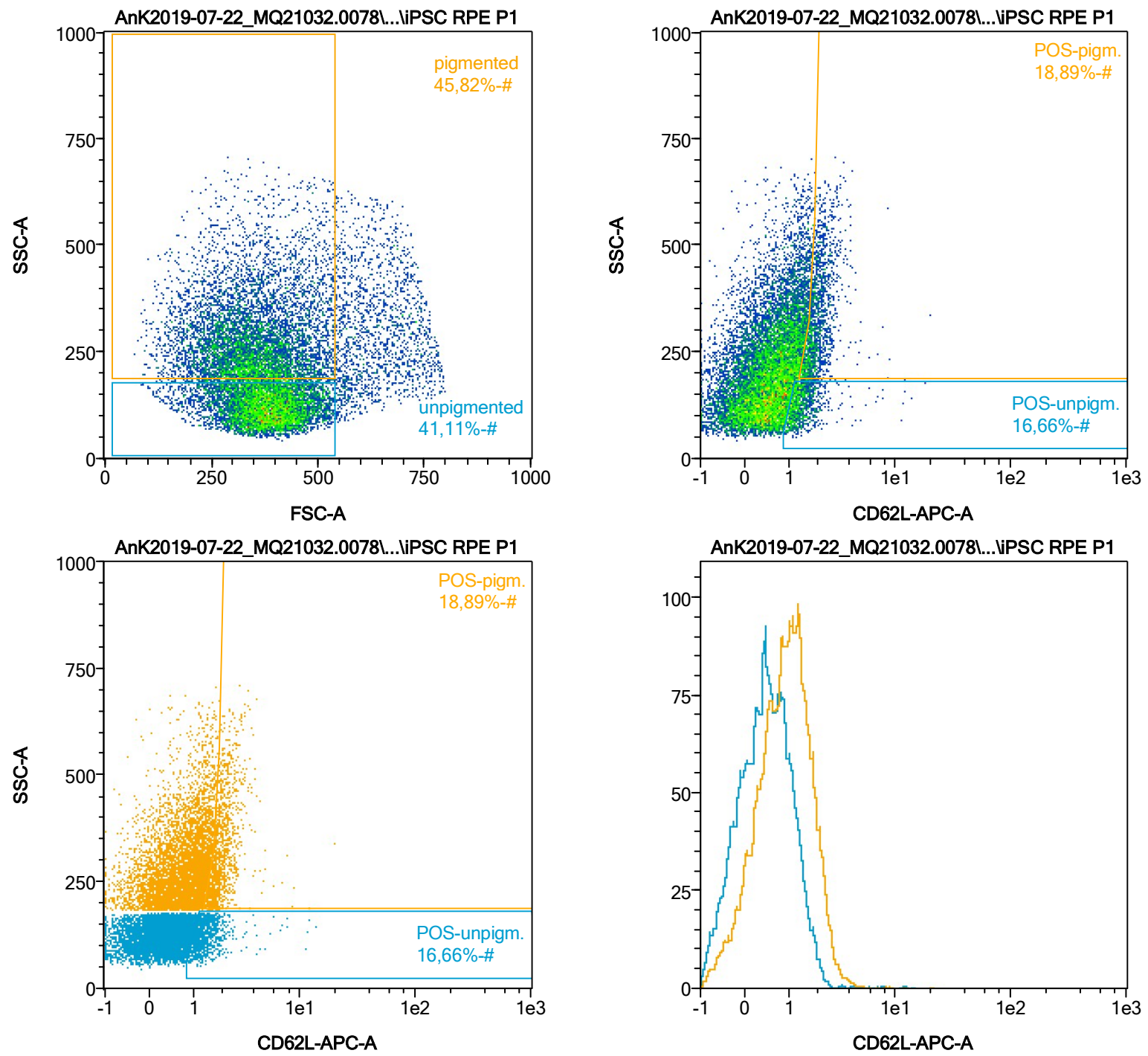

Name	%-#	CD62L-APC-A Mean	CD62L-APC-A Median
POS-unpigm.	16,66	1,47	1,29
POS-pigm.	18,89	2,07	1,90

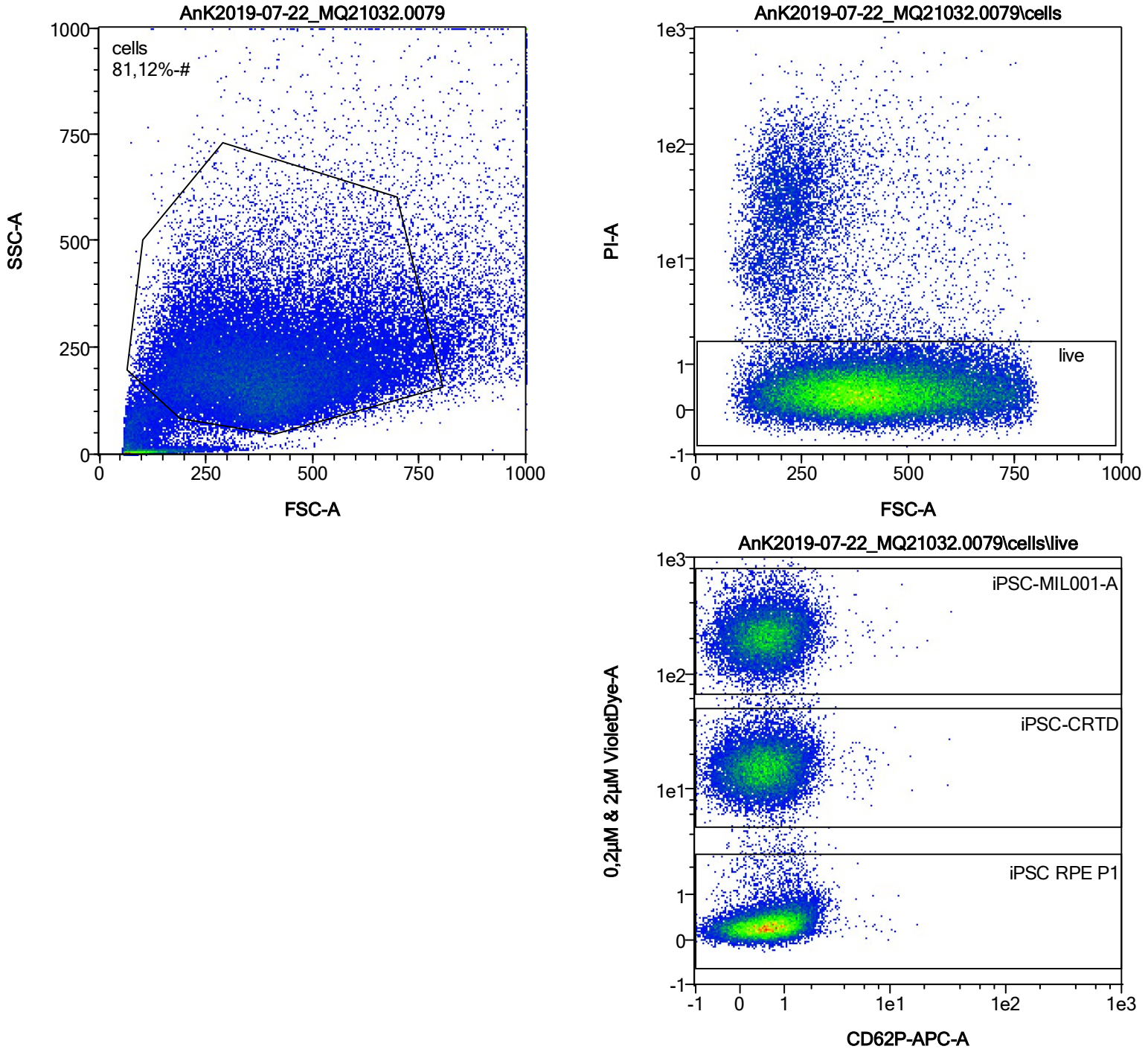

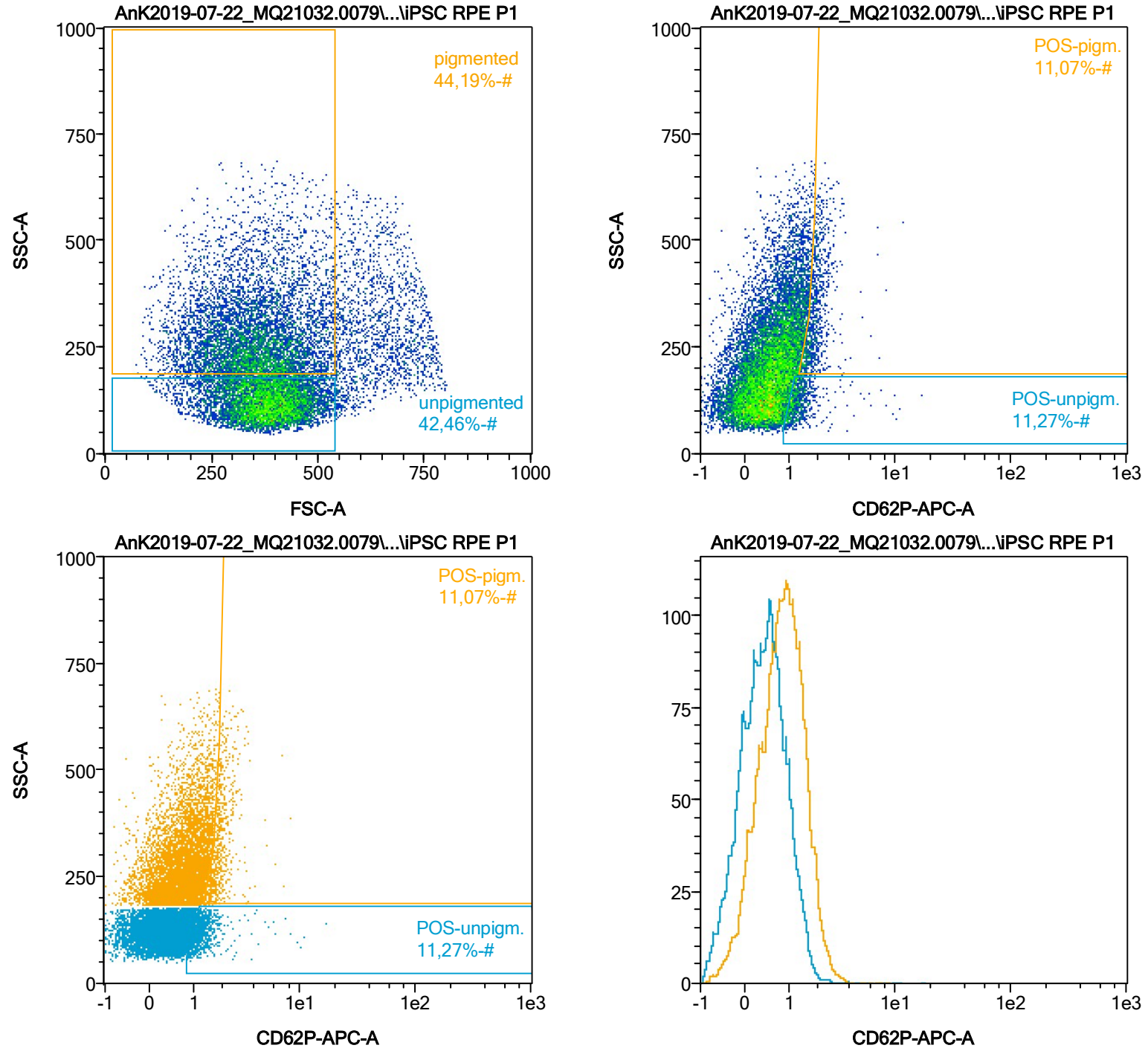

Name	%-#	CD62P-APC-A Mean	CD62P-APC-A Median
POS-unpigm.	11,27	1,41	1,22
POS-pigm.	11,07	1,95	1,86

Date:2024-06-14

Operator: Sebastian Knoebel

Version:2.13.0+54.g95abf0f4

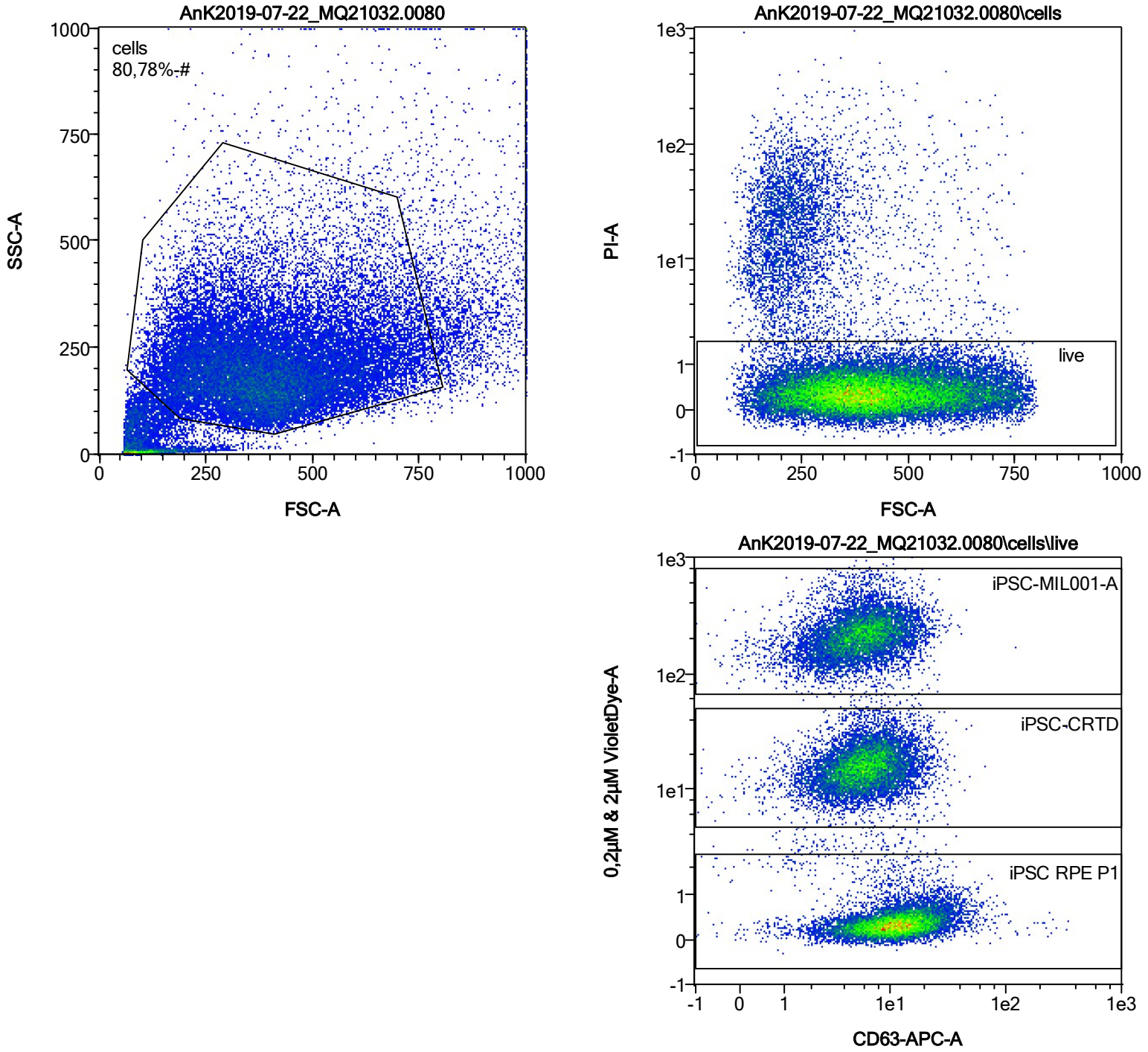

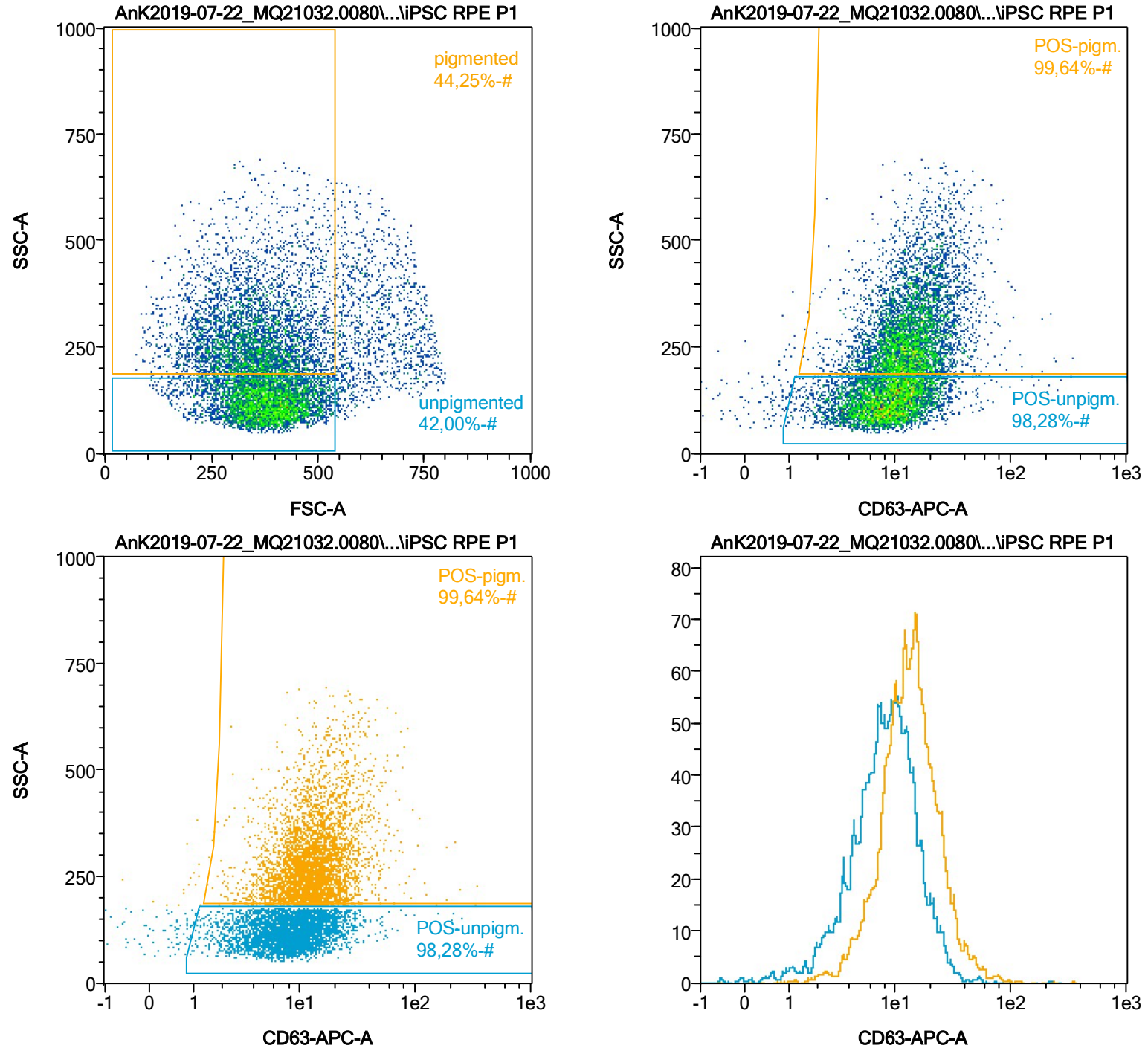

Name	%-#	CD63-APC-A Mean	CD63-APC-A Median
POS-unpigm.	98,28	10,02	8,48
POS-pigm.	99,64	15,58	13,04

Date:2024-06-14

Operator: Sebastian Knoebel

Version:2.13.0+54.g95abf0f4

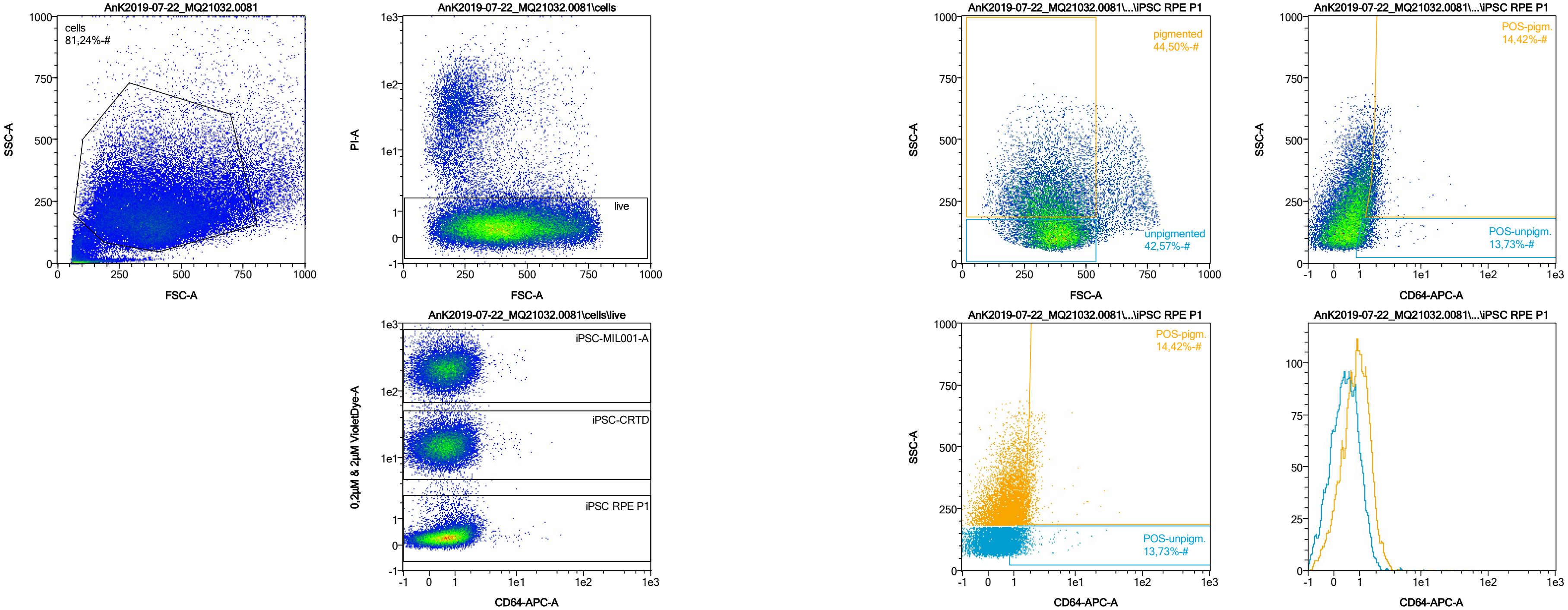

Name	%-#	CD64-APC-A Mean	CD64-APC-A Median
POS-unpigm.	13,73	1,49	1,25
POS-pigm.	14,42	2,09	1,83

Date:2024-06-14

Operator: Sebastian Knoebel

Version:2.13.0+54.g95abf0f4

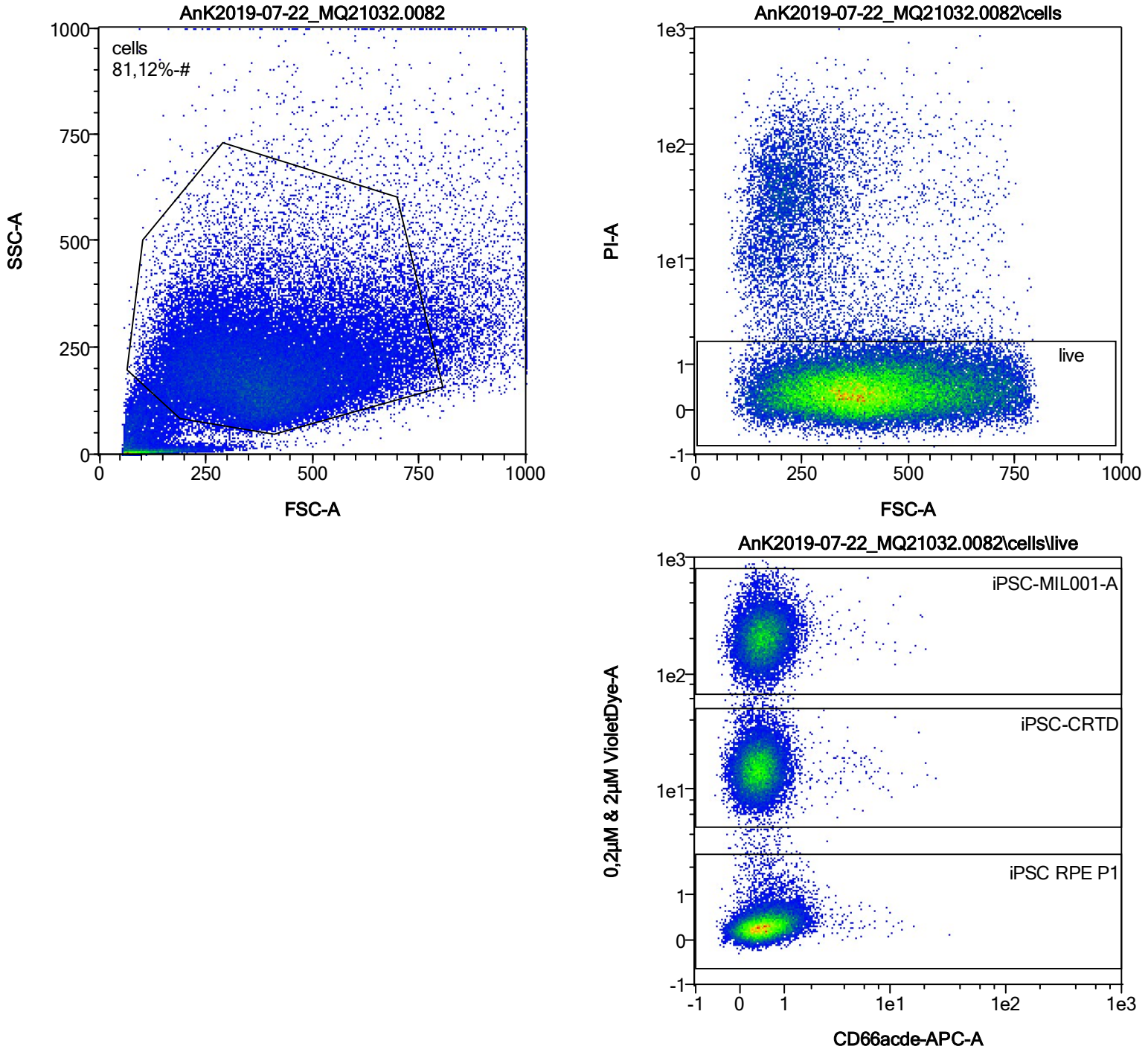

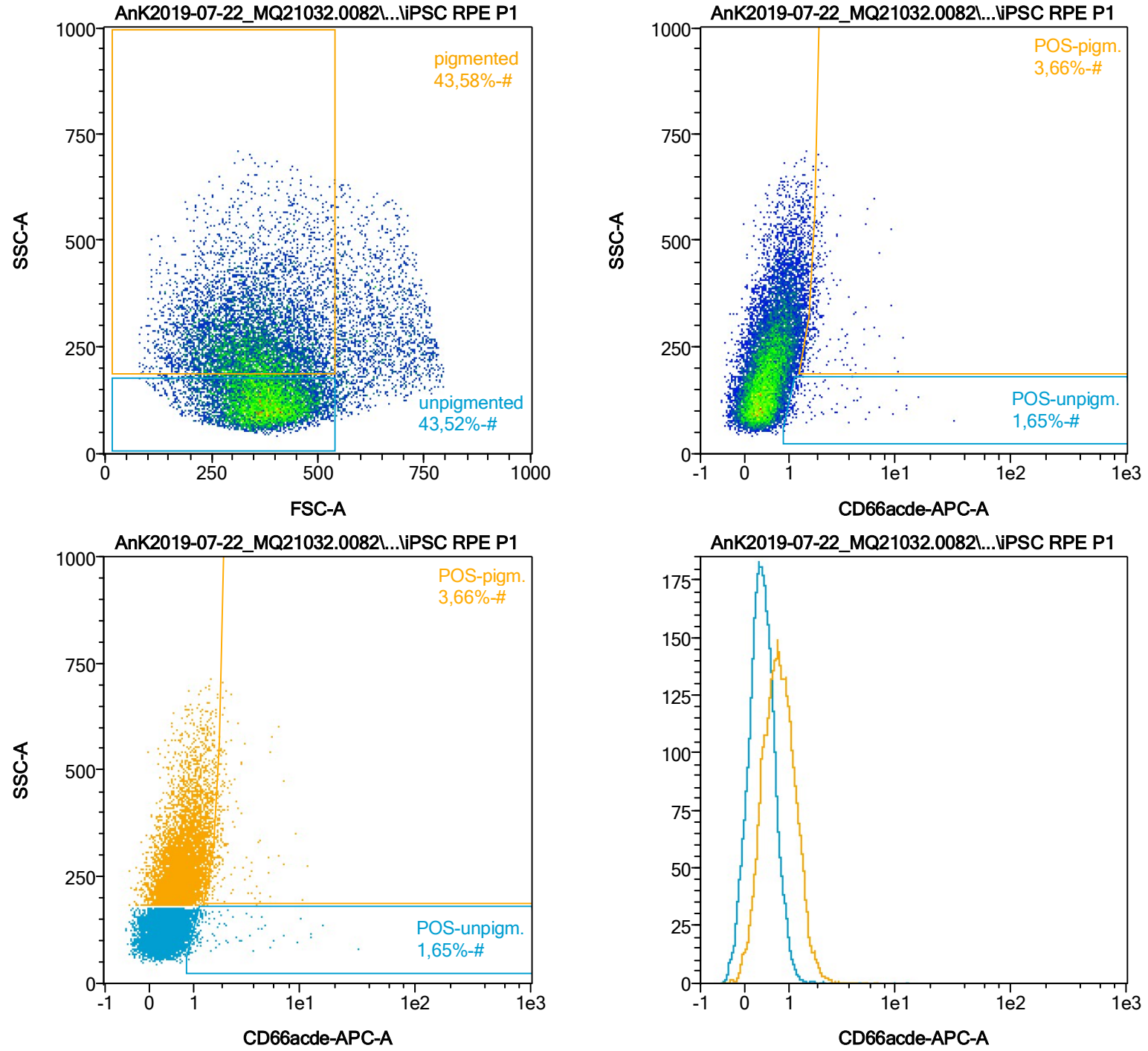

Name	%-#	CD66acde-APC-A Mean	CD66acde-APC-A Median
POS-unpigm.	1,65	2,42	1,24
POS-pigm.	3,66	2,24	1,86

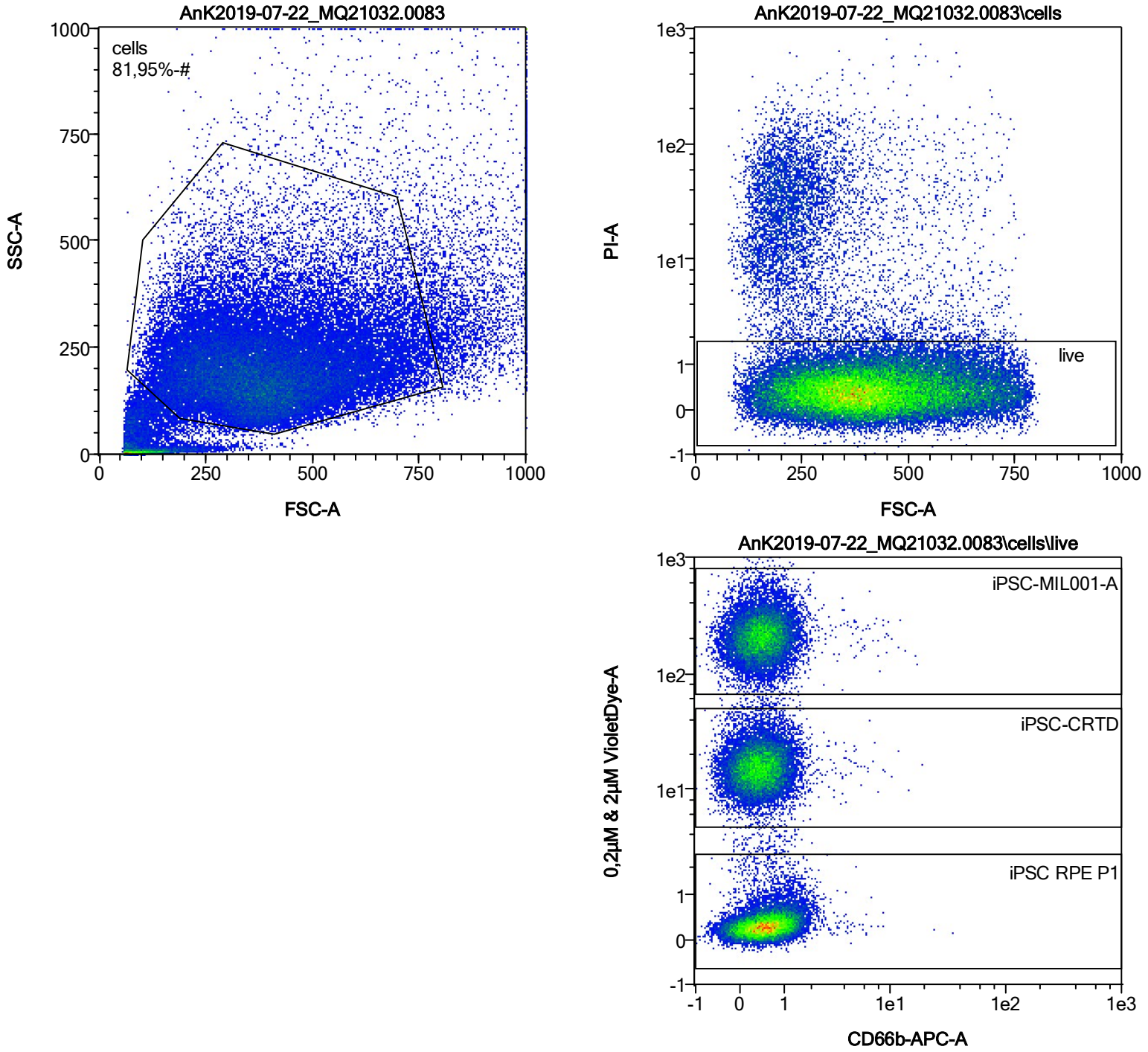

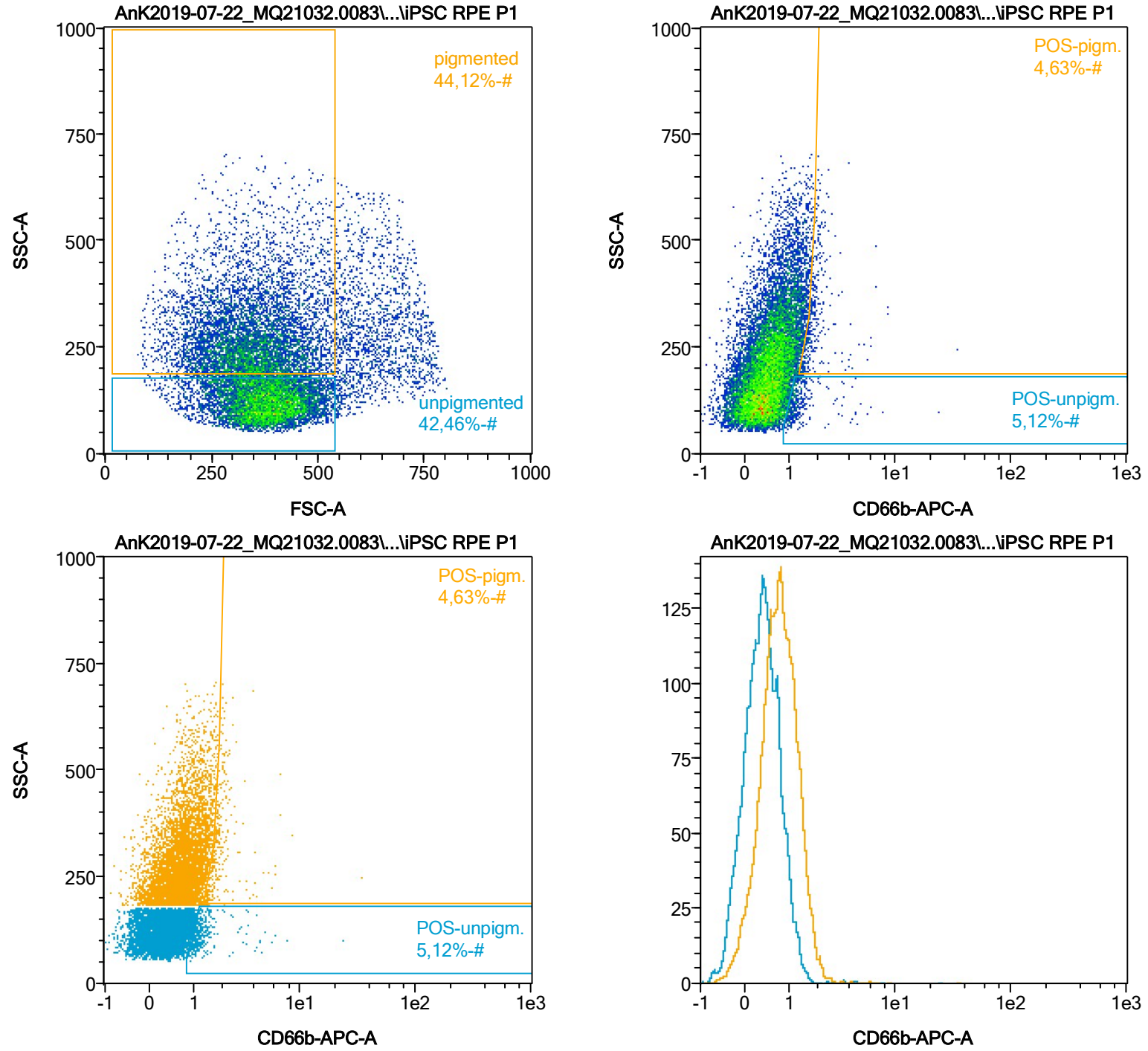

Name	%-#	CD66b-APC-A Mean	CD66b-APC-A Median
POS-unpigm.	5,12	1,45	1,17
POS-pigm.	4,63	2,10	1,80

Date:2024-06-14

Operator: Sebastian Knoebel

Version:2.13.0+54.g95abf0f4

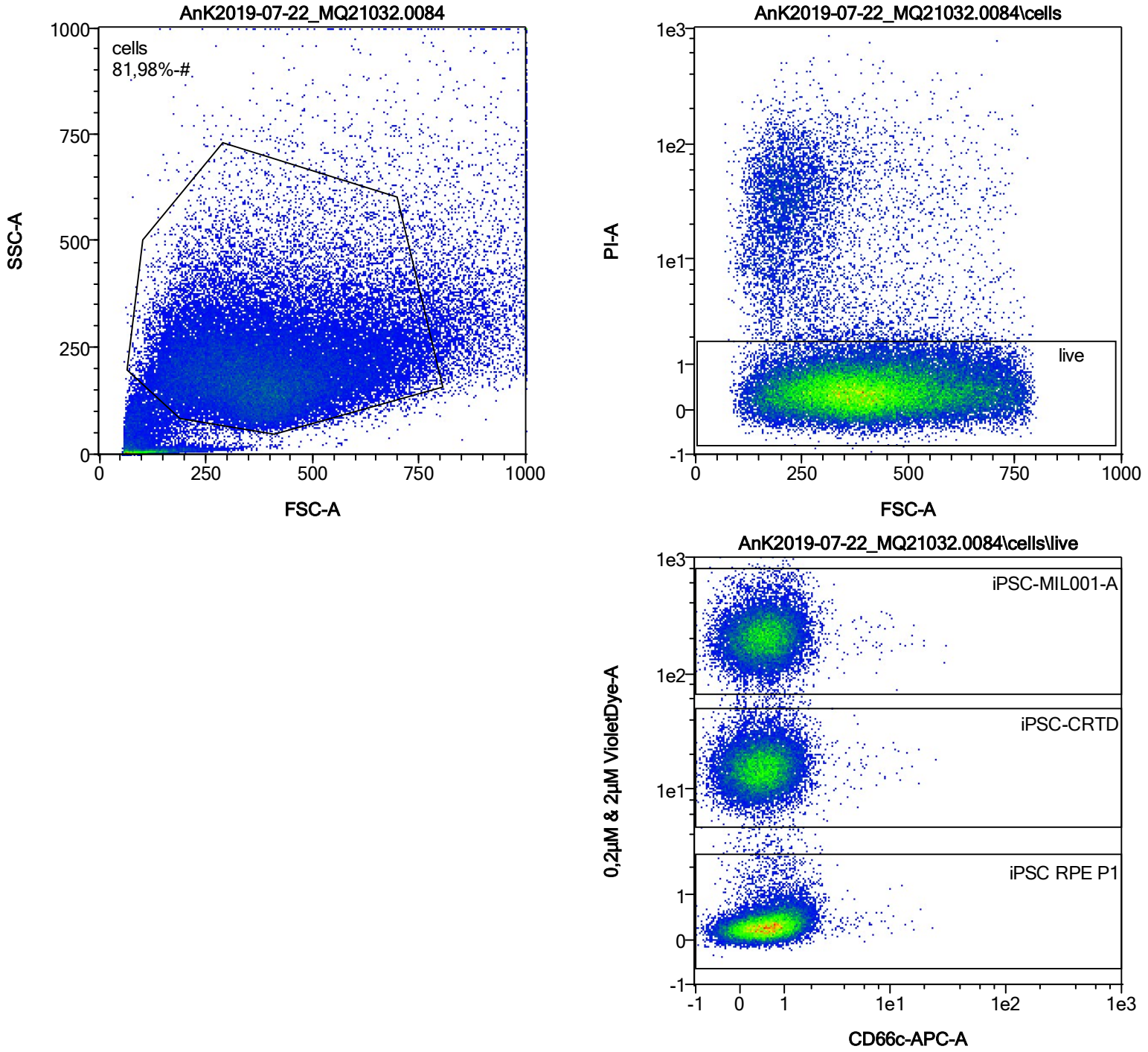

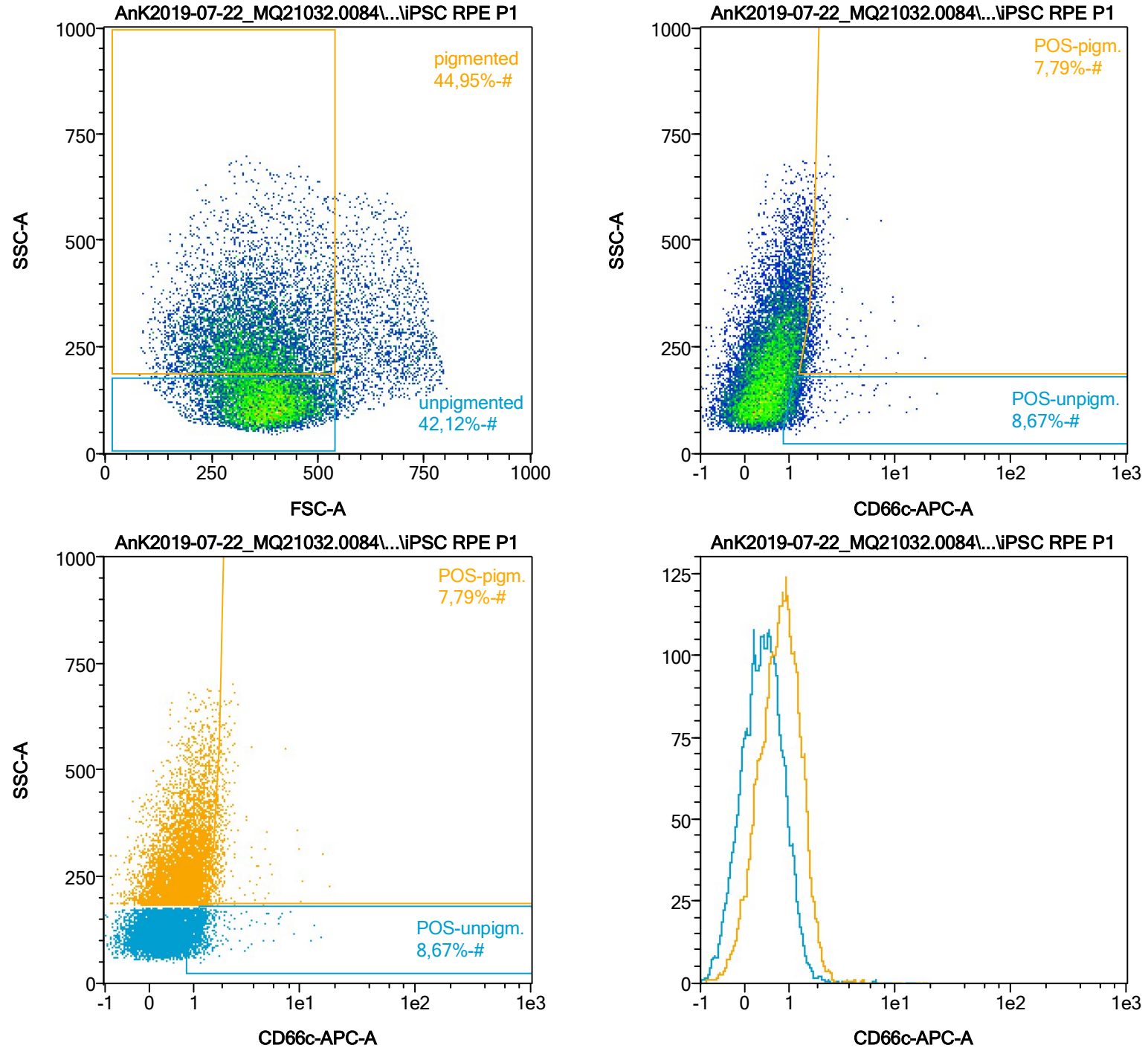

Name	%-#	CD66c-APC-A Mean	CD66c-APC-A Median
POS-unpigm.	8,67	1,47	1,20
POS-pigm.	7,79	2,04	1,80

Date:2024-06-14

Operator: Sebastian Knoebel

Version:2.13.0+54.g95abf0f4

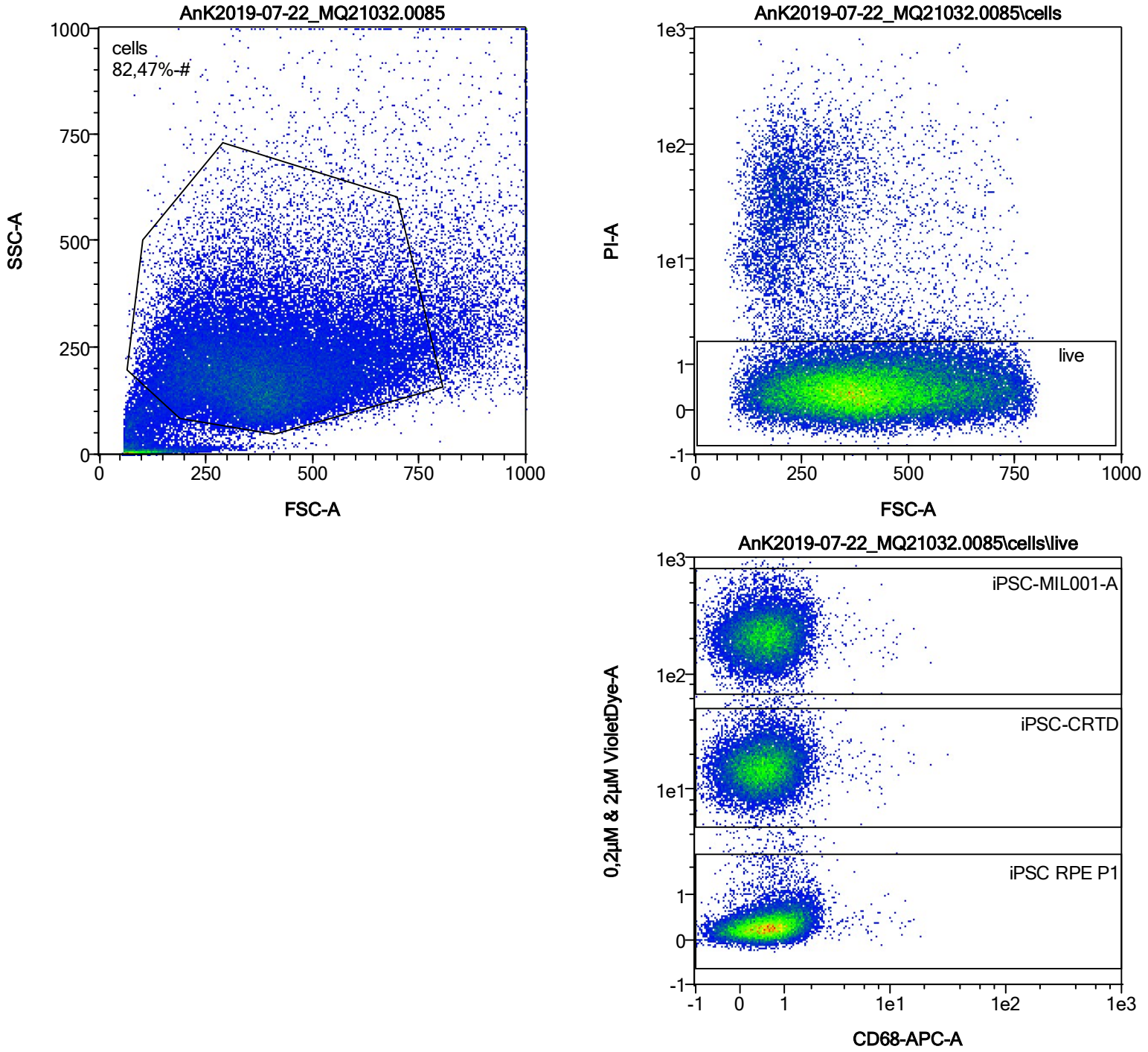

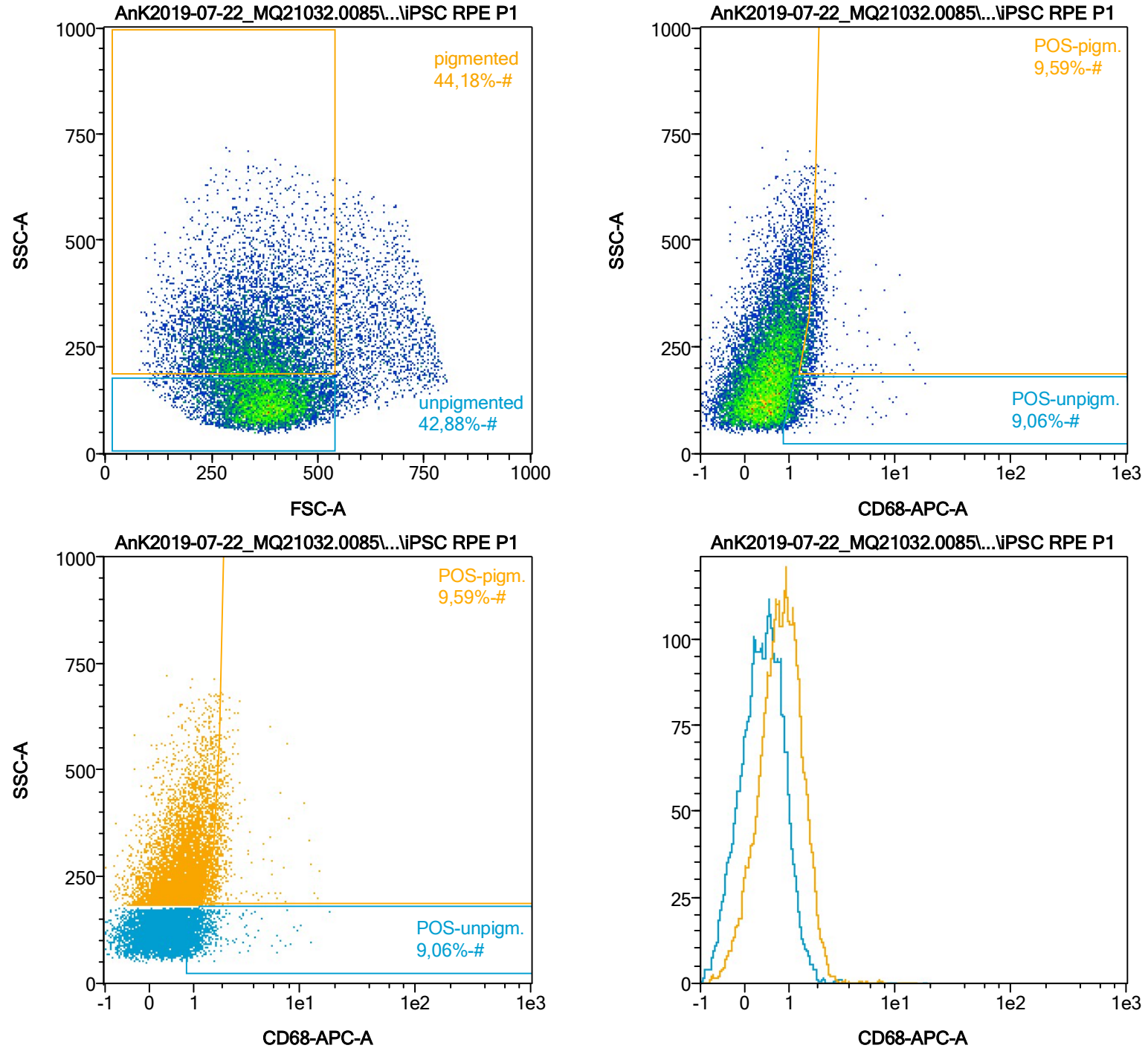

Name	%-#	CD68-APC-A Mean	CD68-APC-A Median
POS-unpigm.	9,06	1,50	1,22
POS-pigm.	9,59	2,09	1,83

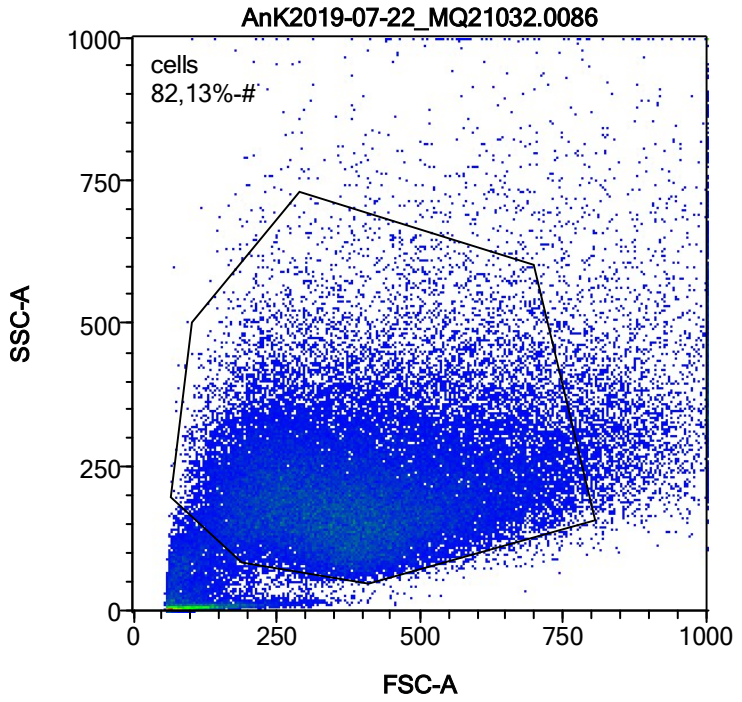

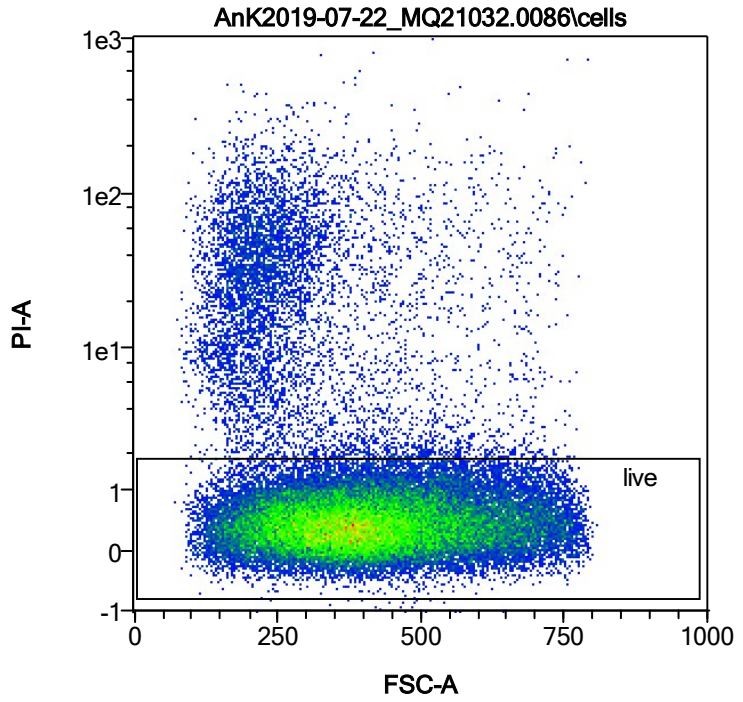

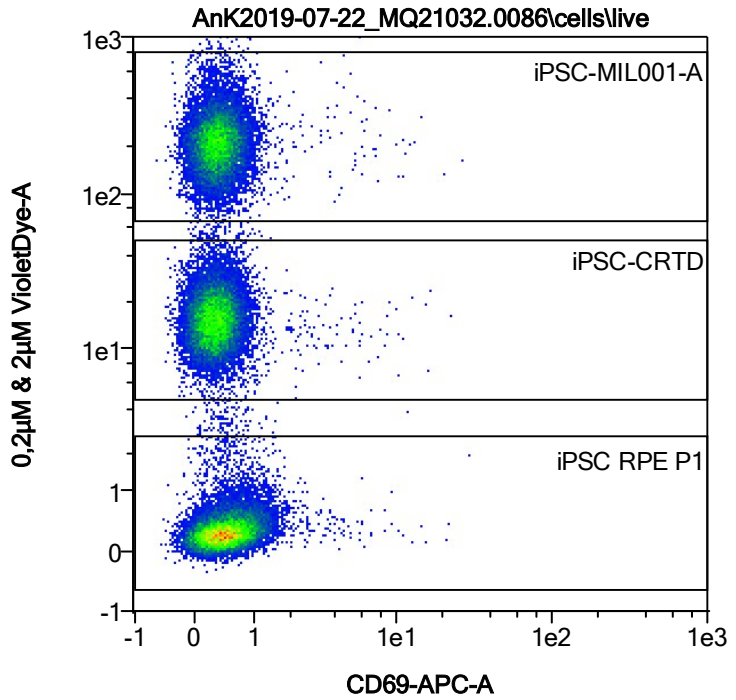

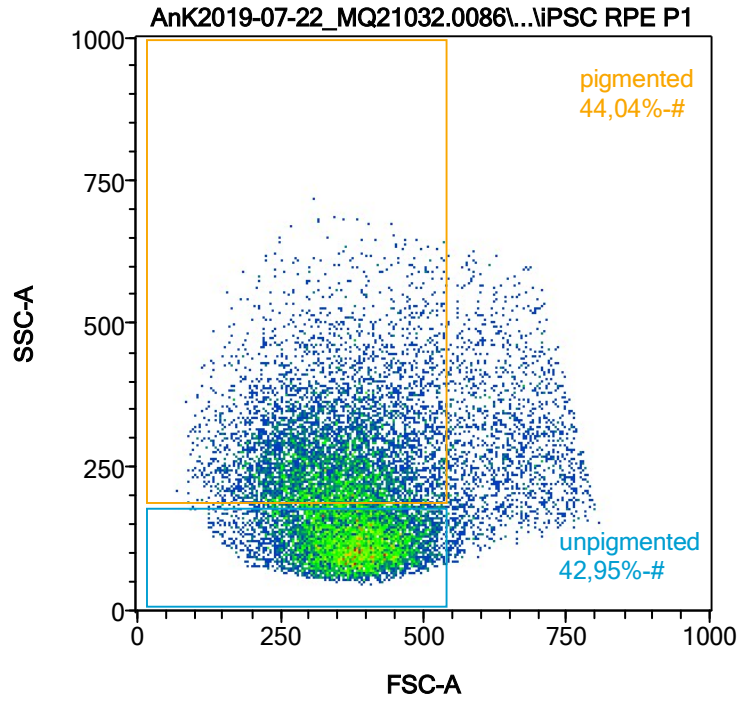

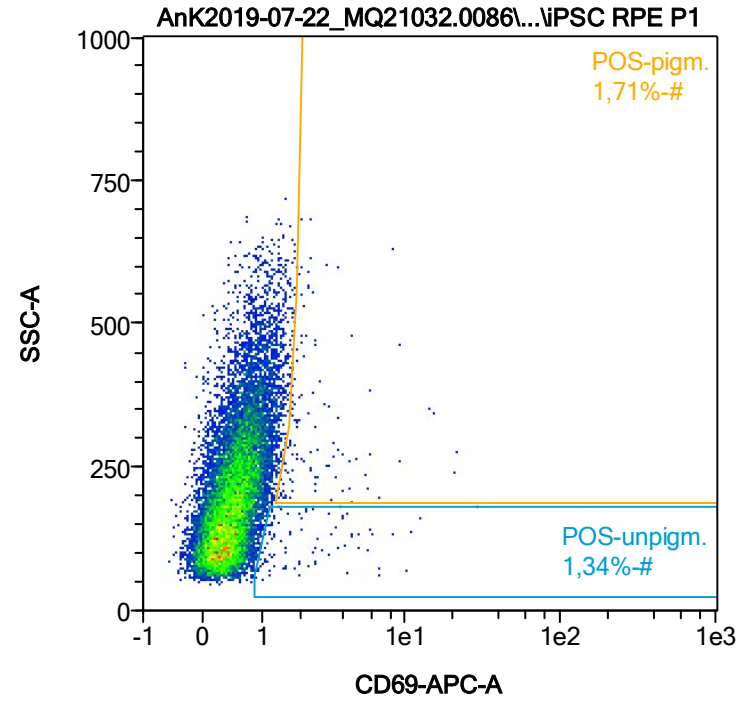

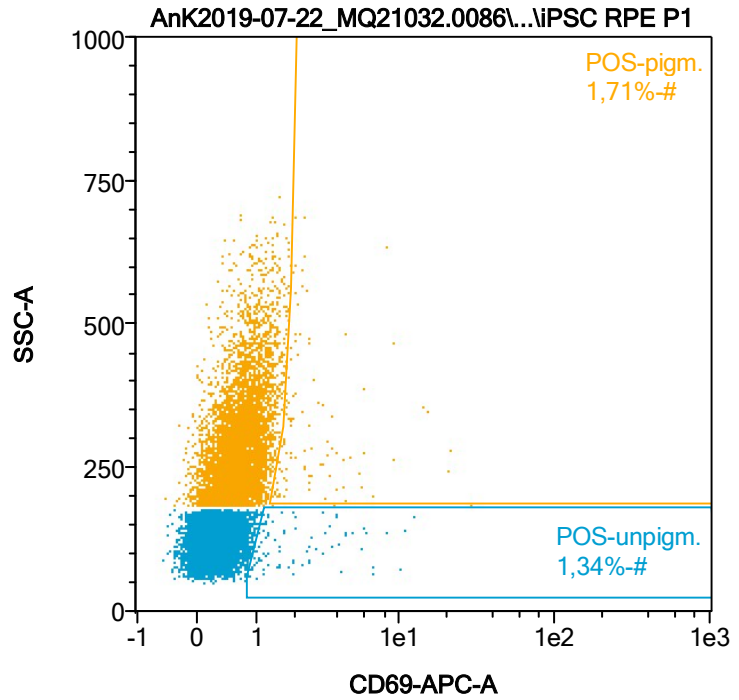

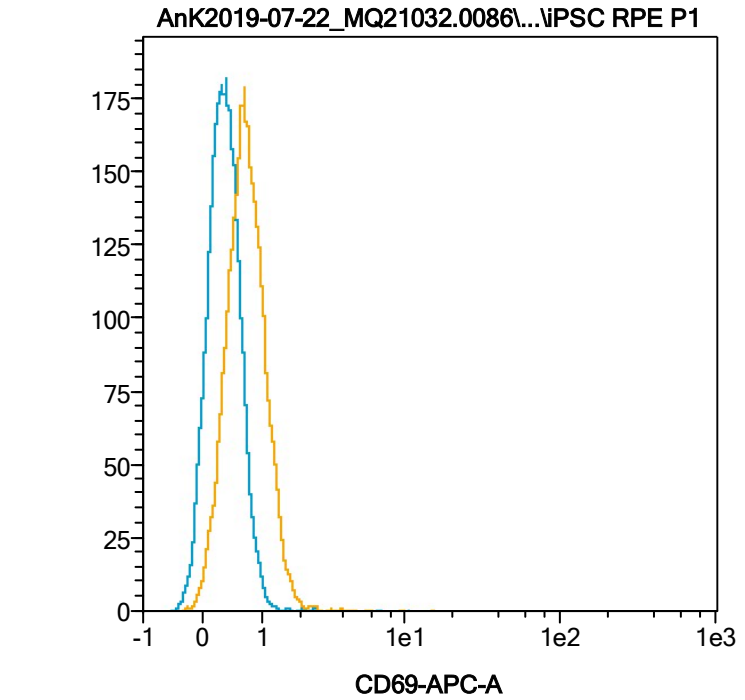

Name	%-#	CD69-APC-A Mean	CD69-APC-A Median
POS-unpigm.	1,34	2,45	1,50
POS-pigm.	1,71	3,36	2,03

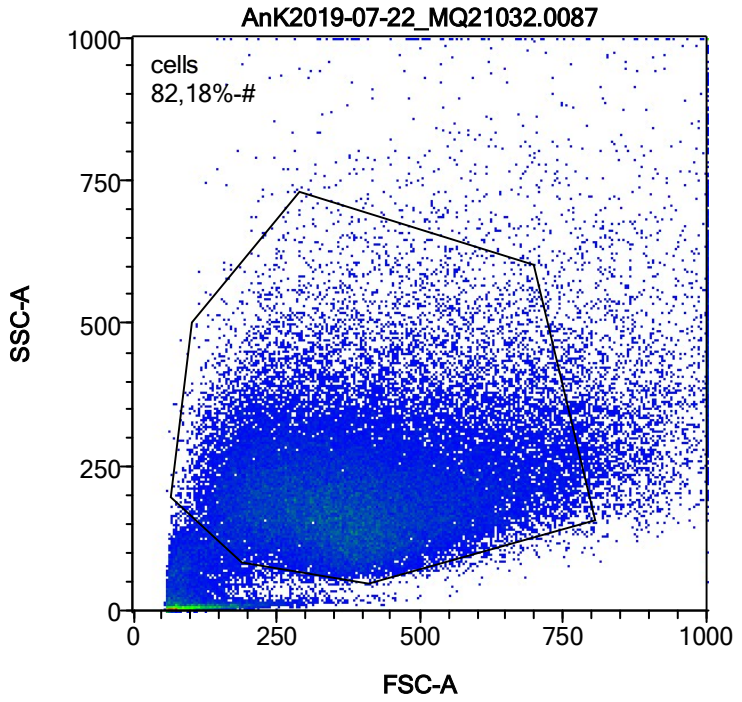

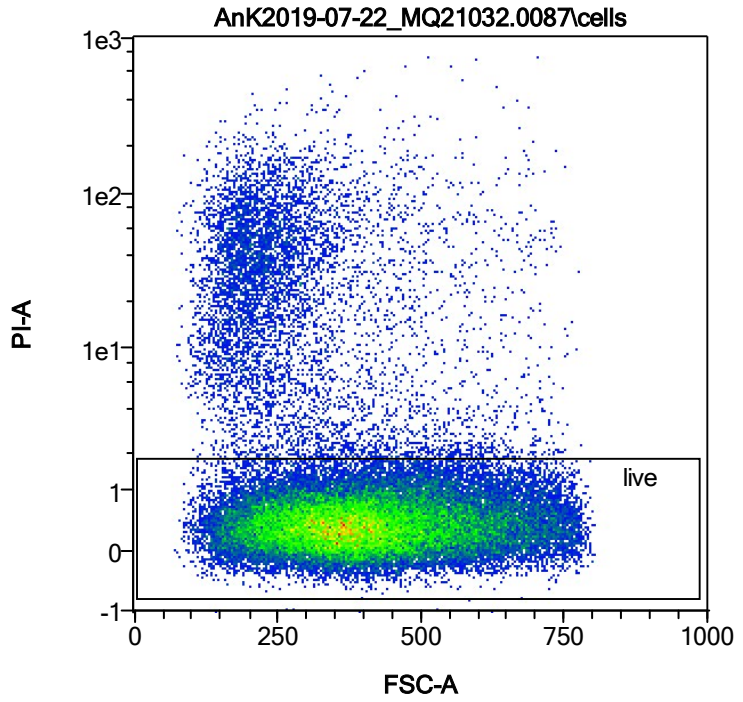

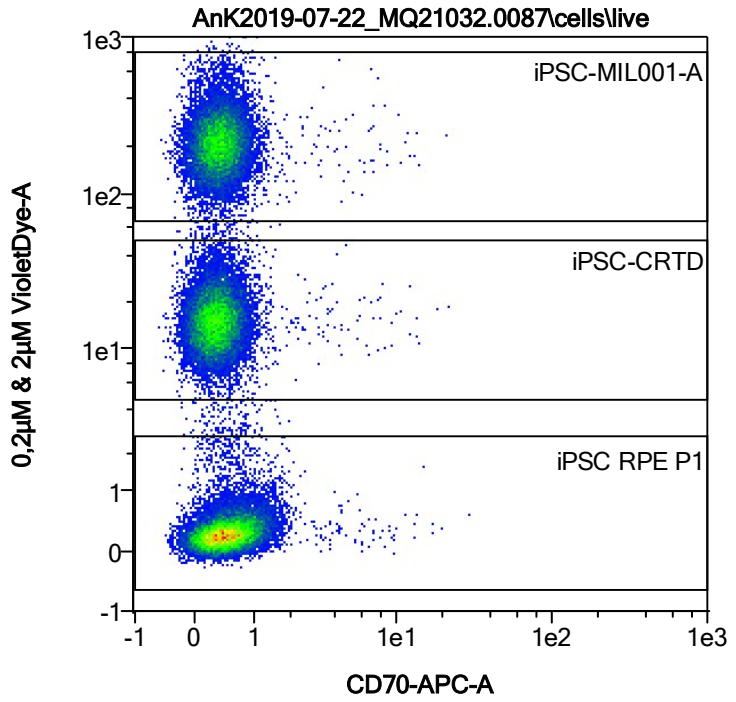

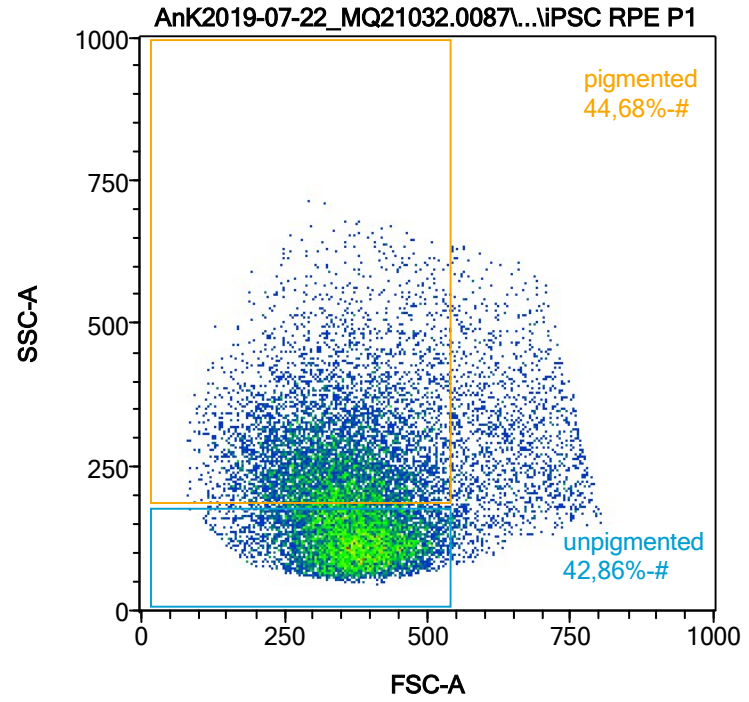

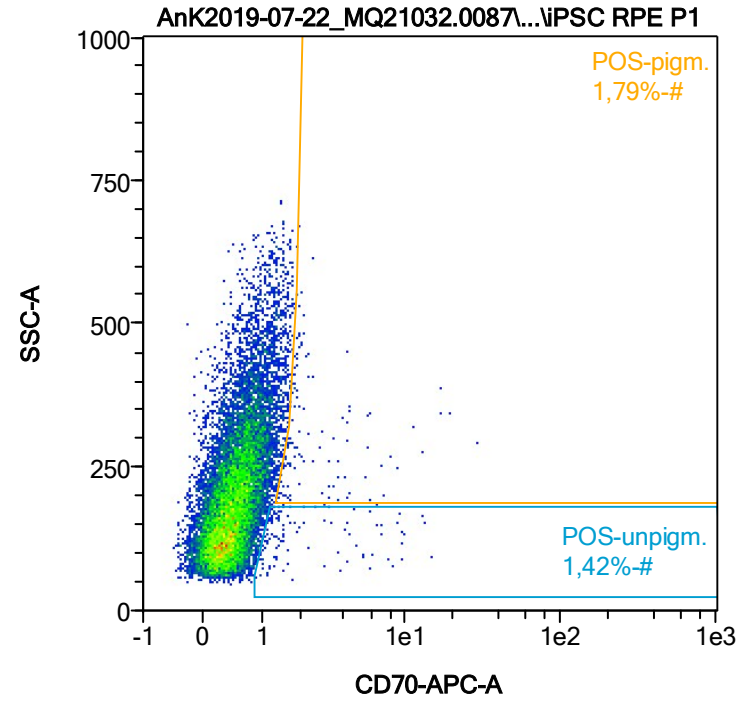

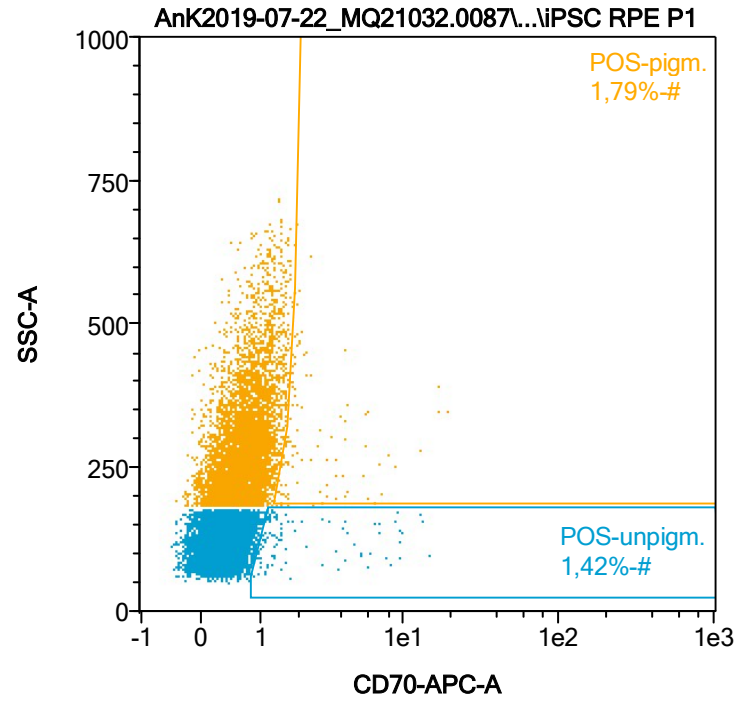

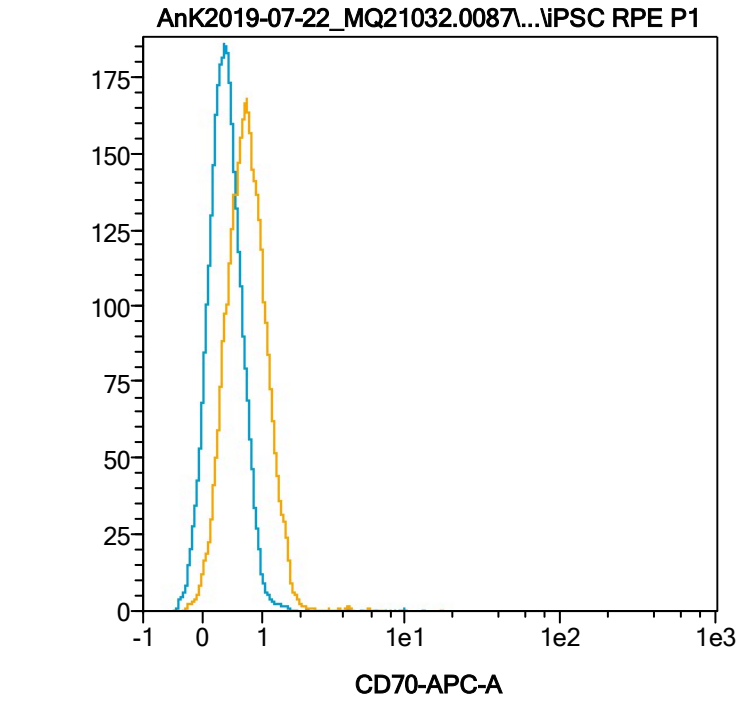

Name	%-#	CD70-APC-A Mean	CD70-APC-A Median
POS-unpigm.	1,42	2,97	1,37
POS-pigm.	1,79	2,95	1,89

Date:2024-06-14

Operator: Sebastian Knoebel

Version:2.13.0+54.g95abf0f4

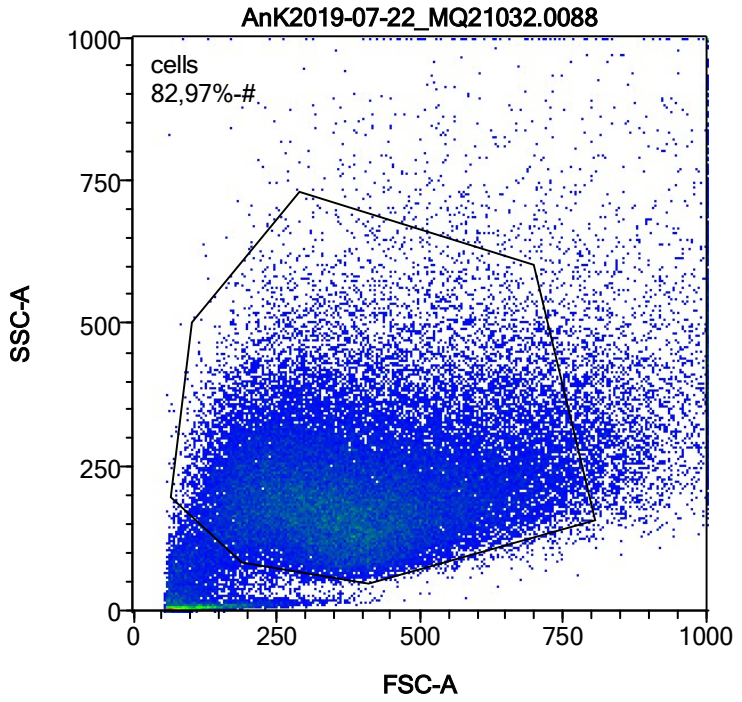

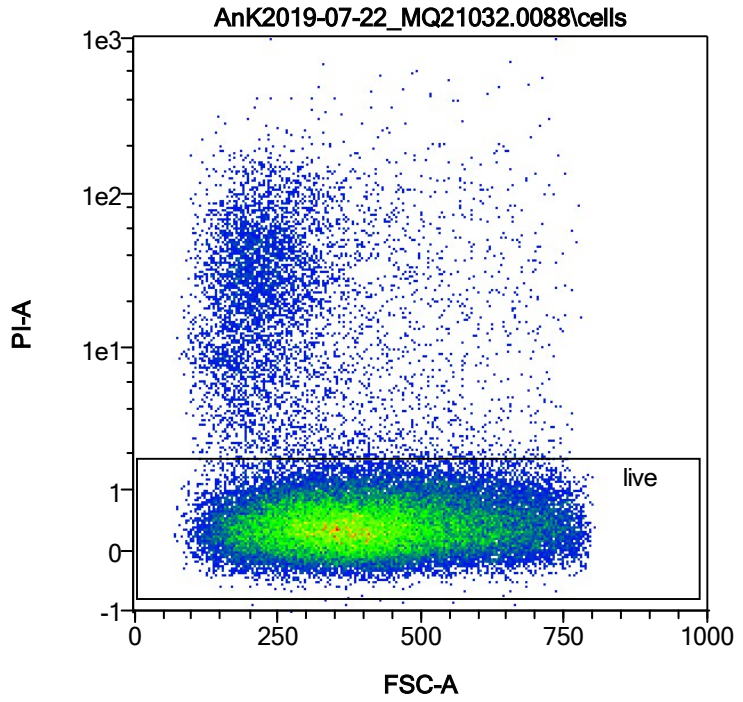

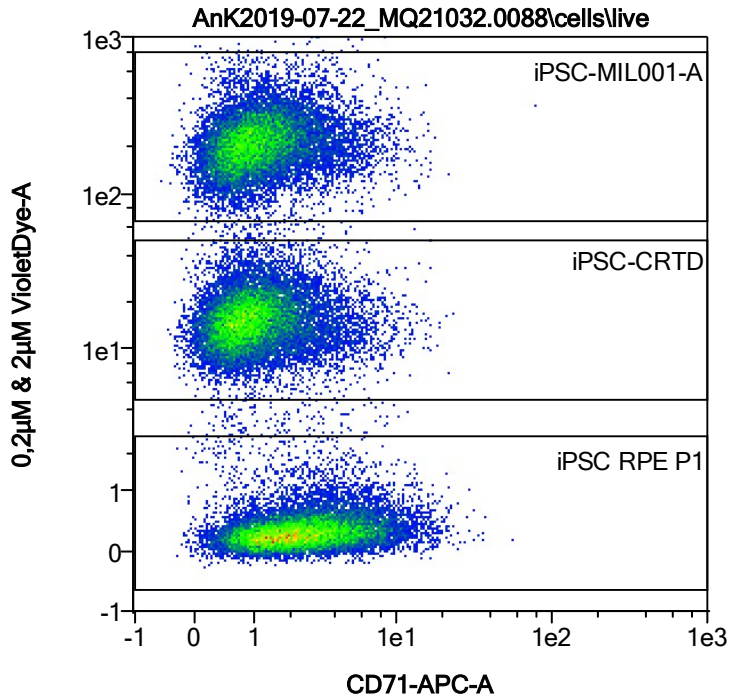

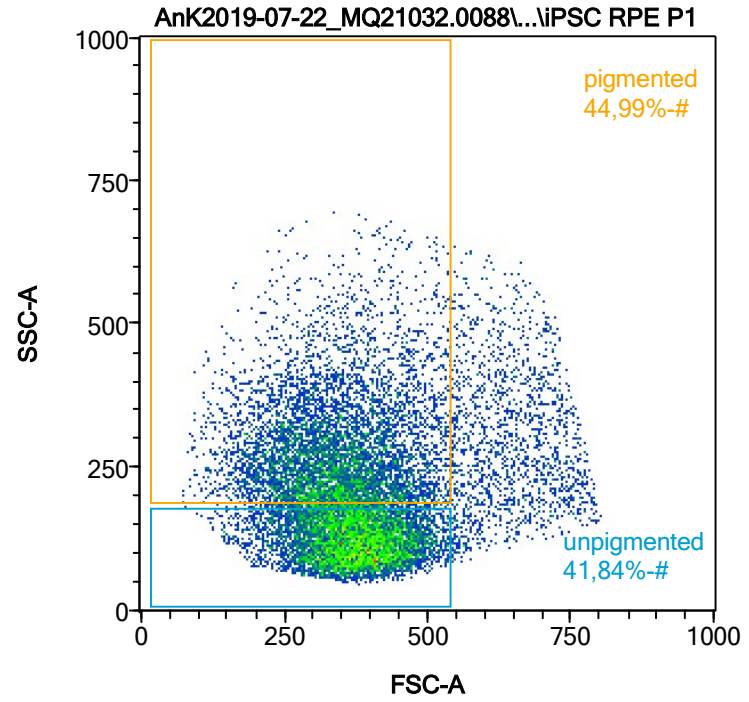

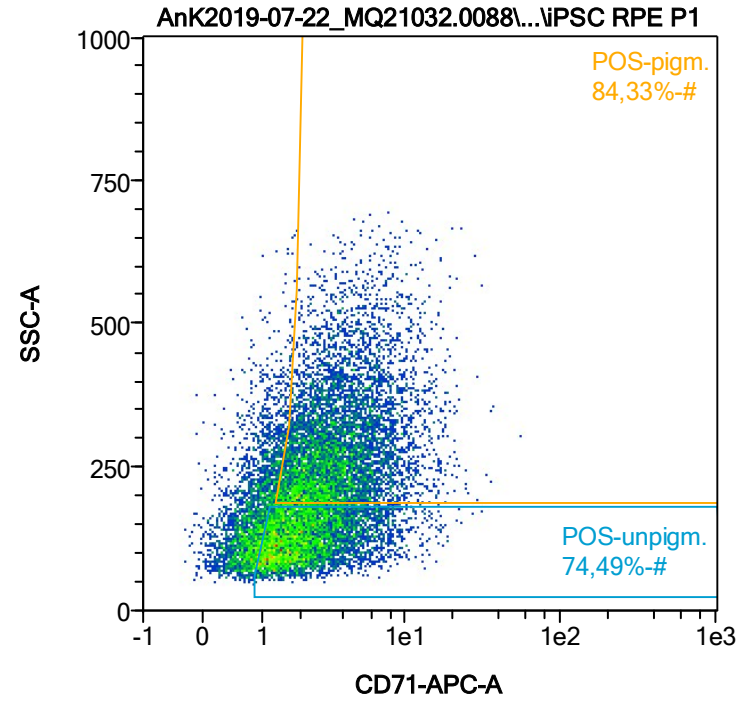

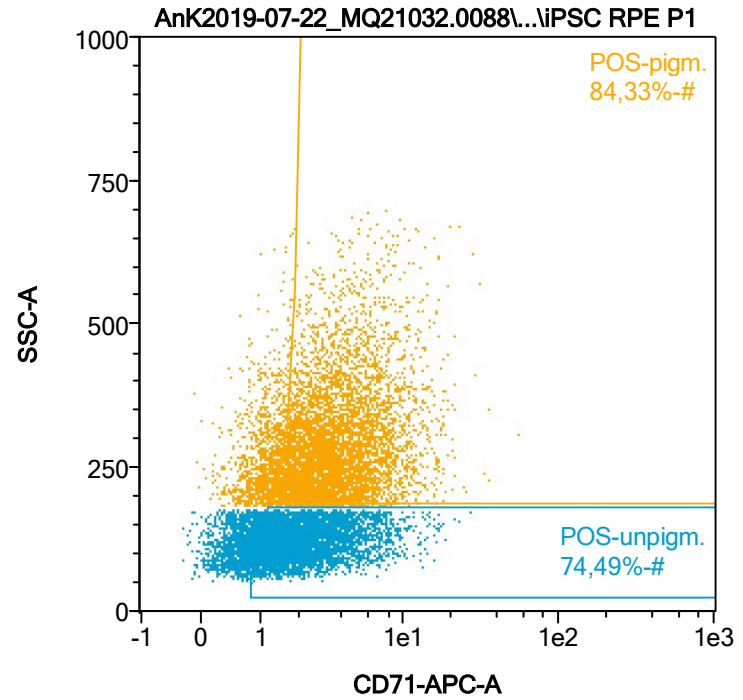

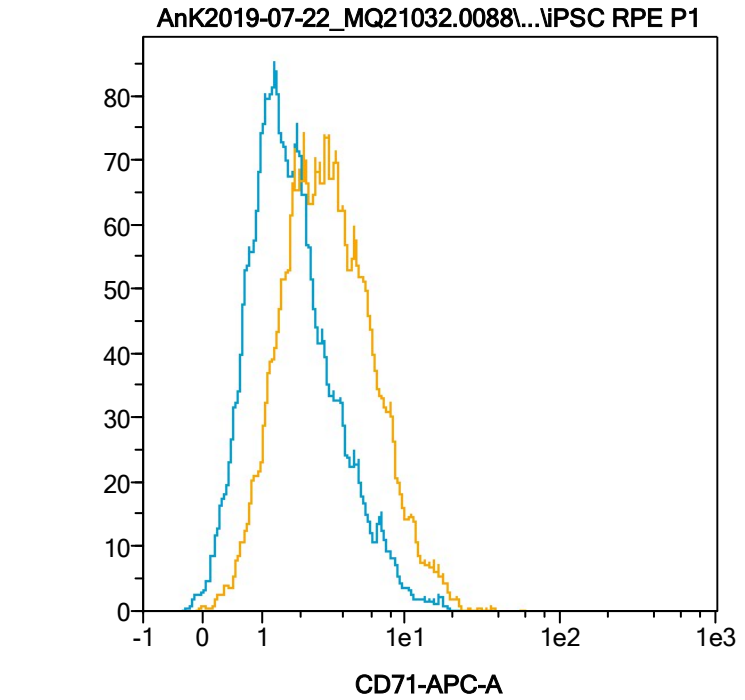

Name	%-#	CD71-APC-A Mean	CD71-APC-A Median
POS-unpigm.	74,49	2,66	1,99
POS-pigm.	84,33	4,34	3,40

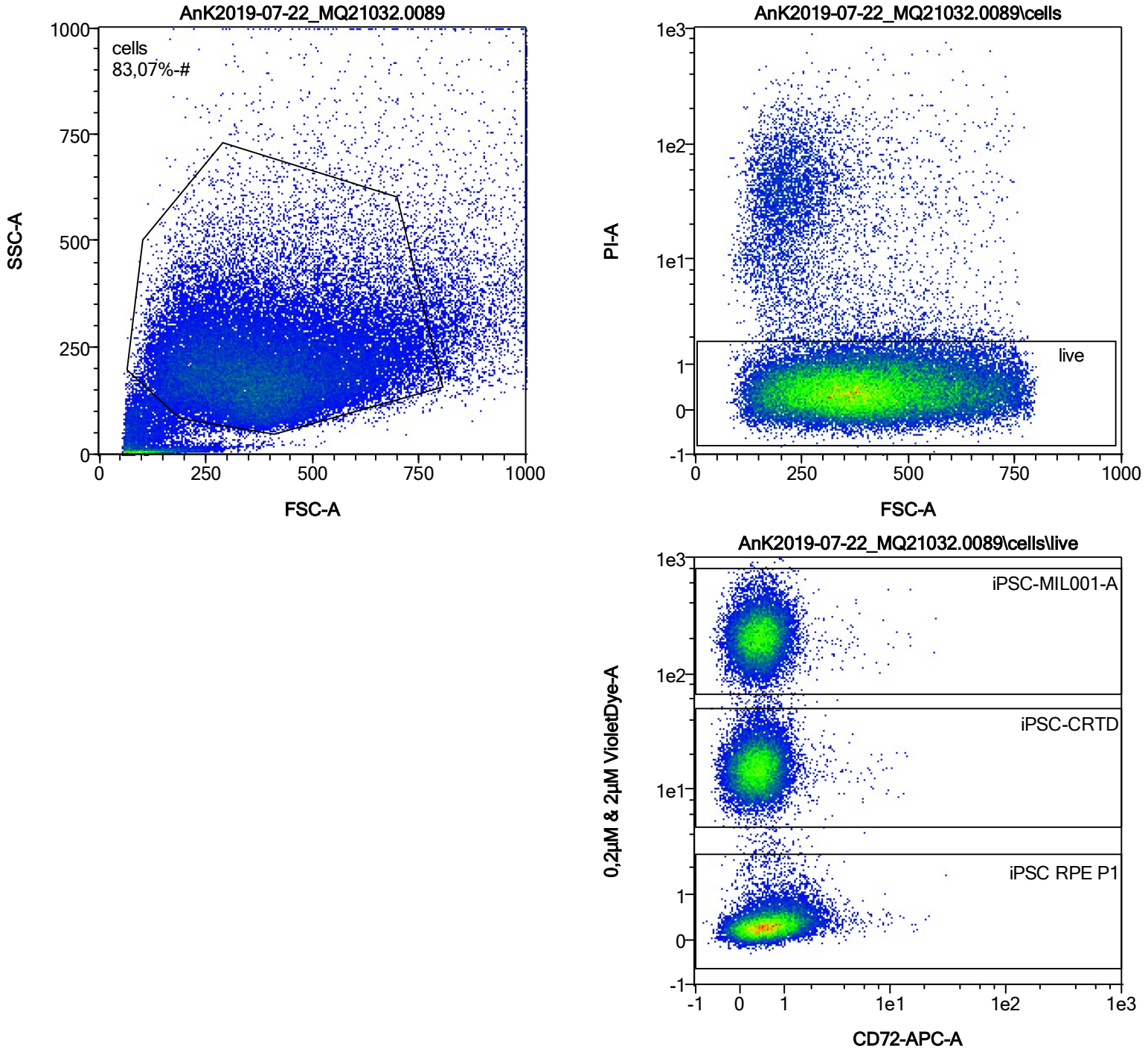

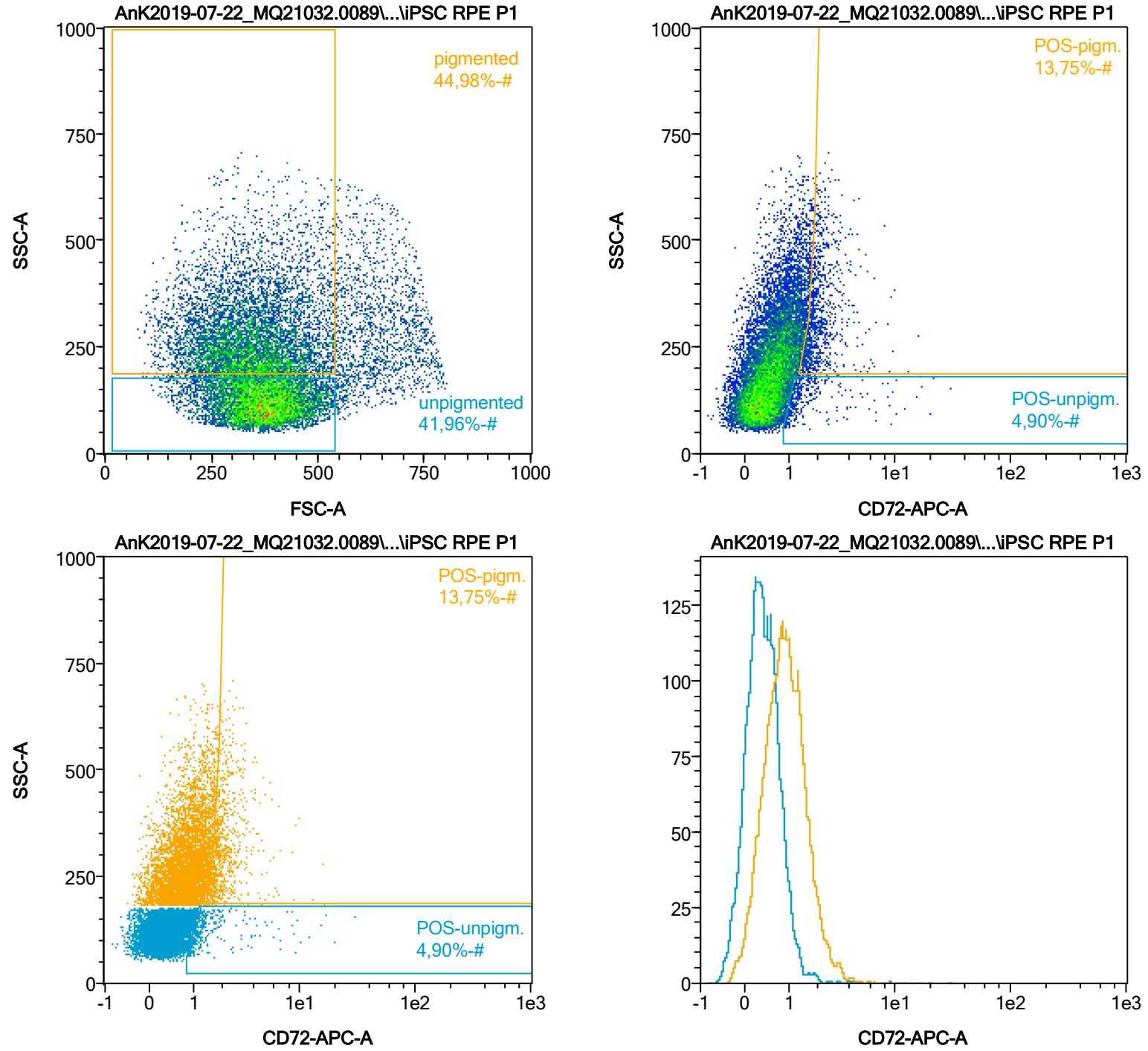

Name	%-#	CD72-APC-A Mean	CD72-APC-A Median
POS-unpigm.	4,90	1,99	1,24
POS-pigm.	13,75	2,20	1,93

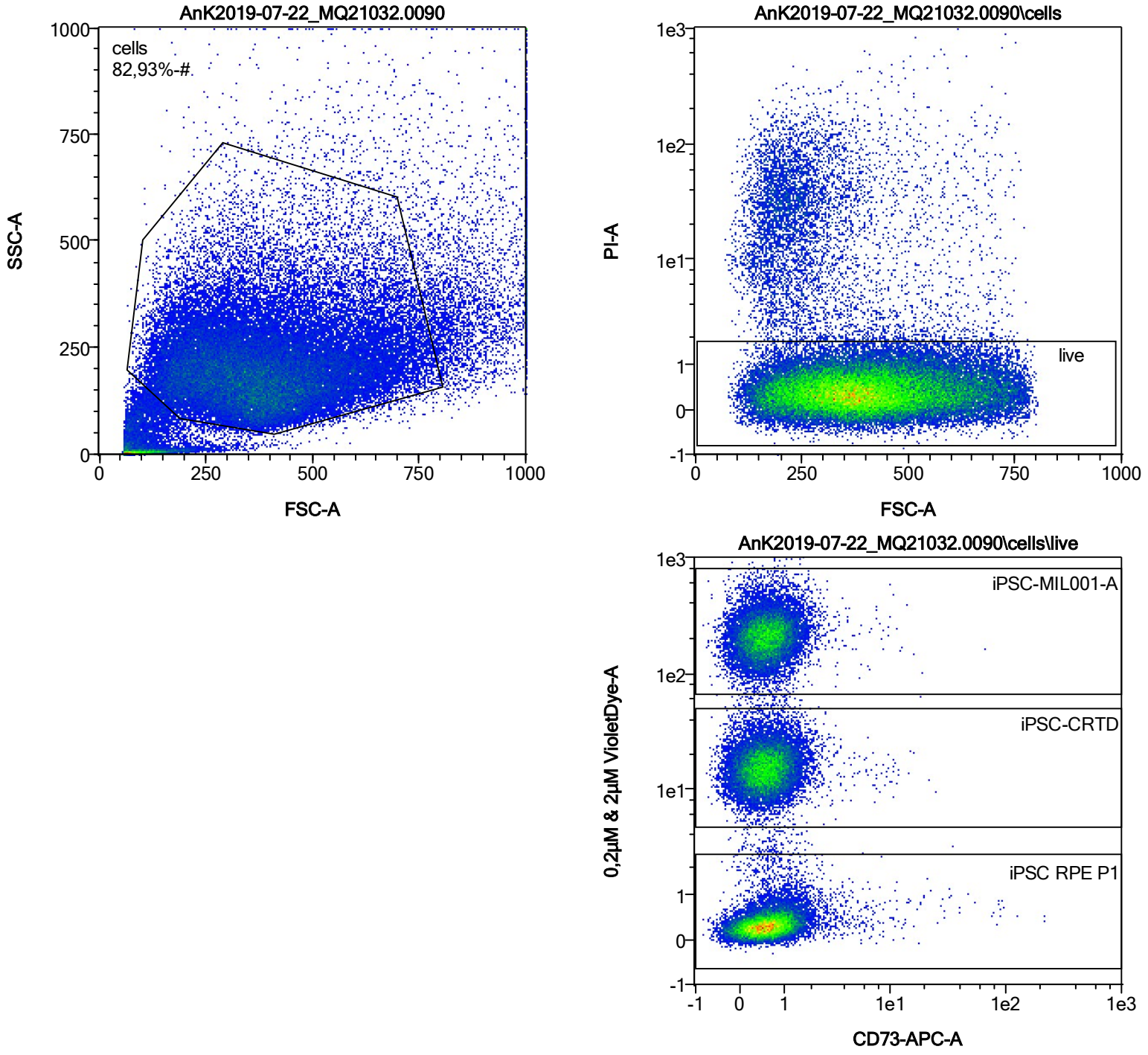

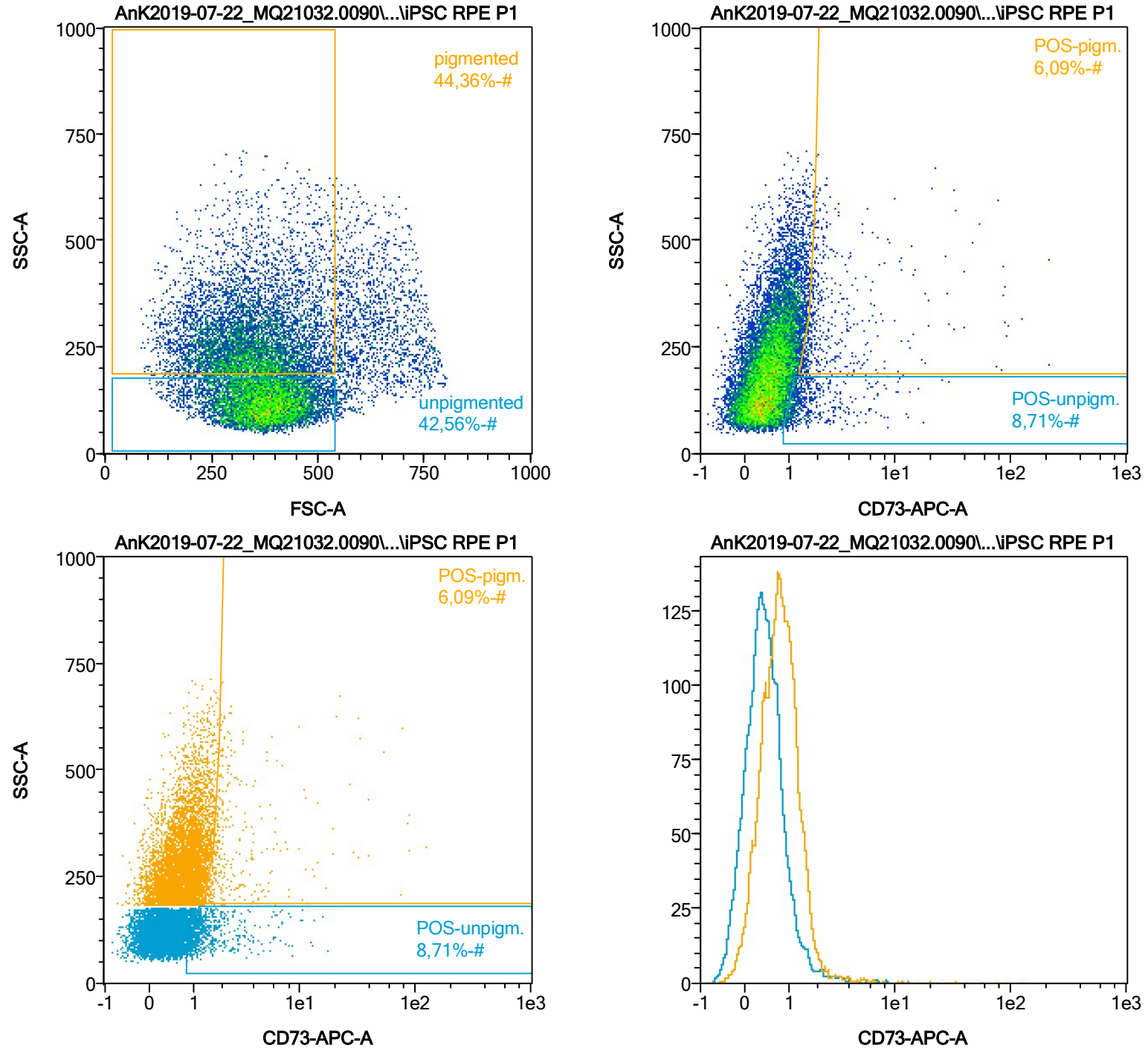

Name	%-#	CD73-APC-A Mean	CD73-APC-A Median
POS-unpigm.	8,71	1,90	1,31
POS-pigm.	6,09	4,74	1,95

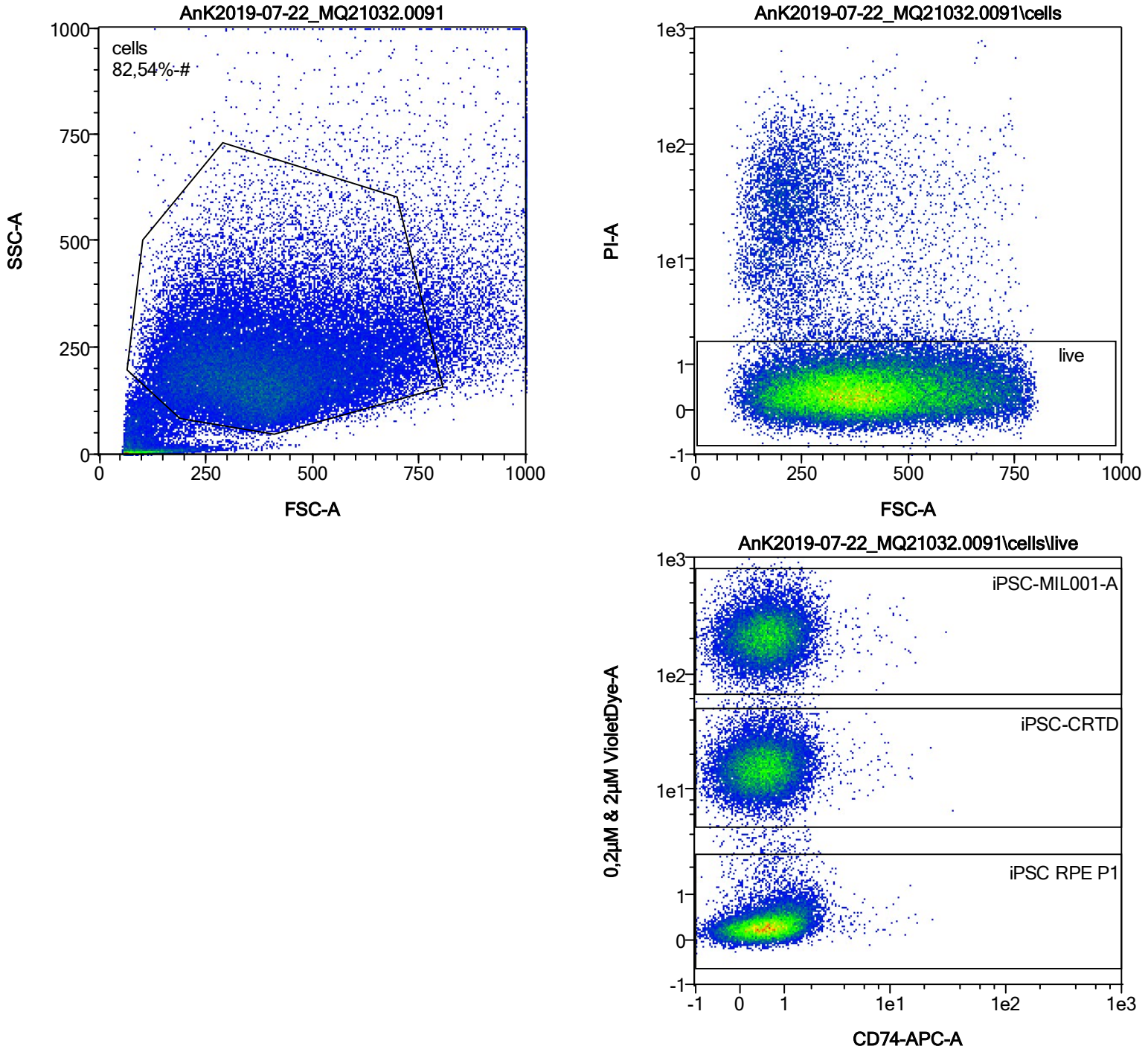

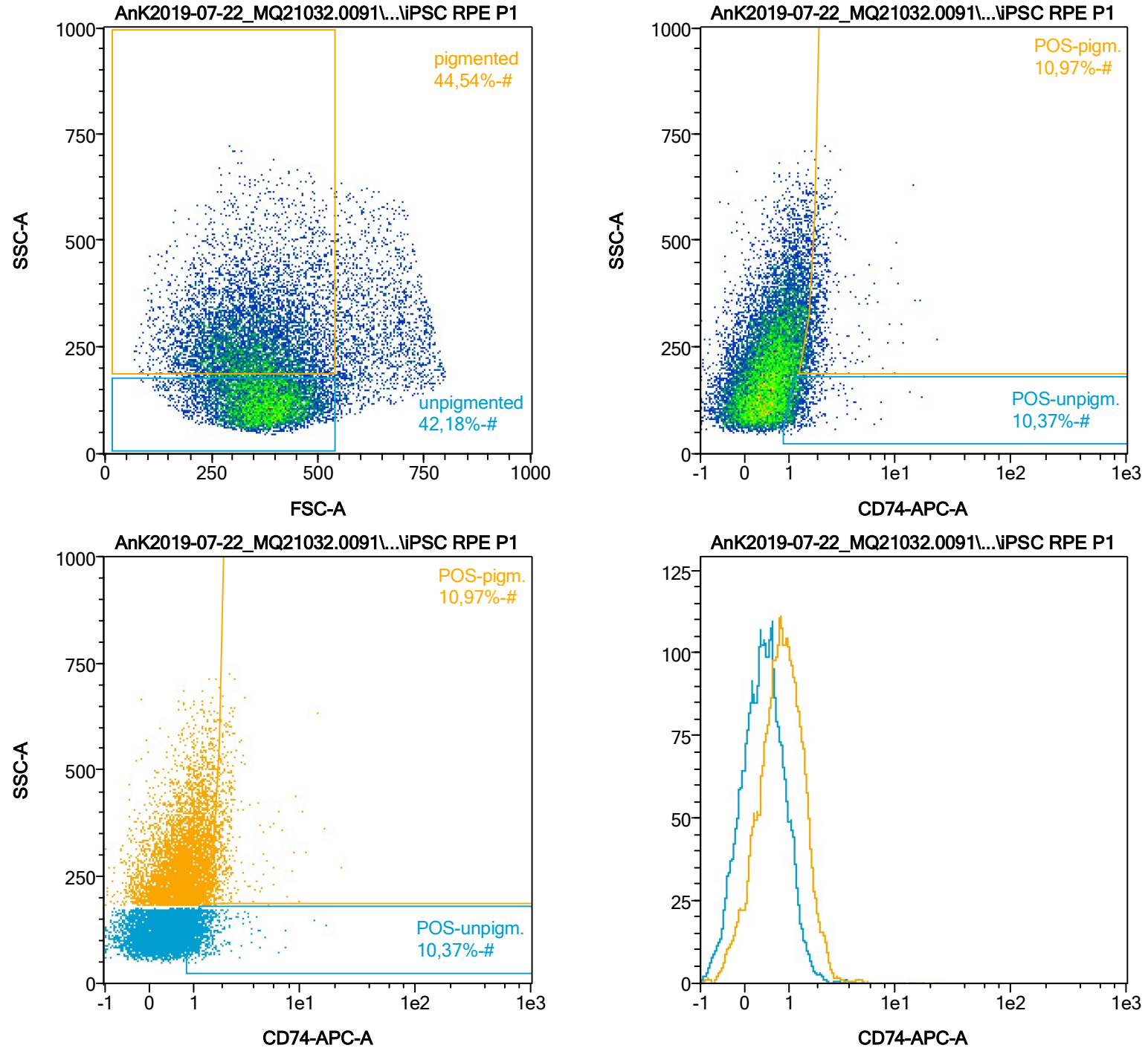

Name	%-#	CD74-APC-A Mean	CD74-APC-A Median
POS-unpigm.	10,37	1,43	1,21
POS-pigm.	10,97	2,12	1,84

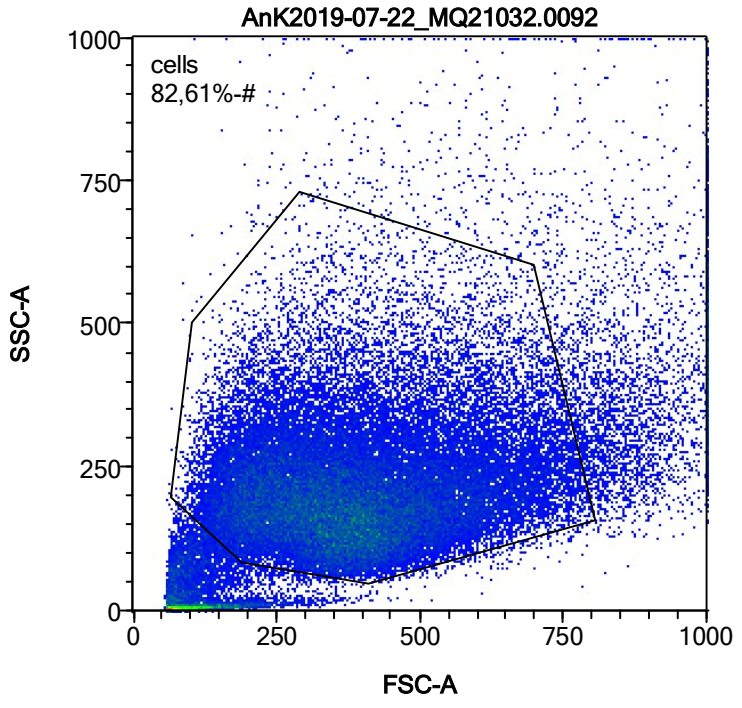

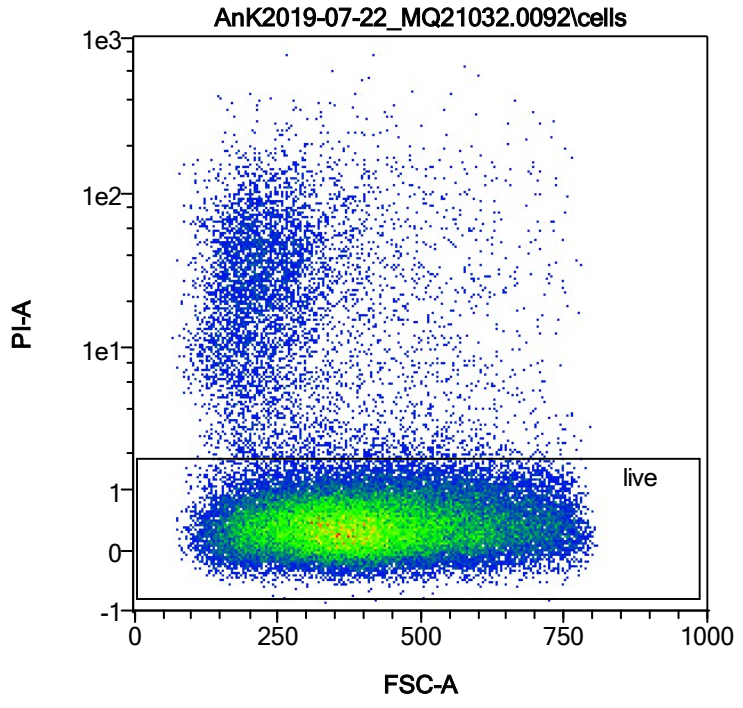

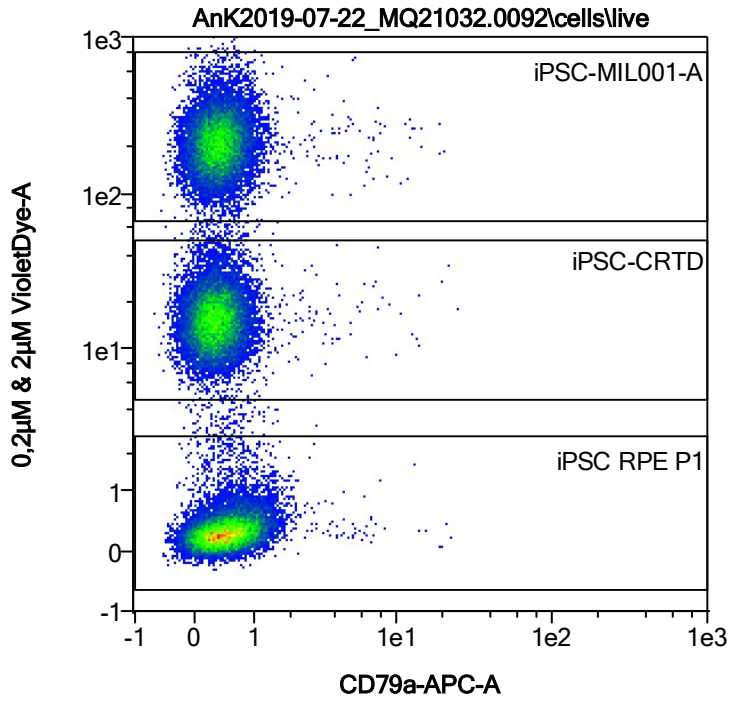

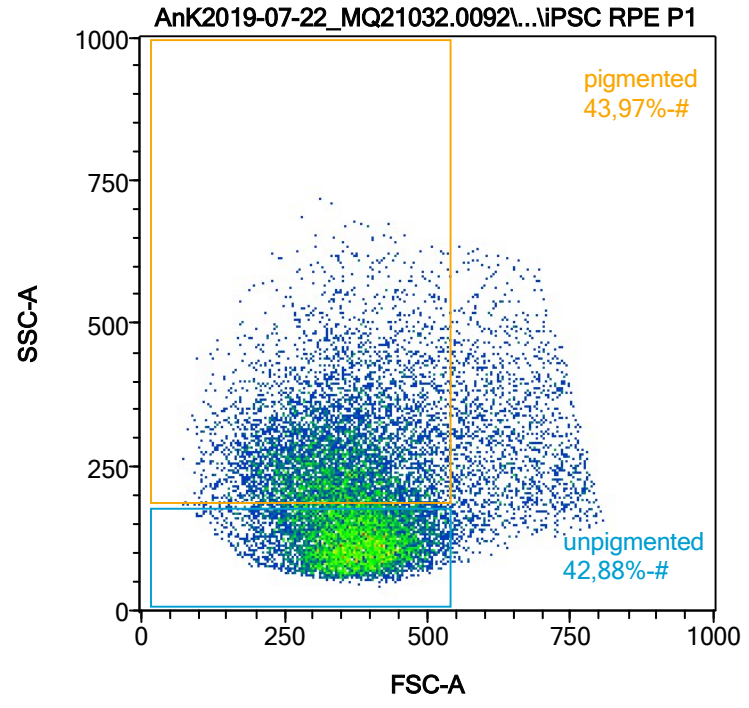

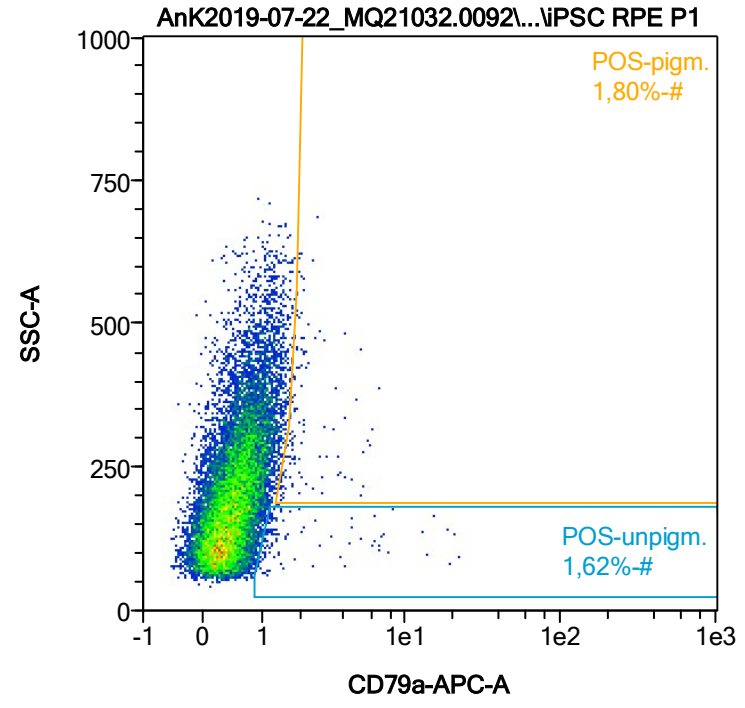

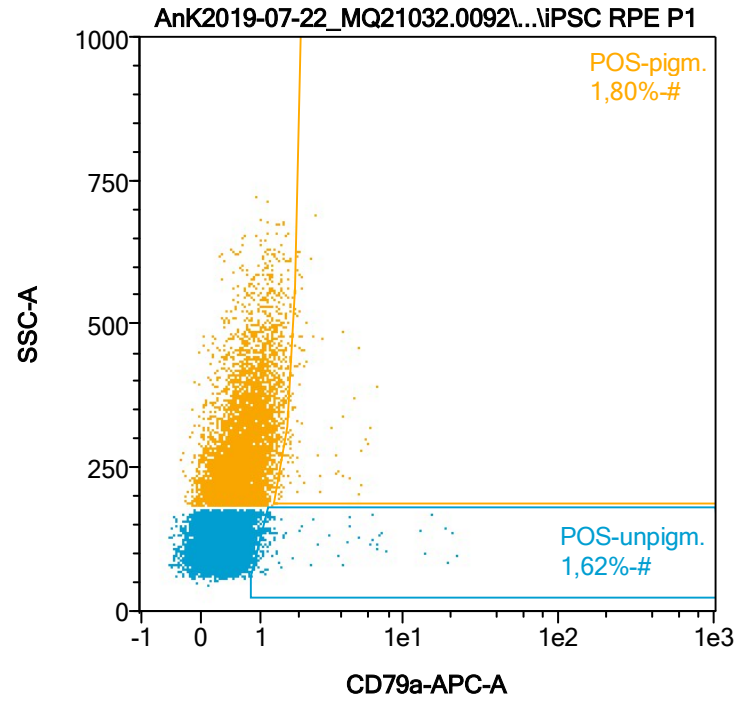

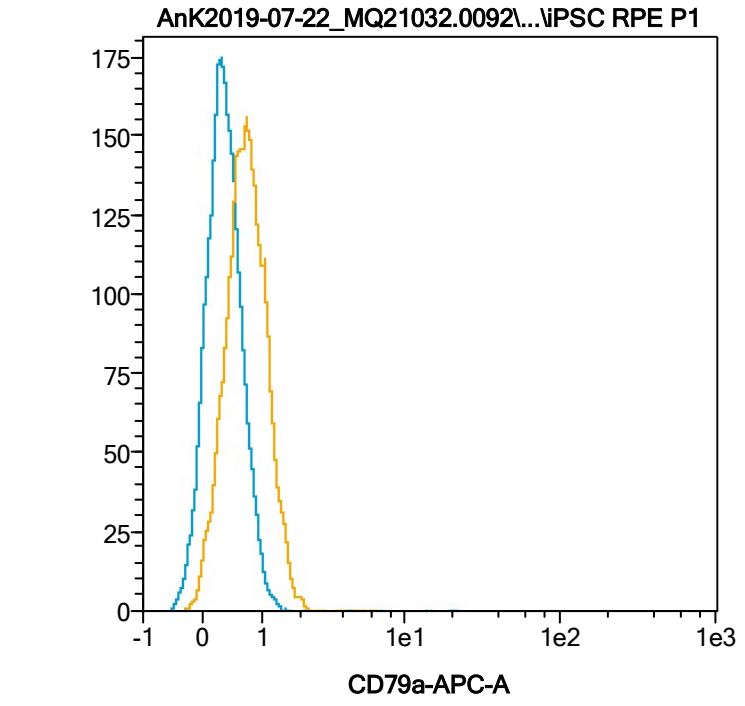

Name	%-#	CD79a-APC-A Mean	CD79a-APC-A Median
POS-unpigm.	1,62	2,97	1,22
POS-pigm.	1,80	2,28	1,87

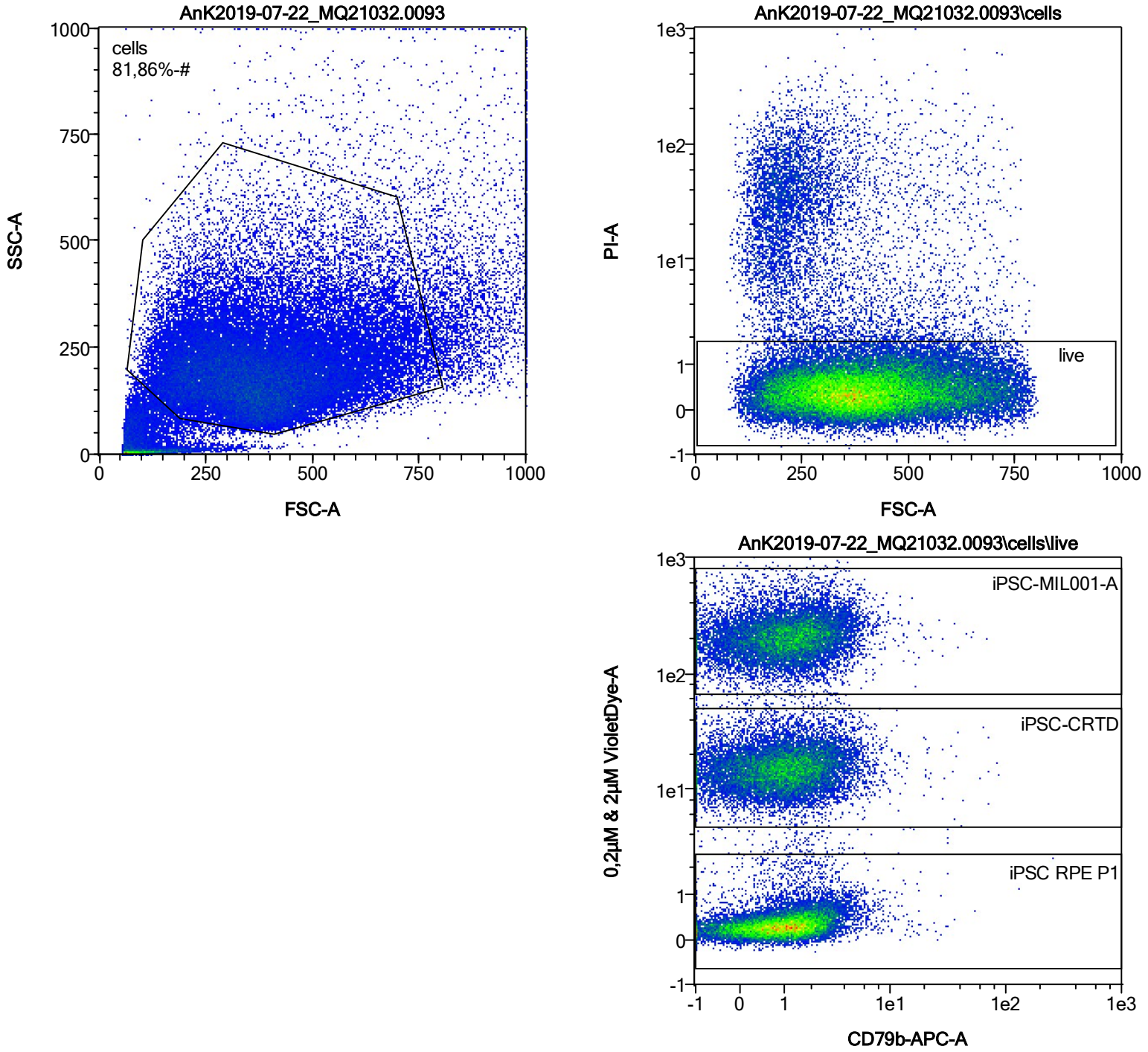

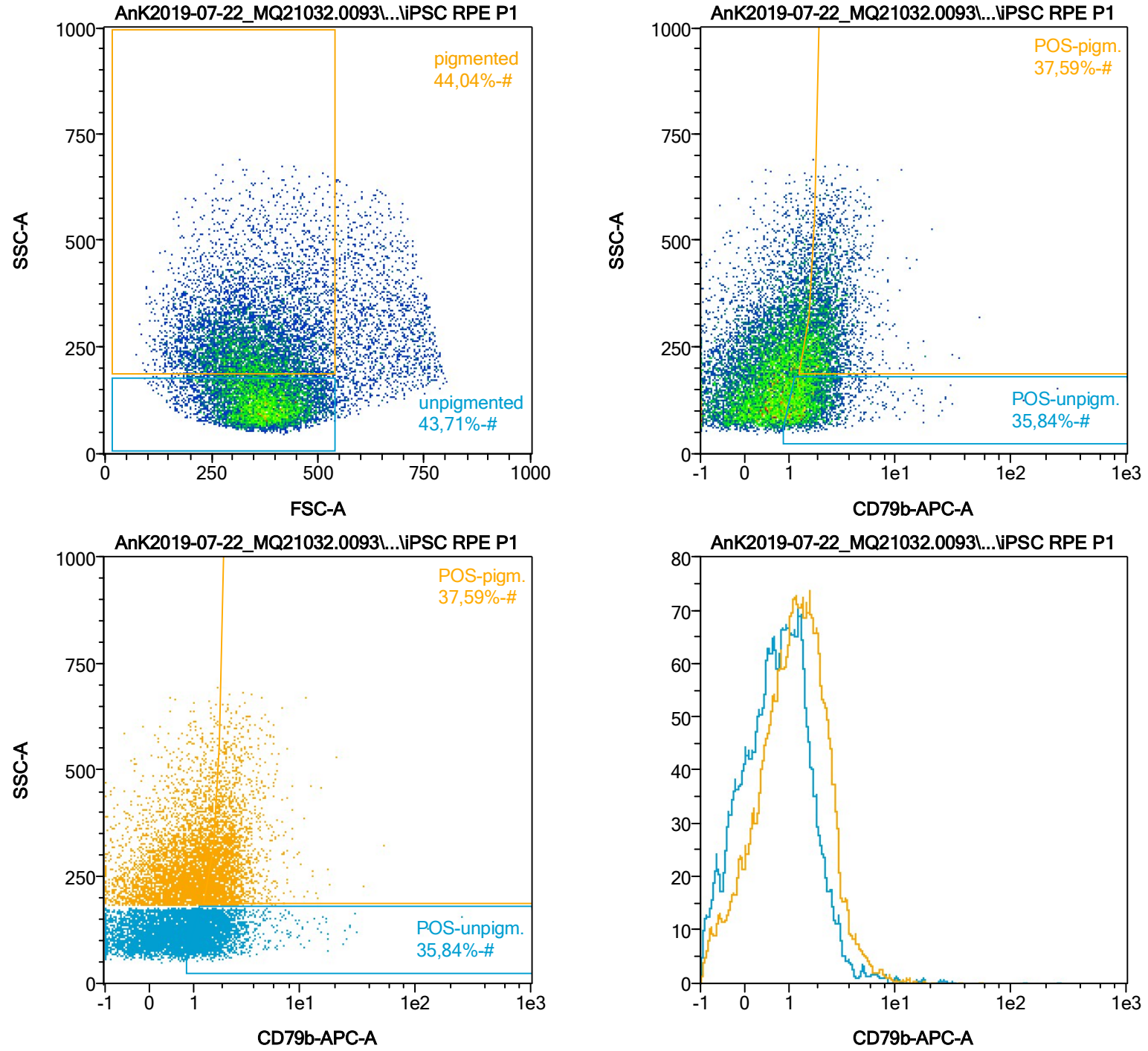

Name	%-#	CD79b-APC-A Mean	CD79b-APC-A Median
POS-unpigm.	35,84	2,13	1,52
POS-pigm.	37,59	2,56	2,17

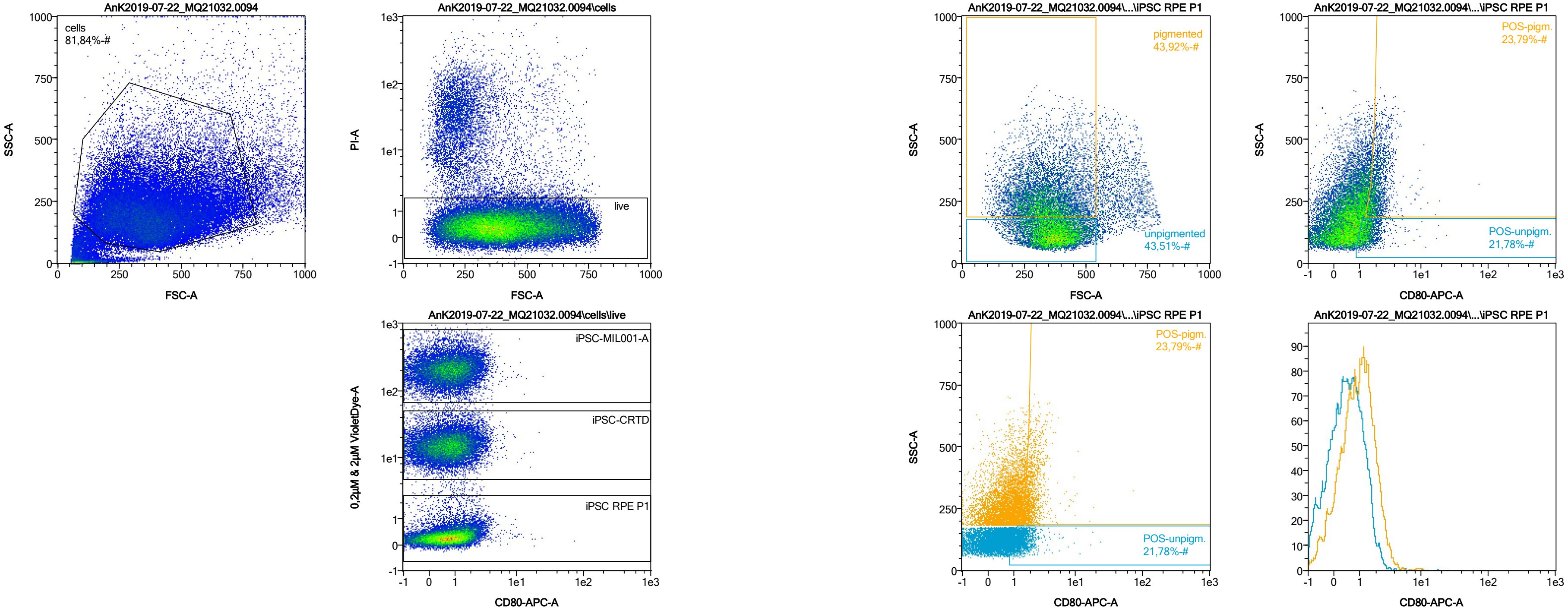

Name	%-#	CD80-APC-A Mean	CD80-APC-A Median
POS-unpigm.	21,78	1,54	1,36
POS-pigm.	23,79	2,23	1,97

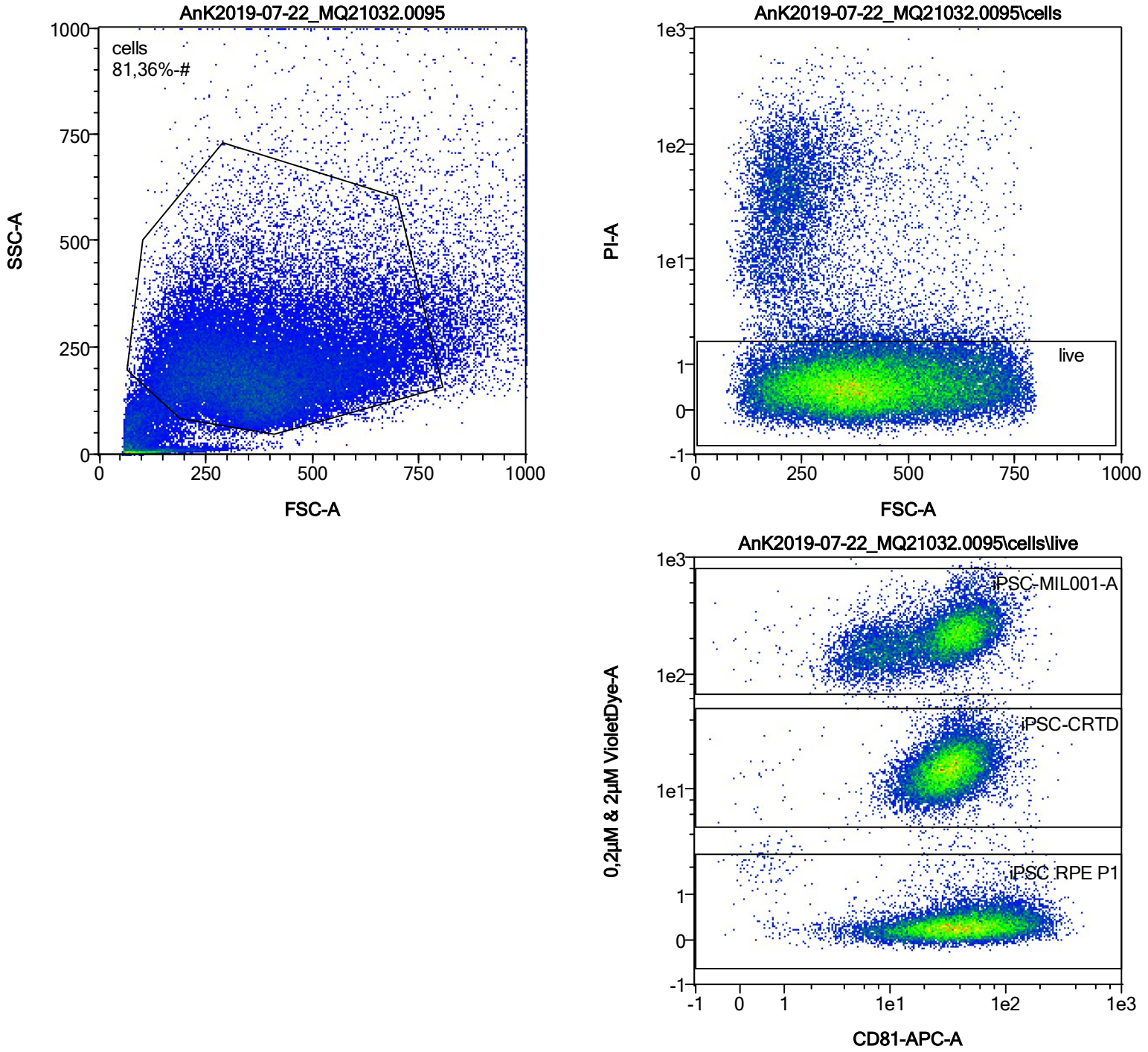

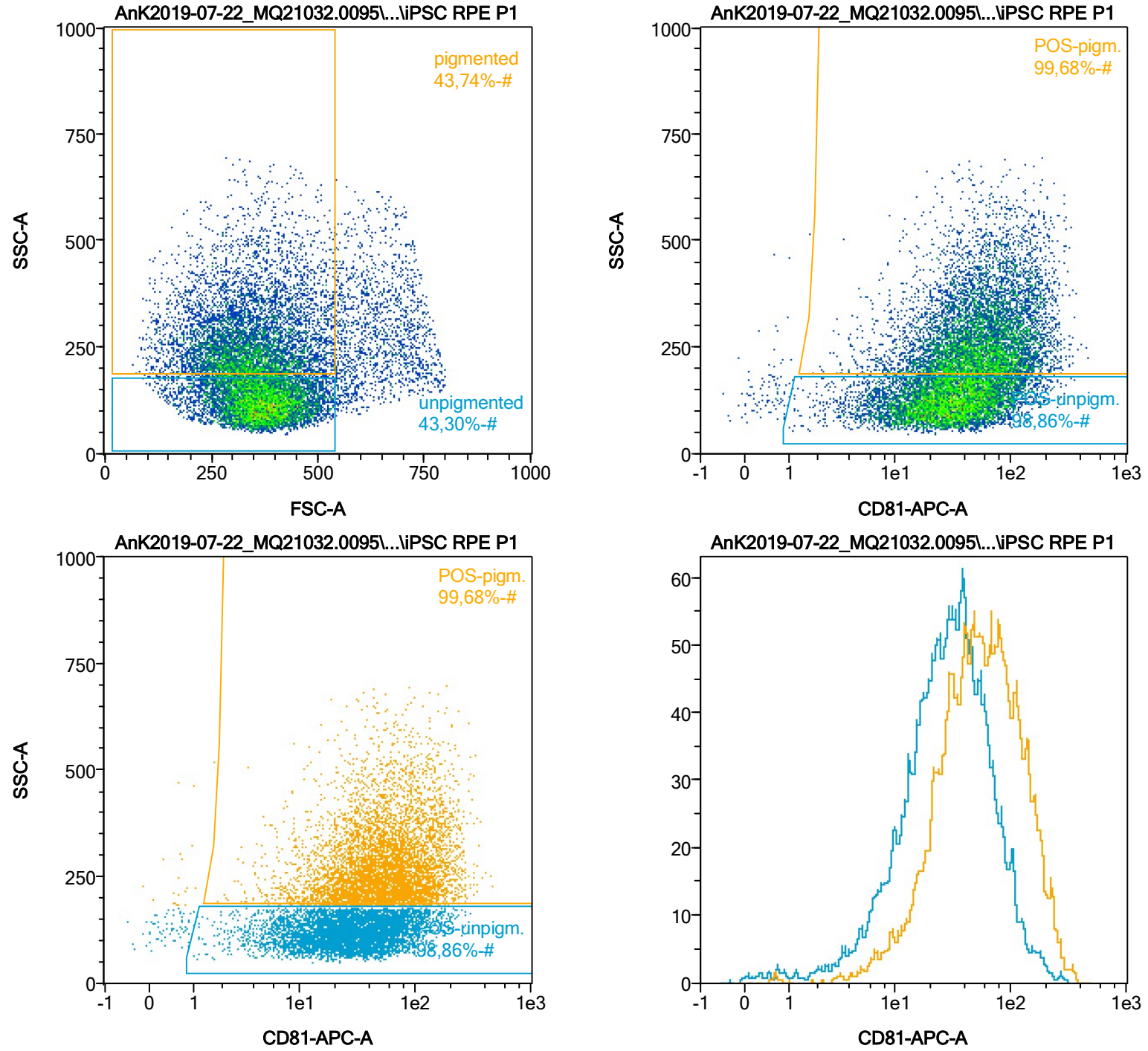

Name	%-#	CD81-APC-A Mean	CD81-APC-A Median
POS-unpigm.	98,86	37,87	28,98
POS-pigm.	99,68	70,51	54,59

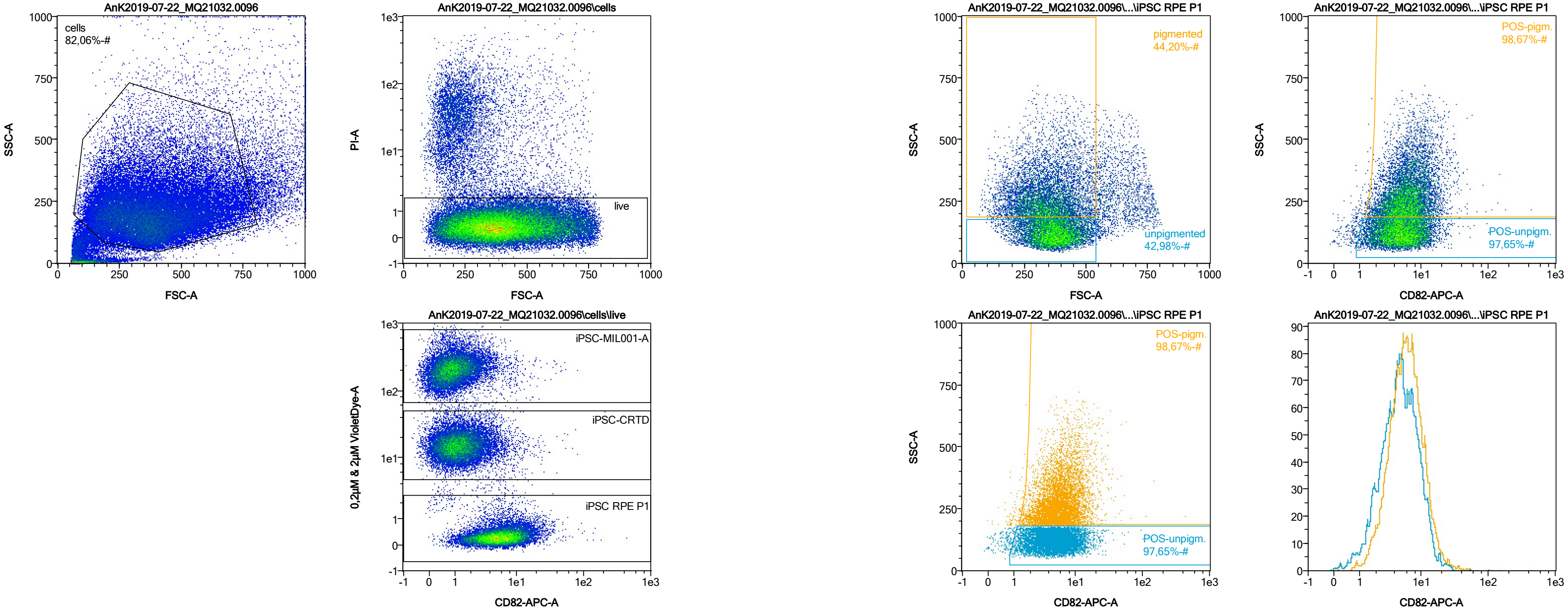

Name	%-#	CD82-APC-A Mean	CD82-APC-A Median
POS-unpigm.	97,65	5,75	4,85
POS-pigm.	98,67	6,98	5,89

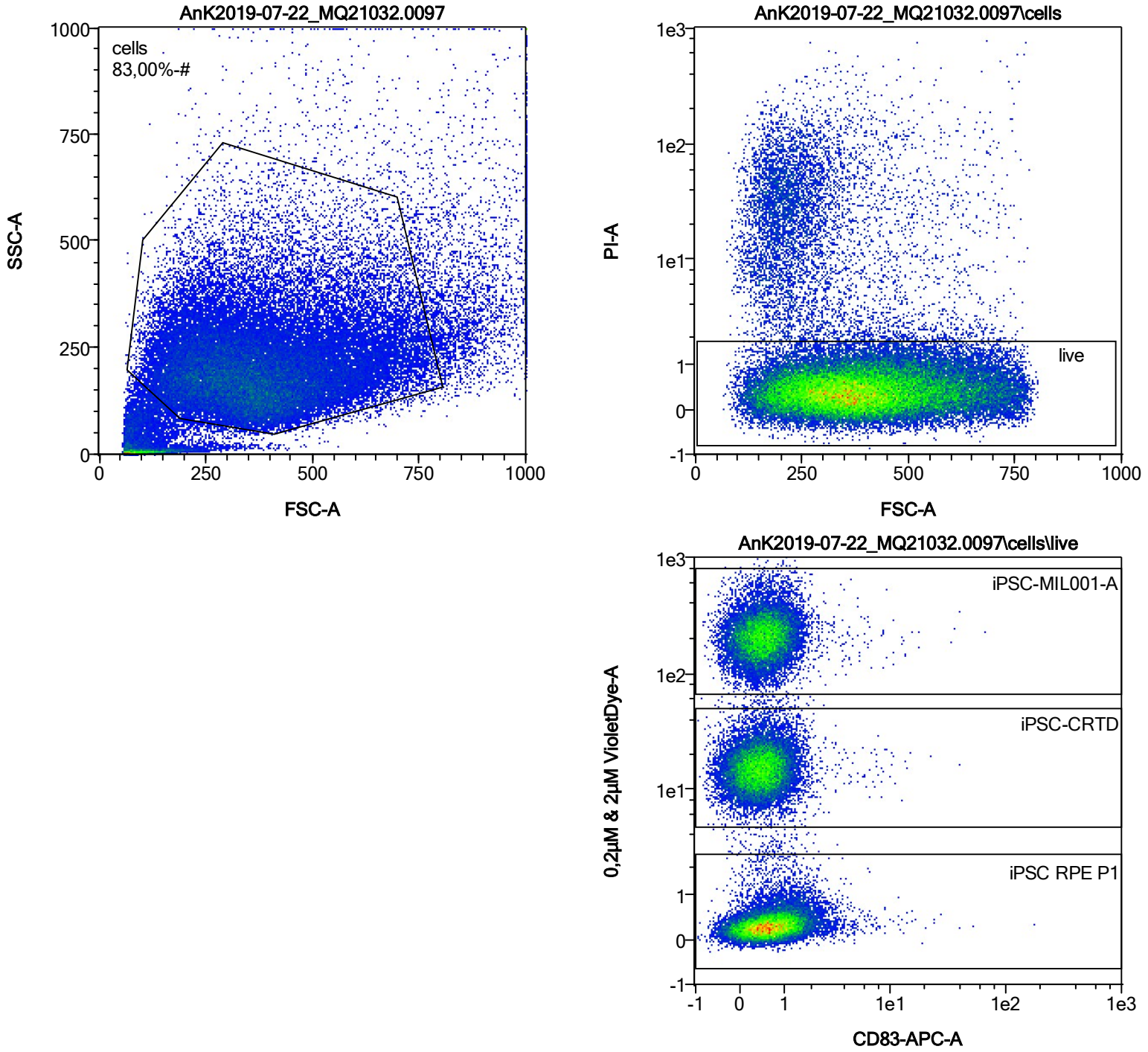

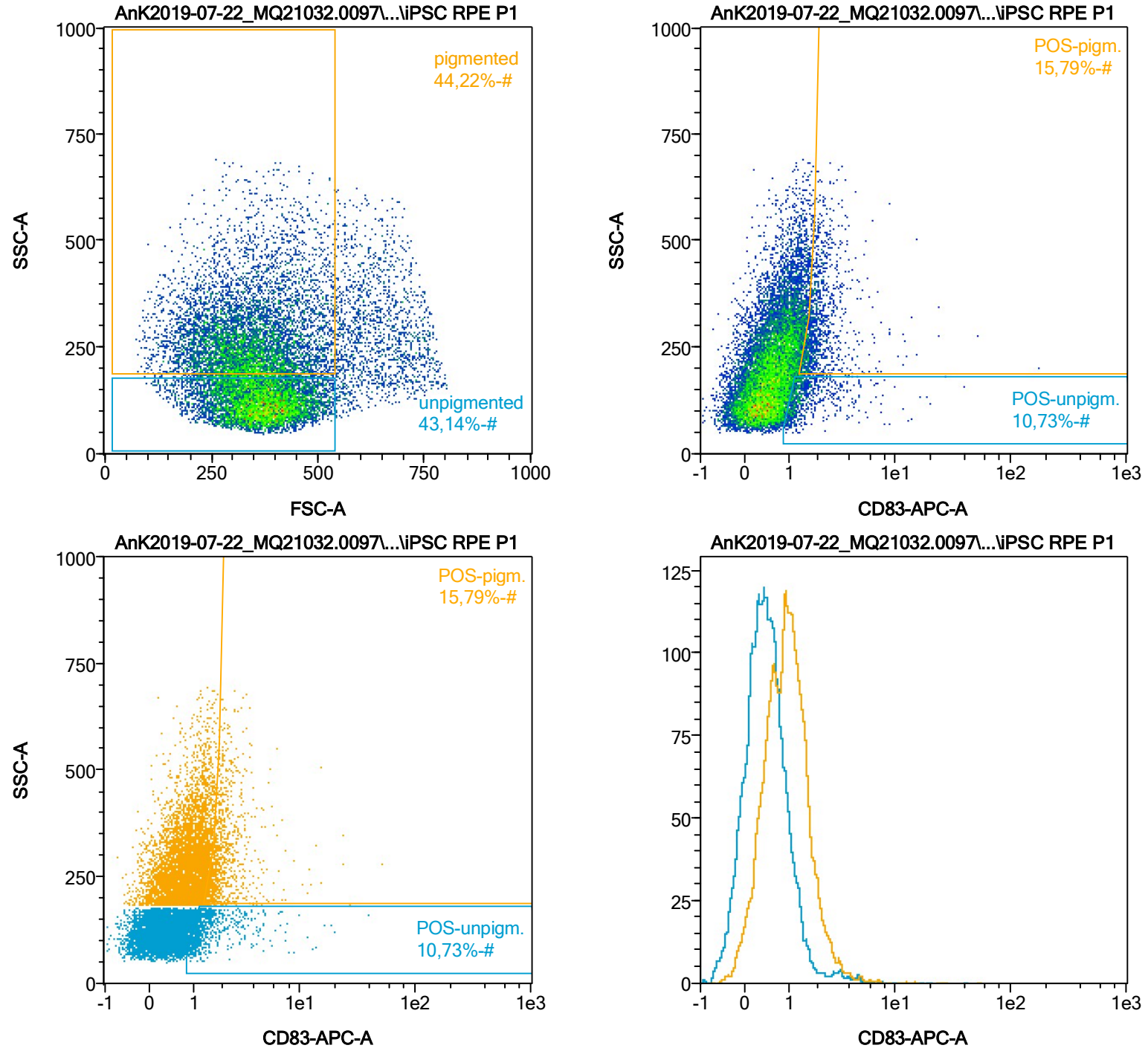

Name	%-#	CD83-APC-A Mean	CD83-APC-A Median
POS-unpigm.	10,73	1,85	1,30
POS-pigm.	15,79	2,33	1,91

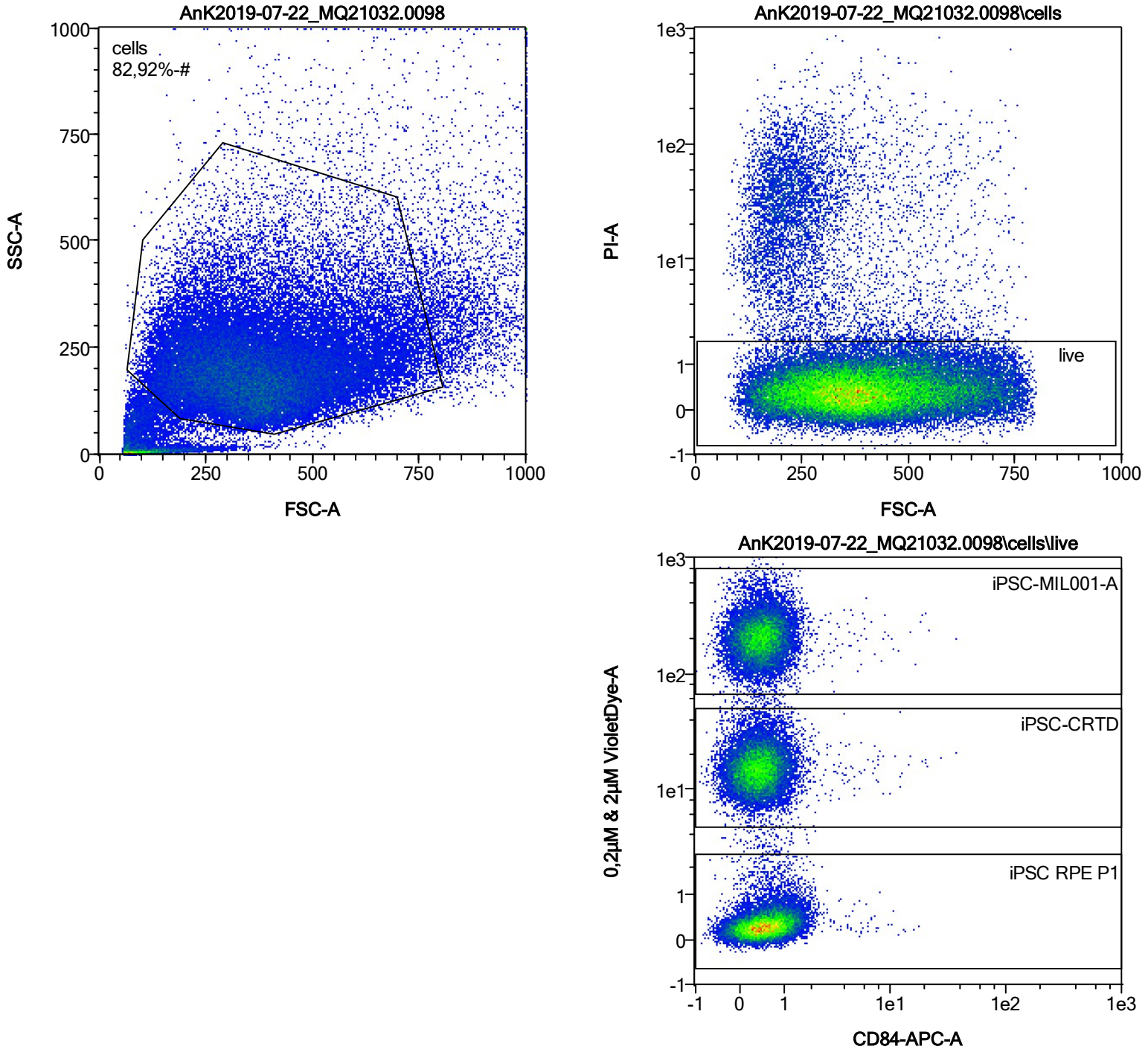

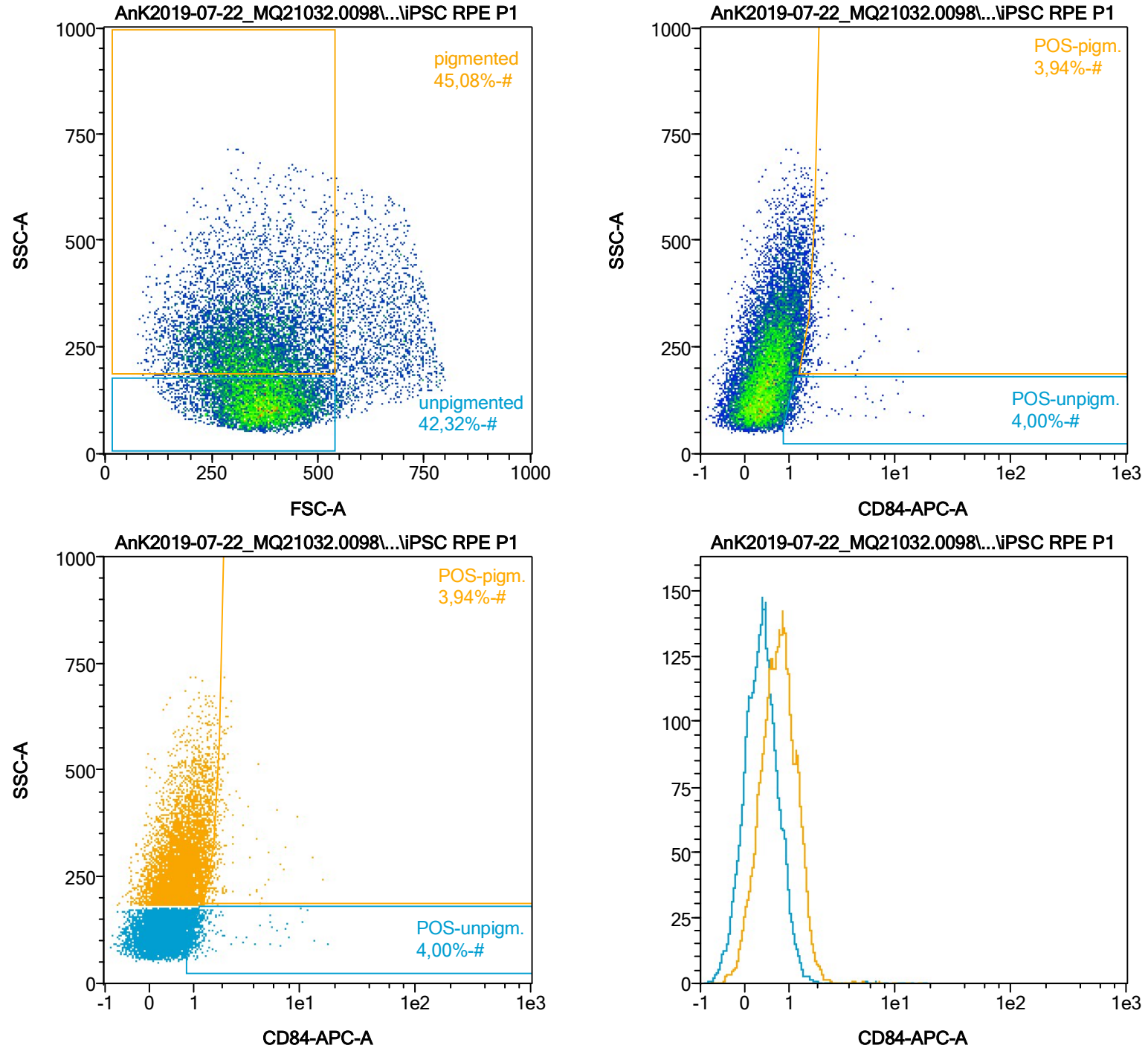

Name	%-#	CD84-APC-A Mean	CD84-APC-A Median
POS-unpigm.	4,00	1,69	1,18
POS-pigm.	3,94	2,11	1,79

Date:2024-06-14
Operator: Sebastian Knoebel
Version:2.13.0+54.g95abf0f4

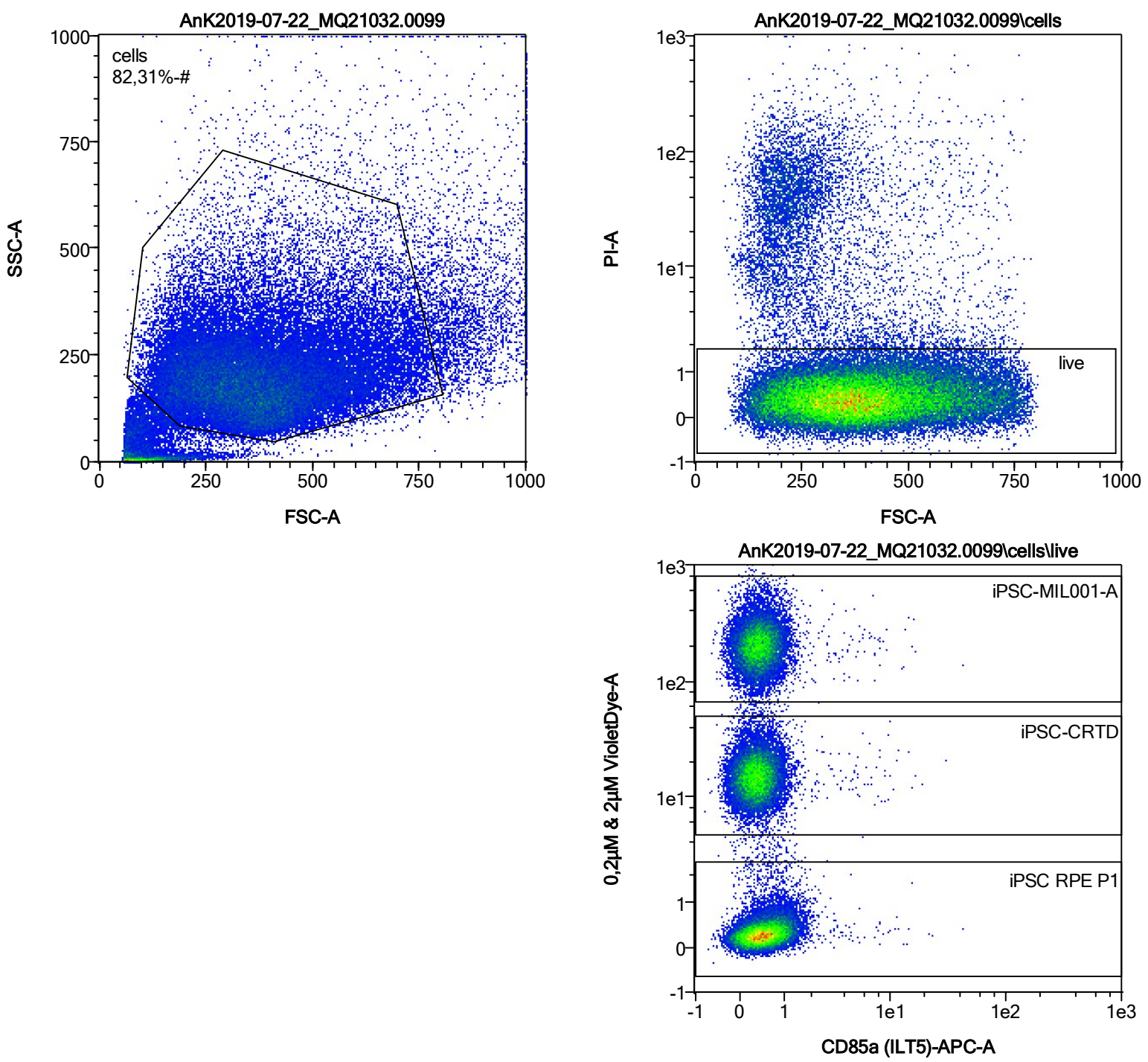

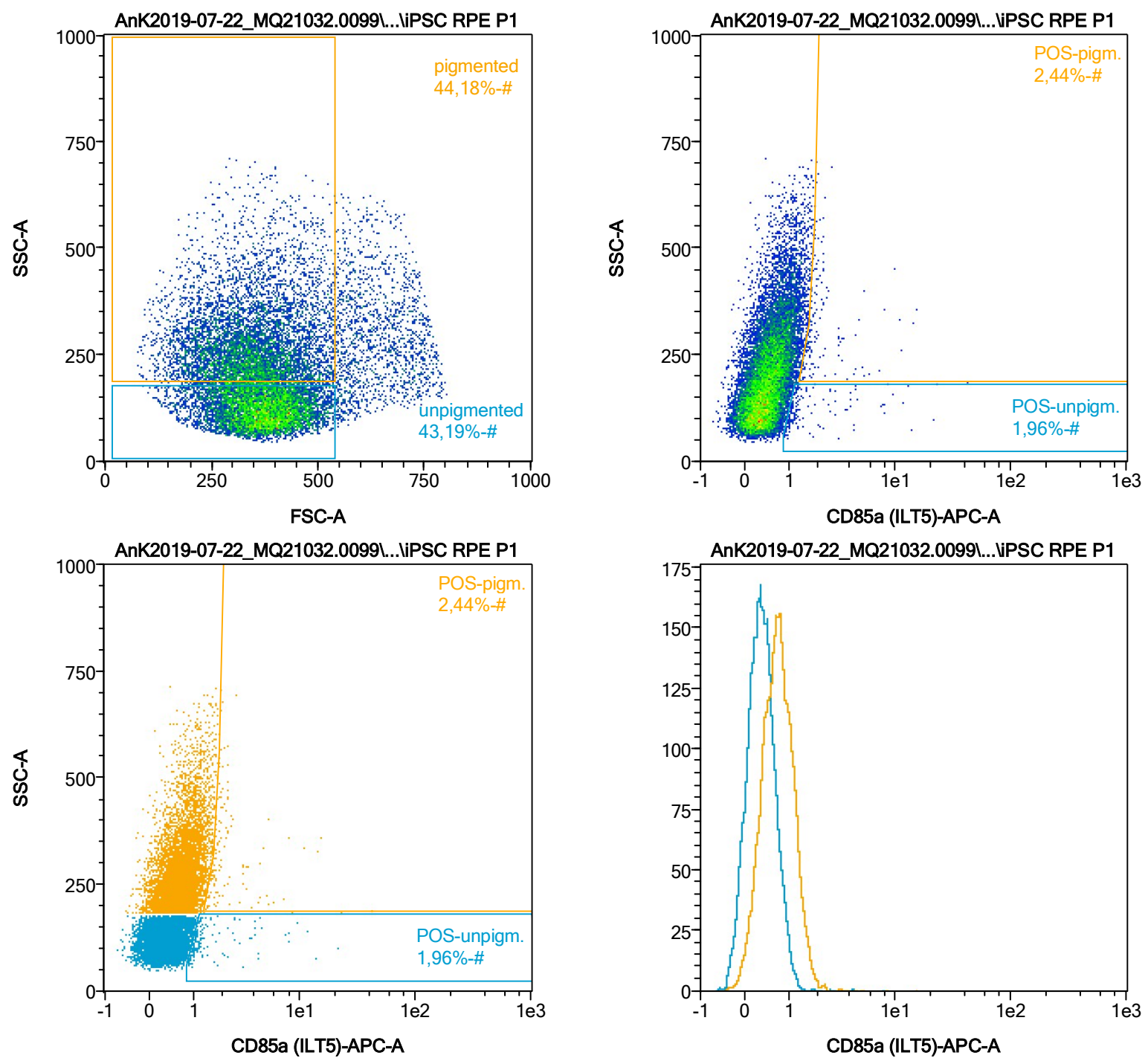

Name	%-#	CD85a (ILT5)-APC-A Mean	CD85a (ILT5)-APC-A Median
POS-unpigm.	1,96	2,05	1,19
POS-pigm.	2,44	2,75	1,83

Date:2024-06-14

Operator: Sebastian Knoebel

Version:2.13.0+54.g95abf0f4

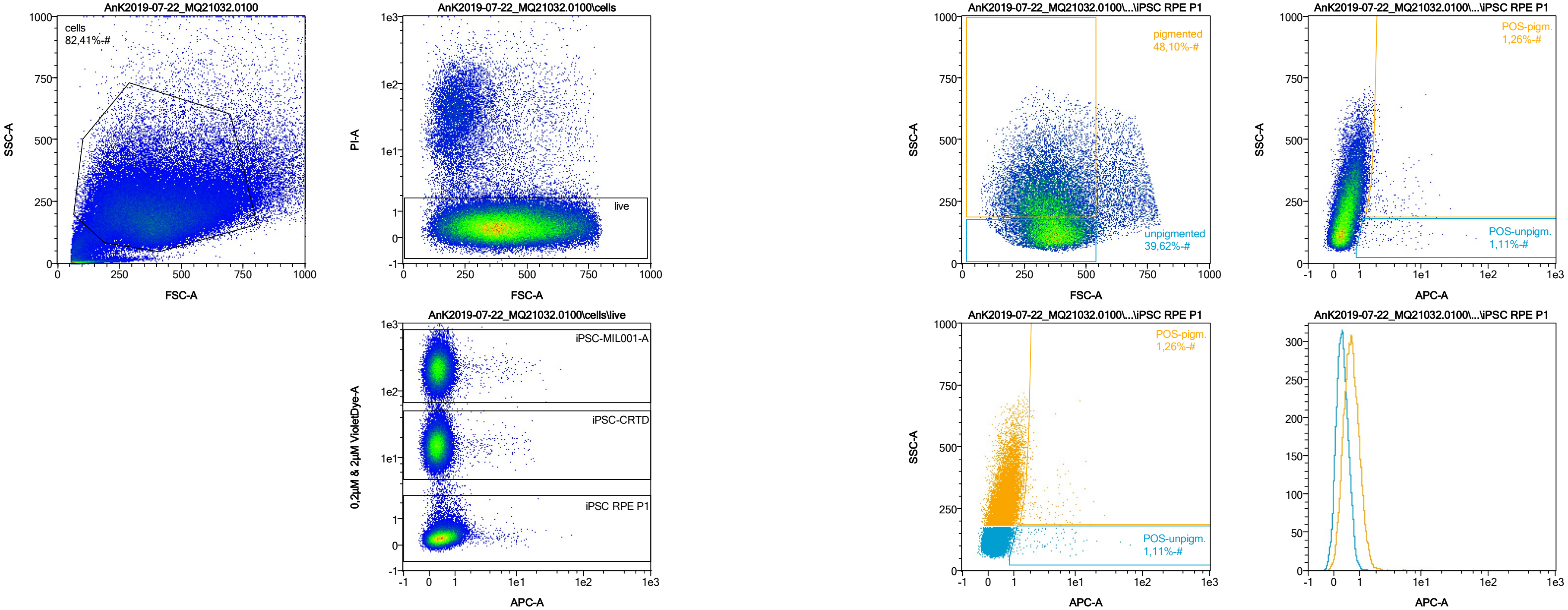

Name	%-#	APC-A Mean	APC-A Median
POS-unpigm.	1,11	4,61	2,37
POS-pigm.	1,26	3,83	2,24

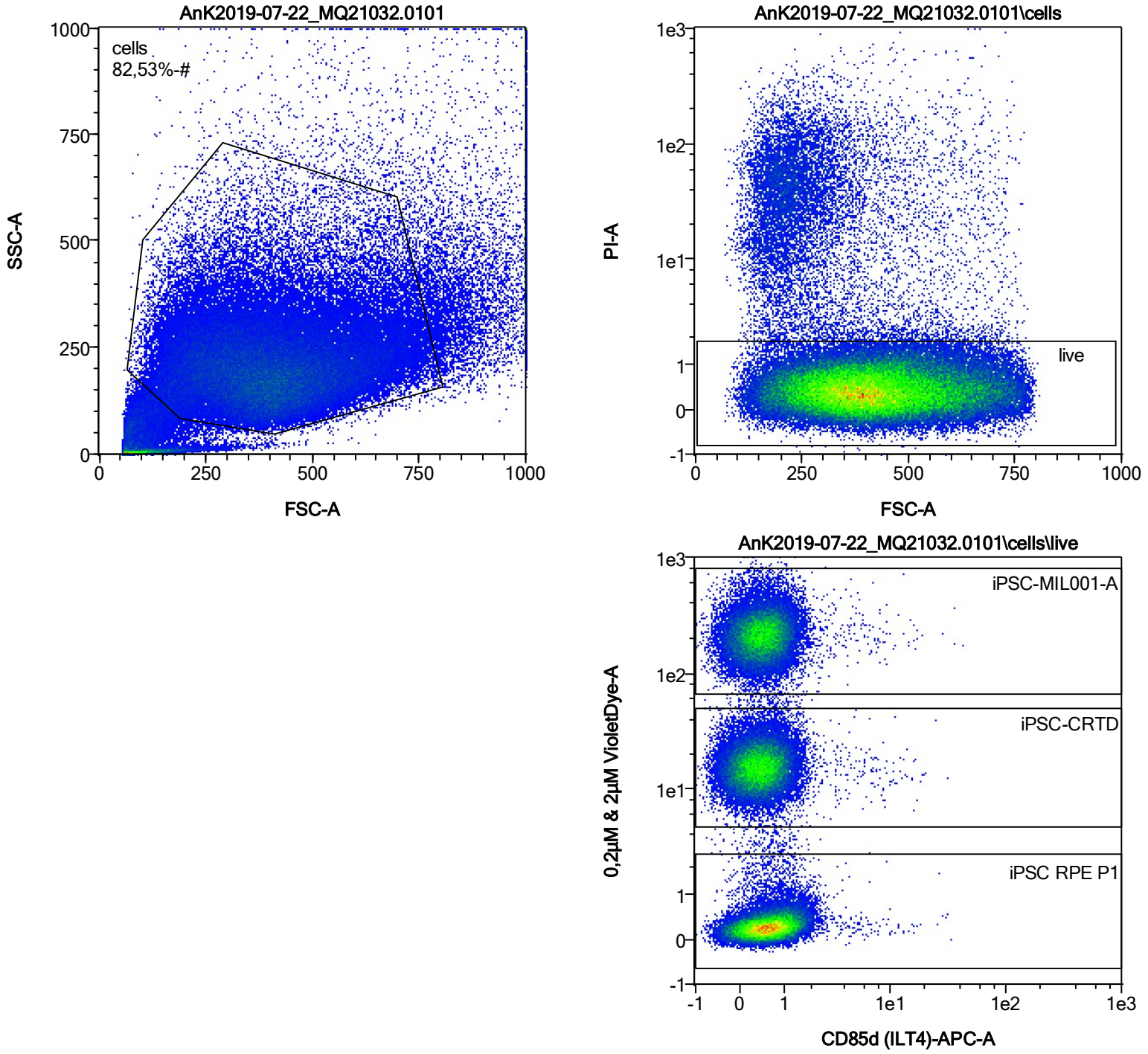

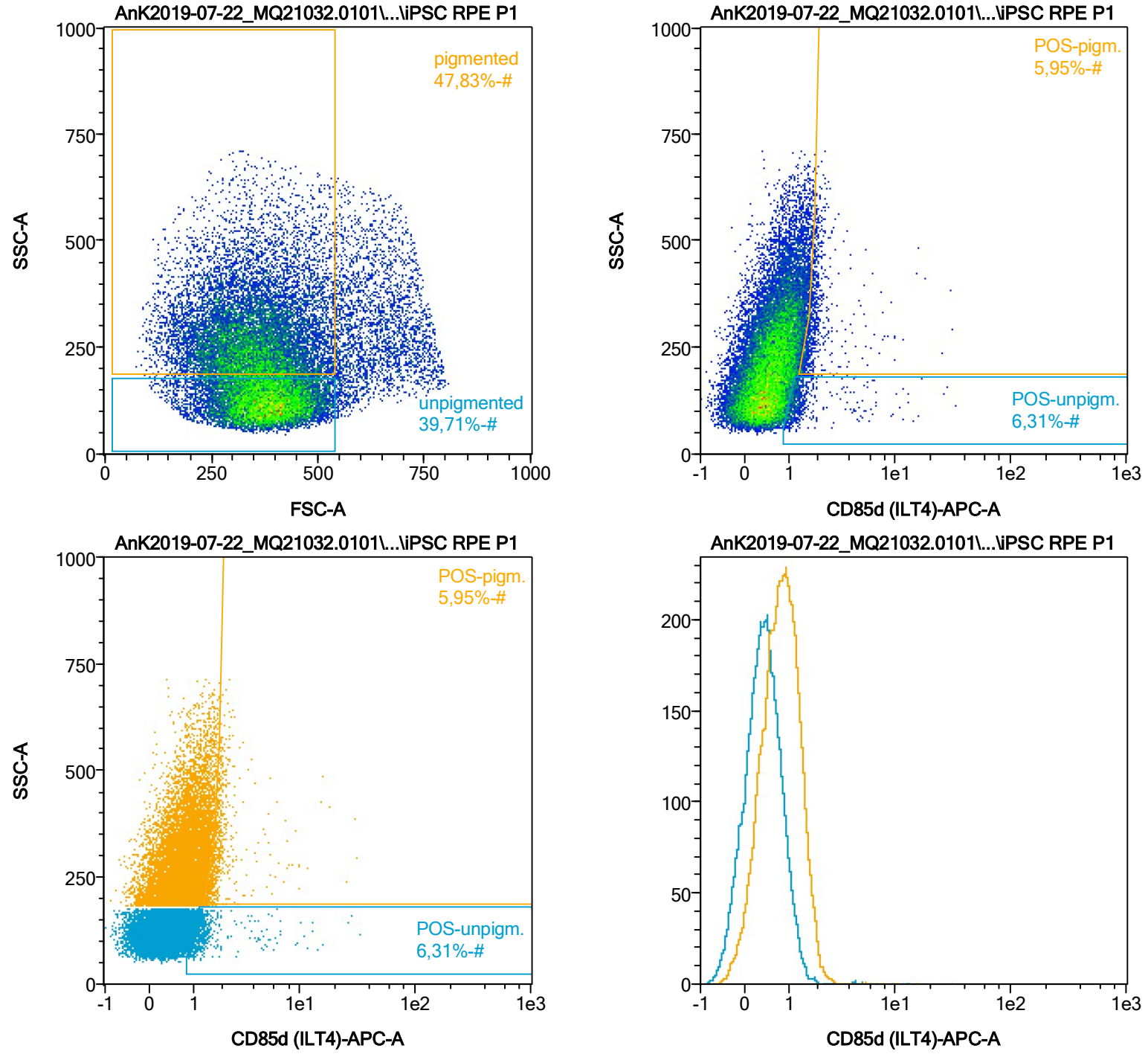

Name	%-#	CD85d (ILT4)-APC-A Mean	CD85d (ILT4)-APC-A Median
POS-unpigm.	6,31	1,70	1,20
POS-pigm.	5,95	2,18	1,81

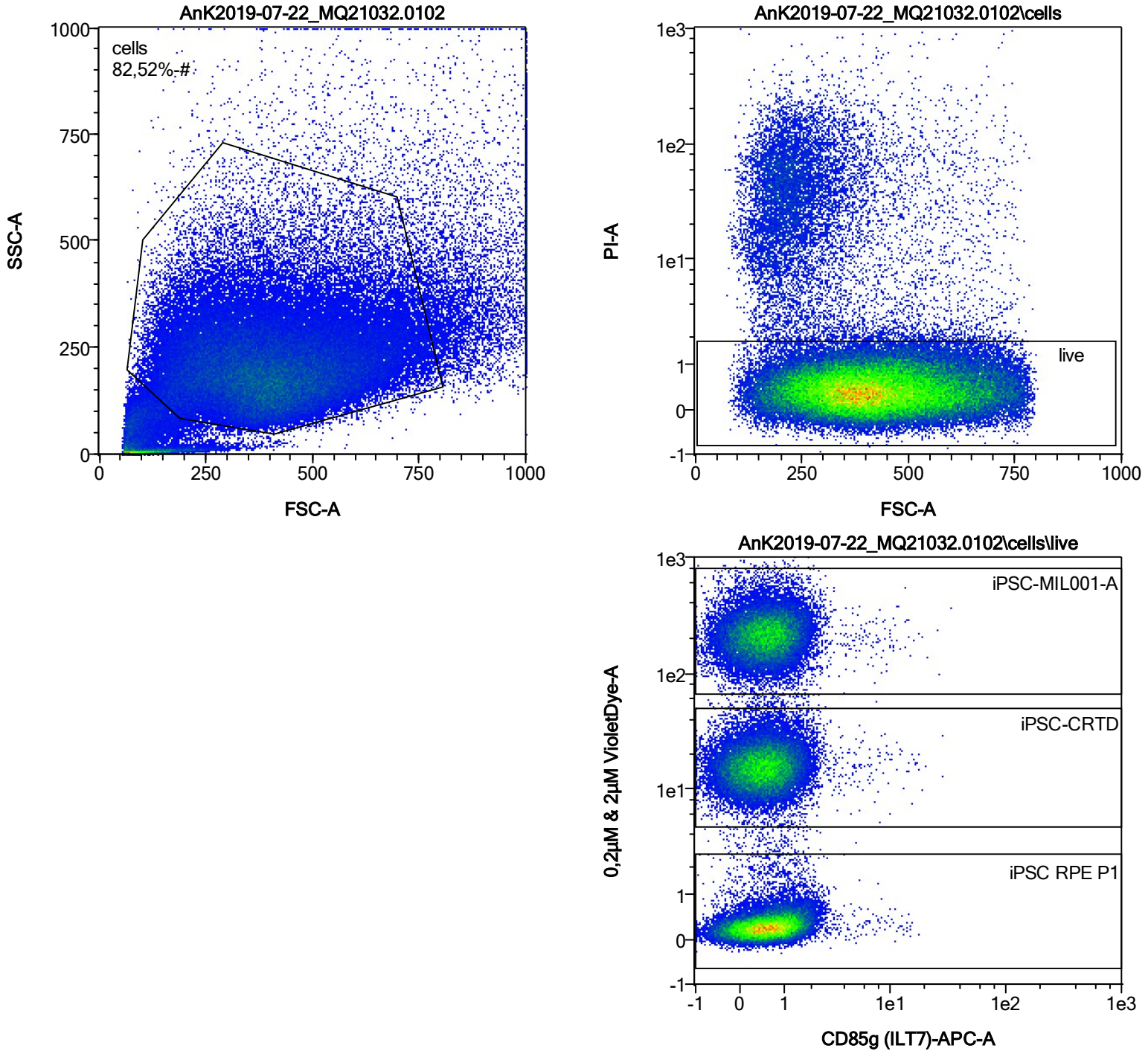

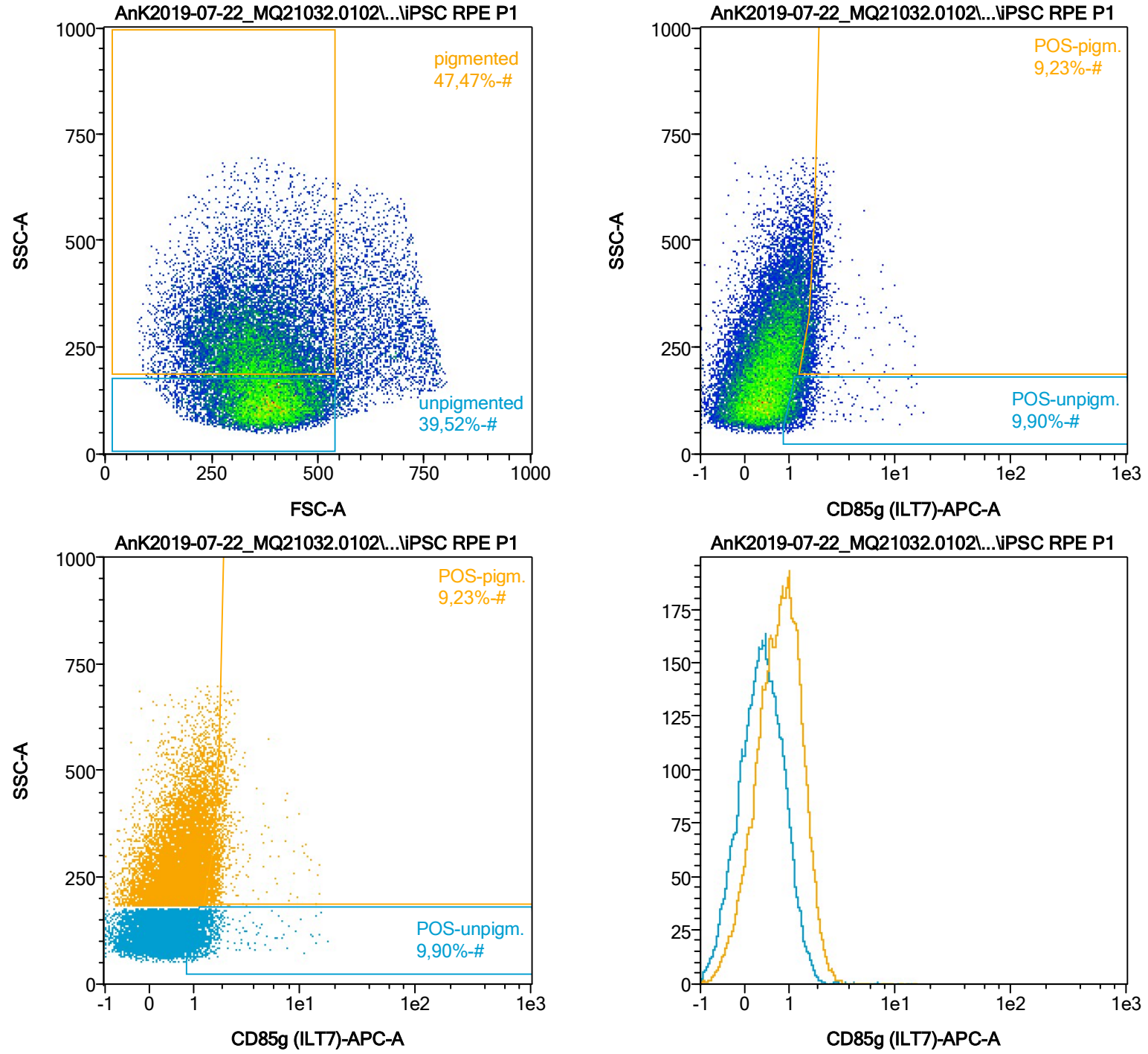

Name	%-#	CD85g (ILT7)-APC-A Mean	CD85g (ILT7)-APC-A Median
POS-unpigm.	9,90	1,52	1,23
POS-pigm.	9,23	2,08	1,83

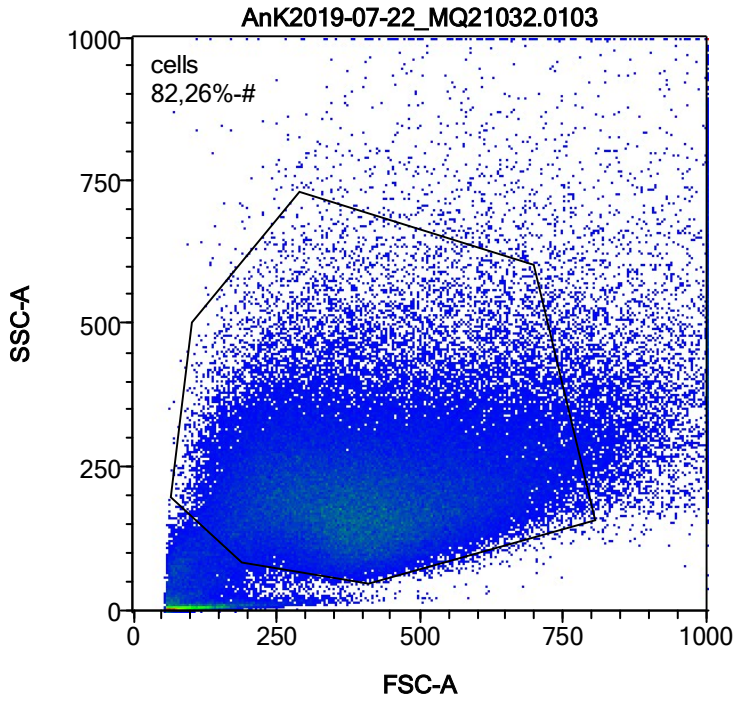

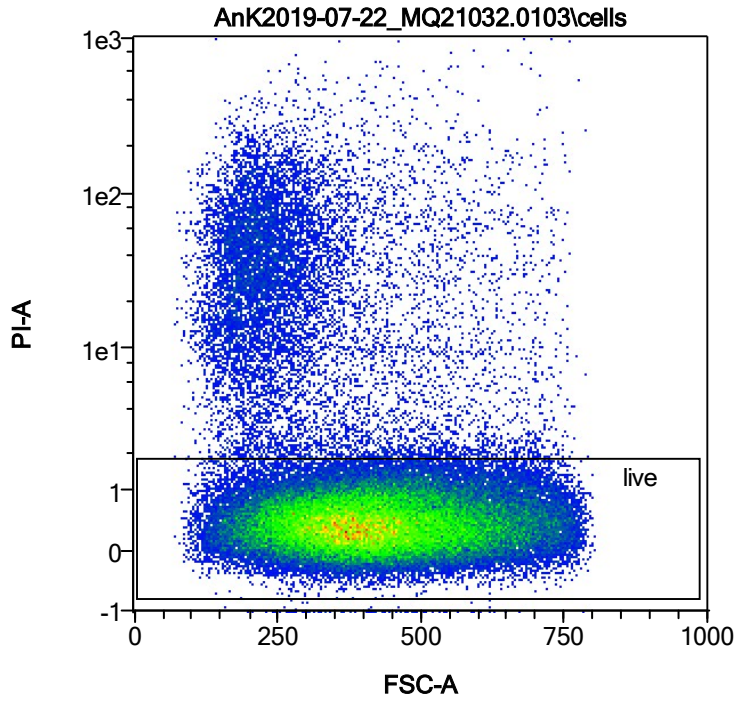

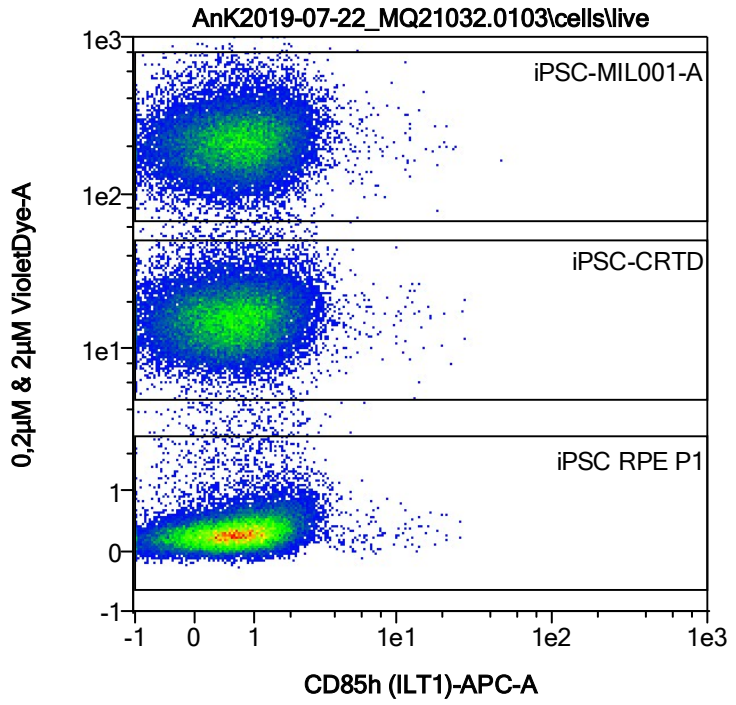

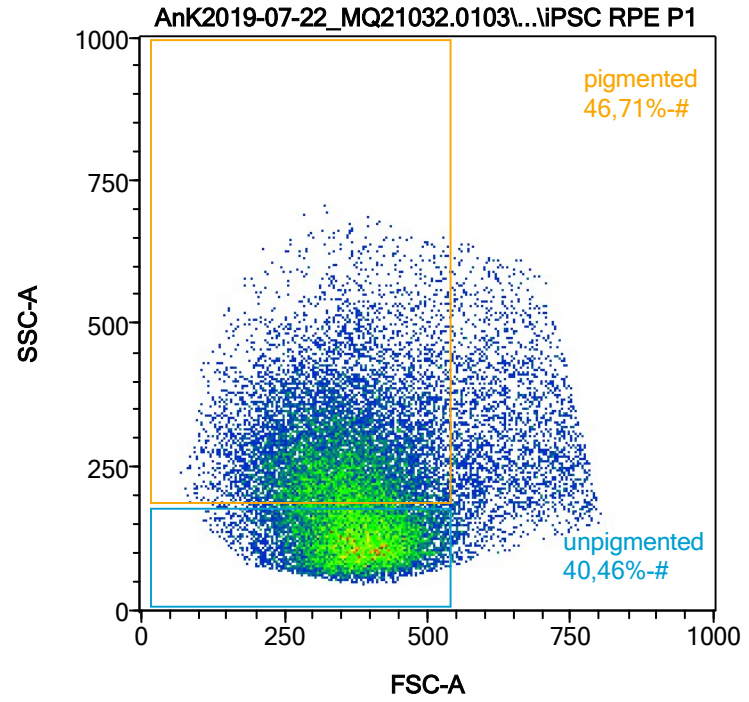

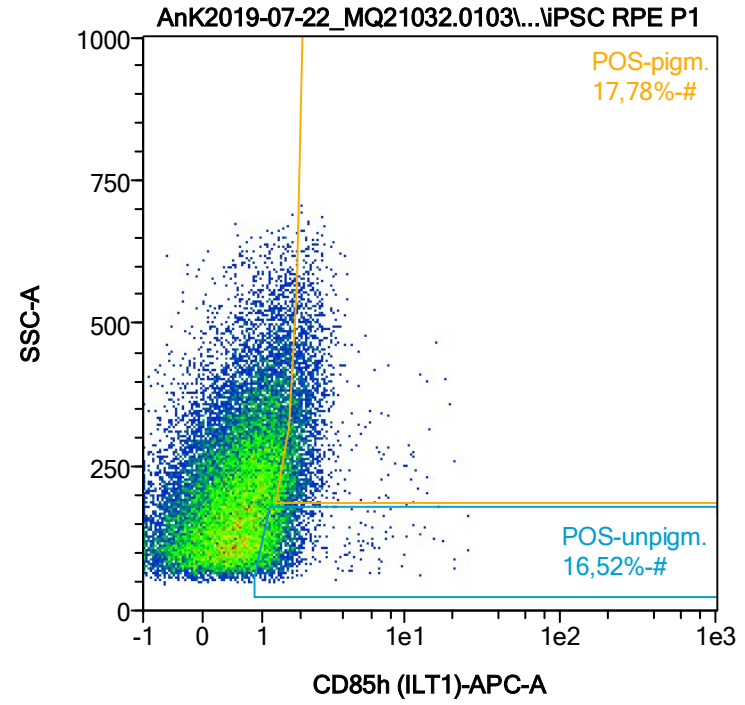

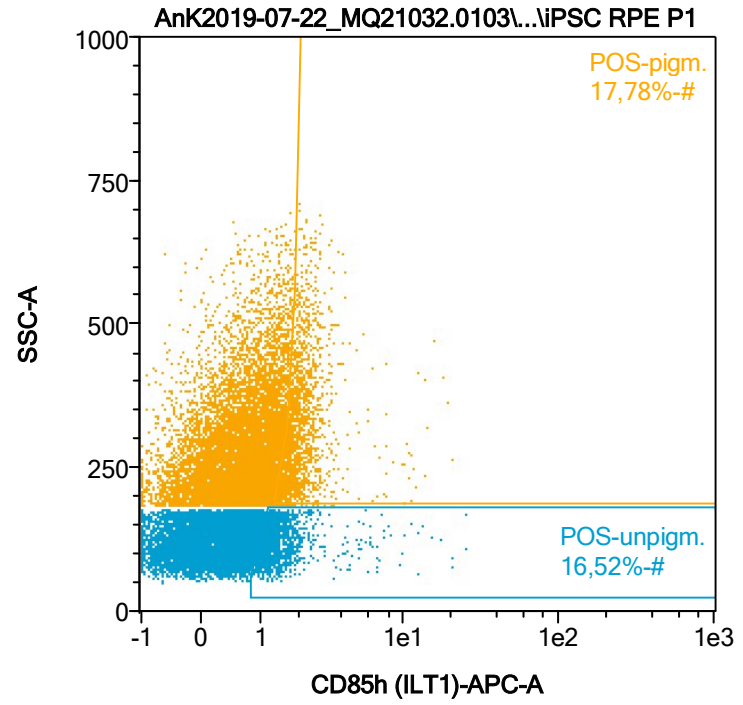

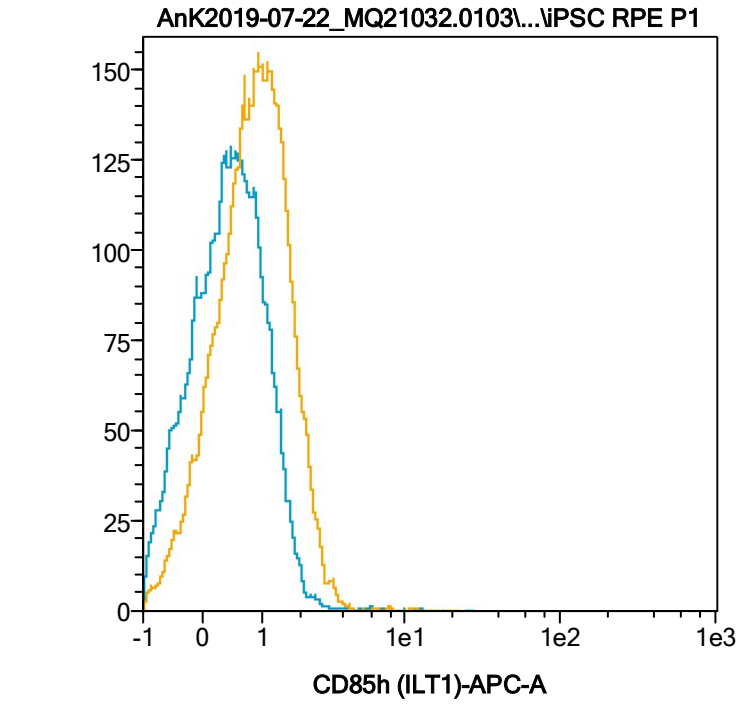

Name	%-#	CD85h (ILT1)-APC-A Mean	CD85h (ILT1)-APC-A Median
POS-unpigm.	16,52	1,60	1,30
POS-pigm.	17,78	2,15	1,90

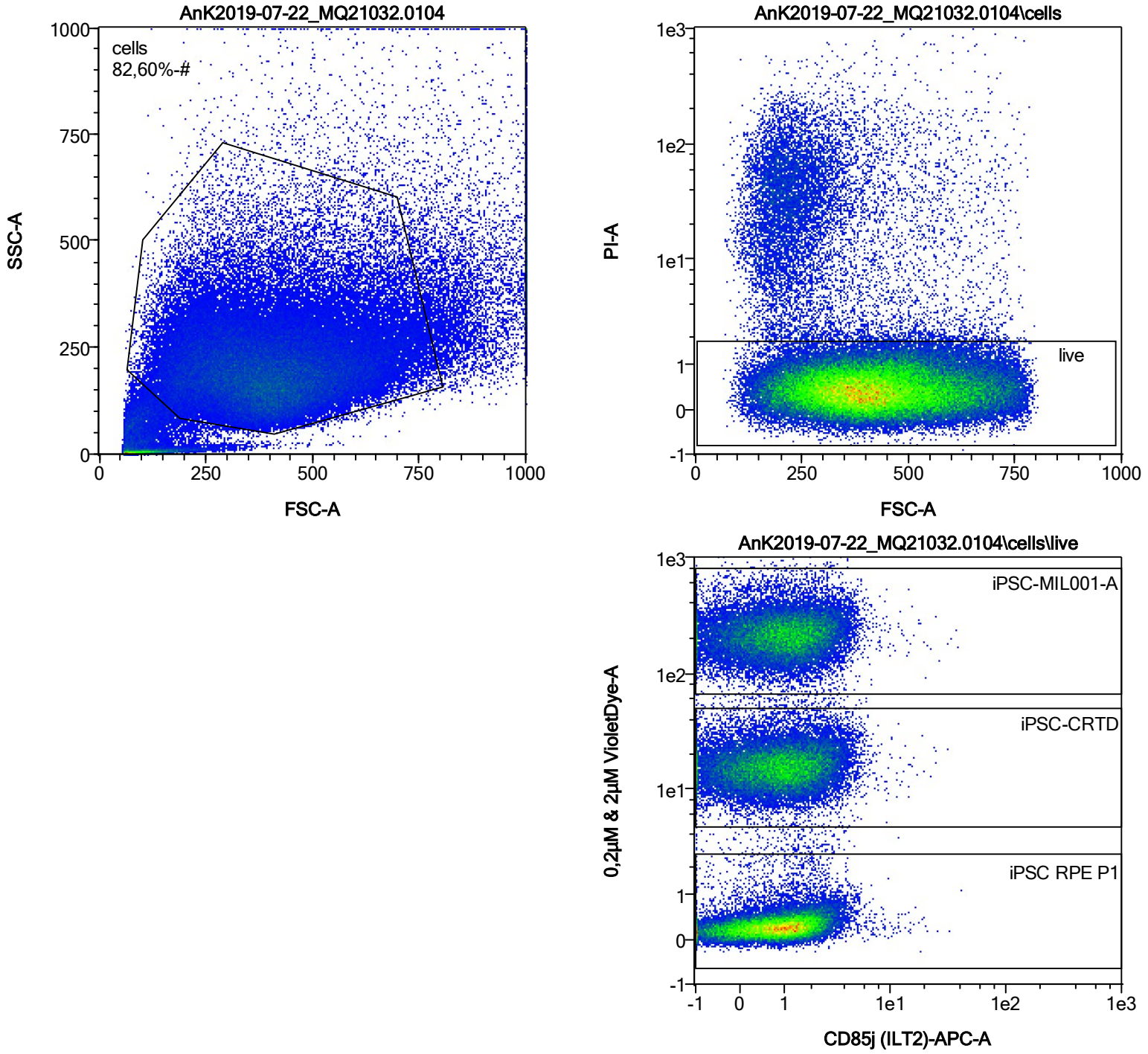

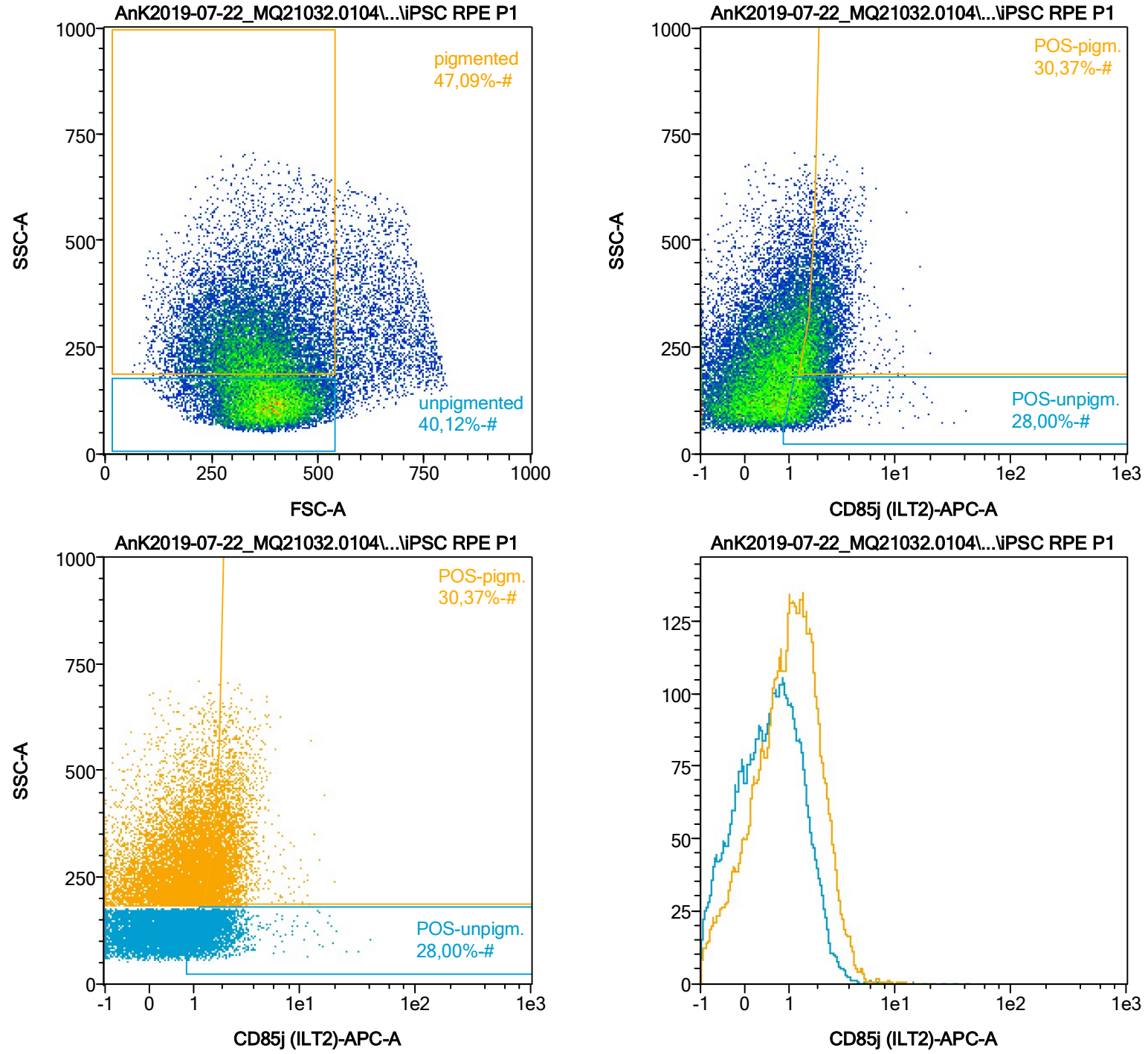

Name	%-#	CD85j (ILT2)-APC-A Mean	CD85j (ILT2)-APC-A Median
POS-unpigm.	28,00	1,76	1,47
POS-pigm.	30,37	2,34	2,07

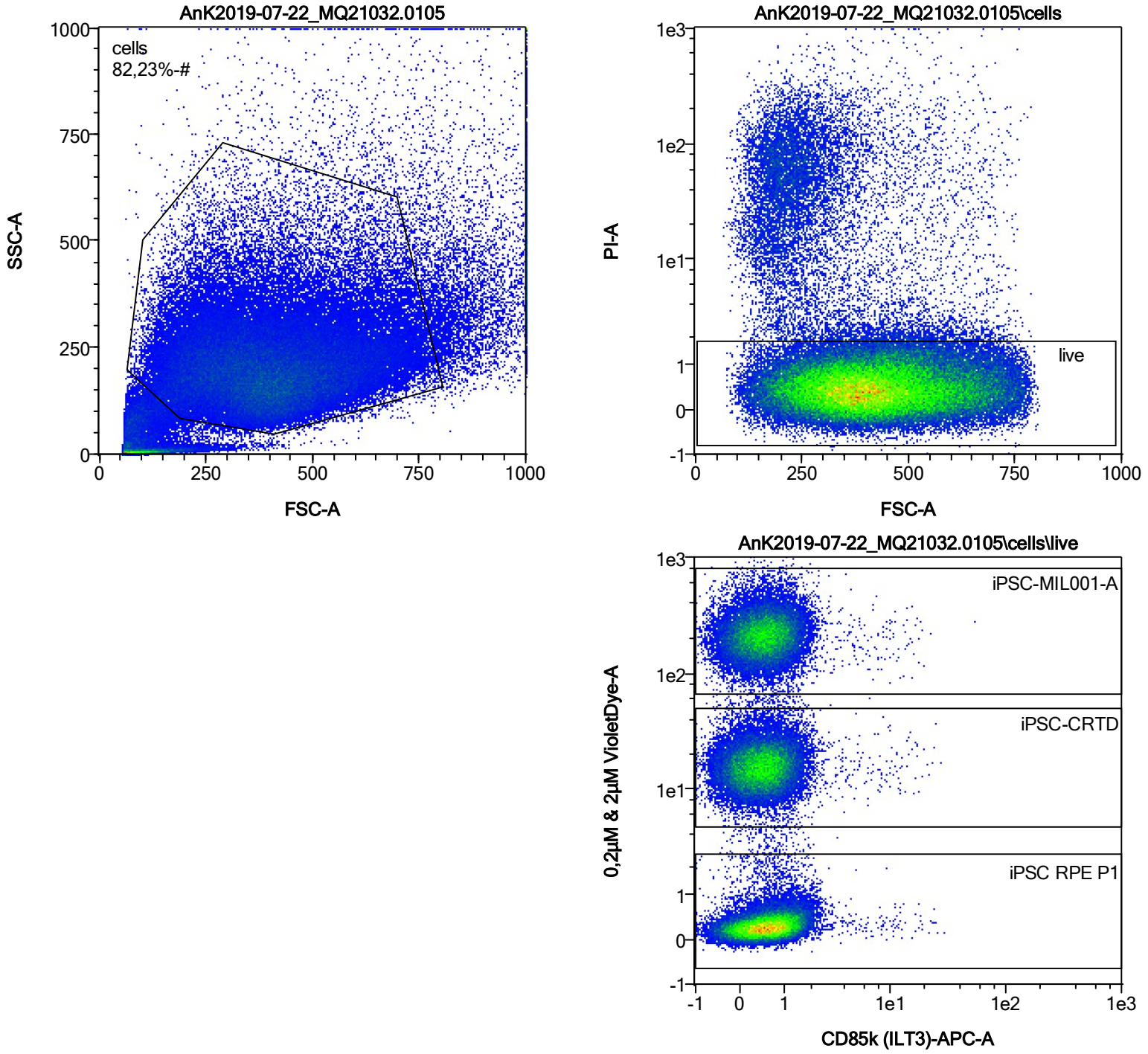

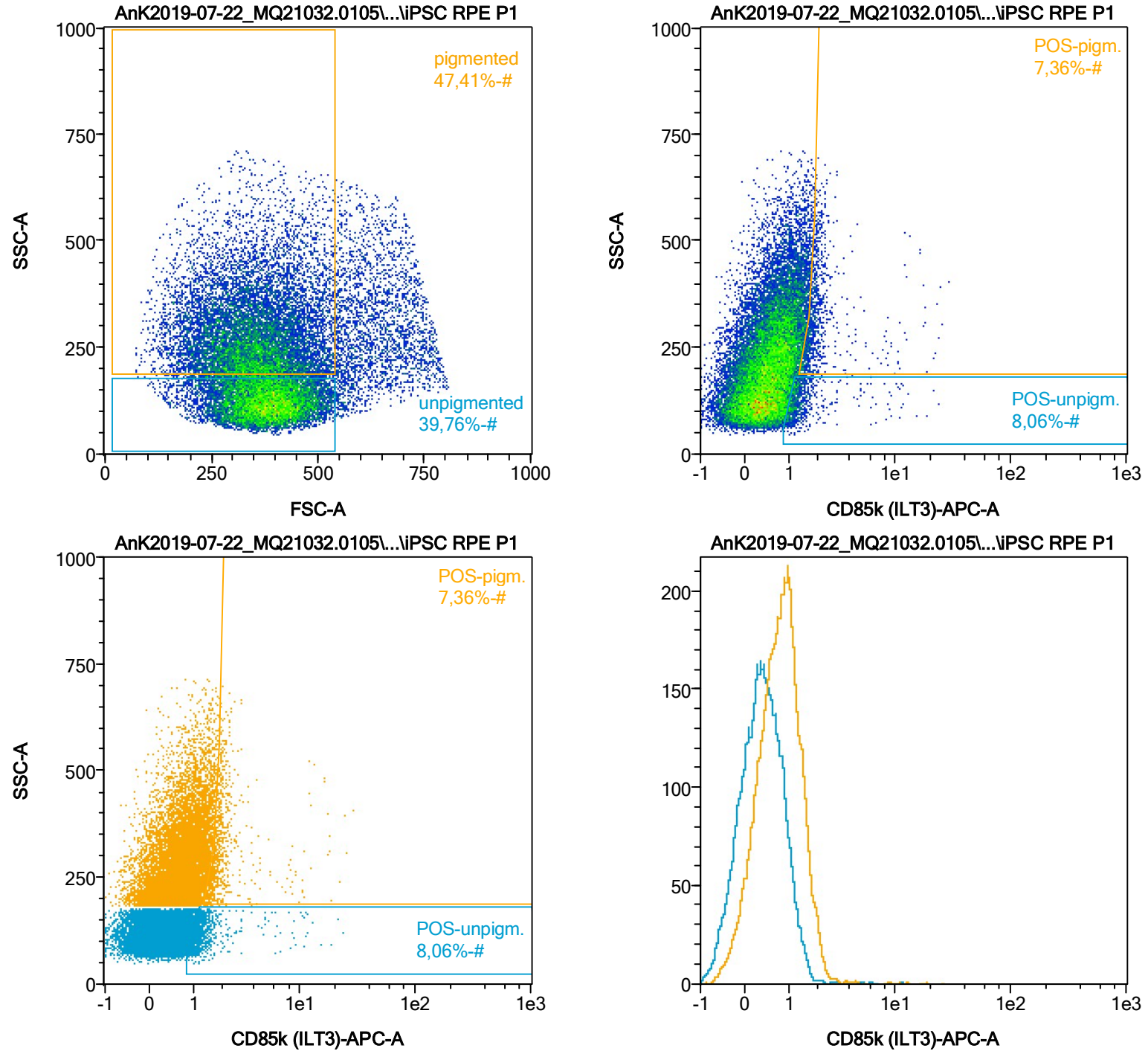

Name	%-#	CD85k (ILT3)-APC-A Mean	CD85k (ILT3)-APC-A Median
POS-unpigm.	8,06	1,63	1,21
POS-pigm.	7,36	2,37	1,85

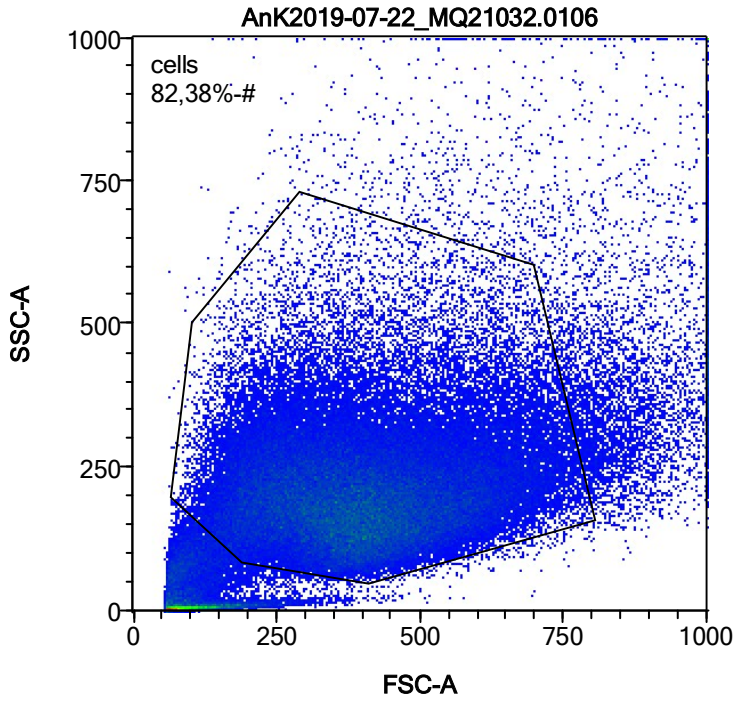

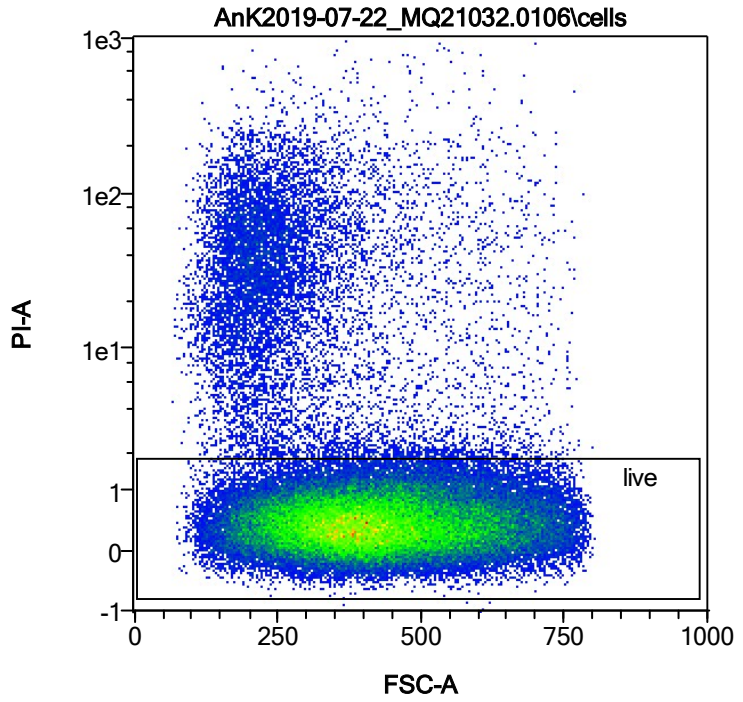

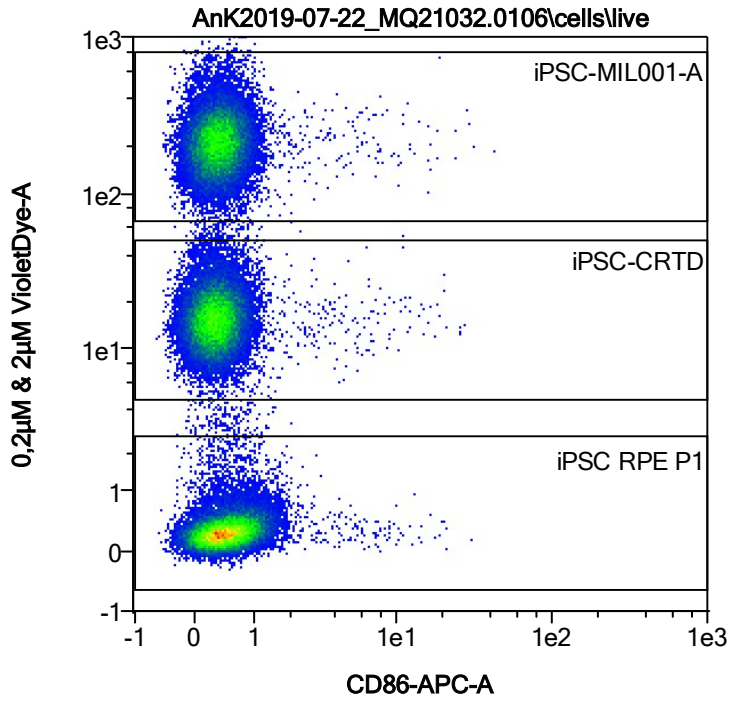

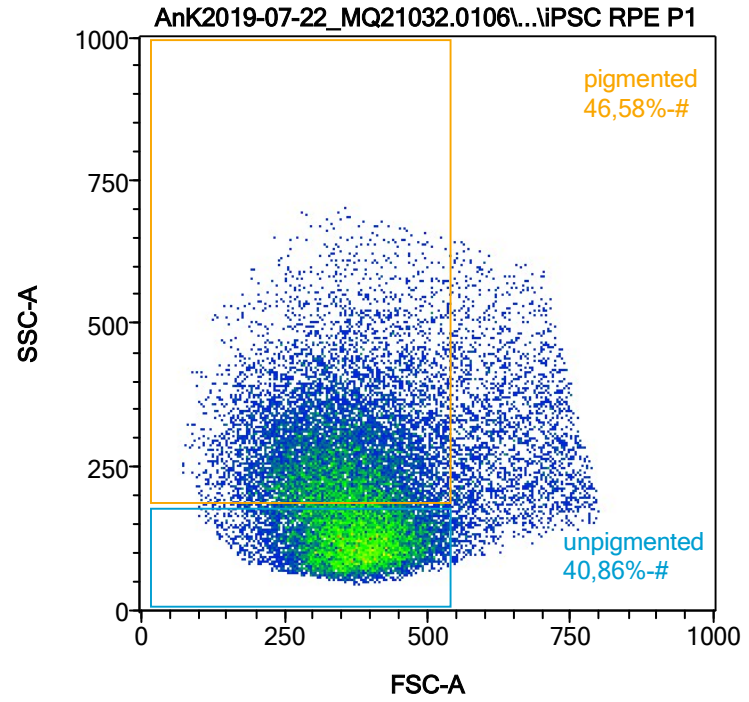

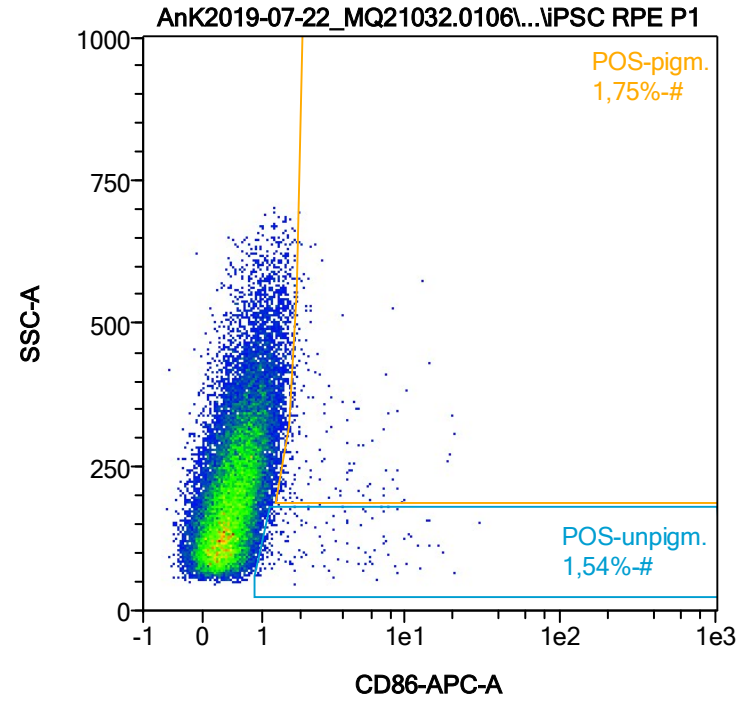

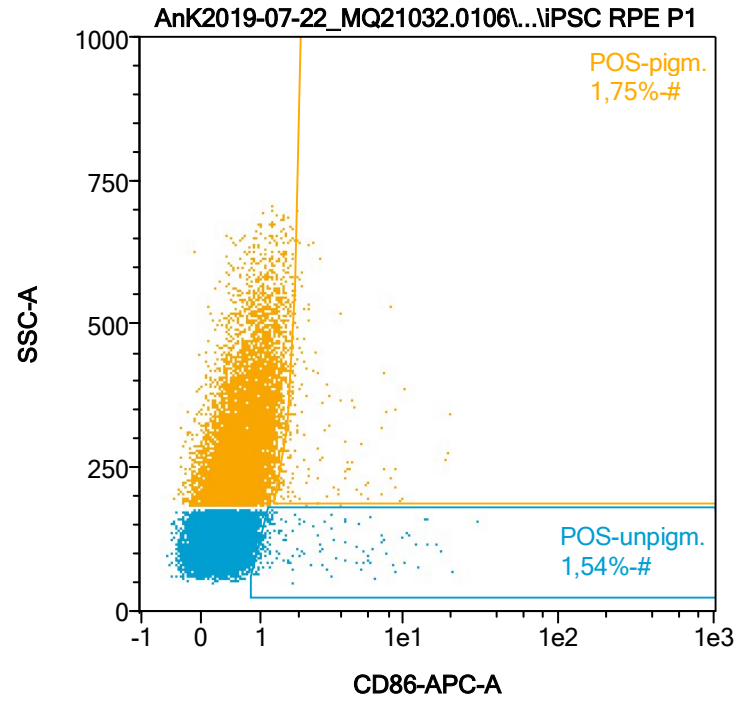

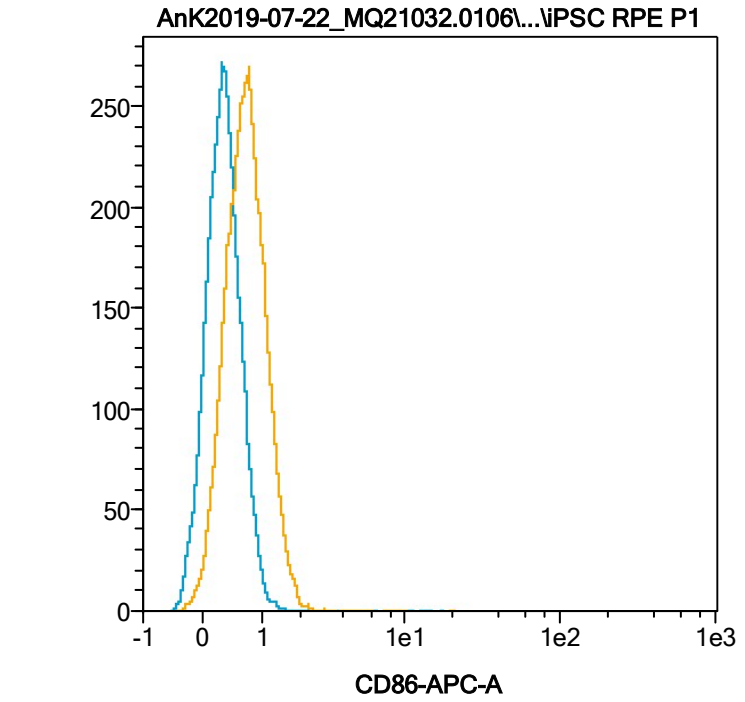

Name	%-#	CD86-APC-A Mean	CD86-APC-A Median
POS-unpigm.	1,54	3,28	1,36
POS-pigm.	1,75	2,88	1,87

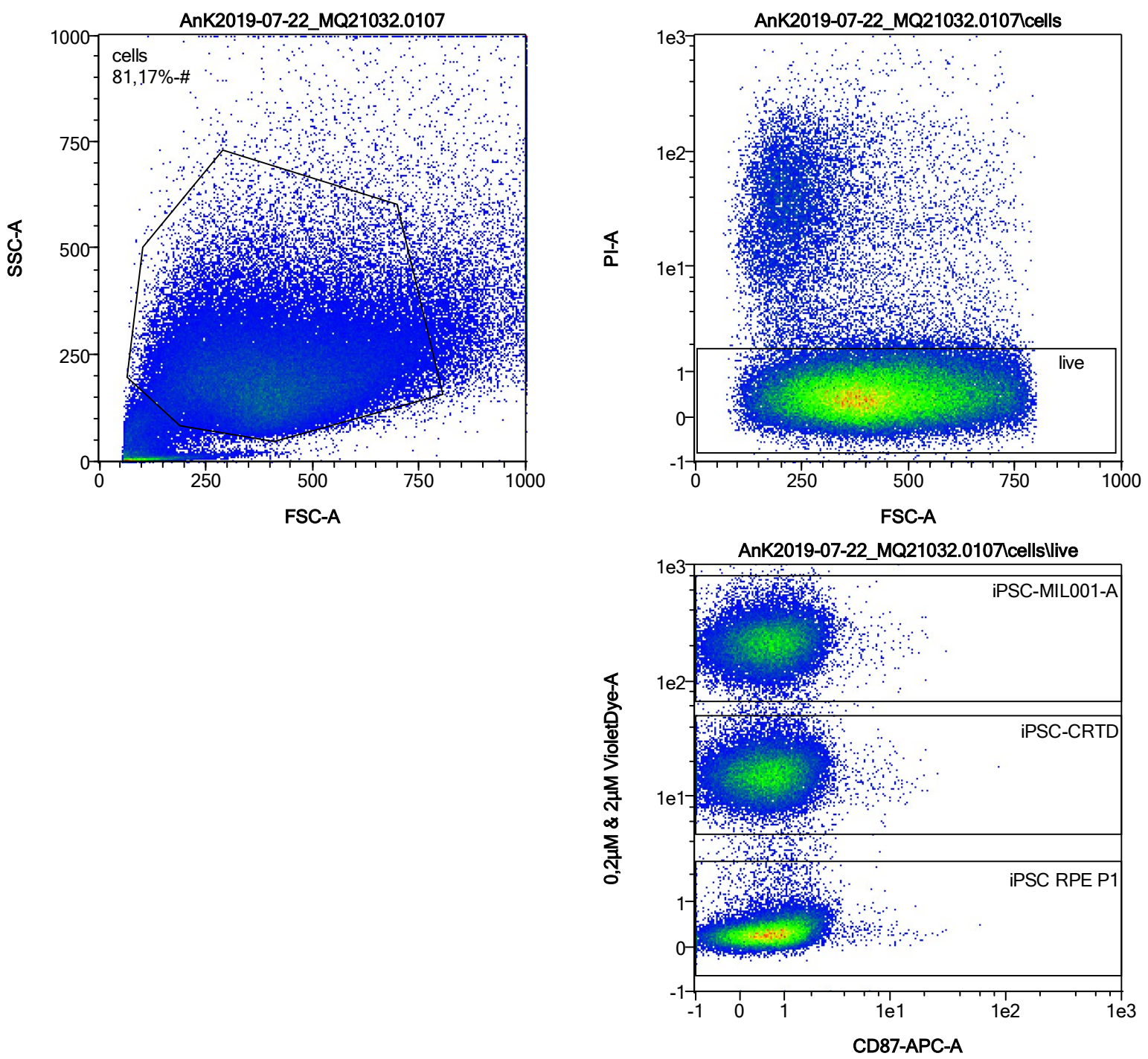

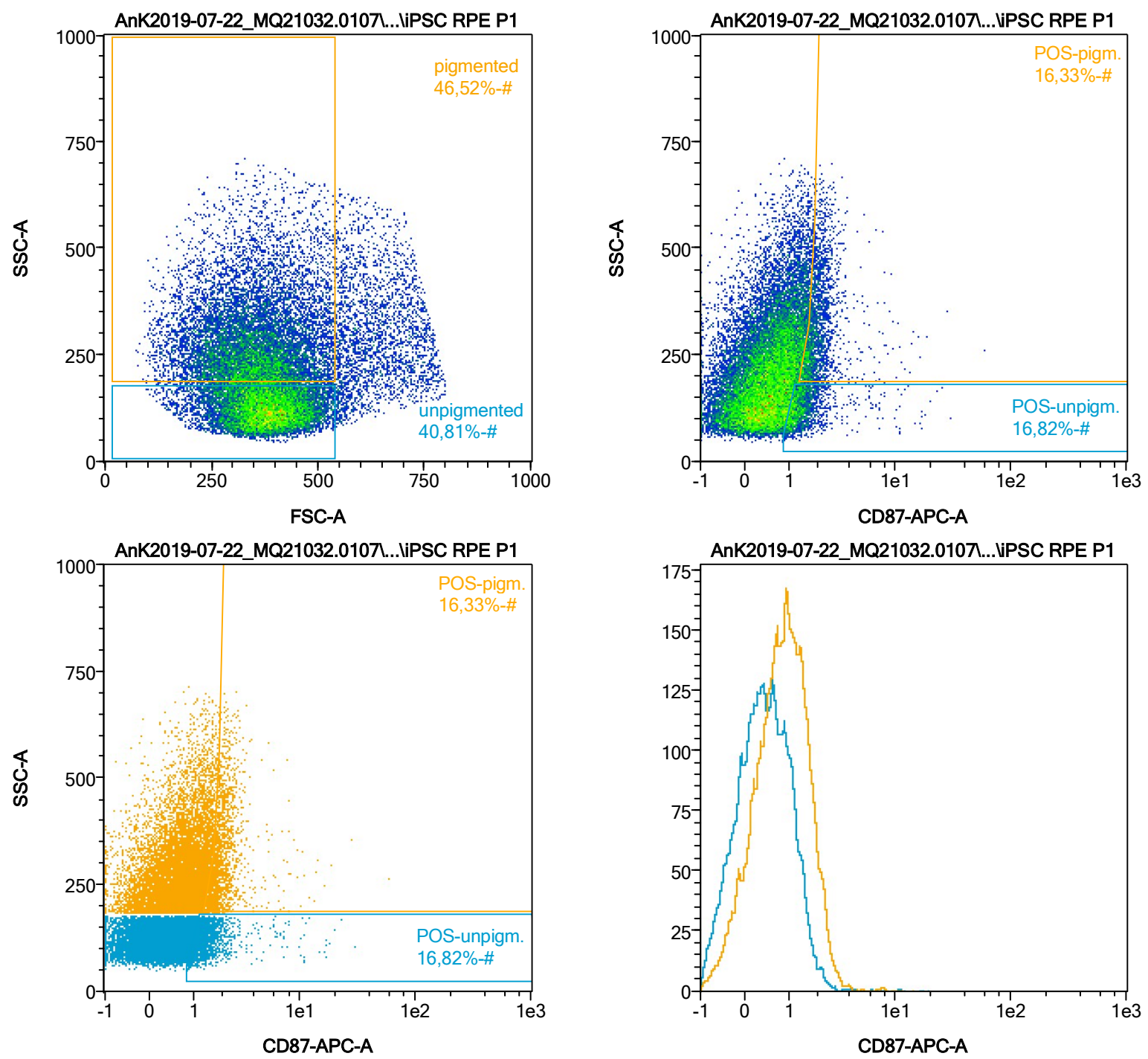

Name	%-#	CD87-APC-A Mean	CD87-APC-A Median
POS-unpigm.	16,82	1,62	1,30
POS-pigm.	16,33	2,16	1,88

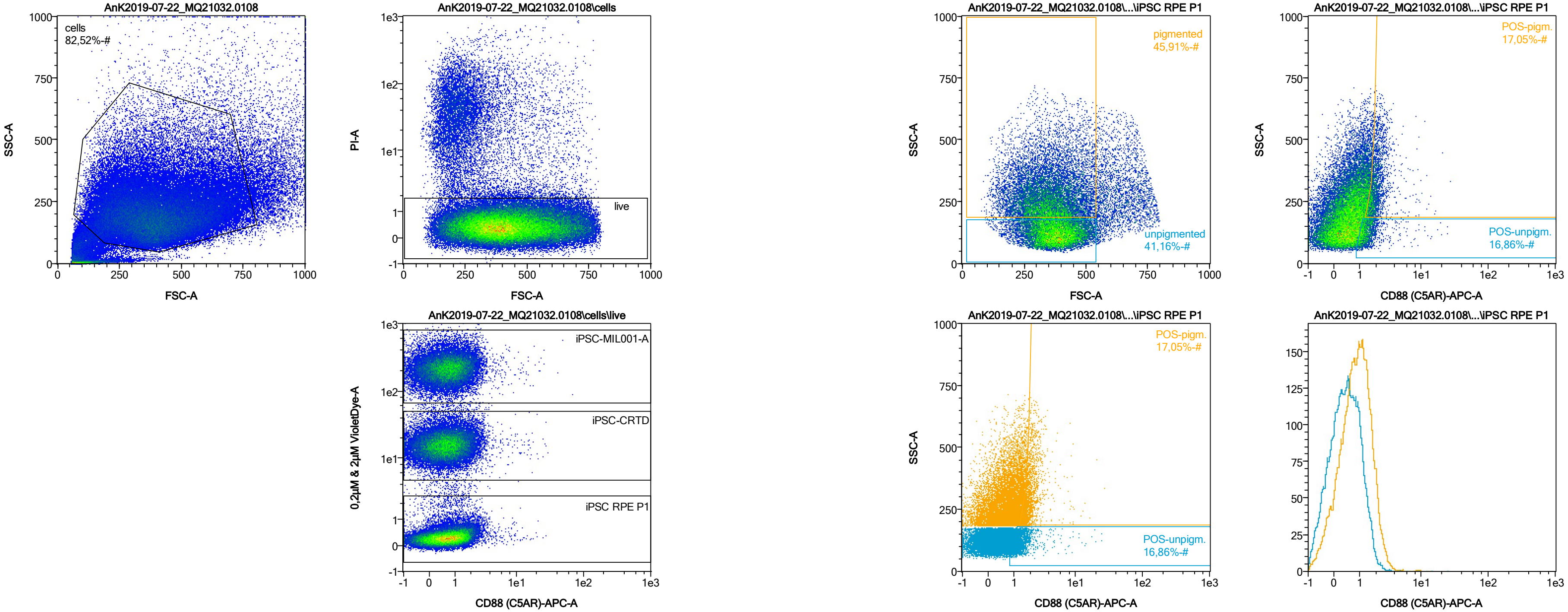

Name	%-#	CD88 (C5AR)-APC-A Mean	CD88 (C5AR)-APC-A Median
POS-unpigm.	16,86	1,54	1,29
POS-pigm.	17,05	2,11	1,88

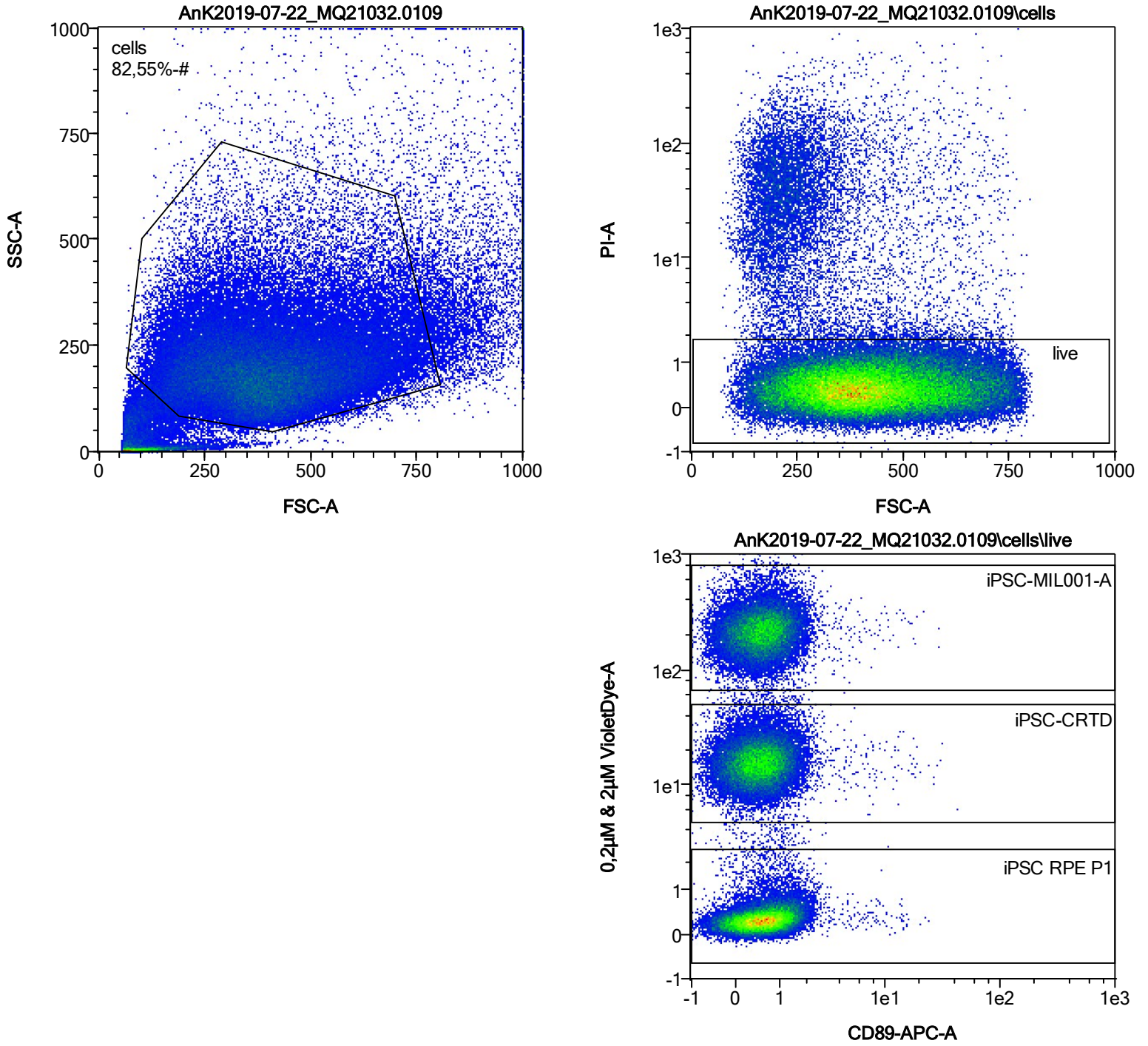

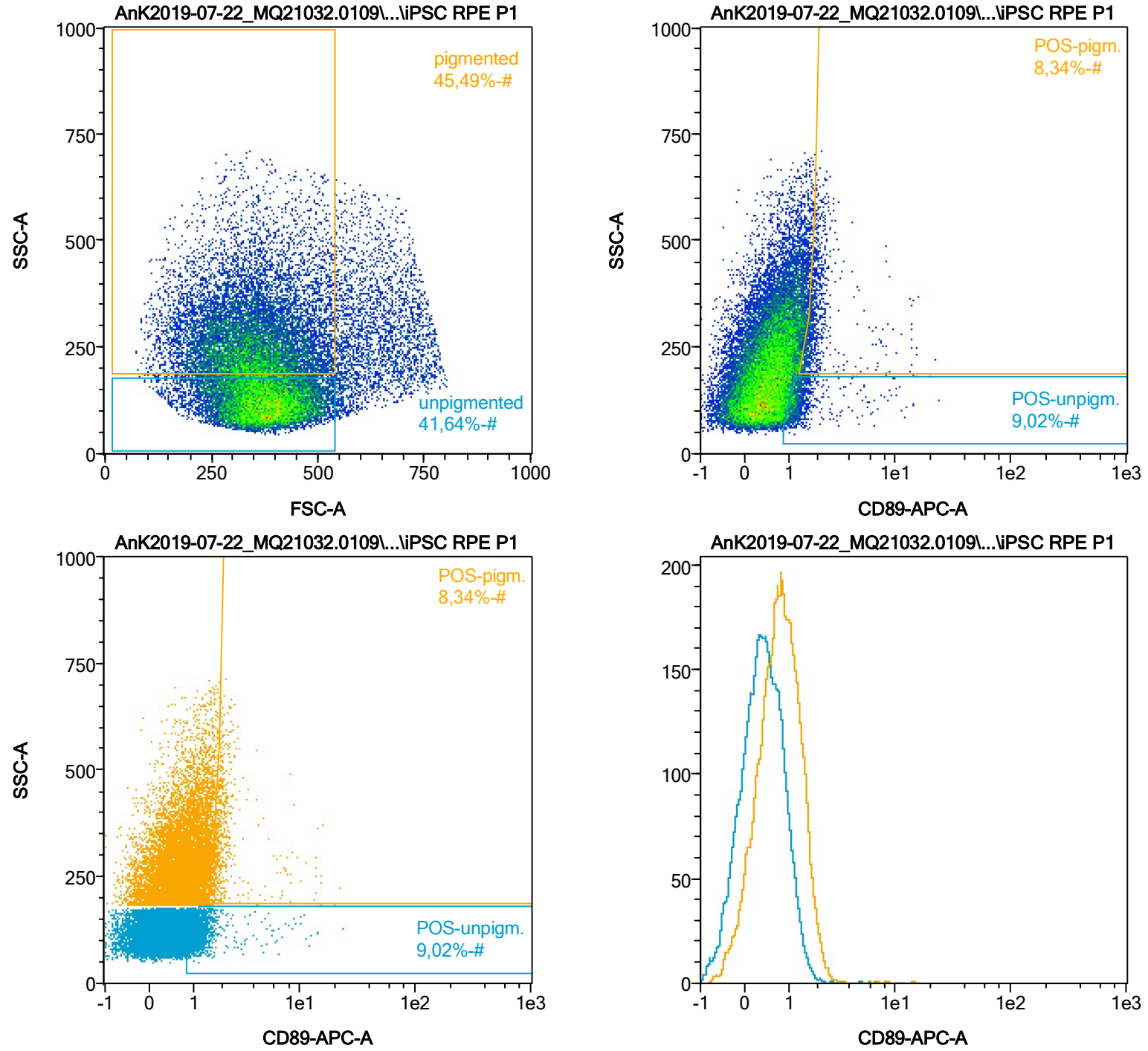

Name	%-#	CD89-APC-A Mean	CD89-APC-A Median
POS-unpigm.	9,02	1,52	1,21
POS-pigm.	8,34	2,21	1,80

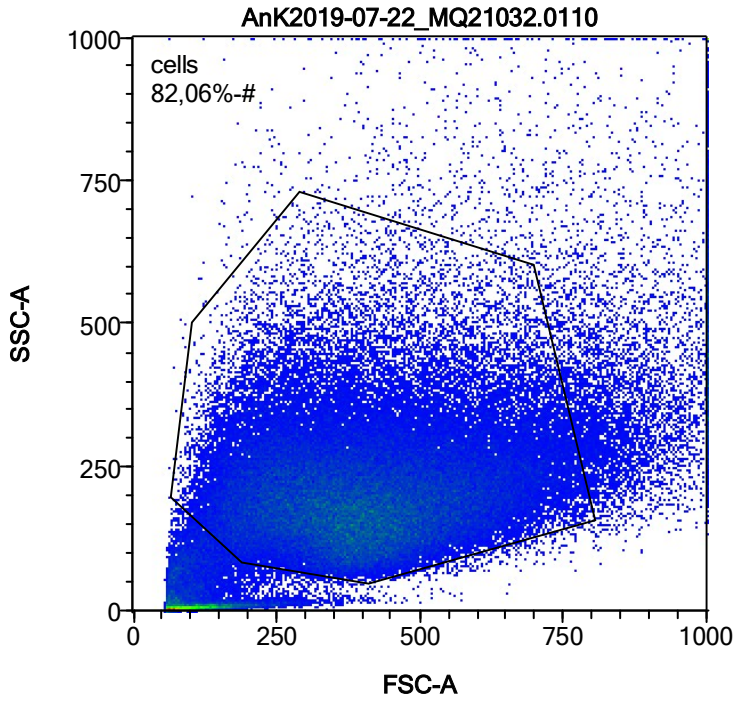

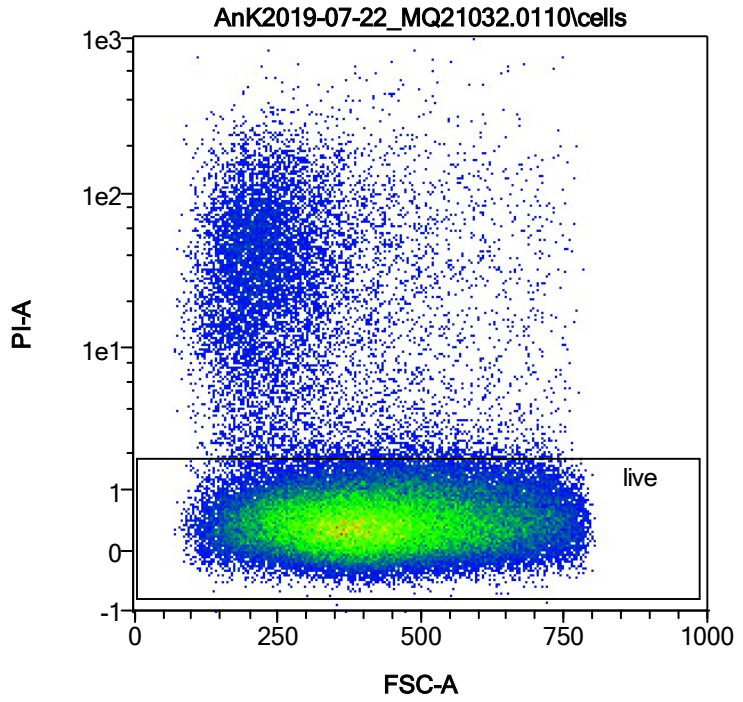

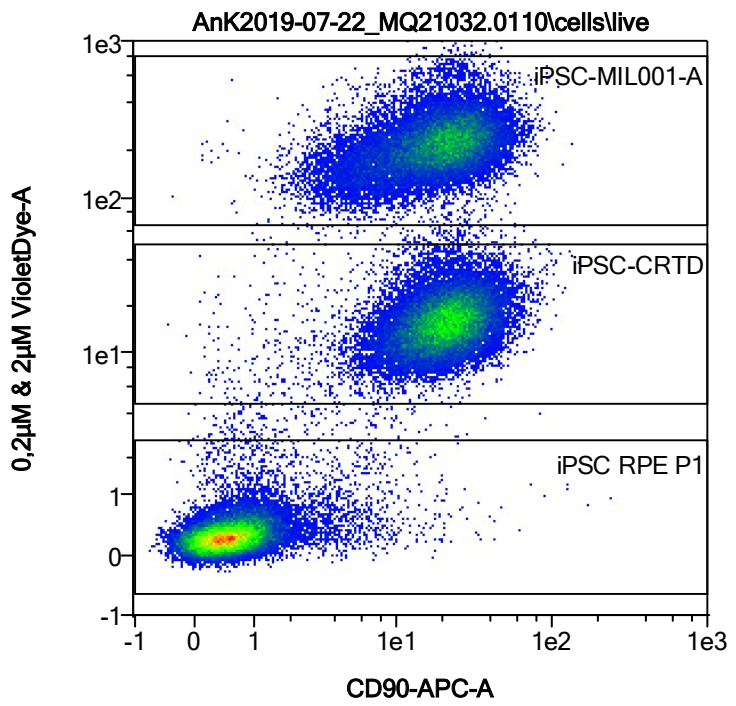

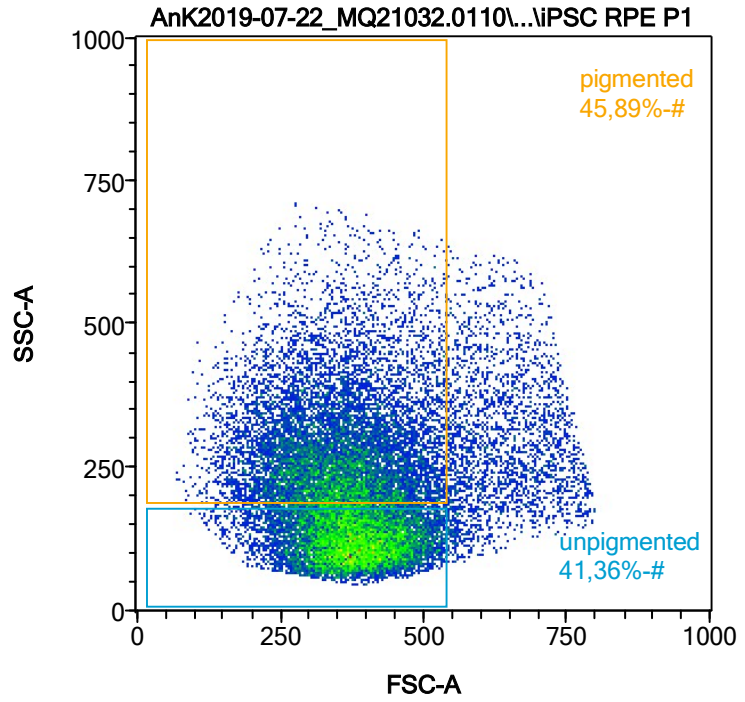

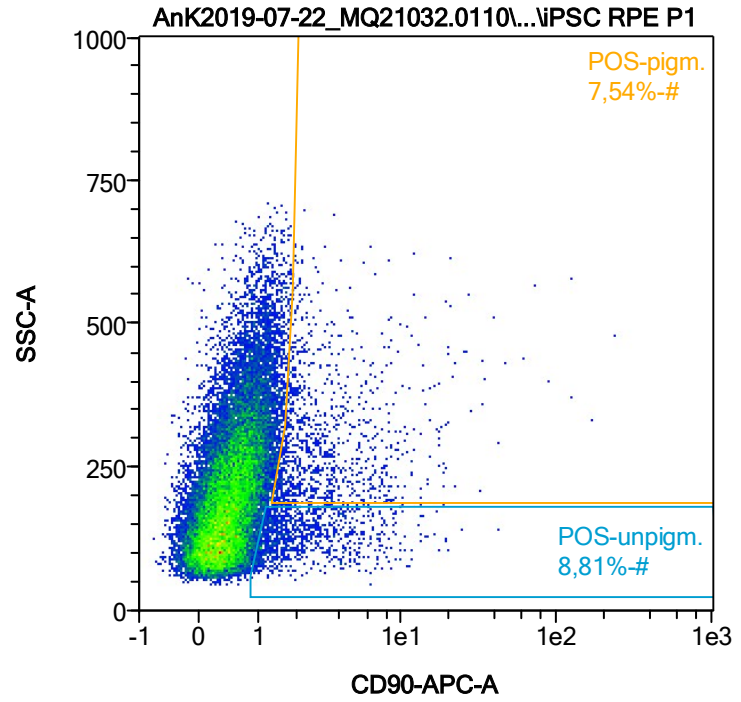

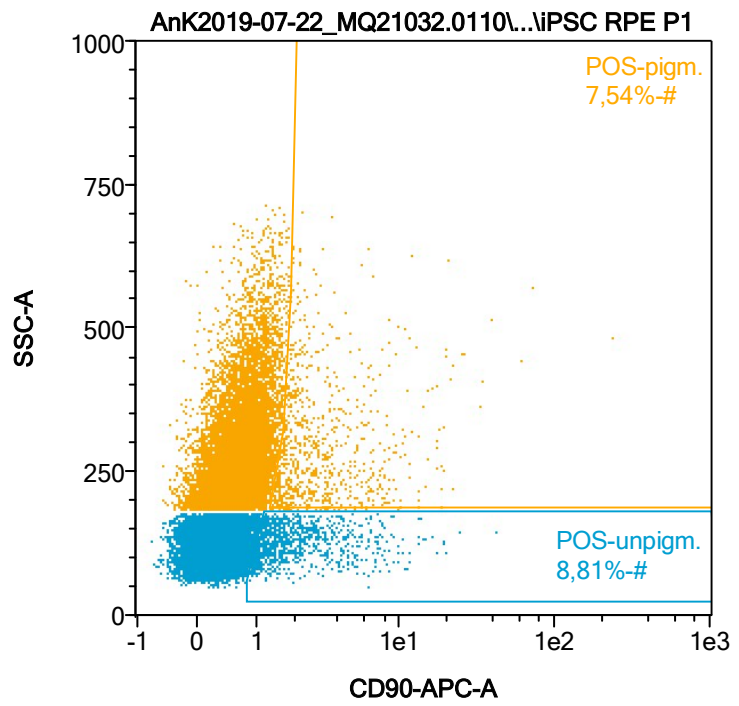

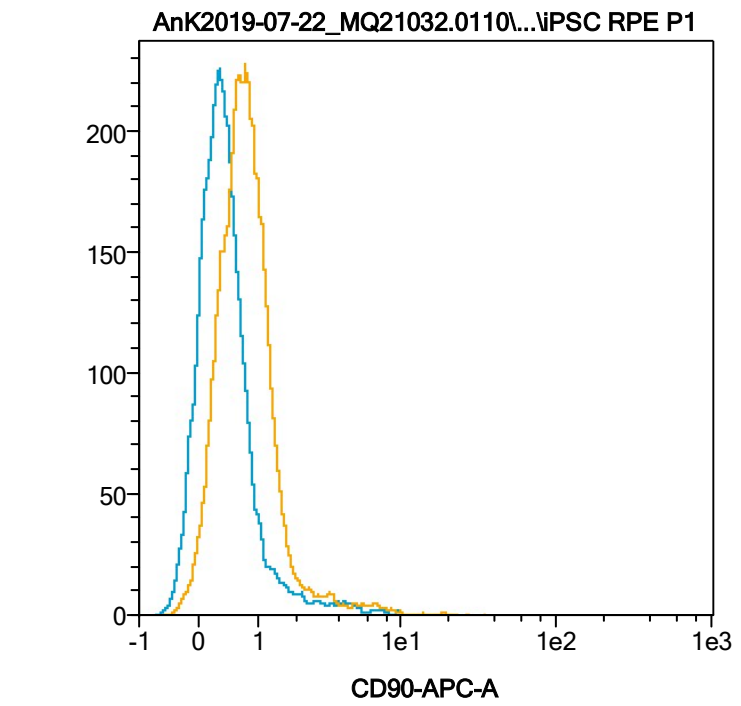

Name	%-#	CD90-APC-A Mean	CD90-APC-A Median
POS-unpigm.	8,81	2,65	1,68
POS-pigm.	7,54	4,20	2,40

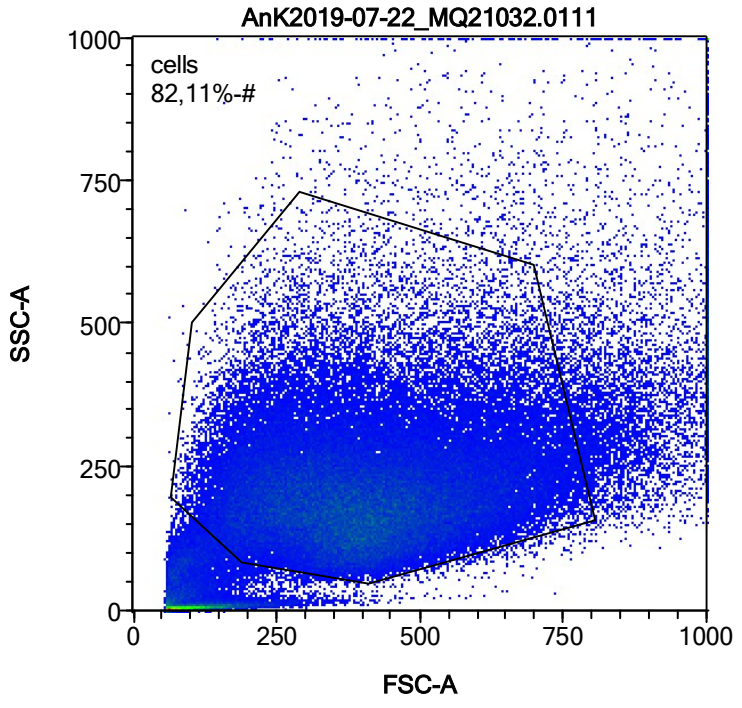

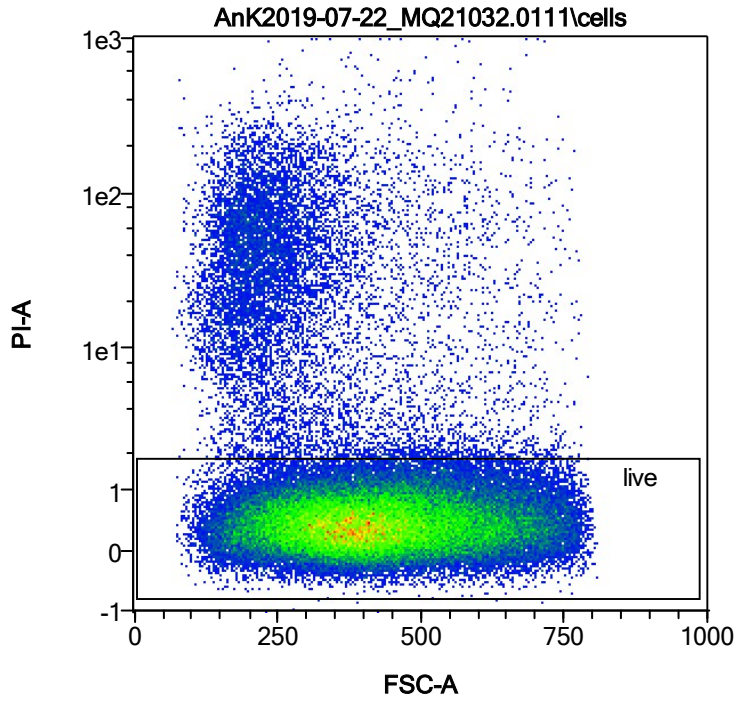

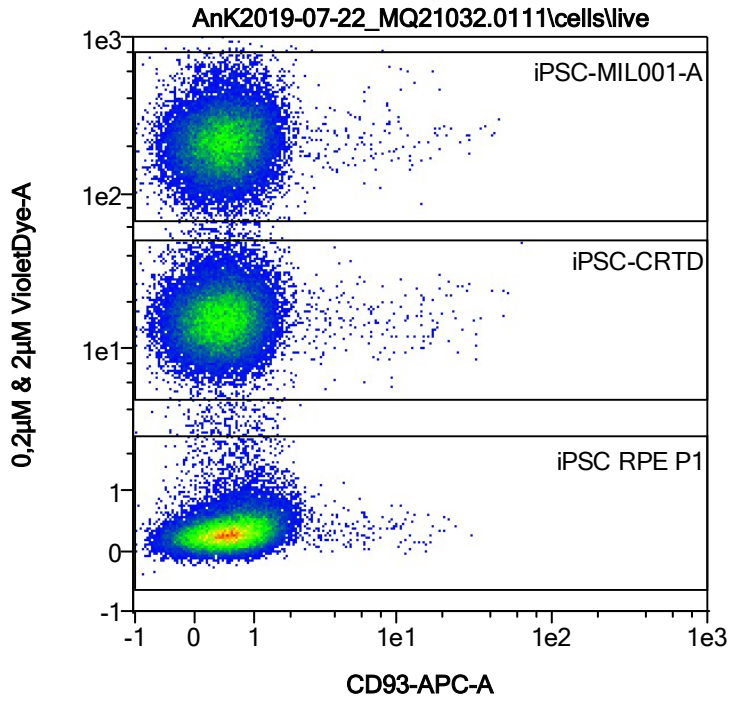

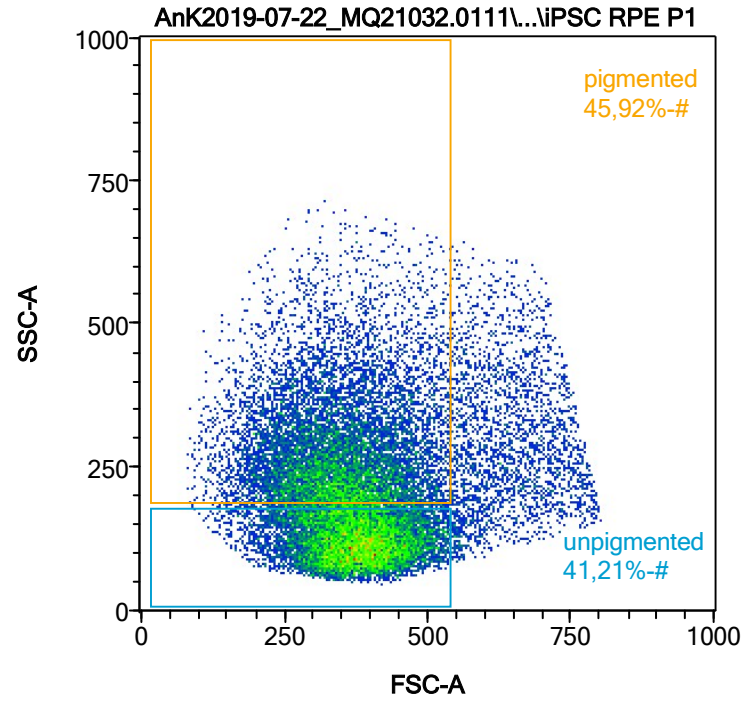

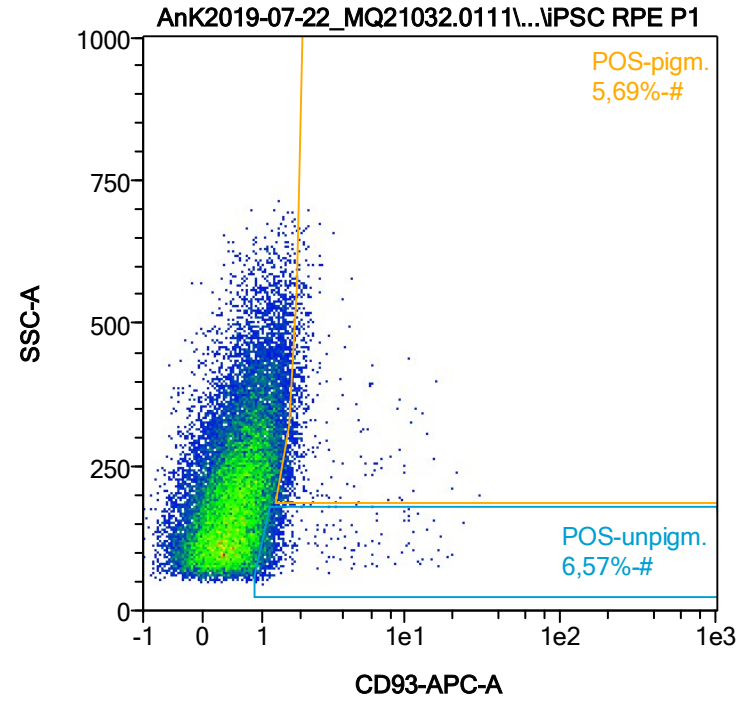

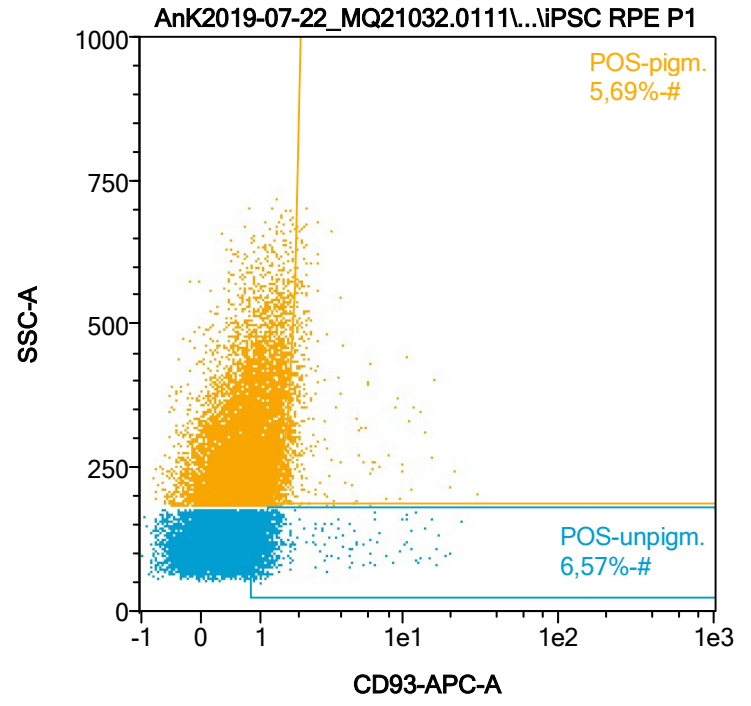

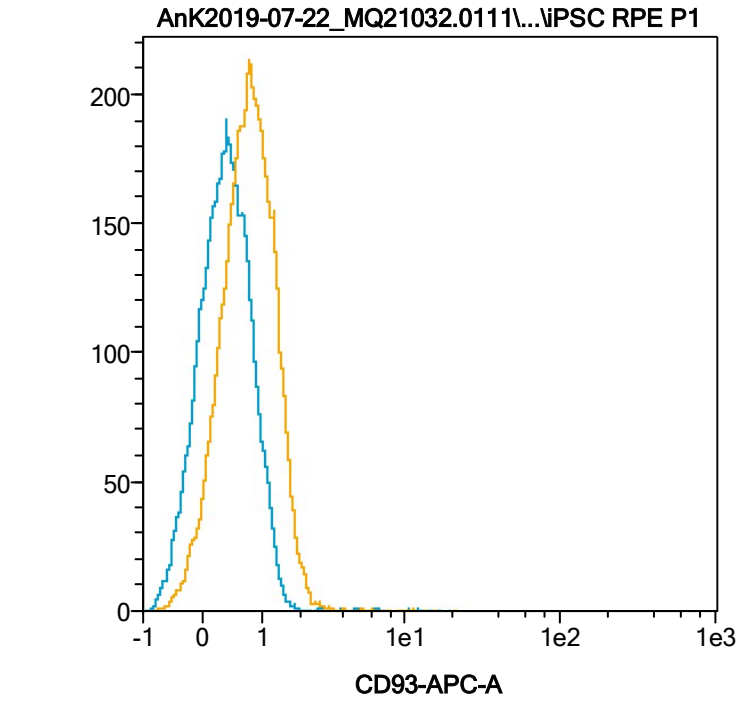

Name	%-#	CD93-APC-A Mean	CD93-APC-A Median
POS-unpigm.	6,57	1,81	1,18
POS-pigm.	5,69	2,28	1,80

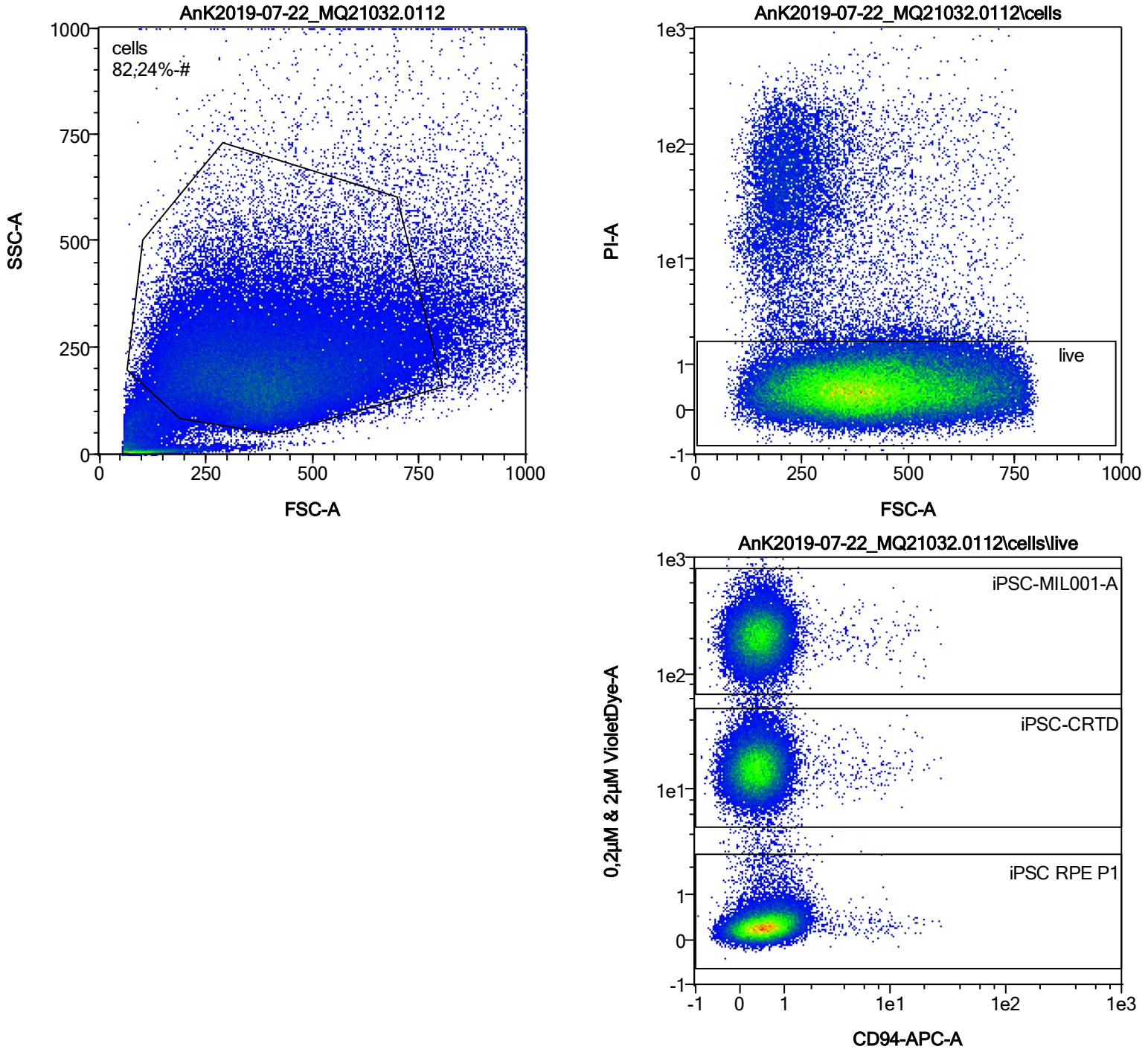

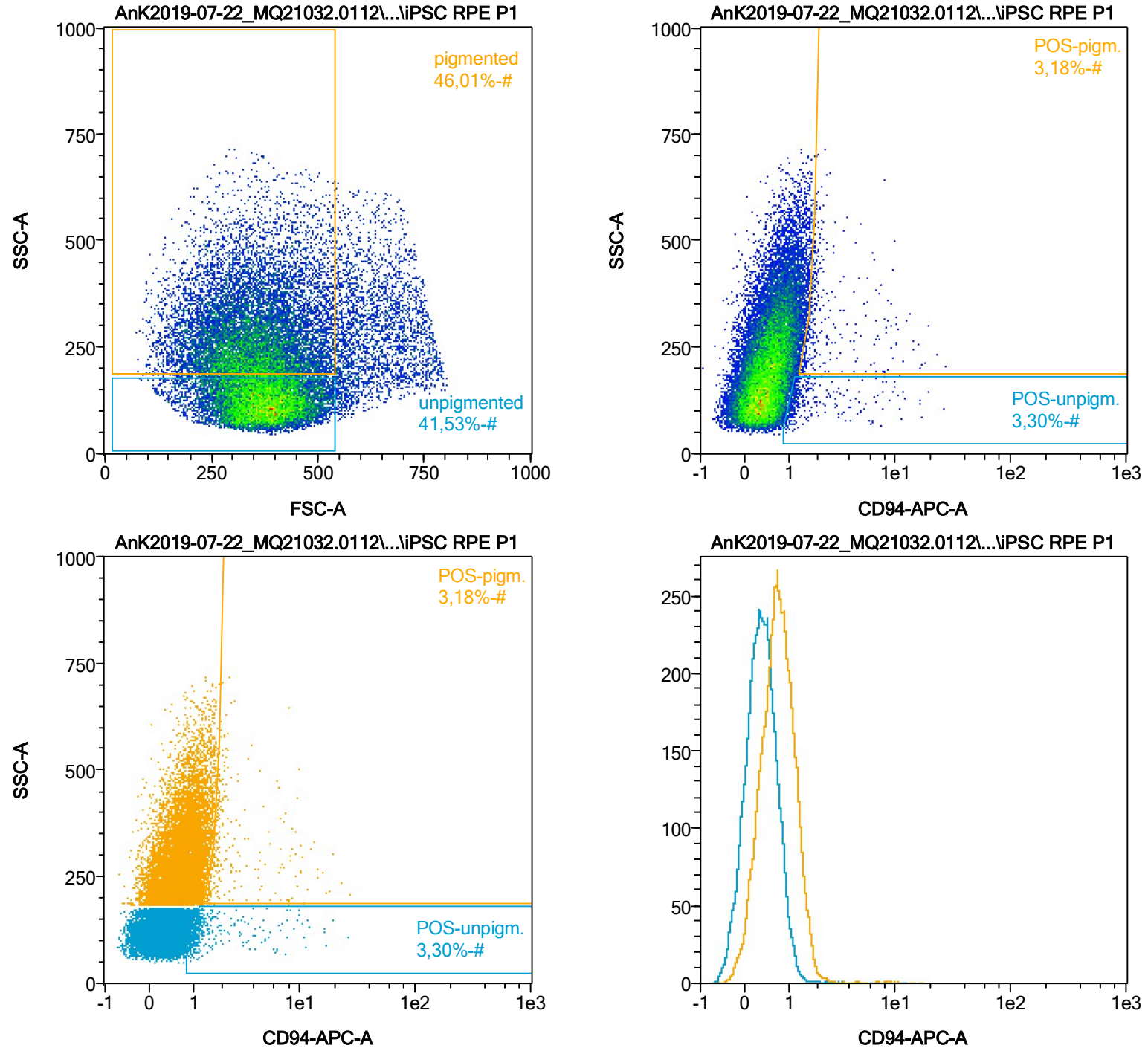

Name	%-#	CD94-APC-A Mean	CD94-APC-A Median
POS-unpigm.	3,30	2,15	1,18
POS-pigm.	3,18	2,94	1,84

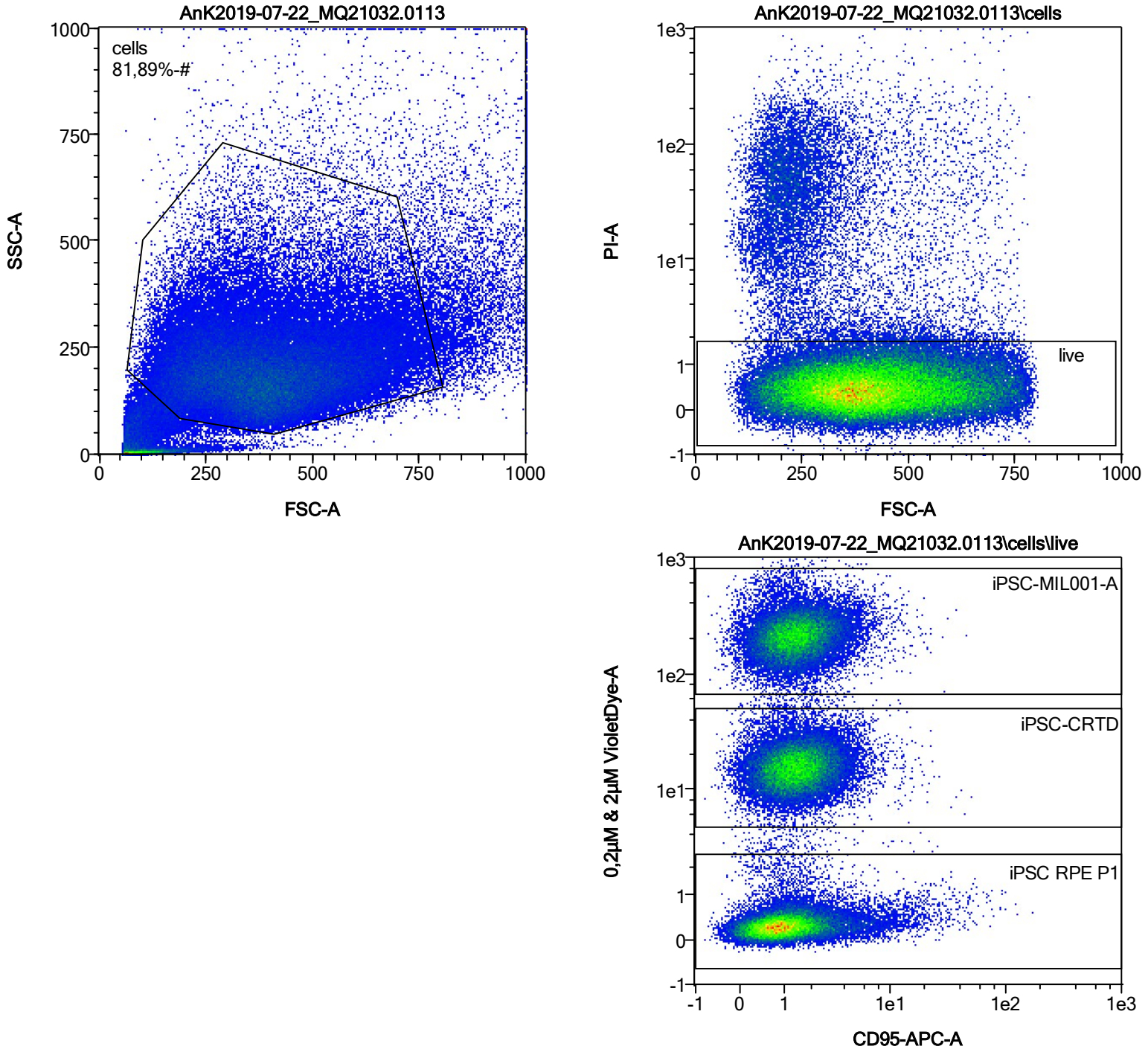

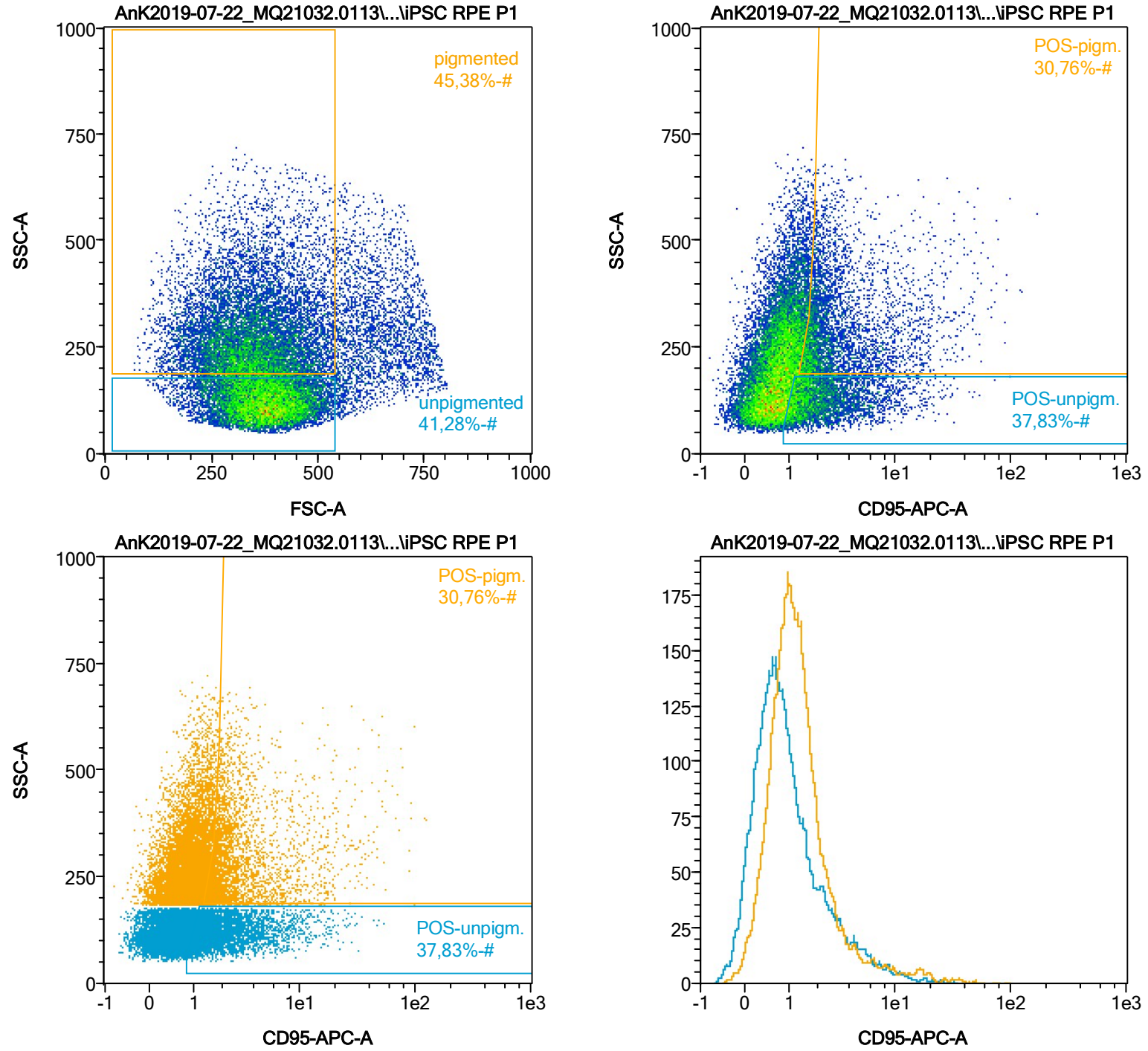

Name	%-#	CD95-APC-A Mean	CD95-APC-A Median
POS-unpigm.	37,83	2,87	1,82
POS-pigm.	30,76	4,44	2,26

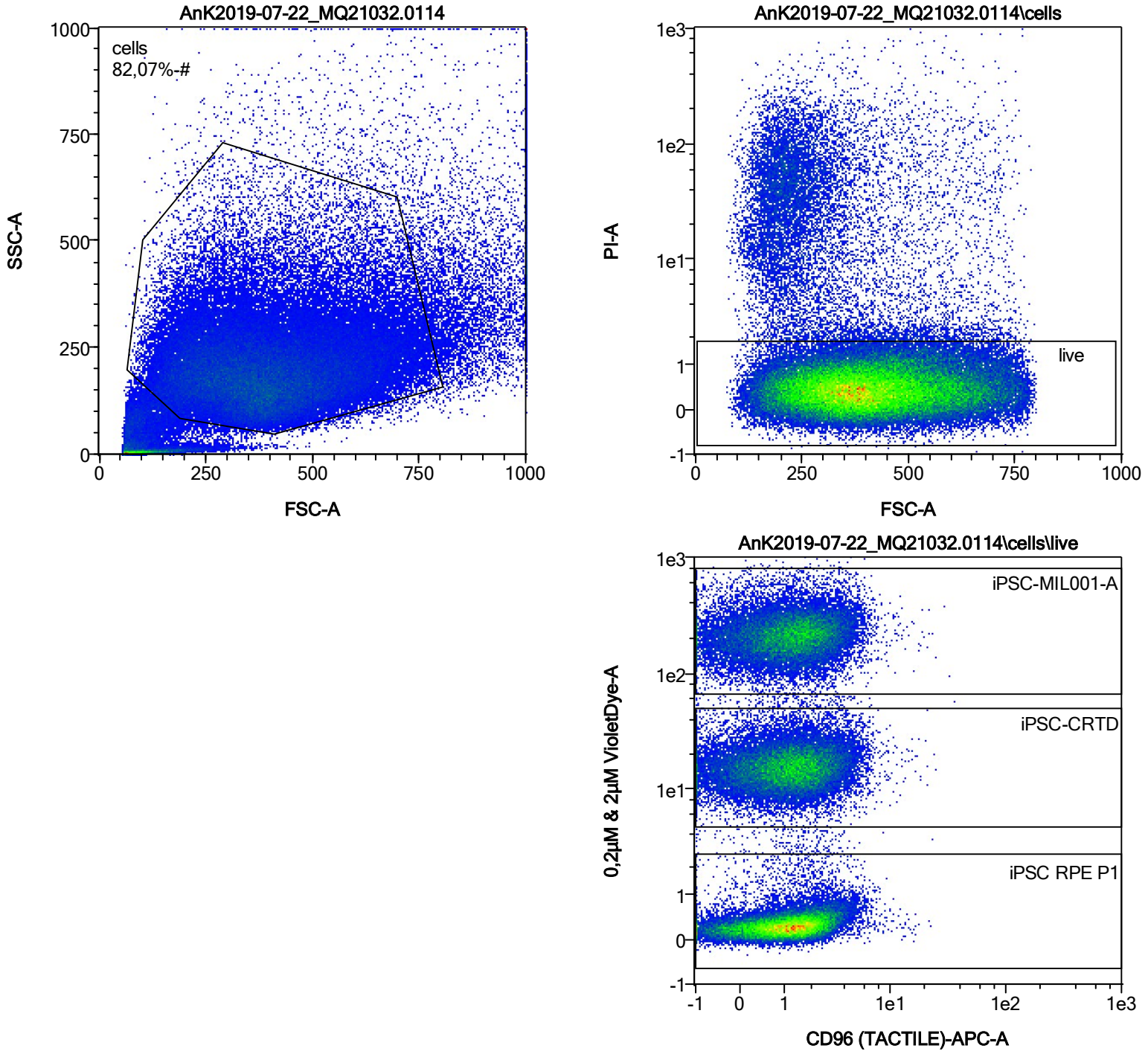

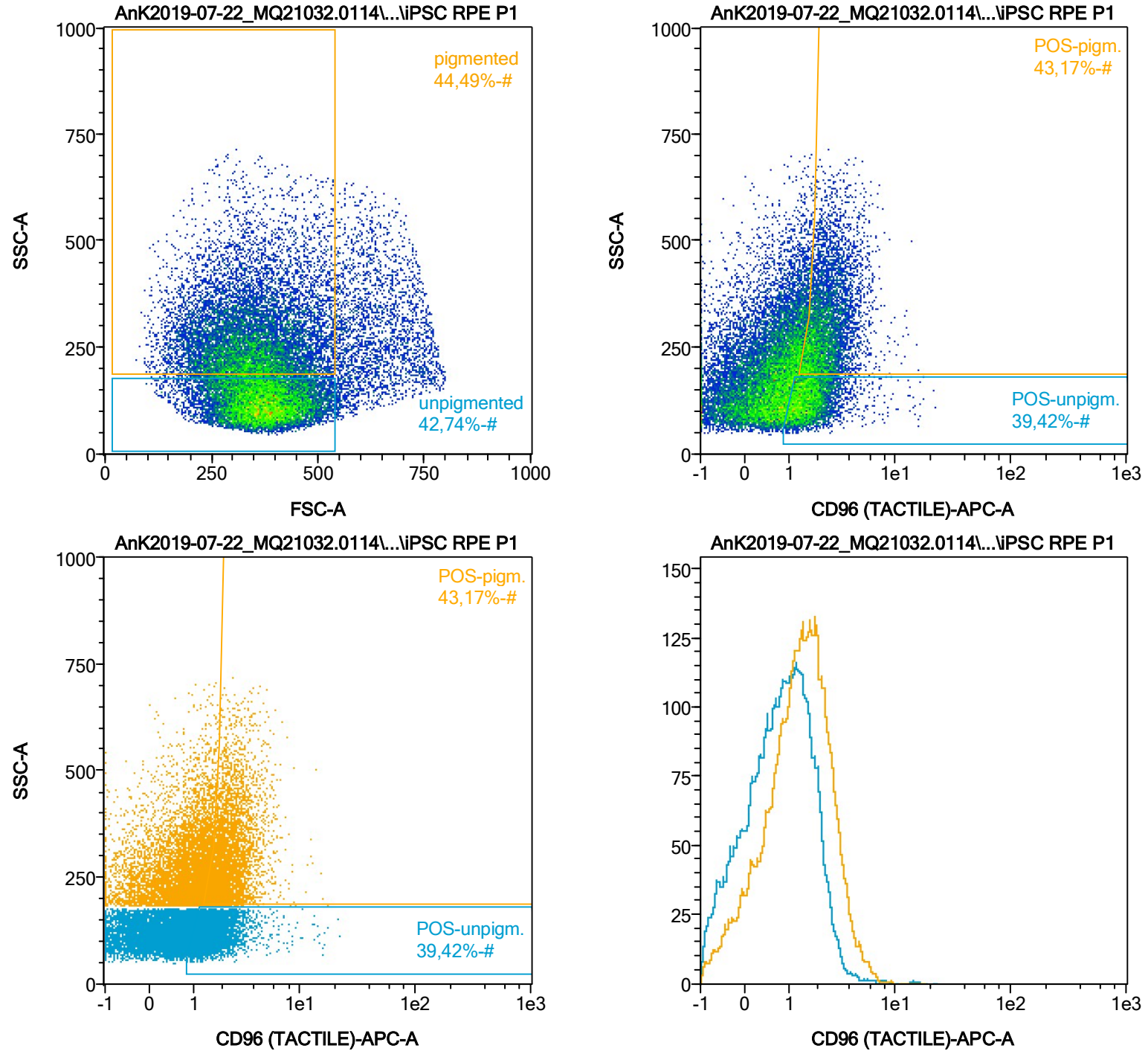

Name	%-#	CD96 (TACTILE)-APC-A Mean	CD96 (TACTILE)-APC-A Median
POS-unpigm.	39,42	1,81	1,57
POS-pigm.	43,17	2,48	2,22

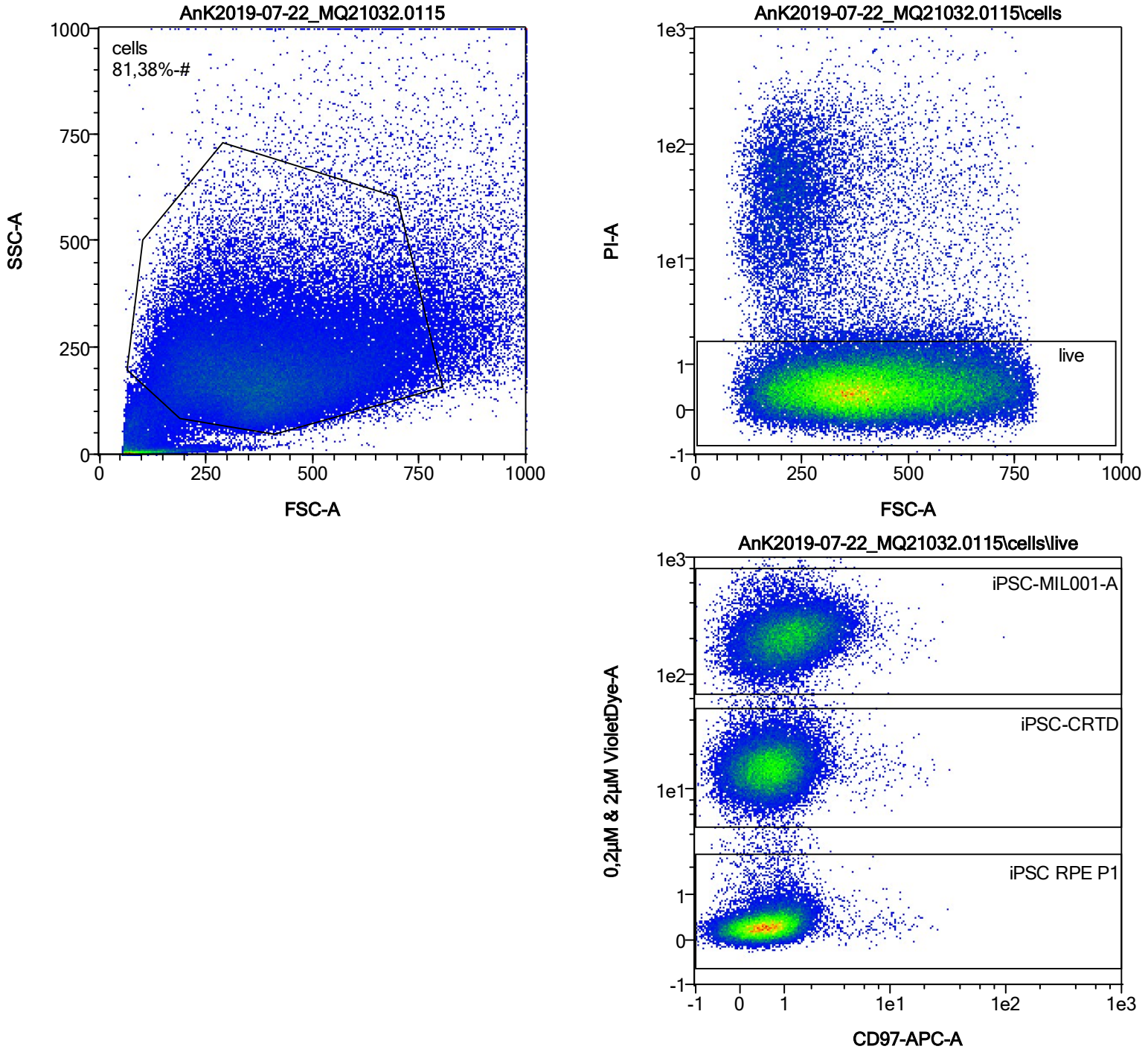

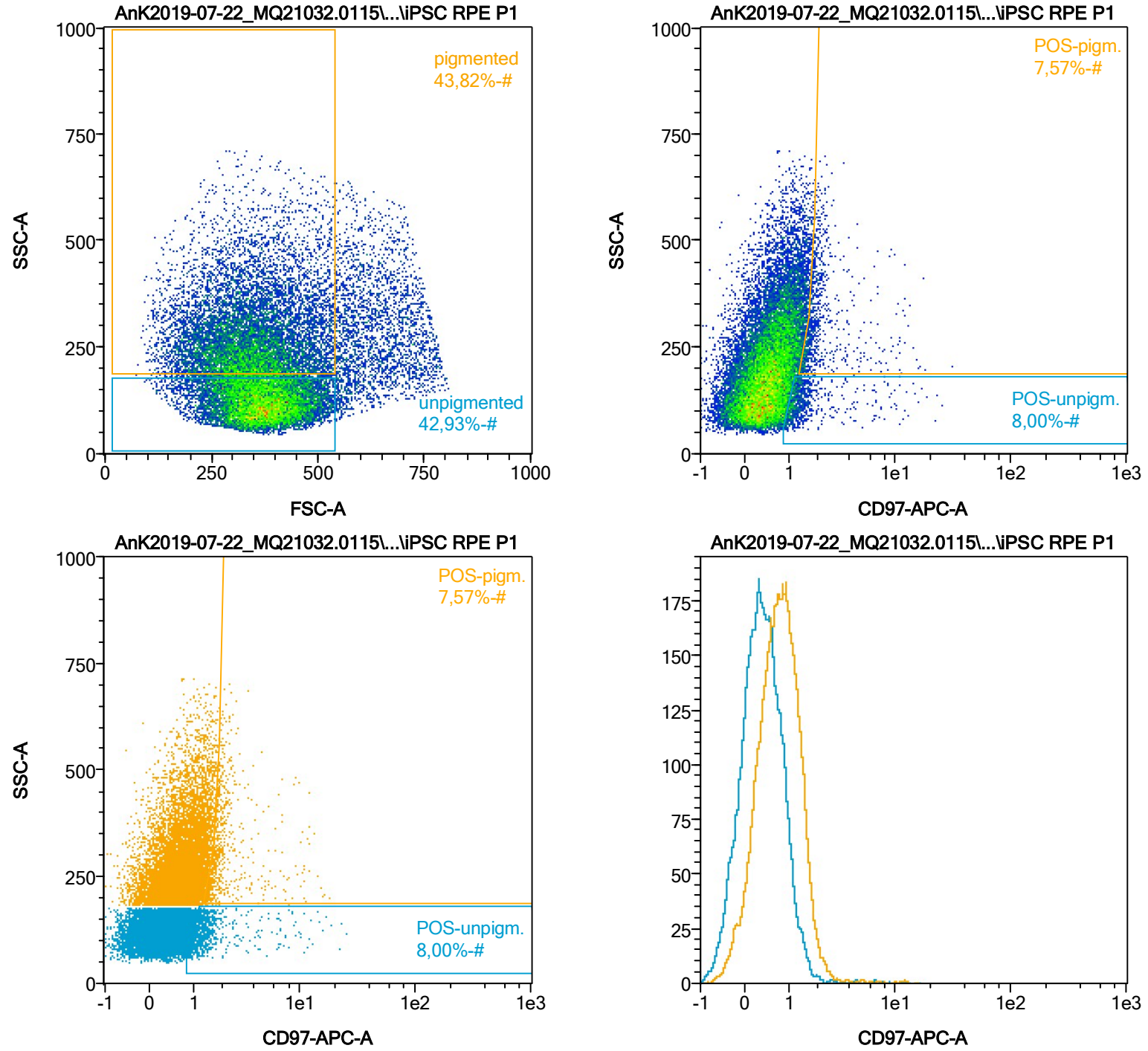

Name	%-#	CD97-APC-A Mean	CD97-APC-A Median
POS-unpigm.	8,00	1,81	1,23
POS-pigm.	7,57	2,40	1,83

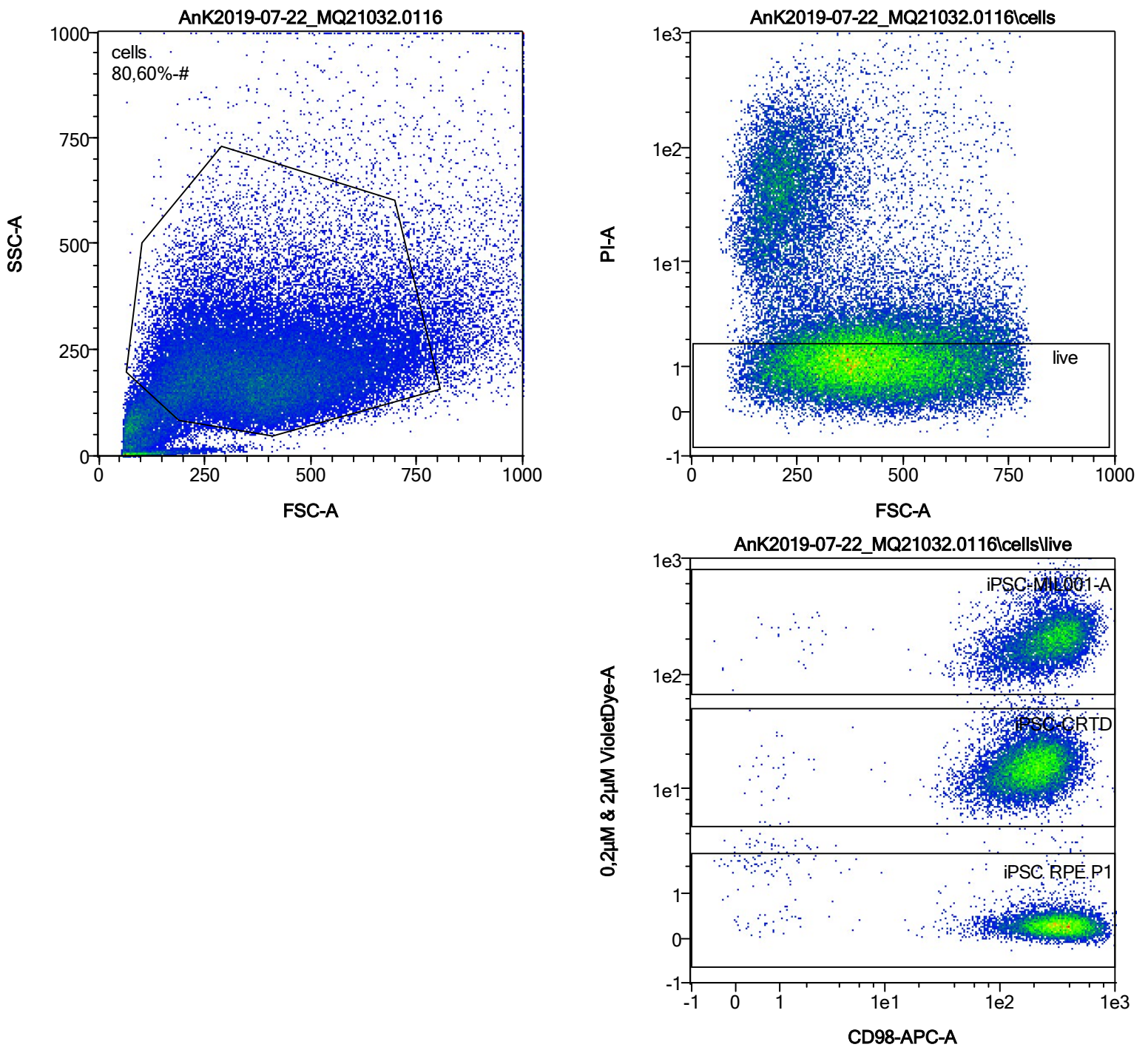

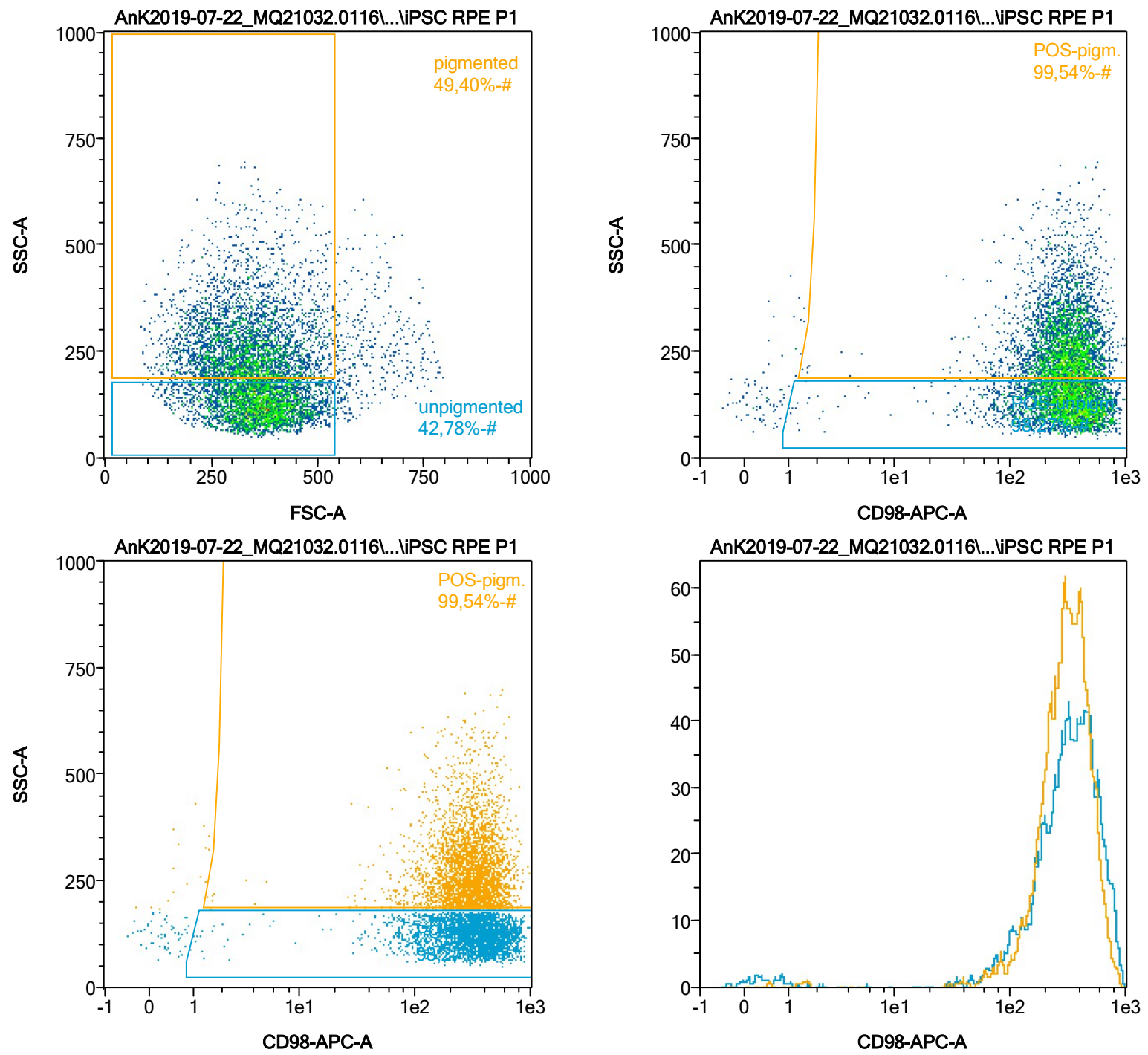

Name	%-#	CD98-APC-A Mean	CD98-APC-A Median
POS-unpigm.	98,27	354,90	330,95
POS-pigm.	99,54	327,07	309,44

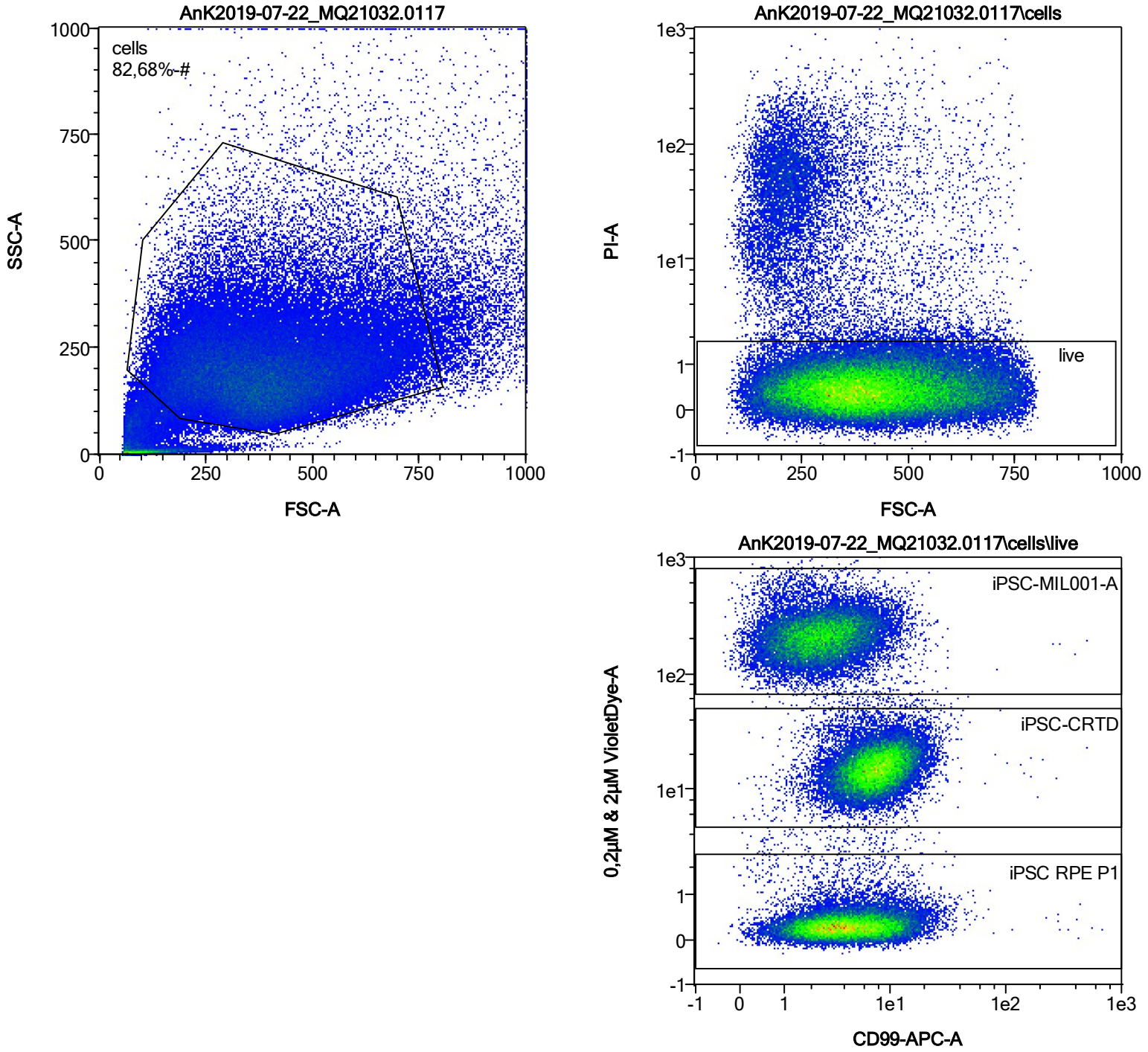

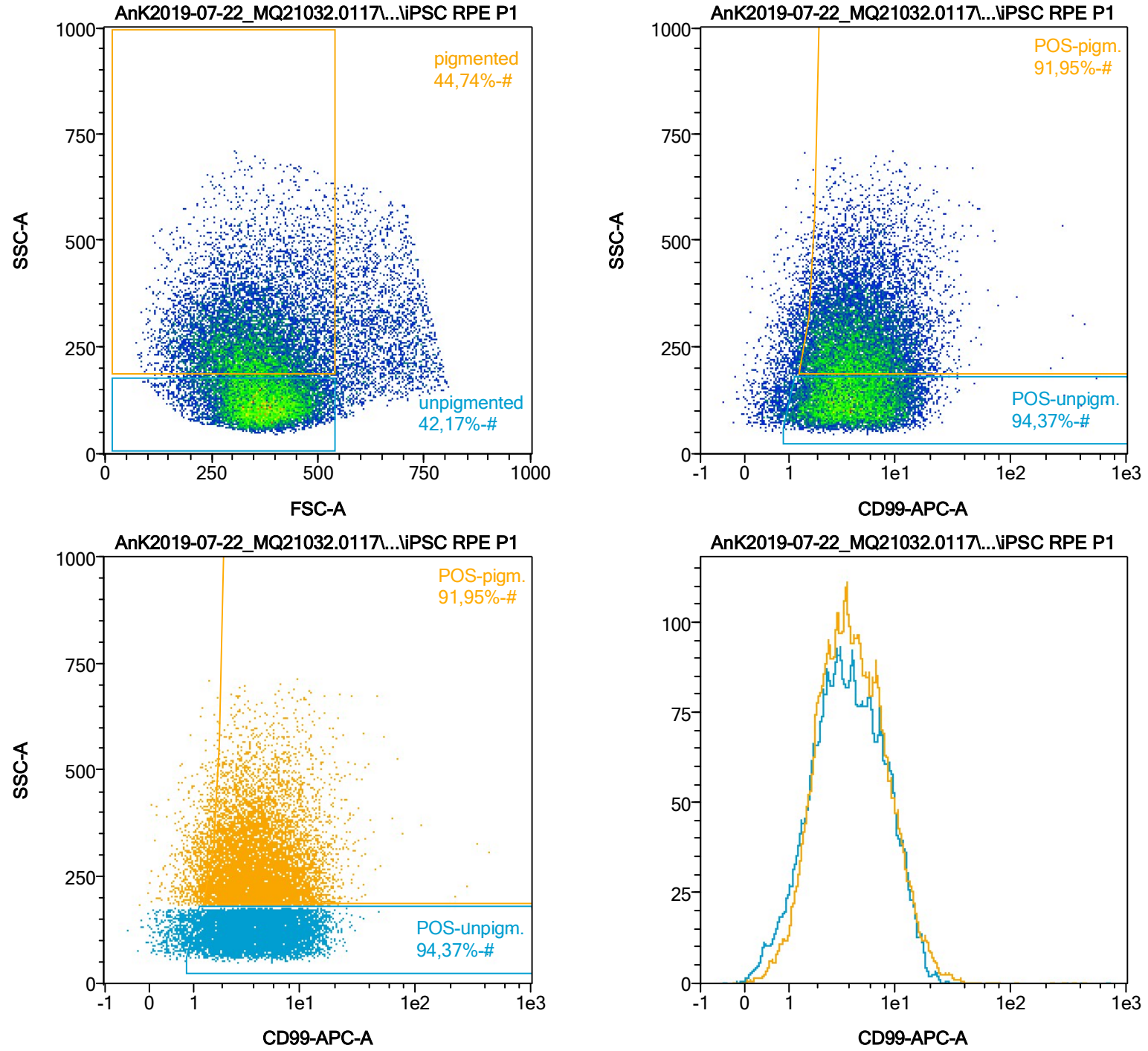

Name	%-#	CD99-APC-A Mean	CD99-APC-A Median
POS-unpigm.	94,37	5,31	4,05
POS-pigm.	91,95	5,74	4,29

Date:2024-06-14

Operator: Sebastian Knoebel

Version:2.13.0+54.g95abf0f4

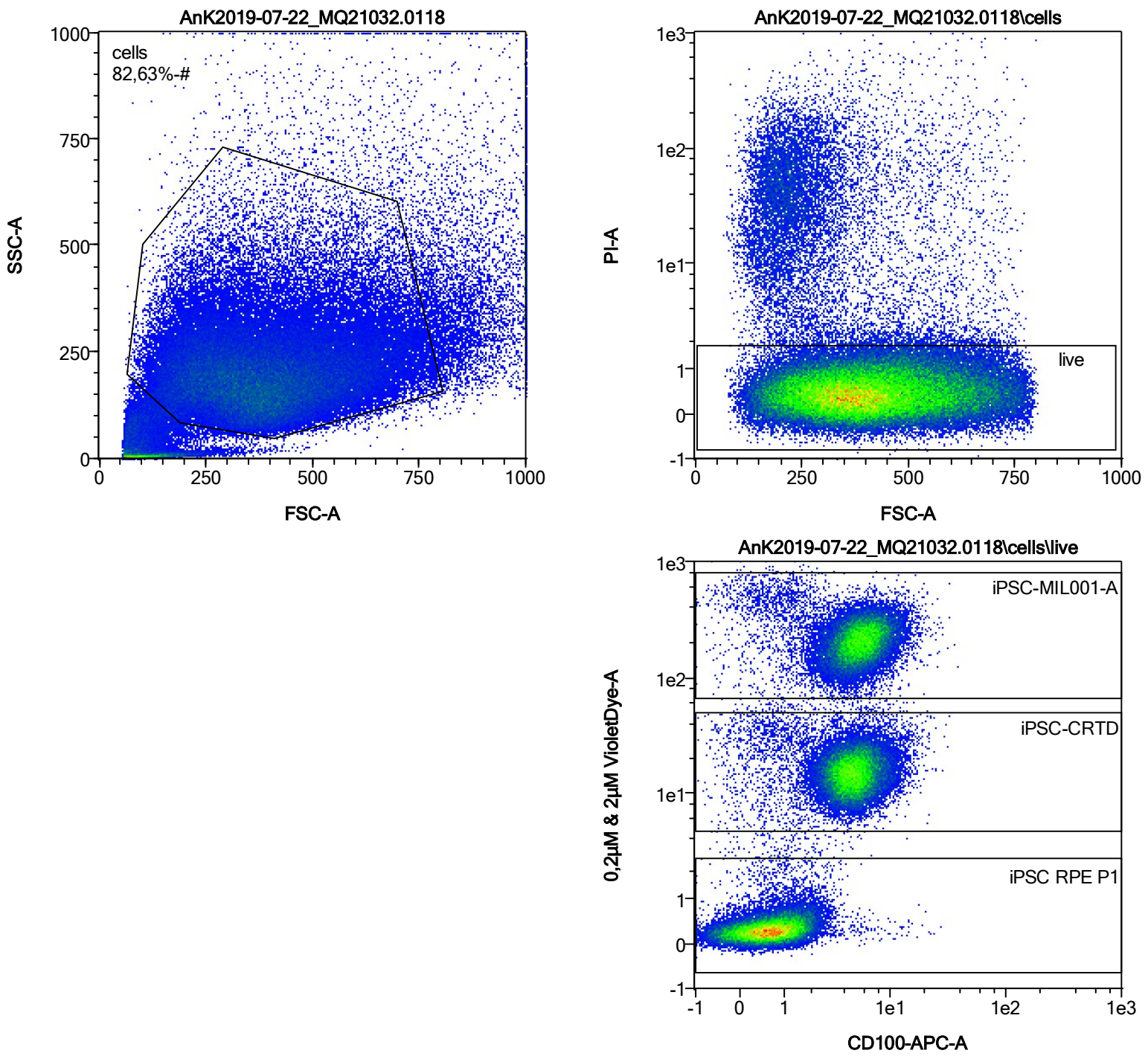

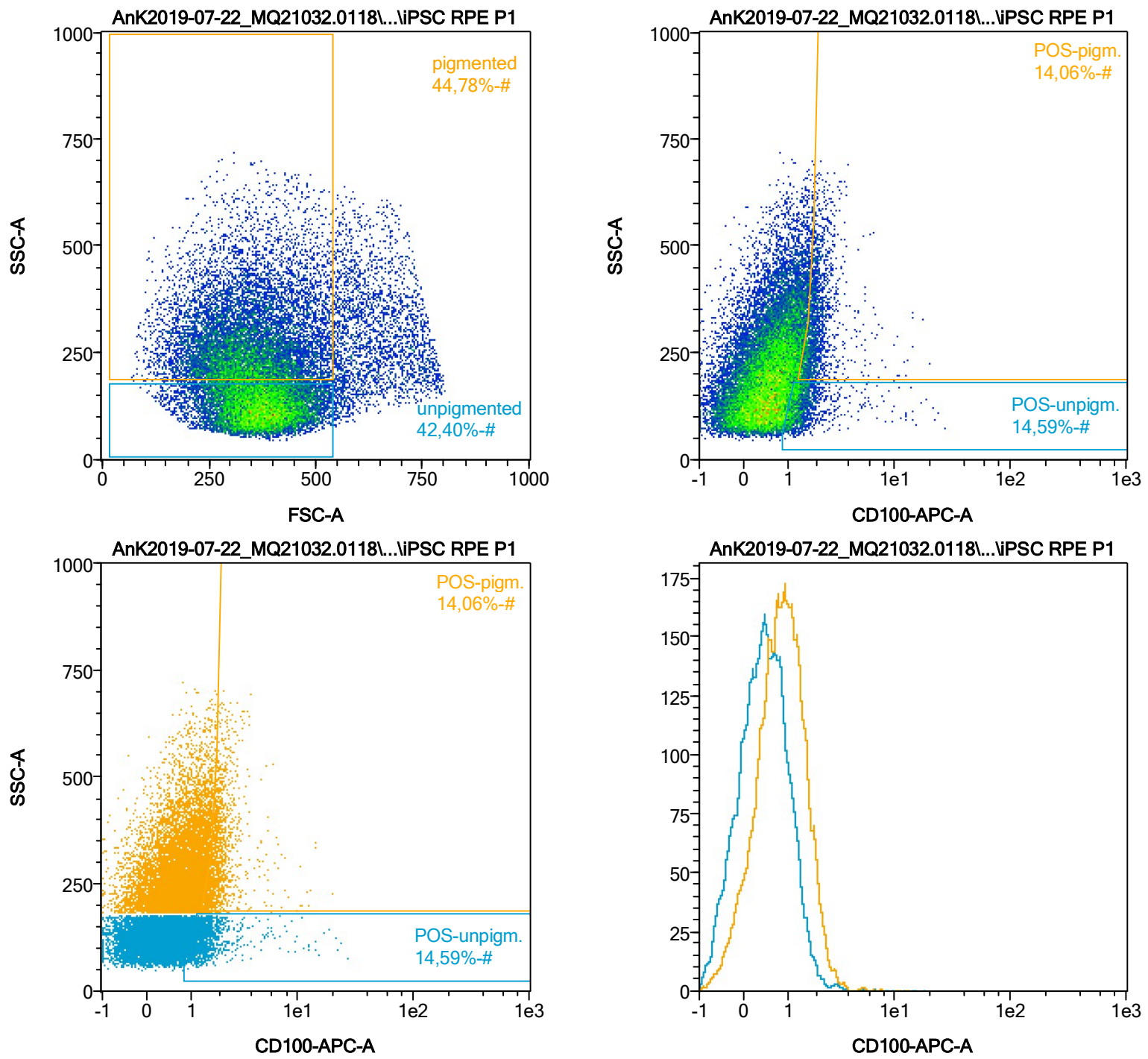

Name	%-#	CD100-APC-A Mean	CD100-APC-A Median
POS-unpigm.	14,59	1,61	1,29
POS-pigm.	14,06	2,09	1,88

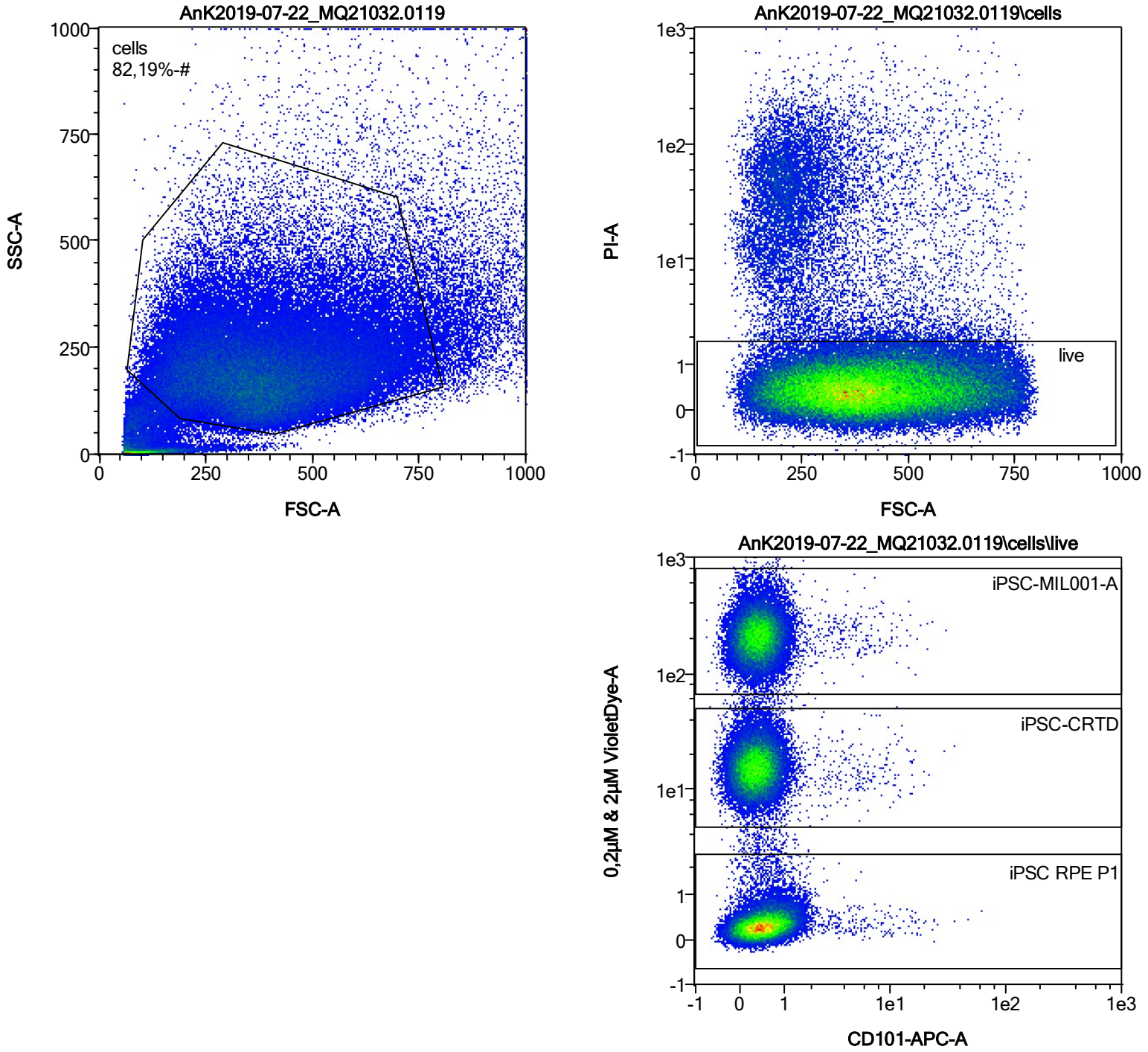

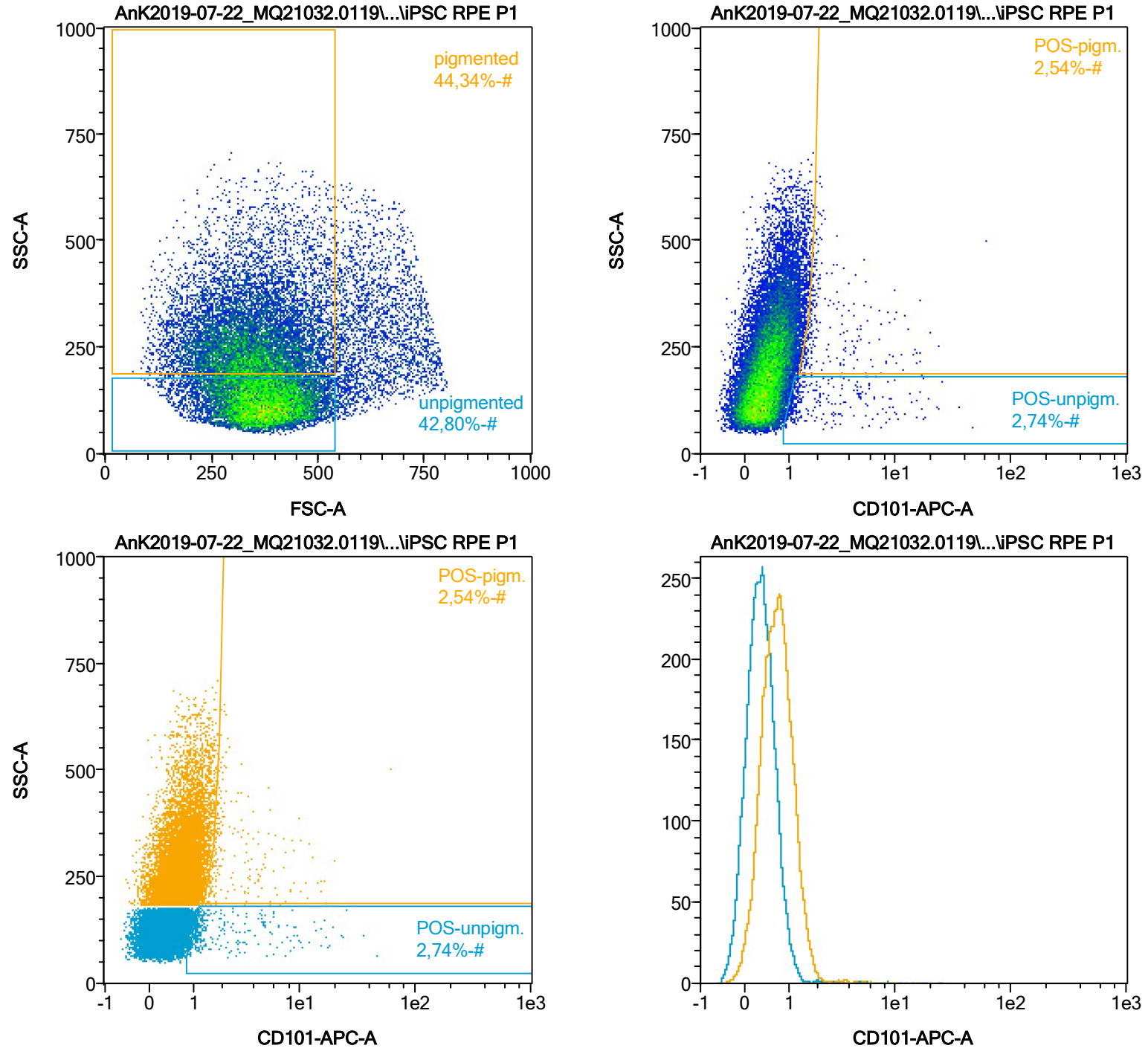

Name	%-#	CD101-APC-A Mean	CD101-APC-A Median
POS-unpigm.	2,74	3,31	1,31
POS-pigm.	2,54	3,25	1,89

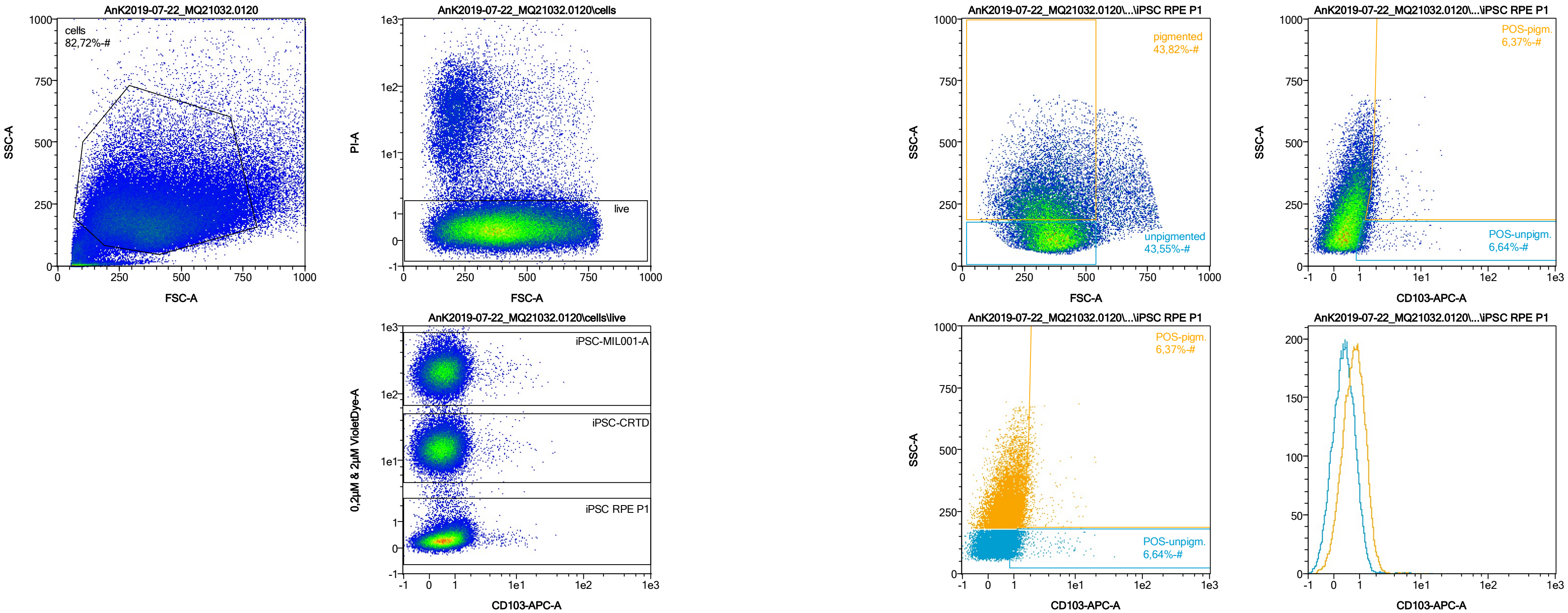

Name	%-#	CD103-APC-A Mean	CD103-APC-A Median
POS-unpigm.	6,64	1,78	1,20
POS-pigm.	6,37	2,21	1,80

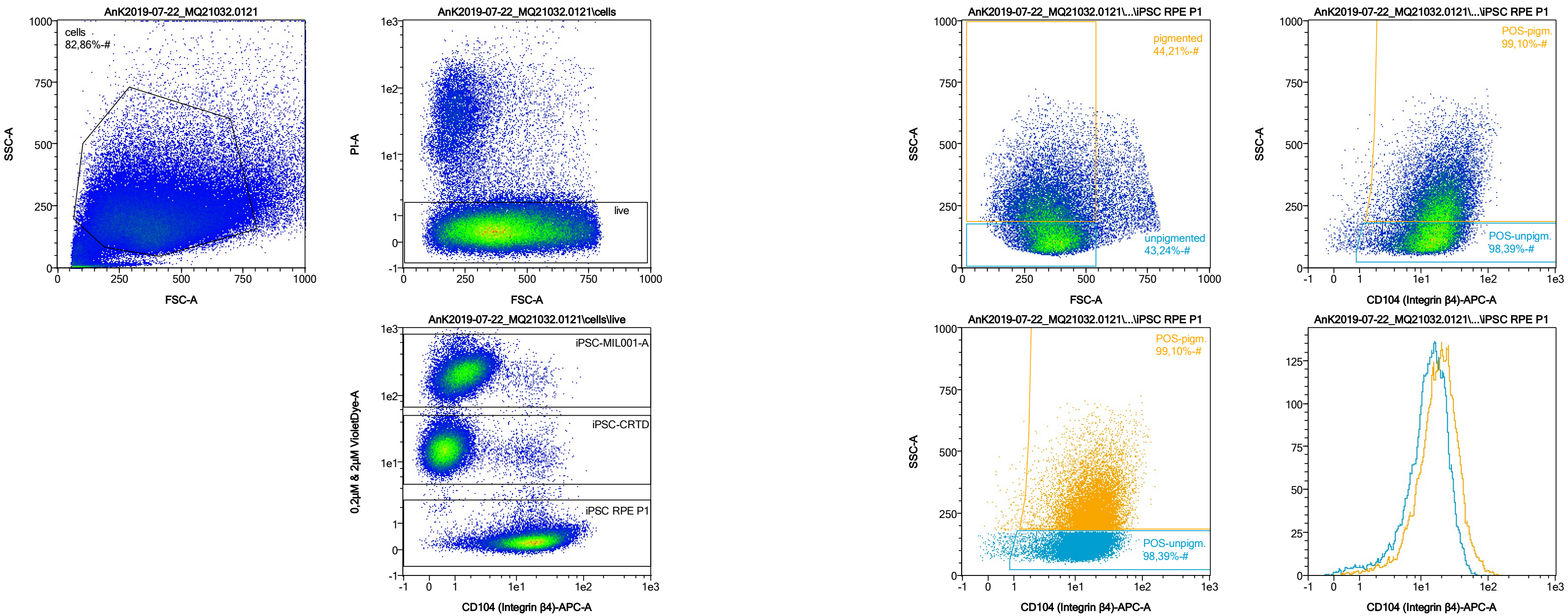

Name	%-#	CD104 (Integrin β4)-APC-A Mean	CD104 (Integrin β4)-APC-A Median
POS-unpigm.	98,39	15,17	13,69
POS-pigm.	99,10	21,60	18,69

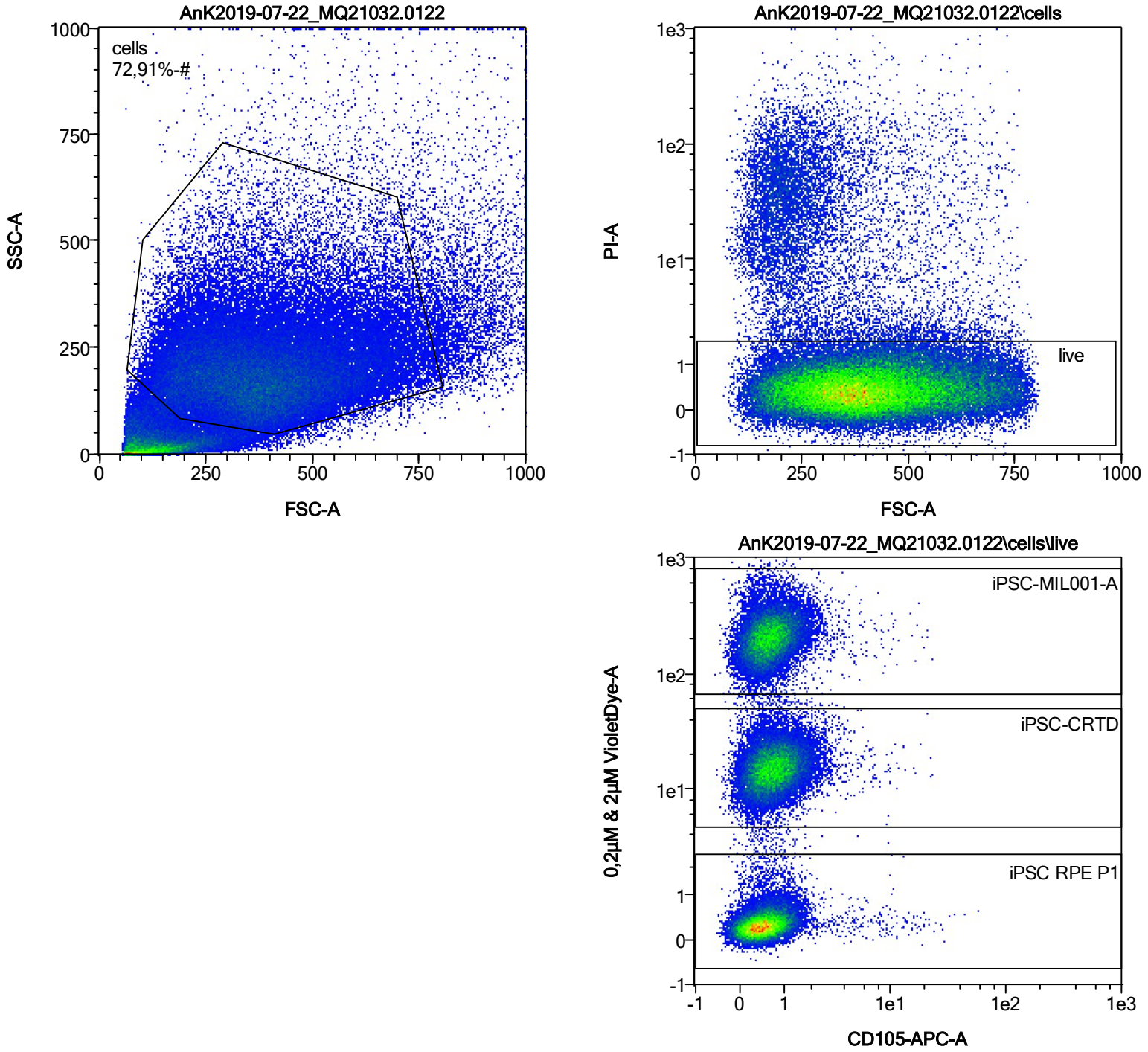

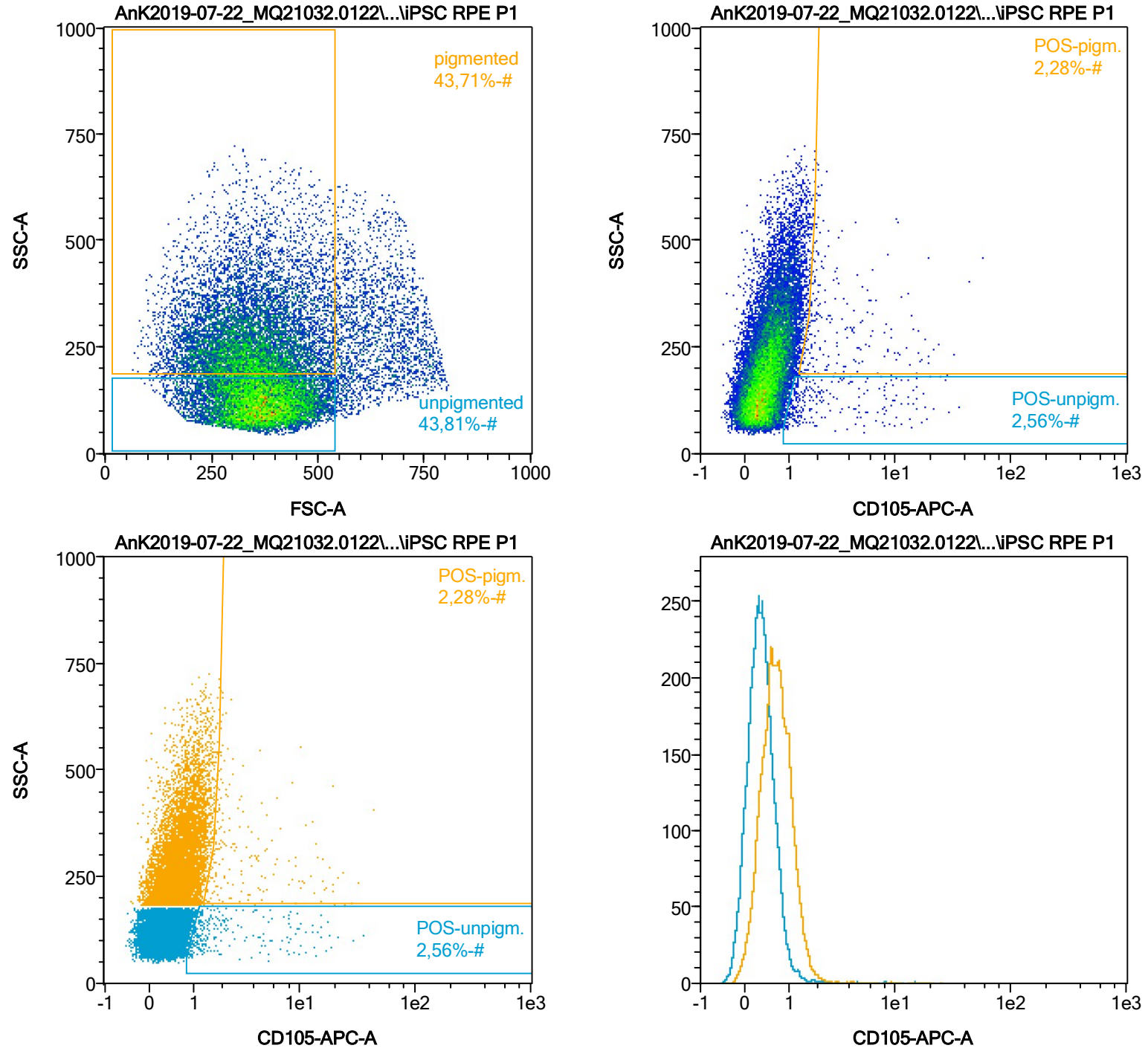

Name	%-#	CD105-APC-A Mean	CD105-APC-A Median
POS-unpigm.	2,56	3,51	1,44
POS-pigm.	2,28	4,73	2,15

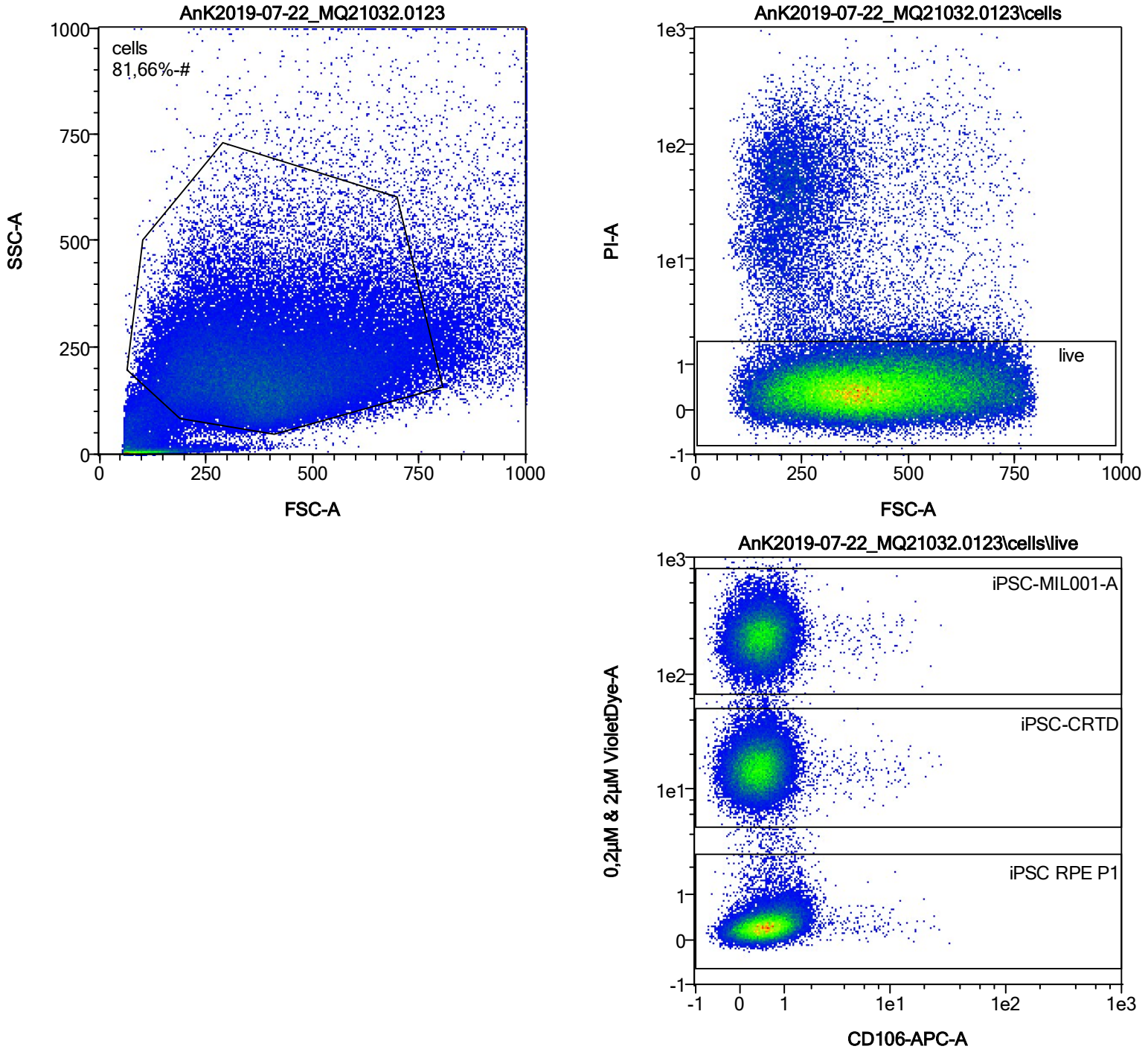

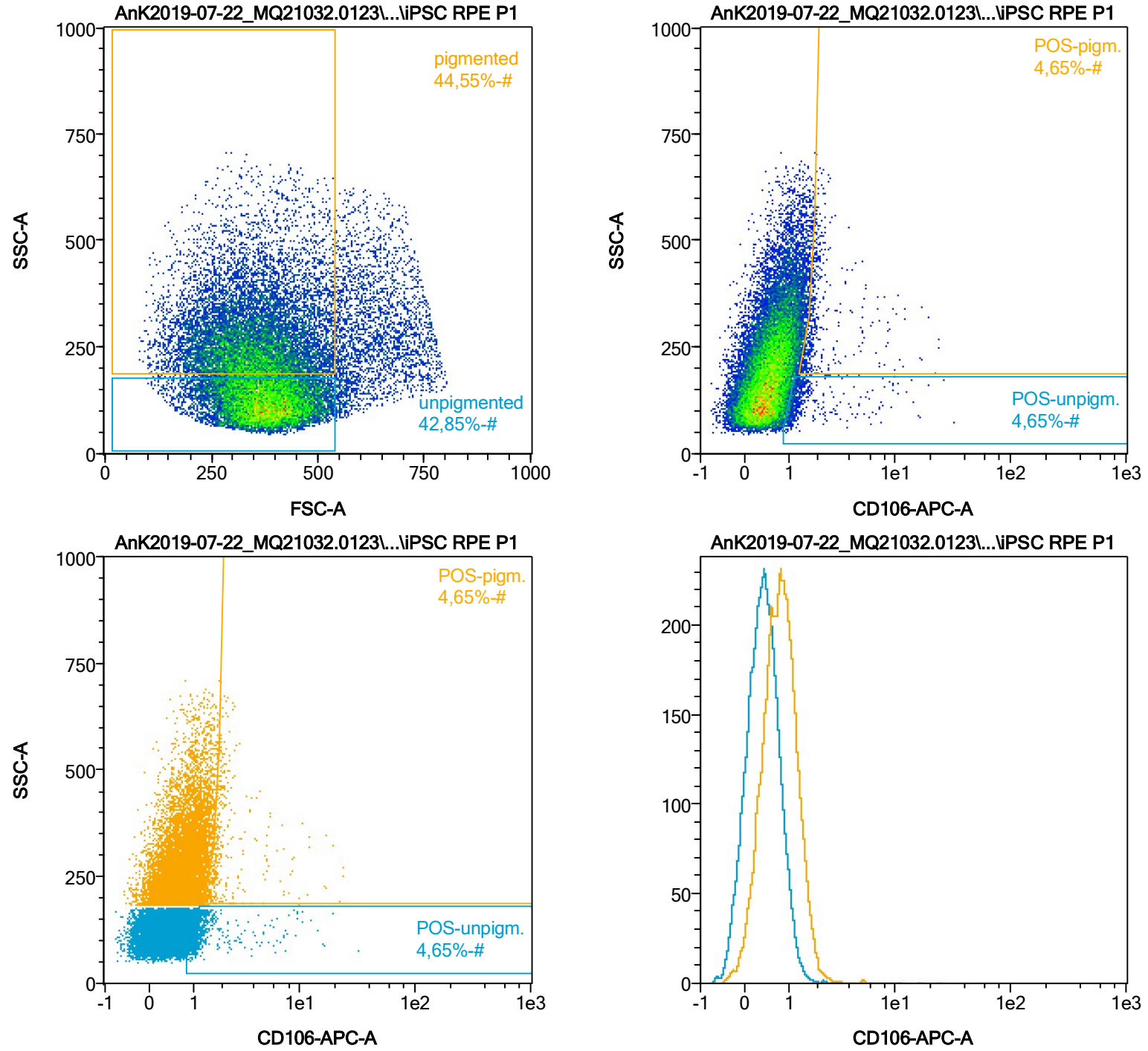

Name	%-#	CD106-APC-A Mean	CD106-APC-A Median
POS-unpigm.	4,65	1,83	1,19
POS-pigm.	4,65	2,43	1,80

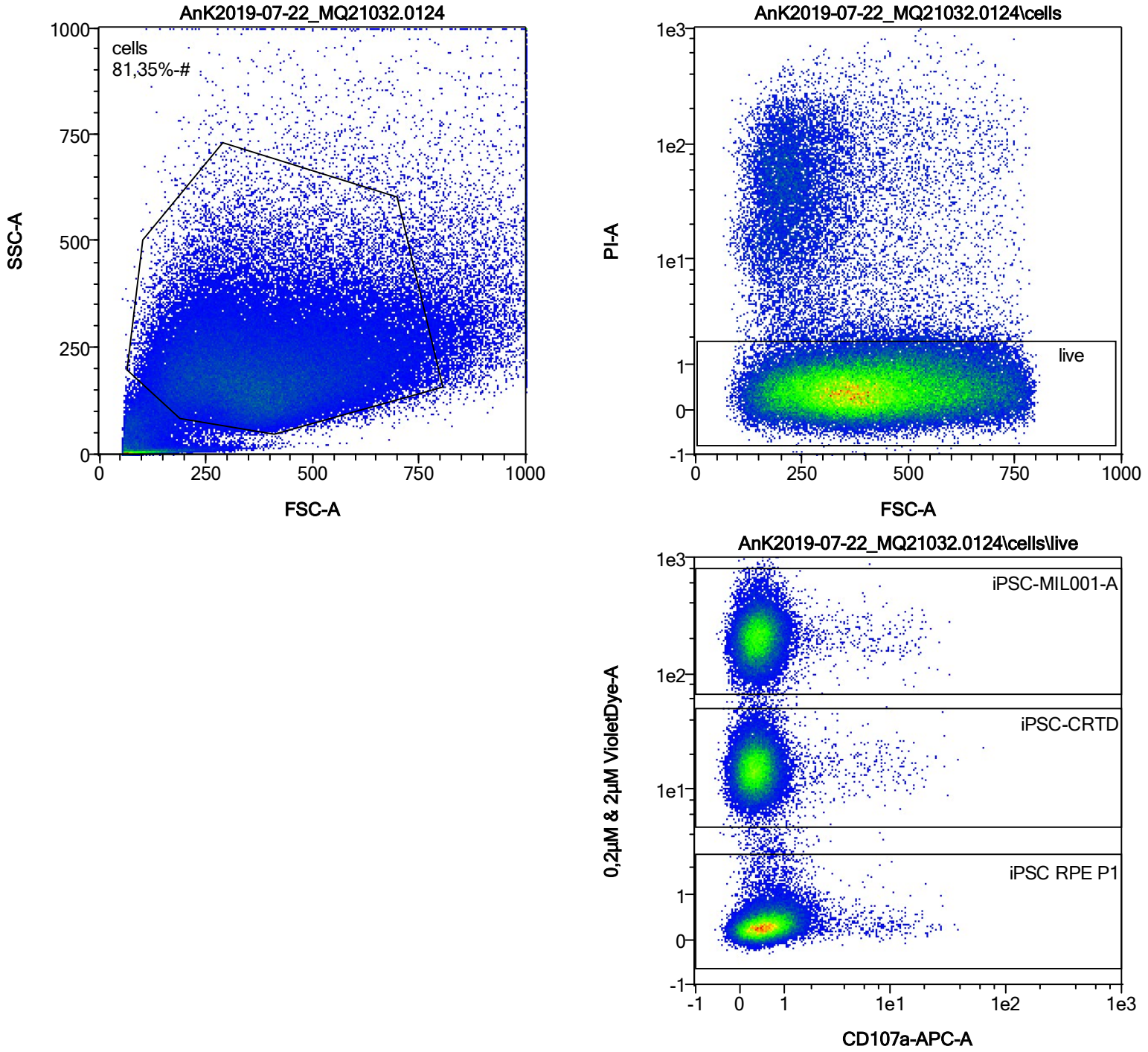

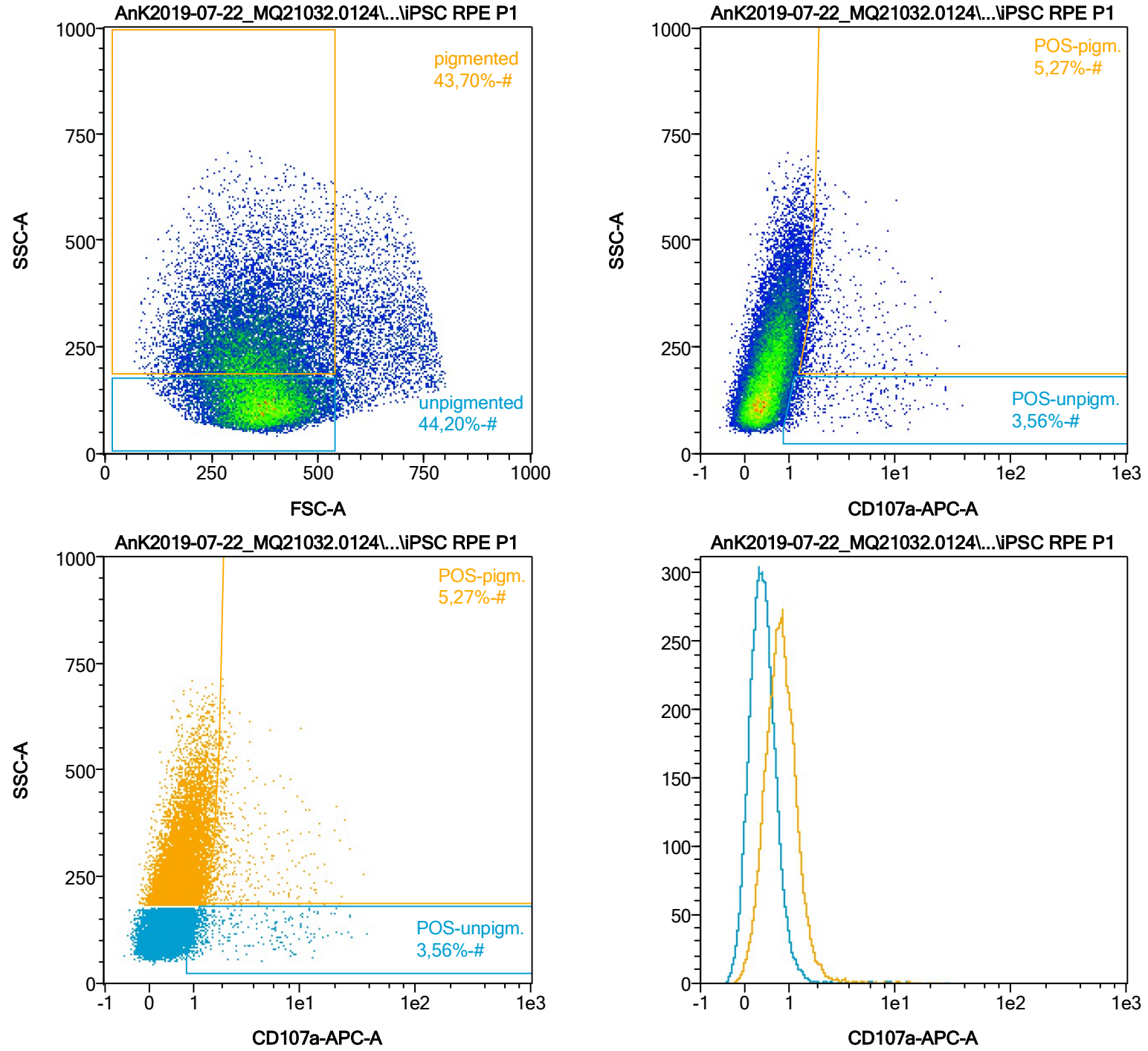

Name	%-#	CD107a-APC-A Mean	CD107a-APC-A Median
POS-unpigm.	3,56	3,39	1,35
POS-pigm.	5,27	3,31	2,00

Date:2024-06-14

Operator: Sebastian Knoebel

Version:2.13.0+54.g95abf0f4

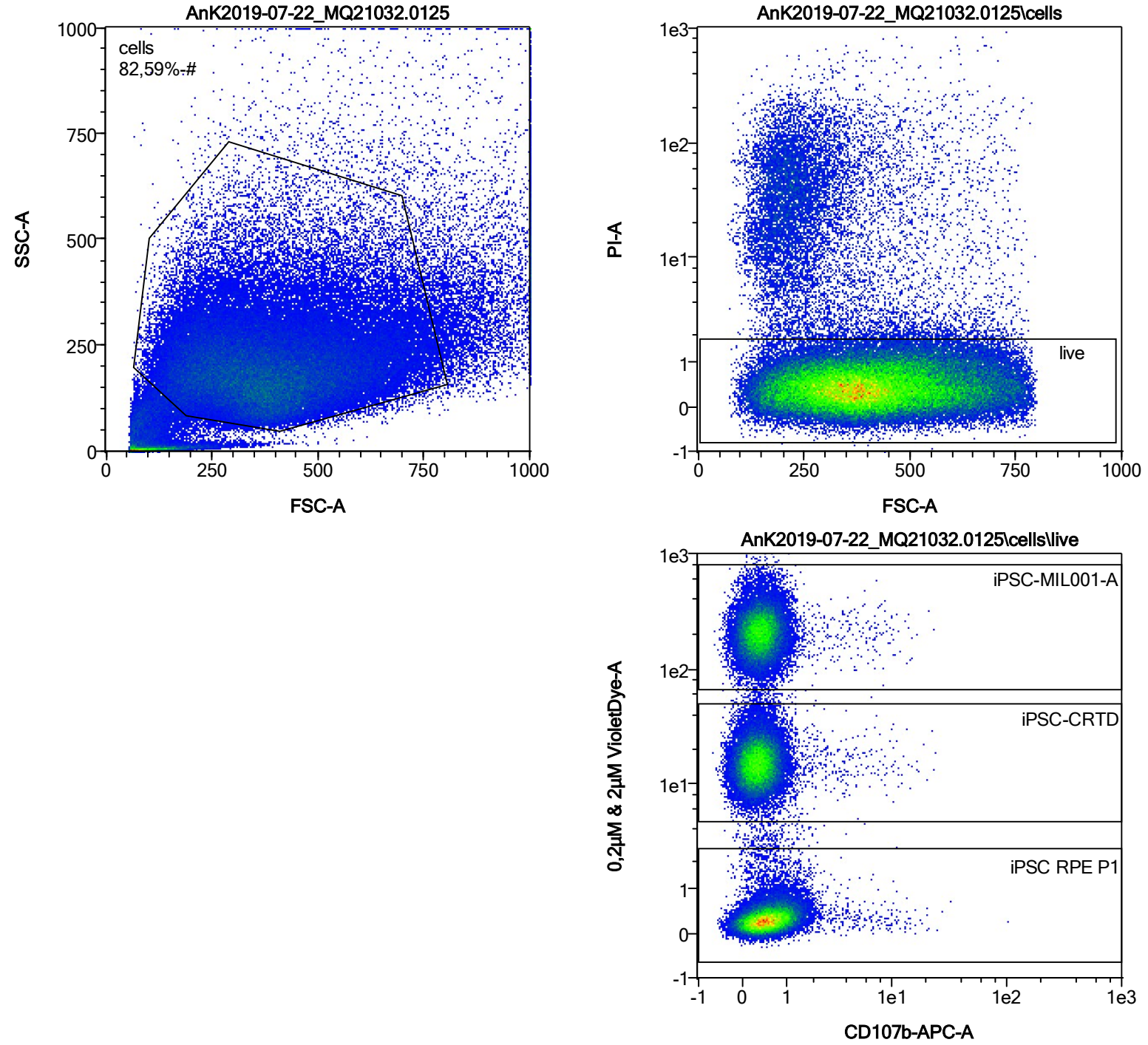

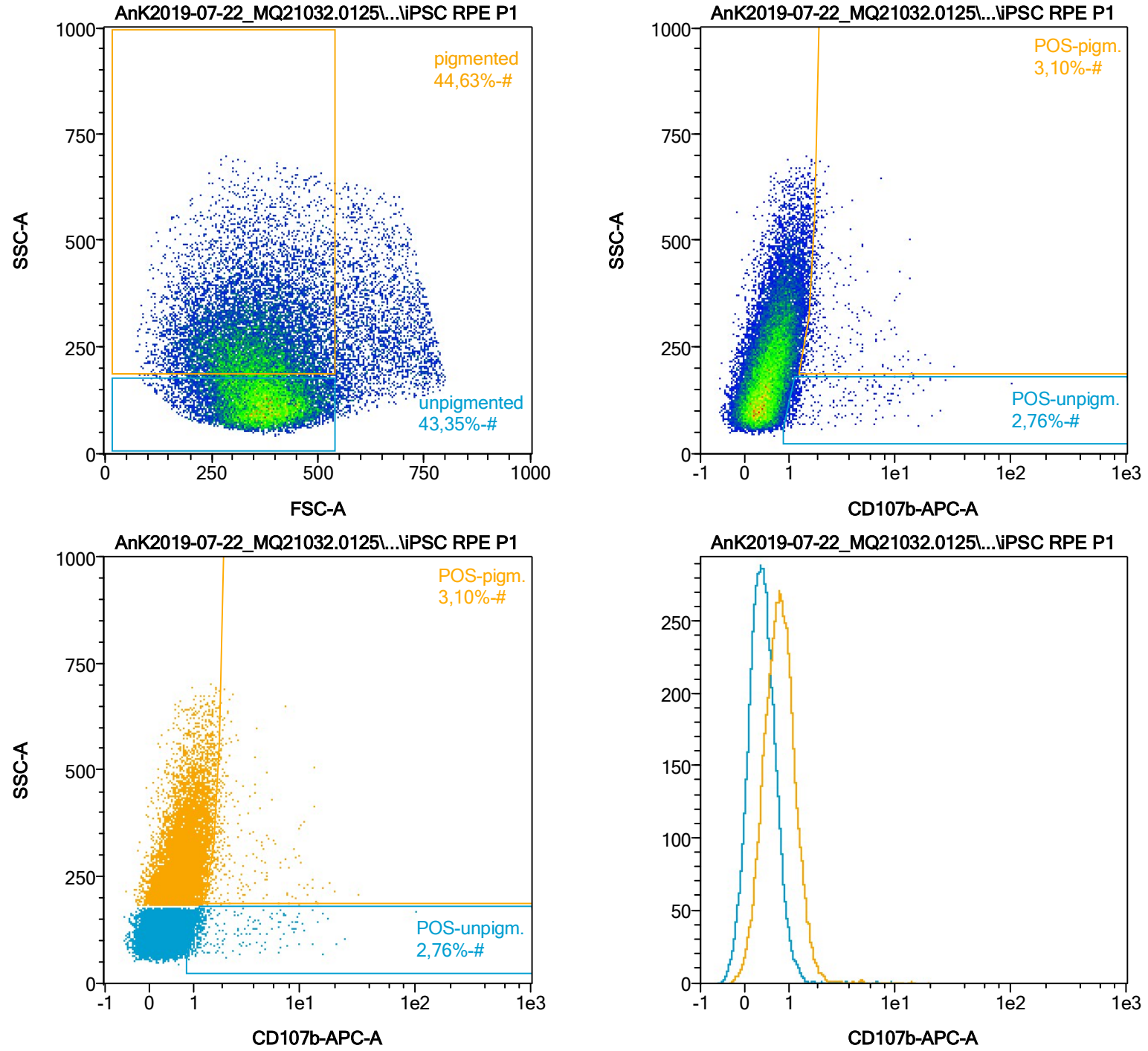

Name	%-#	CD107b-APC-A Mean	CD107b-APC-A Median
POS-unpigm.	2,76	2,95	1,28
POS-pigm.	3,10	3,05	1,95

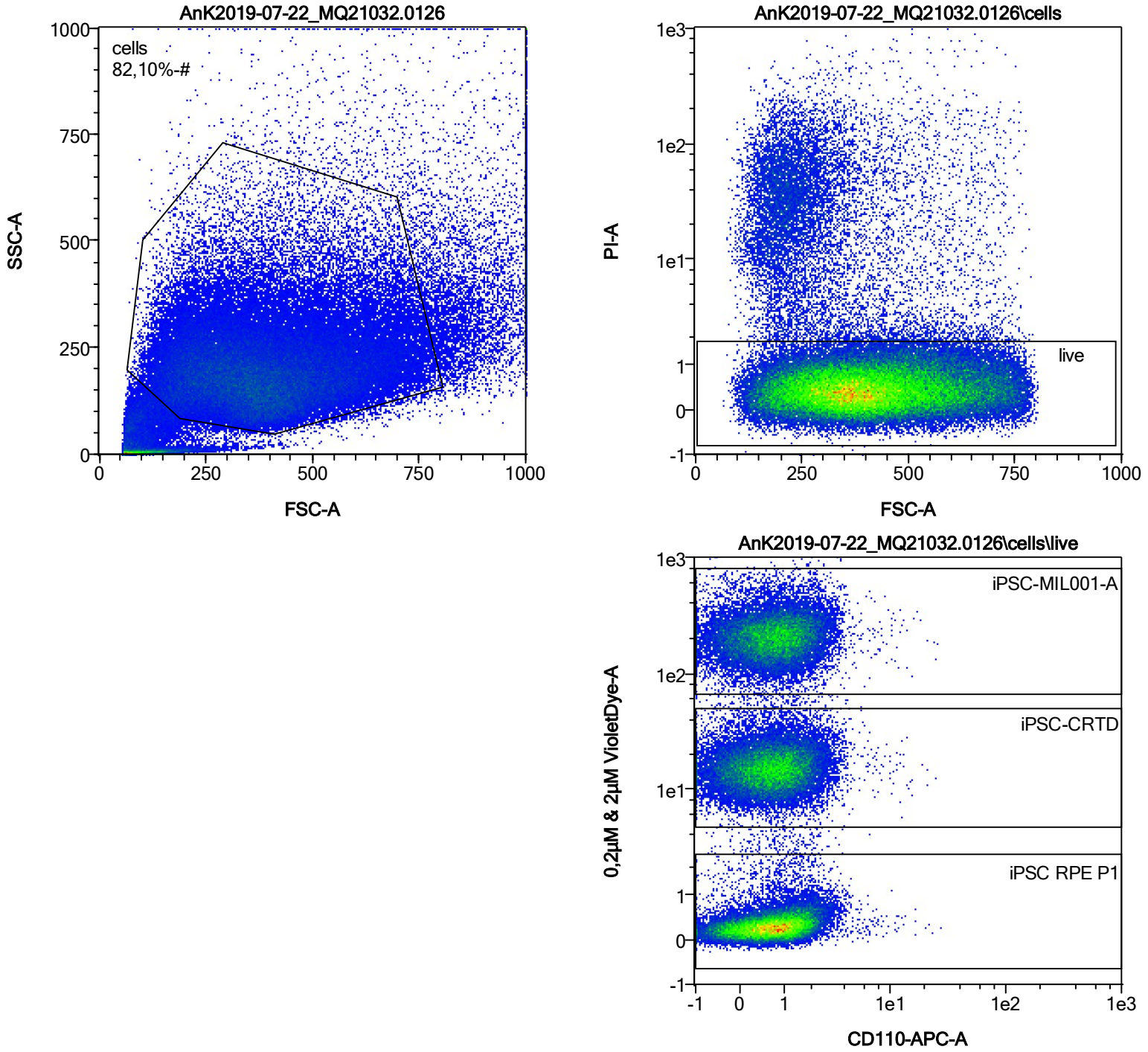

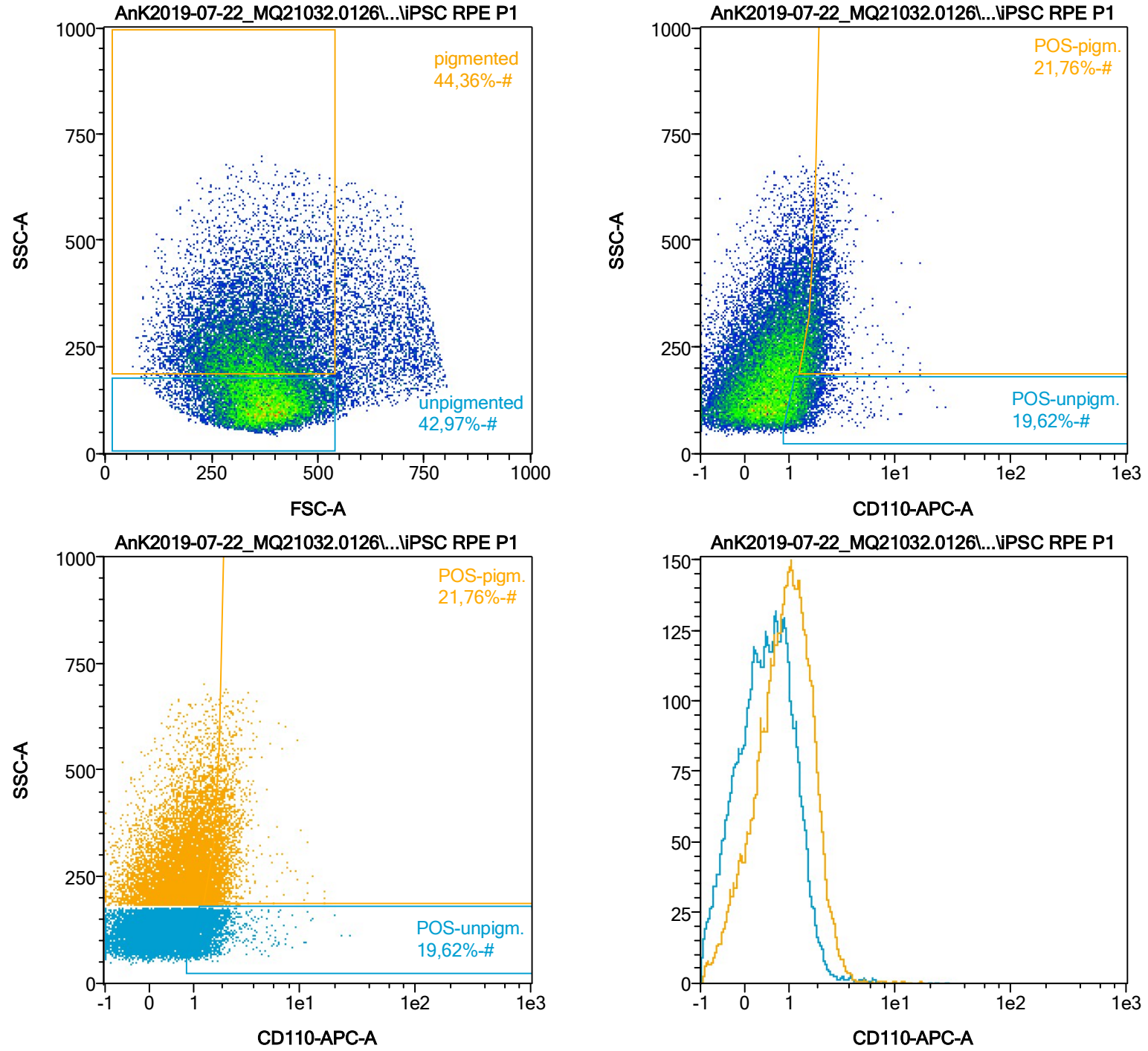

Name	%-#	CD110-APC-A Mean	CD110-APC-A Median
POS-unpigm.	19,62	1,58	1,33
POS-pigm.	21,76	2,11	1,91

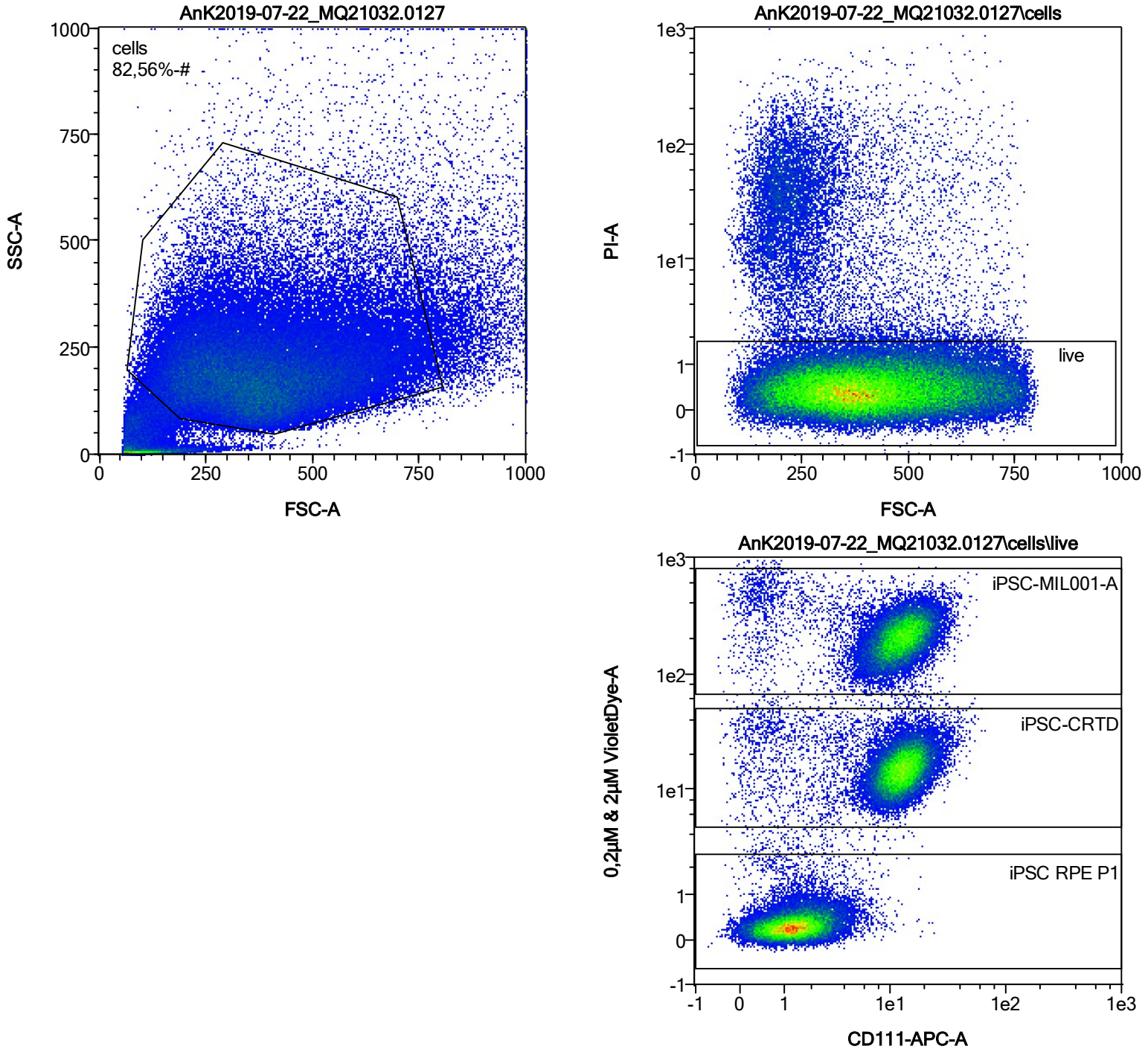

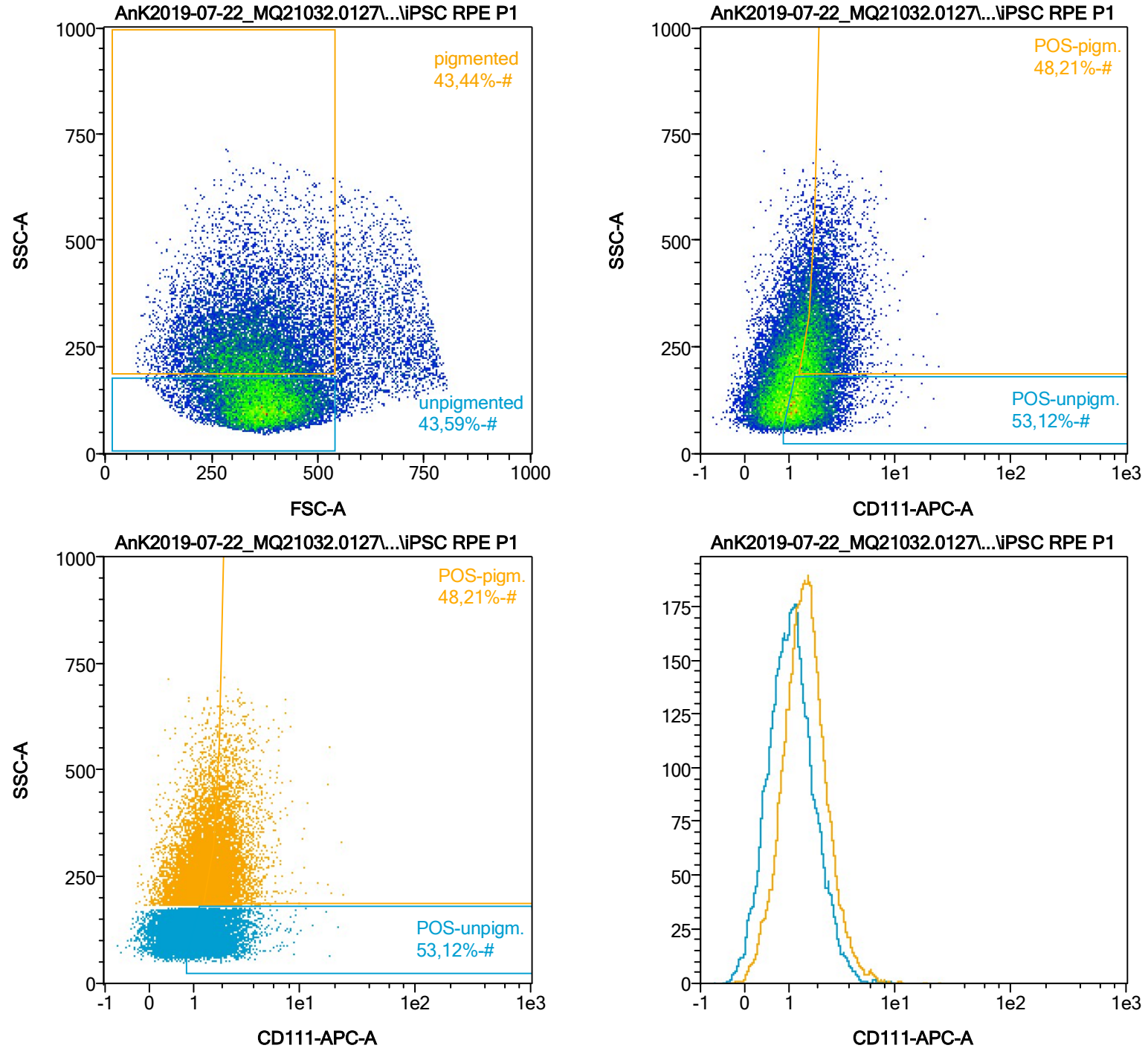

Name	%-#	CD111-APC-A Mean	CD111-APC-A Median
POS-unpigm.	53,12	1,73	1,50
POS-pigm.	48,21	2,33	2,04

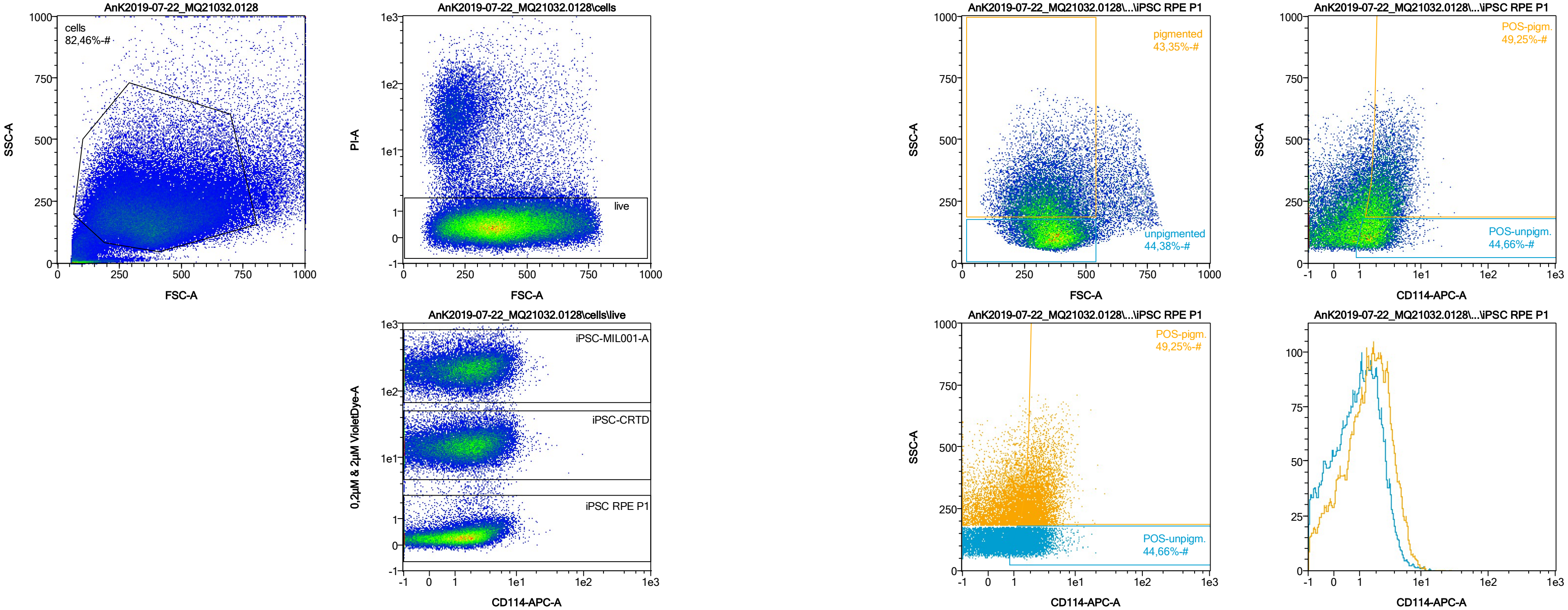

Name	%-#	CD114-APC-A Mean	CD114-APC-A Median
POS-unpigm.	44,66	2,15	1,84
POS-pigm.	49,25	3,02	2,62

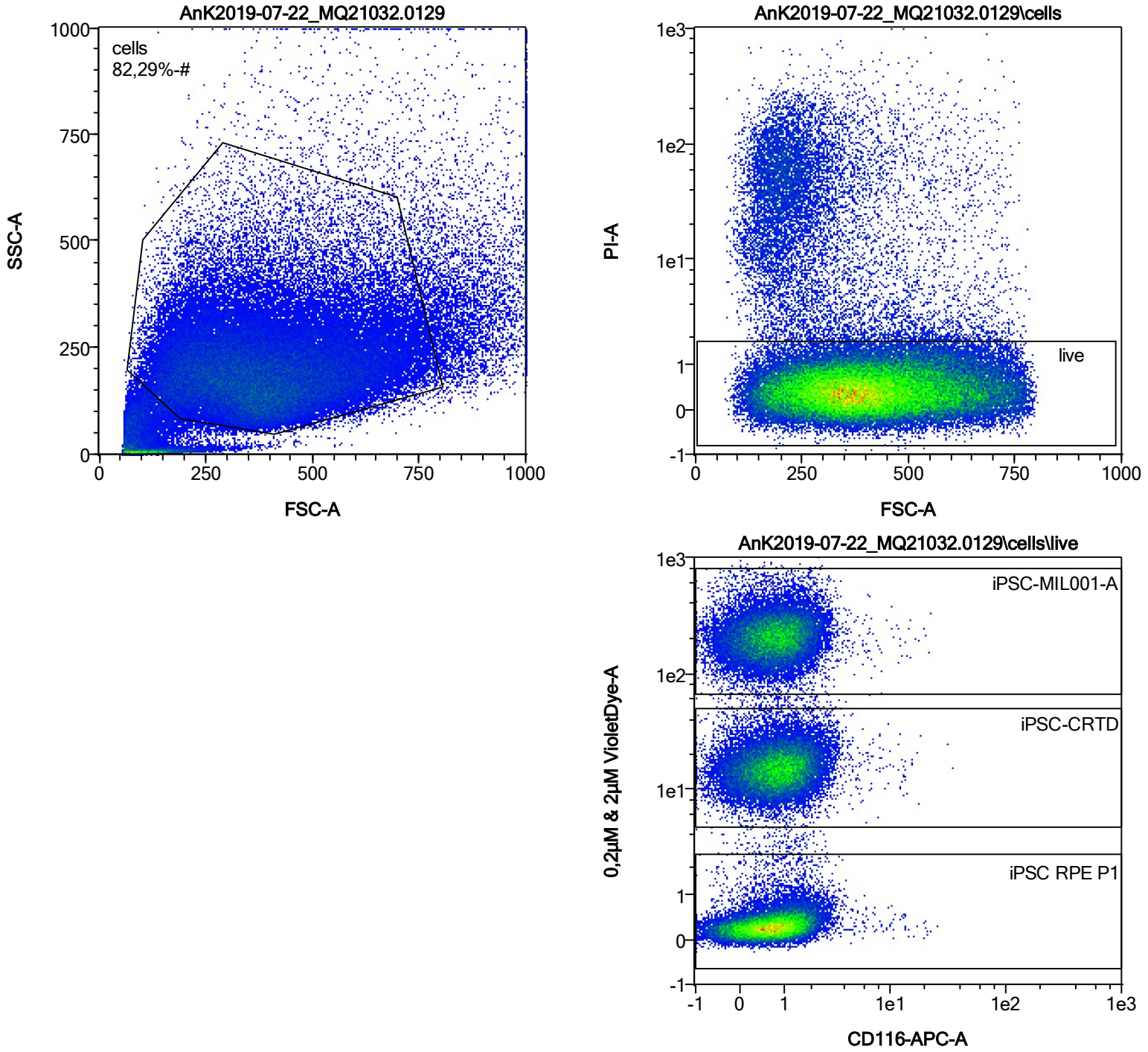

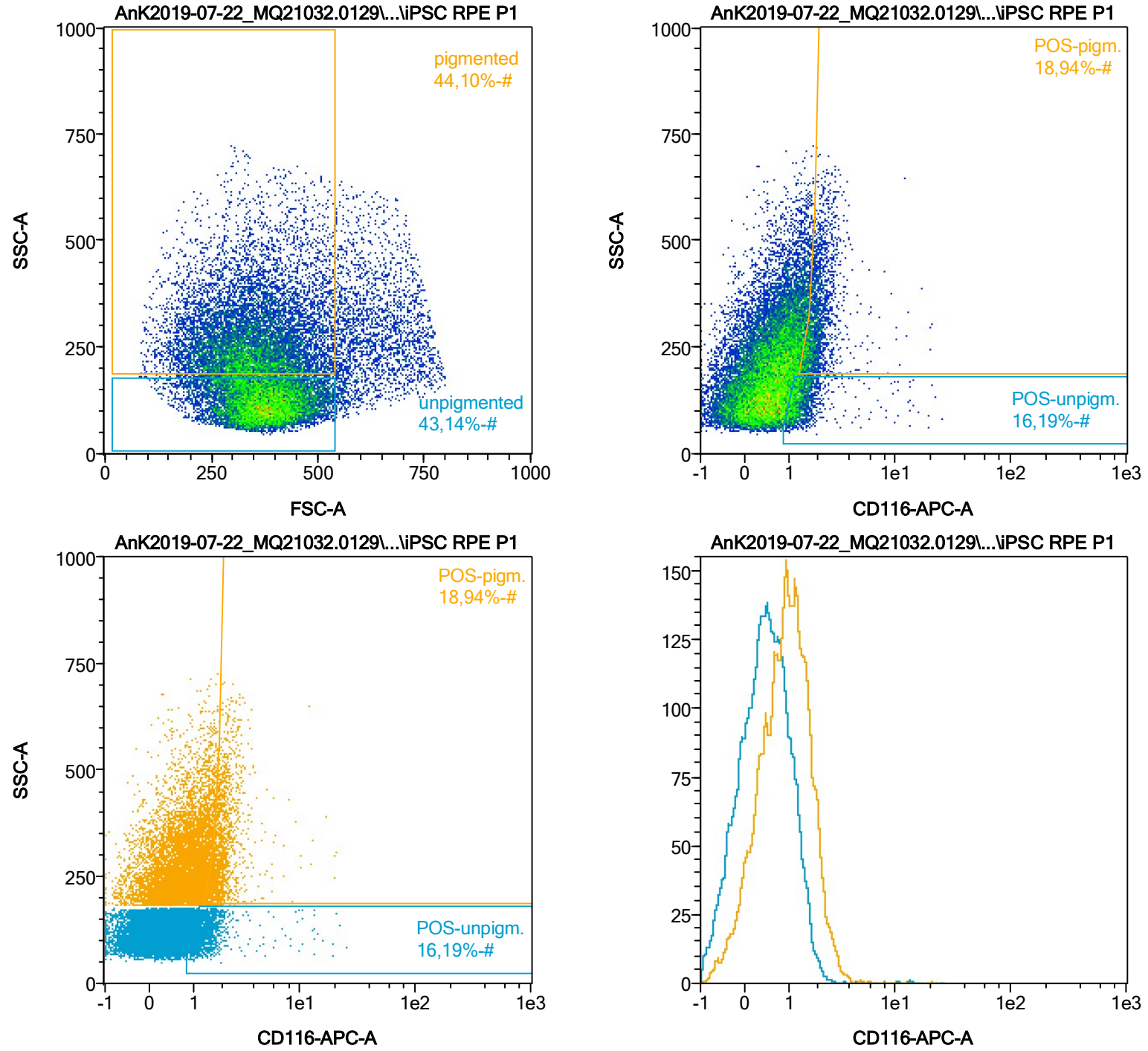

Name	%-#	CD116-APC-A Mean	CD116-APC-A Median
POS-unpigm.	16,19	1,56	1,27
POS-pigm.	18,94	2,08	1,88

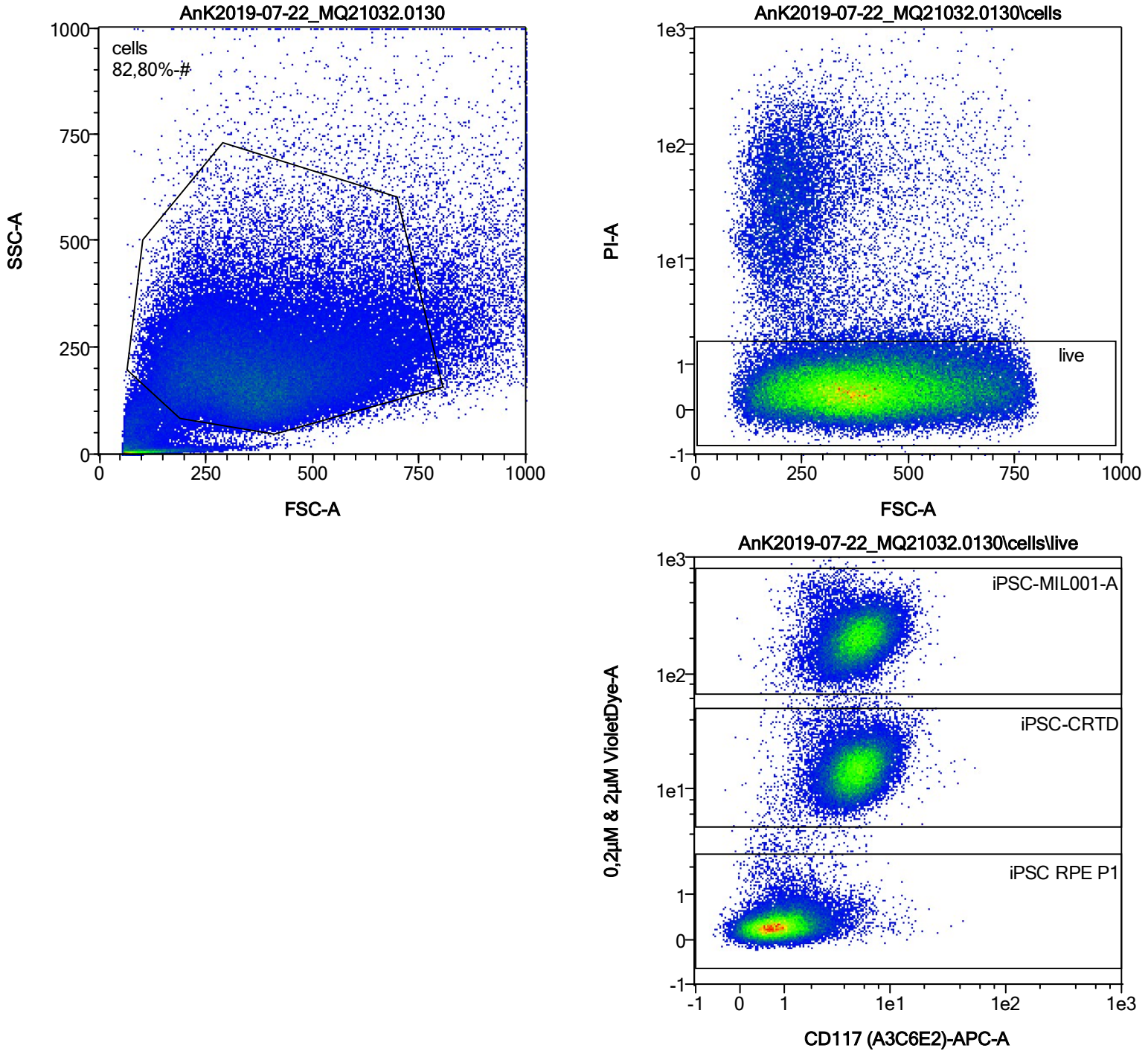

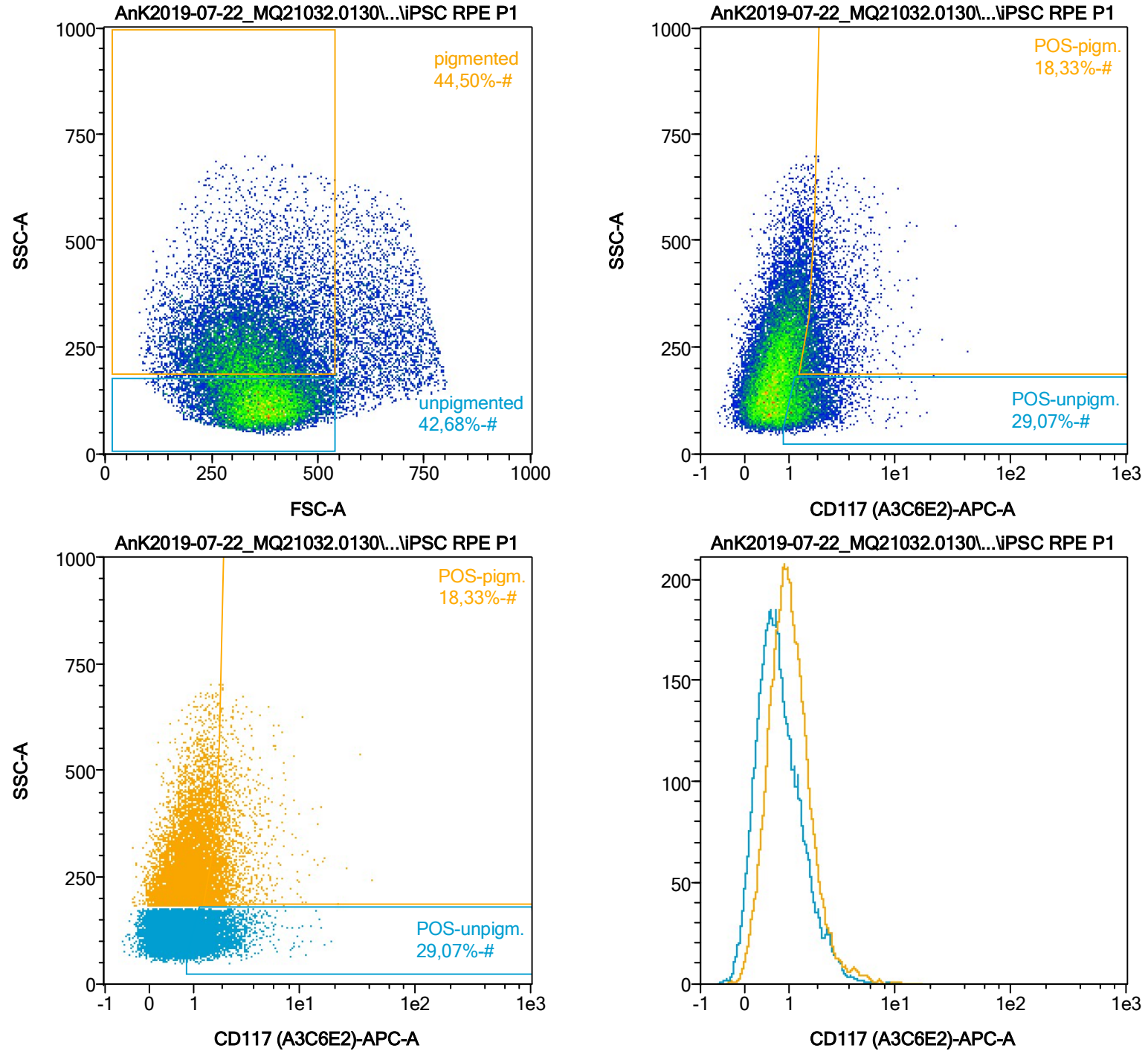

Name	%-#	CD117 (A3C6E2)-APC-A Mean	CD117 (A3C6E2)-APC-A Median
POS-unpigm.	29,07	1,74	1,45
POS-pigm.	18,33	2,46	1,98

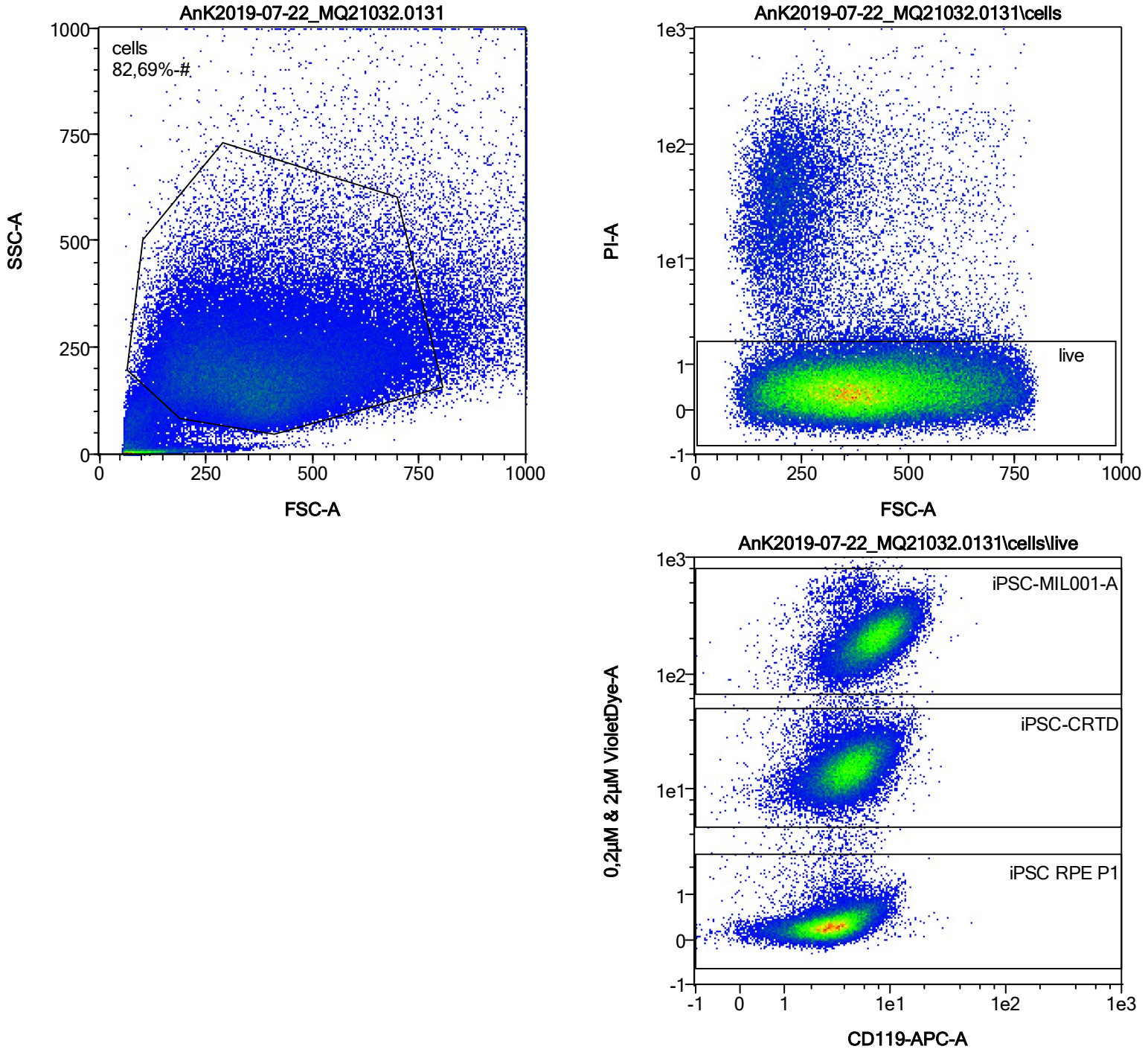

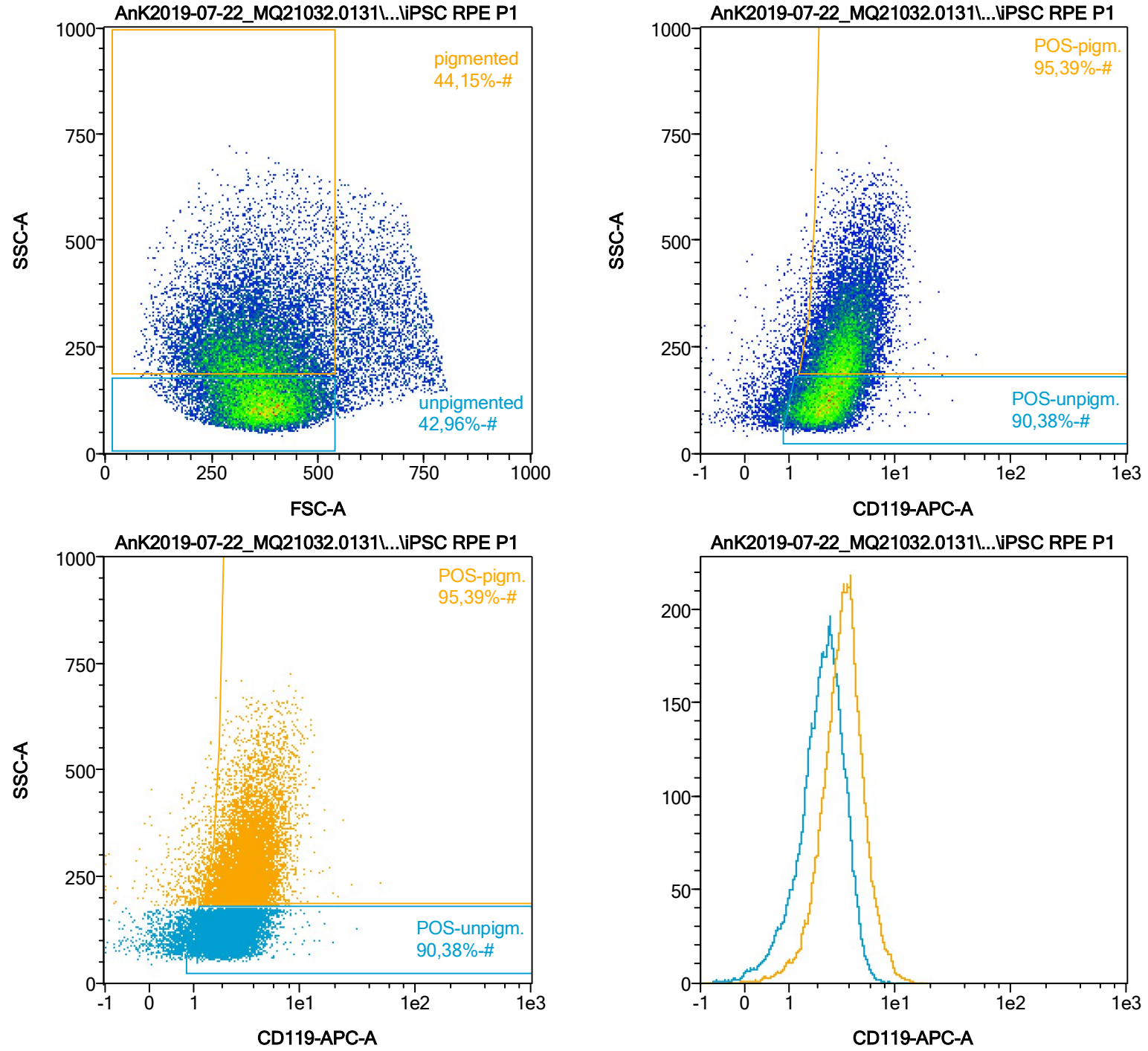

Name	%-#	CD119-APC-A Mean	CD119-APC-A Median
POS-unpigm.	90,38	2,59	2,43
POS-pigm.	95,39	3,85	3,56

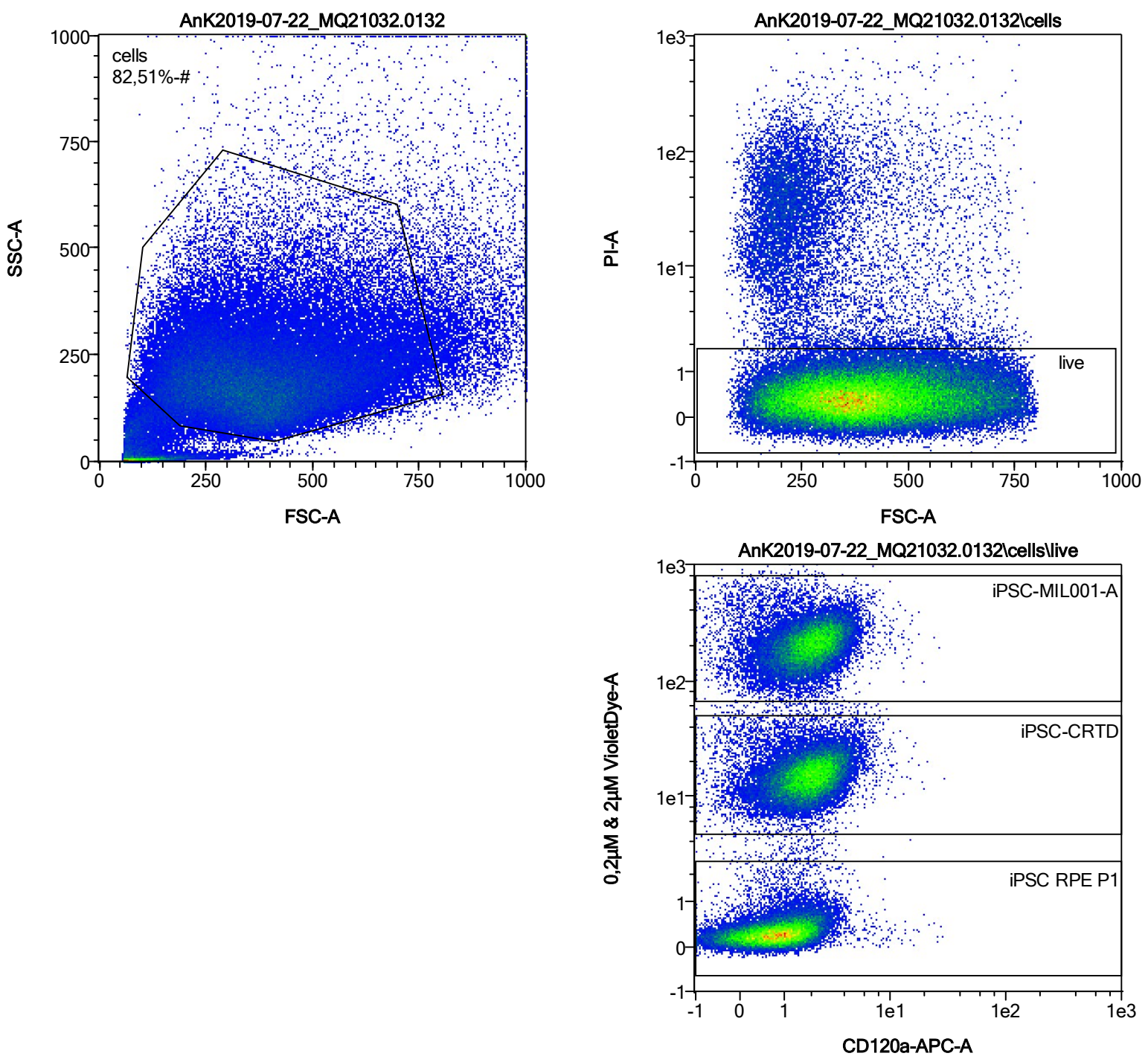

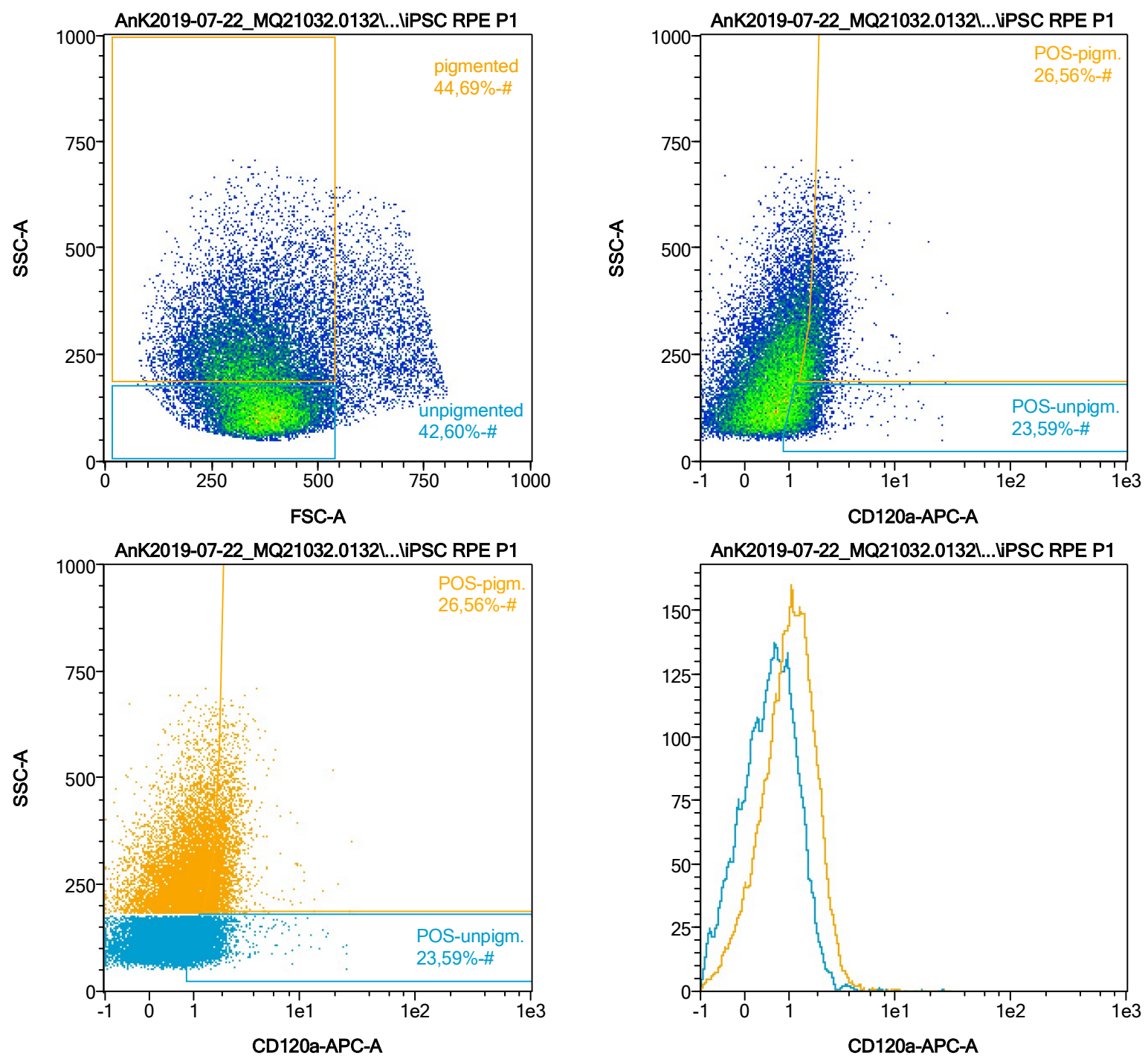

Name	%-#	CD120a-APC-A Mean	CD120a-APC-A Median
POS-unpigm.	23,59	1,60	1,36
POS-pigm.	26,56	2,15	1,94

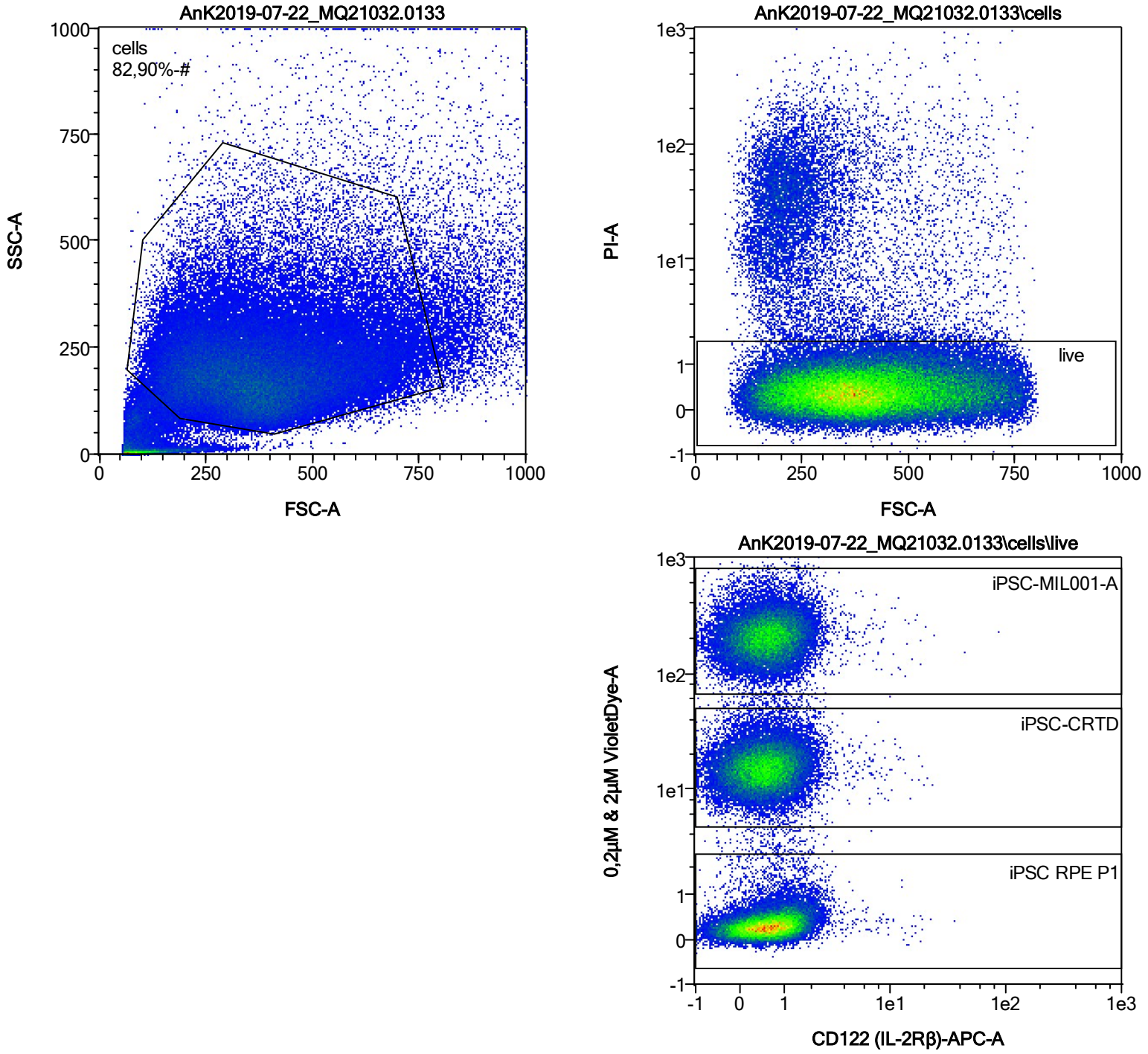

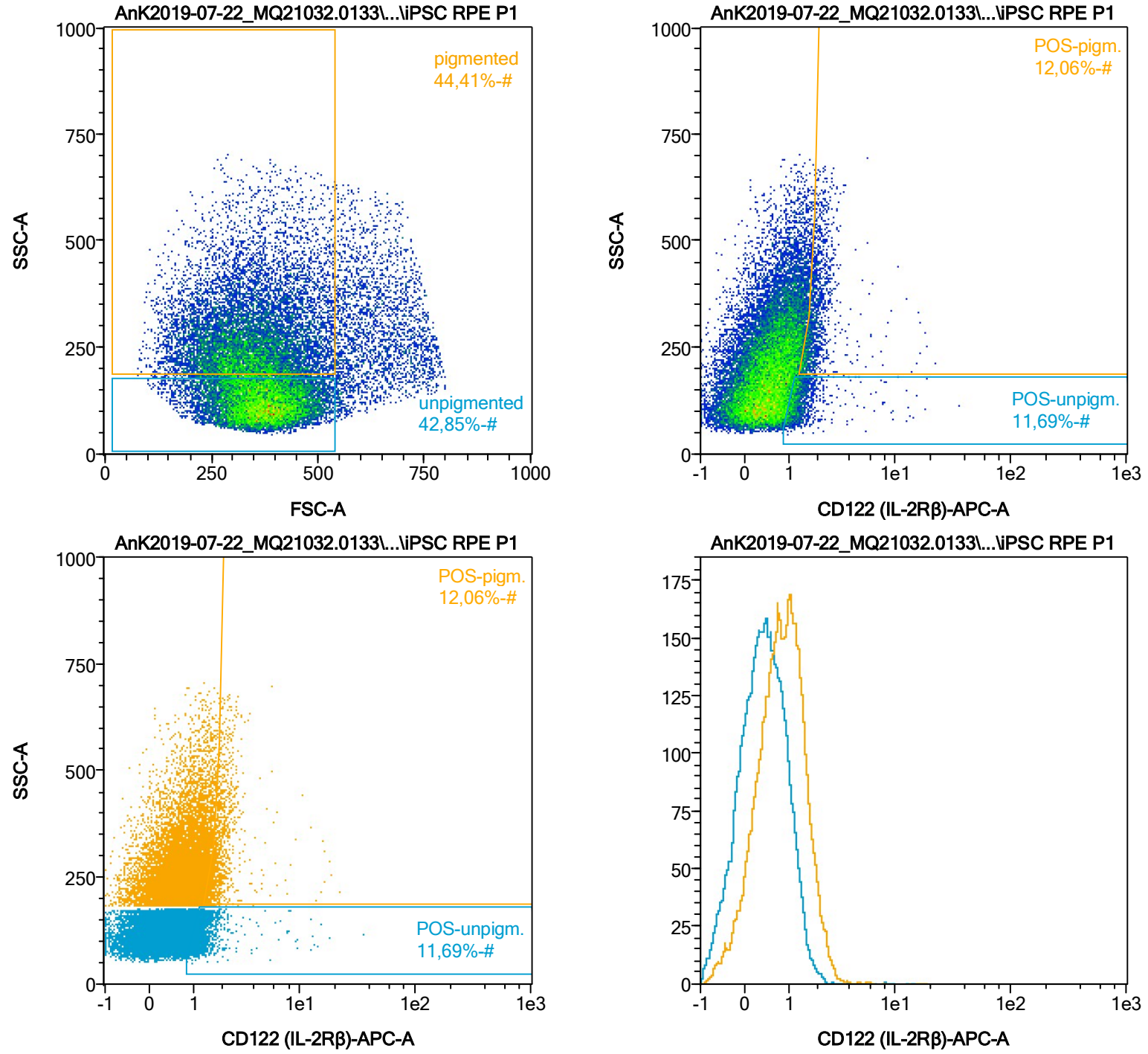

Name	%-#	CD122 (IL-2Rβ)-APC-A Mean	CD122 (IL-2Rβ)-APC-A Median
POS-unpigm.	11,69	1,48	1,24
POS-pigm.	12,06	2,07	1,84

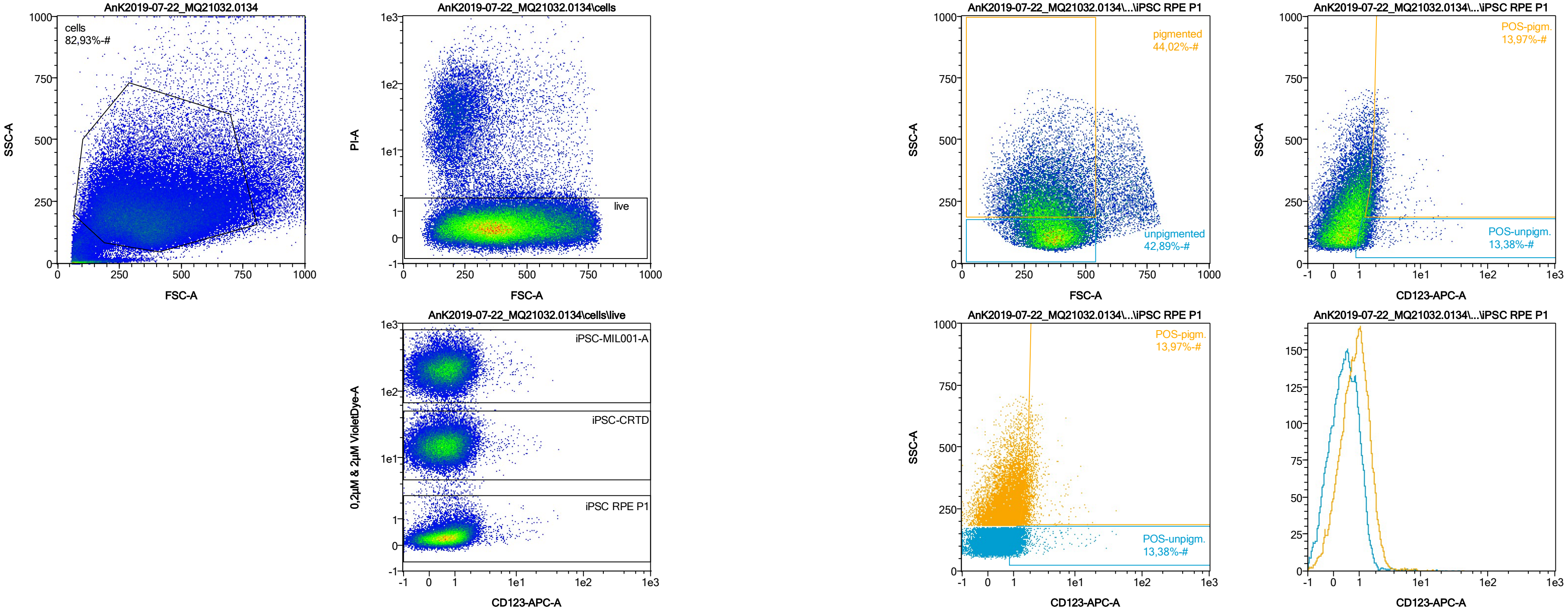

Name	%-#	CD123-APC-A Mean	CD123-APC-A Median
POS-unpigm.	13,38	1,67	1,25
POS-pigm.	13,97	2,15	1,86

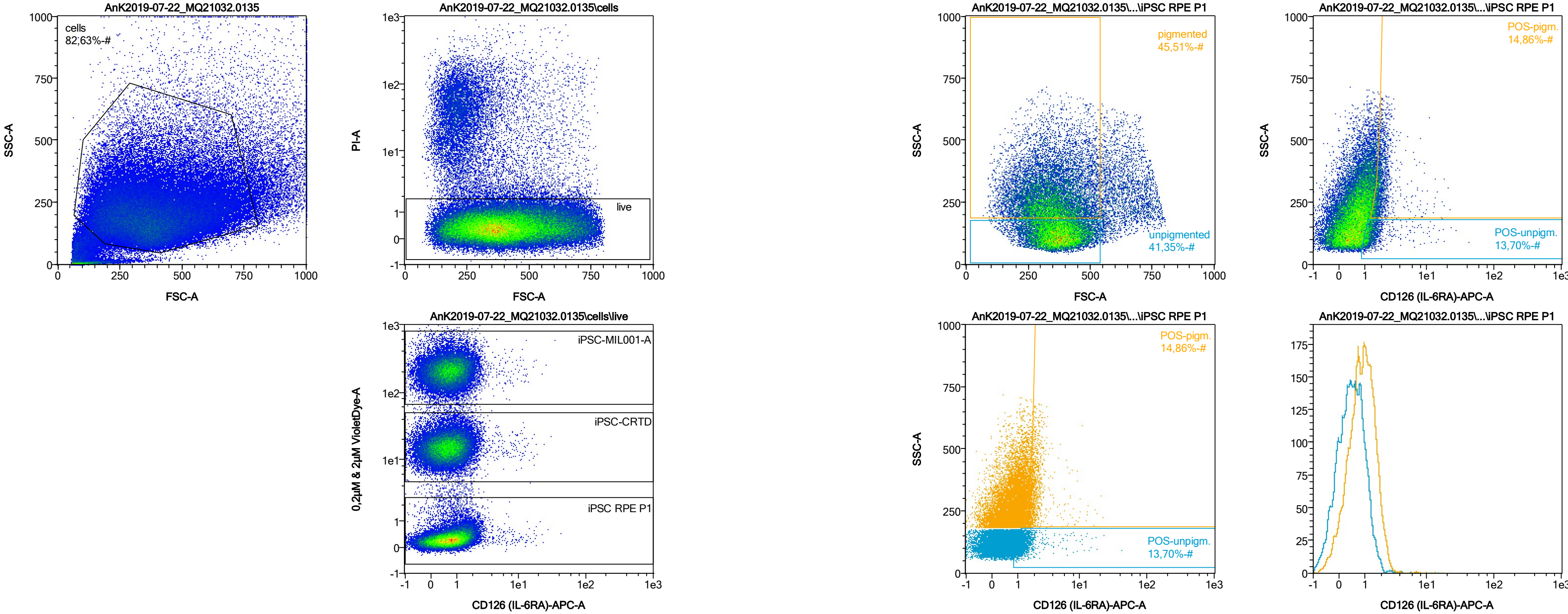

Name	%-#	CD126 (IL-6RA)-APC-A Mean	CD126 (IL-6RA)-APC-A Median
POS-unpigm.	13,70	1,56	1,24
POS-pigm.	14,86	2,11	1,86

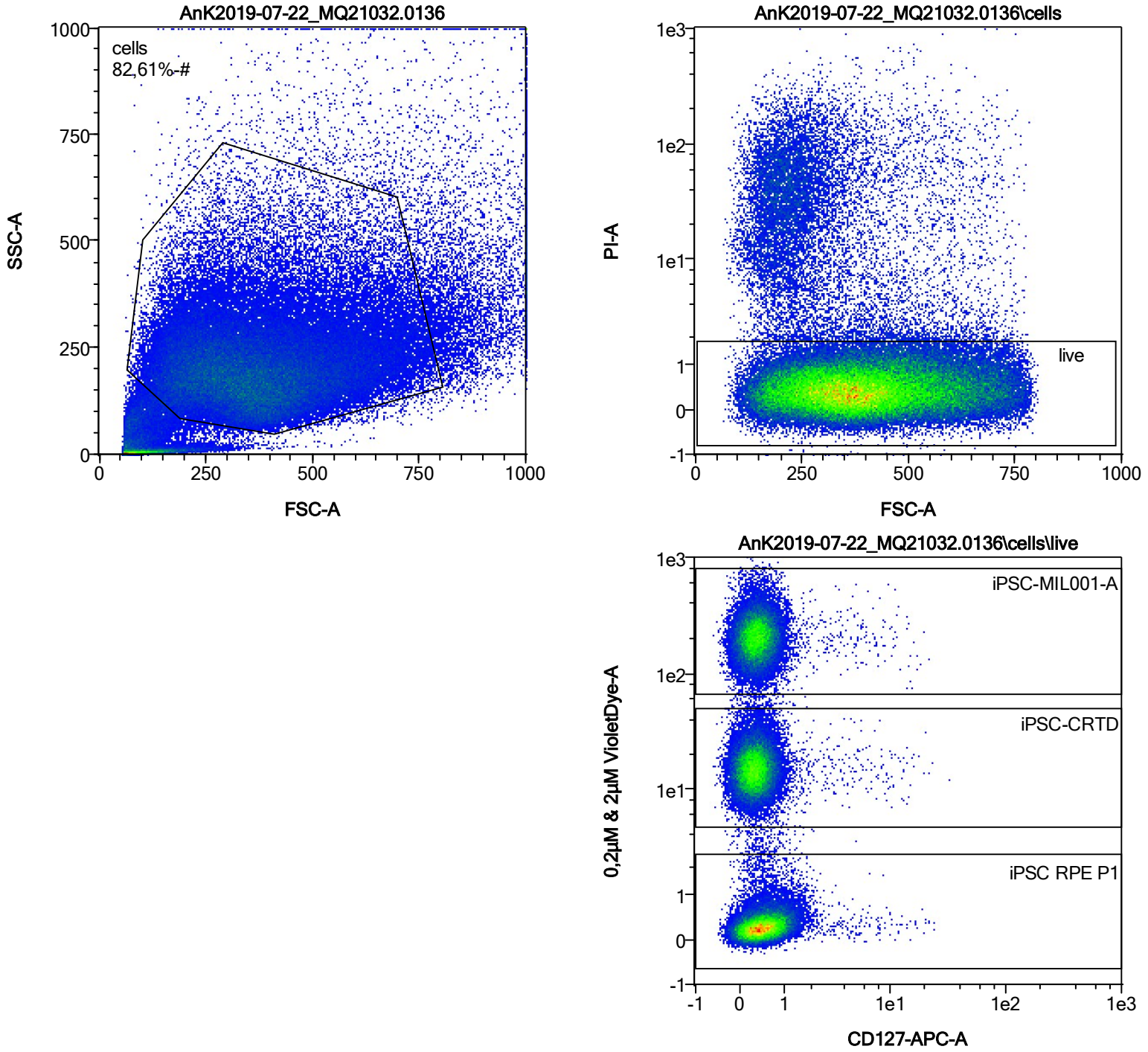

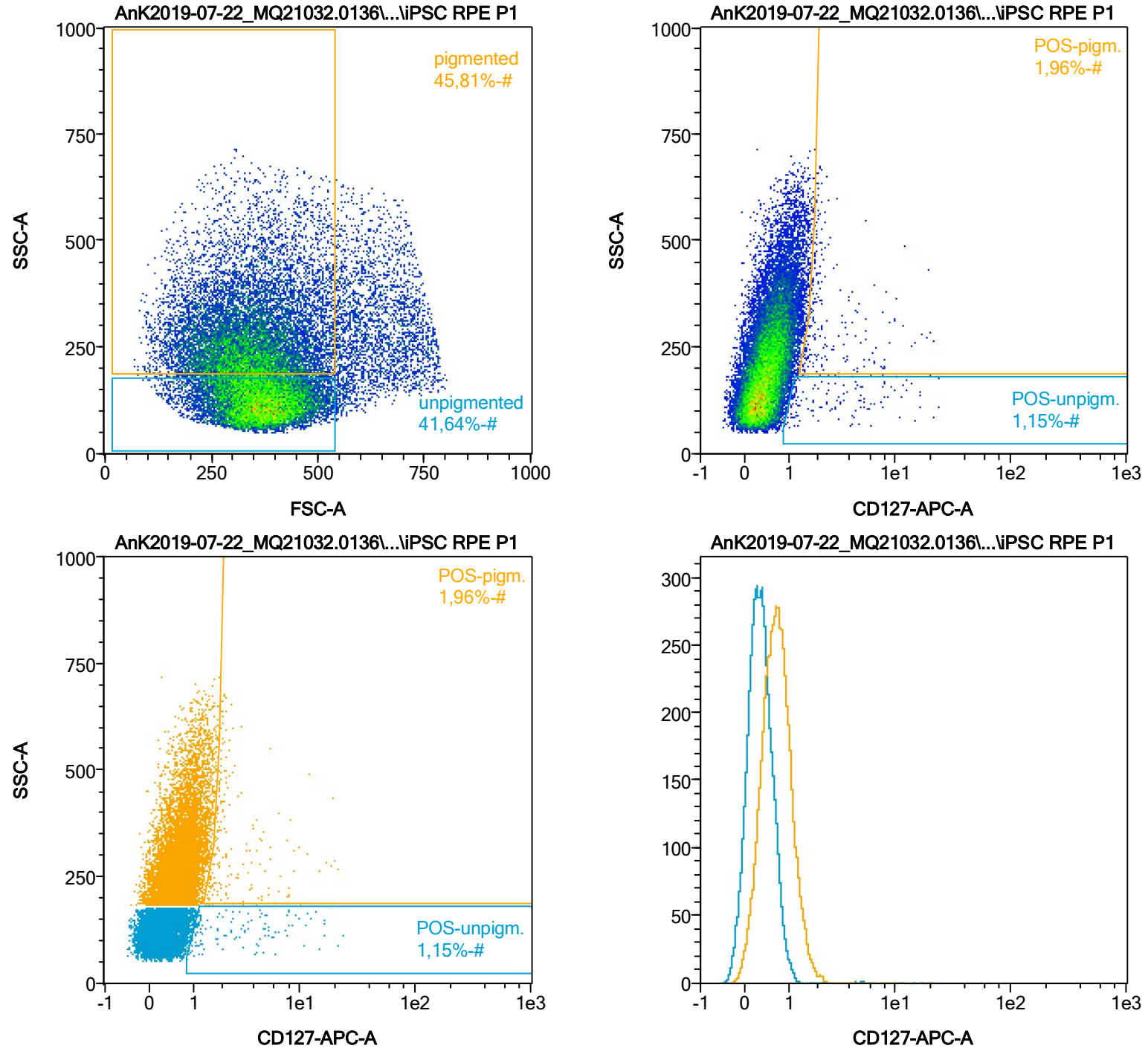

Name	%-#	CD127-APC-A Mean	CD127-APC-A Median
POS-unpigm.	1,15	4,00	2,22
POS-pigm.	1,96	3,20	1,99

Date:2024-06-14

Operator: Sebastian Knoebel

Version:2.13.0+54.g95abf0f4

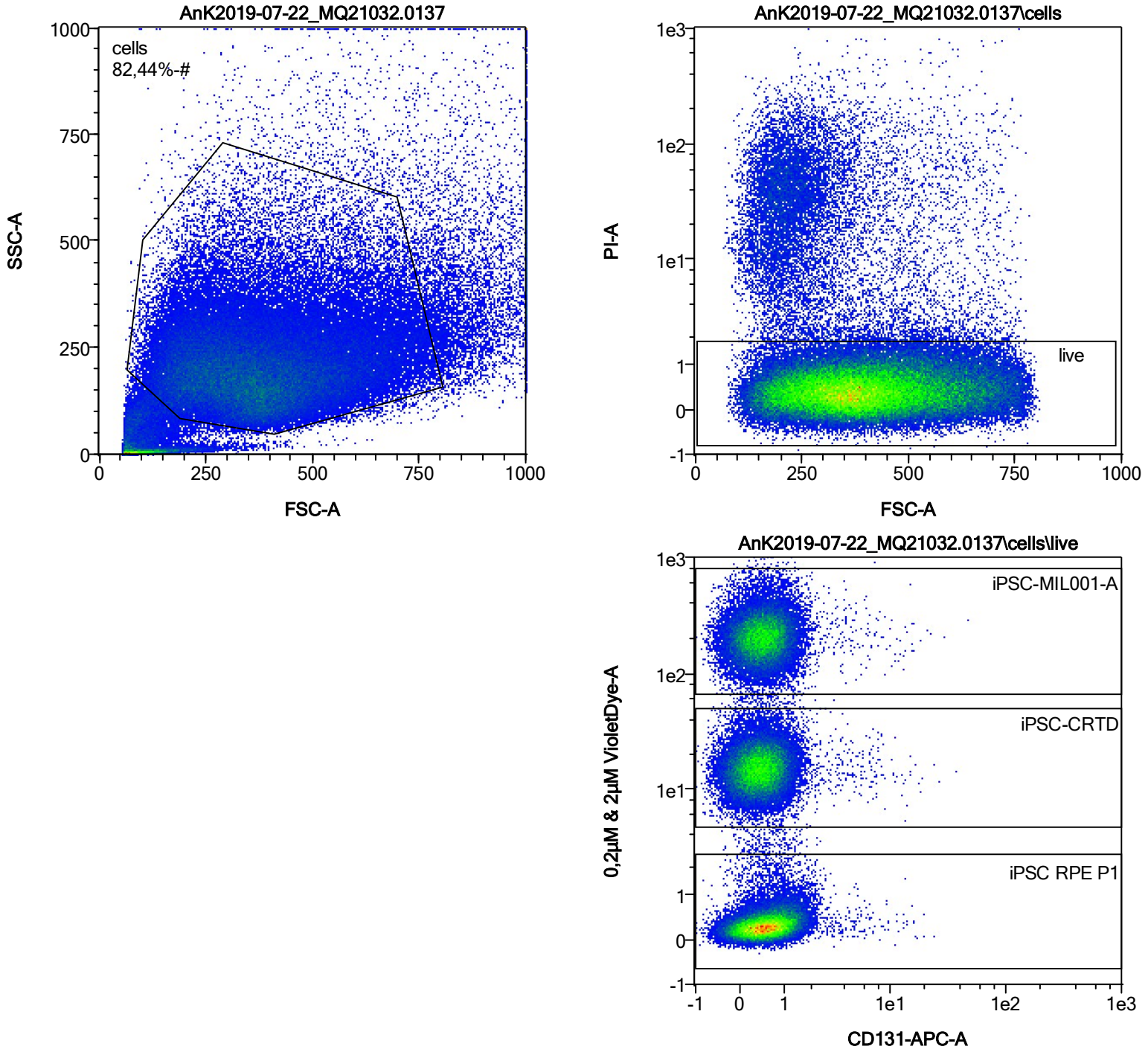

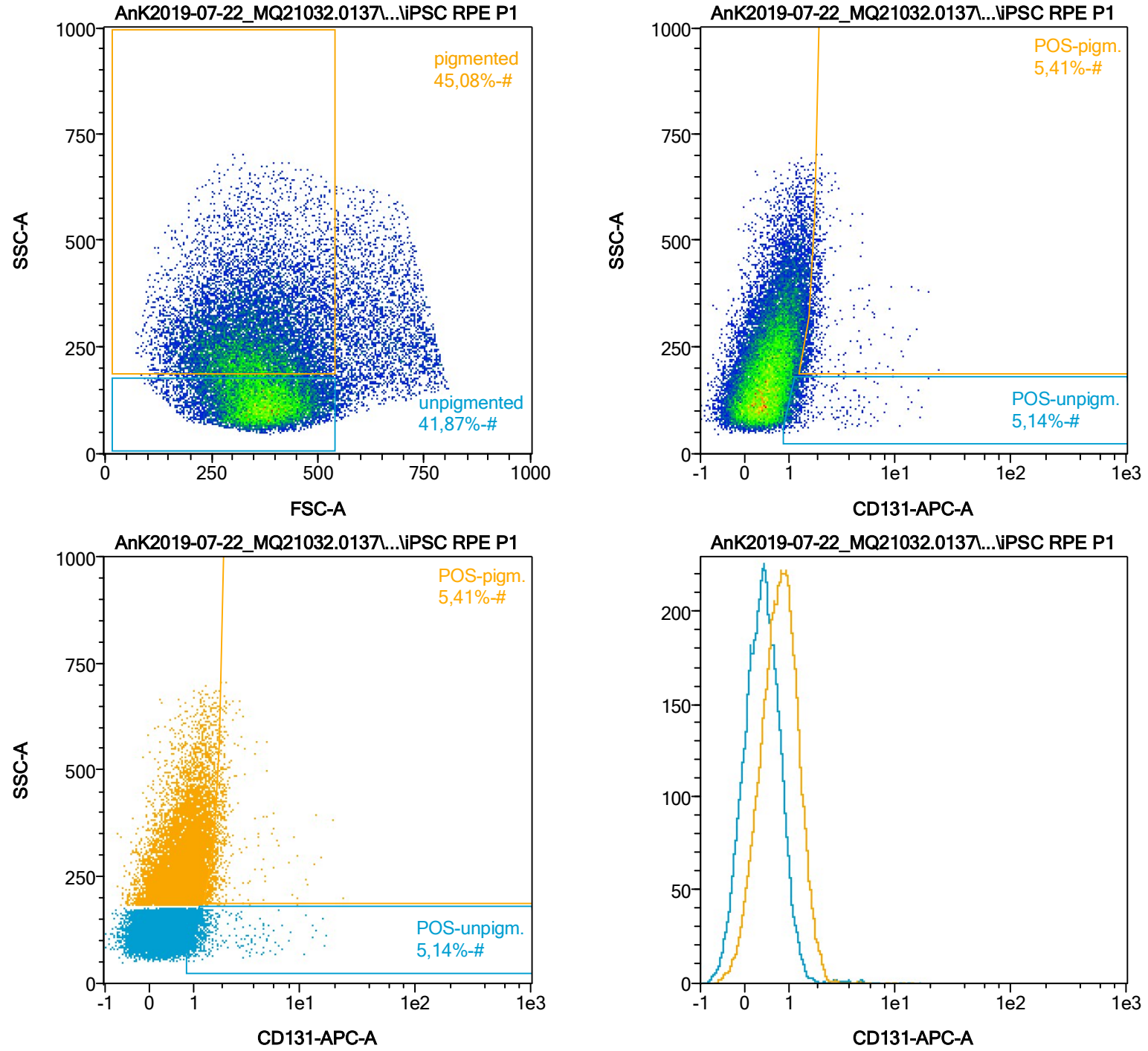

Name	%-#	CD131-APC-A Mean	CD131-APC-A Median
POS-unpigm.	5,14	1,72	1,22
POS-pigm.	5,41	2,17	1,80

Date:2024-06-14

Operator: Sebastian Knoebel

Version:2.13.0+54.g95abf0f4

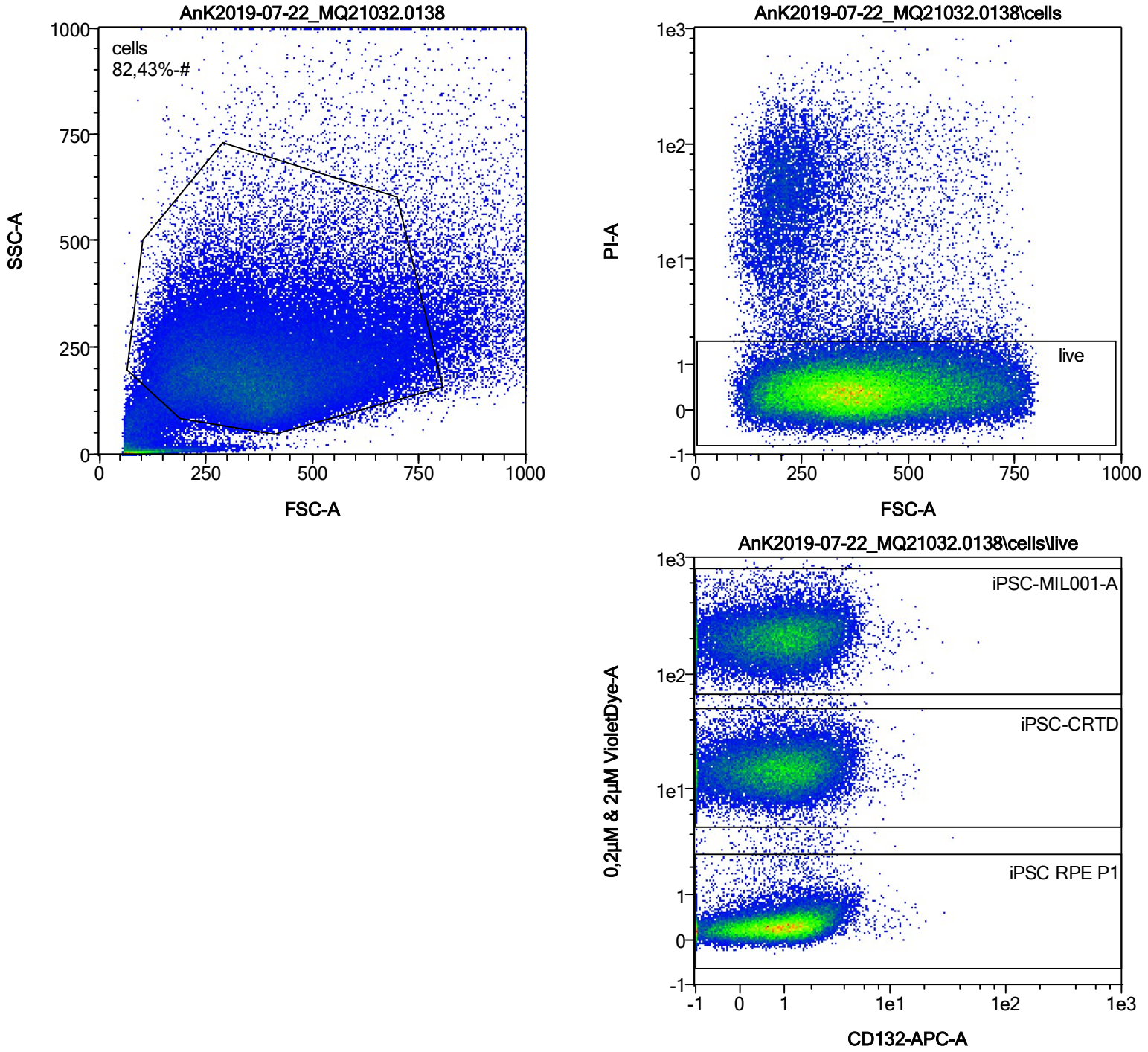

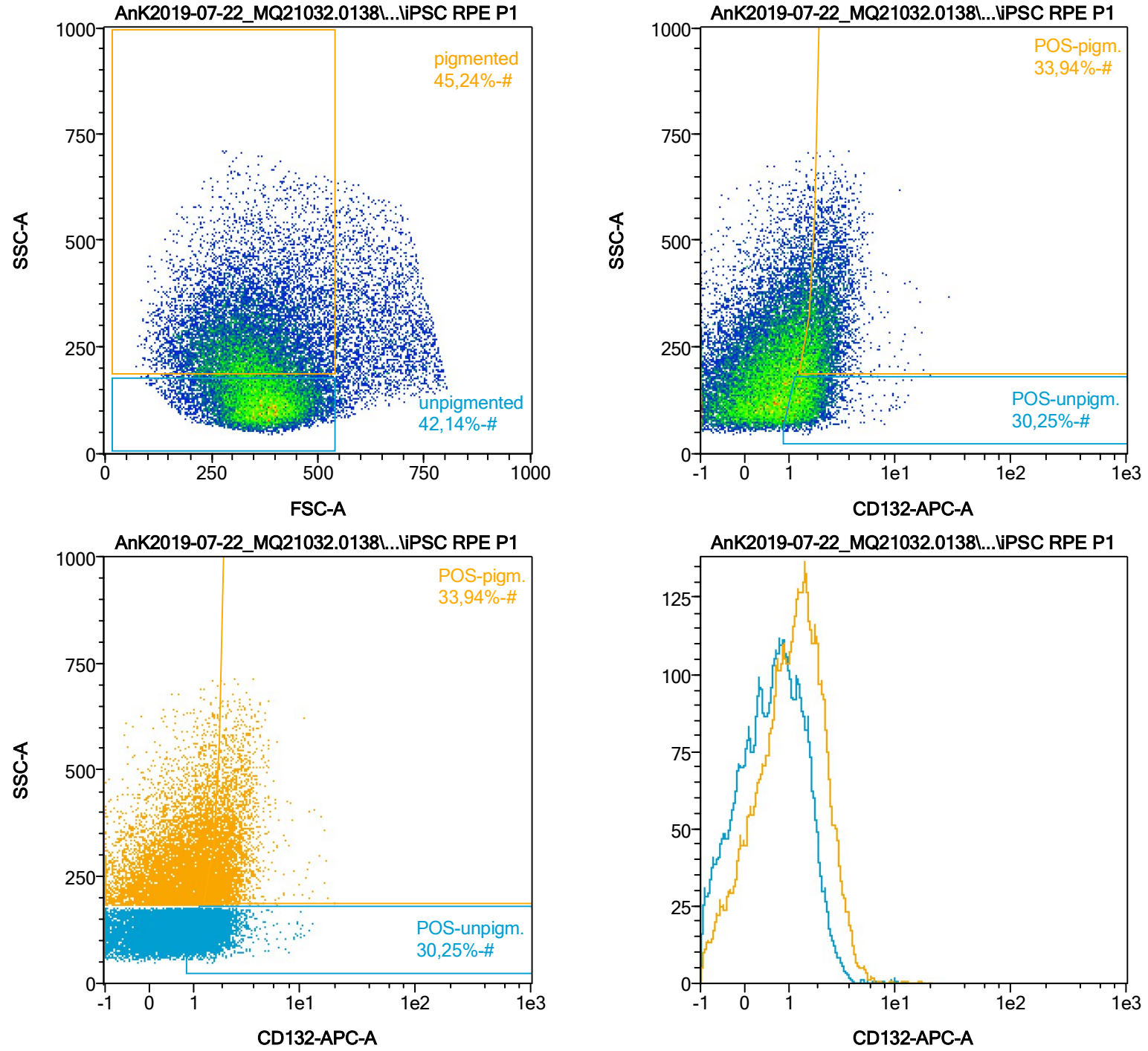

Name	%-#	CD132-APC-A Mean	CD132-APC-A Median
POS-unpigm.	30,25	1,69	1,50
POS-pigm.	33,94	2,37	2,11

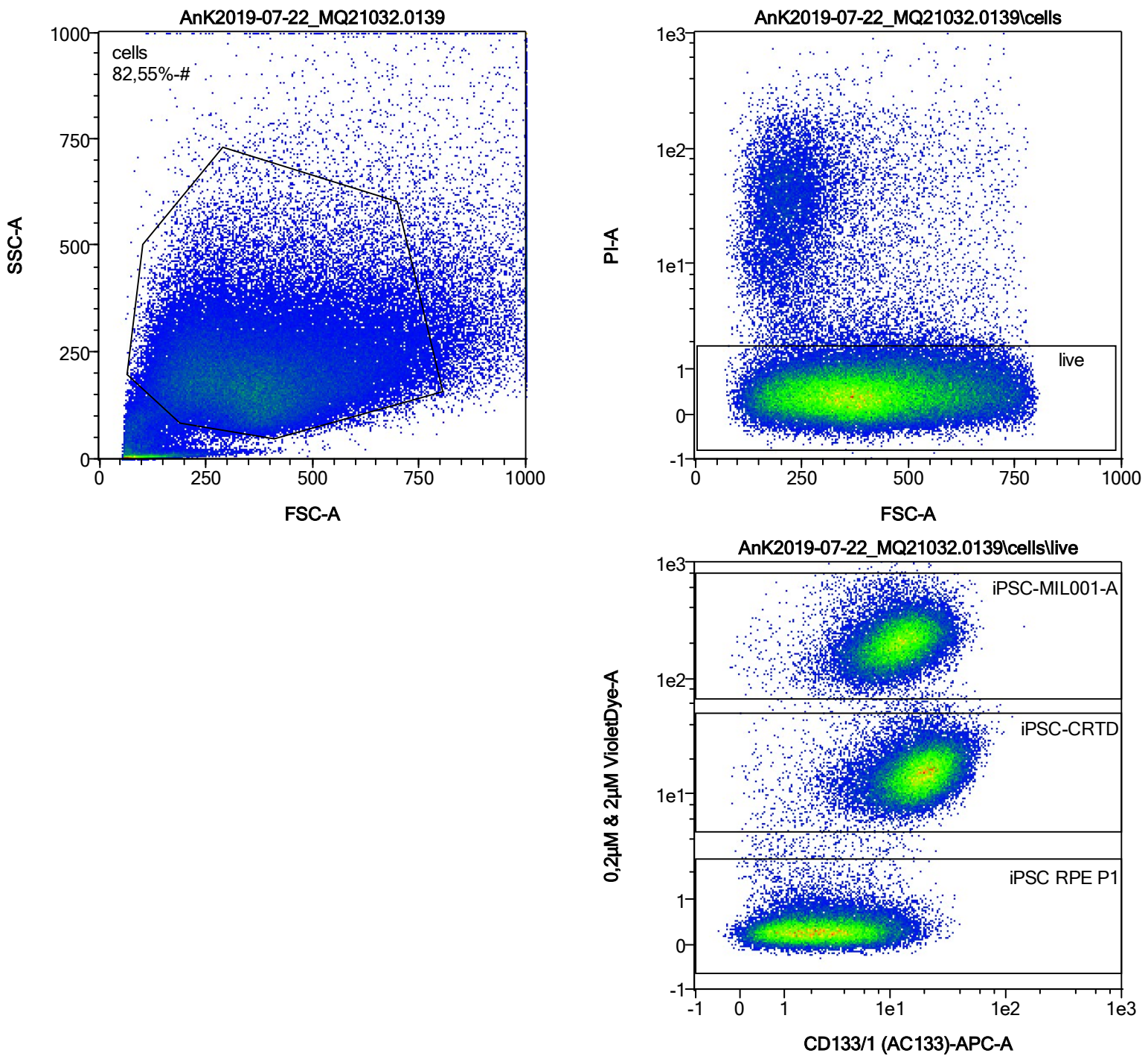

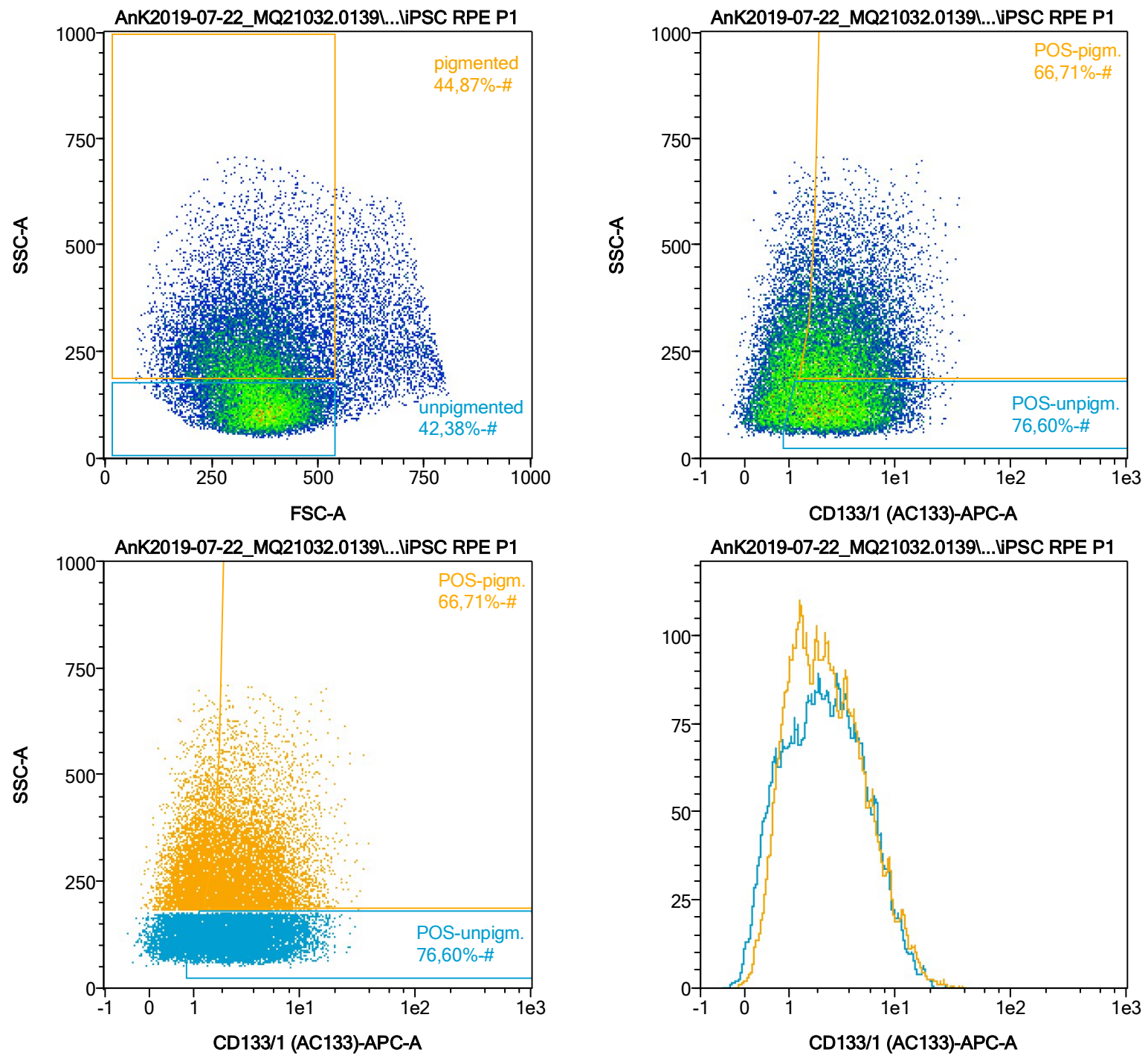

Name	%-#	CD133/1 (AC133)-APC-A Mean	CD133/1 (AC133)-APC-A Median
POS-unpigm.	76,60	3,90	3,03
POS-pigm.	66,71	4,40	3,36

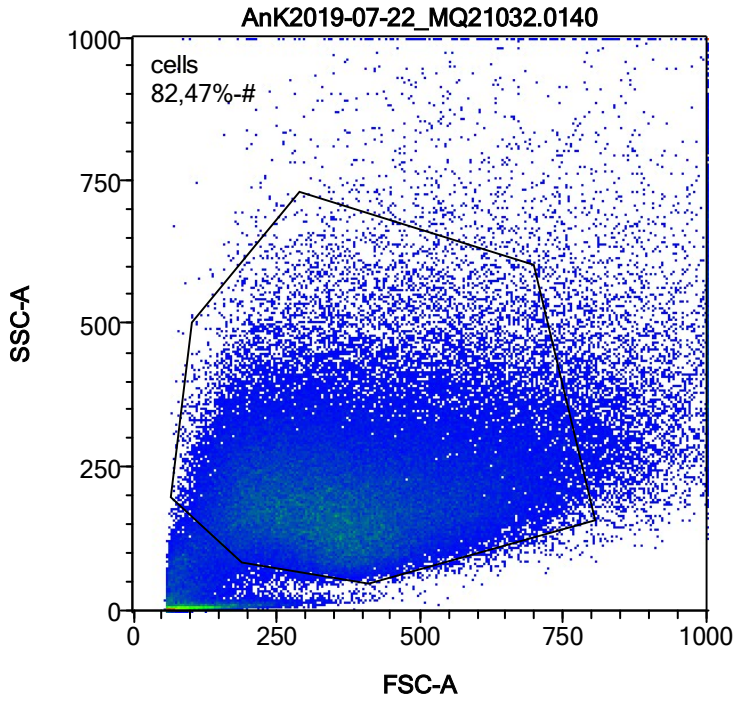

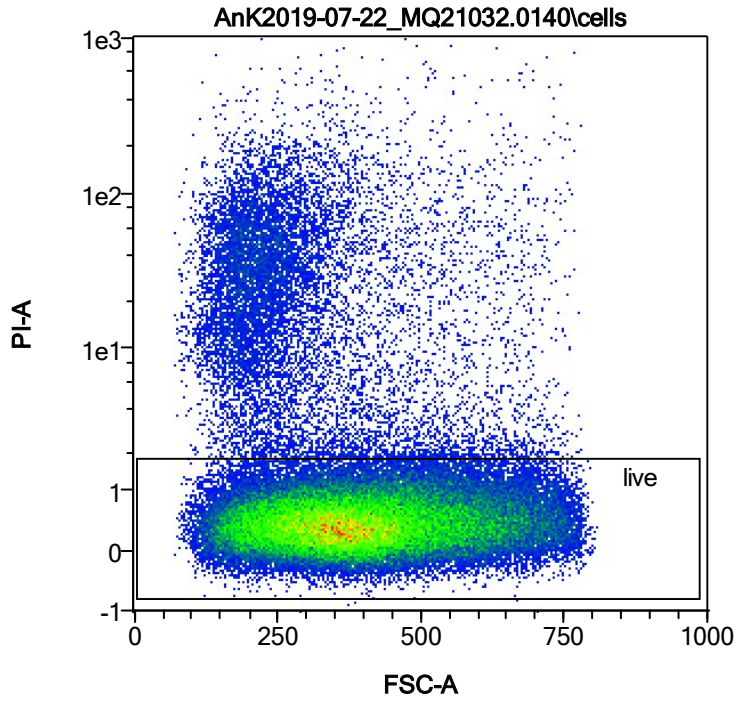

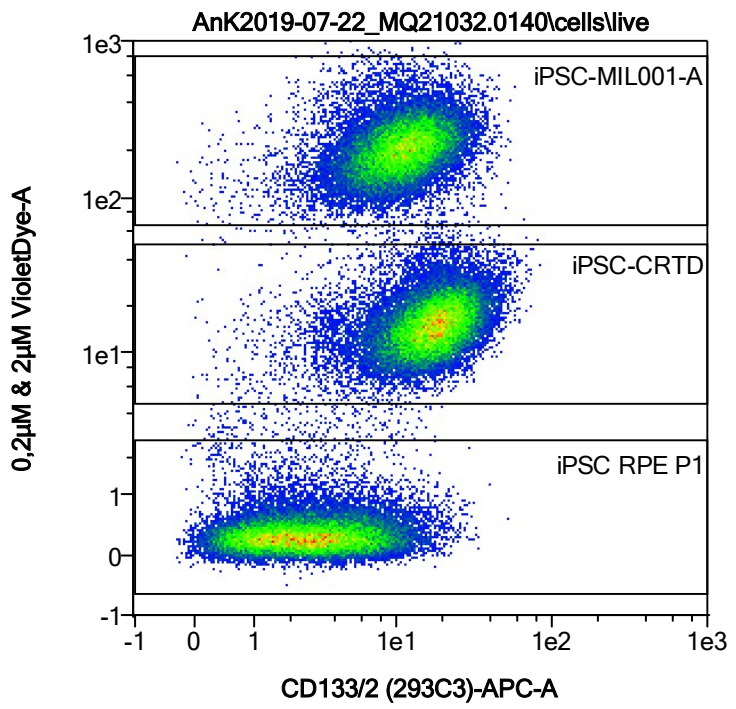

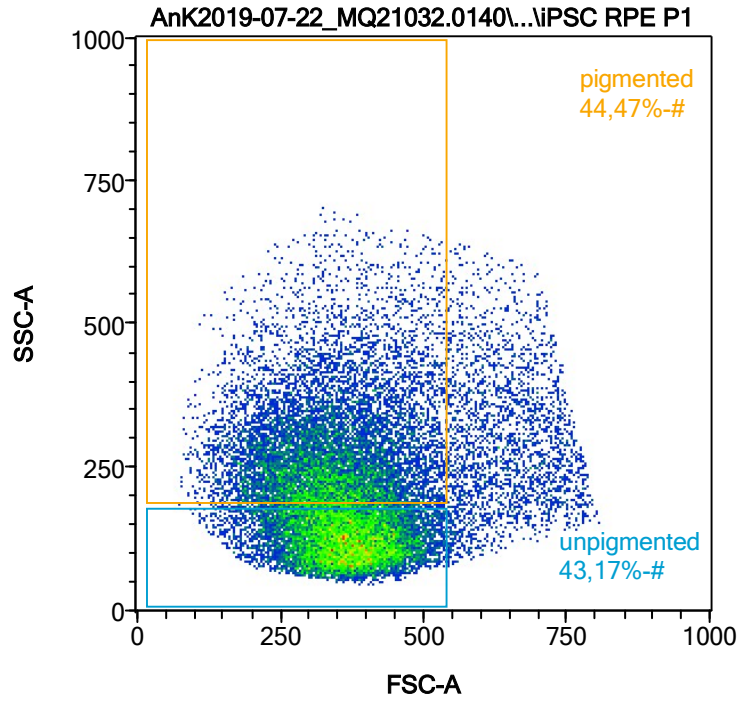

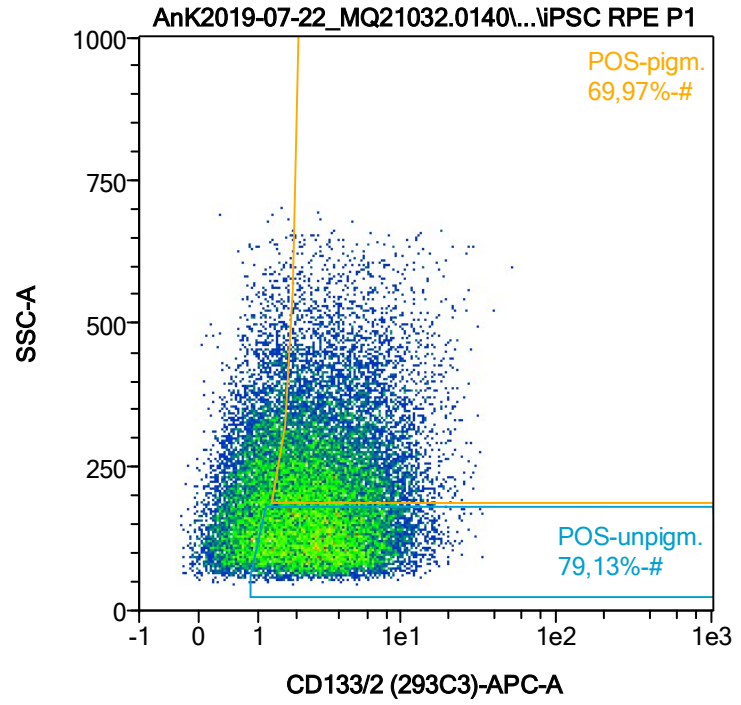

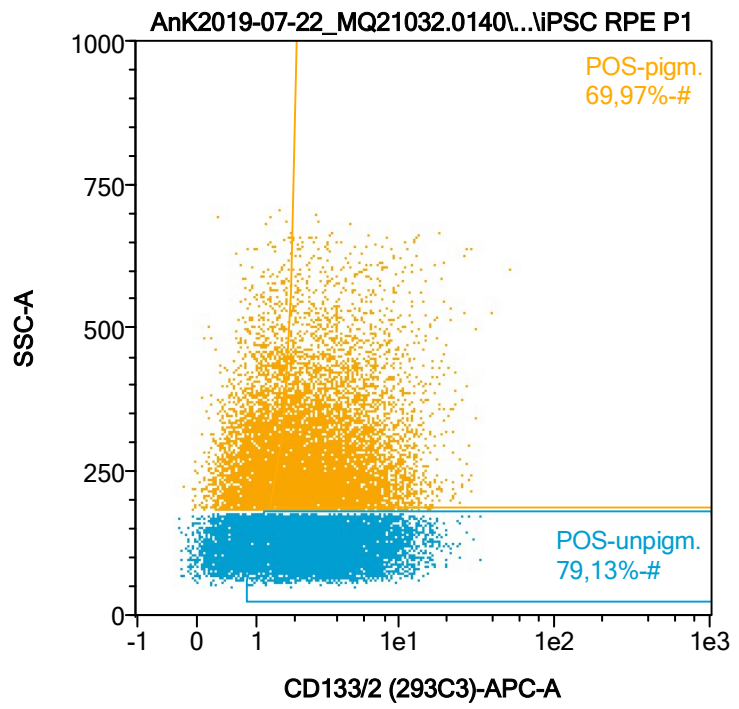

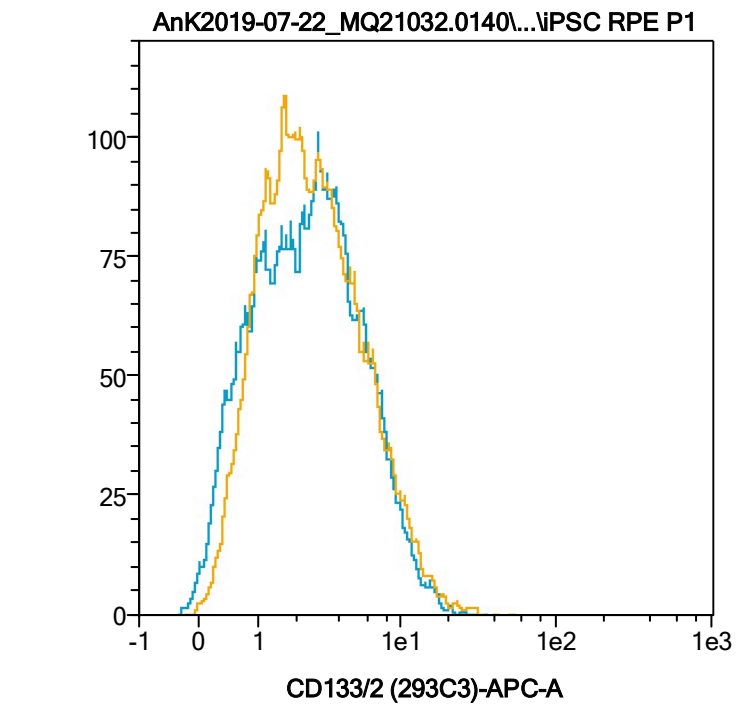

Name	%-#	CD133/2 (293C3)-APC-A Mean	CD133/2 (293C3)-APC-A Median
POS-unpigm.	79,13	3,88	3,05
POS-pigm.	69,97	4,36	3,32

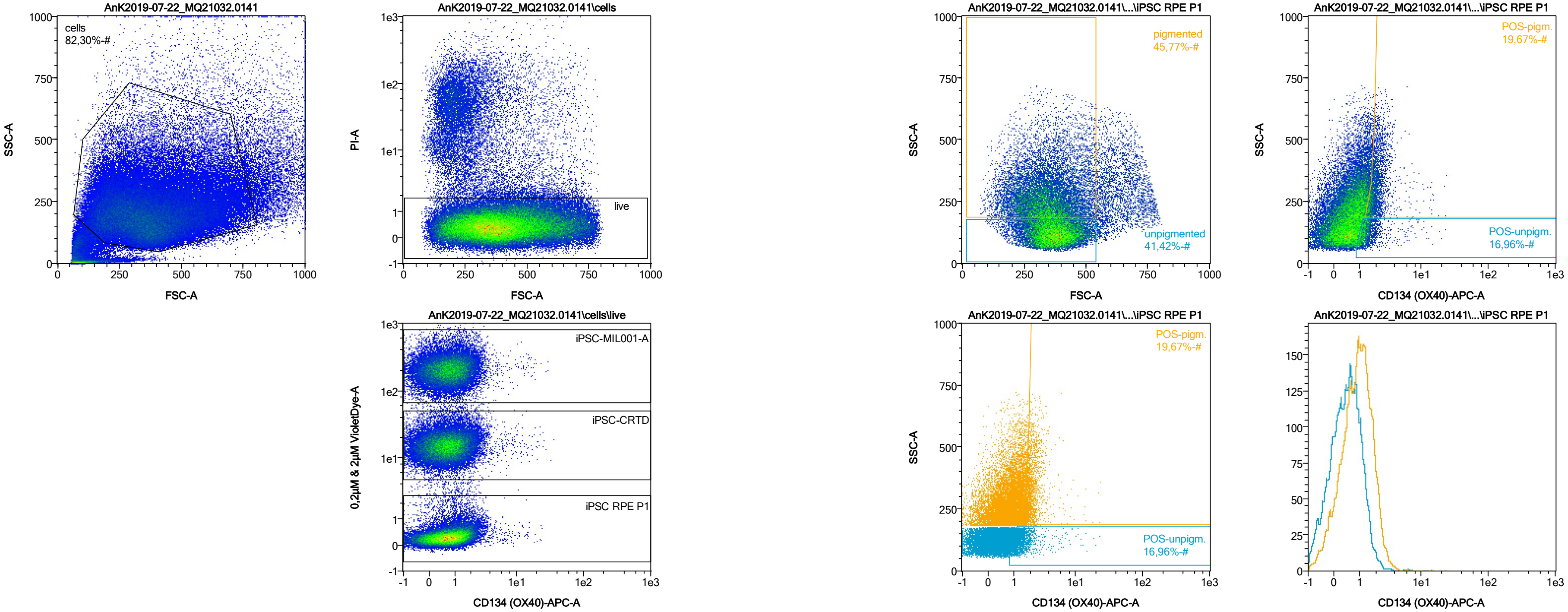

Name	%-#	CD134 (OX40)-APC-A Mean	CD134 (OX40)-APC-A Median
POS-unpigm.	16,96	1,55	1,28
POS-pigm.	19,67	2,12	1,90

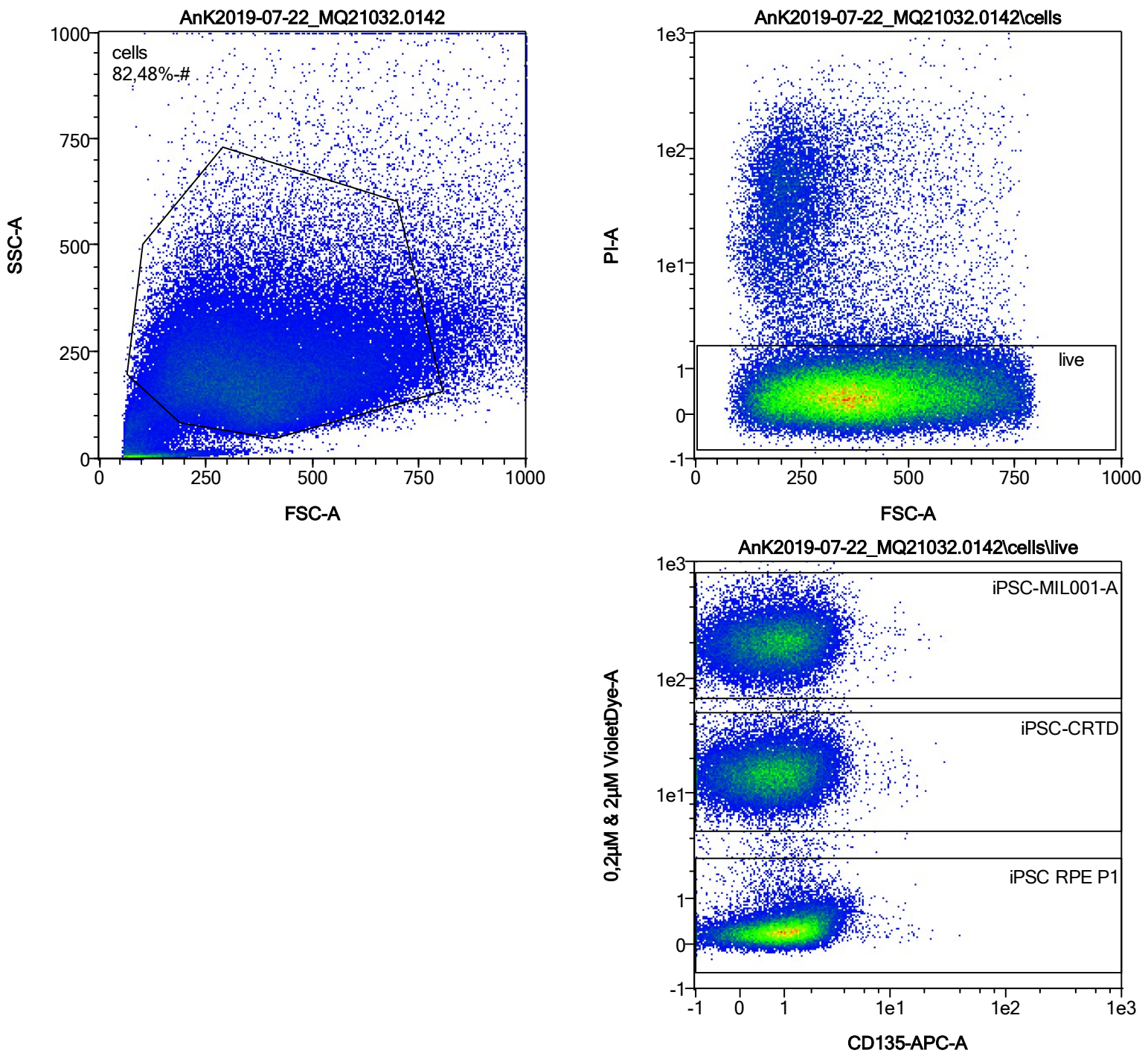

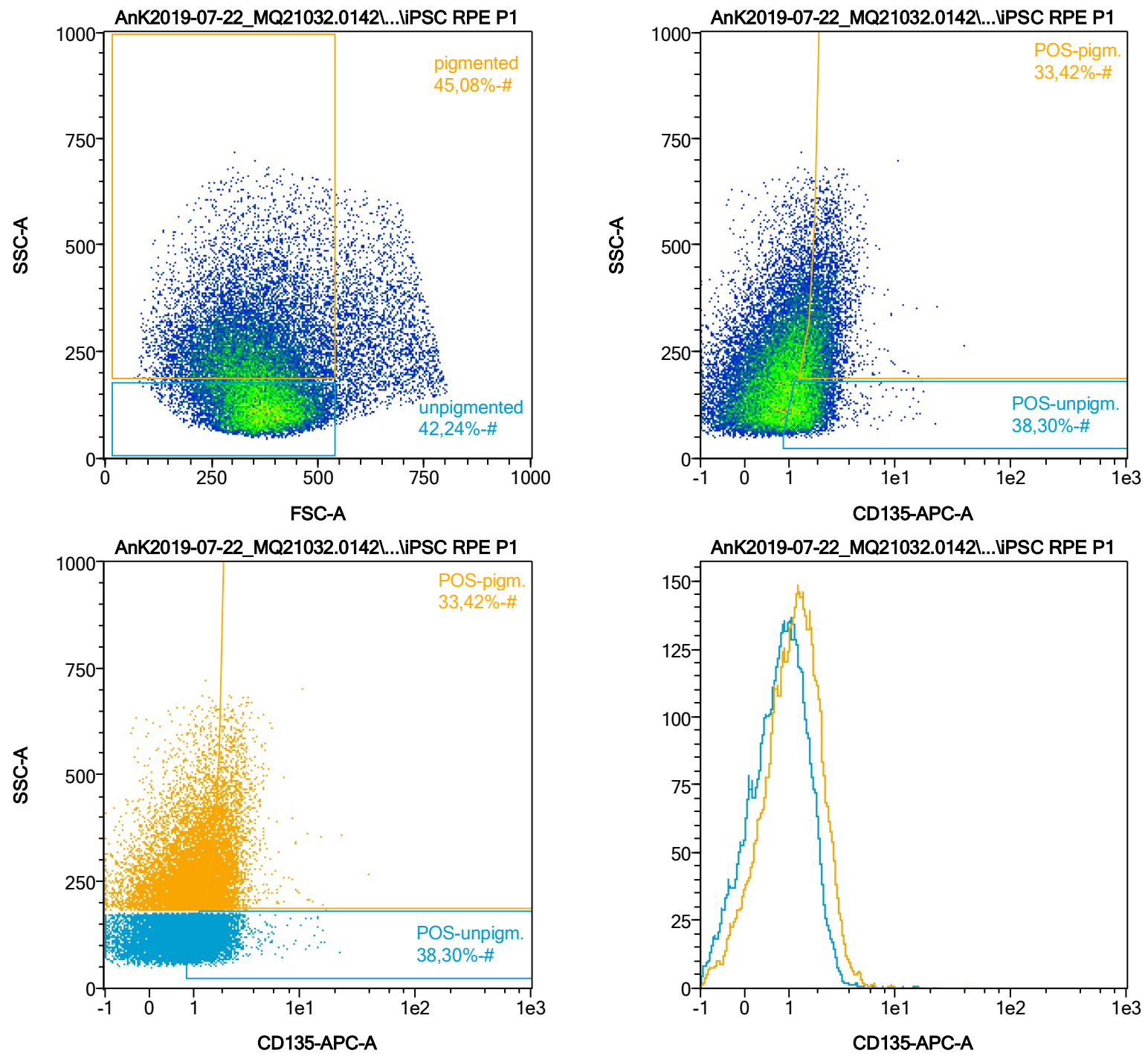

Name	%-#	CD135-APC-A Mean	CD135-APC-A Median
POS-unpigm.	38,30	1,67	1,48
POS-pigm.	33,42	2,25	2,04

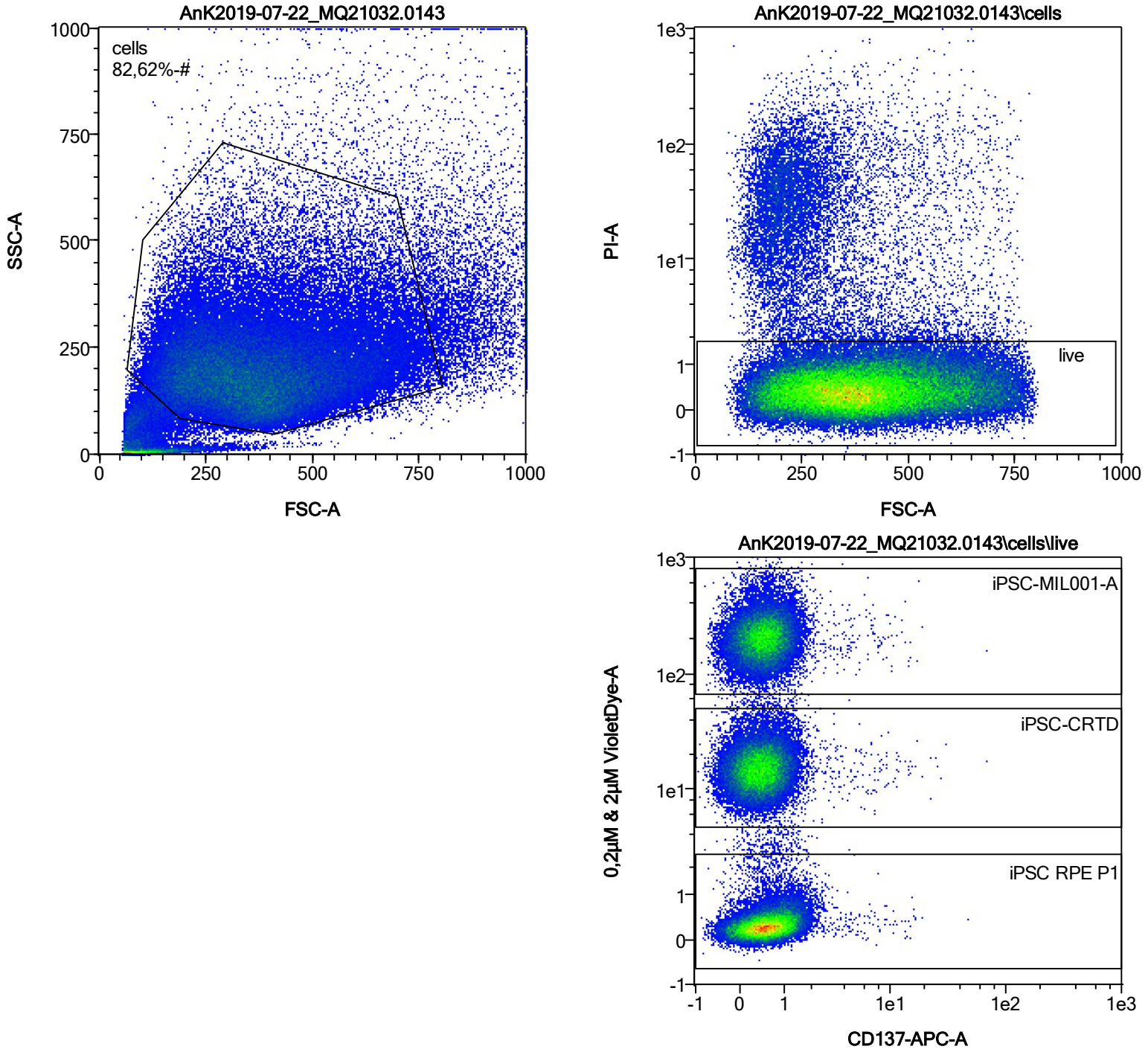

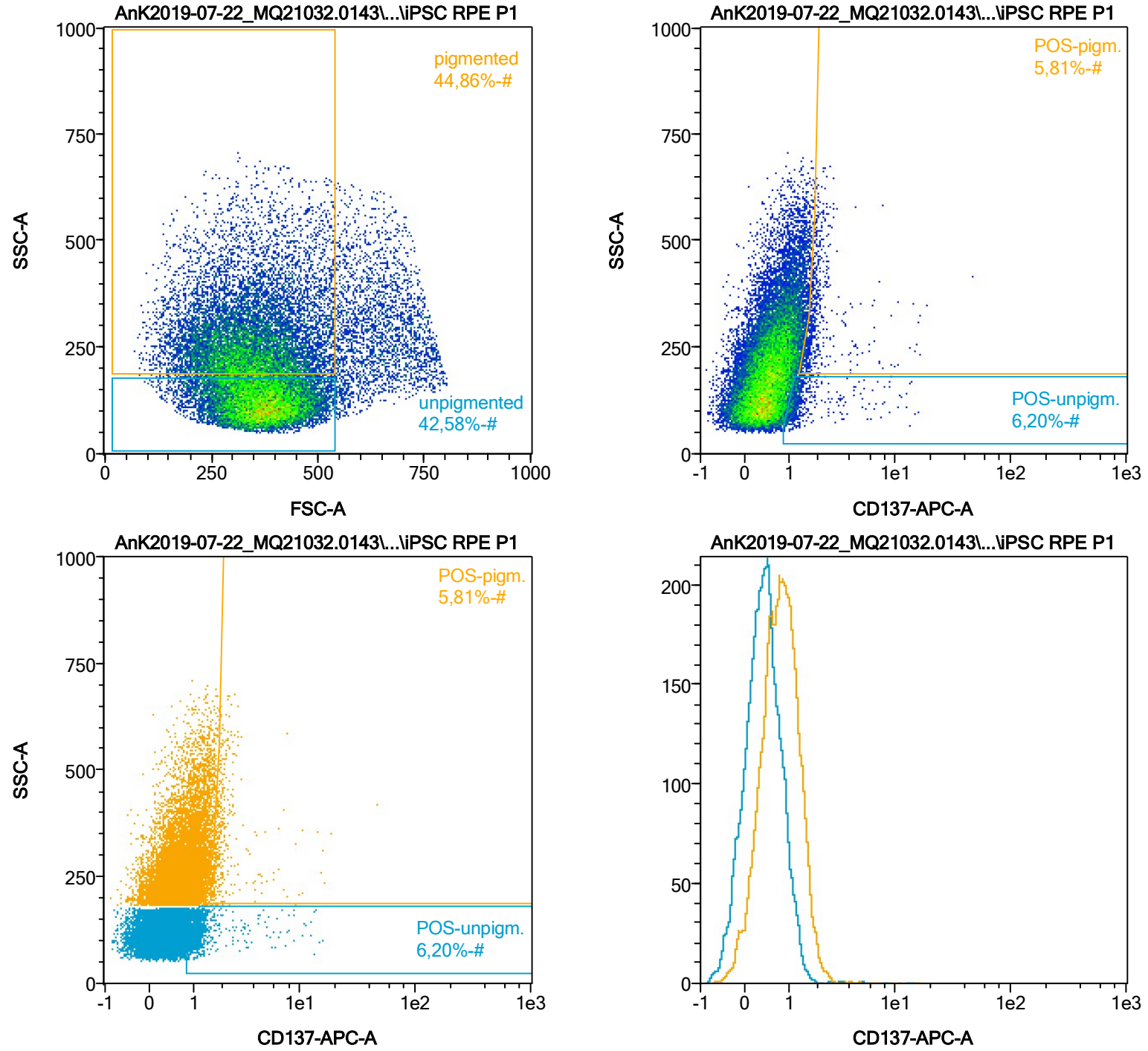

Name	%-#	CD137-APC-A Mean	CD137-APC-A Median
POS-unpigm.	6,20	1,65	1,19
POS-pigm.	5,81	2,19	1,78

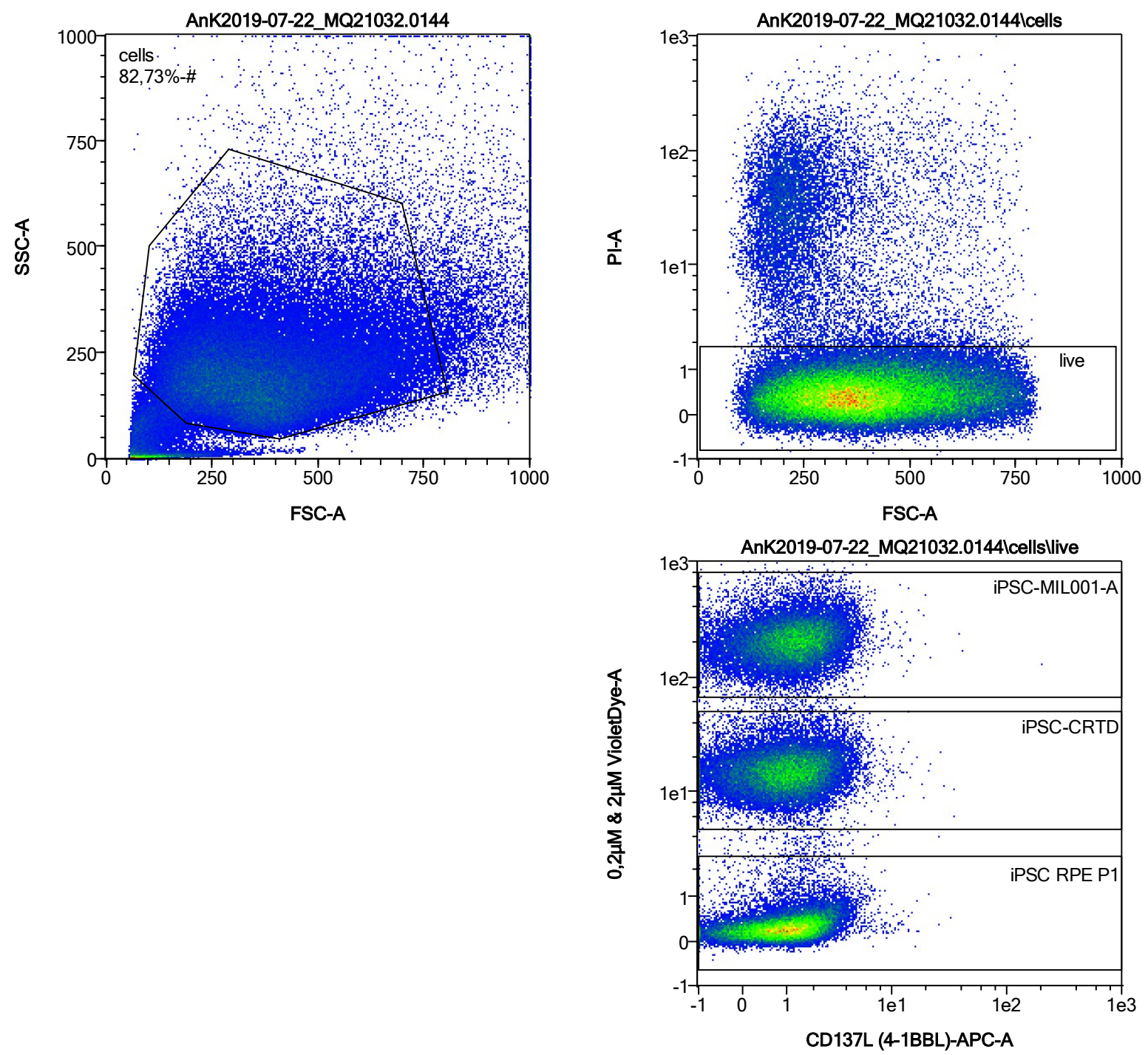

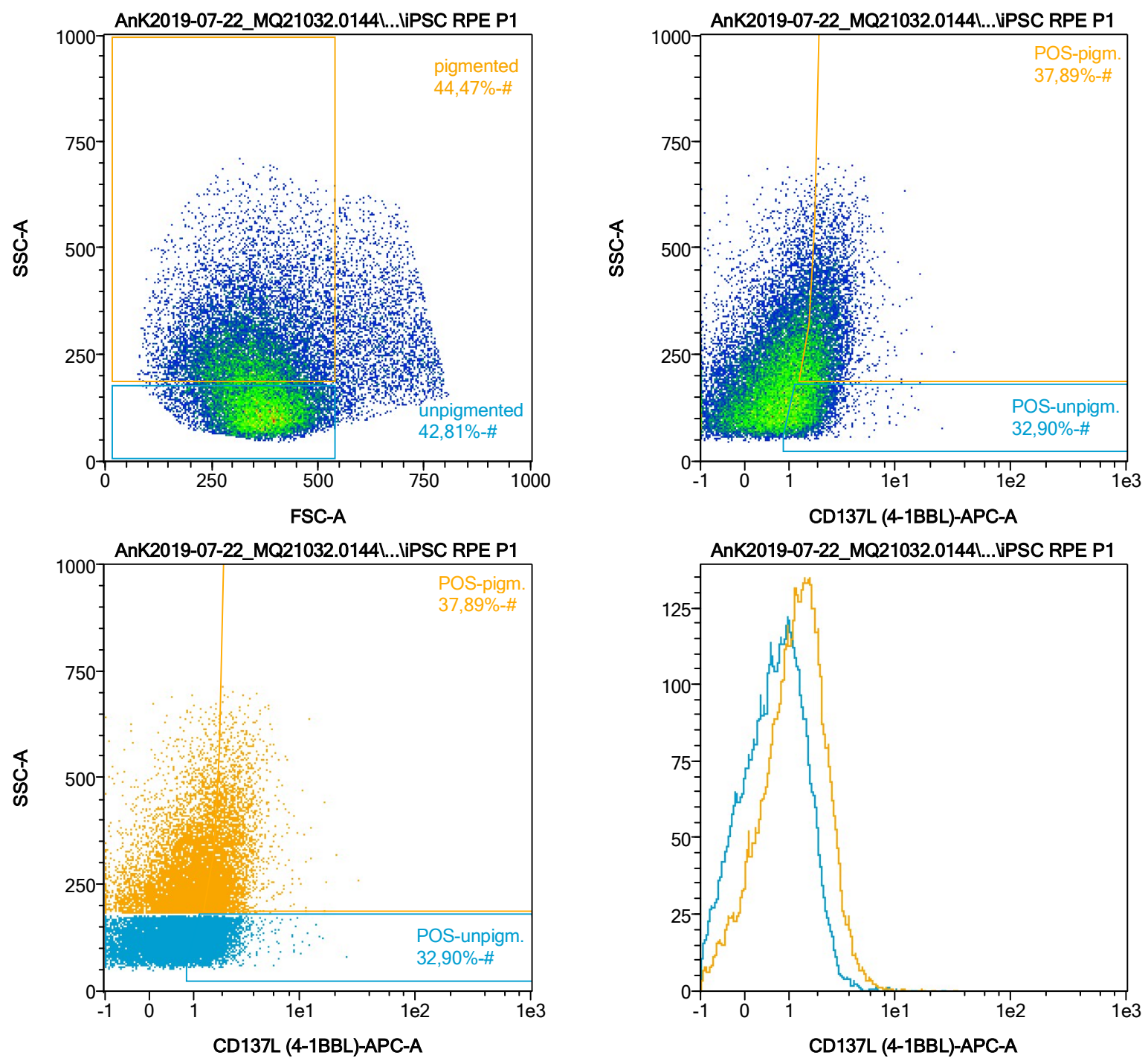

Name	%-#	CD137L (4-1BBL)-APC-A Mean	CD137L (4-1BBL)-APC-A Median
POS-unpigm.	32,90	1,69	1,48
POS-pigm.	37,89	2,41	2,12

Date:2024-06-14

Operator: Sebastian Knoebel

Version:2.13.0+54.g95abf0f4

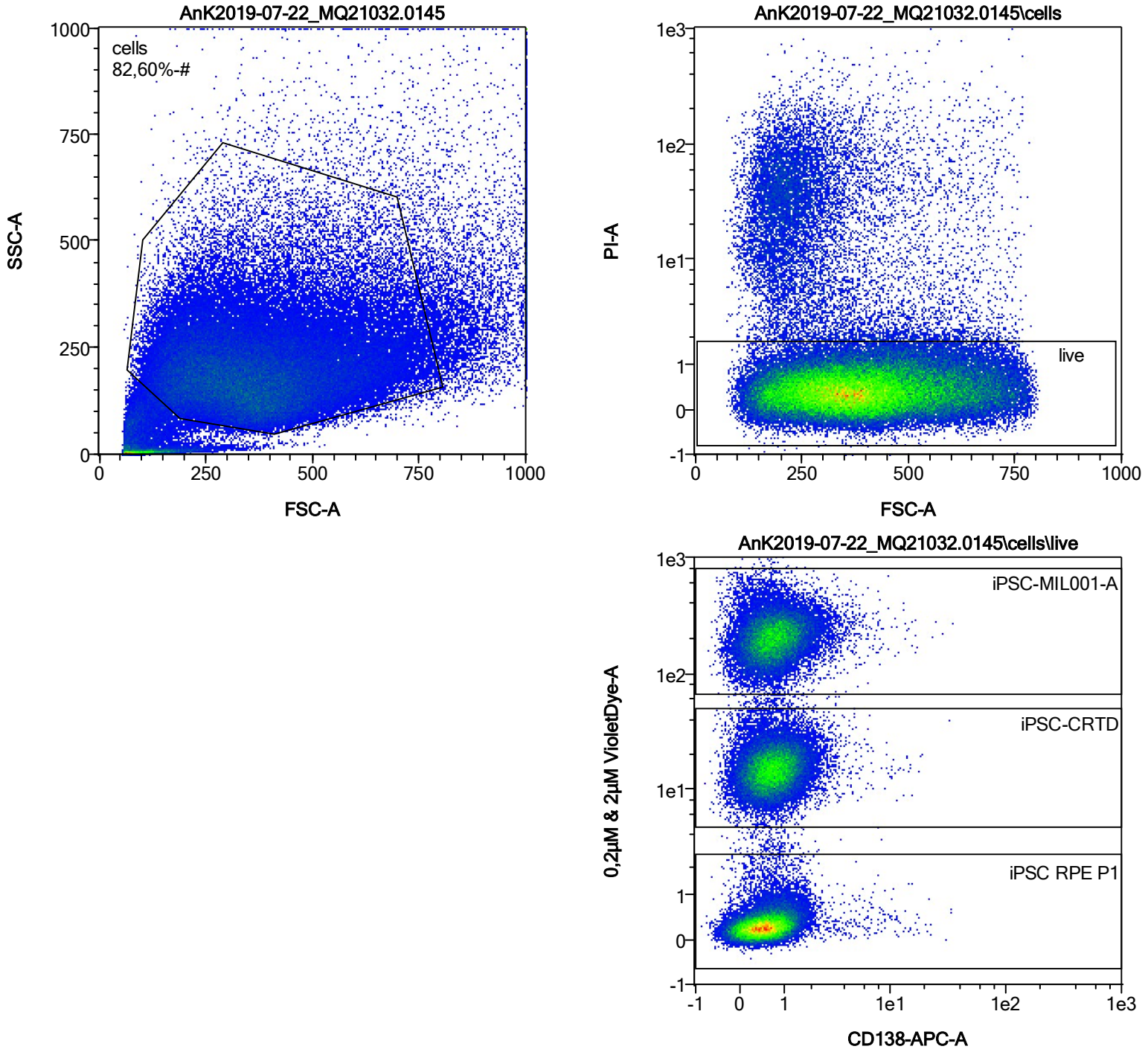

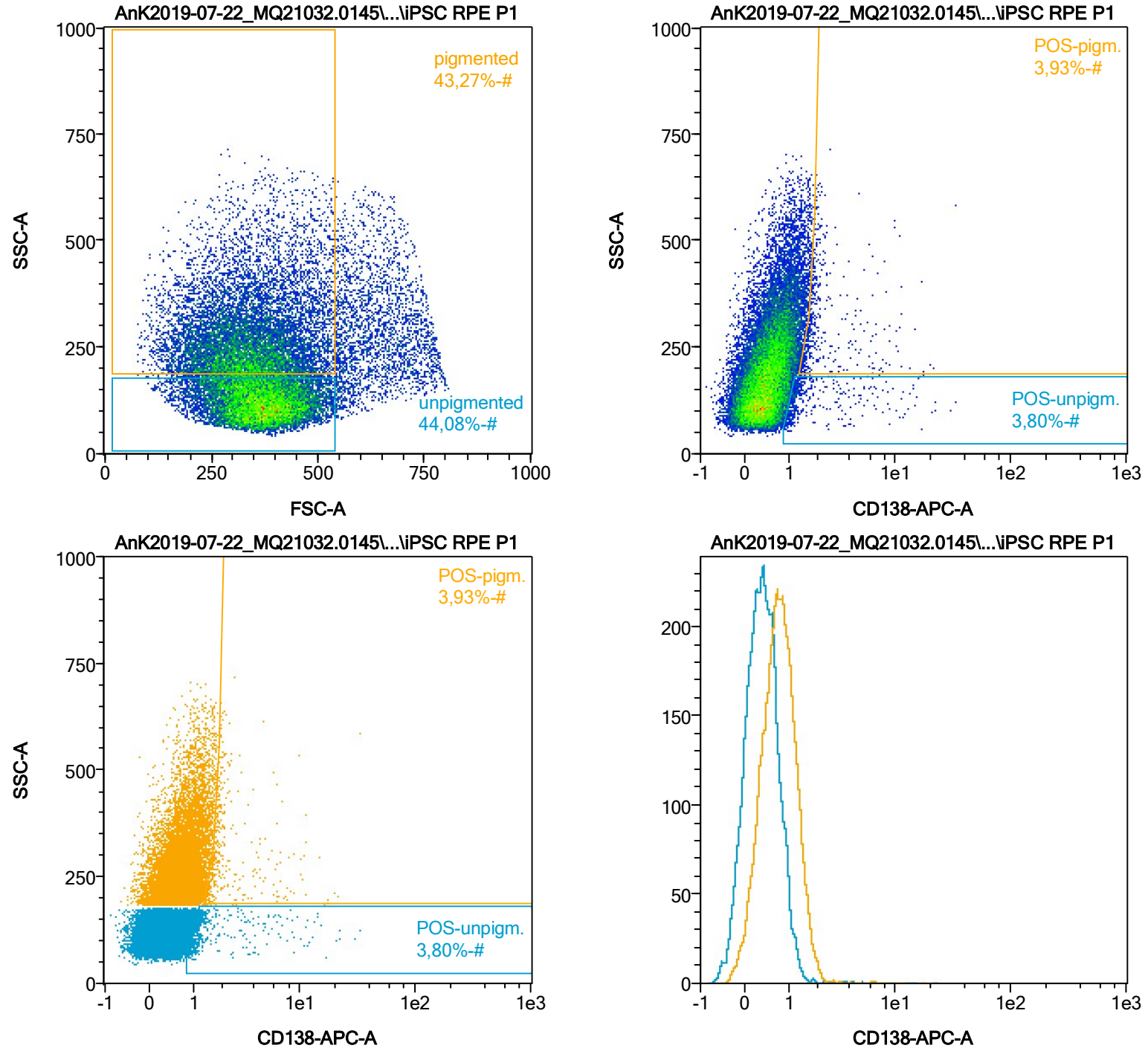

Name	%-#	CD138-APC-A Mean	CD138-APC-A Median
POS-unpigm.	3,80	2,37	1,21
POS-pigm.	3,93	2,74	1,85

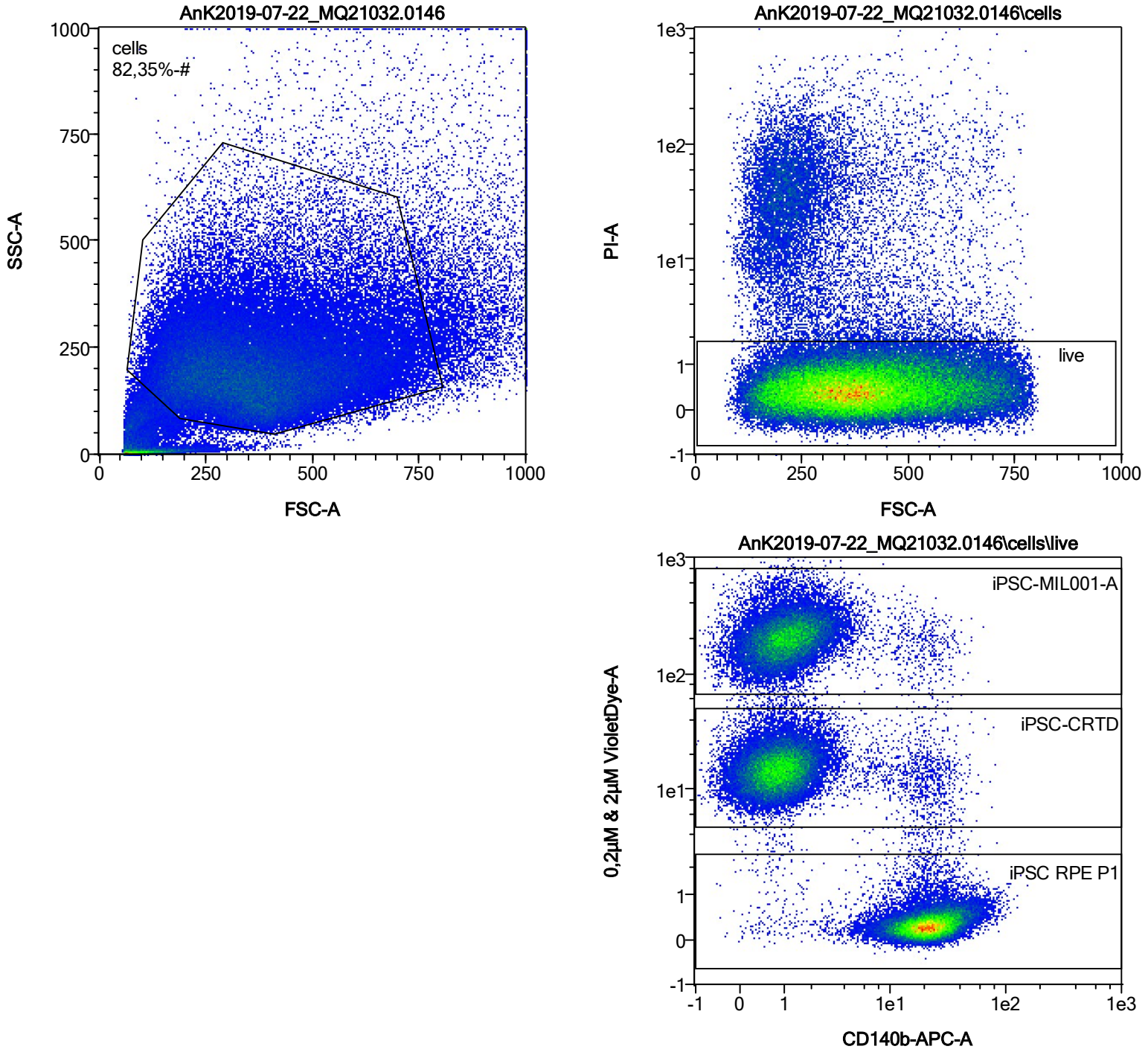

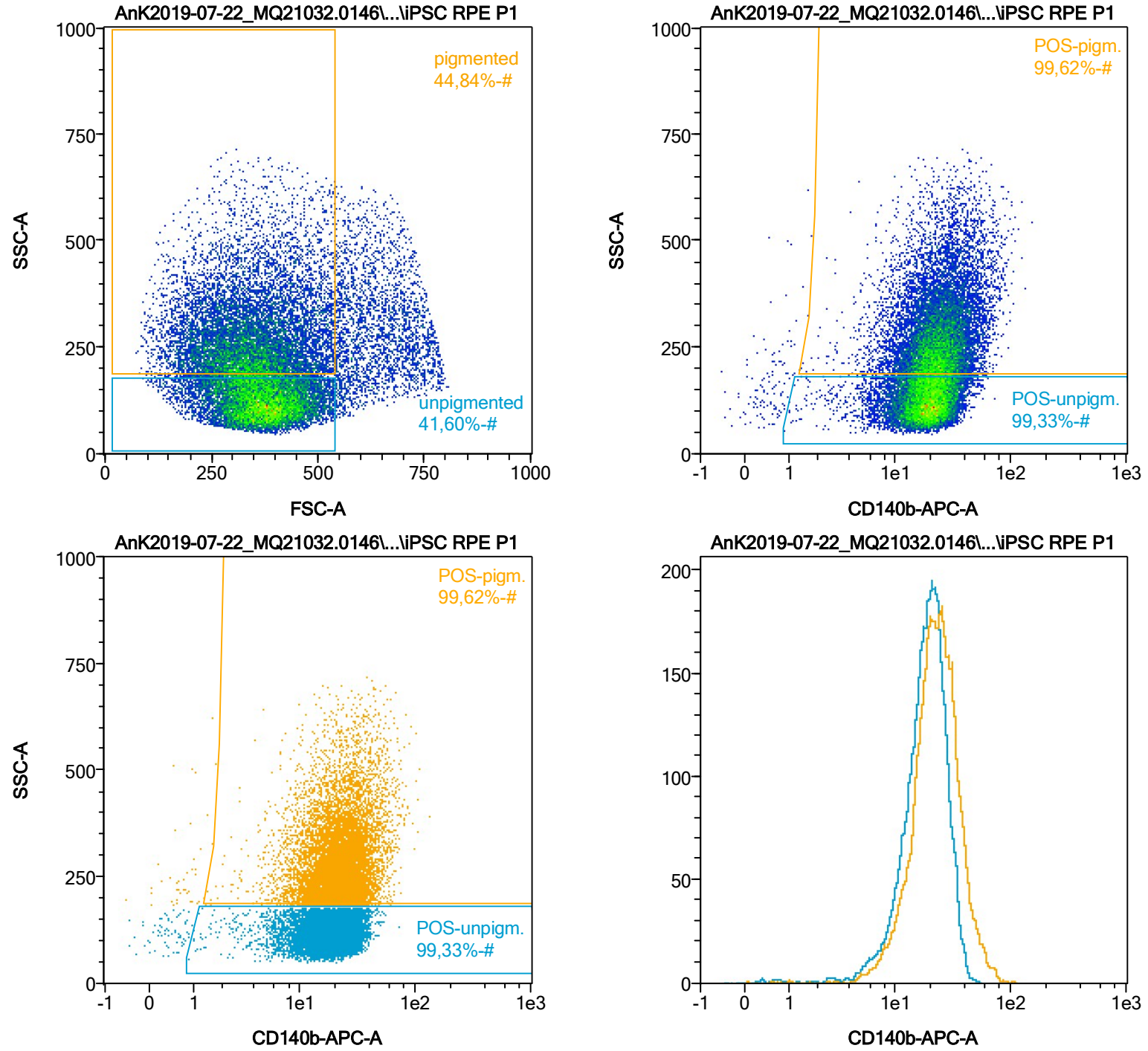

Name	%-#	CD140b-APC-A Mean	CD140b-APC-A Median
POS-unpigm.	99,33	19,26	18,83
POS-pigm.	99,62	24,40	22,55

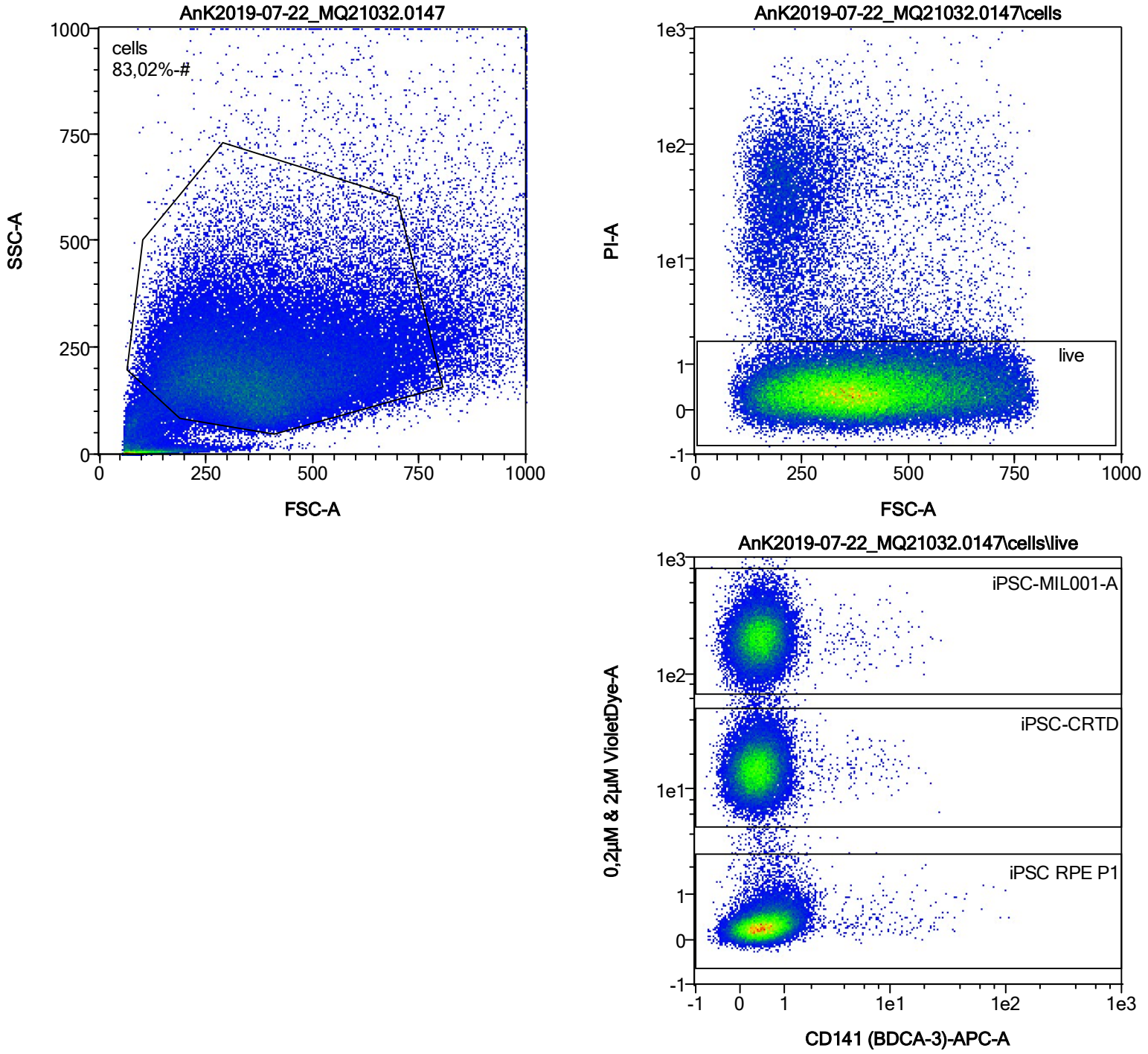

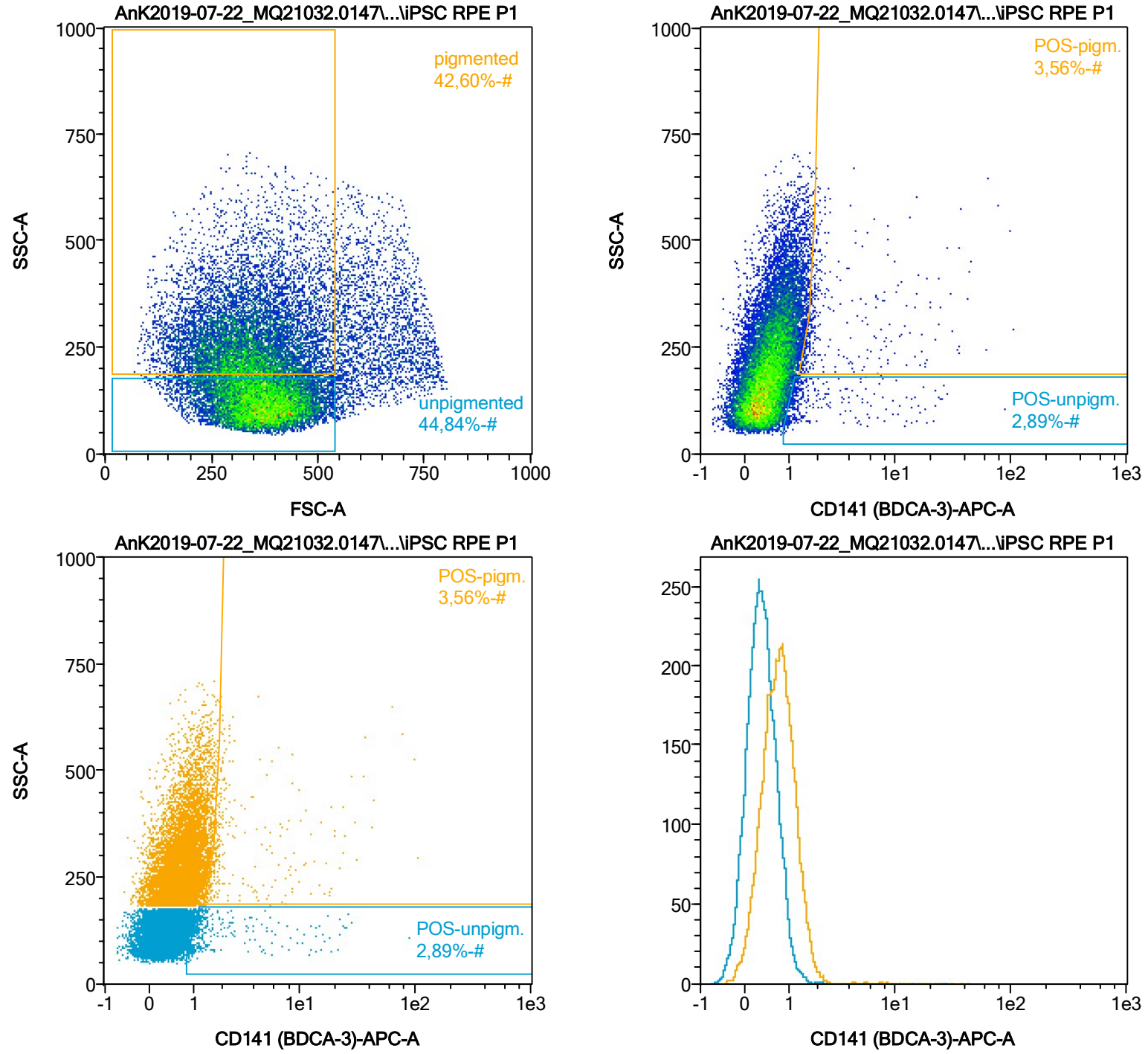

Name	%-#	CD141 (BDCA-3)-APC-A Mean	CD141 (BDCA-3)-APC-A Median
POS-unpigm.	2,89	3,26	1,25
POS-pigm.	3,56	4,92	1,88

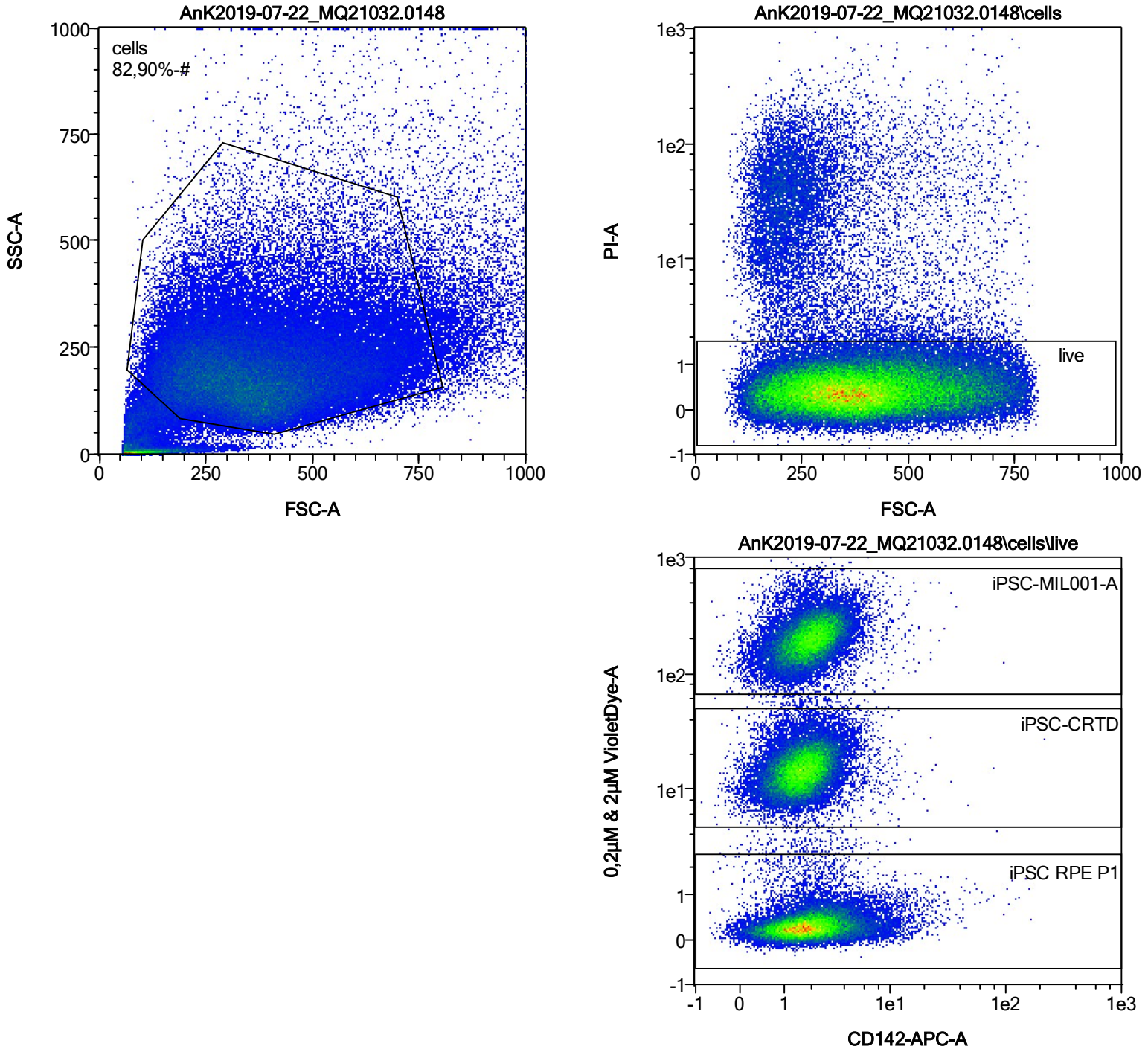

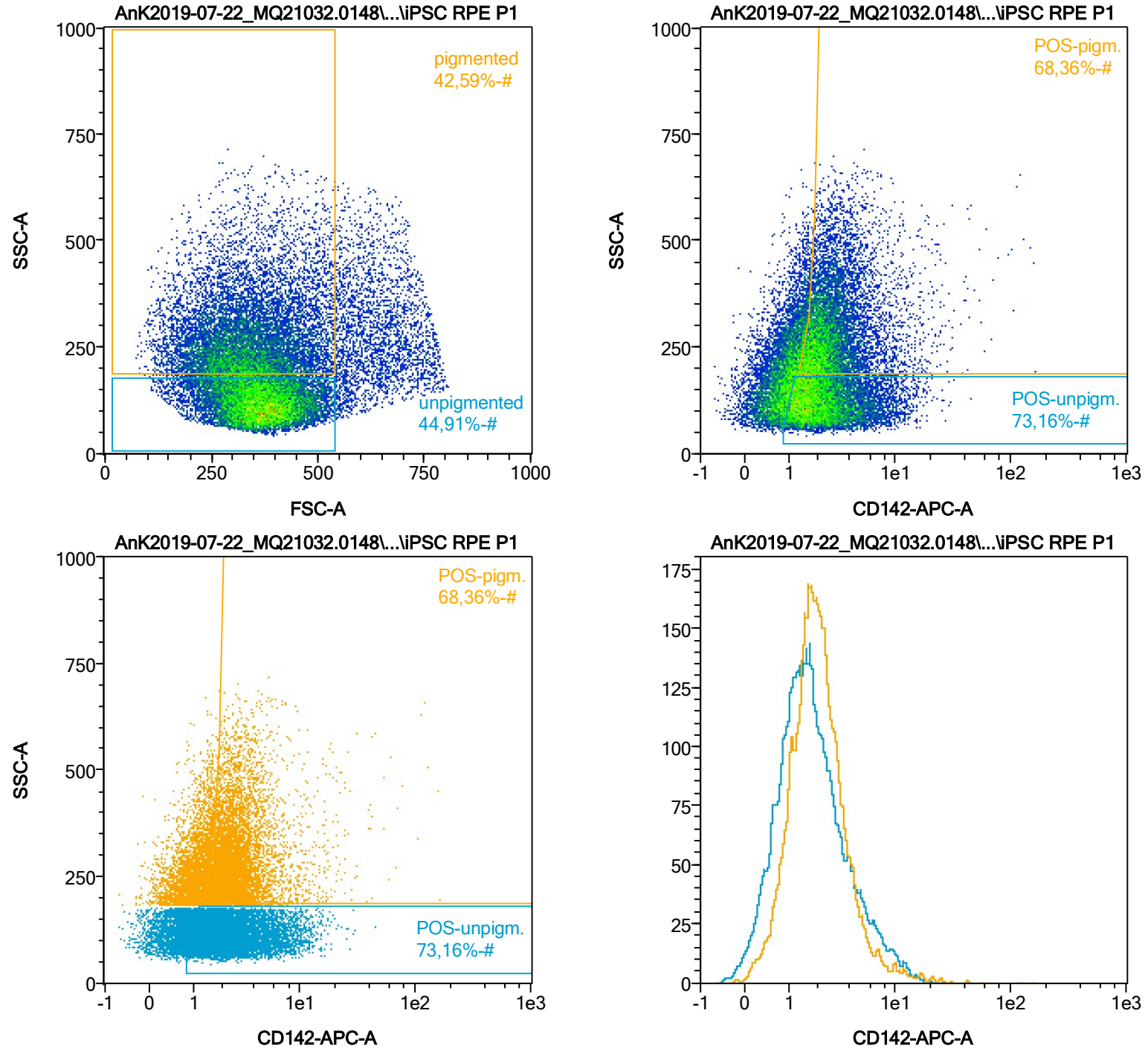

Name	%-#	CD142-APC-A Mean	CD142-APC-A Median
POS-unpigm.	73,16	2,86	2,05
POS-pigm.	68,36	3,23	2,37

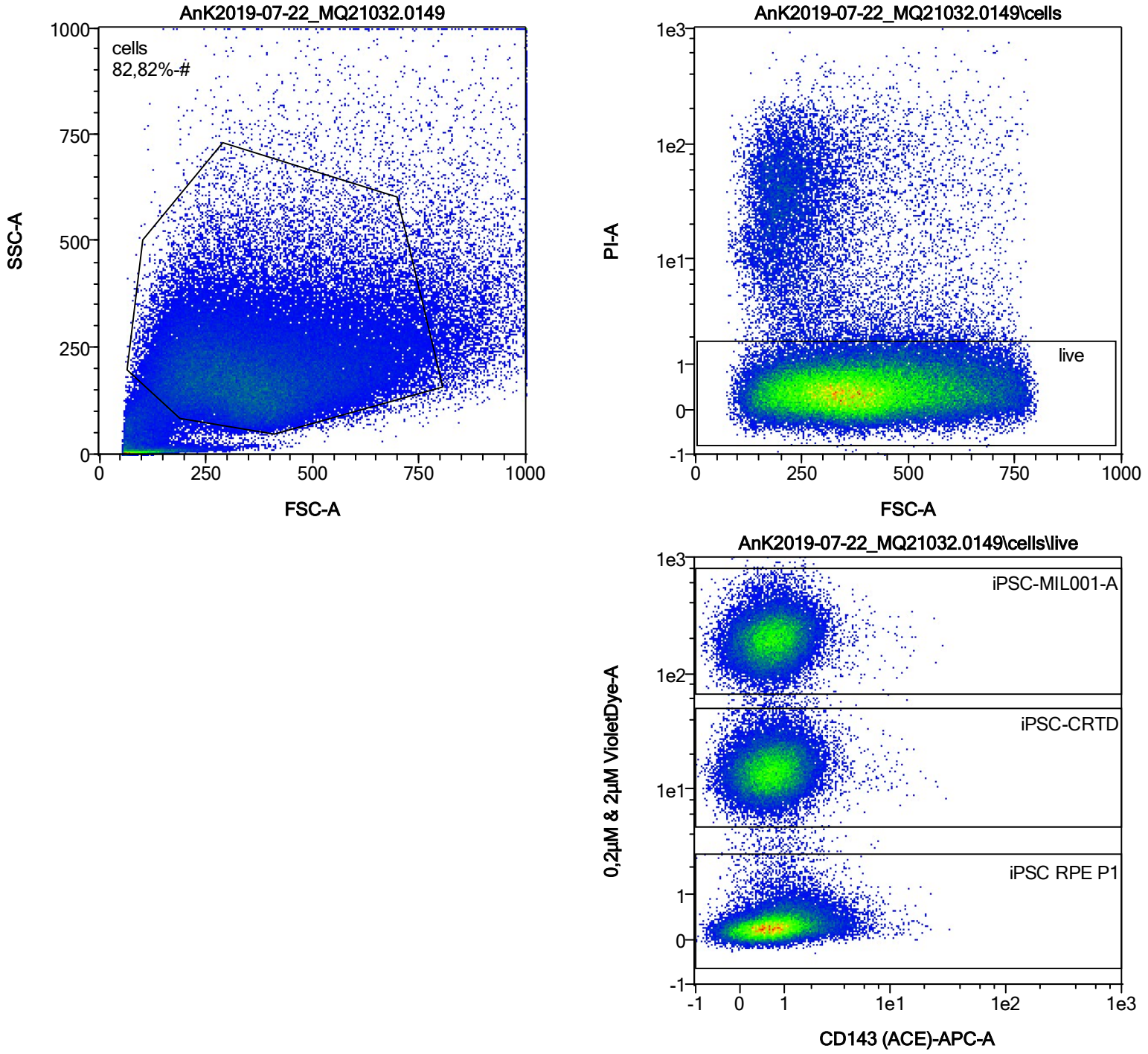

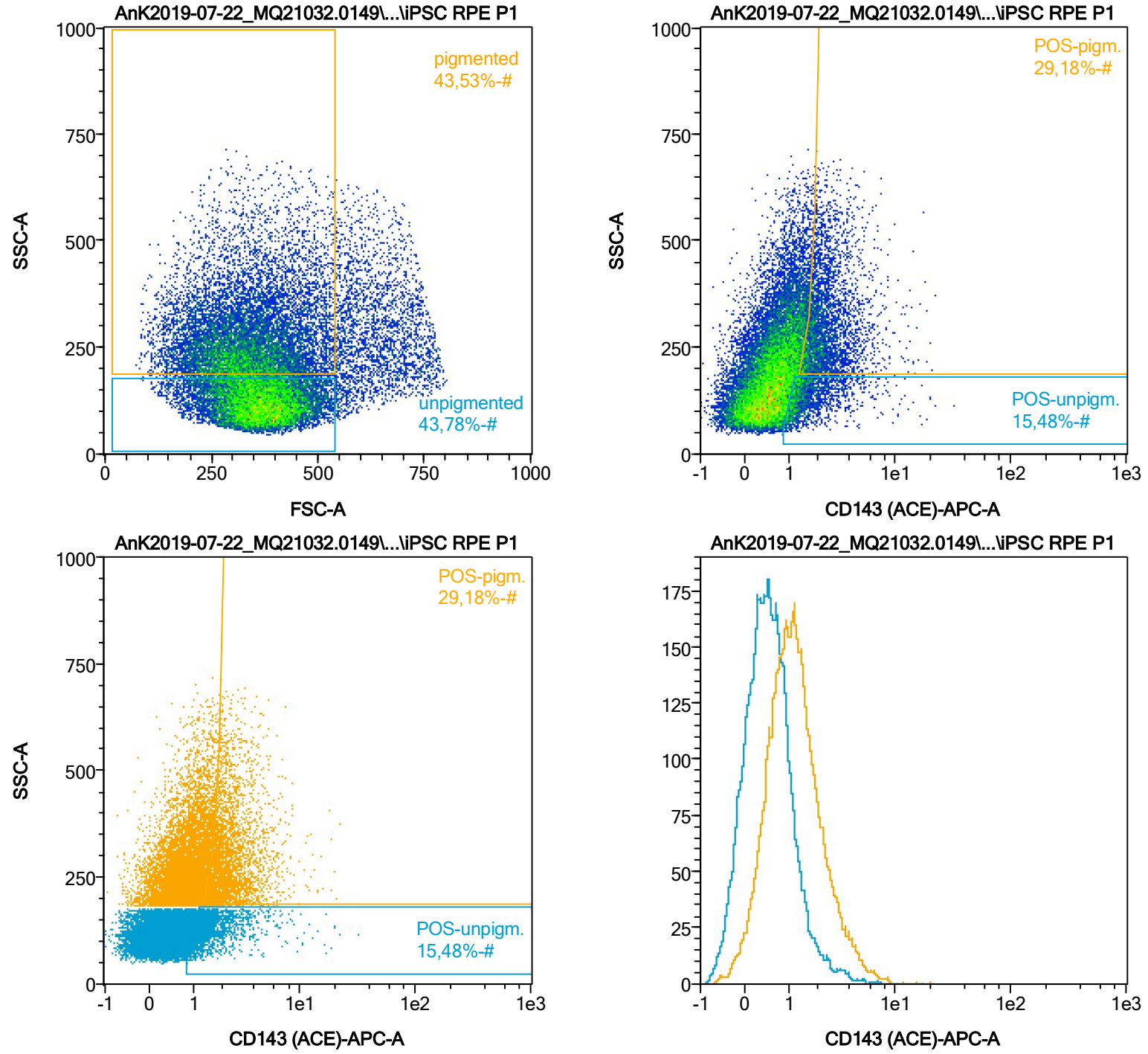

Name	%-#	CD143 (ACE)-APC-A Mean	CD143 (ACE)-APC-A Median
POS-unpigm.	15,48	1,83	1,36
POS-pigm.	29,18	2,63	2,19

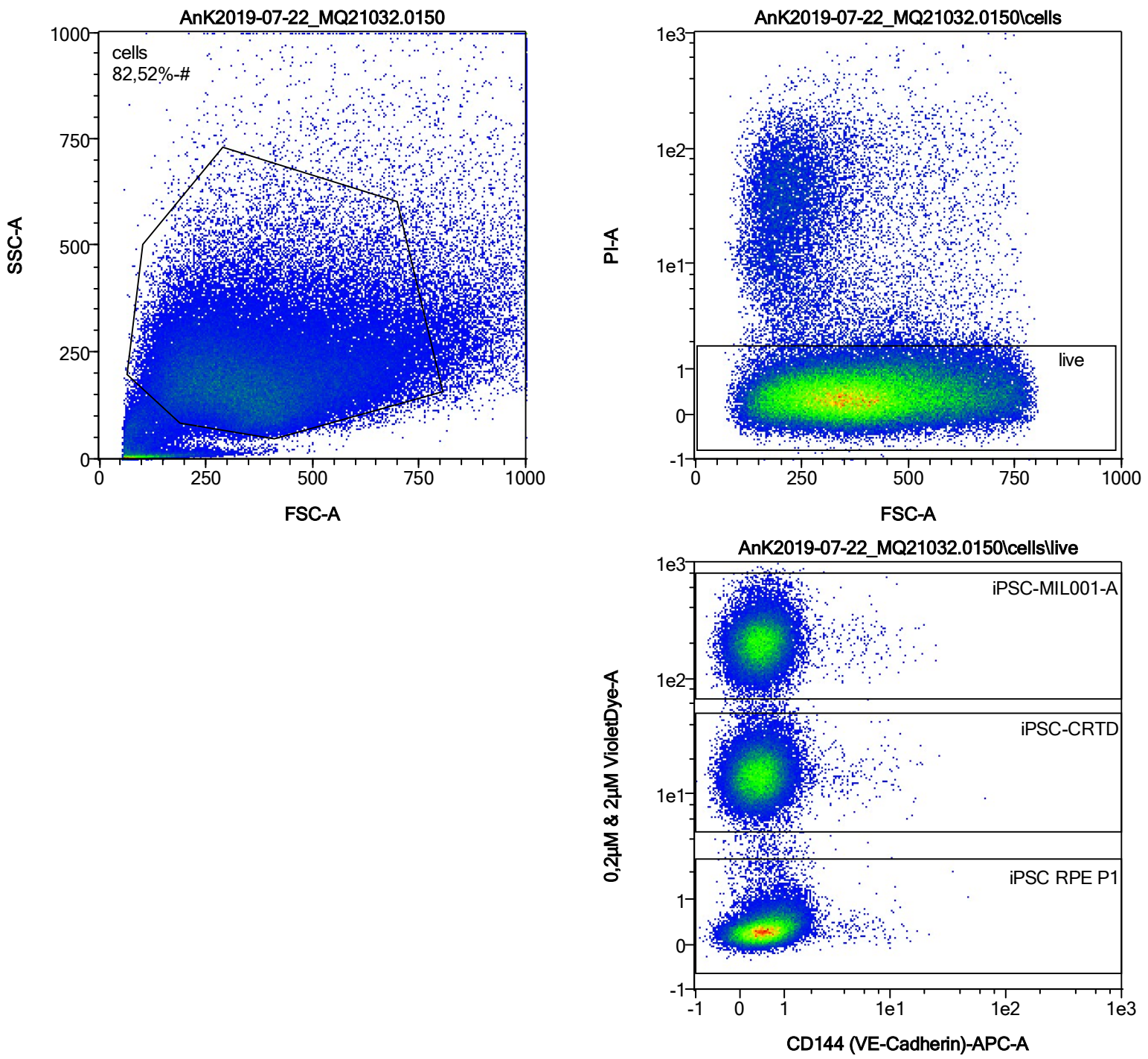

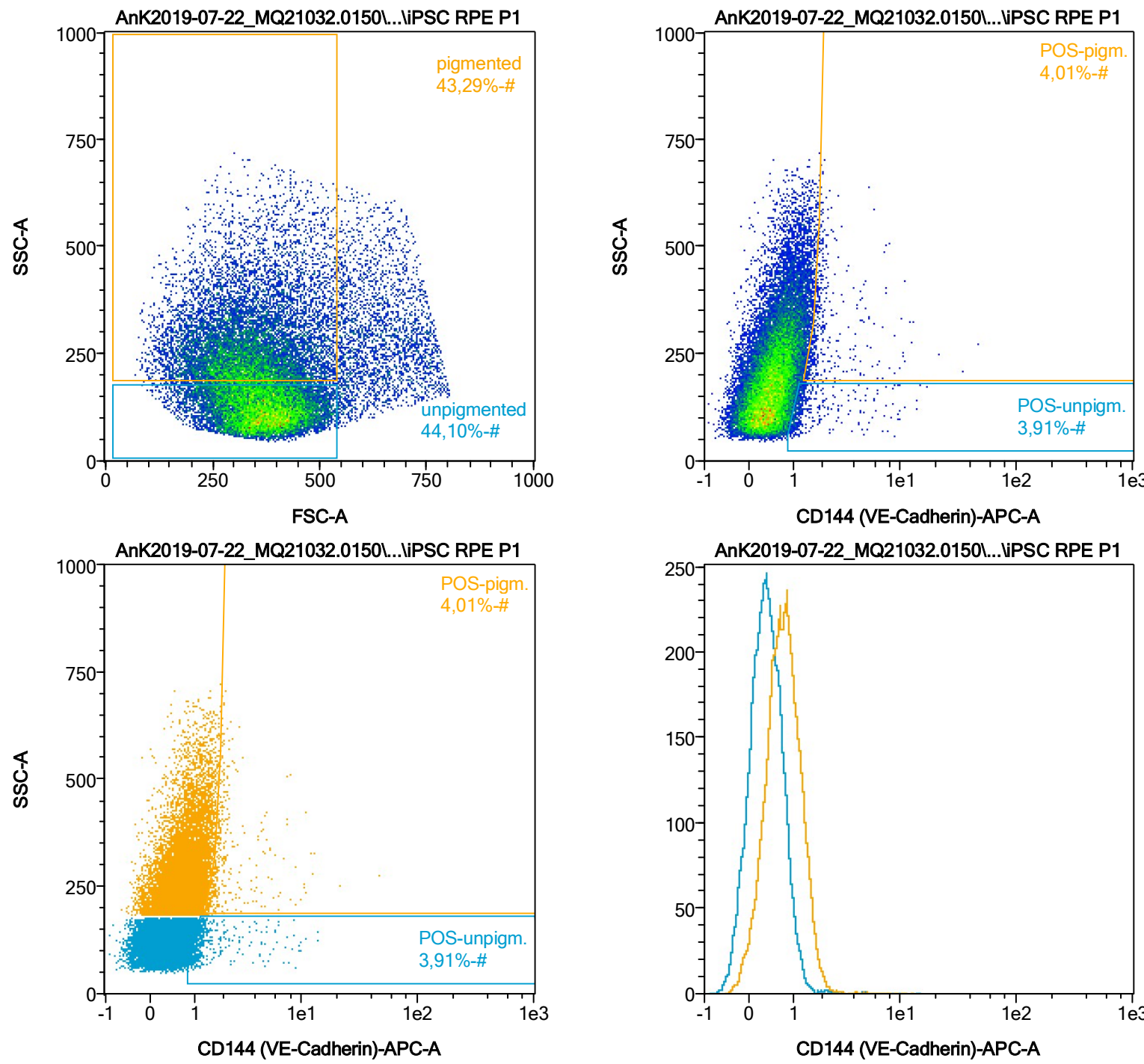

Name	%-#	CD144 (VE-Cadherin)-APC-A Mean	CD144 (VE-Cadherin)-APC-A Median
POS-unpigm.	3,91	1,78	1,18
POS-pigm.	4,01	2,47	1,82

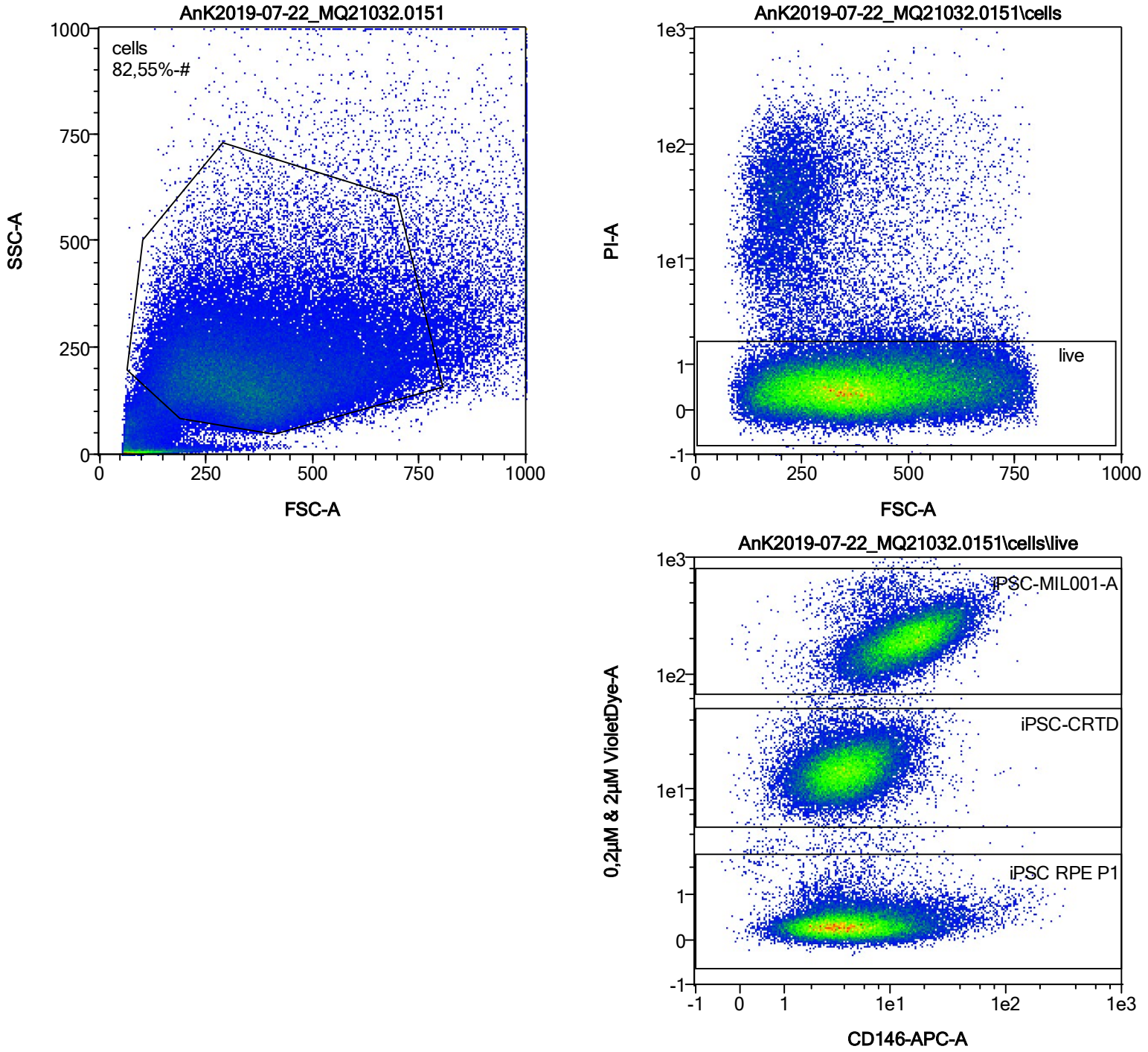

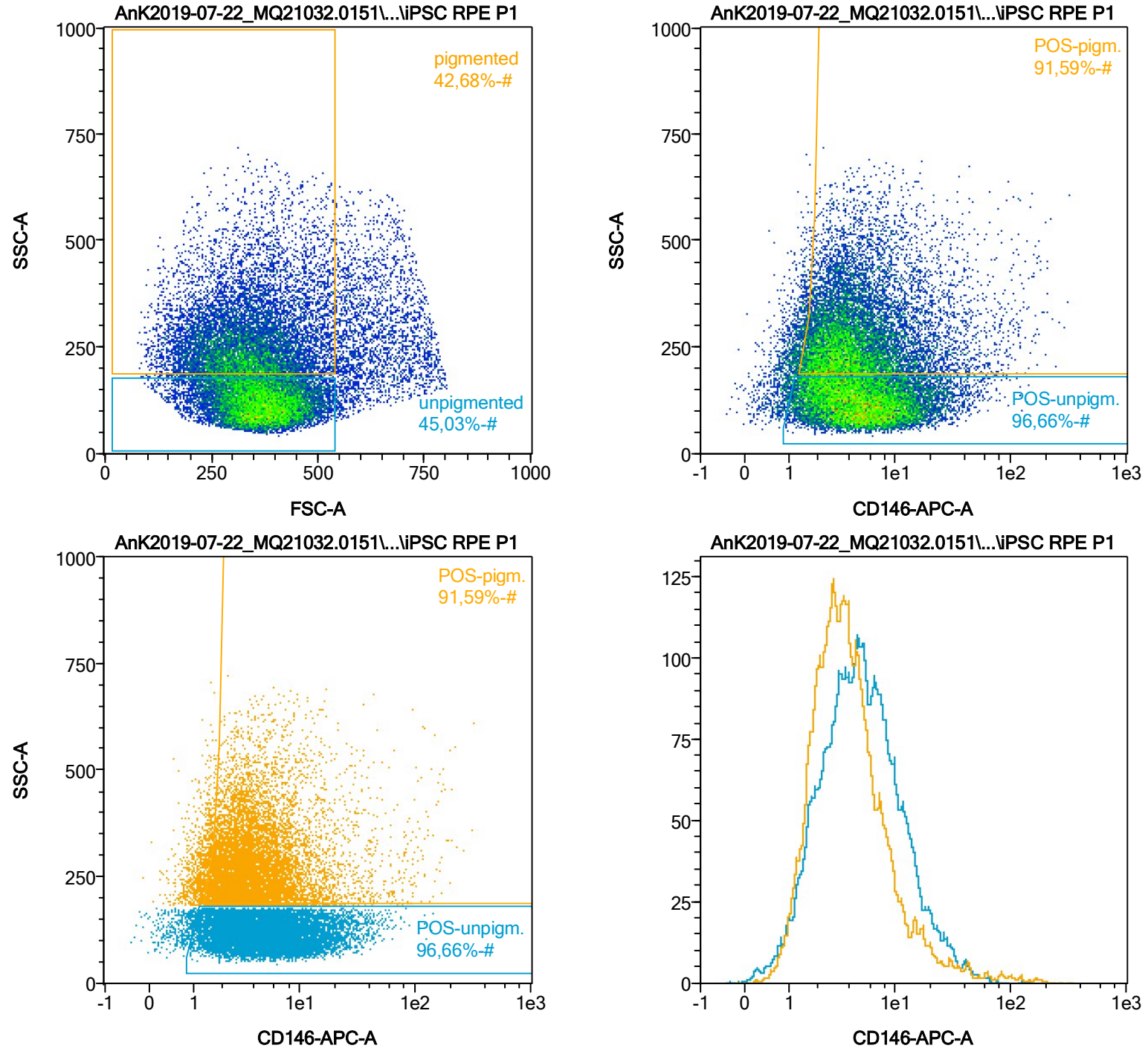

Name	%-#	CD146-APC-A Mean	CD146-APC-A Median
POS-unpigm.	96,66	7,14	4,98
POS-pigm.	91,59	6,88	3,75

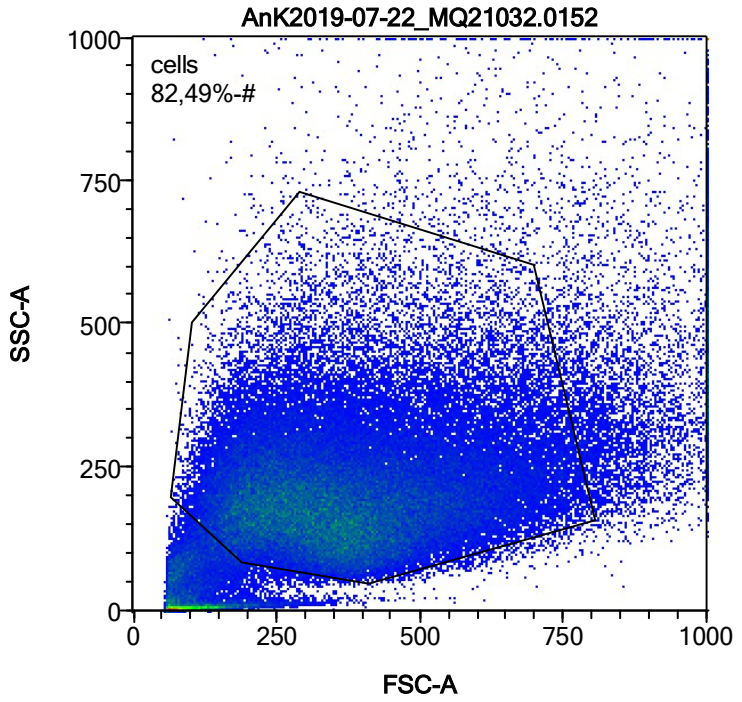

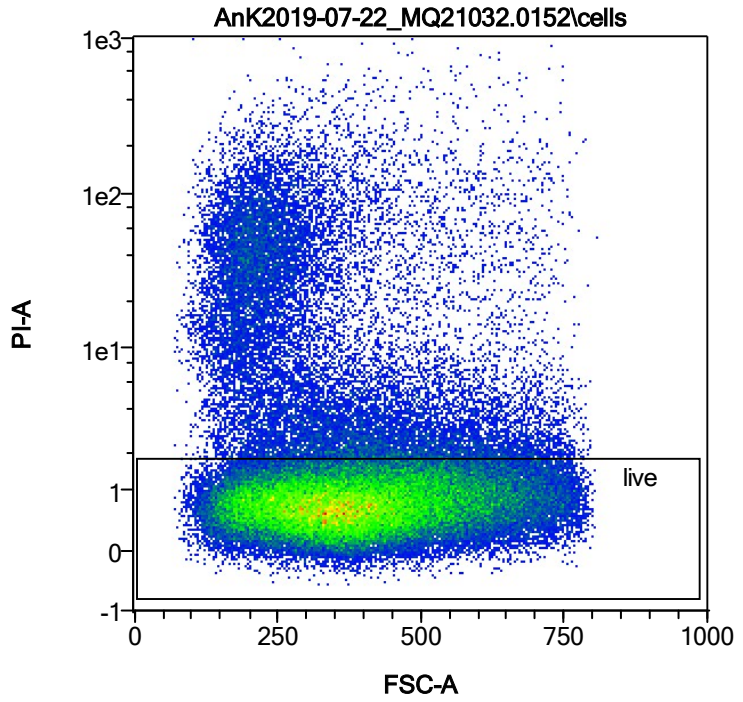

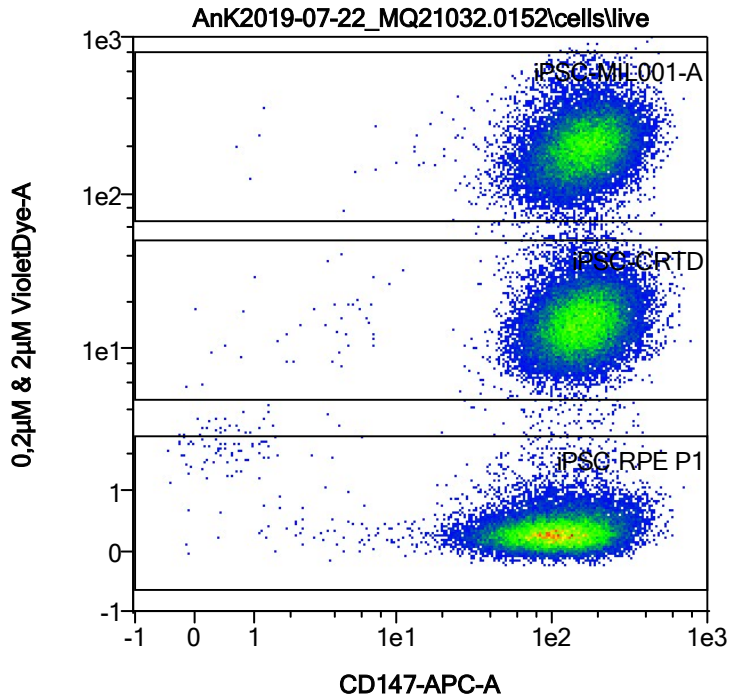

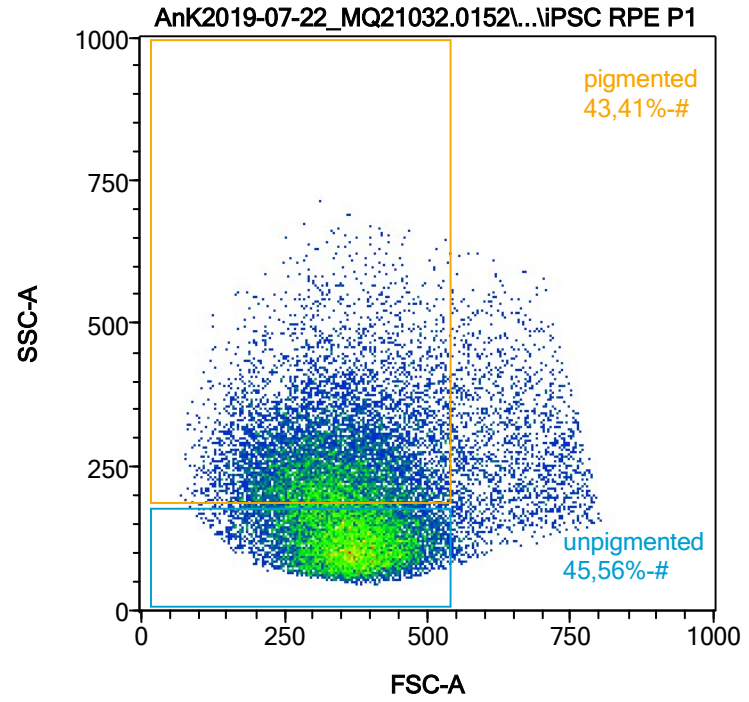

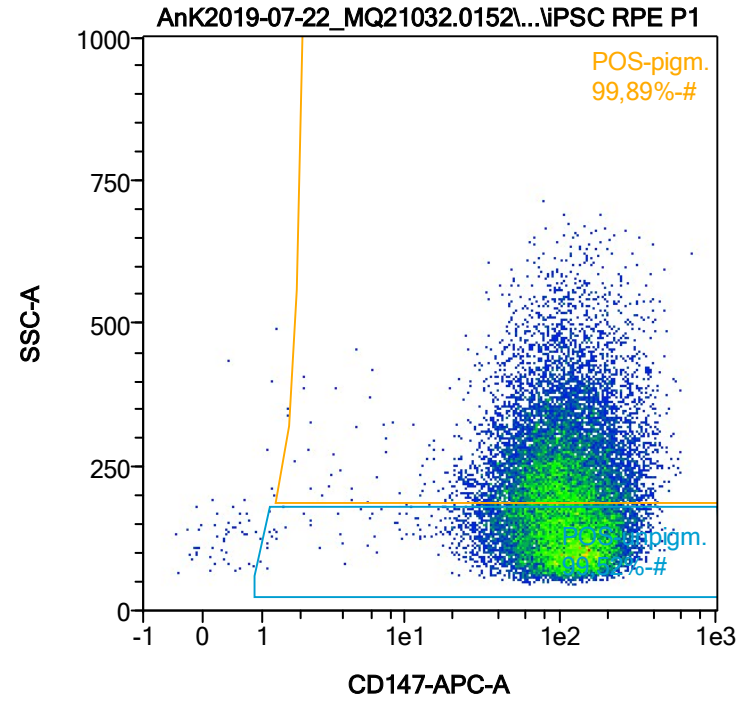

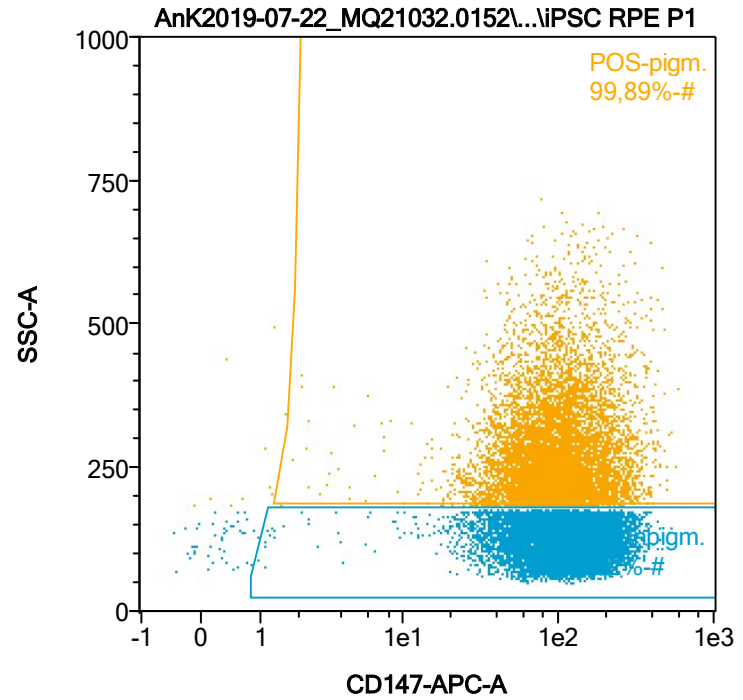

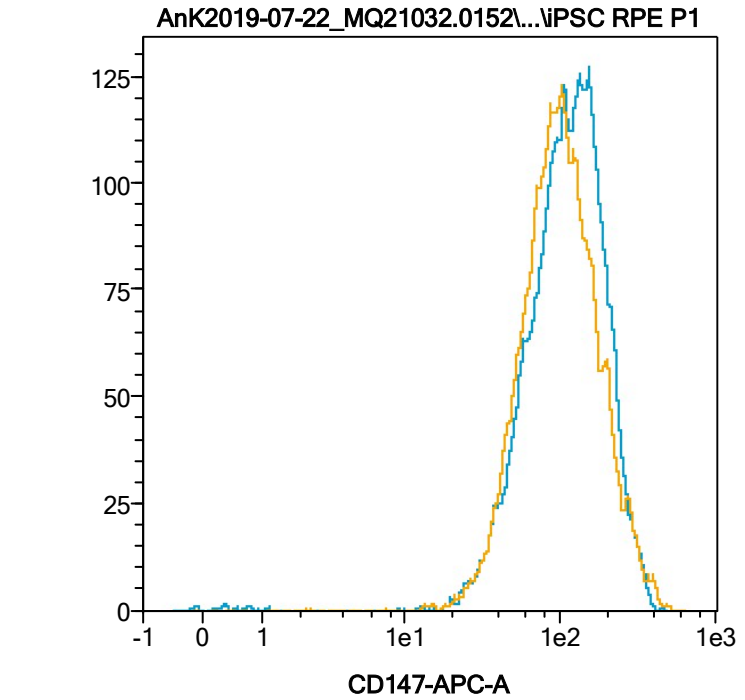

Name	%-#	CD147-APC-A Mean	CD147-APC-A Median
POS-unpigm.	99,52	122,10	111,69
POS-pigm.	99,89	112,97	96,92

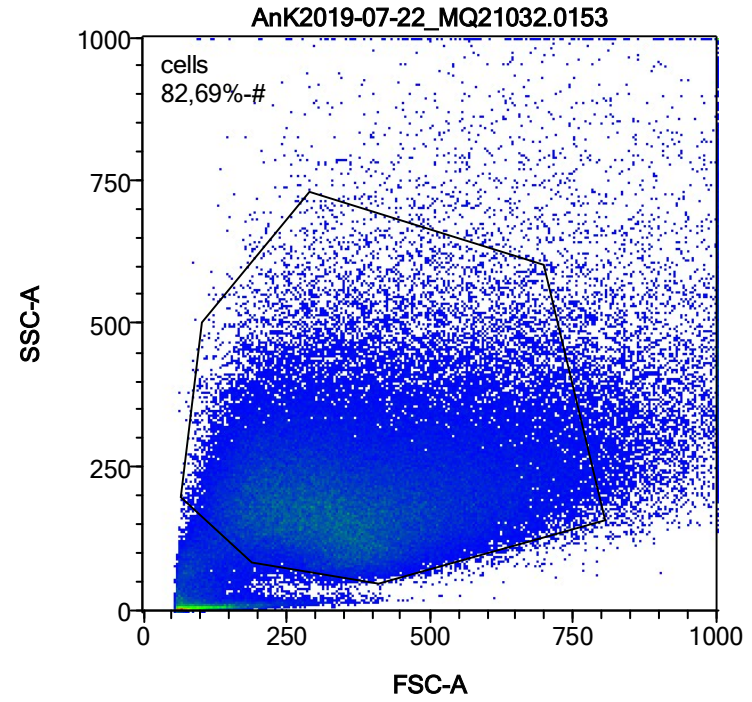

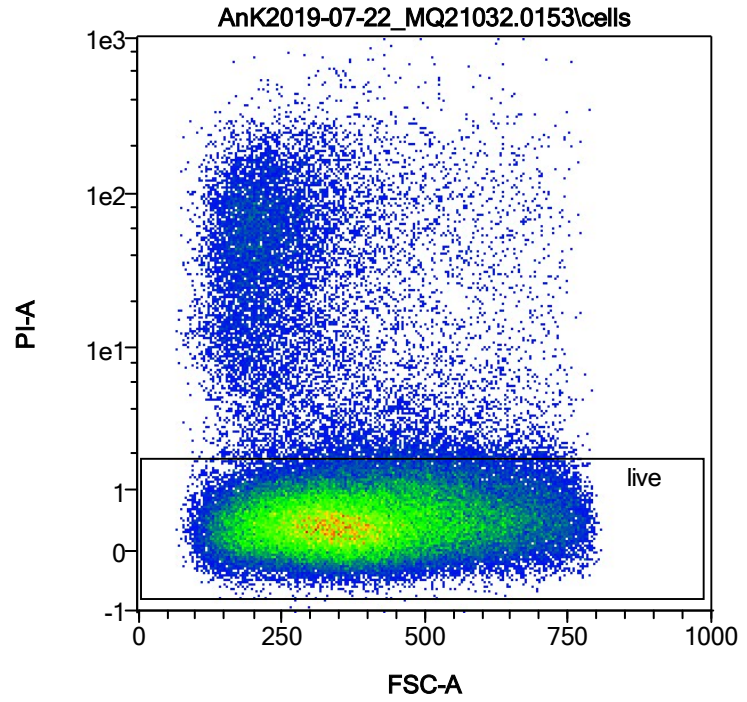

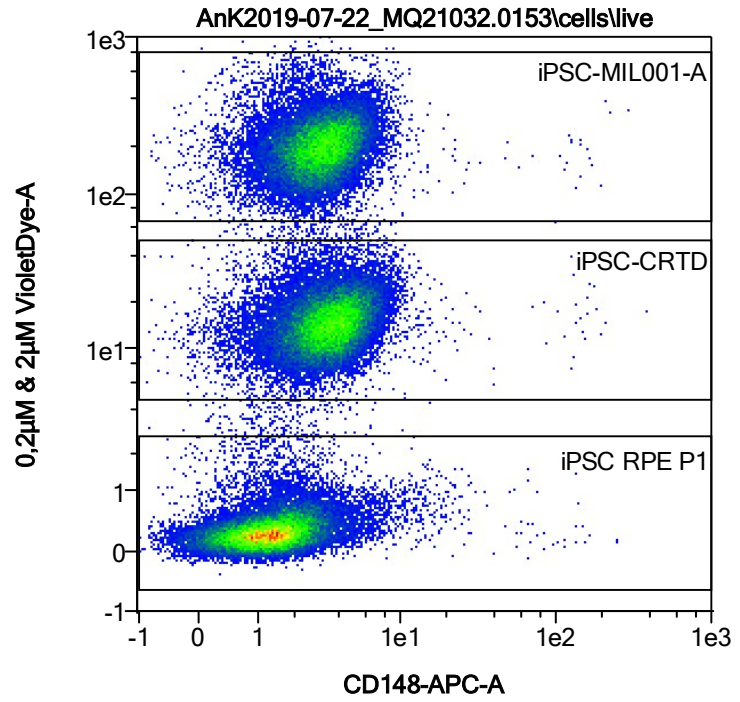

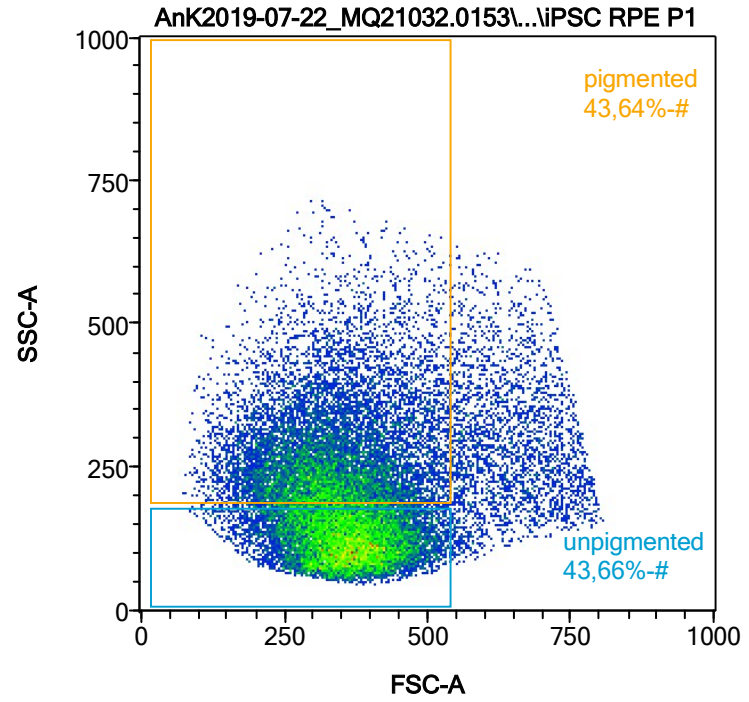

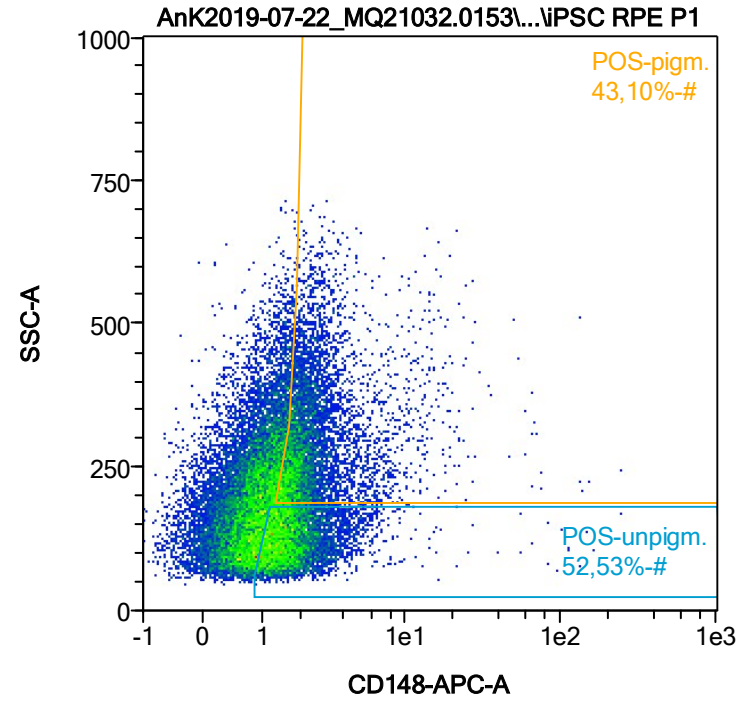

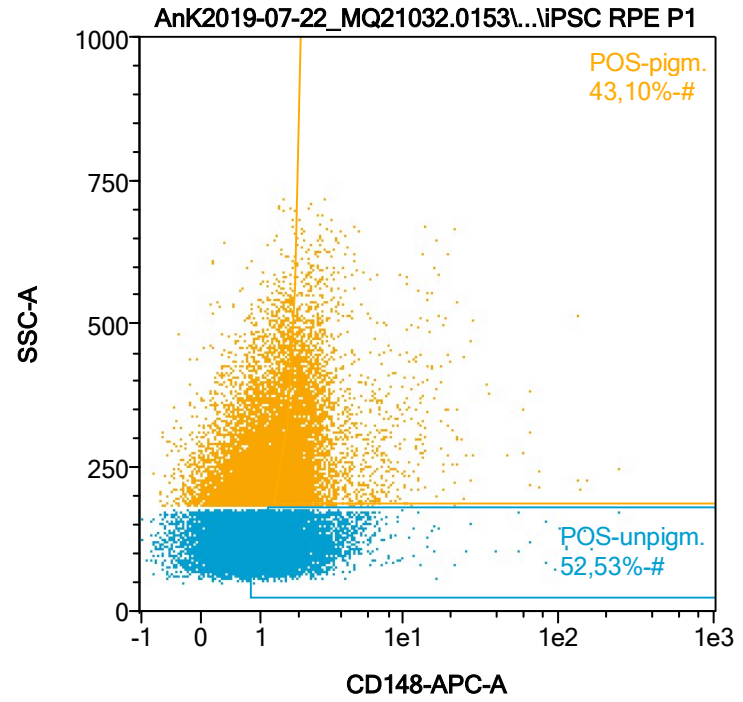

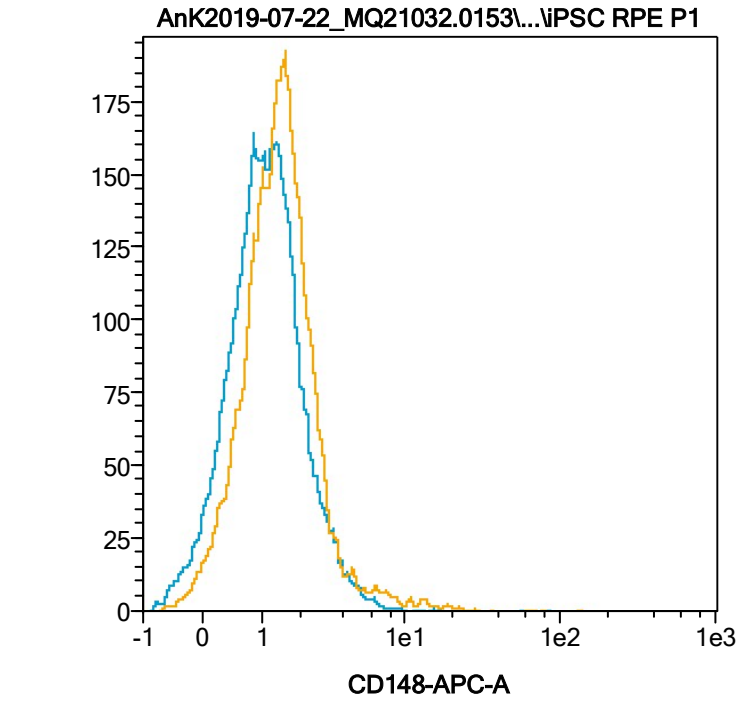

Name	%-#	CD148-APC-A Mean	CD148-APC-A Median
POS-unpigm.	52,53	2,11	1,56
POS-pigm.	43,10	2,95	2,04

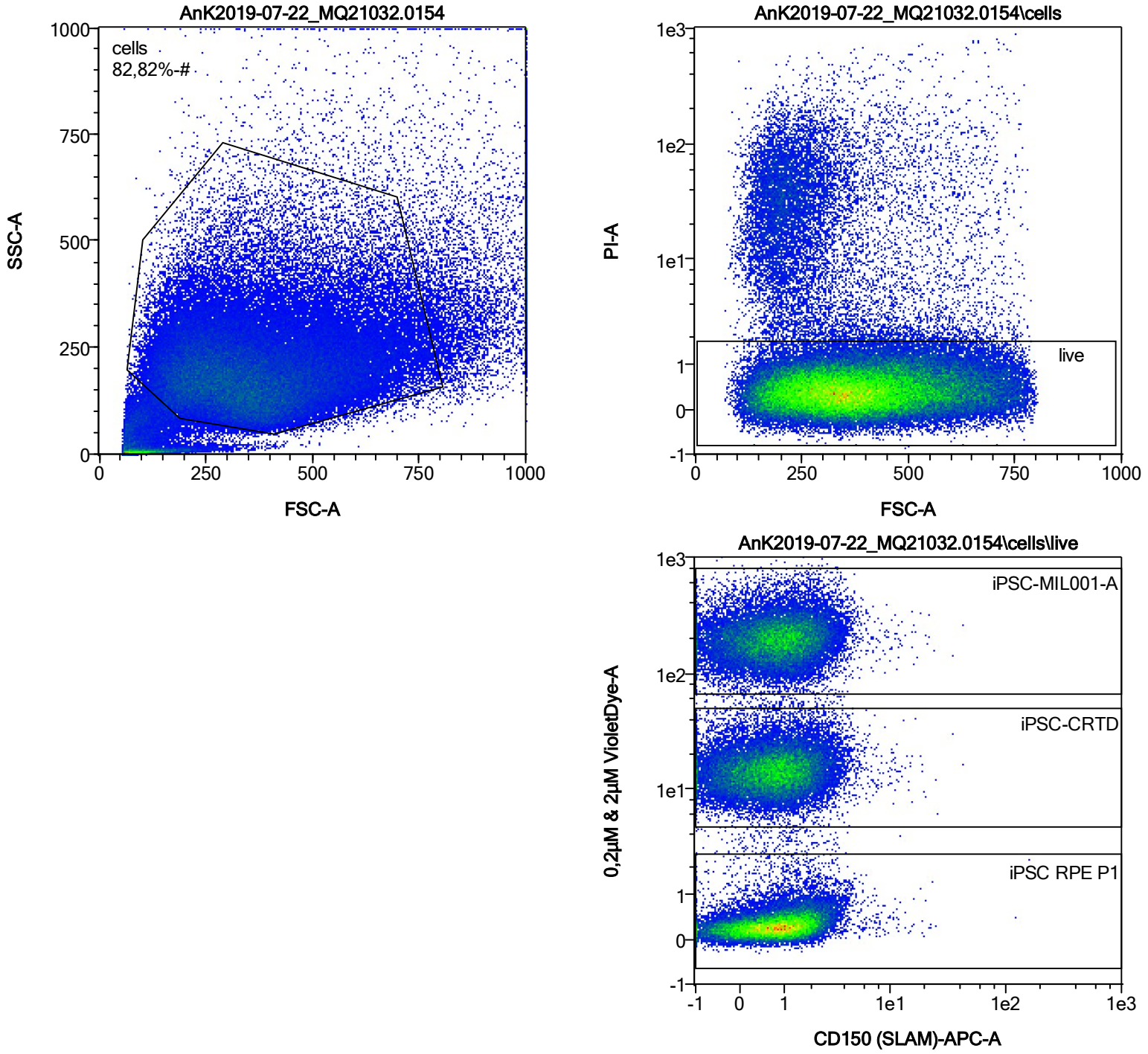

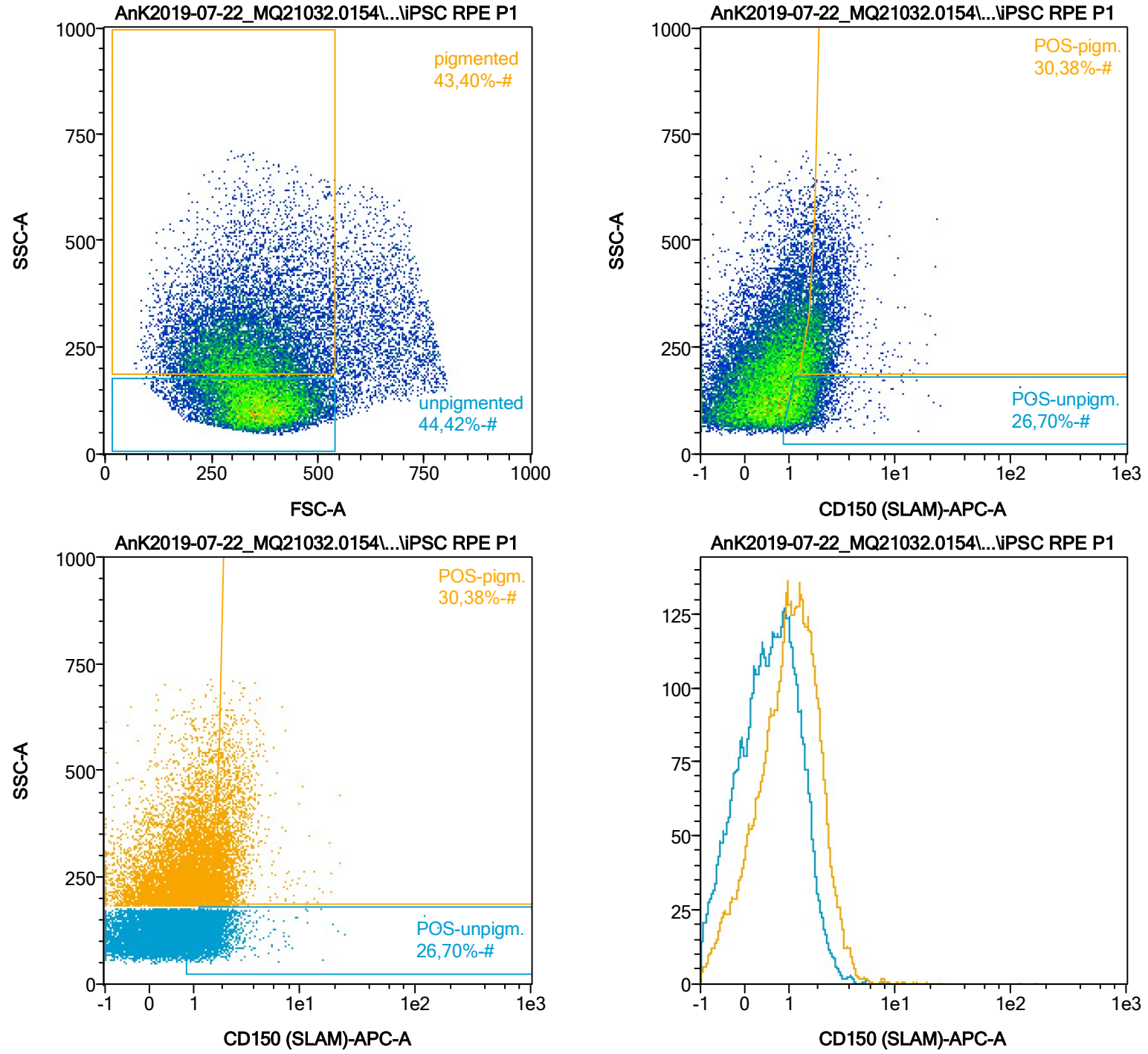

Name	%-#	CD150 (SLAM)-APC-A Mean	CD150 (SLAM)-APC-A Median
POS-unpigm.	26,70	1,70	1,41
POS-pigm.	30,38	2,27	2,02

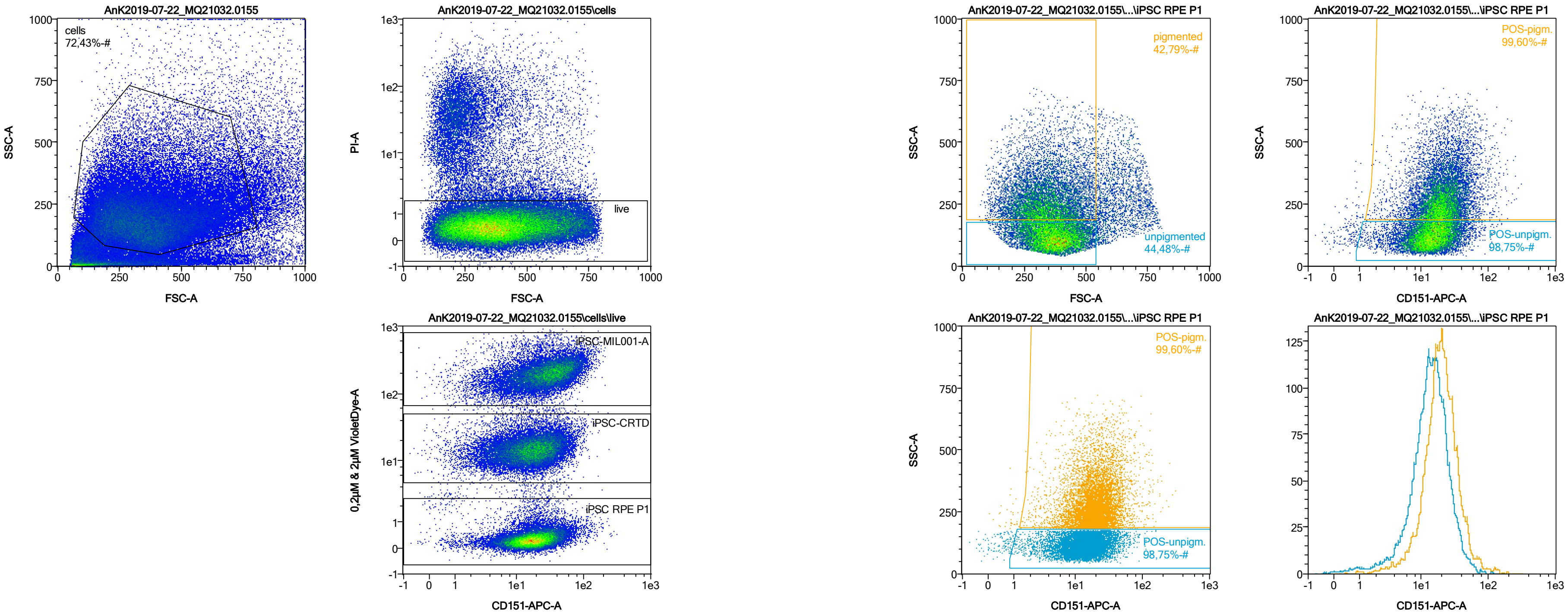

Name	%-#	CD151-APC-A Mean	CD151-APC-A Median
POS-unpigm.	98,75	16,08	13,97
POS-pigm.	99,60	22,93	19,47

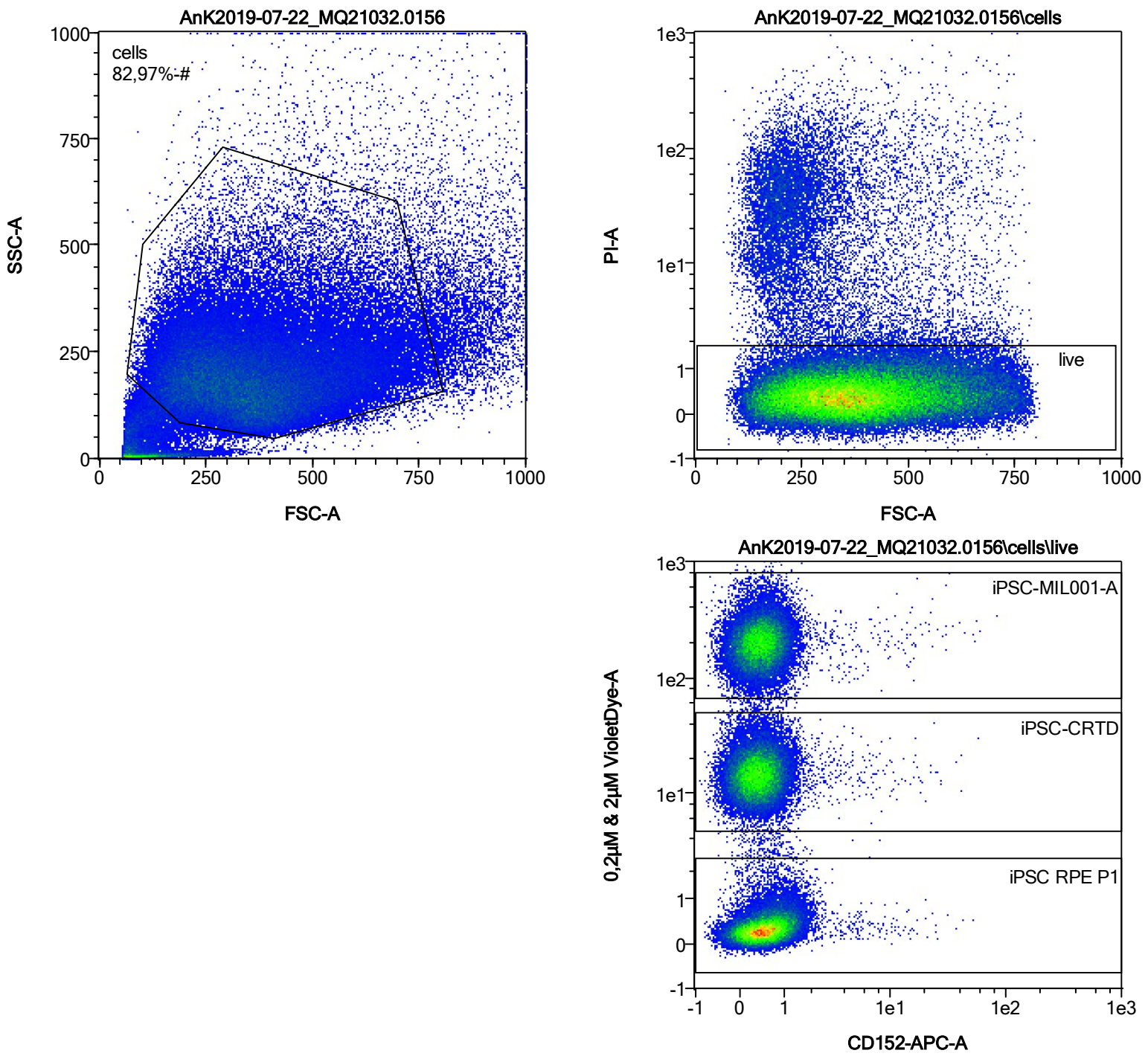

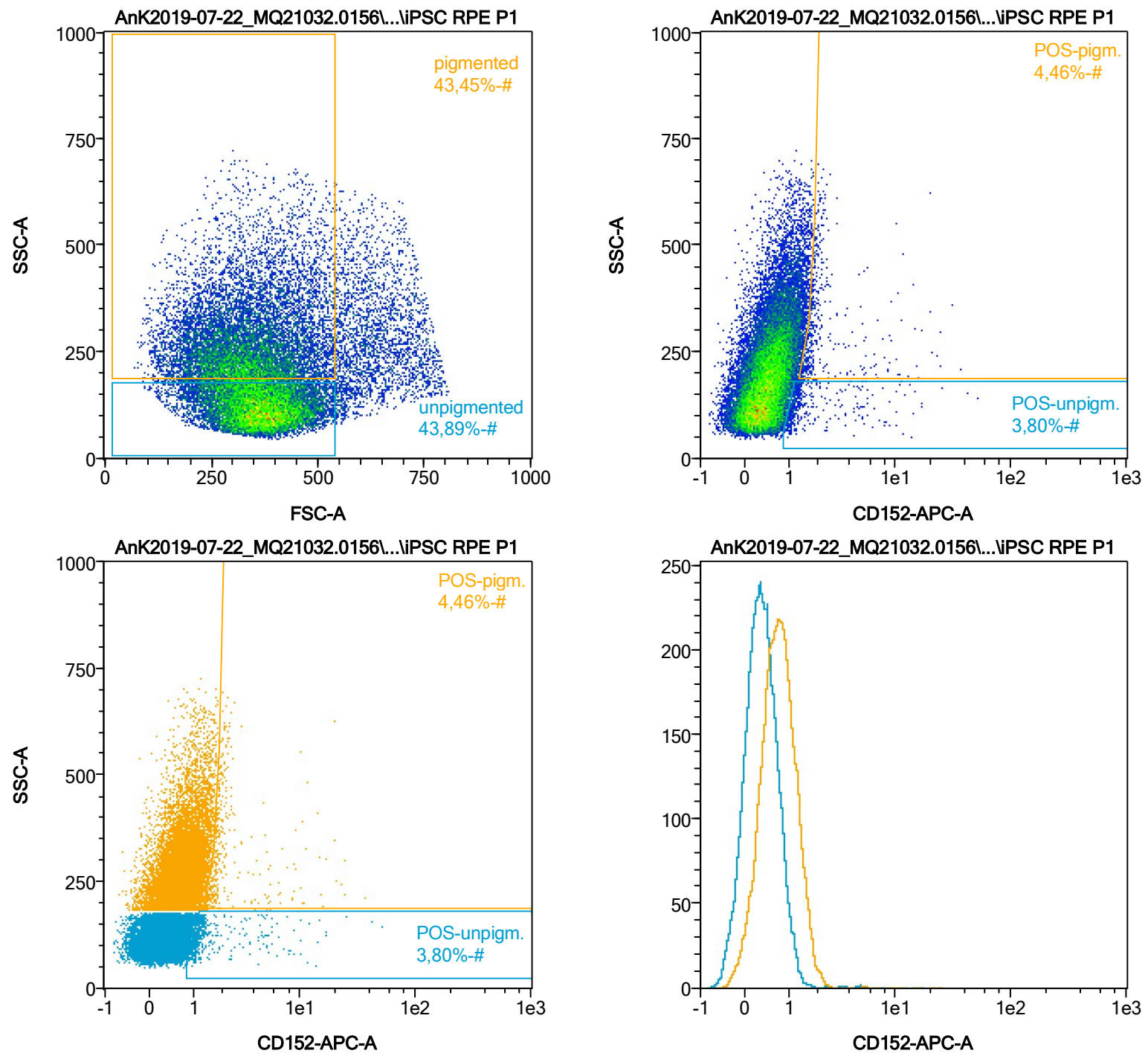

Name	%-#	CD152-APC-A Mean	CD152-APC-A Median
POS-unpigm.	3,80	2,23	1,18
POS-pigm.	4,46	2,68	1,80

Date:2024-06-14

Operator: Sebastian Knoebel

Version:2.13.0+54.g95abf0f4

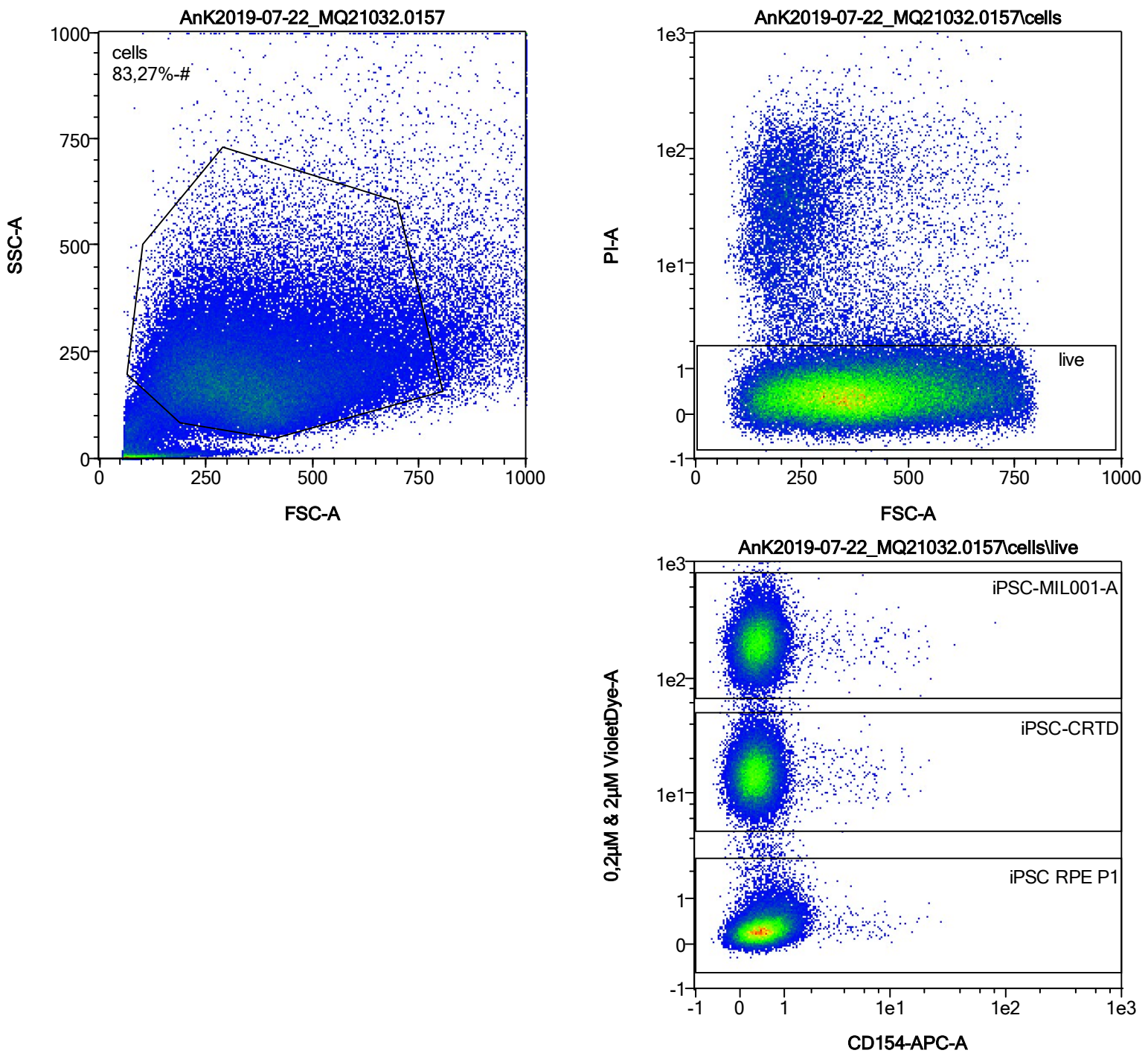

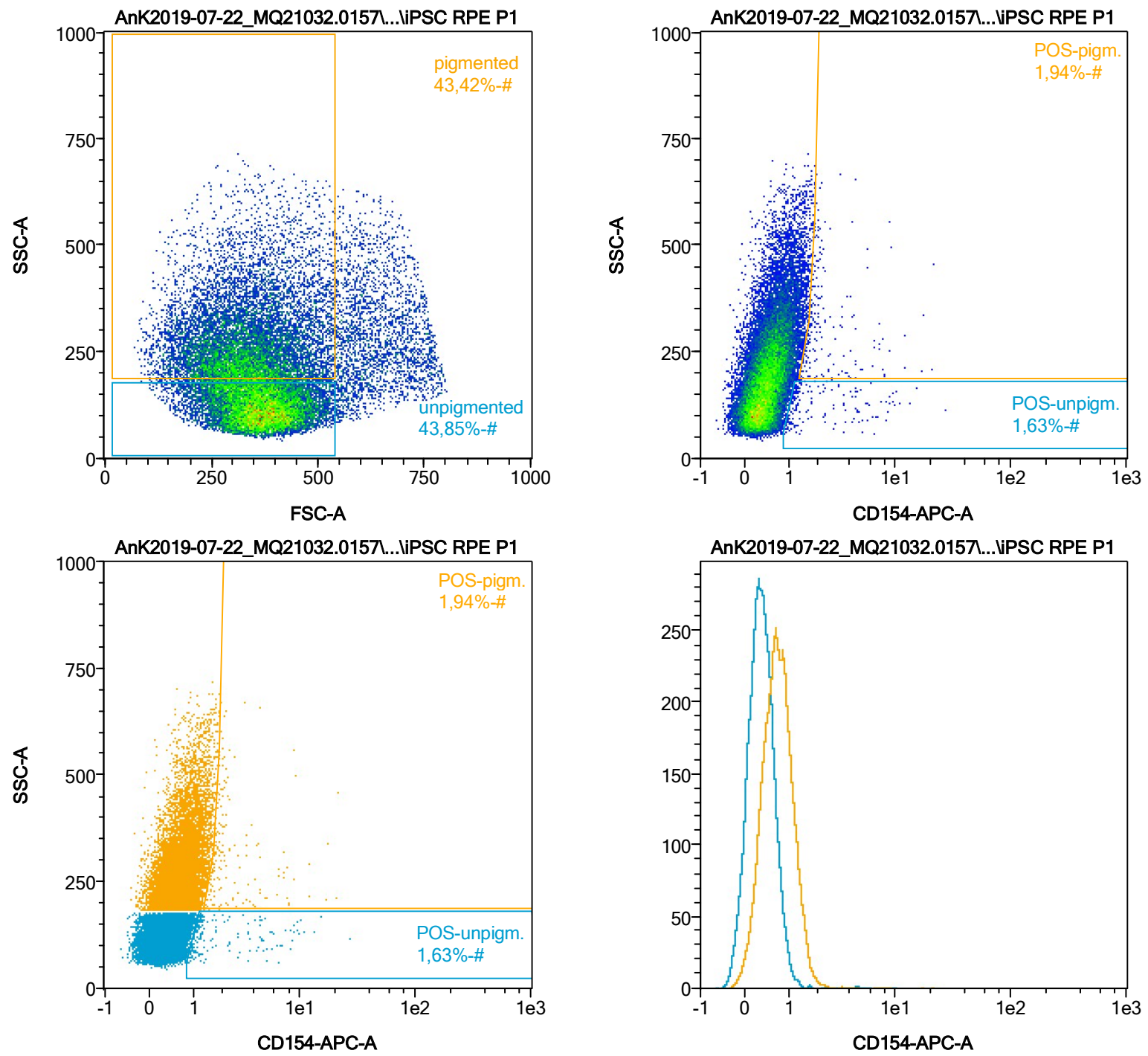

Name	%-#	CD154-APC-A Mean	CD154-APC-A Median
POS-unpigm.	1,63	2,72	1,26
POS-pigm.	1,94	2,86	1,91

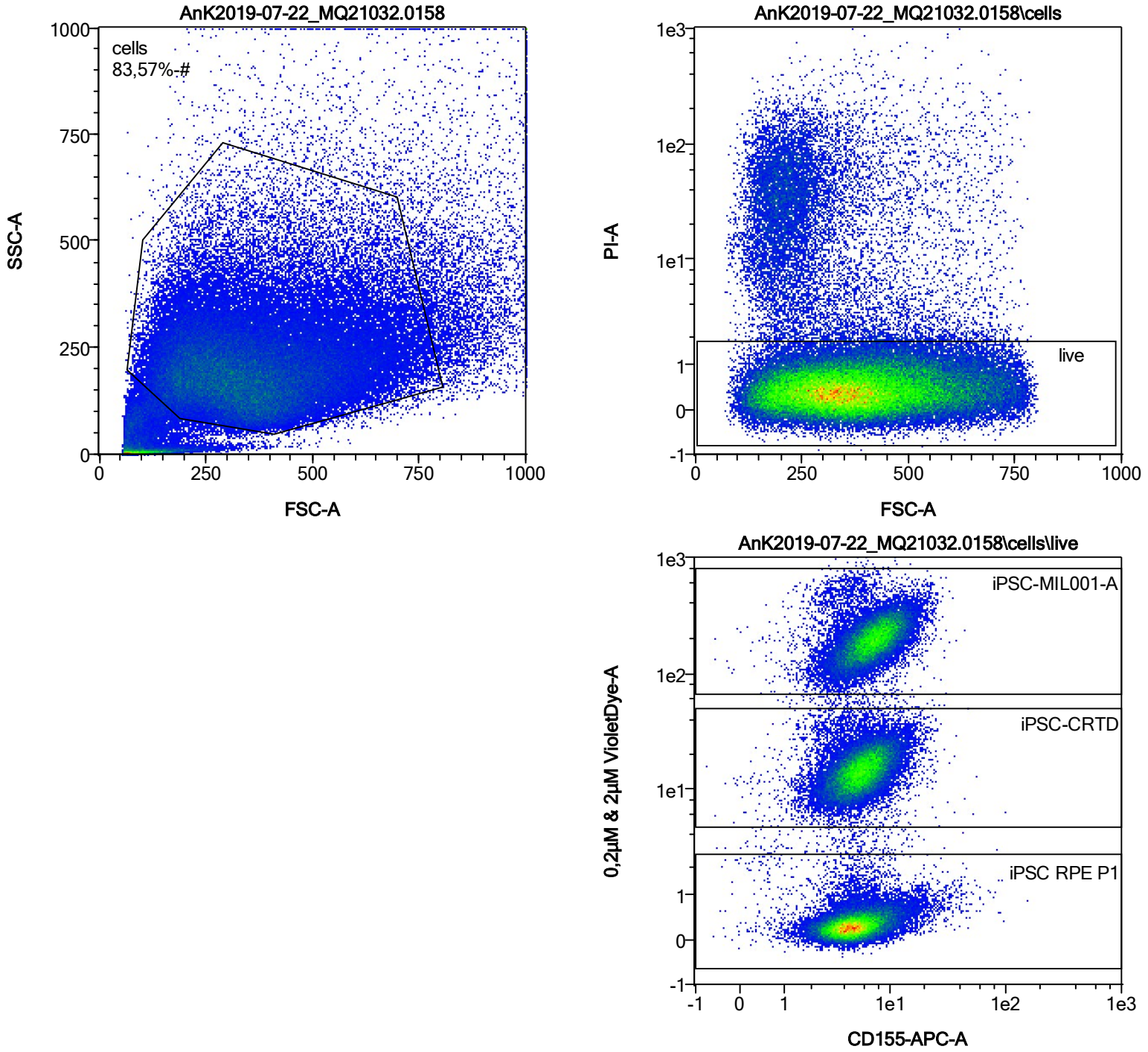

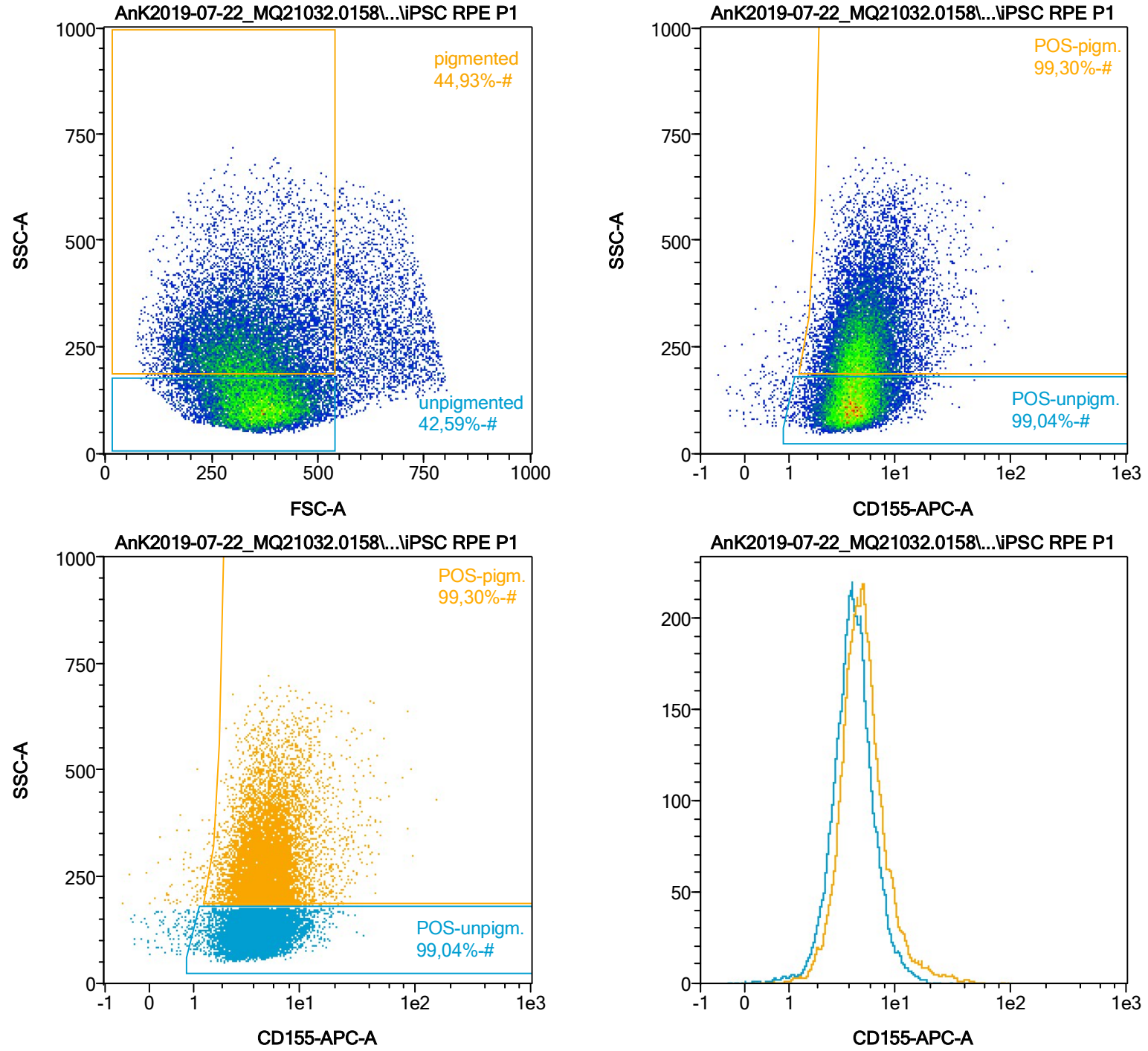

Name	%-#	CD155-APC-A Mean	CD155-APC-A Median
POS-unpigm.	99,04	4,63	4,23
POS-pigm.	99,30	5,95	5,02

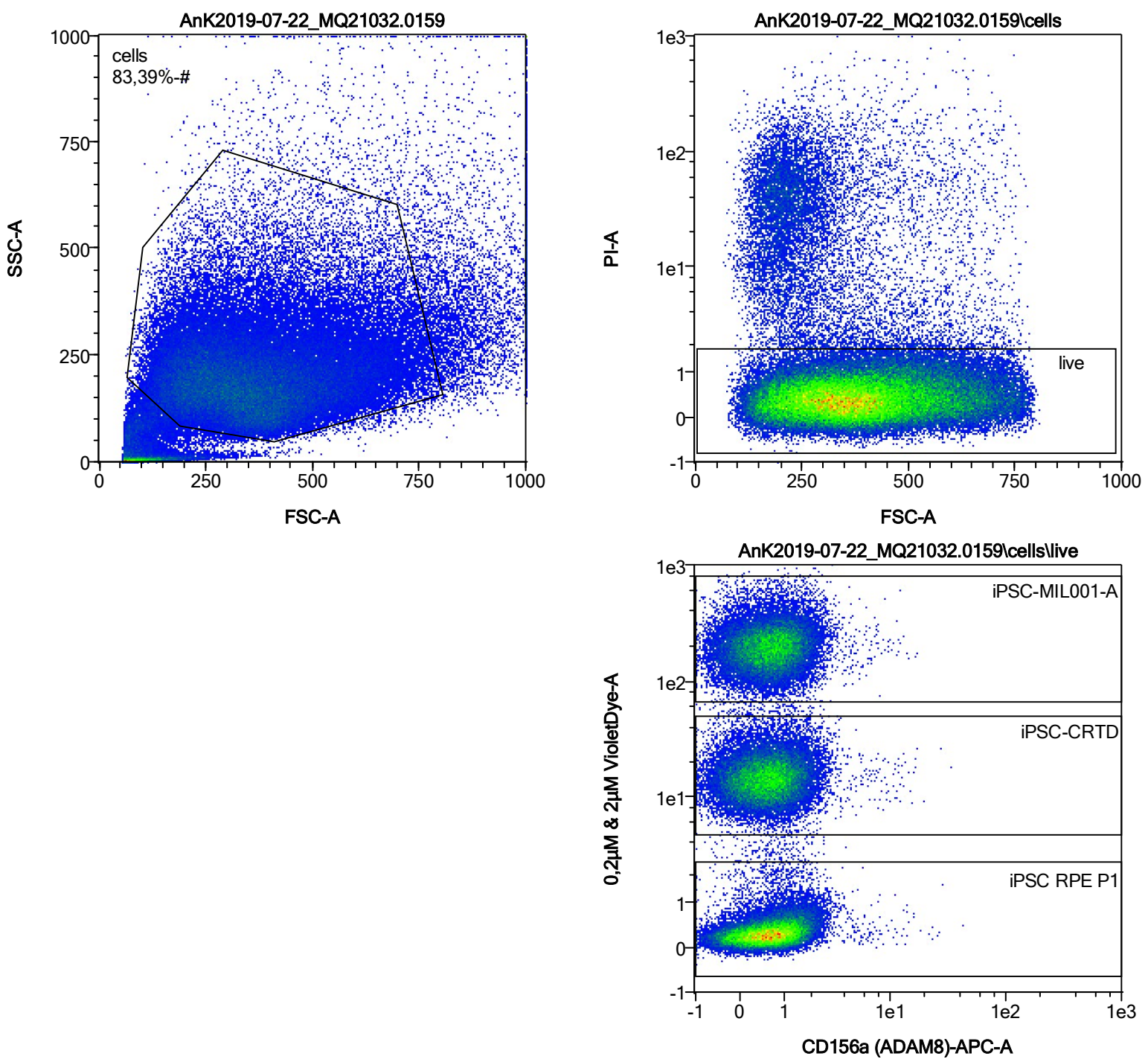

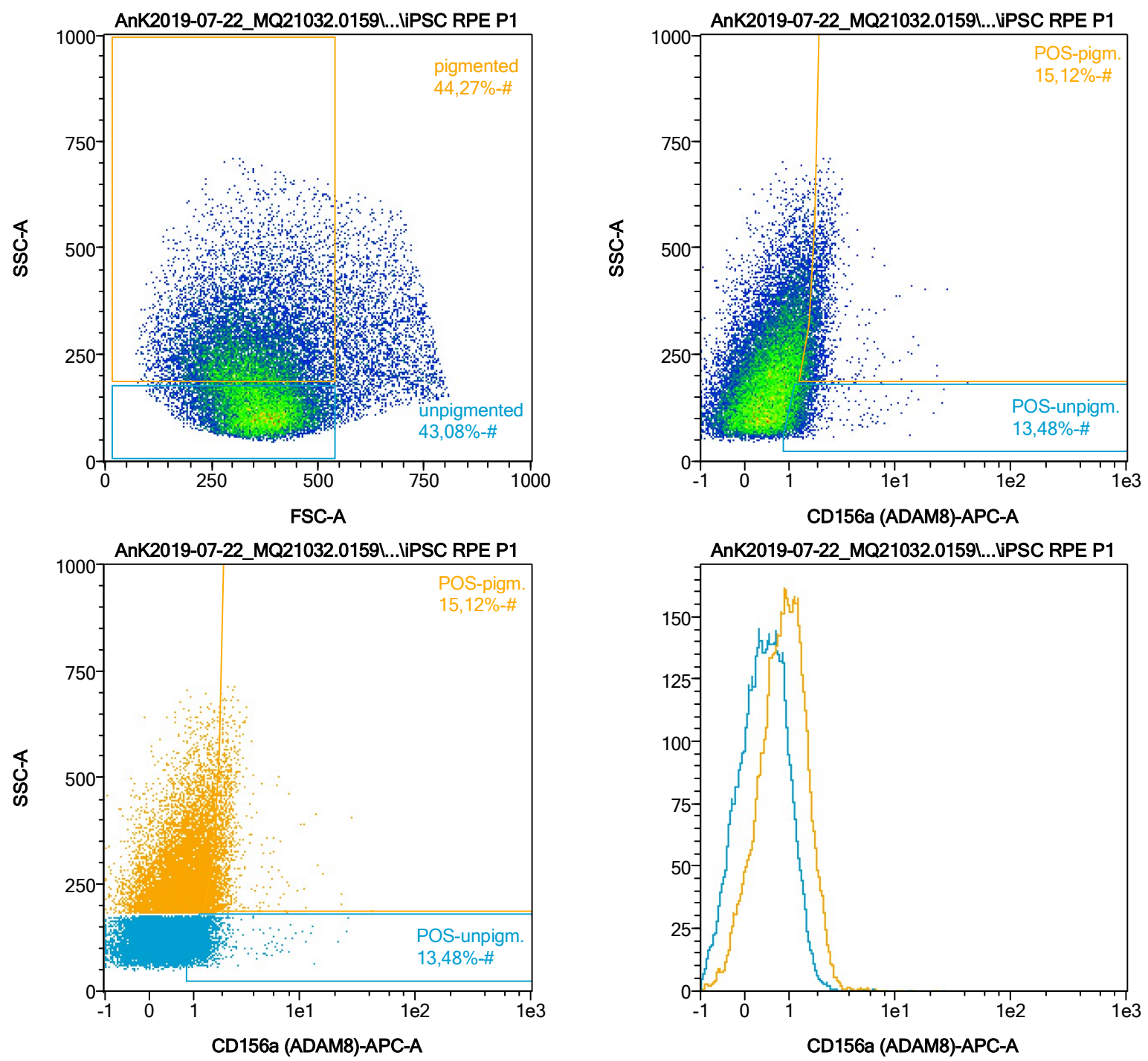

Name	%-#	CD156a (ADAM8)-APC-A Mean	CD156a (ADAM8)-APC-A Median
POS-unpigm.	13,48	1,51	1,26
POS-pigm.	15,12	2,14	1,88

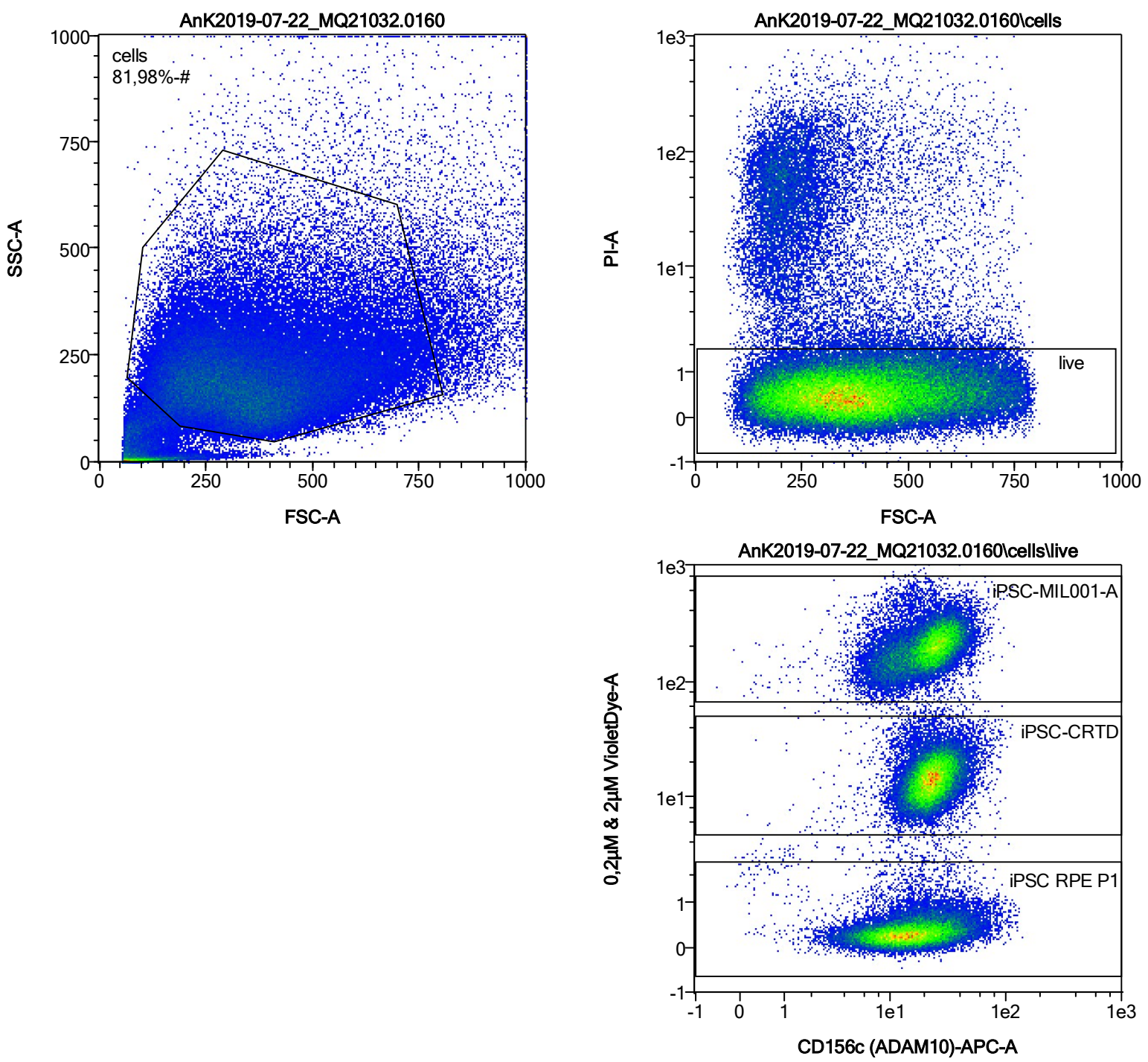

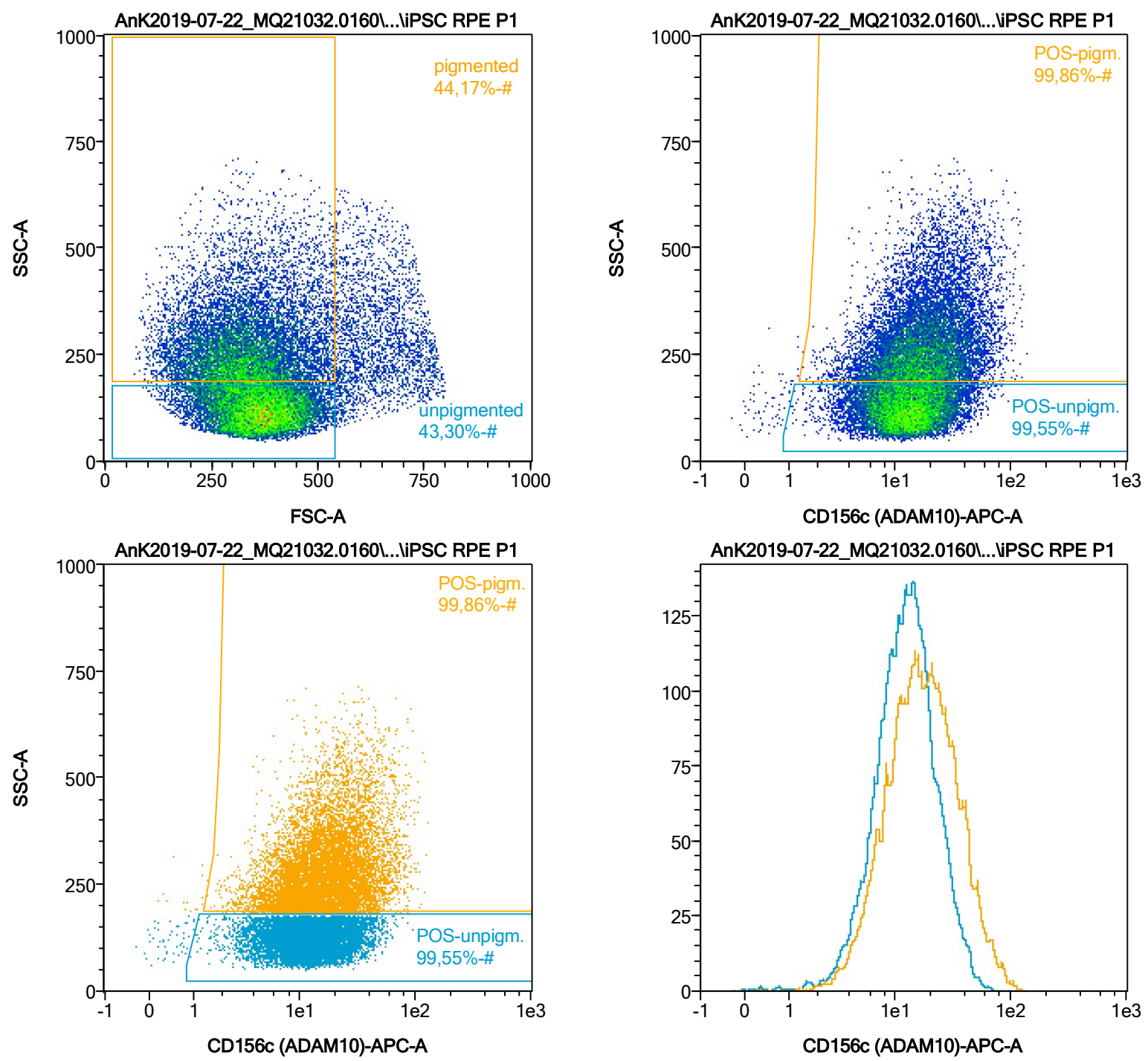

Name	%-#	CD156c (ADAM10)-APC-A Mean	CD156c (ADAM10)-APC-A Median
POS-unpigm.	99,55	14,34	12,48
POS-pigm.	99,86	20,92	16,67

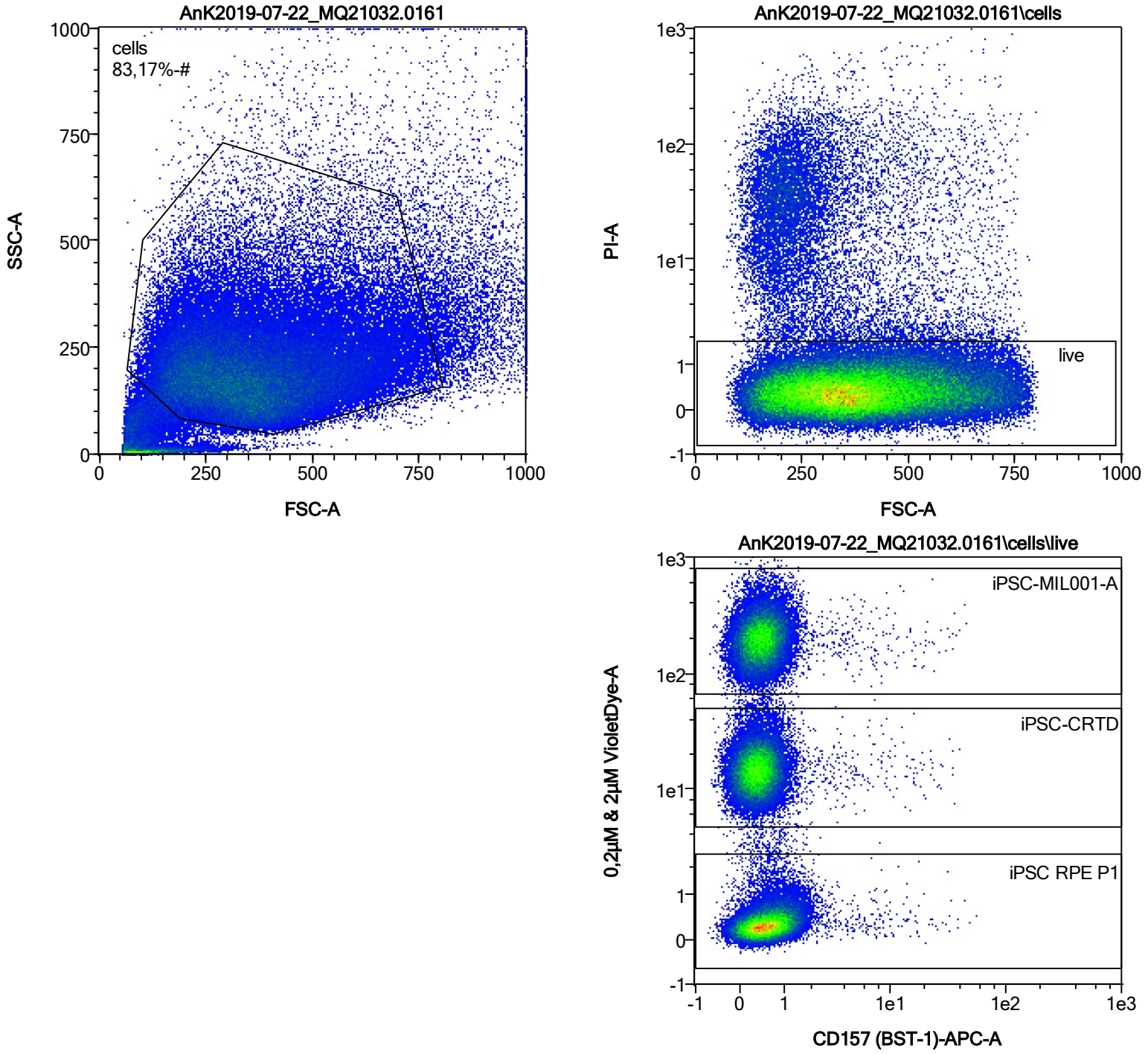

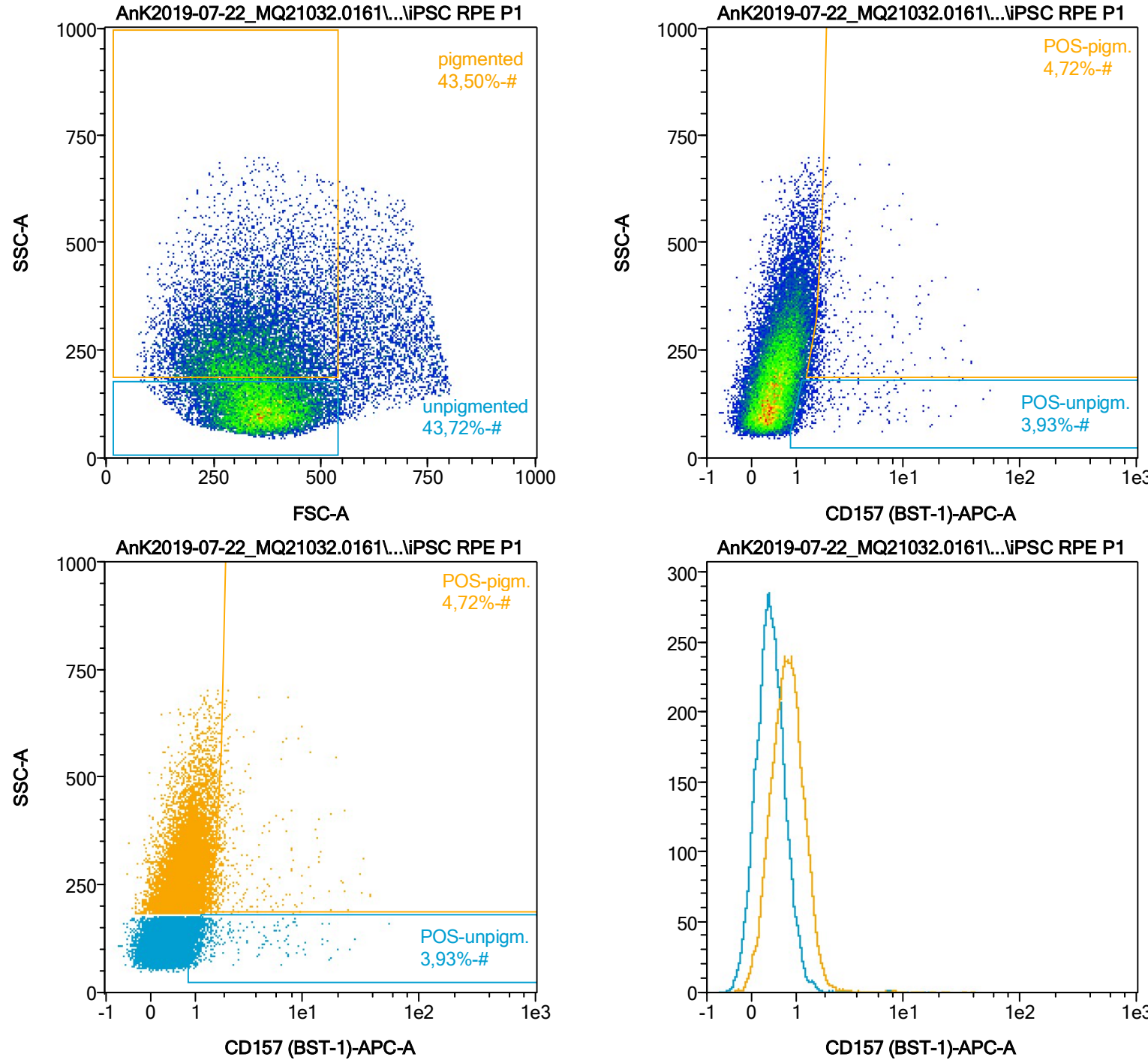

Name	%-#	CD157 (BST-1)-APC-A Mean	CD157 (BST-1)-APC-A Median
POS-unpigm.	3,93	2,31	1,20
POS-pigm.	4,72	3,16	1,87

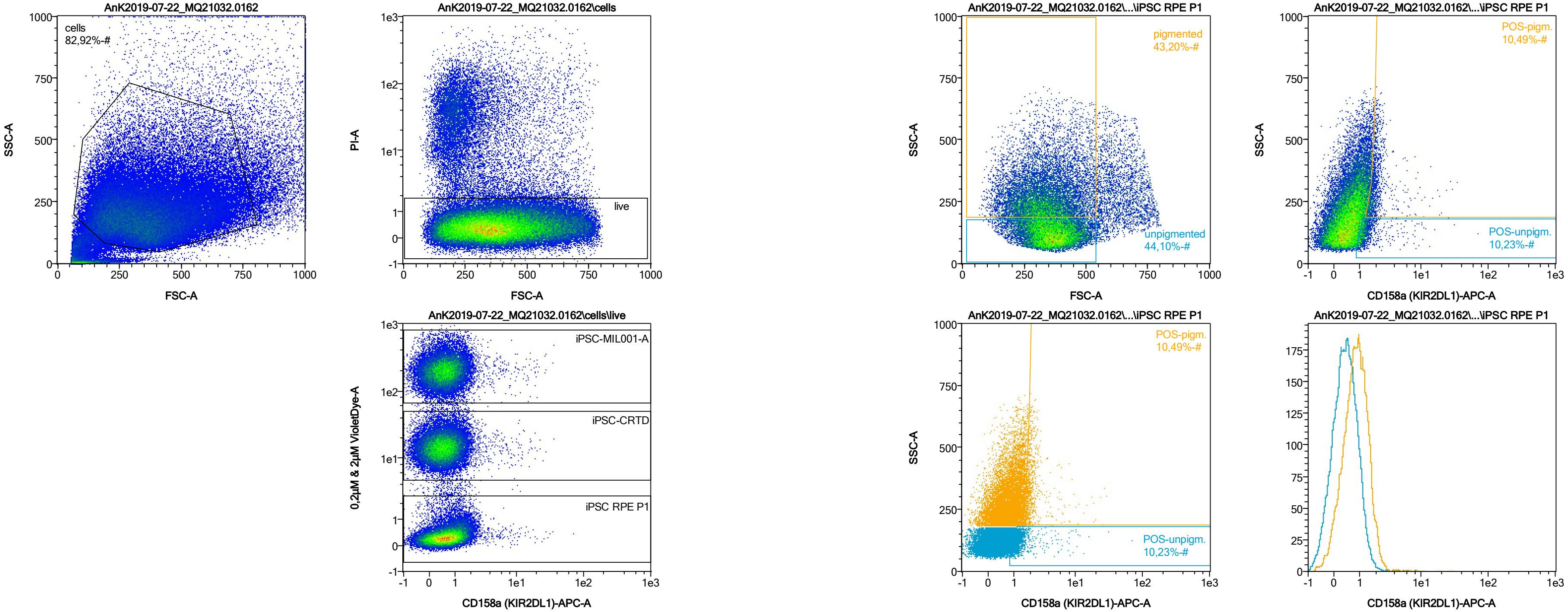

Name	%-#	CD158a (KIR2DL1)-APC-A Mean	CD158a (KIR2DL1)-APC-A Median
POS-unpigm.	10,23	1,56	1,21
POS-pigm.	10,49	2,18	1,81

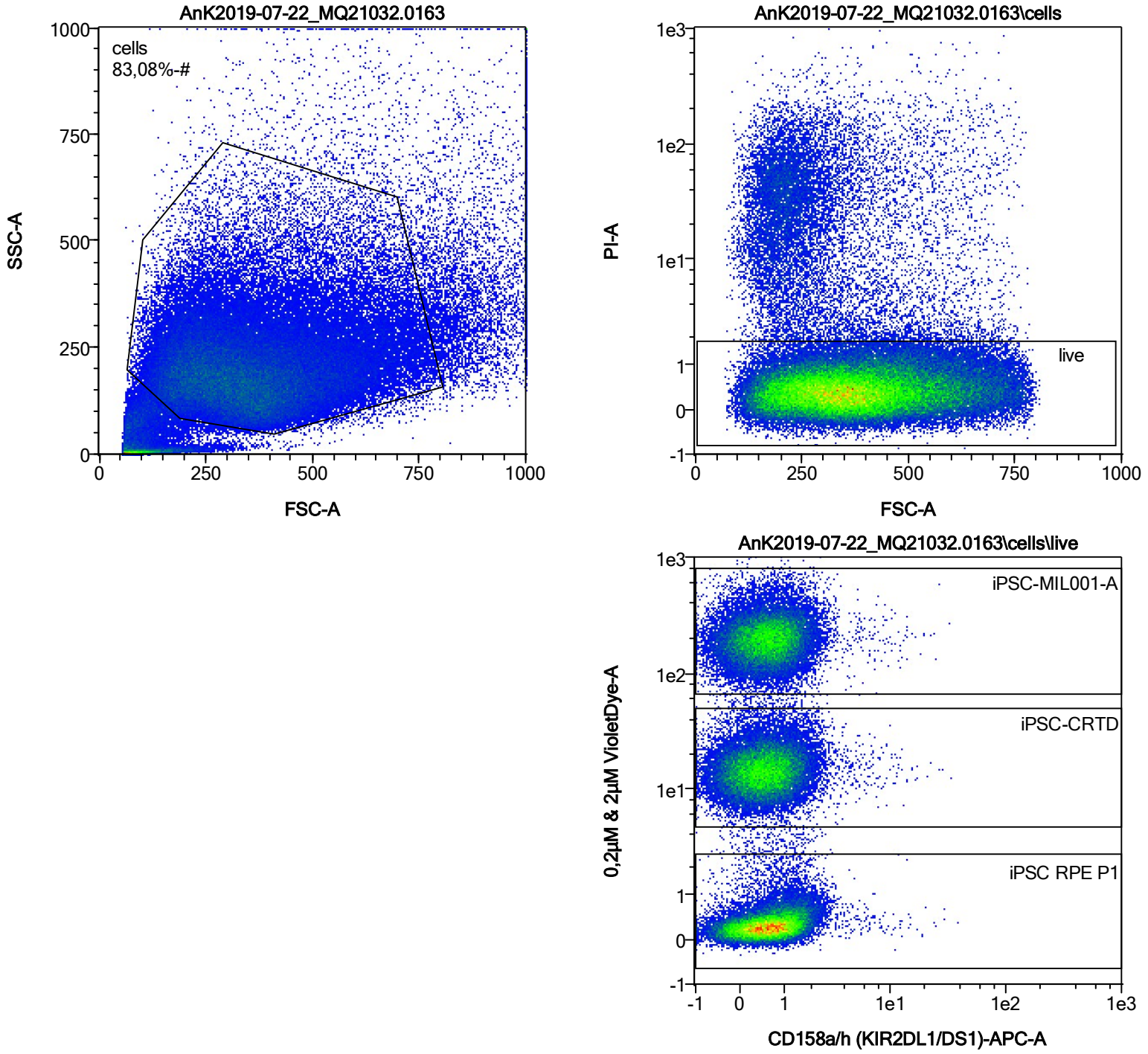

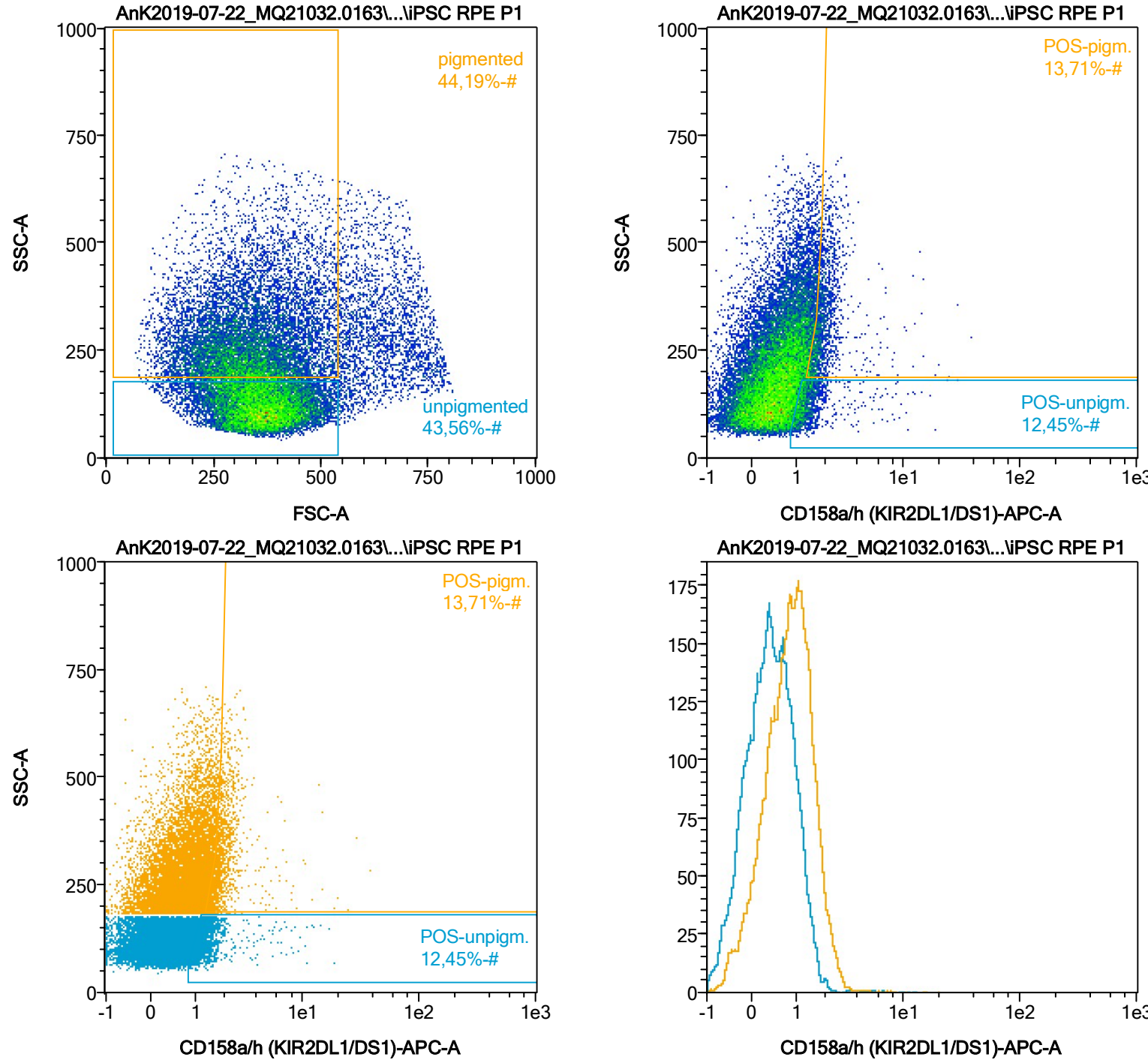

Name	%-#	CD158a/h (KIR2DL1/DS1)-APC-A Mean	CD158a/h (KIR2DL1/DS1)-APC-A Median
POS-unpigm.	12,45	1,49	1,24
POS-pigm.	13,71	2,10	1,83

Date:2024-06-14

Operator: Sebastian Knoebel

Version:2.13.0+54.g95abf0f4

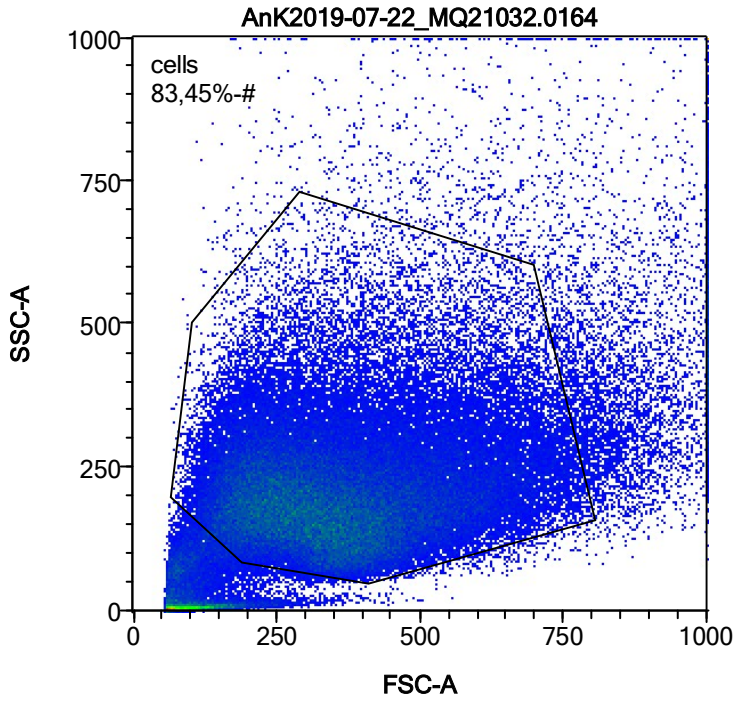

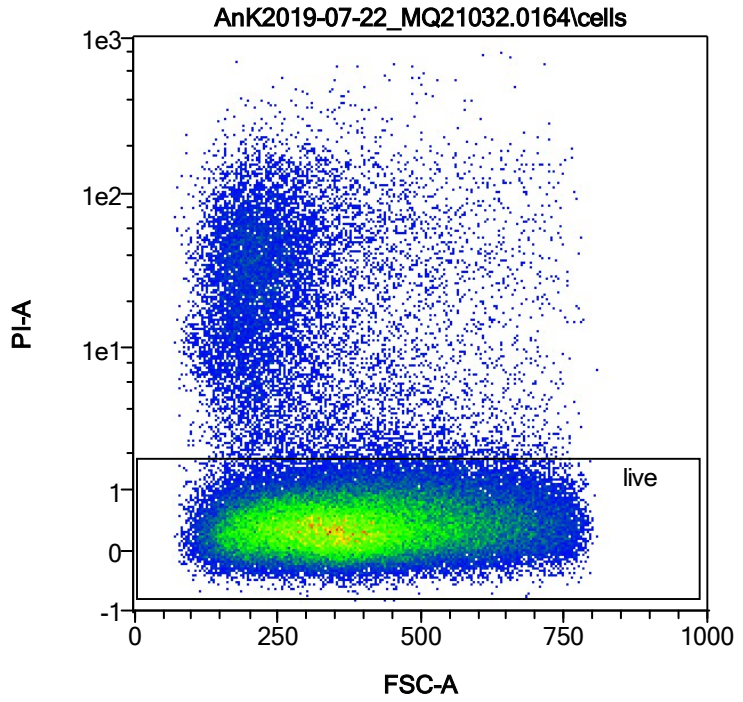

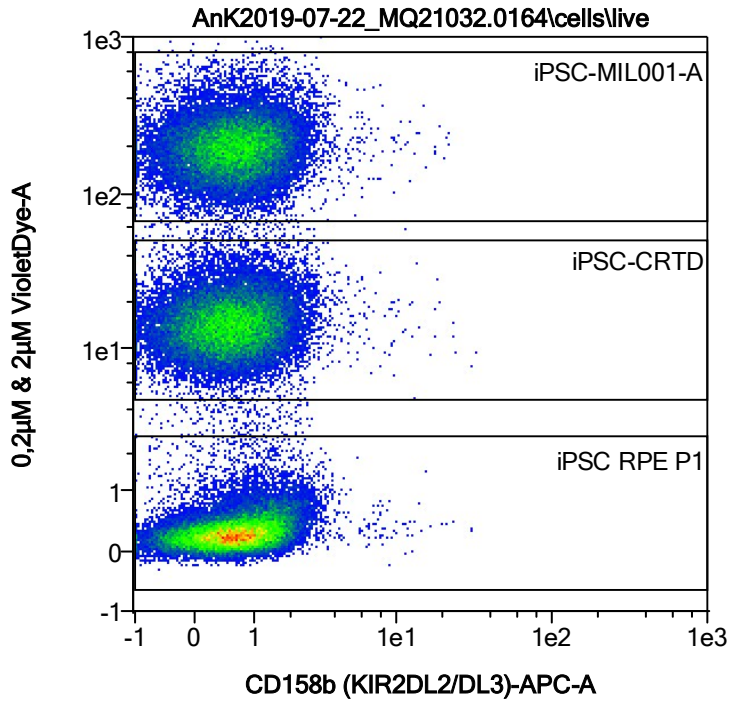

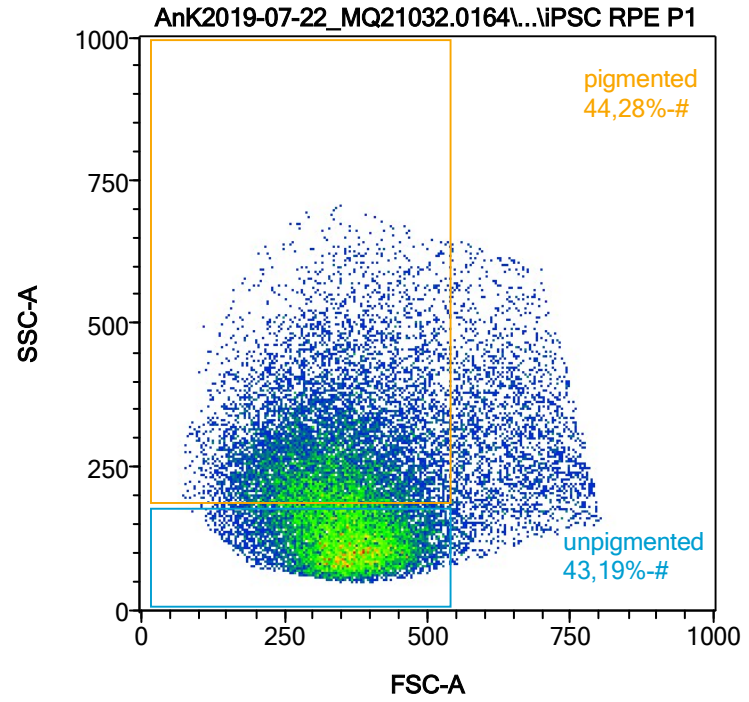

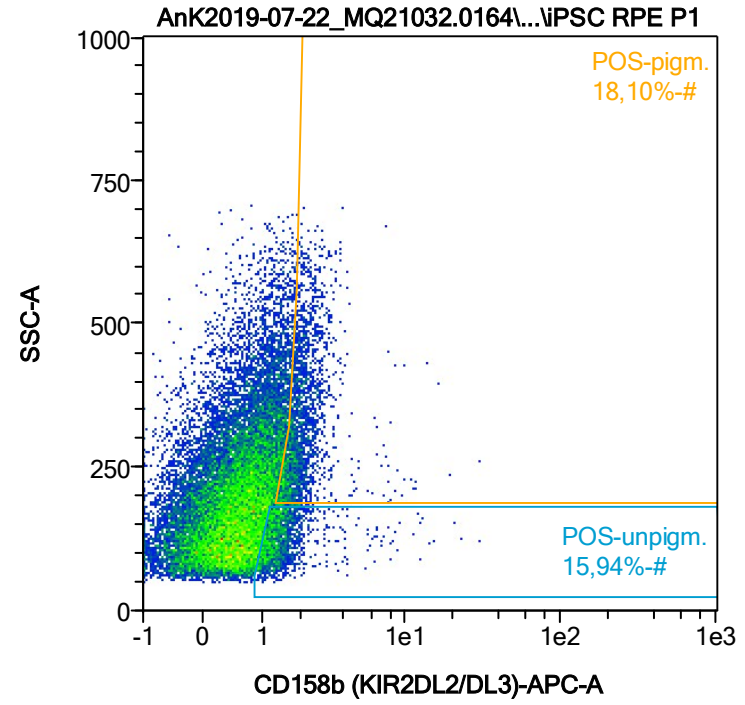

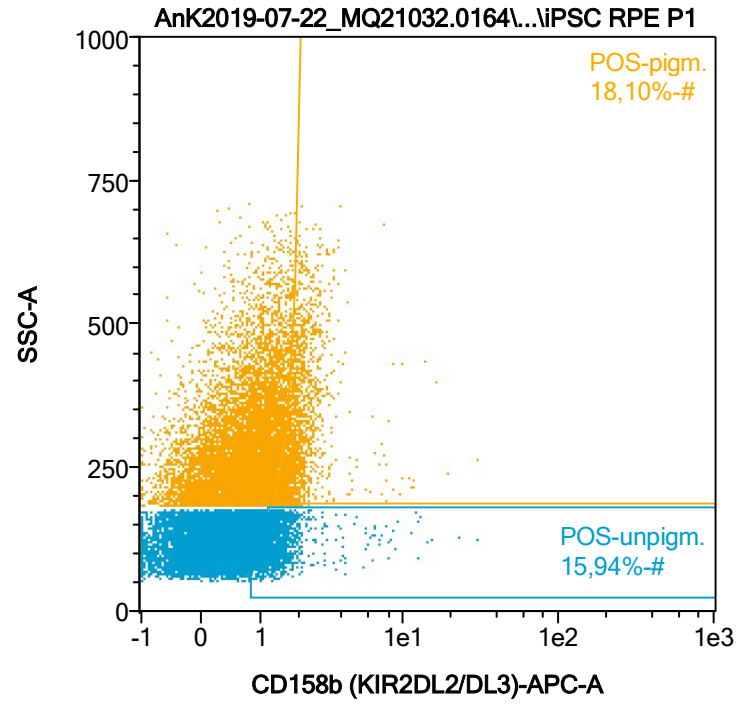

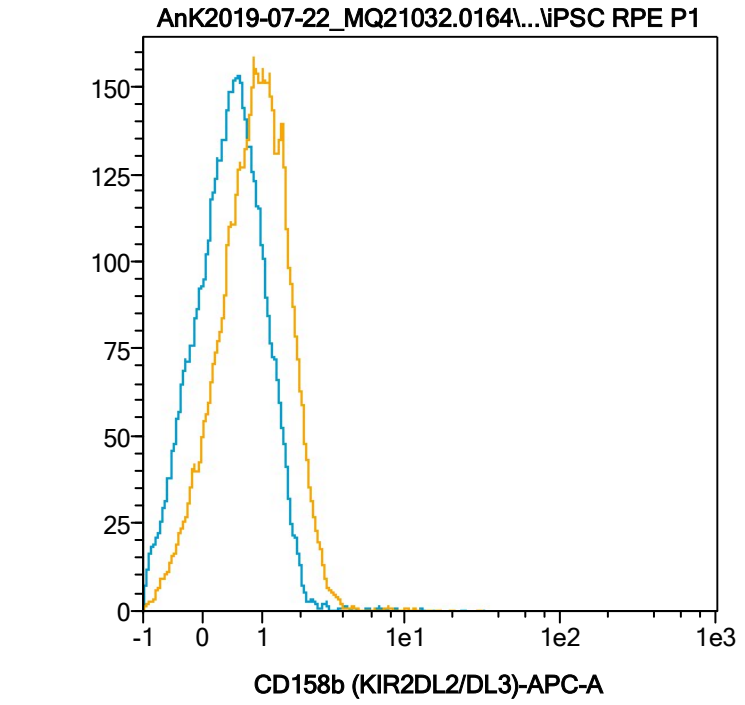

Name	%-#	CD158b (KIR2DL2/DL3)-APC-A Mean	CD158b (KIR2DL2/DL3)-APC-A Median
POS-unpigm.	15,94	1,54	1,30
POS-pigm.	18,10	2,10	1,89

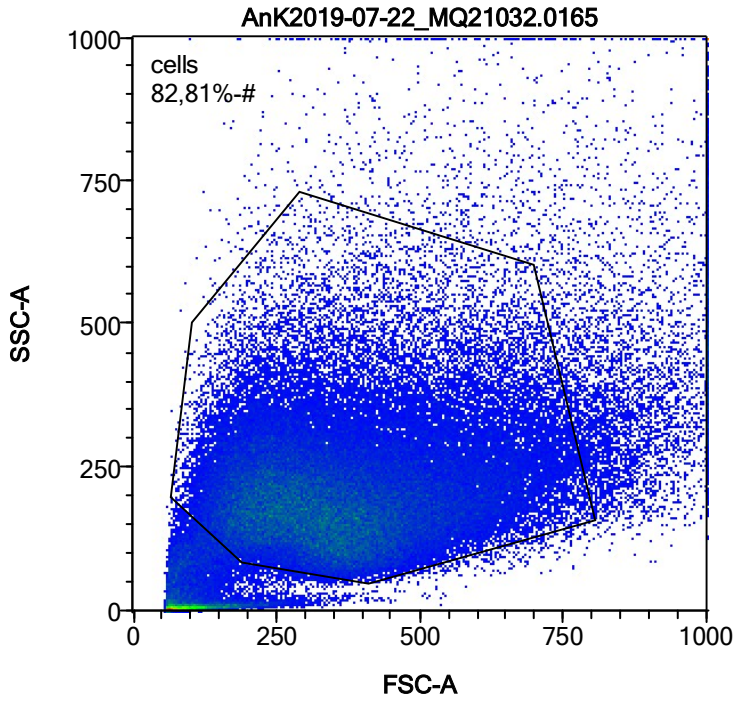

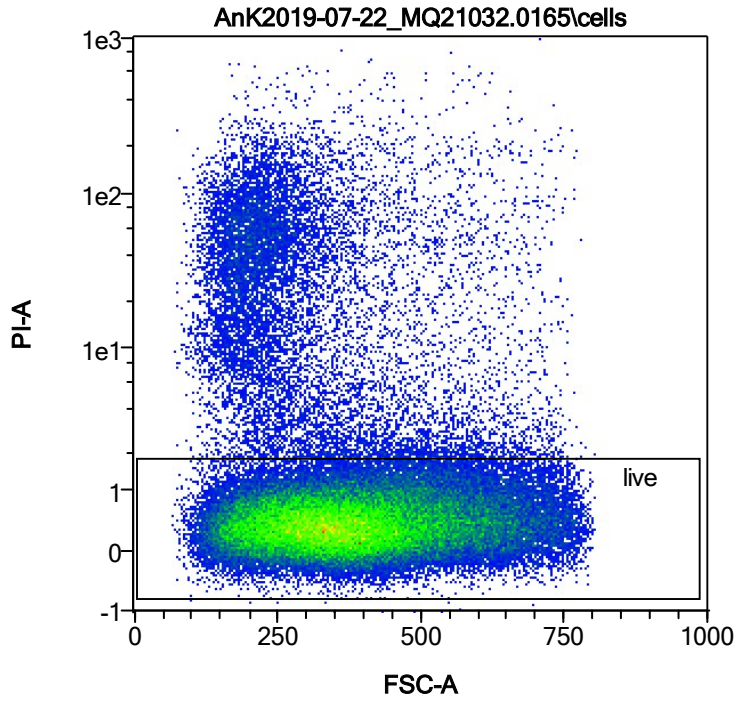

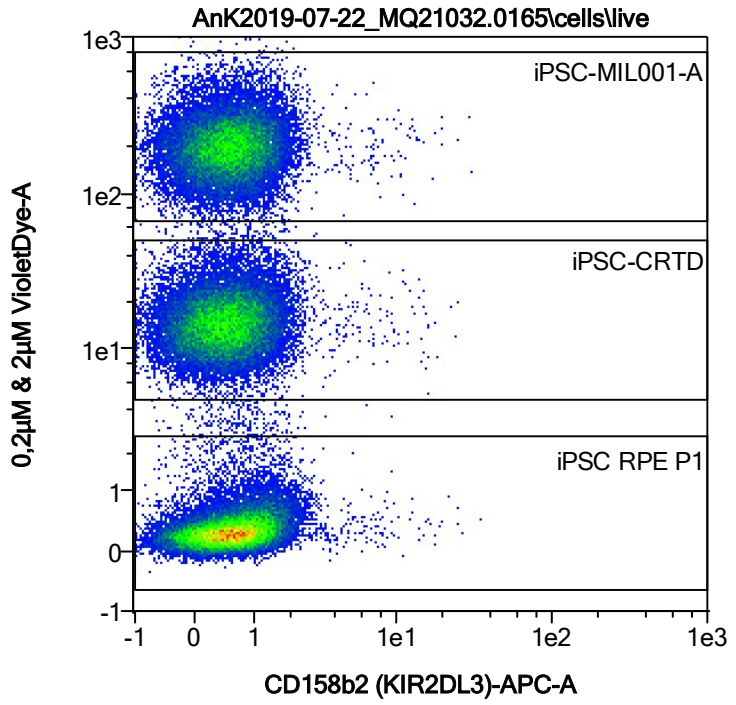

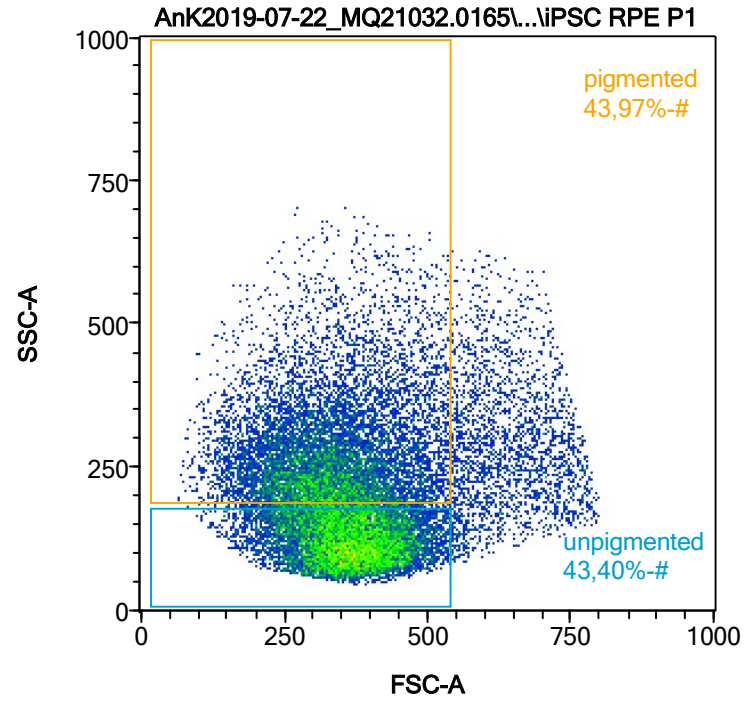

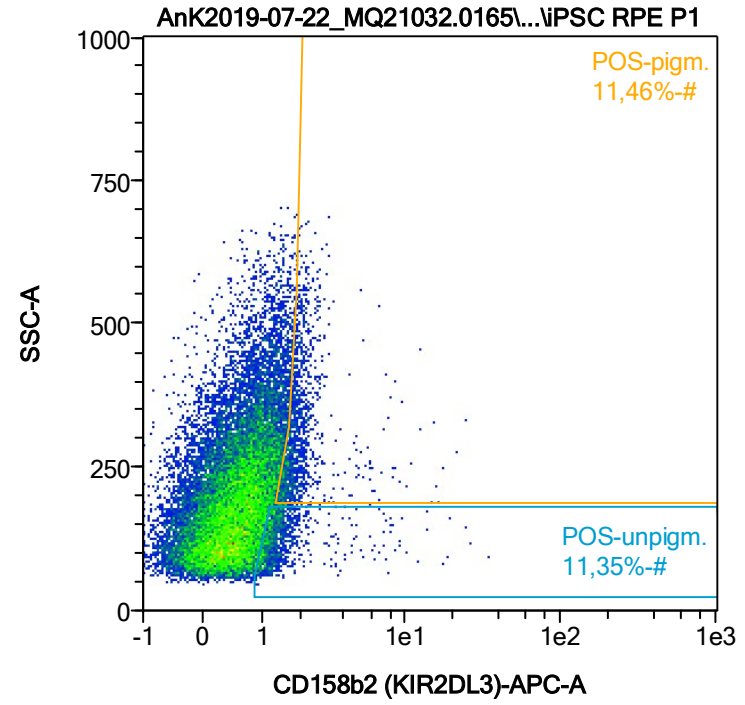

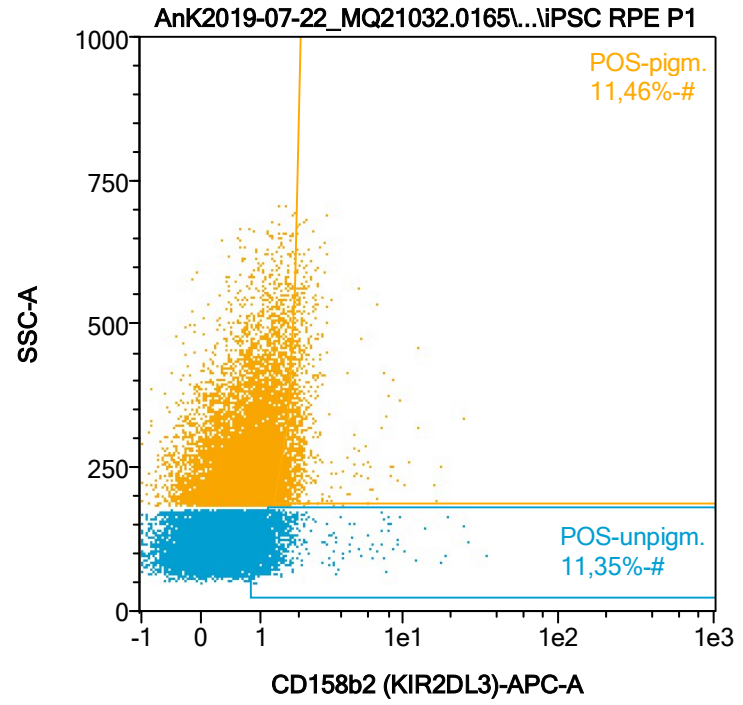

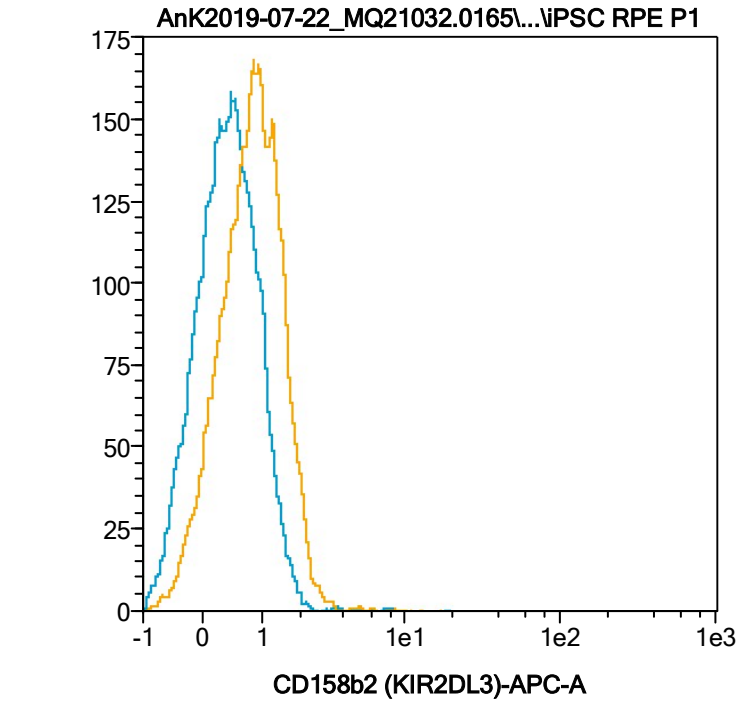

Name	%-#	CD158b2 (KIR2DL3)-APC-A Mean	CD158b2 (KIR2DL3)-APC-A Median
POS-unpigm.	11,35	1,58	1,23
POS-pigm.	11,46	2,10	1,83

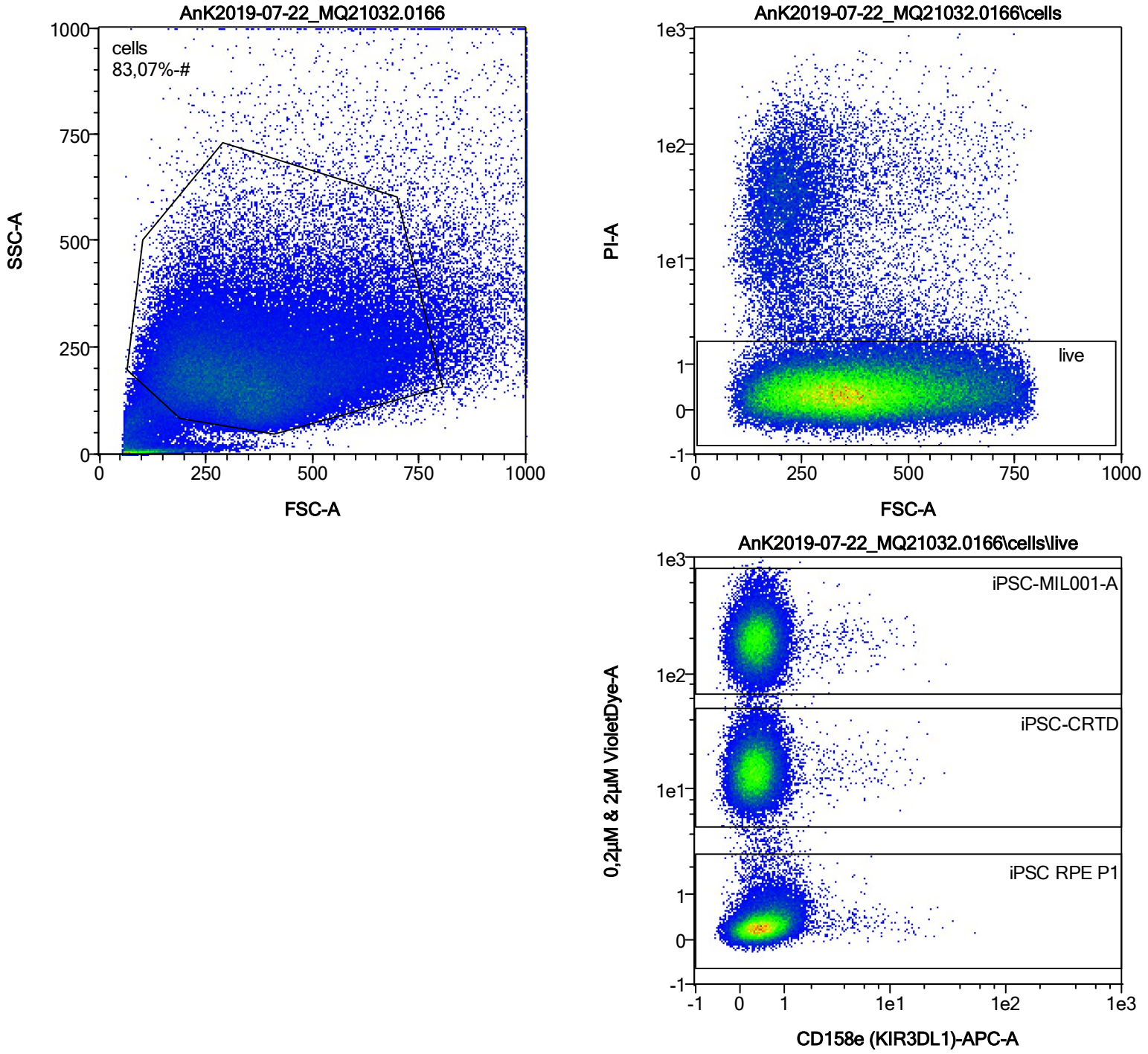

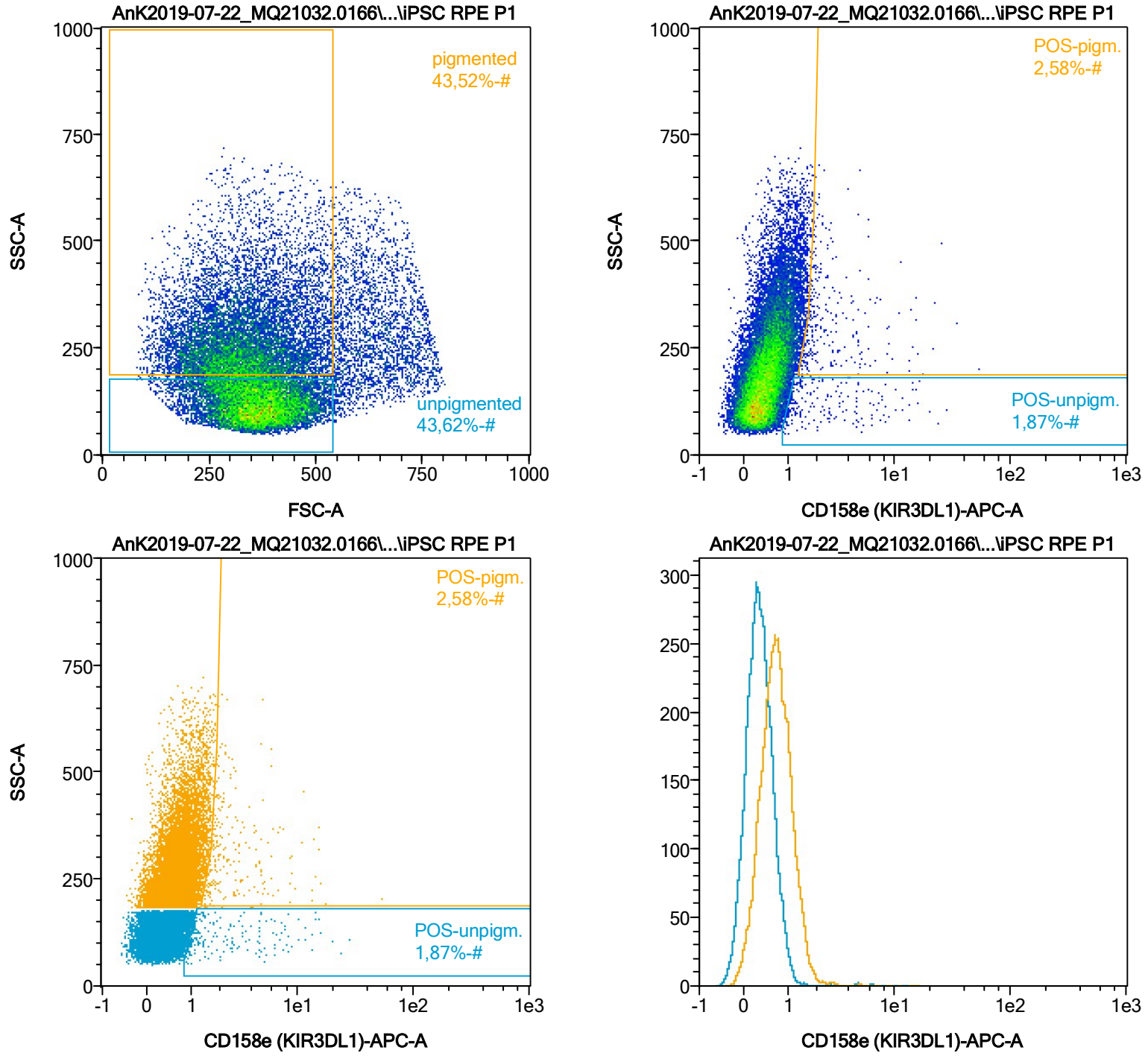

Name	%-#	CD158e (KIR3DL1)-APC-A Mean	CD158e (KIR3DL1)-APC-A Median
POS-unpigm.	1,87	3,18	1,40
POS-pigm.	2,58	3,16	1,96

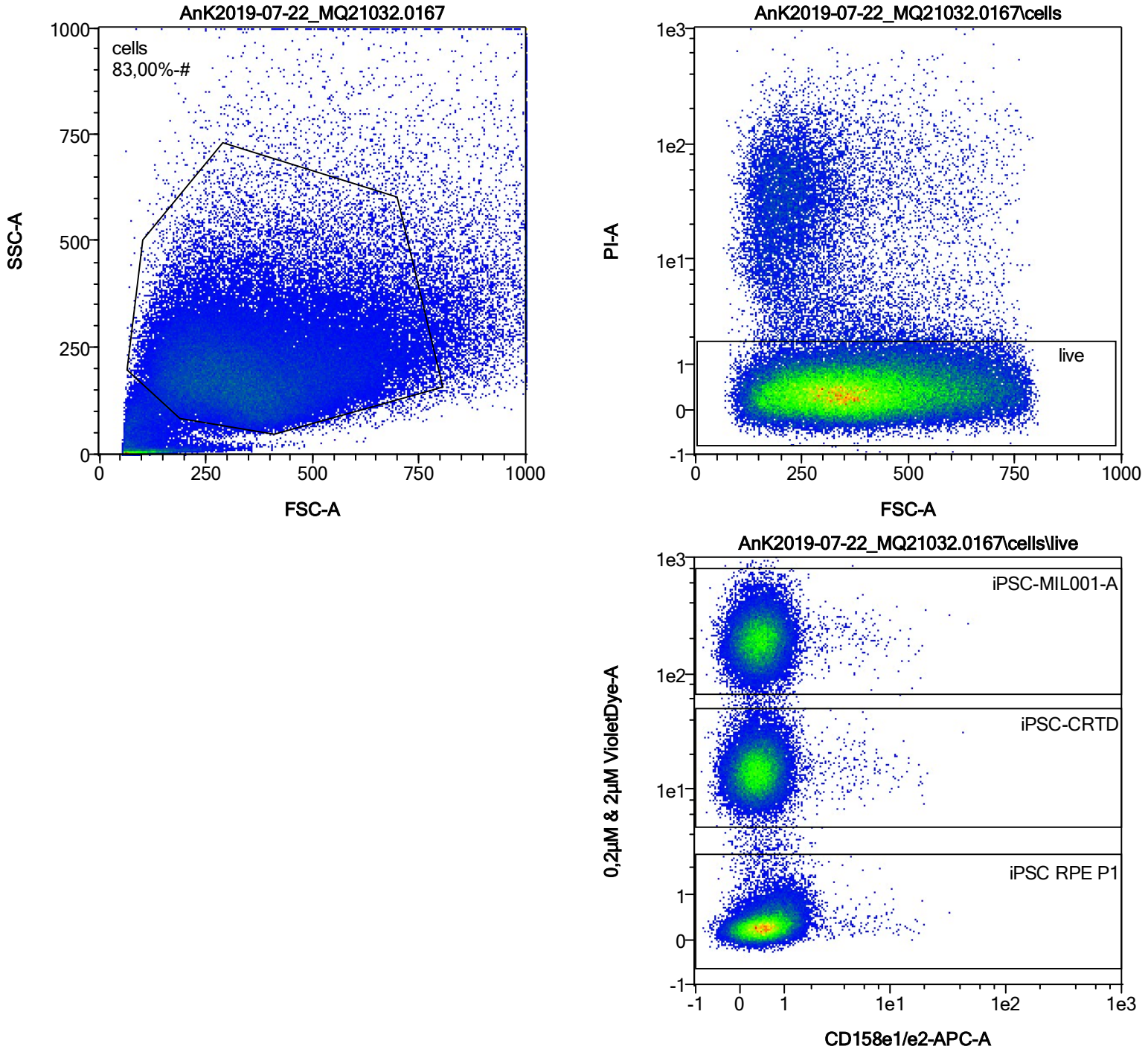

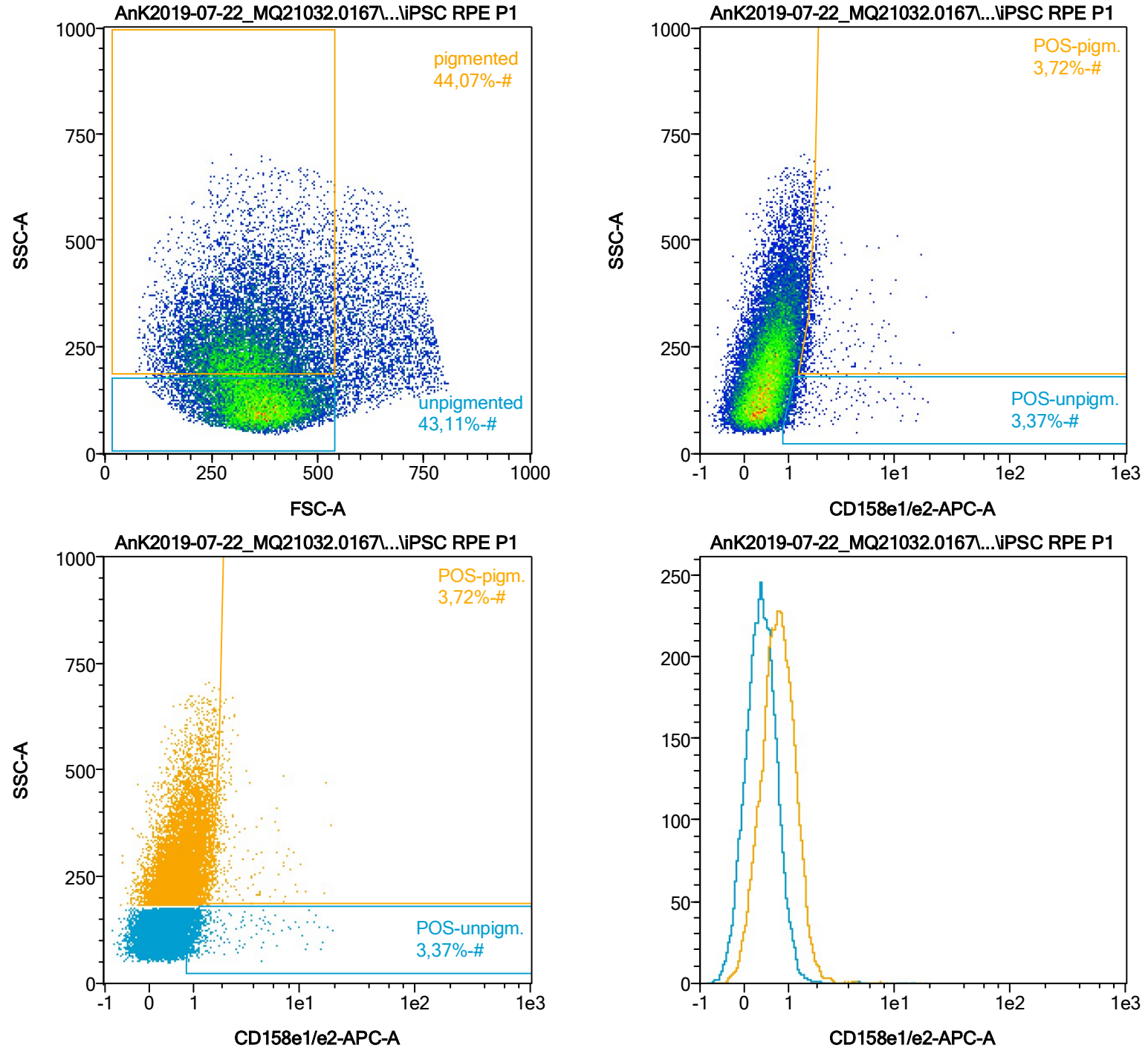

Name	%-#	CD158e1/e2-APC-A Mean	CD158e1/e2-APC-A Median
POS-unpigm.	3,37	2,02	1,18
POS-pigm.	3,72	2,36	1,84

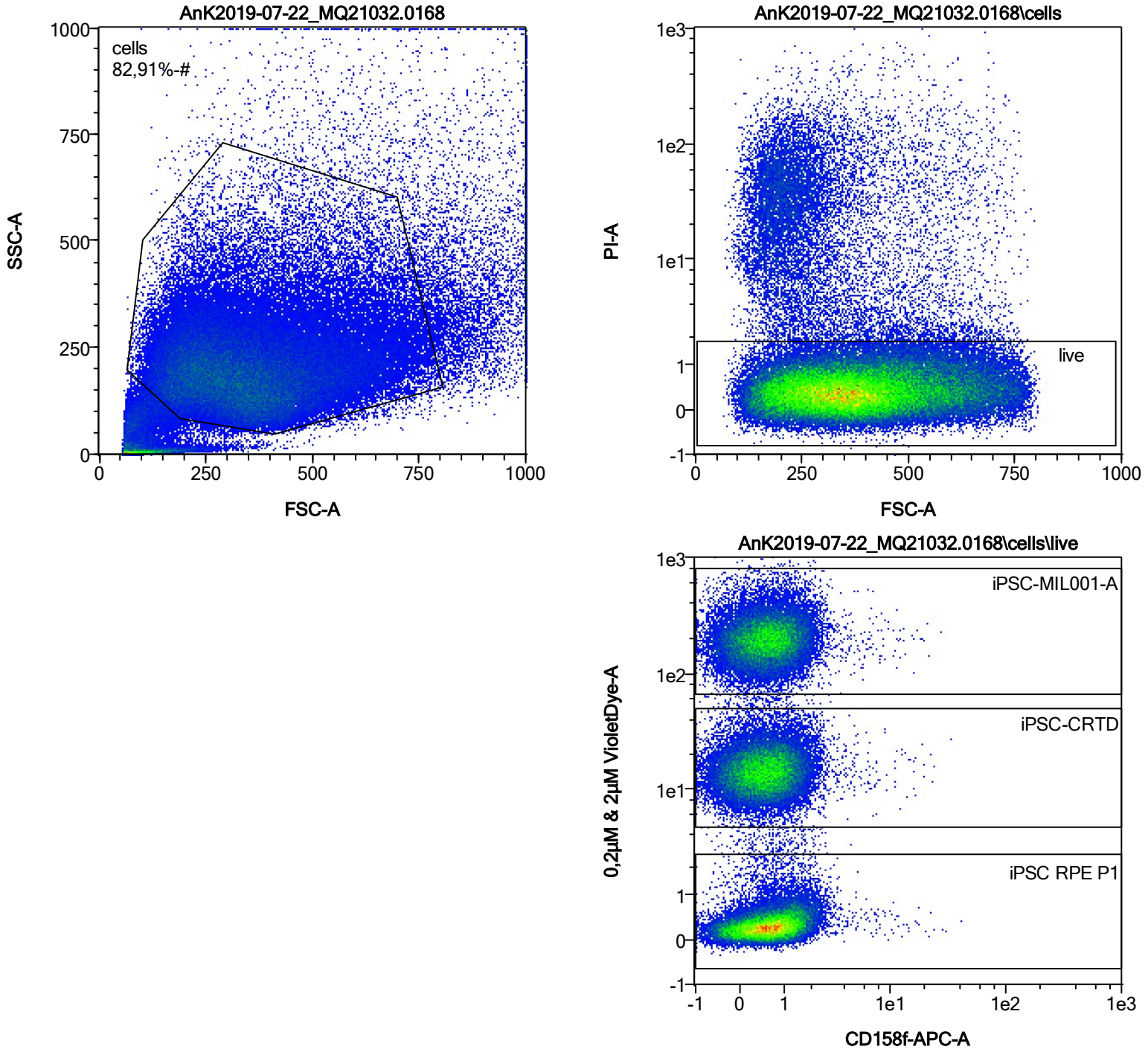

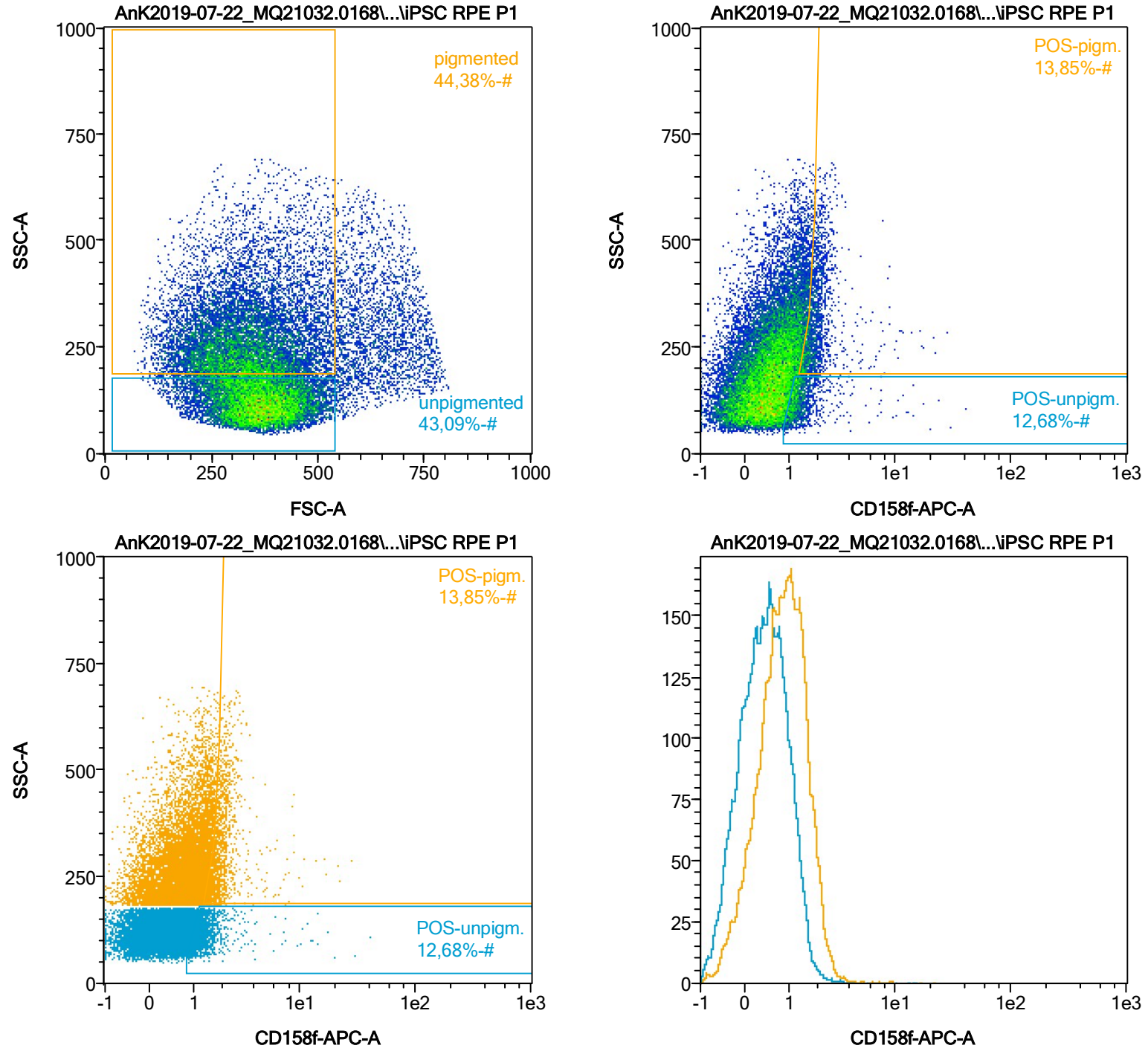

Name	%-#	CD158f-APC-A Mean	CD158f-APC-A Median
POS-unpigm.	12,68	1,50	1,24
POS-pigm.	13,85	2,09	1,87

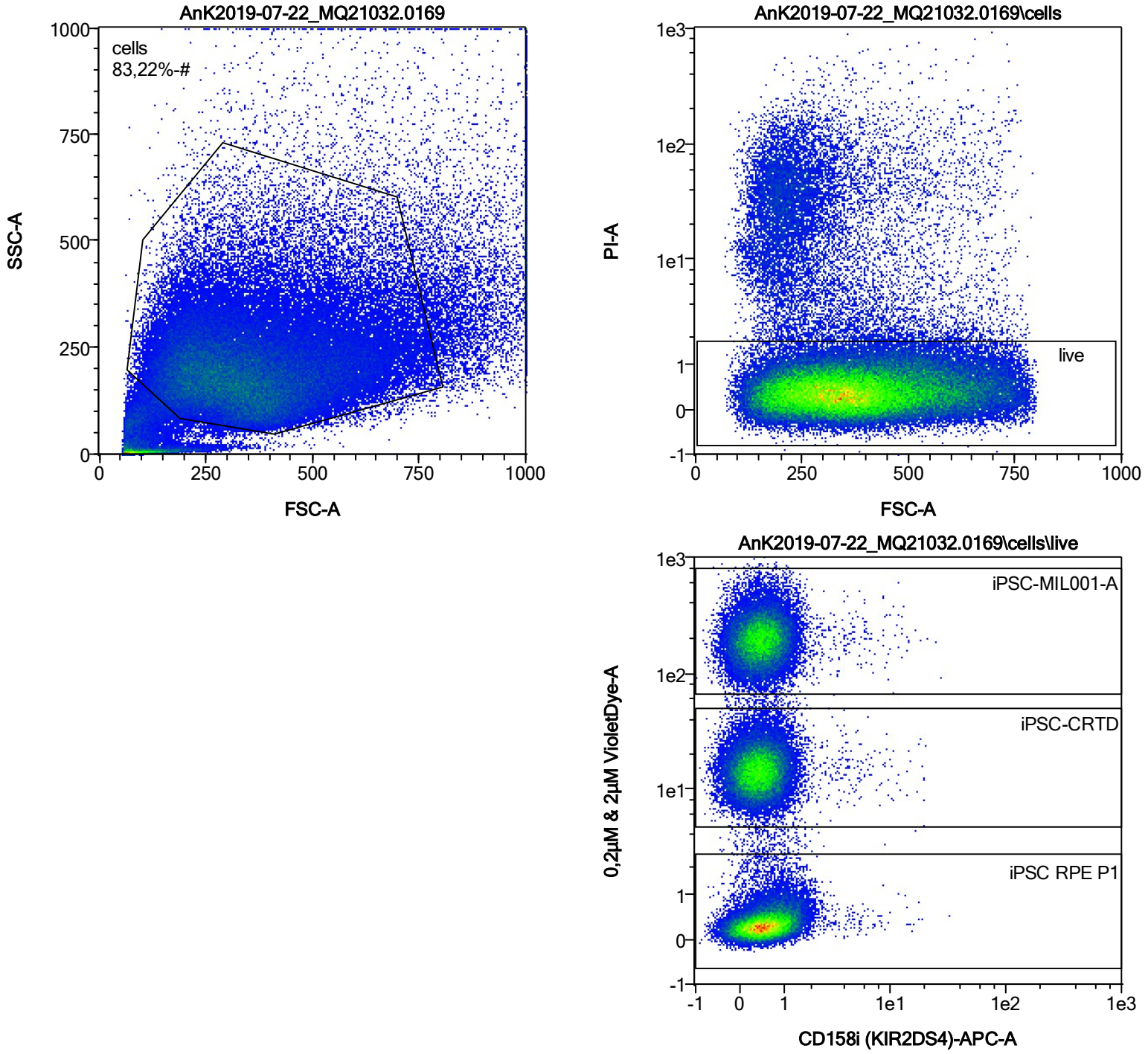

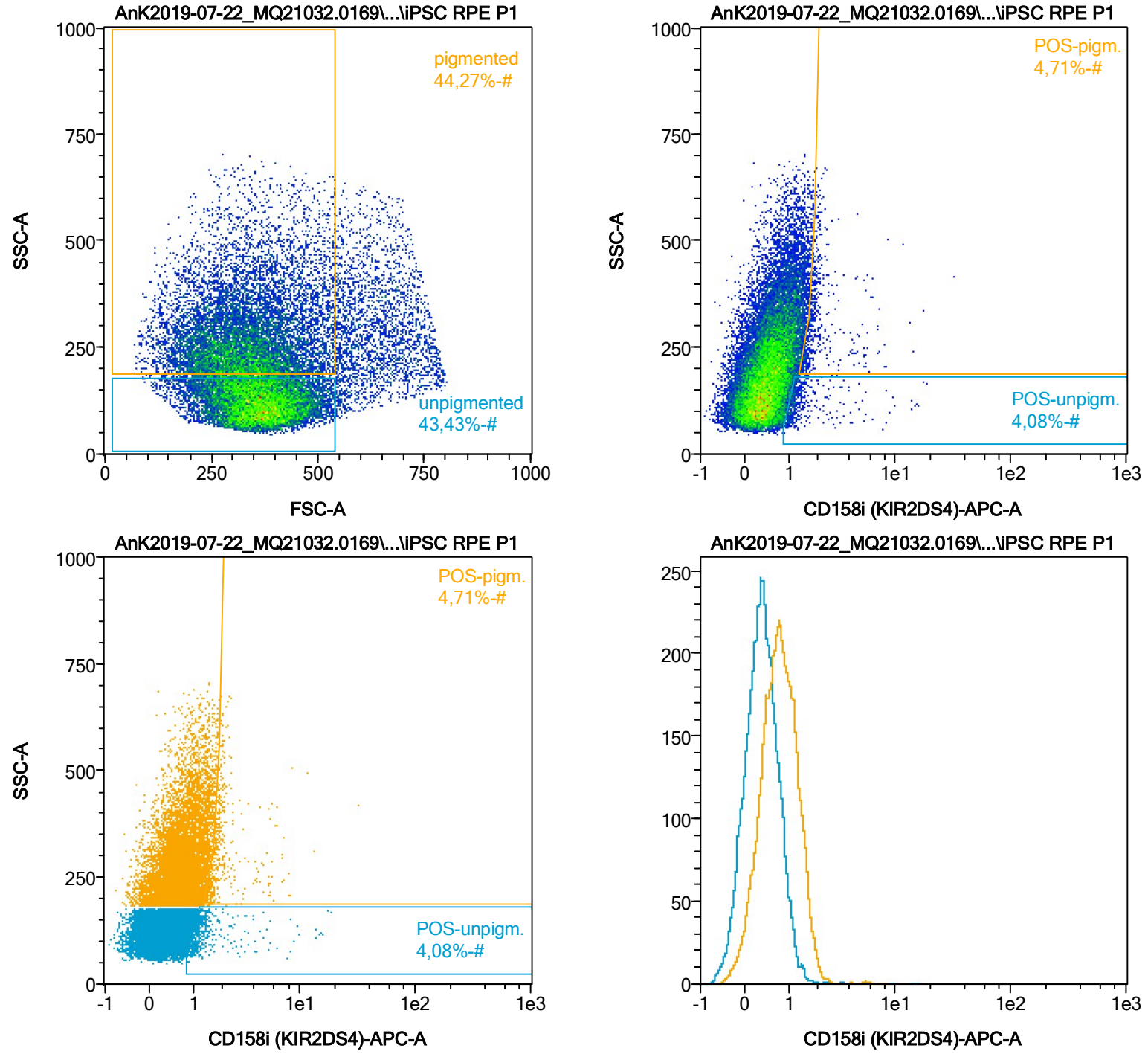

Name	%-#	CD158i (KIR2DS4)-APC-A Mean	CD158i (KIR2DS4)-APC-A Median
POS-unpigm.	4,08	1,72	1,17
POS-pigm.	4,71	2,19	1,79

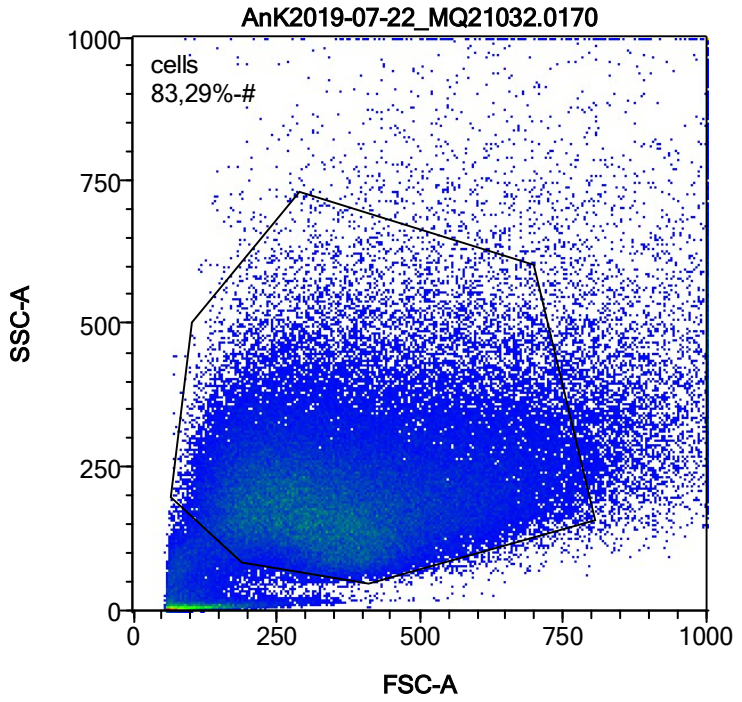

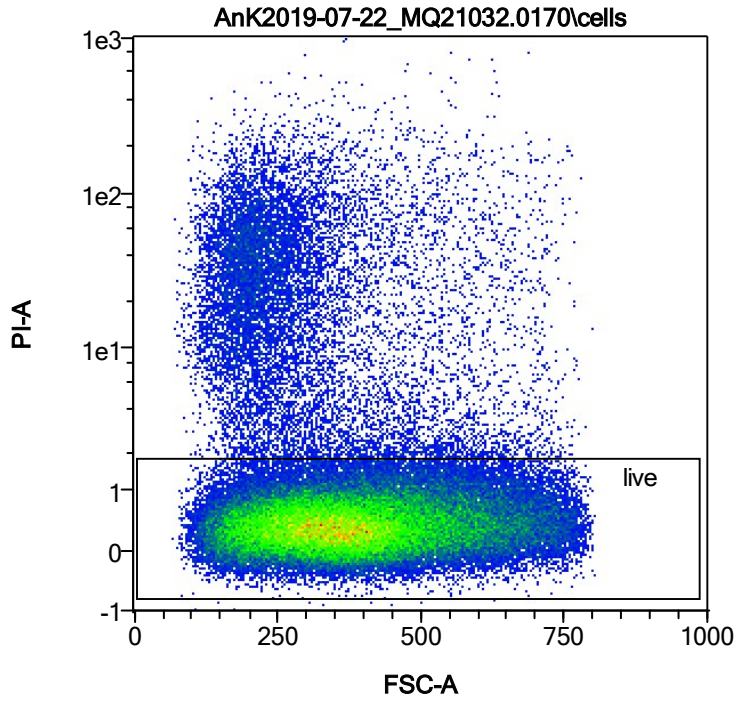

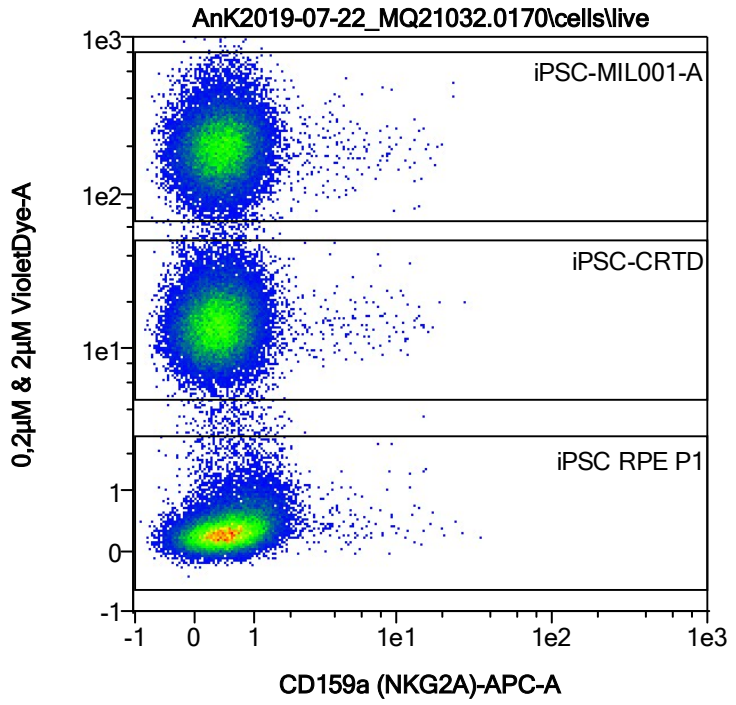

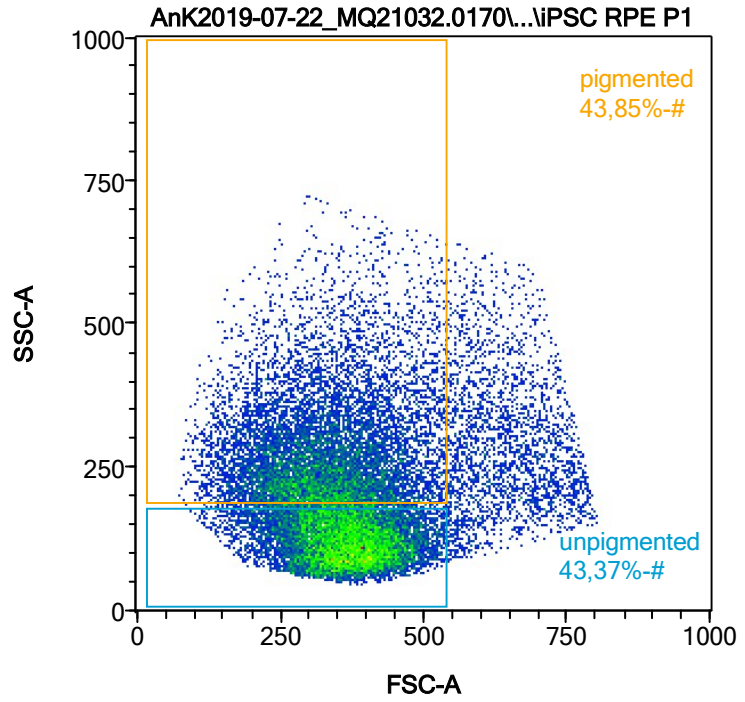

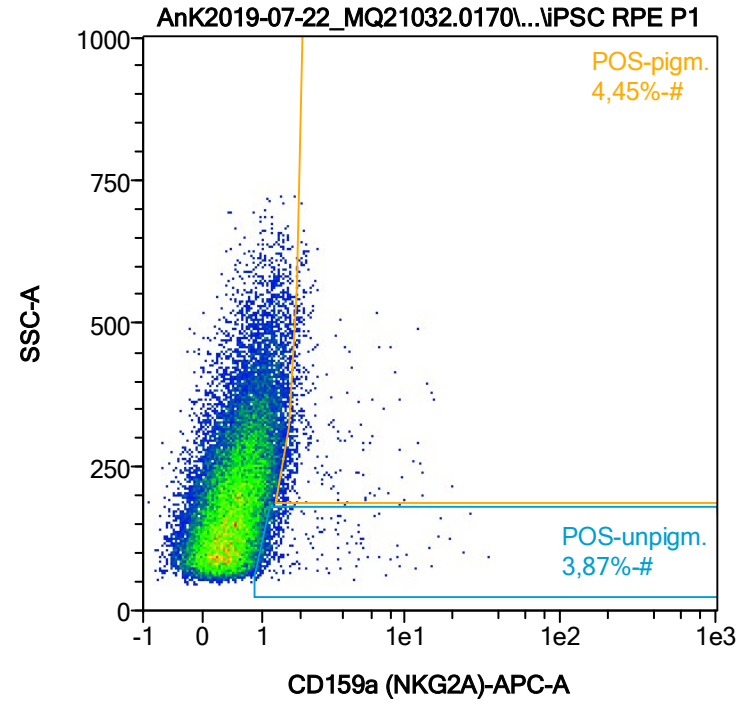

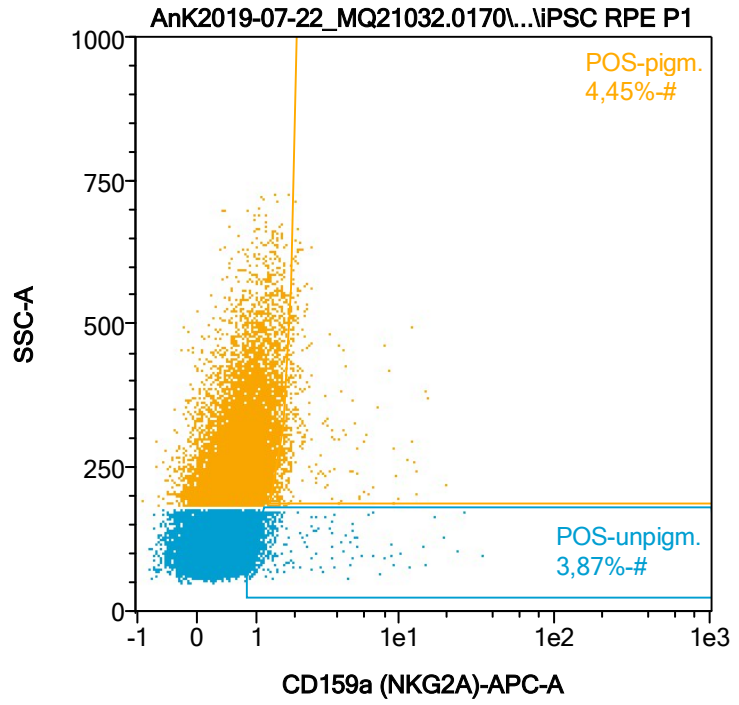

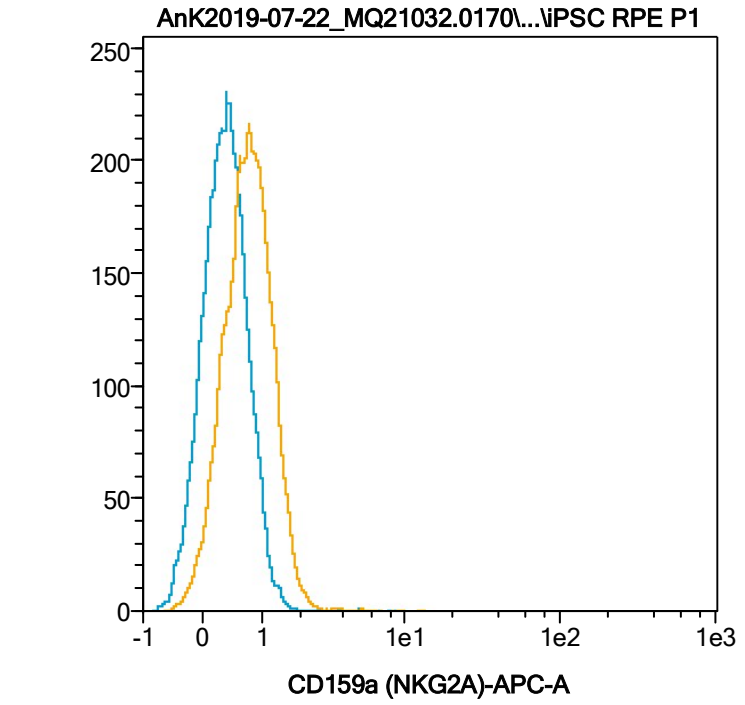

Name	%-#	CD159a (NKG2A)-APC-A Mean	CD159a (NKG2A)-APC-A Median
POS-unpigm.	3,87	1,93	1,18
POS-pigm.	4,45	2,36	1,83

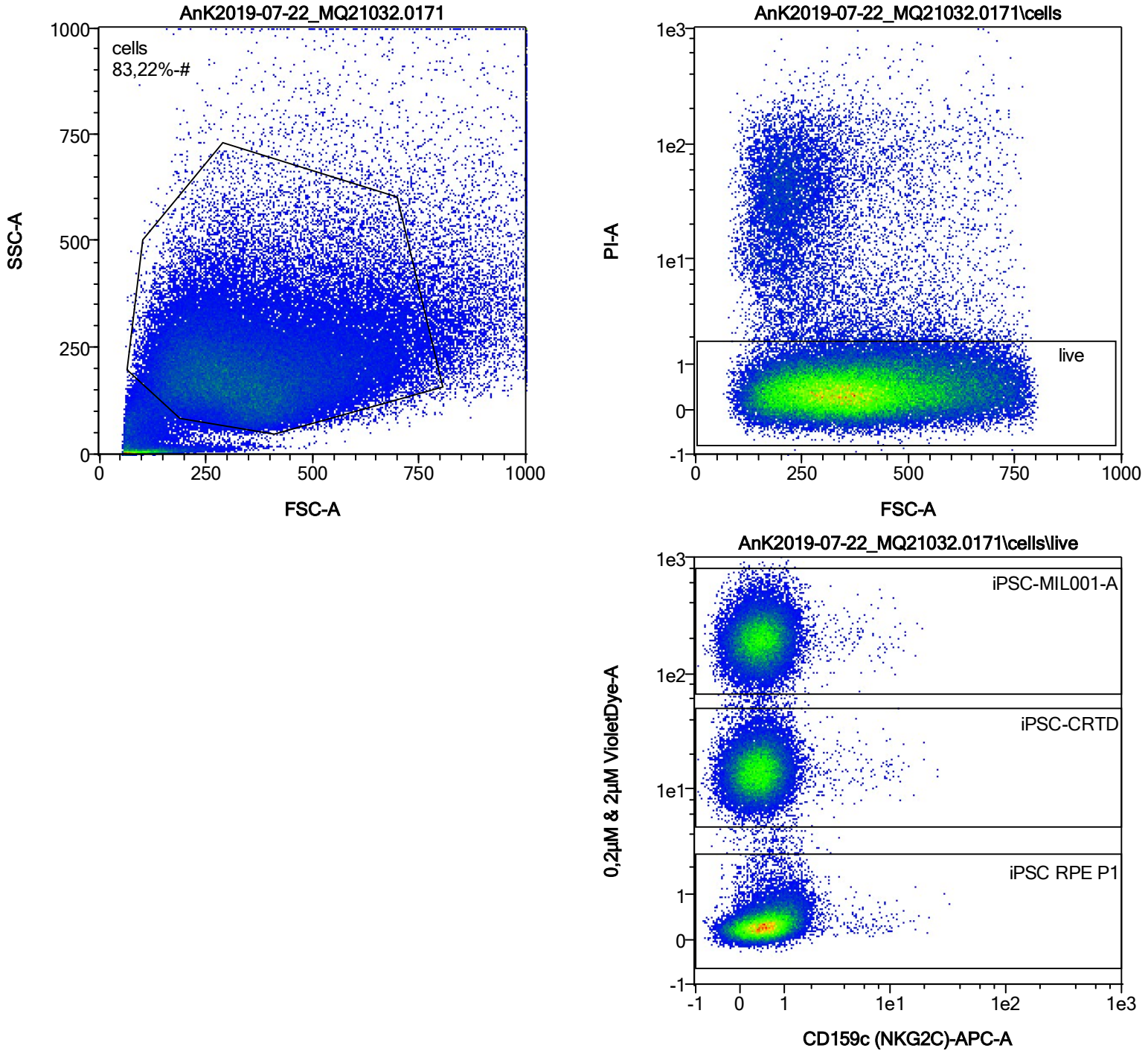

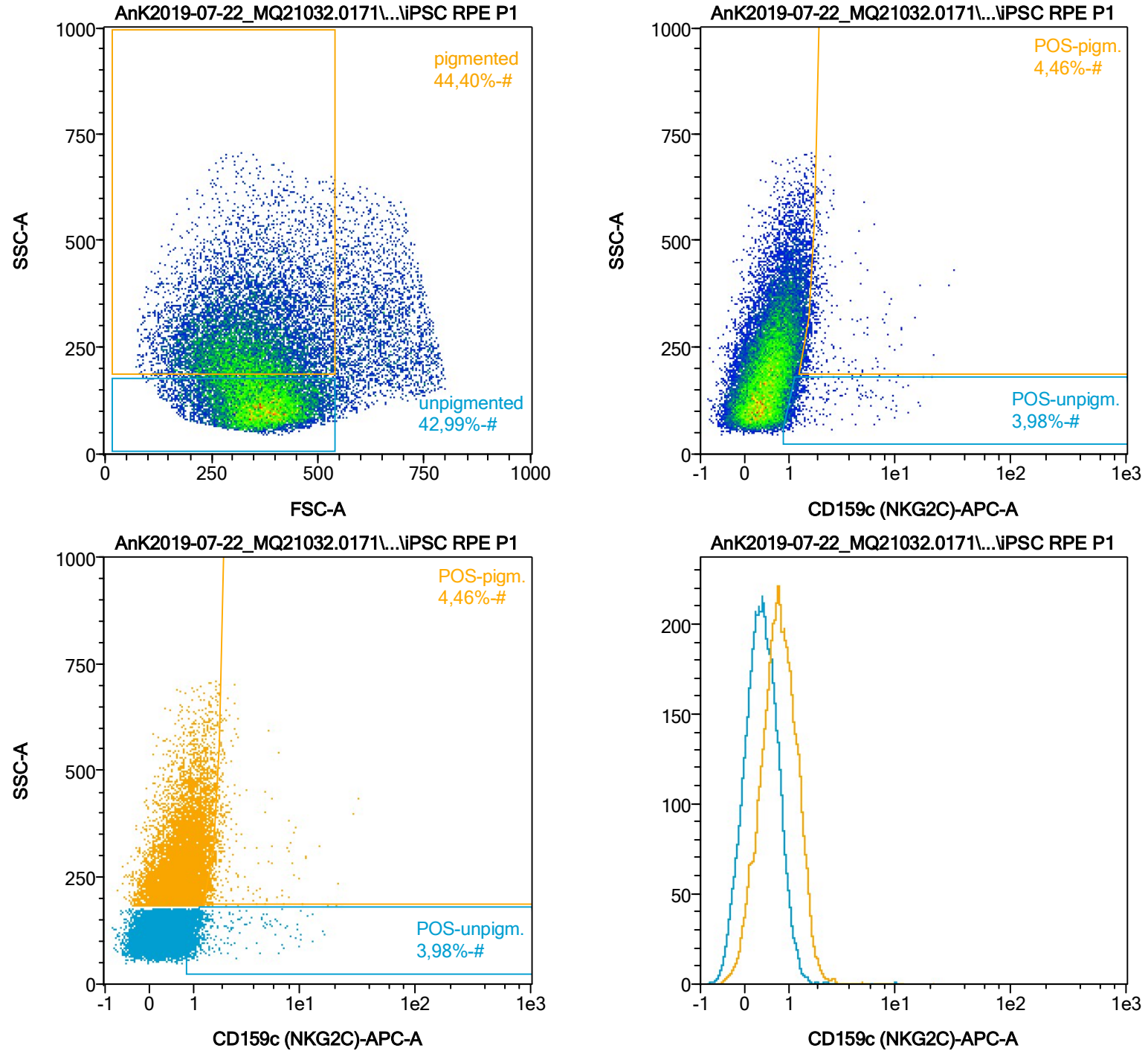

Name	%-#	CD159c (NKG2C)-APC-A Mean	CD159c (NKG2C)-APC-A Median
POS-unpigm.	3,98	1,88	1,19
POS-pigm.	4,46	2,45	1,79

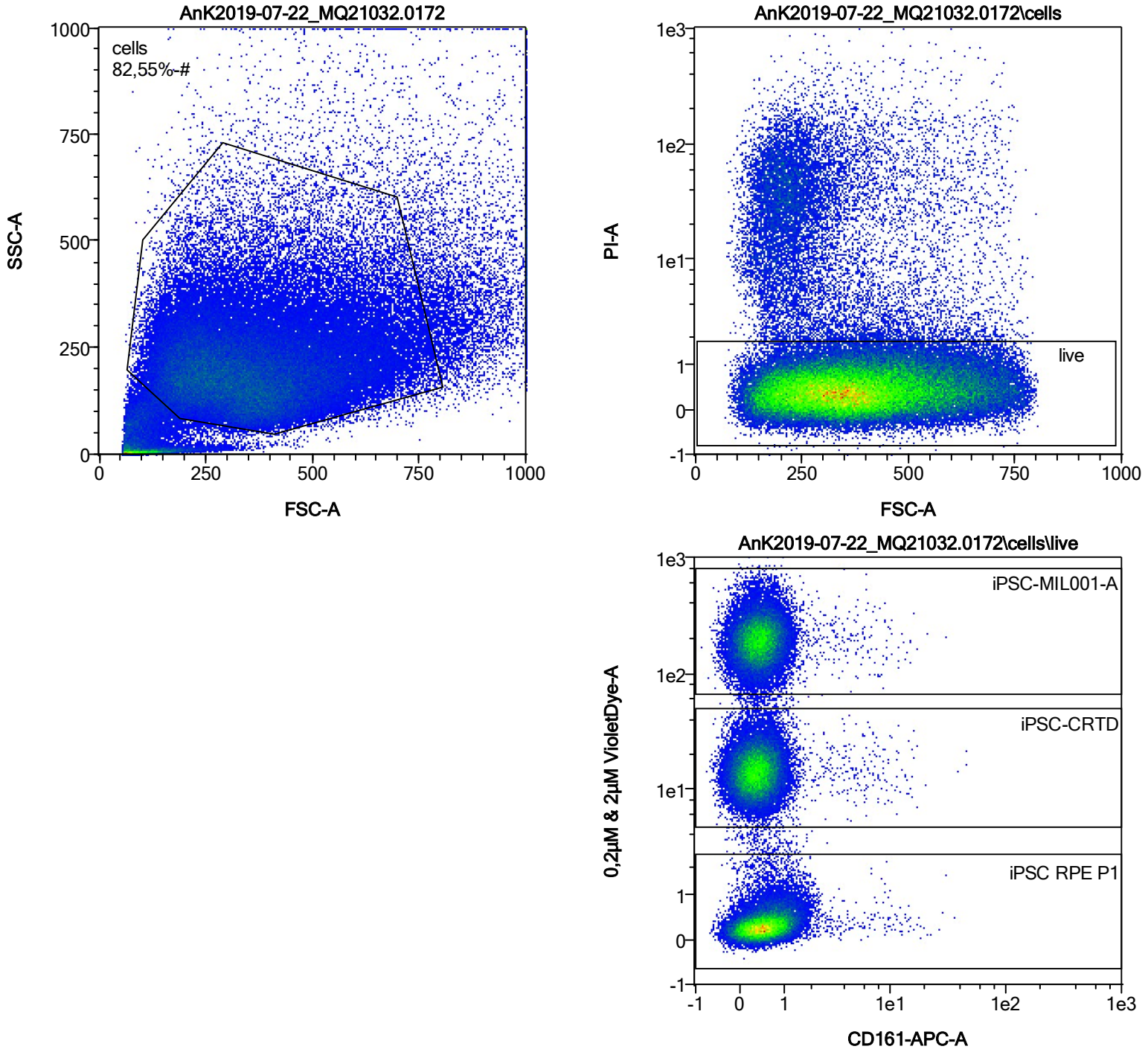

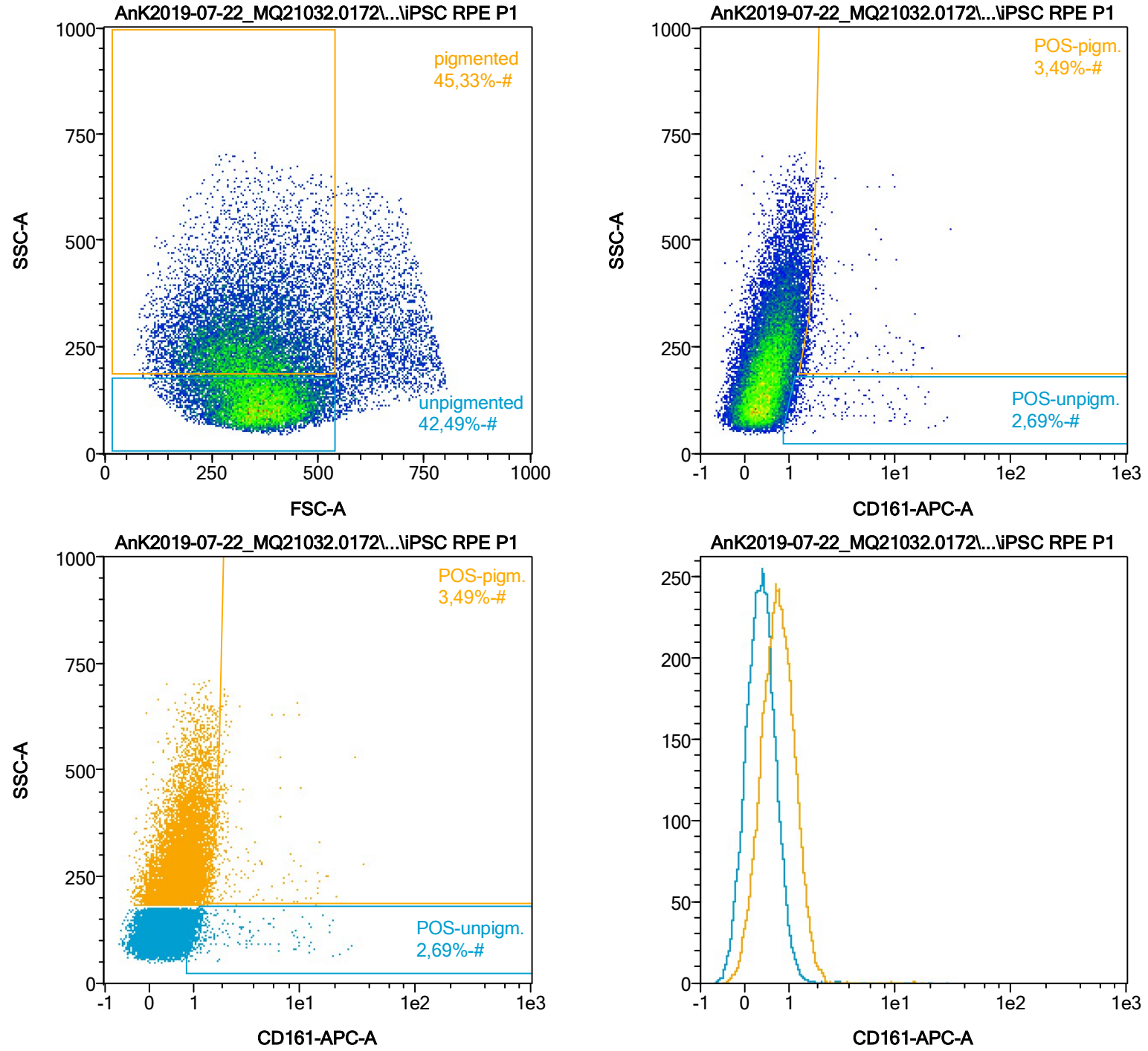

Name	%-#	CD161-APC-A Mean	CD161-APC-A Median
POS-unpigm.	2,69	2,62	1,22
POS-pigm.	3,49	2,79	1,83

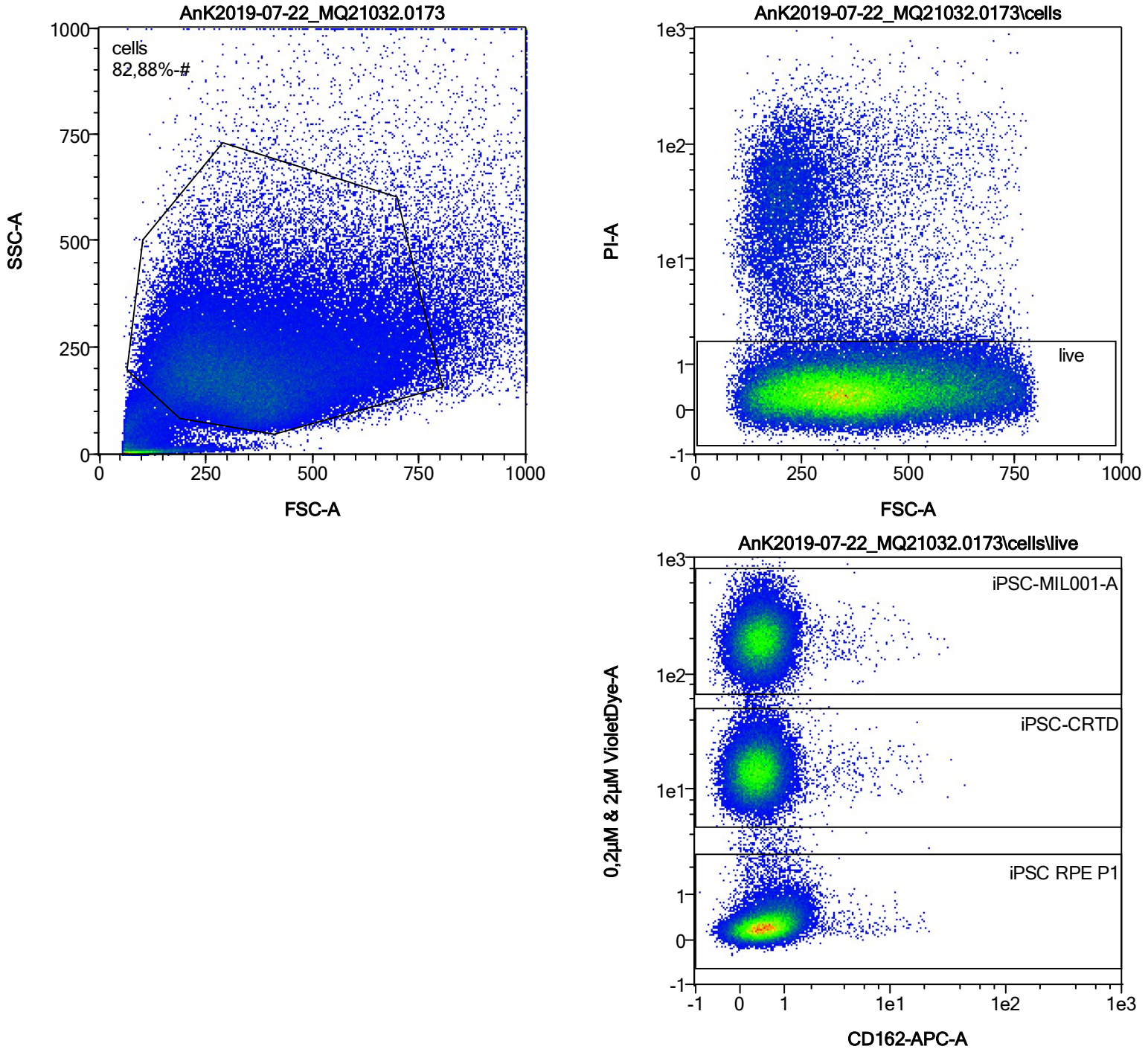

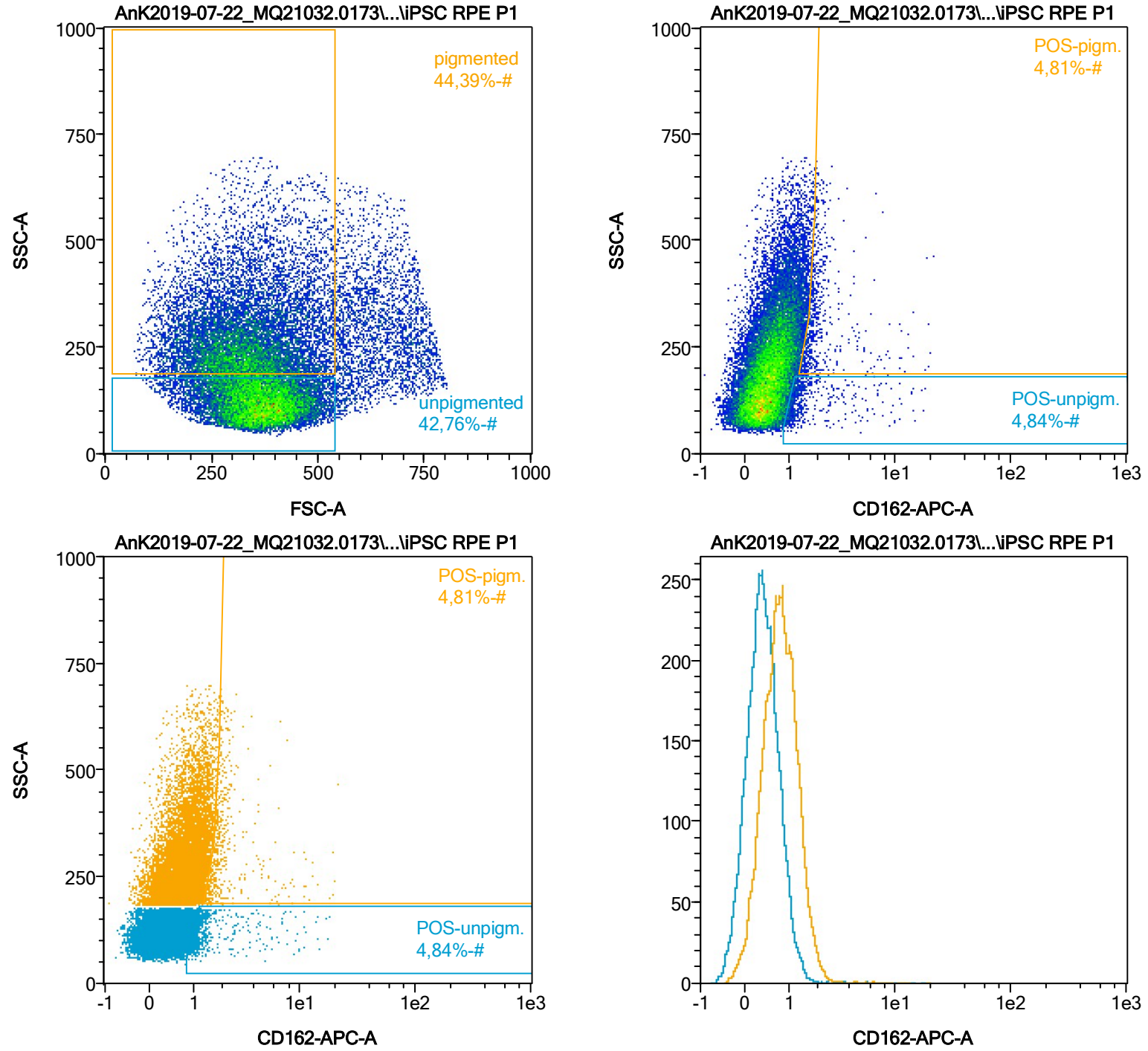

Name	%-#	CD162-APC-A Mean	CD162-APC-A Median
POS-unpigm.	4,84	1,95	1,22
POS-pigm.	4,81	2,48	1,85

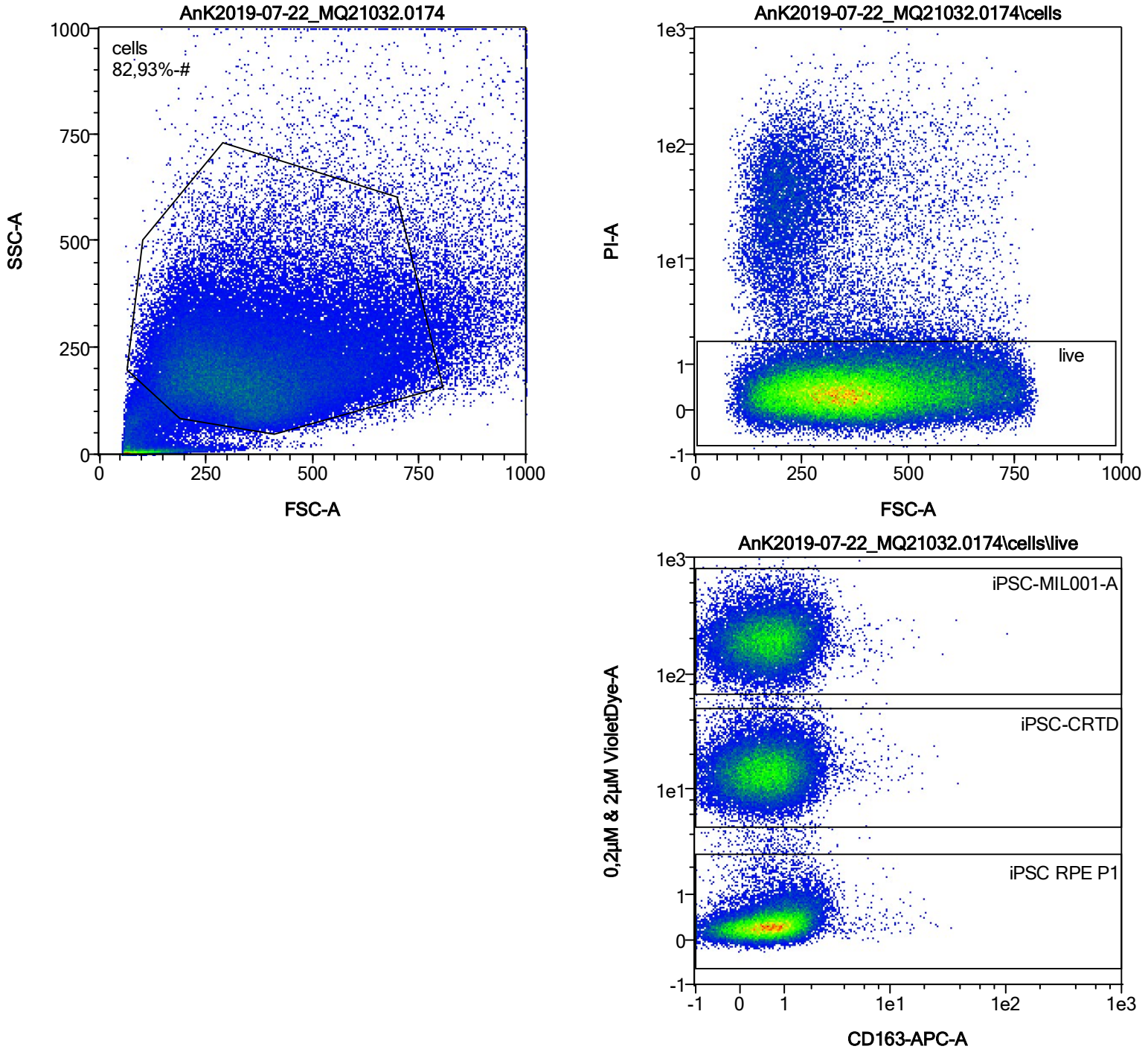

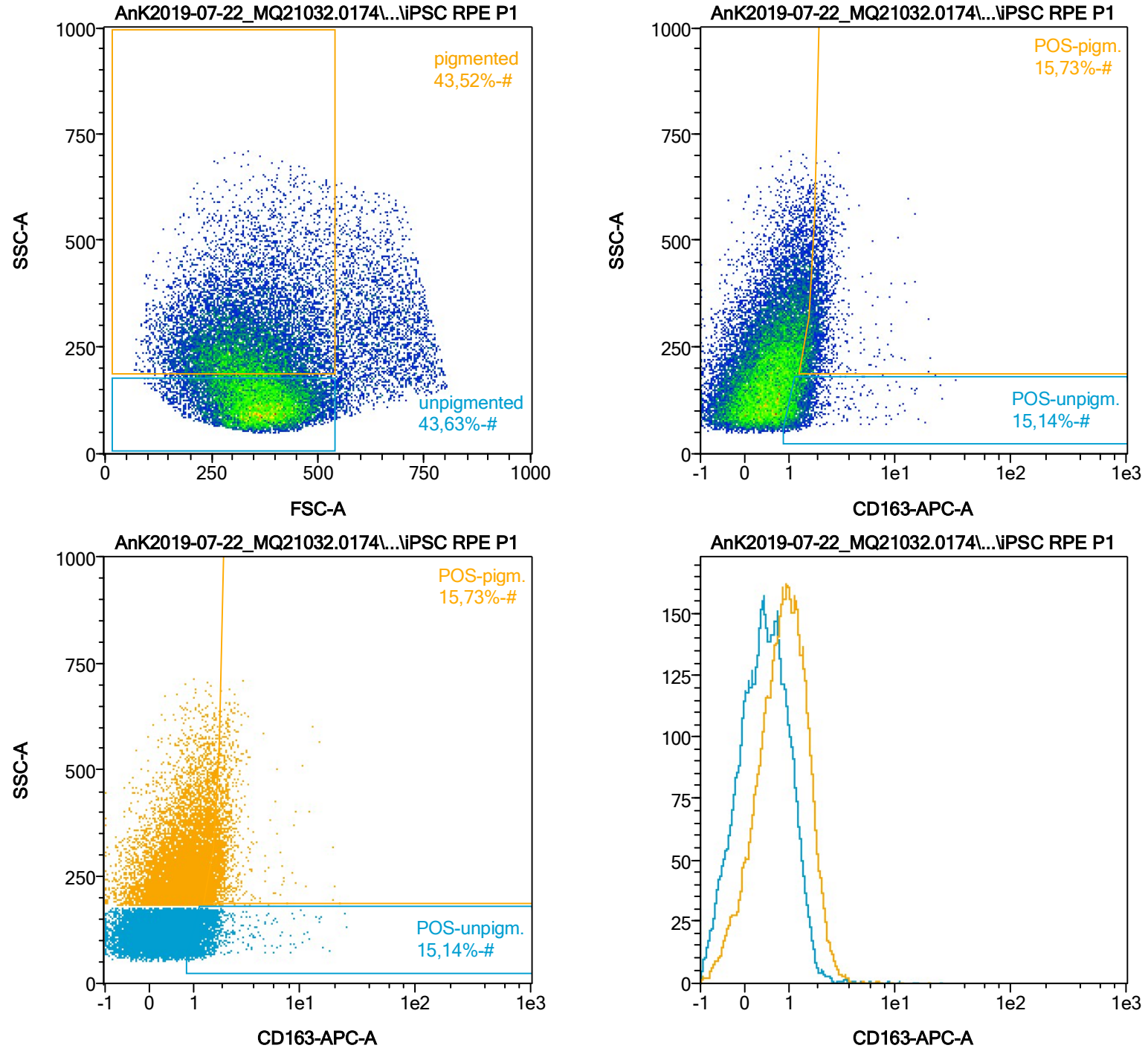

Name	%-#	CD163-APC-A Mean	CD163-APC-A Median
POS-unpigm.	15,14	1,55	1,26
POS-pigm.	15,73	2,08	1,85

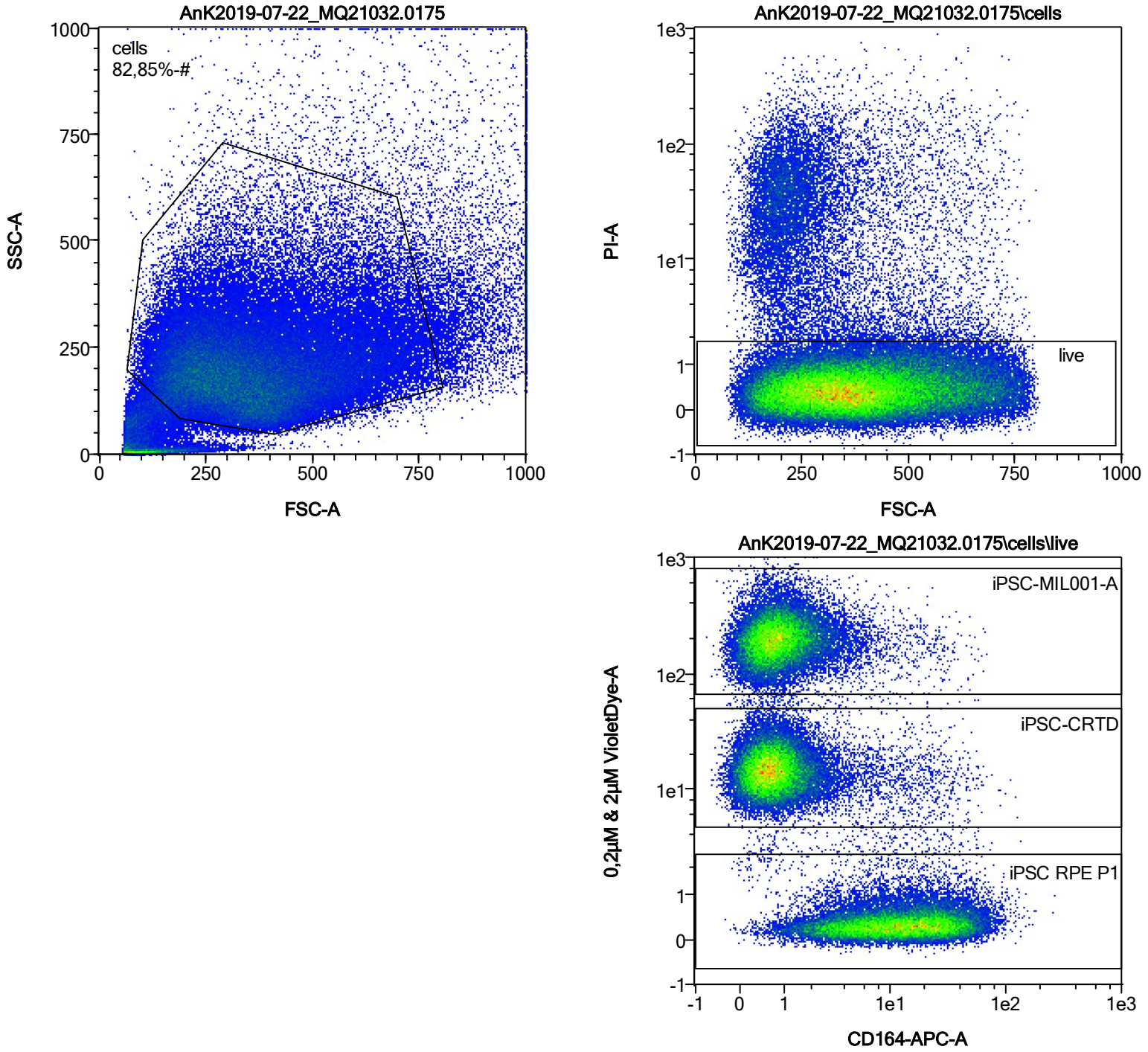

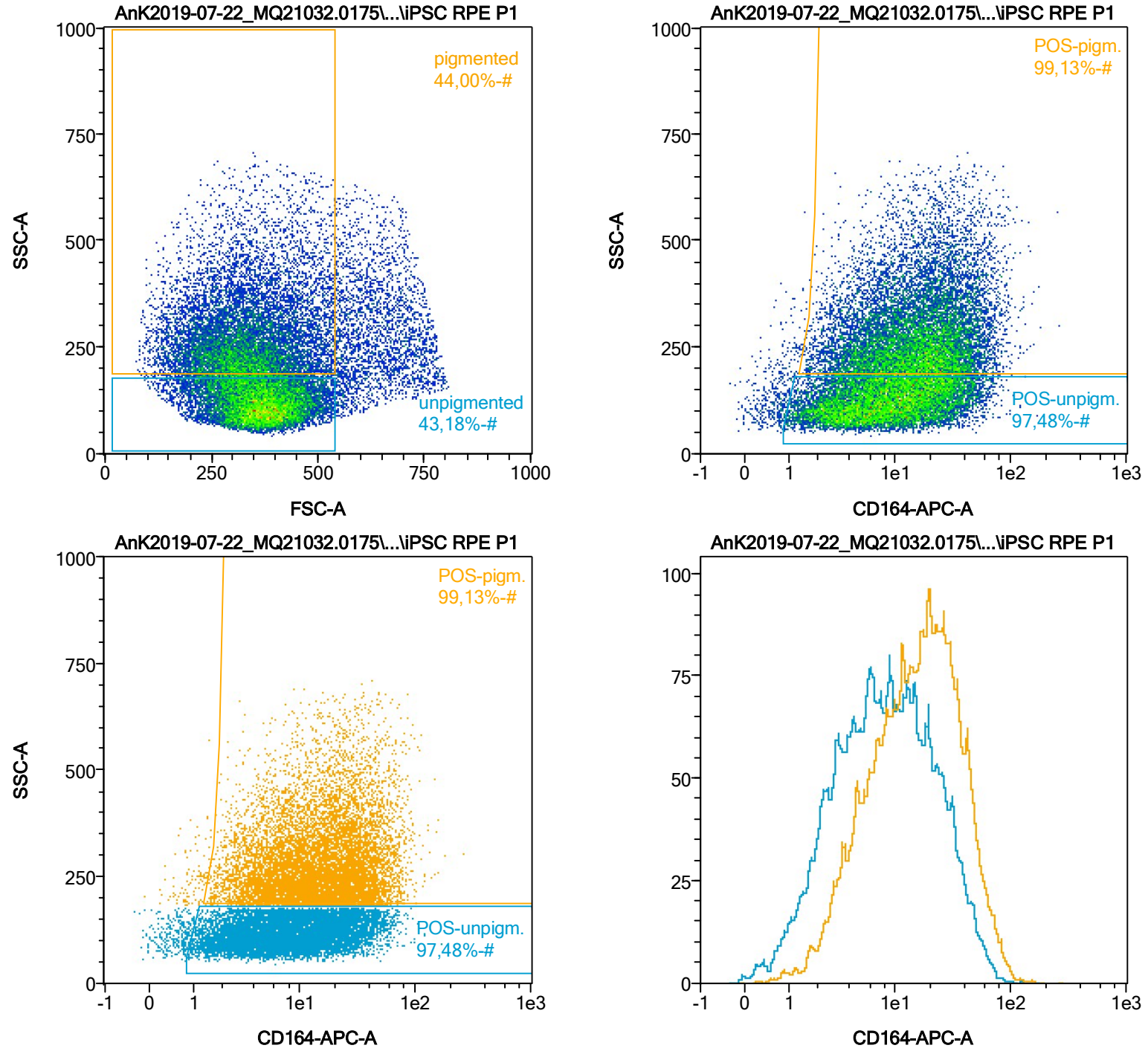

Name	%-#	CD164-APC-A Mean	CD164-APC-A Median
POS-unpigm.	97,48	12,49	8,39
POS-pigm.	99,13	20,30	15,76

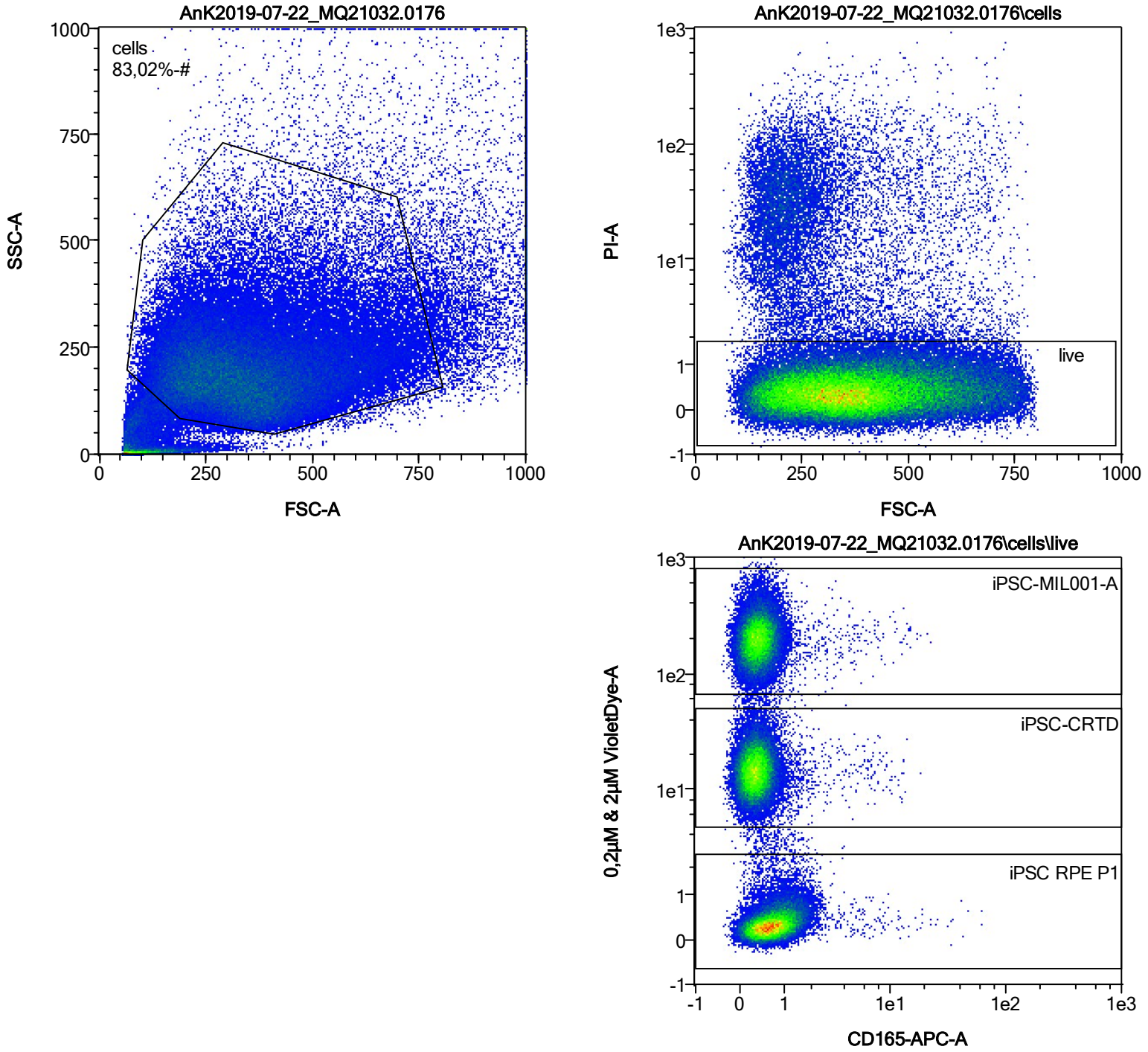

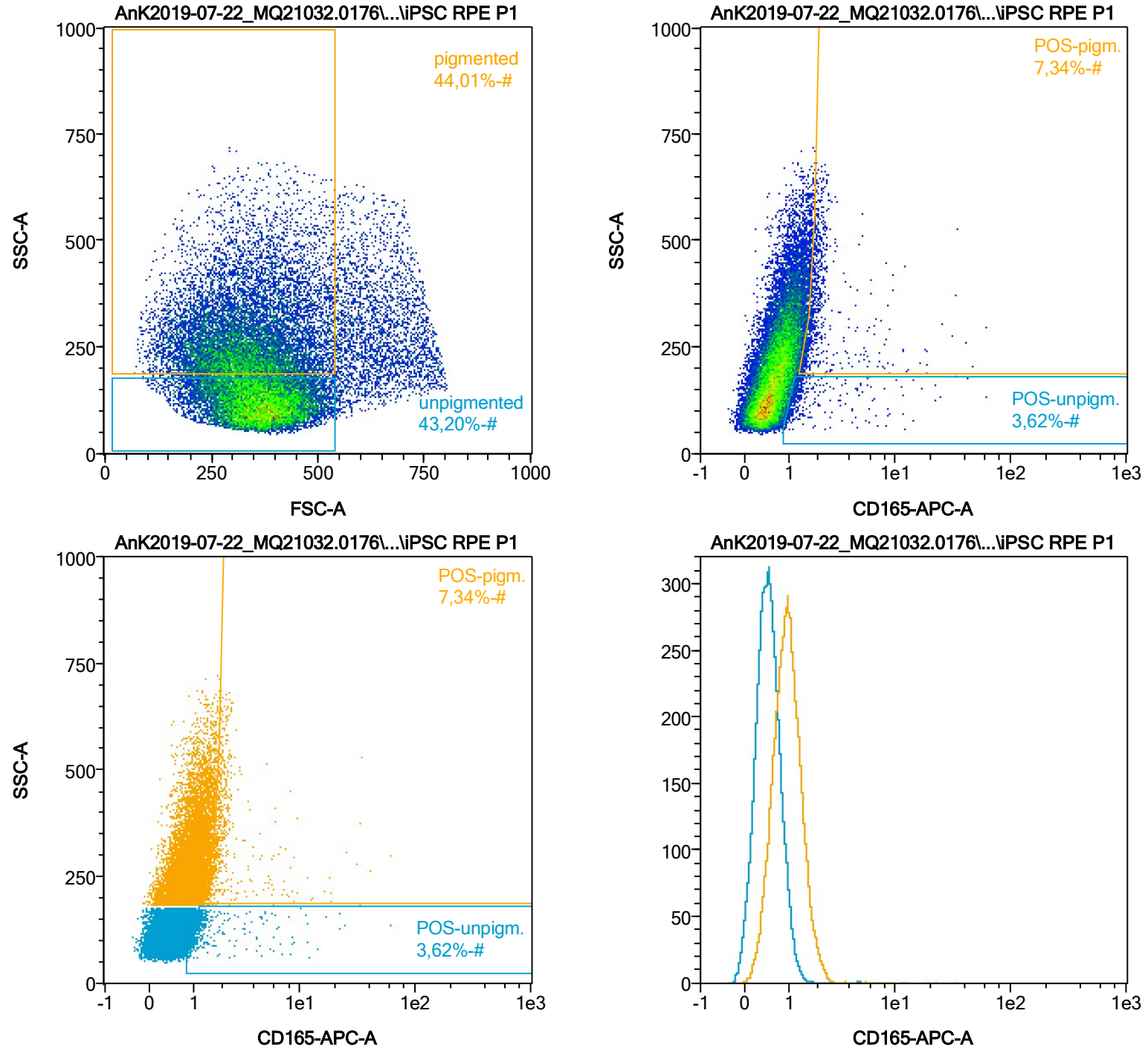

Name	%-#	CD165-APC-A Mean	CD165-APC-A Median
POS-unpigm.	3,62	2,12	1,20
POS-pigm.	7,34	2,48	1,83

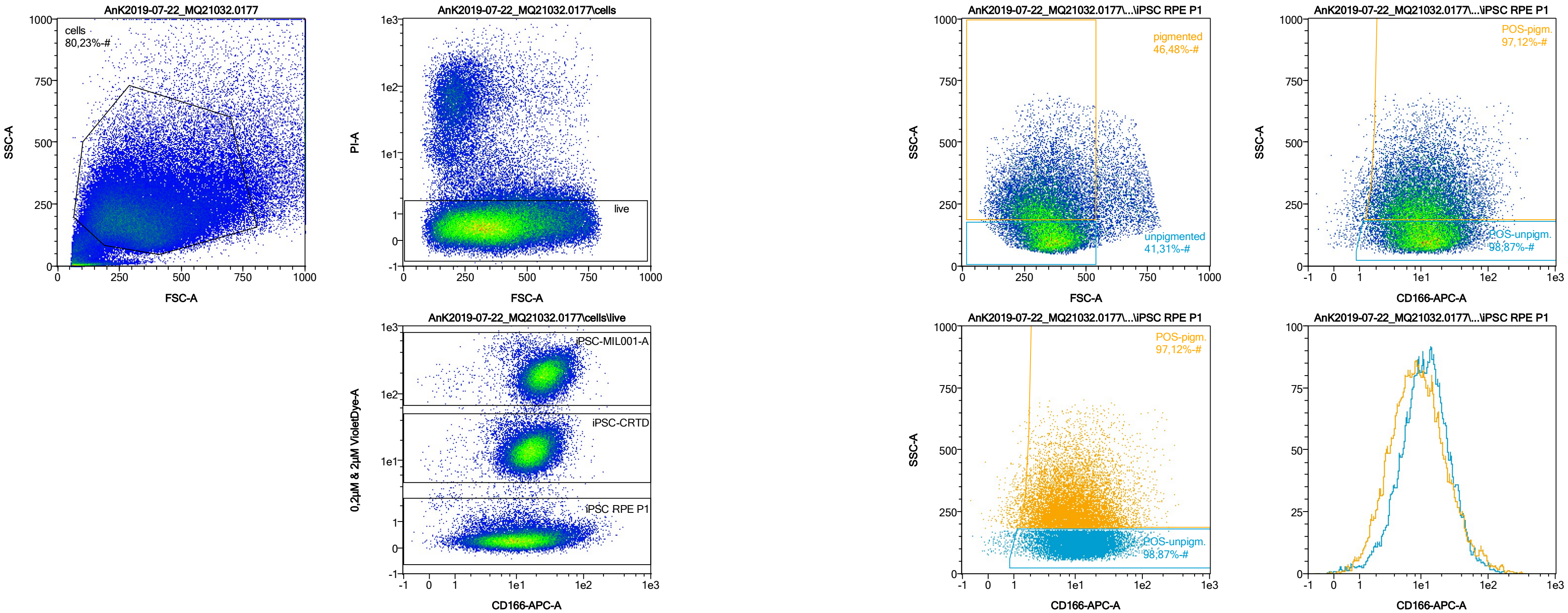

Name	%-#	CD166-APC-A Mean	CD166-APC-A Median
POS-unpigm.	98,87	15,68	11,57
POS-pigm.	97,12	14,91	8,97

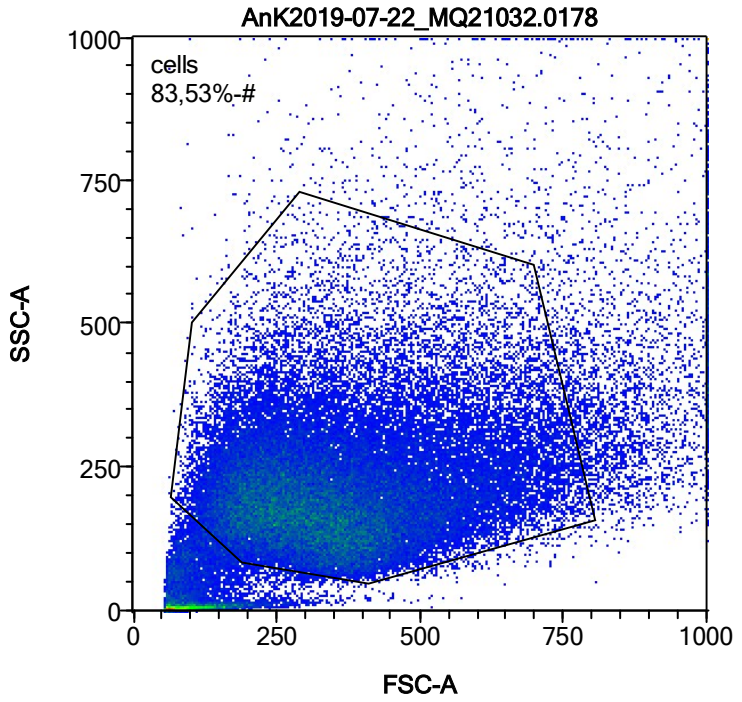

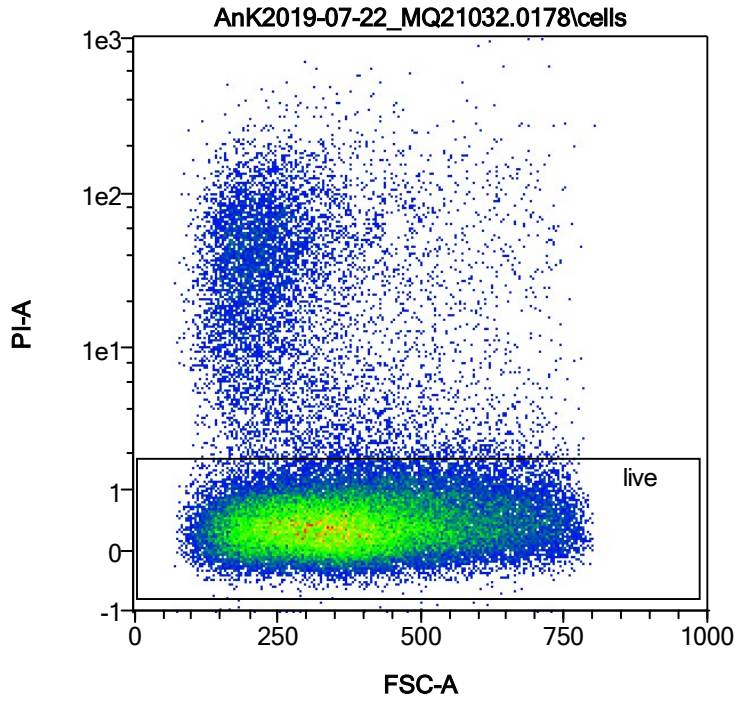

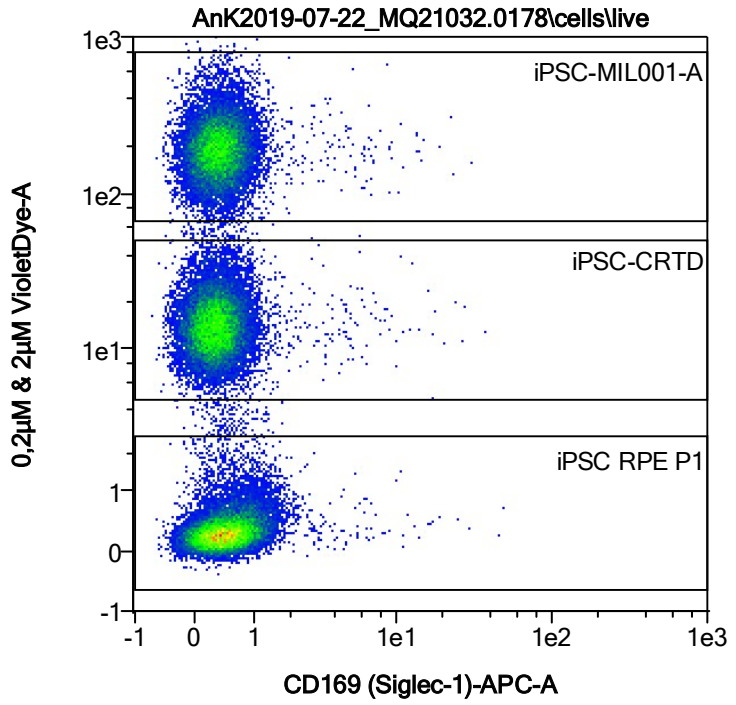

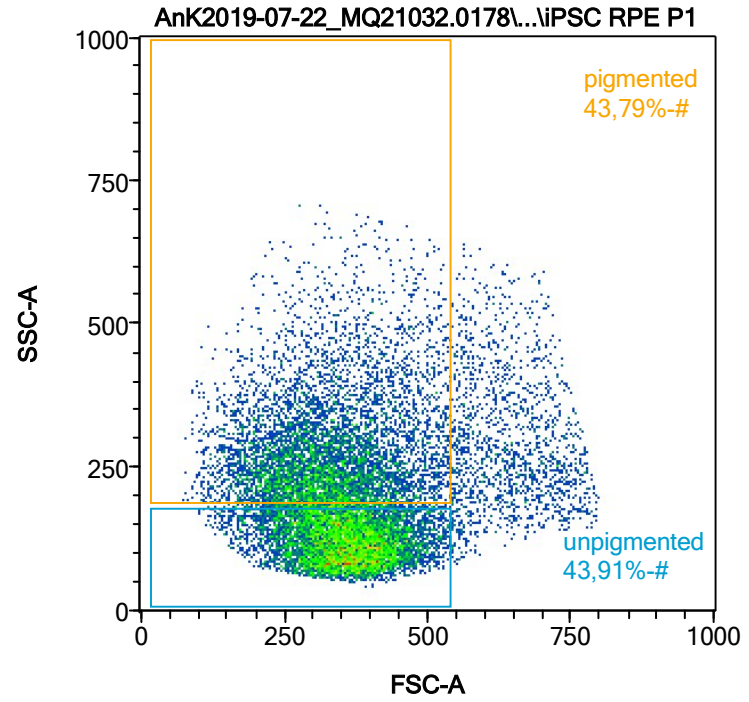

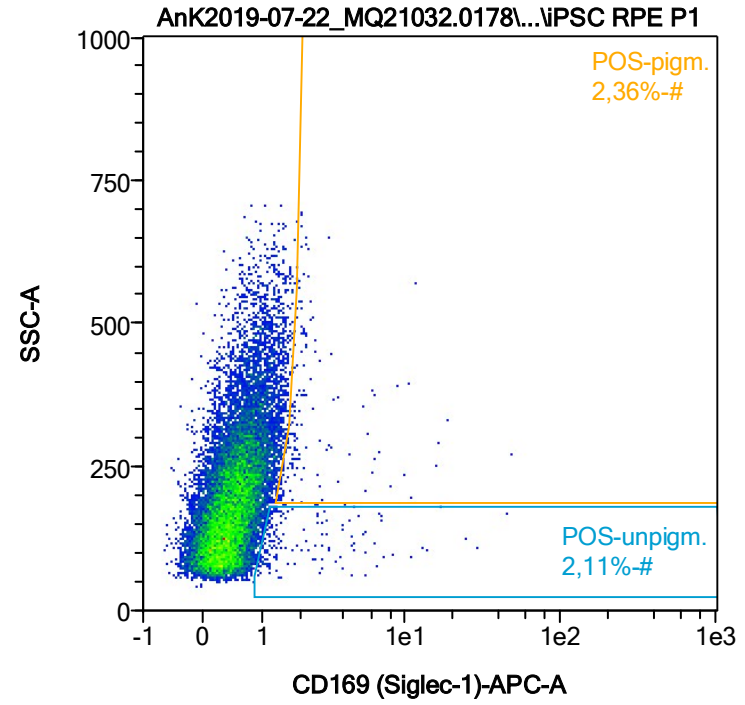

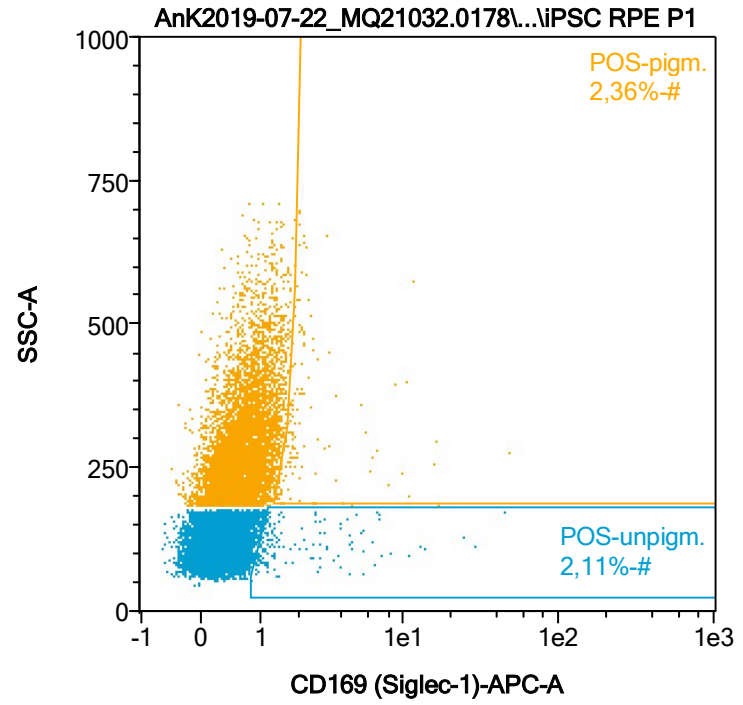

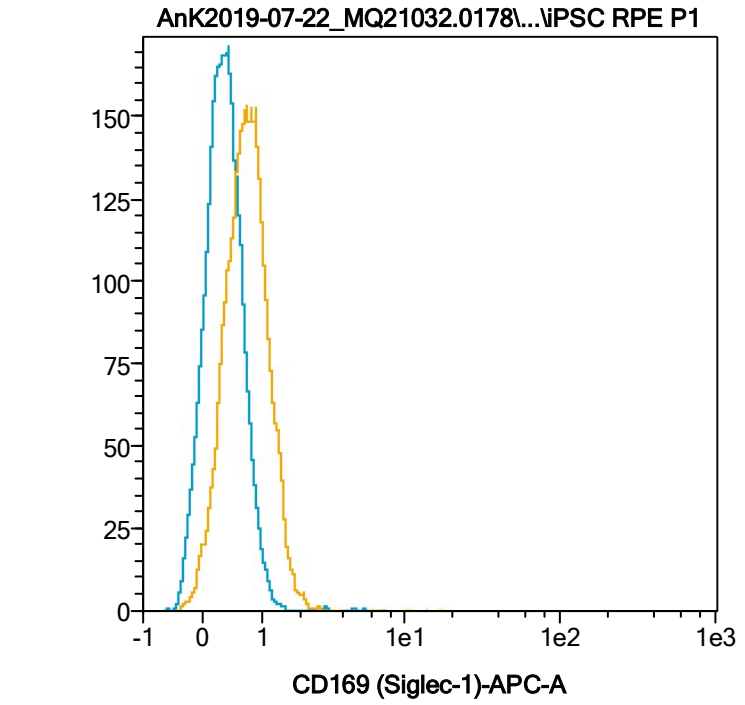

Name	%-#	CD169 (Siglec-1)-APC-A Mean	CD169 (Siglec-1)-APC-A Median
POS-unpigm.	2,11	2,94	1,30
POS-pigm.	2,36	2,93	1,87

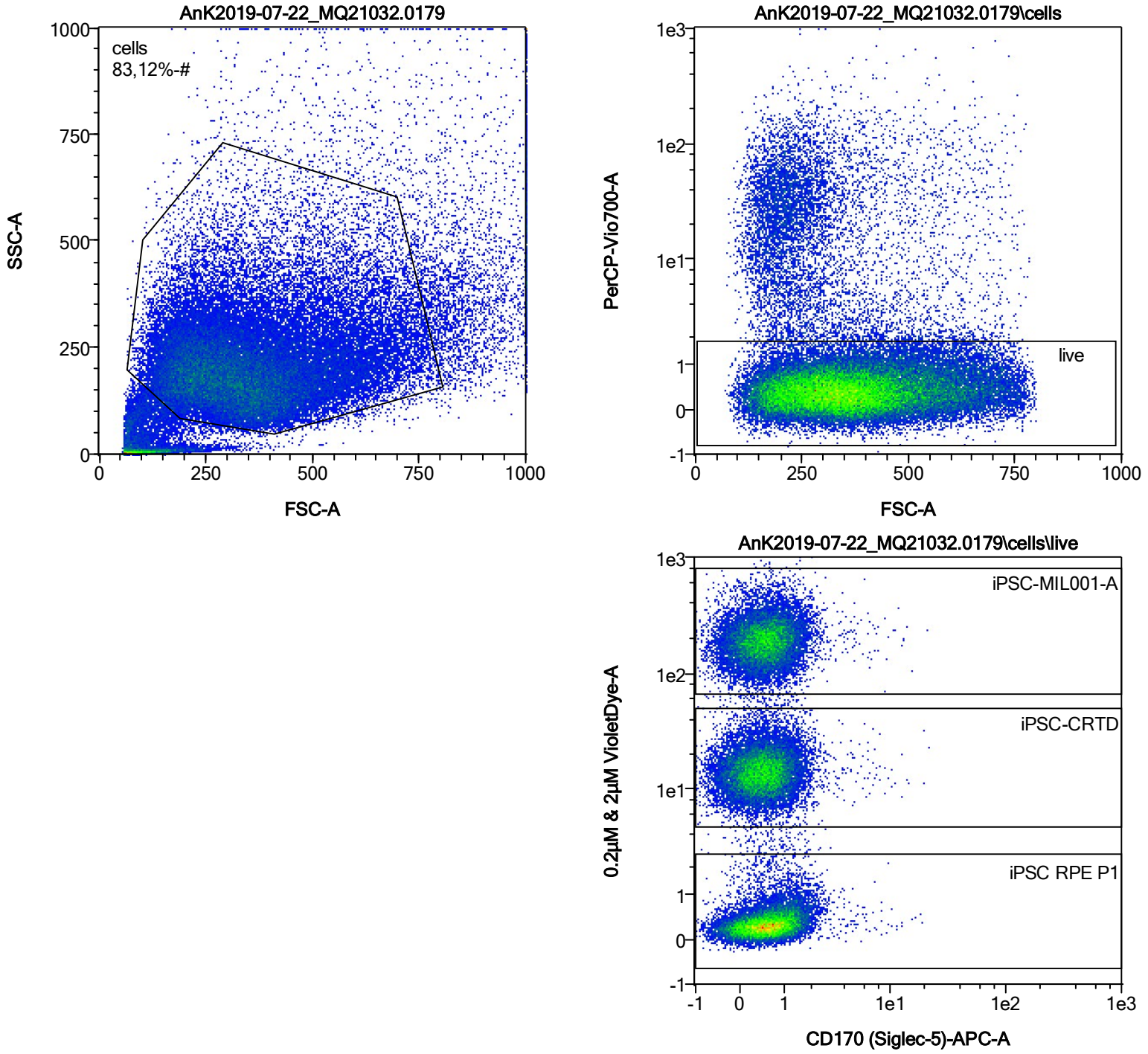

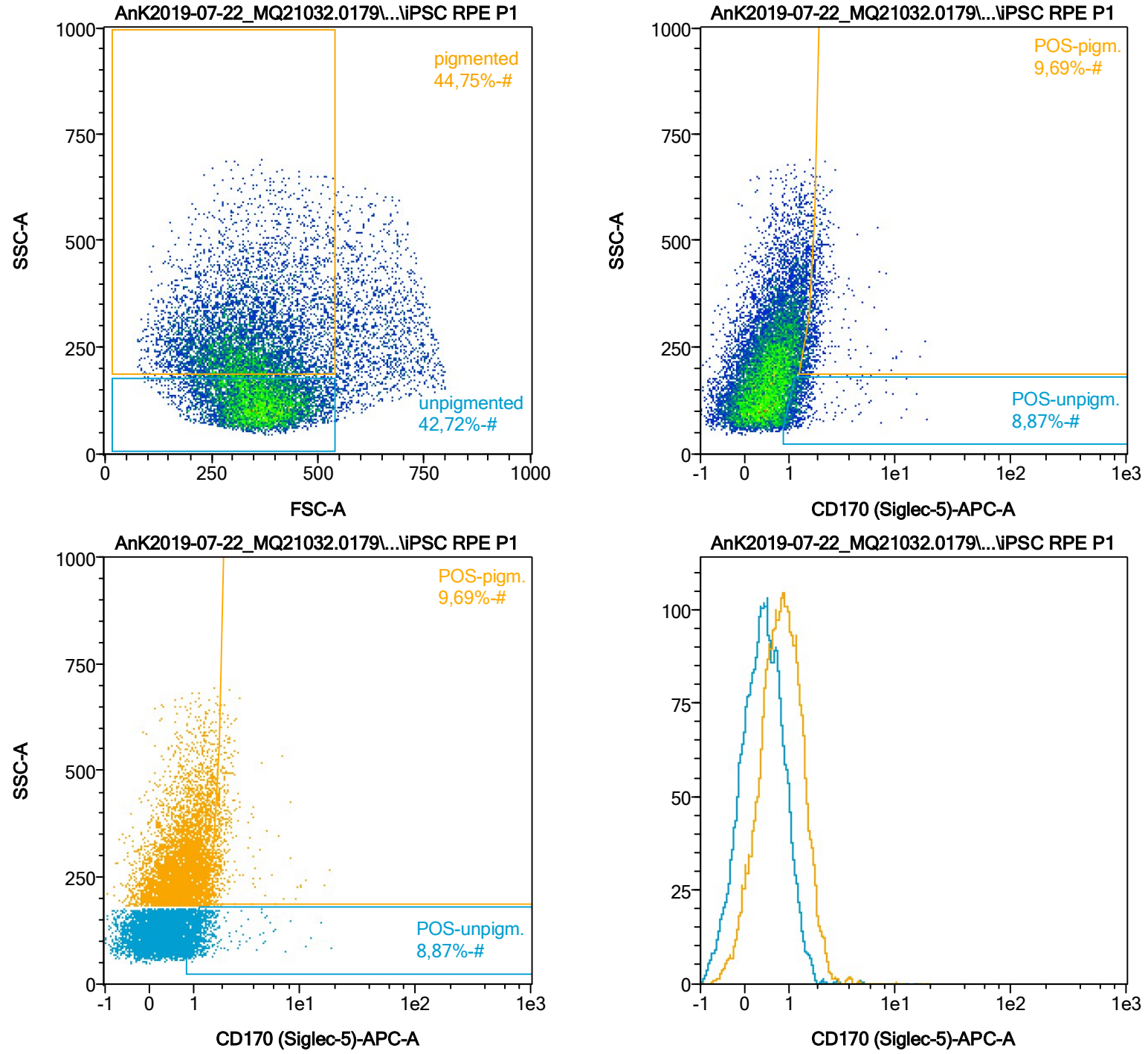

Name	%-#	CD170 (Siglec-5)-APC-A Mean	CD170 (Siglec-5)-APC-A Median
POS-unpigm.	8,87	1,51	1,22
POS-pigm.	9,69	2,10	1,84

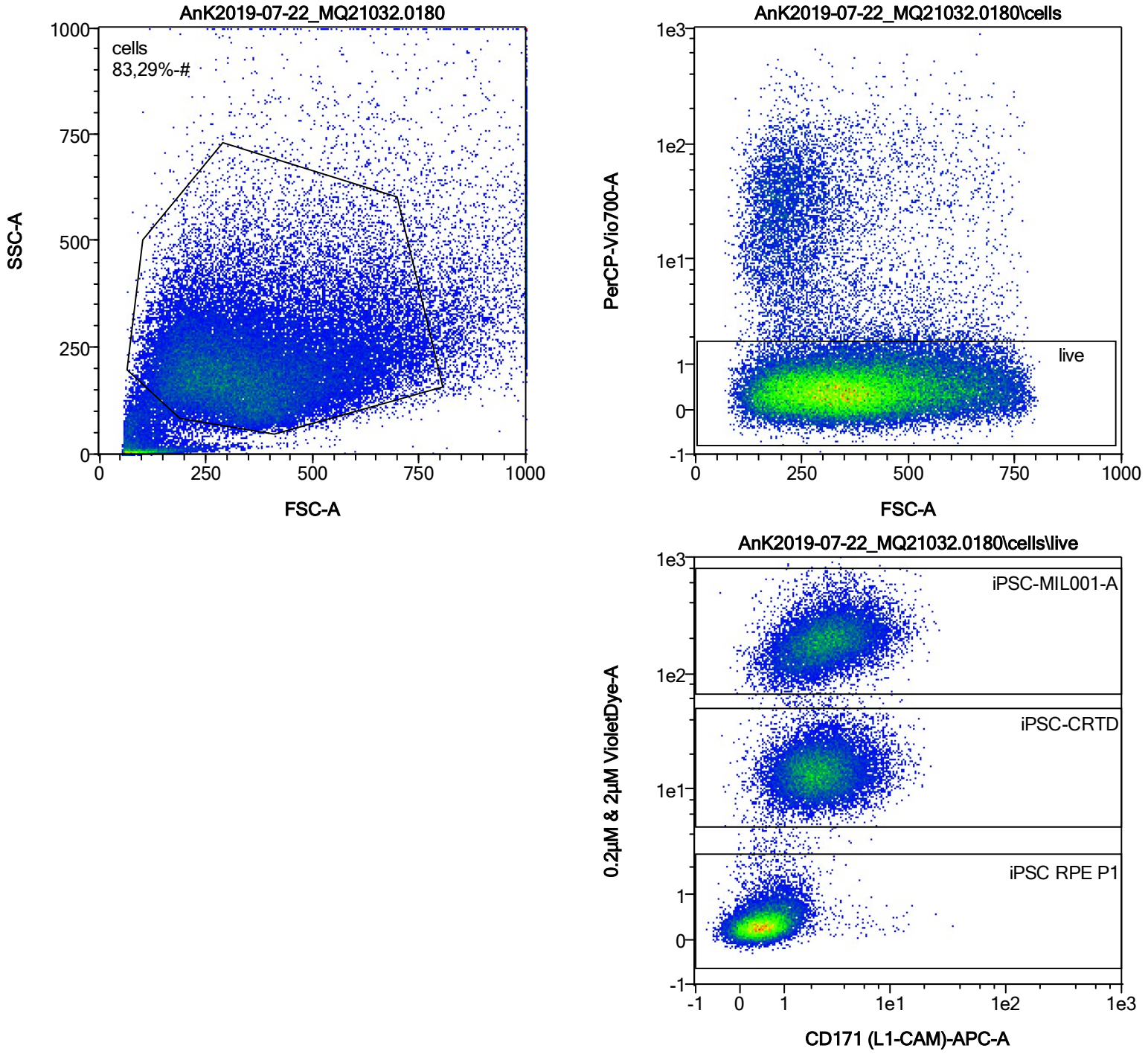

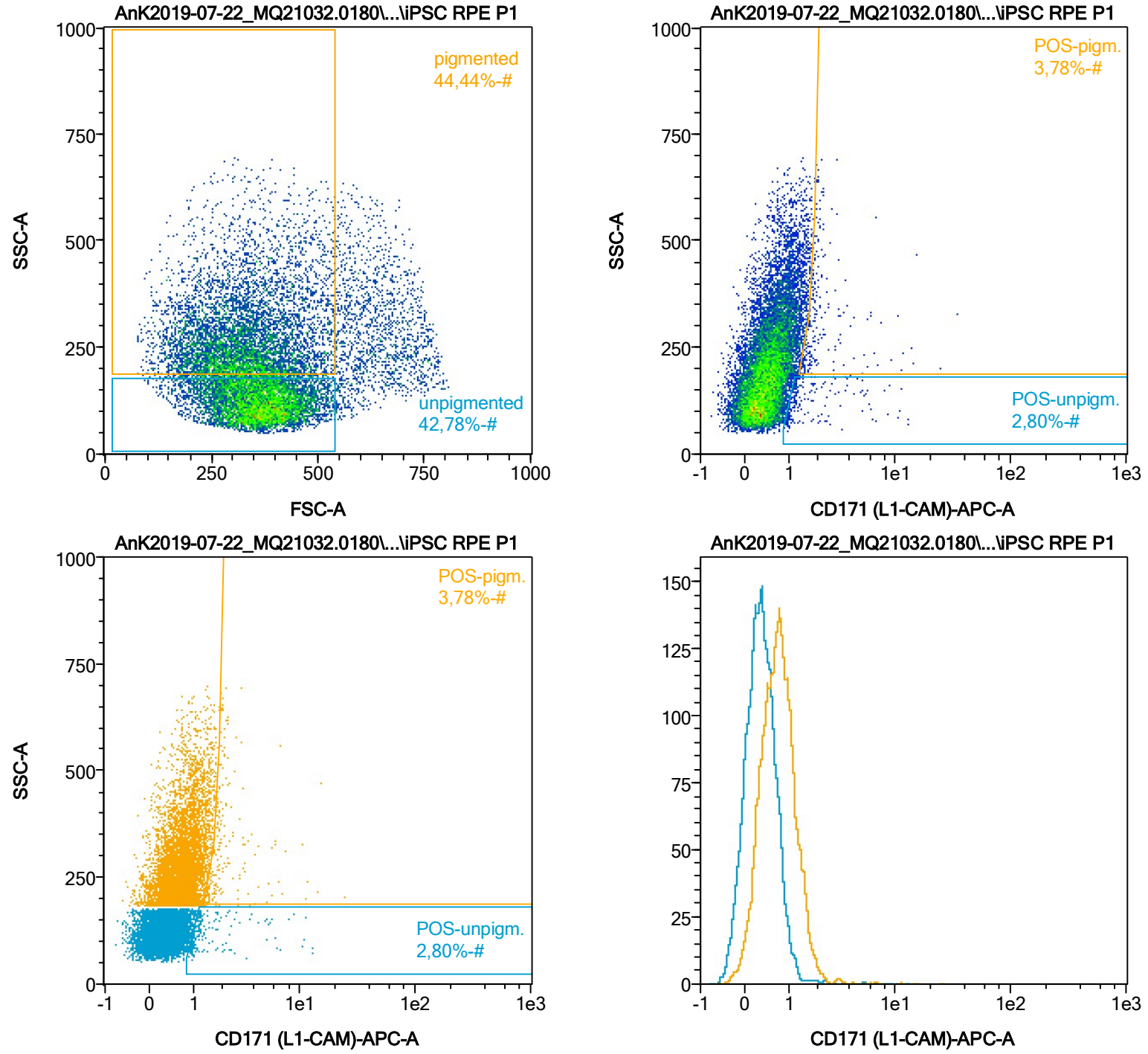

Name	%-#	CD171 (L1-CAM)-APC-A Mean	CD171 (L1-CAM)-APC-A Median
POS-unpigm.	2,80	2,00	1,20
POS-pigm.	3,78	2,46	1,86

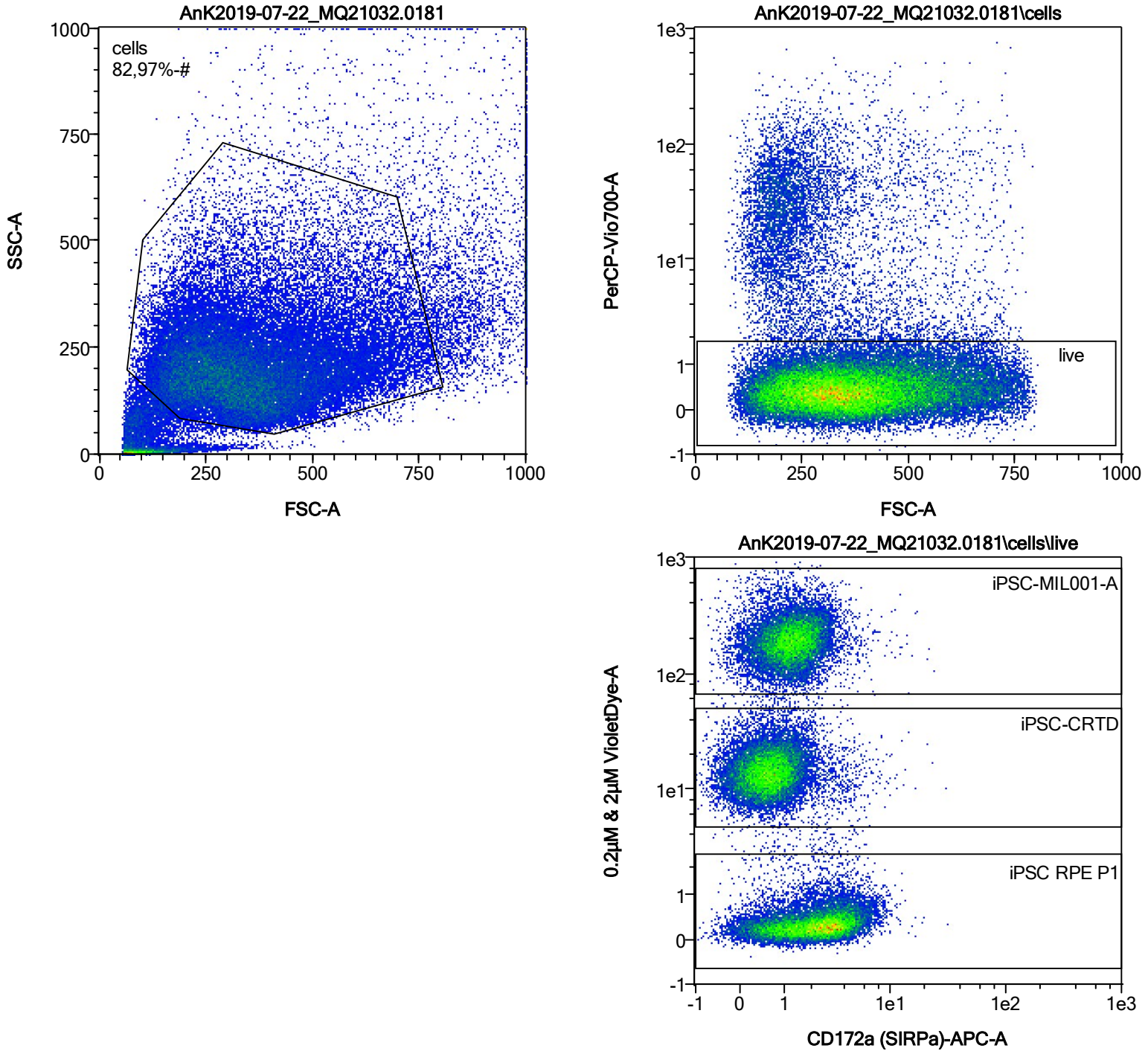

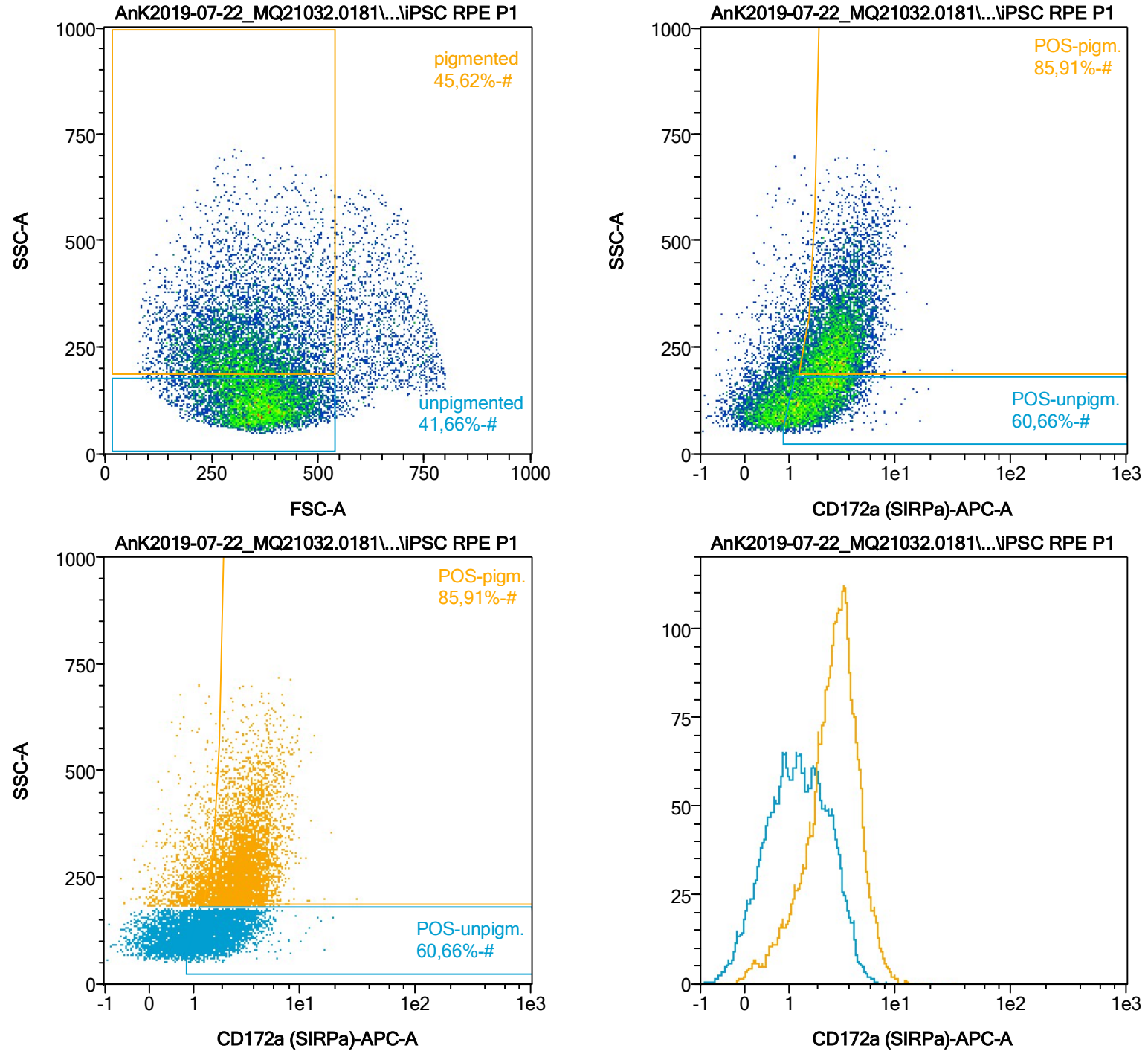

Name	%-#	CD172a (SIRPa)-APC-A Mean	CD172a (SIRPa)-APC-A Median
POS-unpigm.	60,66	2,15	1,91
POS-pigm.	85,91	3,44	3,19

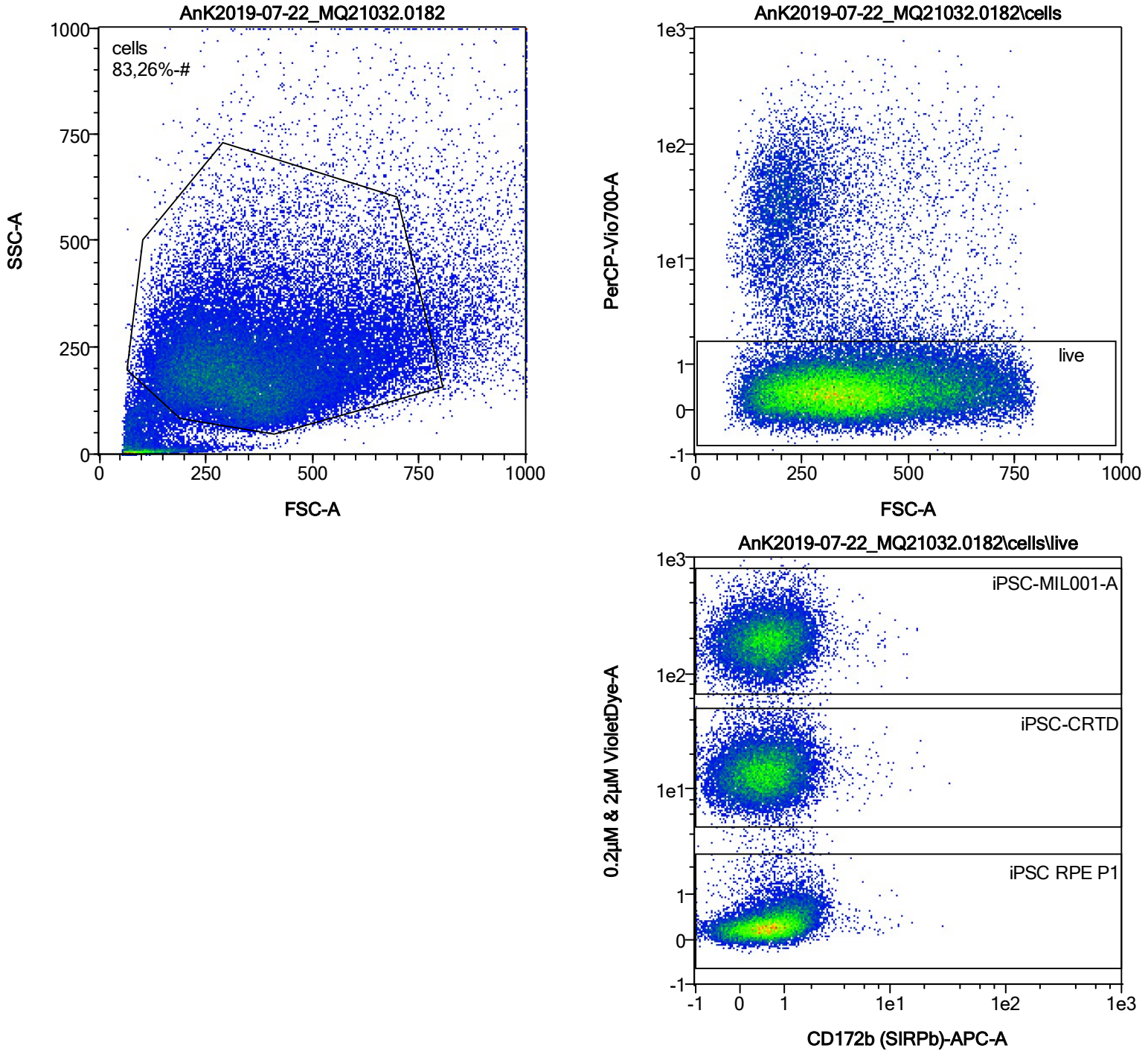

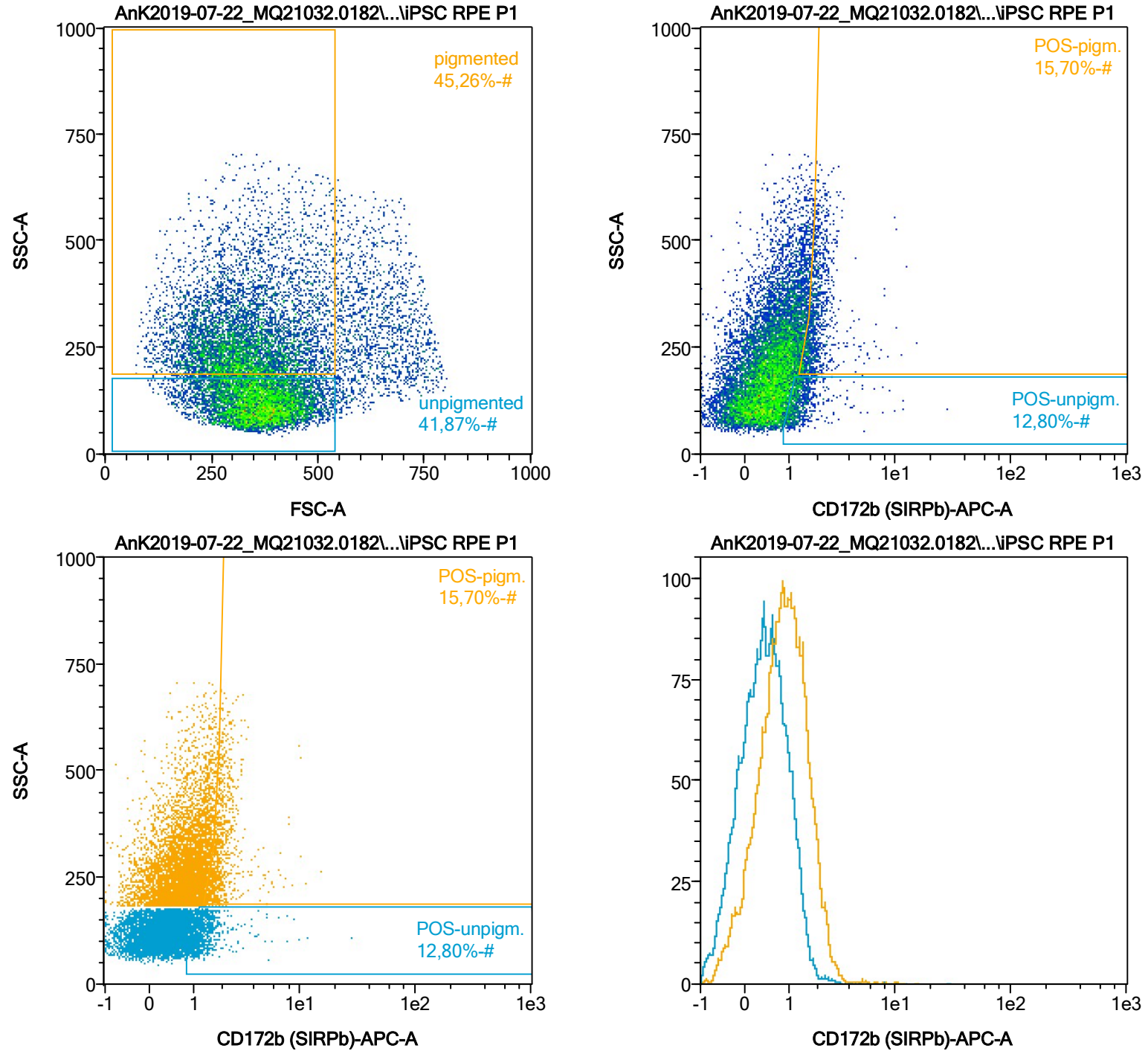

Name	%-#	CD172b (SIRPb)-APC-A Mean	CD172b (SIRPb)-APC-A Median
POS-unpigm.	12,80	1,46	1,24
POS-pigm.	15,70	2,08	1,88

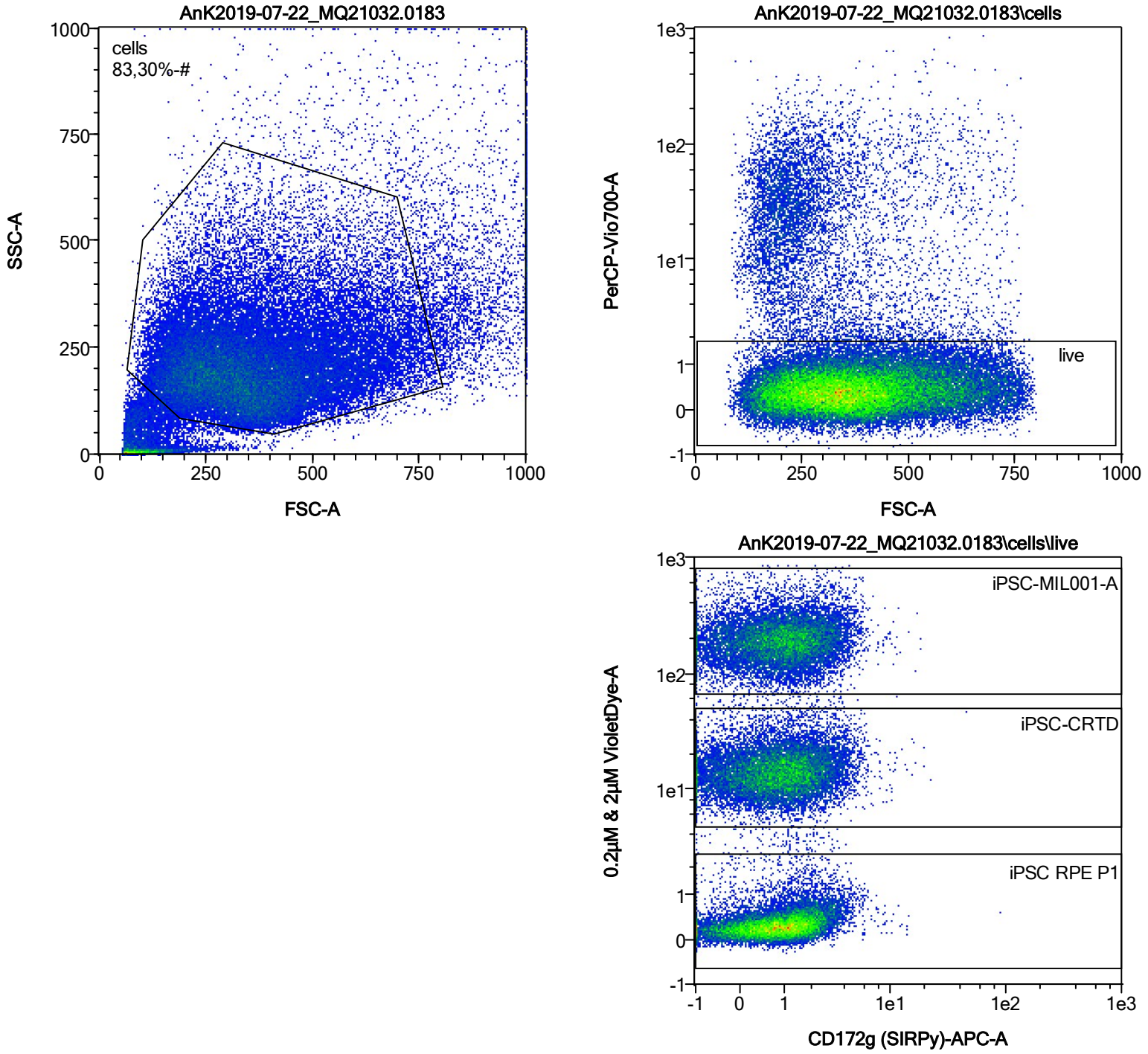

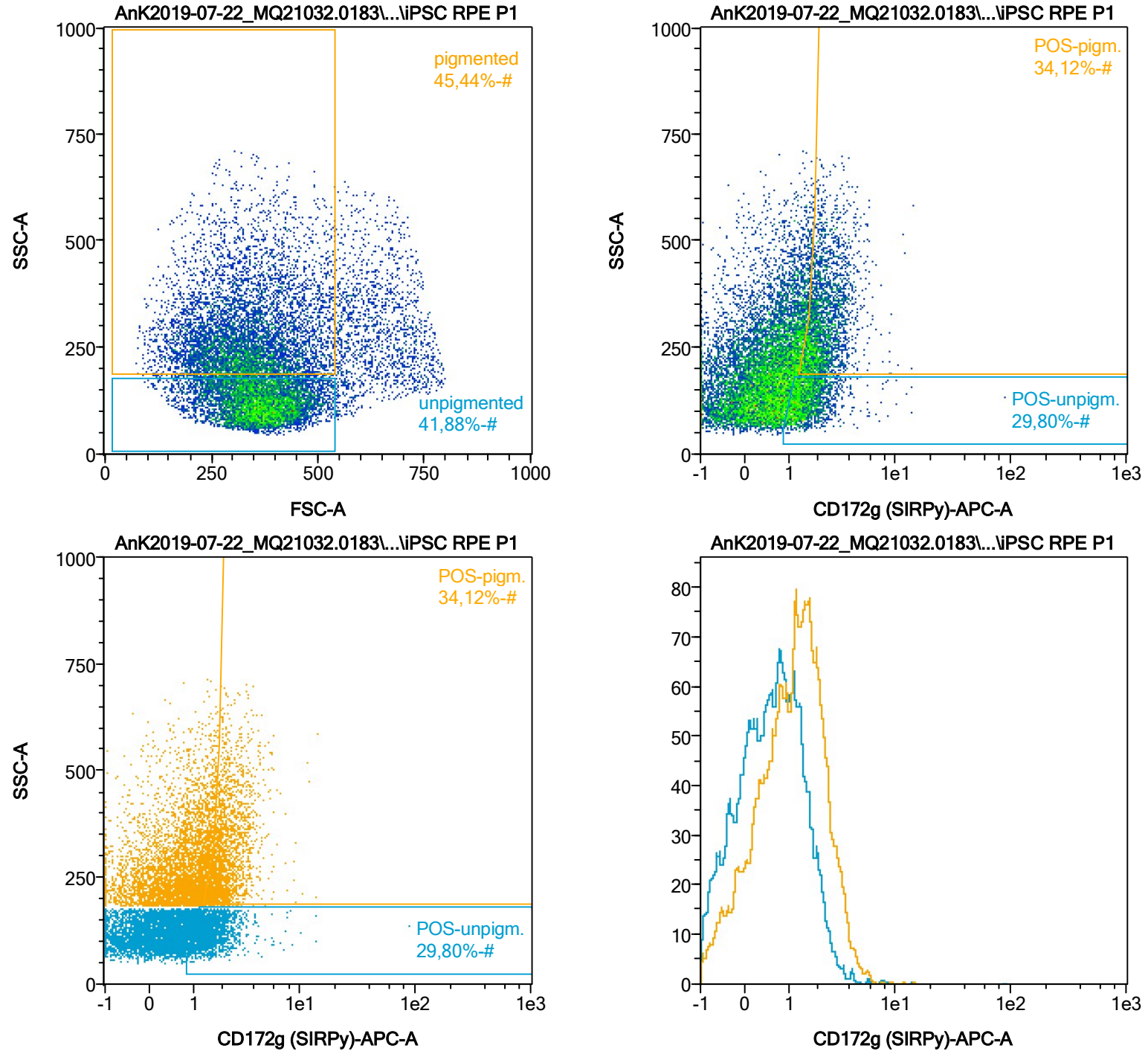

Name	%-#	CD172g (SIRPγ)-APC-A Mean	CD172g (SIRPγ)-APC-A Median
POS-unpigm.	29,80	1,70	1,45
POS-pigm.	34,12	2,36	2,11

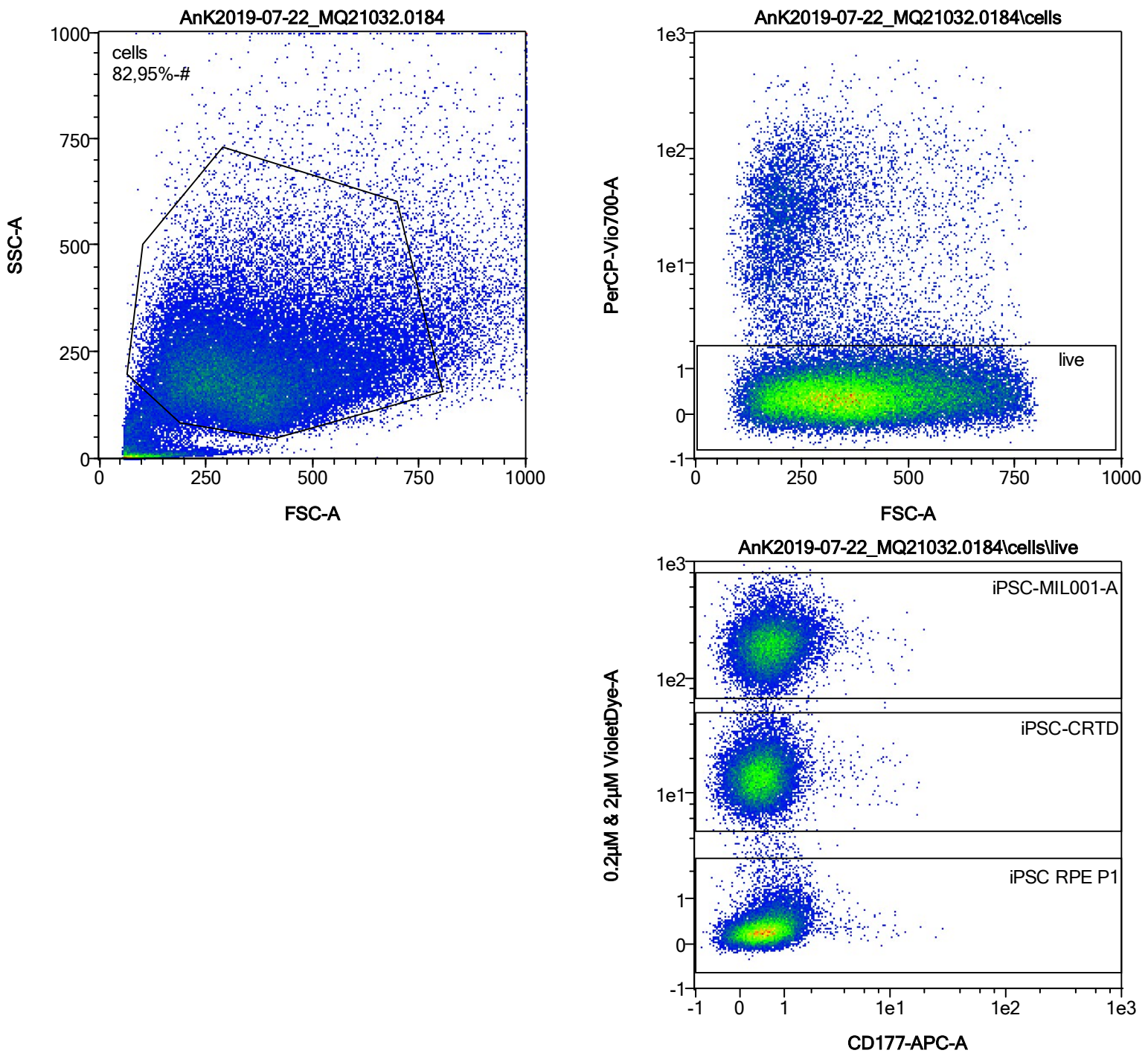

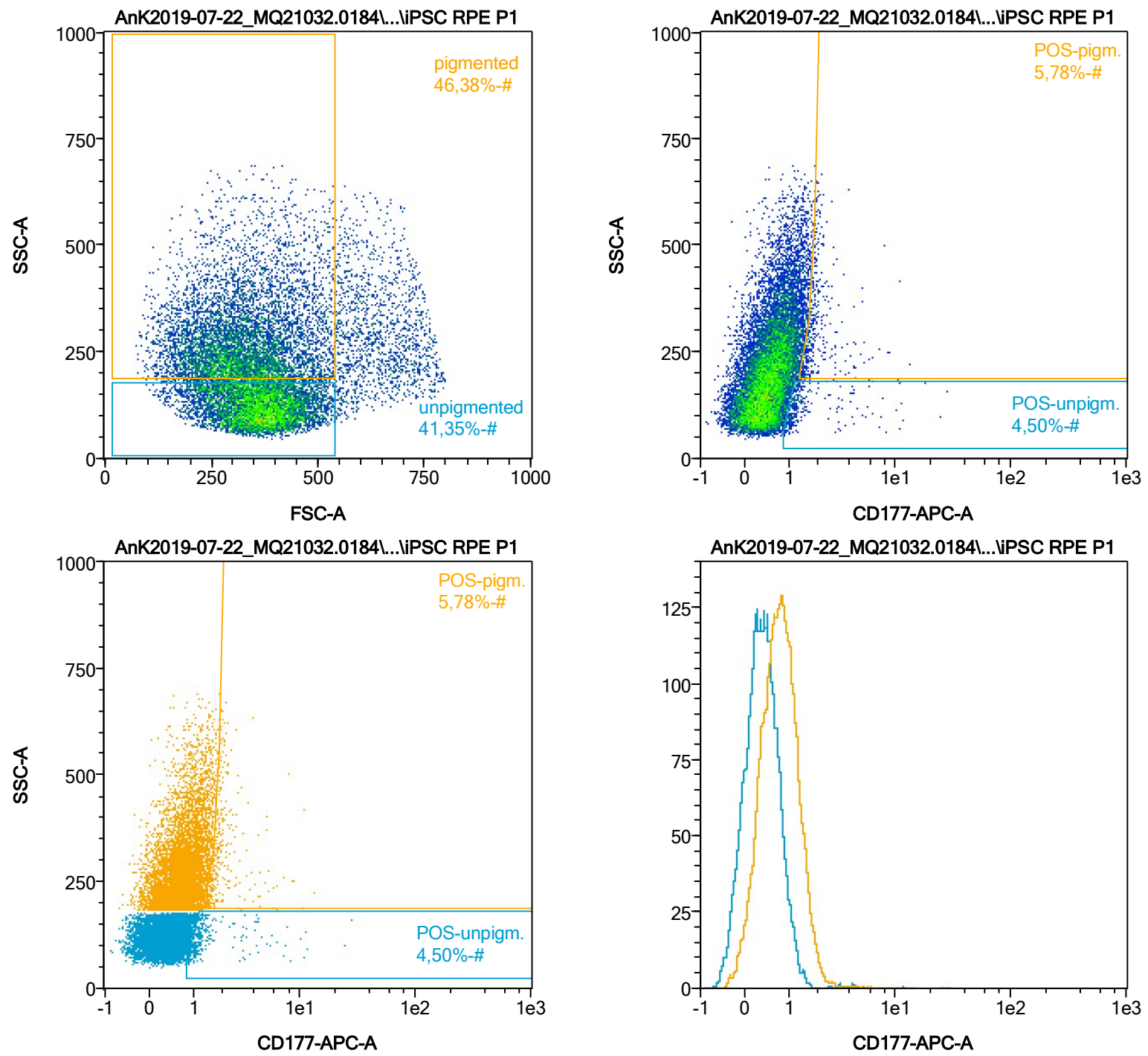

Name	%-#	CD177-APC-A Mean	CD177-APC-A Median
POS-unpigm.	4,50	1,91	1,19
POS-pigm.	5,78	2,16	1,81

Date:2024-06-14

Operator: Sebastian Knoebel

Version:2.13.0+54.g95abf0f4

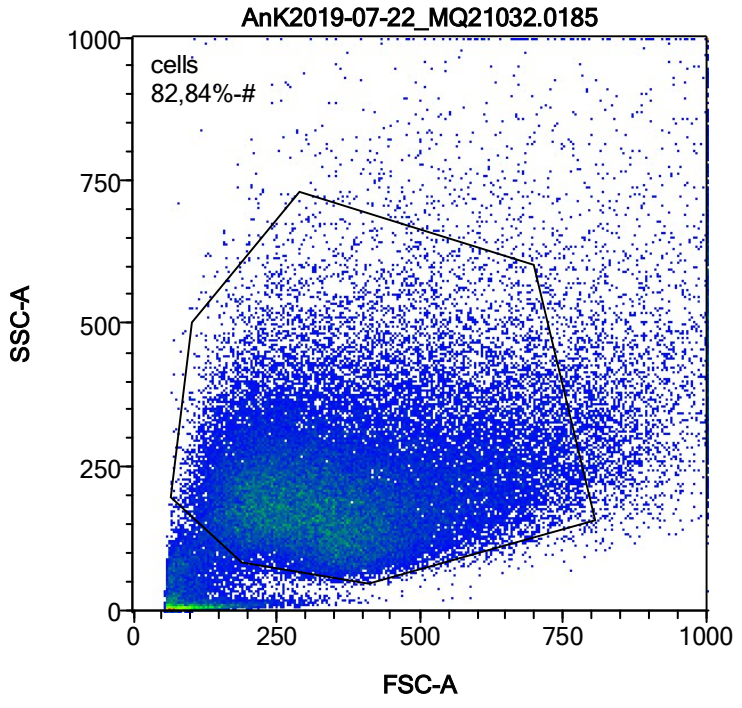

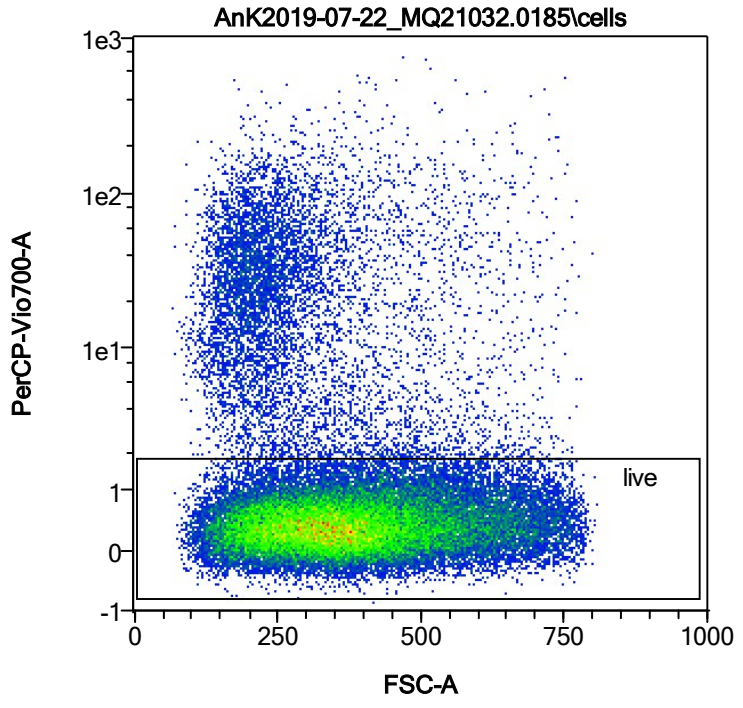

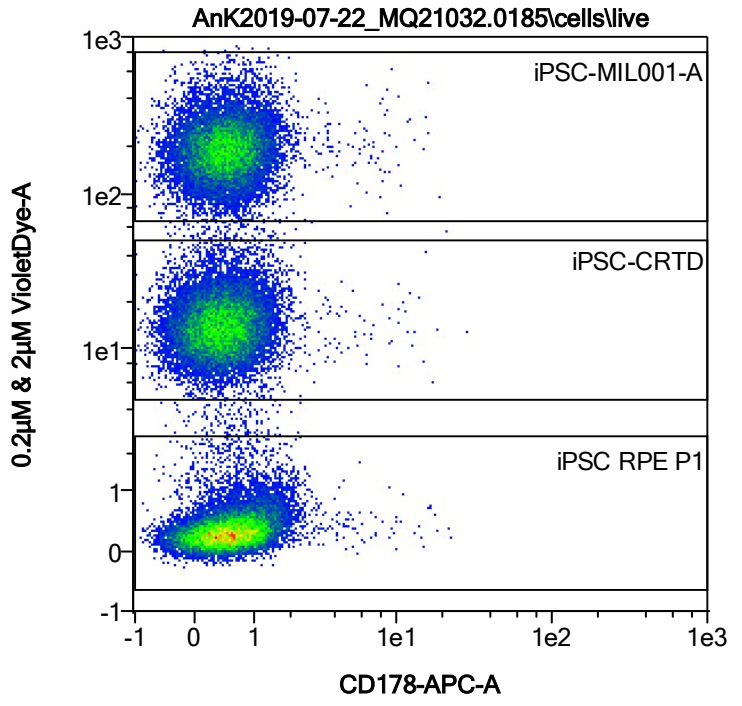

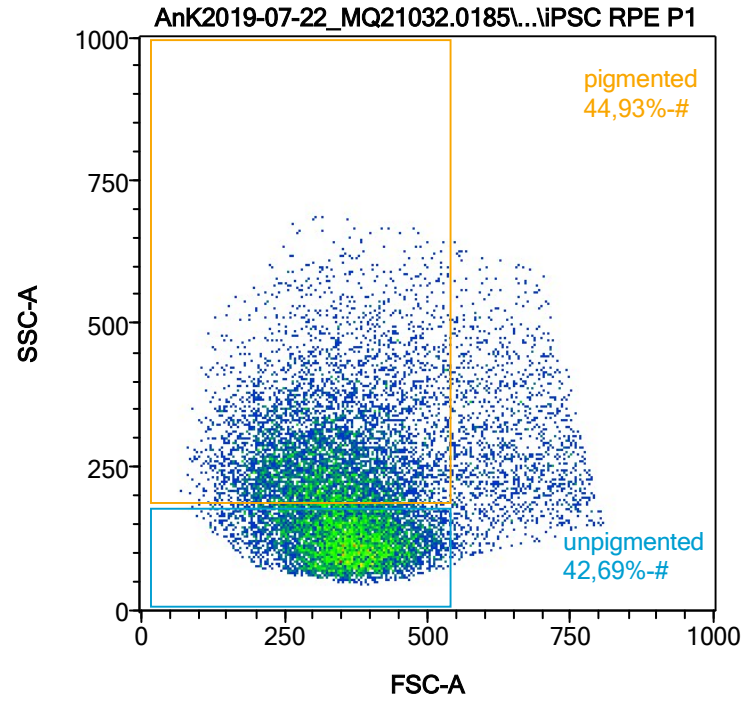

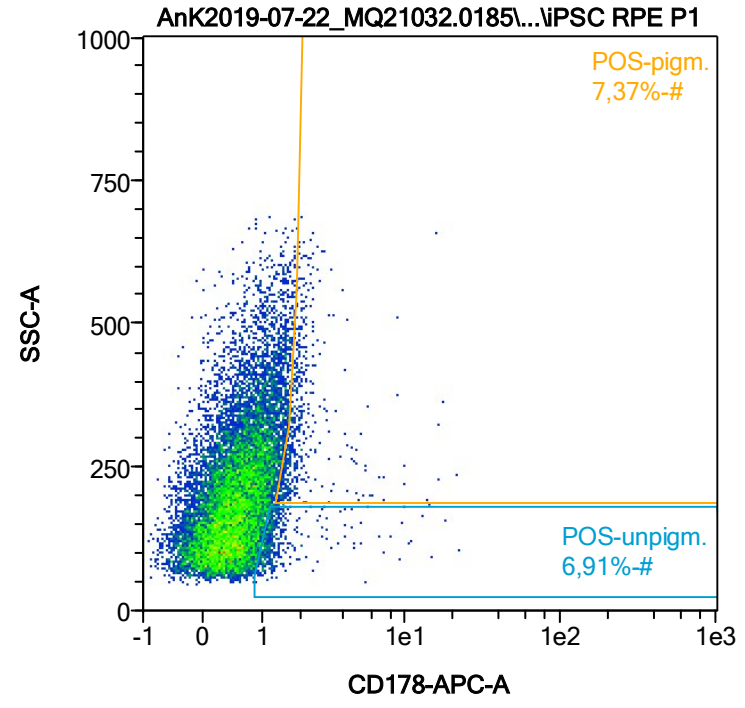

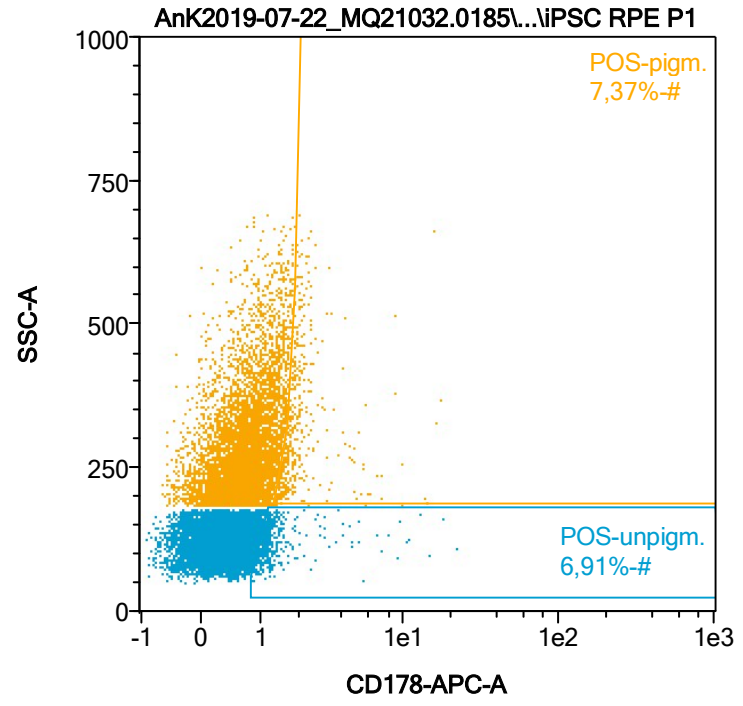

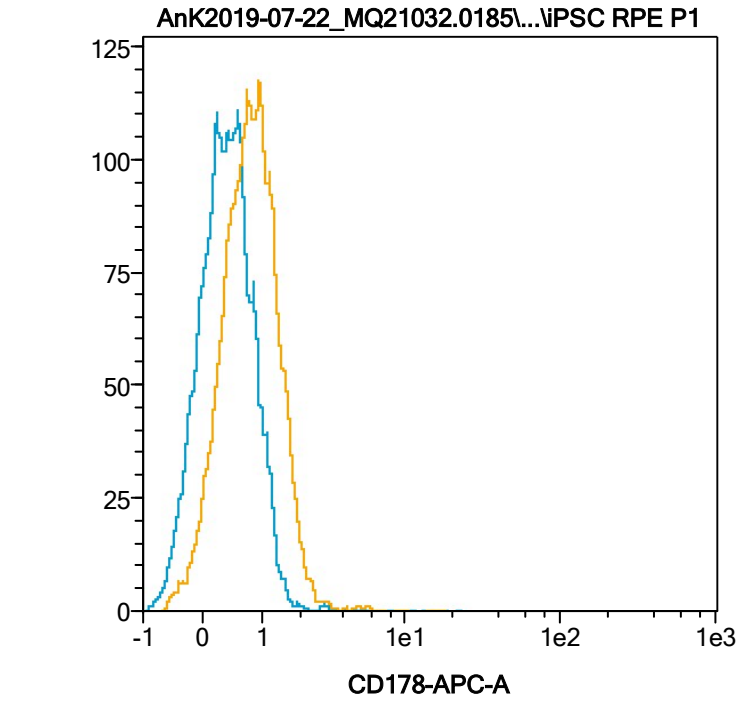

Name	%-#	CD178-APC-A Mean	CD178-APC-A Median
POS-unpigm.	6,91	1,61	1,18
POS-pigm.	7,37	2,26	1,84

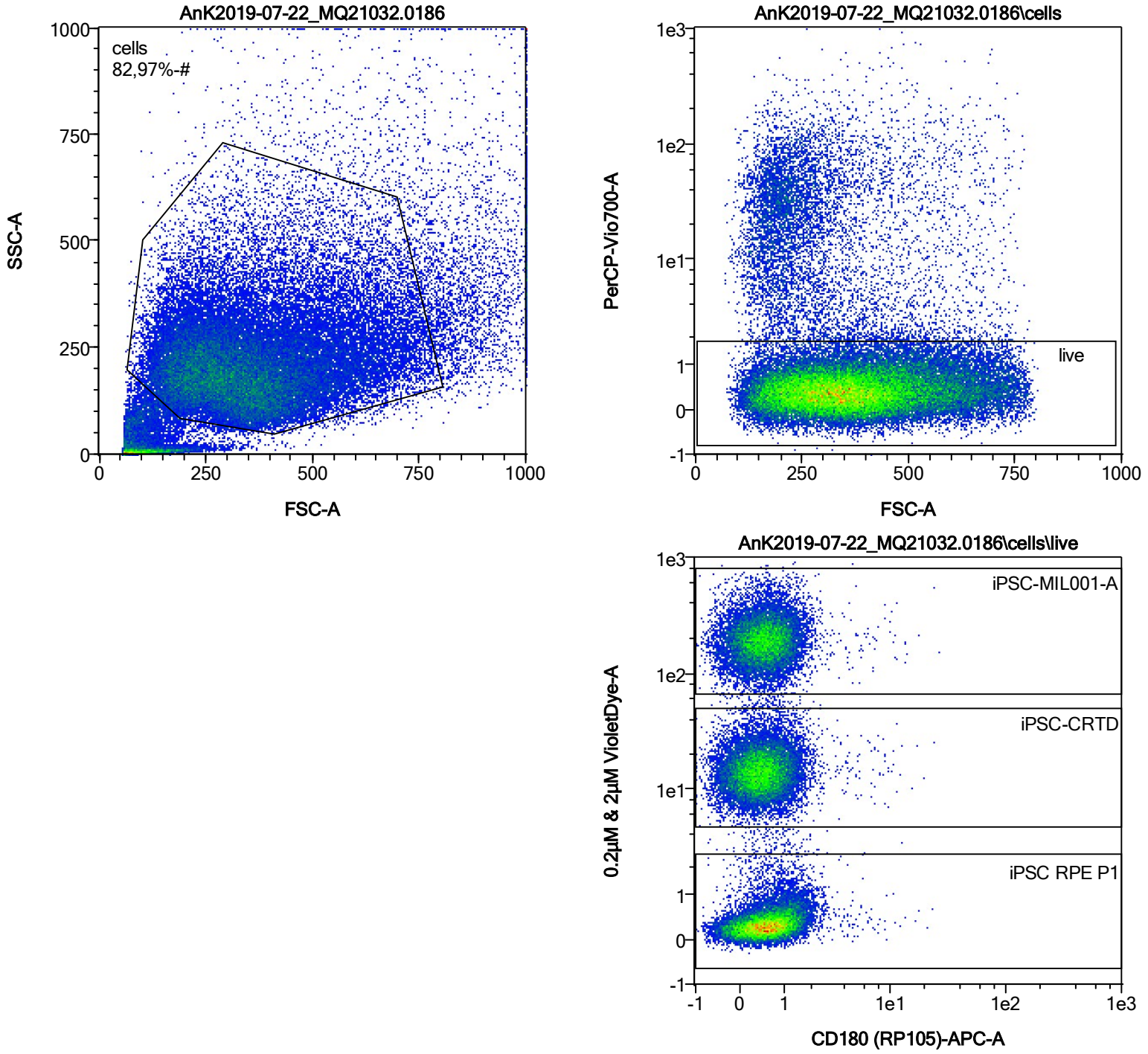

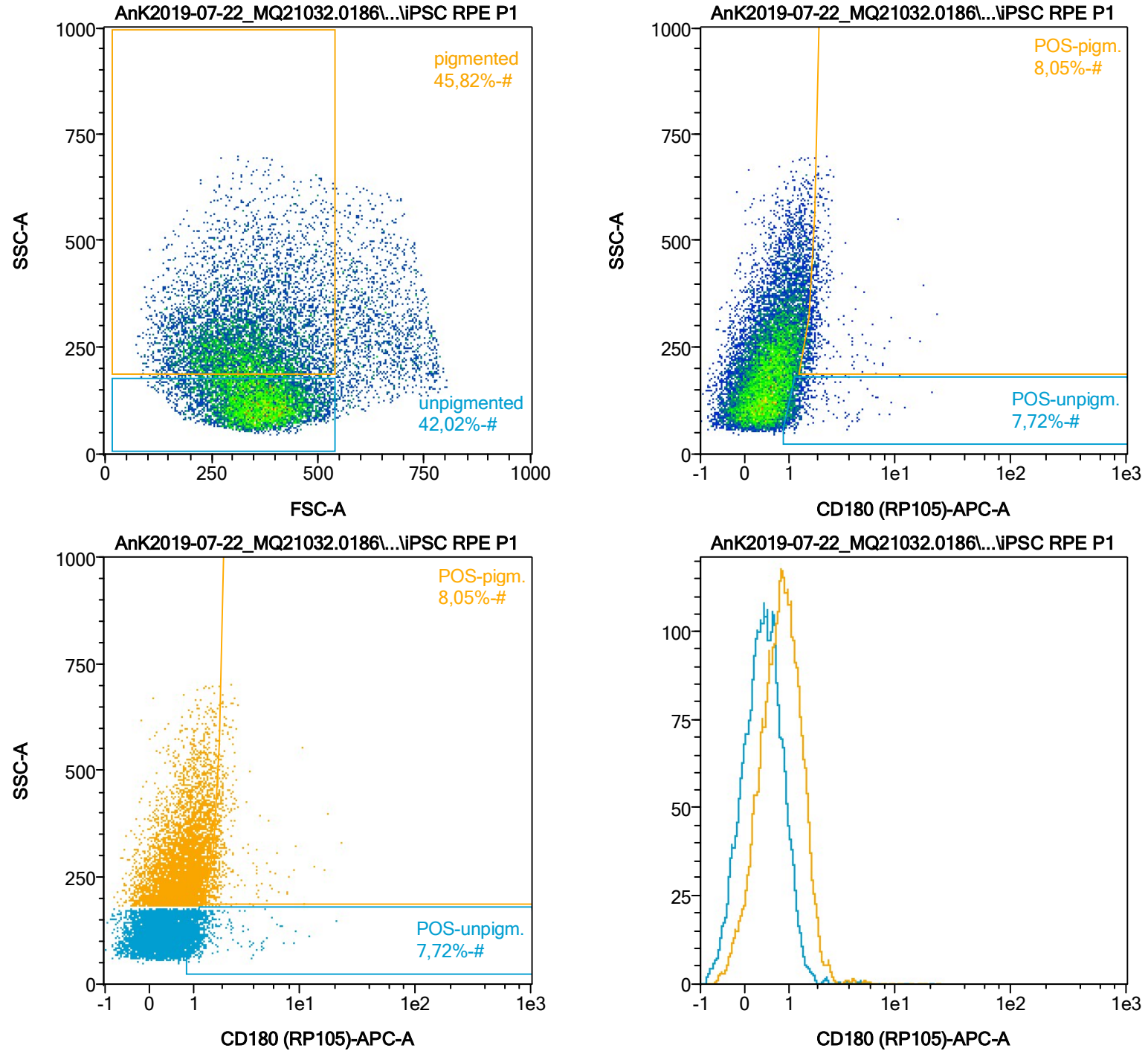

Name	%-#	CD180 (RP105)-APC-A Mean	CD180 (RP105)-APC-A Median
POS-unpigm.	7,72	1,54	1,20
POS-pigm.	8,05	2,21	1,84

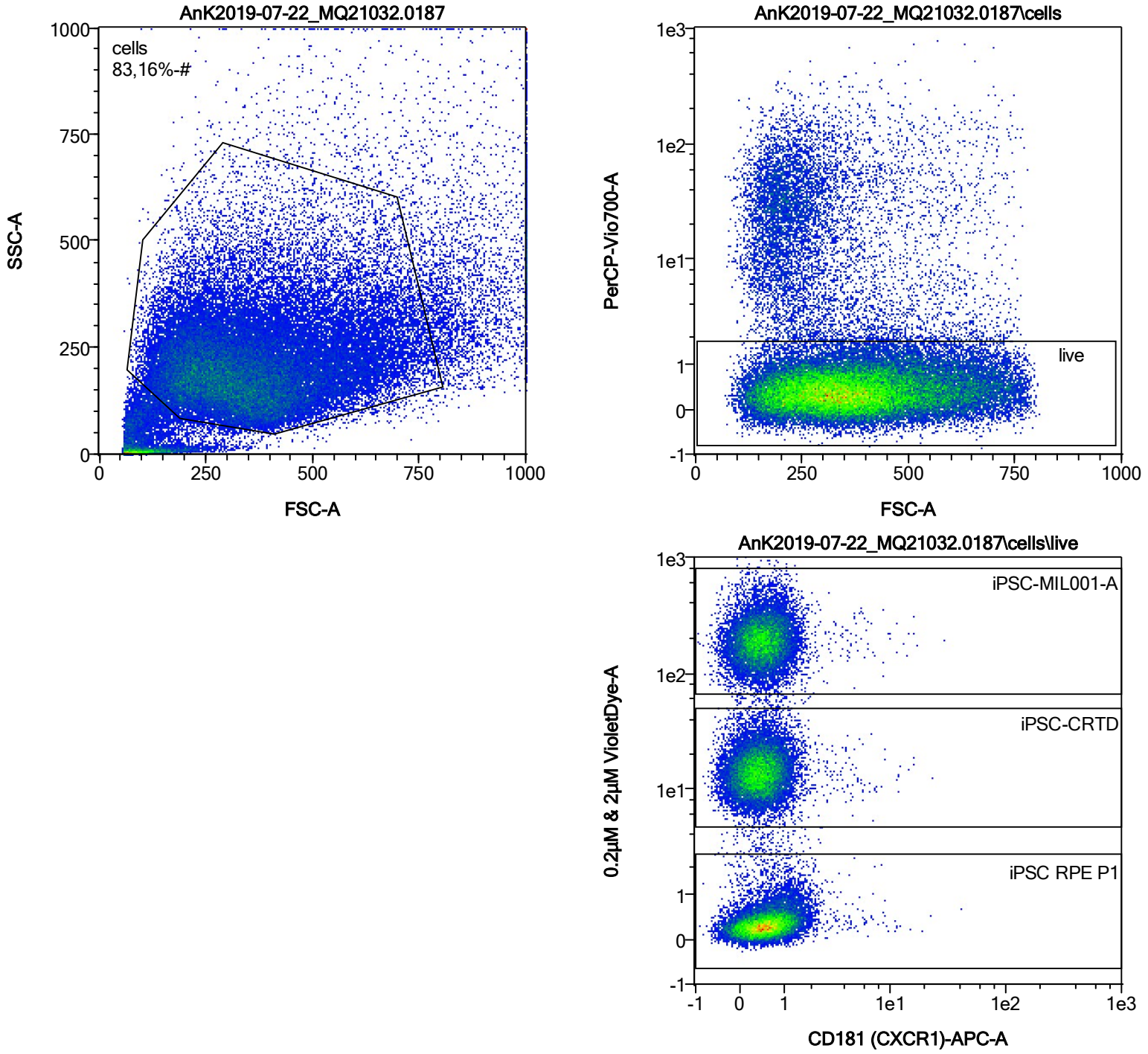

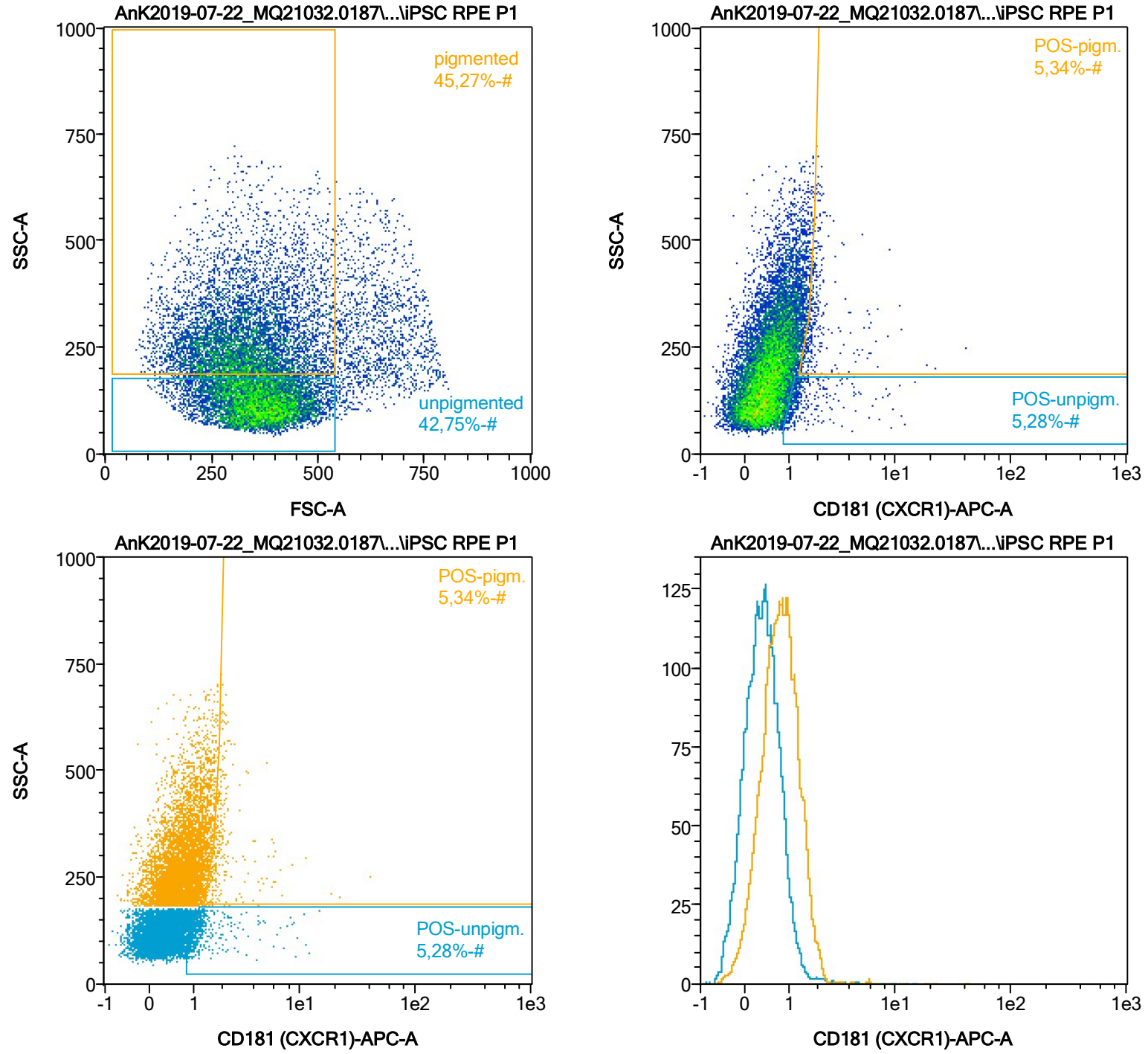

Name	%-#	CD181 (CXCR1)-APC-A Mean	CD181 (CXCR1)-APC-A Median
POS-unpigm.	5,28	1,78	1,22
POS-pigm.	5,34	2,34	1,85

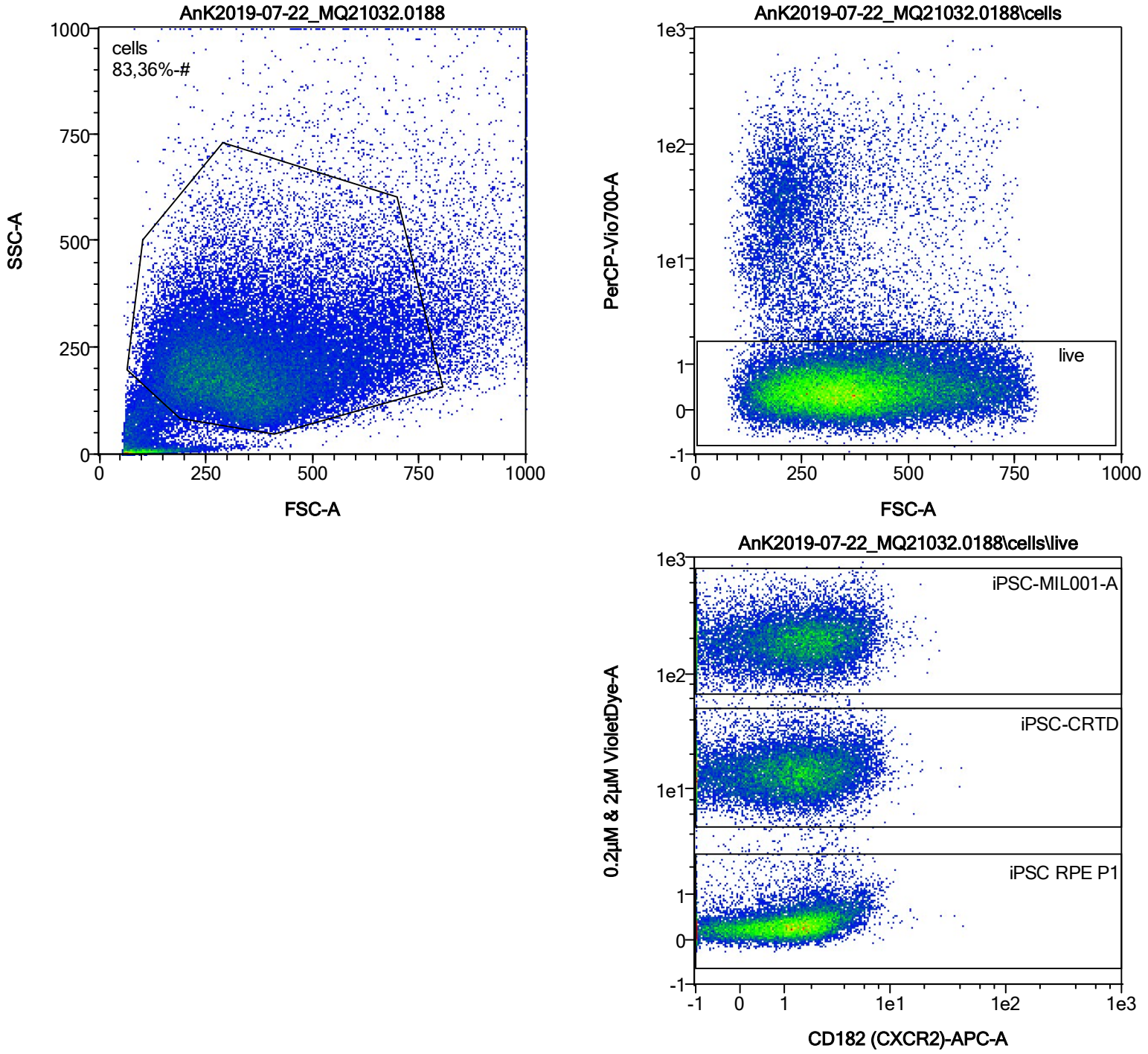

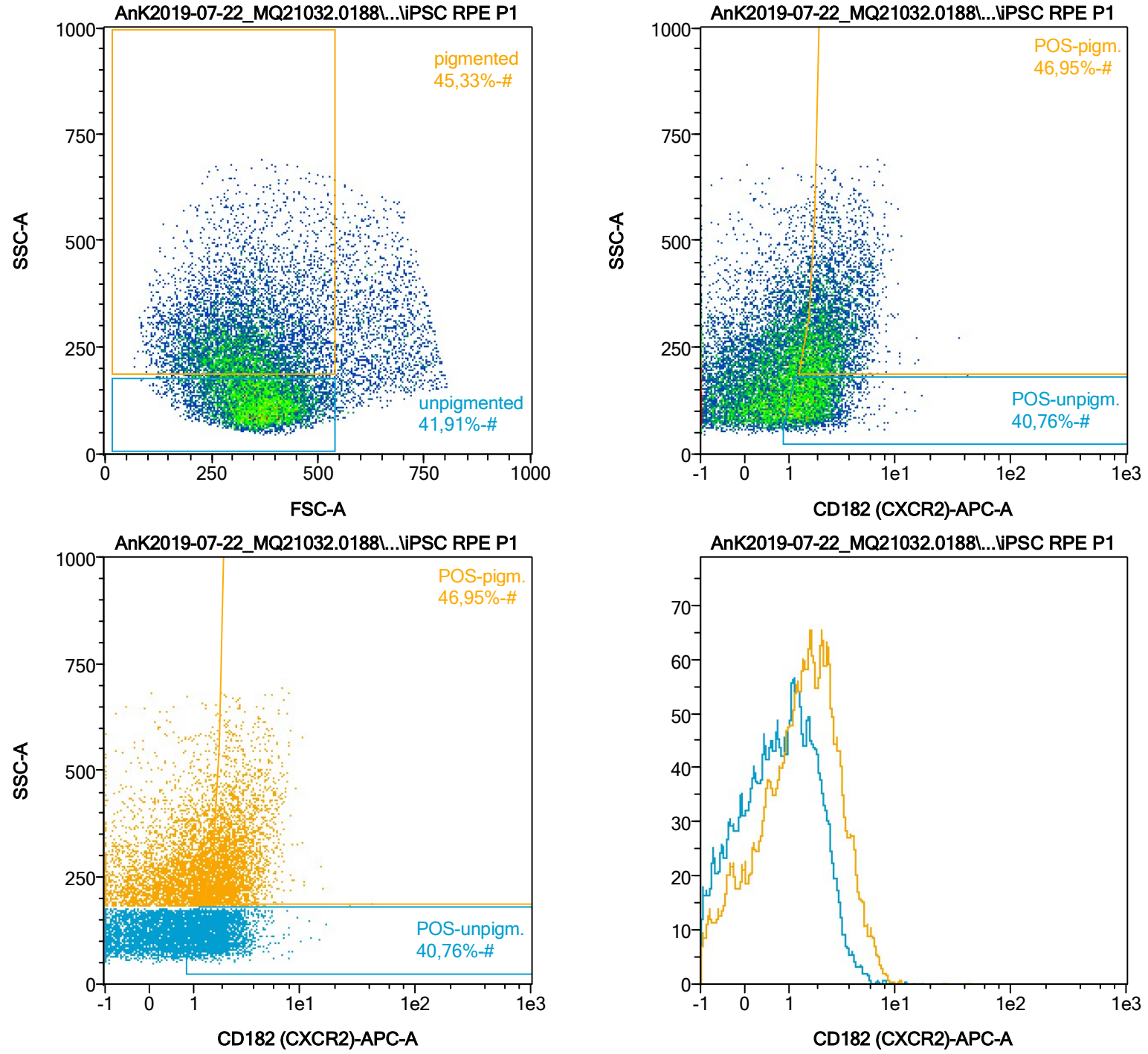

Name	%-#	CD182 (CXCR2)-APC-A Mean	CD182 (CXCR2)-APC-A Median
POS-unpigm.	40,76	1,97	1,73
POS-pigm.	46,95	2,88	2,48

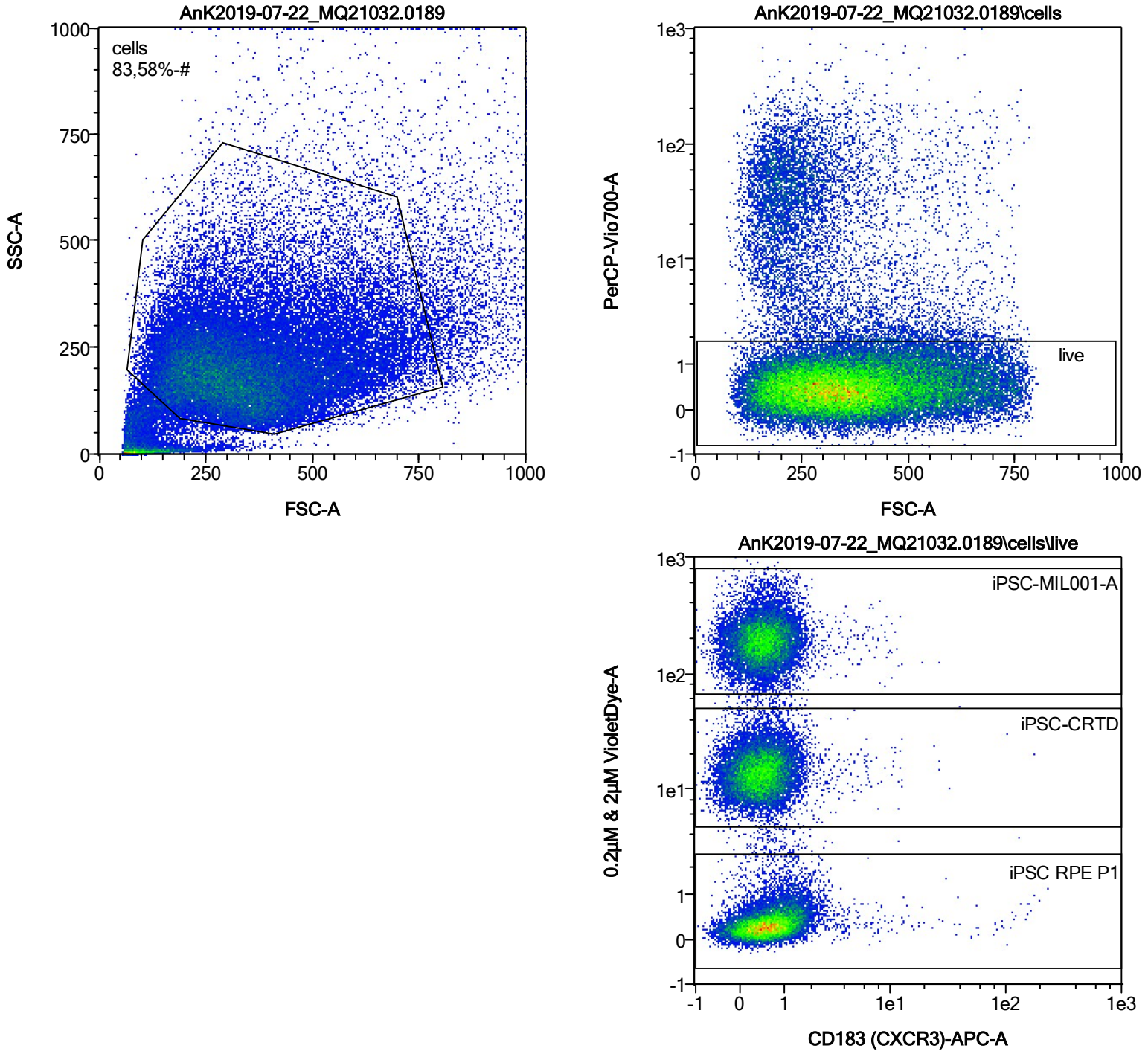

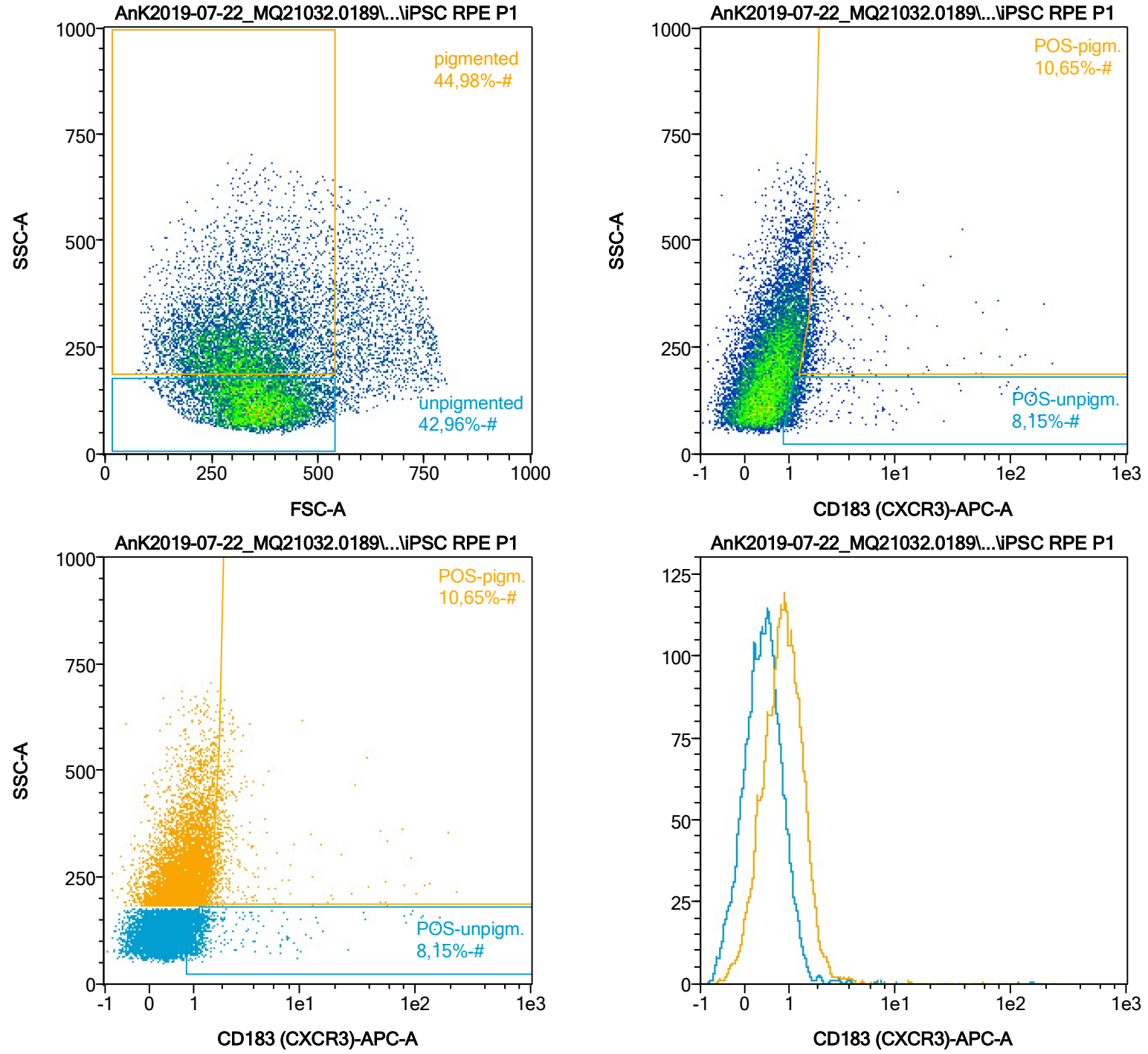

Name	%-#	CD183 (CXCR3)-APC-A Mean	CD183 (CXCR3)-APC-A Median
POS-unpigm.	8,15	5,21	1,25
POS-pigm.	10,65	4,39	1,85

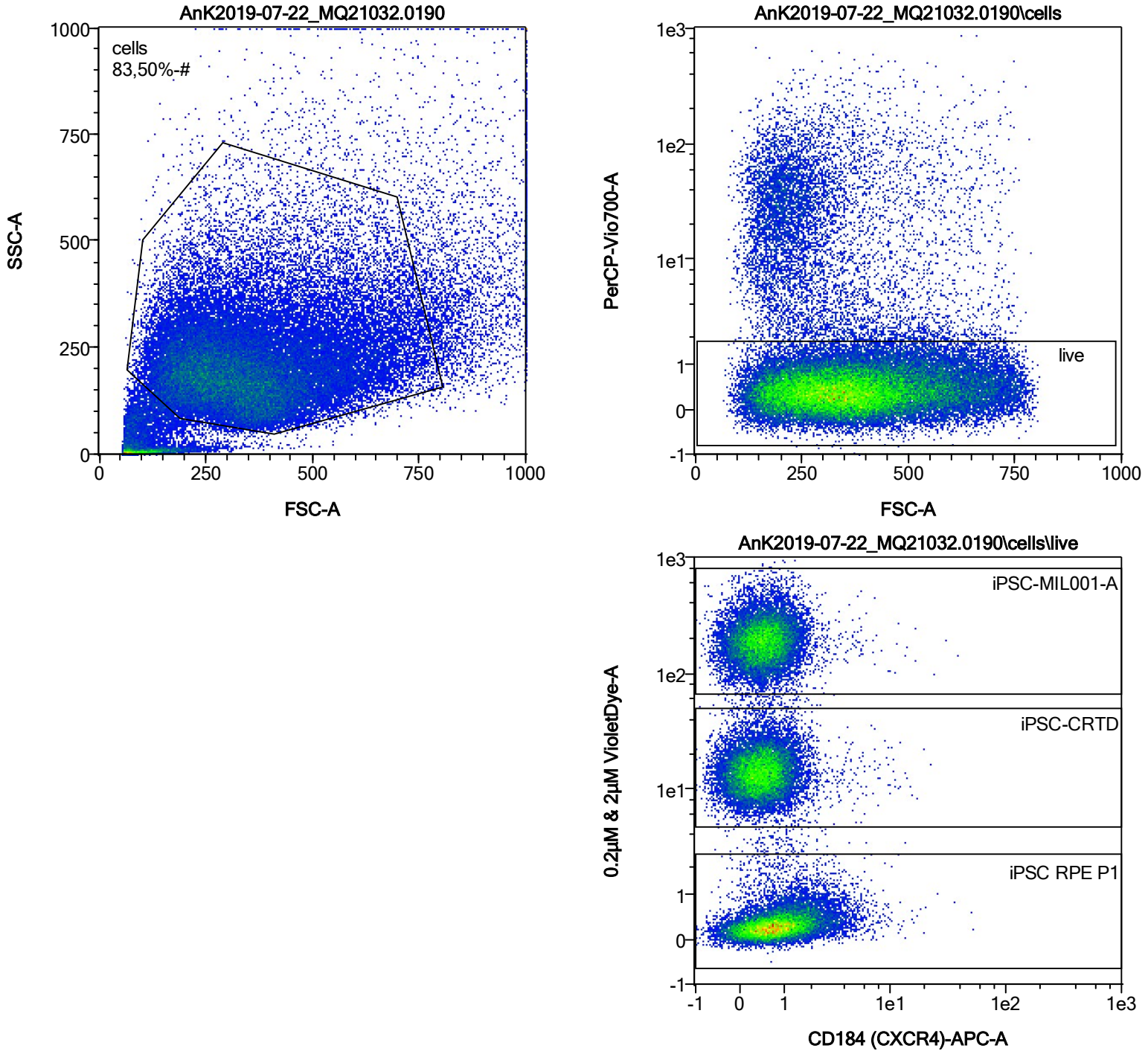

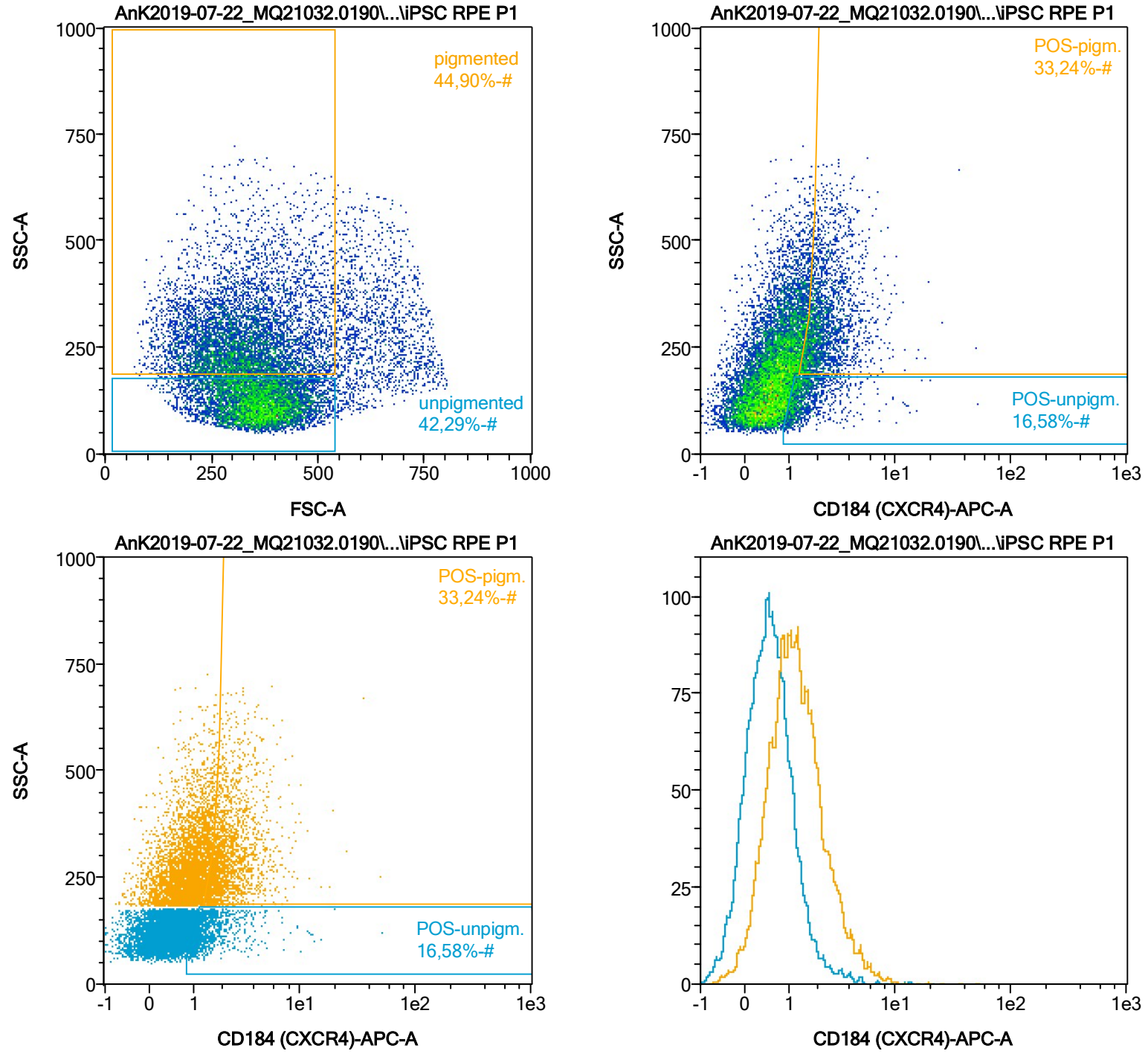

Name	%-#	CD184 (CXCR4)-APC-A Mean	CD184 (CXCR4)-APC-A Median
POS-unpigm.	16,58	1,77	1,35
POS-pigm.	33,24	2,61	2,13

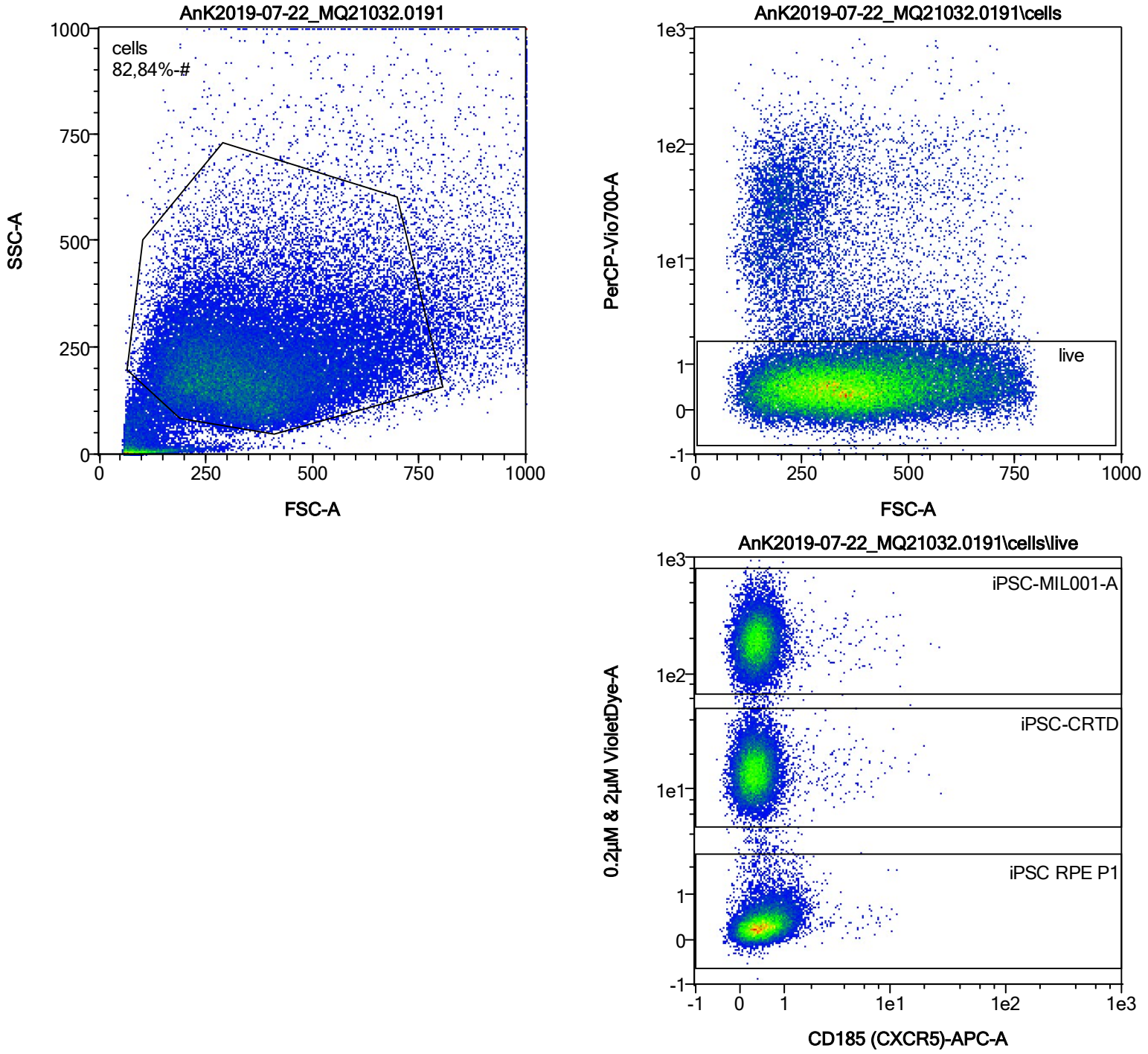

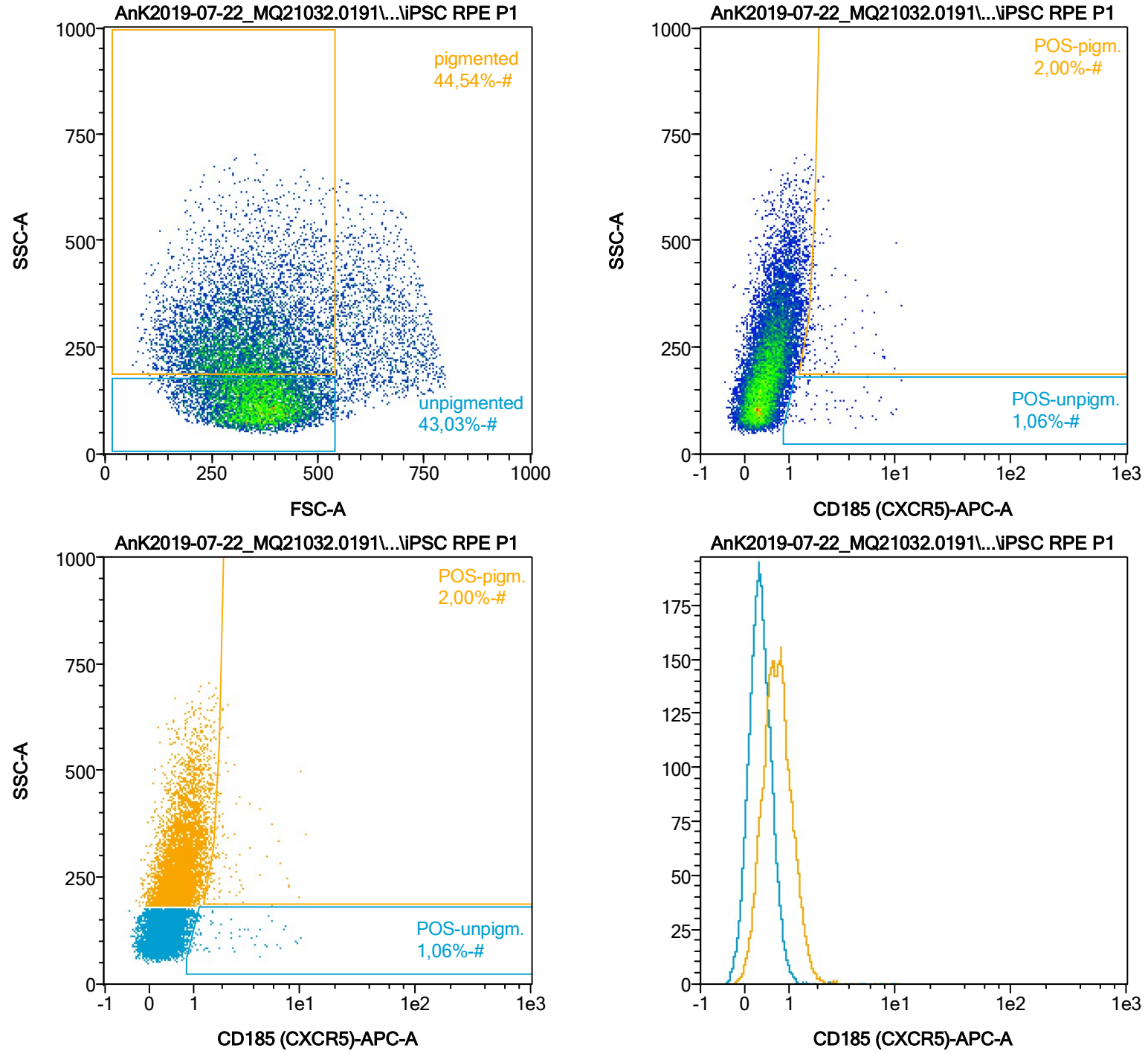

Name	%-#	CD185 (CXCR5)-APC-A Mean	CD185 (CXCR5)-APC-A Median
POS-unpigm.	1,06	2,73	1,62
POS-pigm.	2,00	2,50	1,88

Date:2024-06-14

Operator: Sebastian Knoebel

Version:2.13.0+54.g95abf0f4

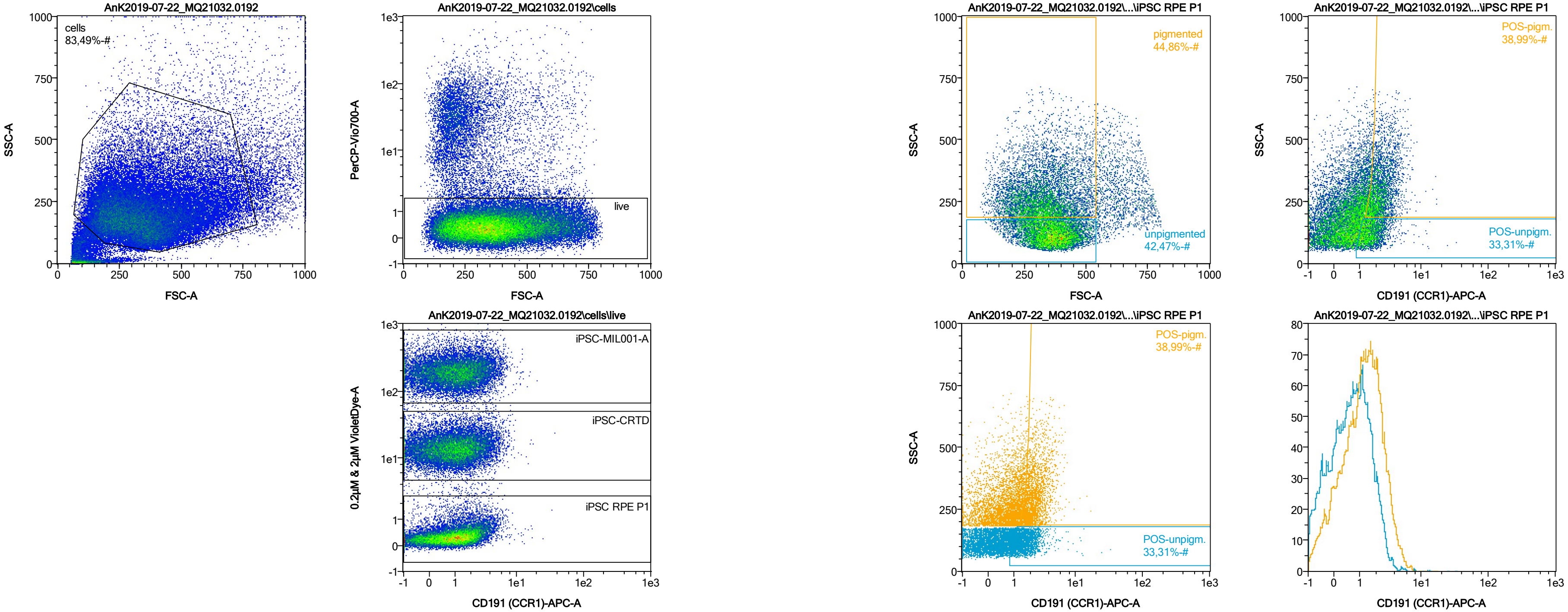

Name	%-#	CD191 (CCR1)-APC-A Mean	CD191 (CCR1)-APC-A Median
POS-unpigm.	33,31	1,76	1,53
POS-pigm.	38,99	2,48	2,21

Date:2024-06-14

Operator: Sebastian Knoebel

Version:2.13.0+54.g95abf0f4

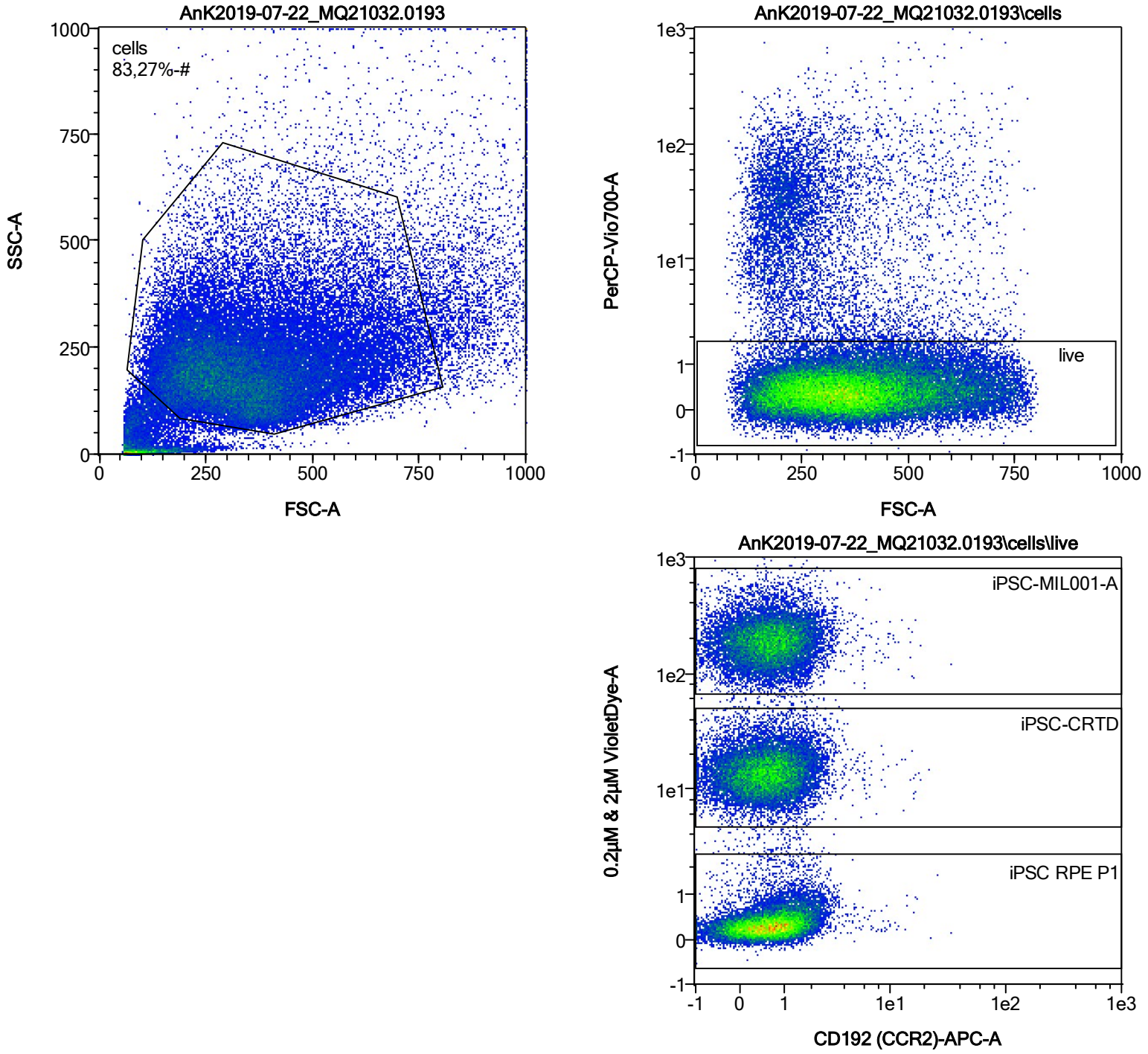

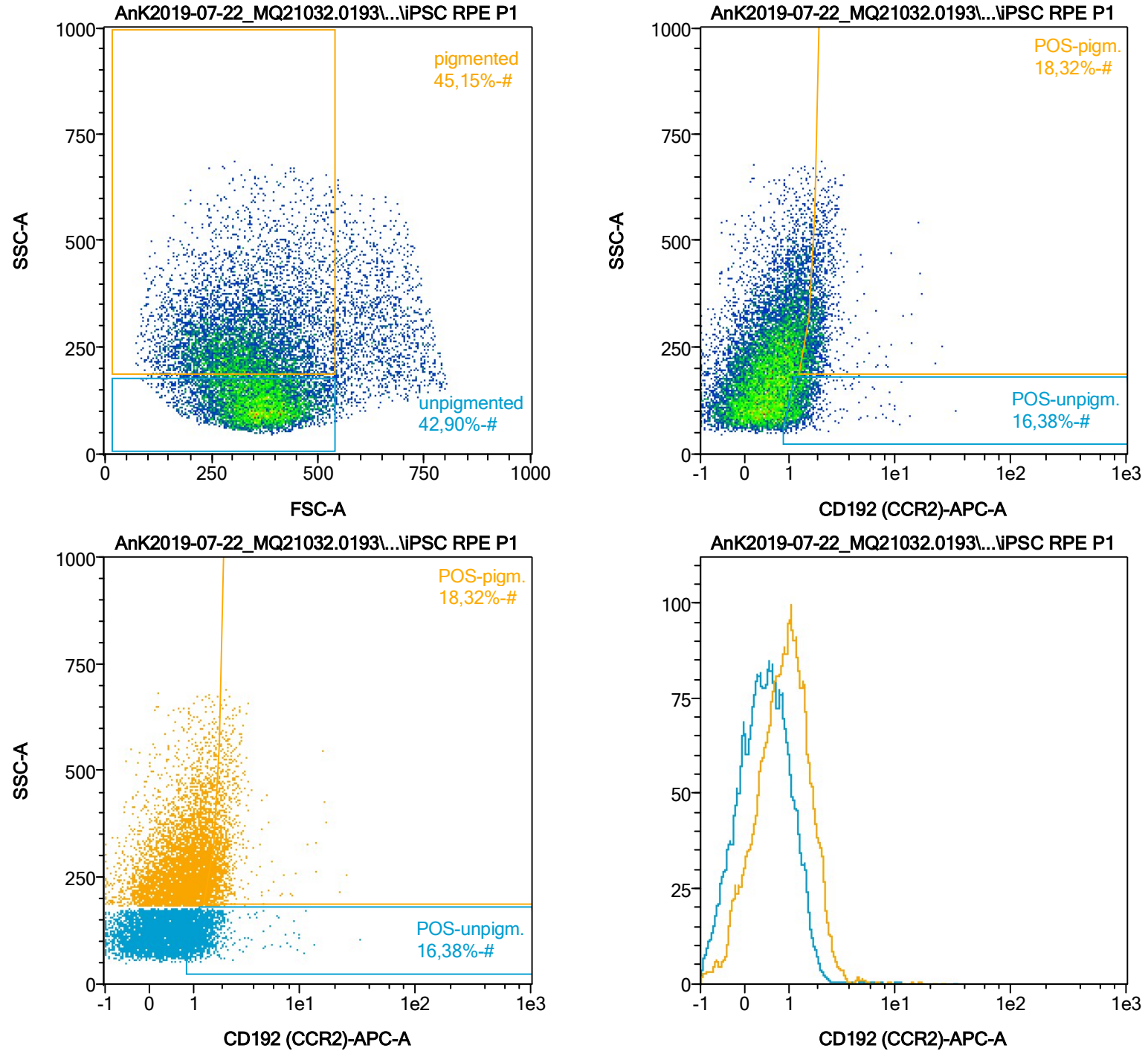

Name	%-#	CD192 (CCR2)-APC-A Mean	CD192 (CCR2)-APC-A Median
POS-unpigm.	16,38	1,54	1,30
POS-pigm.	18,32	2,14	1,91

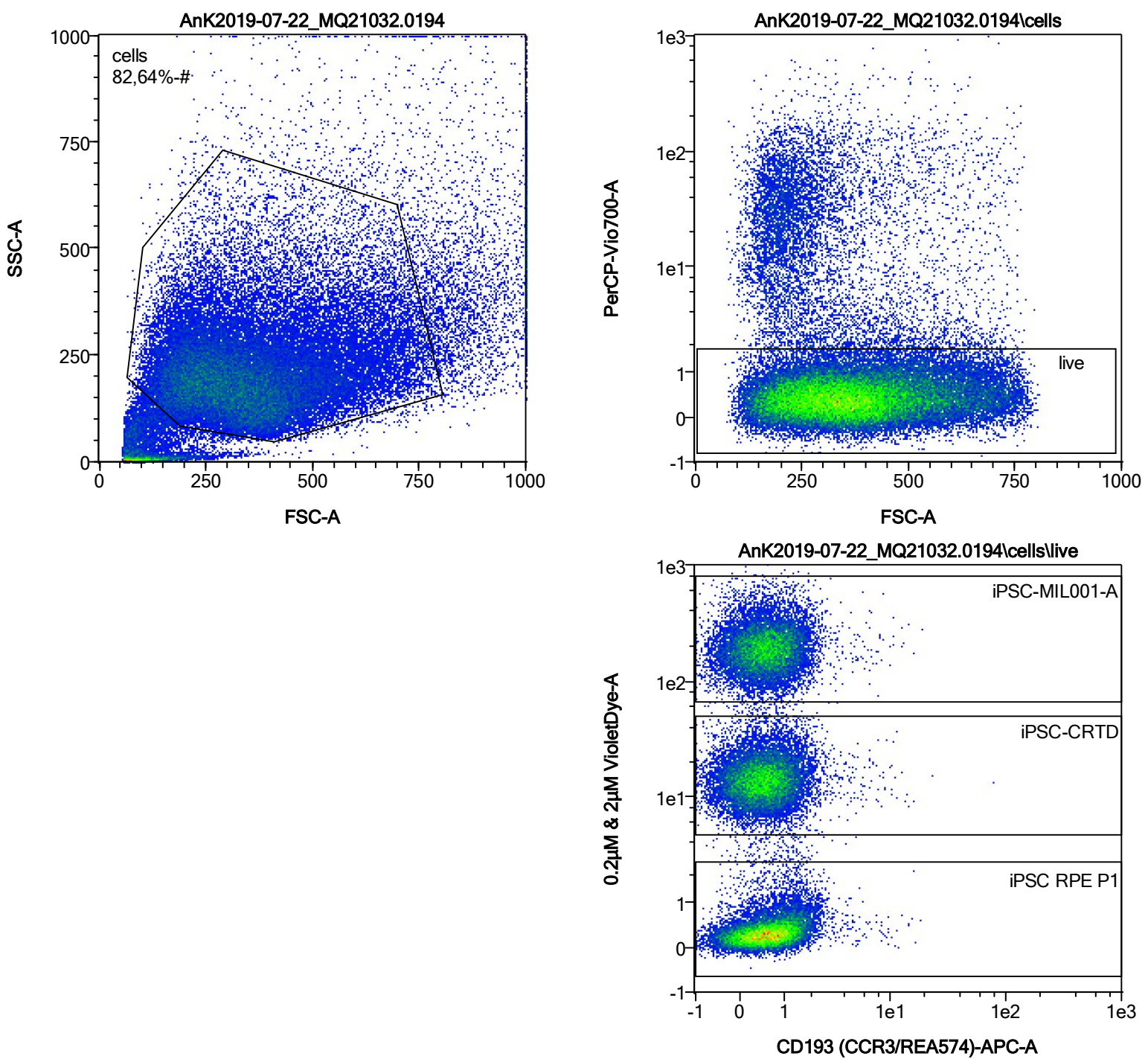

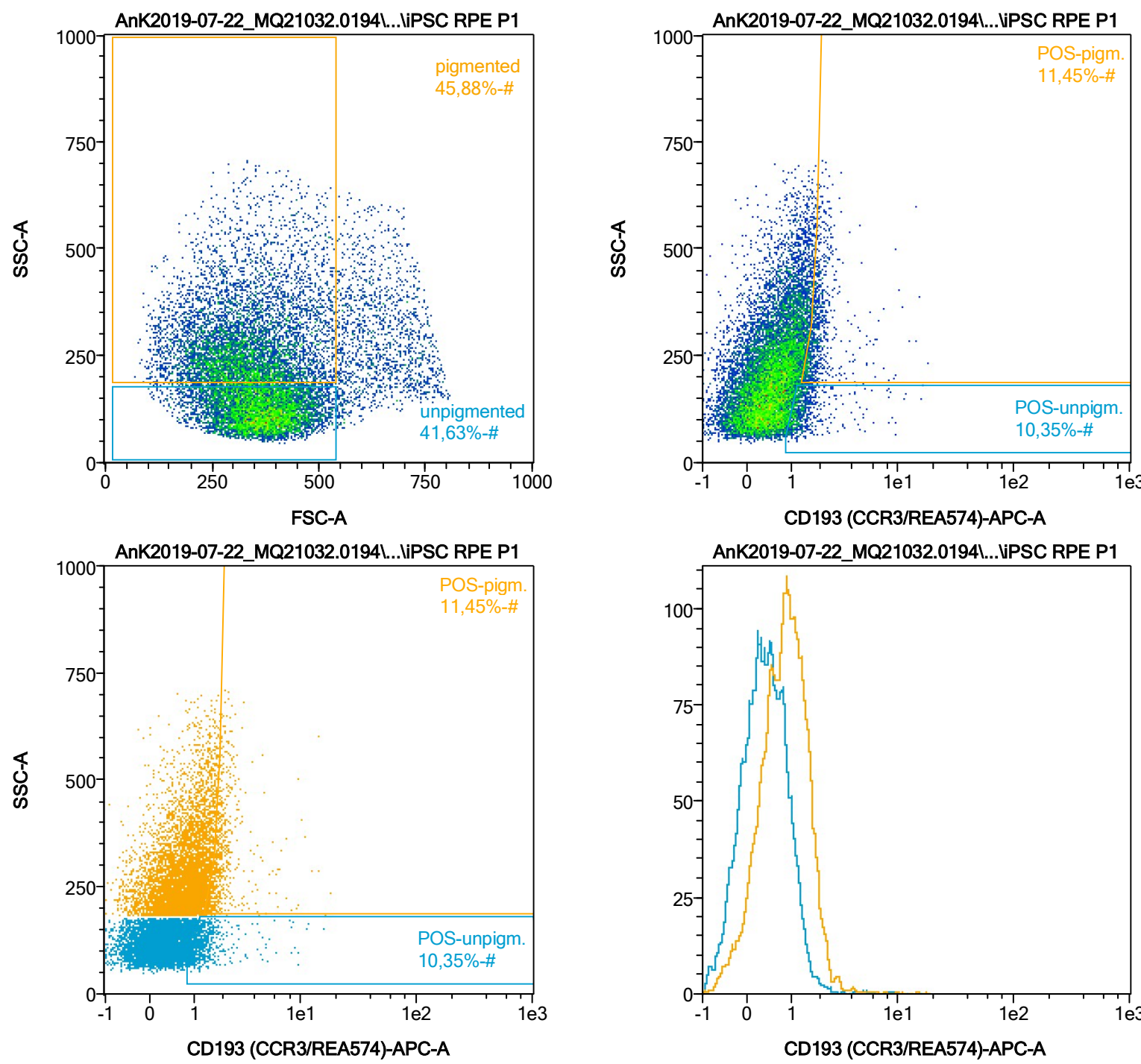

Name	%-#	CD193 (CCR3/REA574)-APC-A Mean	CD193 (CCR3/REA574)-APC-A Median
POS-unpigm.	10,35	1,53	1,23
POS-pigm.	11,45	2,15	1,85

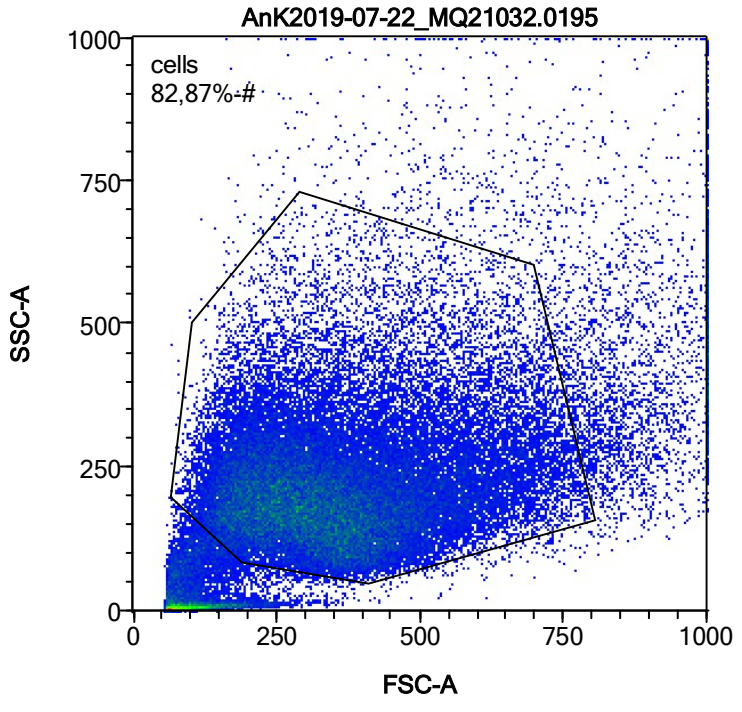

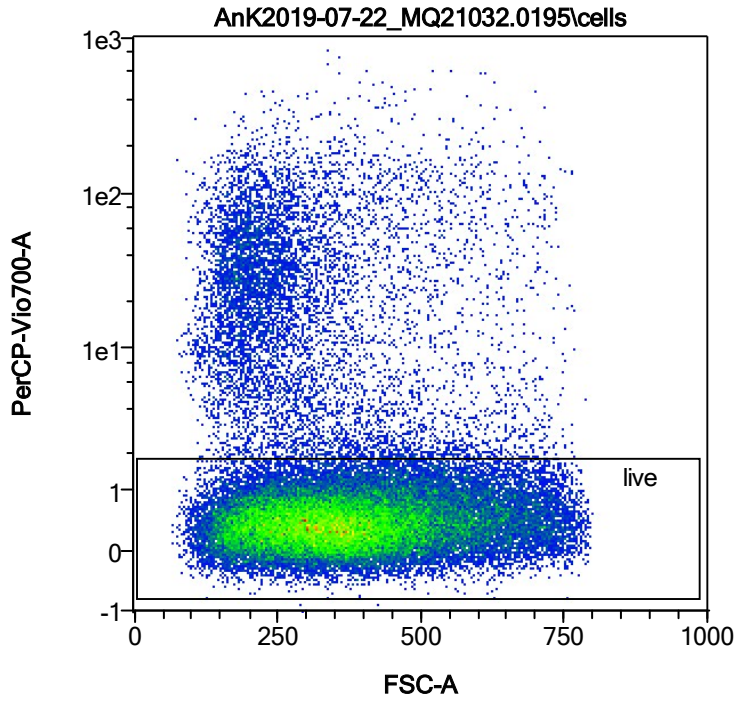

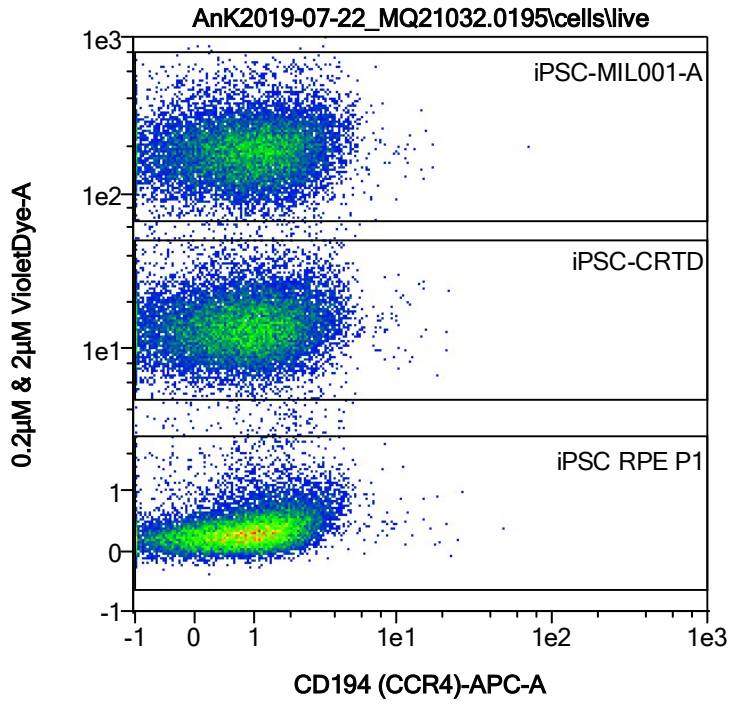

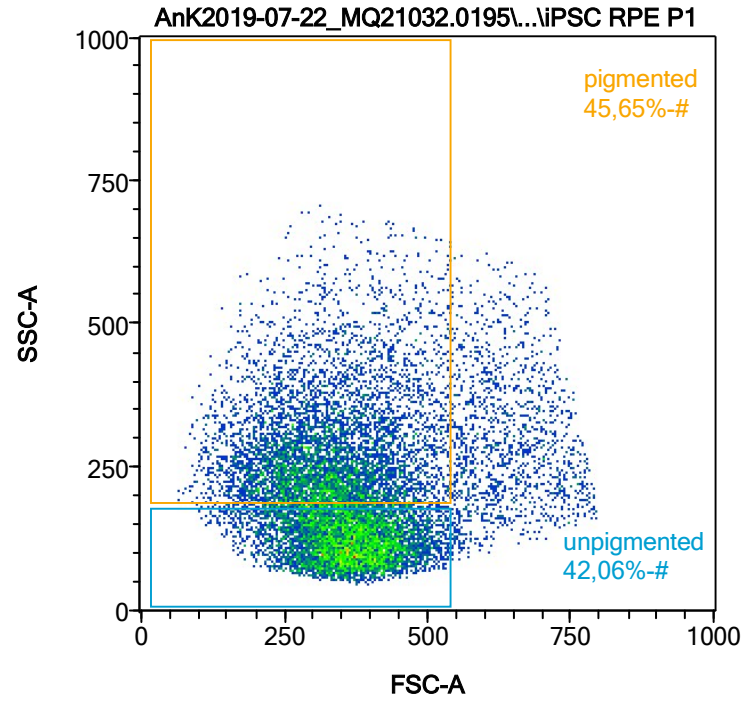

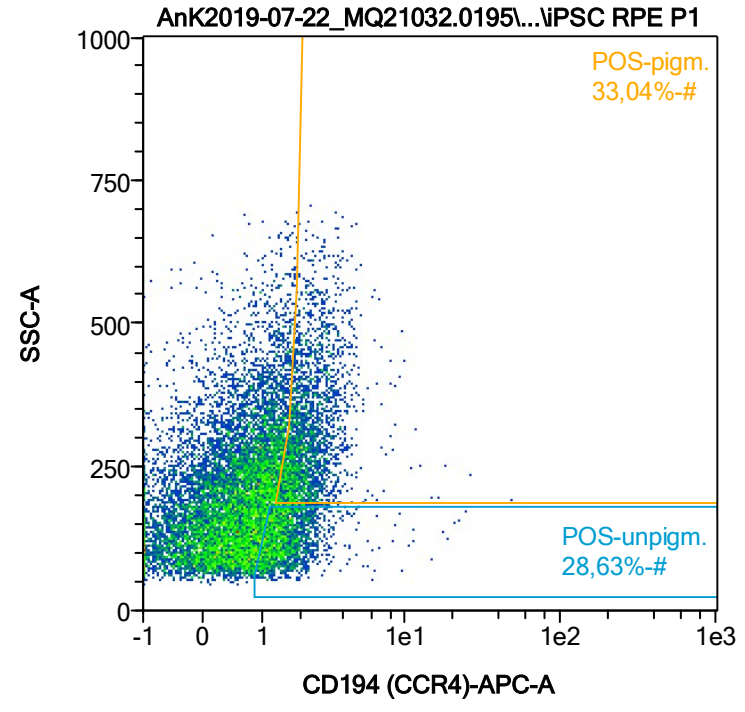

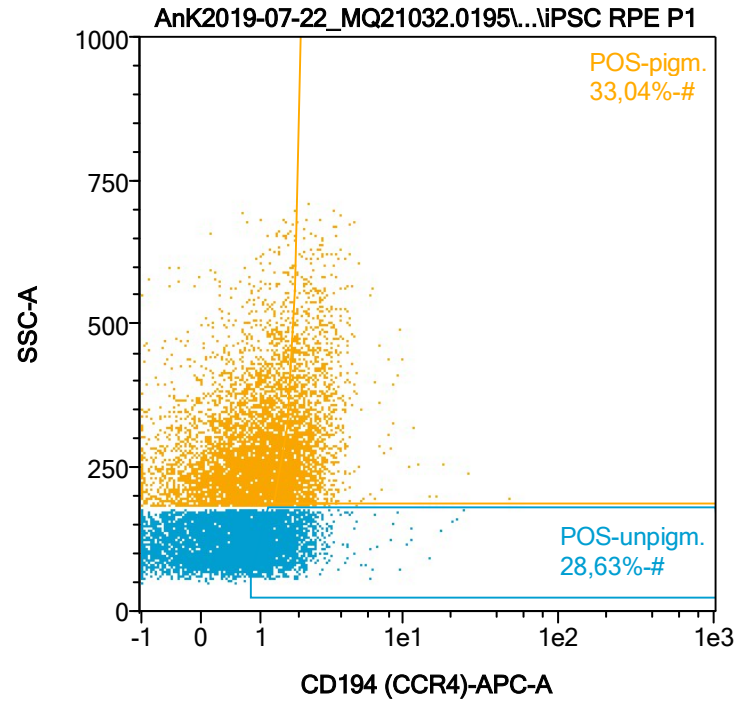

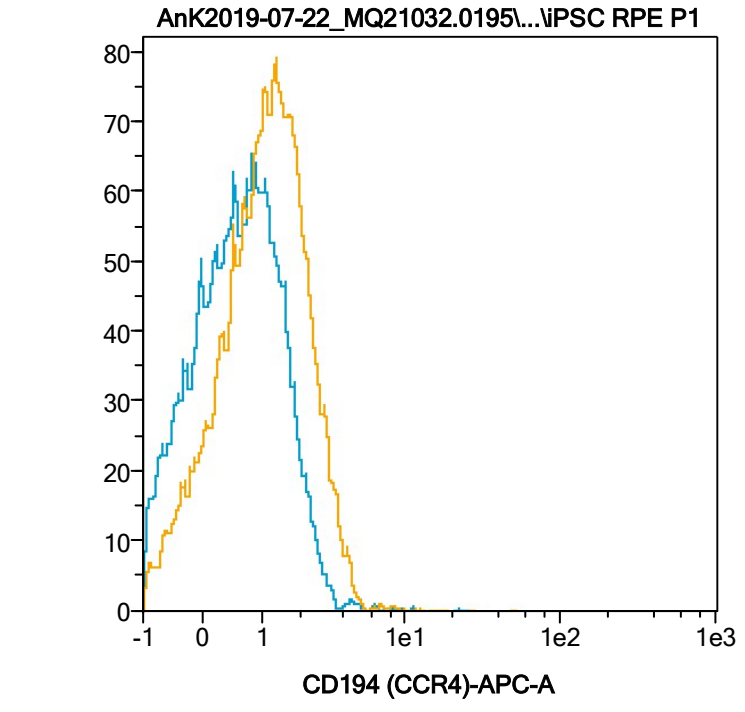

Name	%-#	CD194 (CCR4)-APC-A Mean	CD194 (CCR4)-APC-A Median
POS-unpigm.	28,63	1,69	1,46
POS-pigm.	33,04	2,39	2,12

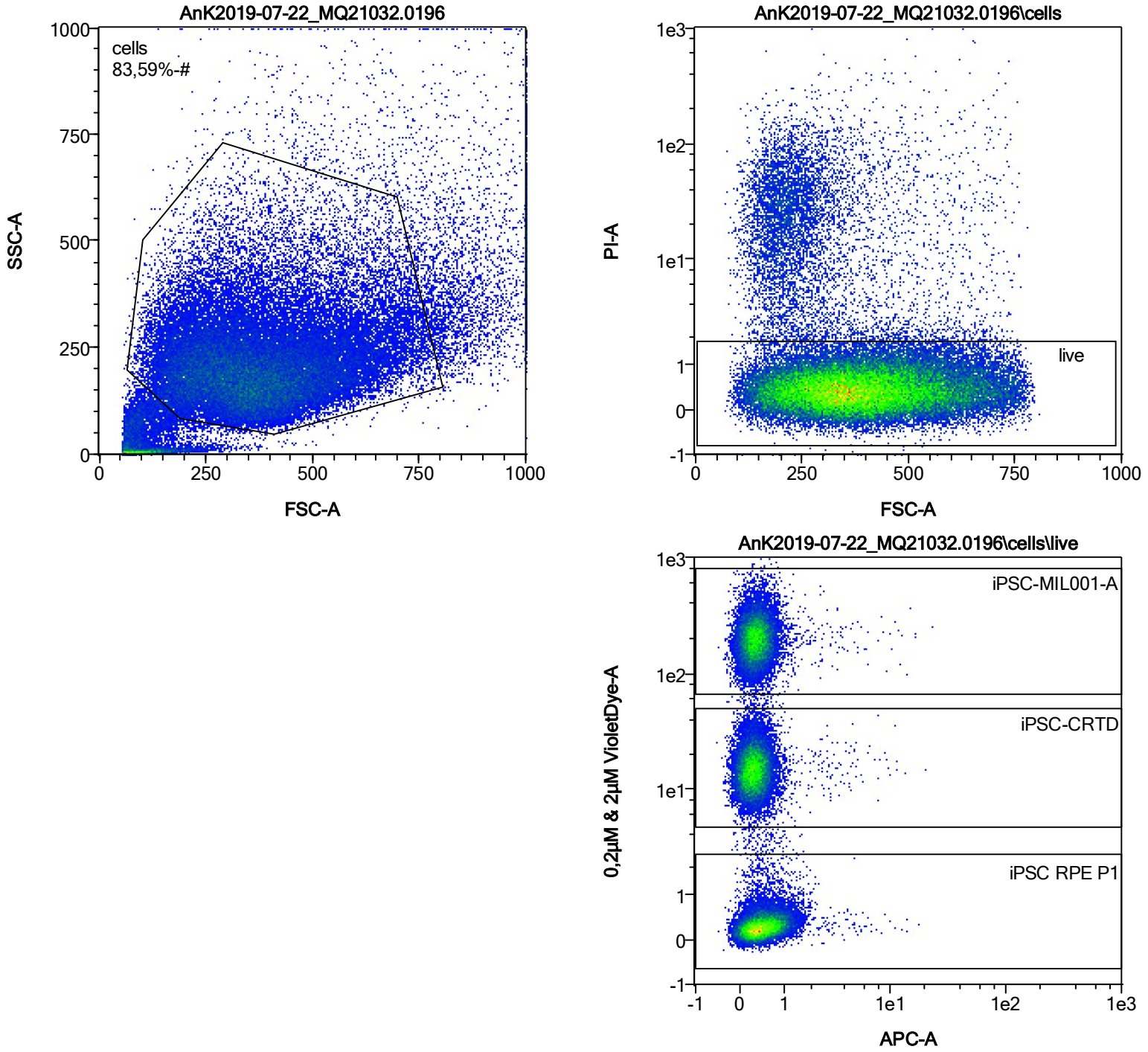

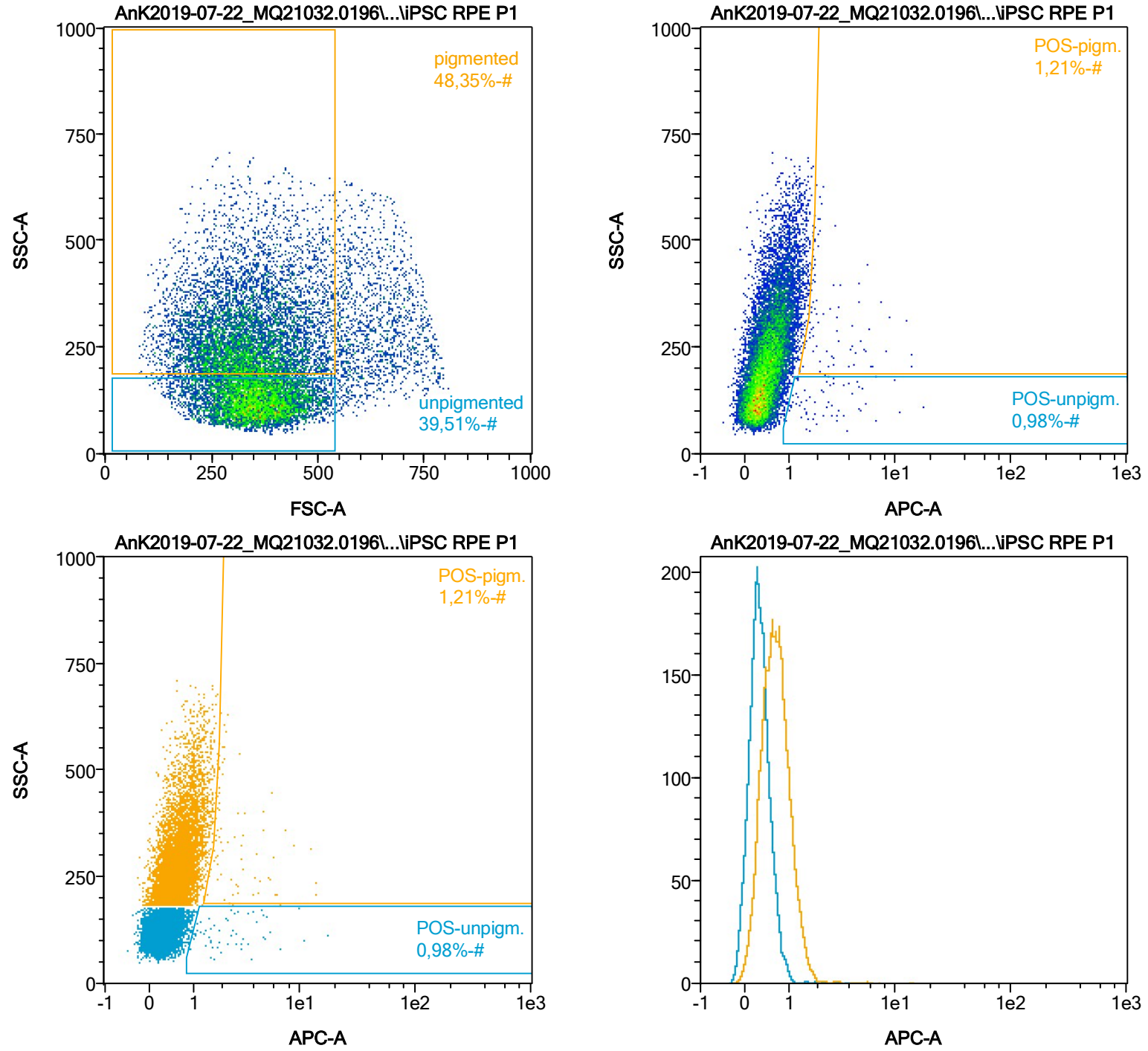

Name	%-#	APC-A Mean	APC-A Median
POS-unpigm.	0,98	2,95	1,98
POS-pigm.	1,21	3,12	2,06

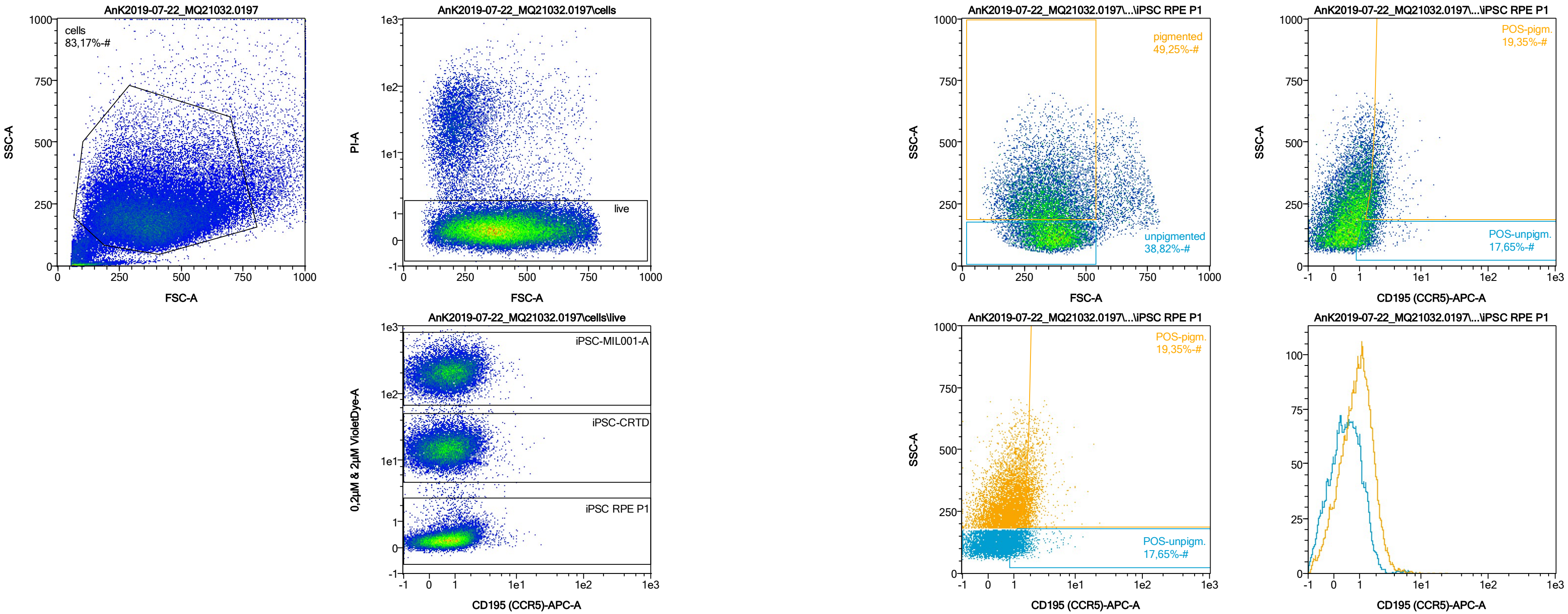

Name	%-#	CD195 (CCR5)-APC-A Mean	CD195 (CCR5)-APC-A Median
POS-unpigm.	17,65	1,56	1,35
POS-pigm.	19,35	2,25	1,97

Date:2024-06-14

Operator: Sebastian Knoebel

Version:2.13.0+54.g95abf0f4

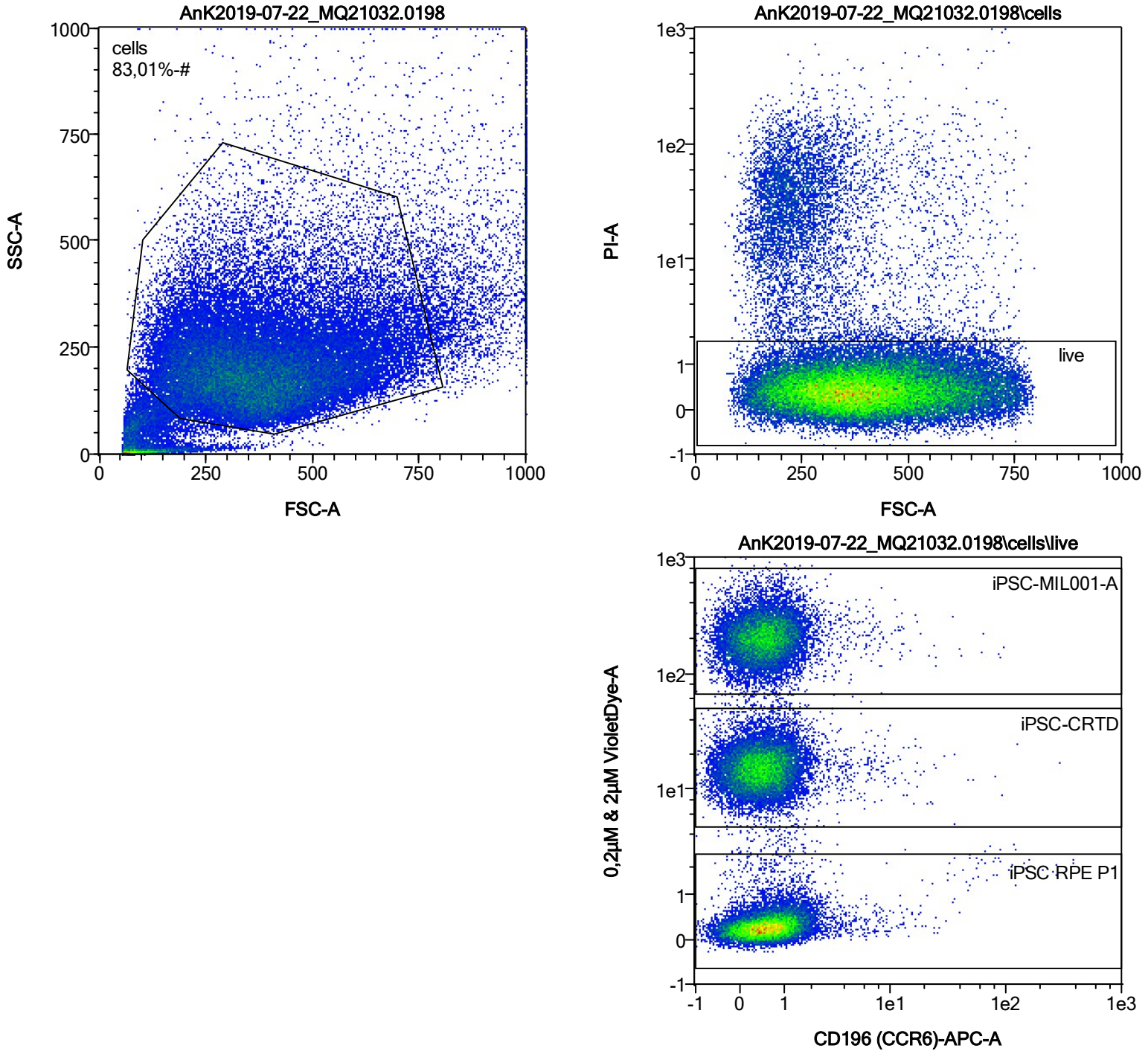

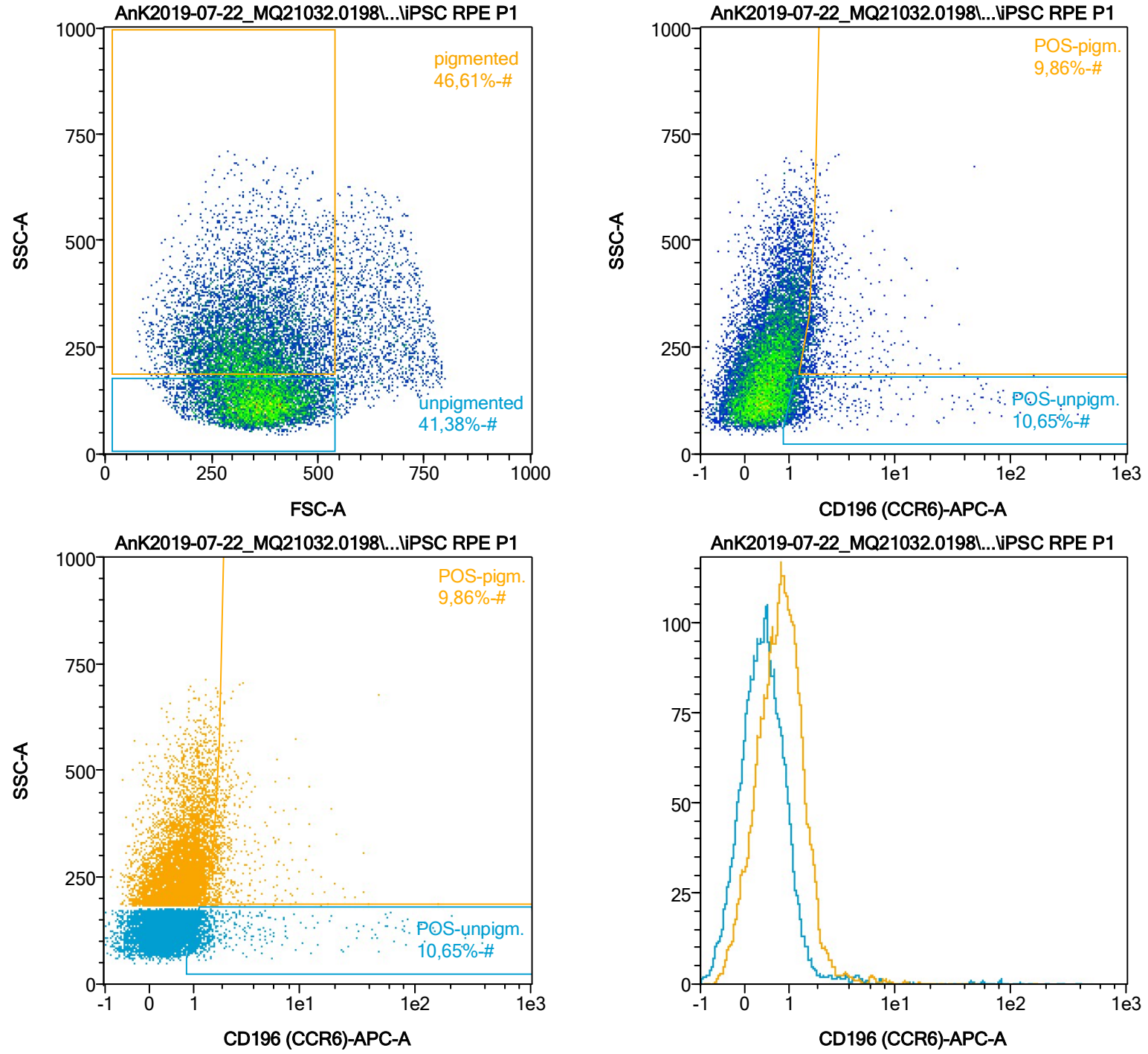

Name	%-#	CD196 (CCR6)-APC-A Mean	CD196 (CCR6)-APC-A Median
POS-unpigm.	10,65	7,95	1,30
POS-pigm.	9,86	3,17	1,94

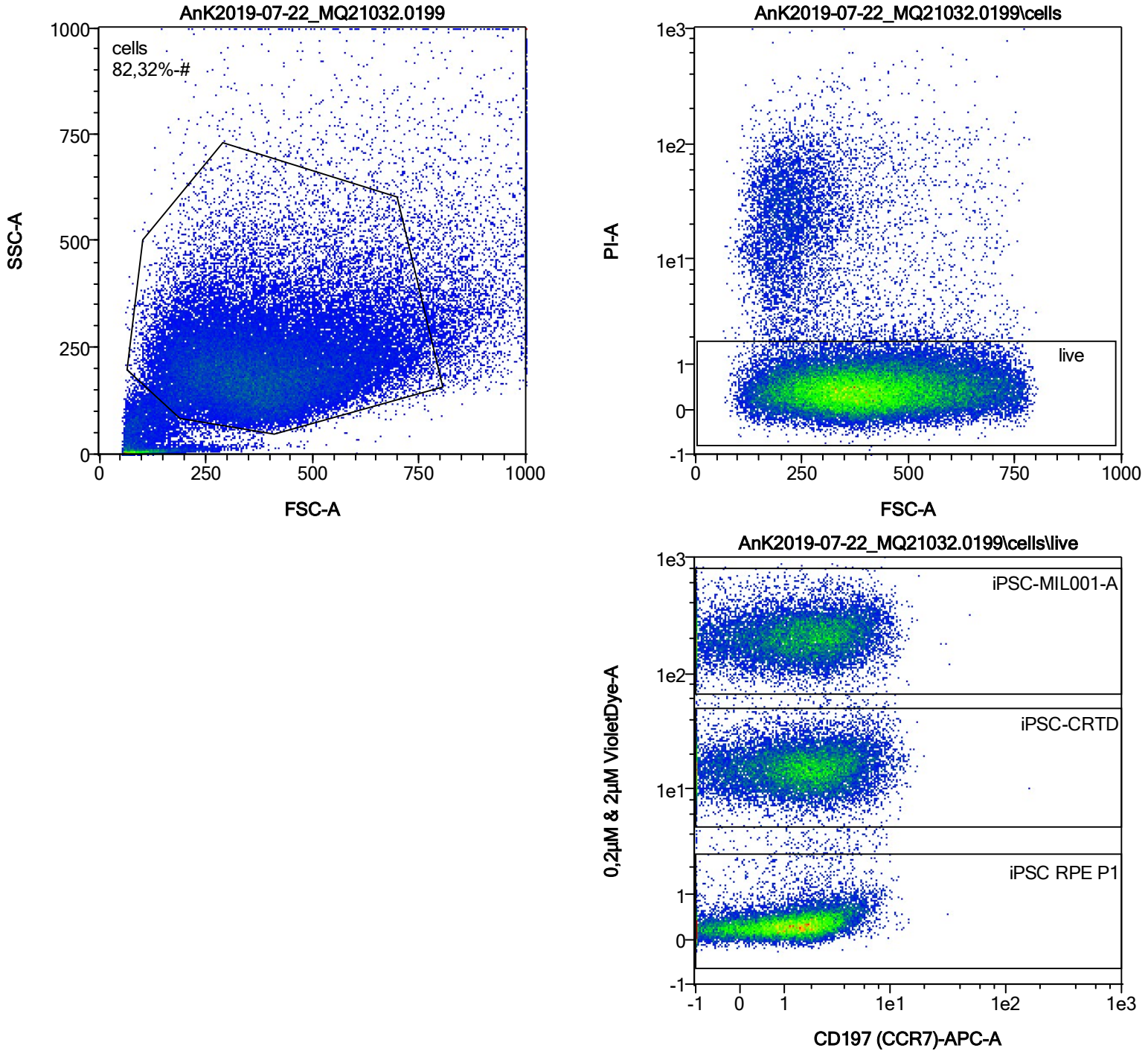

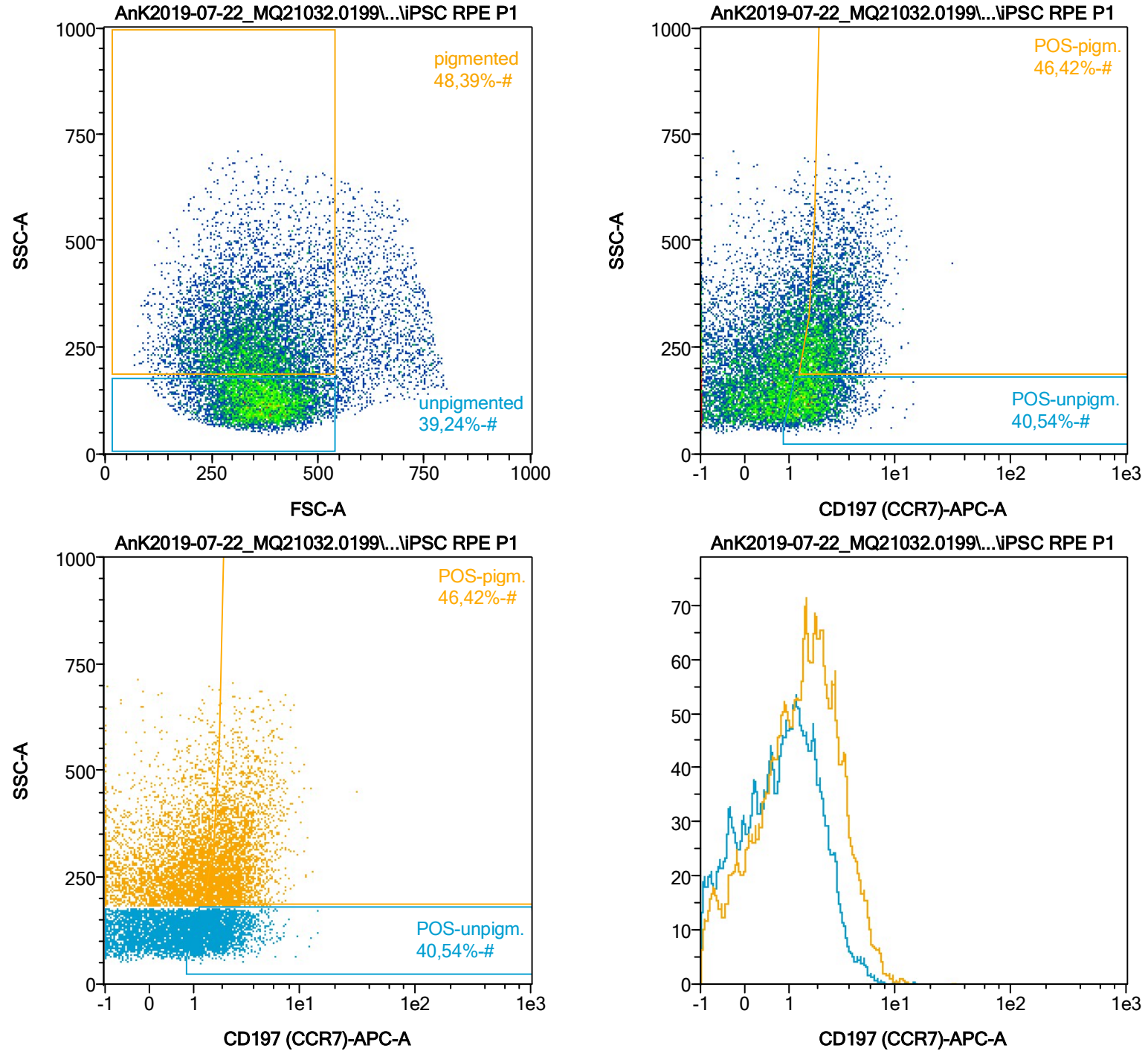

Name	%-#	CD197 (CCR7)-APC-A Mean	CD197 (CCR7)-APC-A Median
POS-unpigm.	40,54	2,03	1,78
POS-pigm.	46,42	2,93	2,55

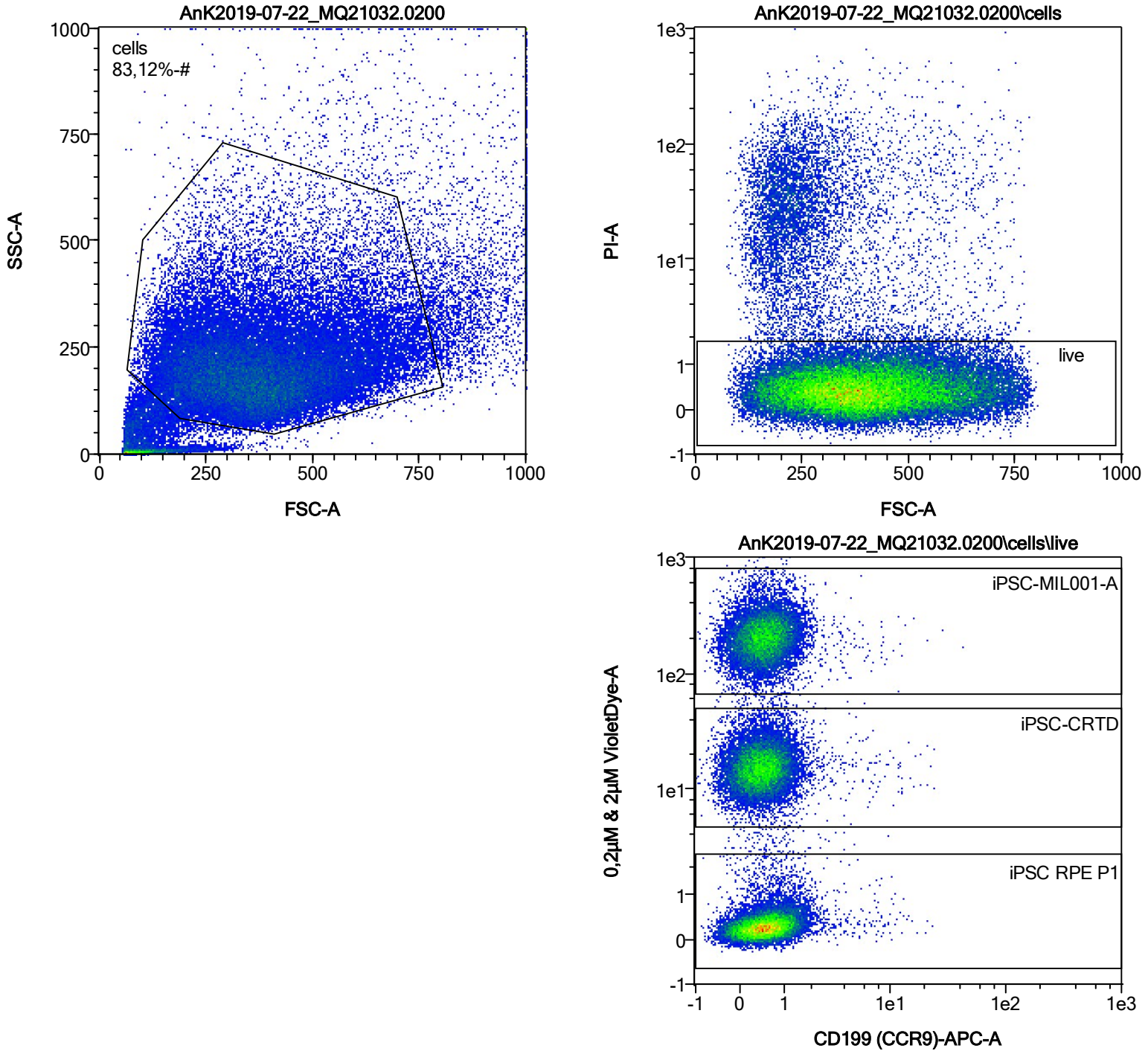

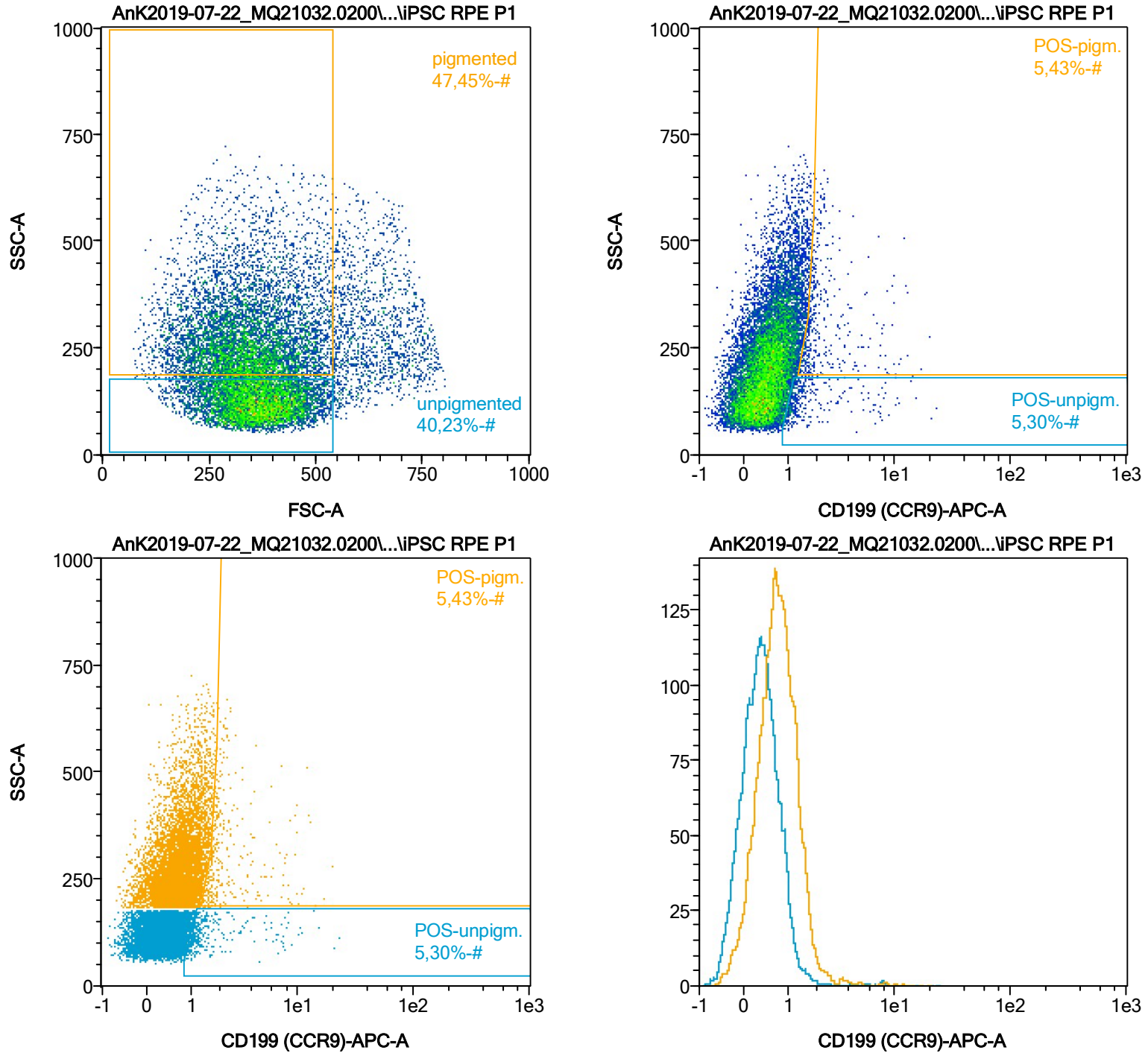

Name	%-#	CD199 (CCR9)-APC-A Mean	CD199 (CCR9)-APC-A Median
POS-unpigm.	5,30	2,09	1,22
POS-pigm.	5,43	2,72	1,91

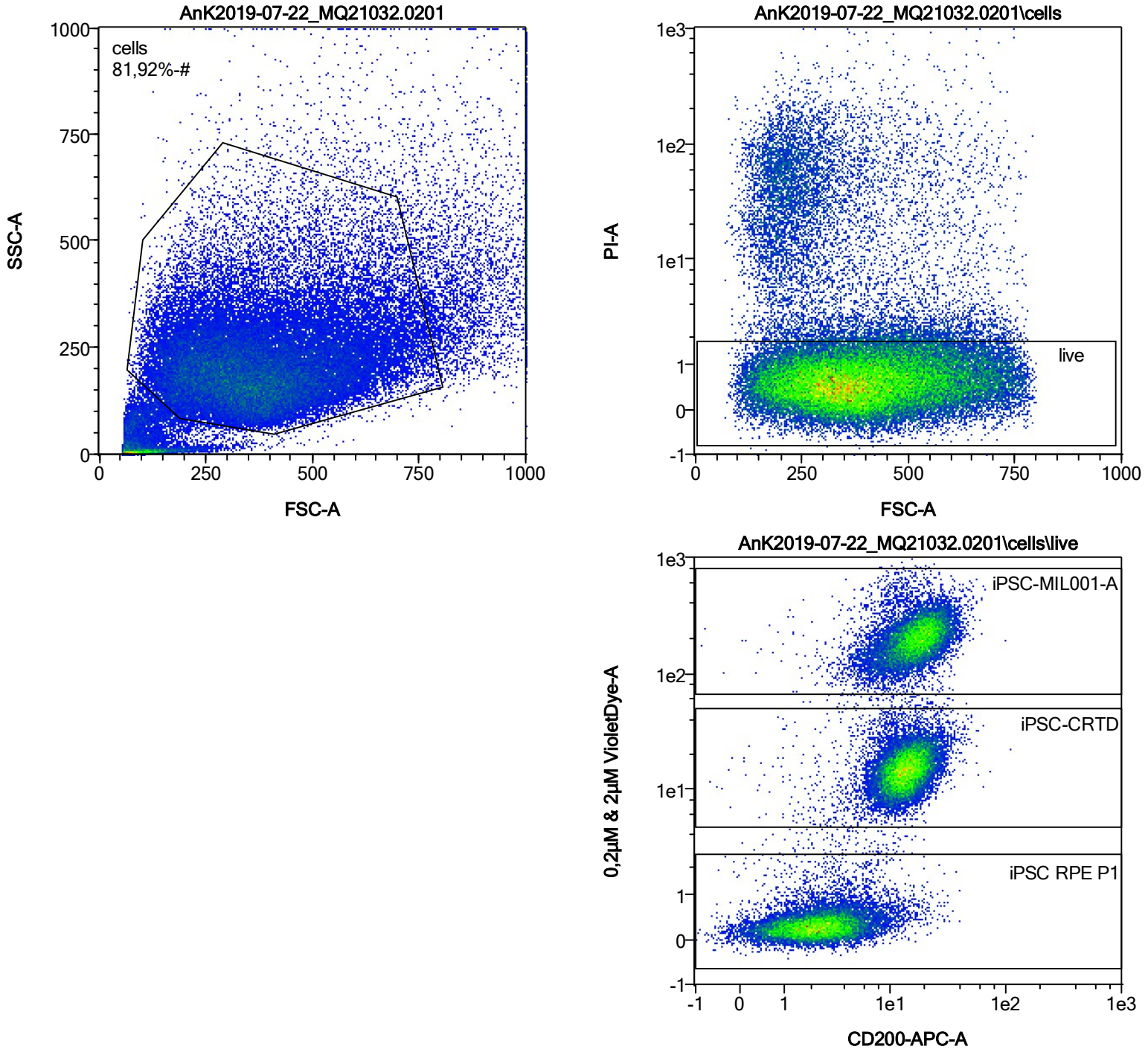

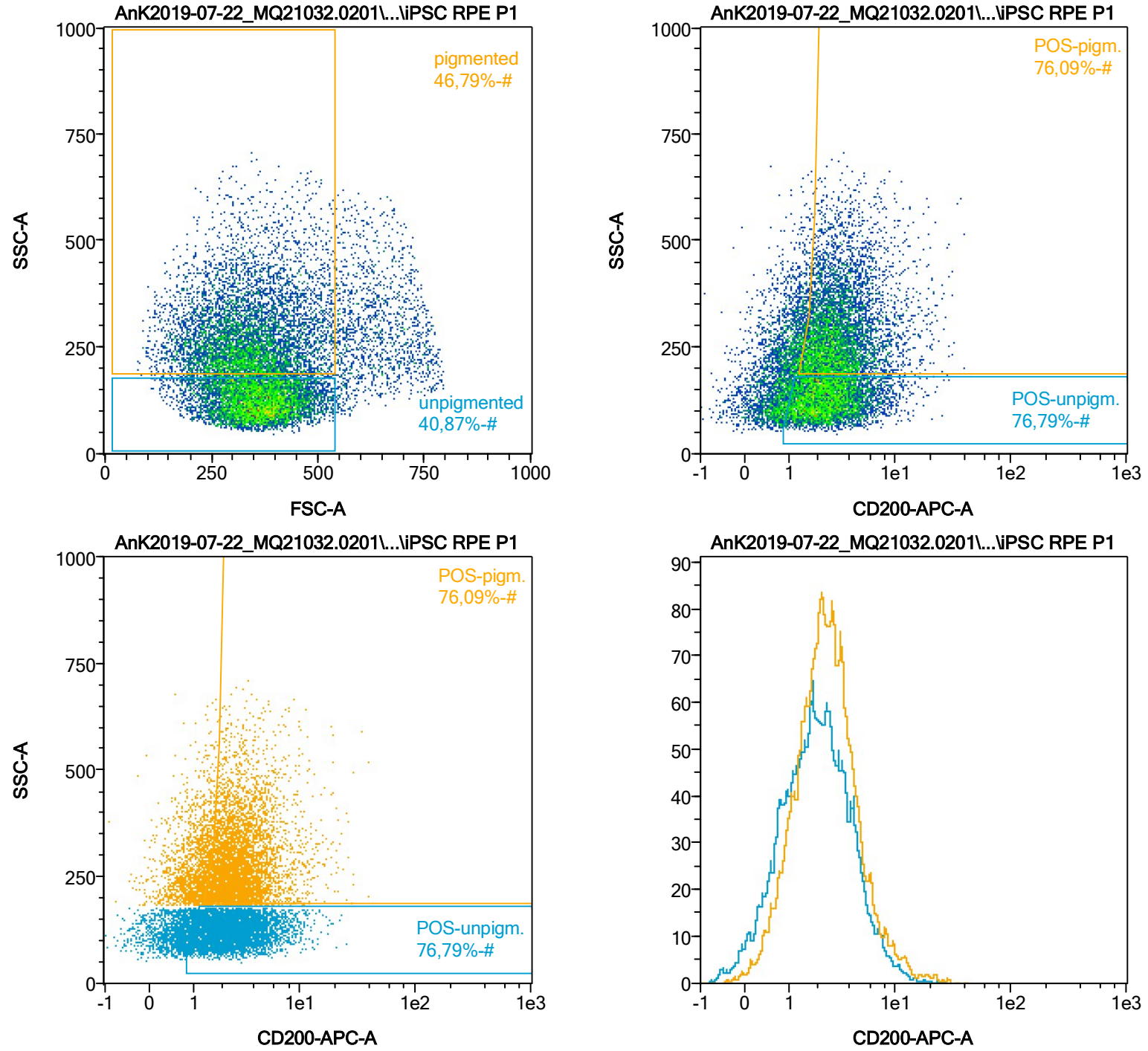

Name	%-#	CD200-APC-A Mean	CD200-APC-A Median
POS-unpigm.	76,79	2,97	2,41
POS-pigm.	76,09	3,57	2,84

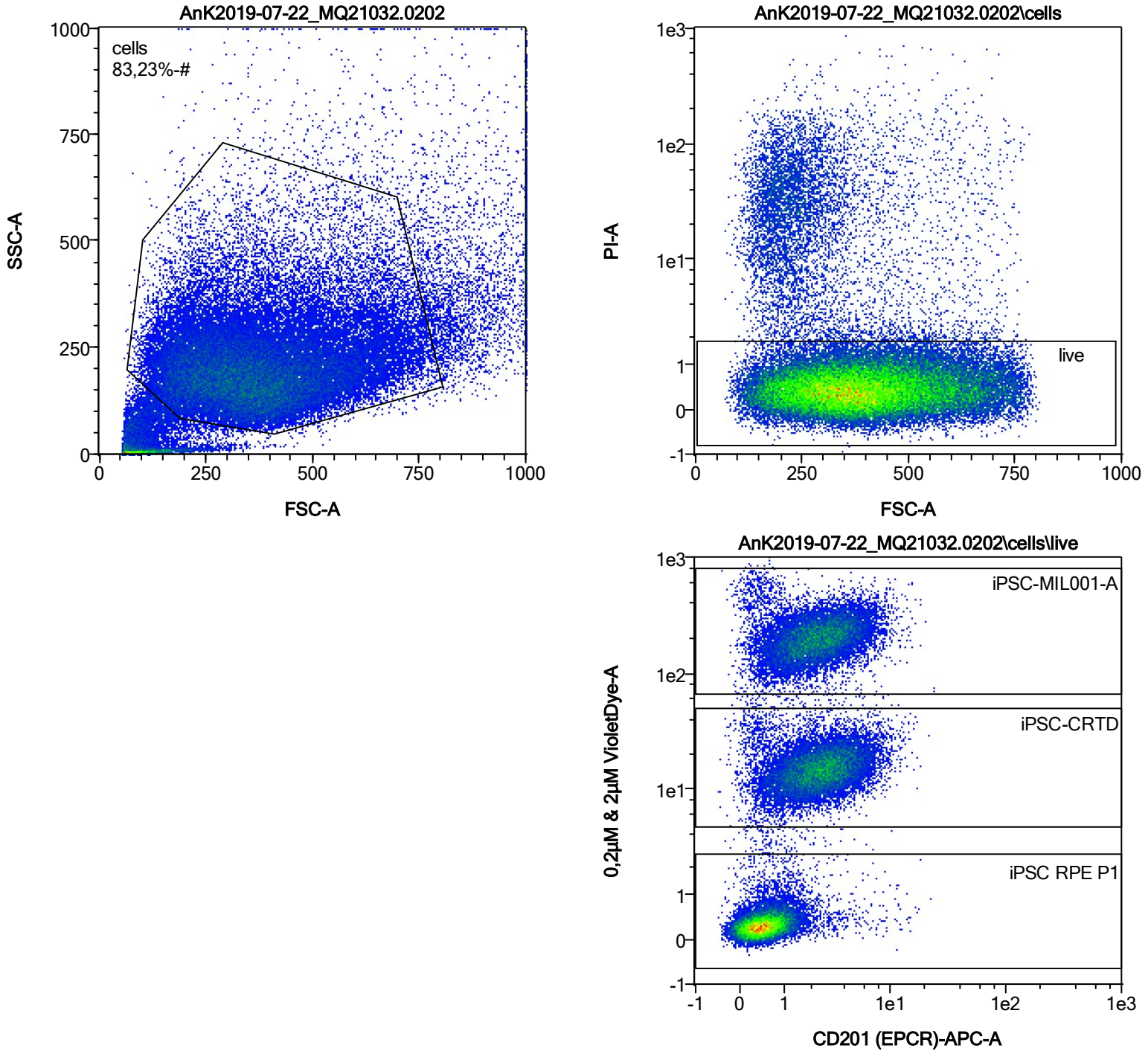

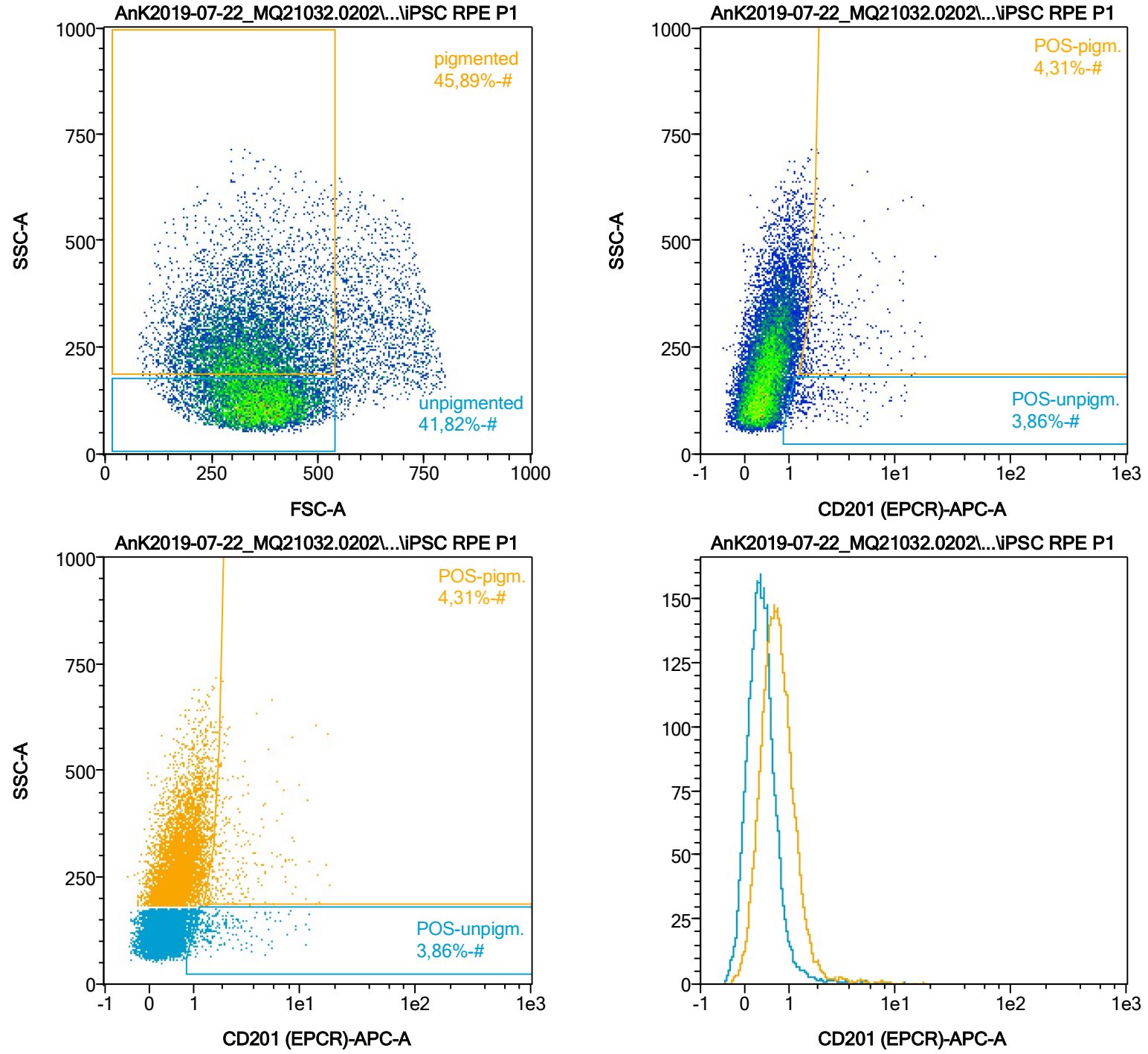

Name	%-#	CD201 (EPCR)-APC-A Mean	CD201 (EPCR)-APC-A Median
POS-unpigm.	3,86	2,24	1,52
POS-pigm.	4,31	3,18	2,12

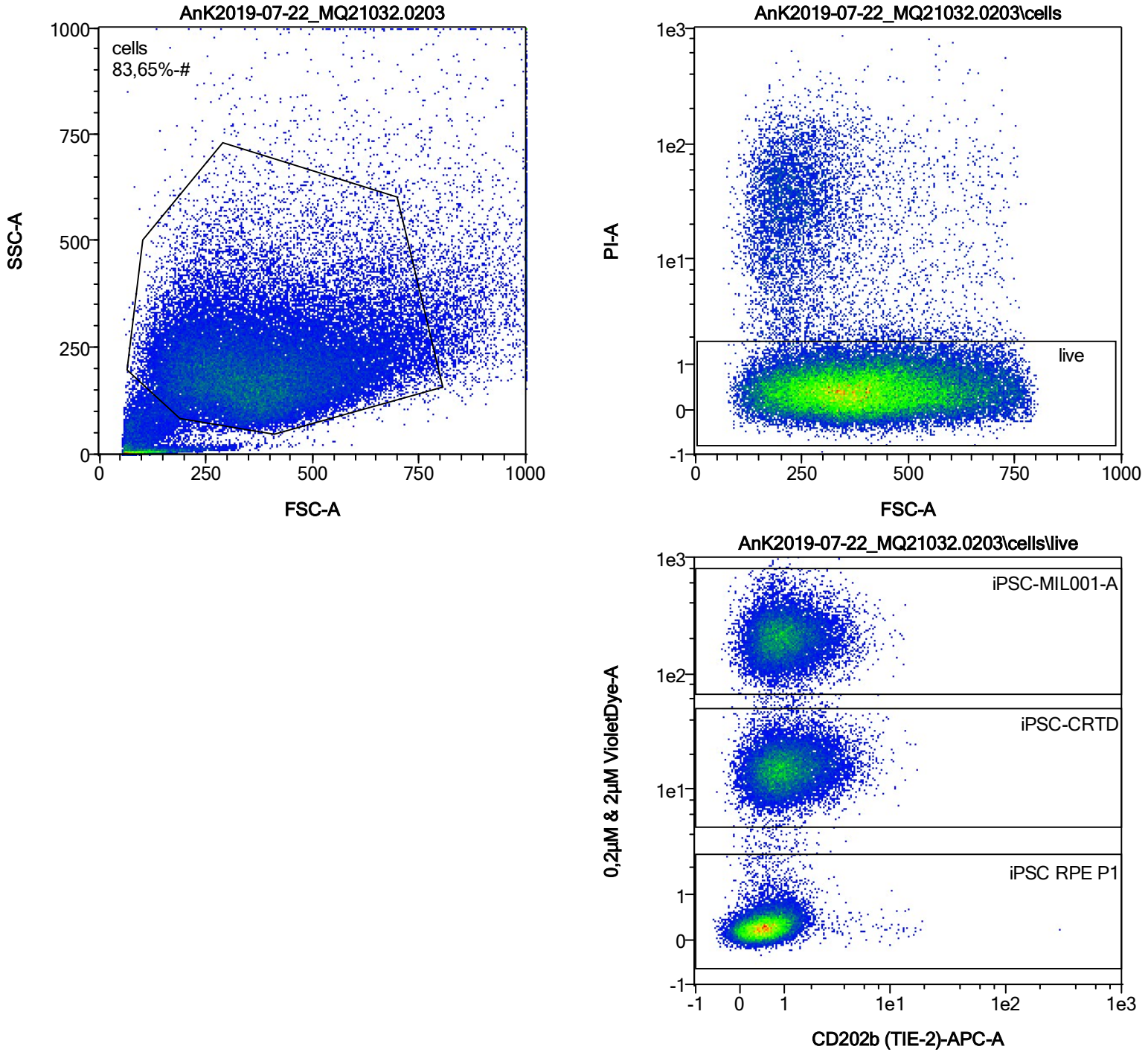

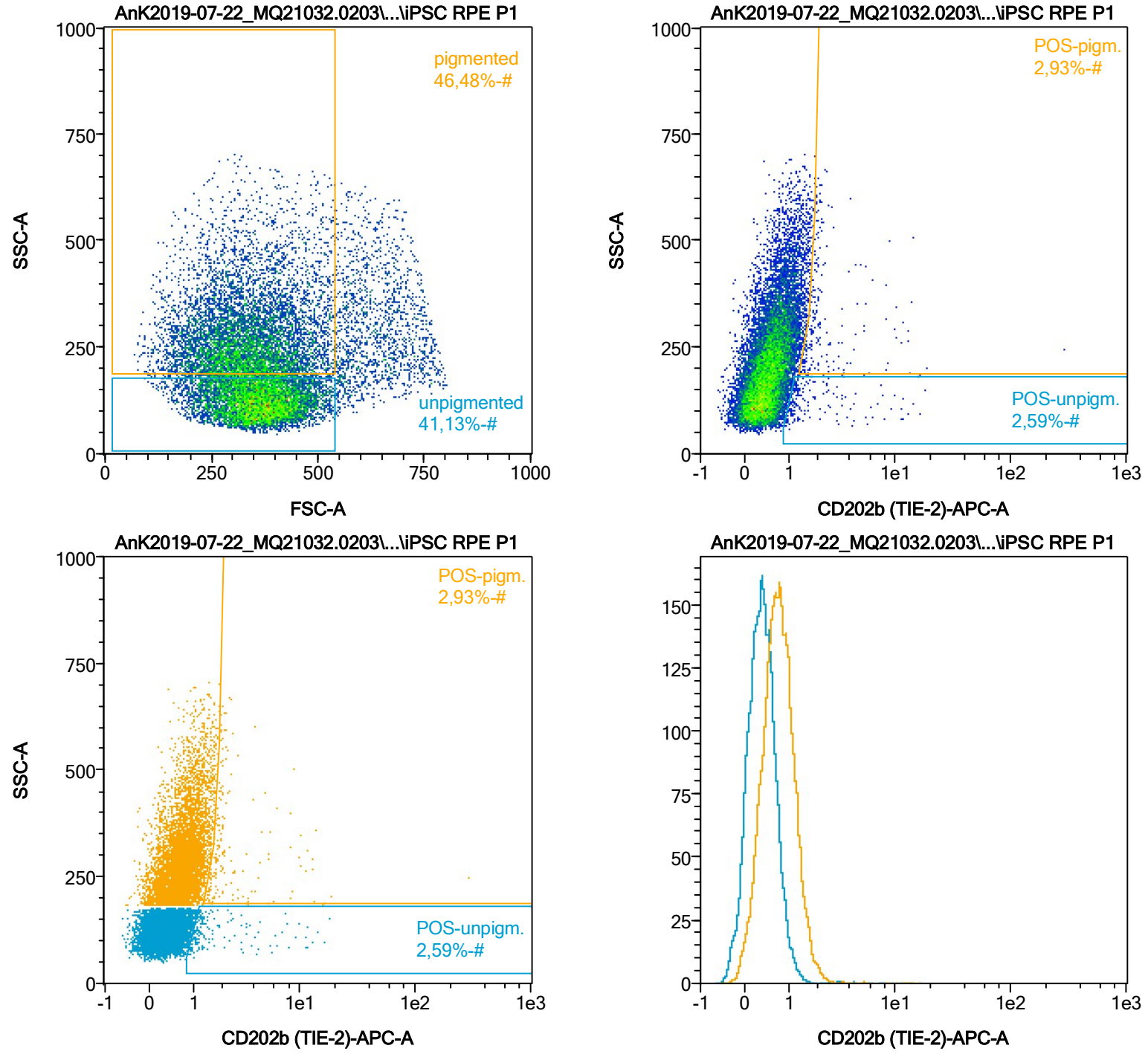

Name	%-#	CD202b (TIE-2)-APC-A Mean	CD202b (TIE-2)-APC-A Median
POS-unpigm.	2,59	2,49	1,26
POS-pigm.	2,93	4,32	1,94

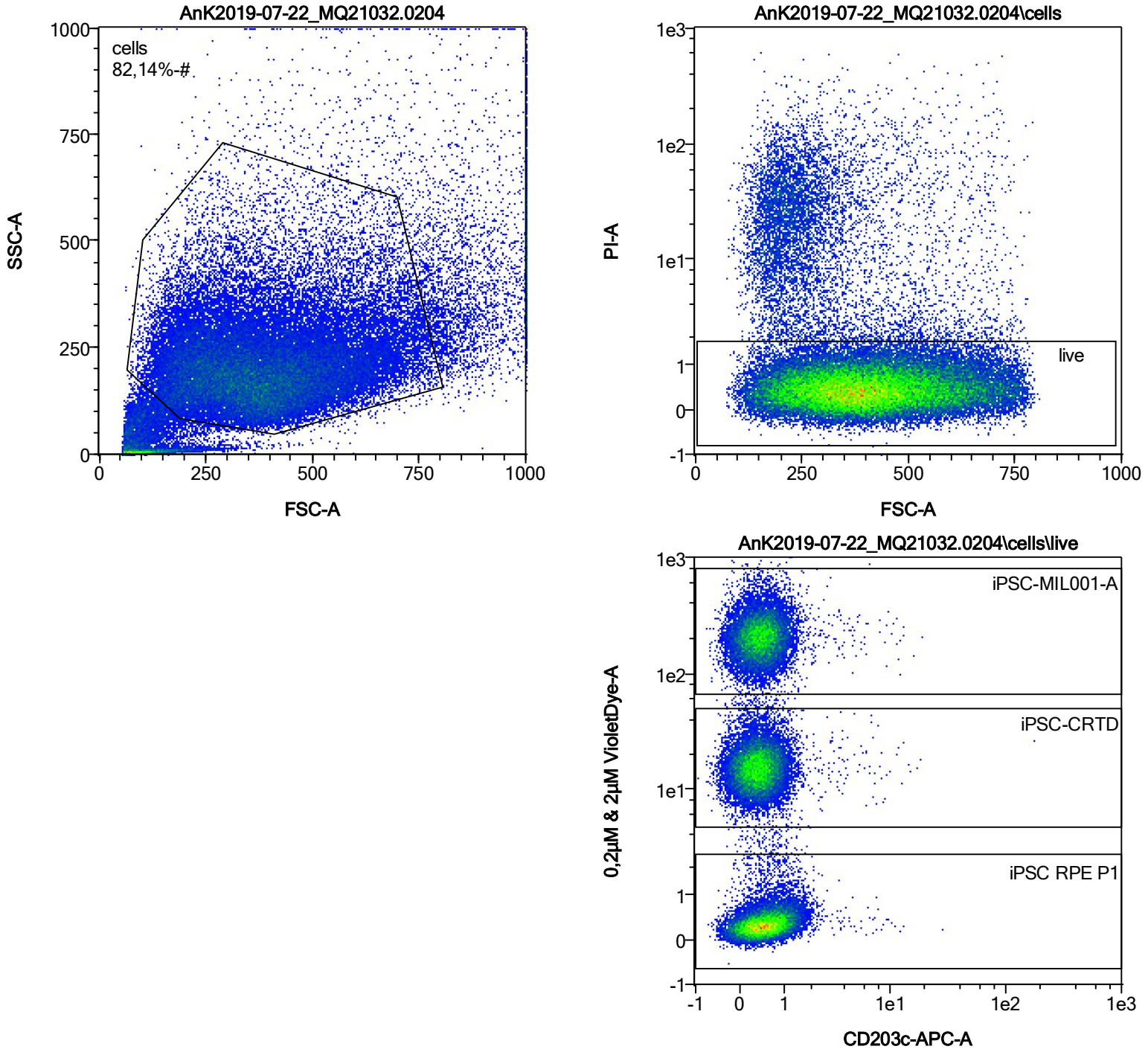

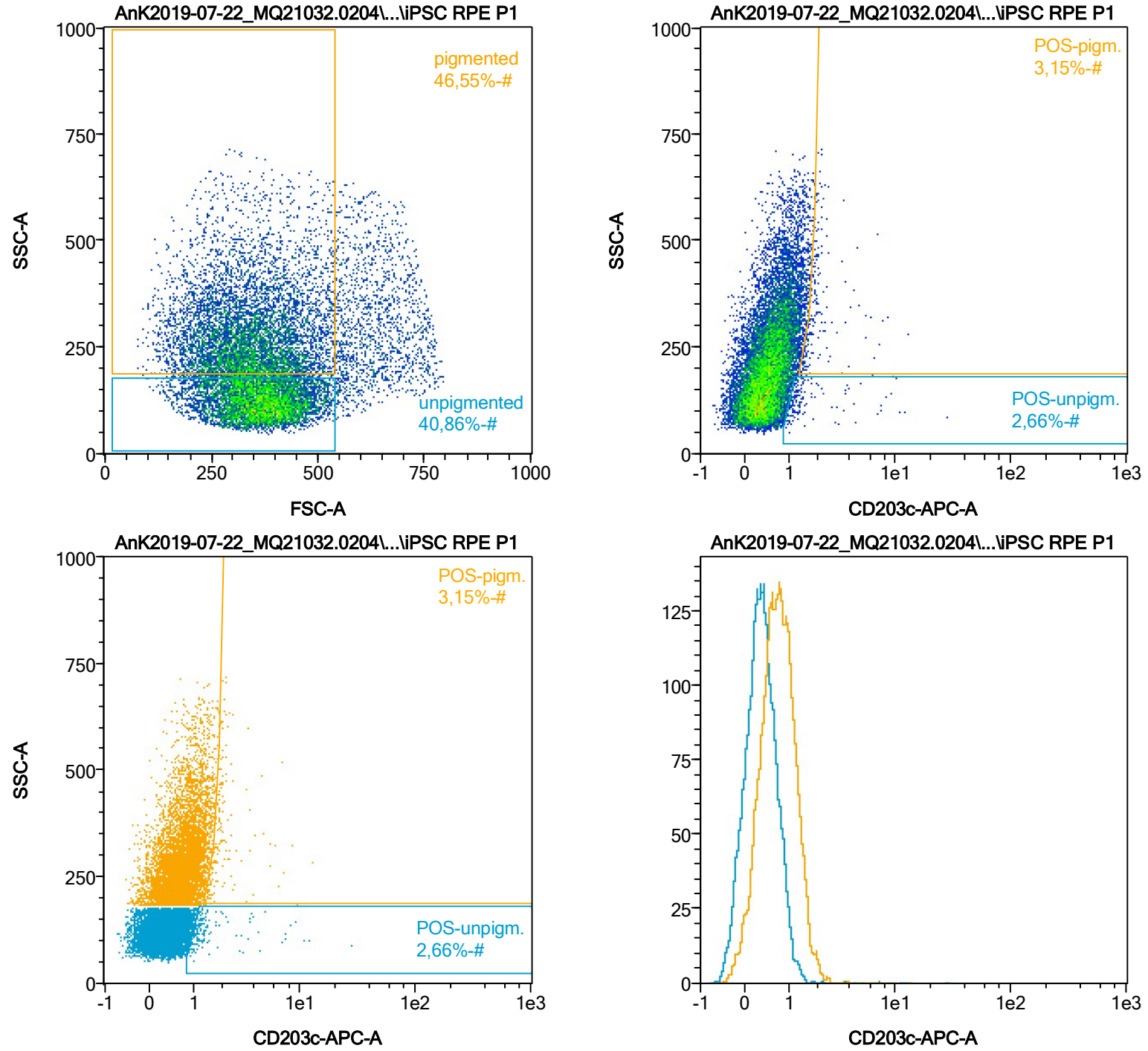

Name	%-#	CD203c-APC-A Mean	CD203c-APC-A Median
POS-unpigm.	2,66	1,95	1,21
POS-pigm.	3,15	2,17	1,86

Date:2024-06-14
Operator: Sebastian Knoebel
Version:2.13.0+54.g95abf0f4

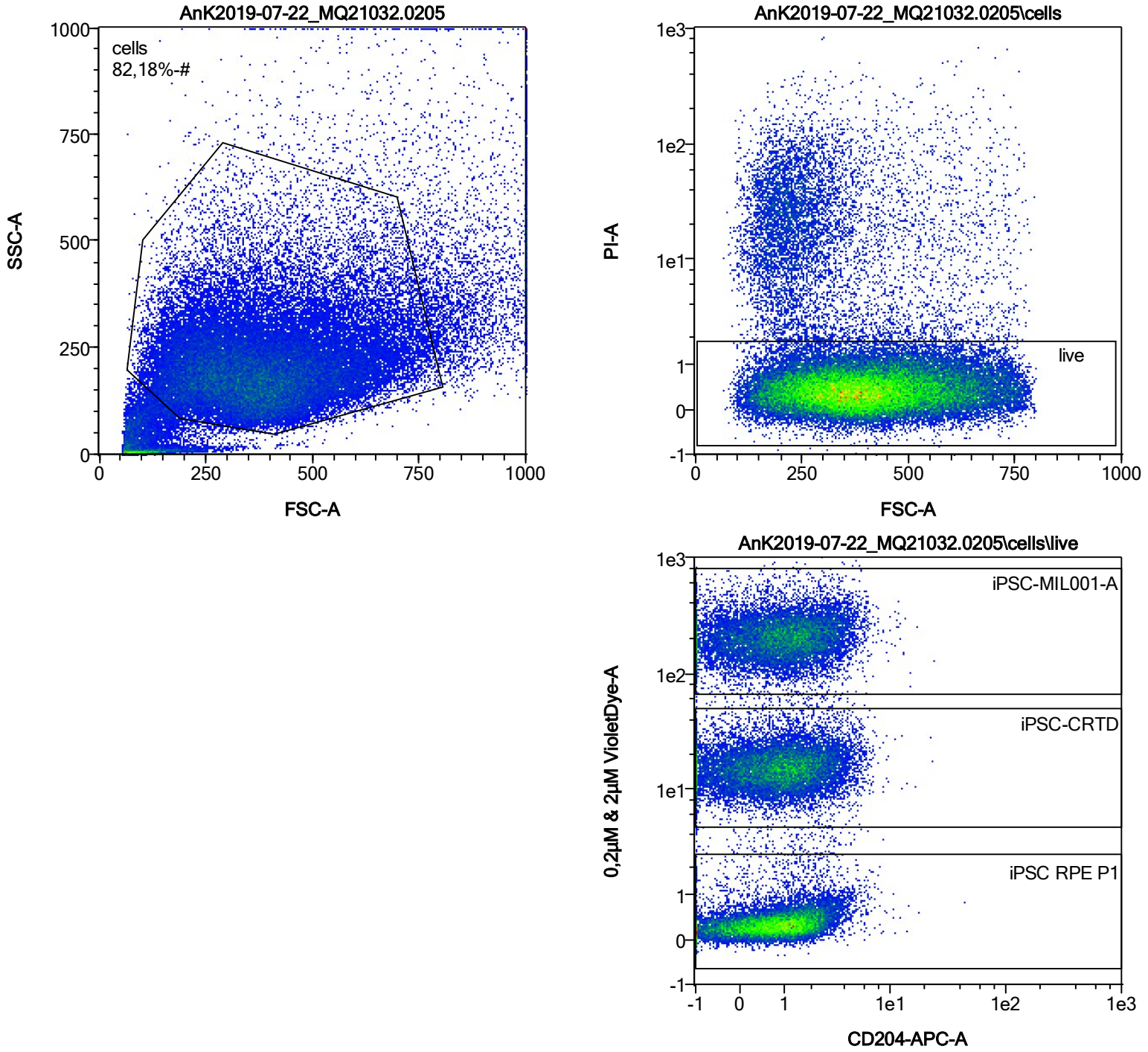

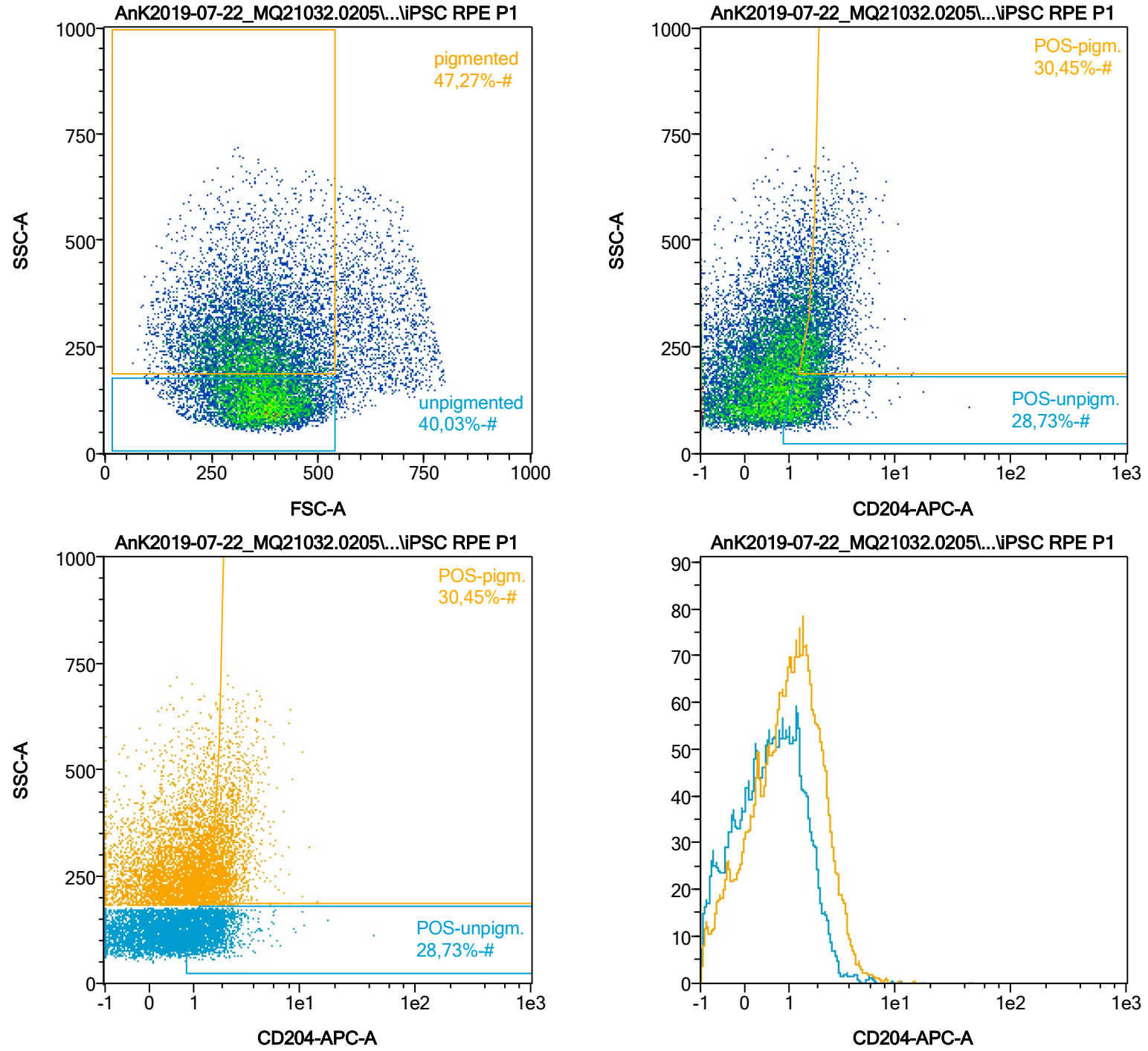

Name	%-#	CD204-APC-A Mean	CD204-APC-A Median
POS-unpigm.	28,73	1,71	1,48
POS-pigm.	30,45	2,39	2,14

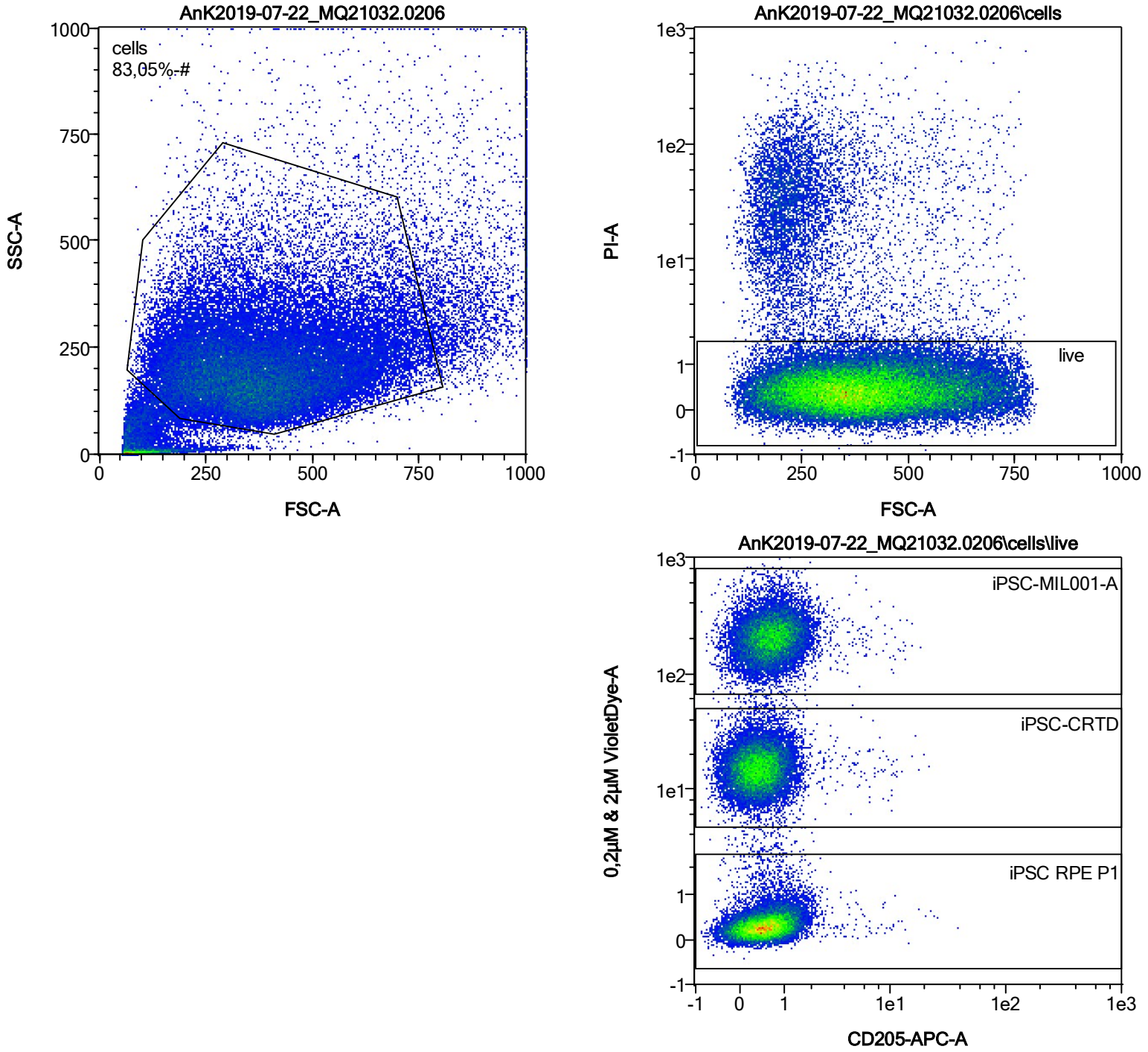

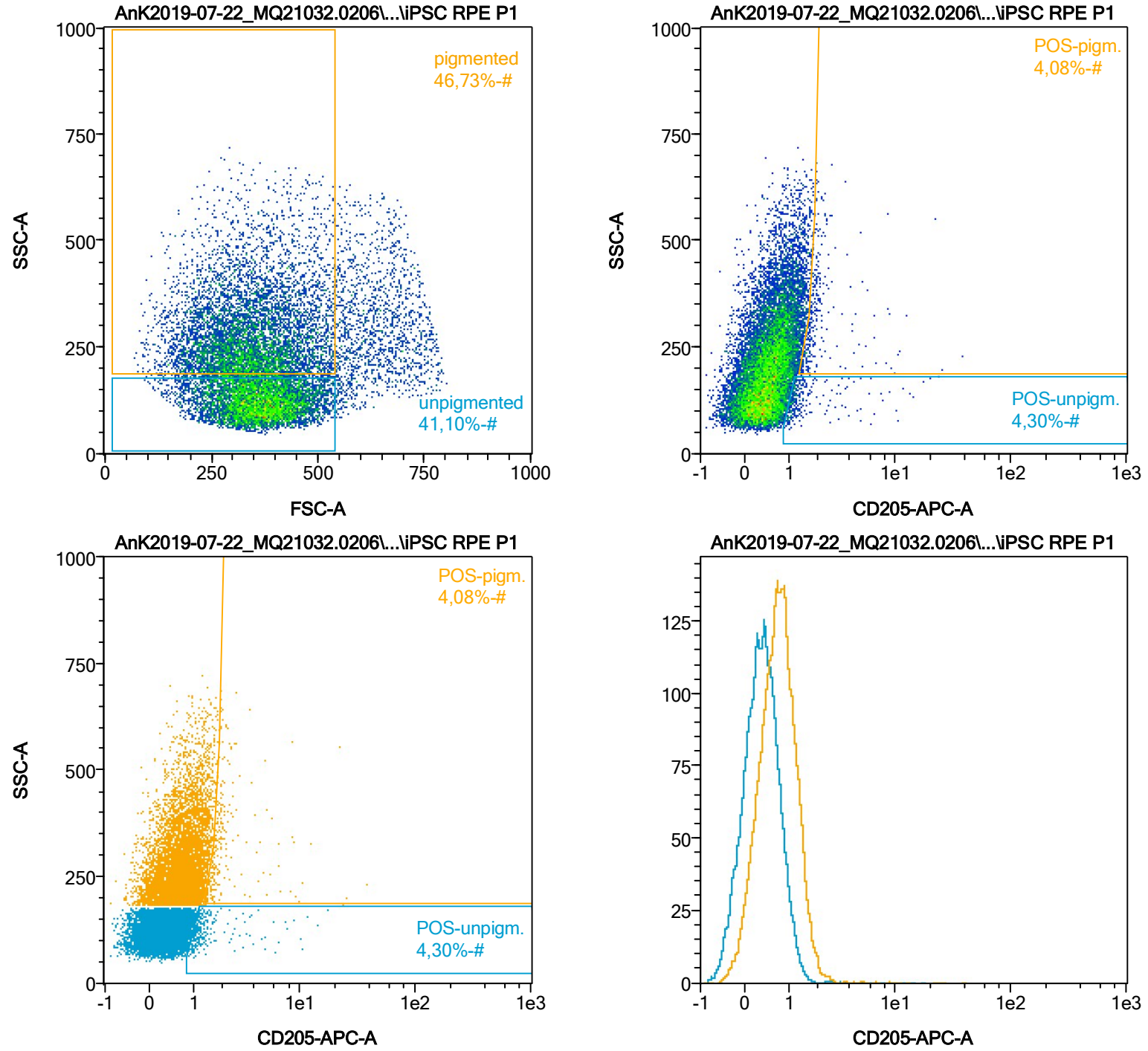

Name	%-#	CD205-APC-A Mean	CD205-APC-A Median
POS-unpigm.	4,30	1,73	1,19
POS-pigm.	4,08	2,59	1,82

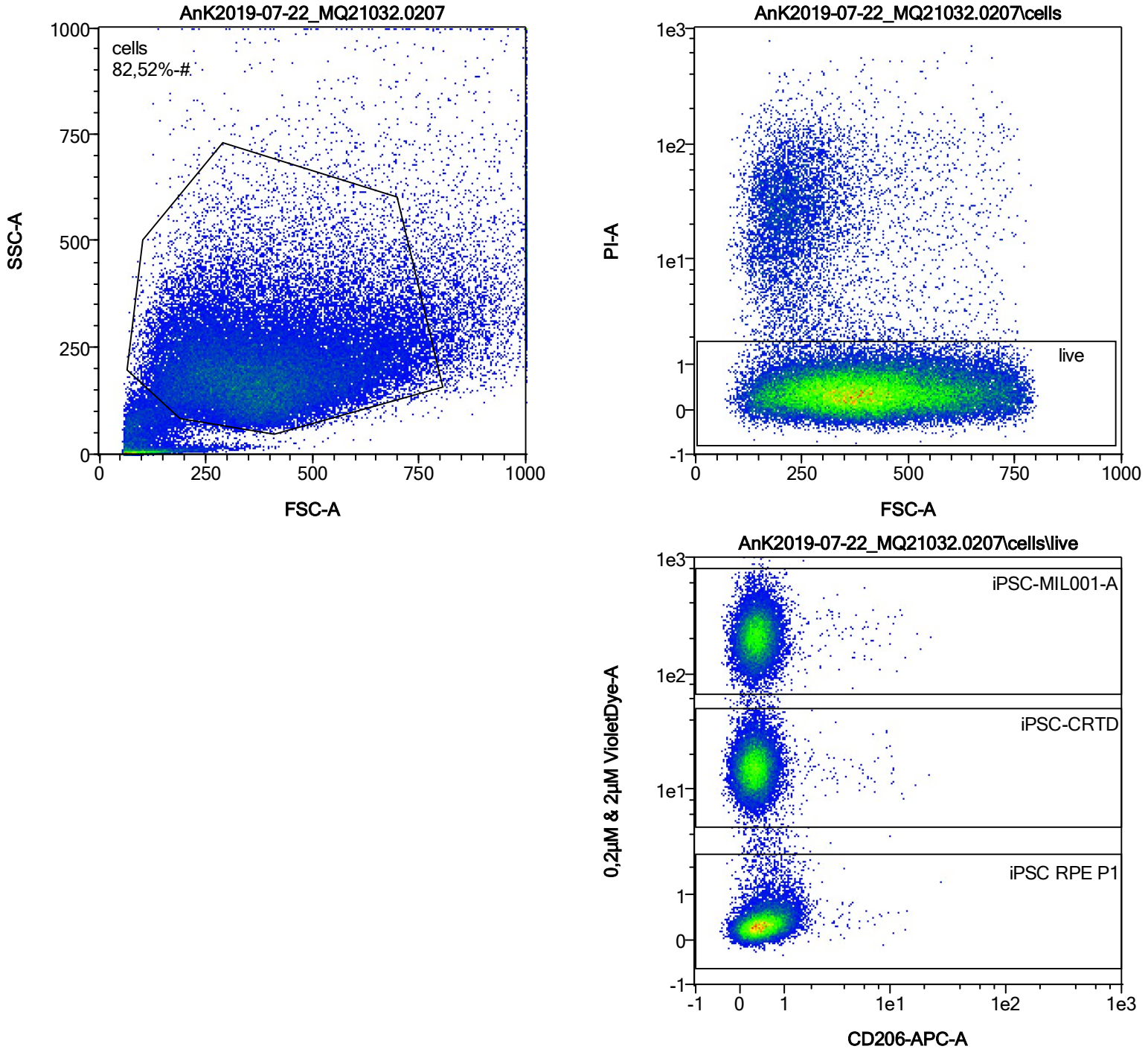

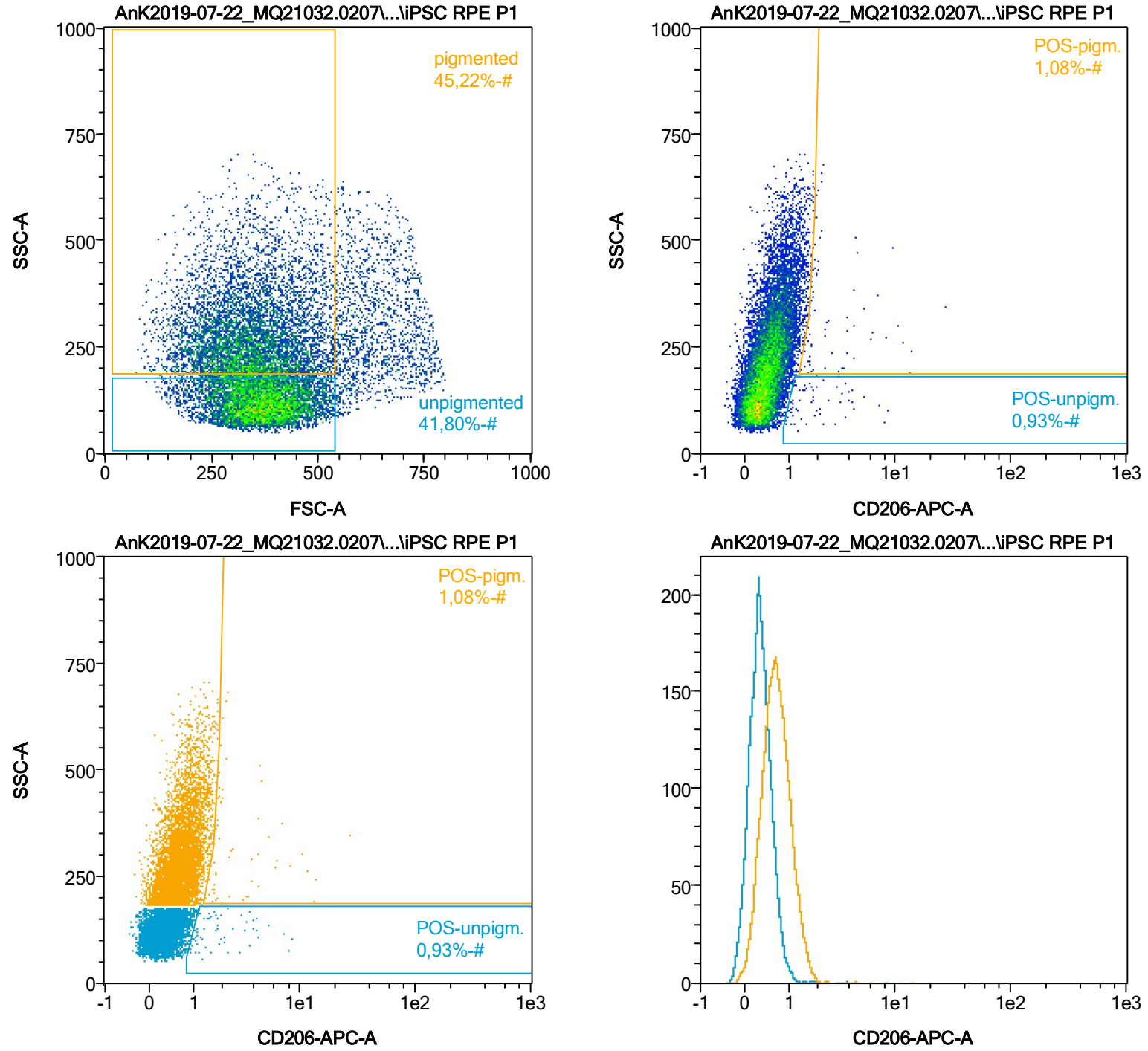

Name	%-#	CD206-APC-A Mean	CD206-APC-A Median
POS-unpigm.	0,93	2,28	1,71
POS-pigm.	1,08	3,40	1,91

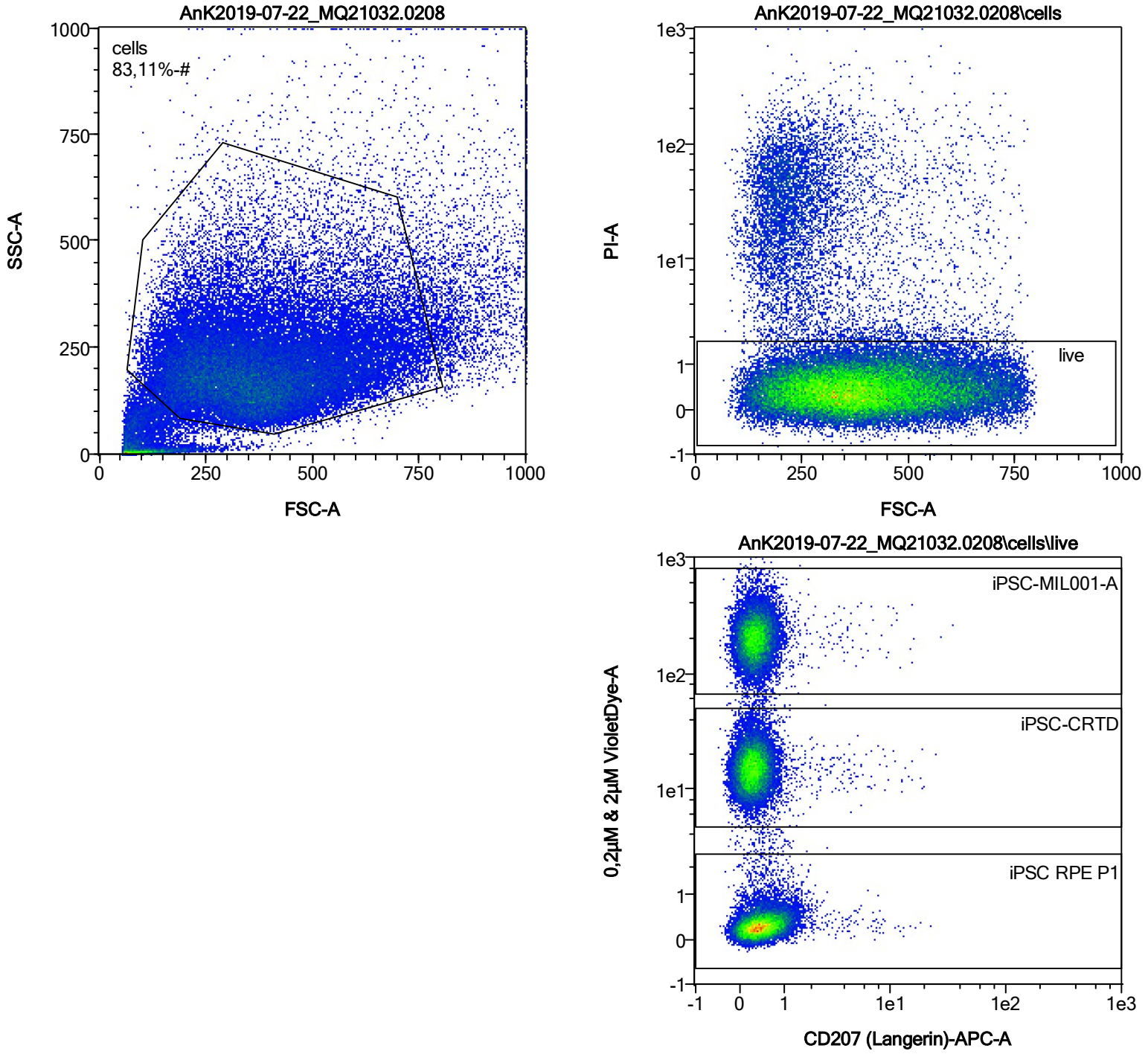

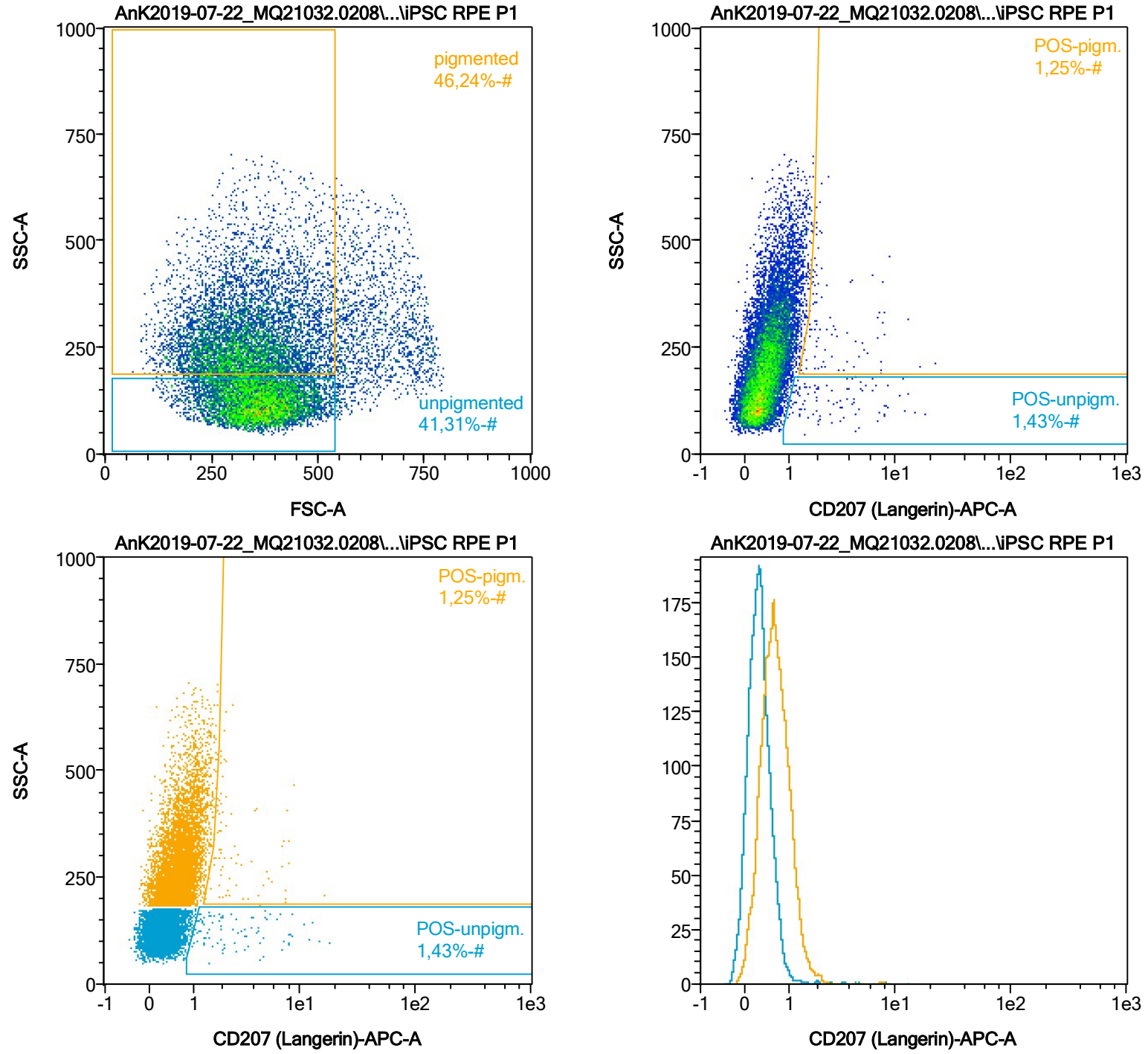

Name	%-#	CD207 (Langerin)-APC-A Mean	CD207 (Langerin)-APC-A Median
POS-unpigm.	1,43	3,62	2,07
POS-pigm.	1,25	3,16	2,10

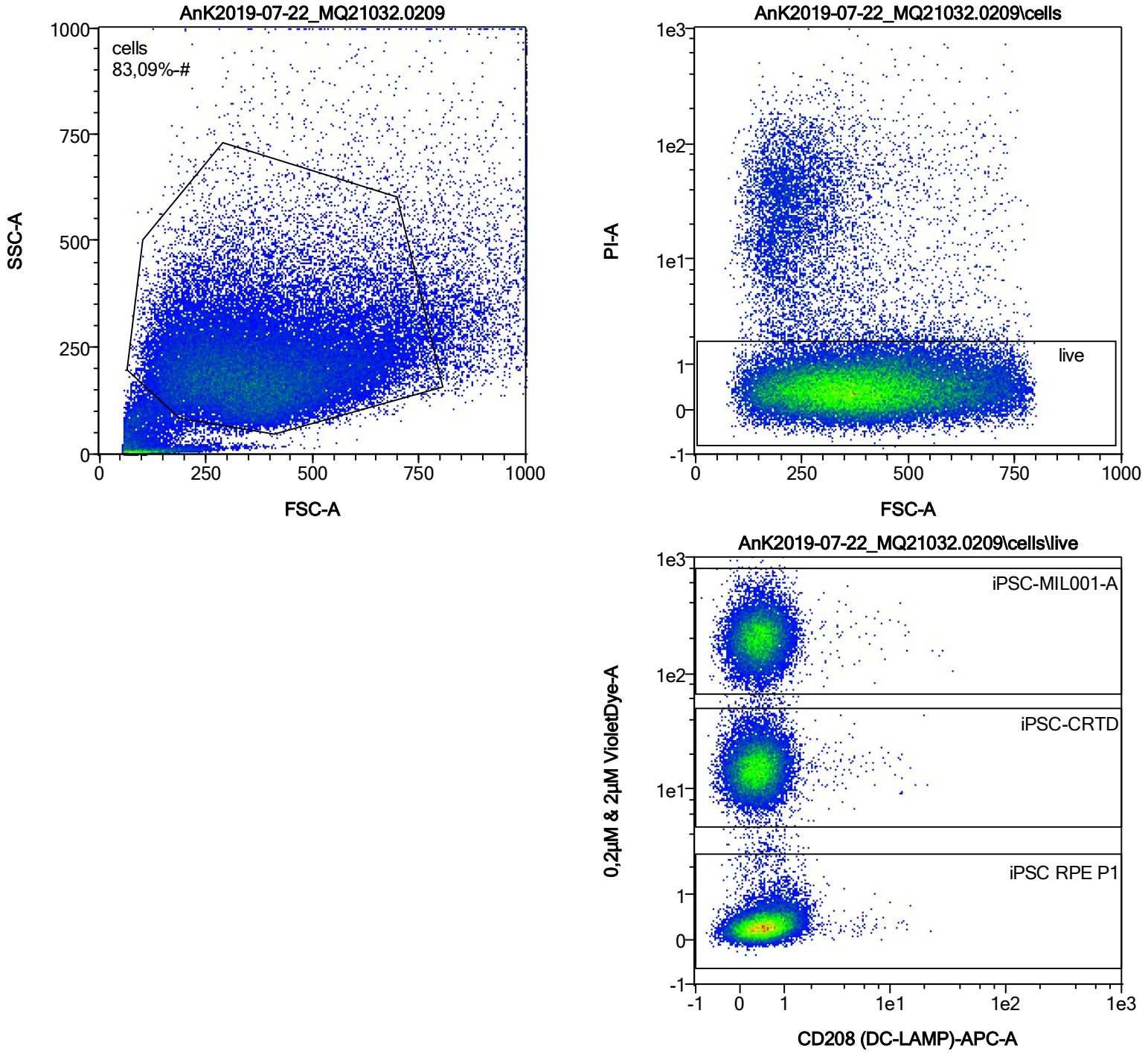

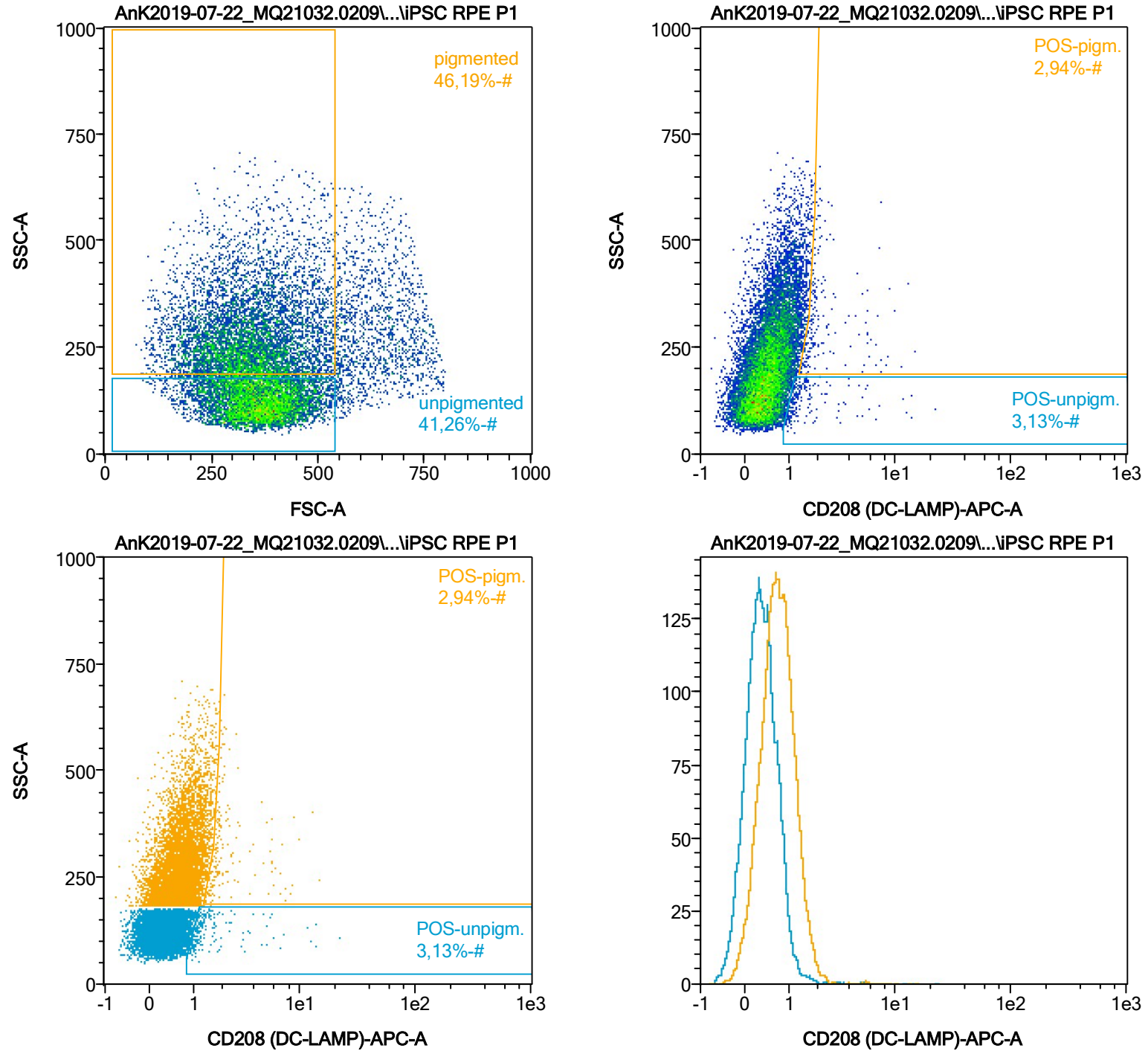

Name	%-#	CD208 (DC-LAMP)-APC-A Mean	CD208 (DC-LAMP)-APC-A Median
POS-unpigm.	3,13	1,93	1,22
POS-pigm.	2,94	2,50	1,84

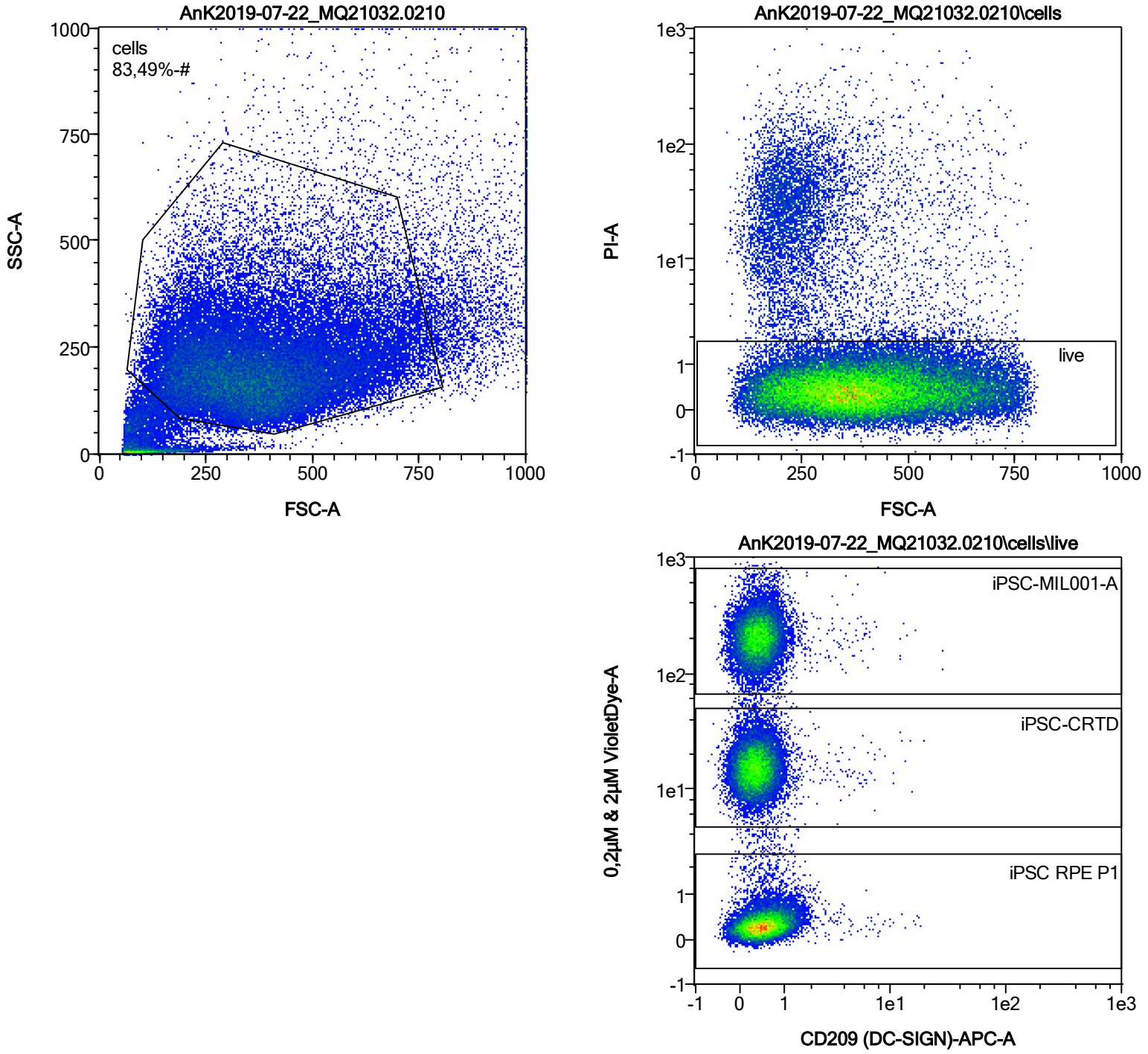

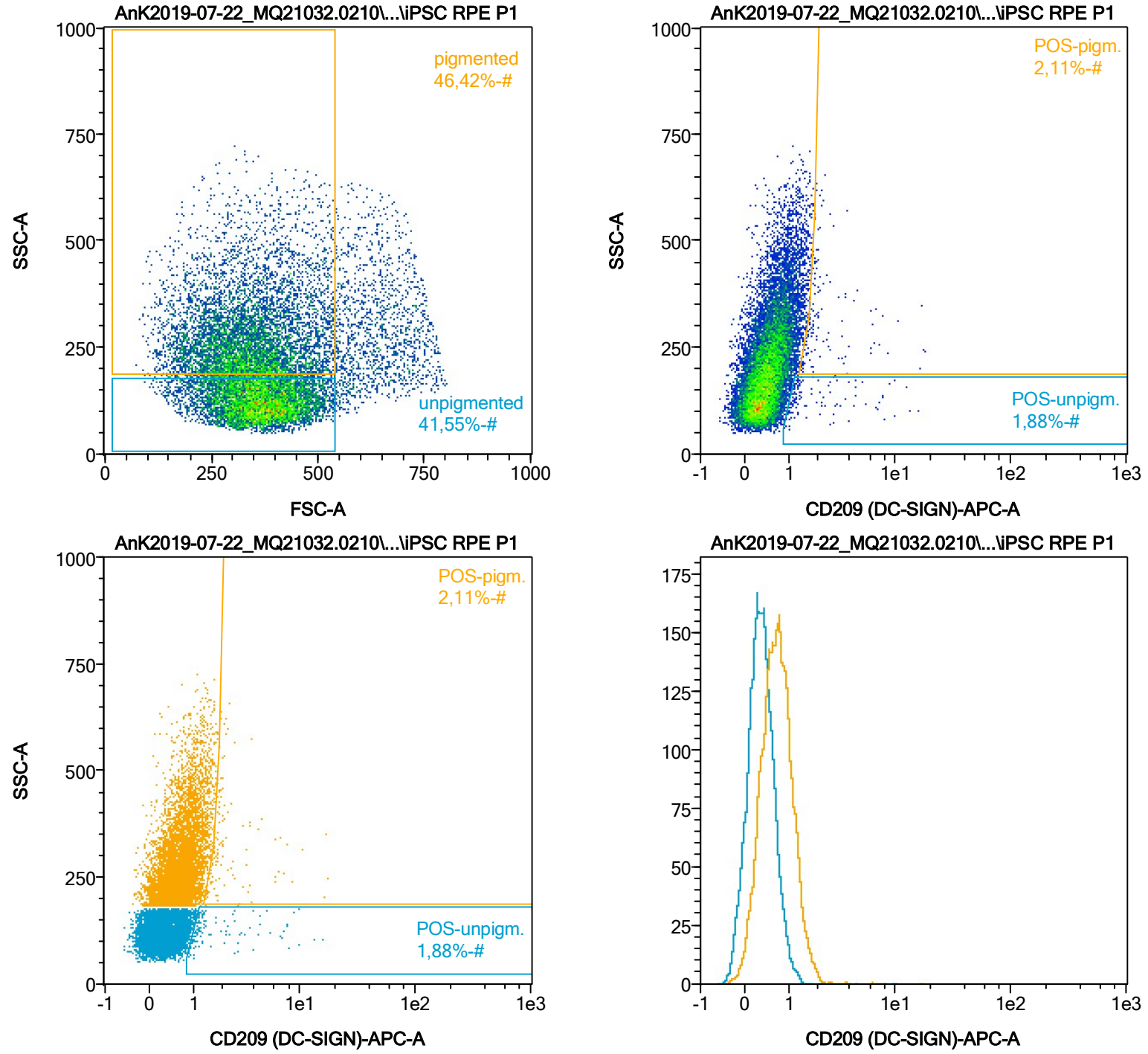

Name	%-#	CD209 (DC-SIGN)-APC-A Mean	CD209 (DC-SIGN)-APC-A Median
POS-unpigm.	1,88	2,38	1,25
POS-pigm.	2,11	2,67	1,90

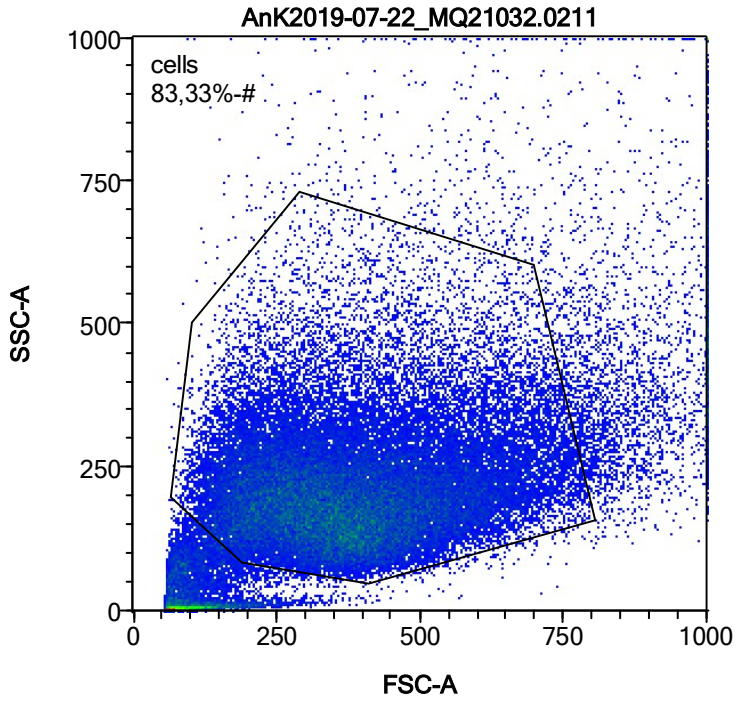

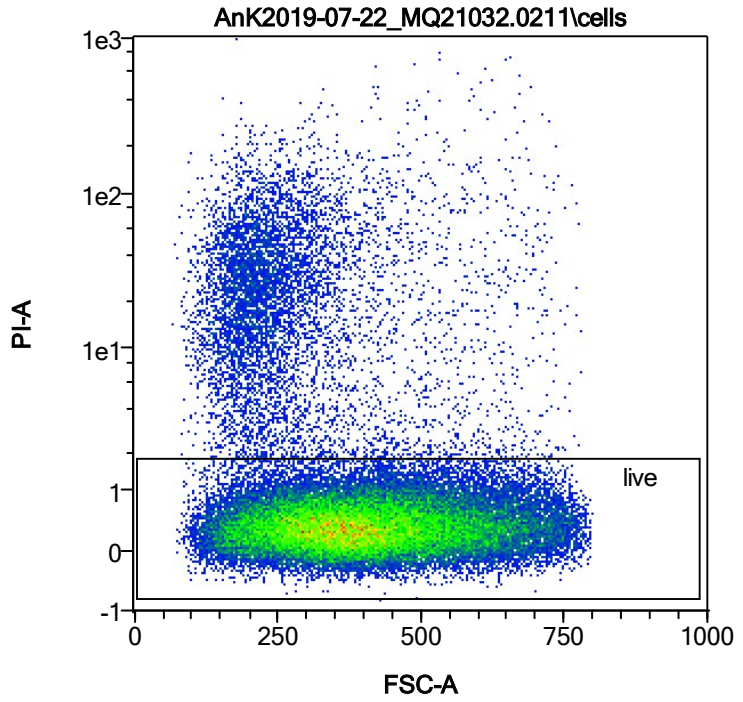

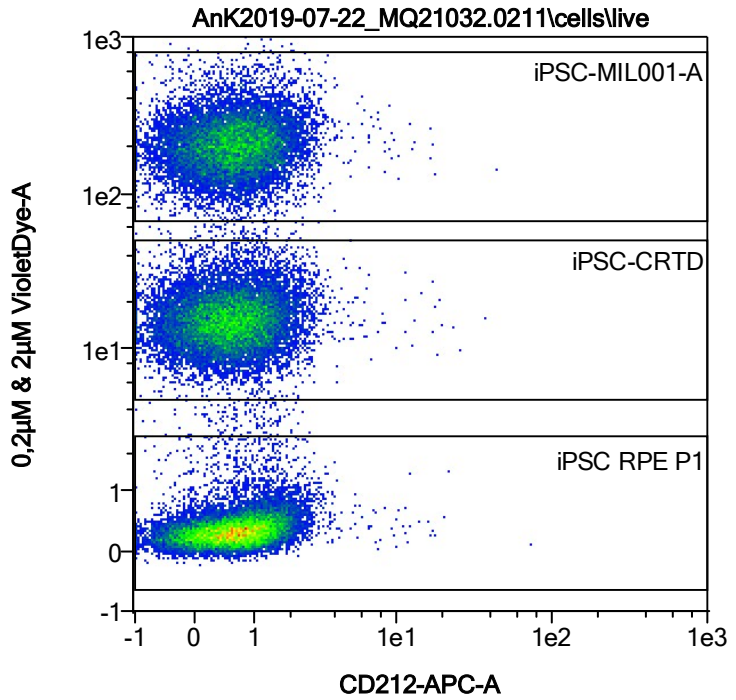

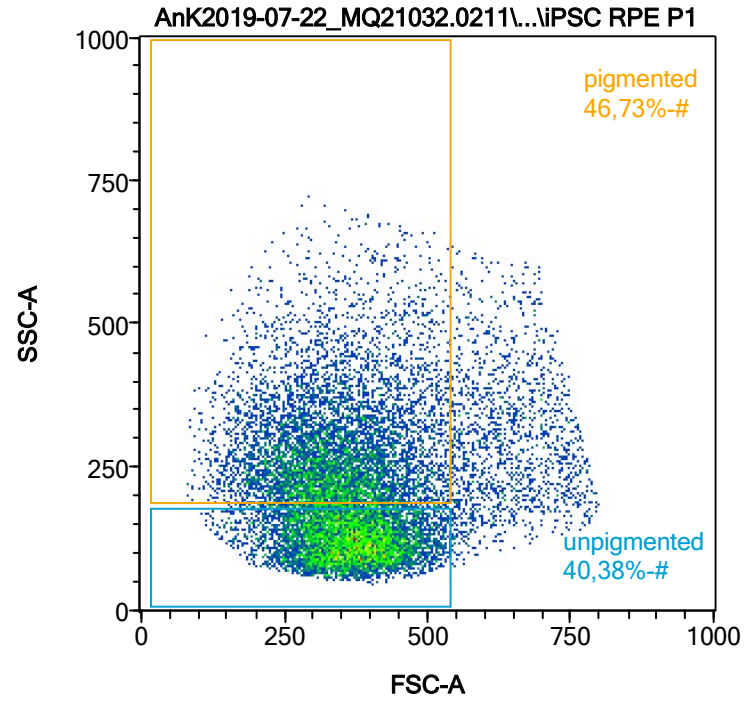

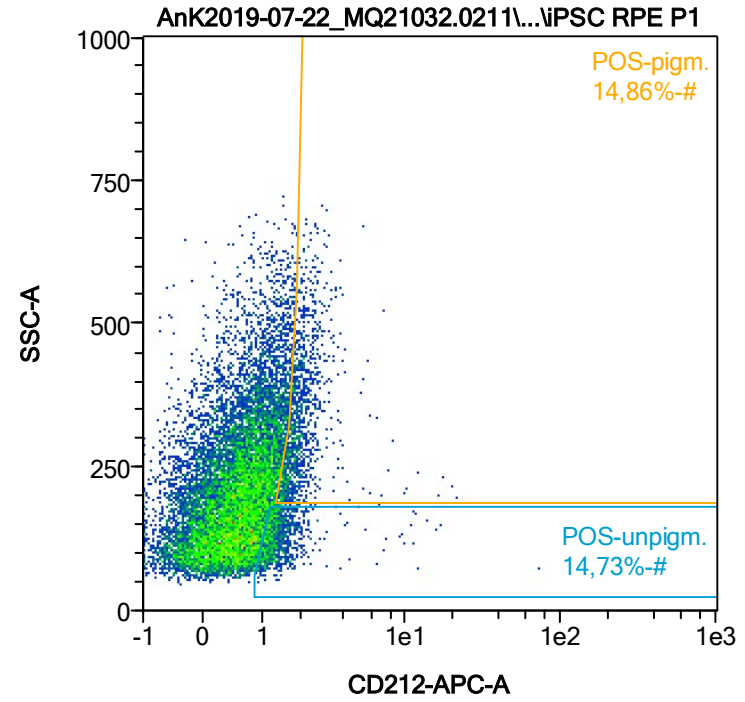

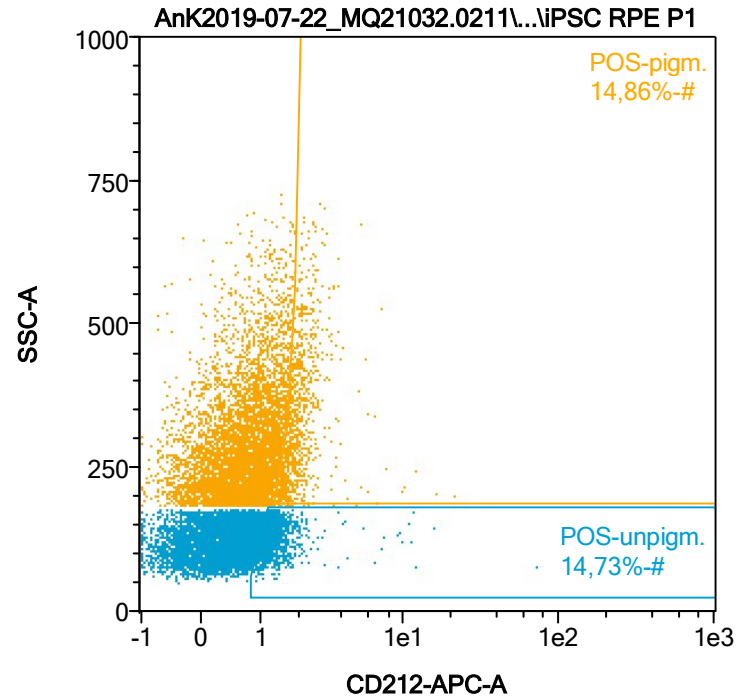

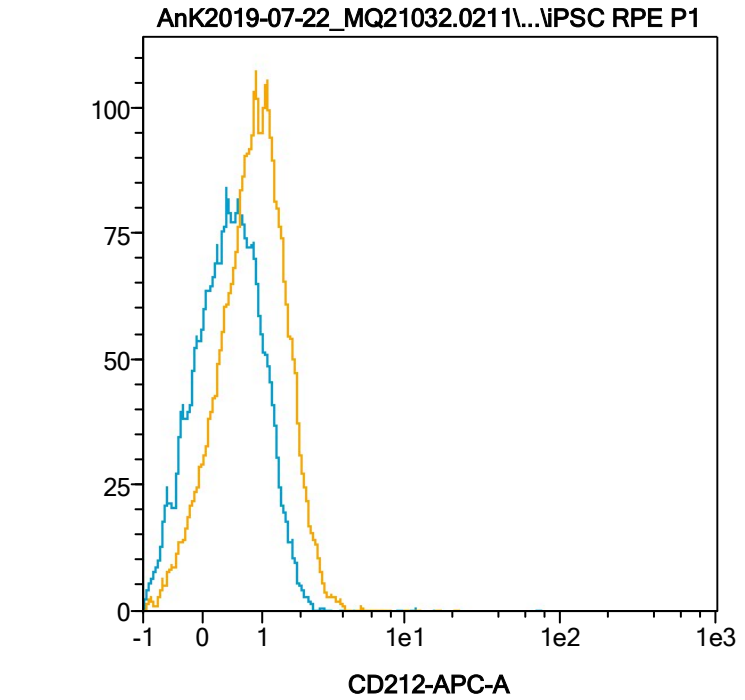

Name	%-#	CD212-APC-A Mean	CD212-APC-A Median
POS-unpigm.	14,73	1,56	1,27
POS-pigm.	14,86	2,09	1,89

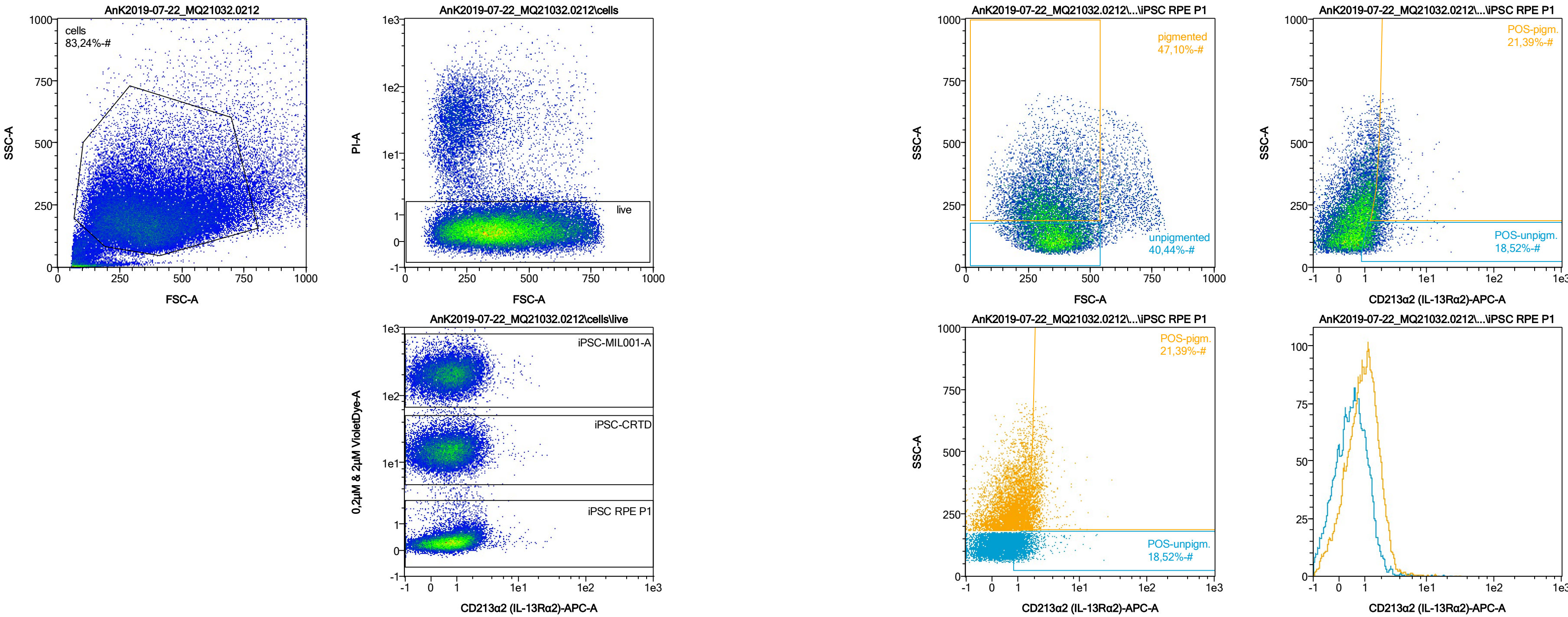

Name	%-#	CD213α2 (IL-13Rα2)-APC-A Mean	CD213α2 (IL-13Rα2)-APC-A Median
POS-unpigm.	18,52	1,60	1,33
POS-pigm.	21,39	2,21	1,96

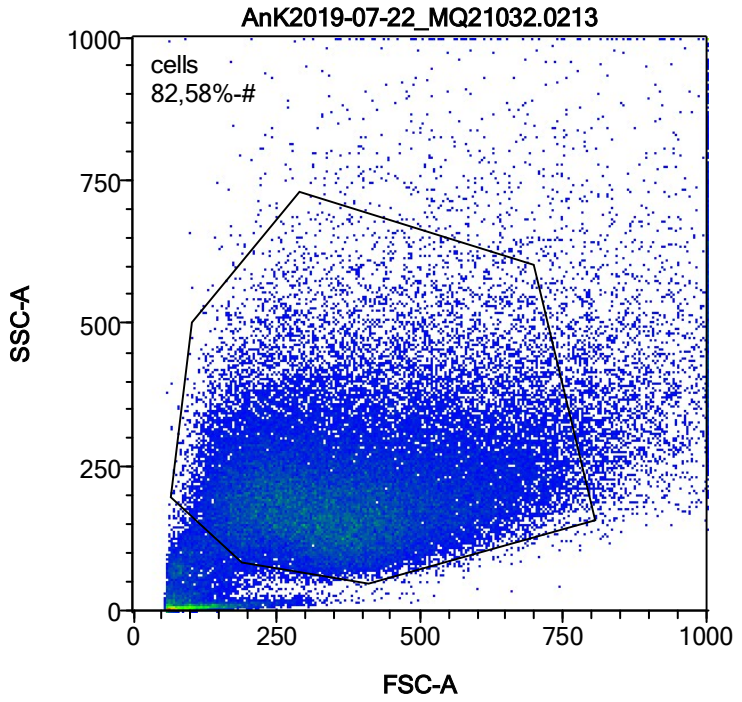

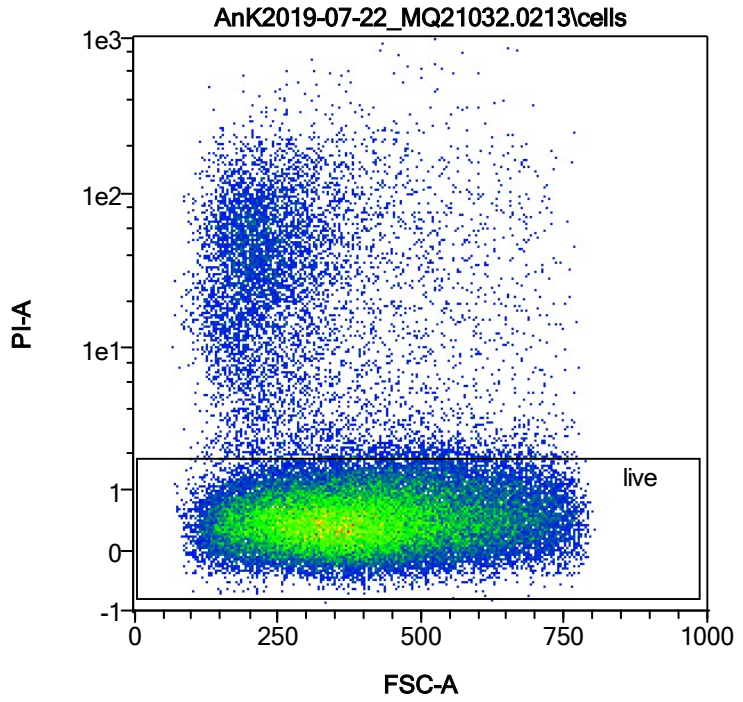

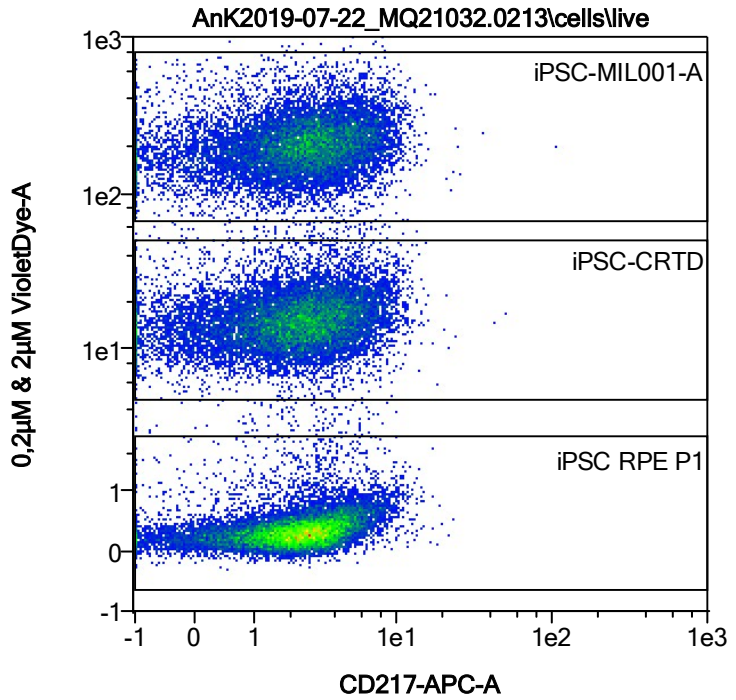

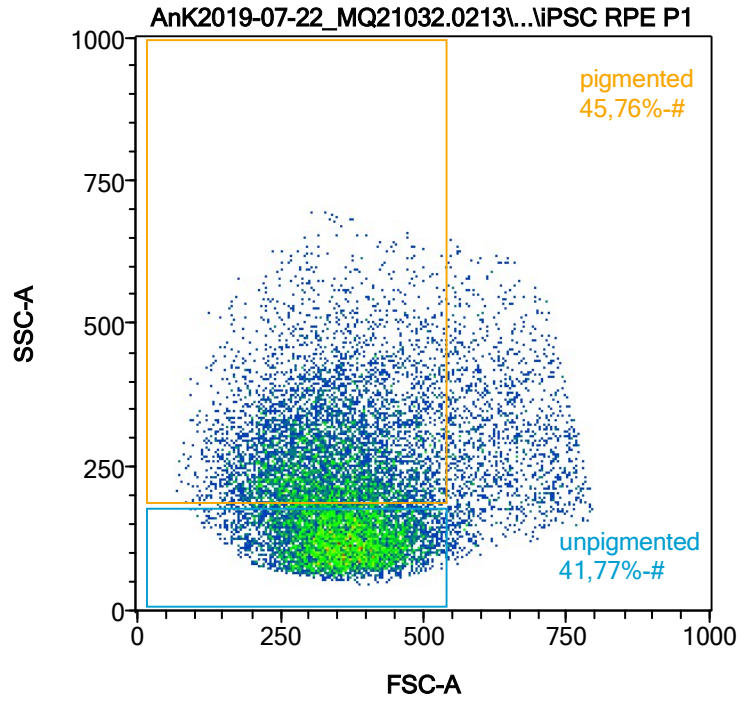

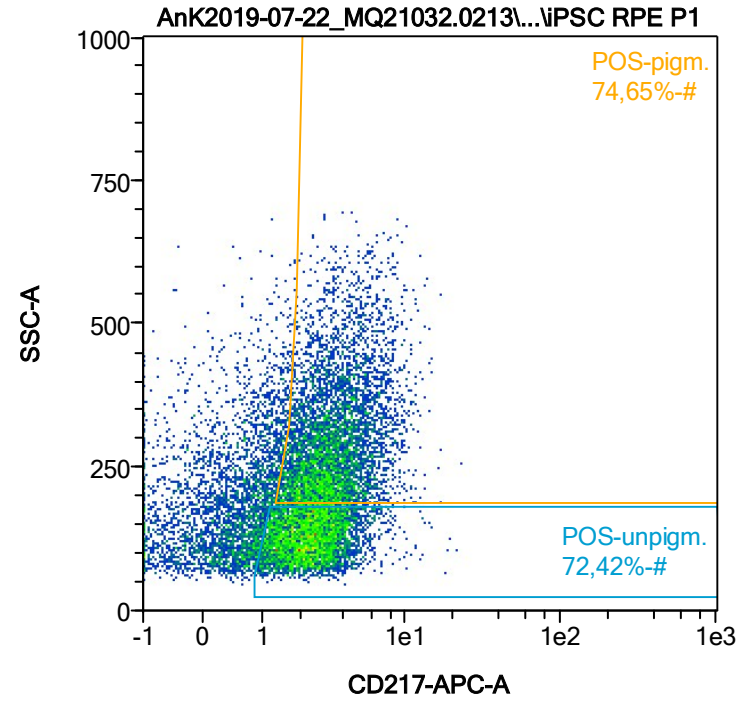

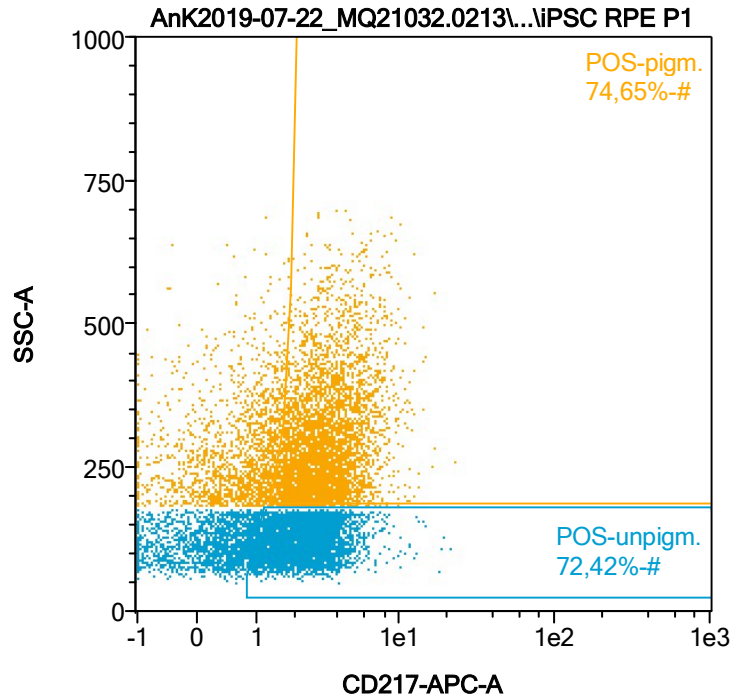

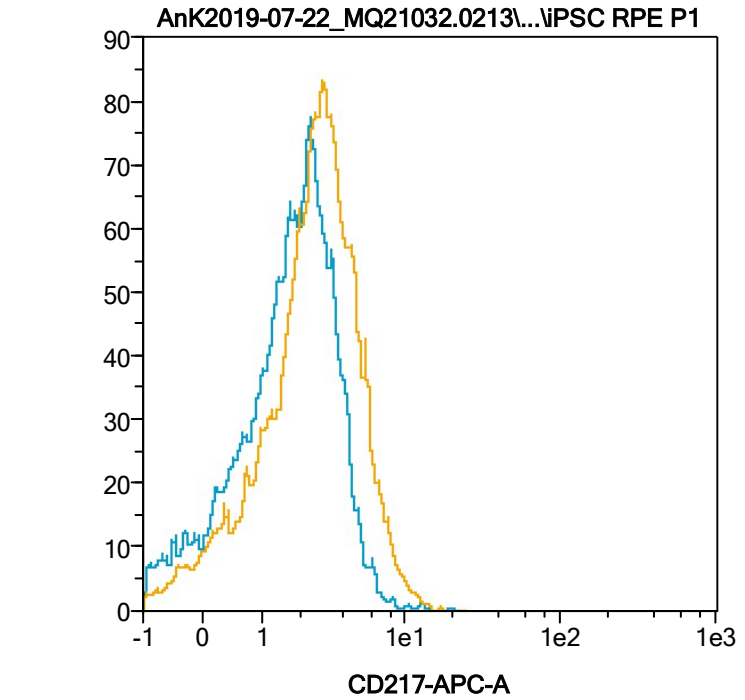

Name	%-#	CD217-APC-A Mean	CD217-APC-A Median
POS-unpigm.	72,42	2,54	2,28
POS-pigm.	74,65	3,54	3,10

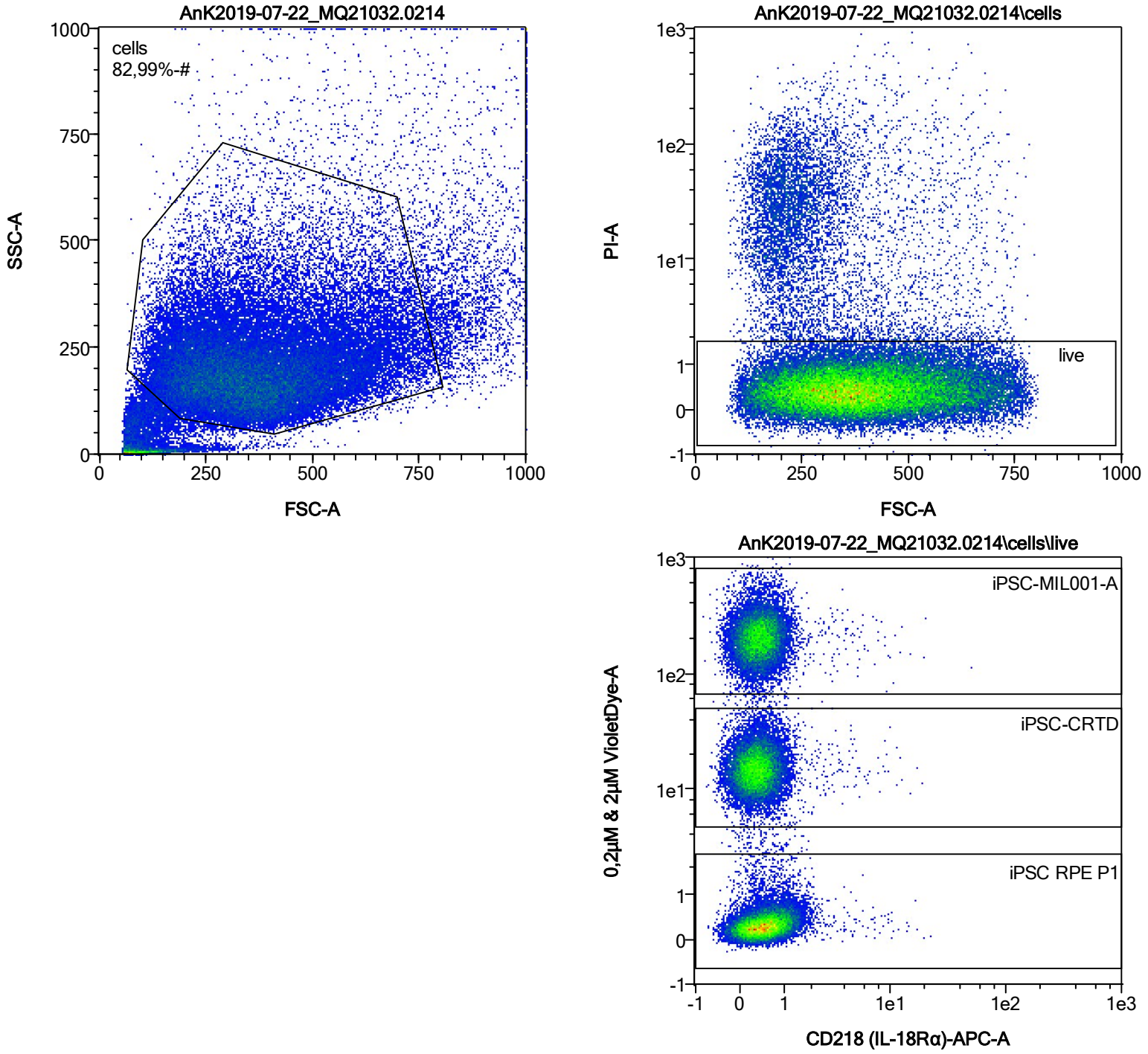

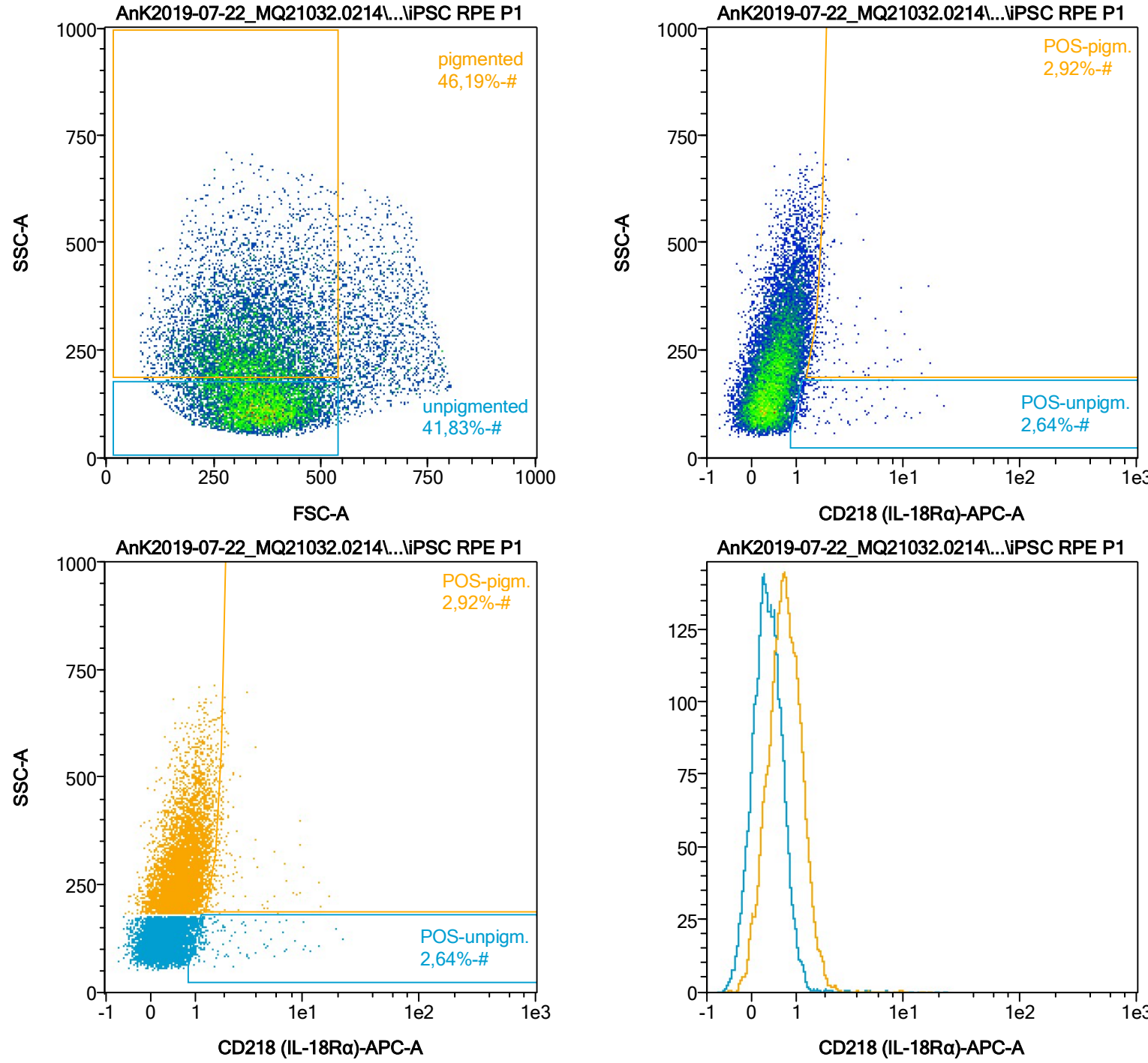

Name	%-#	CD218 (IL-18Rα)-APC-A Mean	CD218 (IL-18Rα)-APC-A Median
POS-unpigm.	2,64	2,38	1,24
POS-pigm.	2,92	2,51	1,87

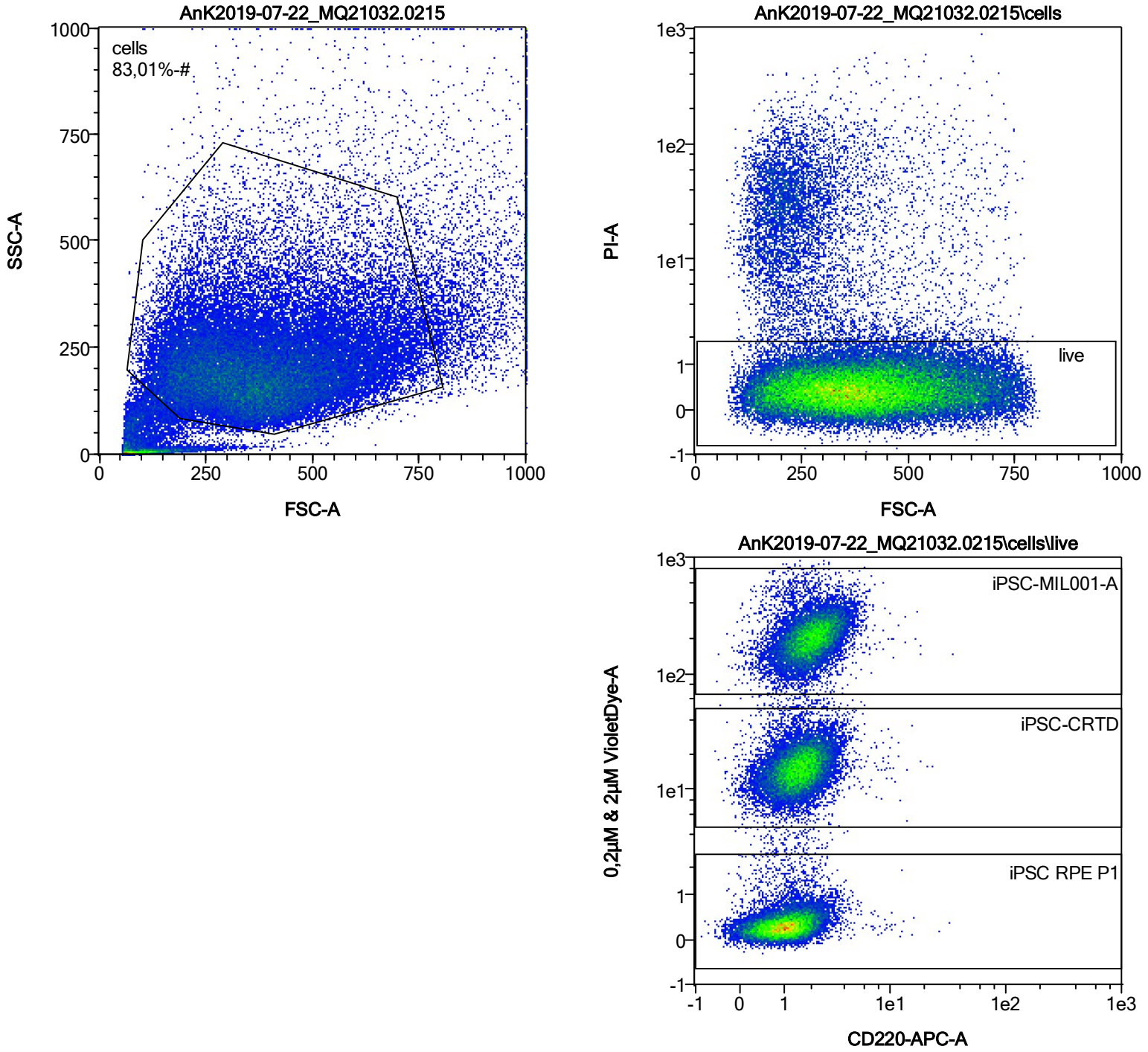

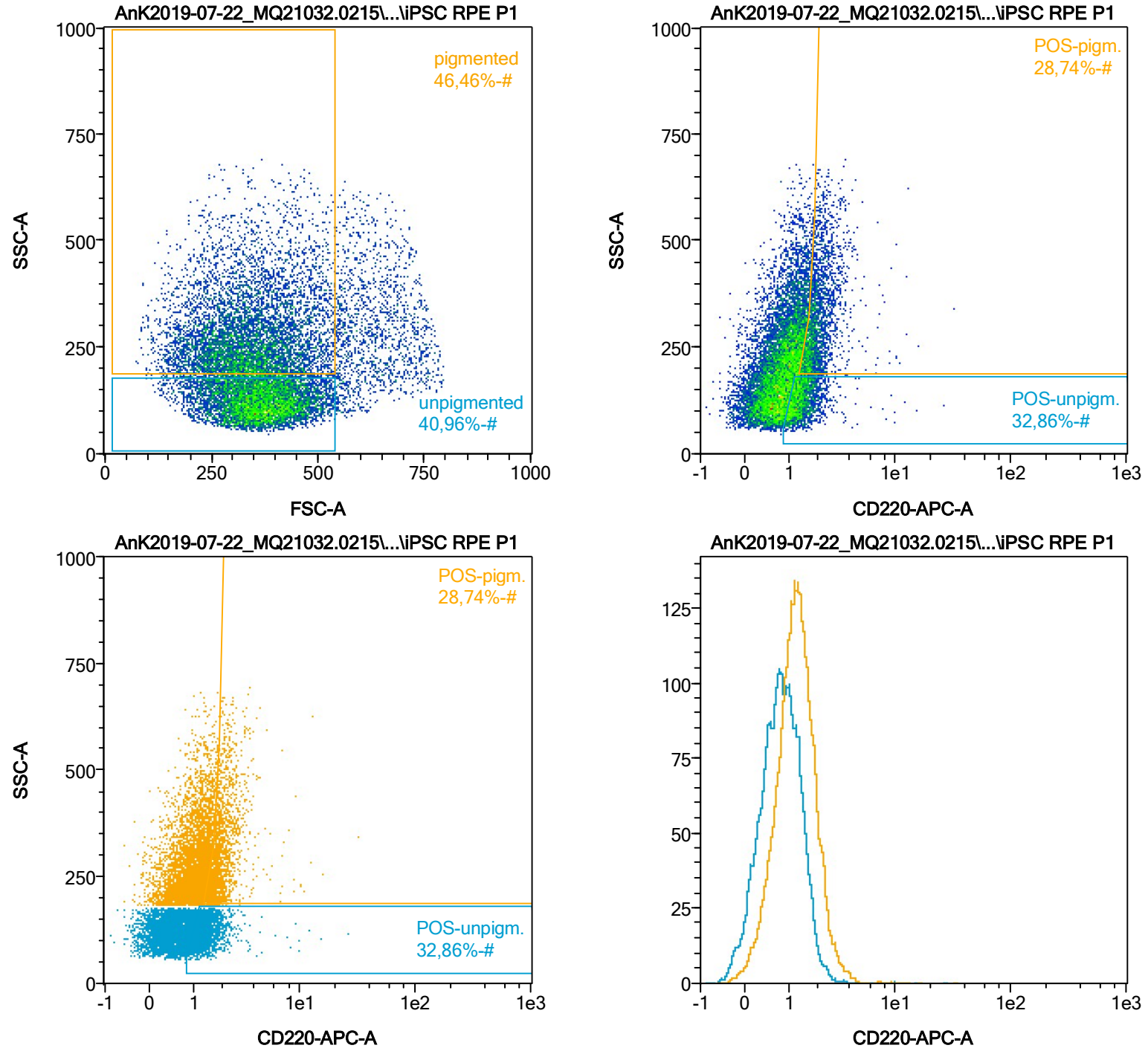

Name	%-#	CD220-APC-A Mean	CD220-APC-A Median
POS-unpigm.	32,86	1,45	1,31
POS-pigm.	28,74	2,08	1,89

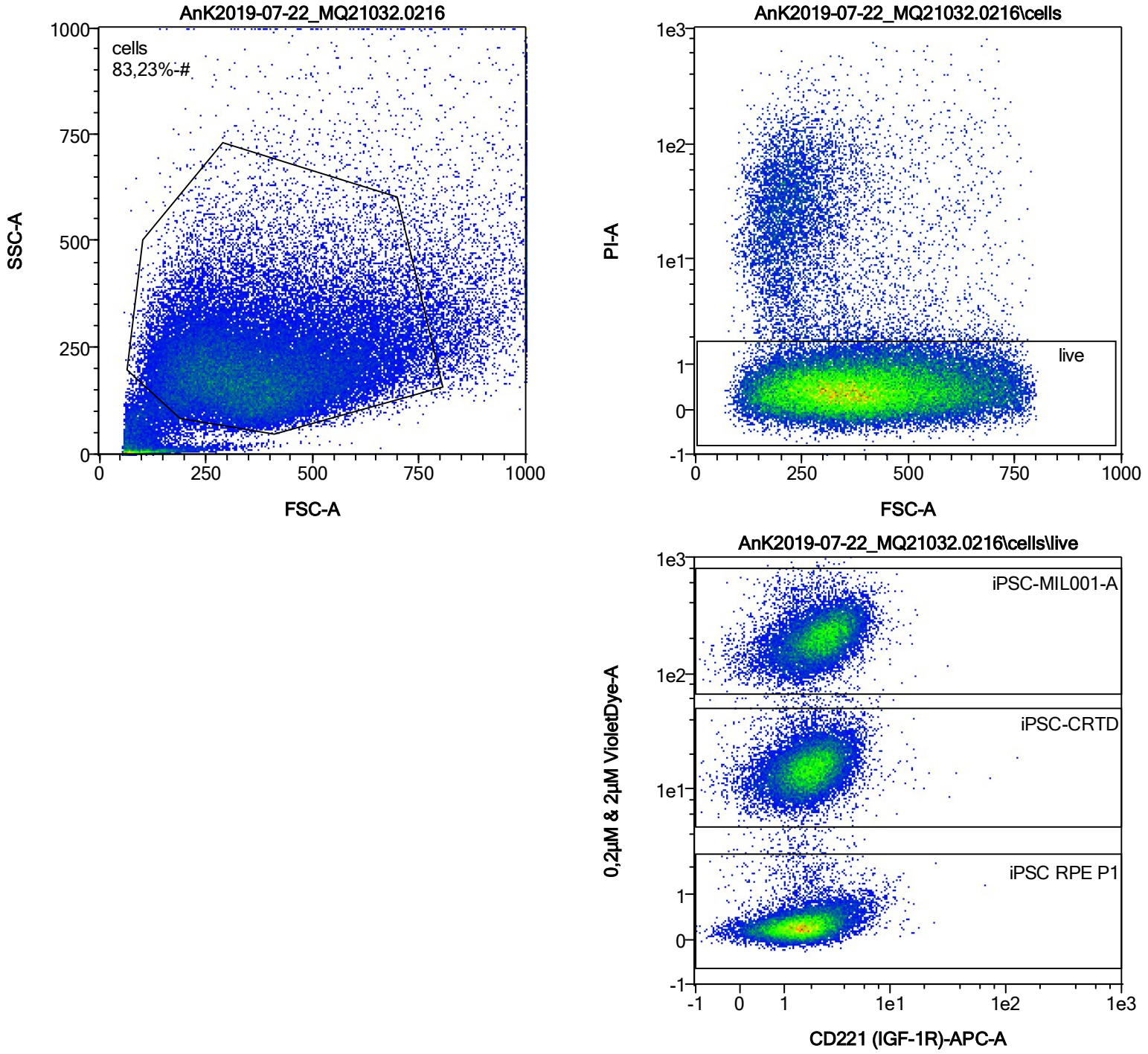

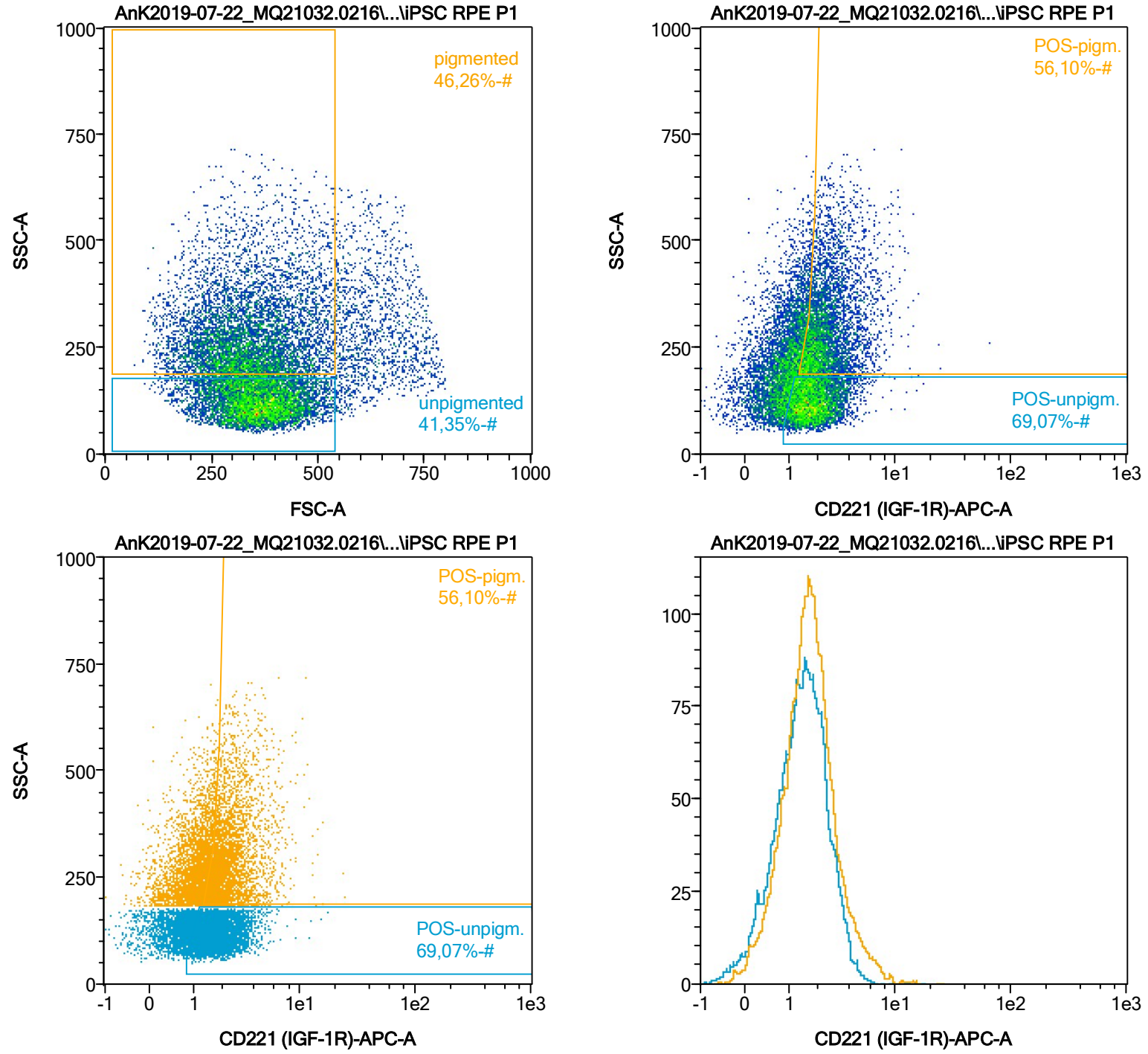

Name	%-#	CD221 (IGF-1R)-APC-A Mean	CD221 (IGF-1R)-APC-A Median
POS-unpigm.	69,07	1,96	1,76
POS-pigm.	56,10	2,59	2,19

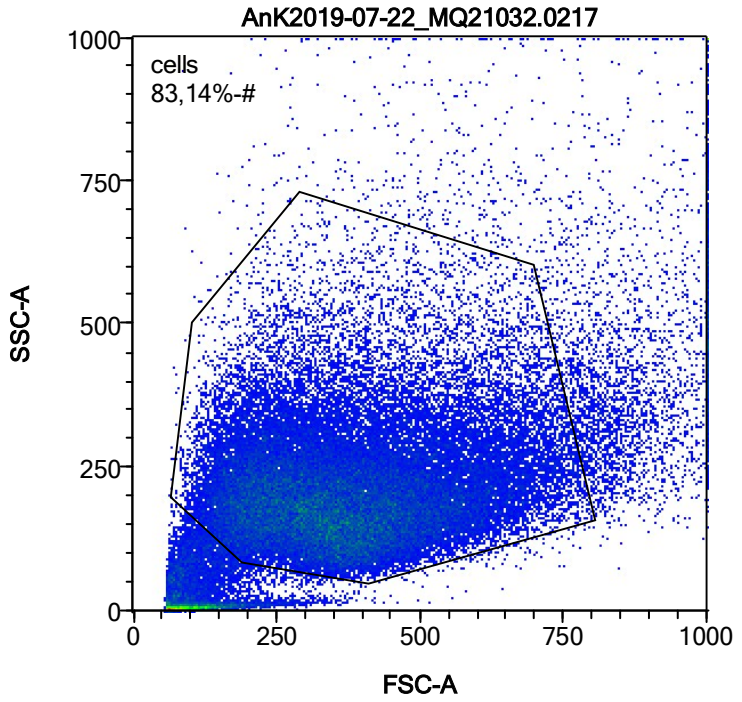

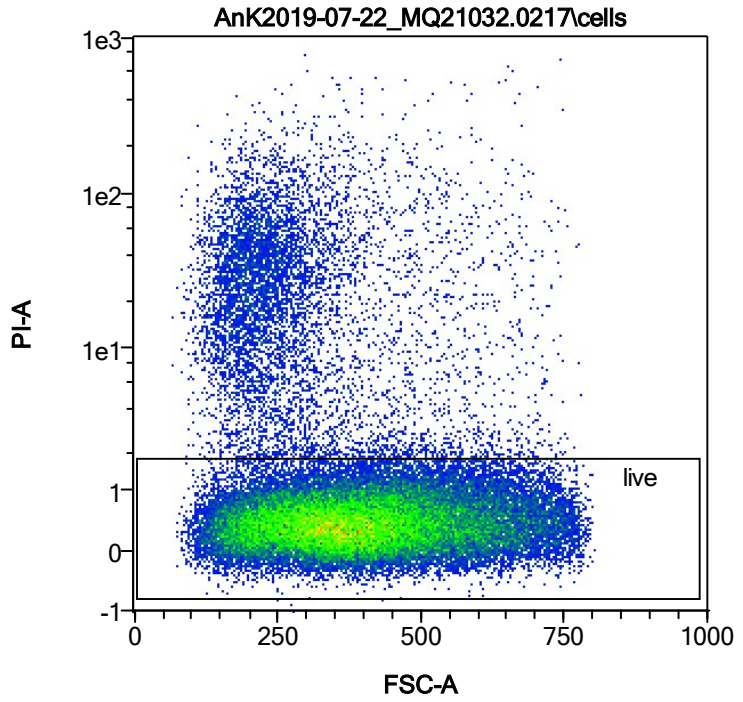

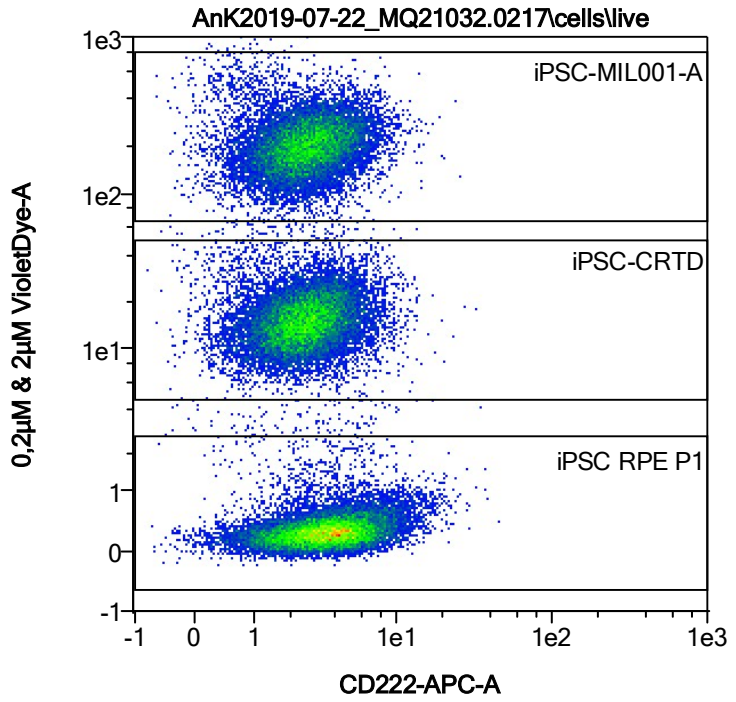

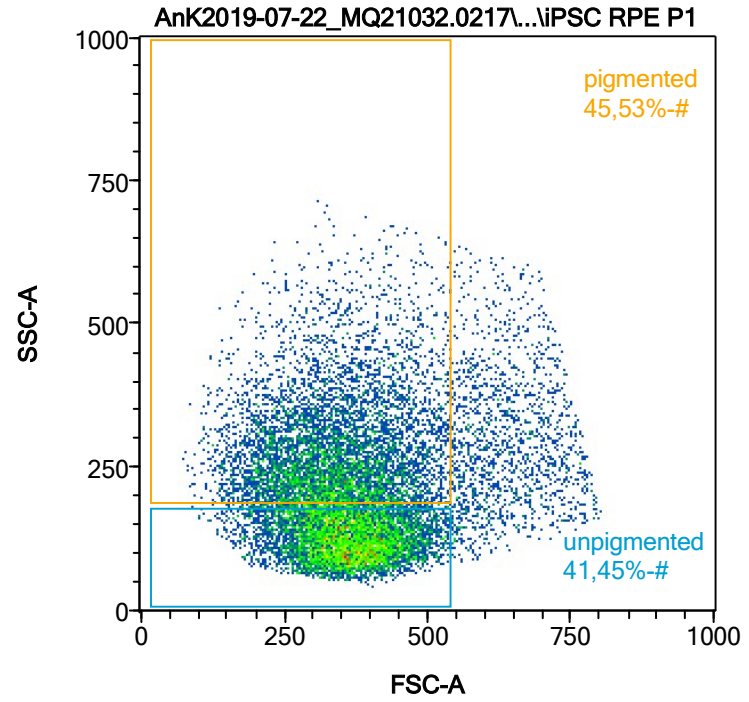

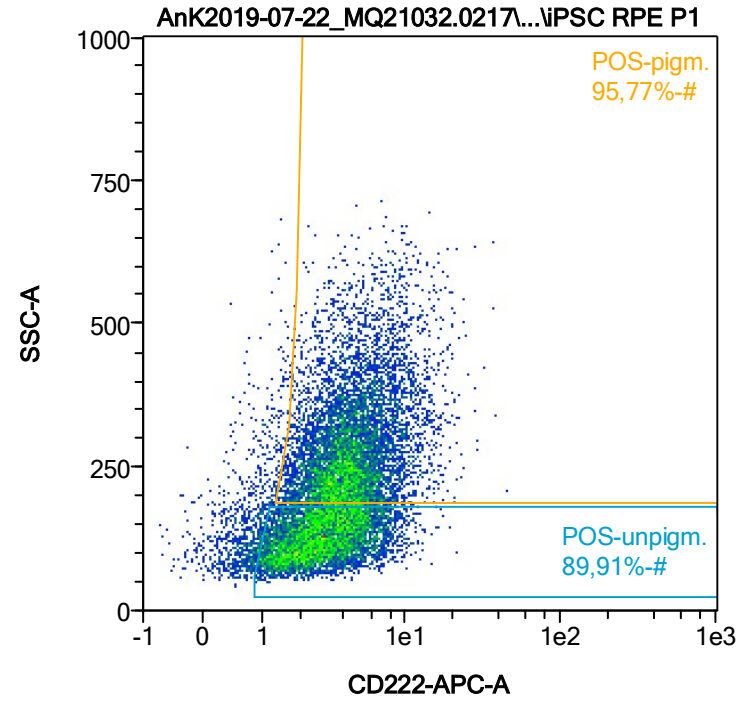

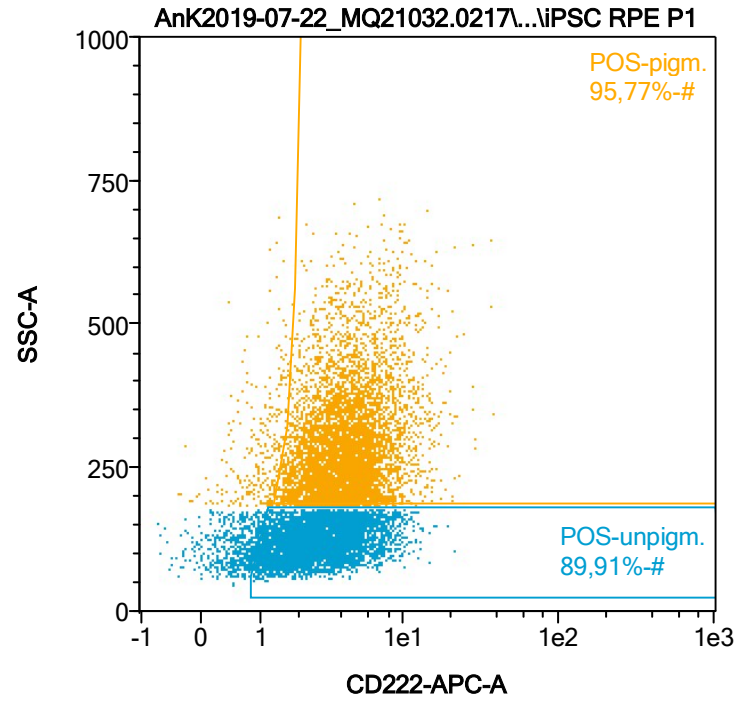

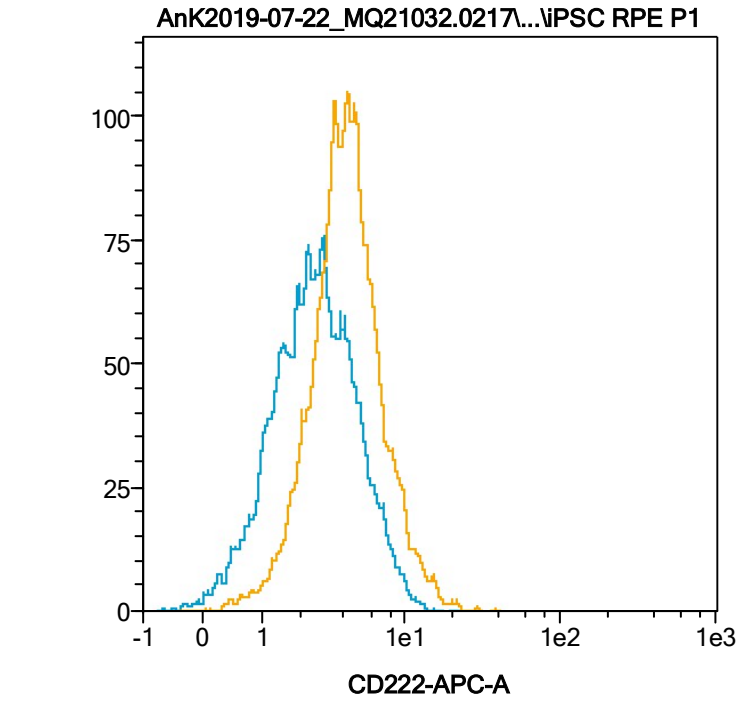

Name	%-#	CD222-APC-A Mean	CD222-APC-A Median
POS-unpigm.	89,91	3,24	2,74
POS-pigm.	95,77	4,81	4,16

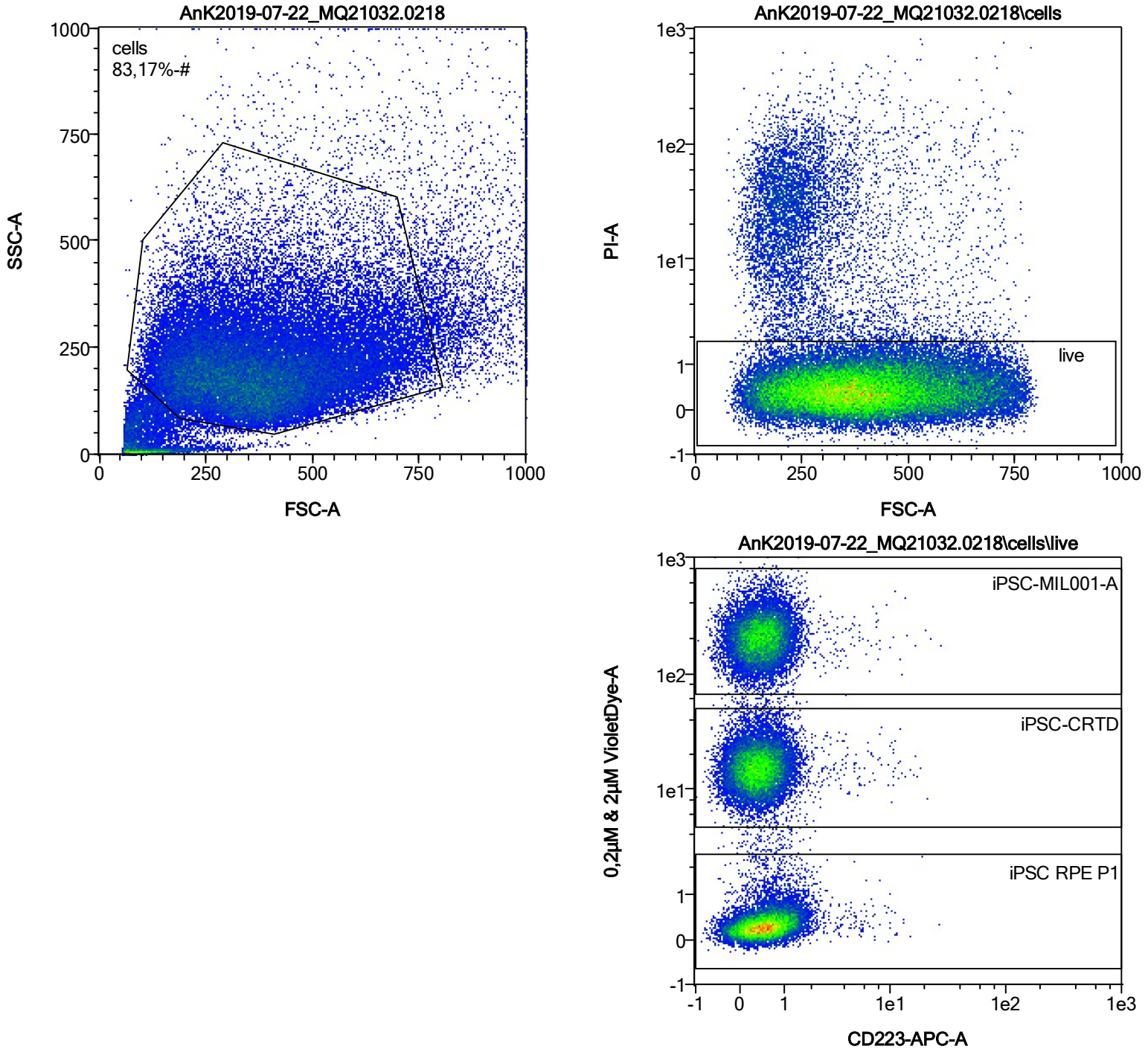

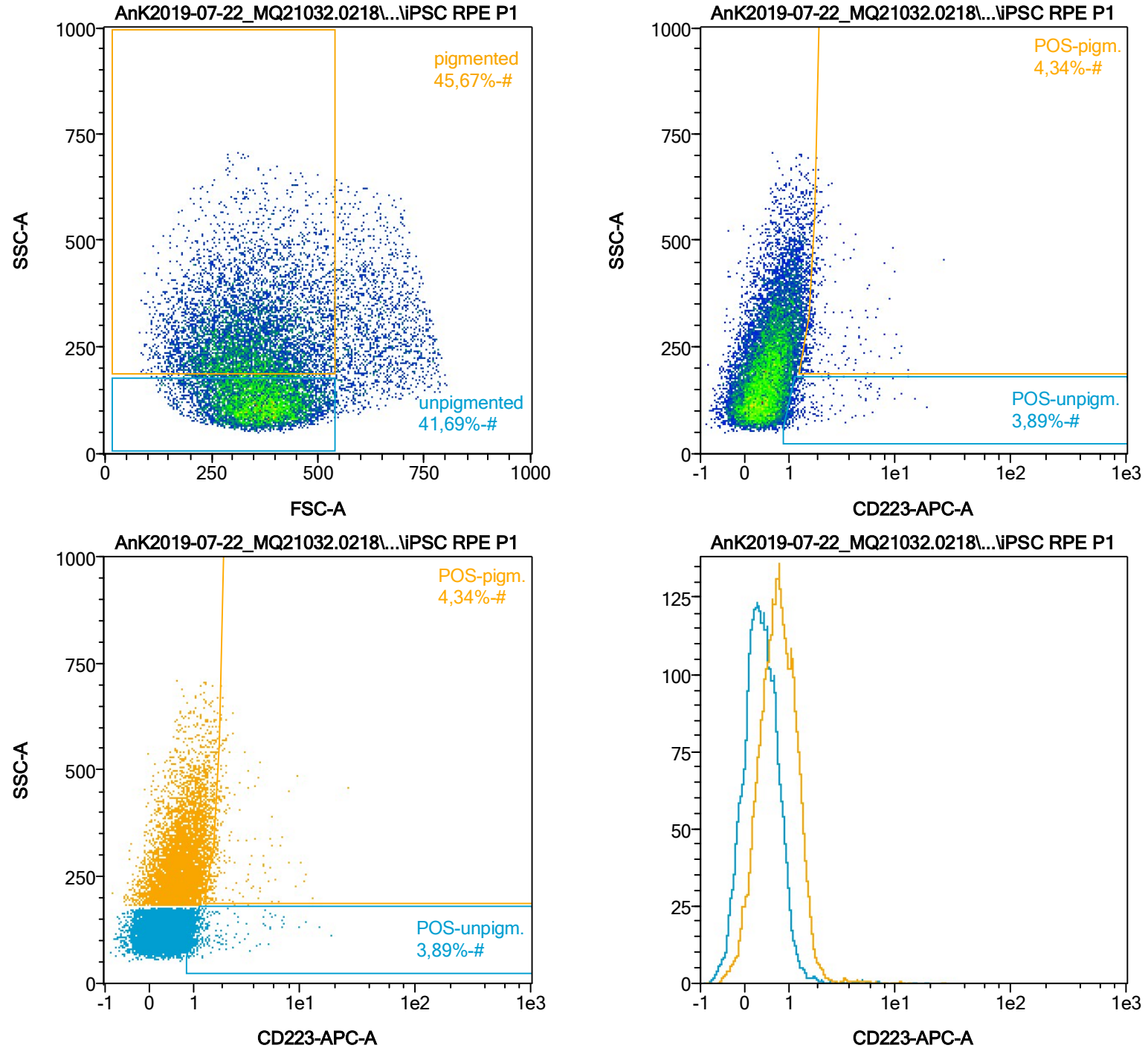

Name	%-#	CD223-APC-A Mean	CD223-APC-A Median
POS-unpigm.	3,89	1,86	1,23
POS-pigm.	4,34	2,49	1,82

Date:2024-06-14

Operator: Sebastian Knoebel

Version:2.13.0+54.g95abf0f4

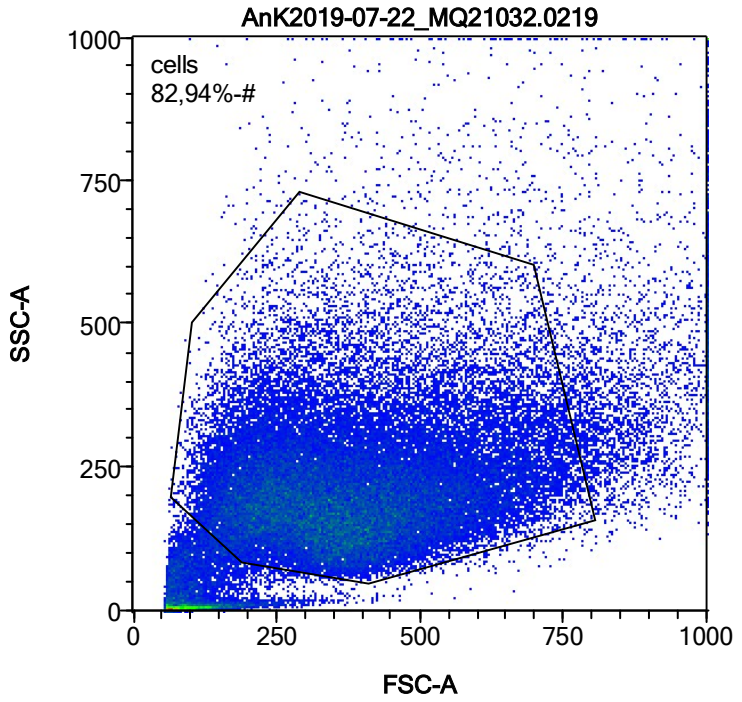

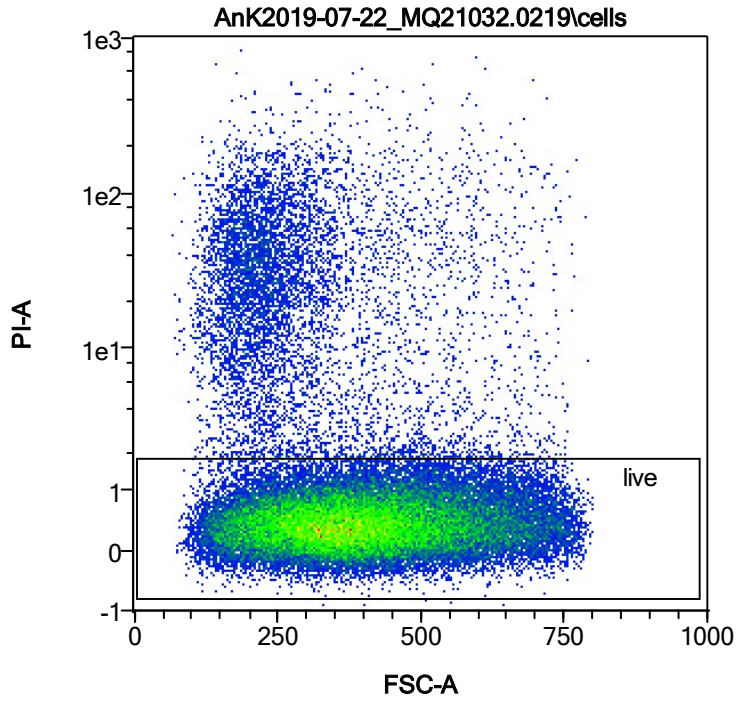

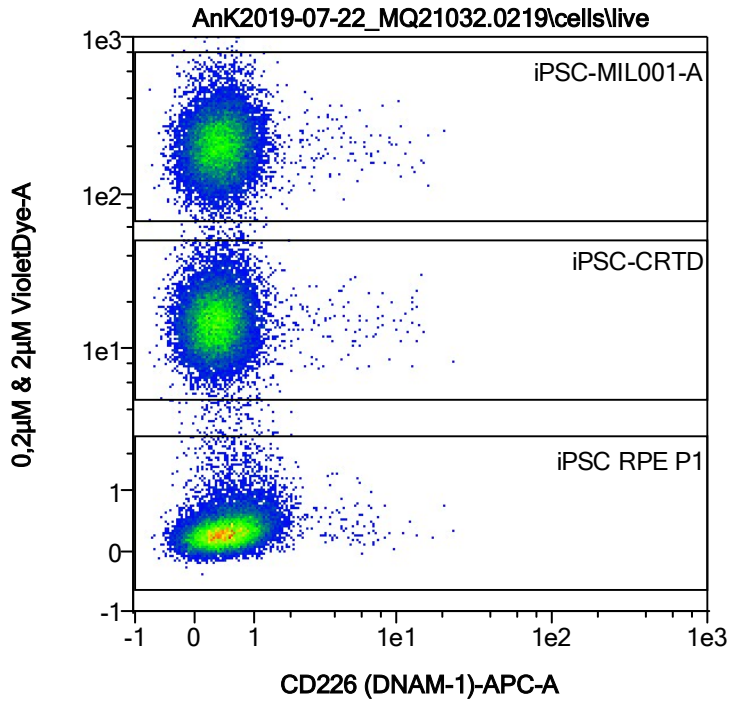

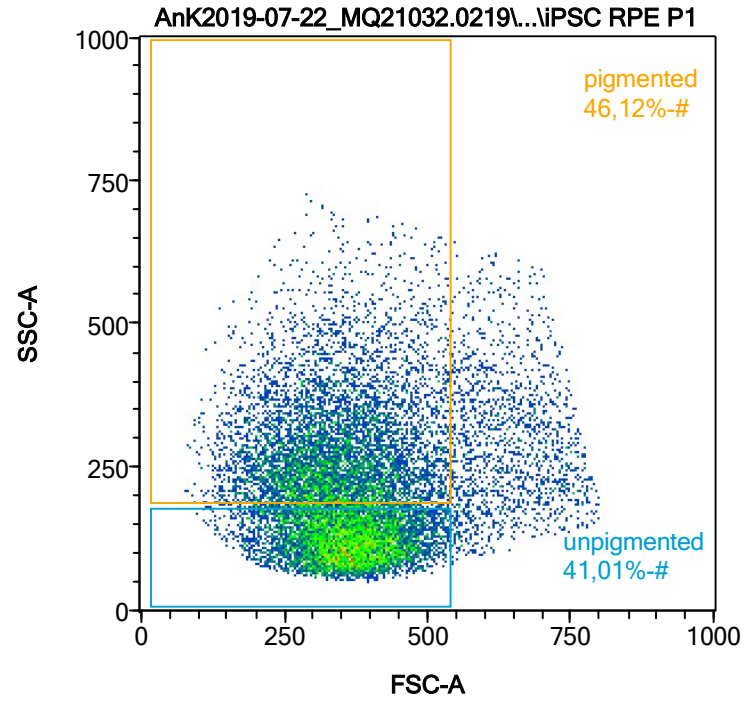

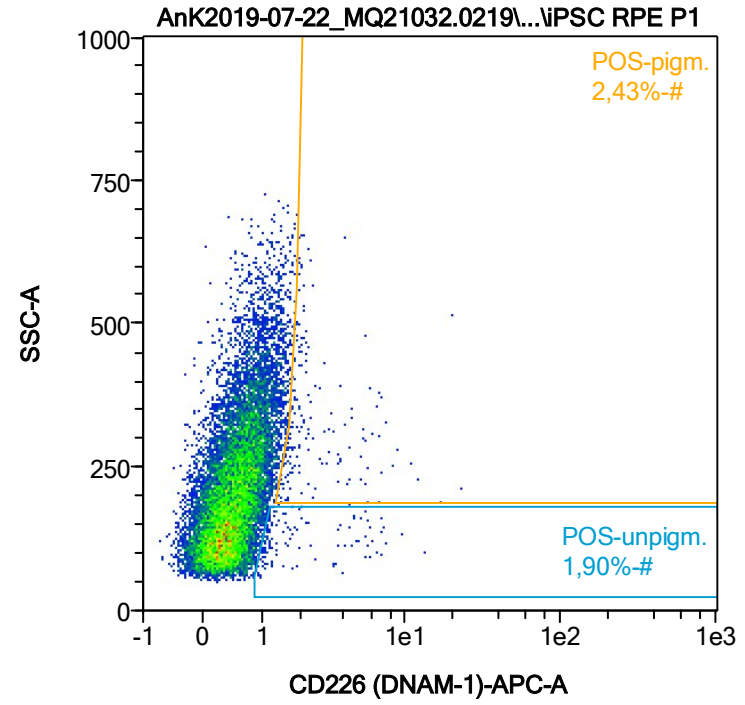

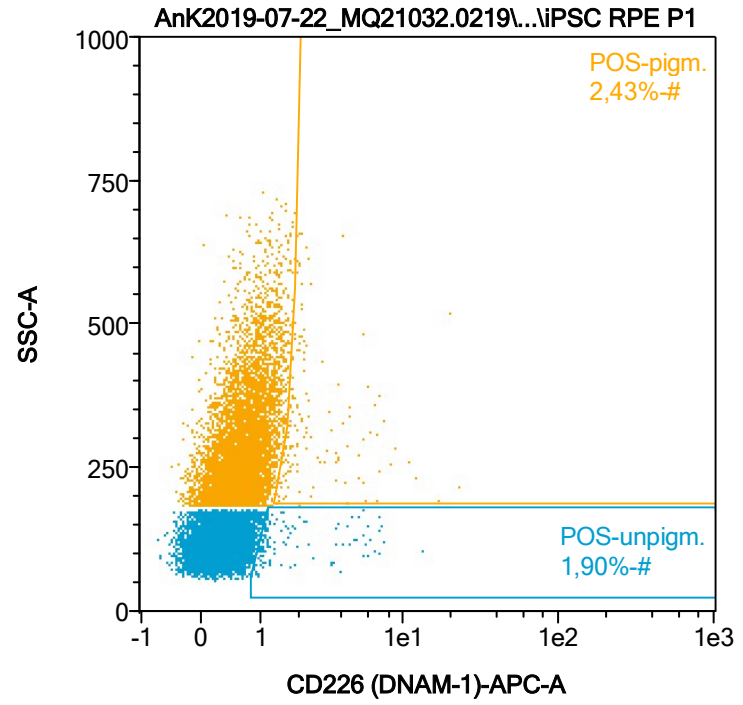

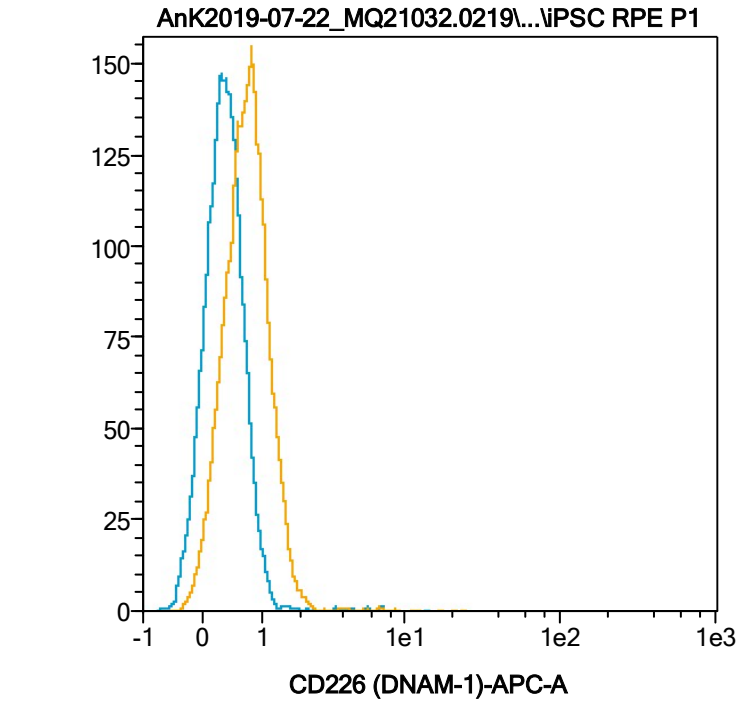

Name	%-#	CD226 (DNAM-1)-APC-A Mean	CD226 (DNAM-1)-APC-A Median
POS-unpigm.	1,90	2,34	1,24
POS-pigm.	2,43	3,04	1,97

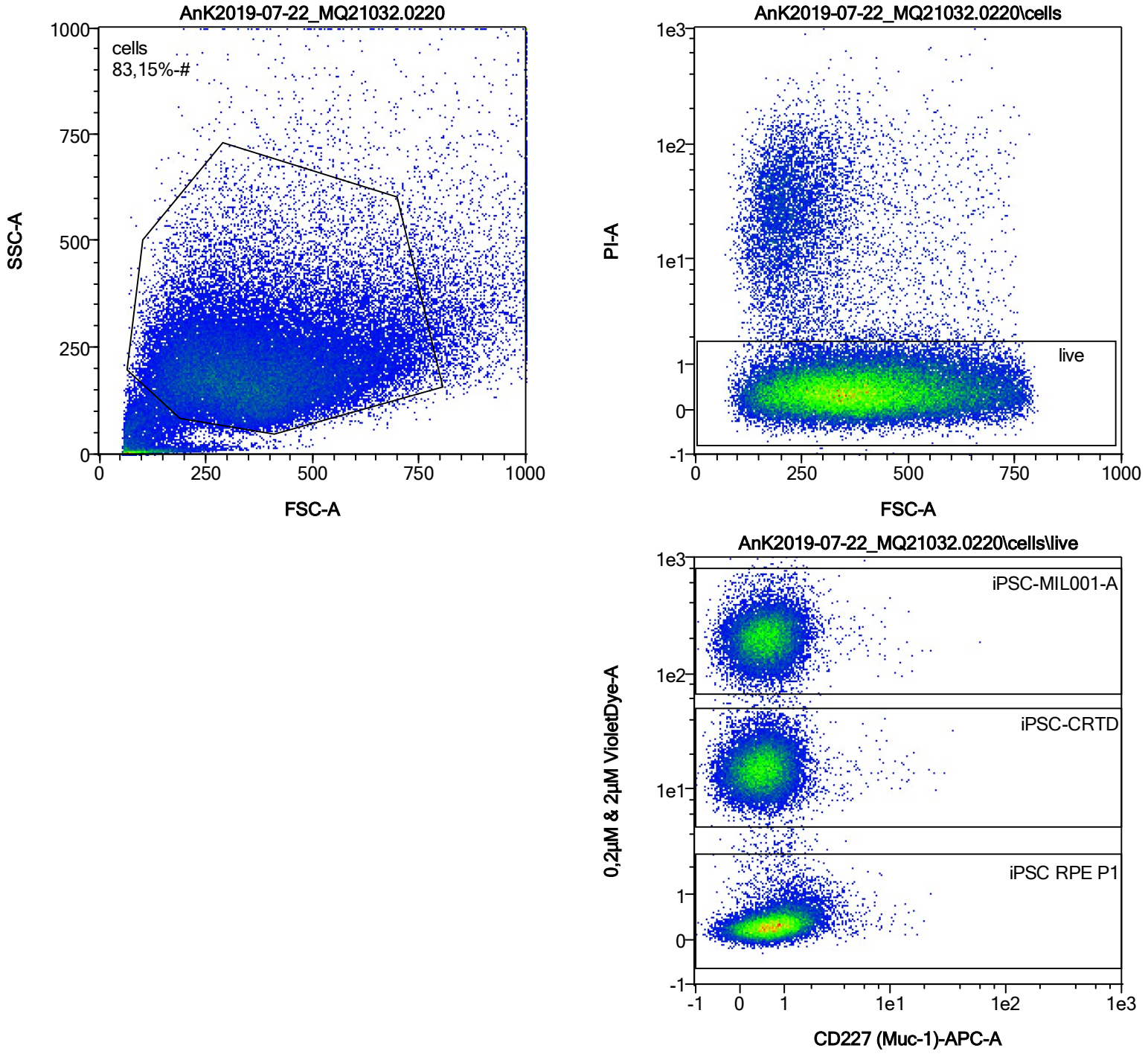

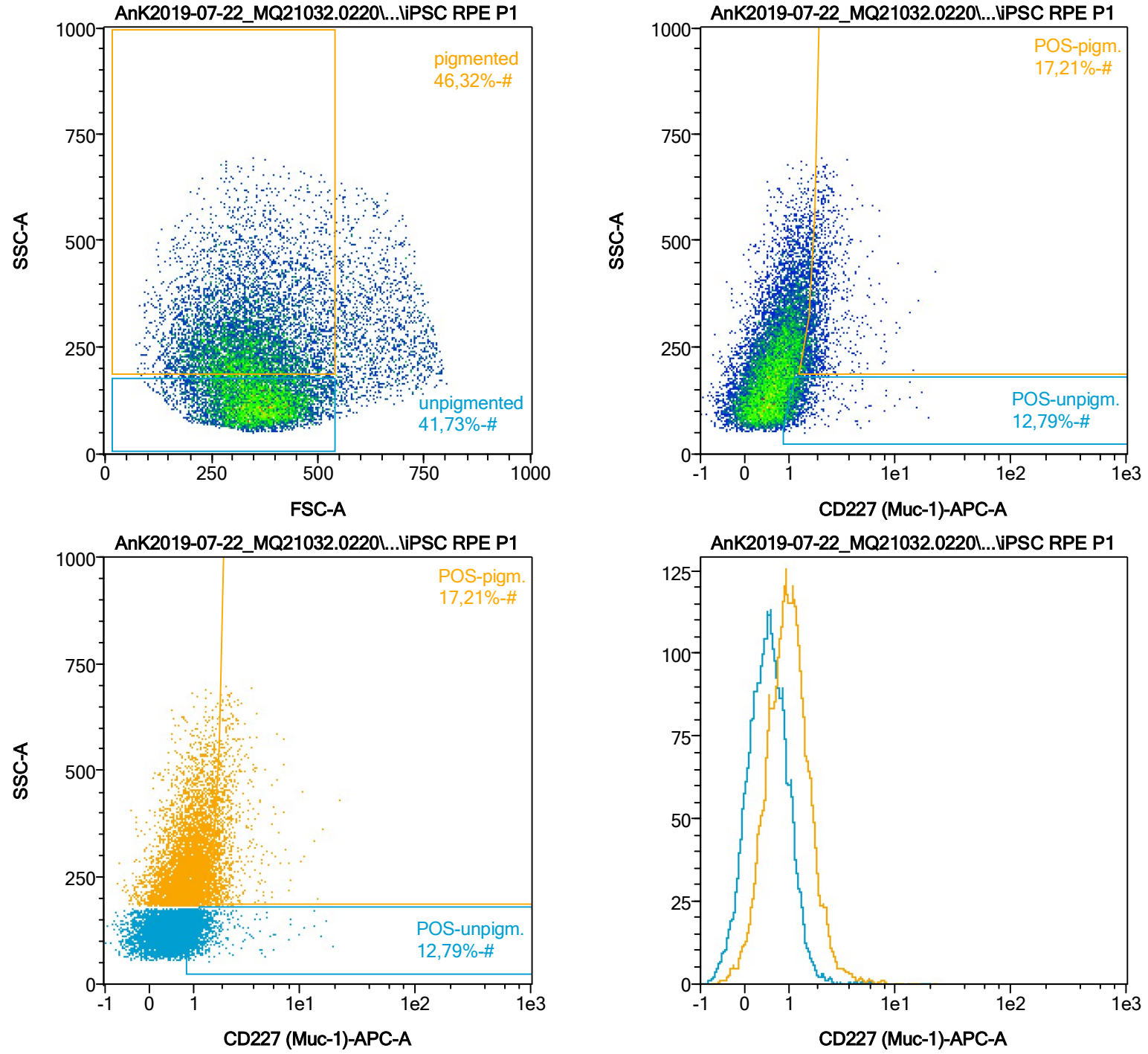

Name	%-#	CD227 (Muc-1)-APC-A Mean	CD227 (Muc-1)-APC-A Median
POS-unpigm.	12,79	1,55	1,24
POS-pigm.	17,21	2,23	1,92

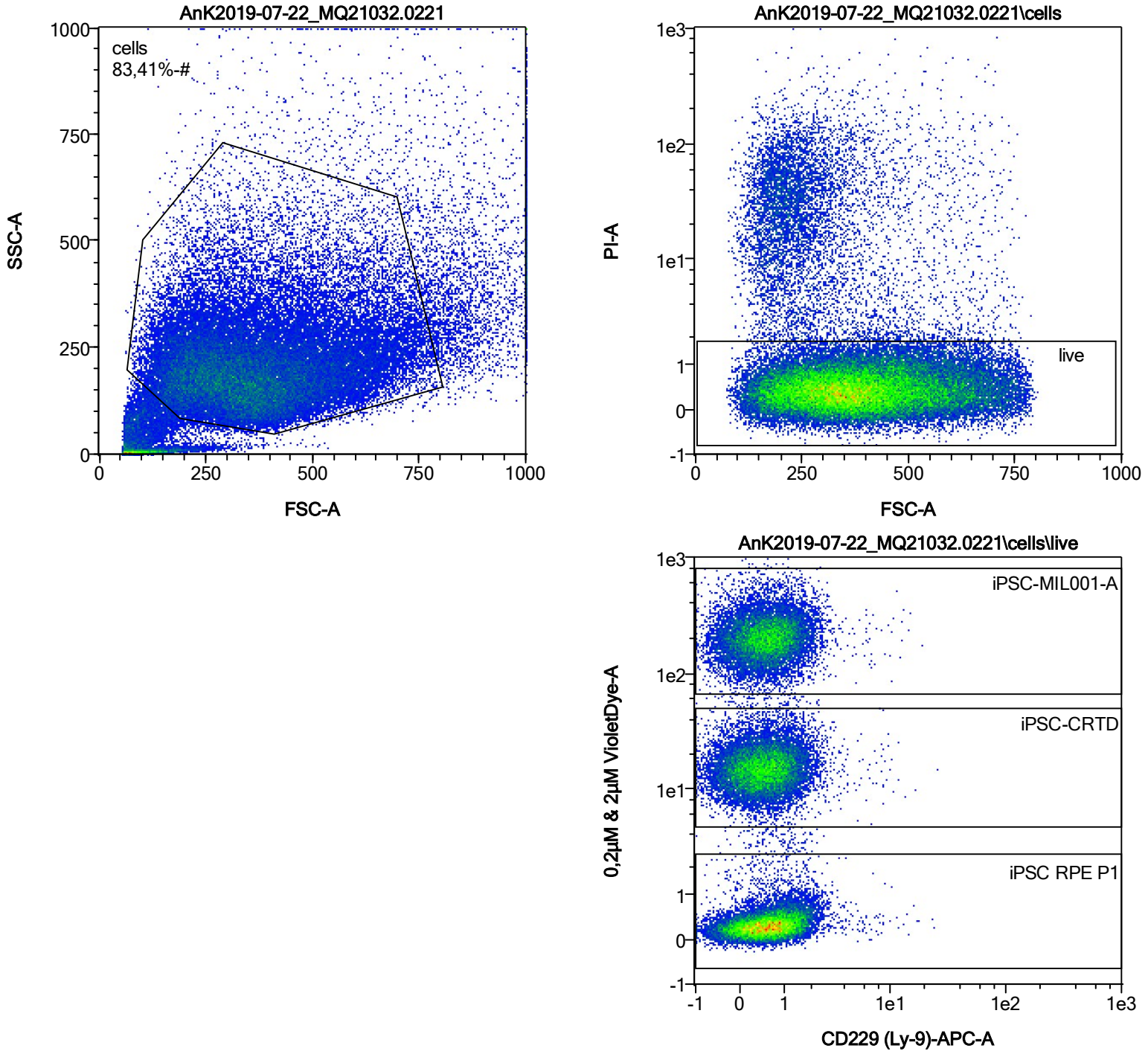

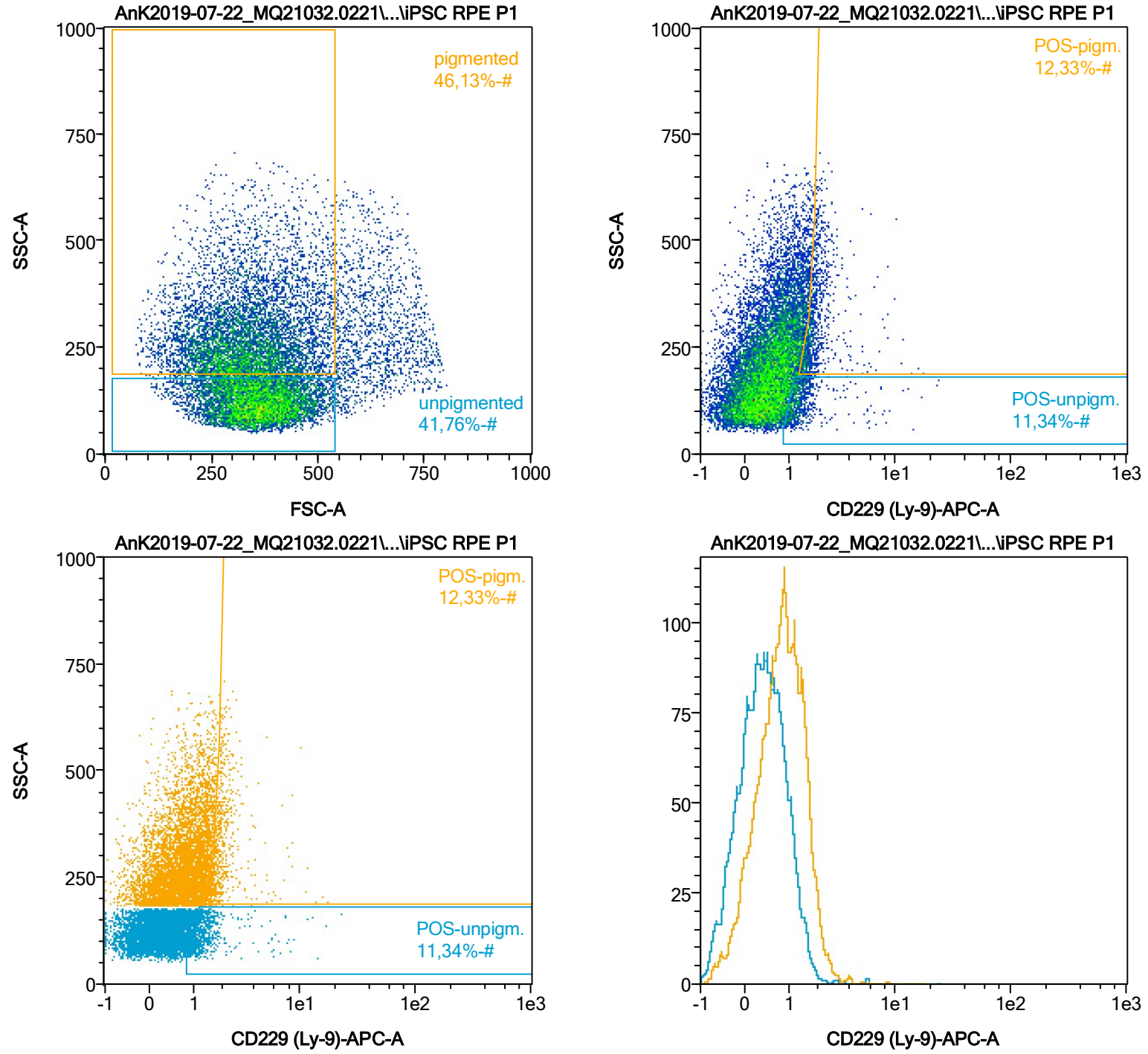

Name	%-#	CD229 (Ly-9)-APC-A Mean	CD229 (Ly-9)-APC-A Median
POS-unpigm.	11,34	1,57	1,24
POS-pigm.	12,33	2,08	1,81

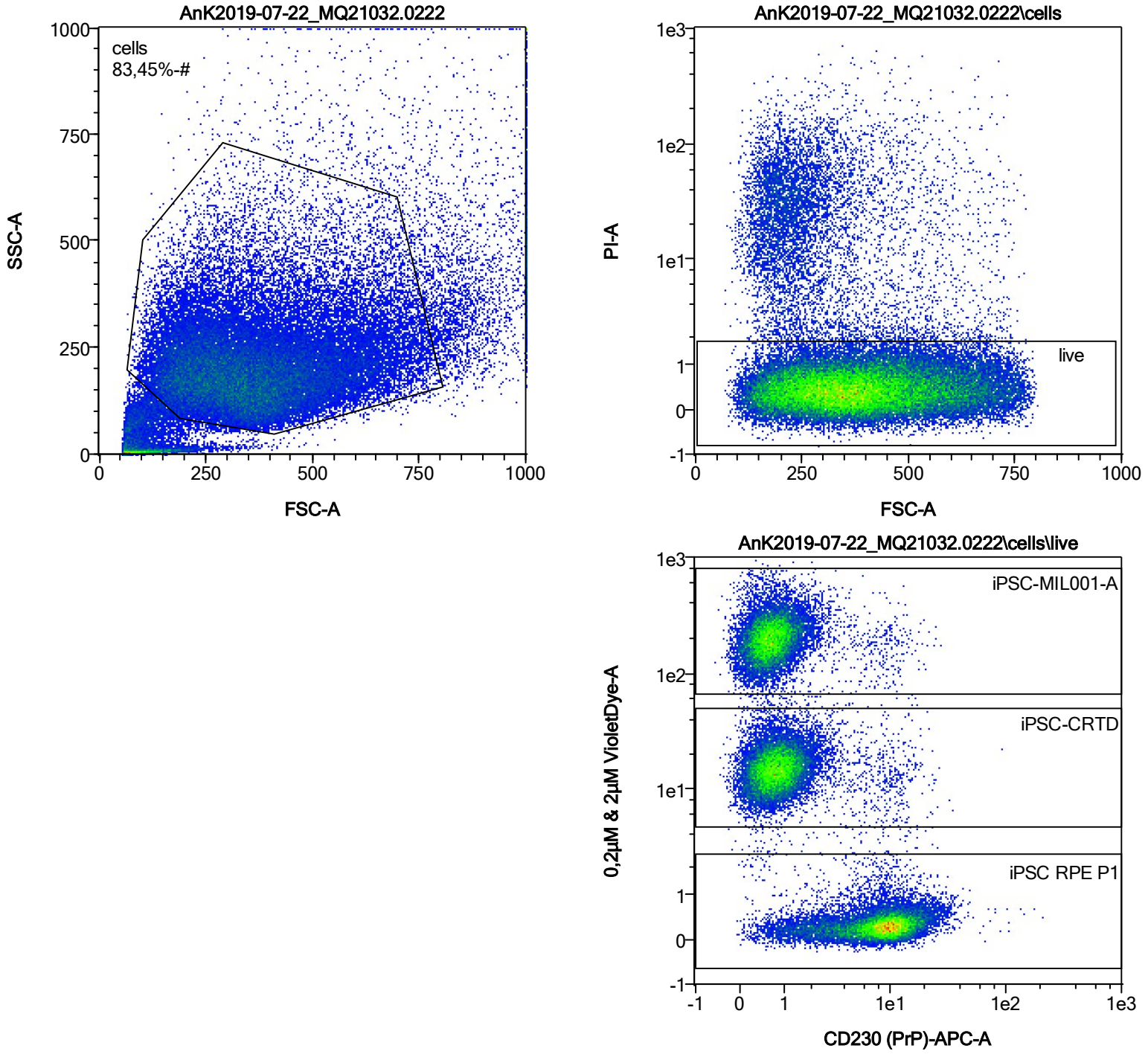

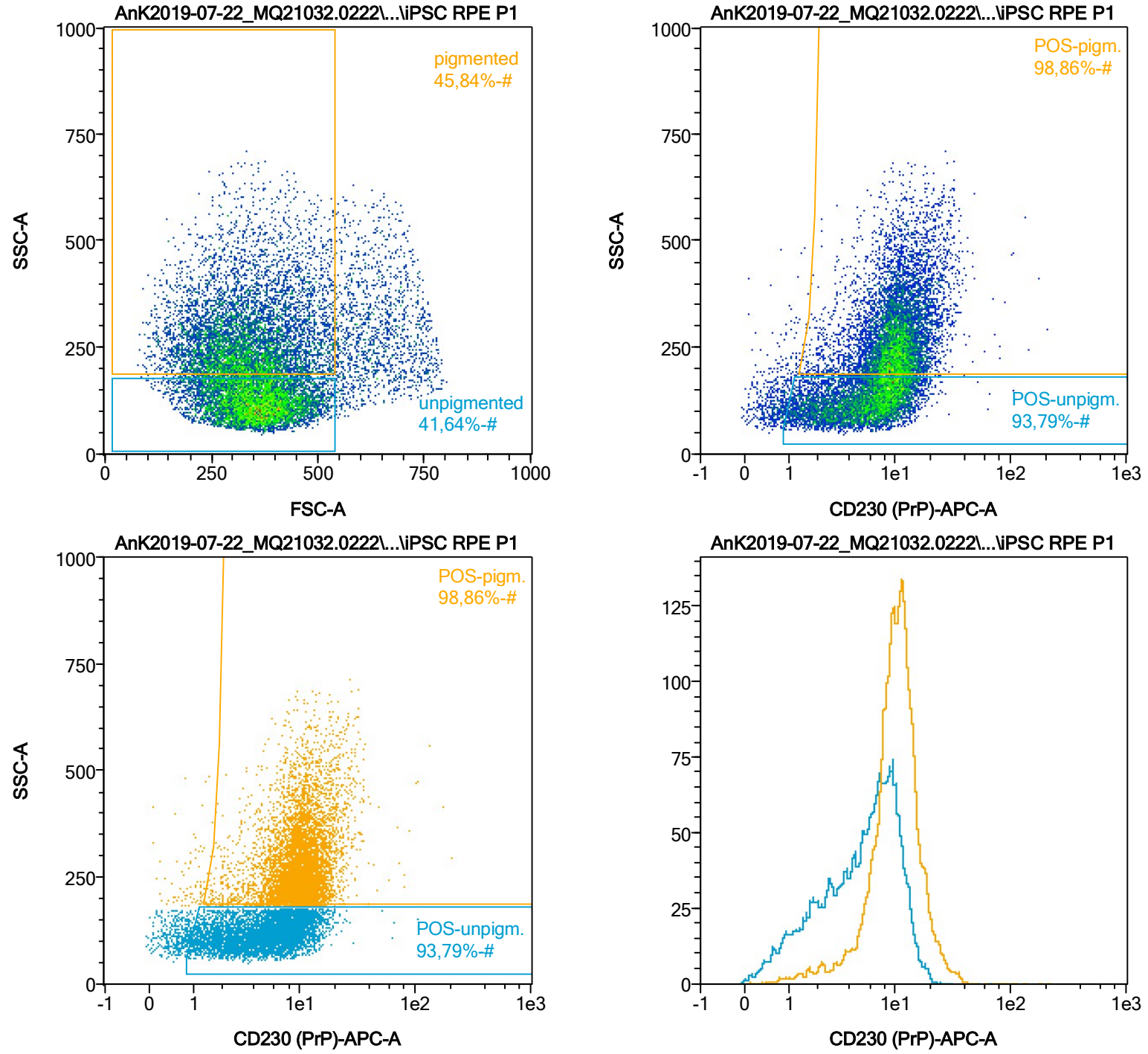

Name	%-#	CD230 (PrP)-APC-A Mean	CD230 (PrP)-APC-A Median
POS-unpigm.	93,79	6,43	5,95
POS-pigm.	98,86	11,10	10,24

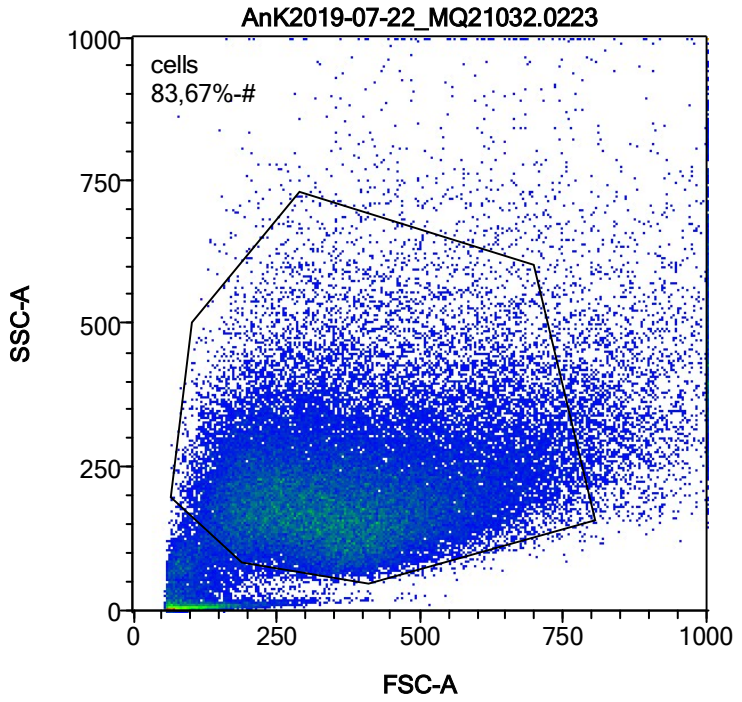

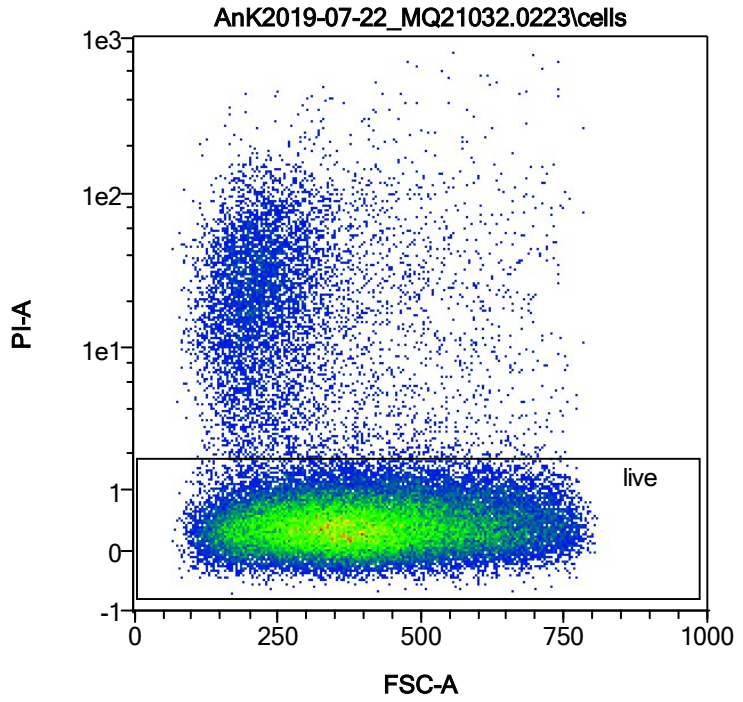

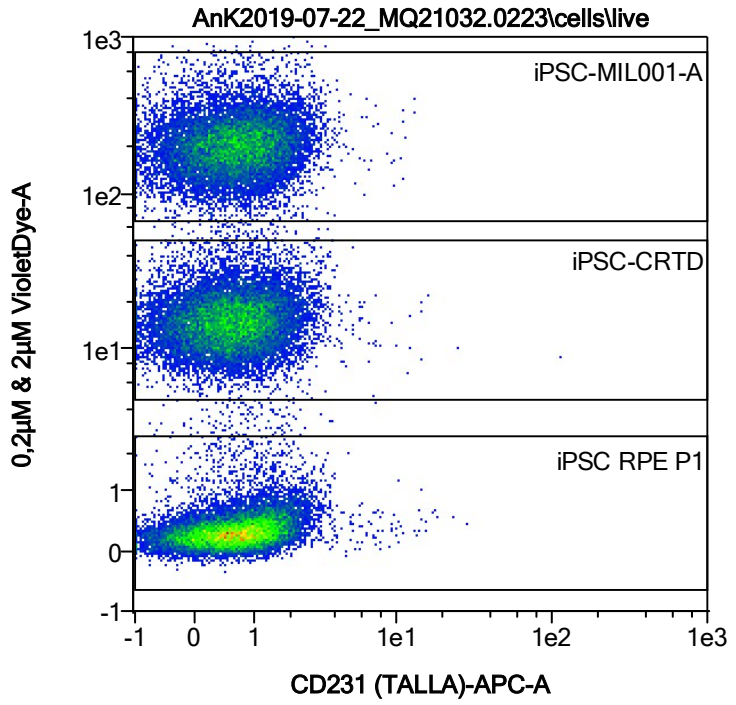

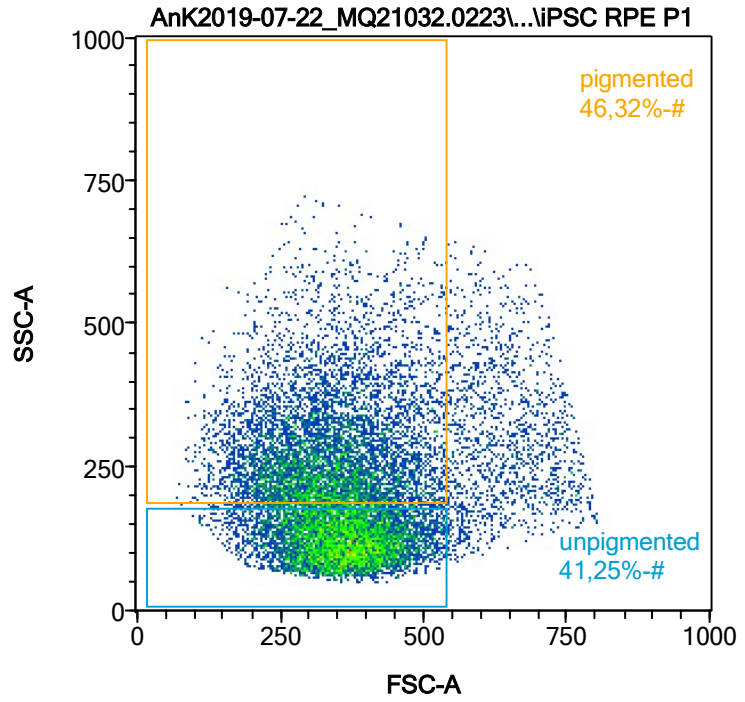

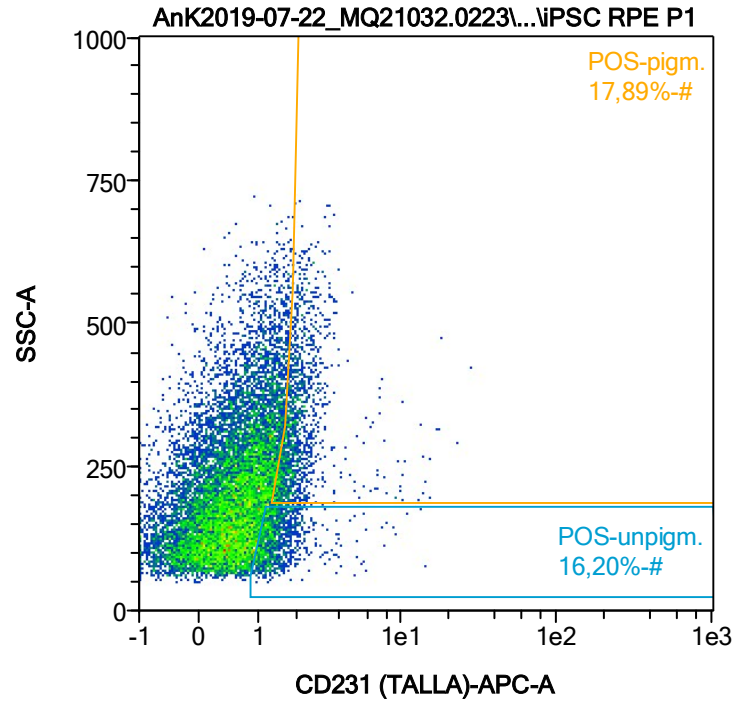

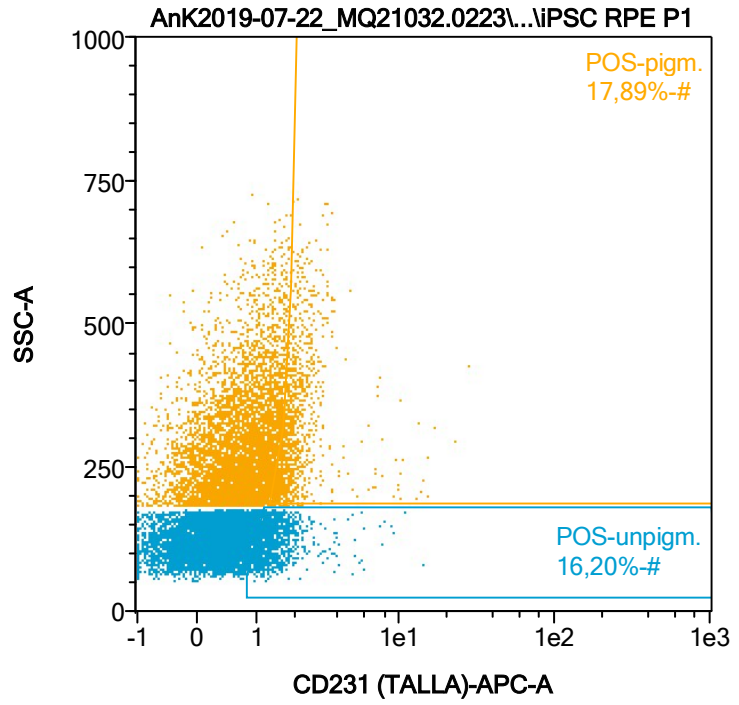

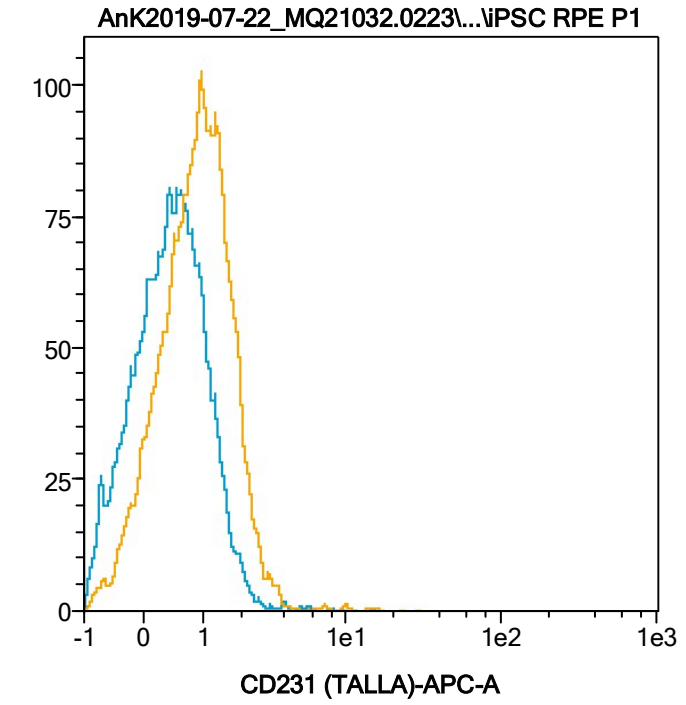

Name	%-#	CD231 (TALLA)-APC-A Mean	CD231 (TALLA)-APC-A Median
POS-unpigm.	16,20	1,53	1,30
POS-pigm.	17,89	2,22	1,90

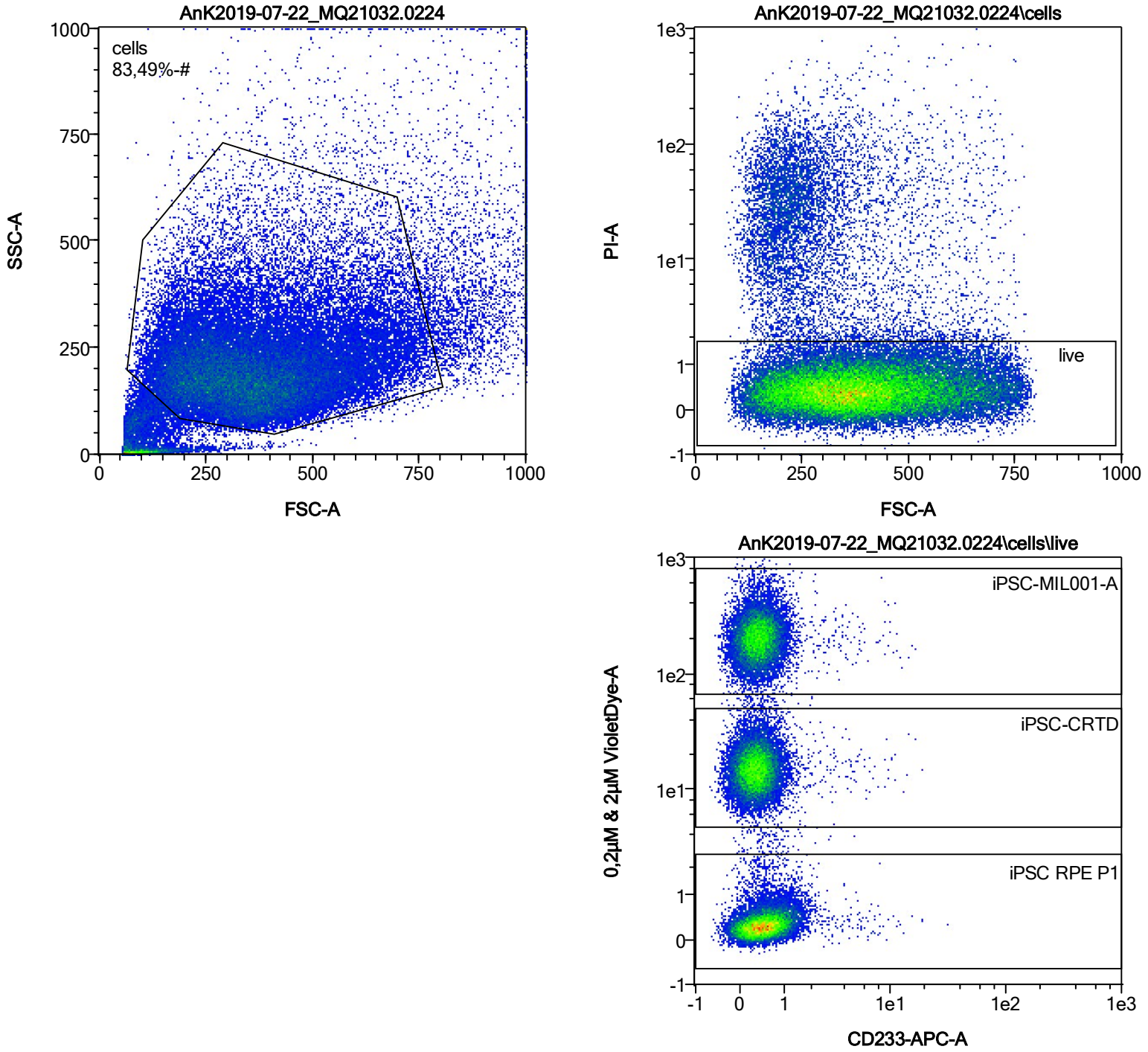

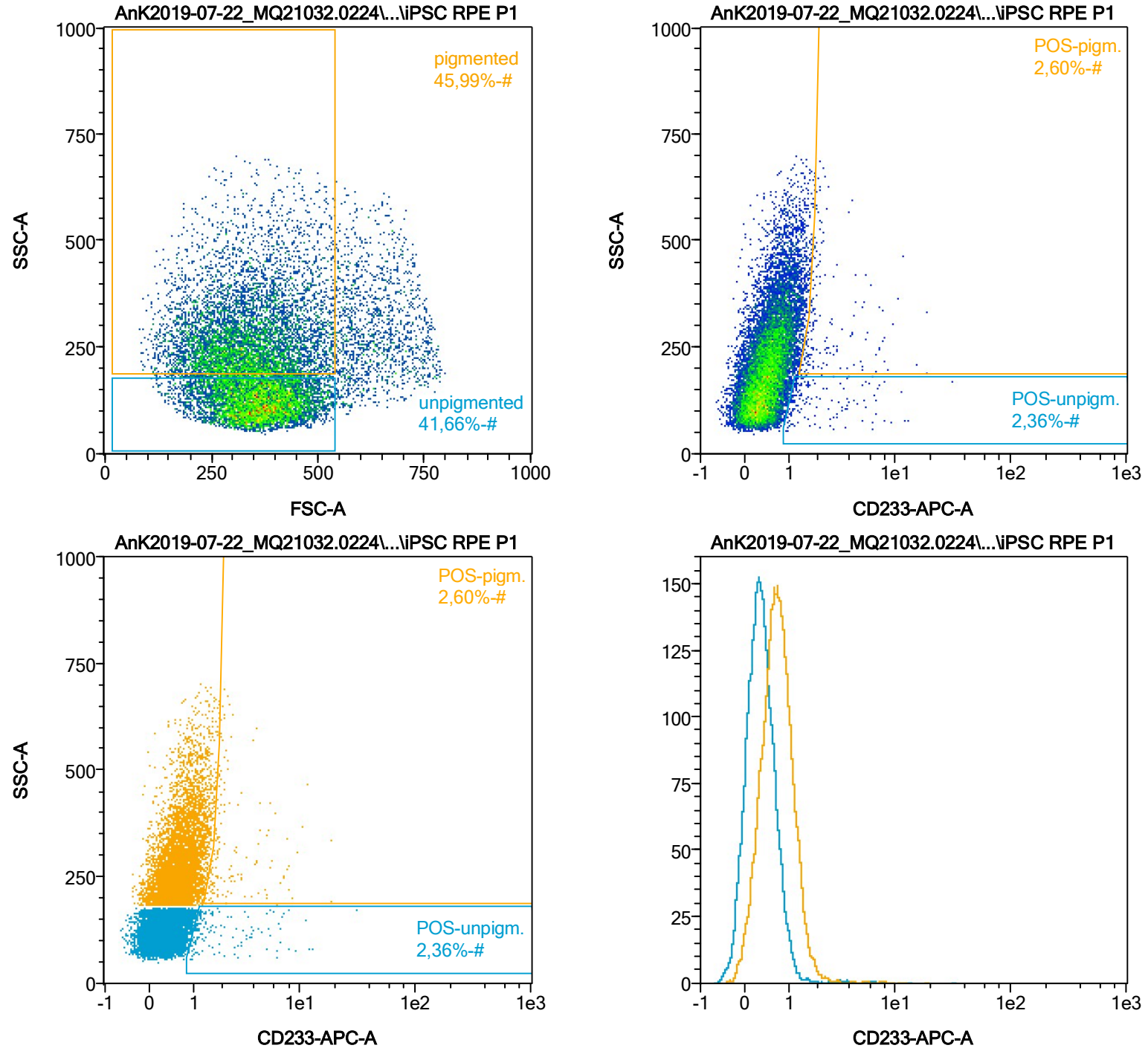

Name	%-#	CD233-APC-A Mean	CD233-APC-A Median
POS-unpigm.	2,36	2,69	1,38
POS-pigm.	2,60	2,90	1,94

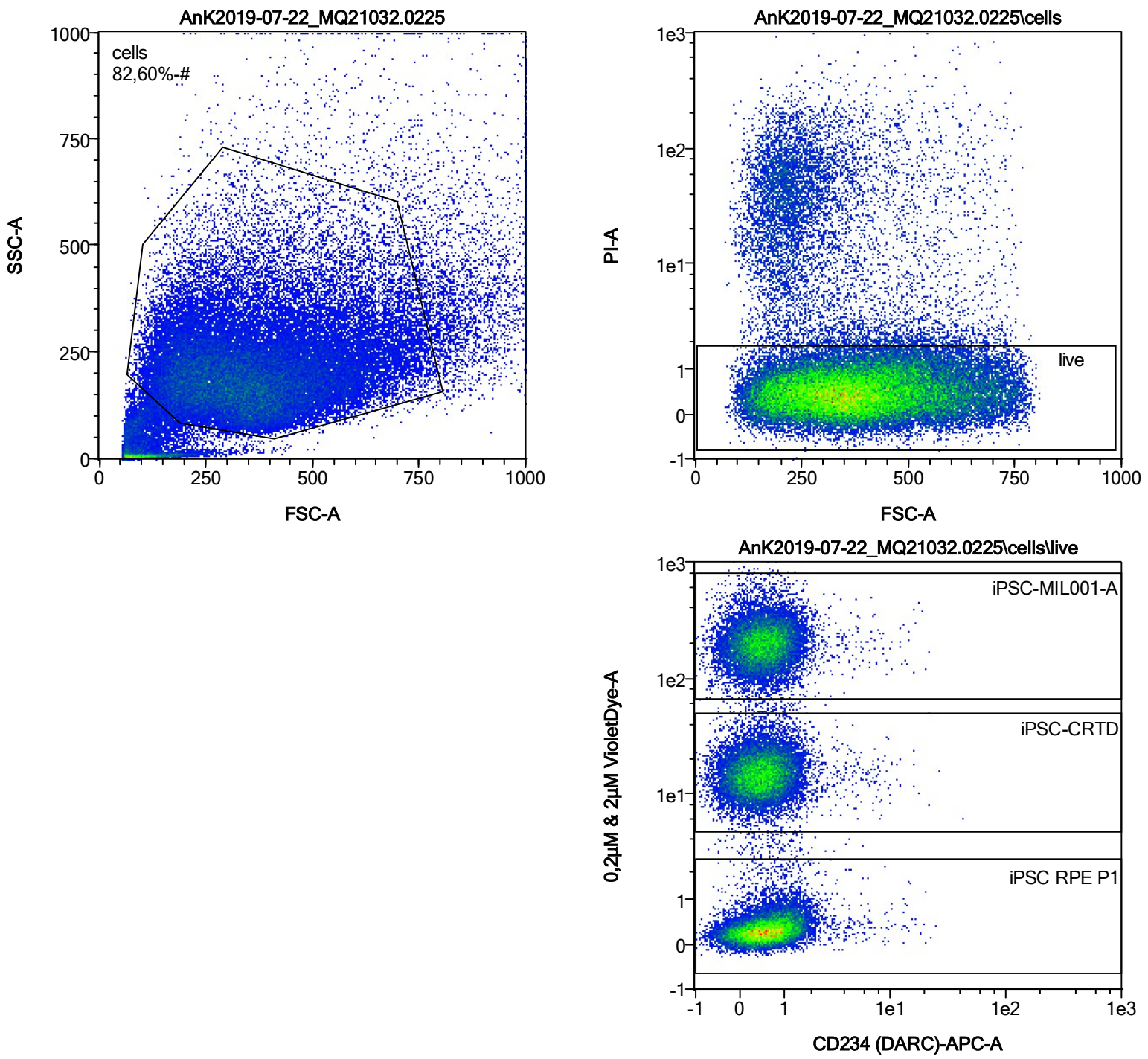

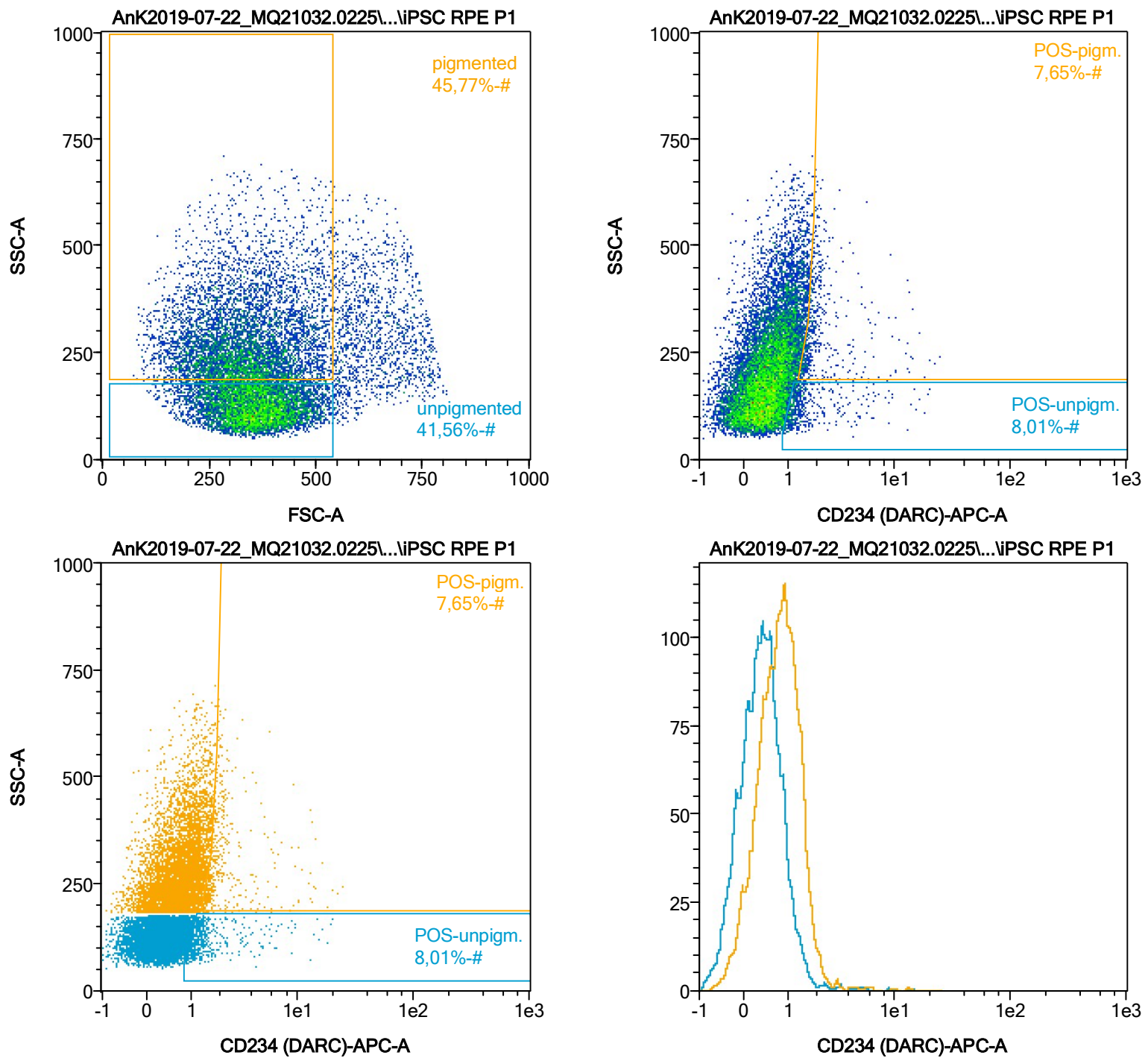

Name	%-#	CD234 (DARC)-APC-A Mean	CD234 (DARC)-APC-A Median
POS-unpigm.	8,01	1,95	1,28
POS-pigm.	7,65	2,64	1,85

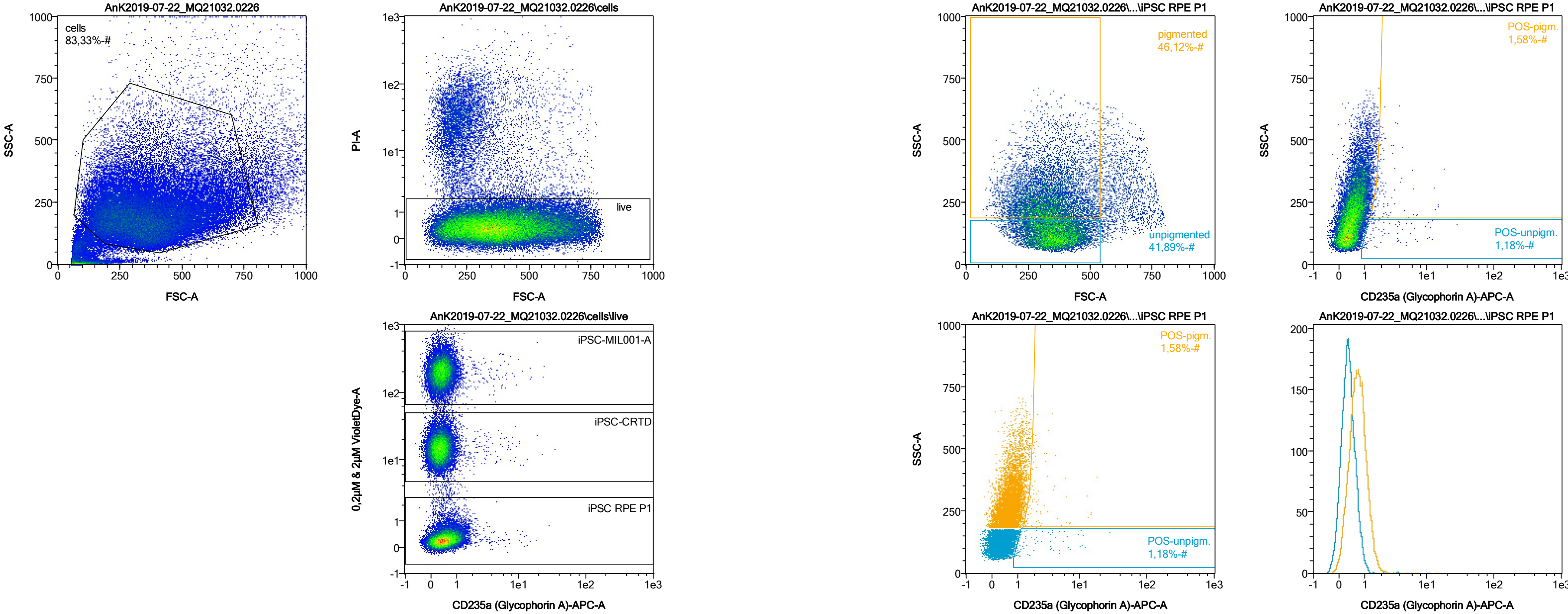

Name	%-#	CD235a (Glycophorin A)-APC-A Mean	CD235a (Glycophorin A)-APC-A Median
POS-unpigm.	1,18	3,32	1,49
POS-pigm.	1,58	2,89	1,94

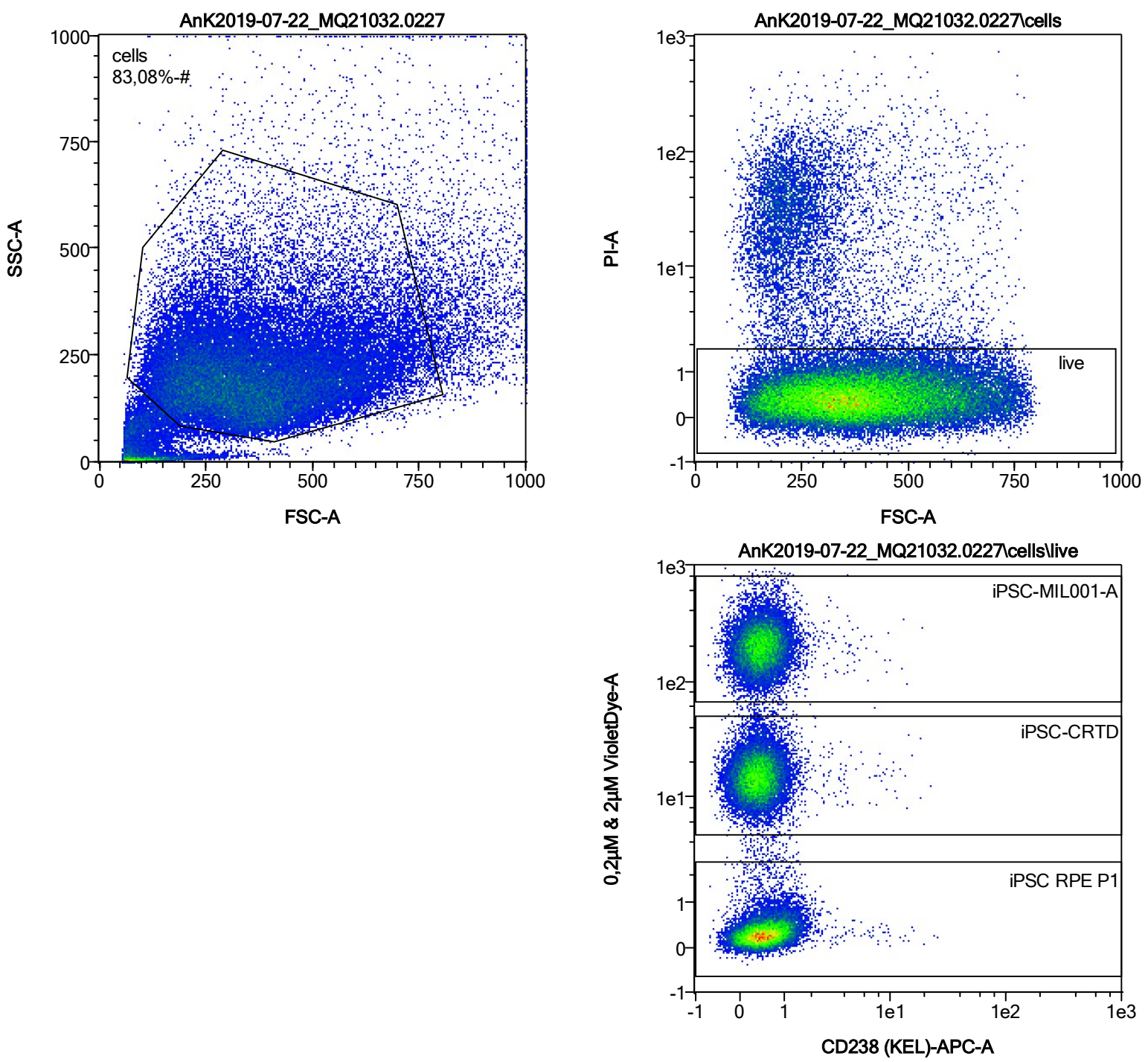

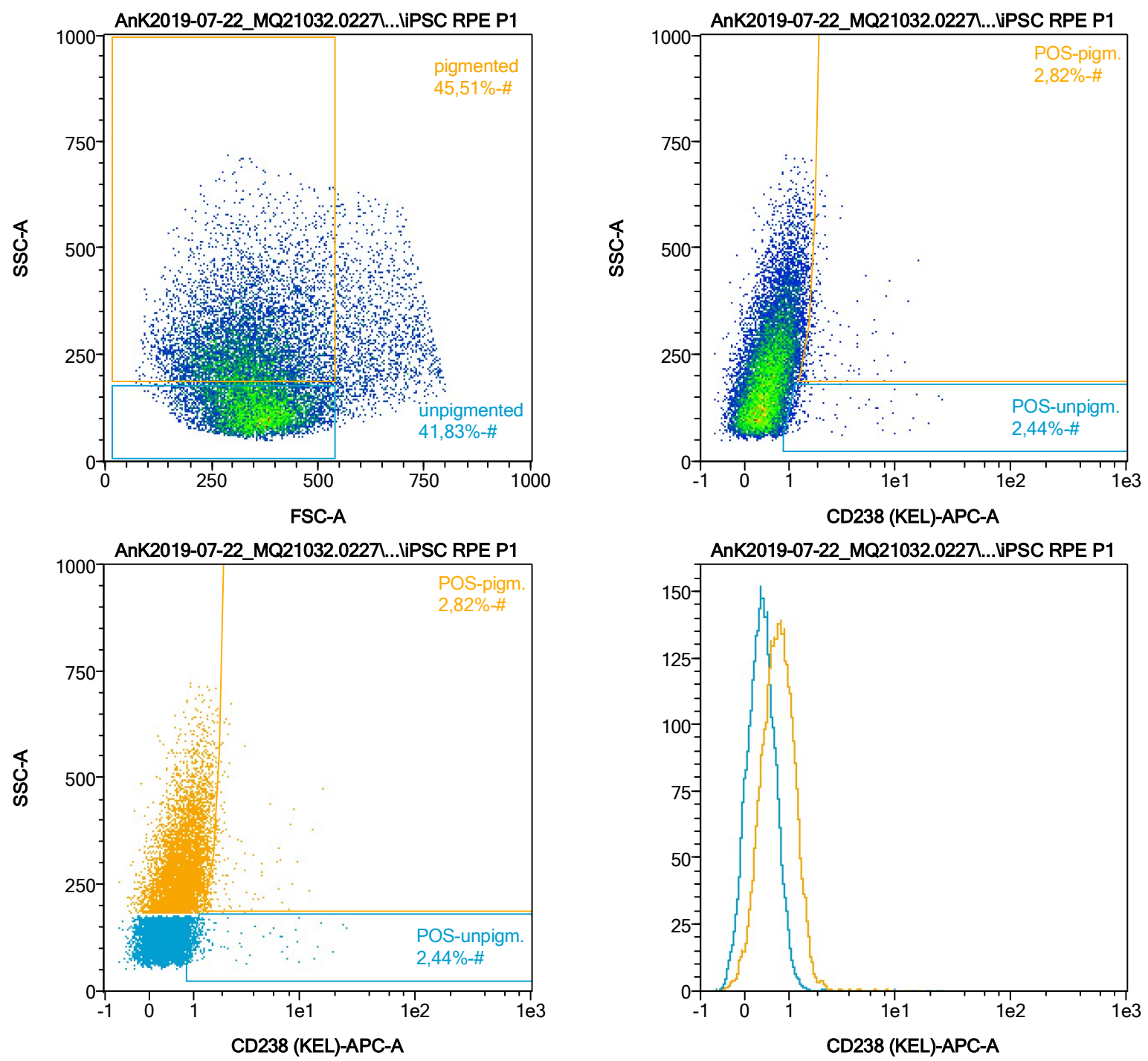

Name	%-#	CD238 (KEL)-APC-A Mean	CD238 (KEL)-APC-A Median
POS-unpigm.	2,44	2,53	1,21
POS-pigm.	2,82	2,49	1,81

Date:2024-06-14
Operator: Sebastian Knoebel
Version:2.13.0+54.g95abf0f4

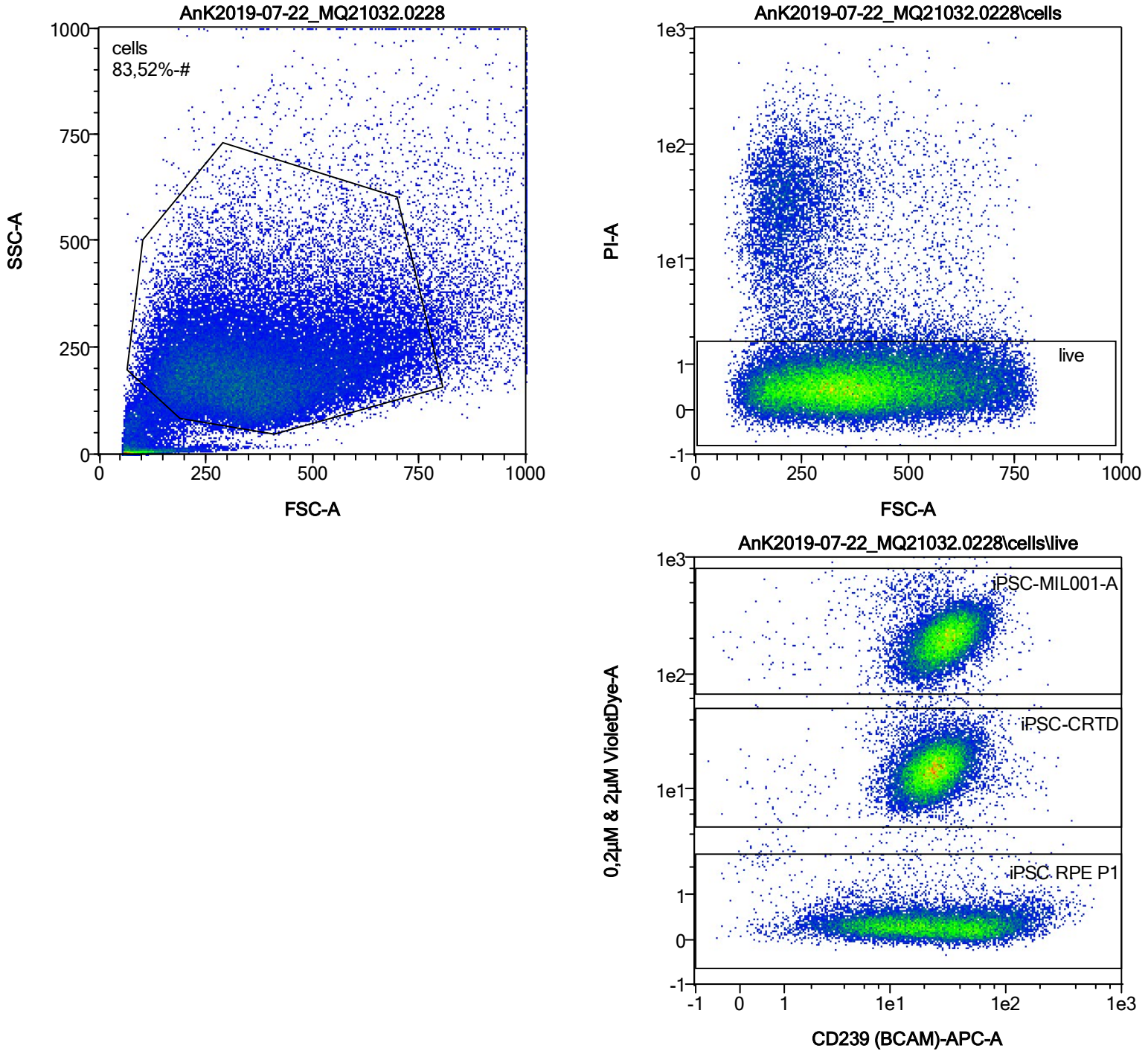

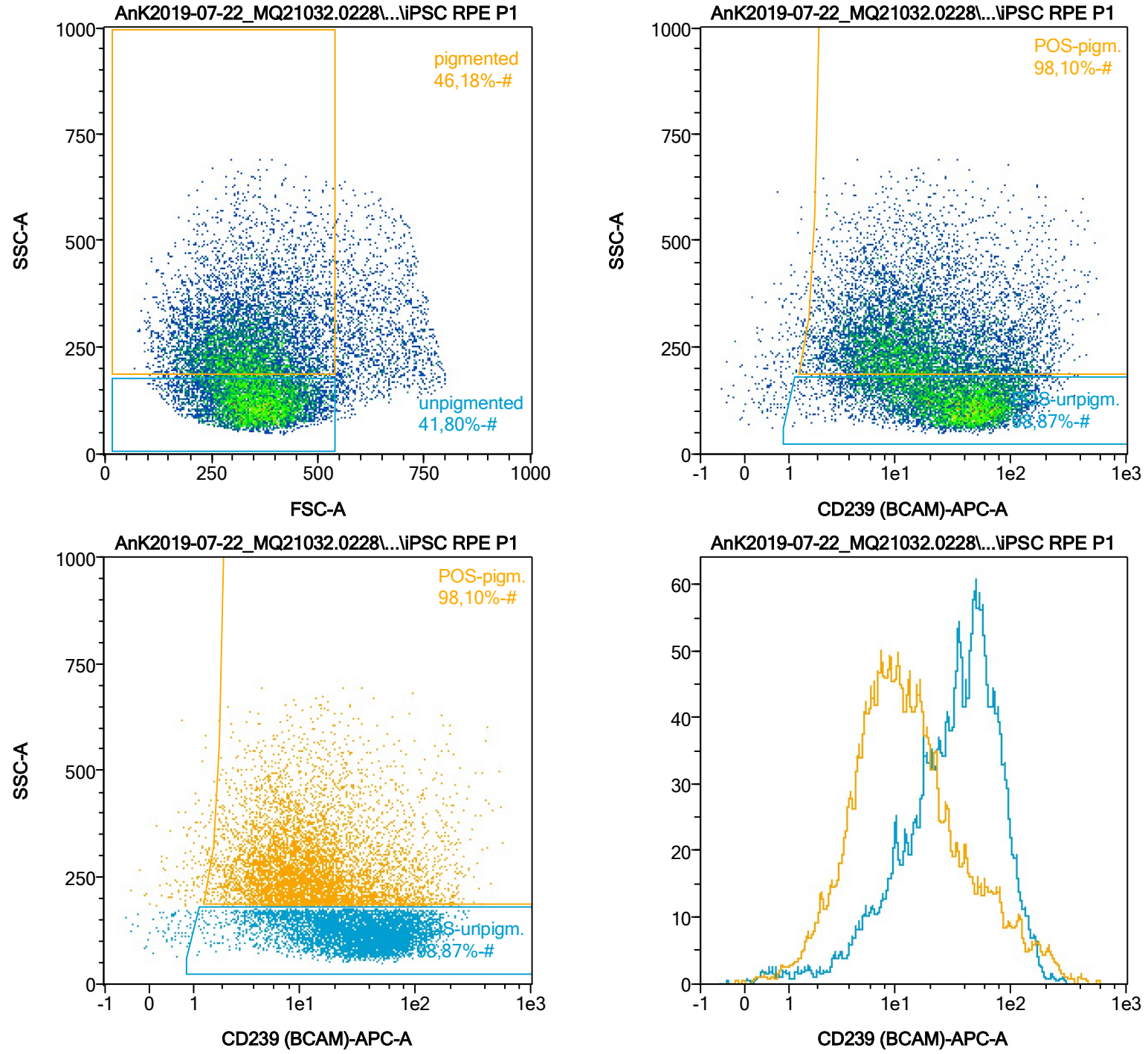

Name	%-#	CD239 (BCAM)-APC-A Mean	CD239 (BCAM)-APC-A Median
POS-unpigm.	98,87	43,32	35,65
POS-pigm.	98,10	26,11	11,83

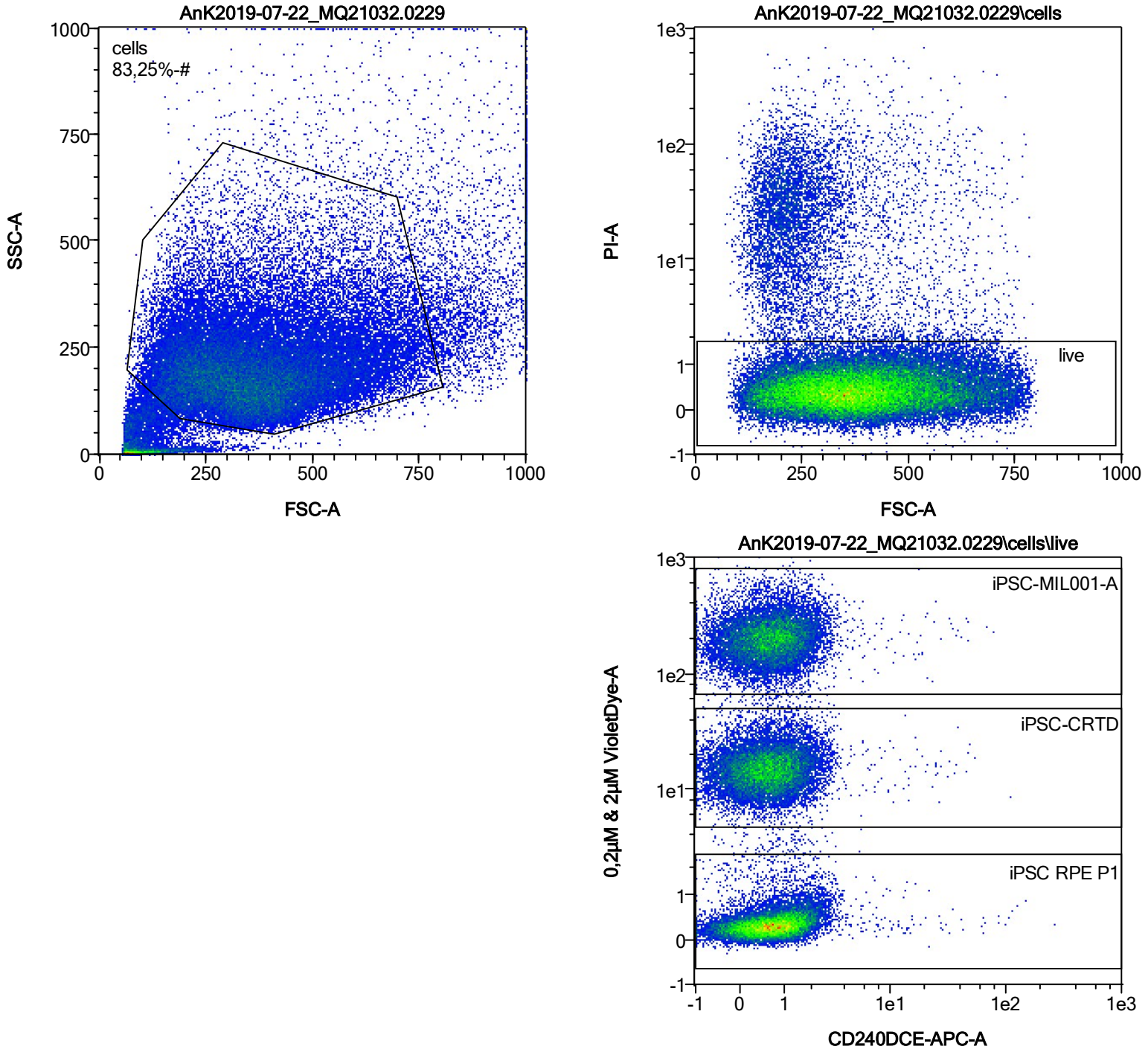

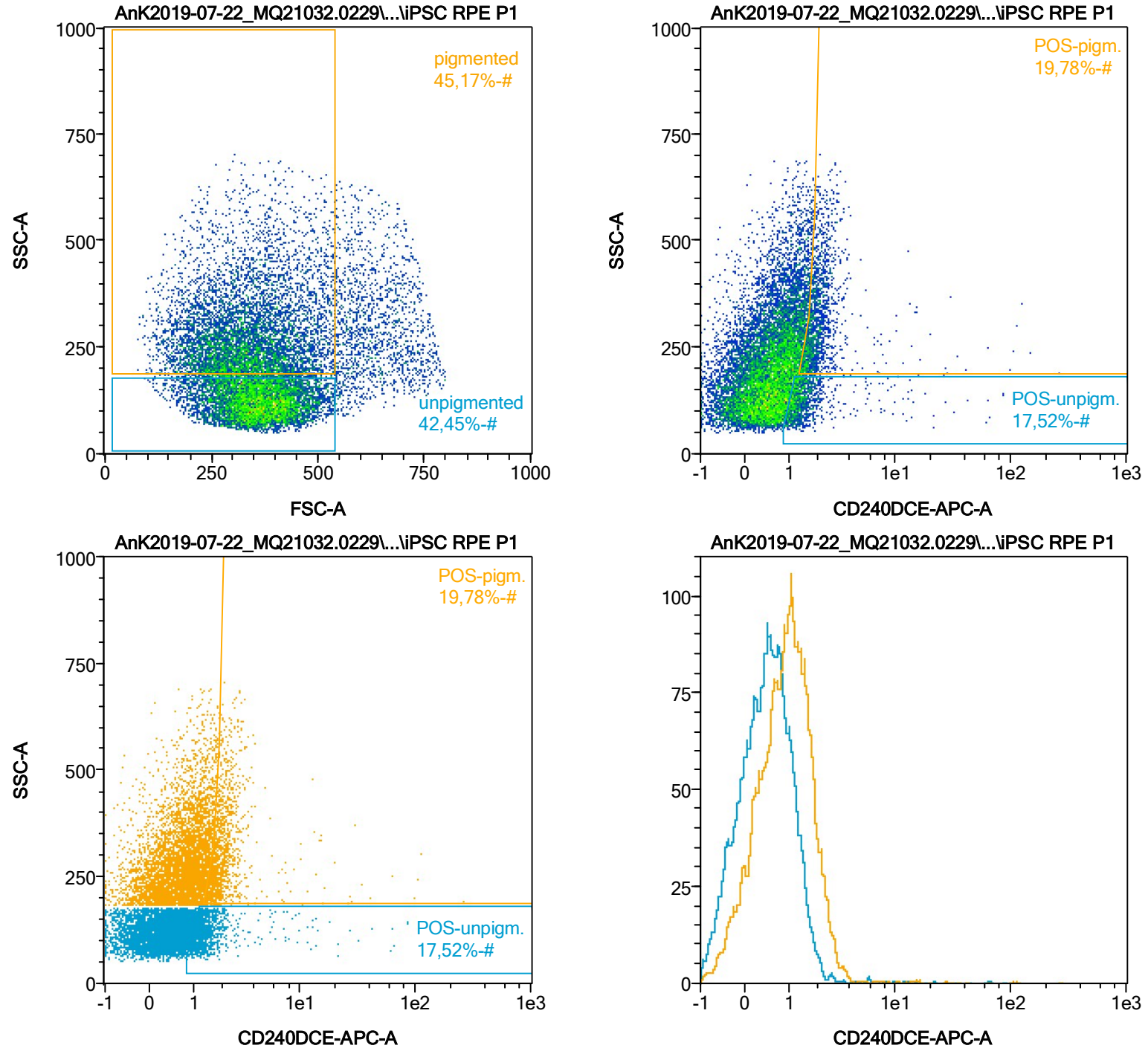

Name	%-#	CD240DCE-APC-A Mean	CD240DCE-APC-A Median
POS-unpigm.	17,52	2,04	1,30
POS-pigm.	19,78	2,78	1,90

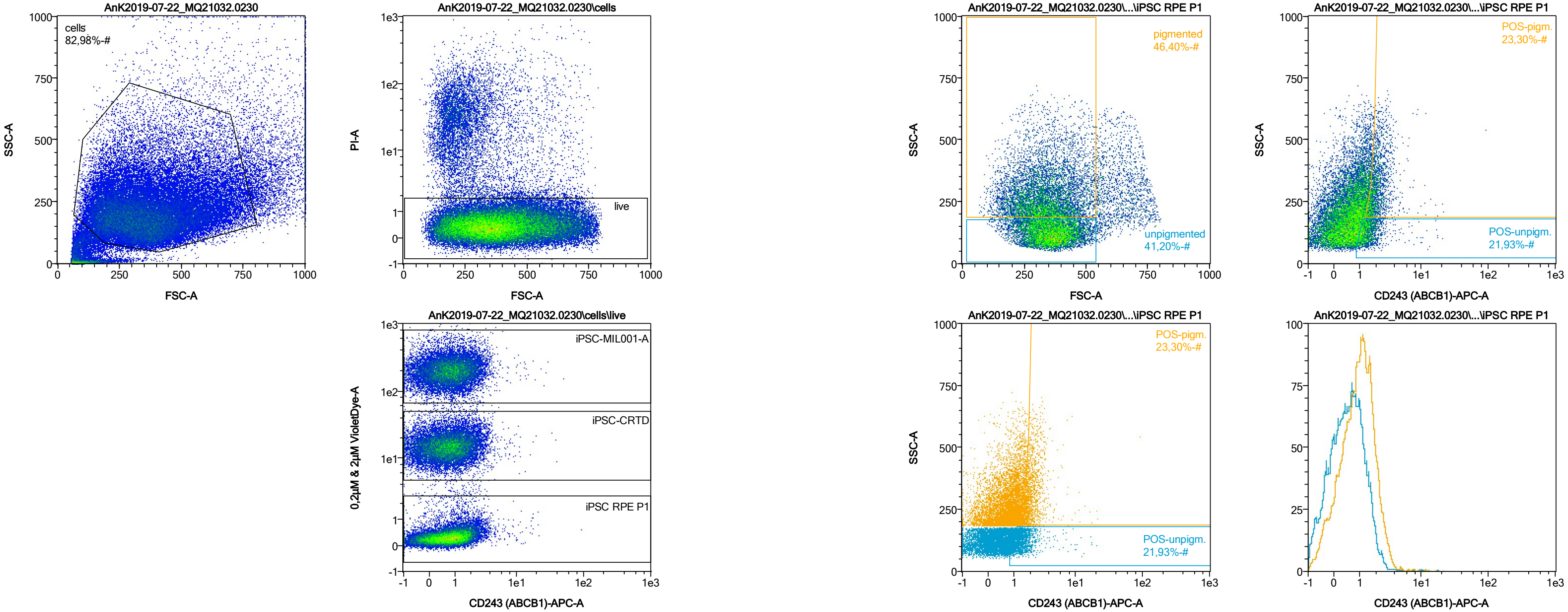

Name	%-#	CD243 (ABCB1)-APC-A Mean	CD243 (ABCB1)-APC-A Median
POS-unpigm.	21,93	1,63	1,37
POS-pigm.	23,30	2,26	1,95

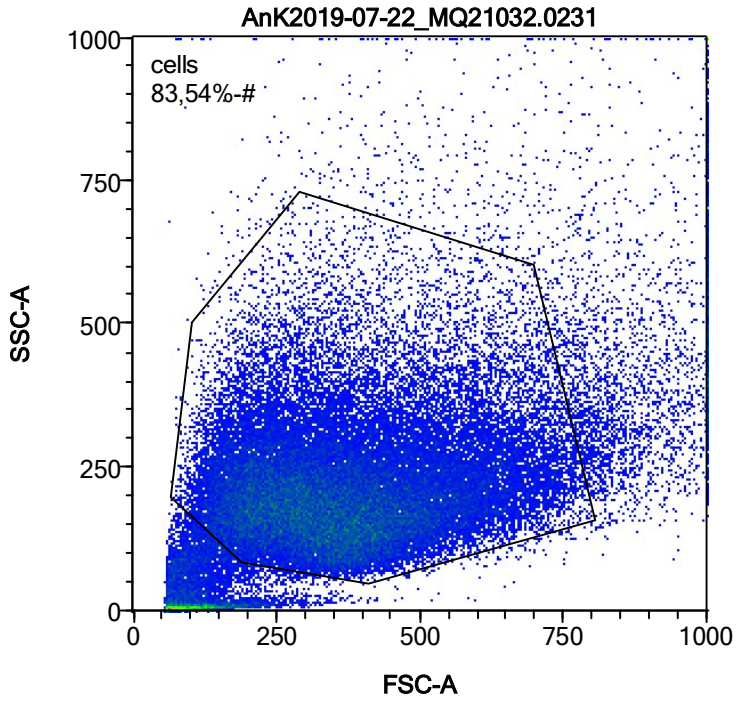

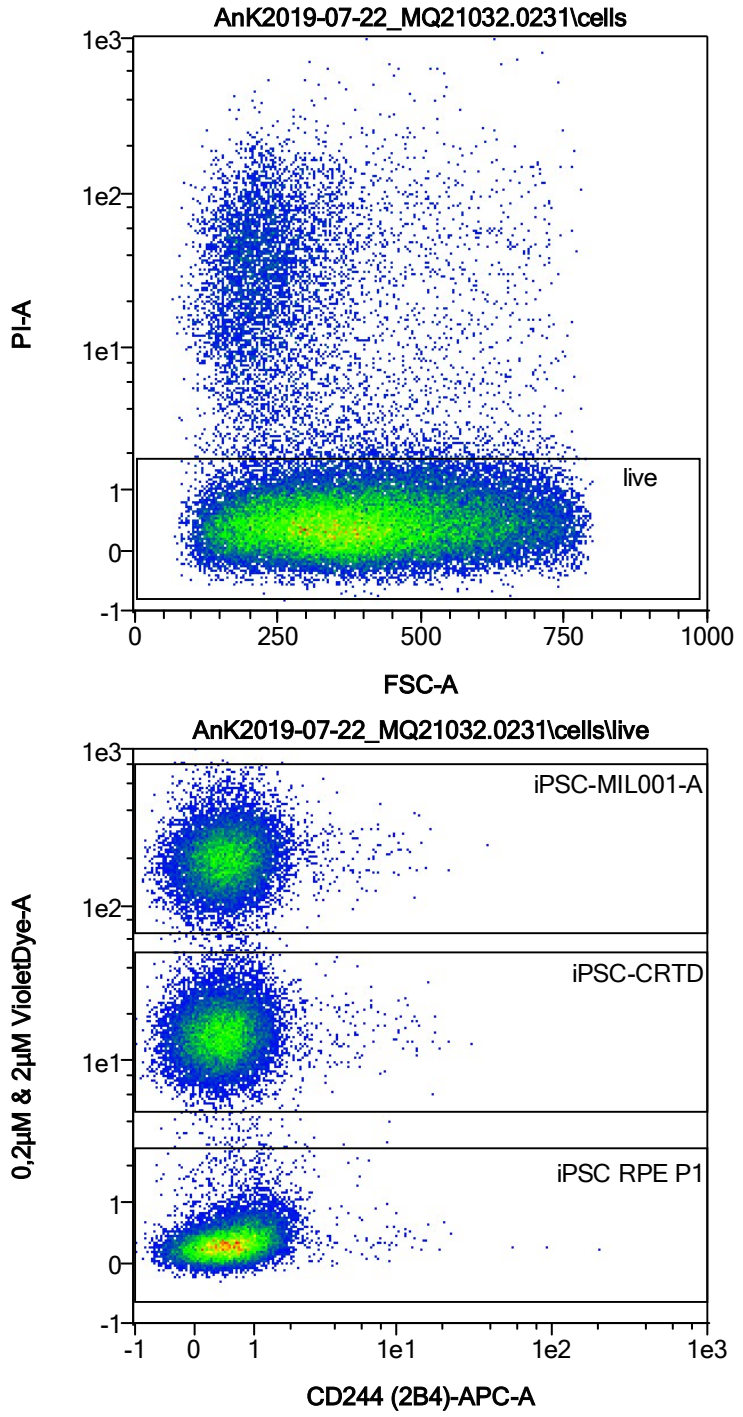

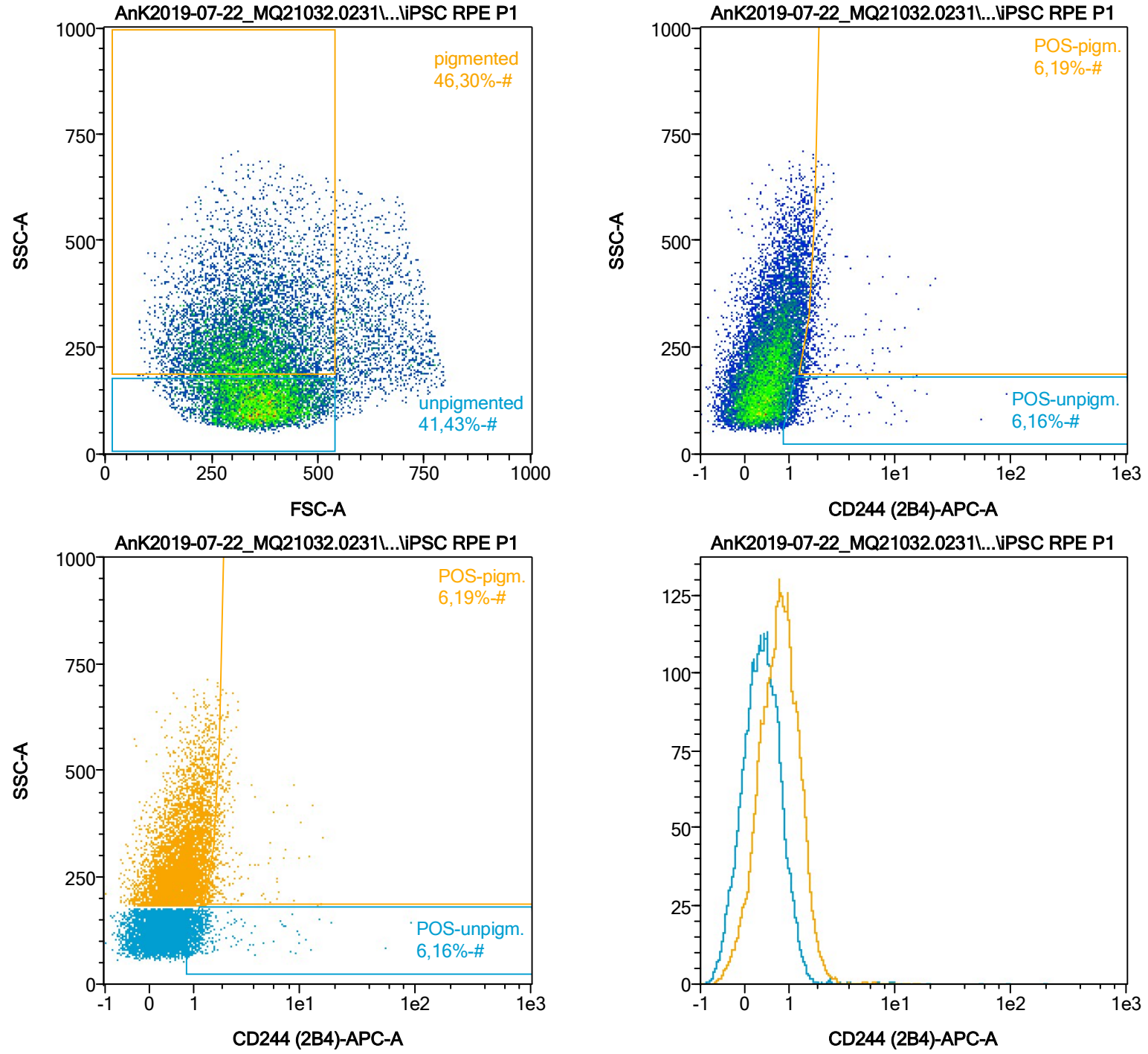

Name	%-#	CD244 (2B4)-APC-A Mean	CD244 (2B4)-APC-A Median
POS-unpigm.	6,16	2,59	1,20
POS-pigm.	6,19	2,26	1,85

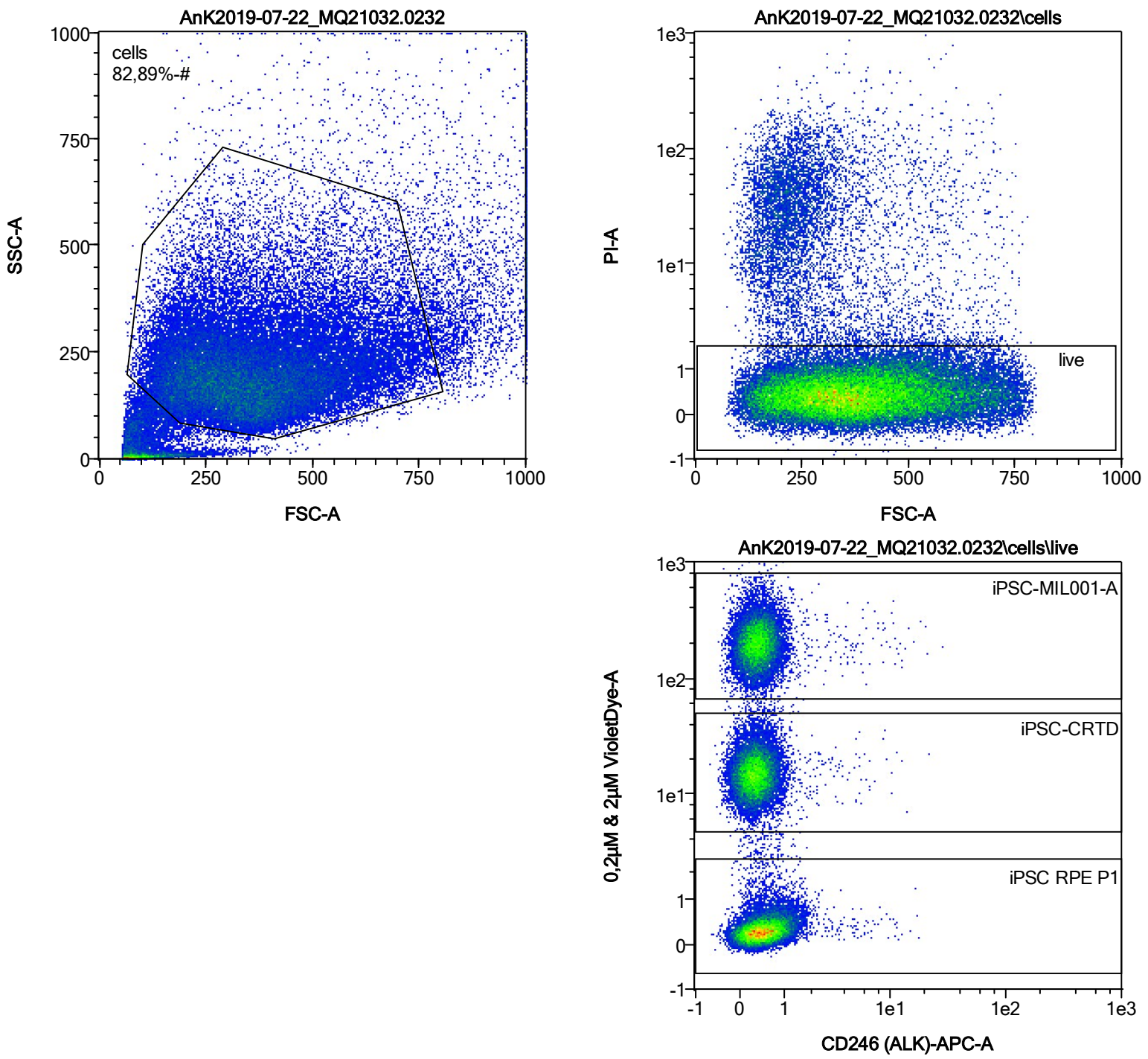

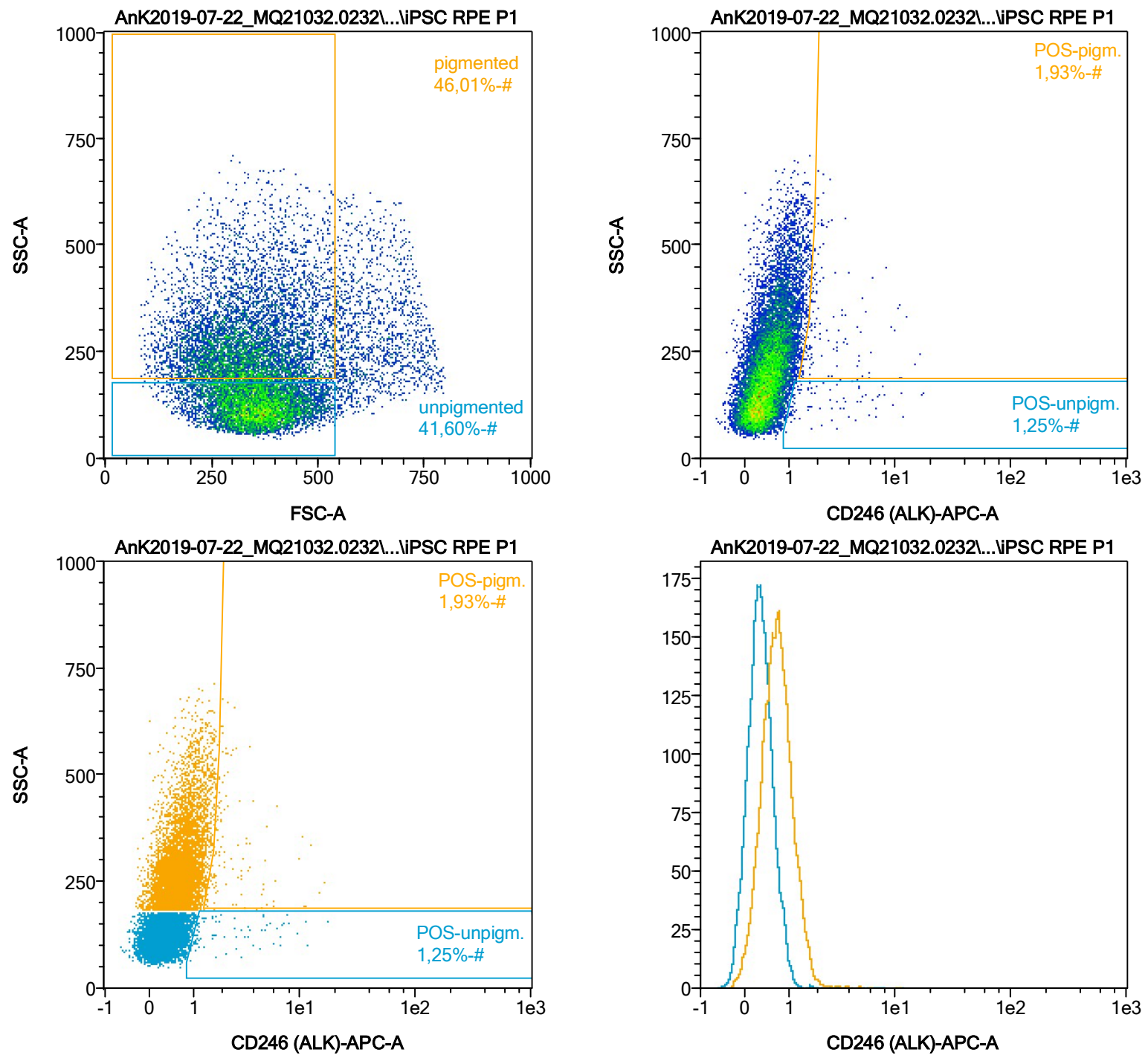

Name	%-#	CD246 (ALK)-APC-A Mean	CD246 (ALK)-APC-A Median
POS-unpigm.	1,25	3,13	1,73
POS-pigm.	1,93	2,93	1,98

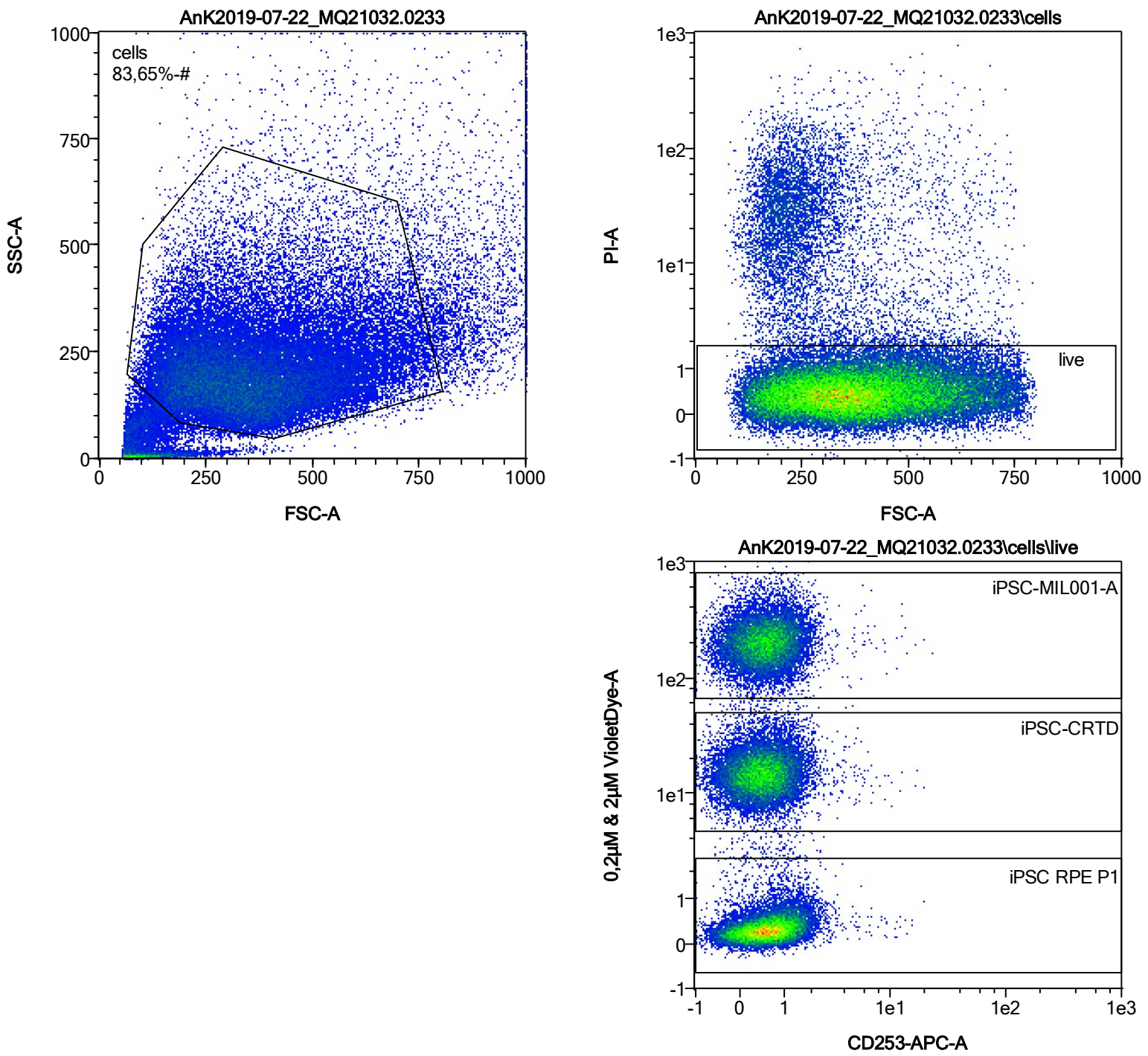

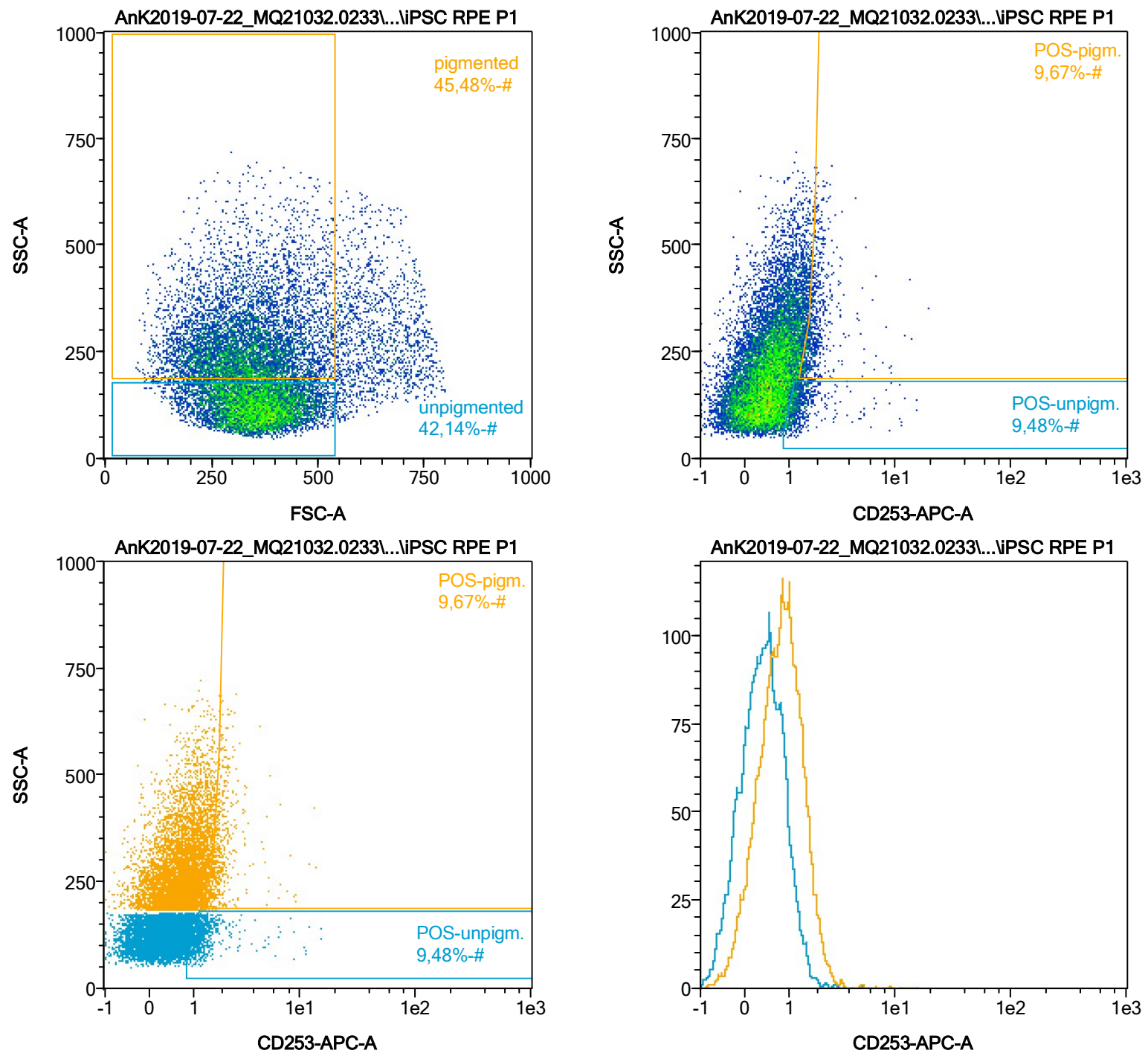

Name	%-#	CD253-APC-A Mean	CD253-APC-A Median
POS-unpigm.	9,48	1,55	1,23
POS-pigm.	9,67	2,06	1,83

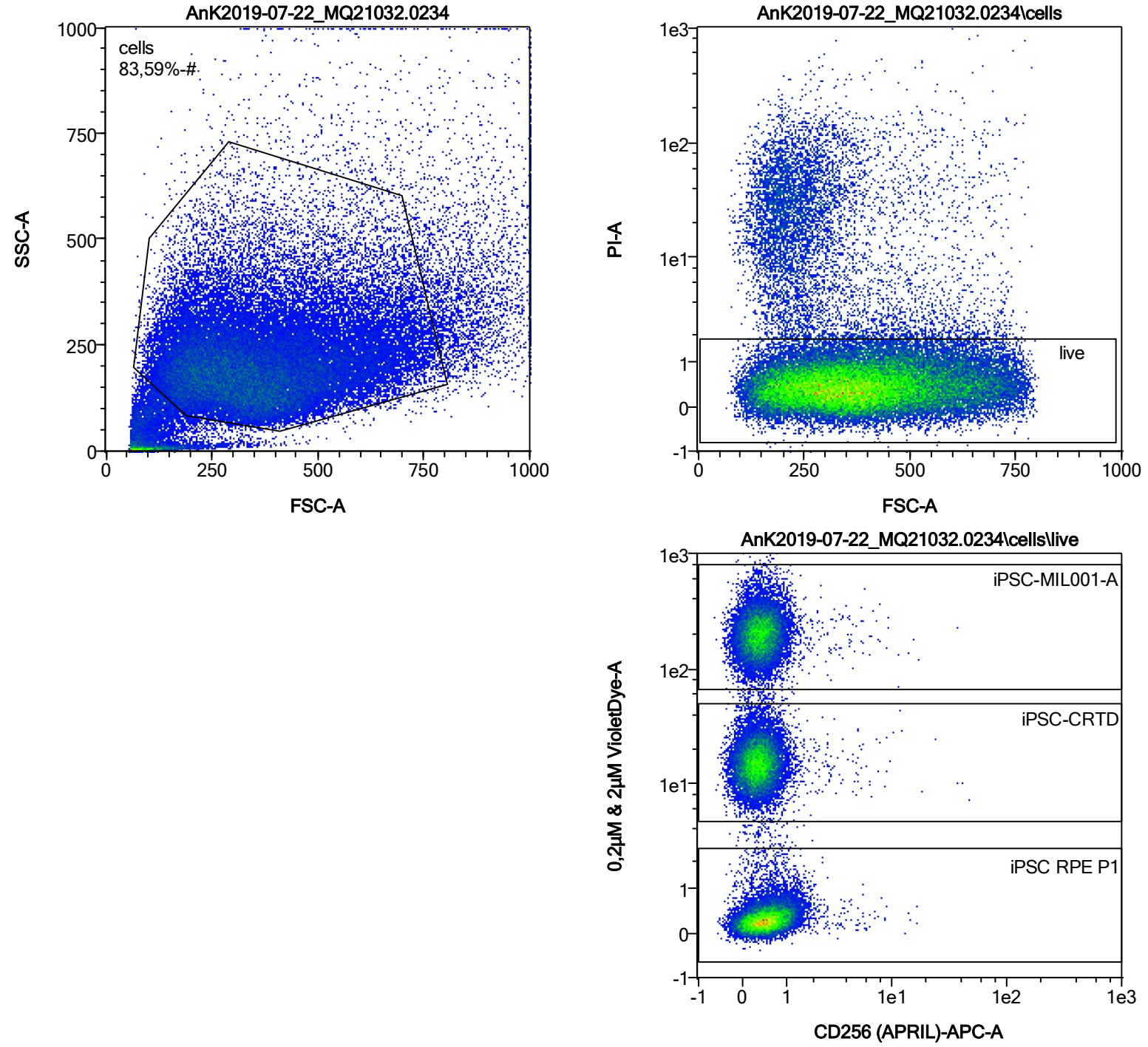

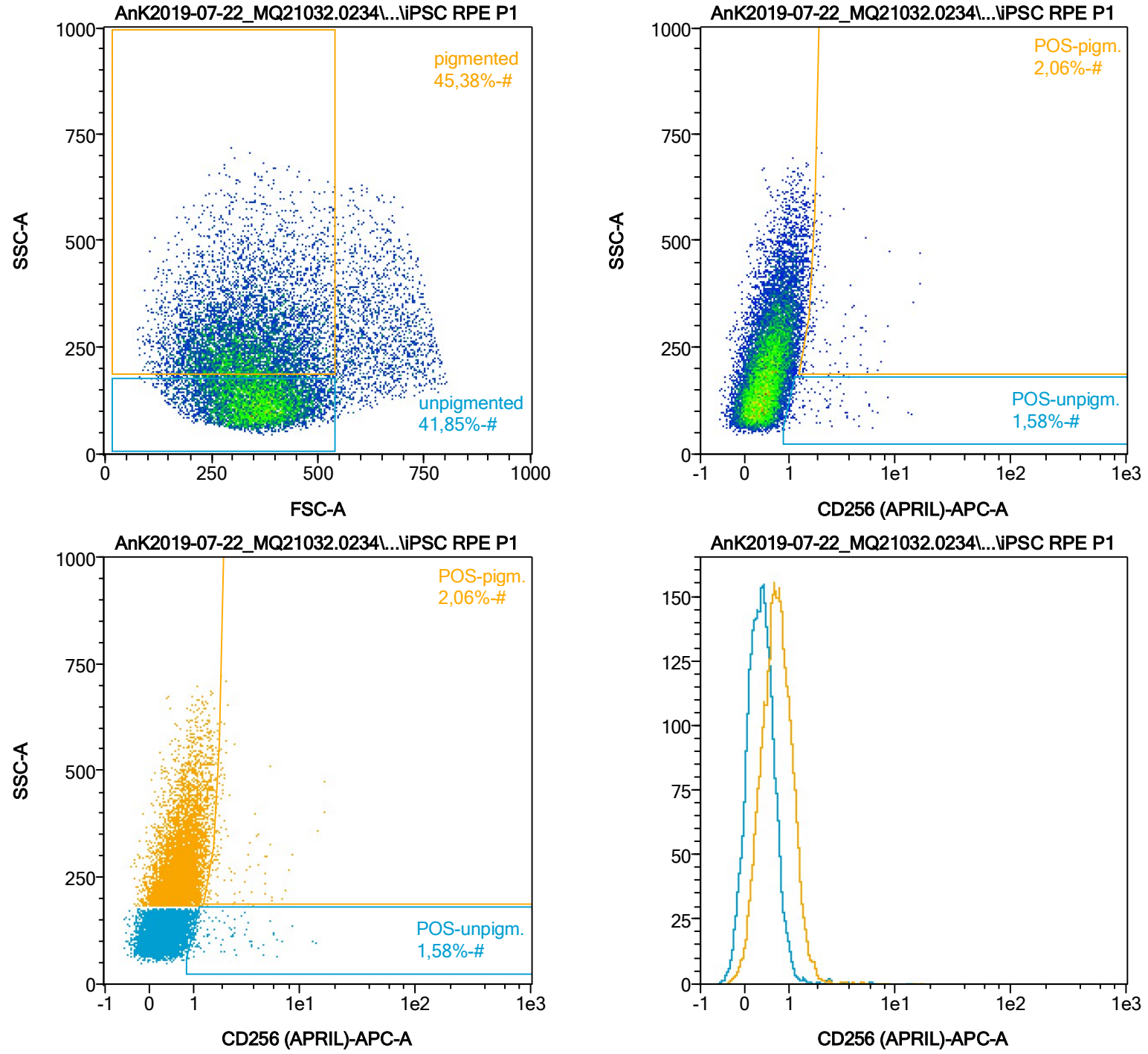

Name	%-#	CD256 (APRIL)-APC-A Mean	CD256 (APRIL)-APC-A Median
POS-unpigm.	1,58	2,40	1,48
POS-pigm.	2,06	2,74	1,91

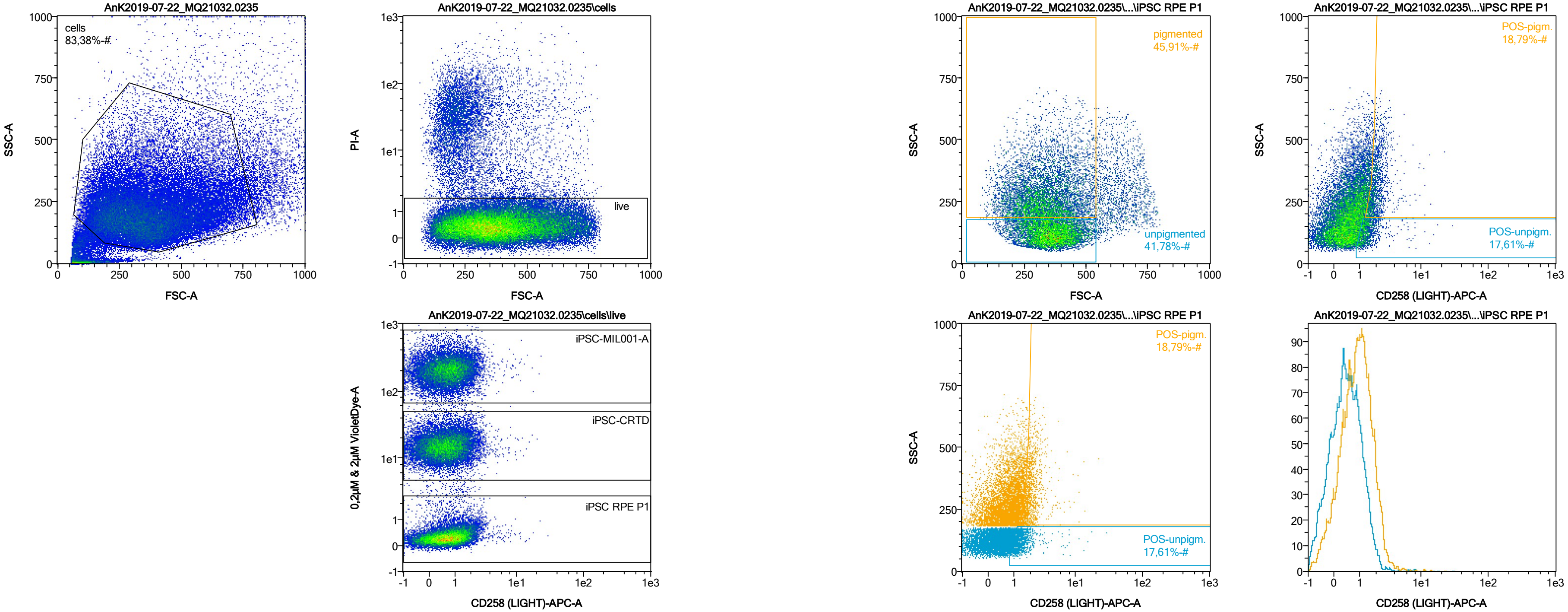

Name	%-#	CD258 (LIGHT)-APC-A Mean	CD258 (LIGHT)-APC-A Median
POS-unpigm.	17,61	1,55	1,31
POS-pigm.	18,79	2,17	1,94

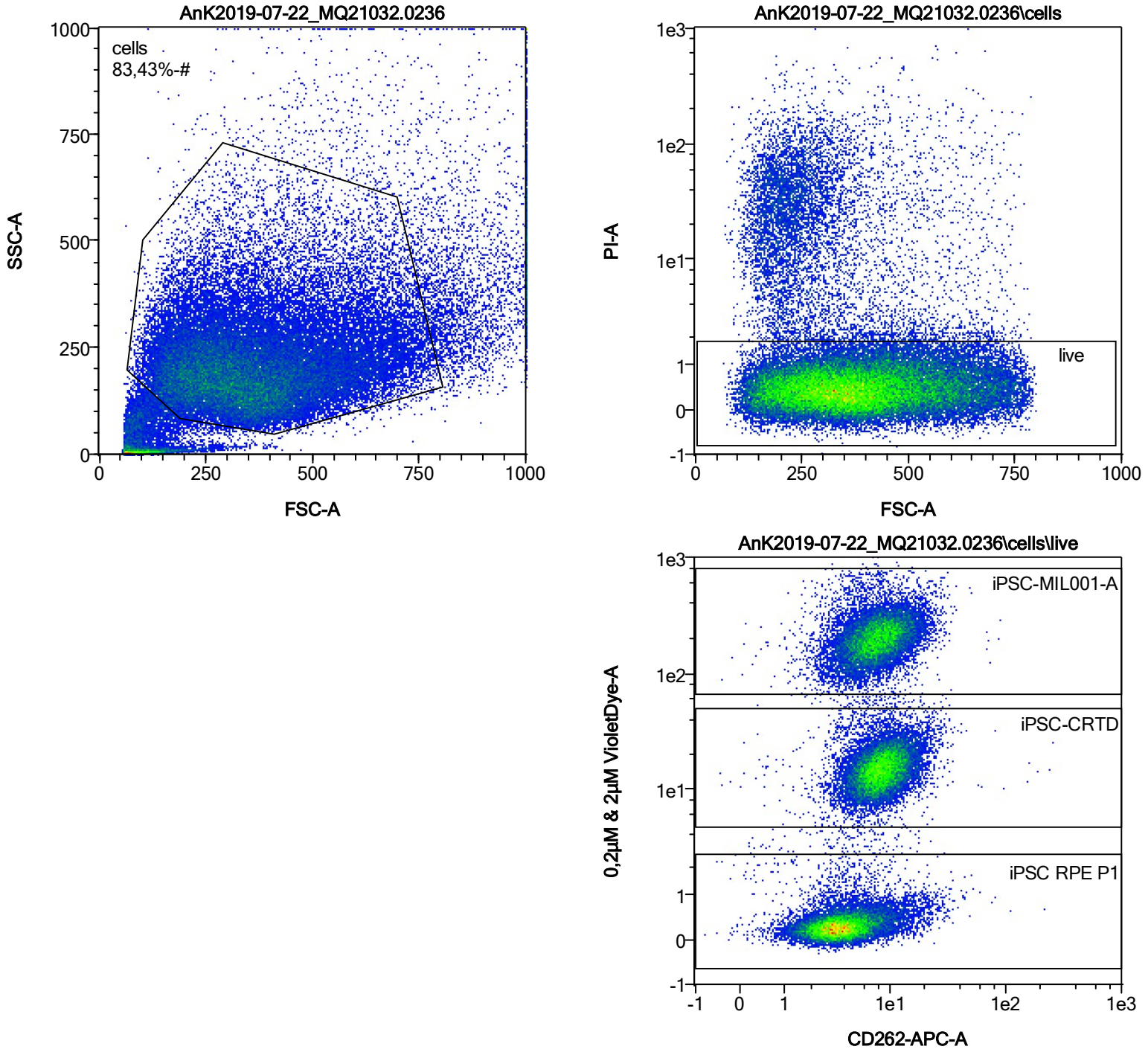

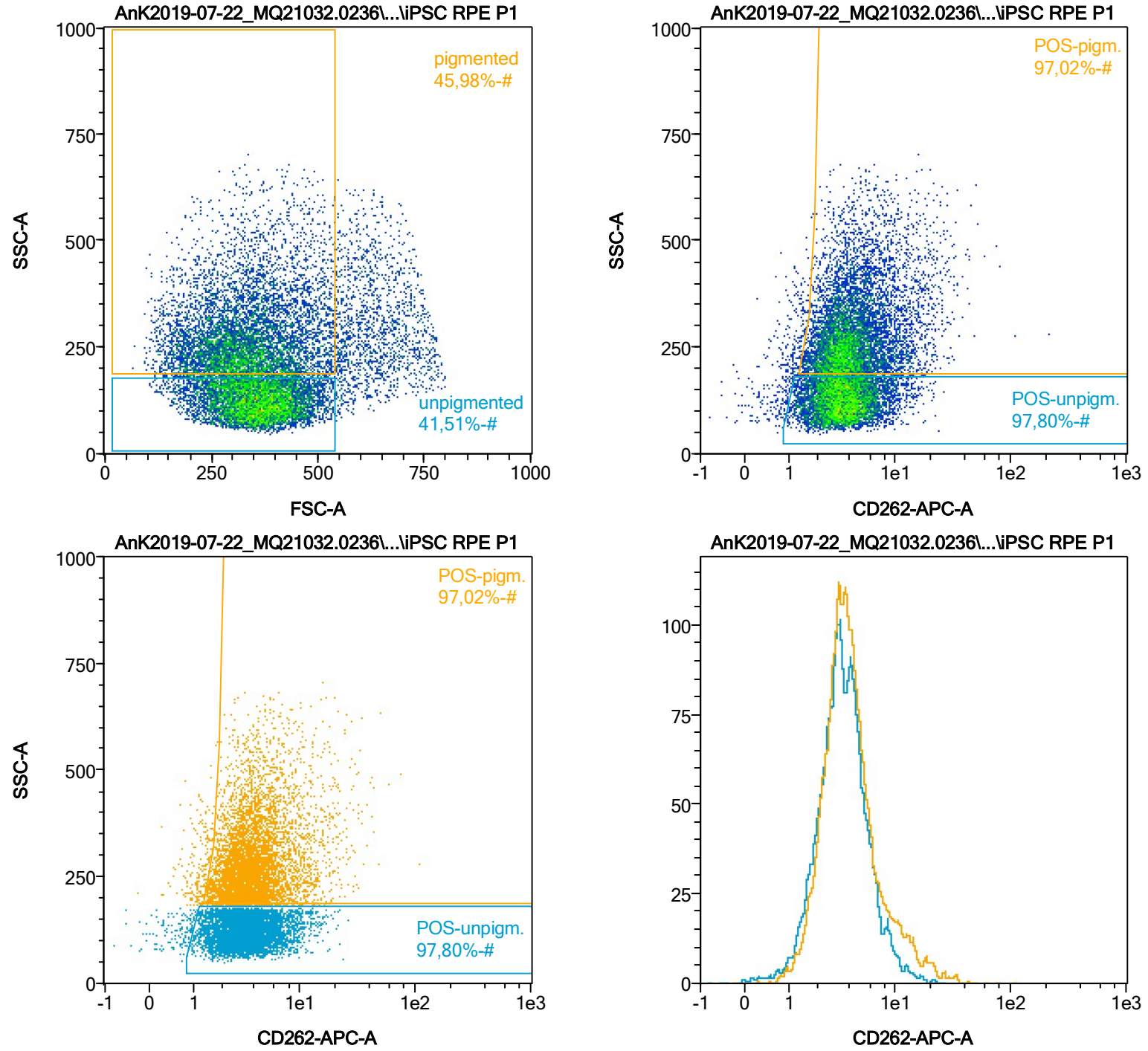

Name	%-#	CD262-APC-A Mean	CD262-APC-A Median
POS-unpigm.	97,80	3,91	3,41
POS-pigm.	97,02	4,86	3,72

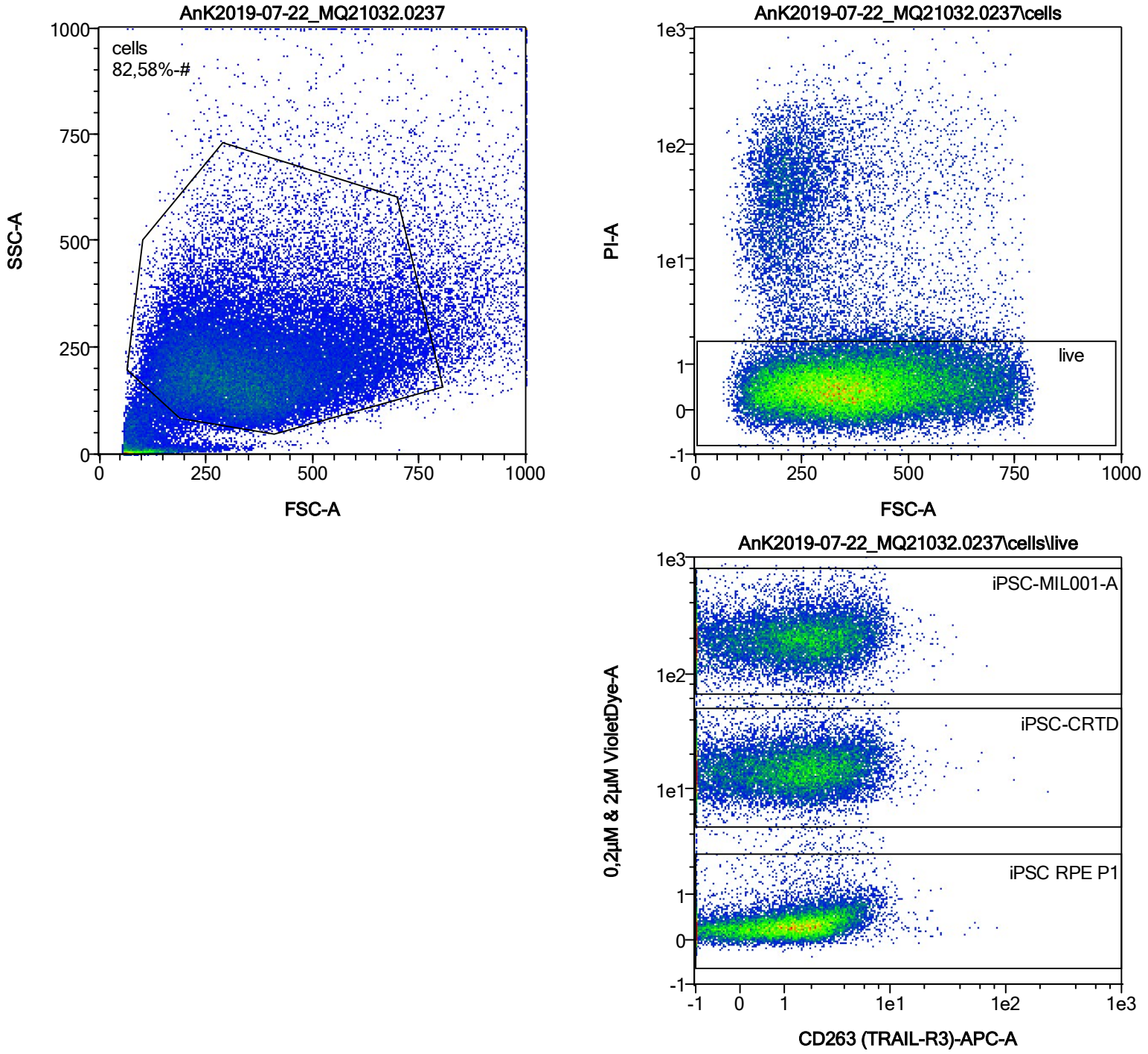

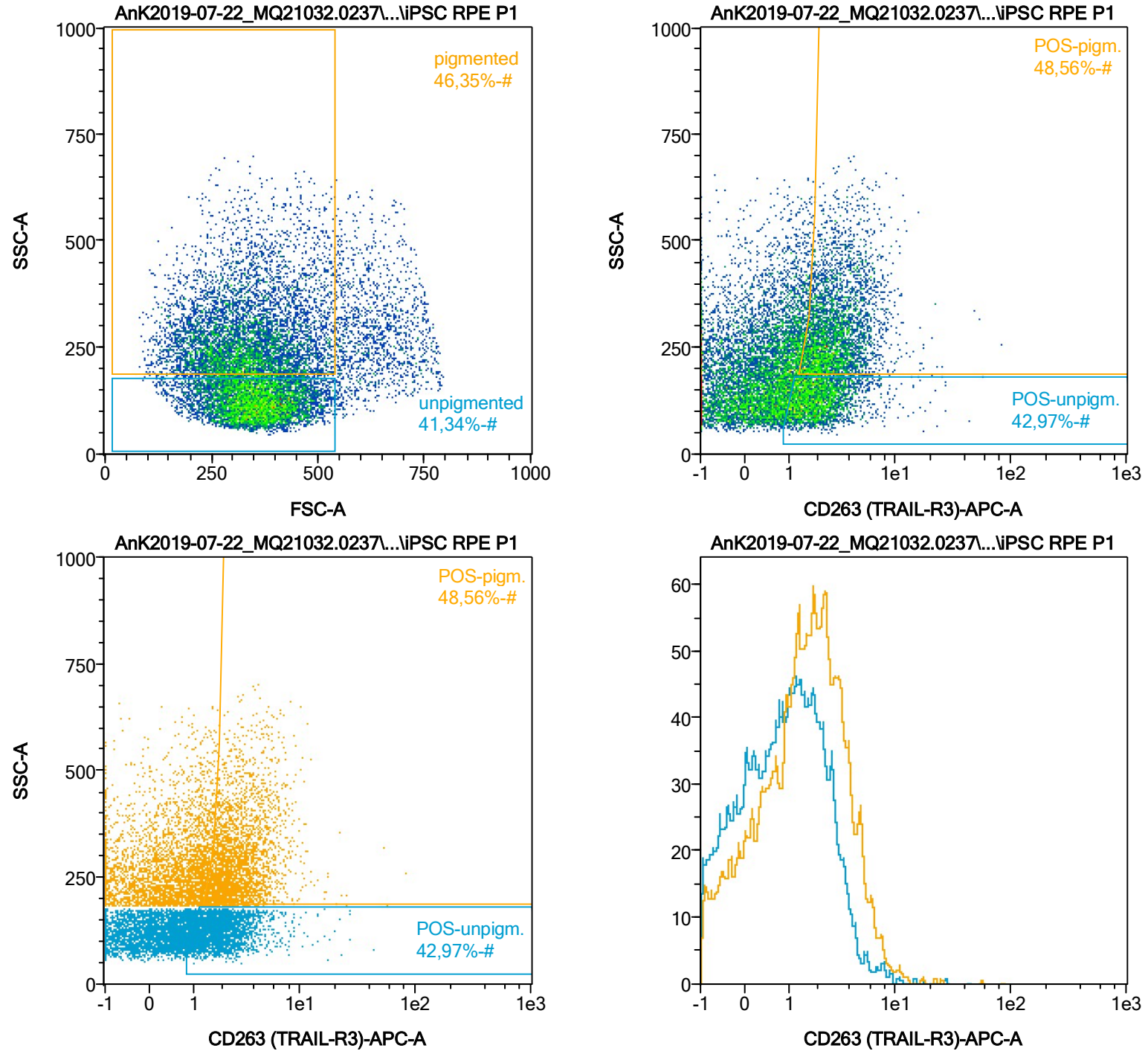

Name	%-#	CD263 (TRAIL-R3)-APC-A Mean	CD263 (TRAIL-R3)-APC-A Median
POS-unpigm.	42,97	2,34	1,92
POS-pigm.	48,56	3,25	2,68

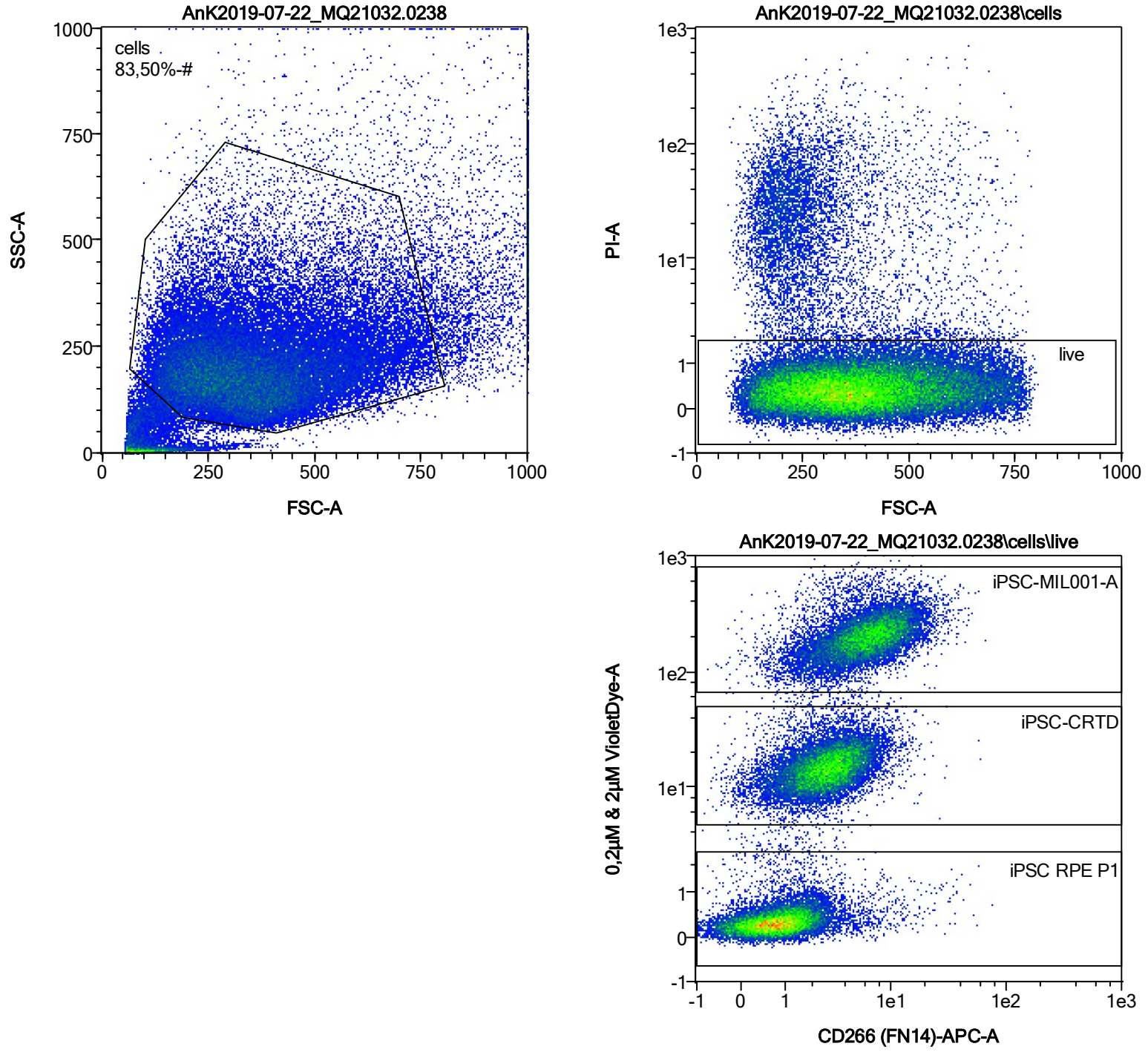

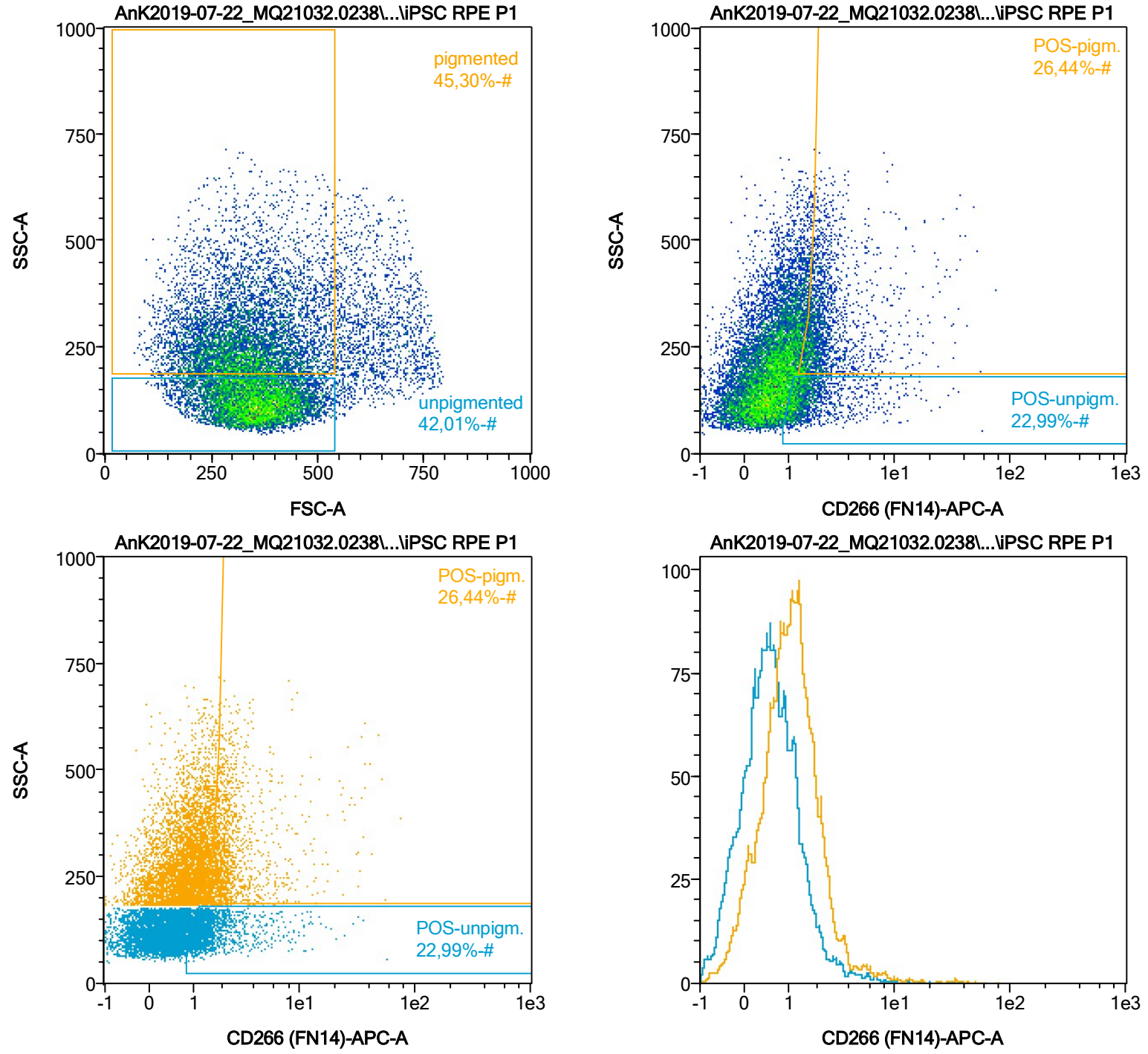

Name	%-#	CD266 (FN14)-APC-A Mean	CD266 (FN14)-APC-A Median
POS-unpigm.	22,99	2,08	1,42
POS-pigm.	26,44	3,13	2,09

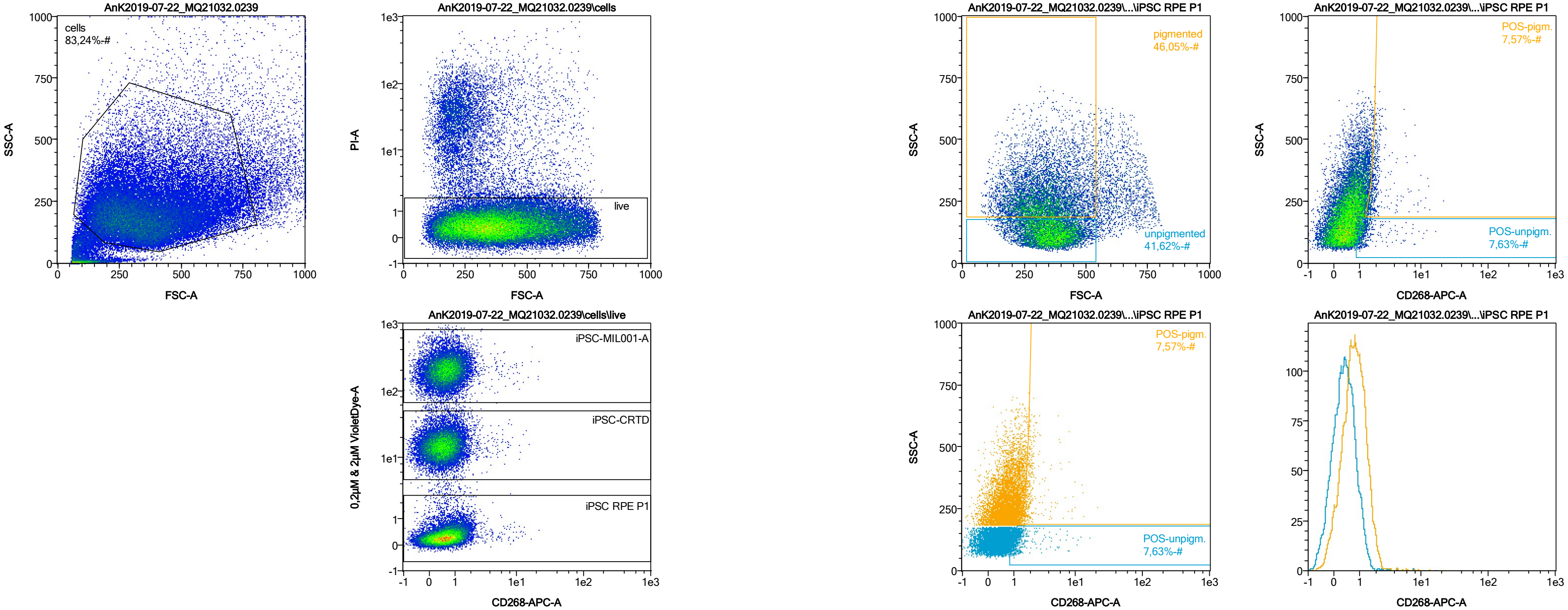

Name	%-#	CD268-APC-A Mean	CD268-APC-A Median
POS-unpigm.	7,63	1,58	1,21
POS-pigm.	7,57	2,19	1,80

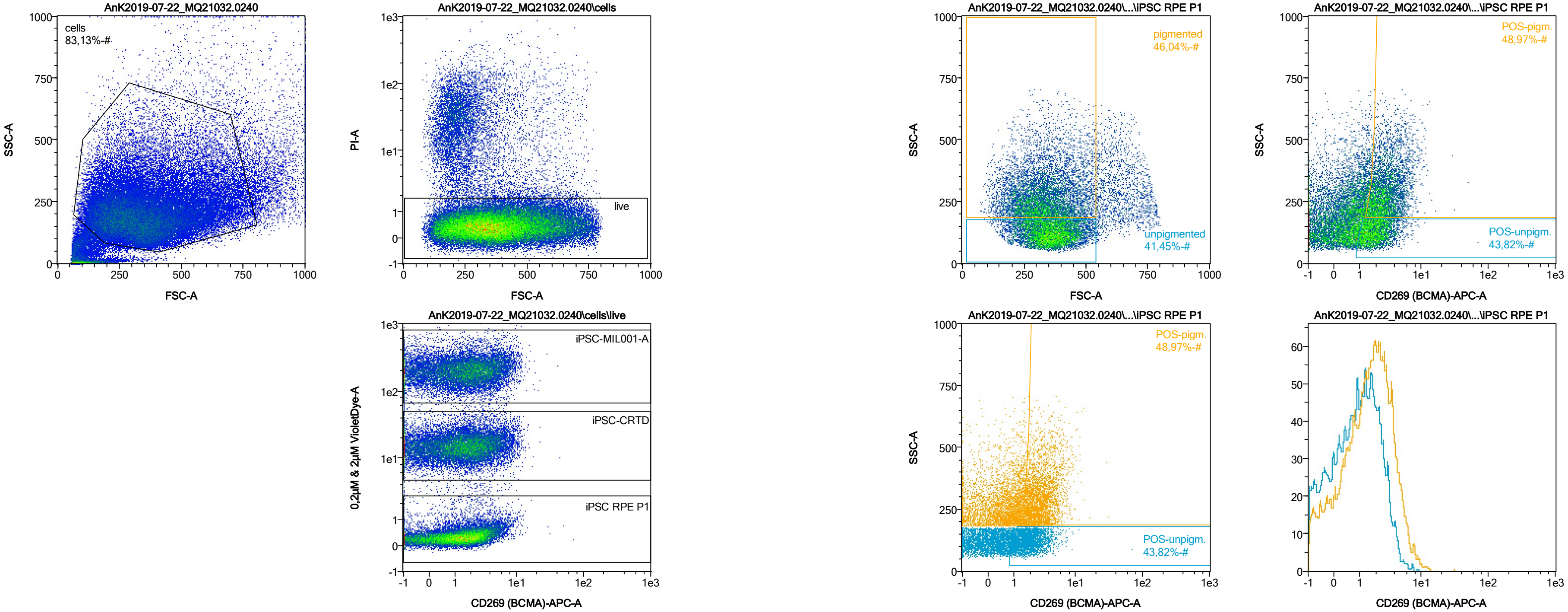

Name	%-#	CD269 (BCMA)-APC-A Mean	CD269 (BCMA)-APC-A Median
POS-unpigm.	43,82	2,11	1,84
POS-pigm.	48,97	3,14	2,69

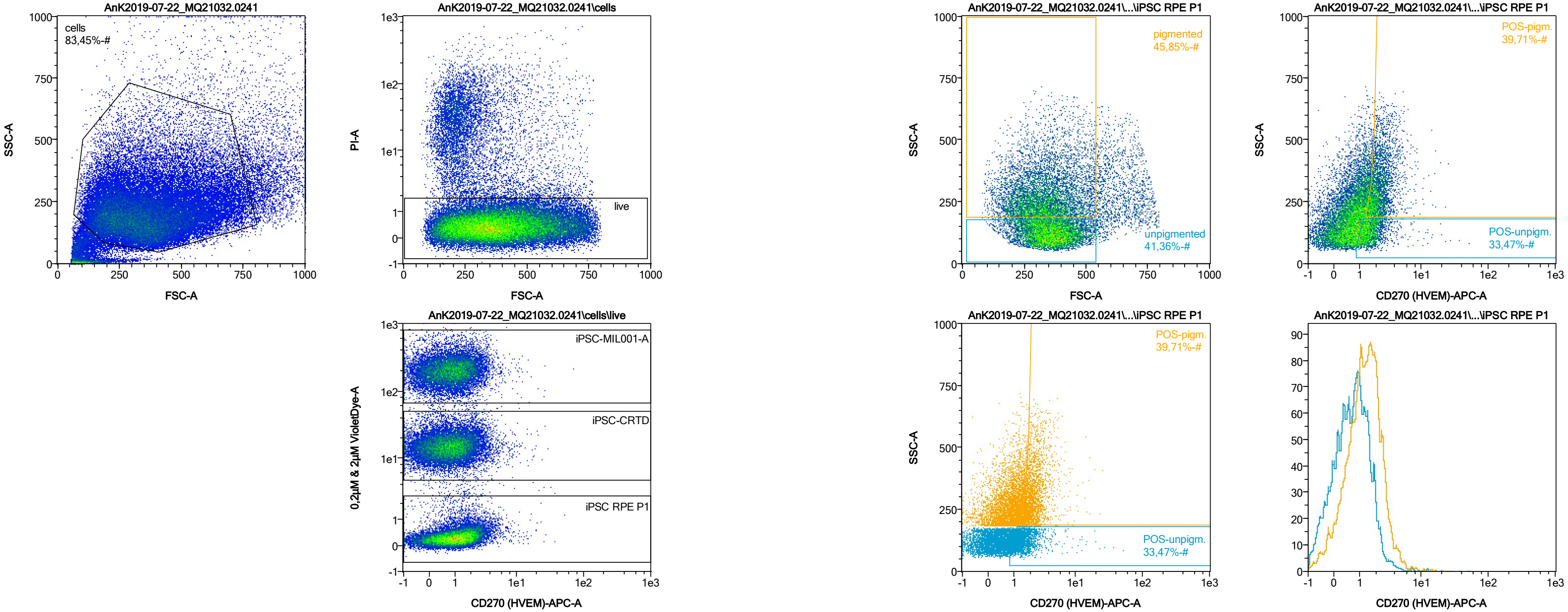

Name	%-#	CD270 (HVEM)-APC-A Mean	CD270 (HVEM)-APC-A Median
POS-unpigm.	33,47	1,76	1,46
POS-pigm.	39,71	2,43	2,12

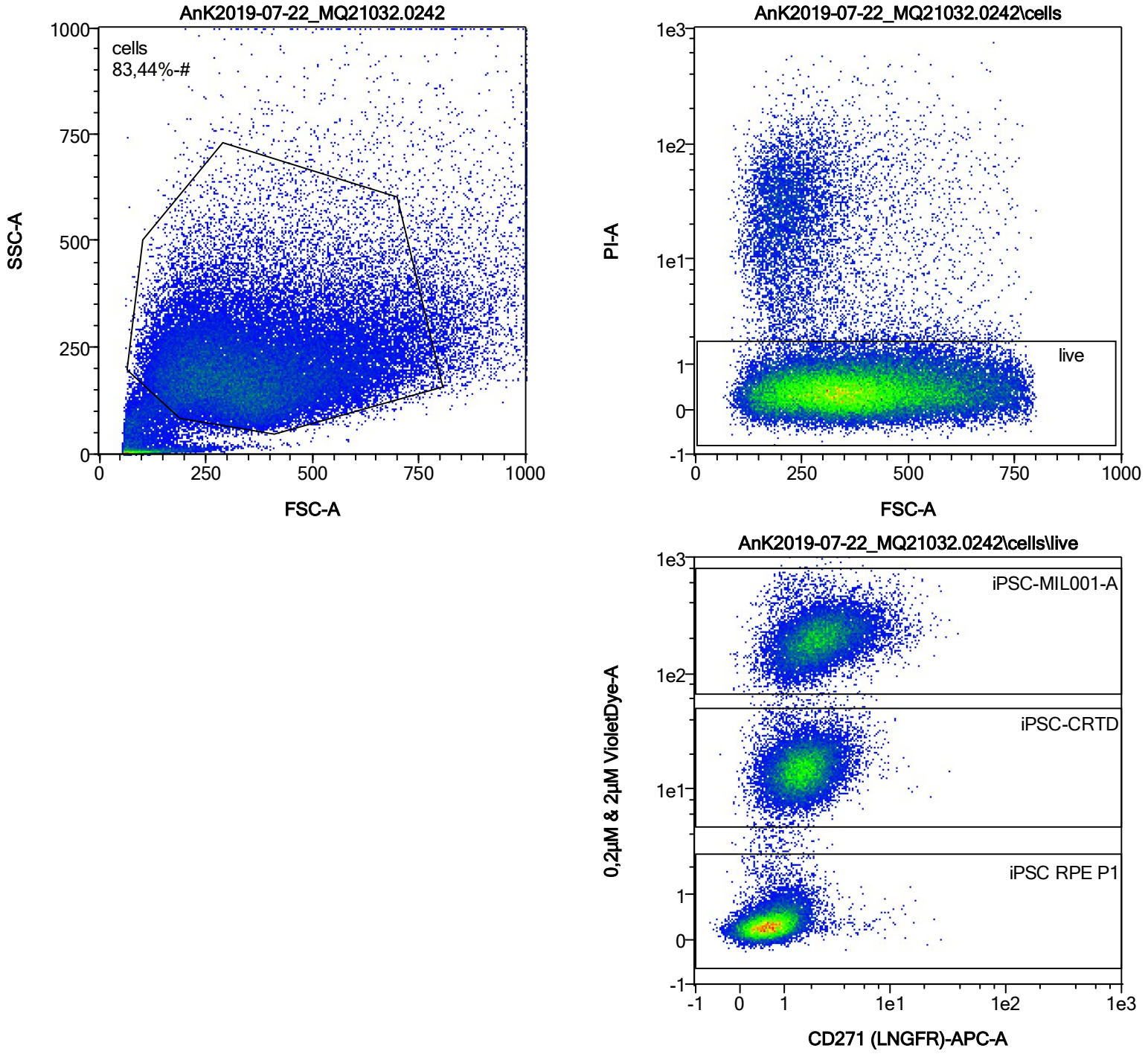

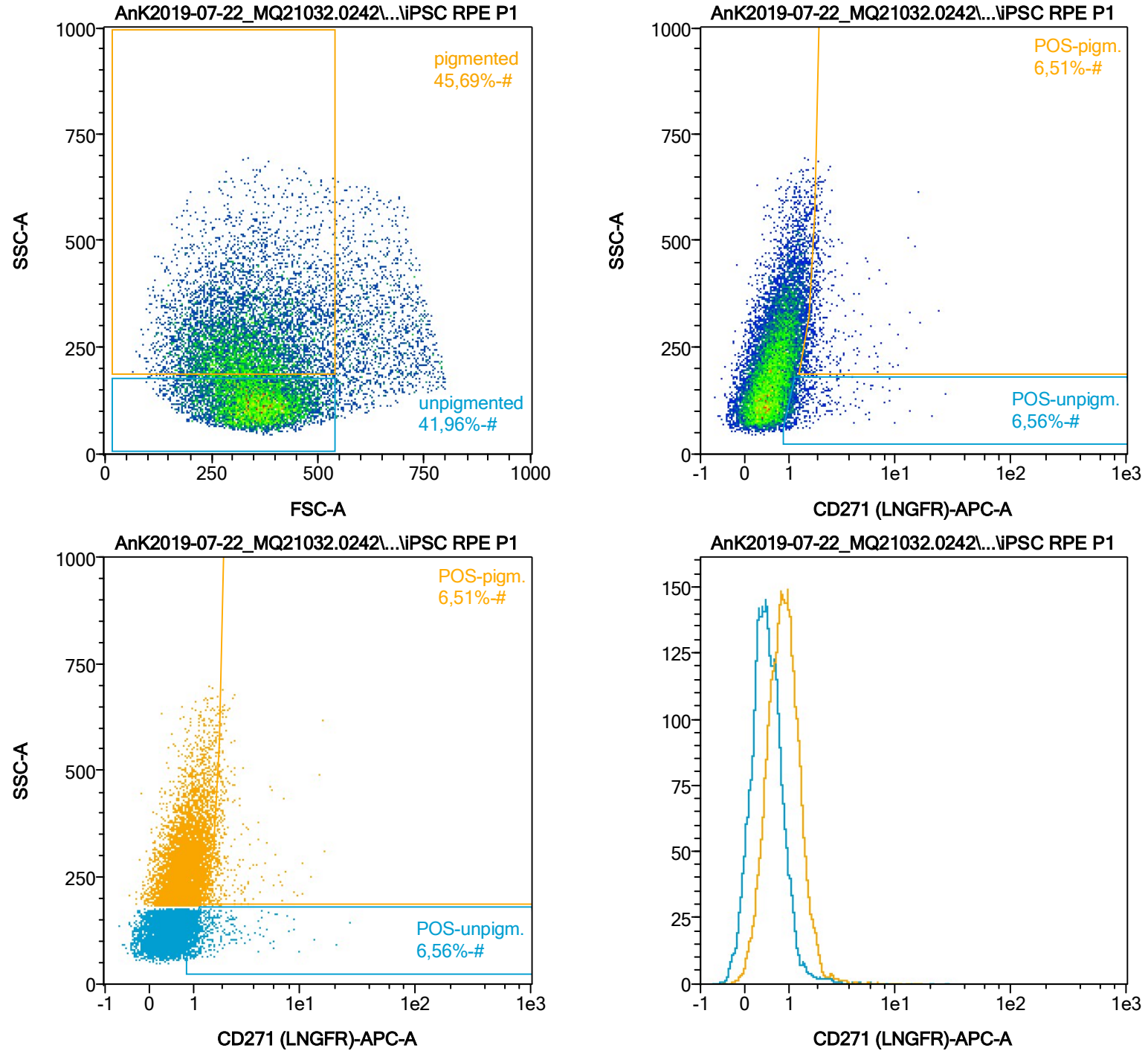

Name	%-#	CD271 (LNGFR)-APC-A Mean	CD271 (LNGFR)-APC-A Median
POS-unpigm.	6,56	1,76	1,23
POS-pigm.	6,51	2,27	1,85

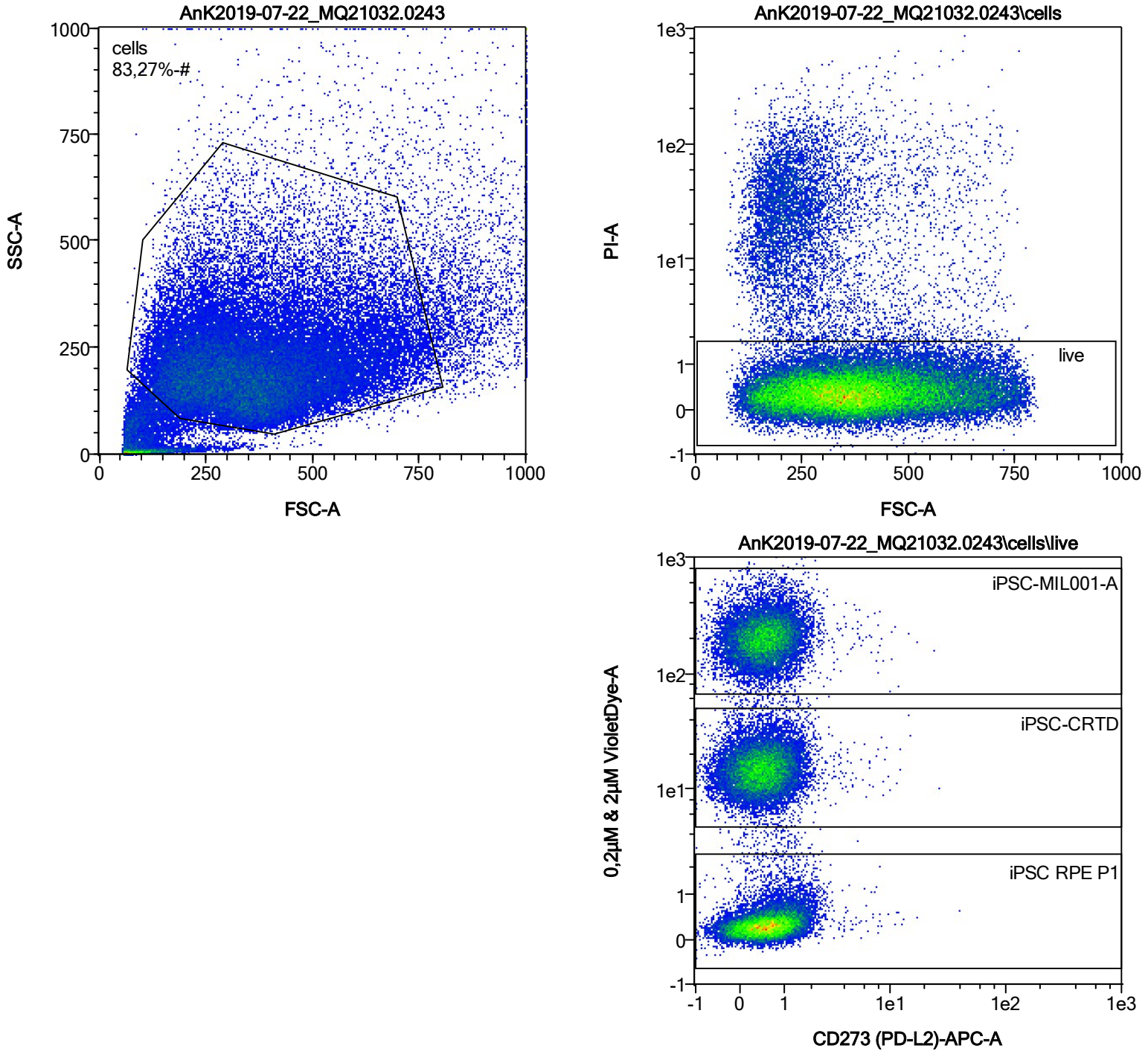

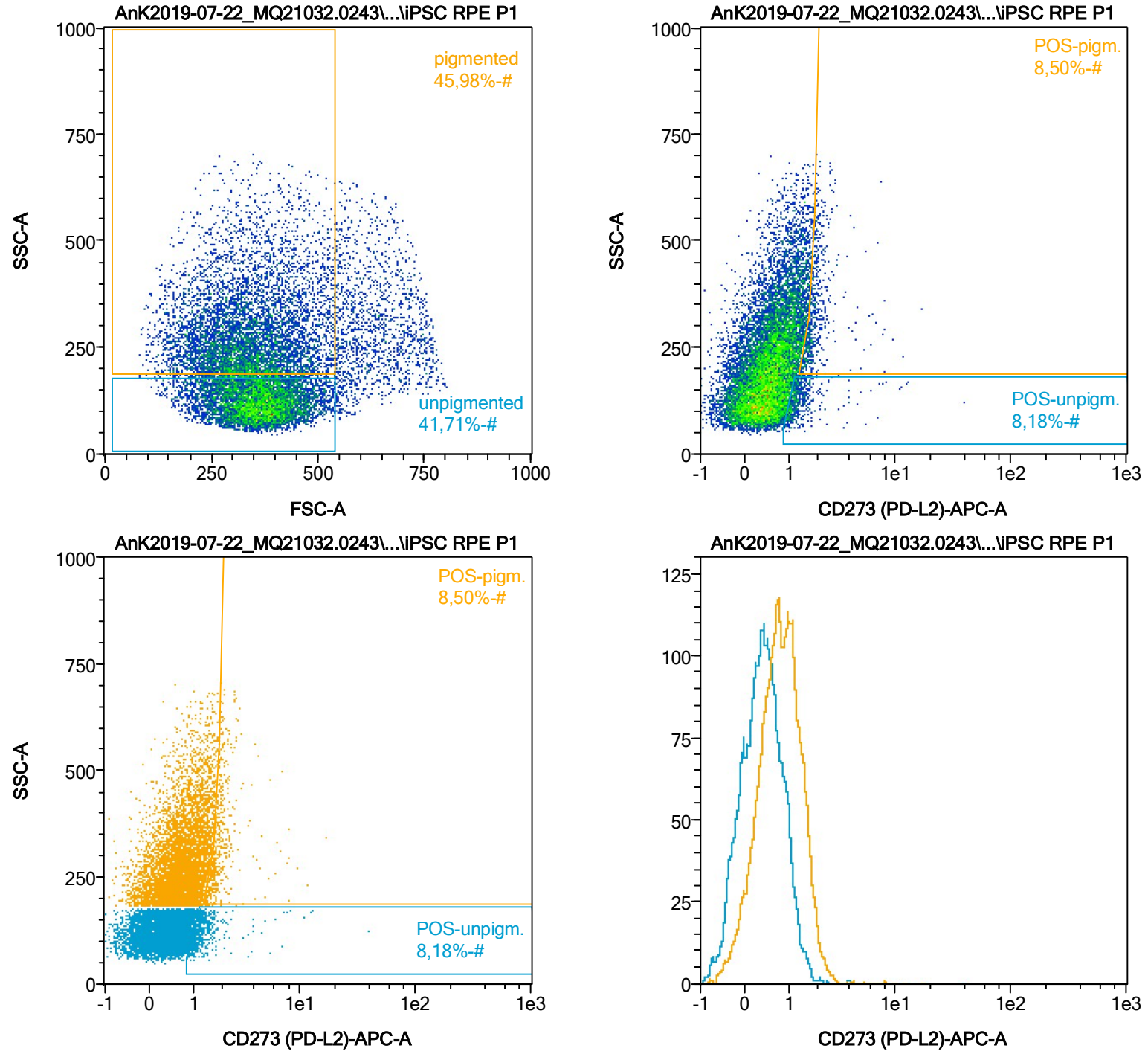

Name	%-#	CD273 (PD-L2)-APC-A Mean	CD273 (PD-L2)-APC-A Median
POS-unpigm.	8,18	1,52	1,20
POS-pigm.	8,50	2,04	1,82

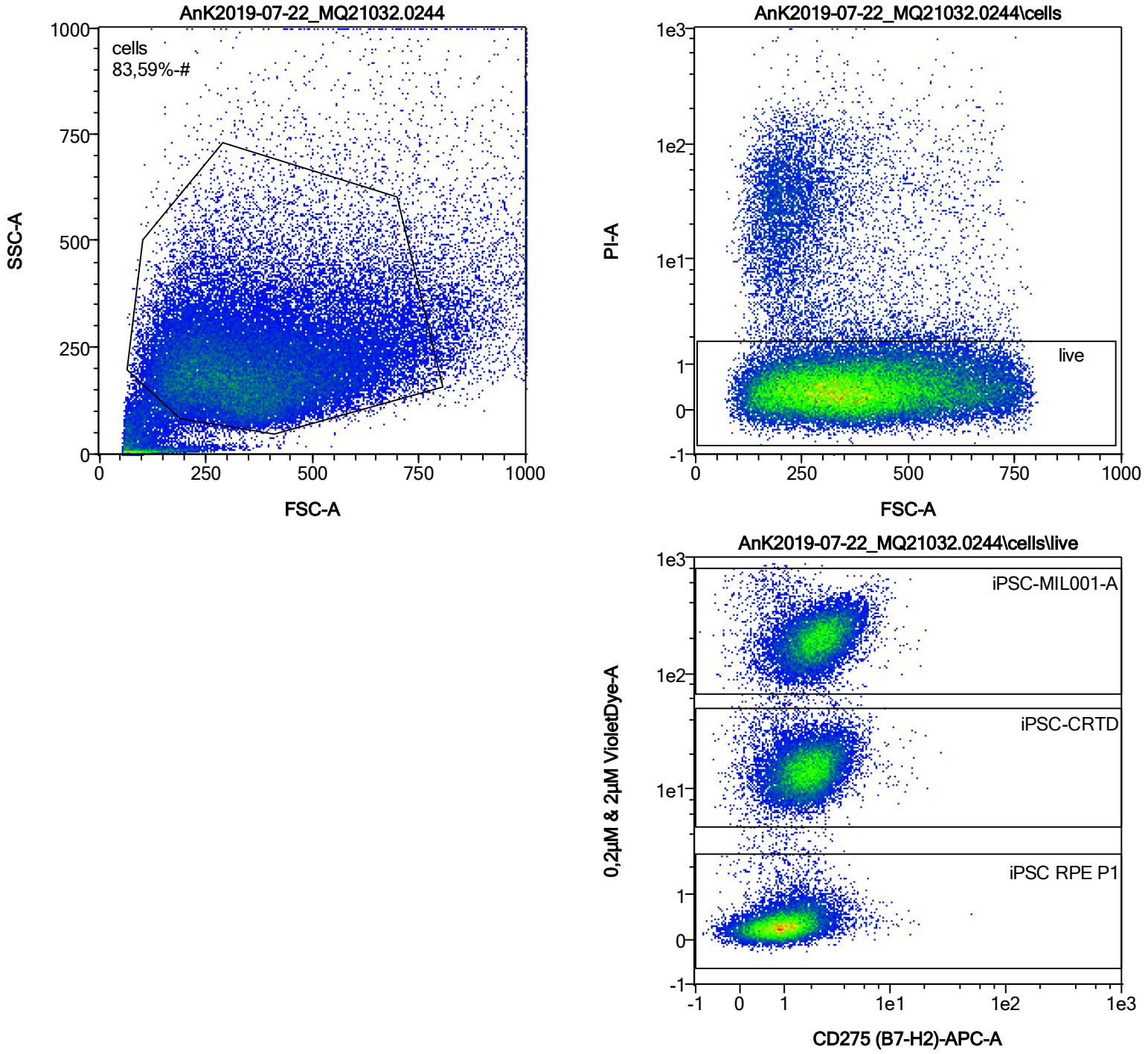

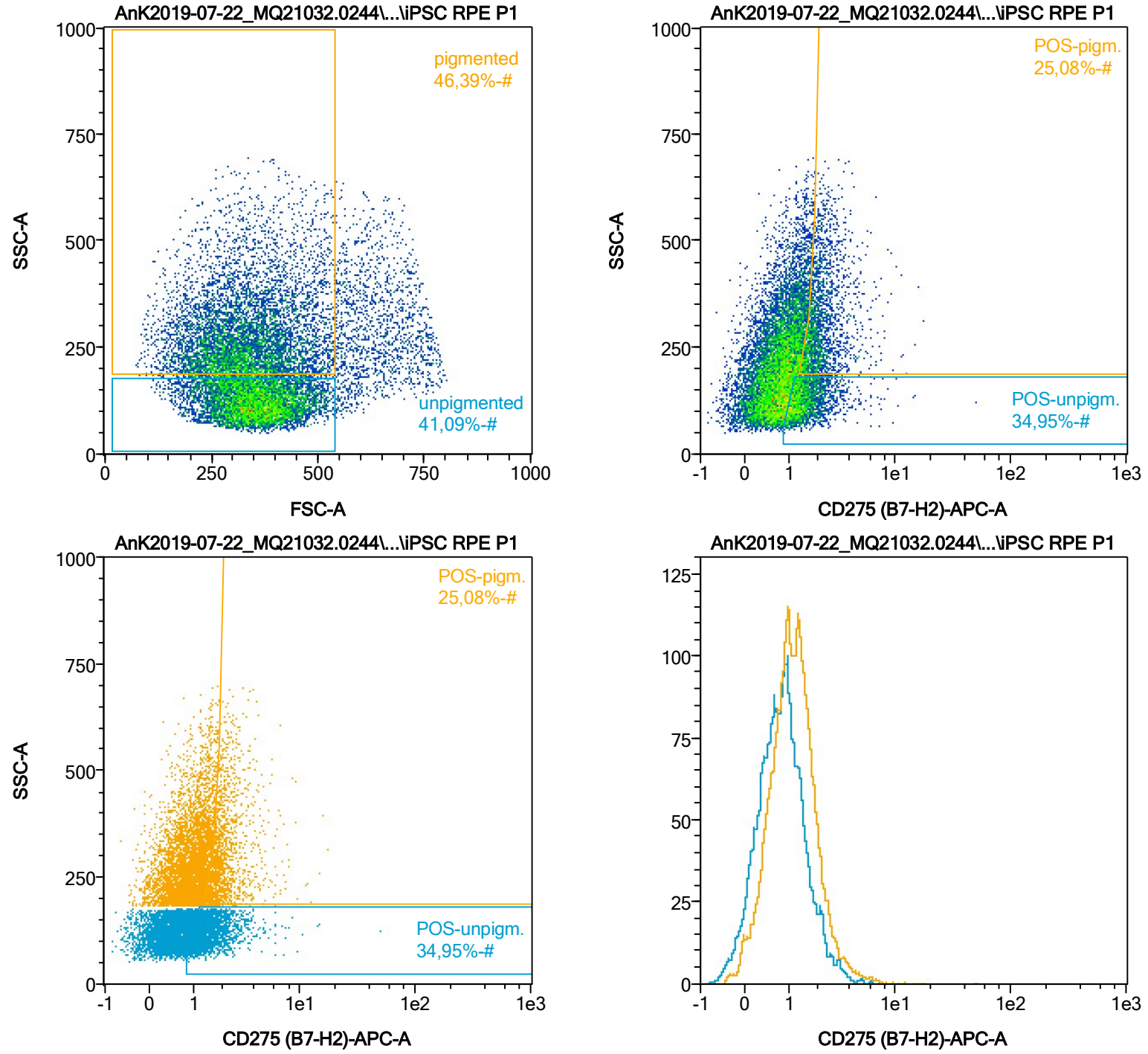

Name	%-#	CD275 (B7-H2)-APC-A Mean	CD275 (B7-H2)-APC-A Median
POS-unpigm.	34,95	1,65	1,39
POS-pigm.	25,08	2,26	1,95

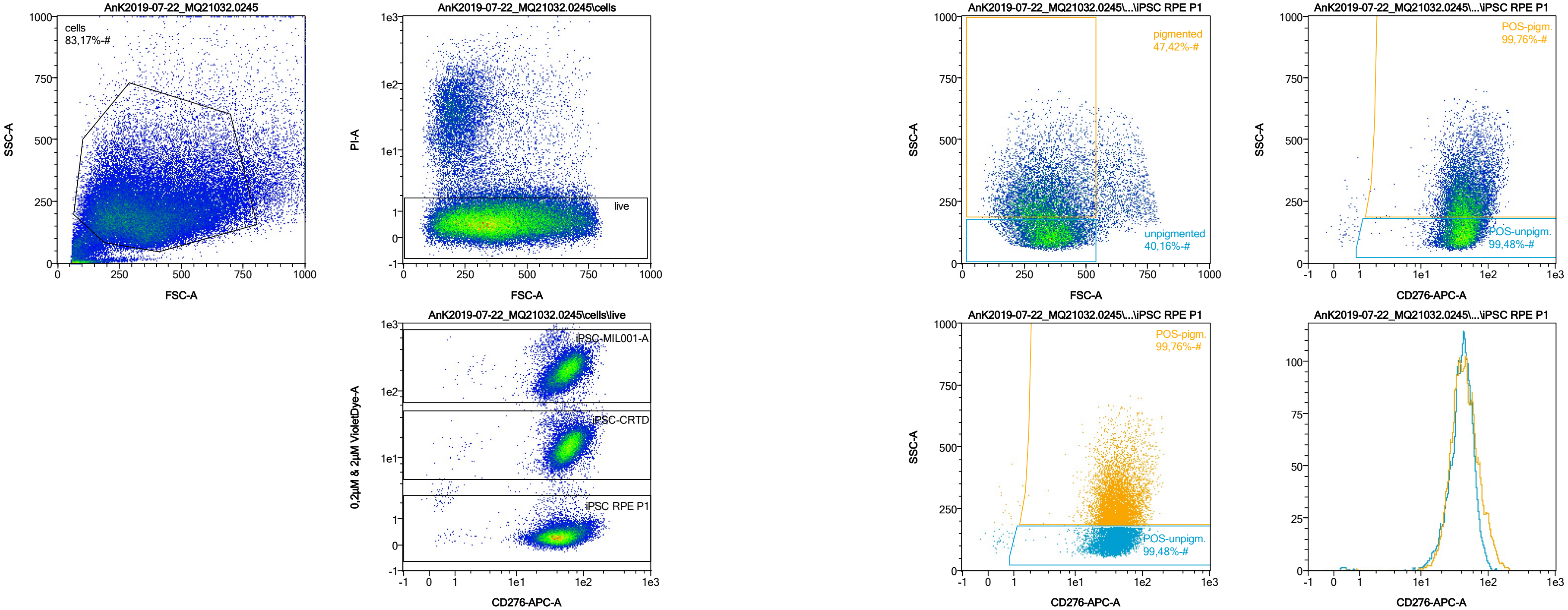

Name	%-#	CD276-APC-A Mean	CD276-APC-A Median
POS-unpigm.	99,48	42,20	40,49
POS-pigm.	99,76	49,12	43,15

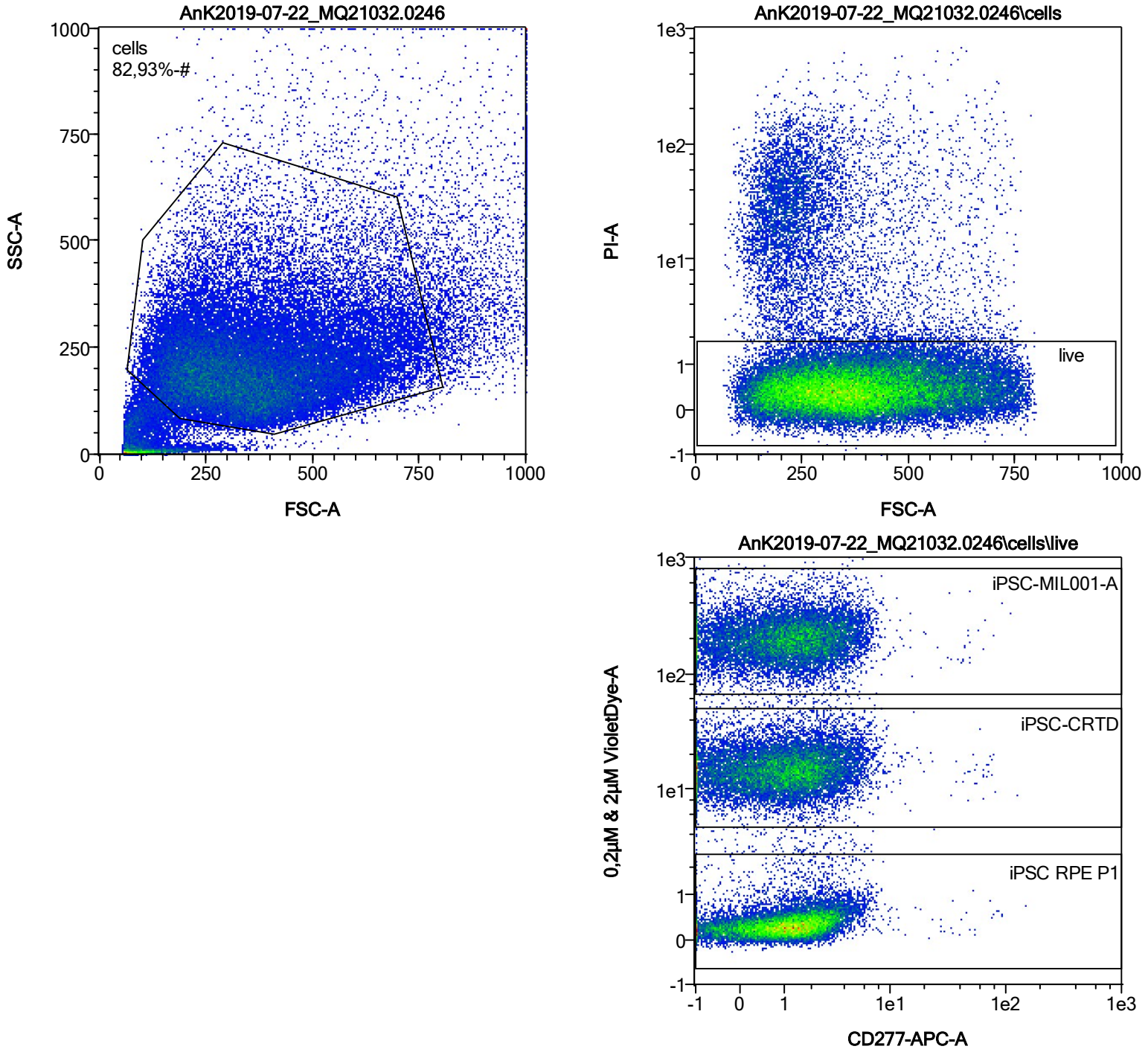

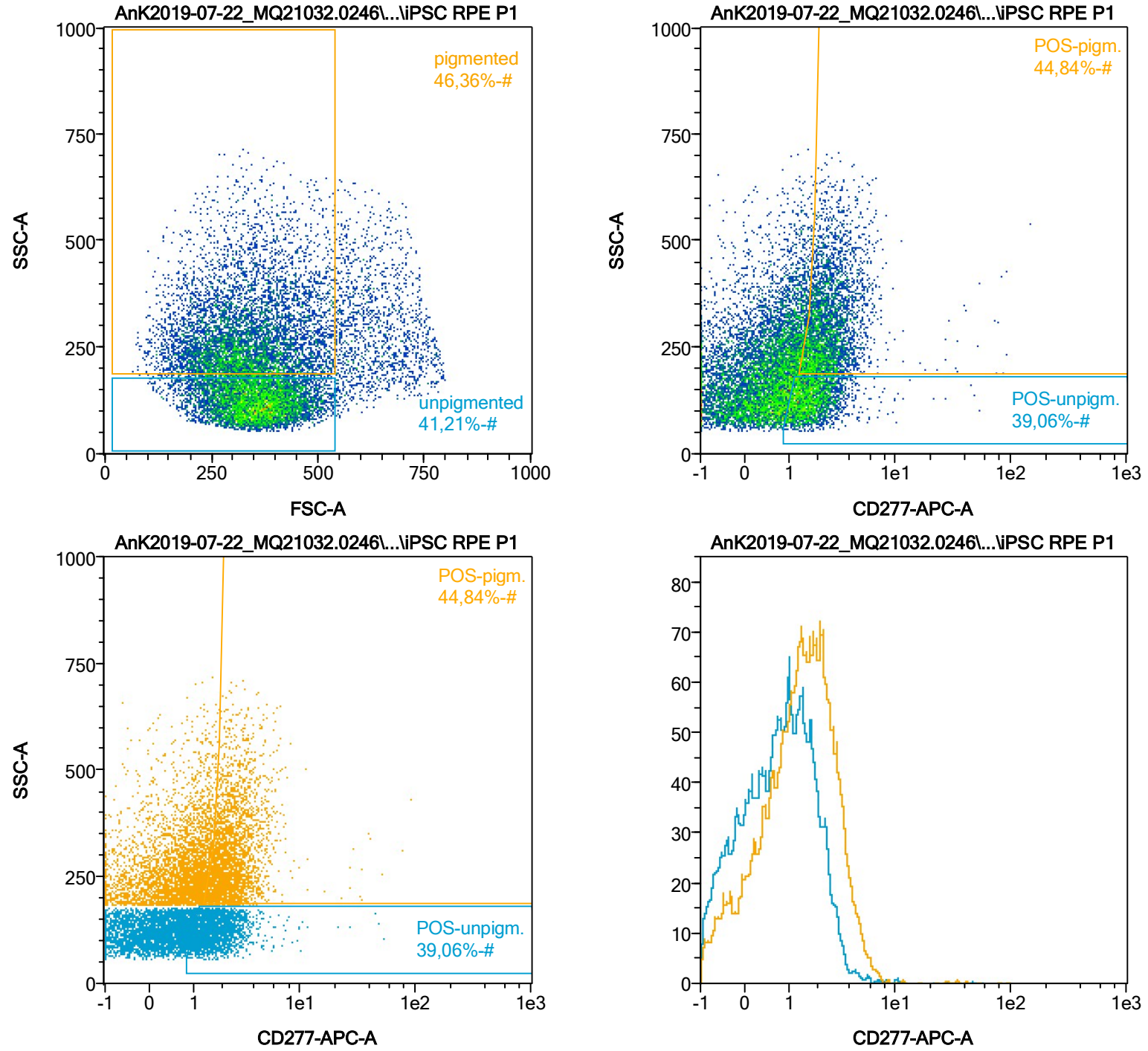

Name	%-#	CD277-APC-A Mean	CD277-APC-A Median
POS-unpigm.	39,06	1,93	1,65
POS-pigm.	44,84	2,80	2,36

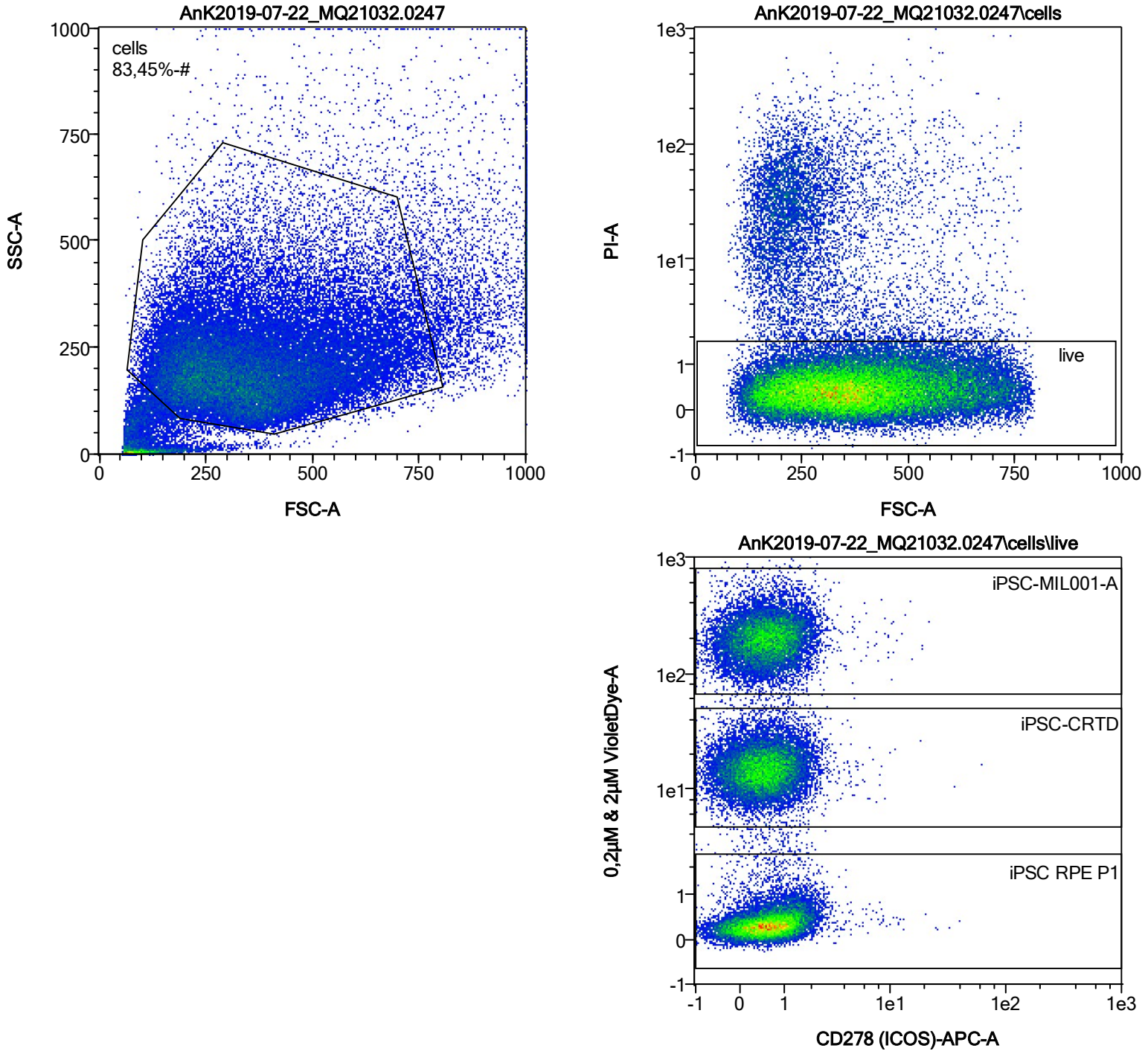

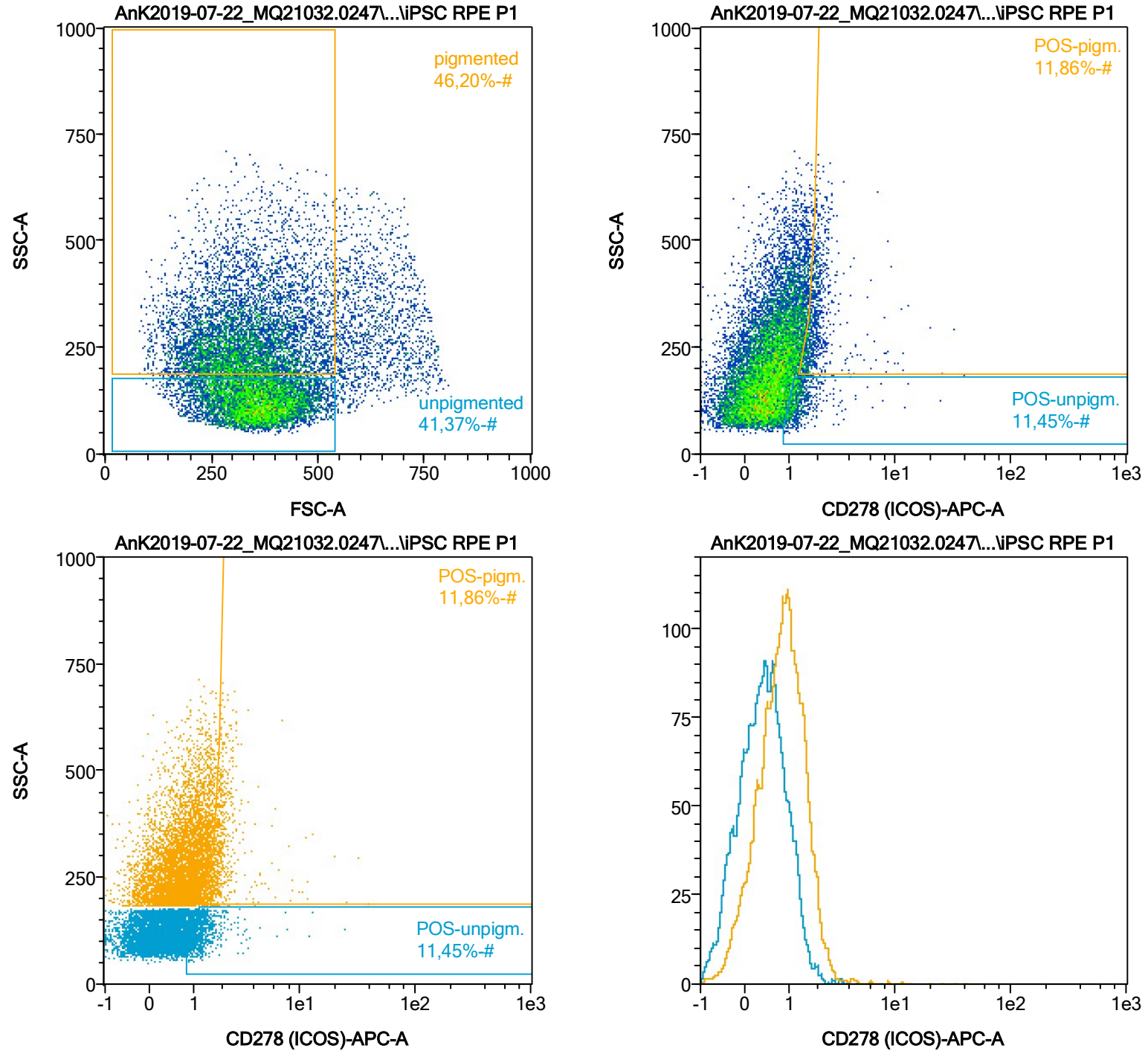

Name	%-#	CD278 (ICOS)-APC-A Mean	CD278 (ICOS)-APC-A Median
POS-unpigm.	11,45	1,43	1,24
POS-pigm.	11,86	2,23	1,86

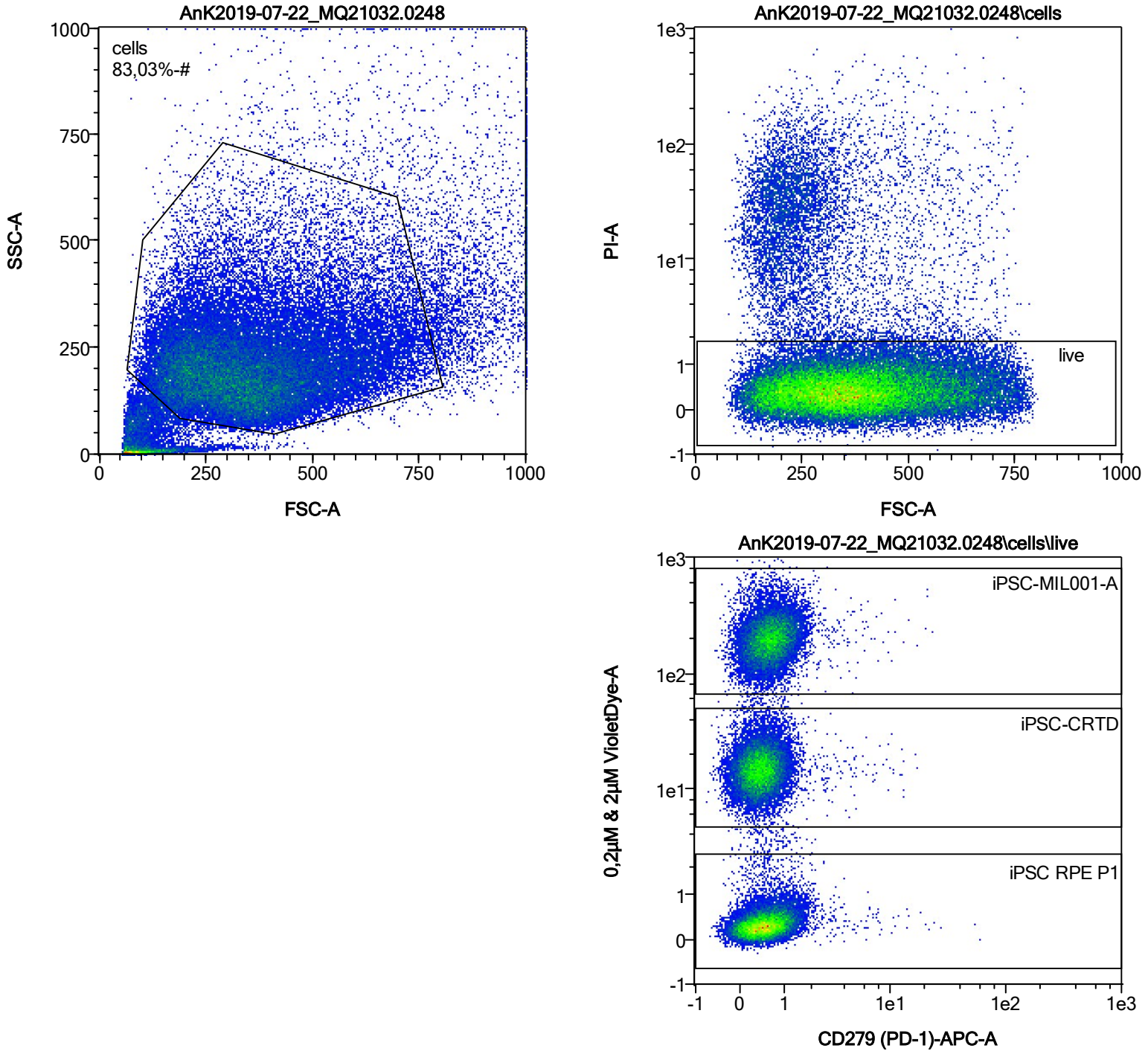

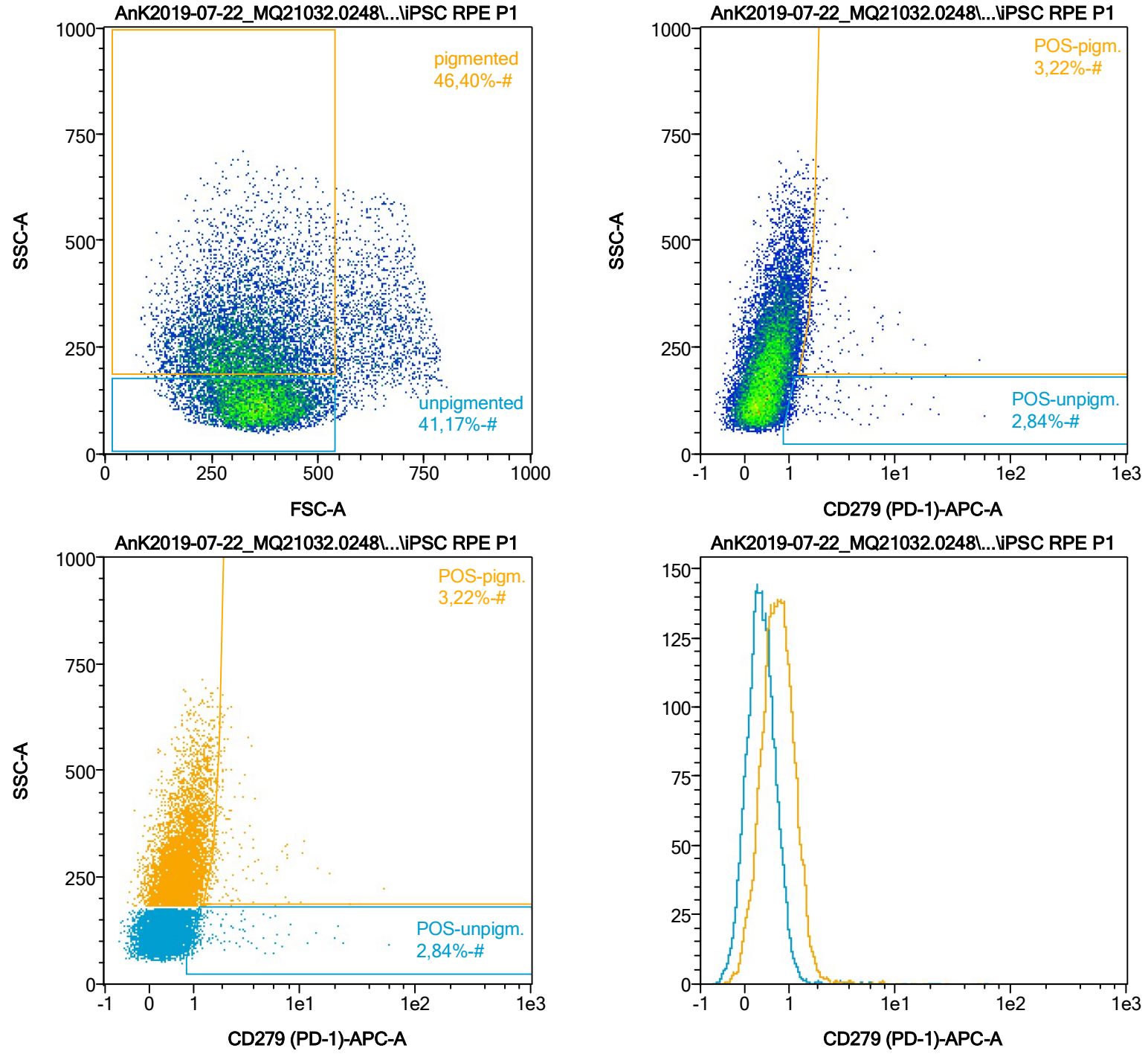

Name	%-#	CD279 (PD-1)-APC-A Mean	CD279 (PD-1)-APC-A Median
POS-unpigm.	2,84	2,61	1,20
POS-pigm.	3,22	2,88	1,87

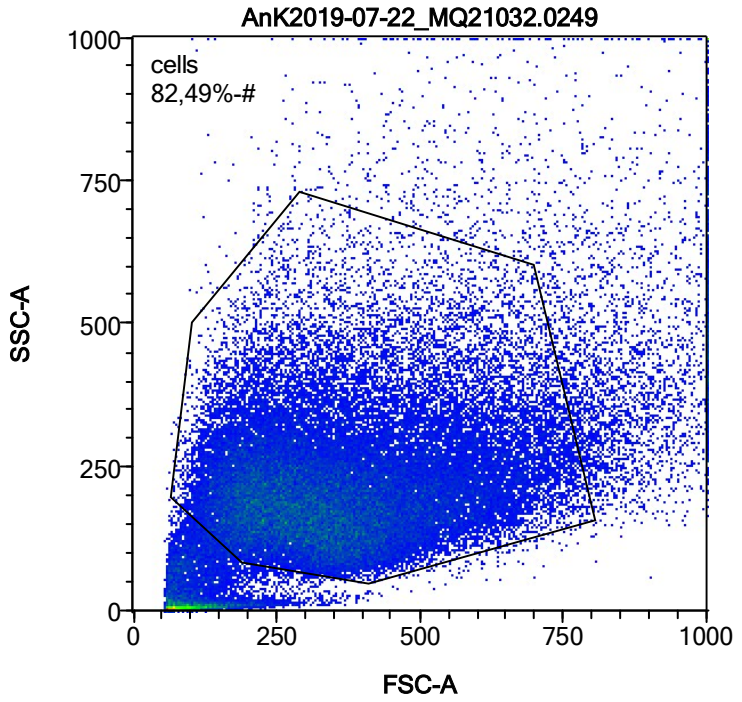

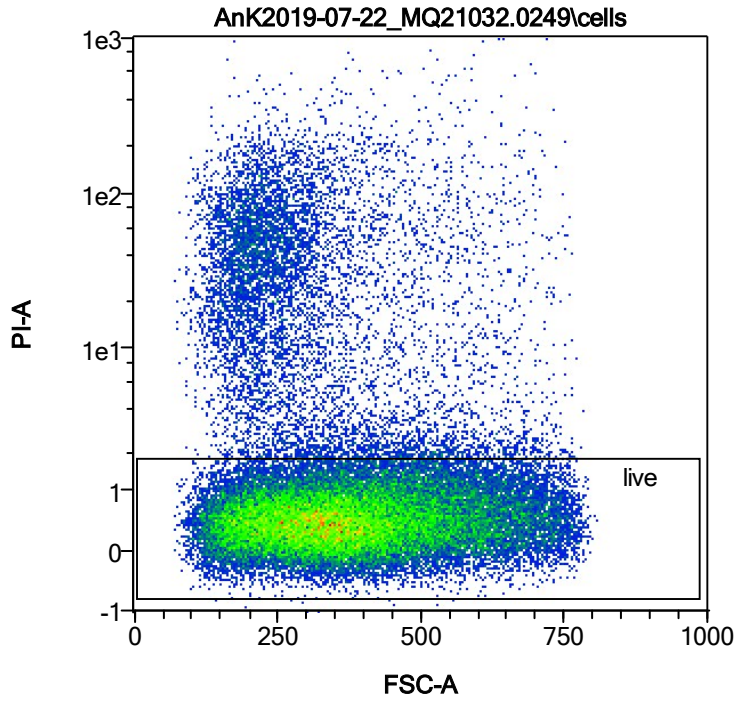

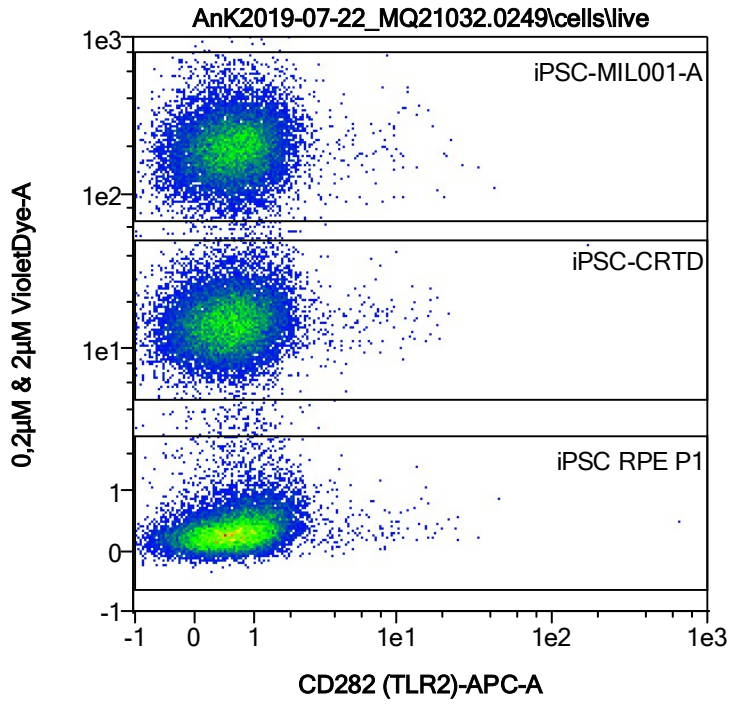

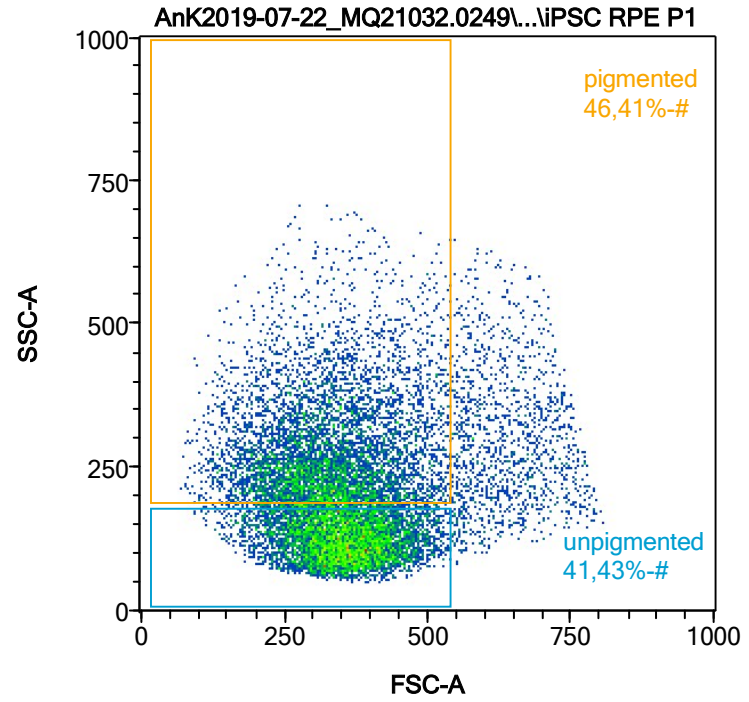

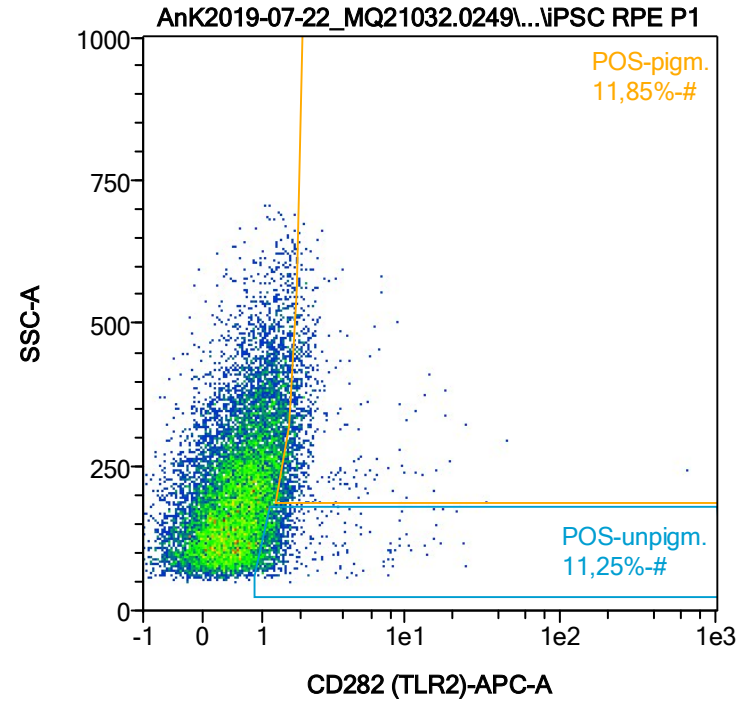

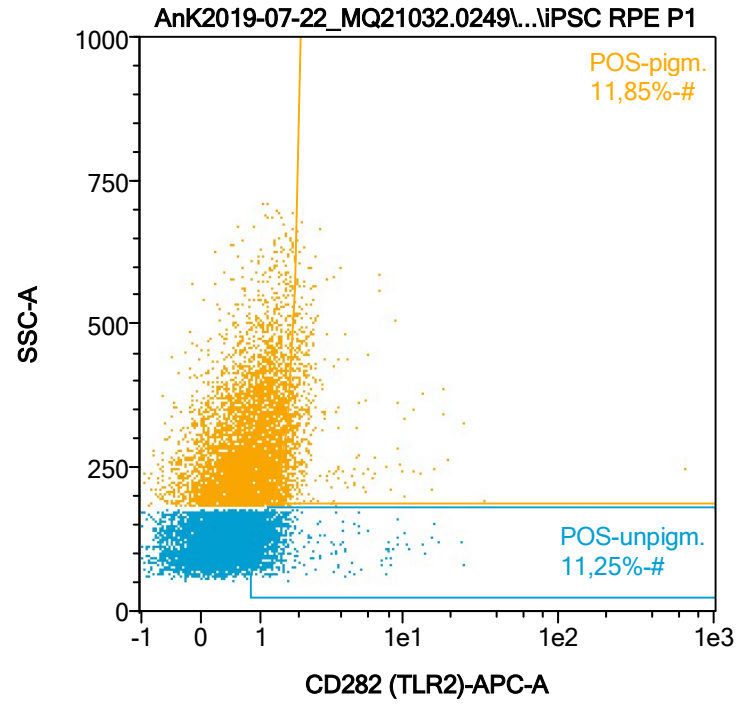

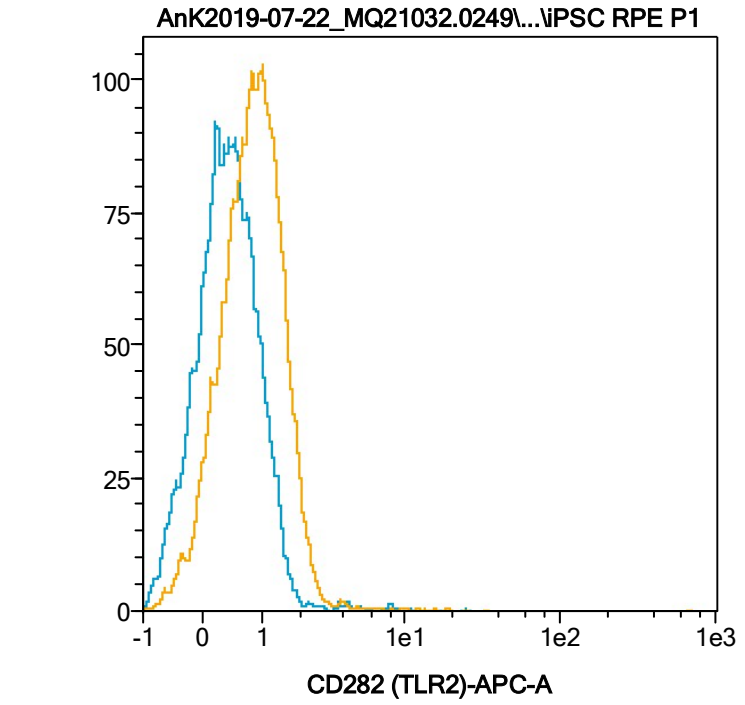

Name	%-#	CD282 (TLR2)-APC-A Mean	CD282 (TLR2)-APC-A Median
POS-unpigm.	11,25	1,80	1,27
POS-pigm.	11,85	3,22	1,86

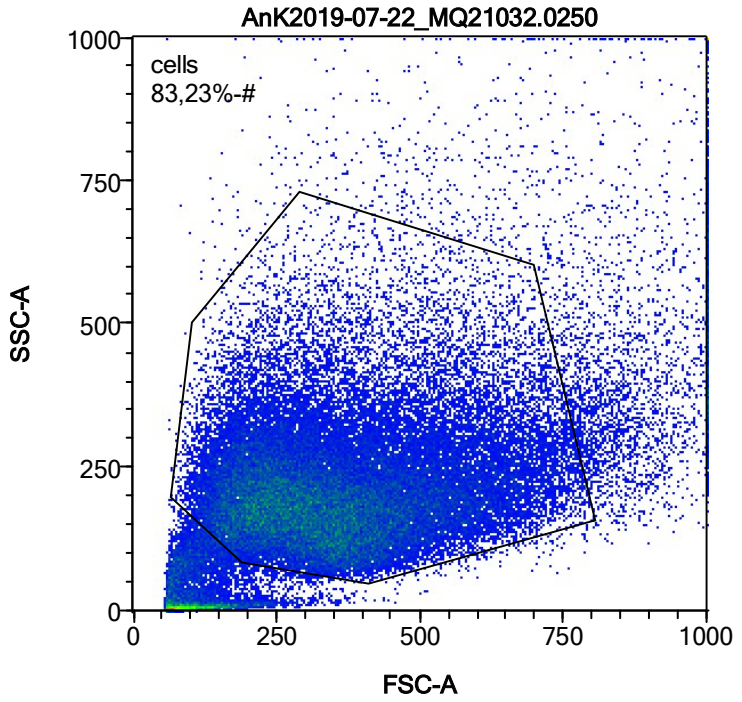

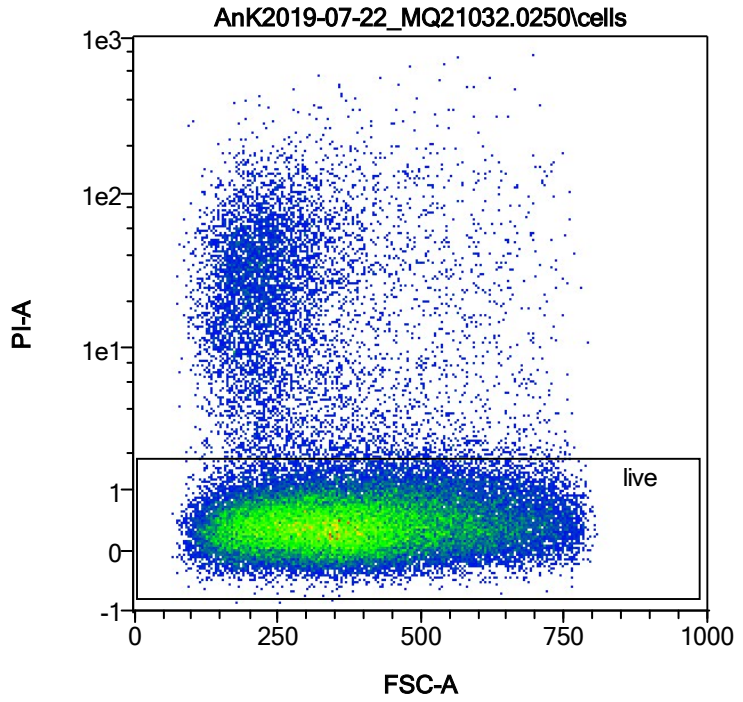

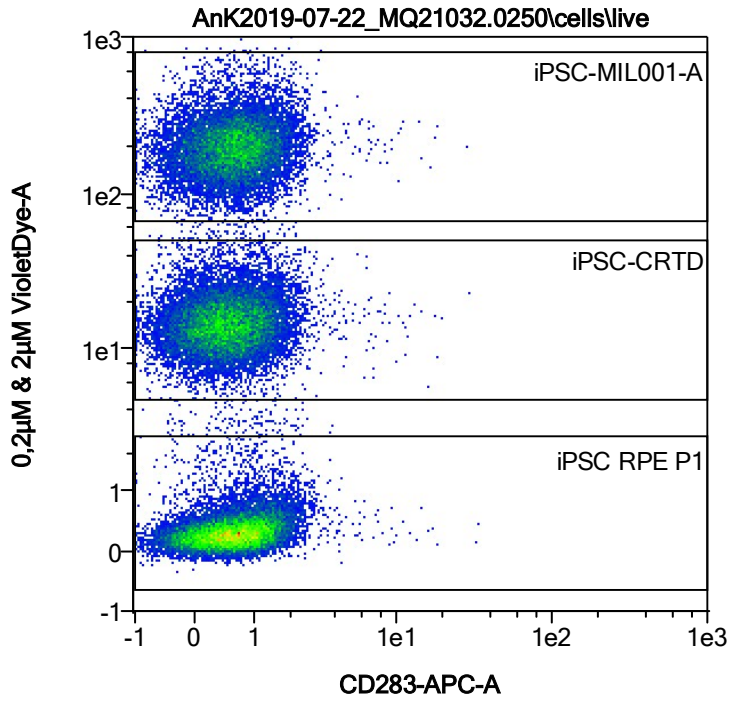

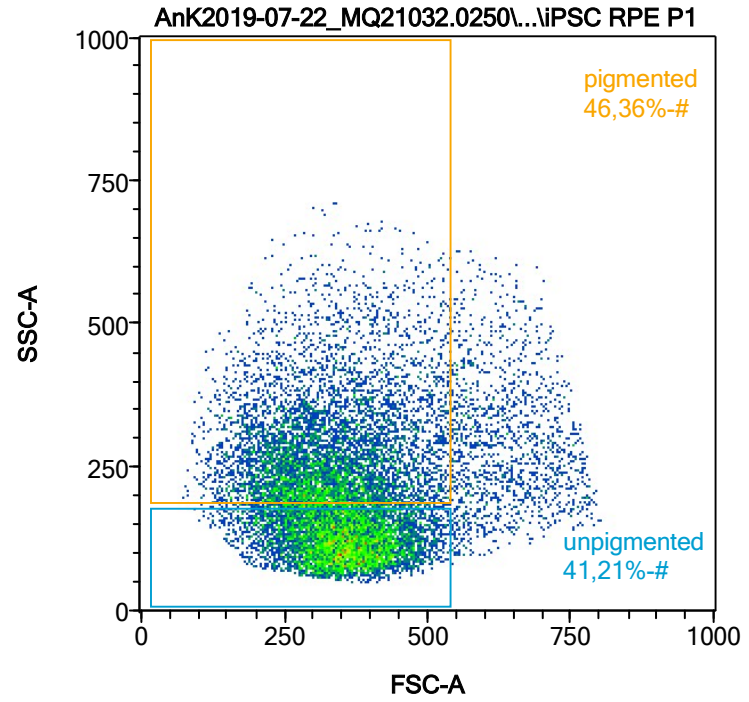

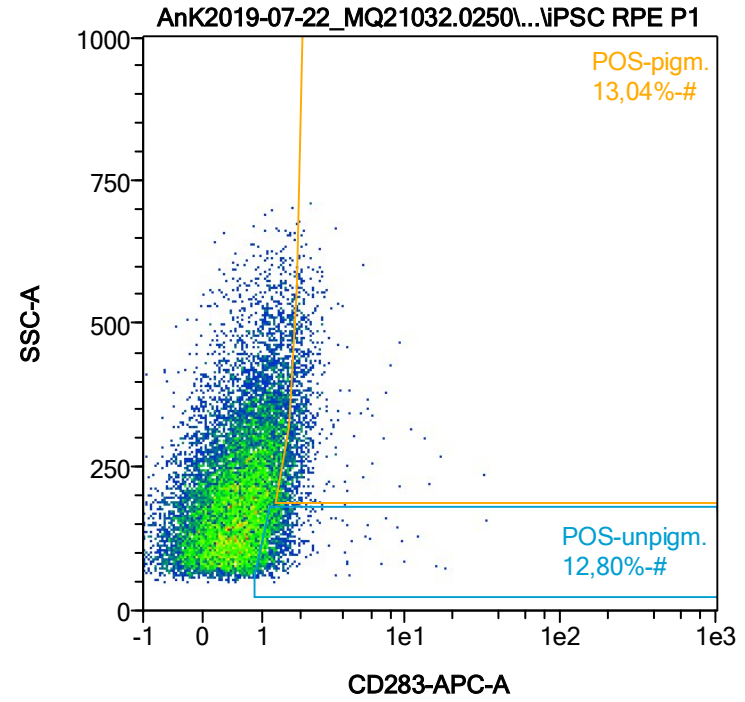

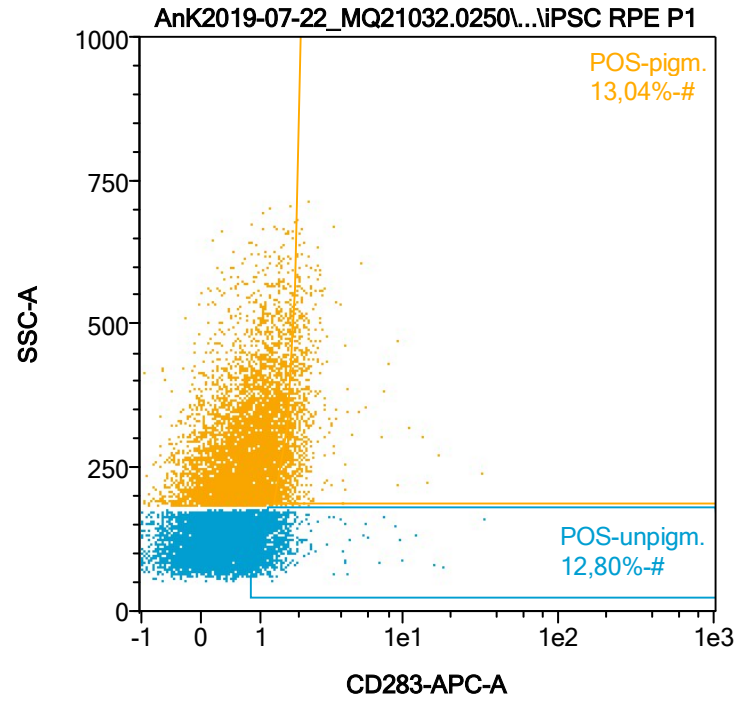

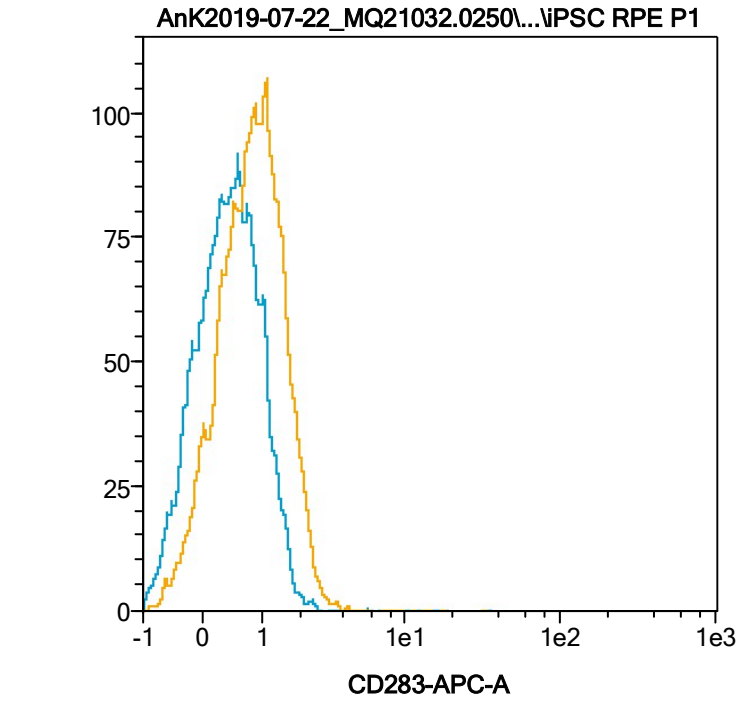

Name	%-#	CD283-APC-A Mean	CD283-APC-A Median
POS-unpigm.	12,80	1,49	1,24
POS-pigm.	13,04	2,09	1,87

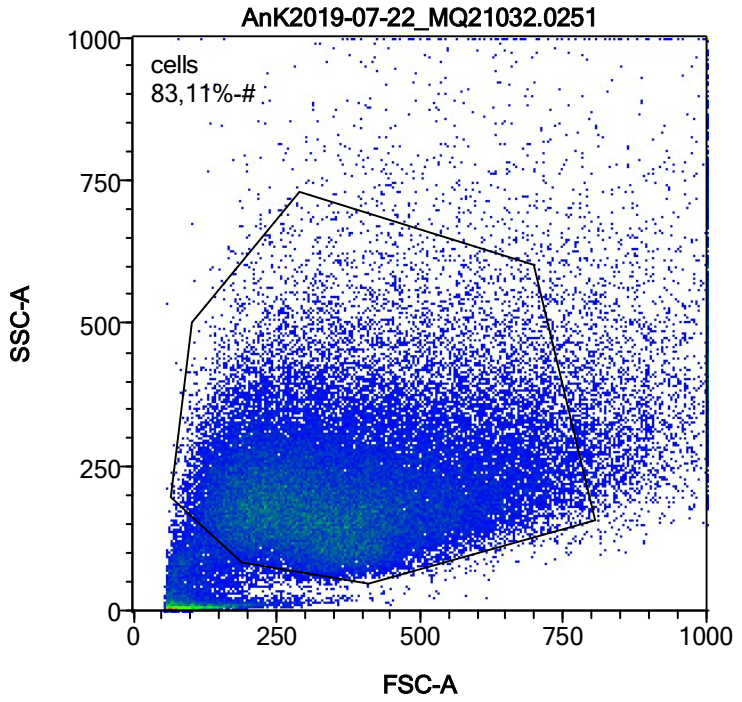

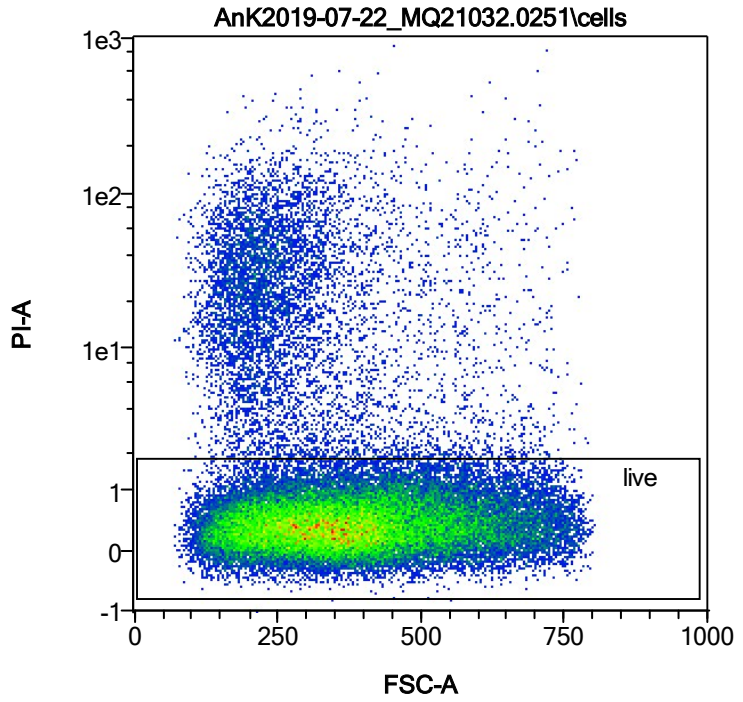

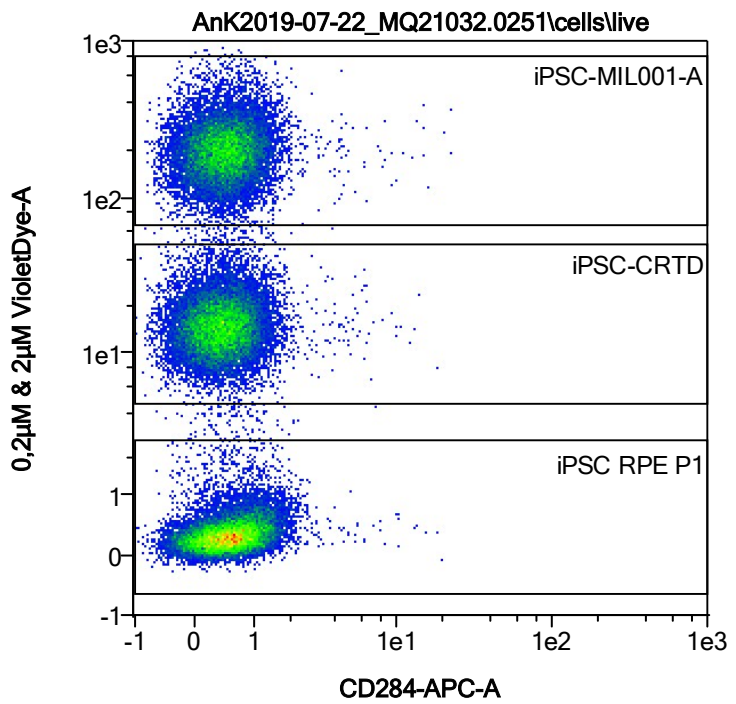

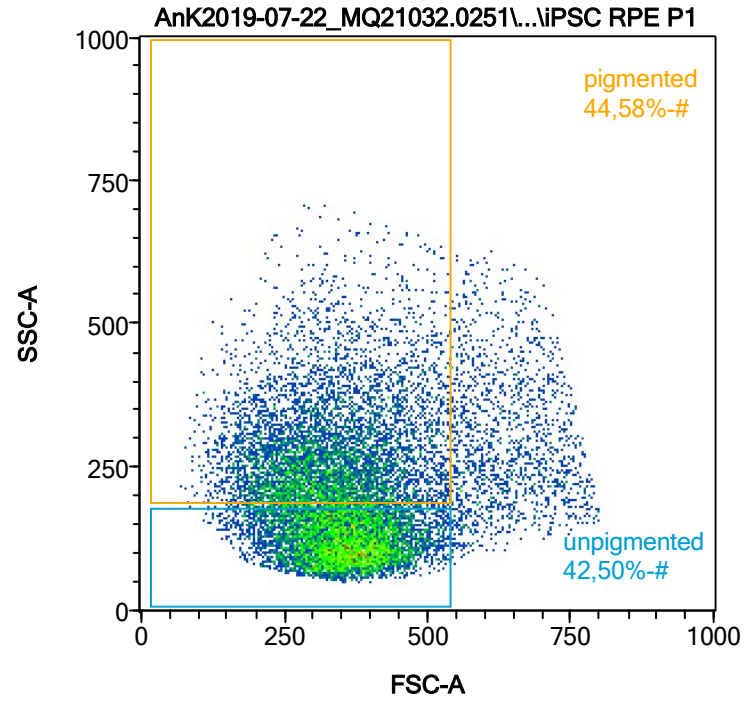

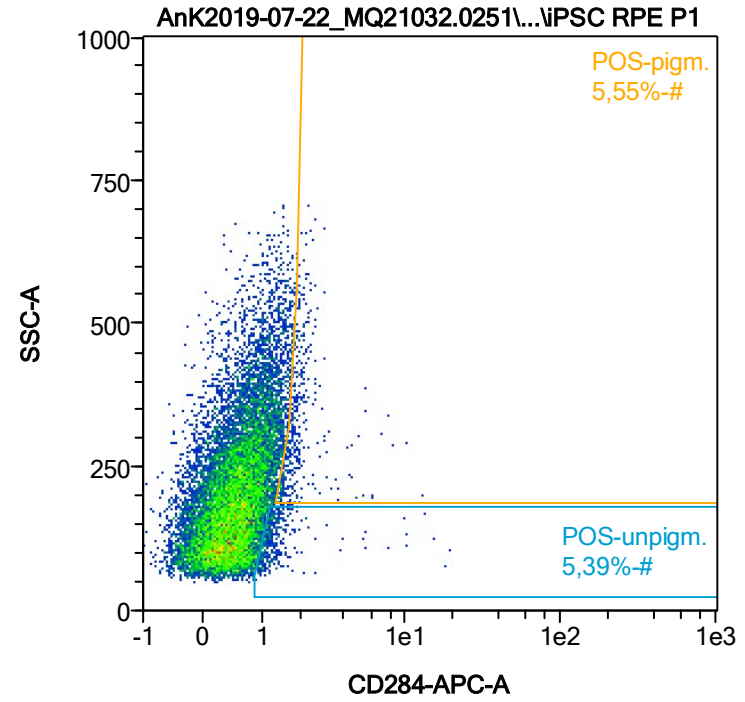

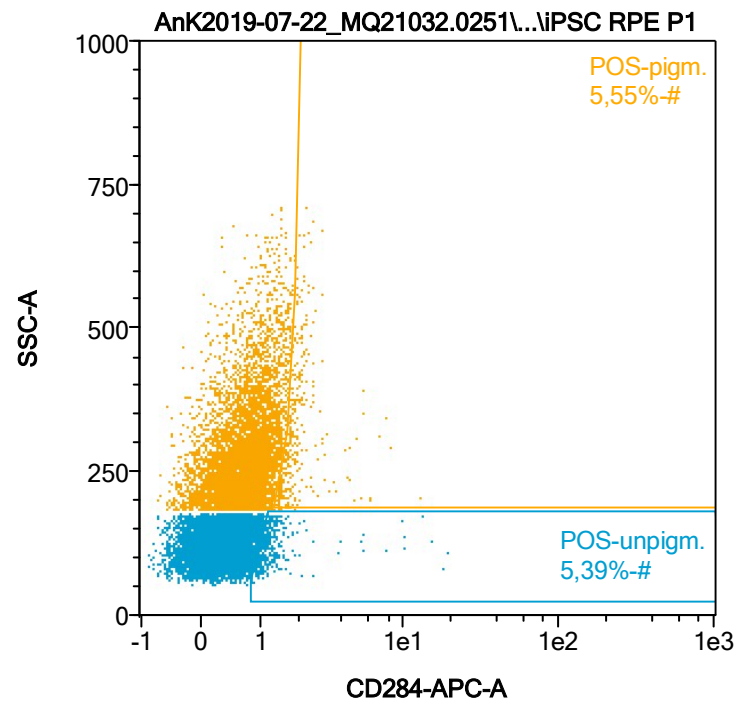

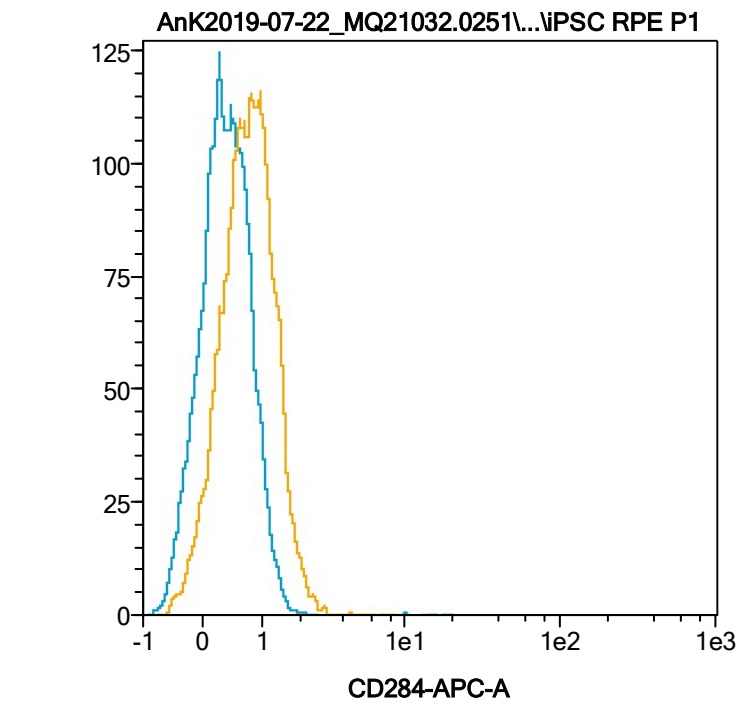

Name	%-#	CD284-APC-A Mean	CD284-APC-A Median
POS-unpigm.	5,39	1,56	1,18
POS-pigm.	5,55	2,04	1,82

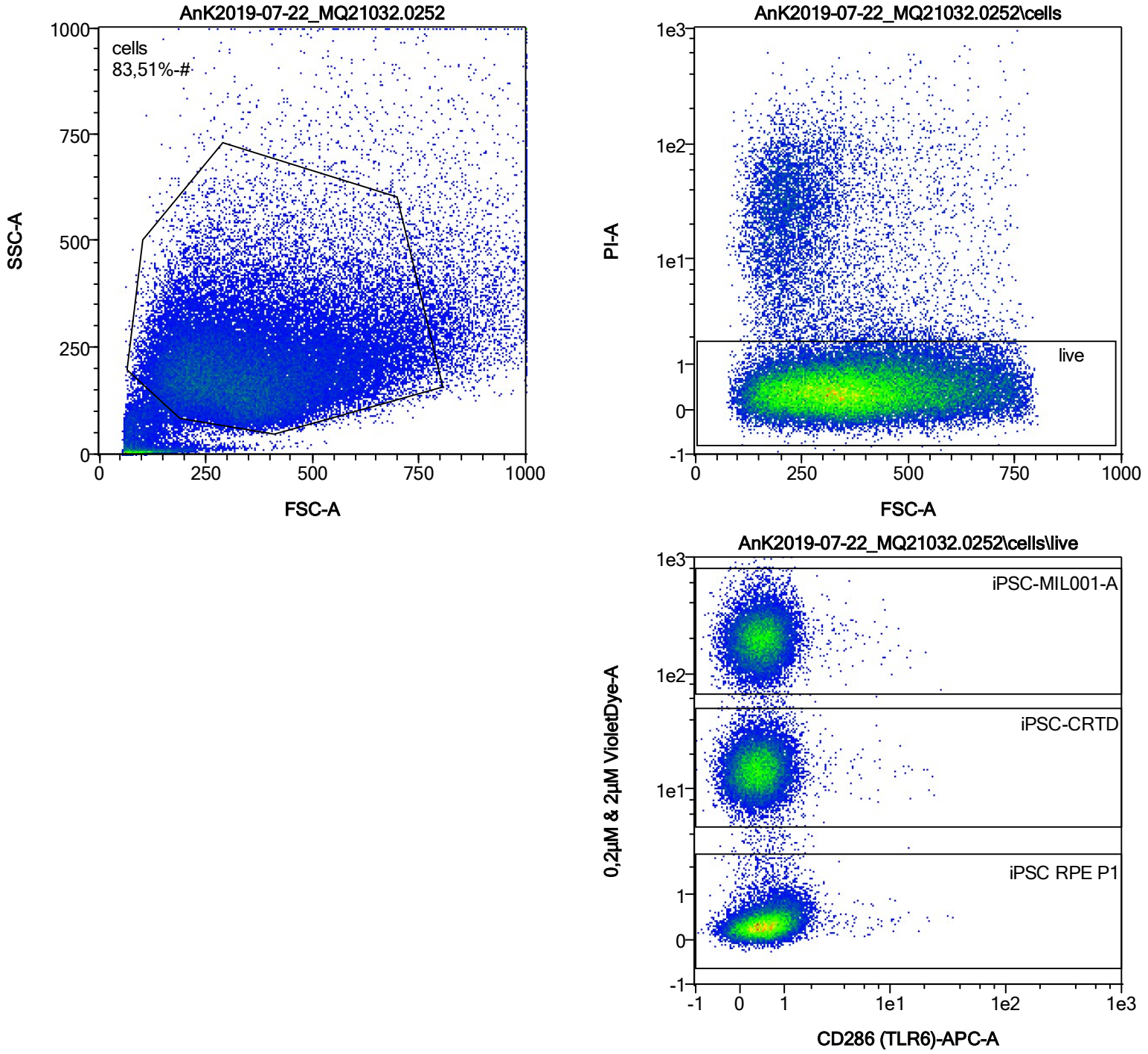

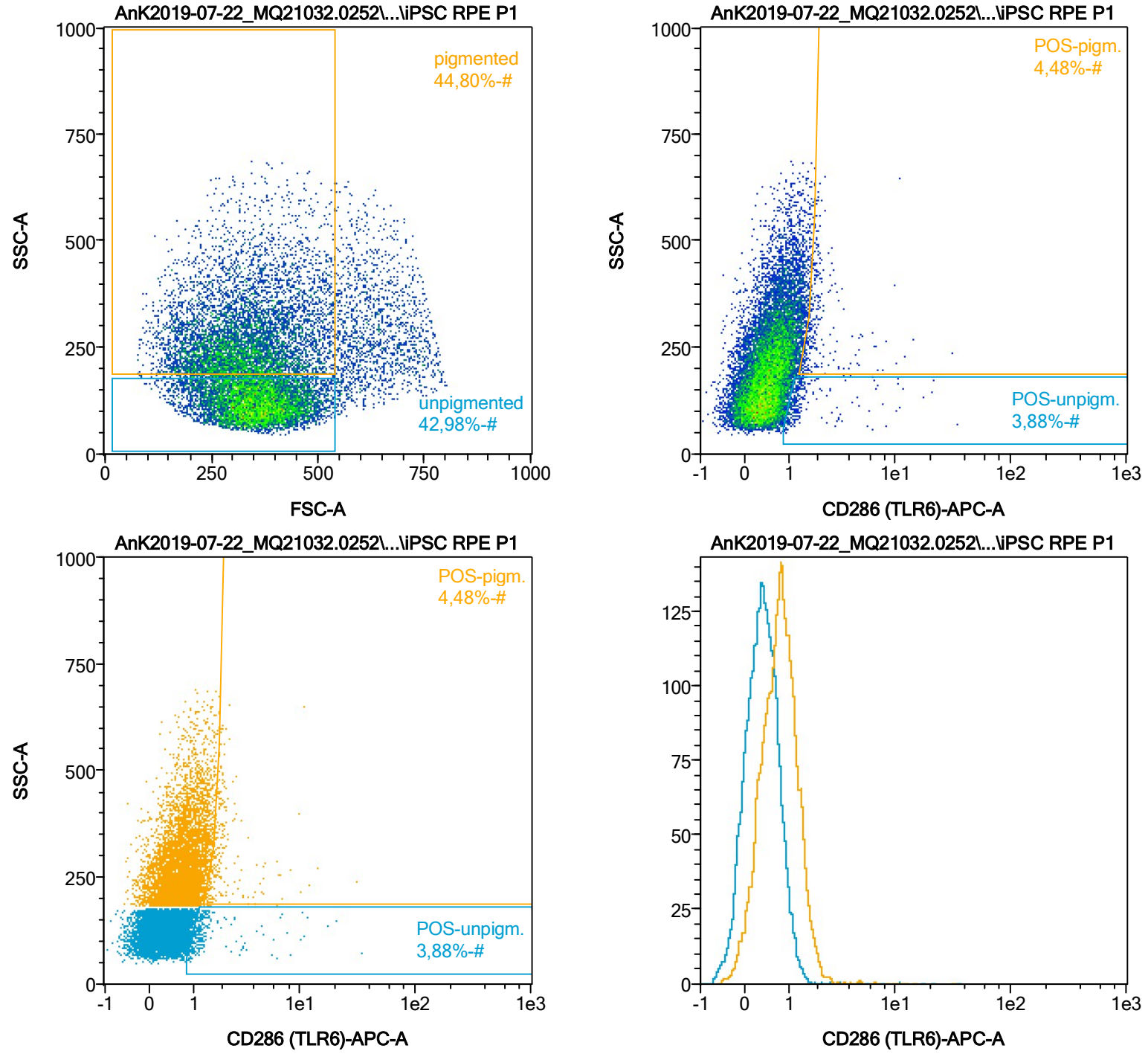

Name	%-#	CD286 (TLR6)-APC-A Mean	CD286 (TLR6)-APC-A Median
POS-unpigm.	3,88	1,92	1,17
POS-pigm.	4,48	2,32	1,81

Date:2024-06-14

Operator: Sebastian Knoebel

Version:2.13.0+54.g95abf0f4

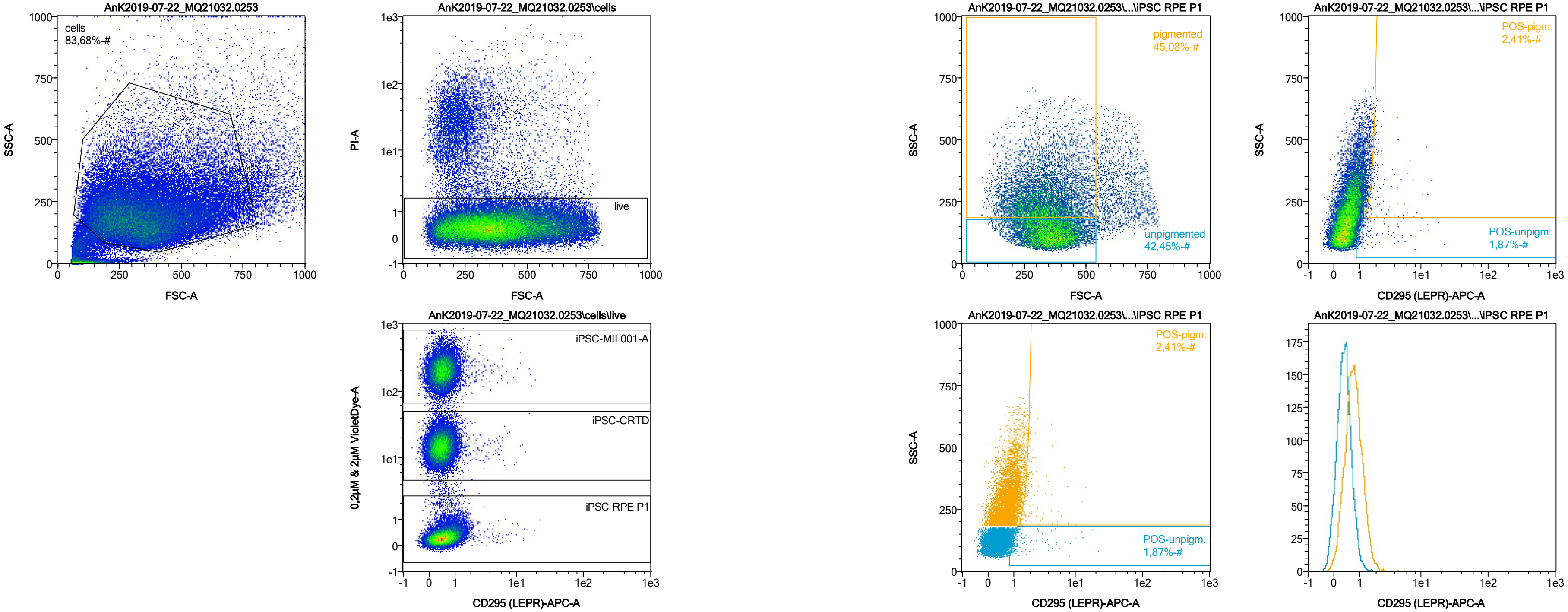

Name	%-#	CD295 (LEPR)-APC-A Mean	CD295 (LEPR)-APC-A Median
POS-unpigm.	1,87	2,37	1,23
POS-pigm.	2,41	2,45	1,90

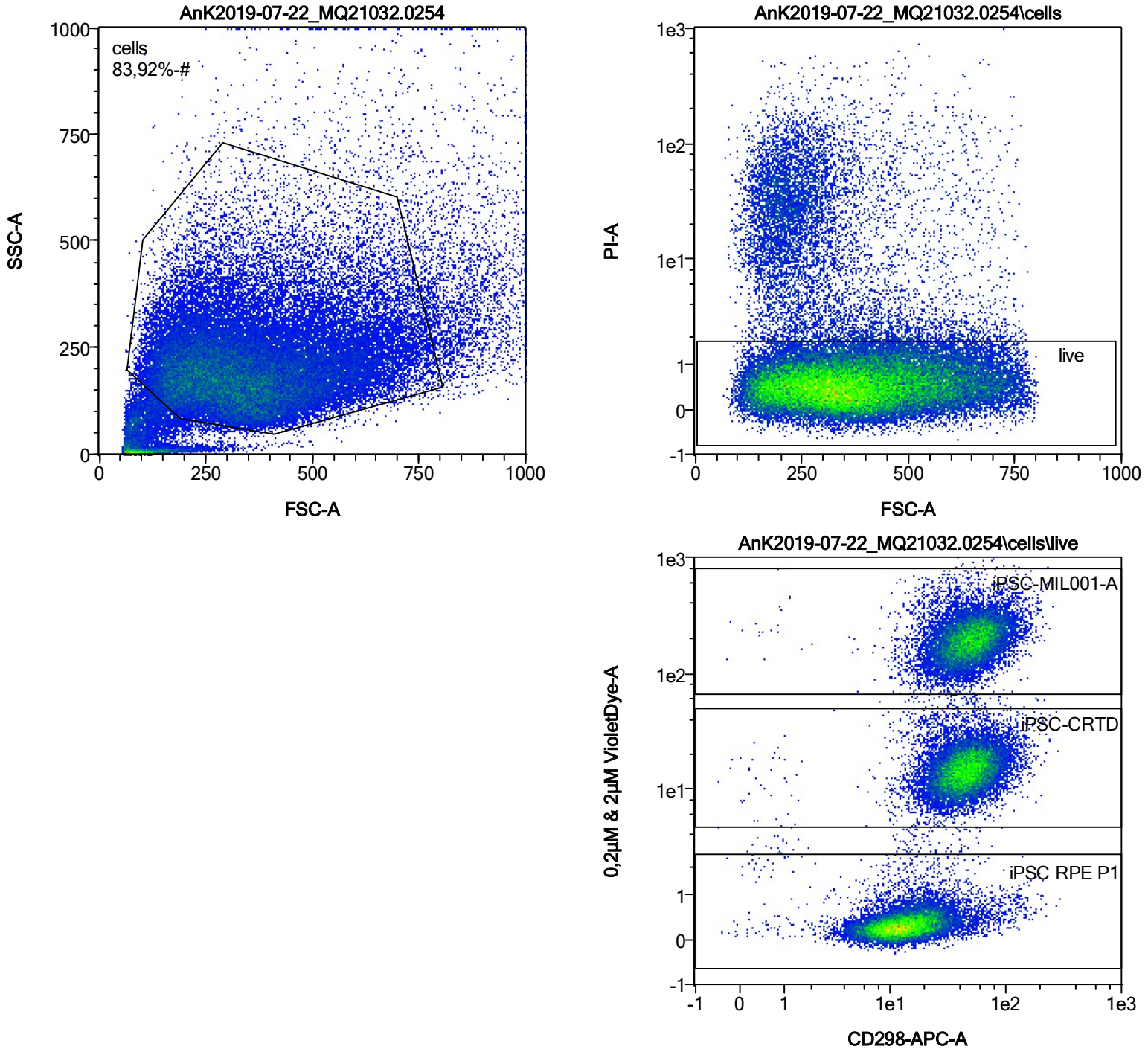

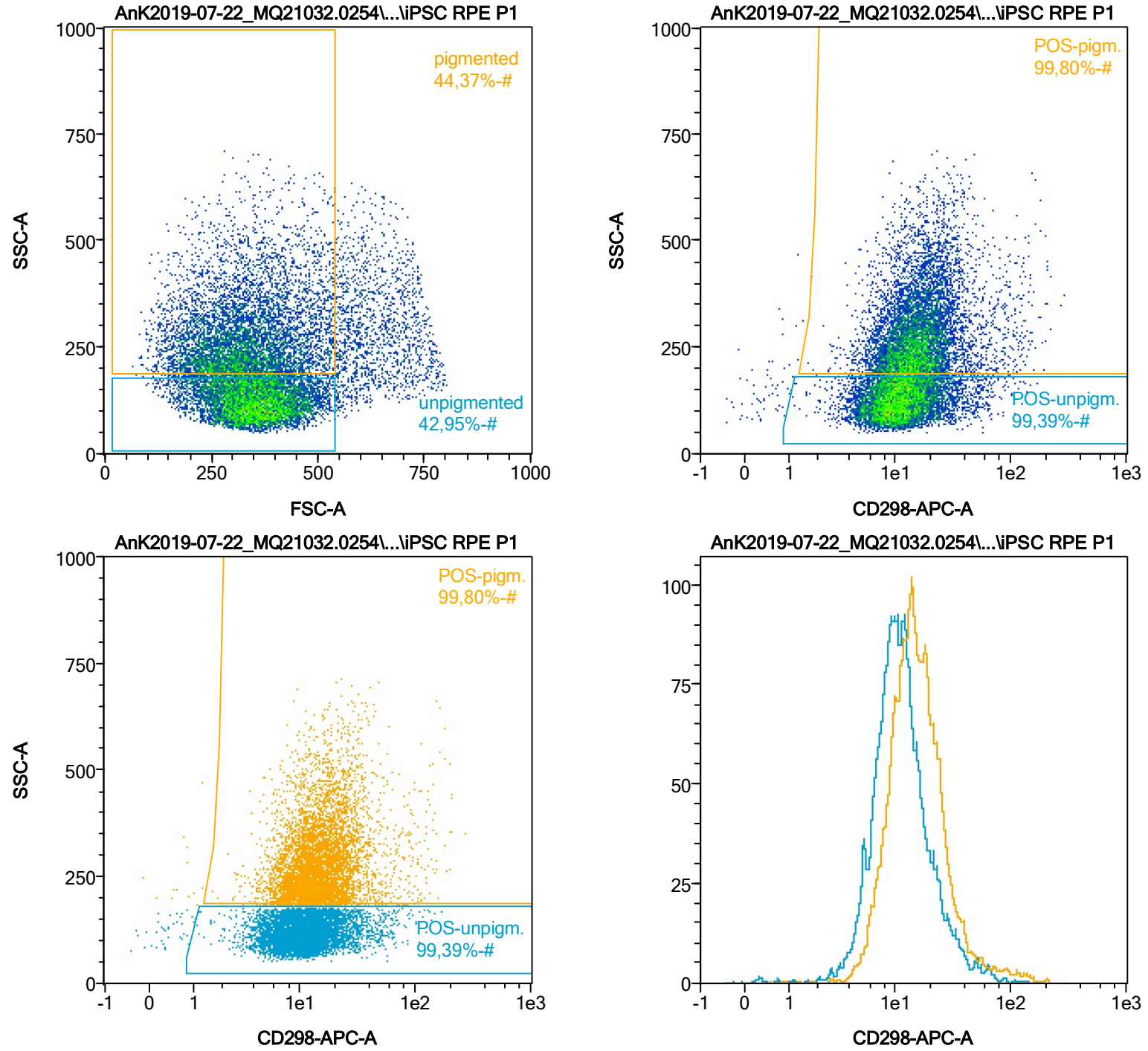

Name	%-#	CD298-APC-A Mean	CD298-APC-A Median
POS-unpigm.	99,39	13,36	10,72
POS-pigm.	99,80	18,43	14,62

Date:2024-06-14
Operator: Sebastian Knoebel
Version:2.13.0+54.g95abf0f4

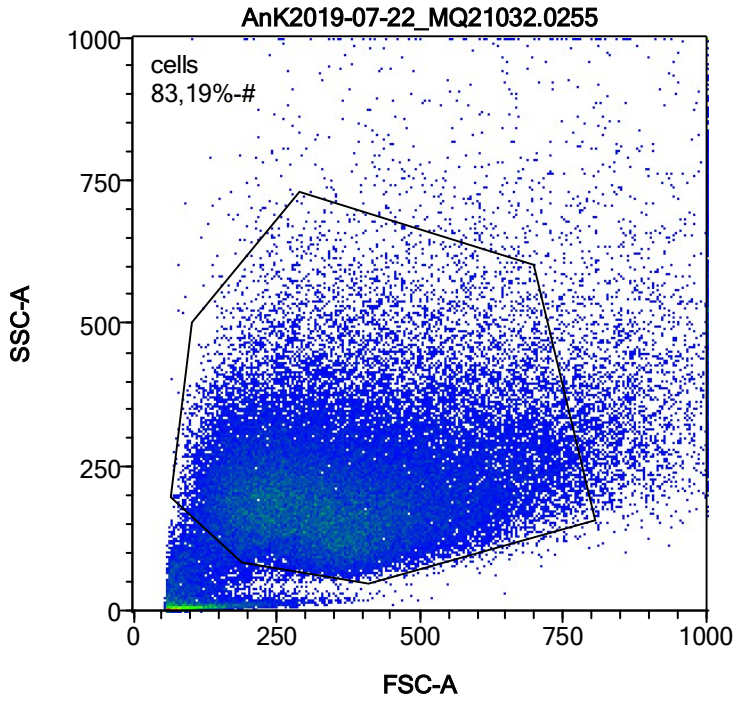

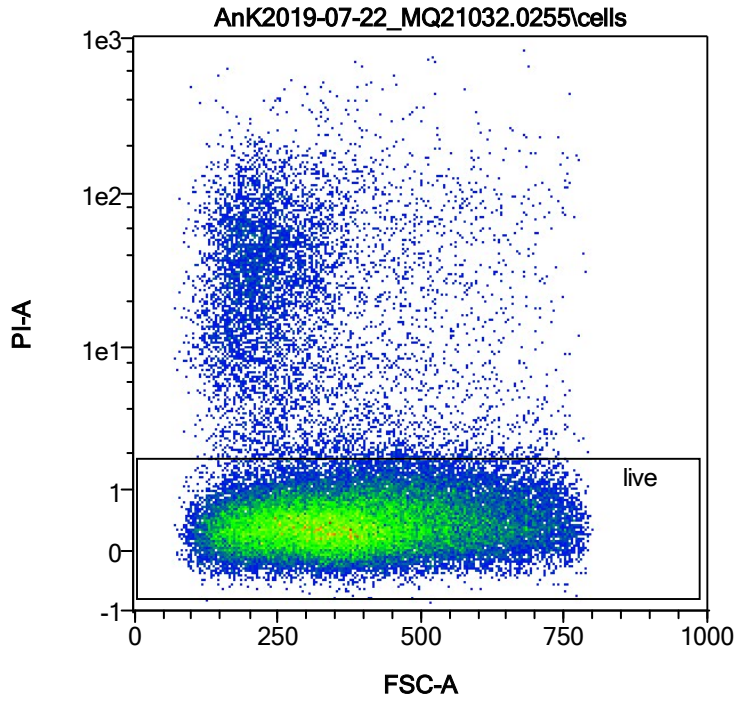

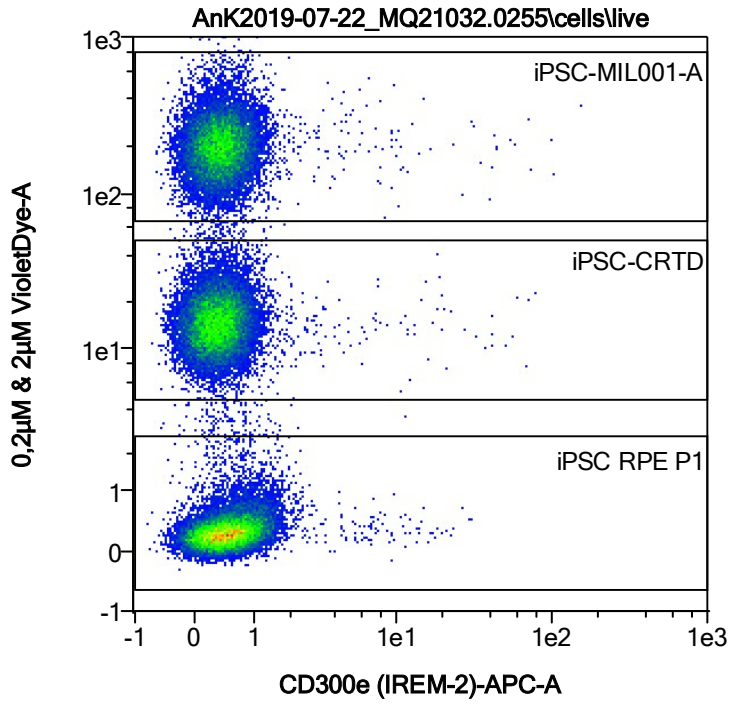

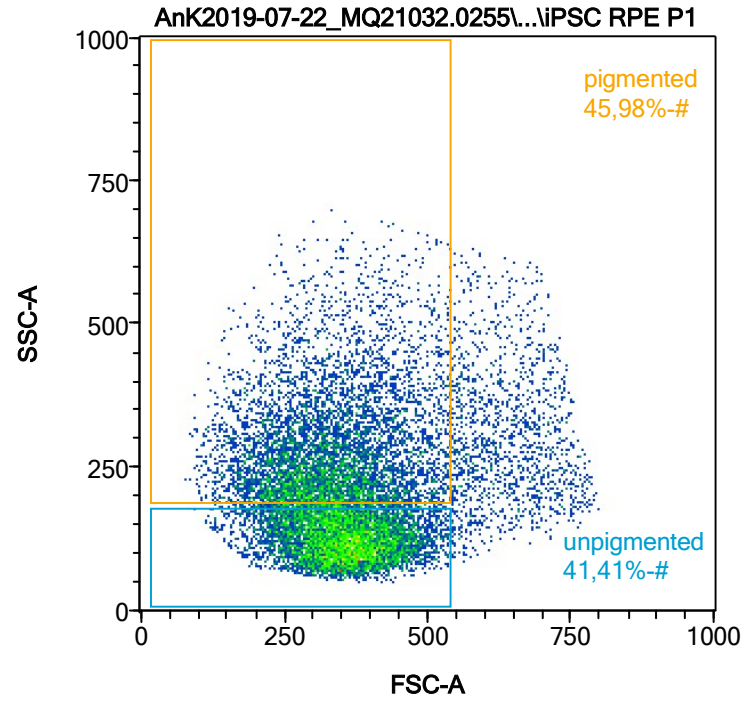

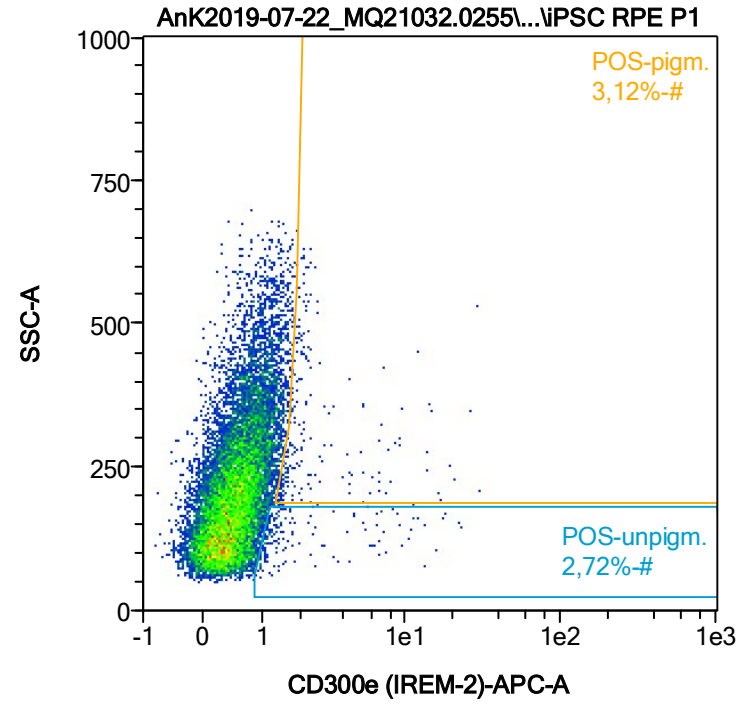

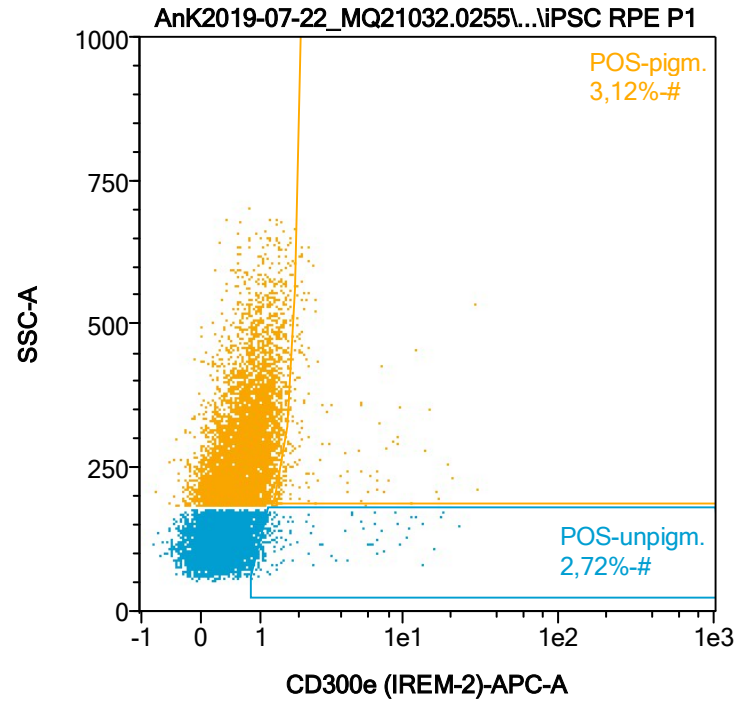

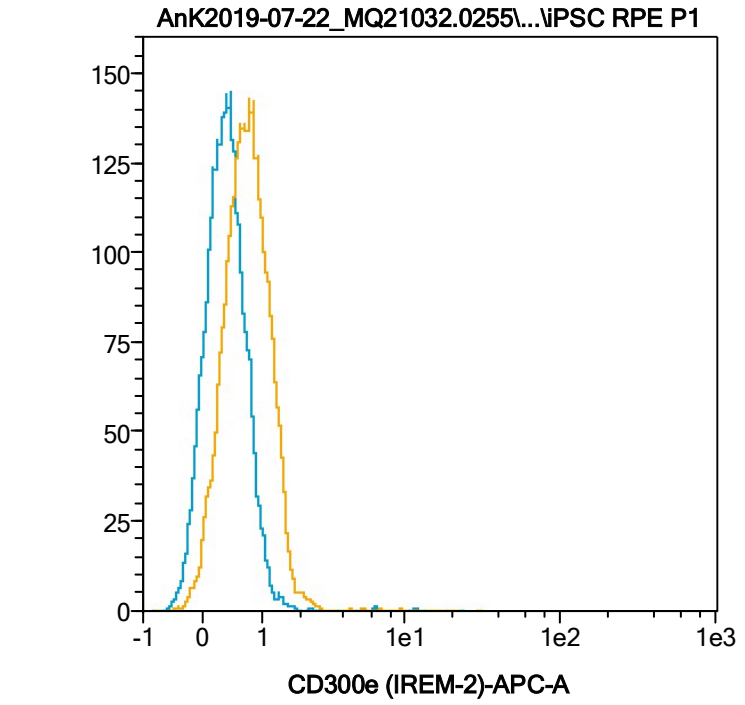

Name	%-#	CD300e (IREM-2)-APC-A Mean	CD300e (IREM-2)-APC-A Median
POS-unpigm.	2,72	2,77	1,29
POS-pigm.	3,12	3,21	1,87

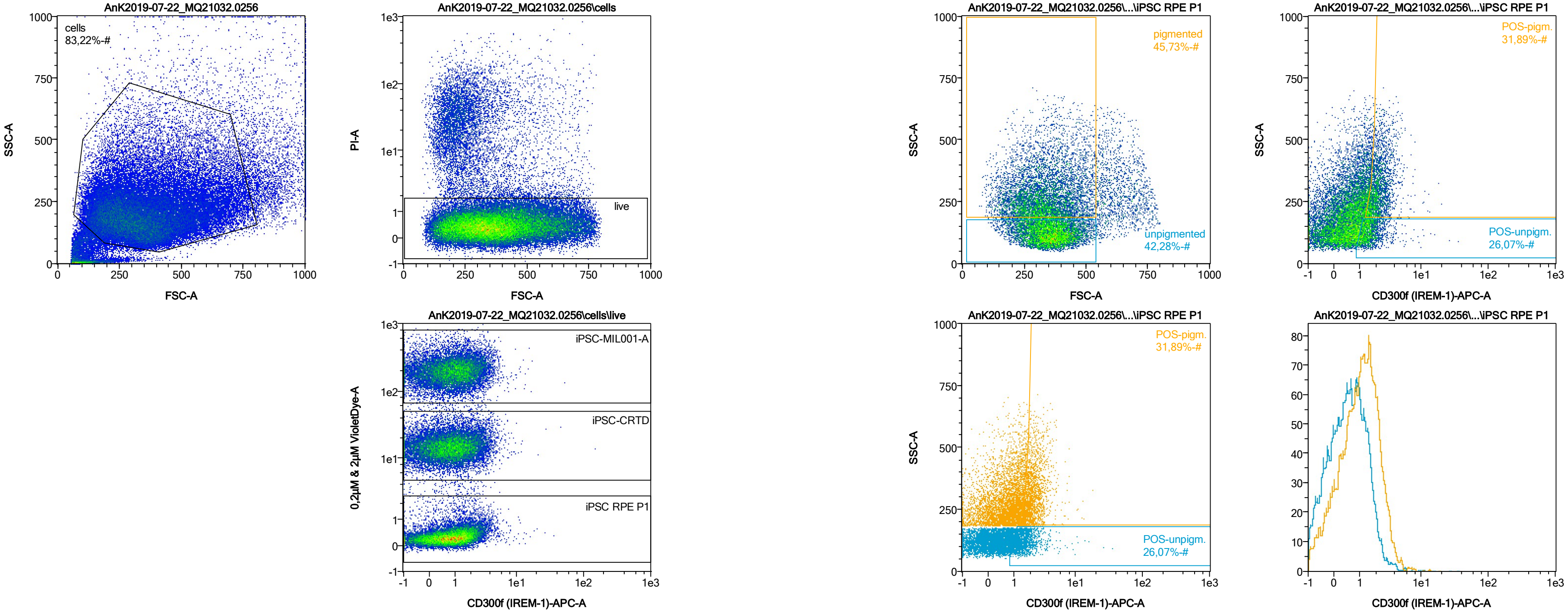

Name	%-#	CD300f (IREM-1)-APC-A Mean	CD300f (IREM-1)-APC-A Median
POS-unpigm.	26,07	1,68	1,44
POS-pigm.	31,89	2,32	2,08

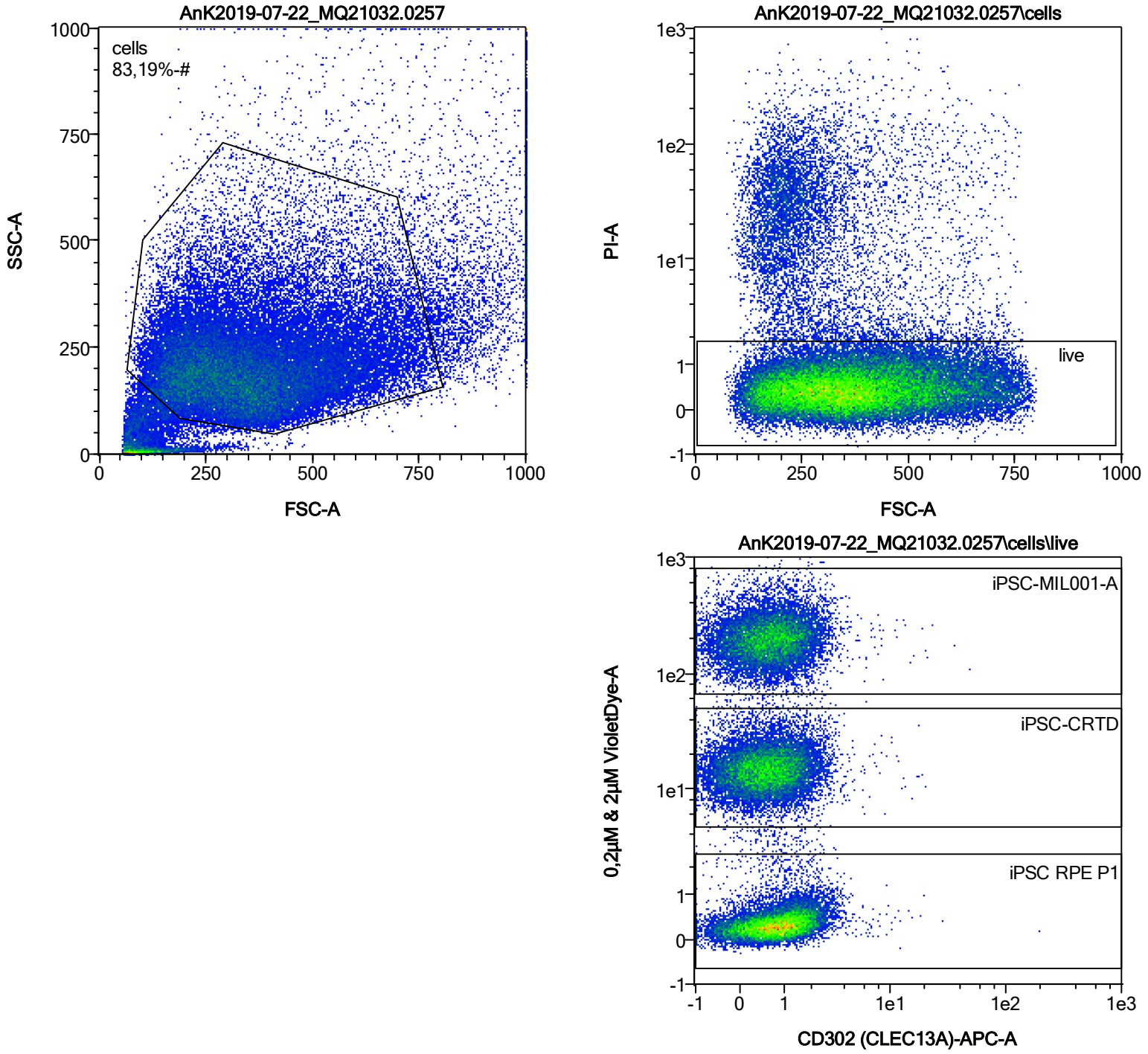

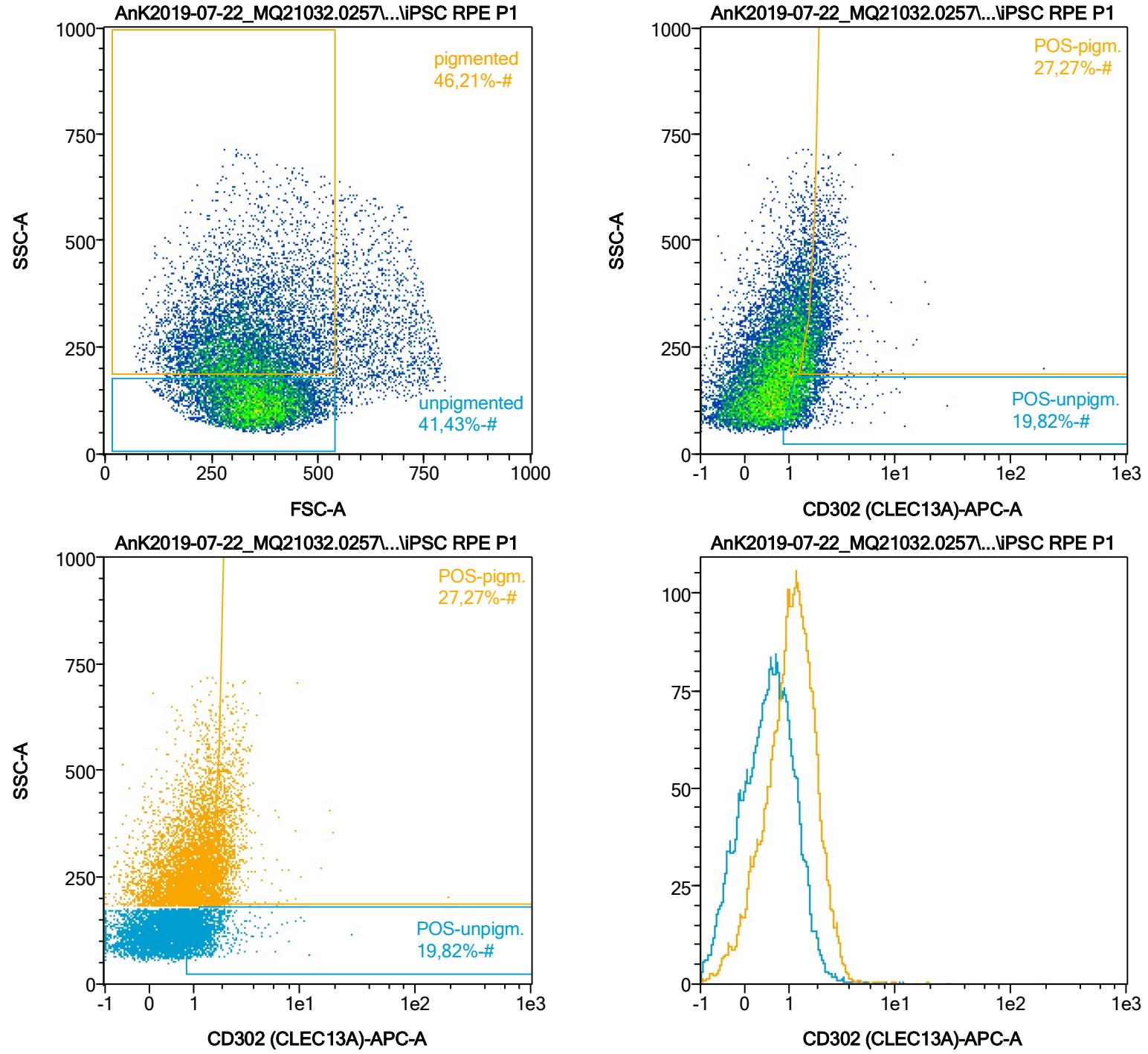

Name	%-#	CD302 (CLEC13A)-APC-A Mean	CD302 (CLEC13A)-APC-A Median
POS-unpigm.	19,82	1,53	1,30
POS-pigm.	27,27	2,22	1,92

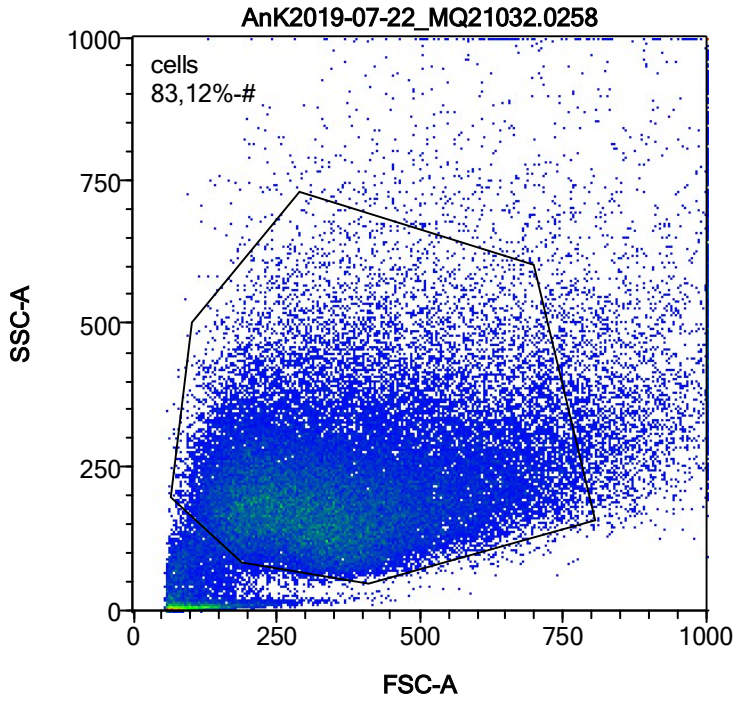

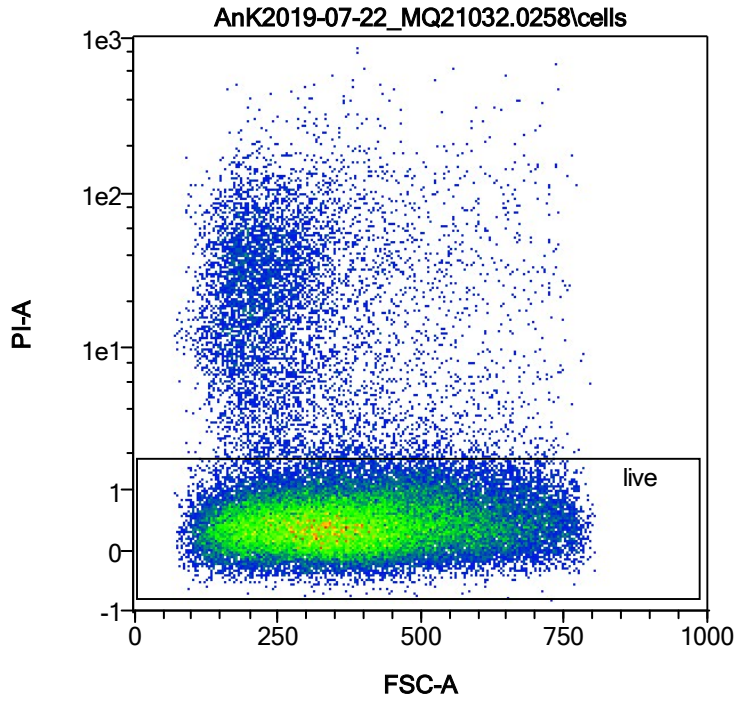

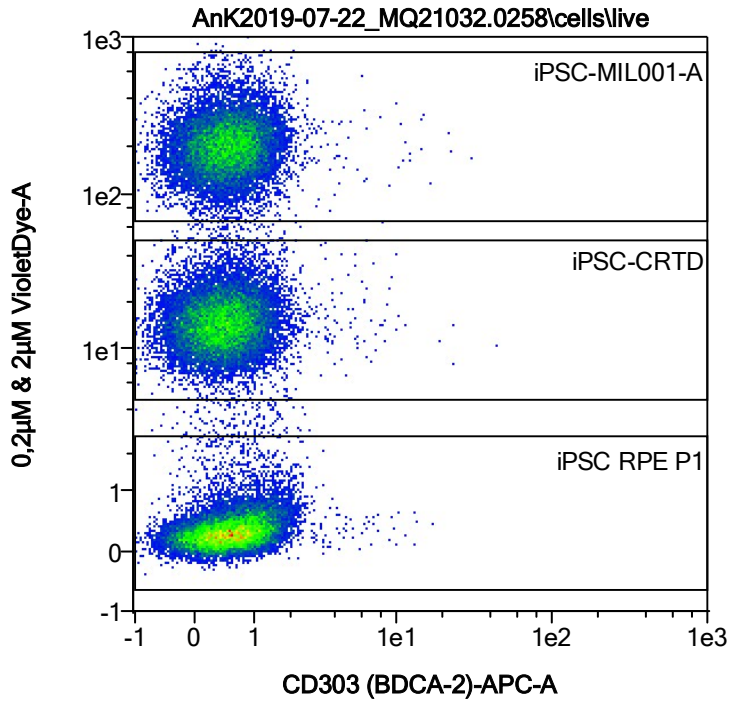

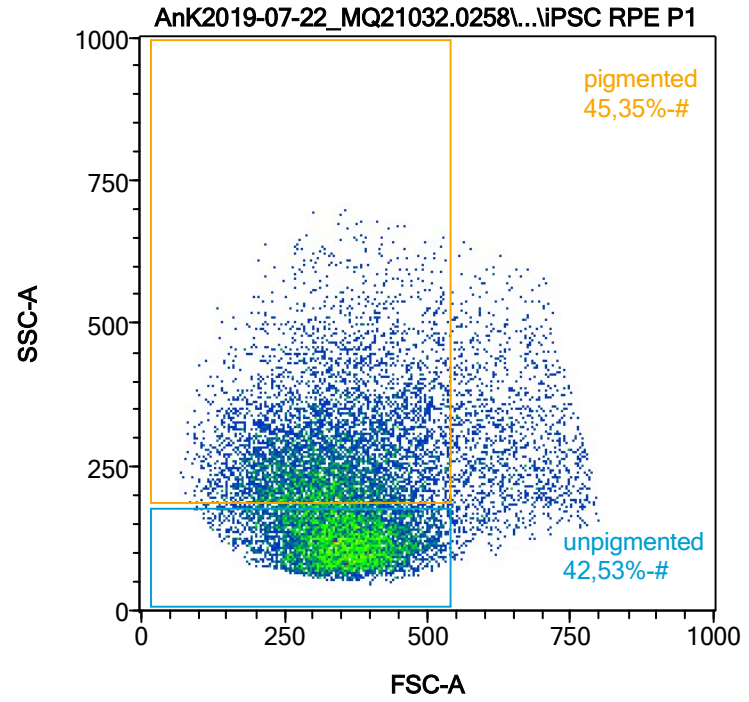

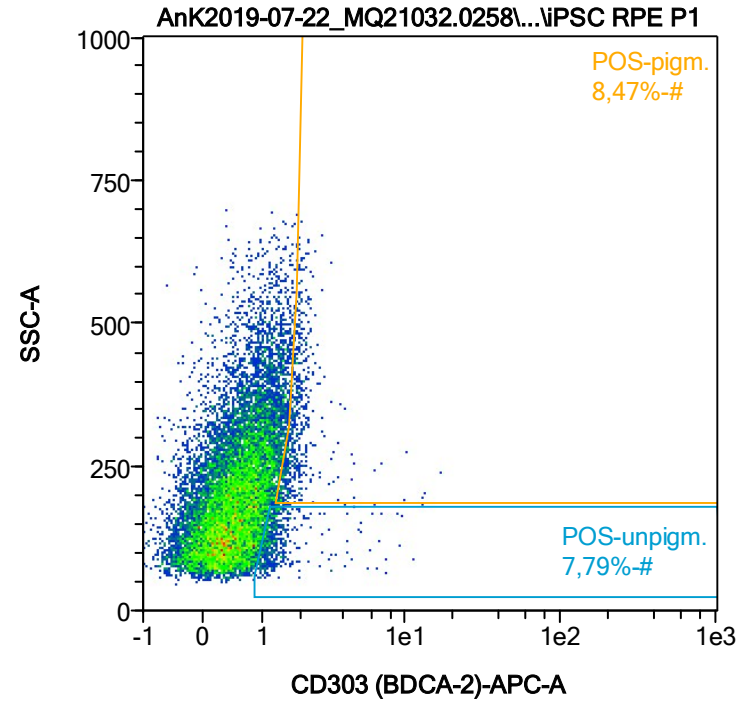

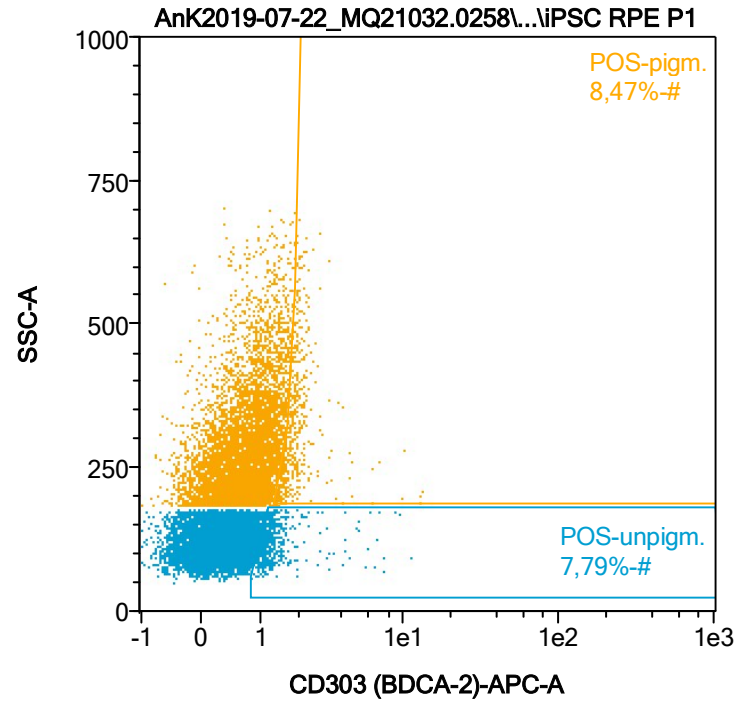

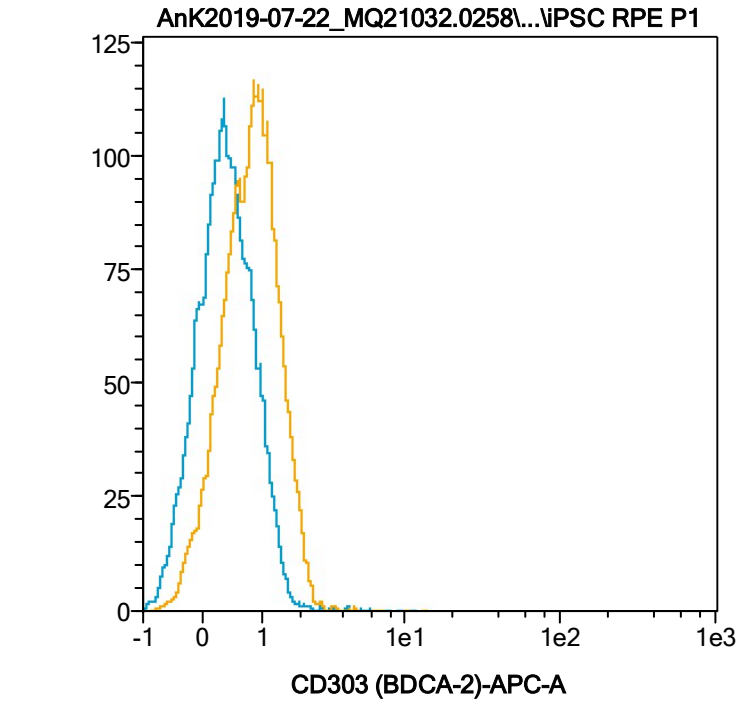

Name	%-#	CD303 (BDCA-2)-APC-A Mean	CD303 (BDCA-2)-APC-A Median
POS-unpigm.	7,79	1,50	1,22
POS-pigm.	8,47	2,00	1,82

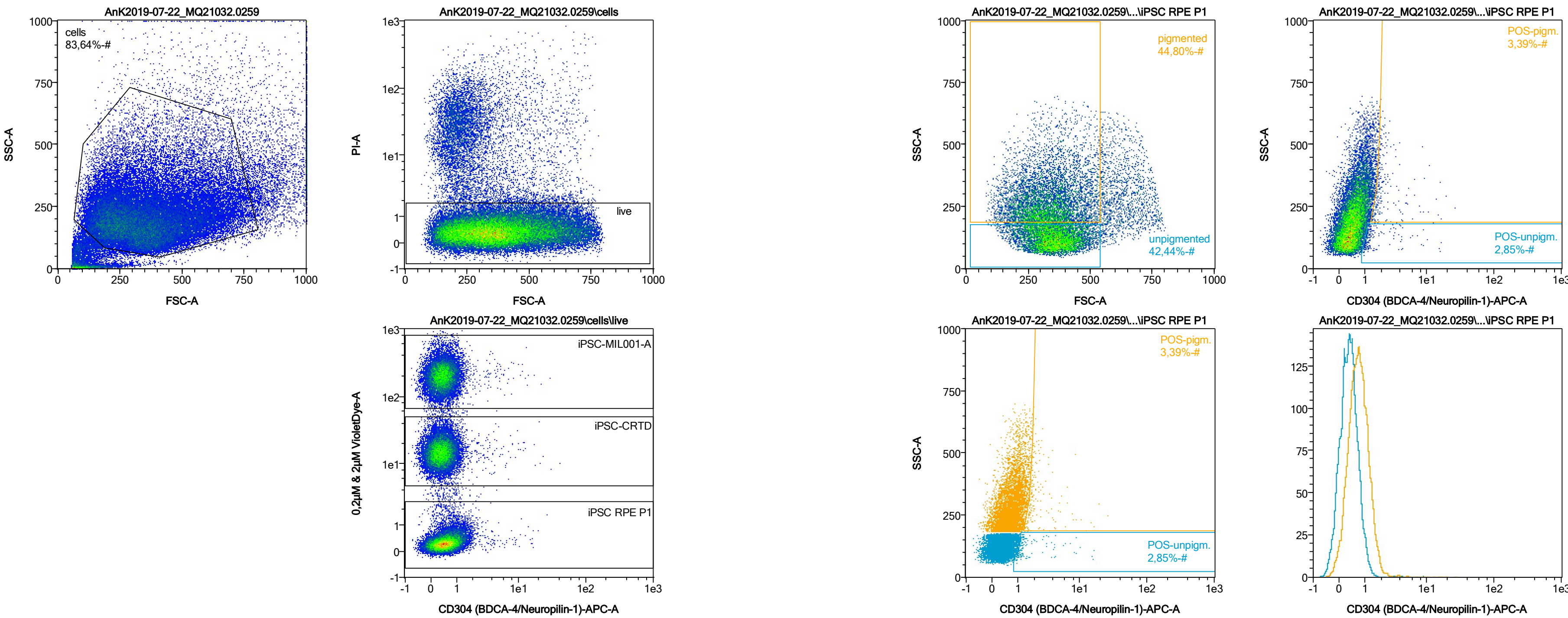

Name	%-#	CD304 (BDCA-4/Neuropilin-1)-APC-A Mean	CD304 (BDCA-4/Neuropilin-1)-APC-A Median
POS-unpigm.	2,85	1,92	1,17
POS-pigm.	3,39	2,64	1,86

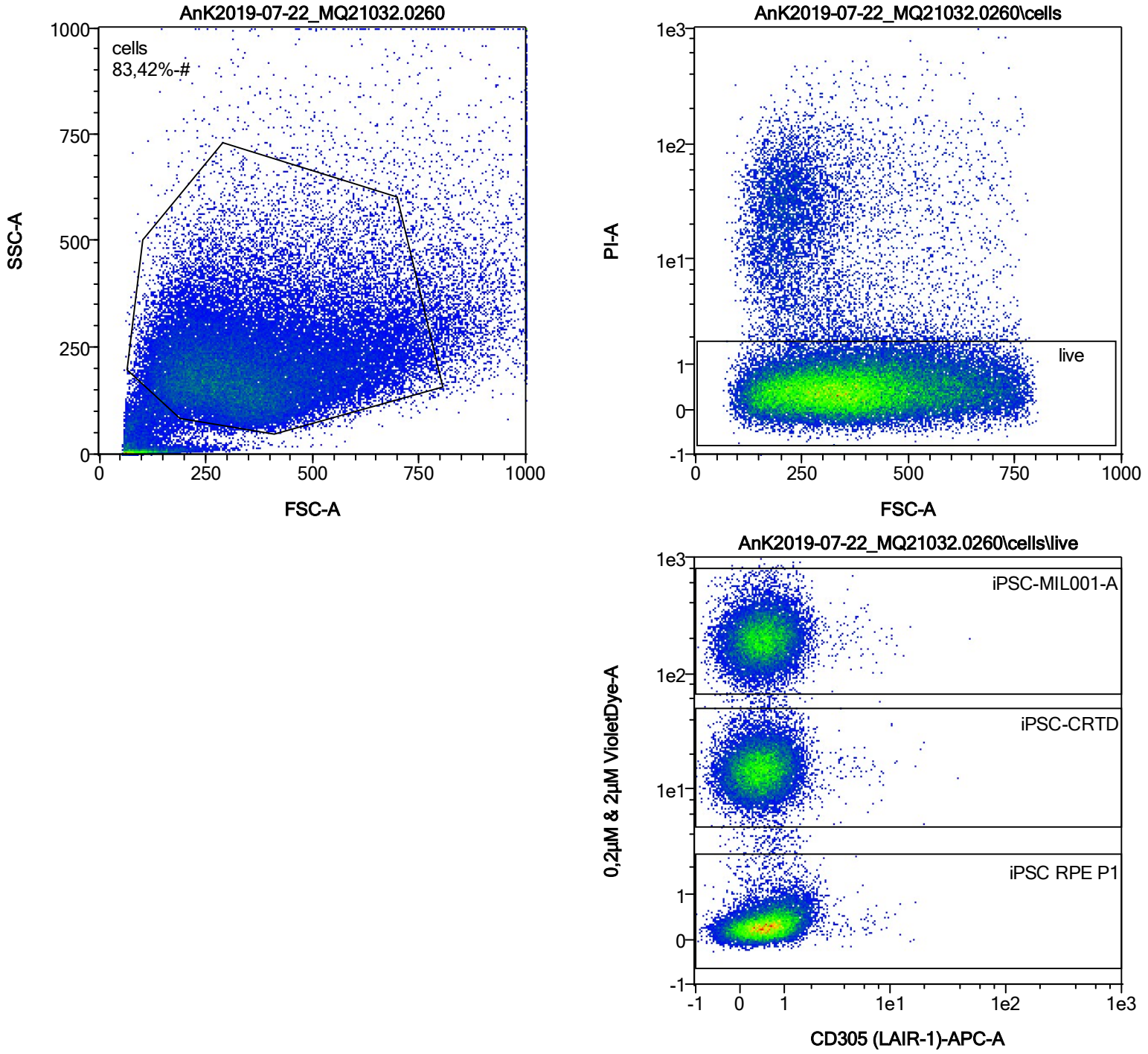

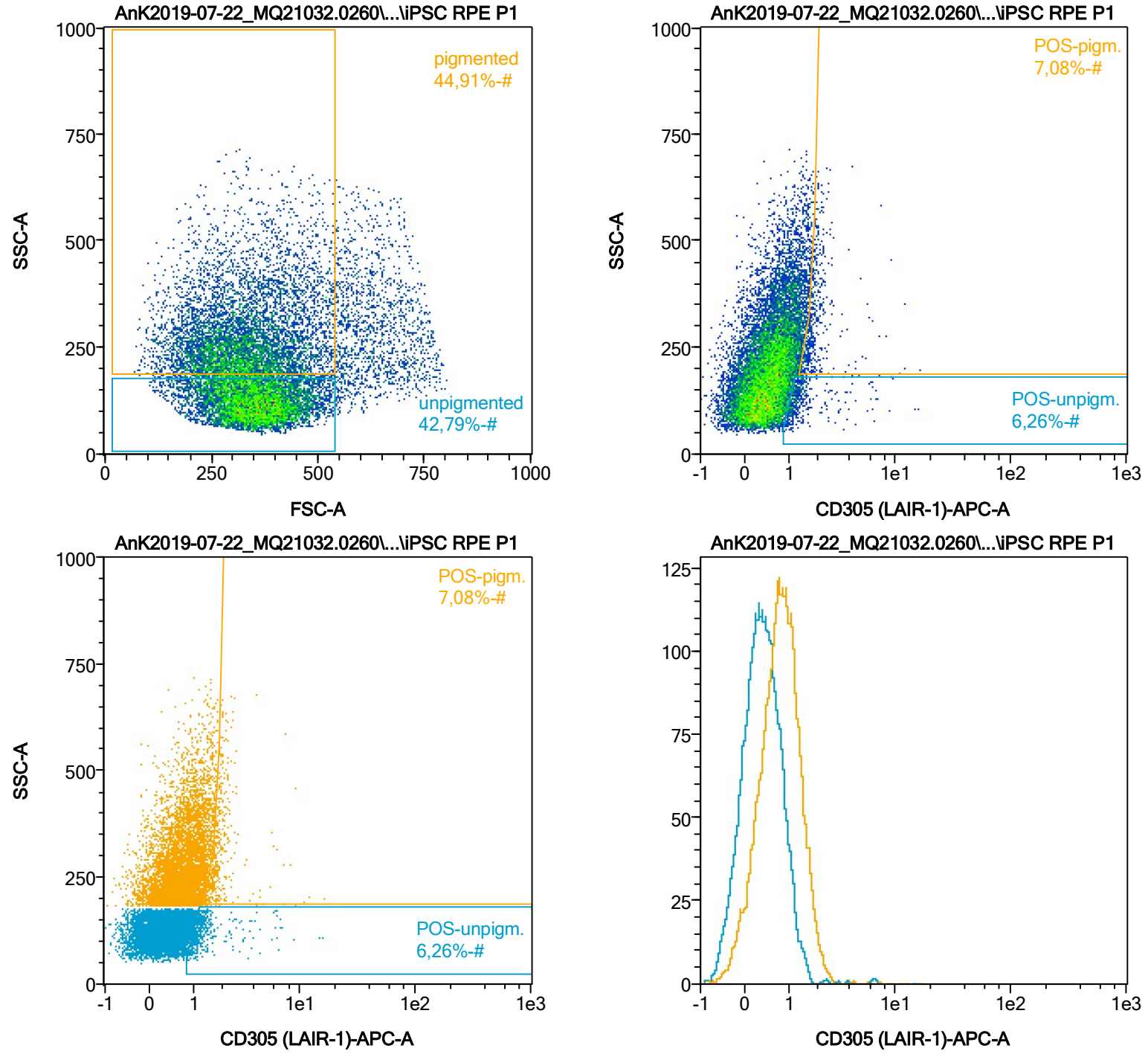

Name	%-#	CD305 (LAIR-1)-APC-A Mean	CD305 (LAIR-1)-APC-A Median
POS-unpigm.	6,26	1,63	1,21
POS-pigm.	7,08	2,07	1,81

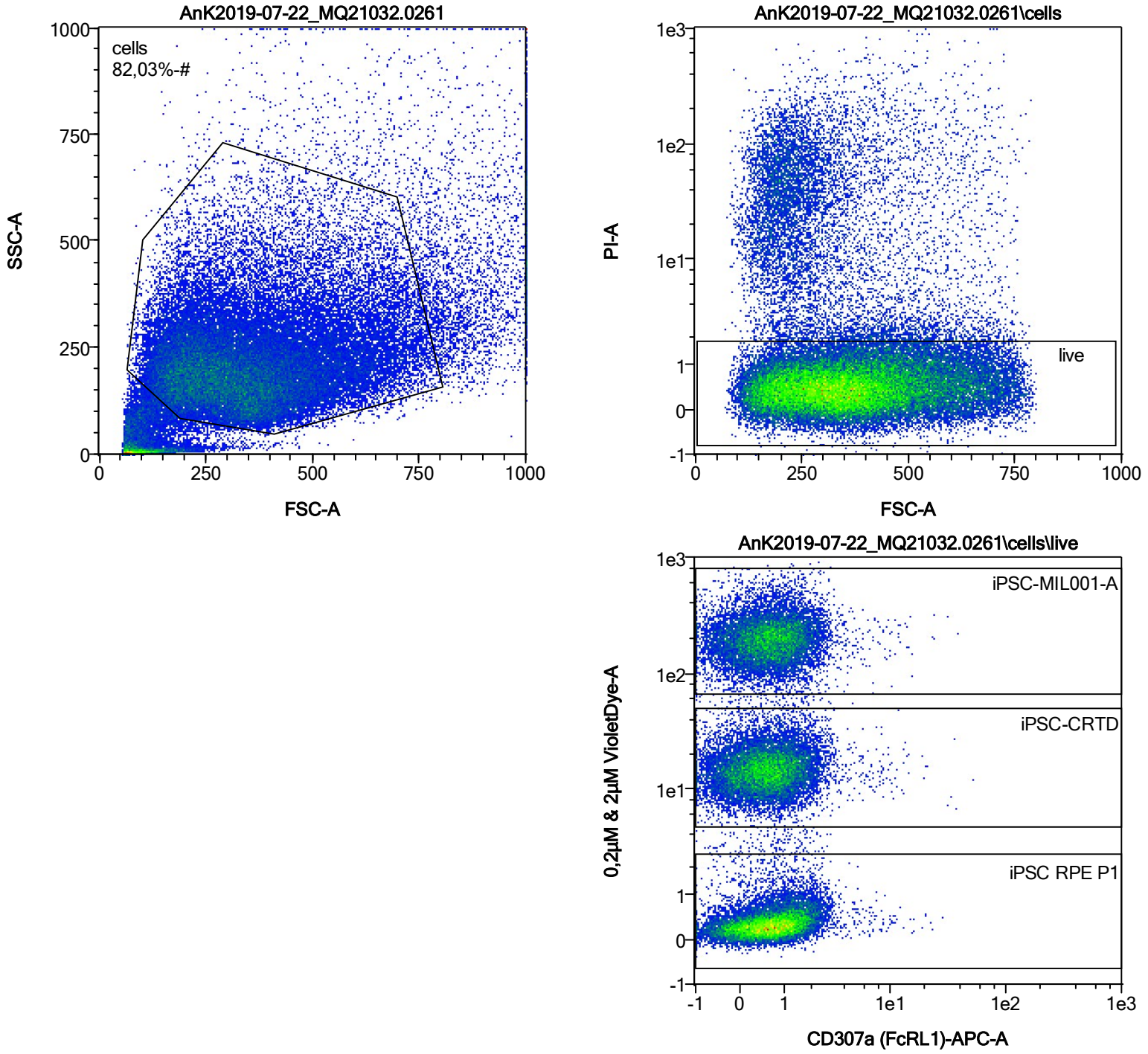

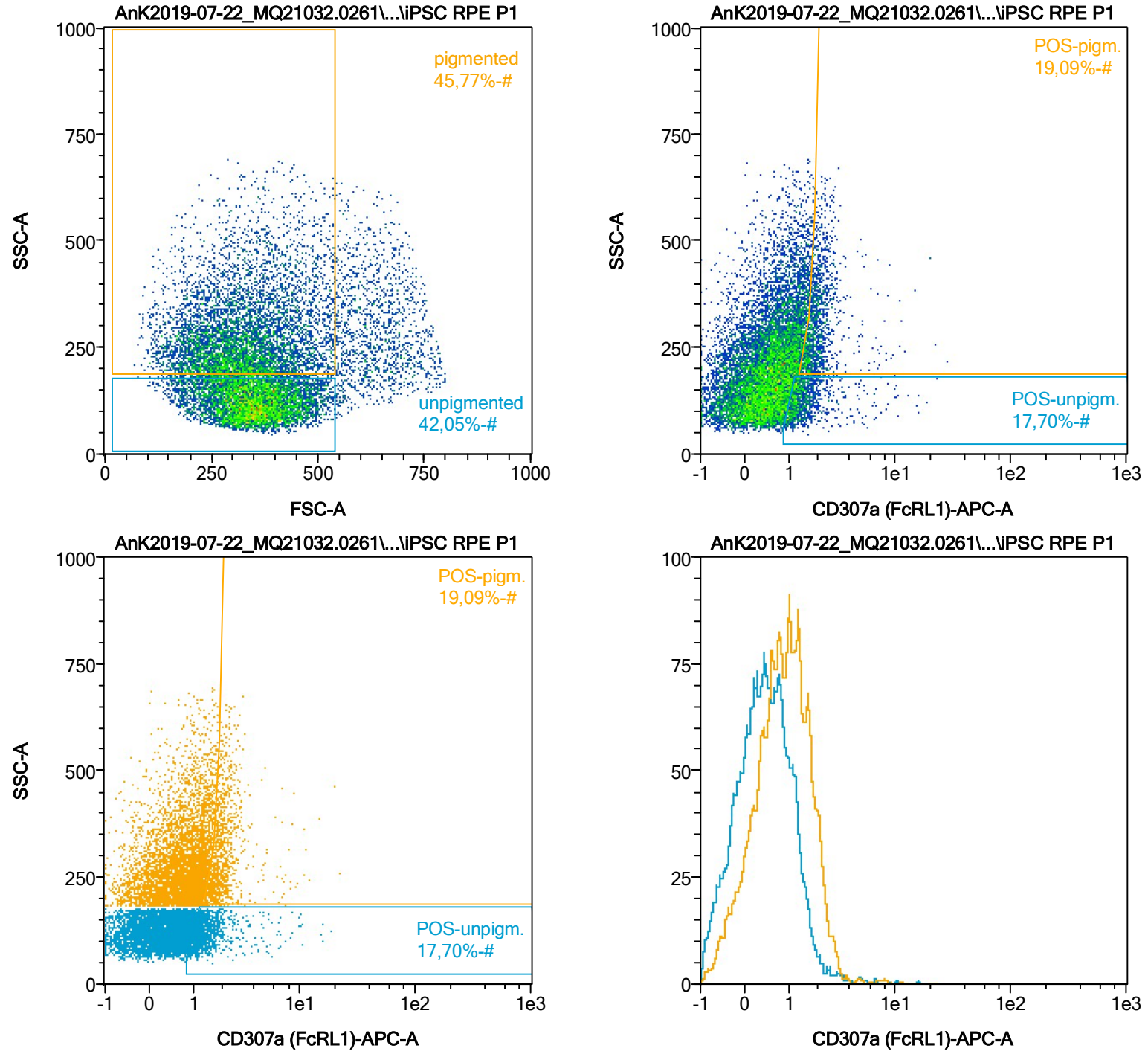

Name	%-#	CD307a (FcRL1)-APC-A Mean	CD307a (FcRL1)-APC-A Median
POS-unpigm.	17,70	1,67	1,31
POS-pigm.	19,09	2,20	1,93

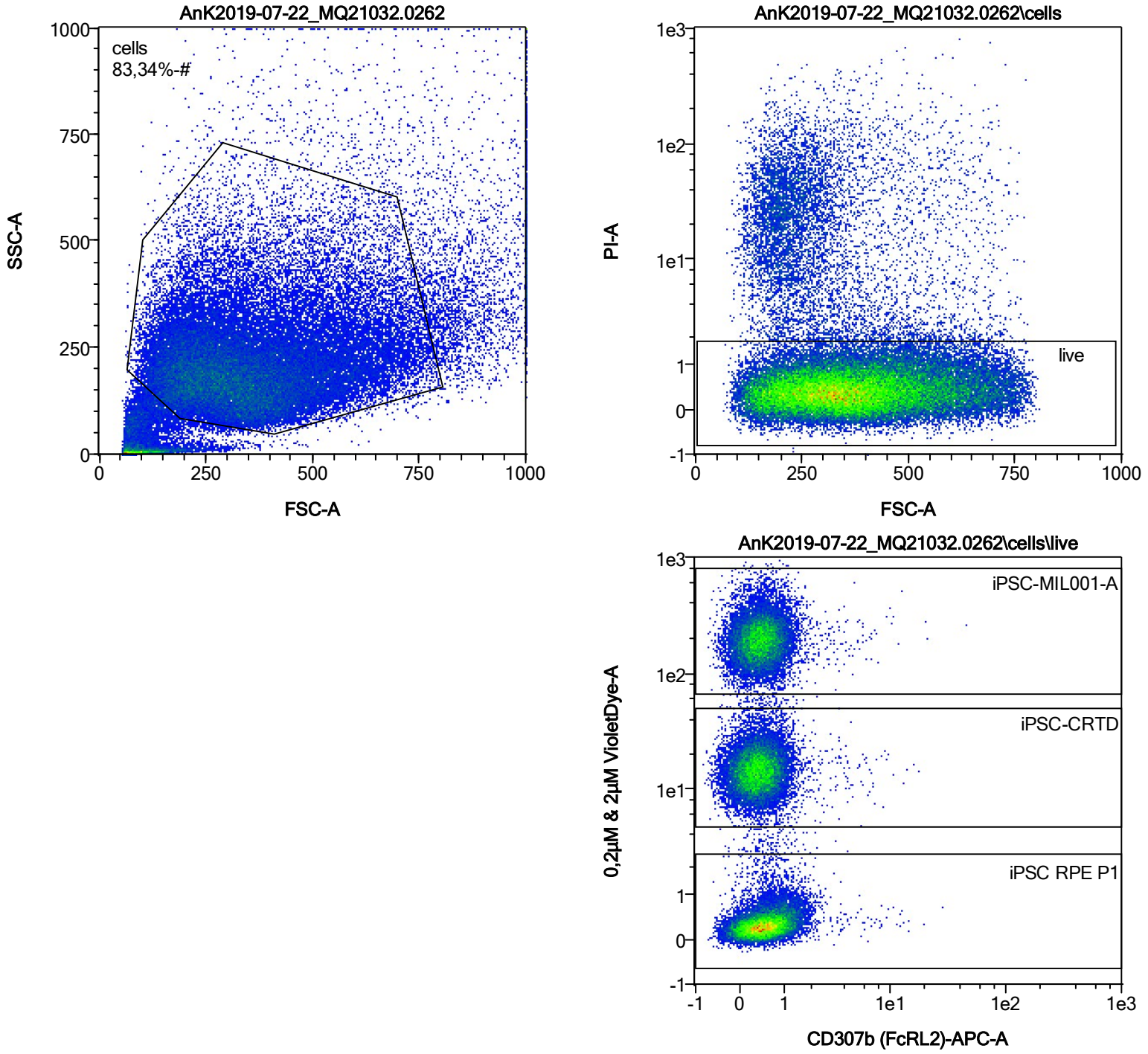

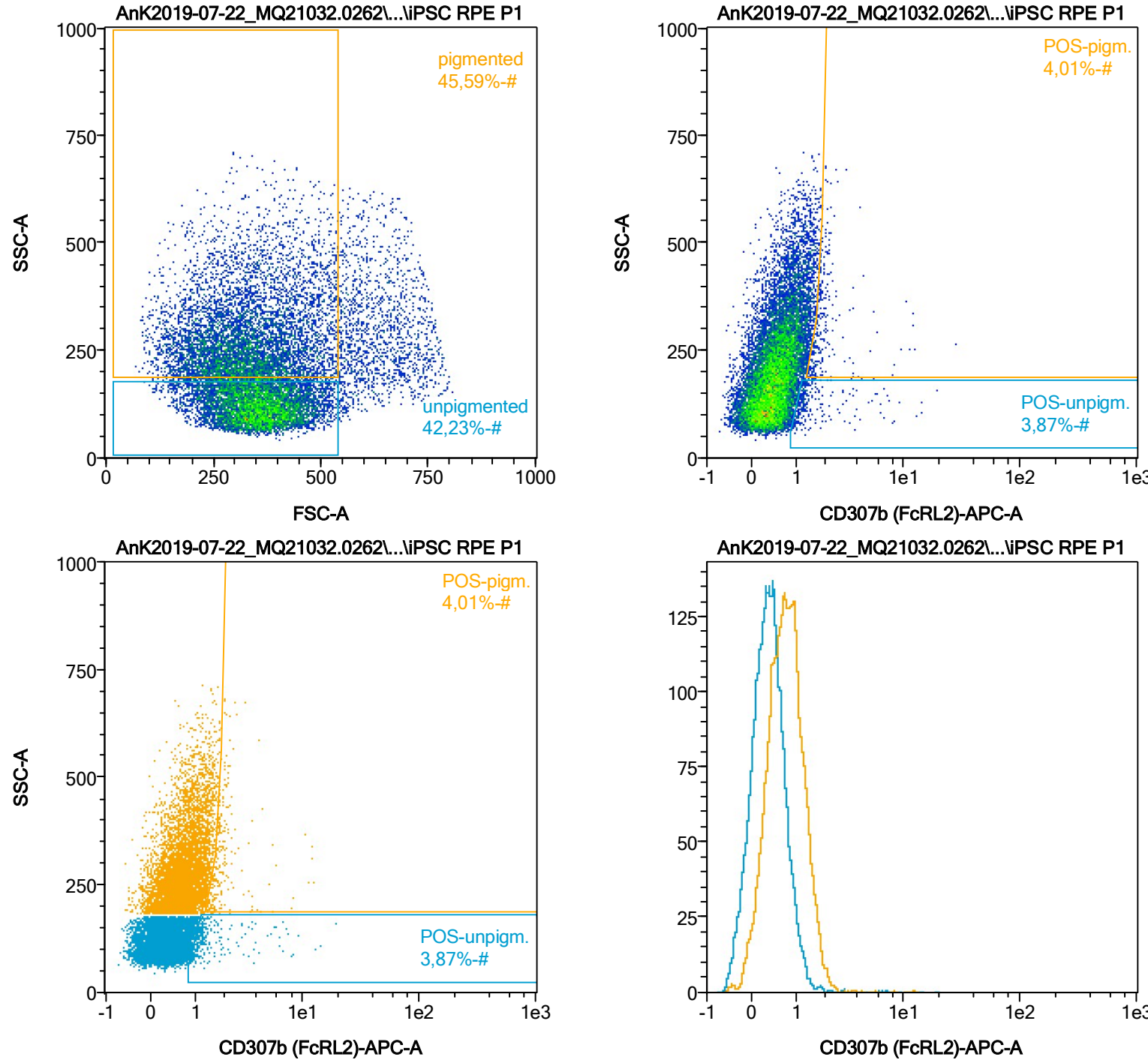

Name	%-#	CD307b (FcRL2)-APC-A Mean	CD307b (FcRL2)-APC-A Median
POS-unpigm.	3,87	1,88	1,23
POS-pigm.	4,01	2,22	1,81

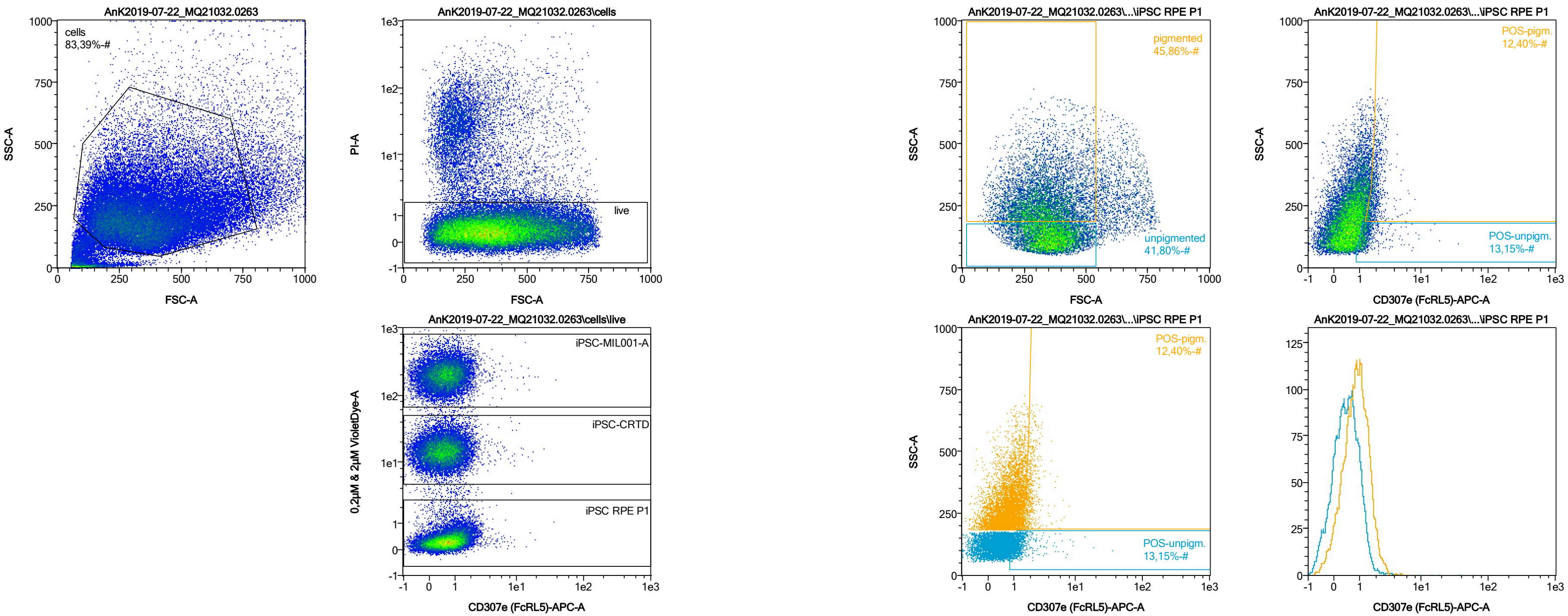

Name	%-#	CD307e (FcRL5)-APC-A Mean	CD307e (FcRL5)-APC-A Median
POS-unpigm.	13,15	1,50	1,24
POS-pigm.	12,40	1,97	1,83

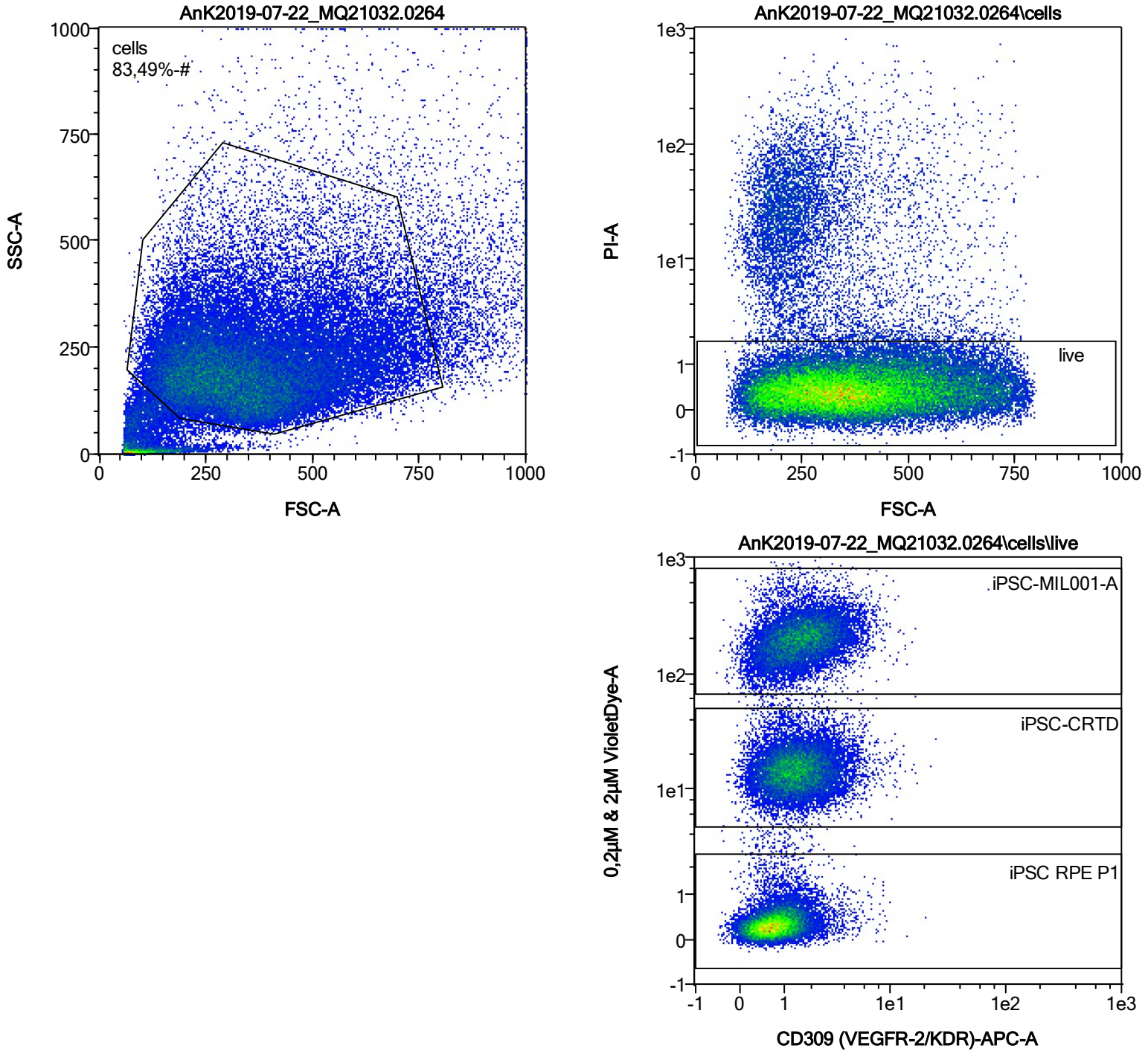

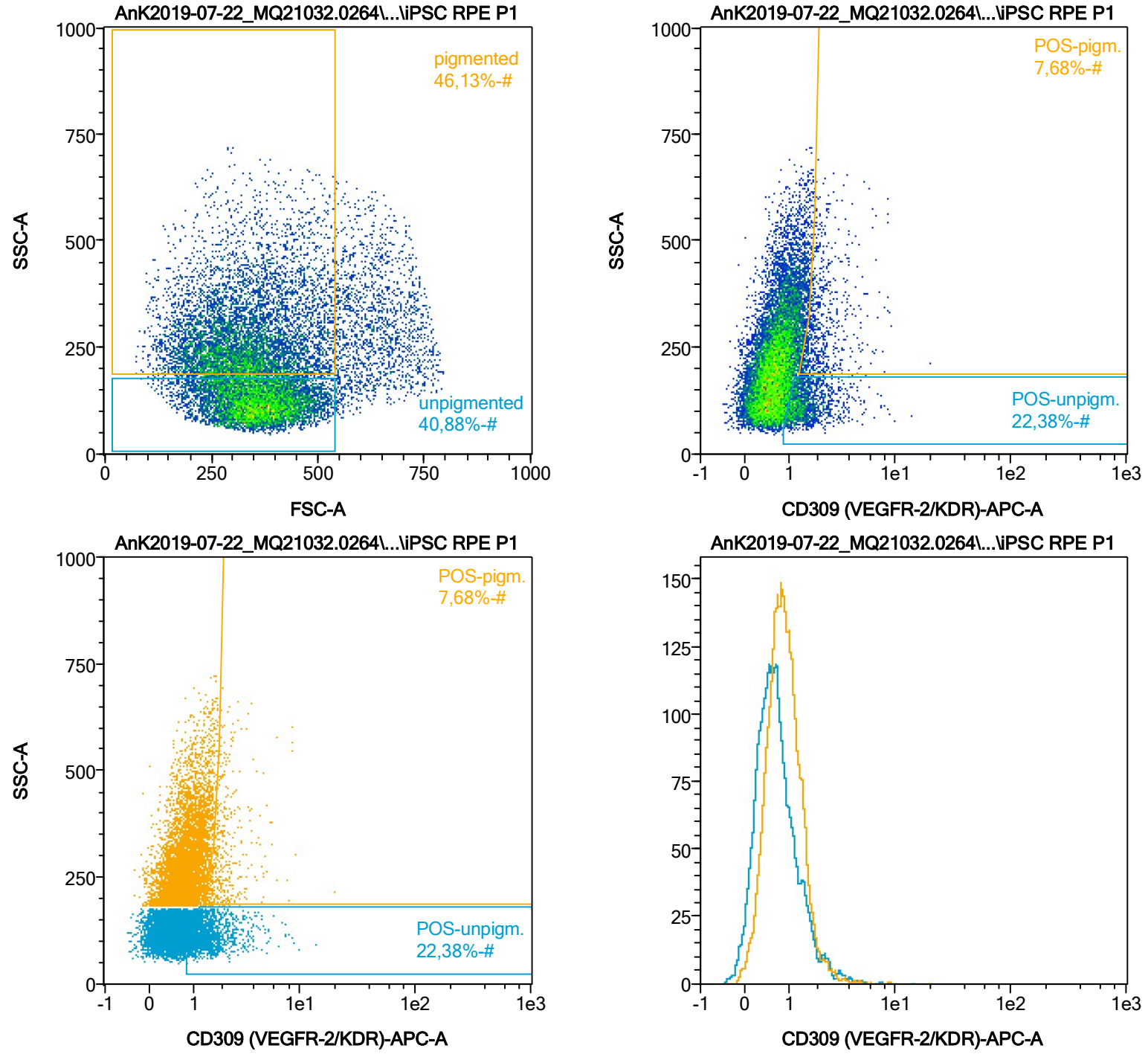

Name	%-#	CD309 (VEGFR-2/KDR)-APC-A Mean	CD309 (VEGFR-2/KDR)-APC-A Median
POS-unpigm.	22,38	1,65	1,39
POS-pigm.	7,68	2,24	1,90

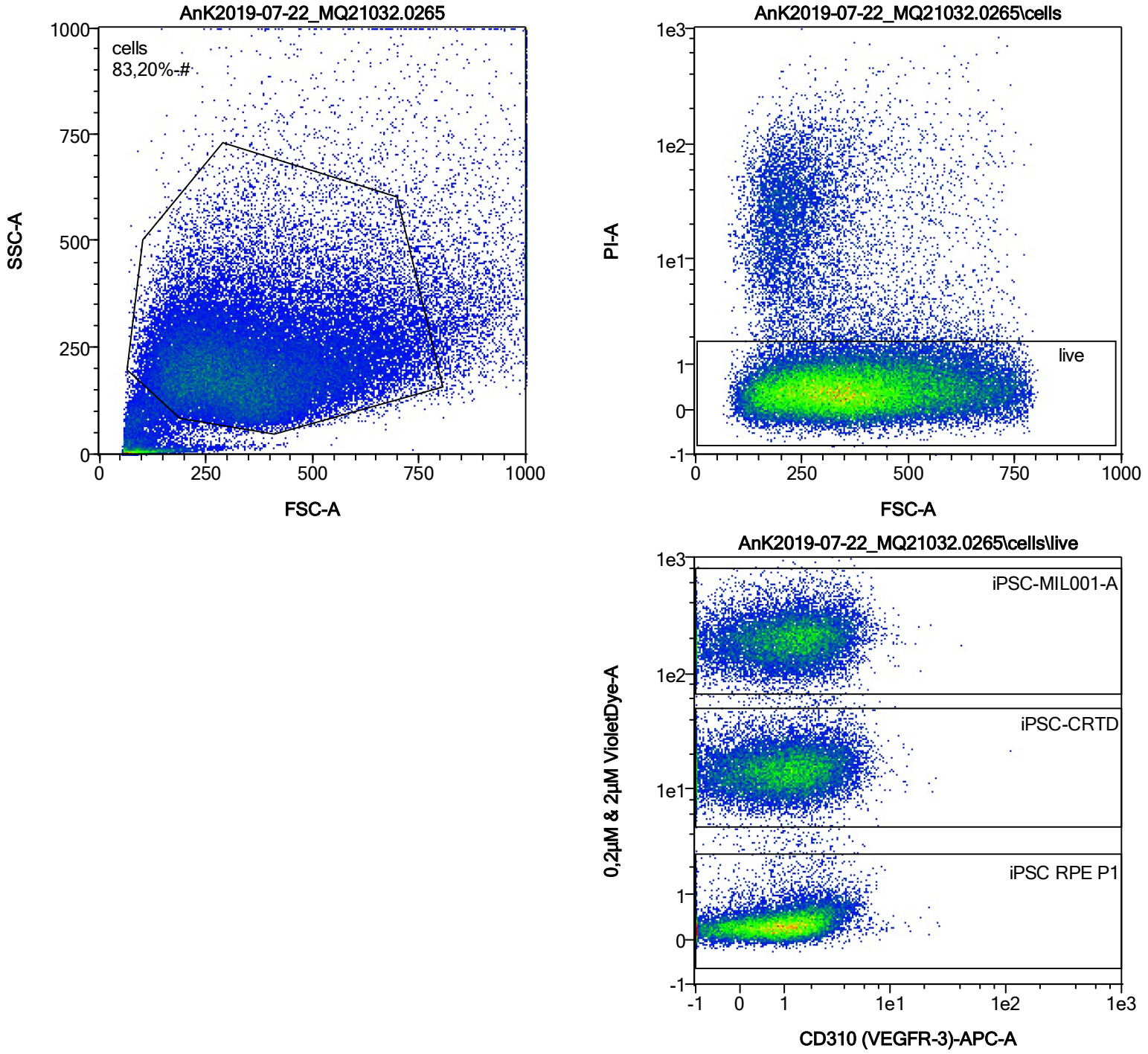

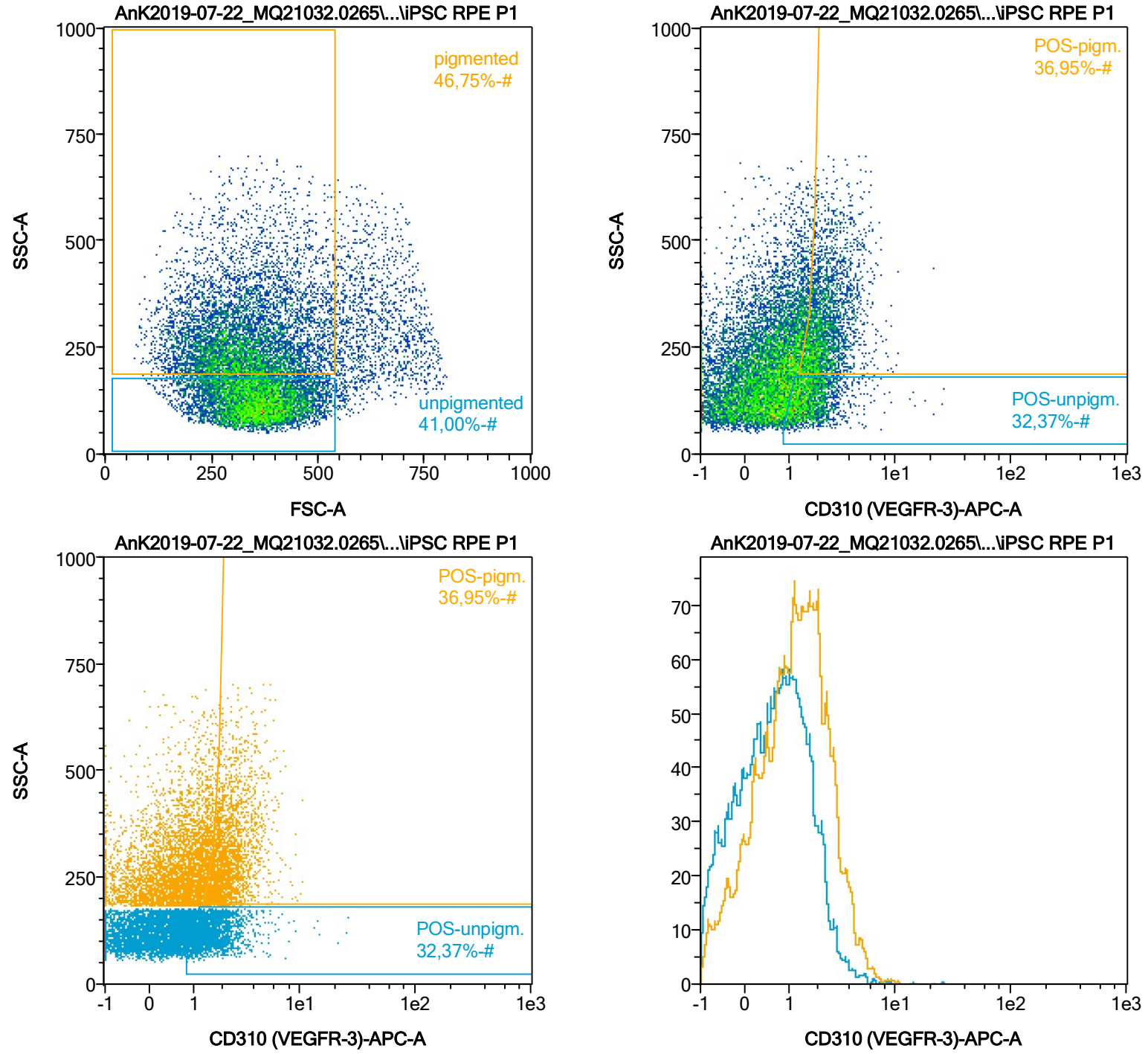

Name	%-#	CD310 (VEGFR-3)-APC-A Mean	CD310 (VEGFR-3)-APC-A Median
POS-unpigm.	32,37	1,81	1,55
POS-pigm.	36,95	2,51	2,21

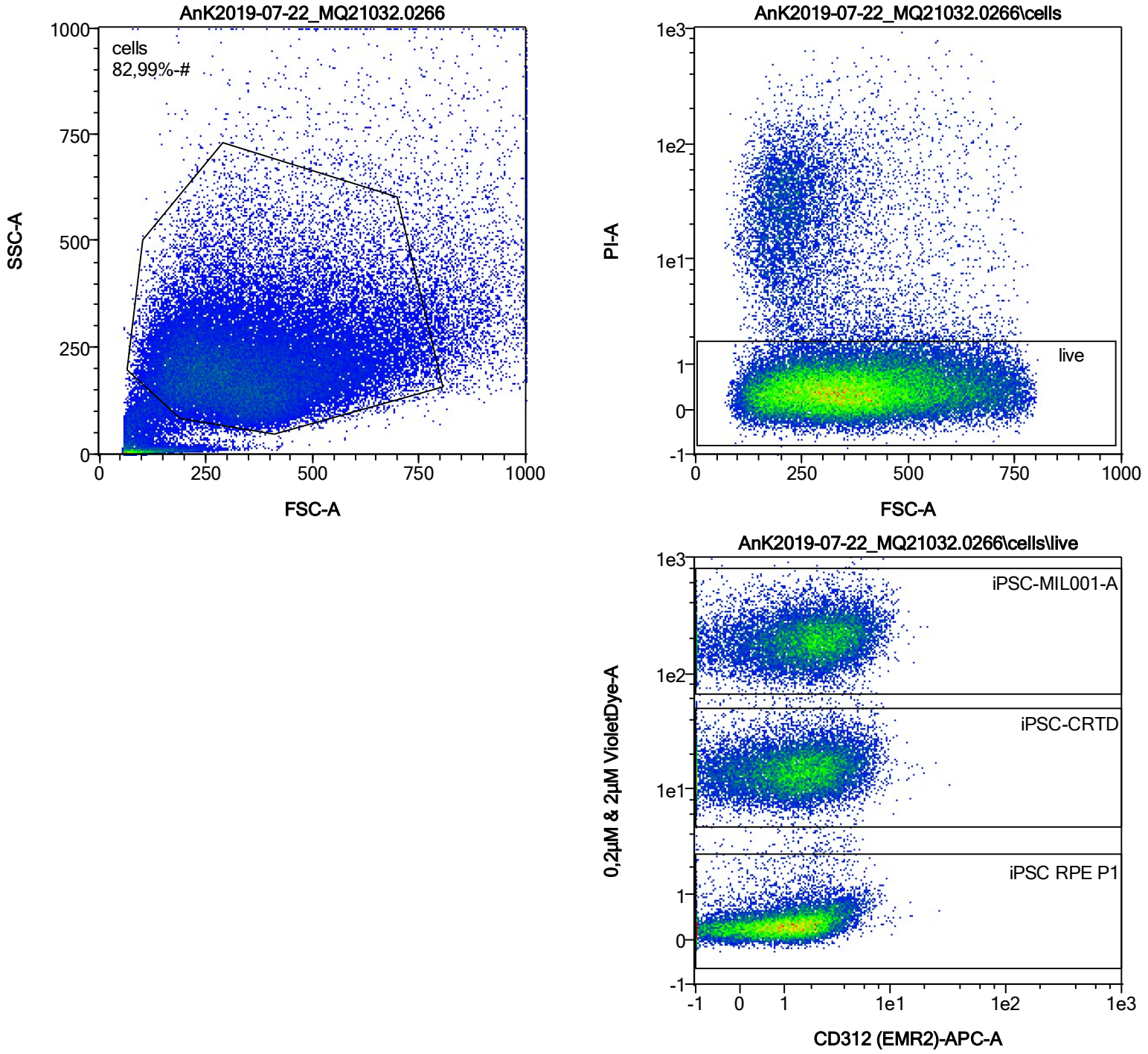

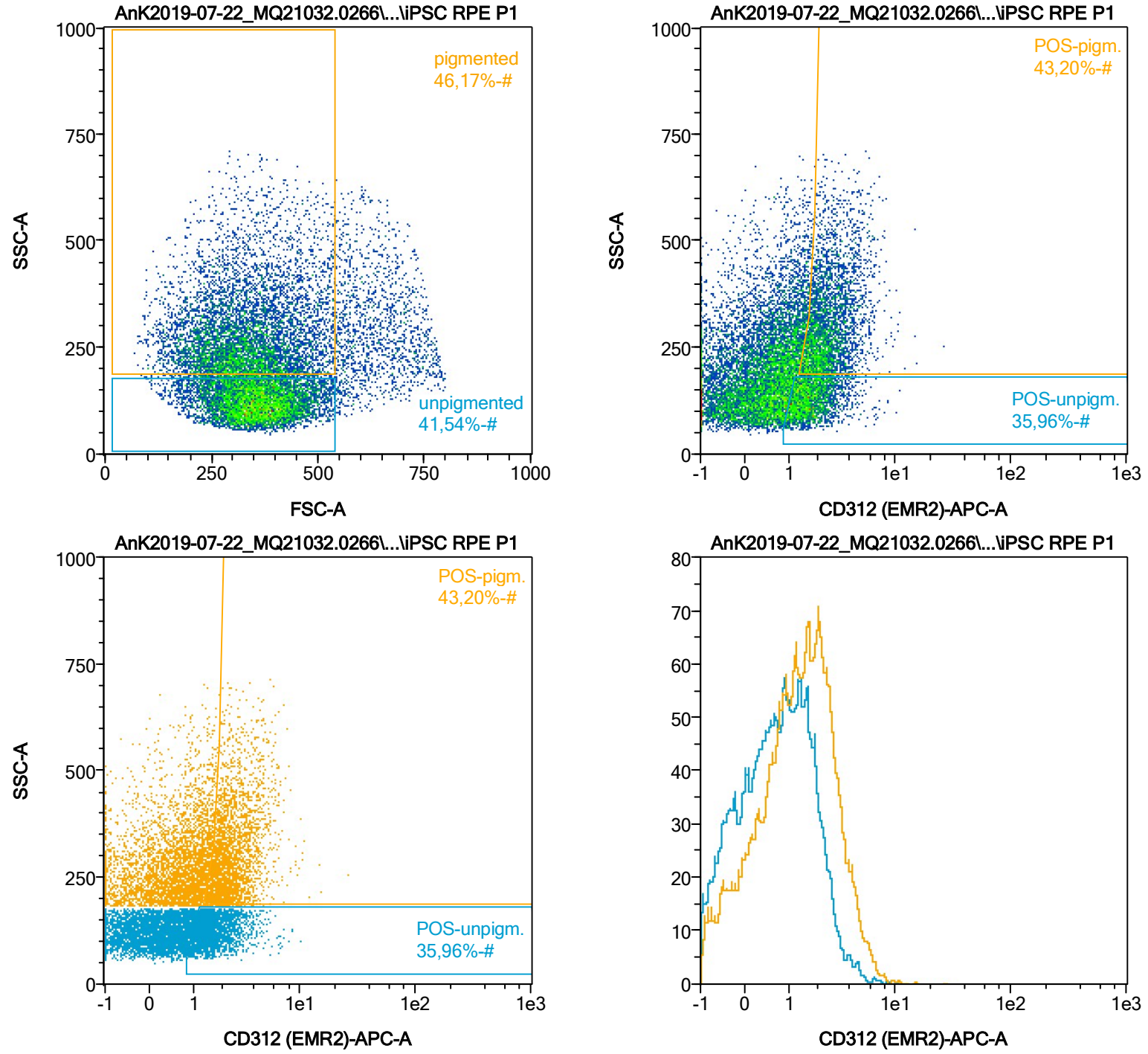

Name	%-#	CD312 (EMR2)-APC-A Mean	CD312 (EMR2)-APC-A Median
POS-unpigm.	35,96	1,85	1,62
POS-pigm.	43,20	2,68	2,35

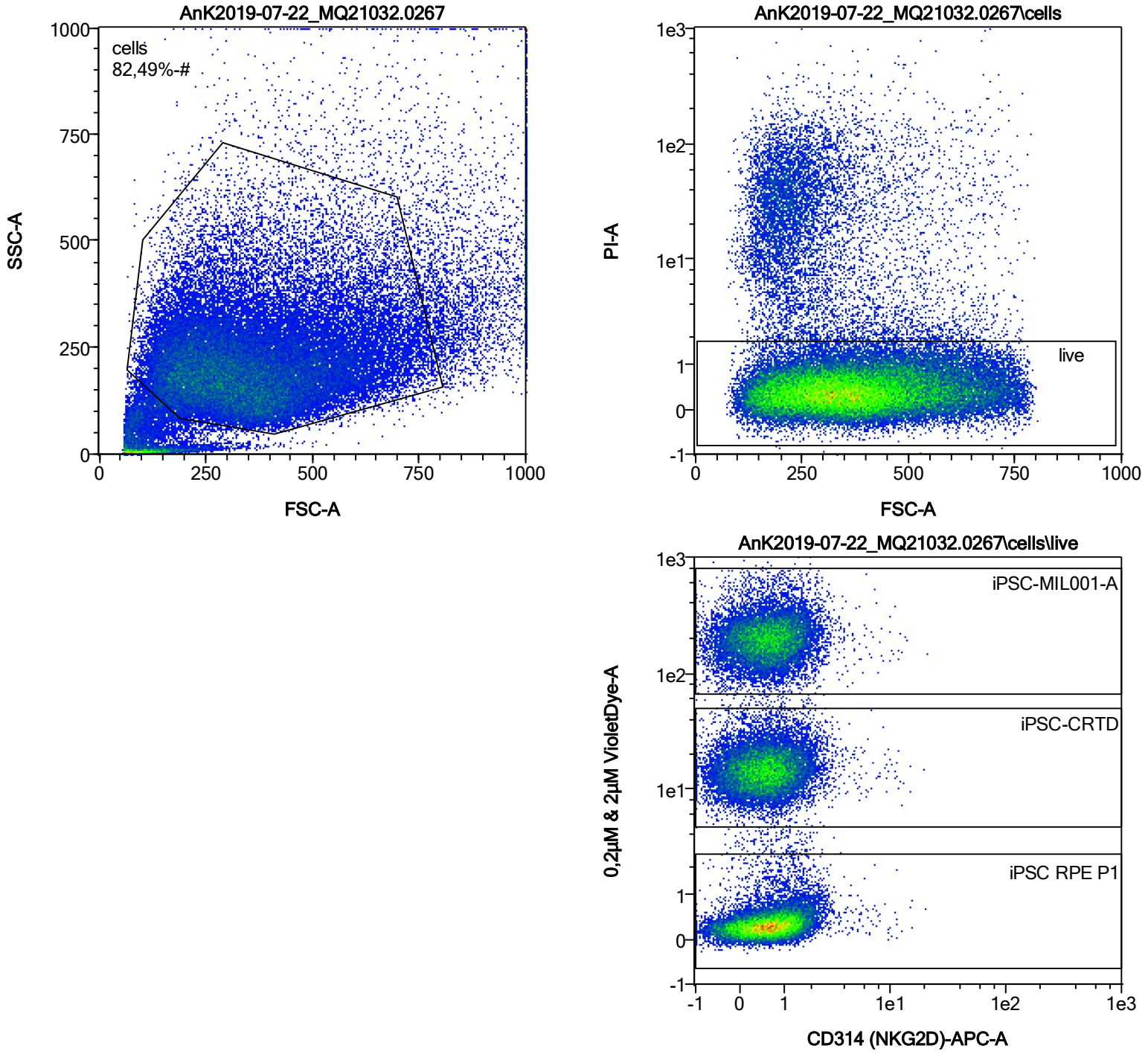

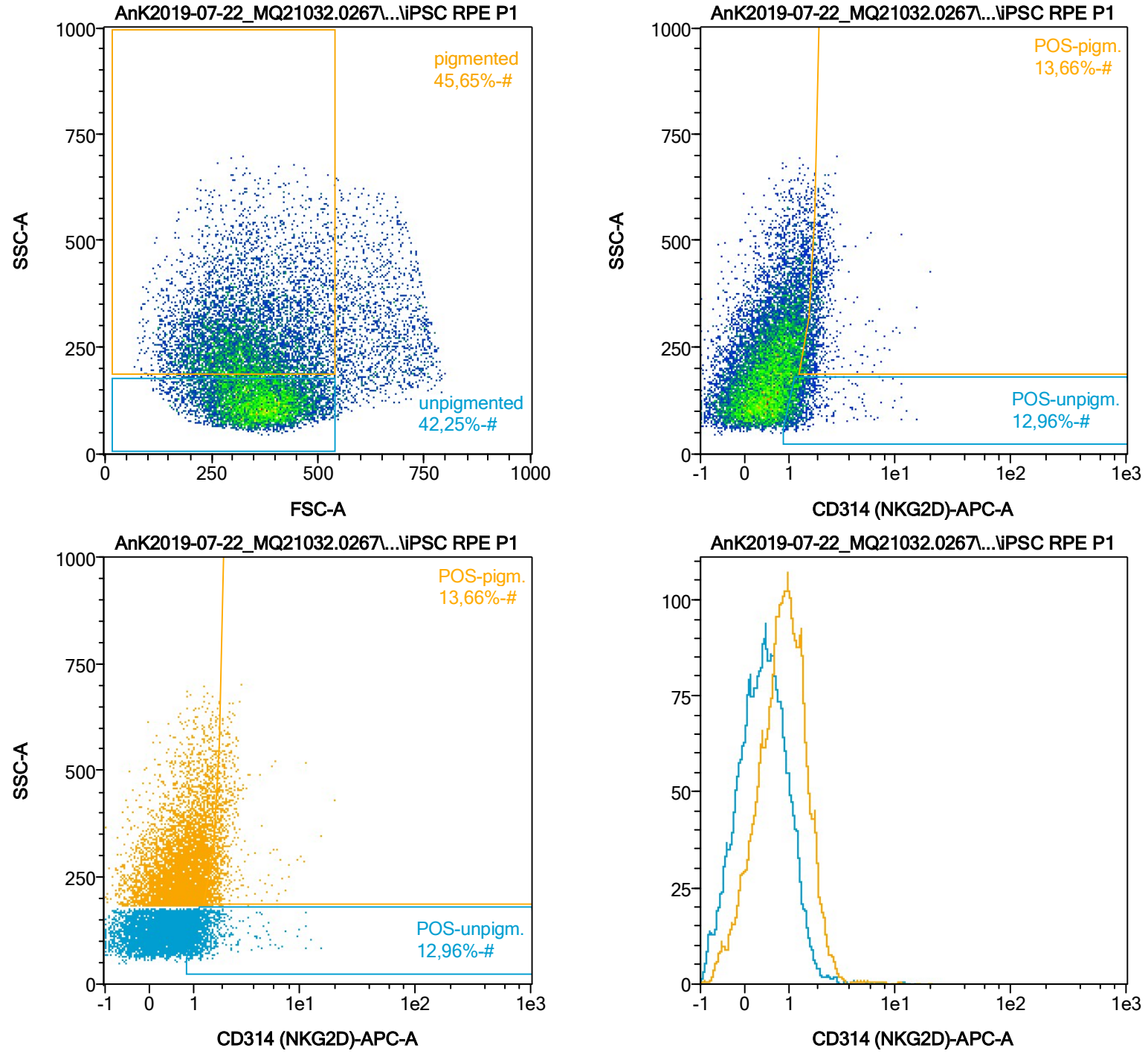

Name	%-#	CD314 (NKG2D)-APC-A Mean	CD314 (NKG2D)-APC-A Median
POS-unpigm.	12,96	1,54	1,27
POS-pigm.	13,66	2,10	1,87

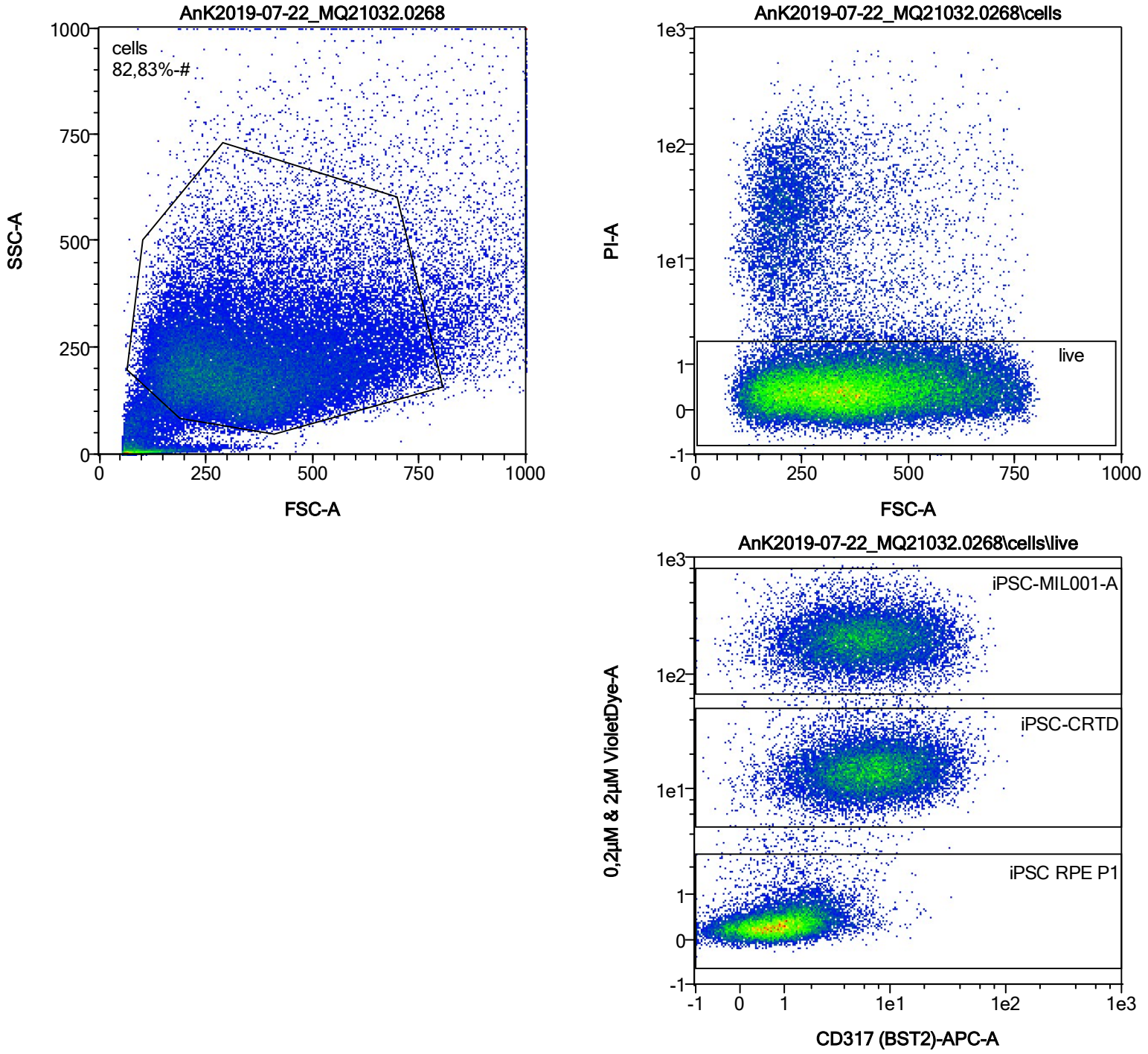

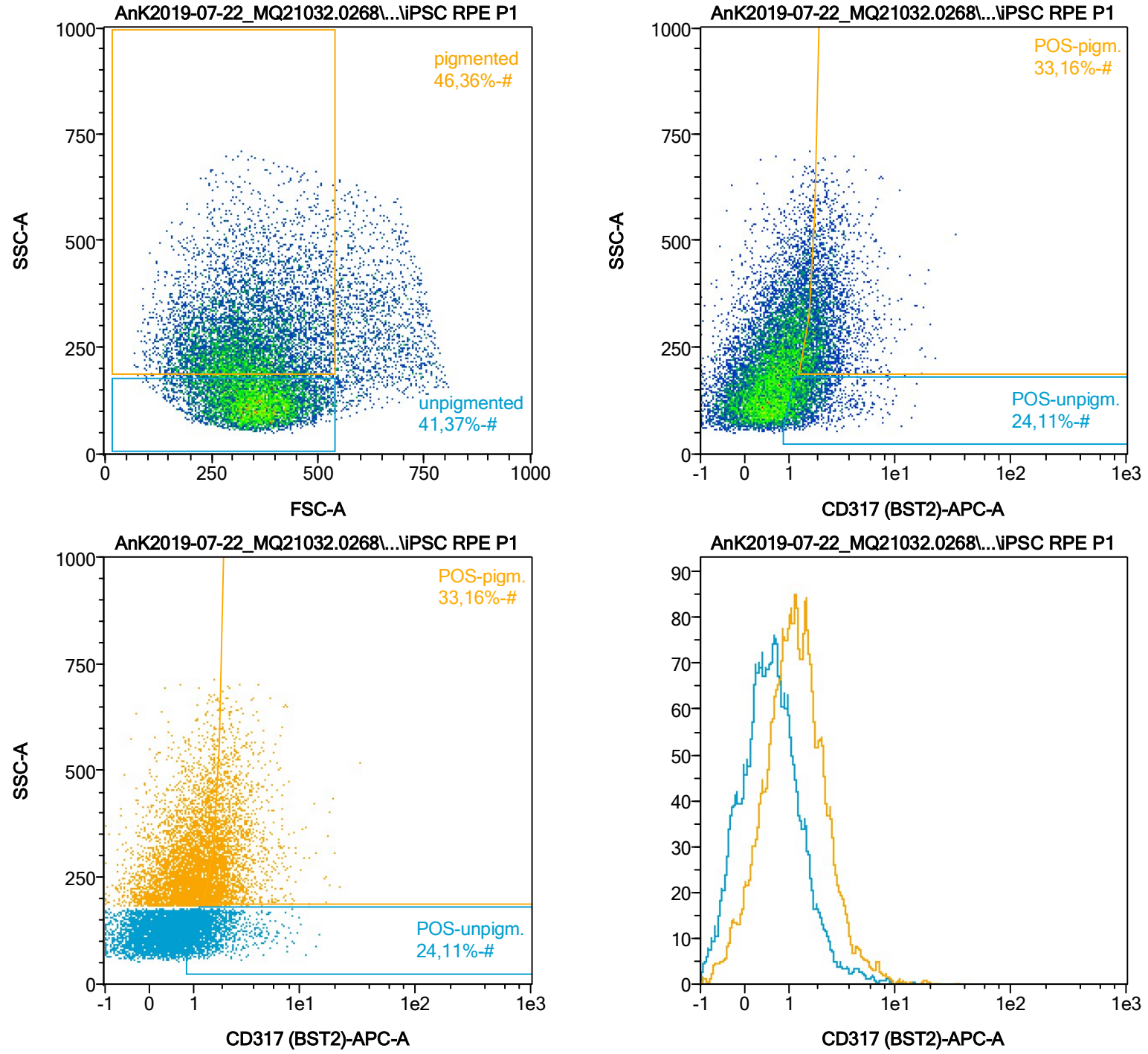

Name	%-#	CD317 (BST2)-APC-A Mean	CD317 (BST2)-APC-A Median
POS-unpigm.	24,11	1,84	1,47
POS-pigm.	33,16	2,67	2,19

Date:2024-06-14

Operator: Sebastian Knoebel

Version:2.13.0+54.g95abf0f4

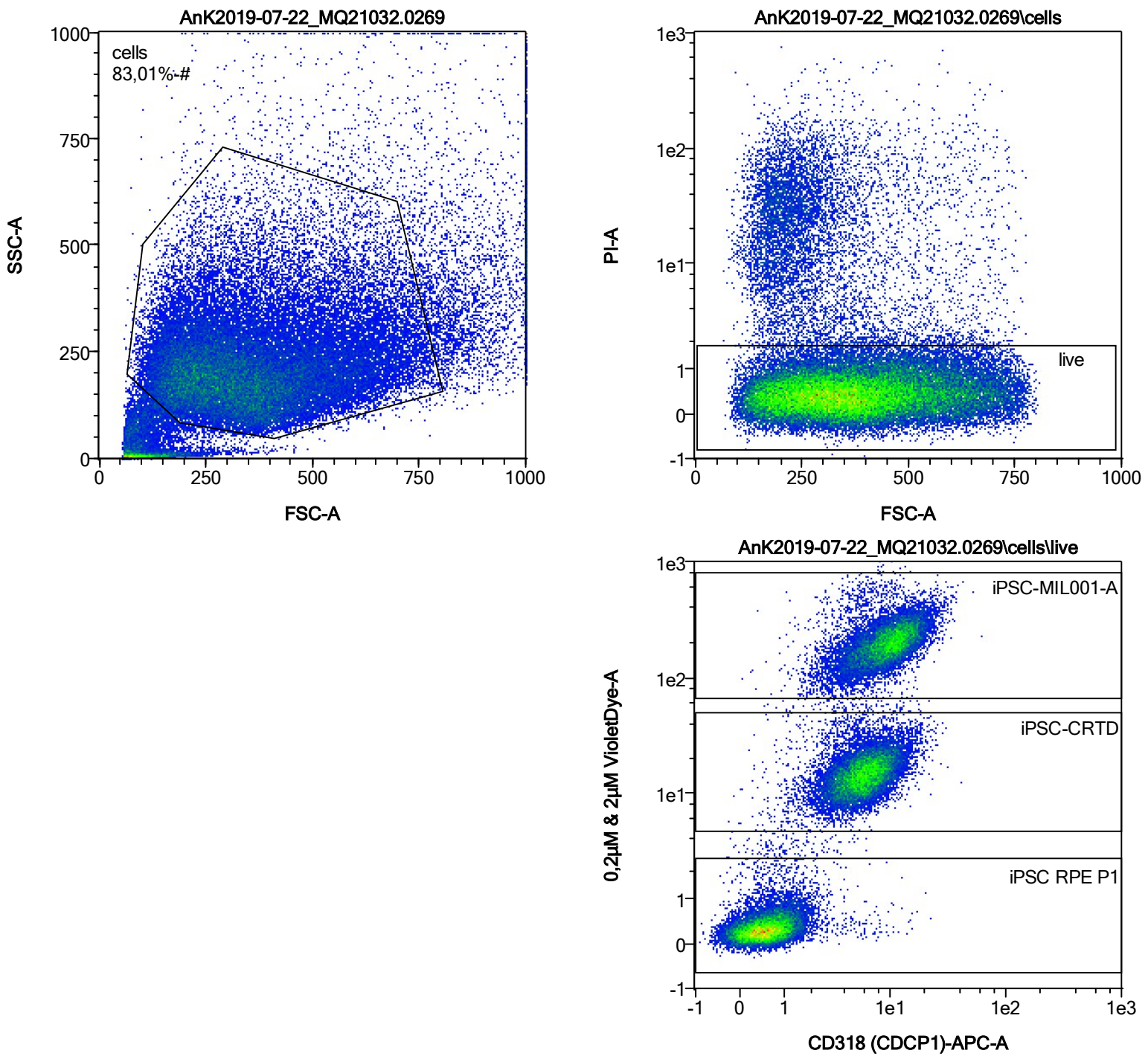

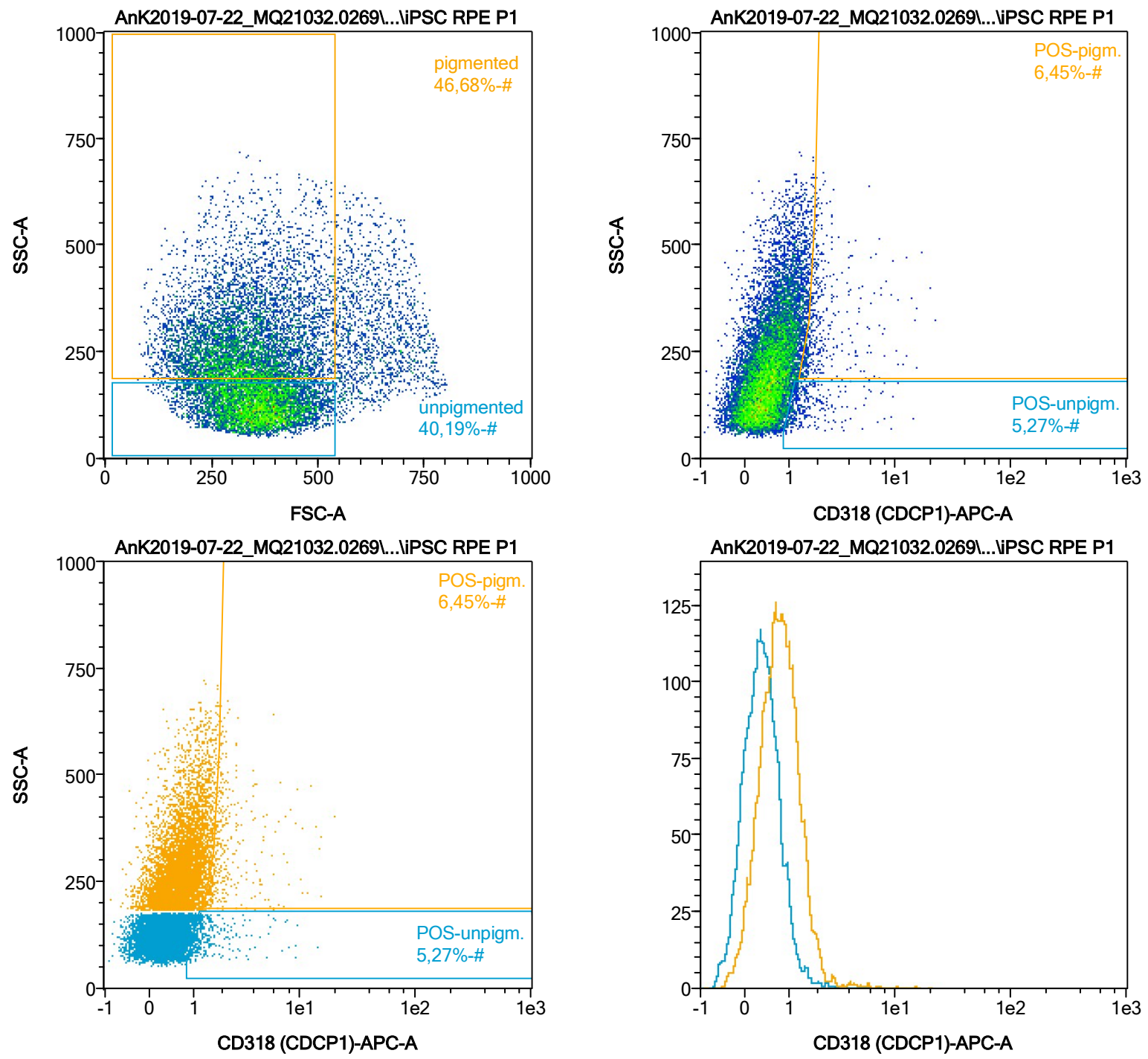

Name	%-#	CD318 (CDCP1)-APC-A Mean	CD318 (CDCP1)-APC-A Median
POS-unpigm.	5,27	1,74	1,26
POS-pigm.	6,45	2,55	1,93

Date:2024-06-14
Operator: Sebastian Knoebel
Version:2.13.0+54.g95abf0f4

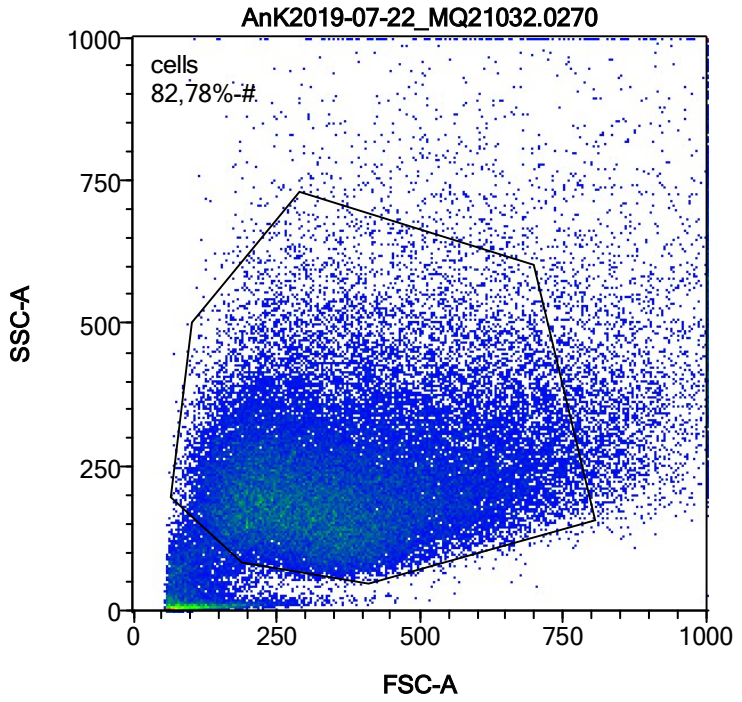

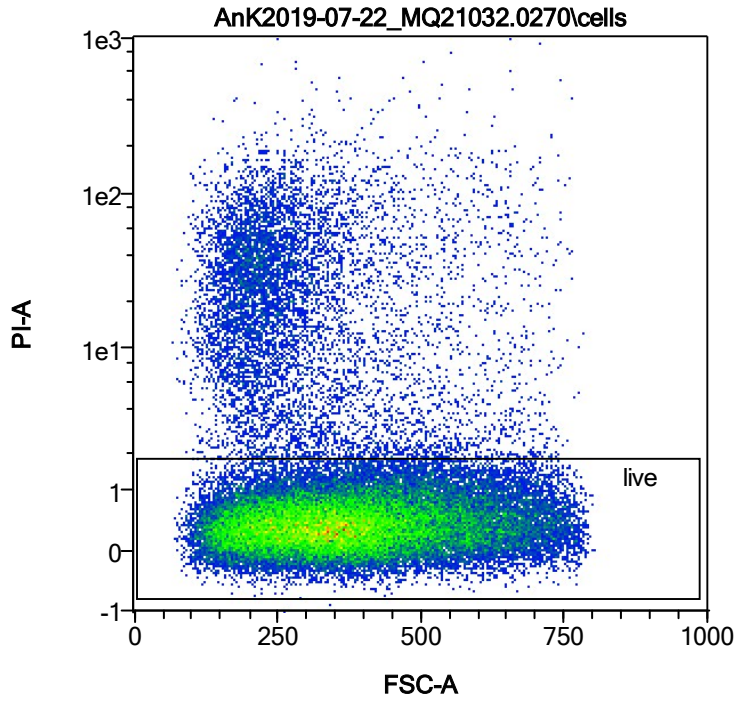

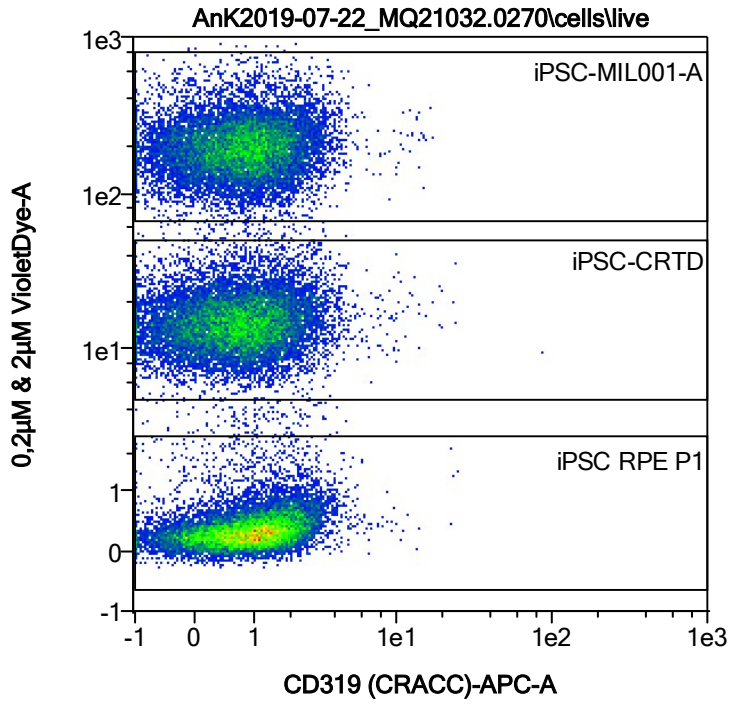

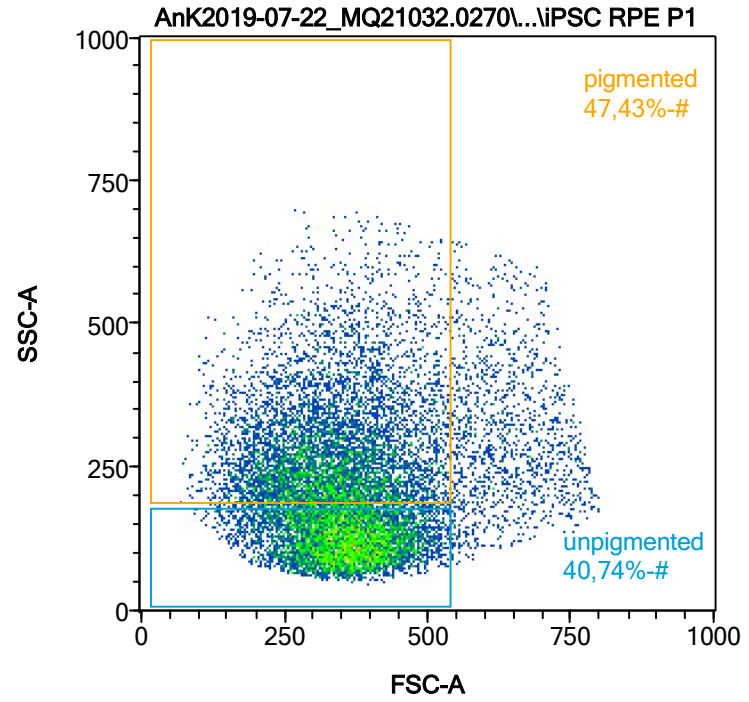

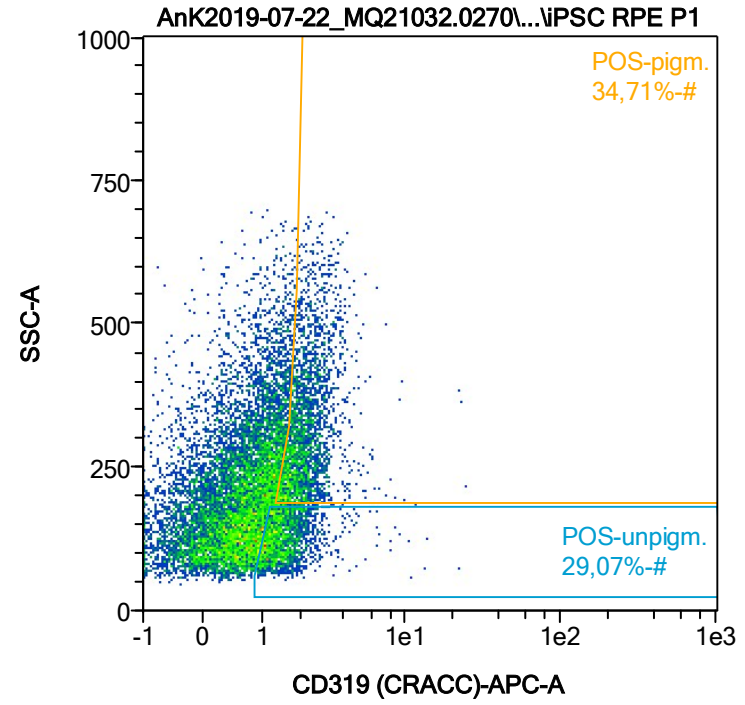

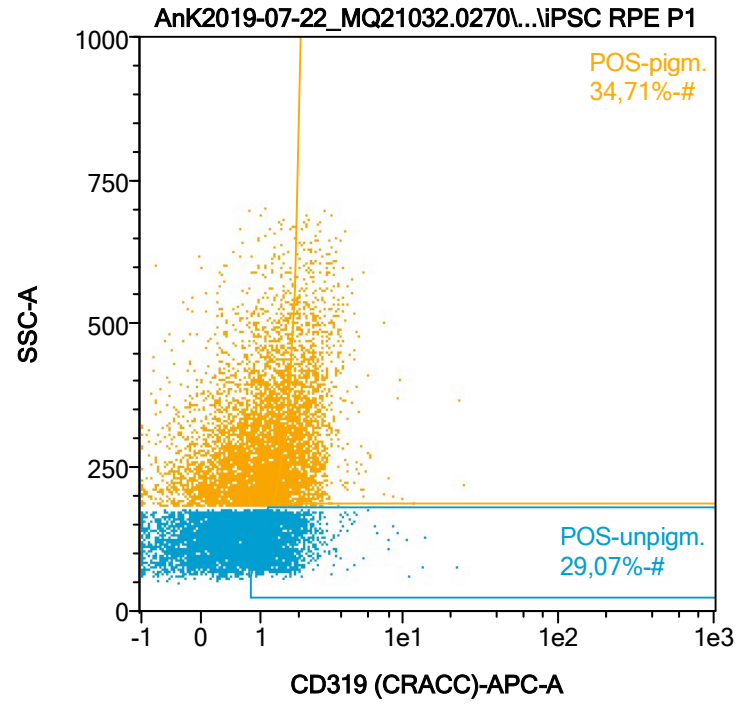

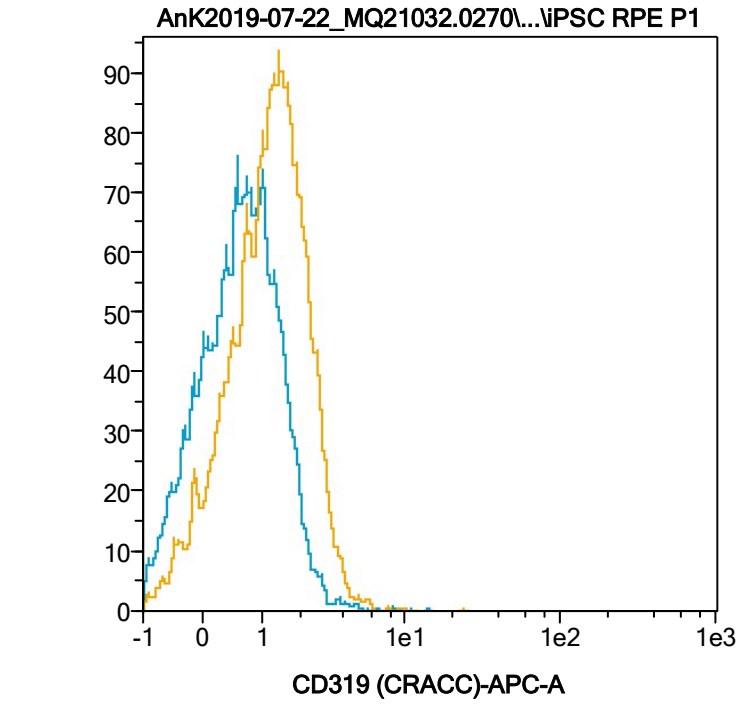

Name	%-#	CD319 (CRACC)-APC-A Mean	CD319 (CRACC)-APC-A Median
POS-unpigm.	29,07	1,60	1,41
POS-pigm.	34,71	2,25	2,06

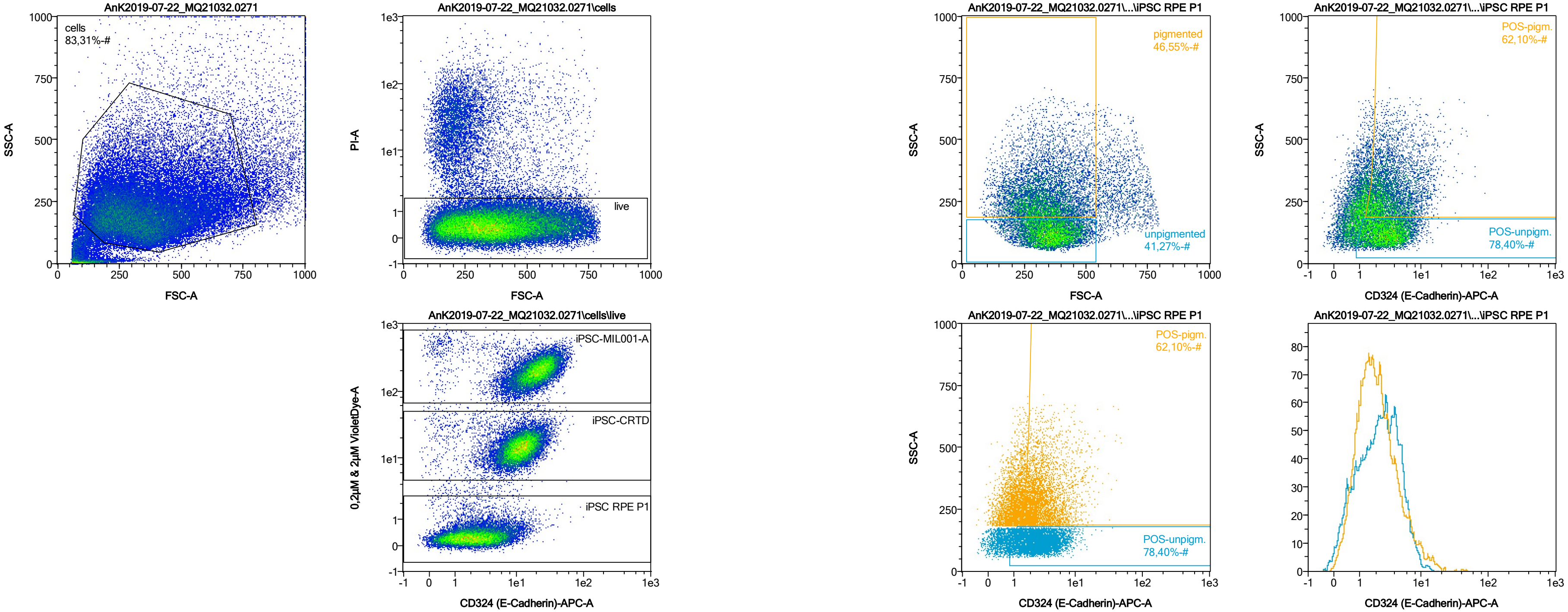

Name	%-#	CD324 (E-Cadherin)-APC-A Mean	CD324 (E-Cadherin)-APC-A Median
POS-unpigm.	78,40	3,27	2,83
POS-pigm.	62,10	3,76	2,74

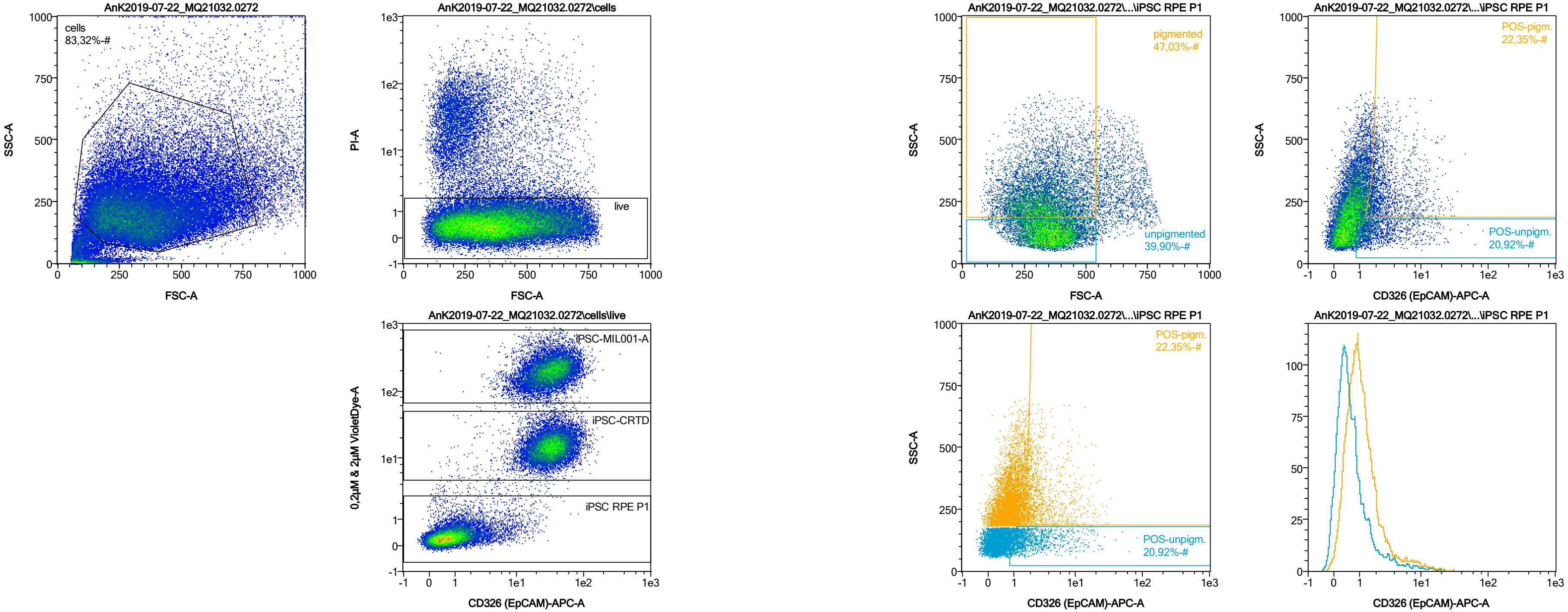

Name	%-#	CD326 (EpCAM)-APC-A Mean	CD326 (EpCAM)-APC-A Median
POS-unpigm.	20,92	3,10	1,66
POS-pigm.	22,35	3,63	2,28

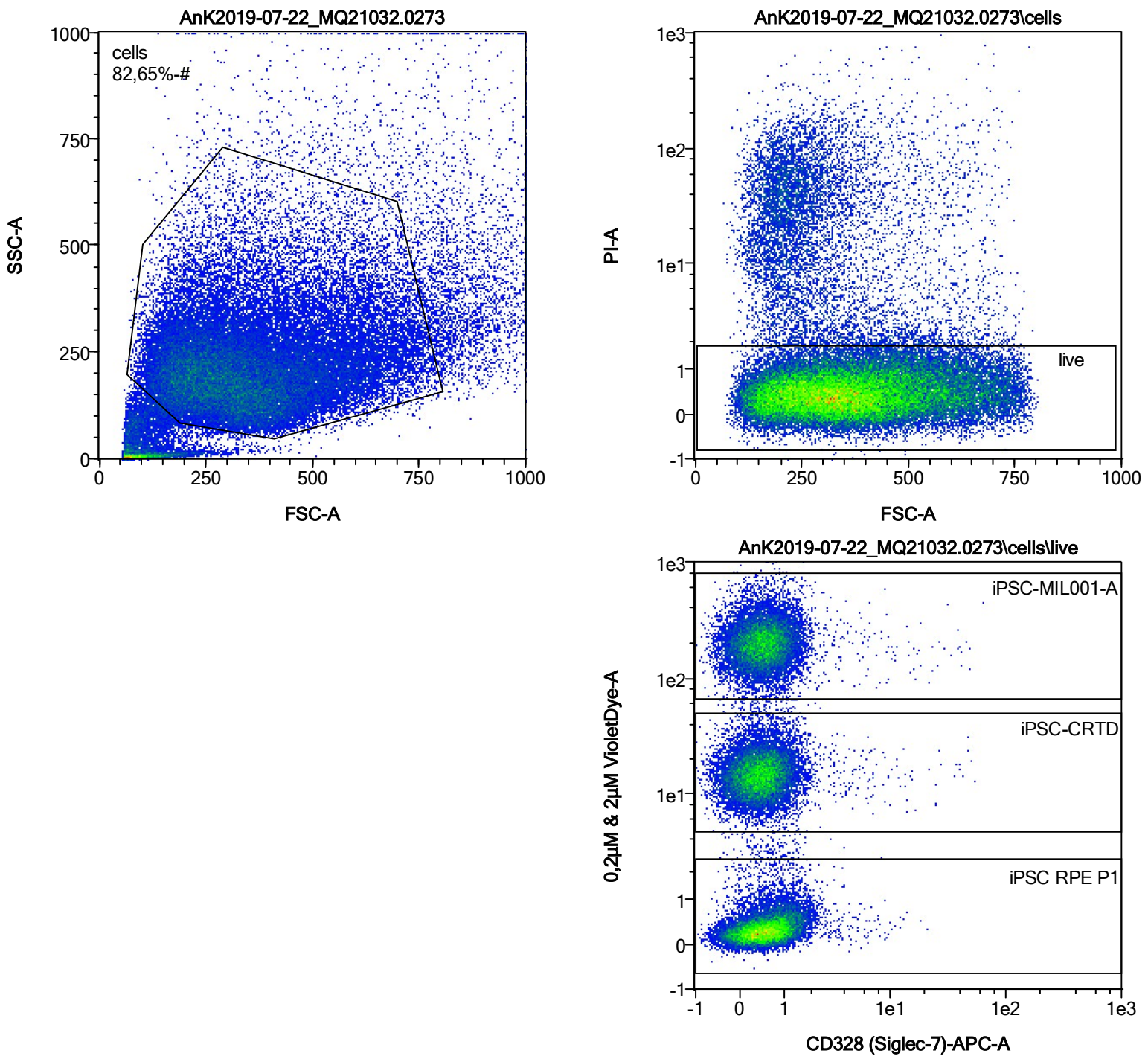

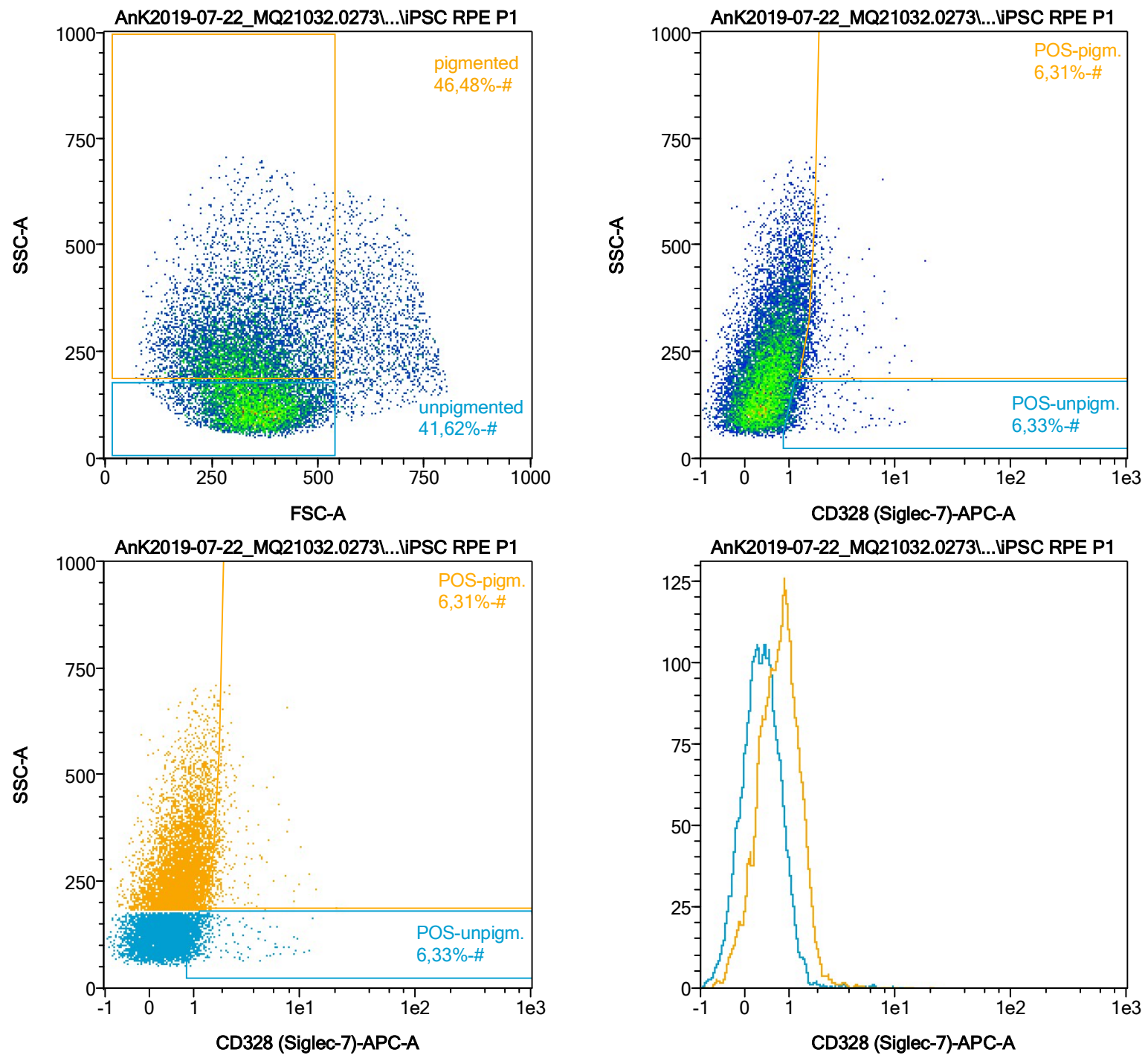

Name	%-#	CD328 (Siglec-7)-APC-A Mean	CD328 (Siglec-7)-APC-A Median
POS-unpigm.	6,33	1,72	1,21
POS-pigm.	6,31	2,24	1,84

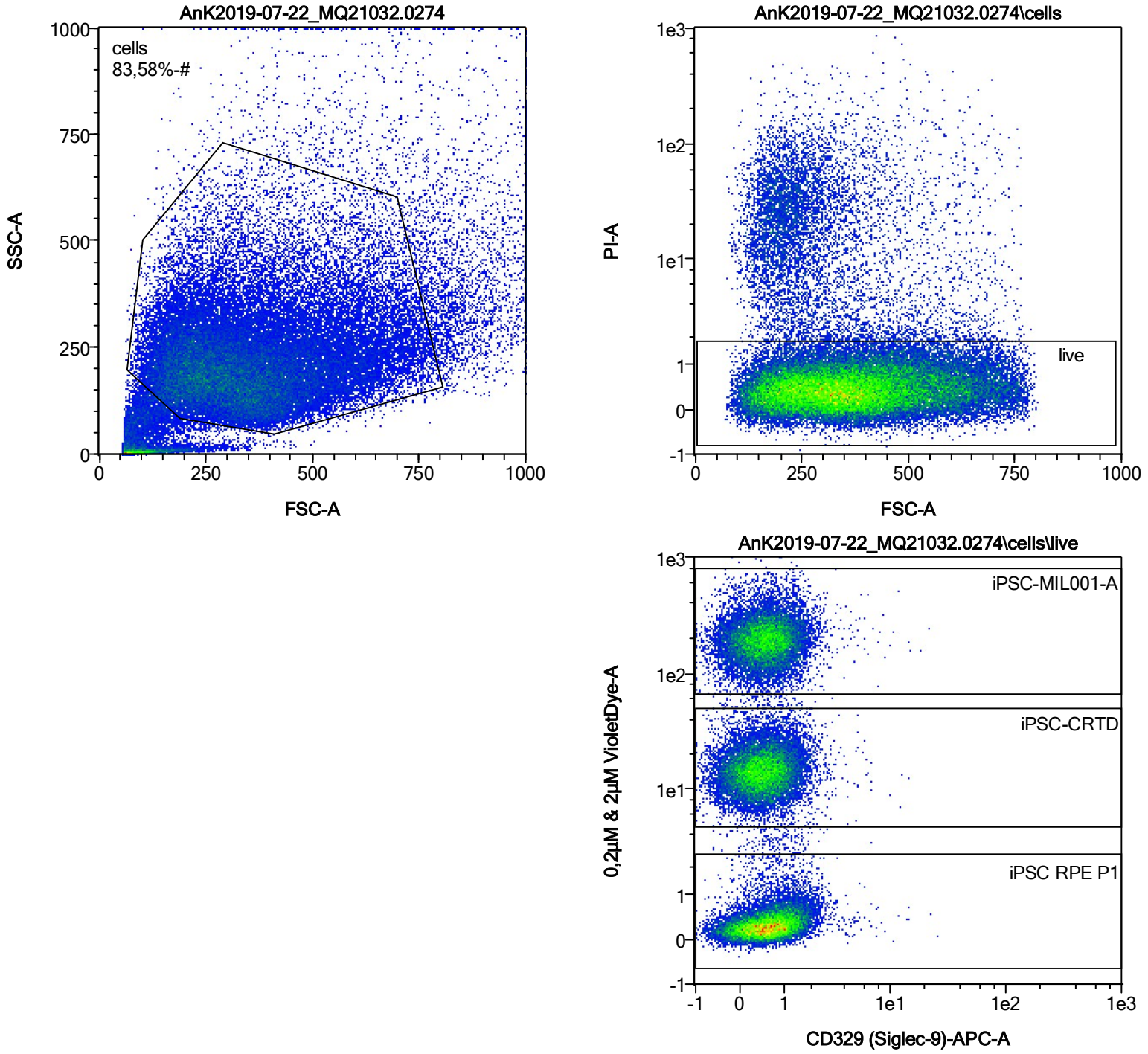

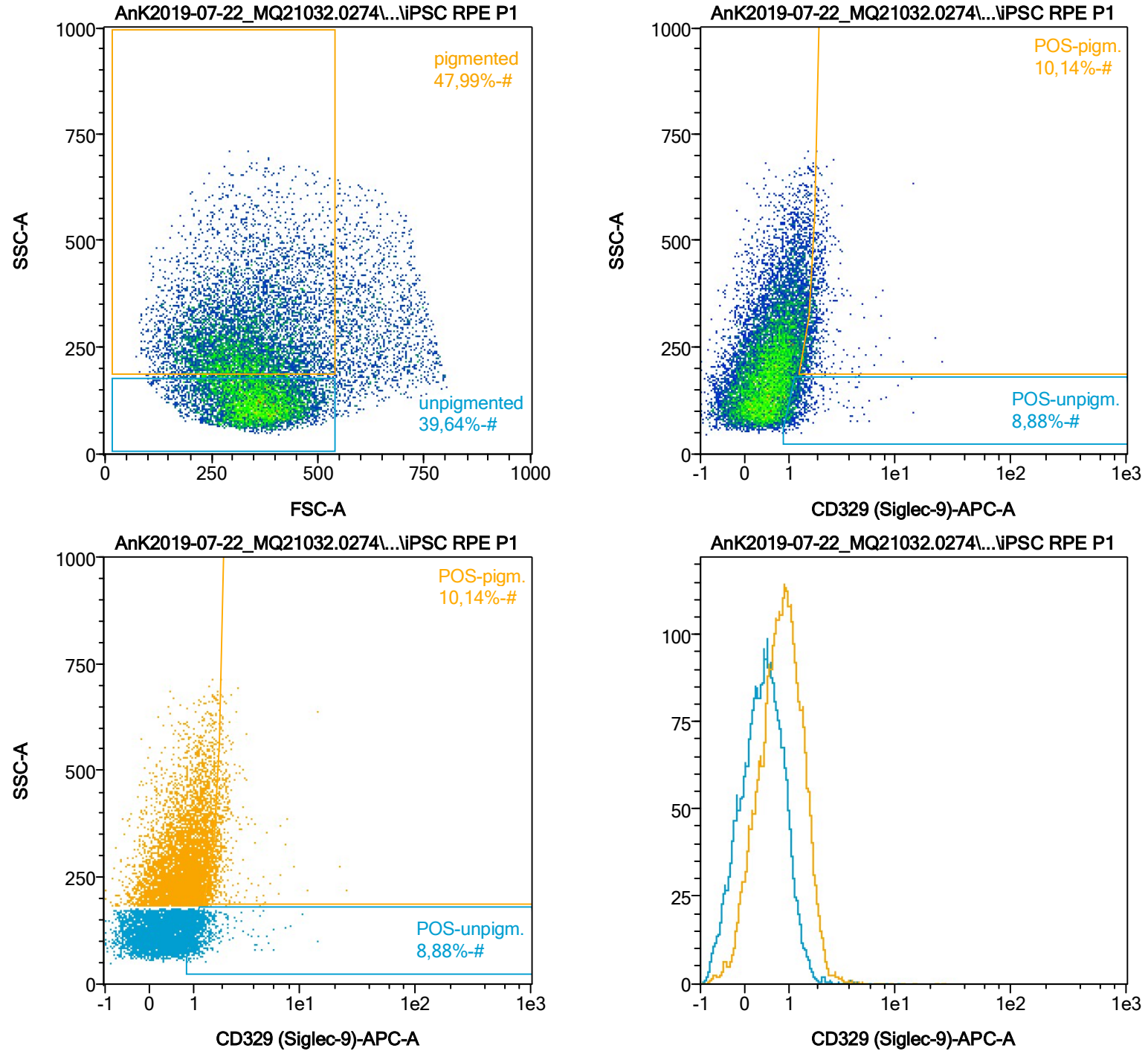

Name	%-#	CD329 (Siglec-9)-APC-A Mean	CD329 (Siglec-9)-APC-A Median
POS-unpigm.	8,88	1,46	1,20
POS-pigm.	10,14	2,06	1,82

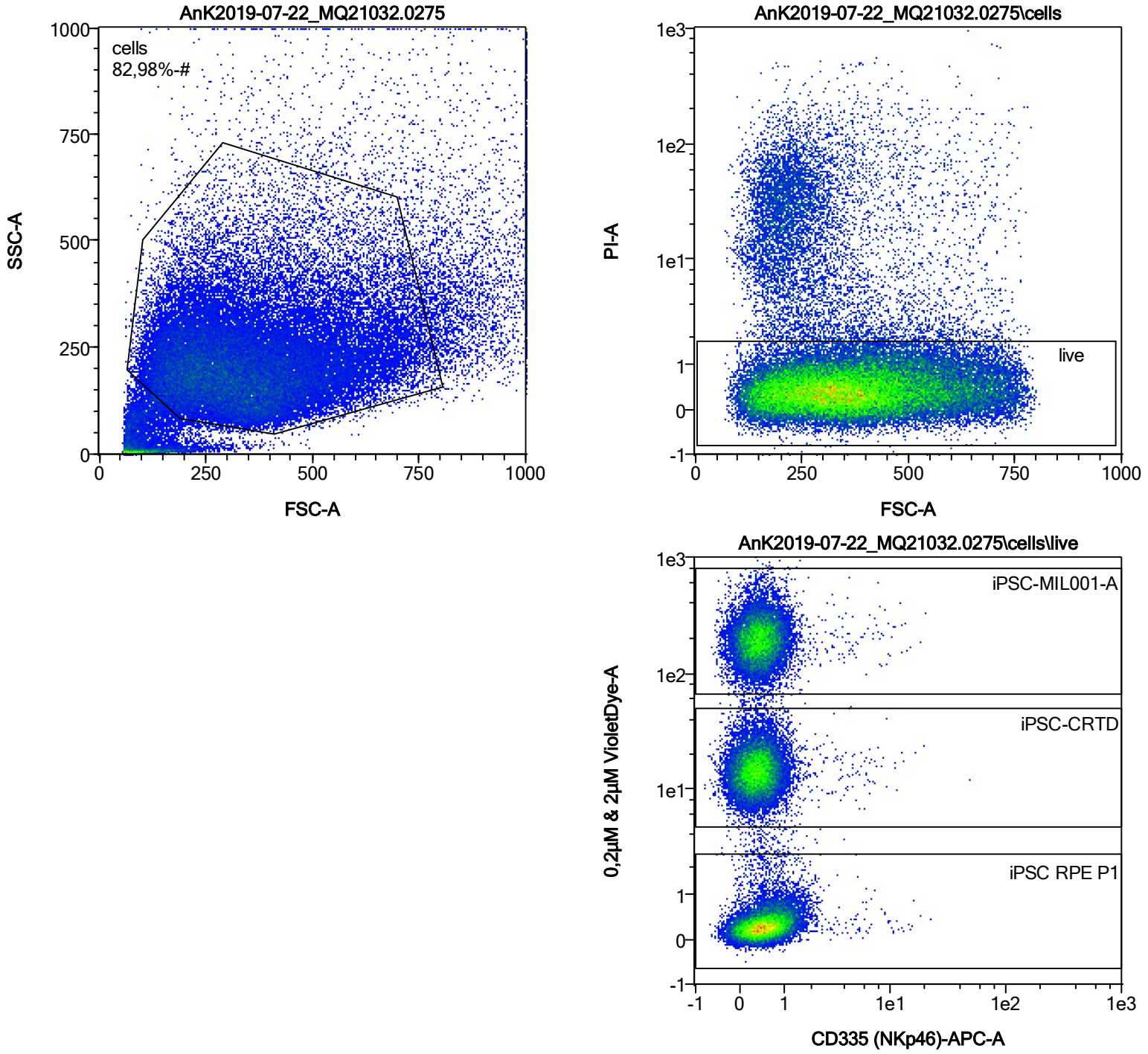

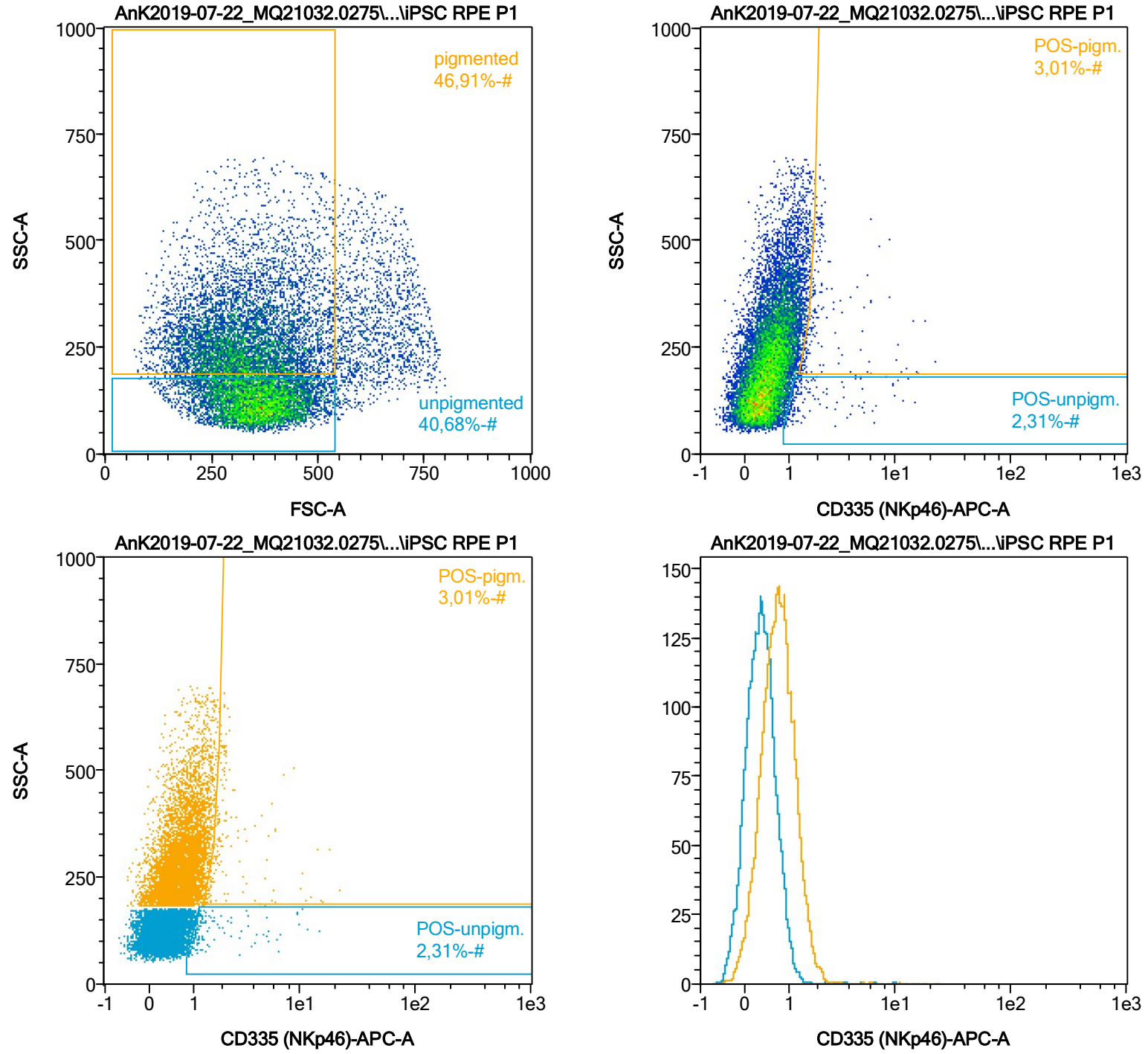

Name	%-#	CD335 (NKp46)-APC-A Mean	CD335 (NKp46)-APC-A Median
POS-unpigm.	2,31	2,15	1,22
POS-pigm.	3,01	2,75	1,87

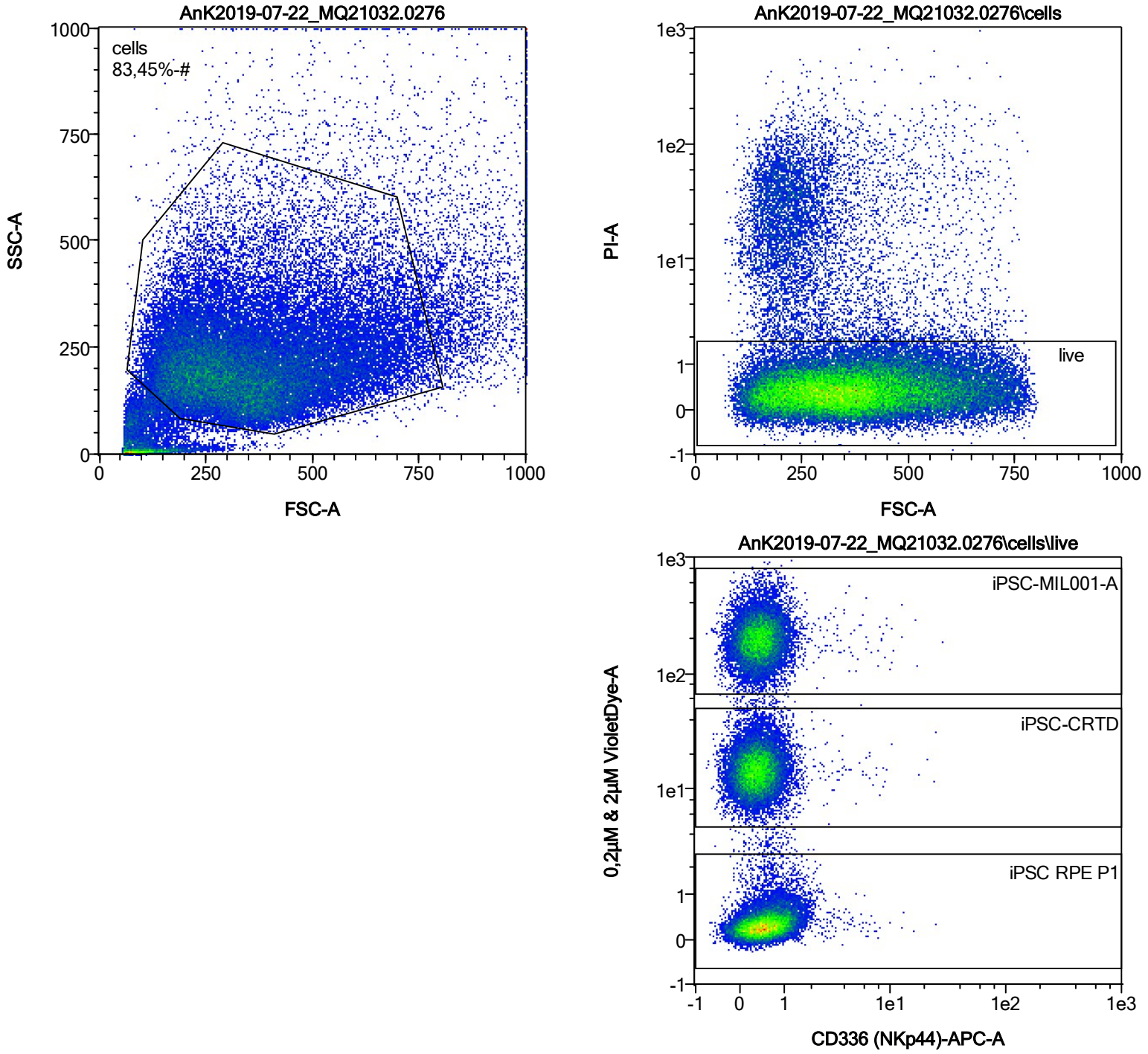

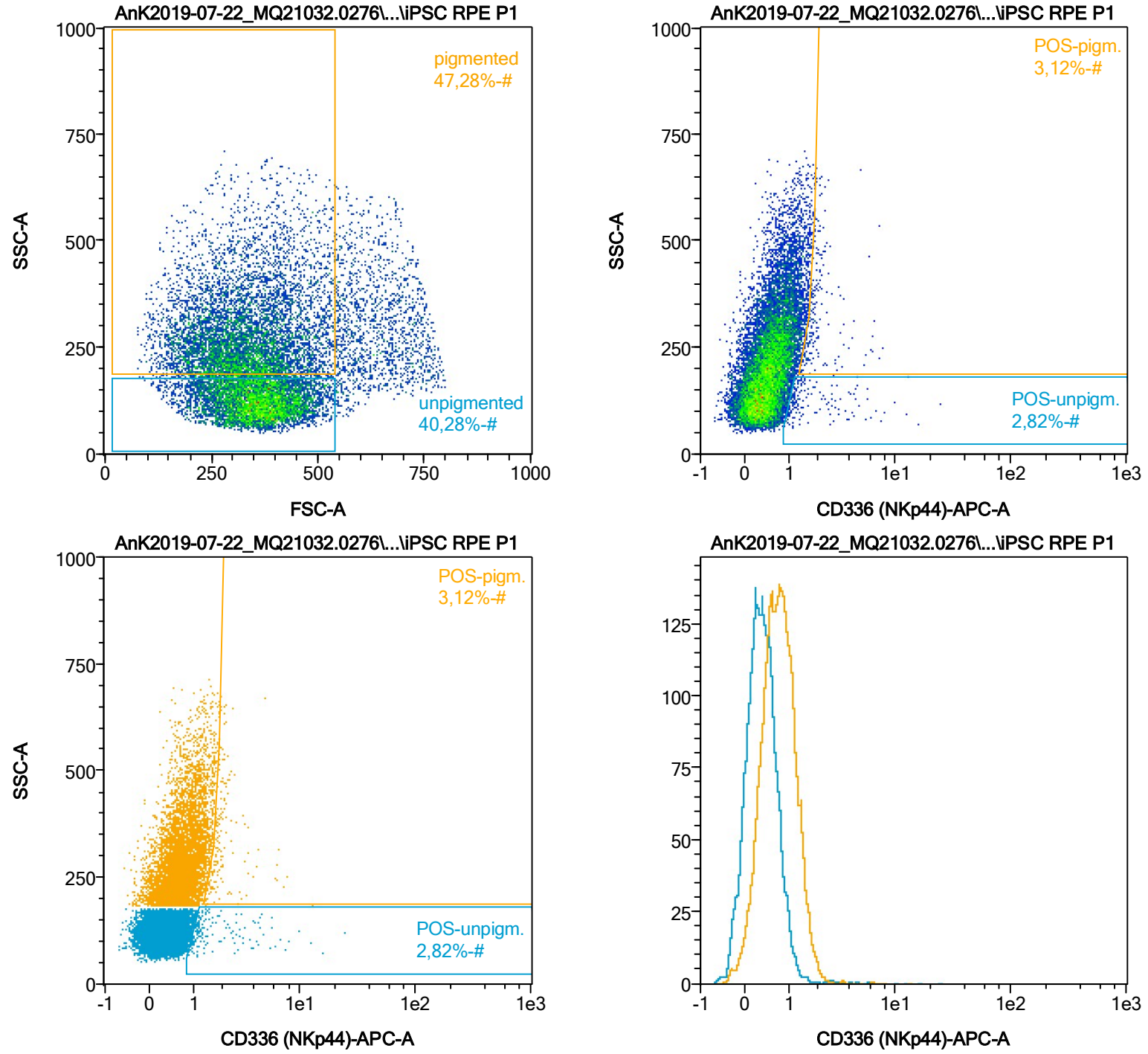

Name	%-#	CD336 (NKp44)-APC-A Mean	CD336 (NKp44)-APC-A Median
POS-unpigm.	2,82	2,21	1,26
POS-pigm.	3,12	2,13	1,85

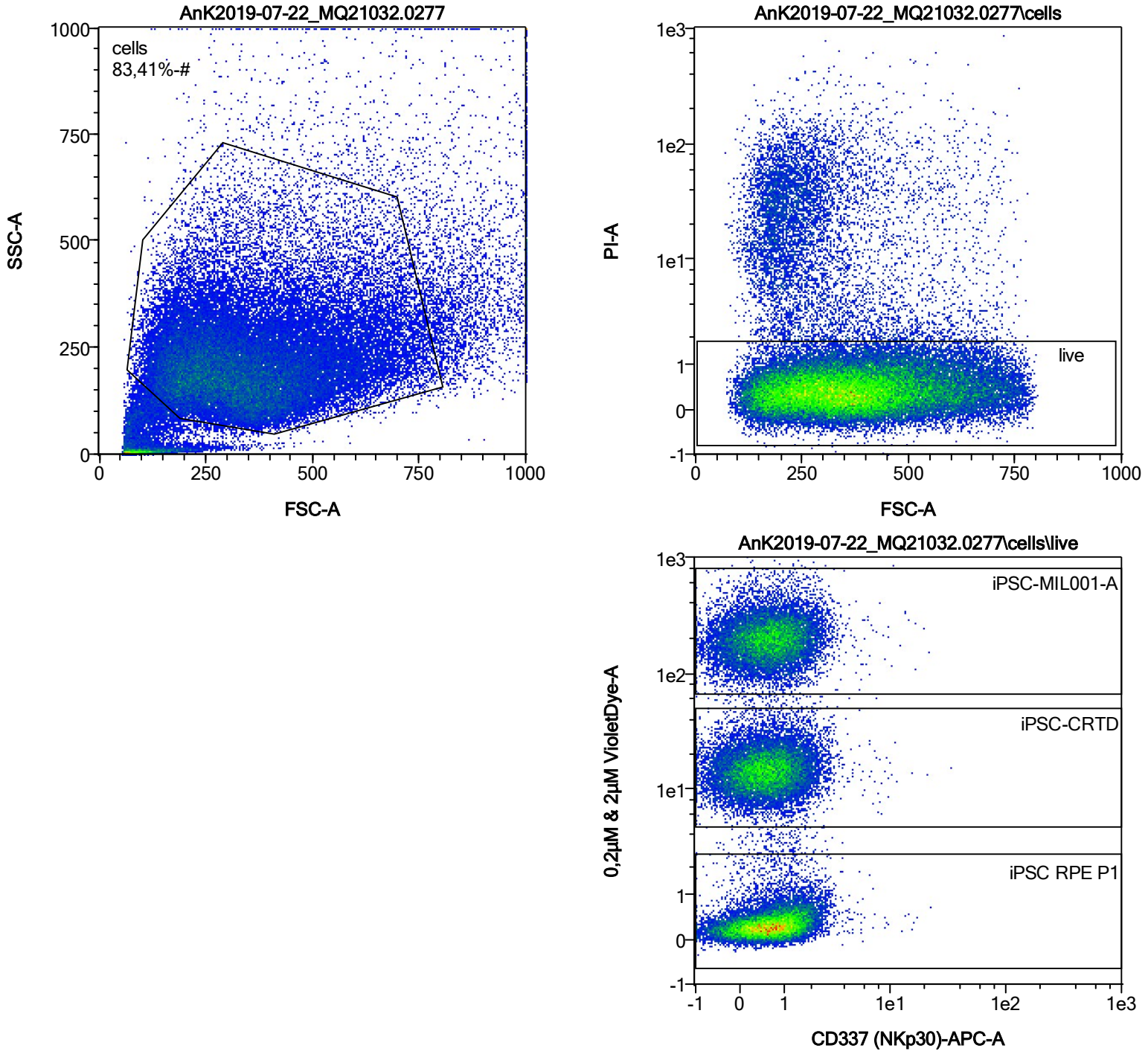

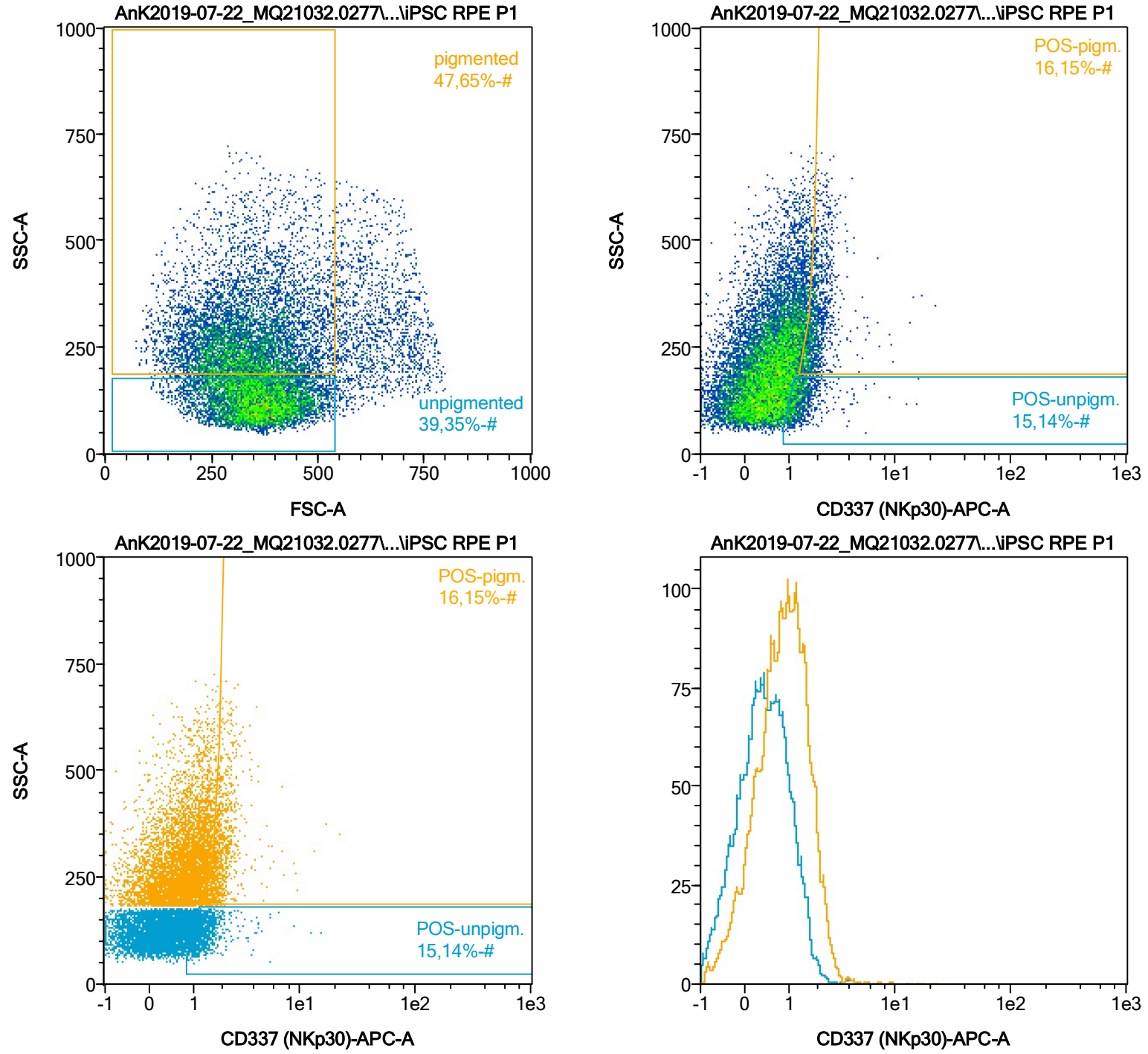

Name	%-#	CD337 (NKp30)-APC-A Mean	CD337 (NKp30)-APC-A Median
POS-unpigm.	15,14	1,45	1,29
POS-pigm.	16,15	2,07	1,88

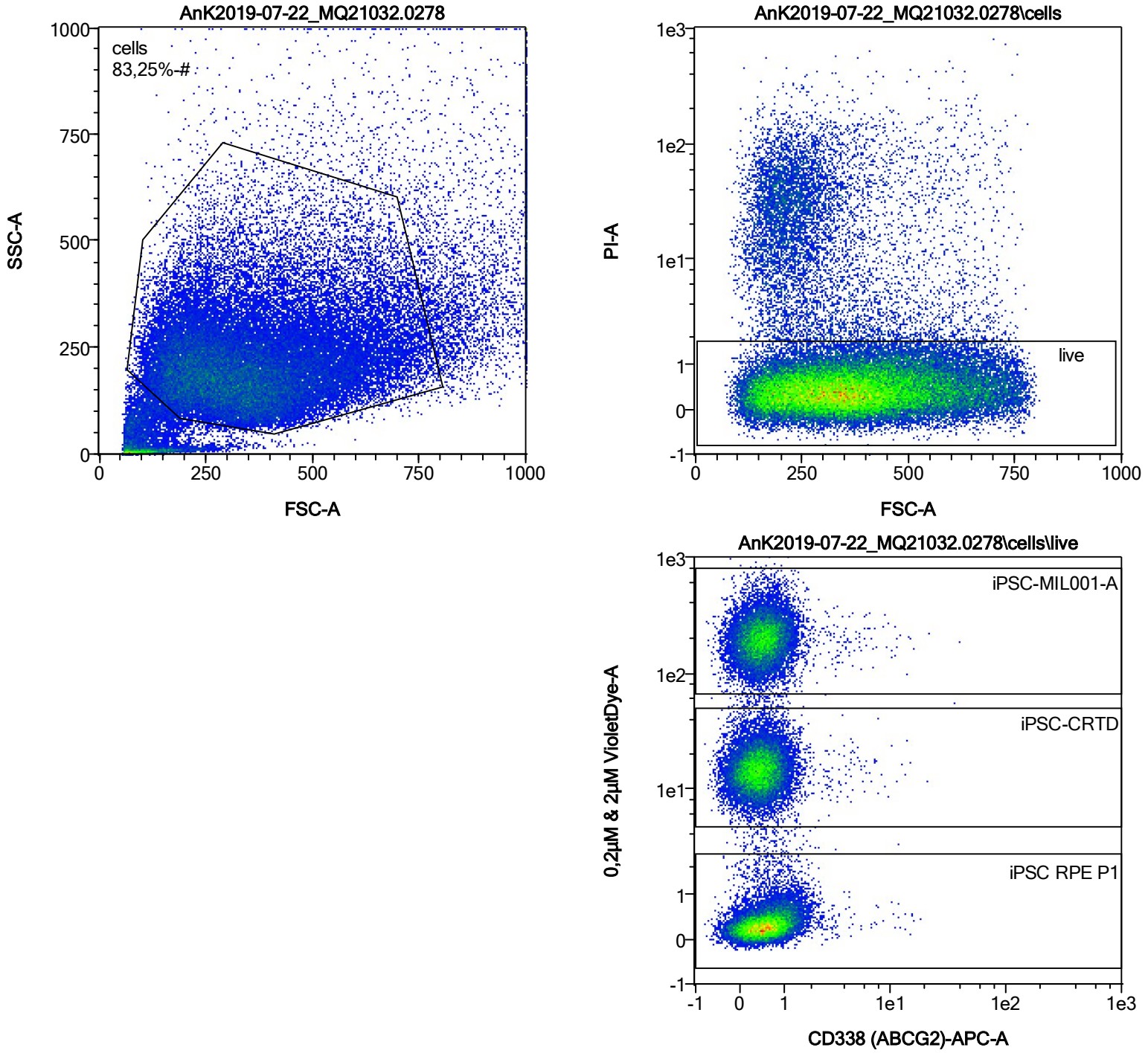

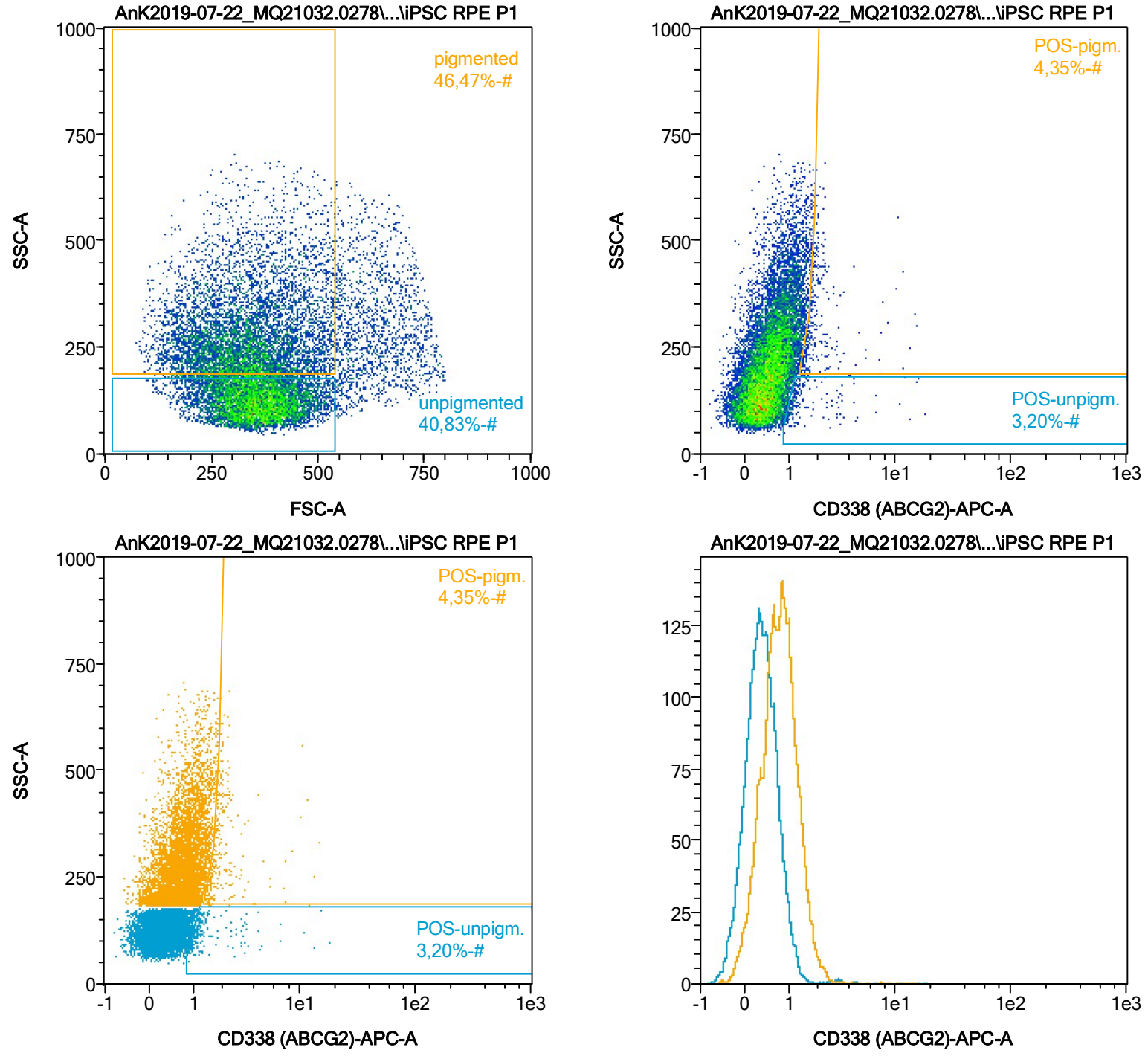

Name	%-#	CD338 (ABCG2)-APC-A Mean	CD338 (ABCG2)-APC-A Median
POS-unpigm.	3,20	1,80	1,19
POS-pigm.	4,35	2,25	1,84

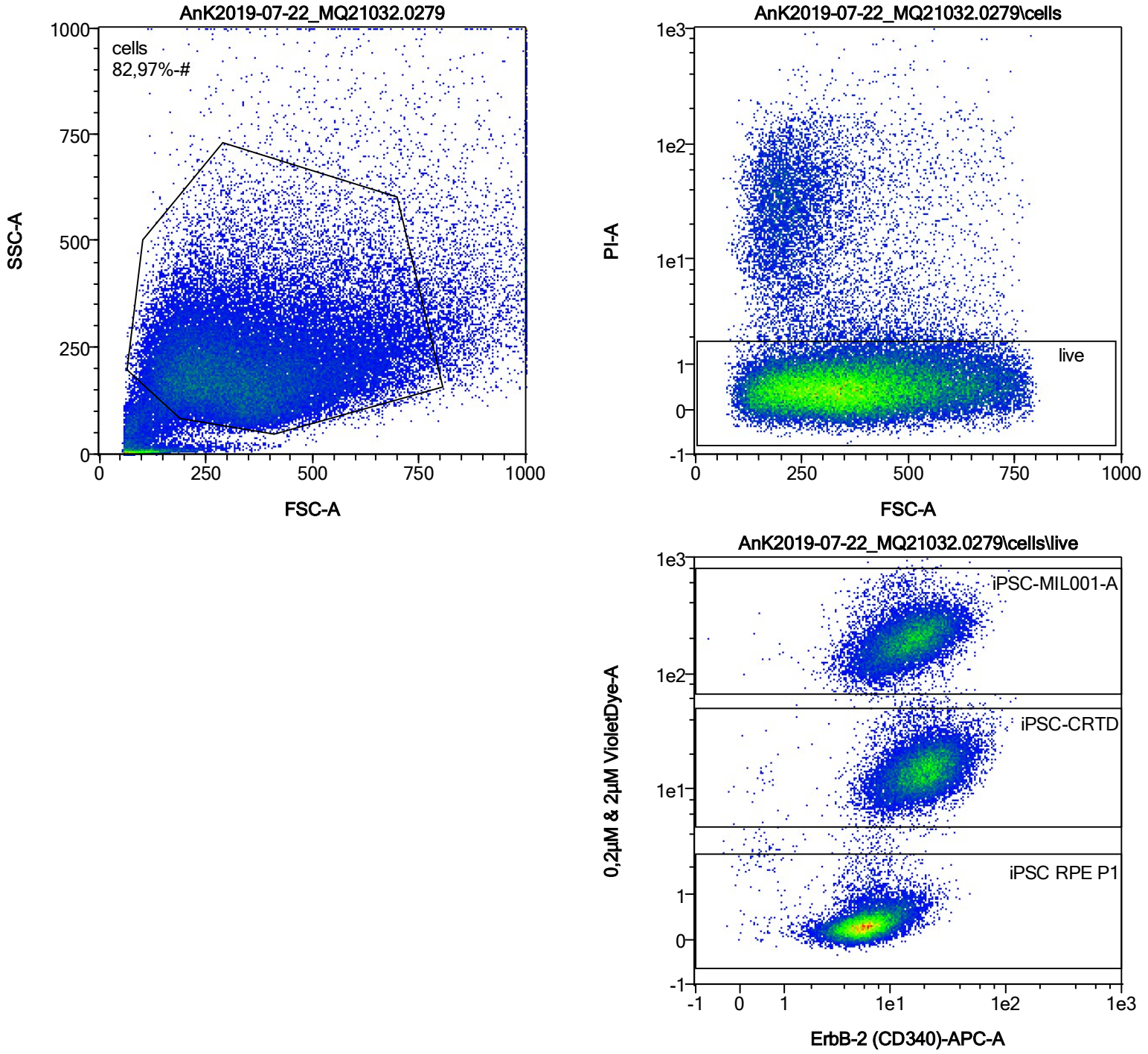

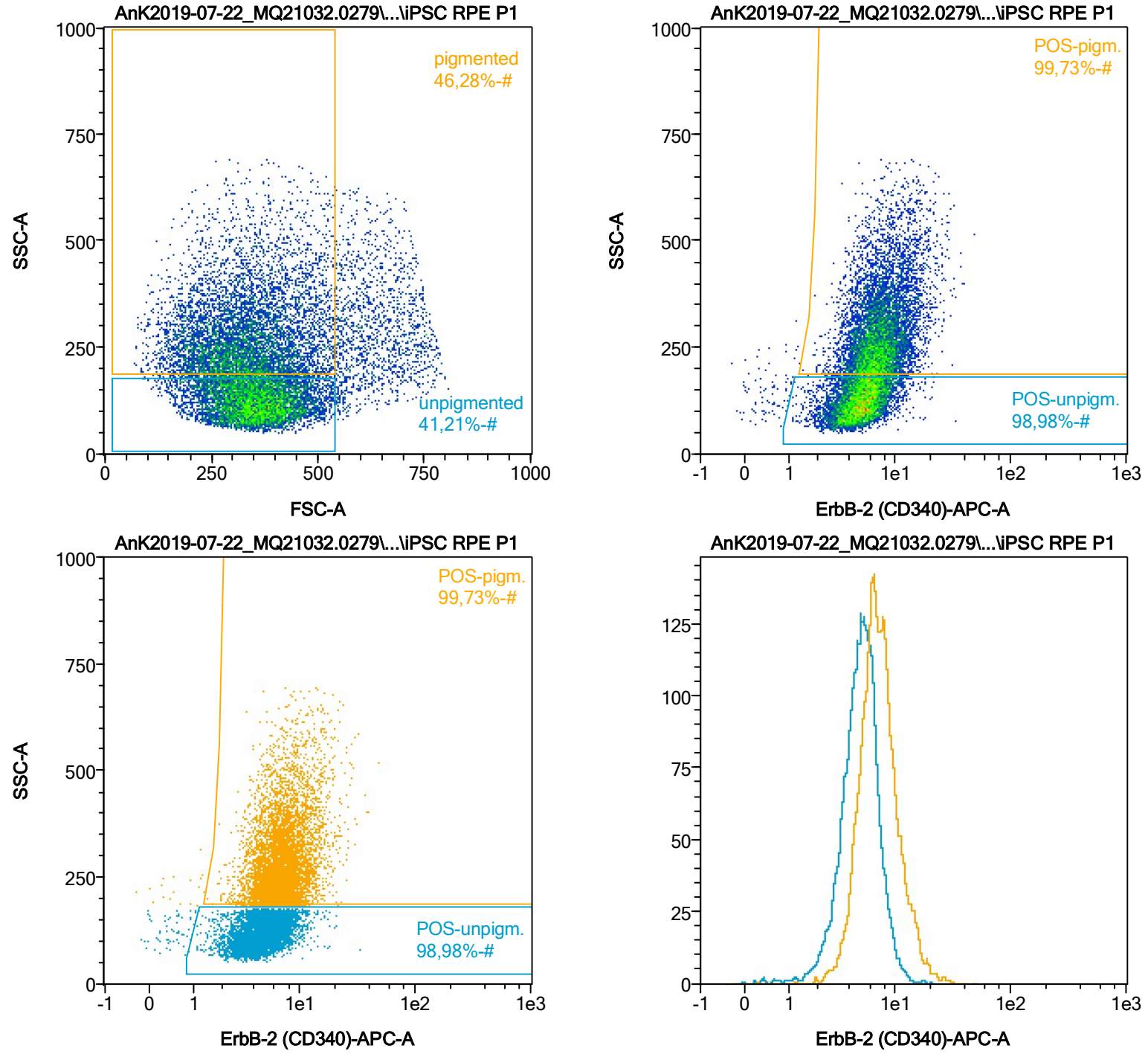

Name	%-#	ErbB-2 (CD340)-APC-A Mean	ErbB-2 (CD340)-APC-A Median
POS-unpigm.	98,98	5,18	4,97
POS-pigm.	99,73	7,62	6,91

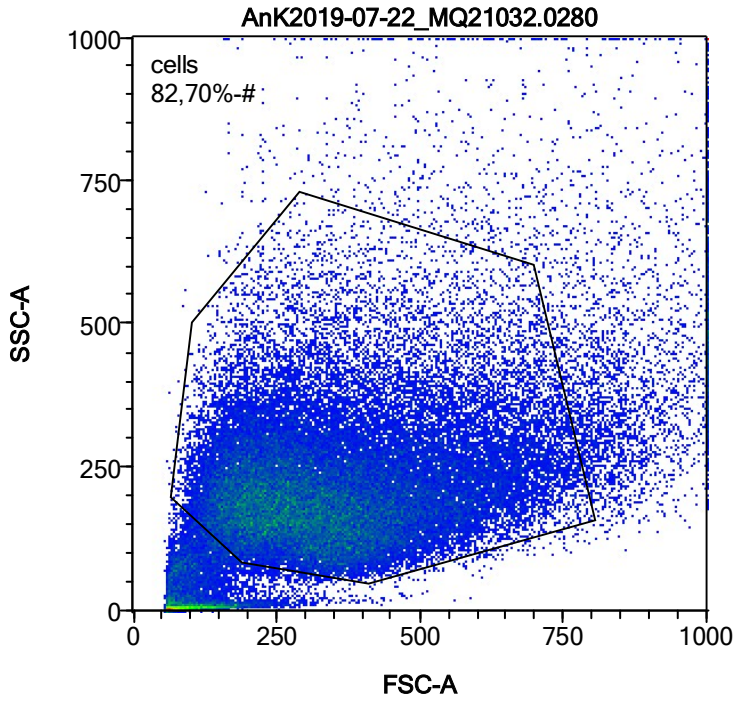

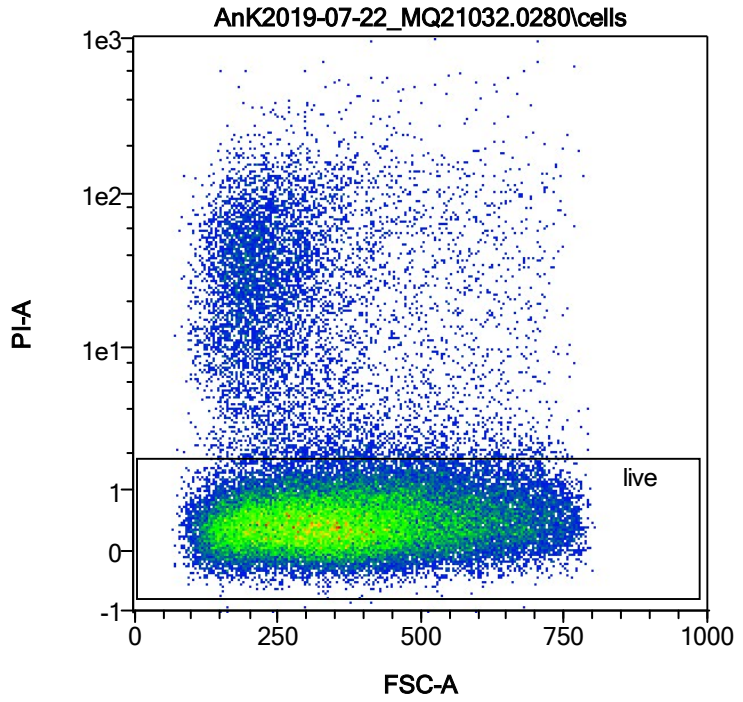

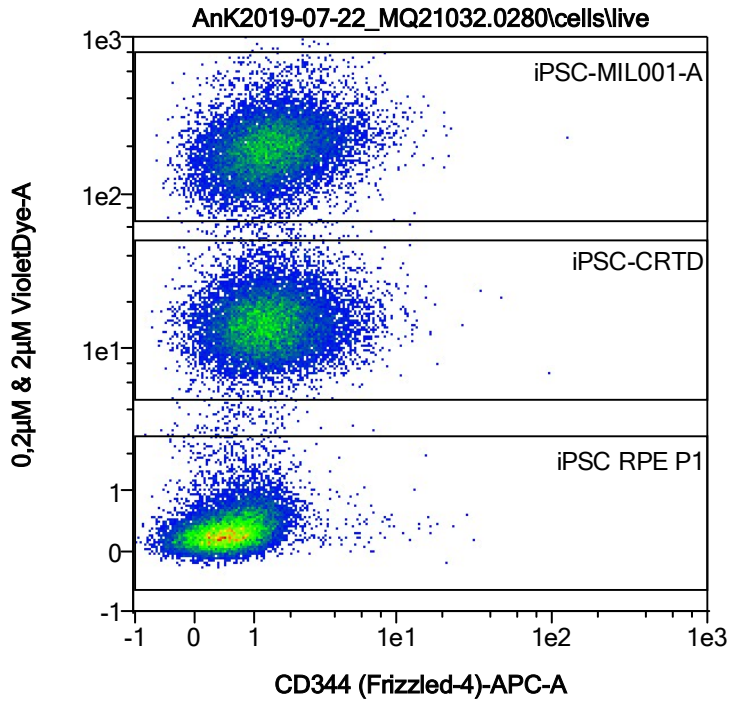

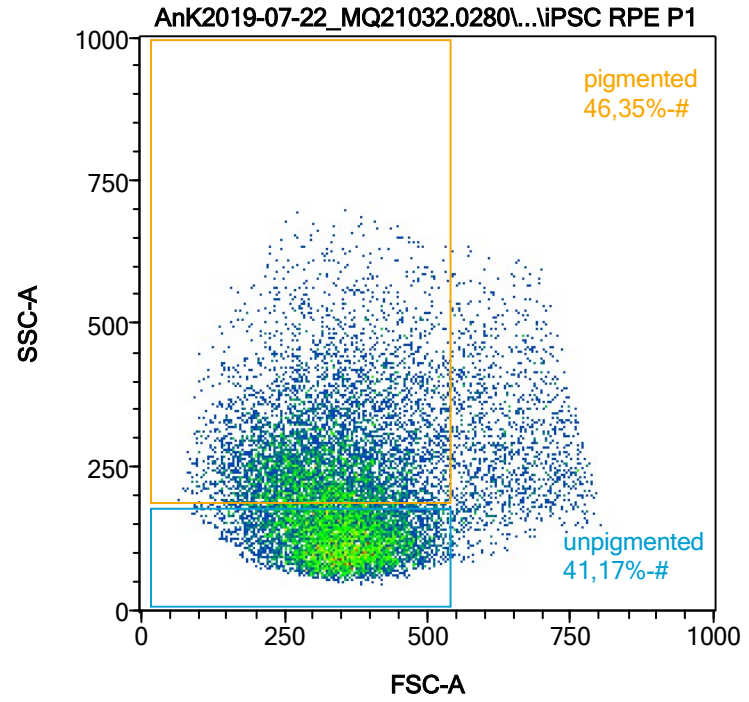

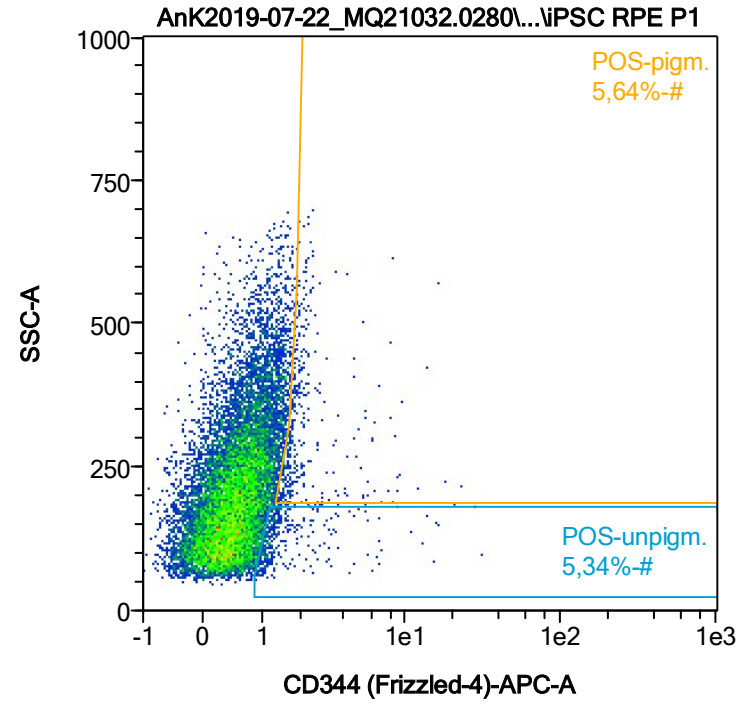

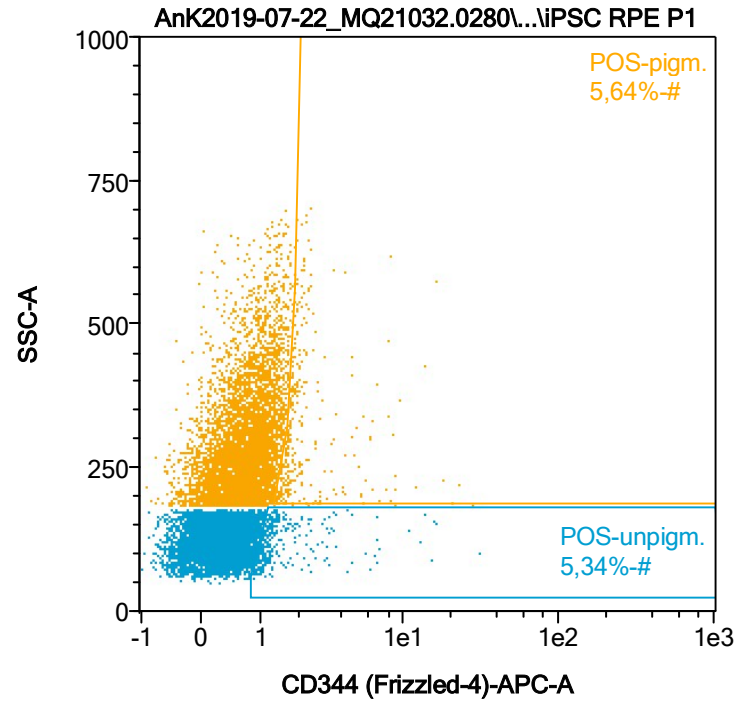

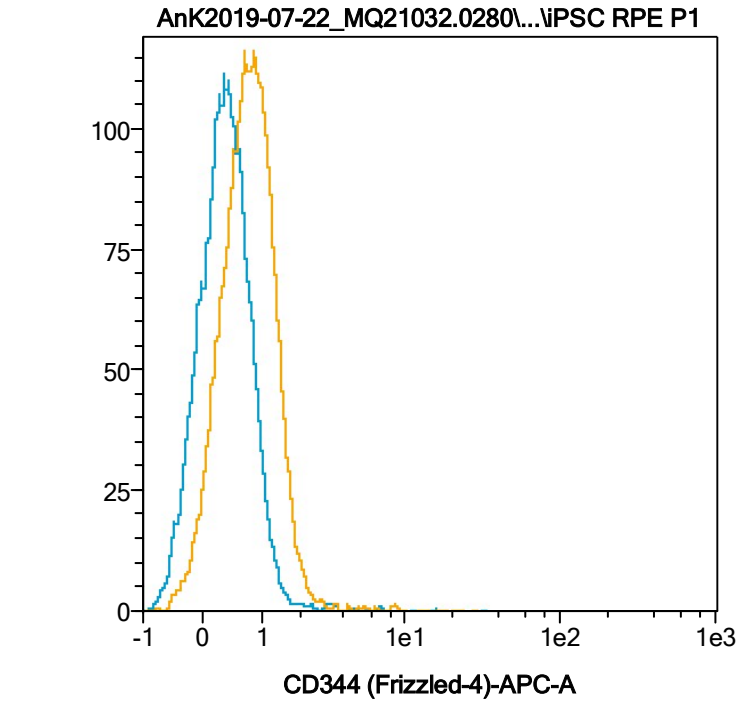

Name	%-#	CD344 (Frizzled-4)-APC-A Mean	CD344 (Frizzled-4)-APC-A Median
POS-unpigm.	5,34	1,91	1,22
POS-pigm.	5,64	2,61	1,85

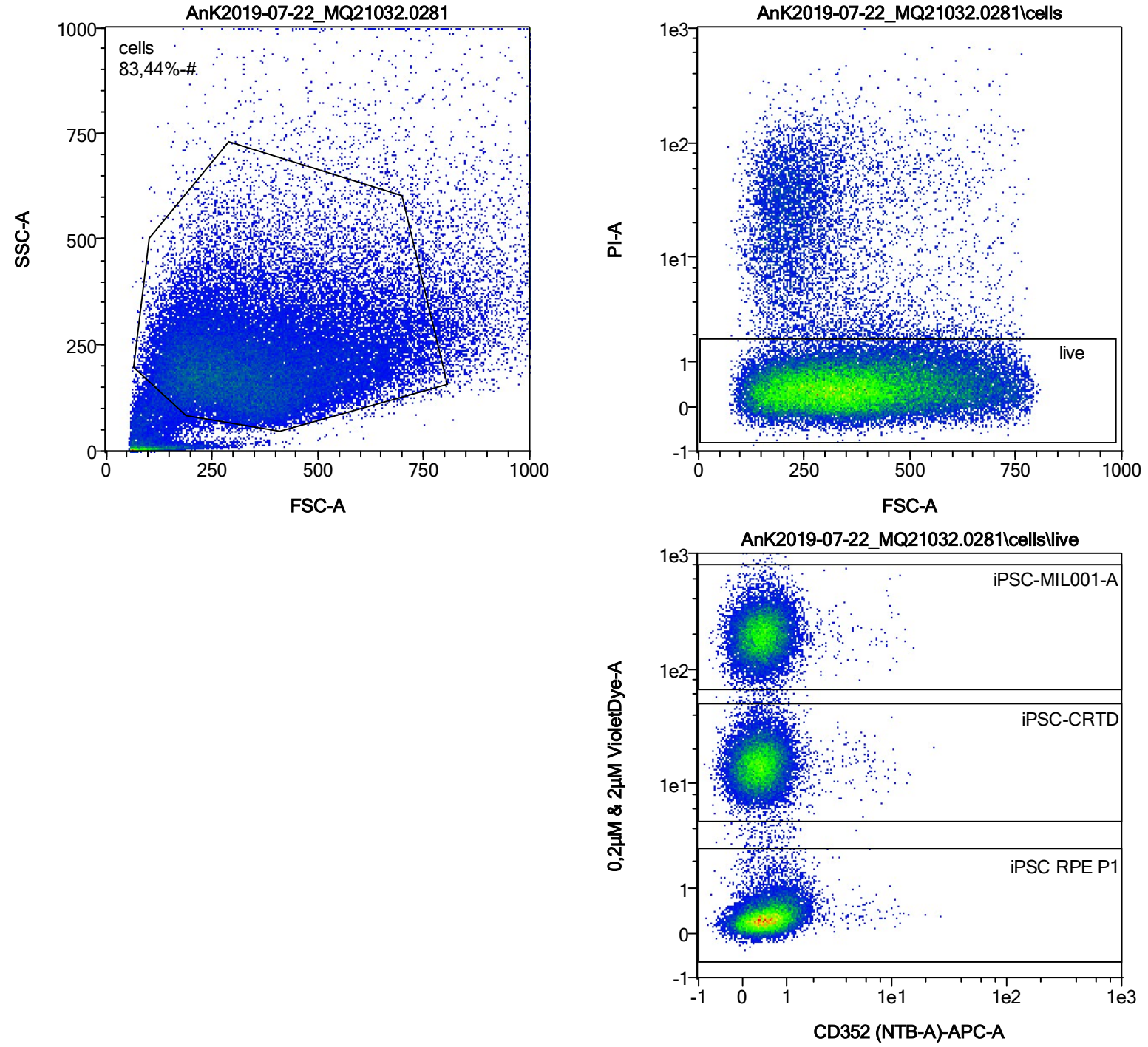

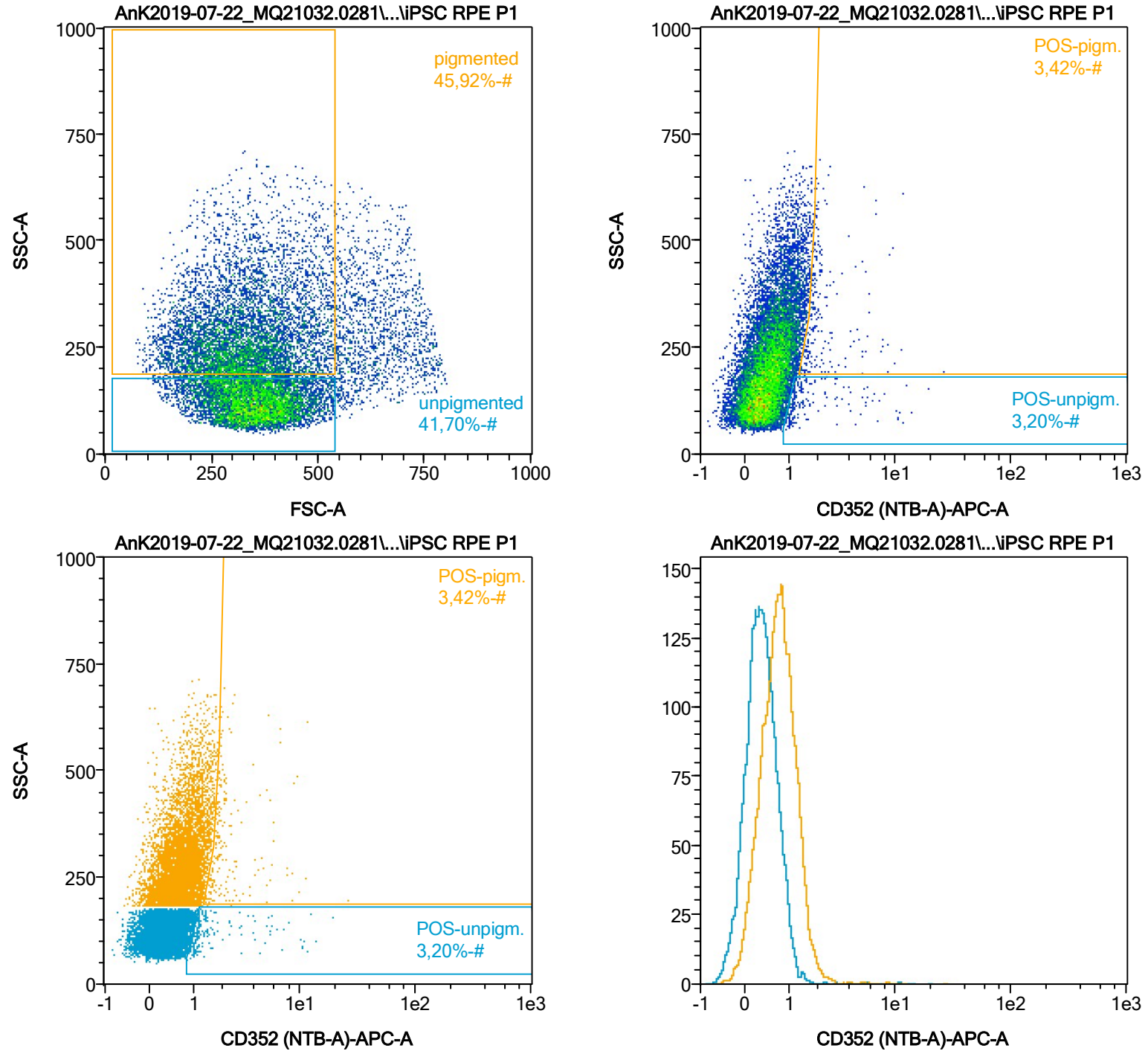

Name	%-#	CD352 (NTB-A)-APC-A Mean	CD352 (NTB-A)-APC-A Median
POS-unpigm.	3,20	2,08	1,18
POS-pigm.	3,42	2,58	1,91

Date:2024-06-14
Operator: Sebastian Knoebel
Version:2.13.0+54.g95abf0f4

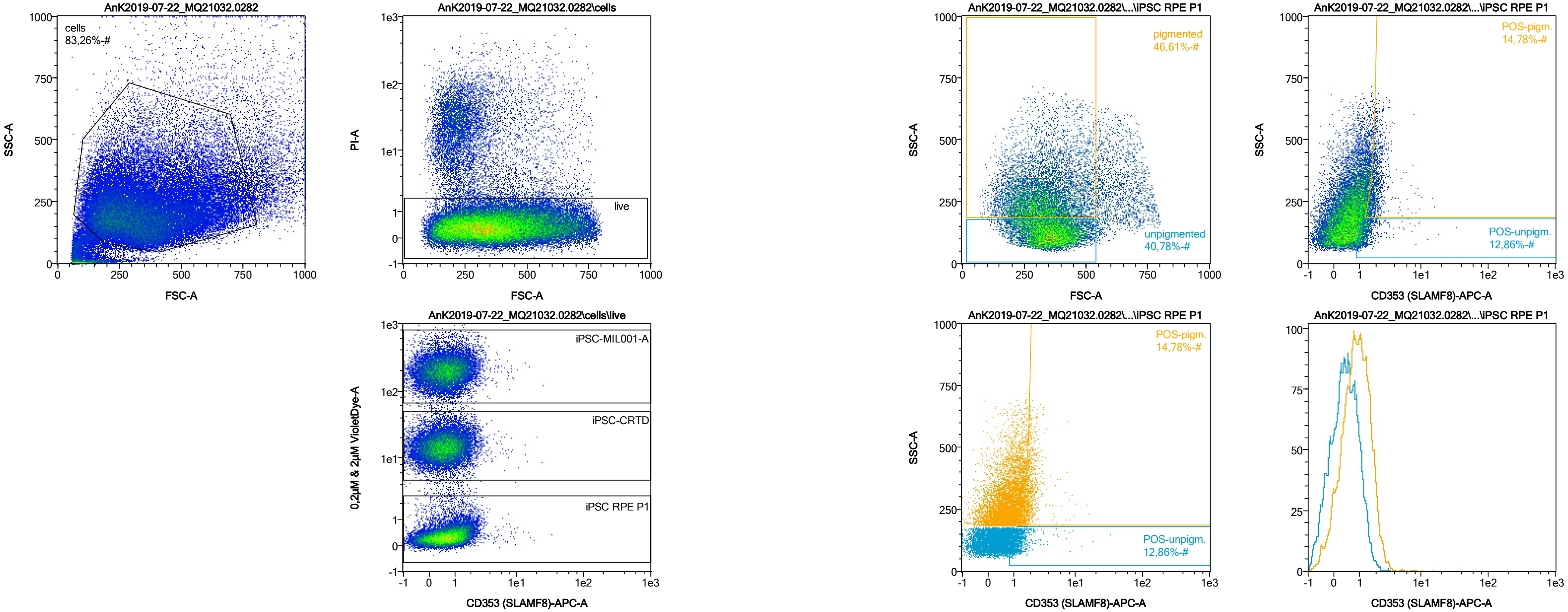

Name	%-#	CD353 (SLAMF8)-APC-A Mean	CD353 (SLAMF8)-APC-A Median
POS-unpigm.	12,86	1,47	1,25
POS-pigm.	14,78	2,05	1,87

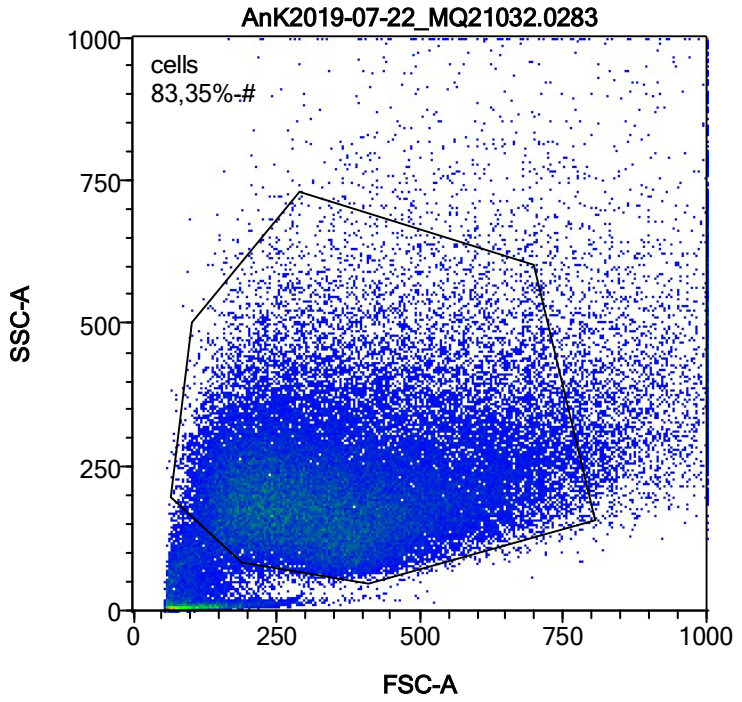

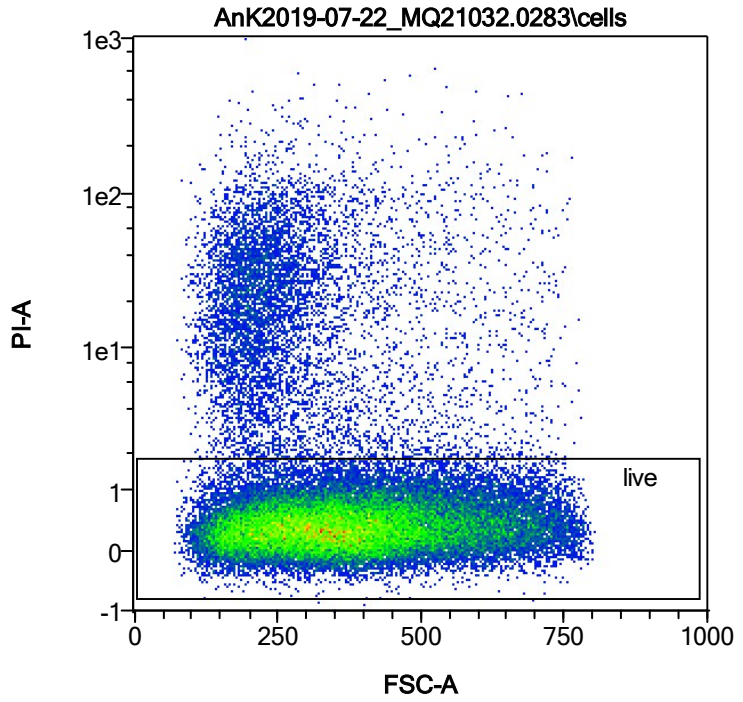

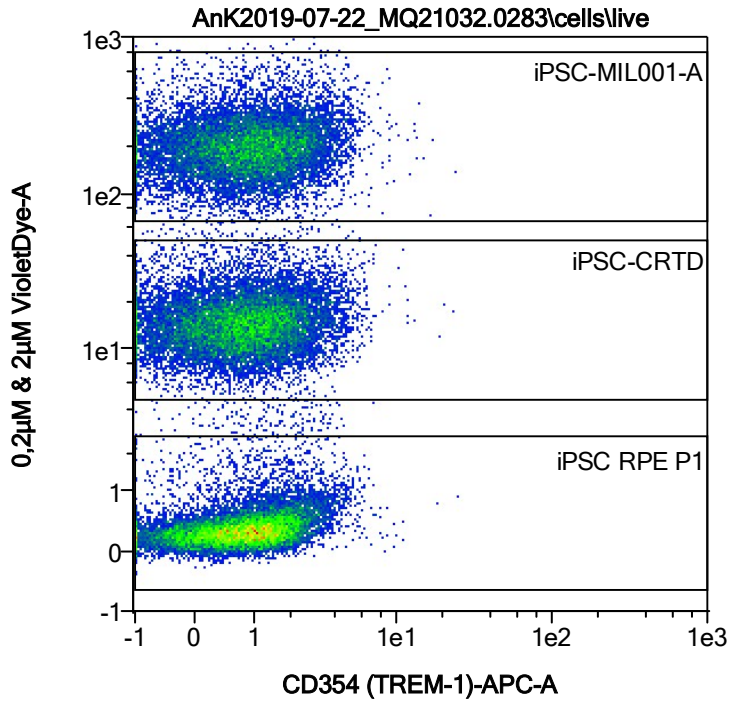

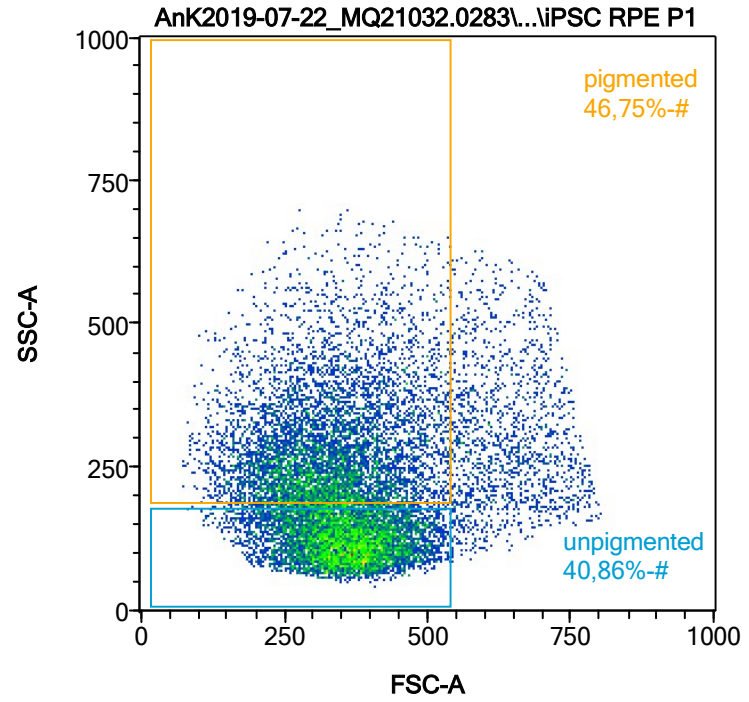

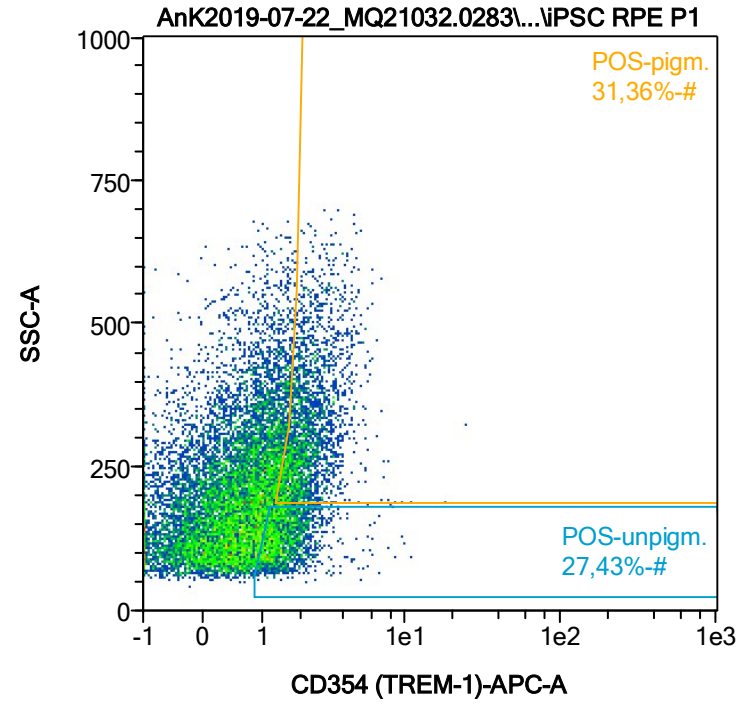

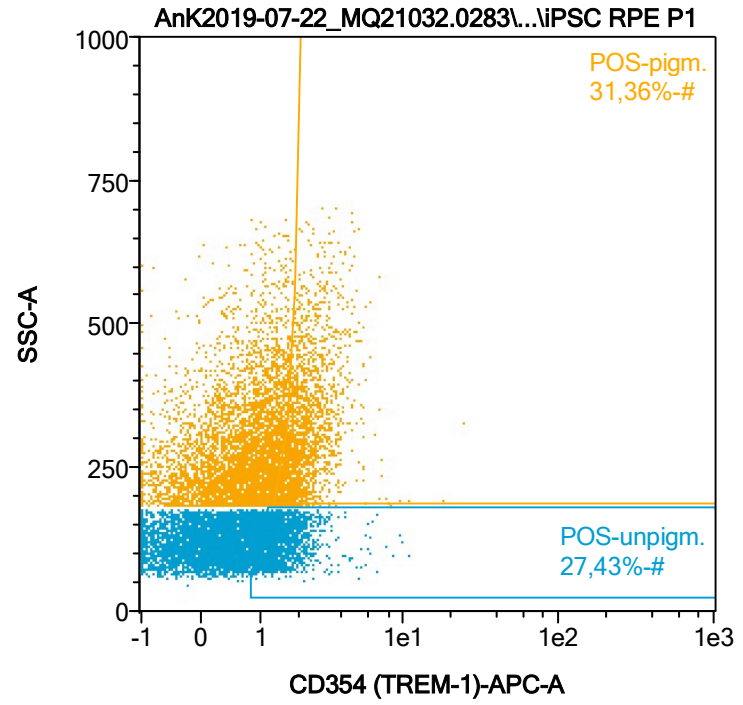

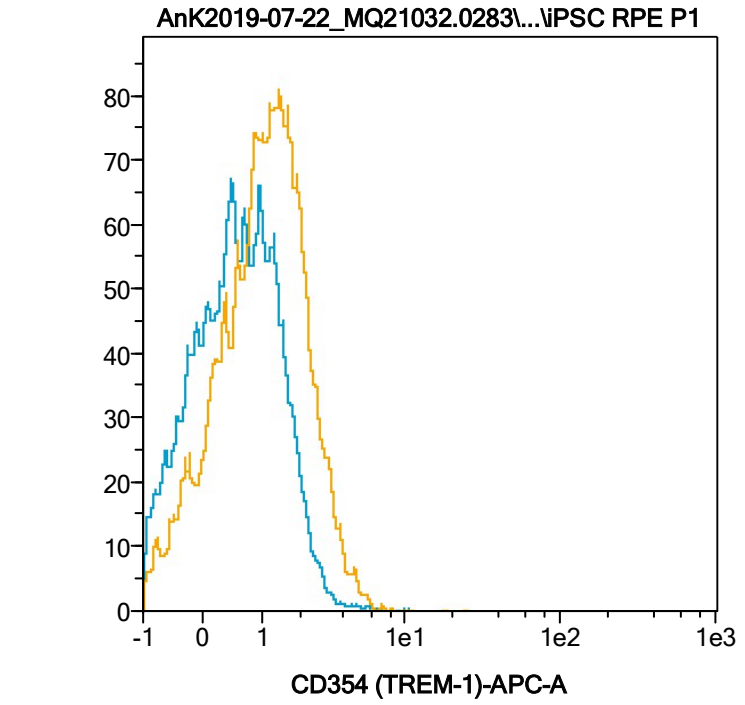

Name	%-#	CD354 (TREM-1)-APC-A Mean	CD354 (TREM-1)-APC-A Median
POS-unpigm.	27,43	1,62	1,43
POS-pigm.	31,36	2,35	2,09

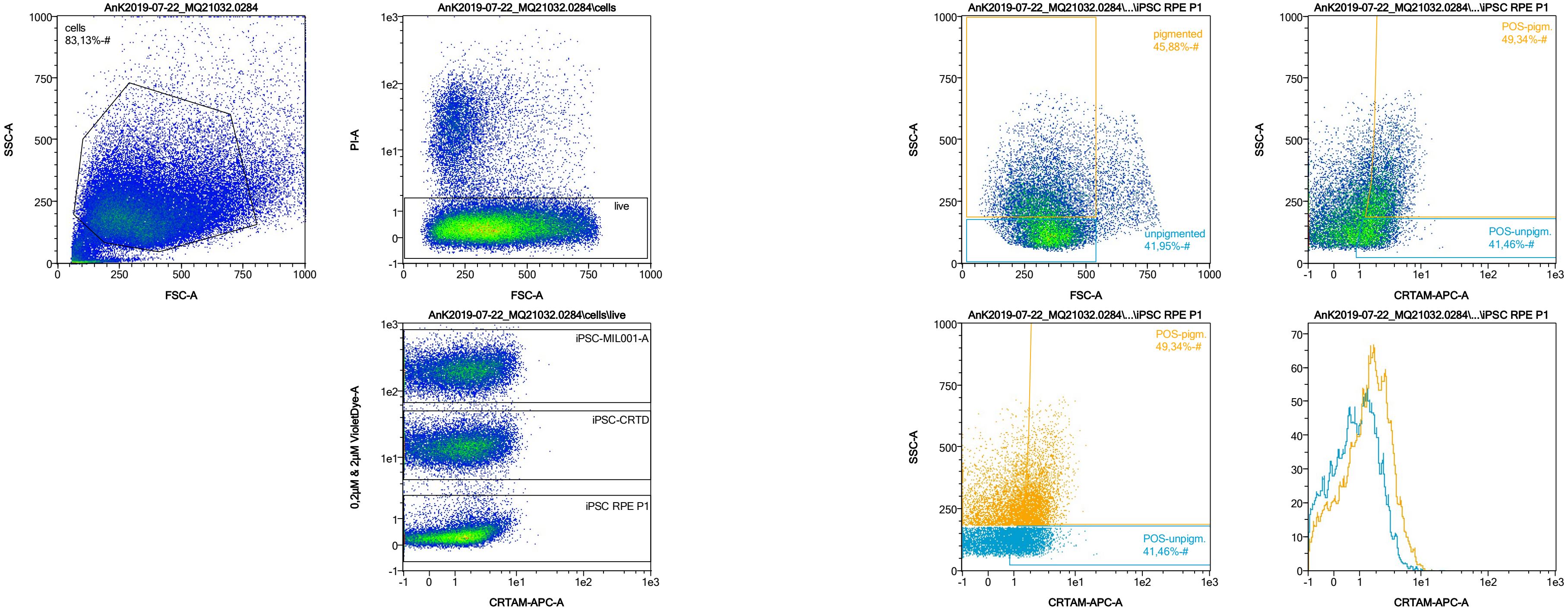

Name	%-#	CRTAM-APC-A Mean	CRTAM-APC-A Median
POS-unpigm.	41,46	2,08	1,78
POS-pigm.	49,34	2,97	2,58

Date:2024-06-14

Operator: Sebastian Knoebel

Version:2.13.0+54.g95abf0f4

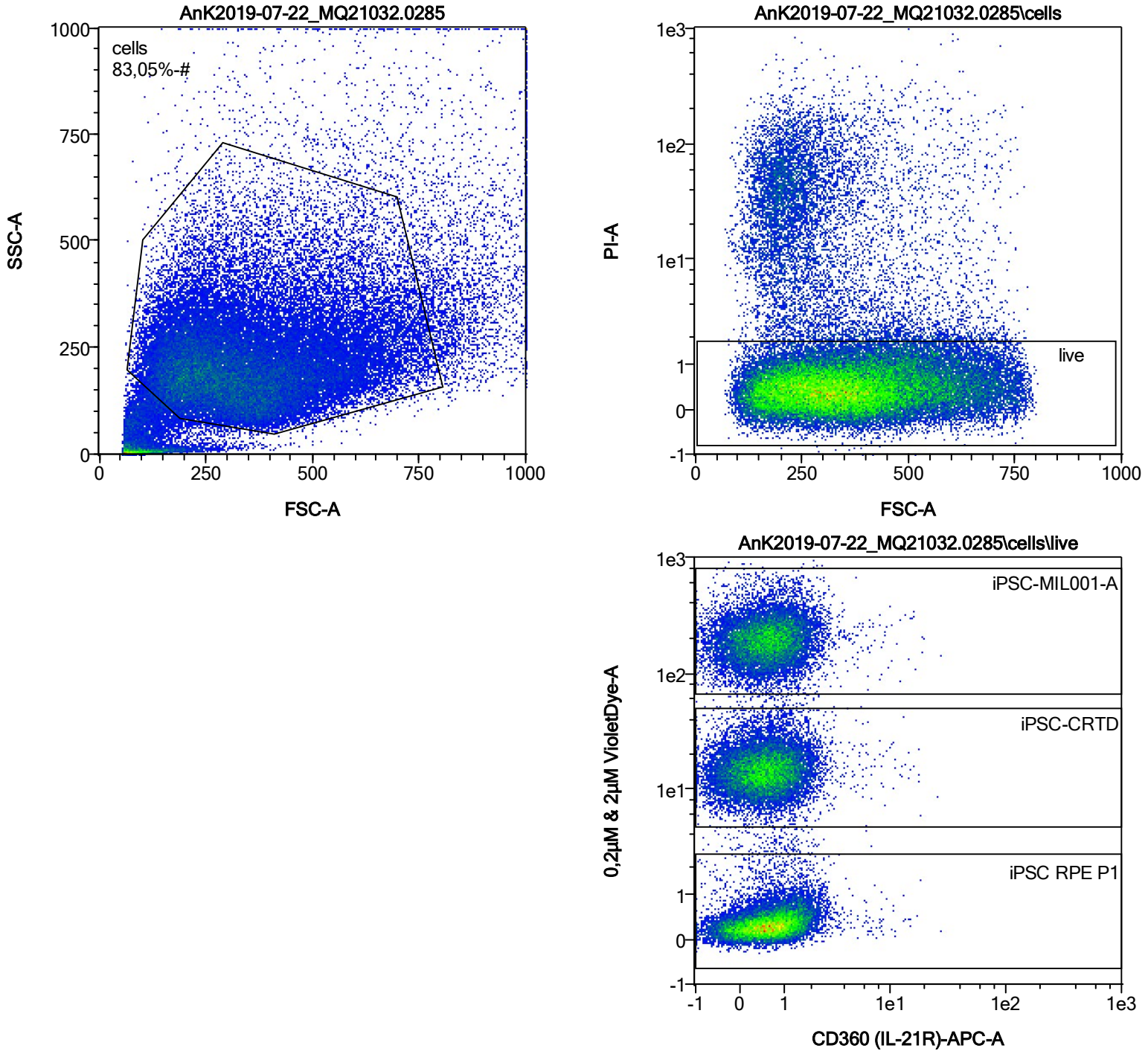

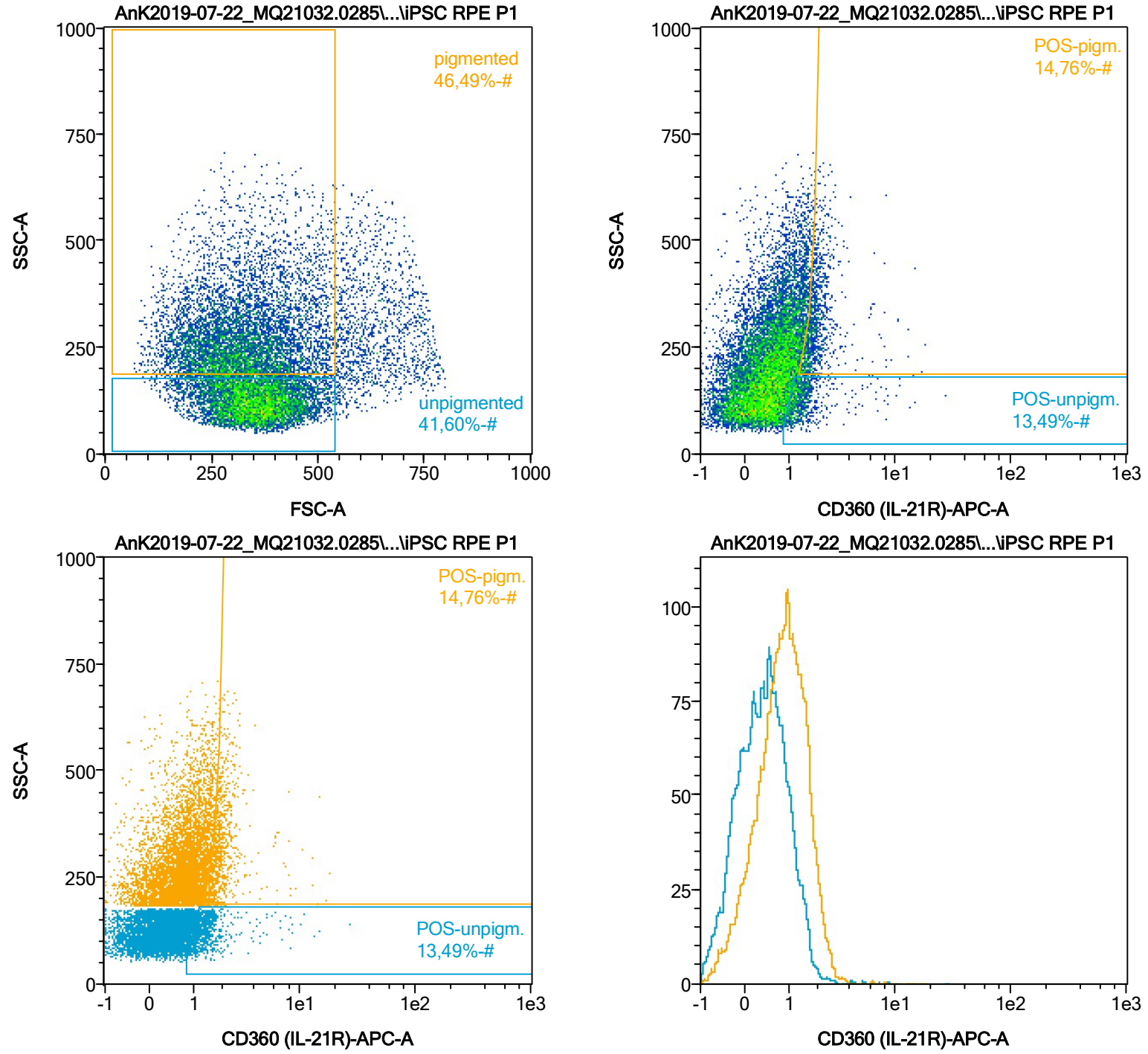

Name	%-#	CD360 (IL-21R)-APC-A Mean	CD360 (IL-21R)-APC-A Median
POS-unpigm.	13,49	1,59	1,29
POS-pigm.	14,76	2,11	1,86

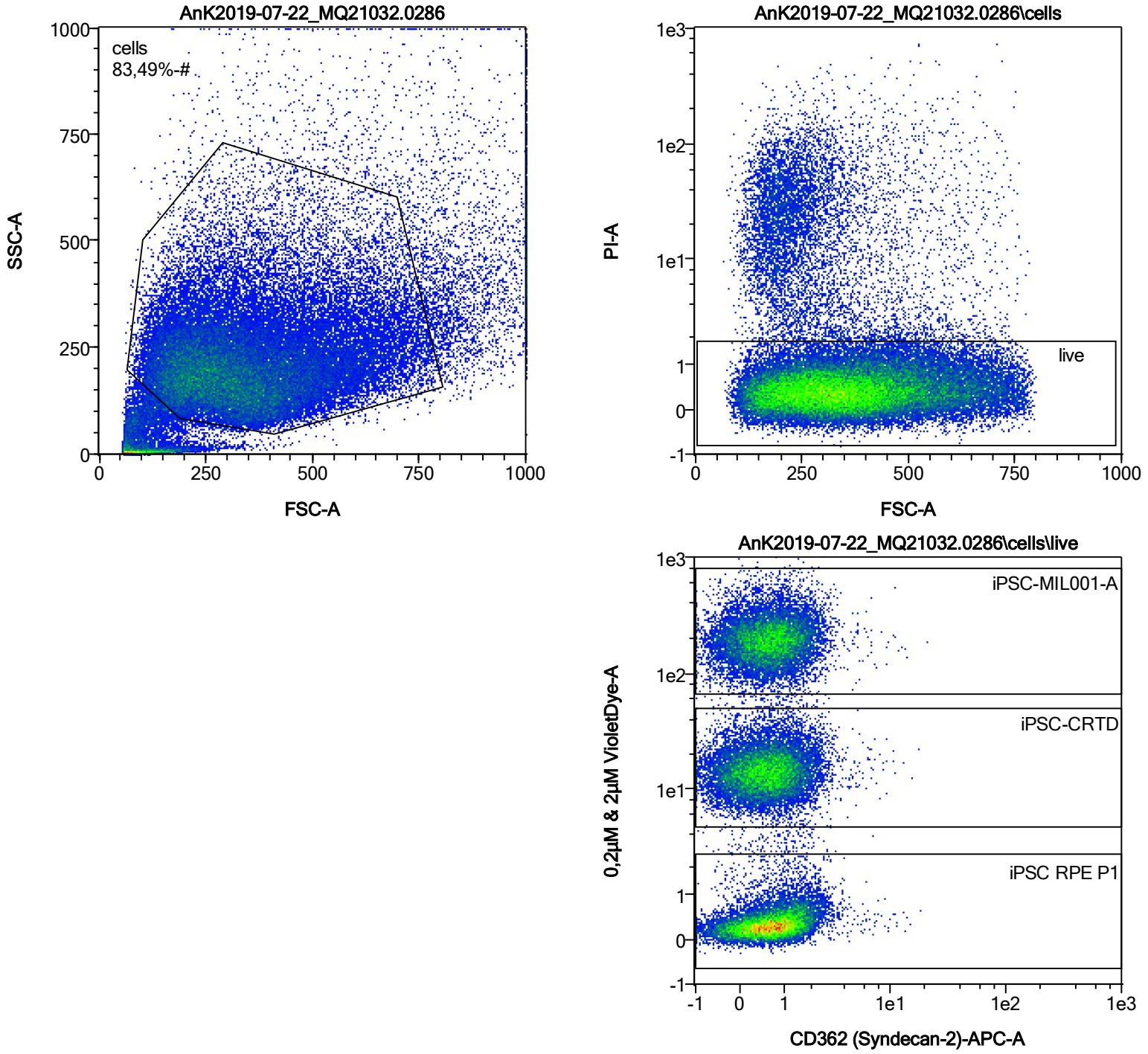

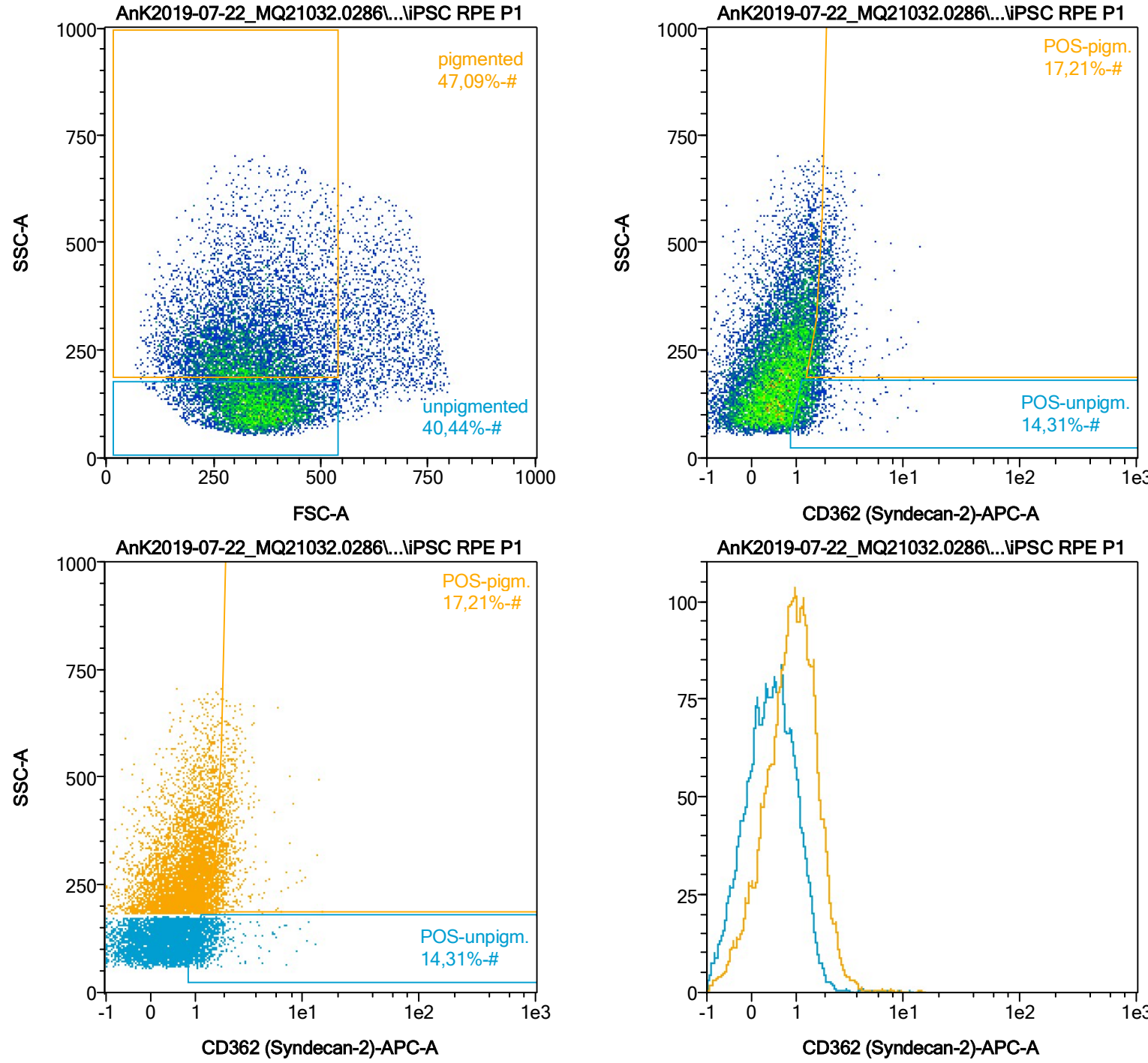

Name	%-#	CD362 (Syndecan-2)-APC-A Mean	CD362 (Syndecan-2)-APC-A Median
POS-unpigm.	14,31	1,50	1,27
POS-pigm.	17,21	2,12	1,91

Date:2024-06-14

Operator: Sebastian Knoebel

Version:2.13.0+54.g95abf0f4

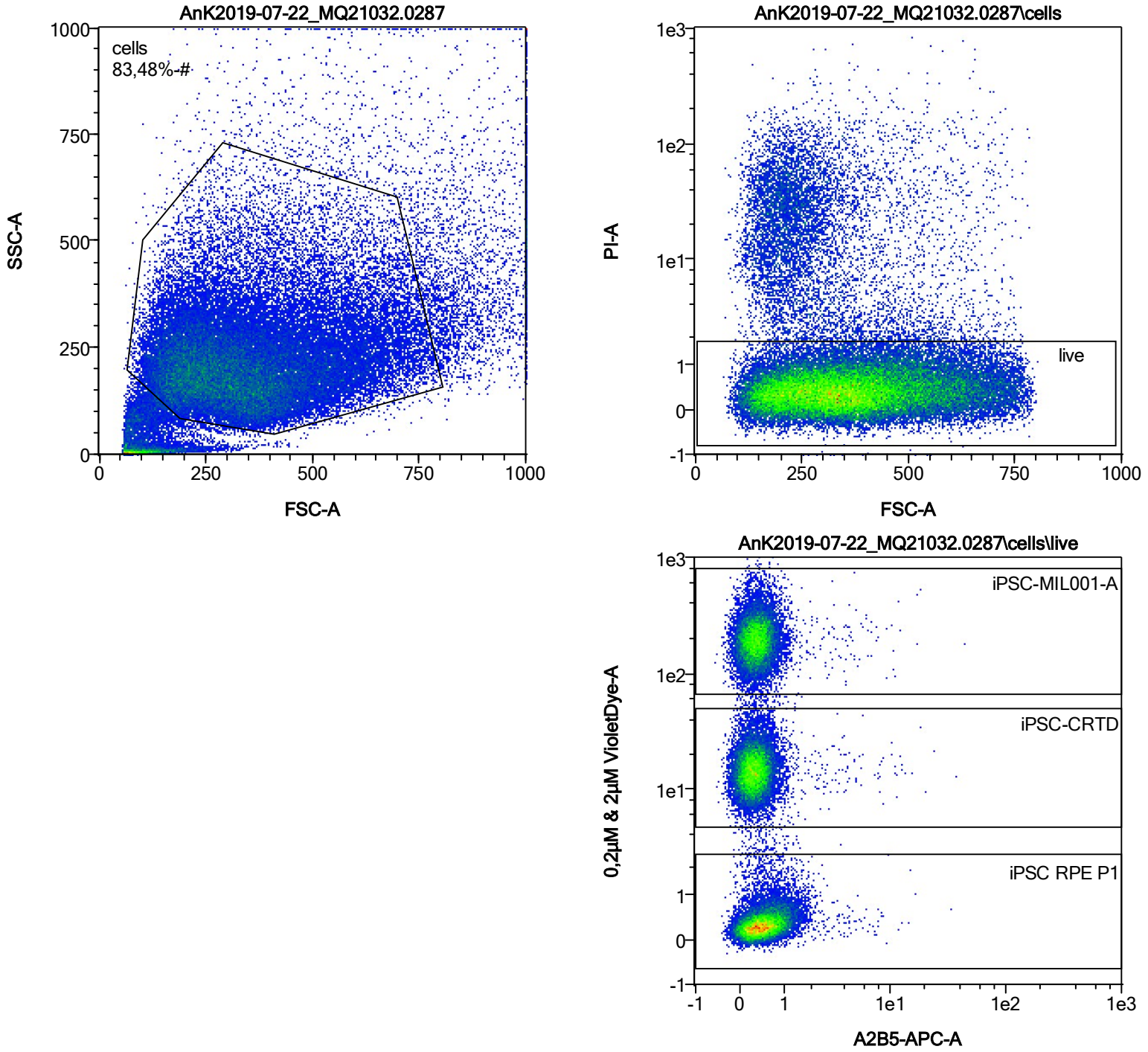

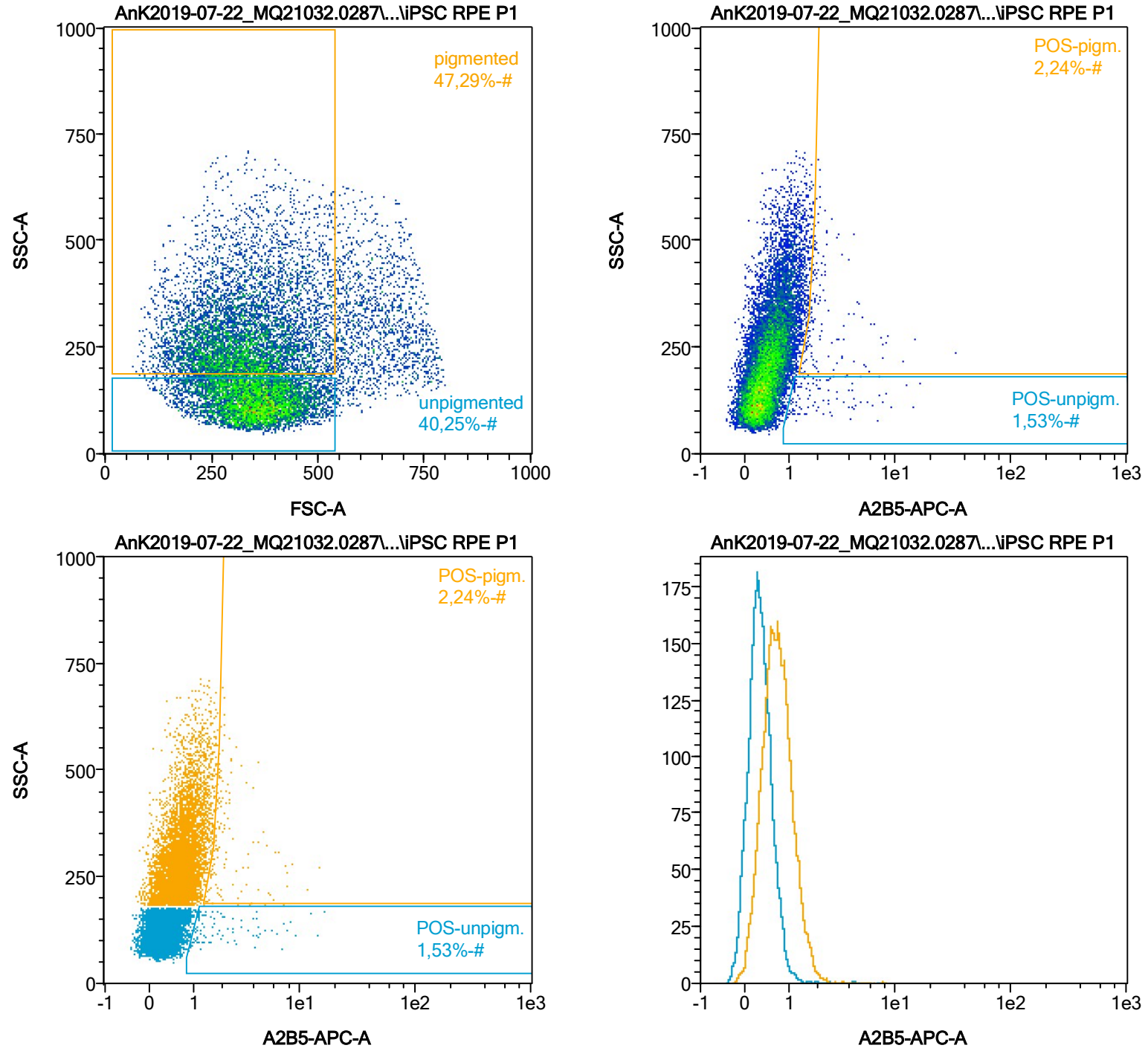

Name	%-#	A2B5-APC-A Mean	A2B5-APC-A Median
POS-unpigm.	1,53	2,77	1,60
POS-pigm.	2,24	2,59	1,92

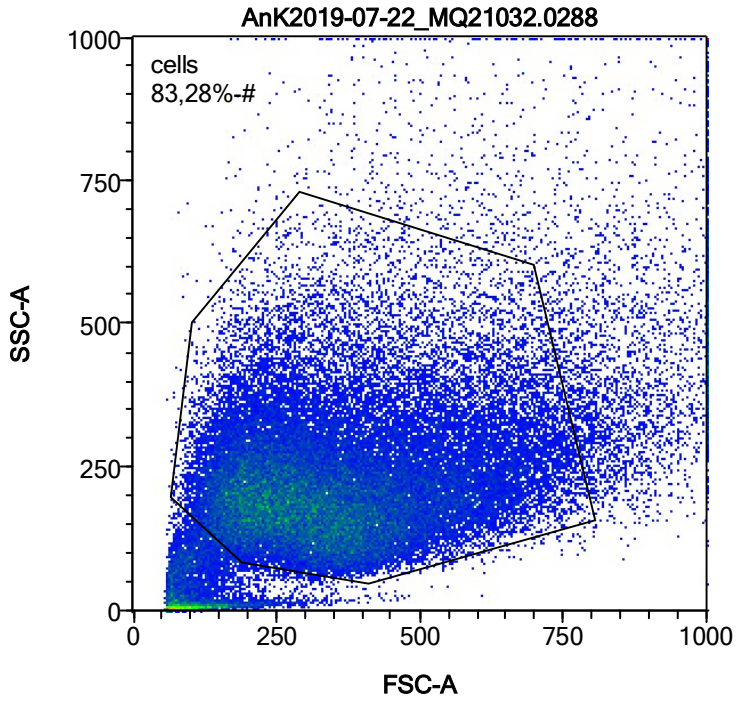

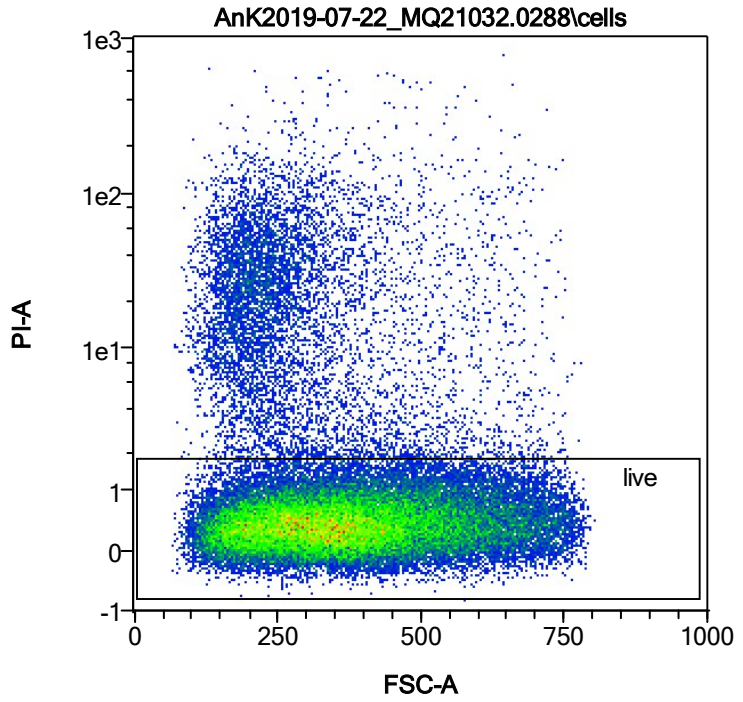

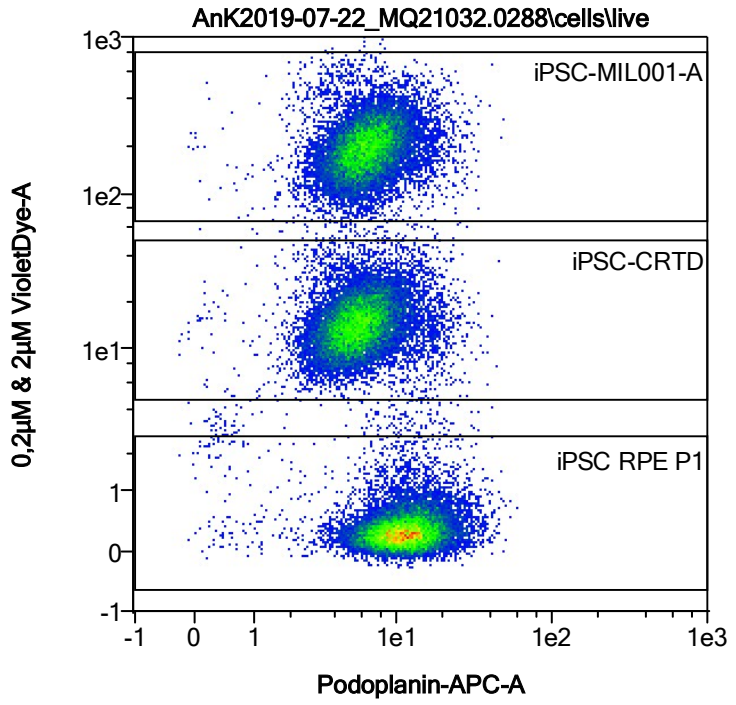

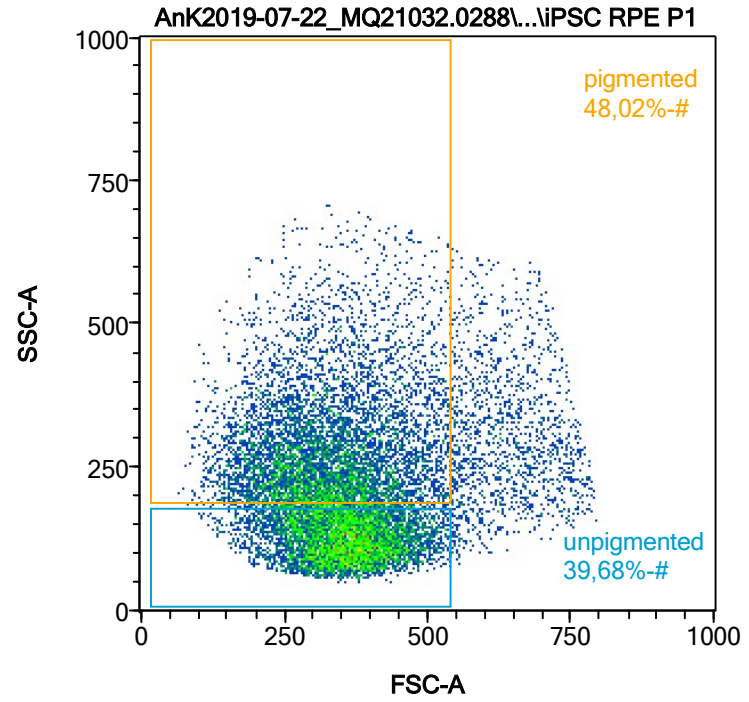

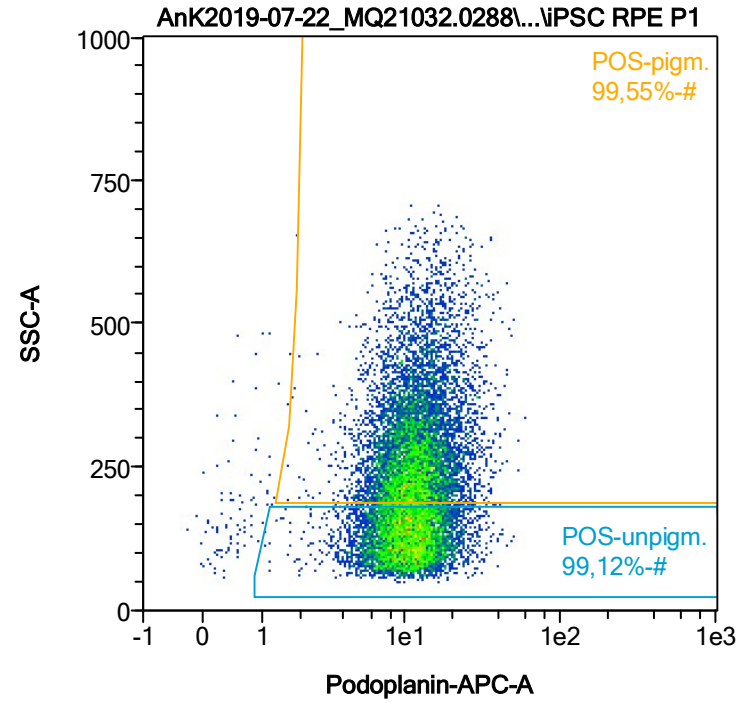

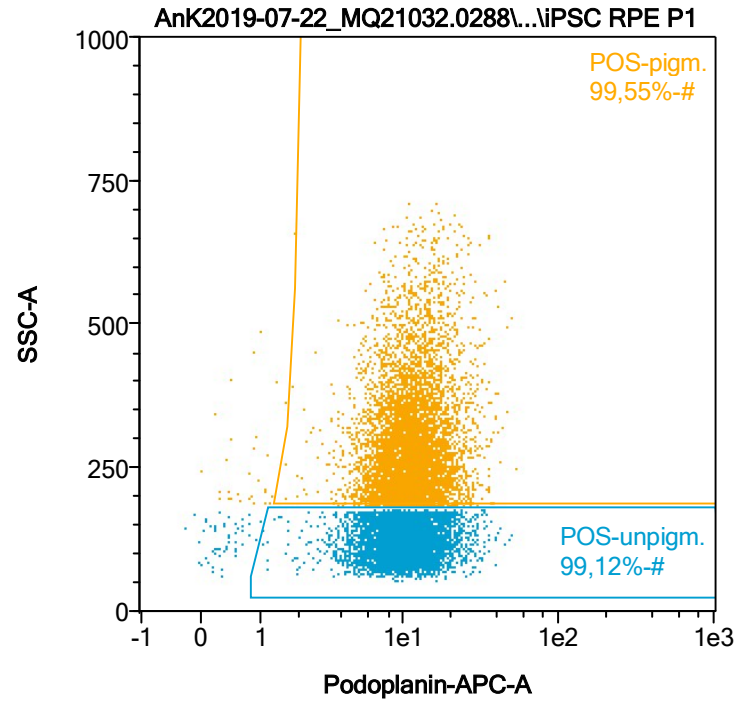

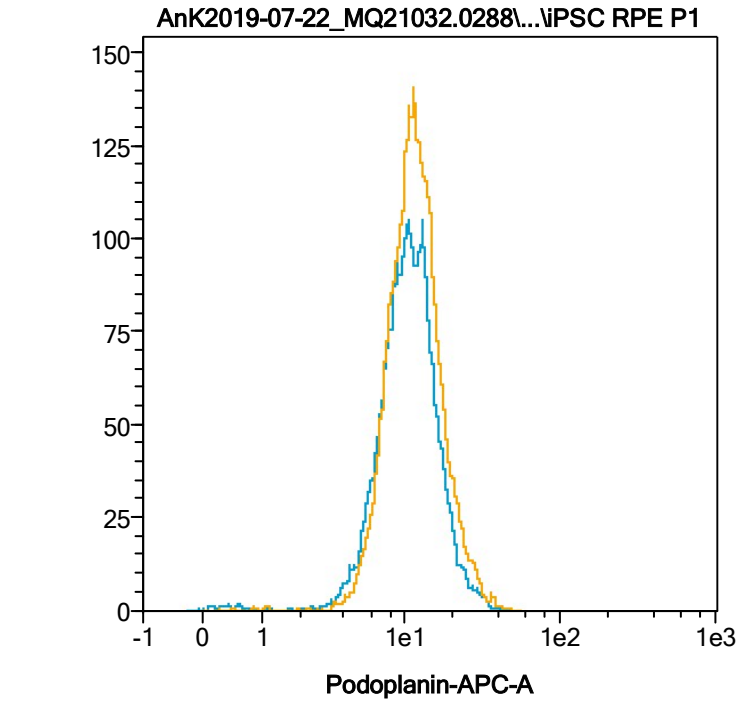

Name	%-#	Podoplanin-APC-A Mean	Podoplanin-APC-A Median
POS-unpigm.	99,12	11,21	10,47
POS-pigm.	99,55	12,20	11,24

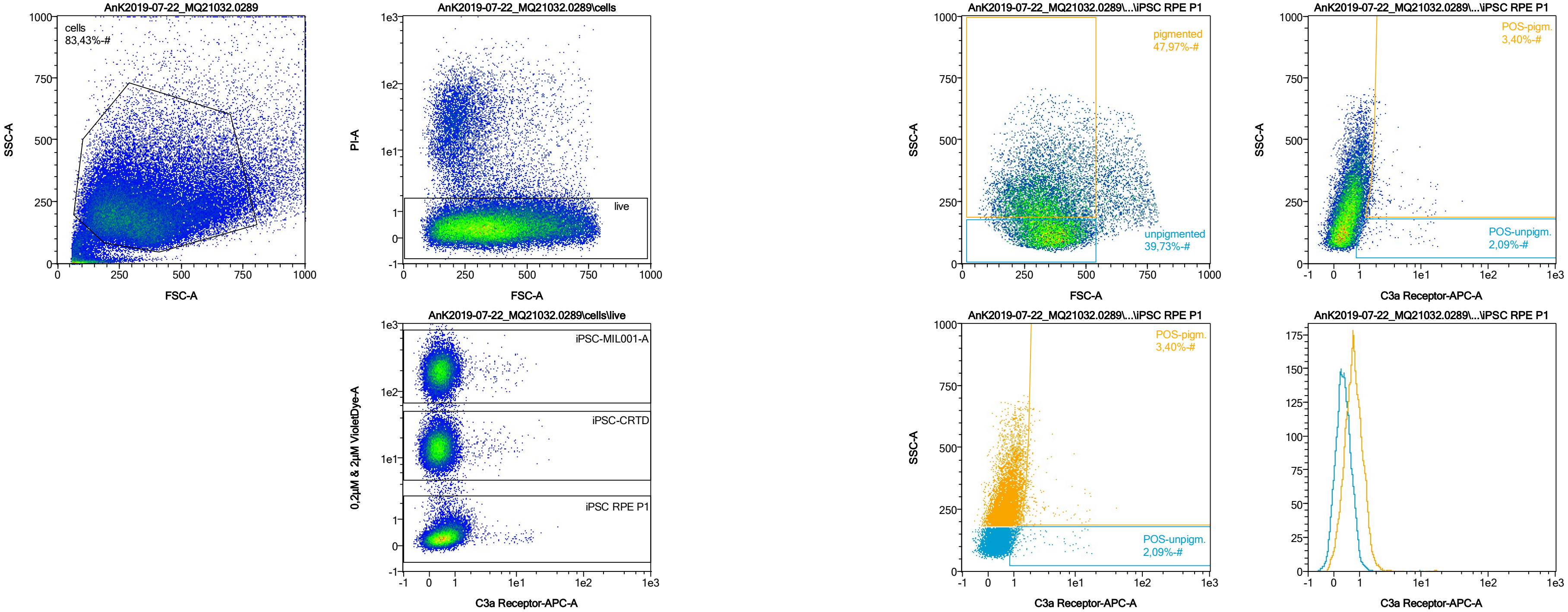

Name	%-#	C3a Receptor-APC-A Mean	C3a Receptor-APC-A Median
POS-unpigm.	2,09	3,01	1,29
POS-pigm.	3,40	3,07	1,89

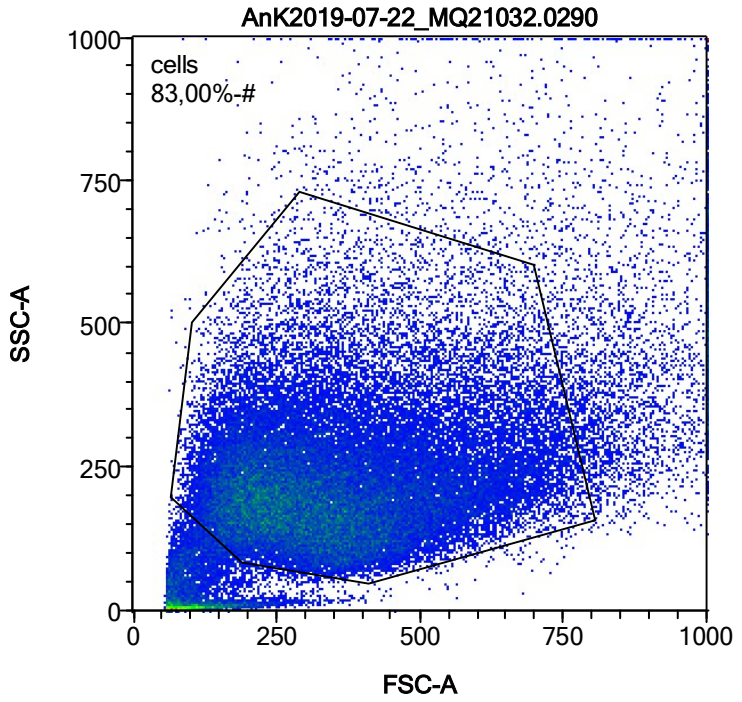

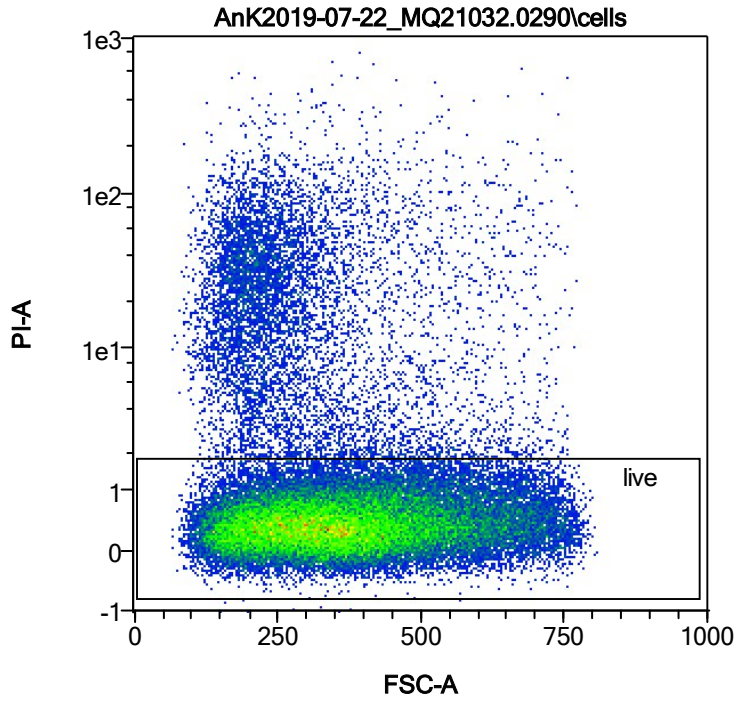

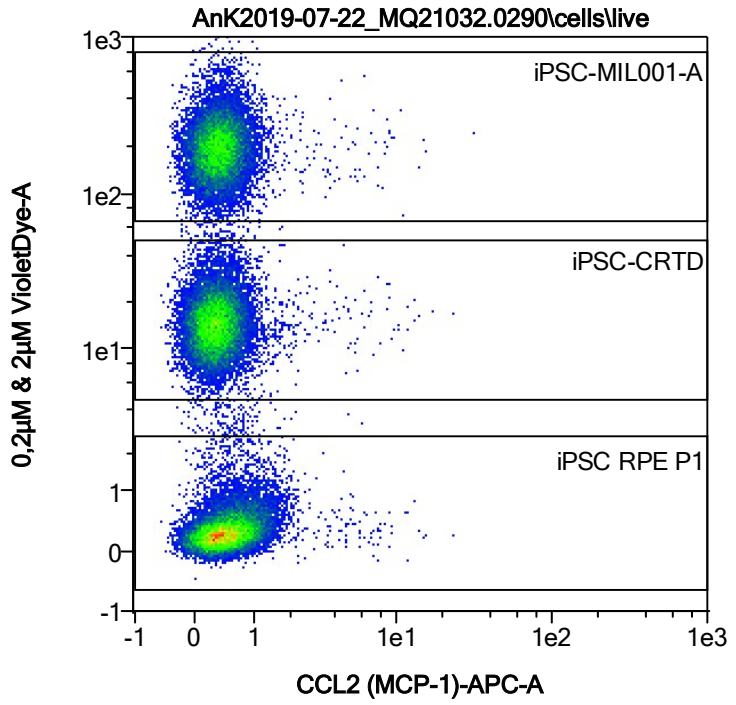

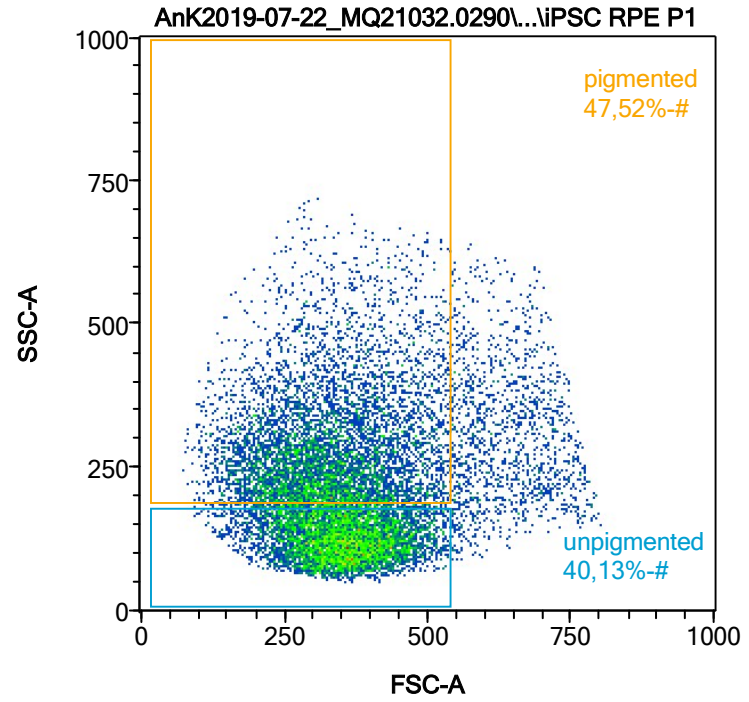

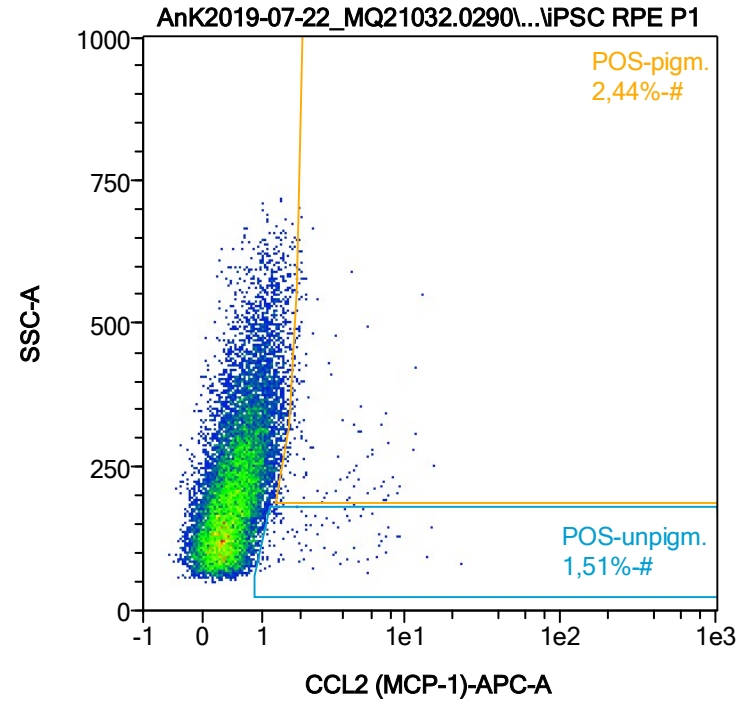

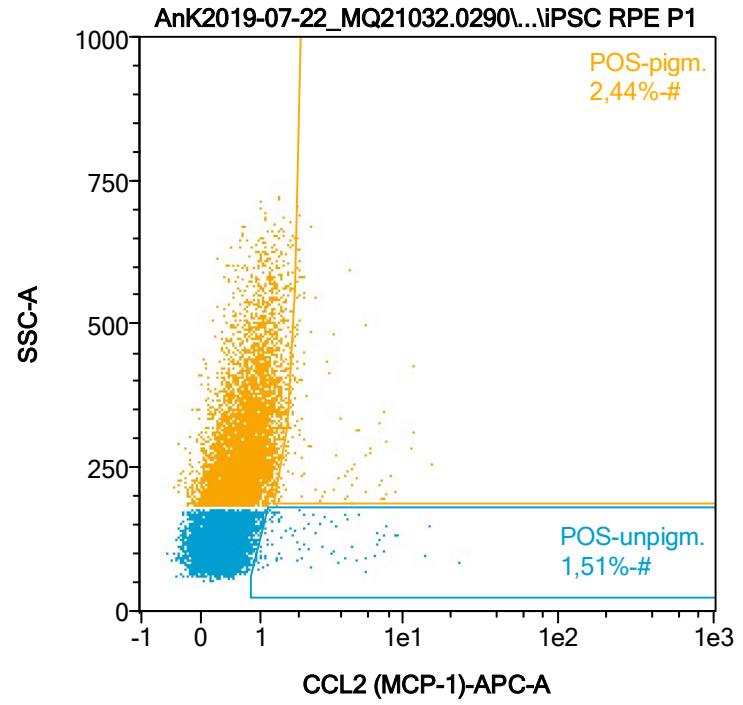

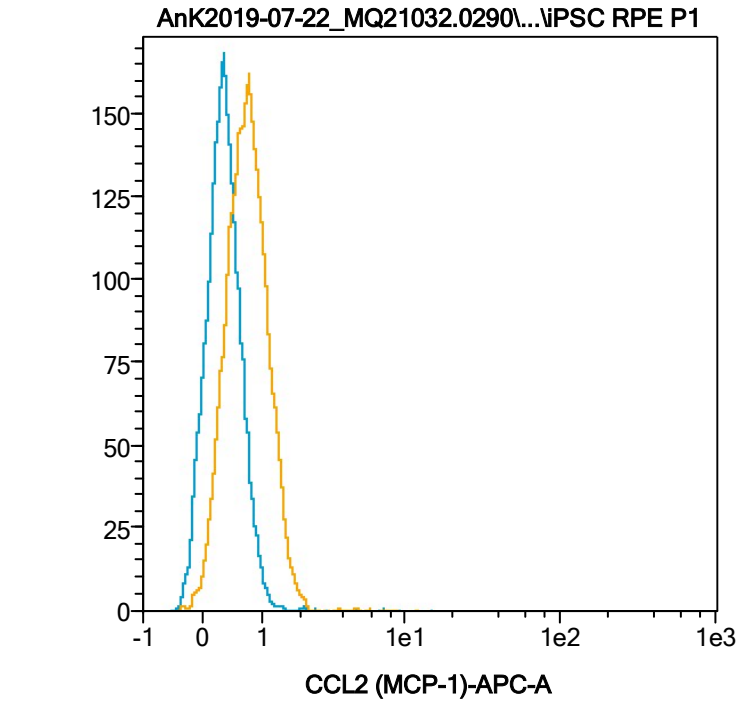

Name	%-#	CCL2 (MCP-1)-APC-A Mean	CCL2 (MCP-1)-APC-A Median
POS-unpigm.	1,51	3,17	1,81
POS-pigm.	2,44	2,87	1,95

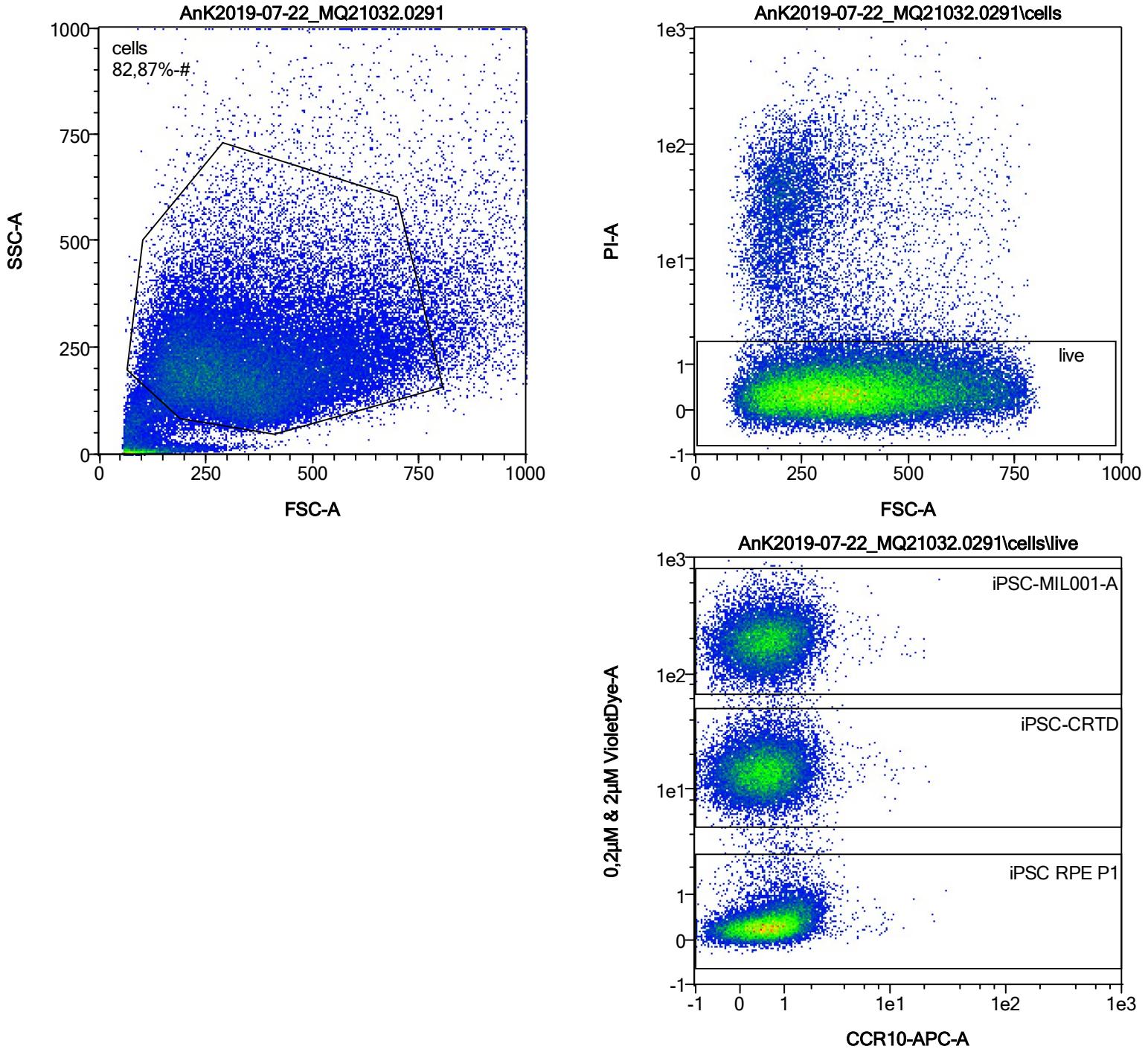

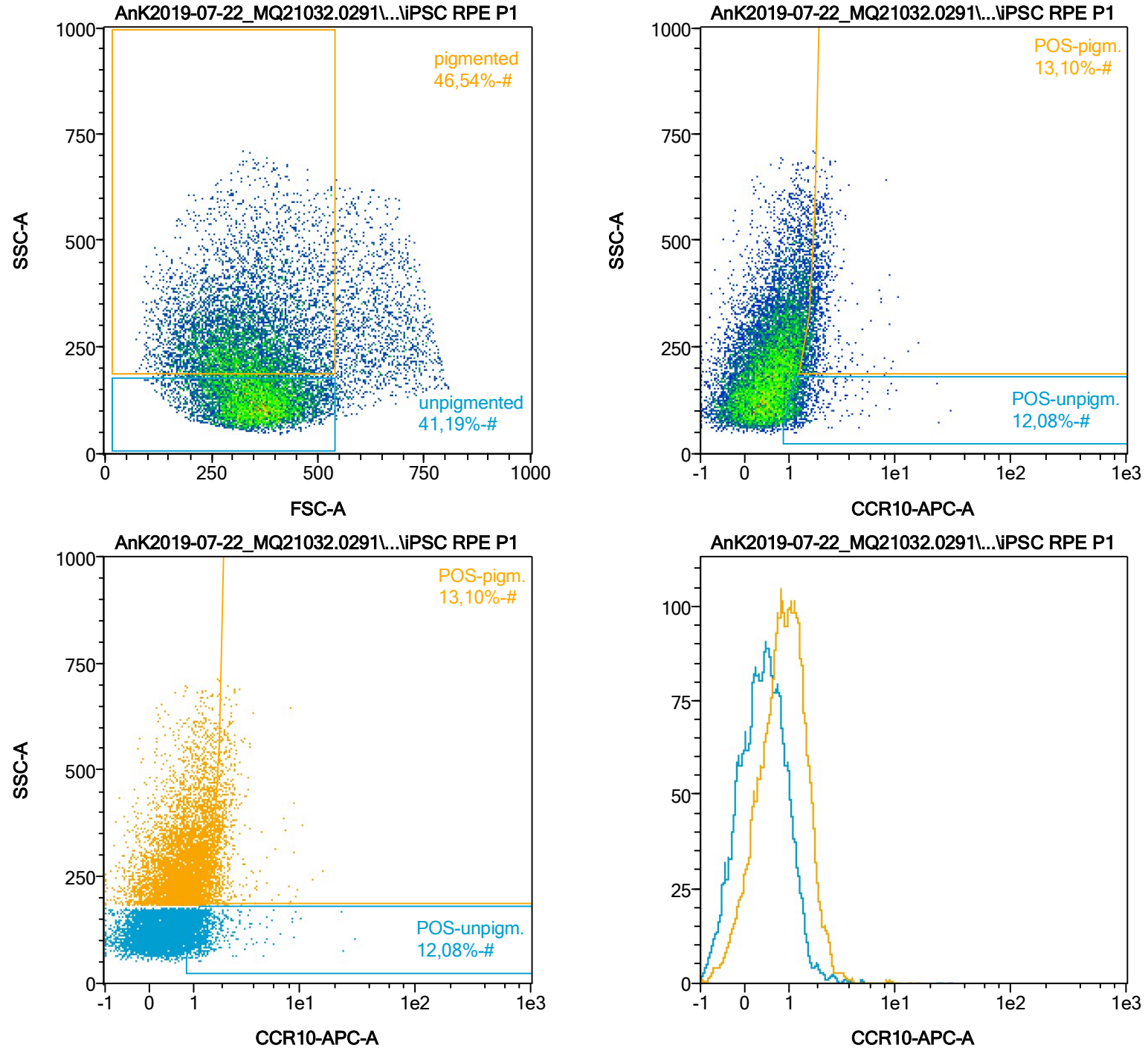

Name	%-#	CCR10-APC-A Mean	CCR10-APC-A Median
POS-unpigm.	12,08	1,59	1,25
POS-pigm.	13,10	2,12	1,89

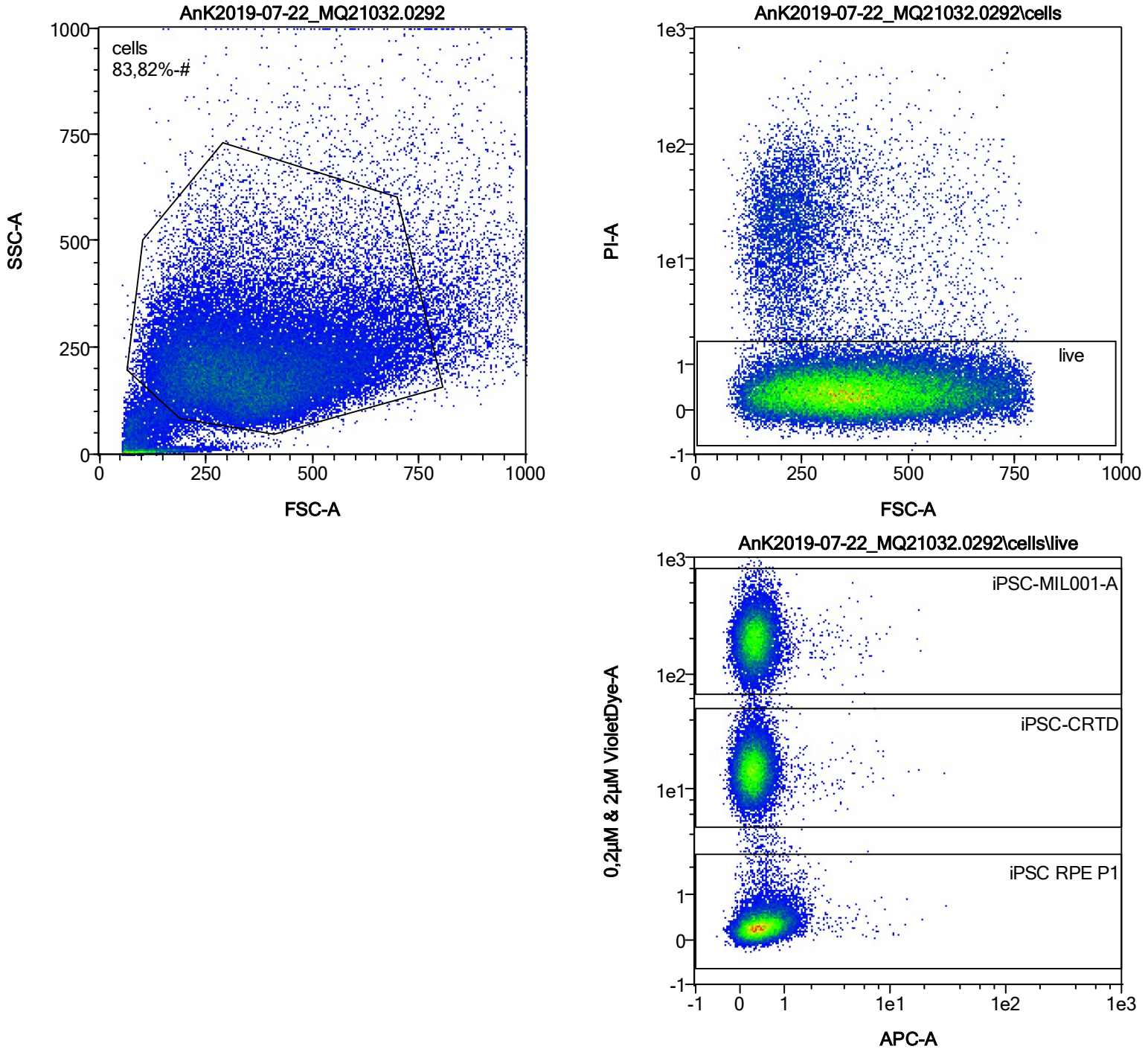

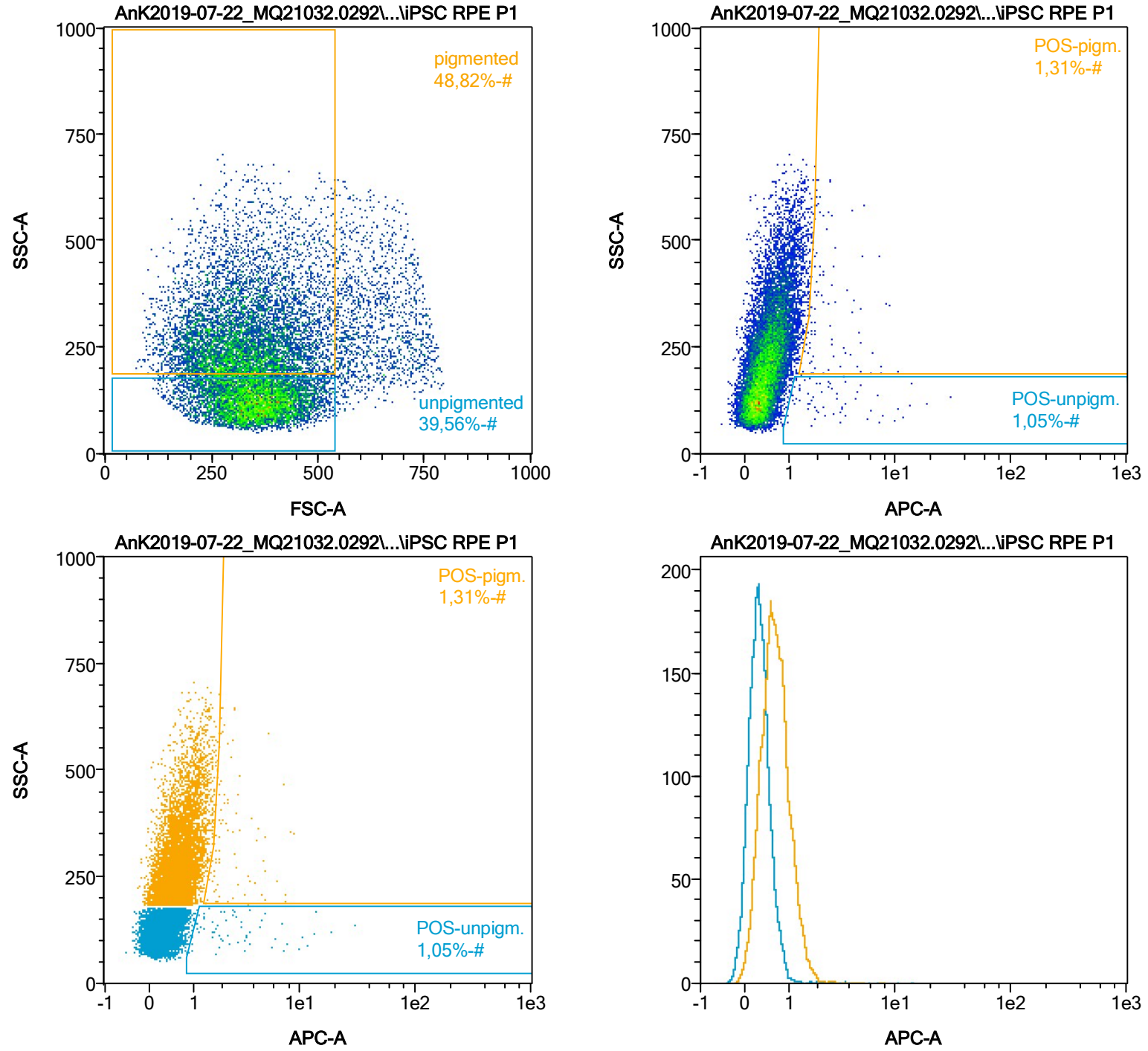

Name	%-#	APC-A Mean	APC-A Median
POS-unpigm.	1,05	4,11	2,28
POS-pigm.	1,31	2,72	1,99

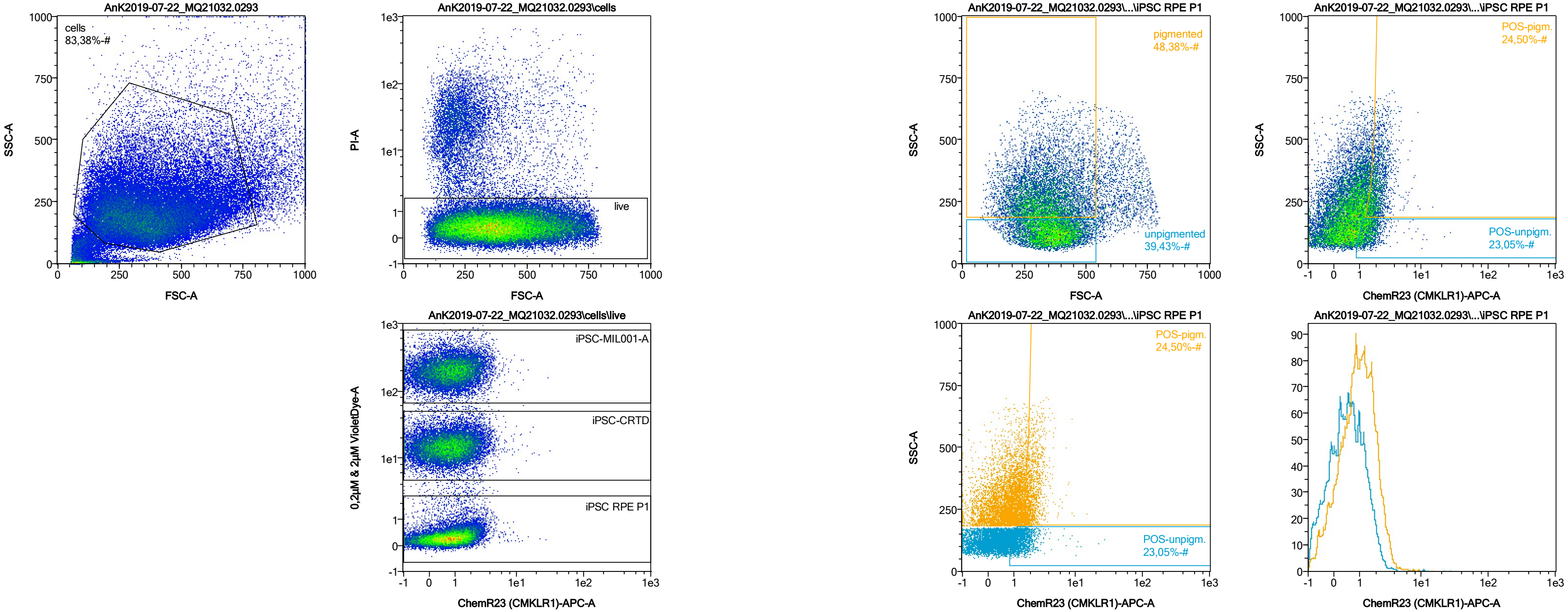

Name	%-#	ChemR23 (CMKLR1)-APC-A Mean	ChemR23 (CMKLR1)-APC-A Median
POS-unpigm.	23,05	1,61	1,41
POS-pigm.	24,50	2,19	2,00

Date:2024-06-14
Operator: Sebastian Knoebel
Version:2.13.0+54.g95abf0f4

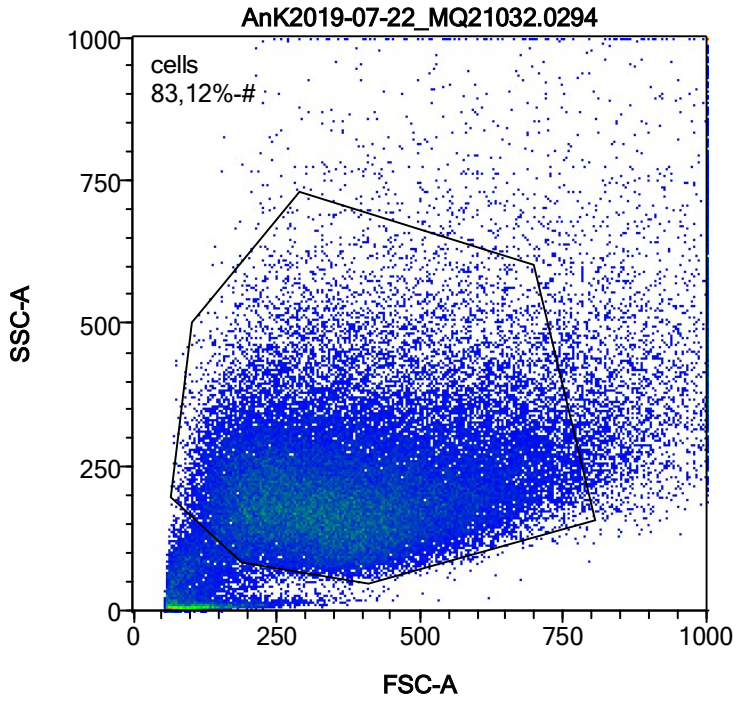

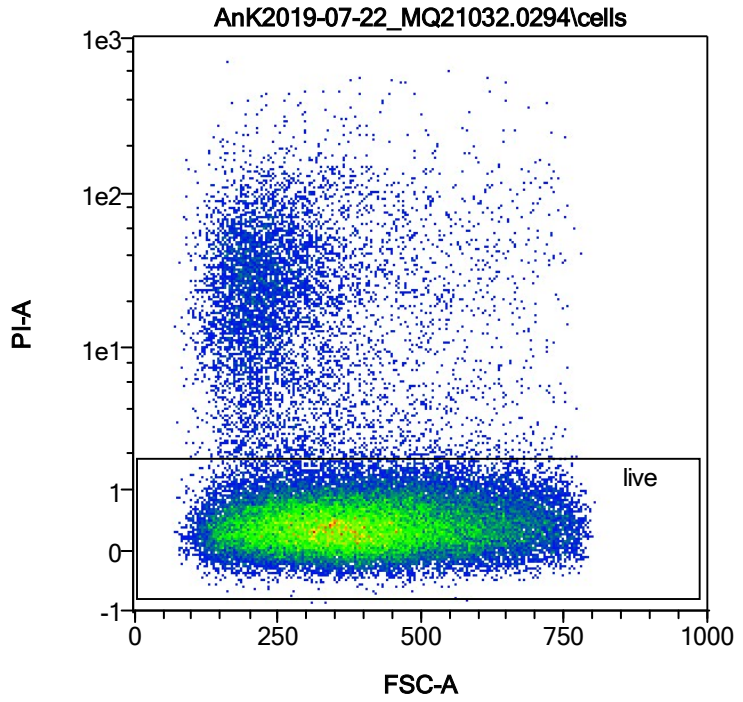

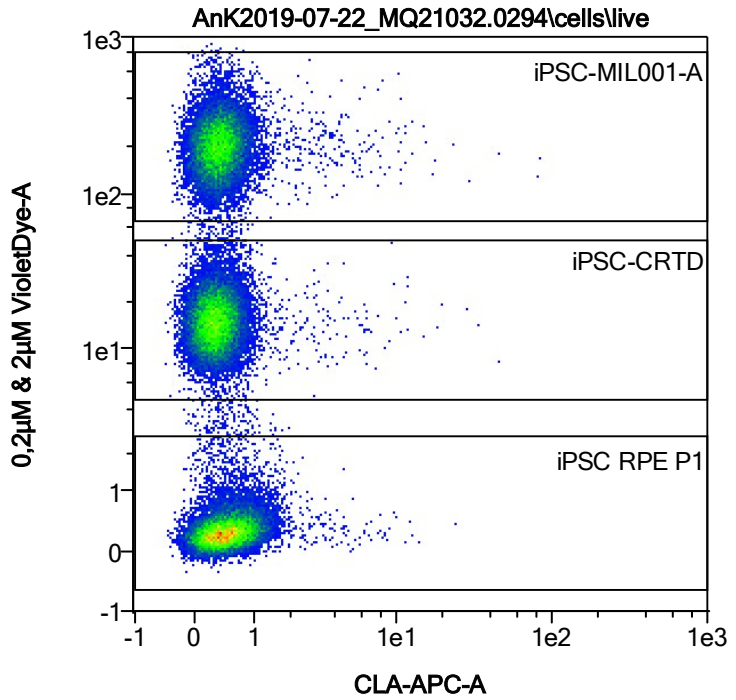

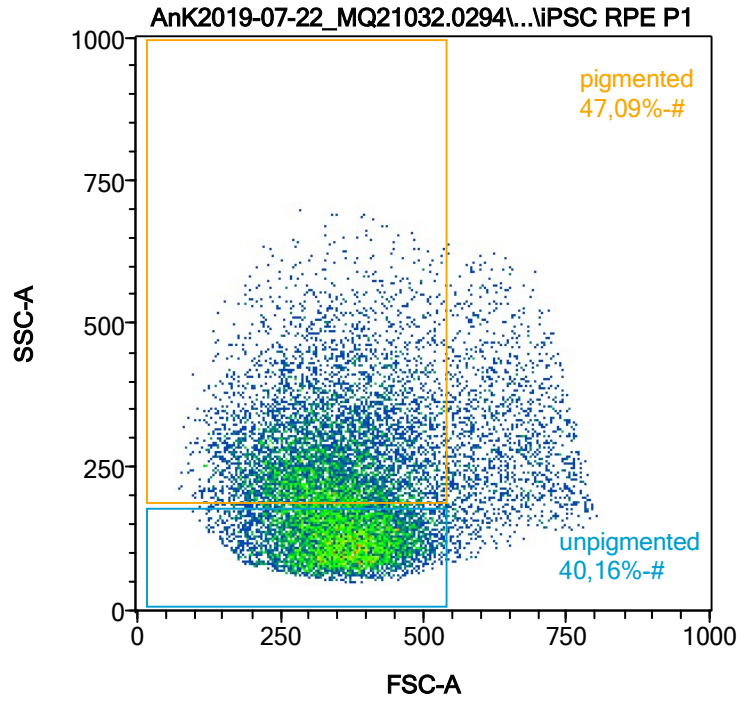

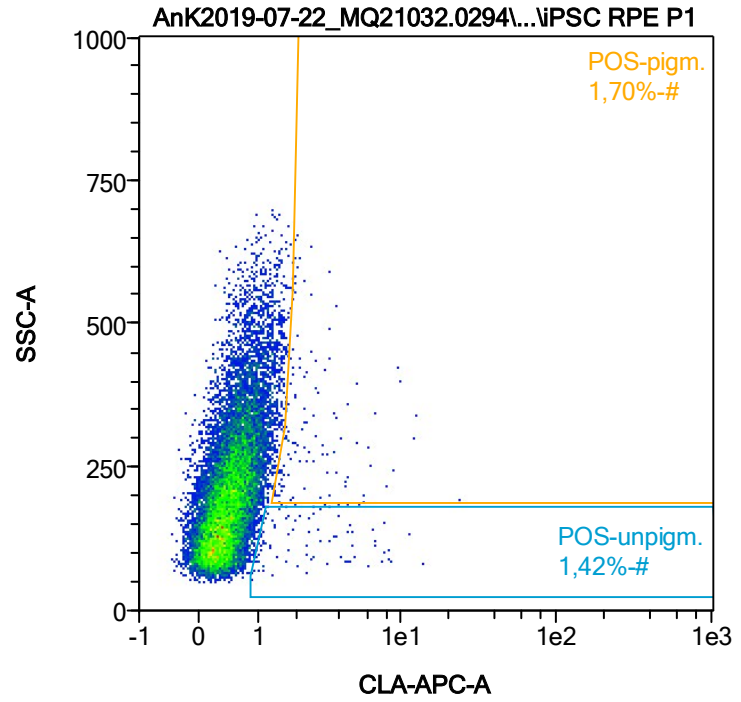

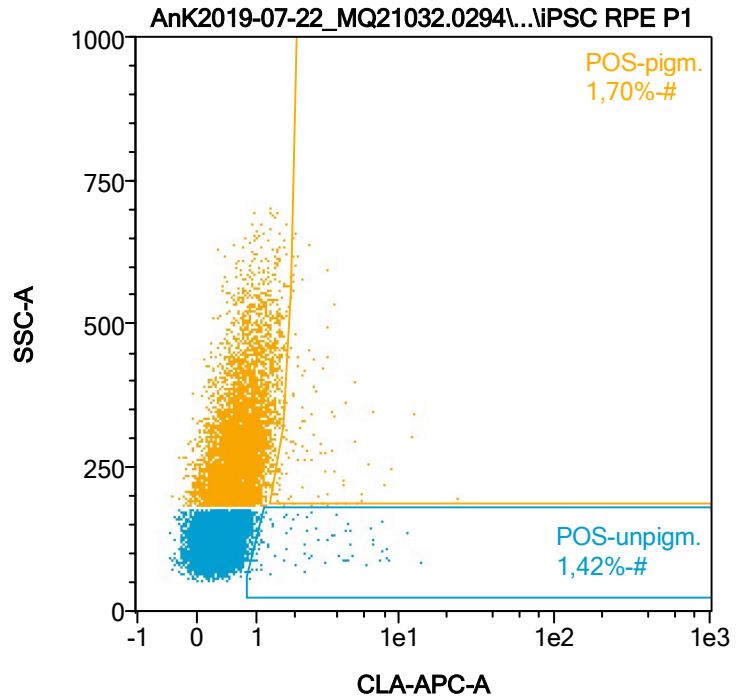

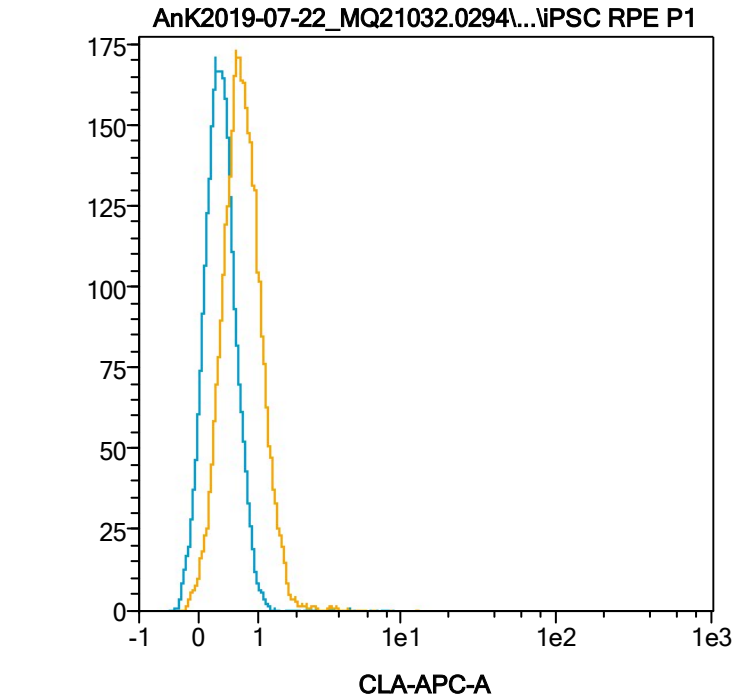

Name	%-#	CLA-APC-A Mean	CLA-APC-A Median
POS-unpigm.	1,42	2,98	1,91
POS-pigm.	1,70	3,01	2,13

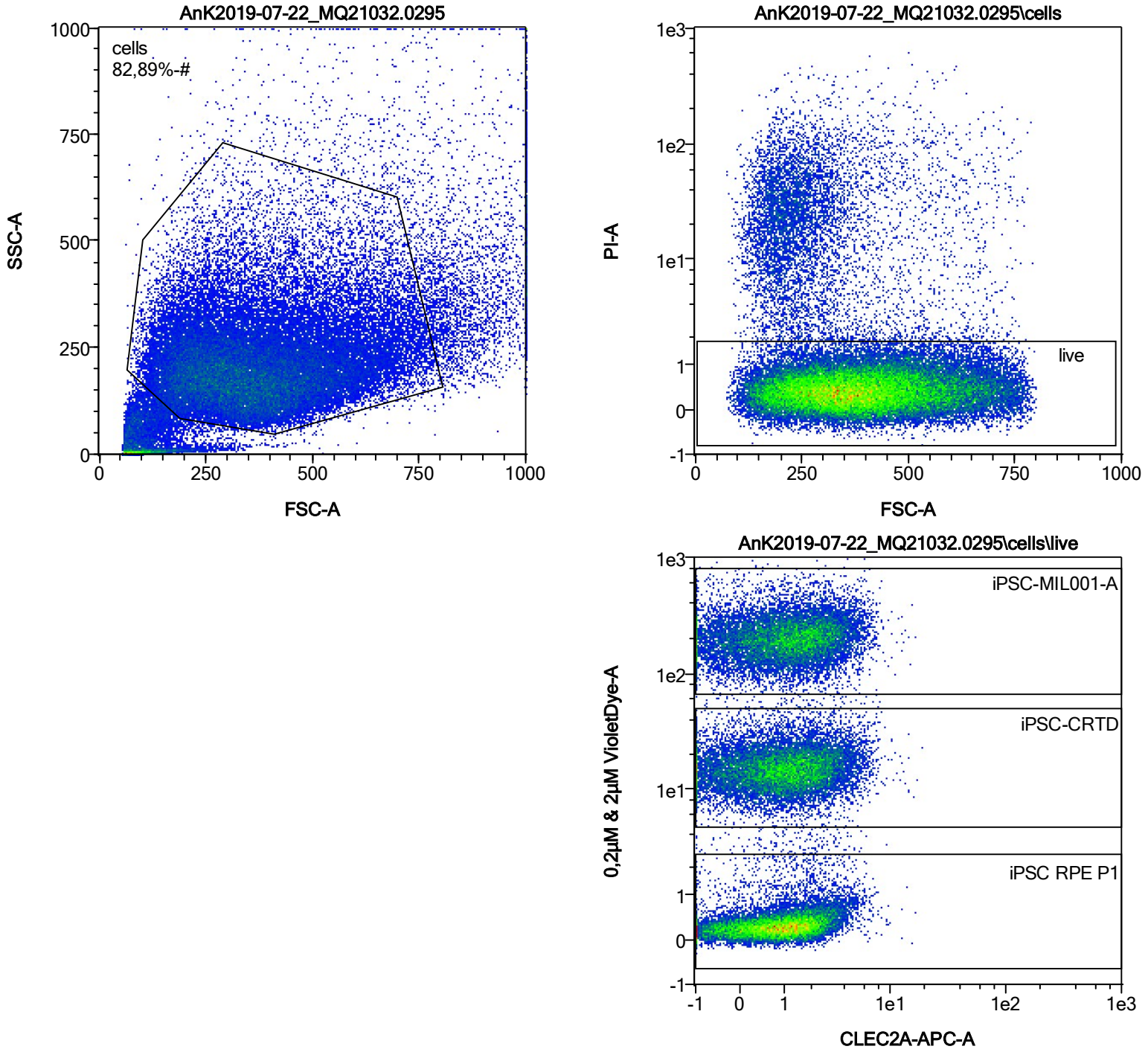

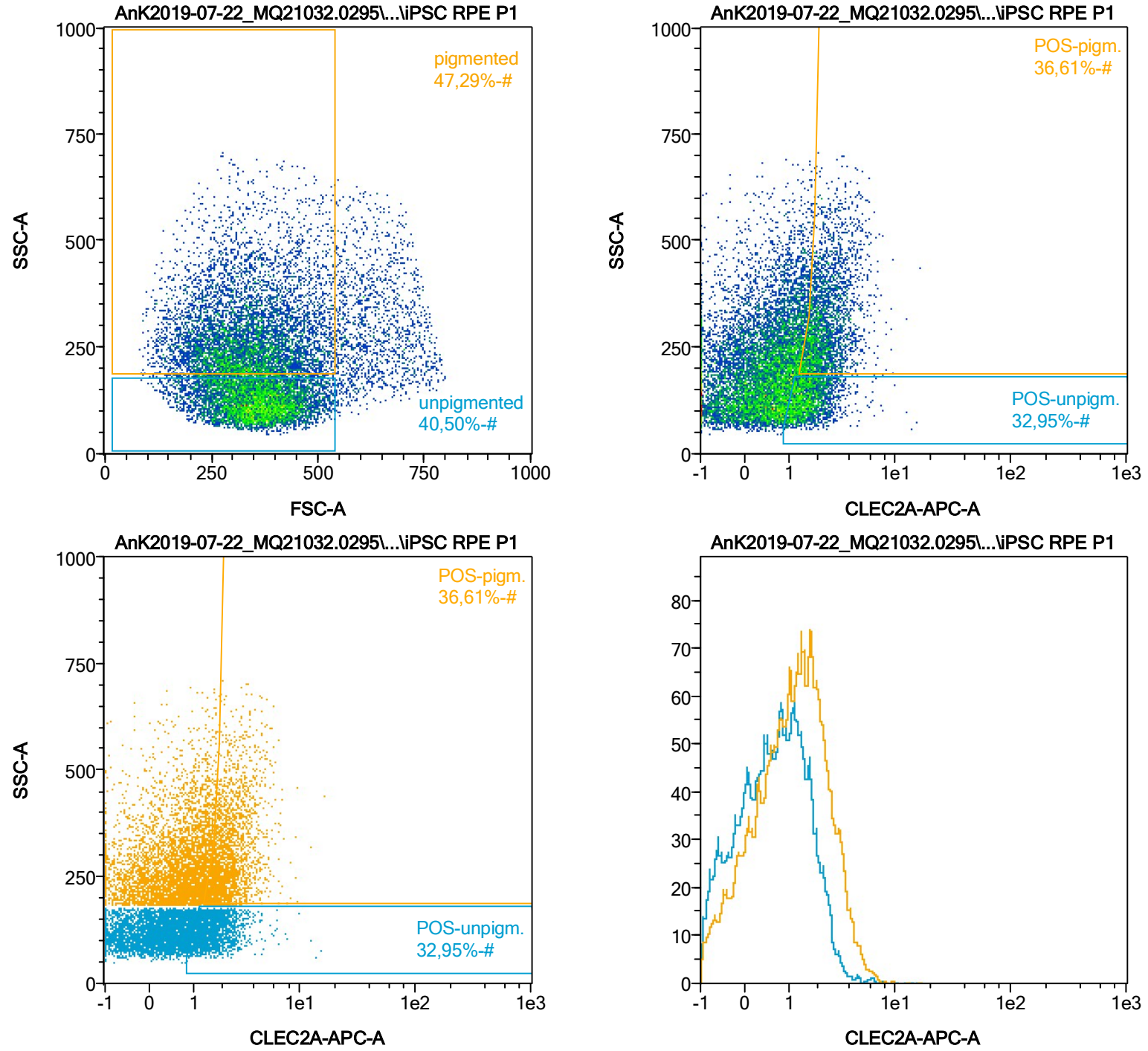

Name	%-#	CLEC2A-APC-A Mean	CLEC2A-APC-A Median
POS-unpigm.	32,95	1,74	1,55
POS-pigm.	36,61	2,50	2,22

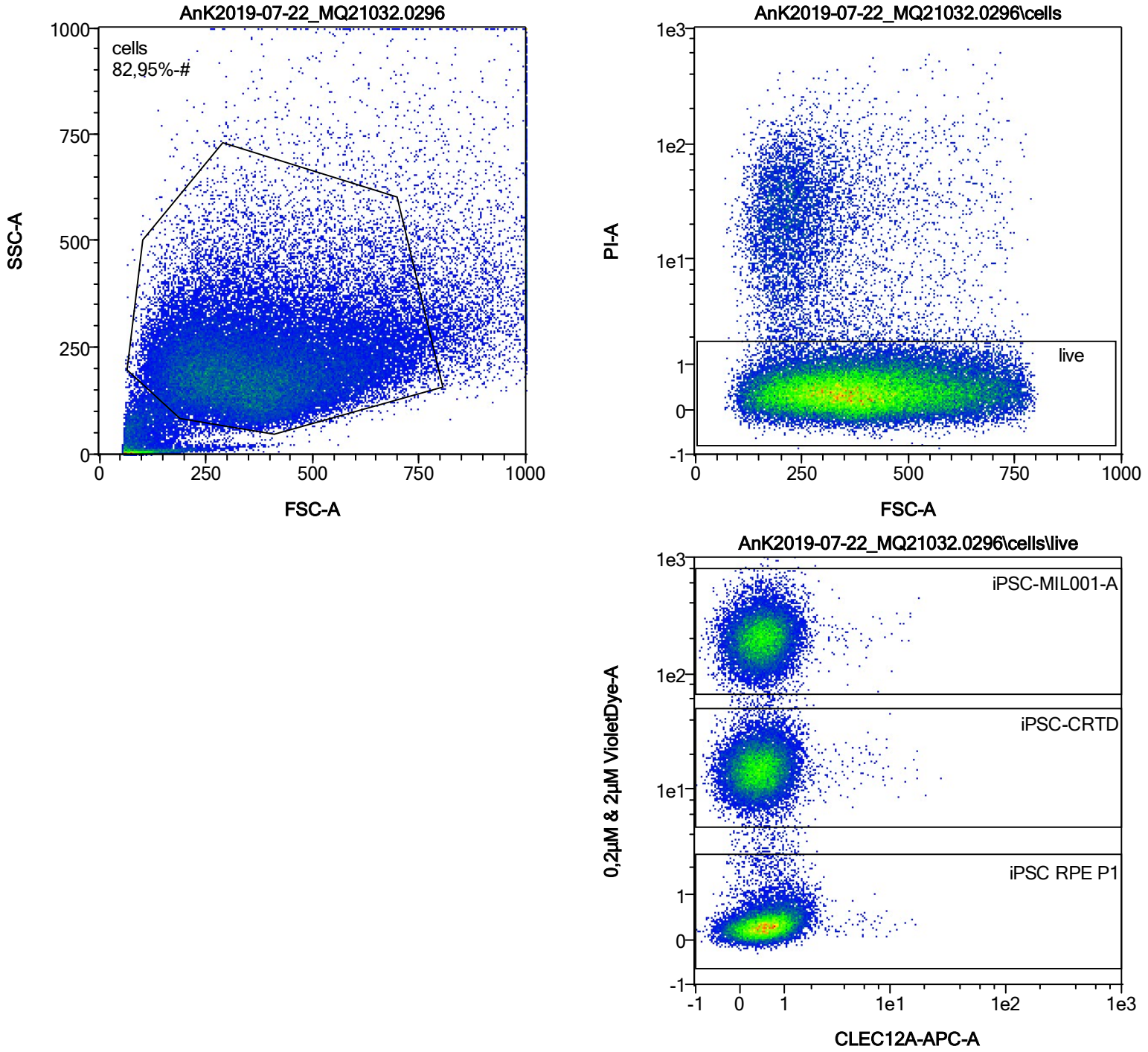

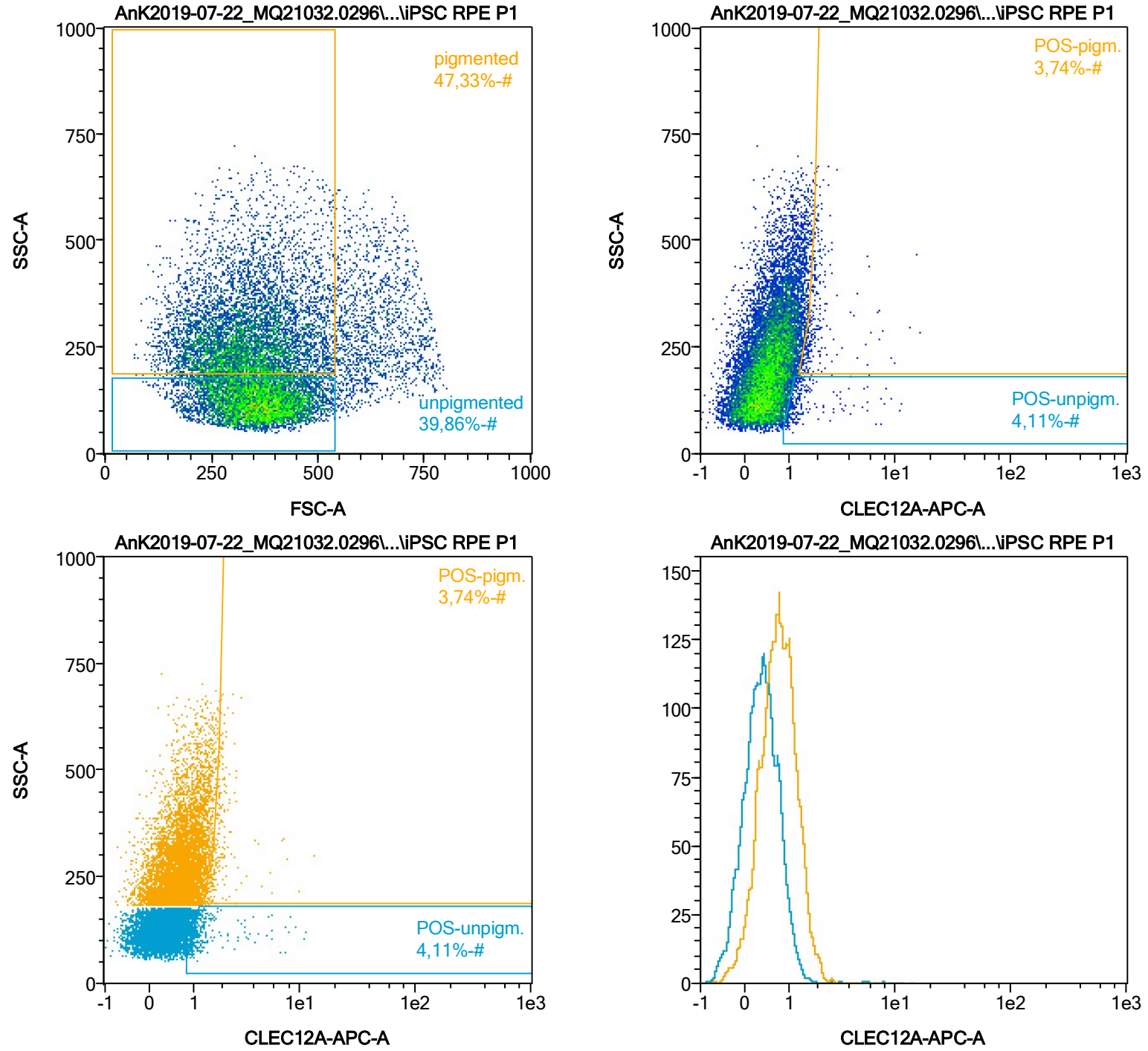

Name	%-#	CLEC12A-APC-A Mean	CLEC12A-APC-A Median
POS-unpigm.	4,11	1,71	1,19
POS-pigm.	3,74	2,06	1,83

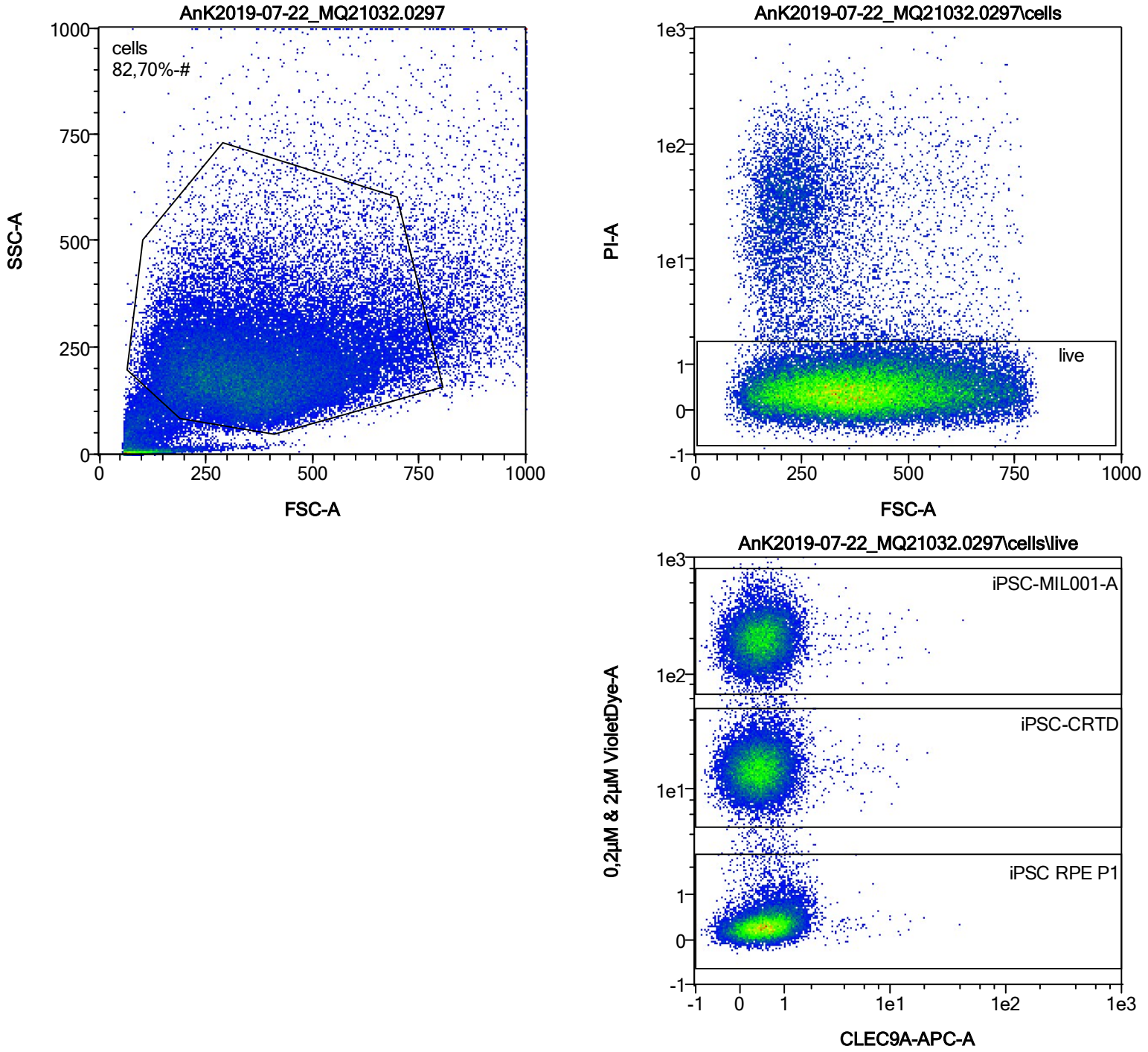

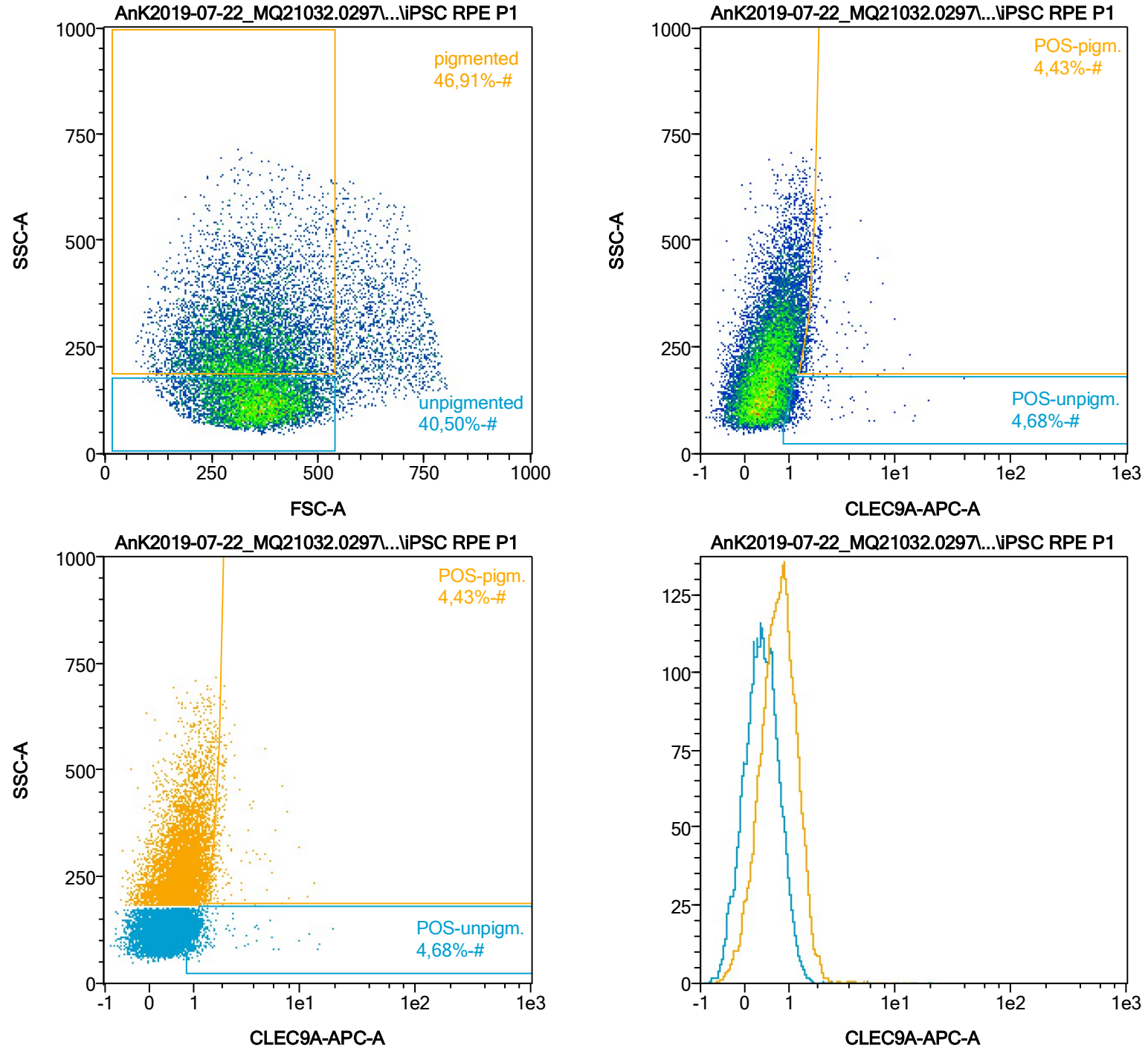

Name	%-#	CLEC9A-APC-A Mean	CLEC9A-APC-A Median
POS-unpigm.	4,68	1,60	1,16
POS-pigm.	4,43	2,14	1,79

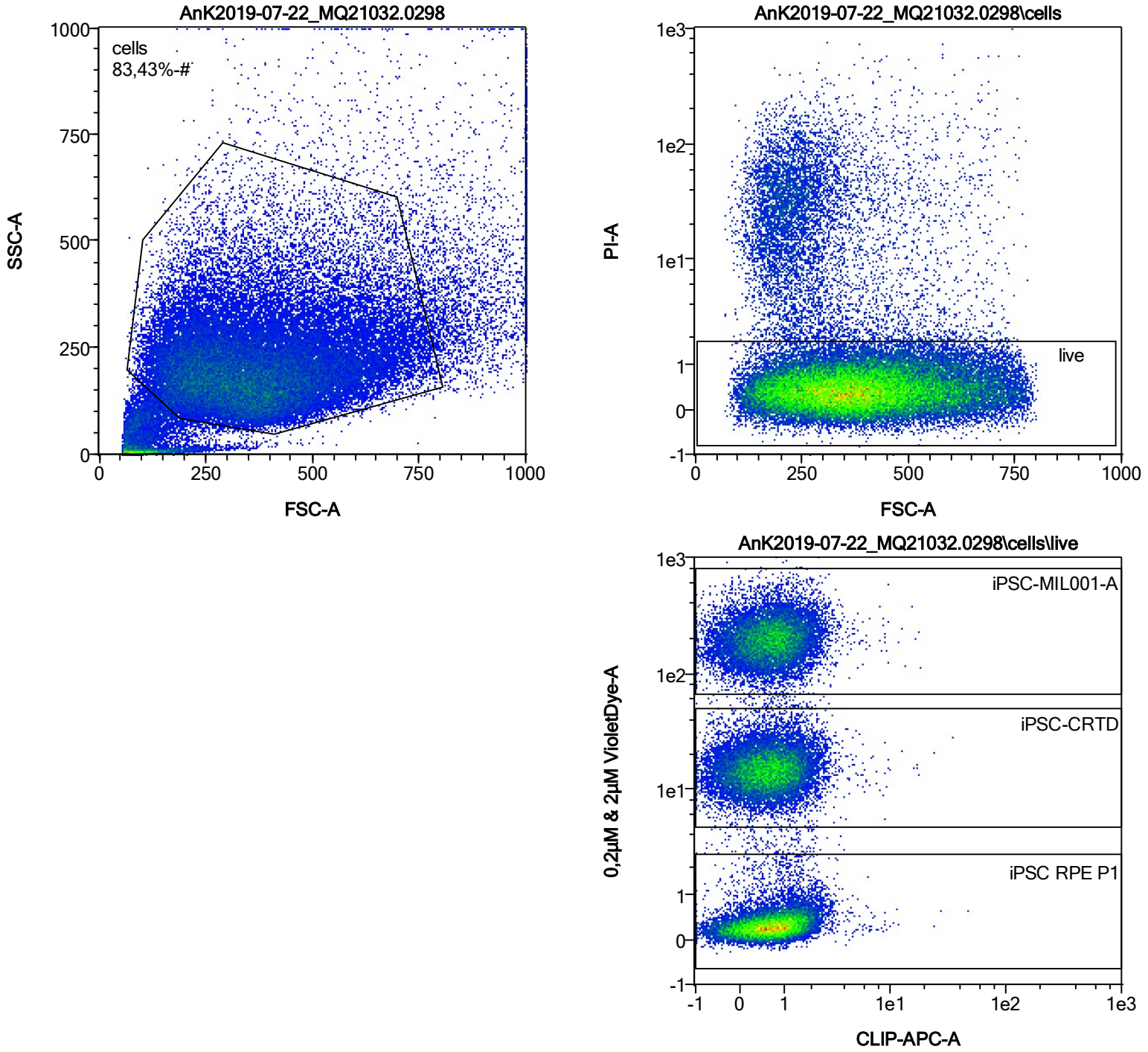

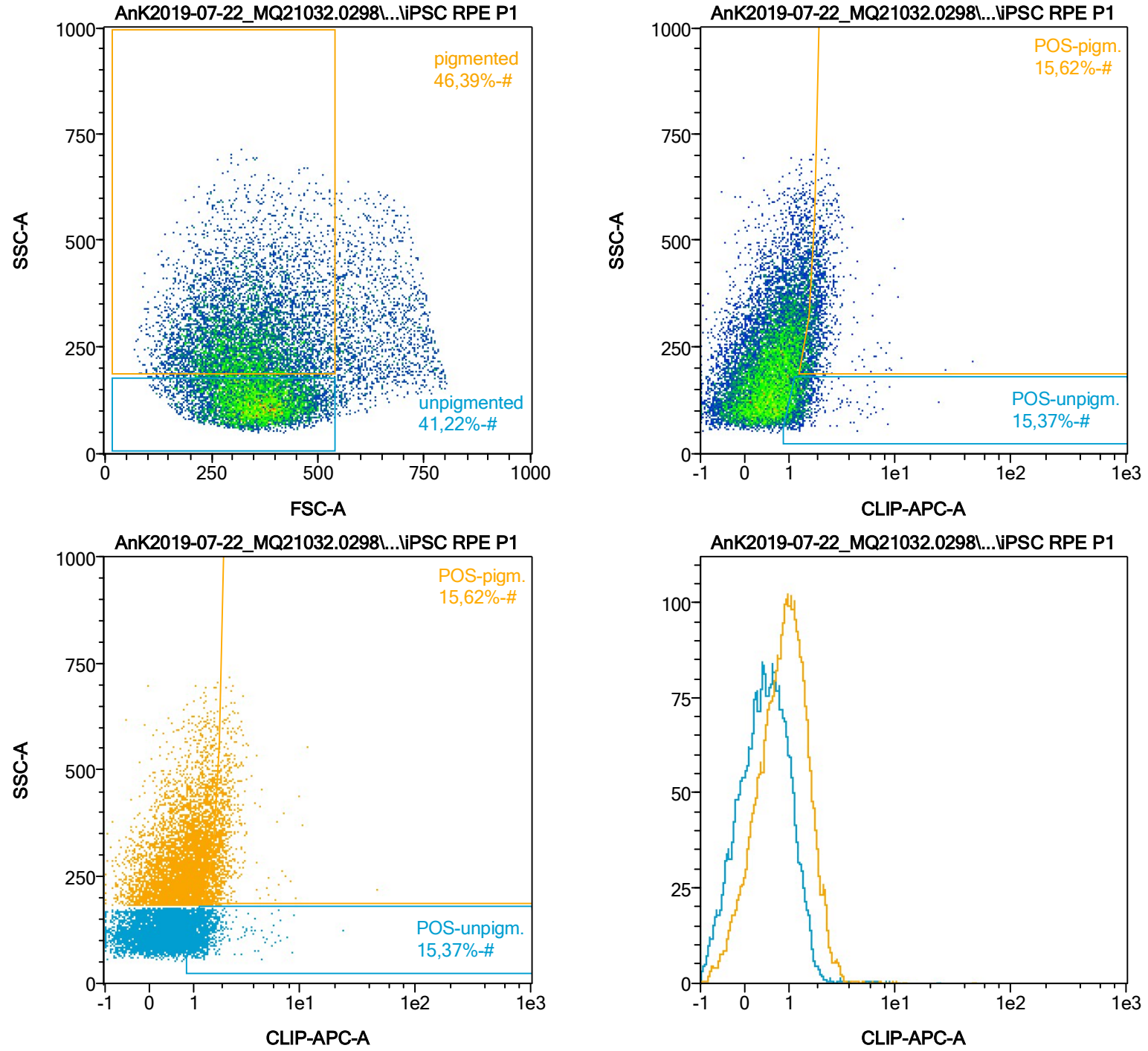

Name	%-#	CLIP-APC-A Mean	CLIP-APC-A Median
POS-unpigm.	15,37	1,47	1,26
POS-pigm.	15,62	2,10	1,91

Date:2024-06-14

Operator: Sebastian Knoebel

Version:2.13.0+54.g95abf0f4

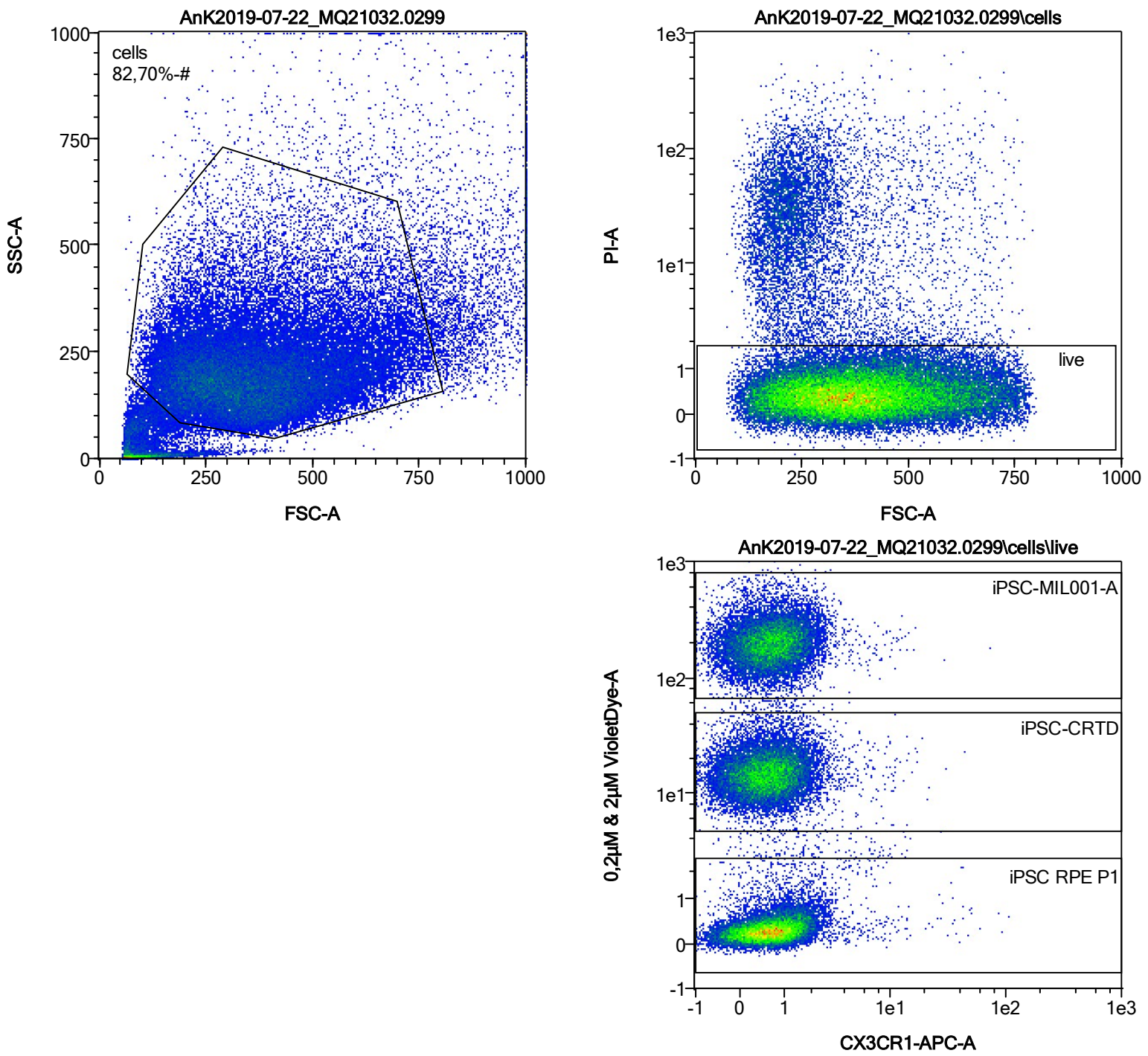

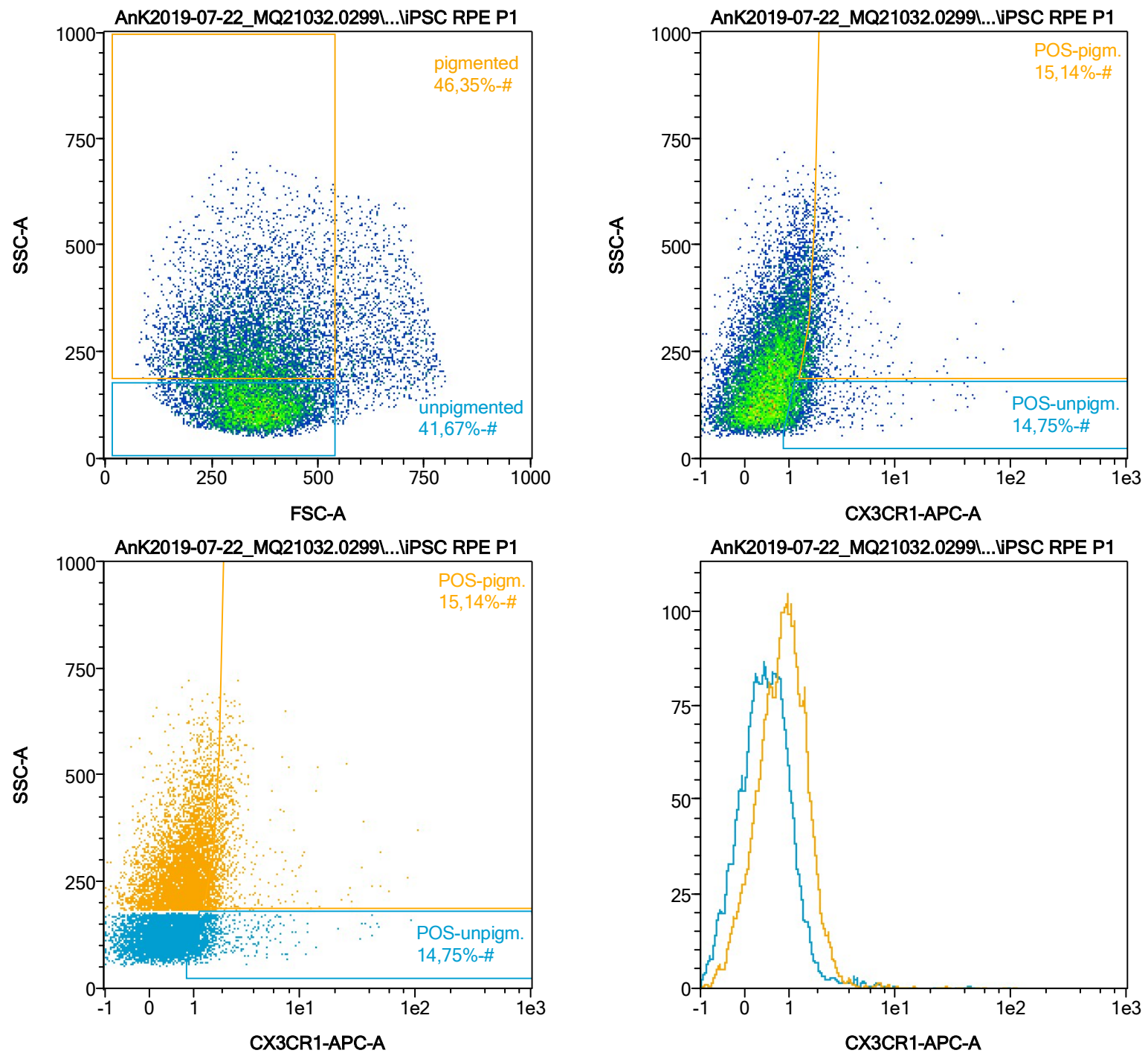

Name	%-#	CX3CR1-APC-A Mean	CX3CR1-APC-A Median
POS-unpigm.	14,75	2,22	1,32
POS-pigm.	15,14	2,81	1,90

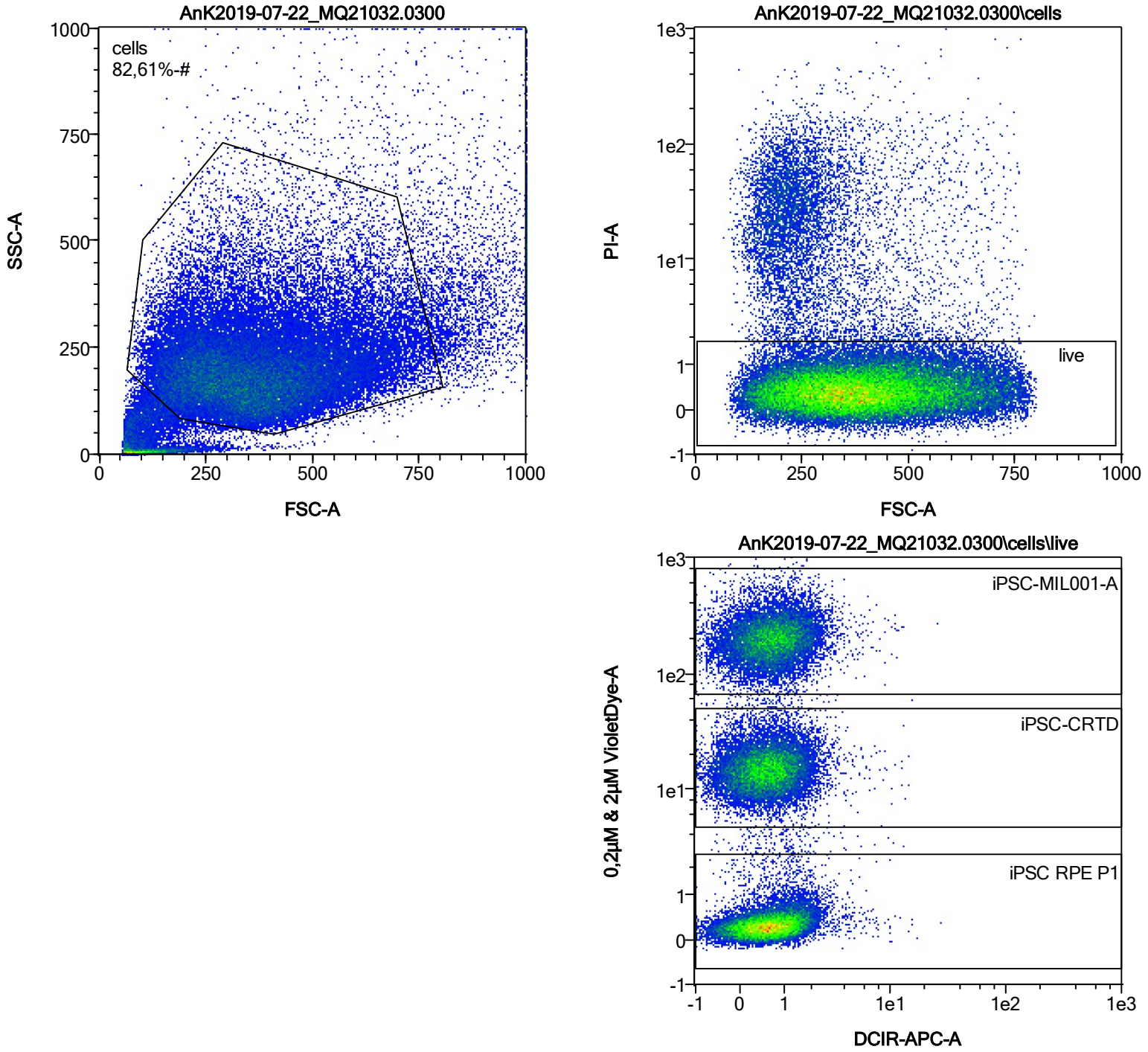

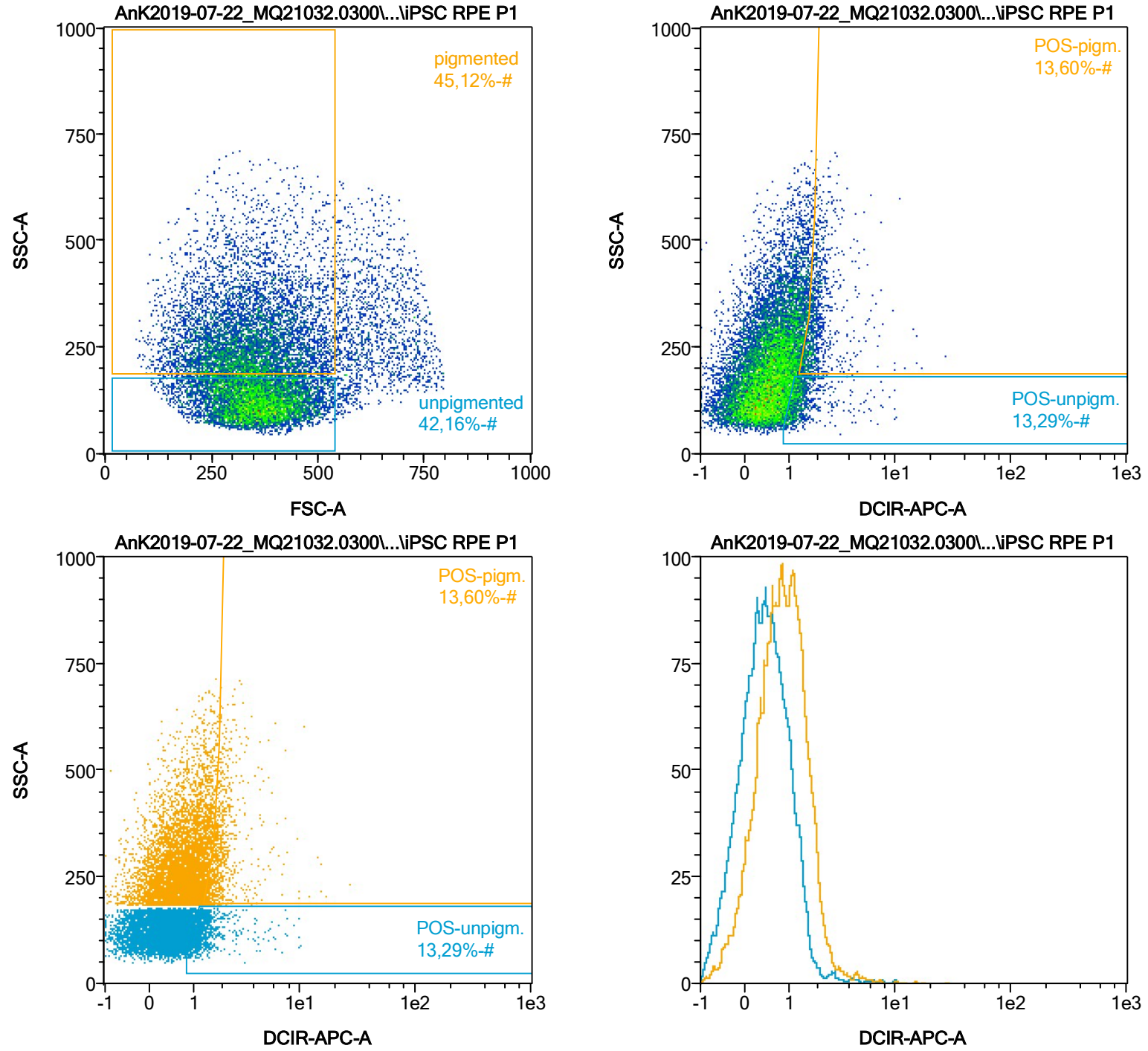

Name	%-#	DCIR-APC-A Mean	DCIR-APC-A Median
POS-unpigm.	13,29	1,54	1,28
POS-pigm.	13,60	2,23	1,87

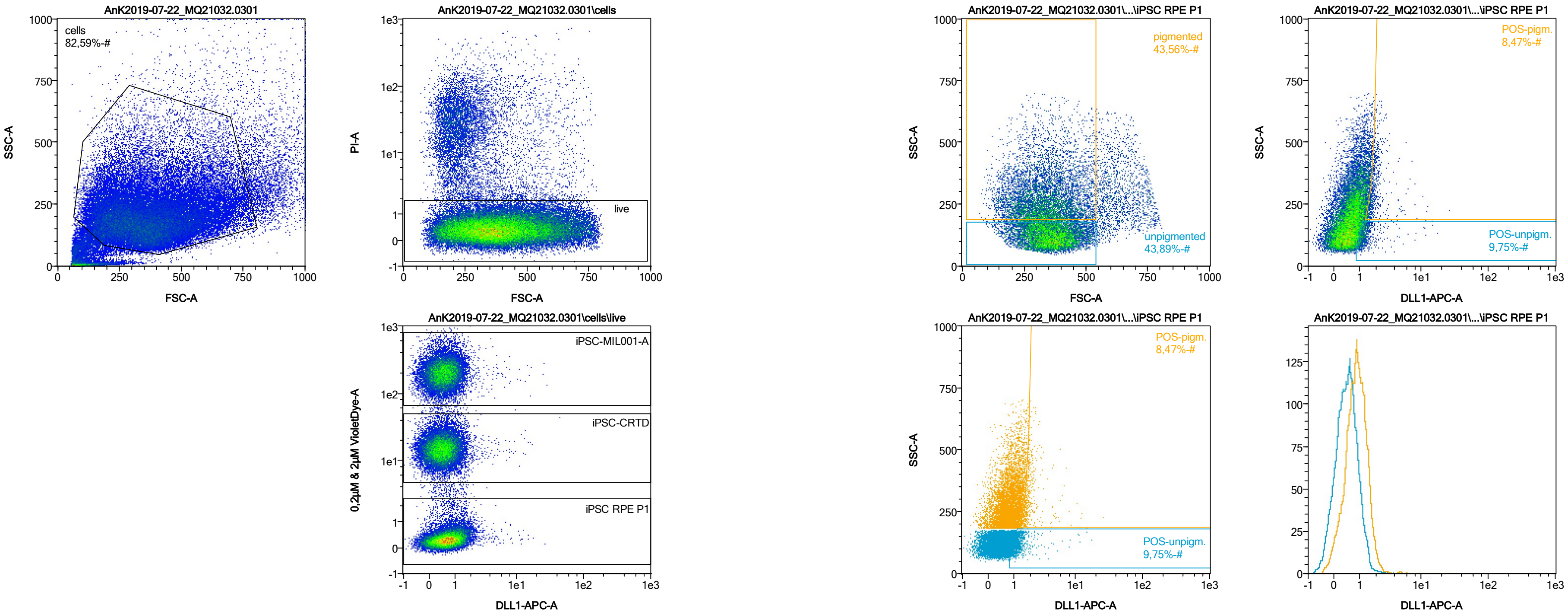

Name	%-#	DLL1-APC-A Mean	DLL1-APC-A Median
POS-unpigm.	9,75	1,45	1,22
POS-pigm.	8,47	2,11	1,77

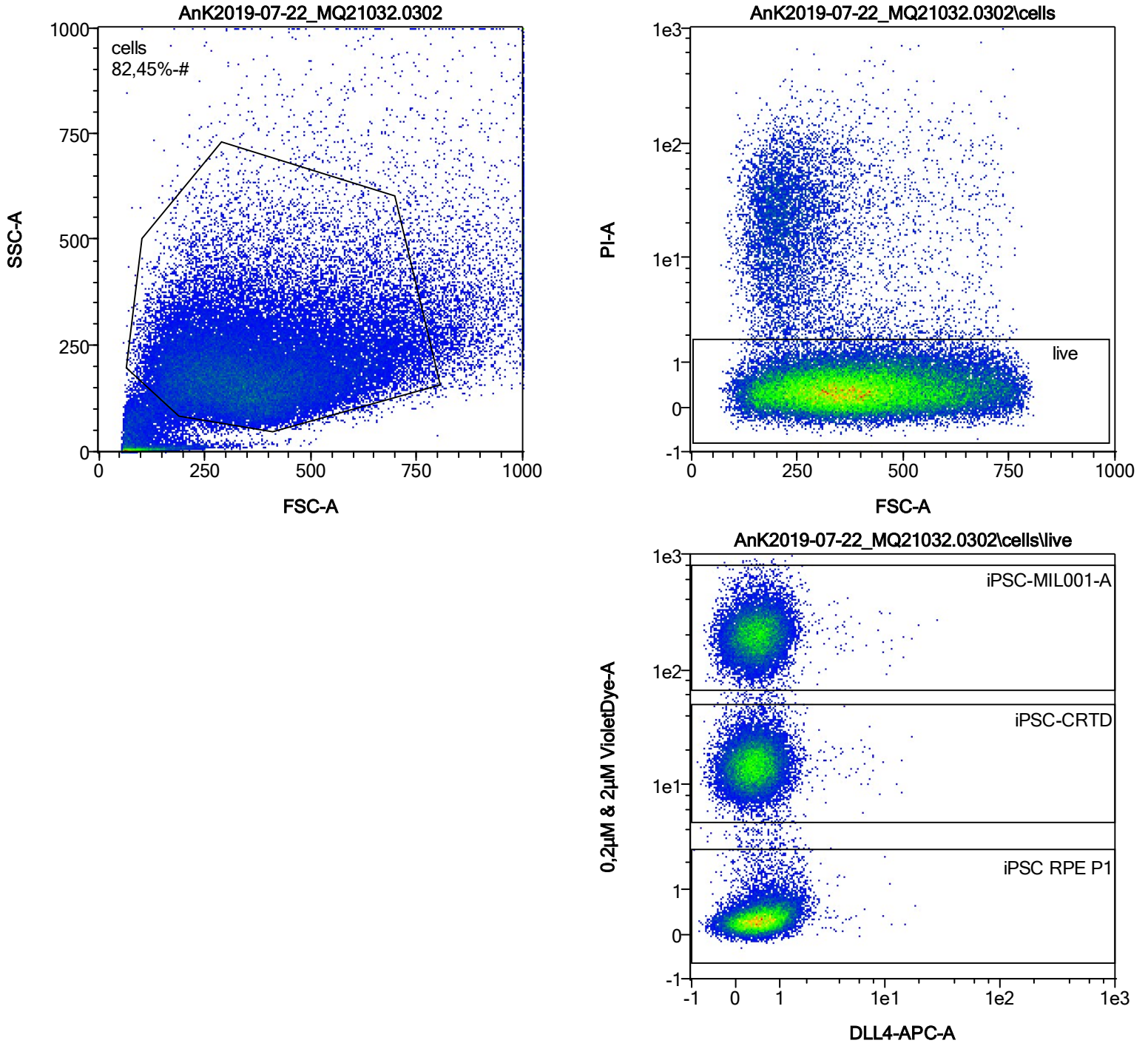

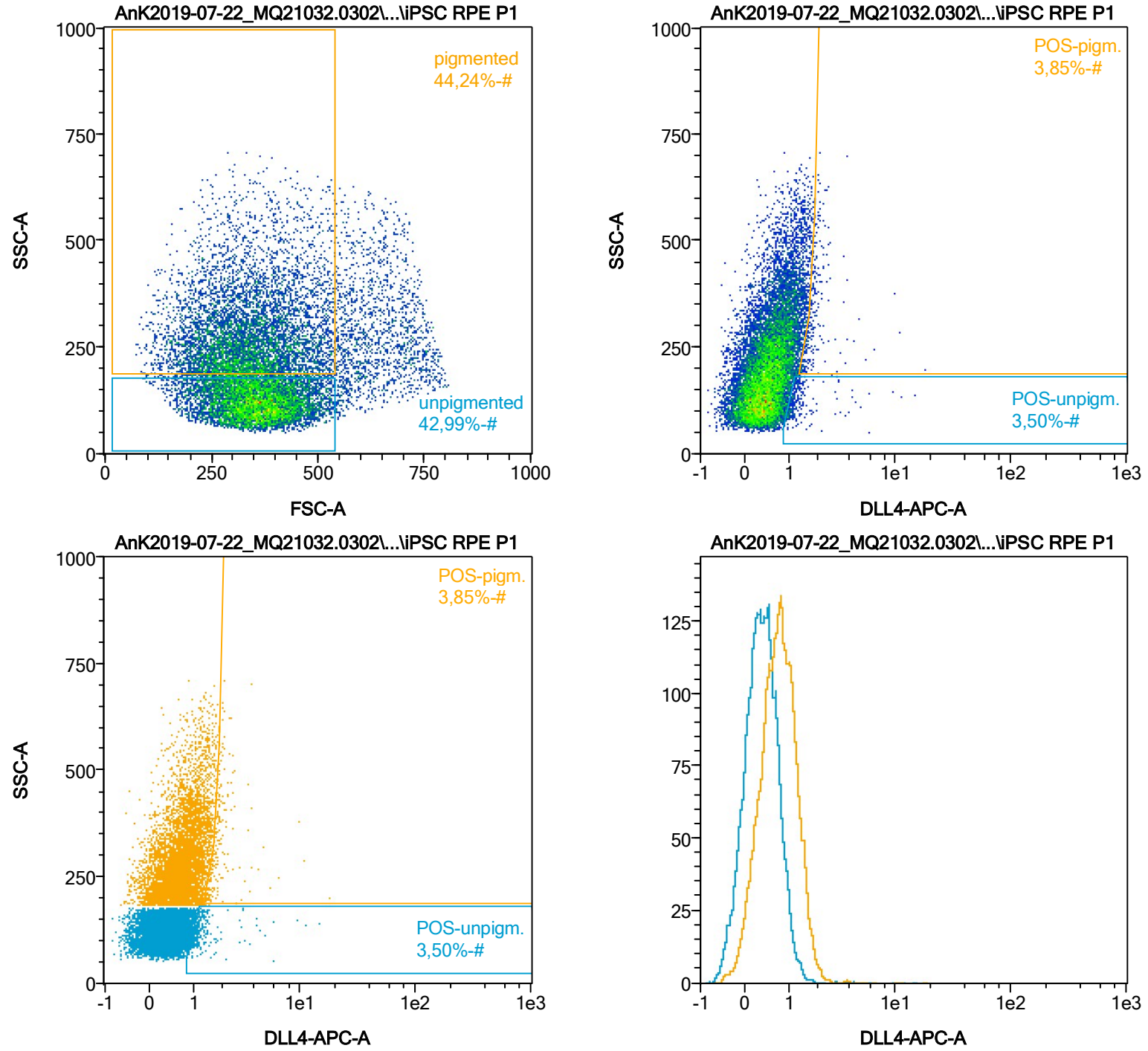

Name	%-#	DLL4-APC-A Mean	DLL4-APC-A Median
POS-unpigm.	3,50	1,51	1,16
POS-pigm.	3,85	2,03	1,78

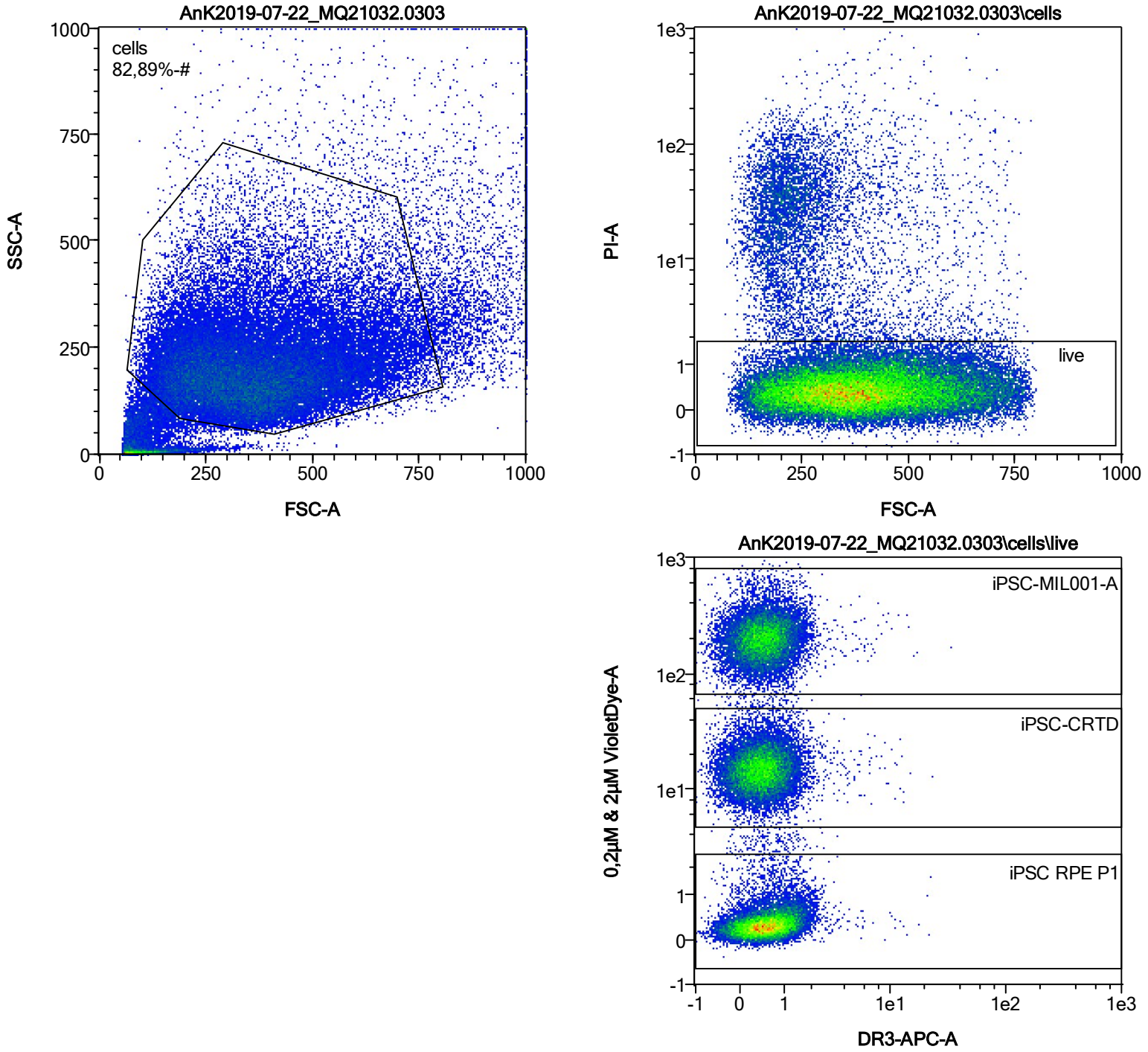

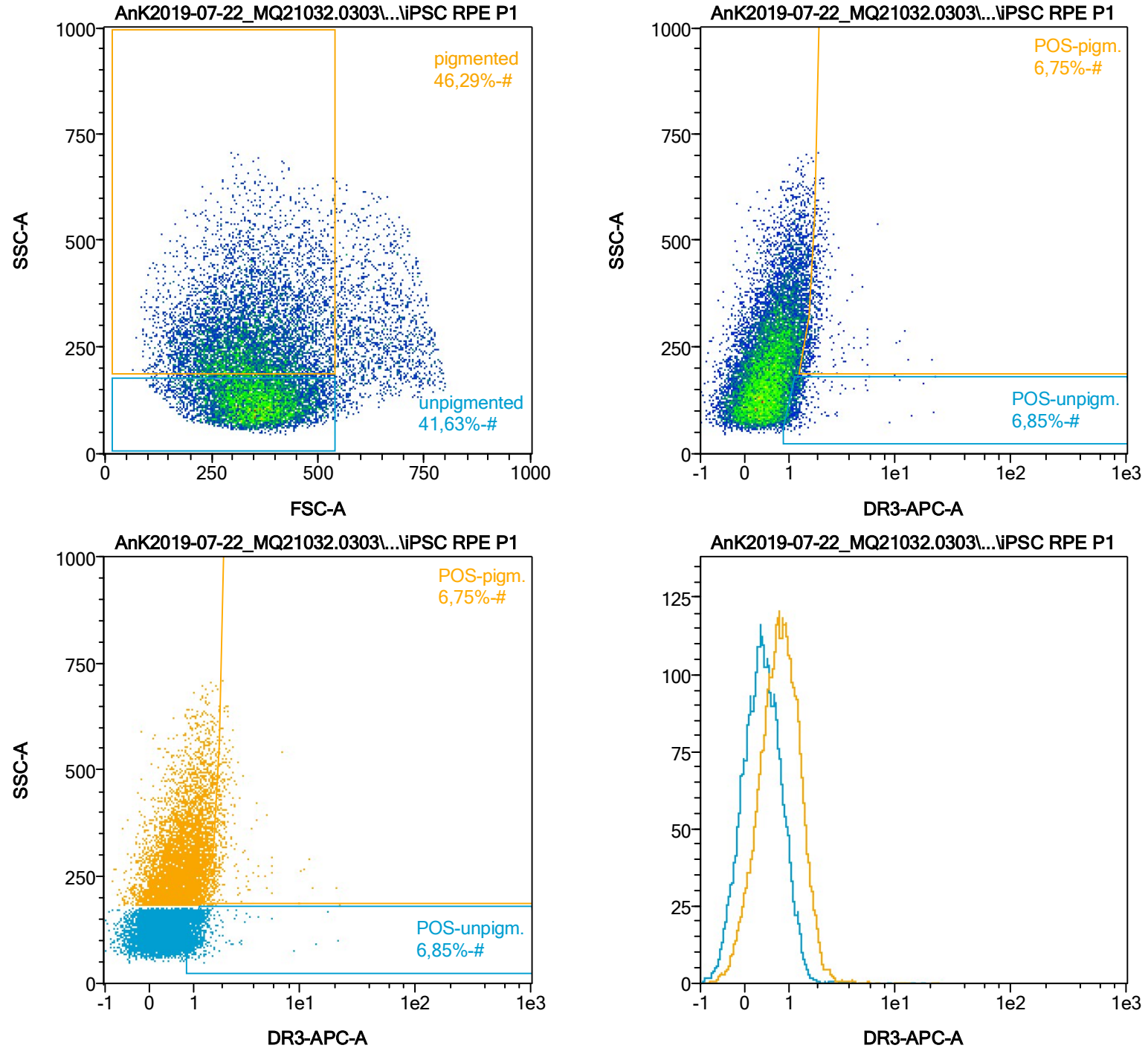

Name	%-#	DR3-APC-A Mean	DR3-APC-A Median
POS-unpigm.	6,85	1,42	1,20
POS-pigm.	6,75	2,18	1,85

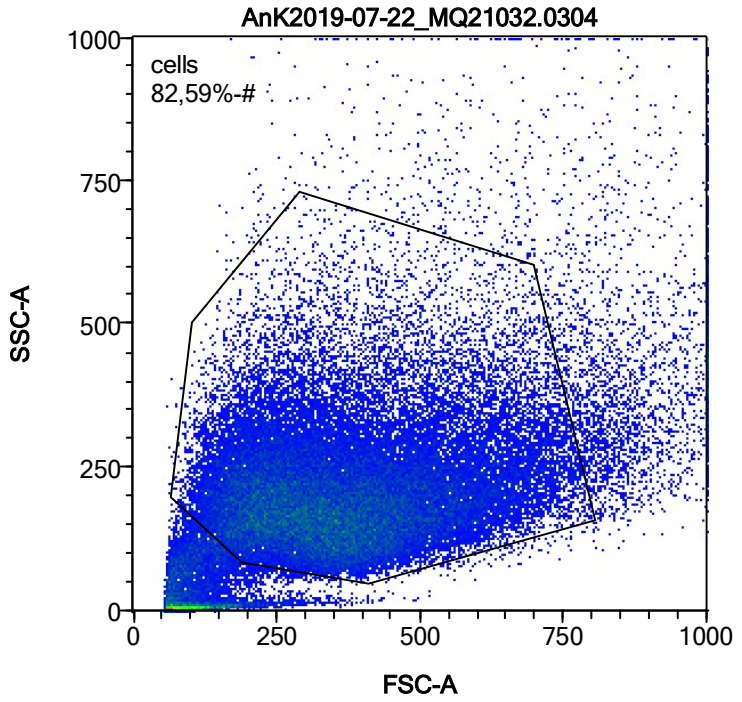

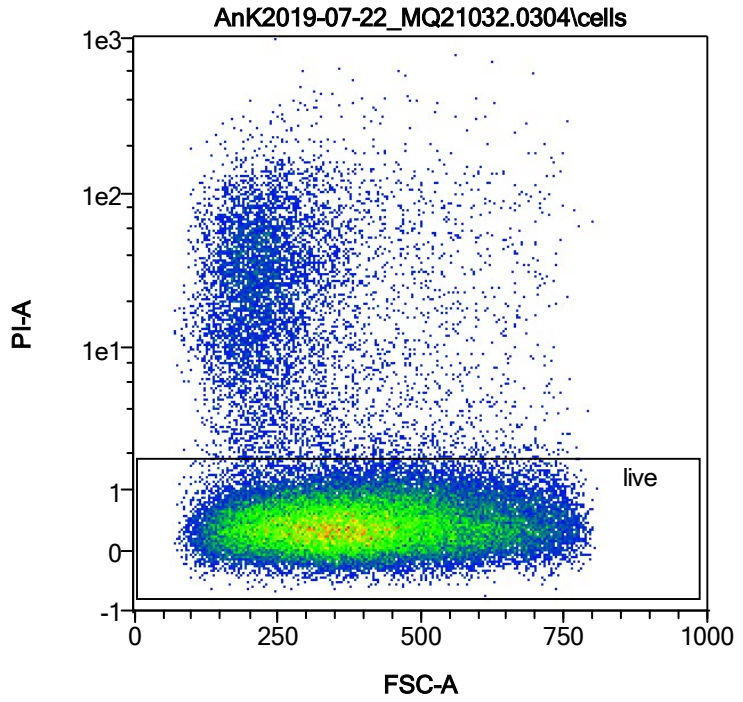

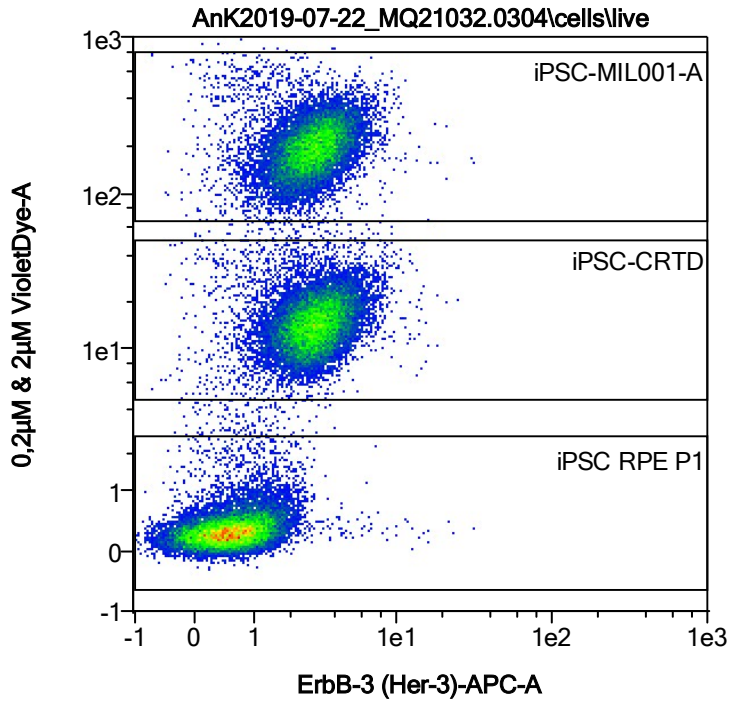

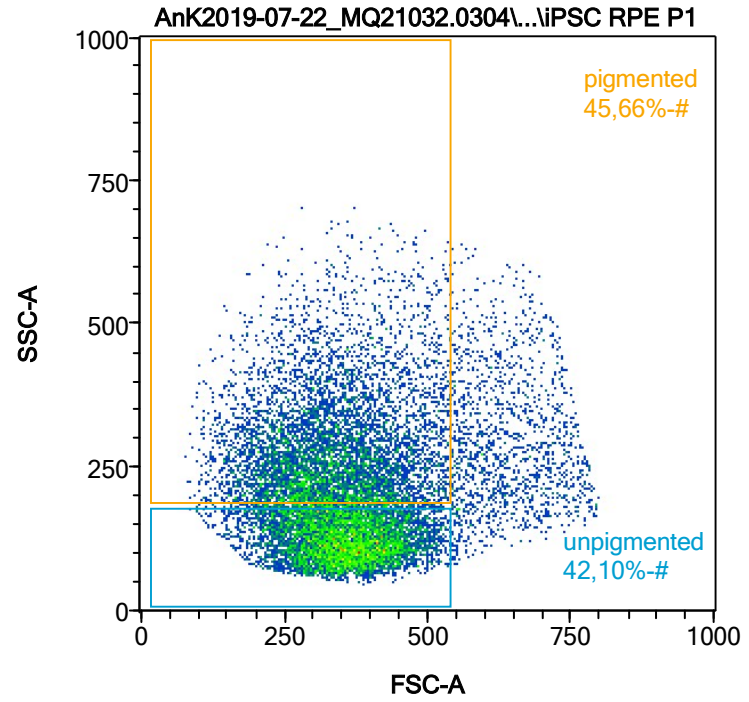

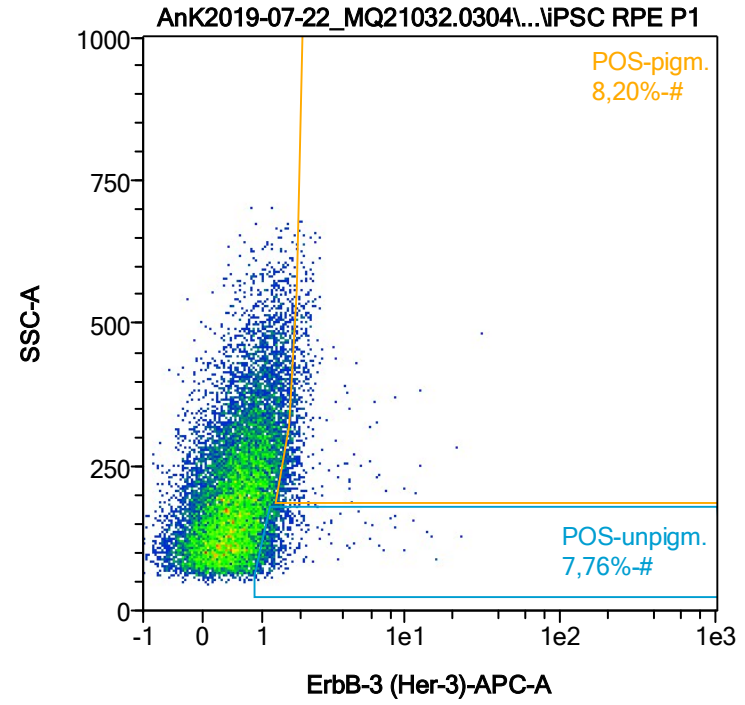

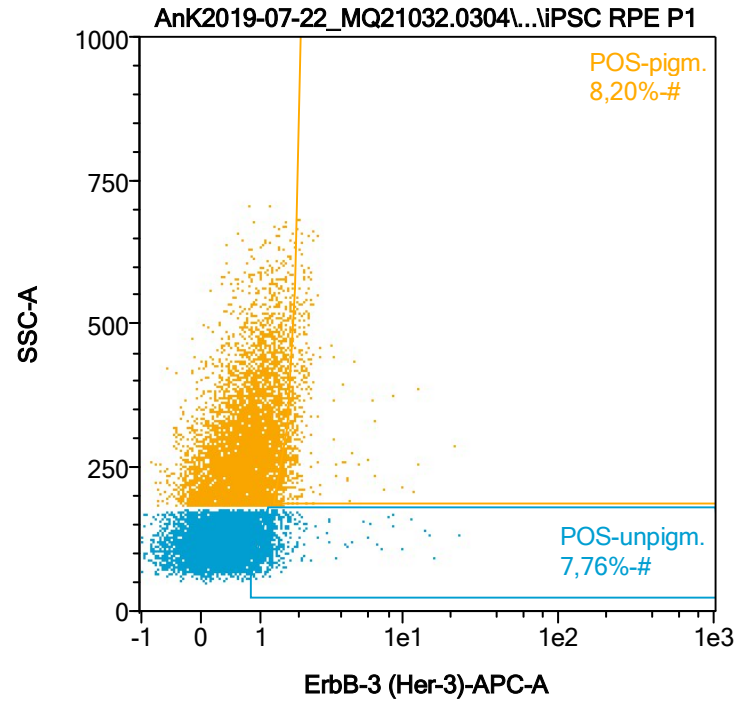

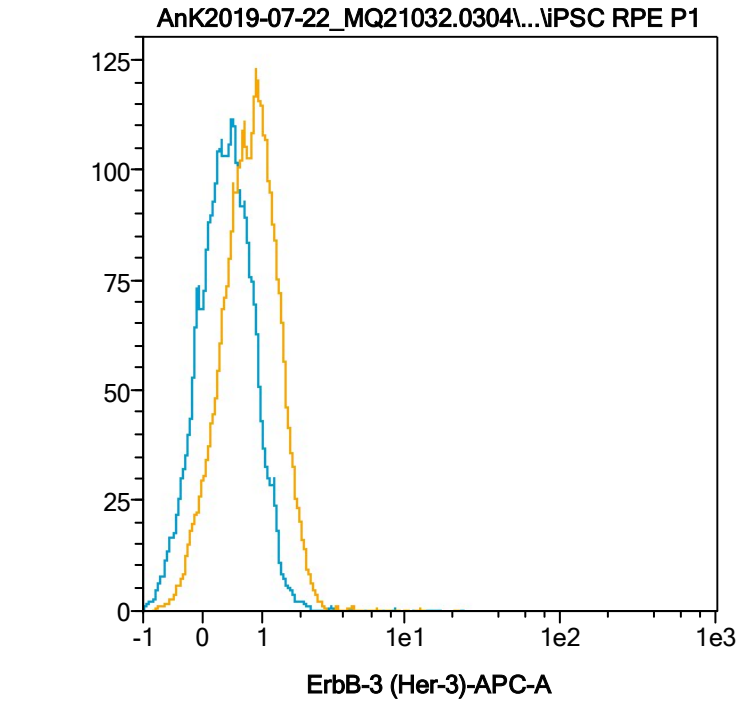

Name	%-#	ErbB-3 (Her-3)-APC-A Mean	ErbB-3 (Her-3)-APC-A Median
POS-unpigm.	7,76	1,61	1,22
POS-pigm.	8,20	2,05	1,81

Date:2024-06-14

Operator: Sebastian Knoebel

Version:2.13.0+54.g95abf0f4

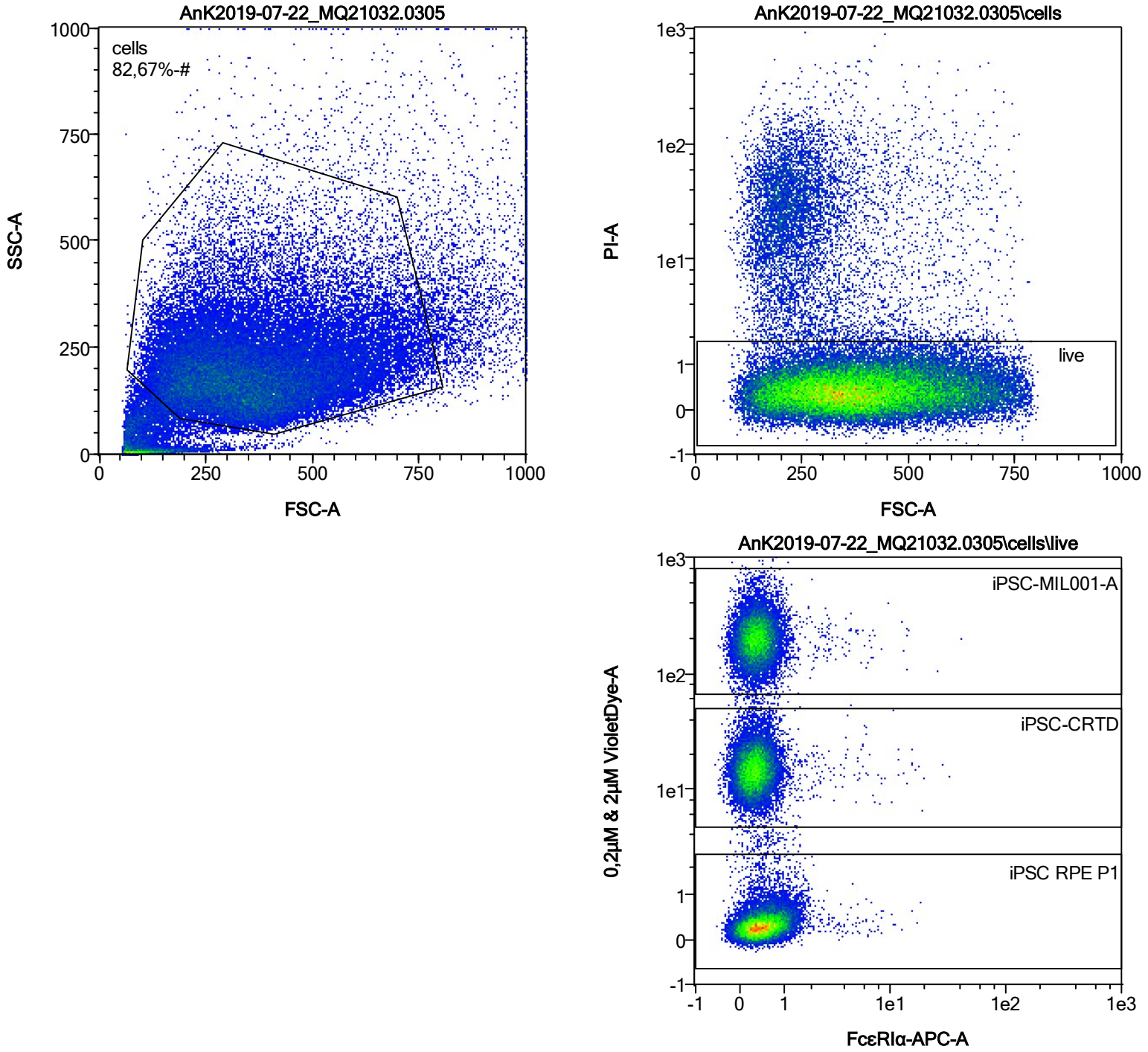

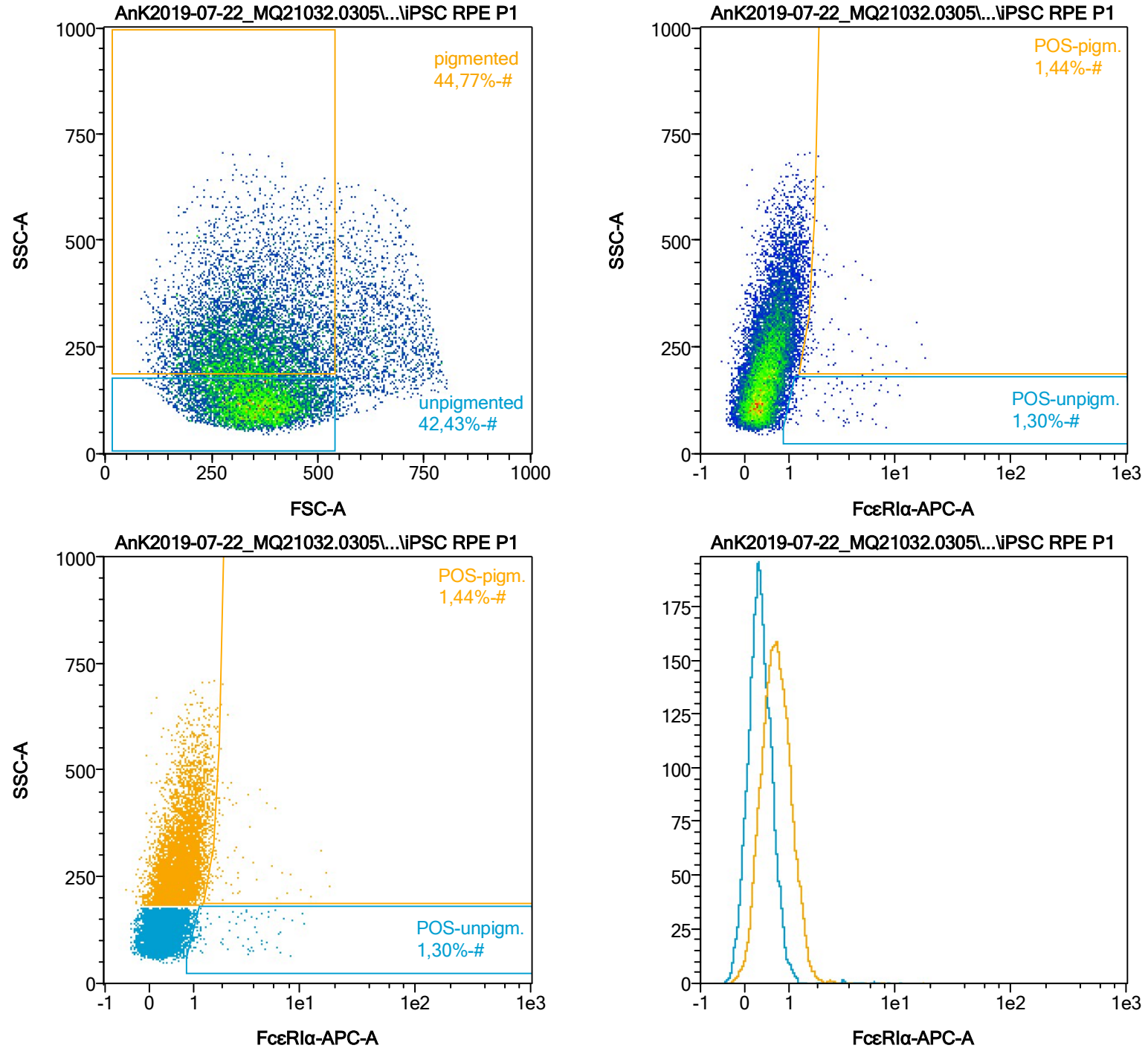

Name	%-#	FcεRIα-APC-A Mean	FcεRIα-APC-A Median
POS-unpigm.	1,30	2,88	2,00
POS-pigm.	1,44	3,08	1,92

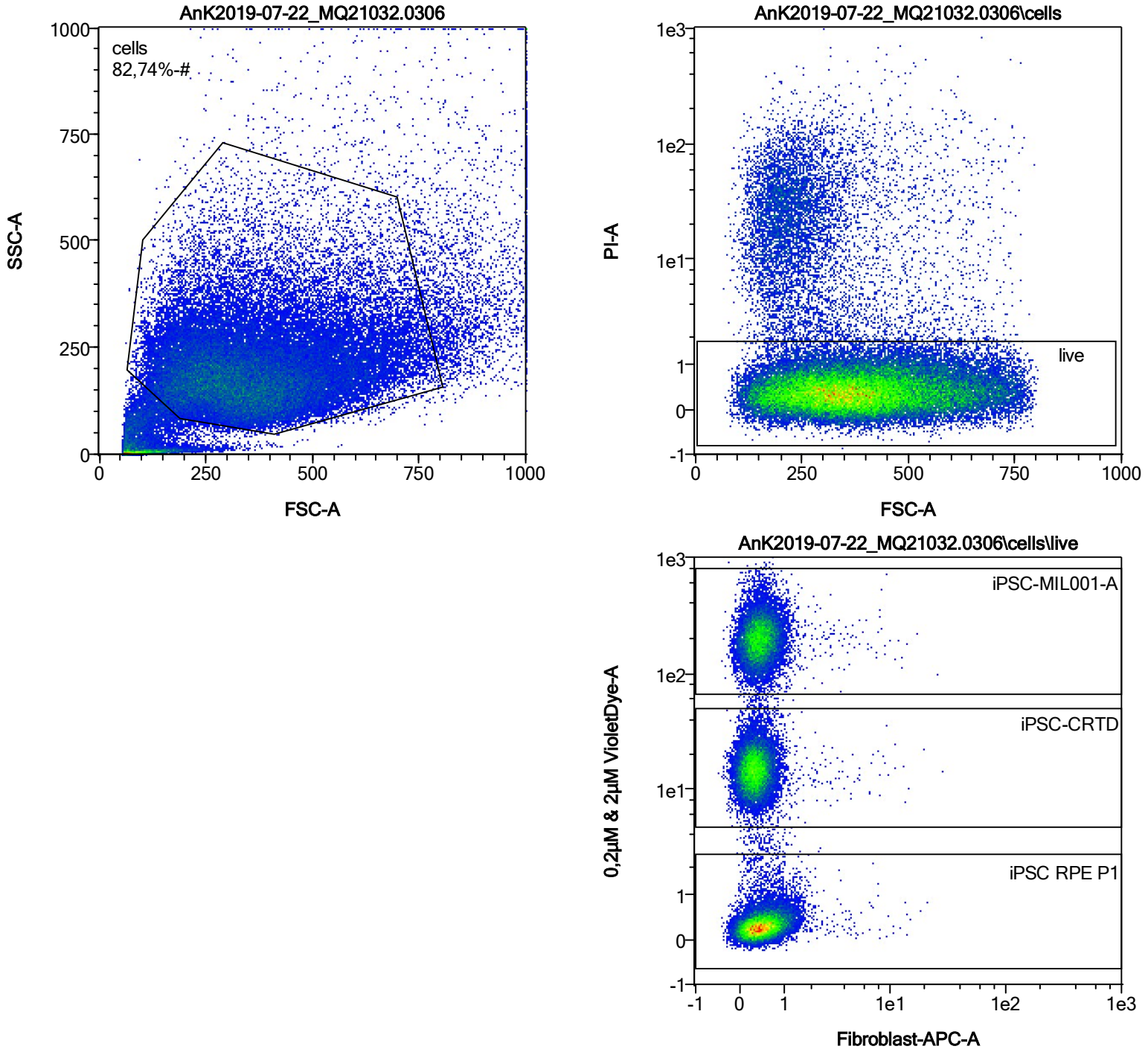

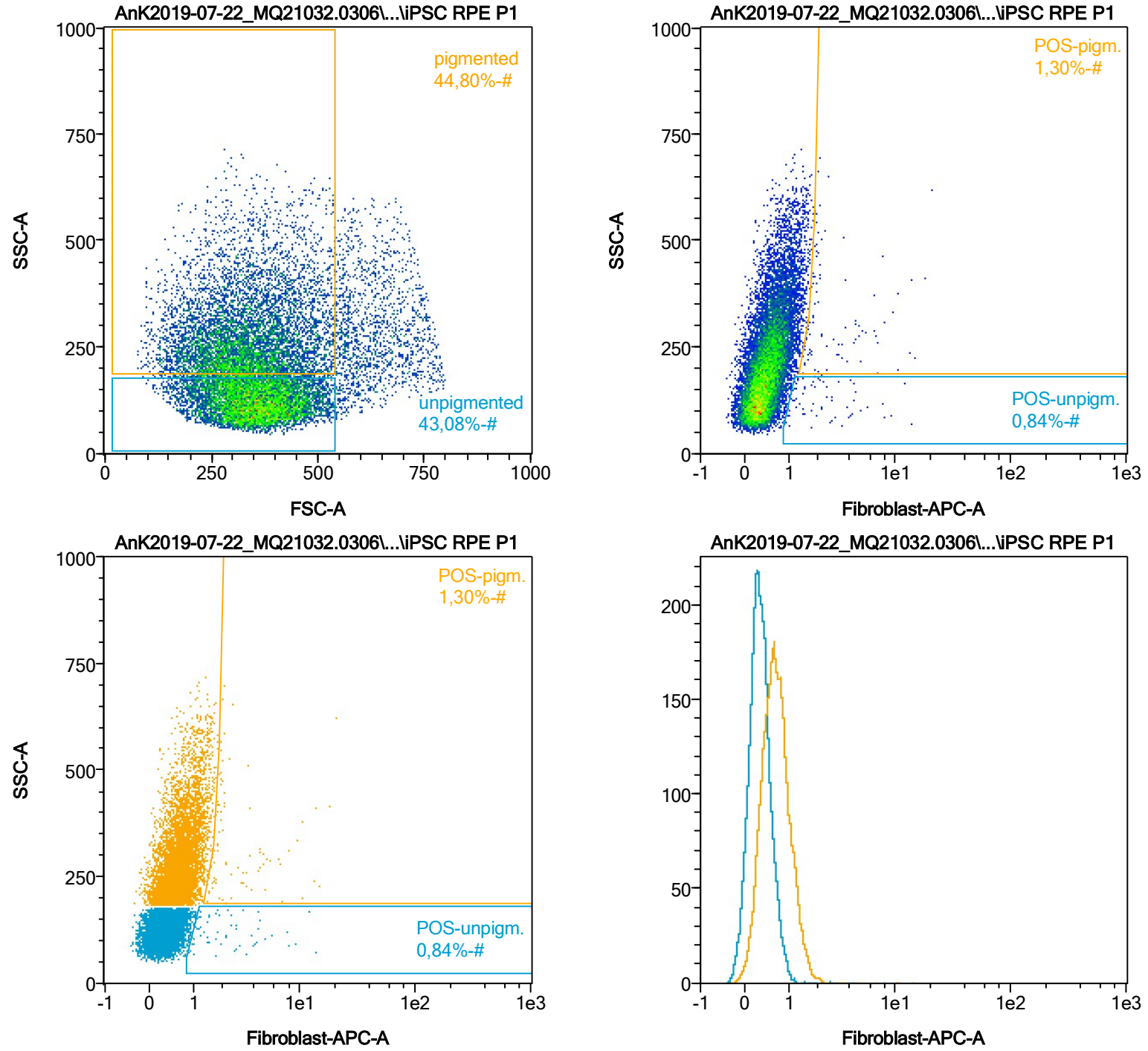

Name	%-#	Fibroblast-APC-A Mean	Fibroblast-APC-A Median
POS-unpigm.	0,84	2,93	1,89
POS-pigm.	1,30	3,64	2,08

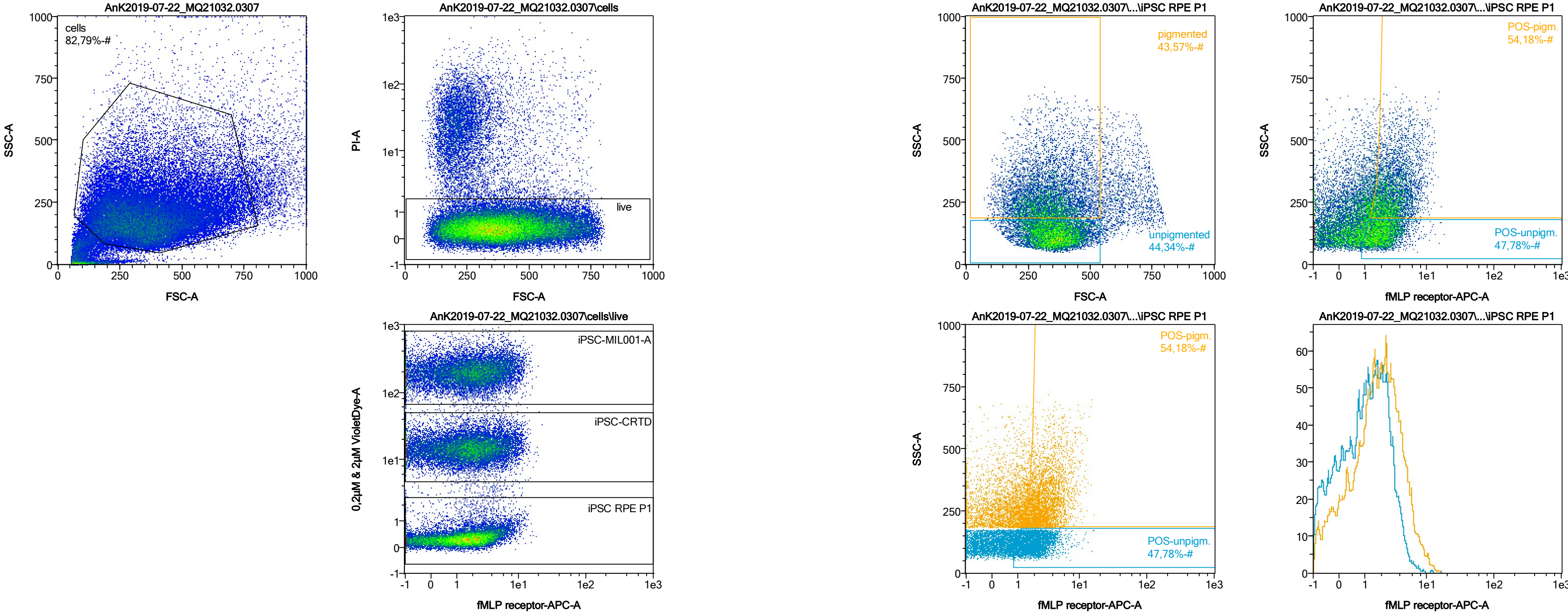

Name	%-#	fMLP receptor-APC-A Mean	fMLP receptor-APC-A Median
POS-unpigm.	47,78	2,25	1,97
POS-pigm.	54,18	3,37	2,84

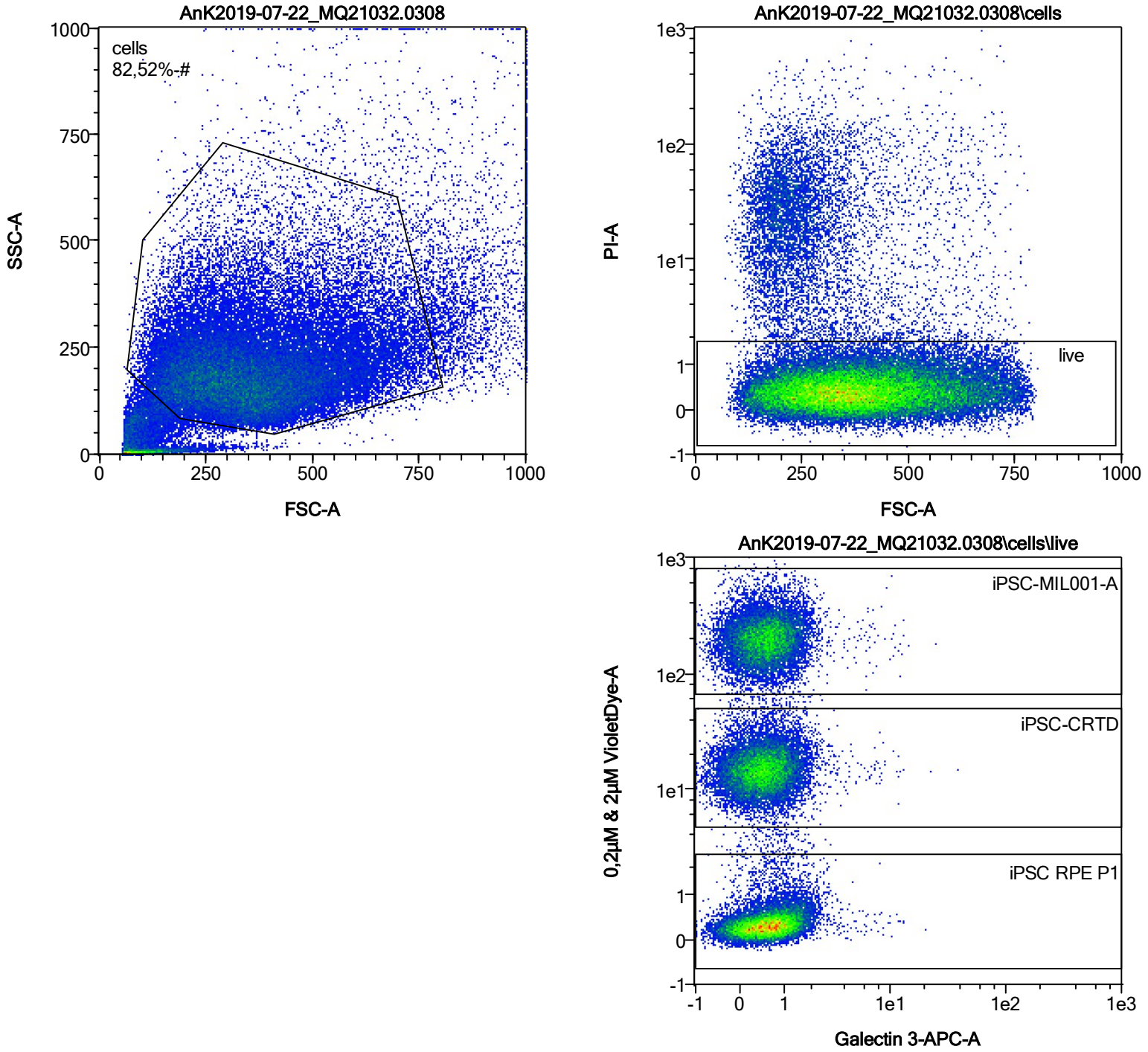

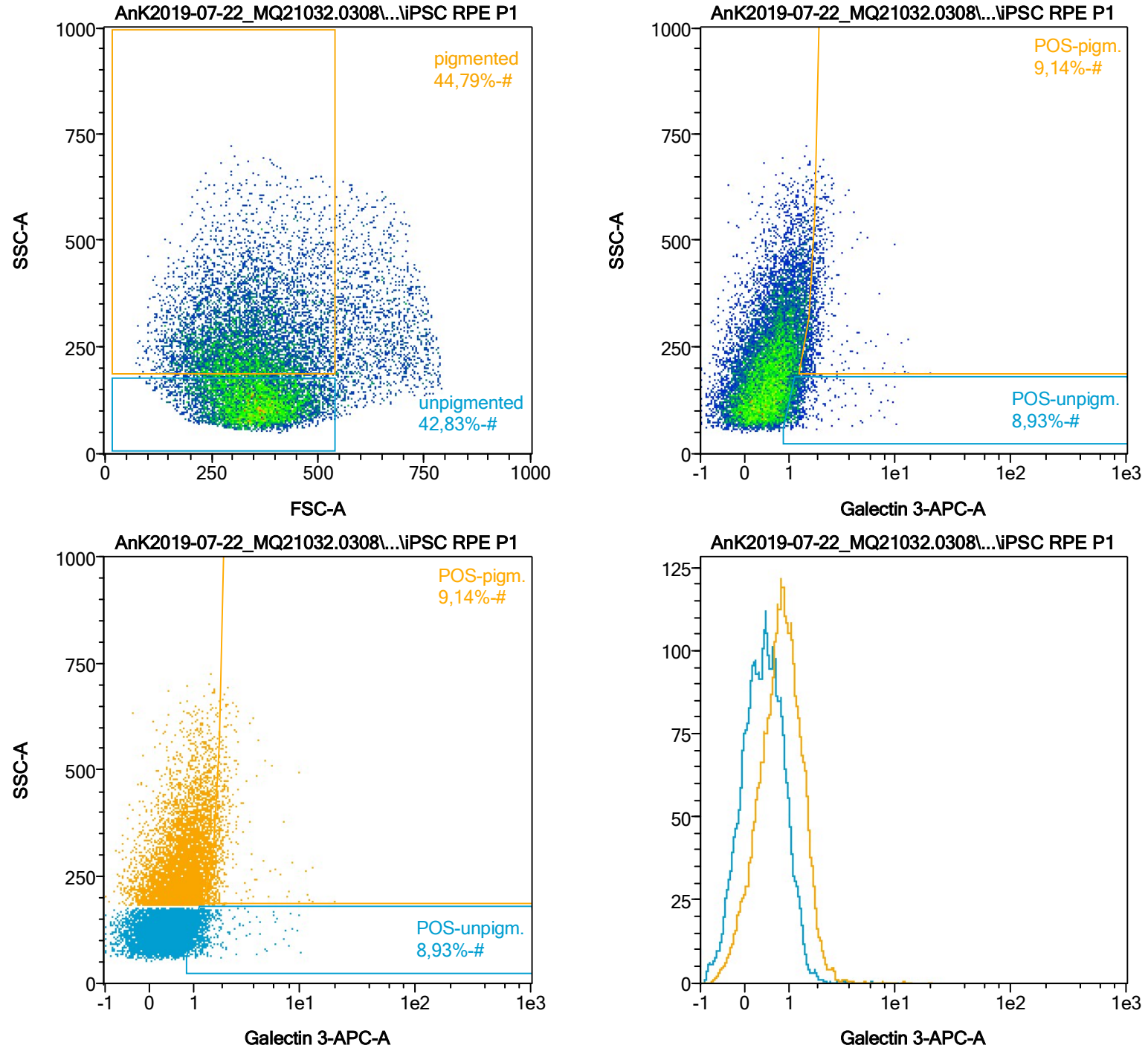

Name	%-#	Galectin 3-APC-A Mean	Galectin 3-APC-A Median
POS-unpigm.	8,93	1,49	1,22
POS-pigm.	9,14	2,14	1,83

Date:2024-06-14

Operator: Sebastian Knoebel

Version:2.13.0+54.g95abf0f4

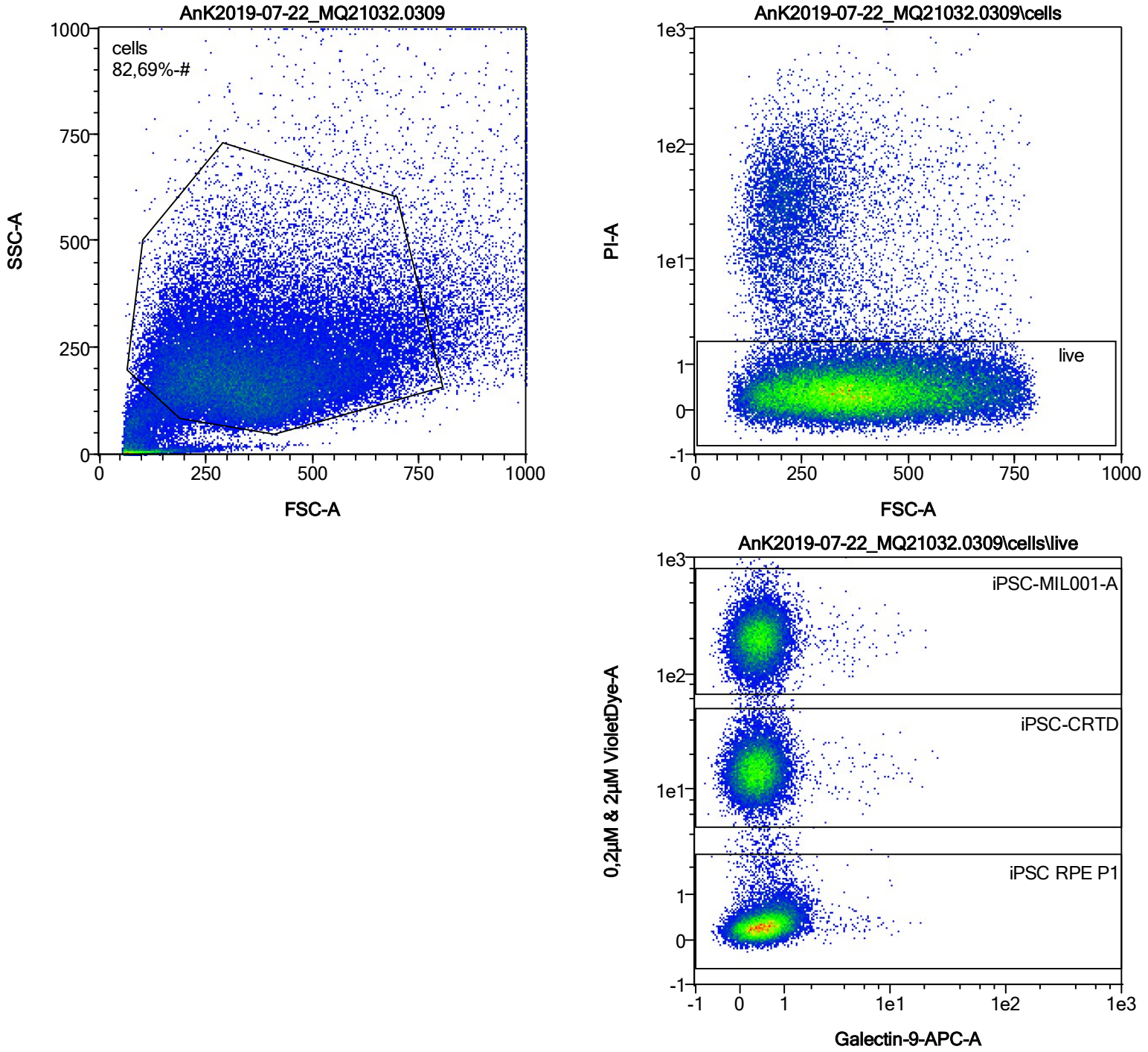

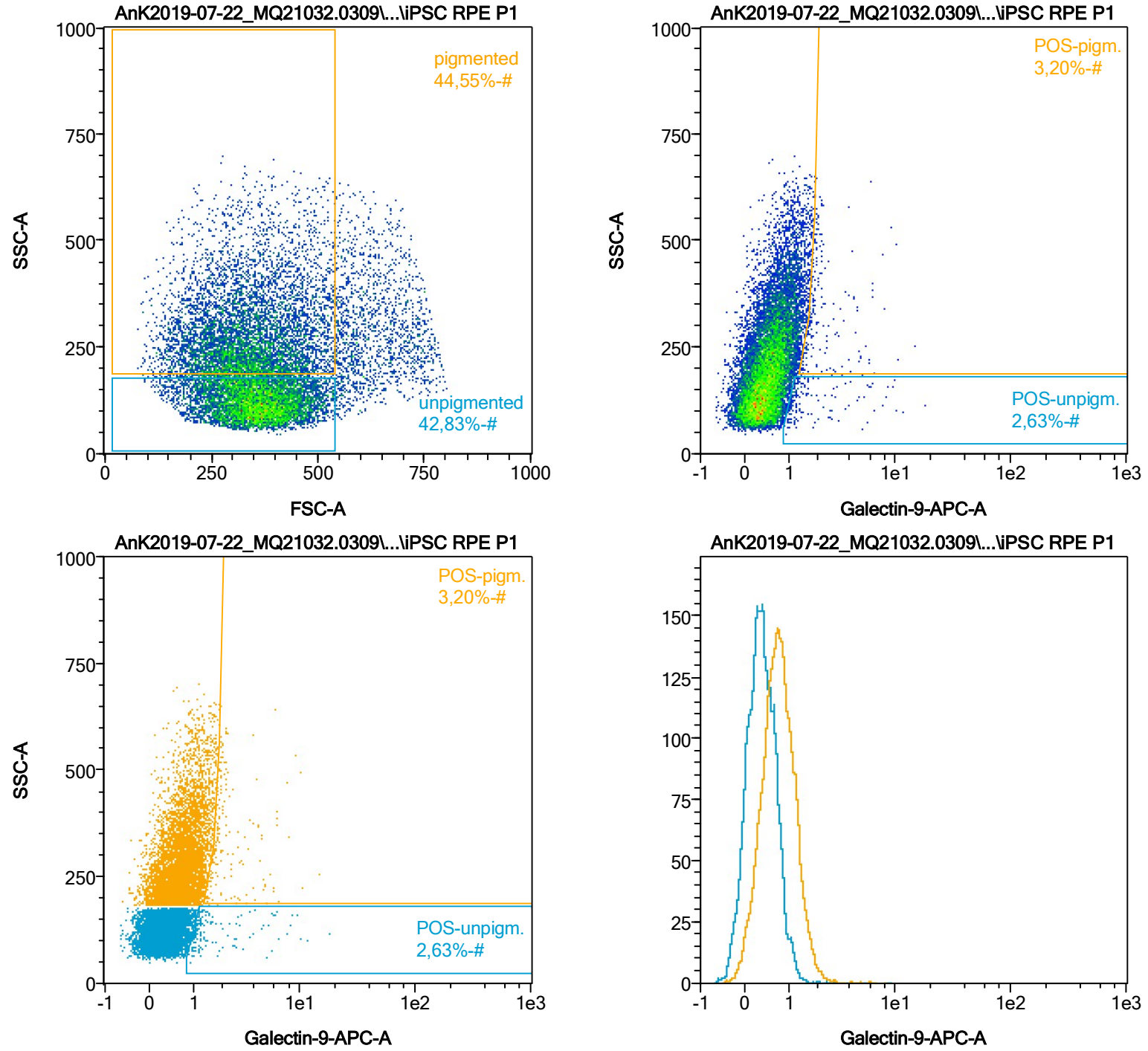

Name	%-#	Galectin-9-APC-A Mean	Galectin-9-APC-A Median
POS-unpigm.	2,63	2,06	1,21
POS-pigm.	3,20	2,50	1,88

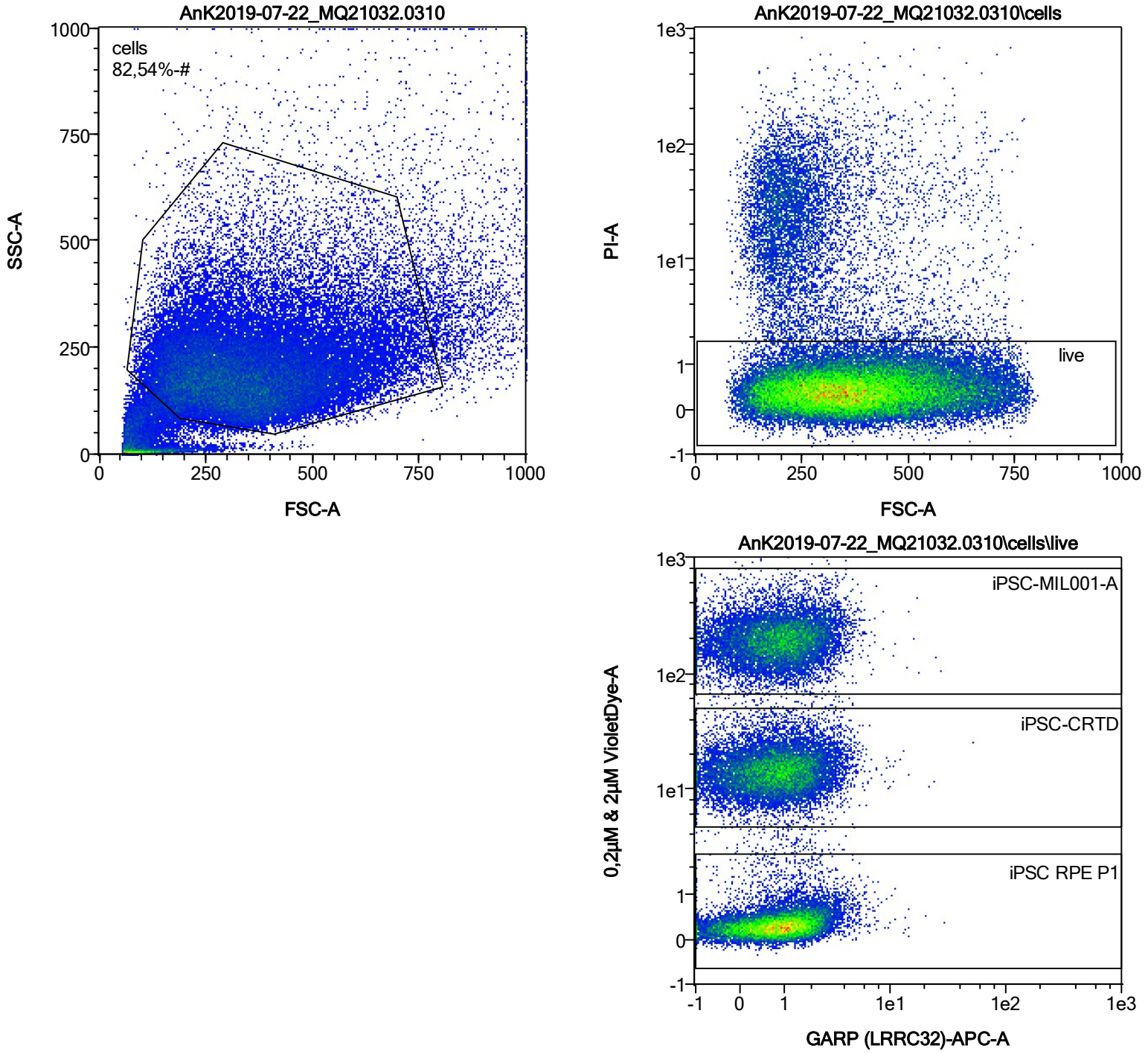

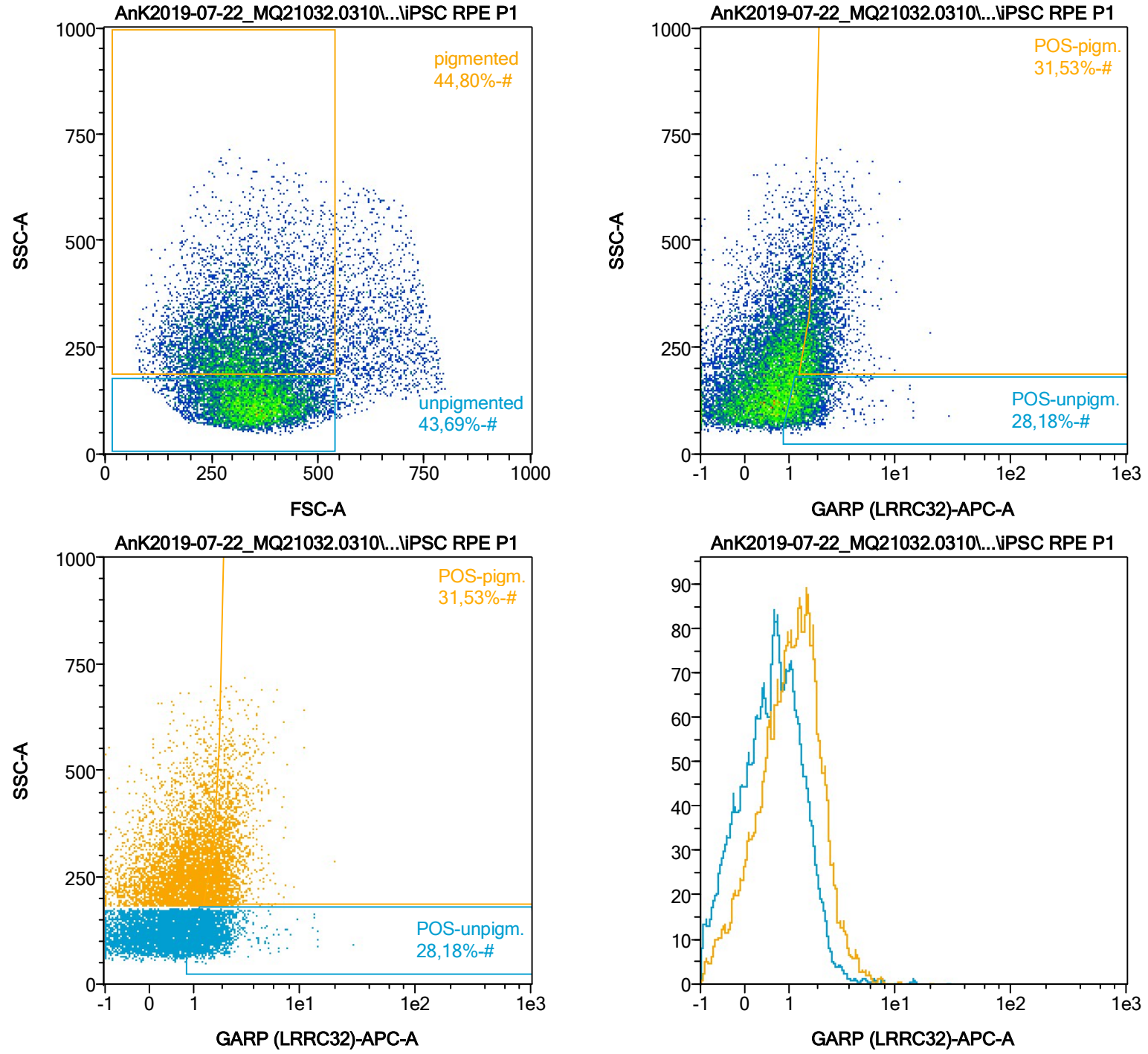

Name	%-#	GARP (LRRC32)-APC-A Mean	GARP (LRRC32)-APC-A Median
POS-unpigm.	28,18	1,68	1,43
POS-pigm.	31,53	2,28	2,03

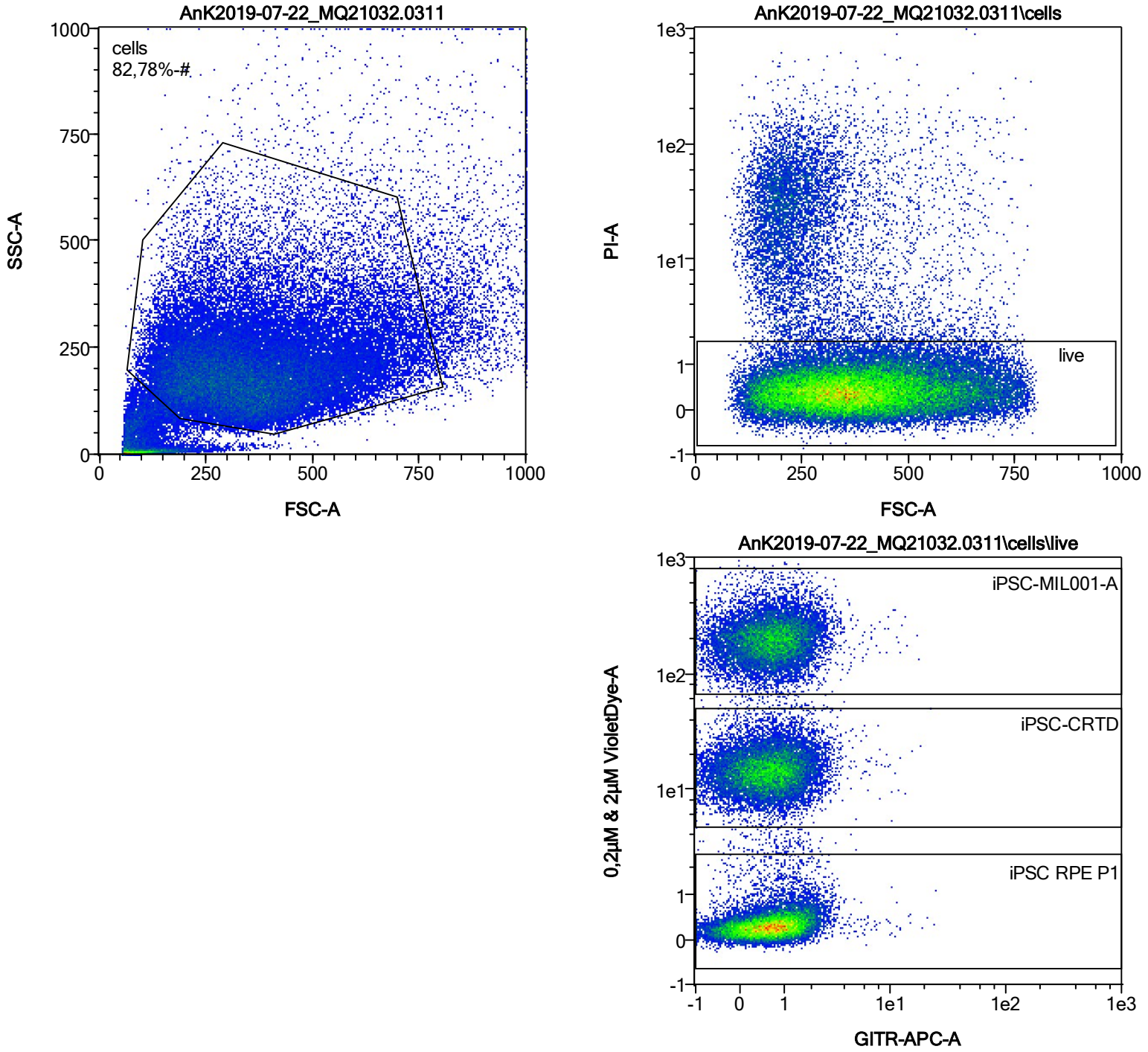

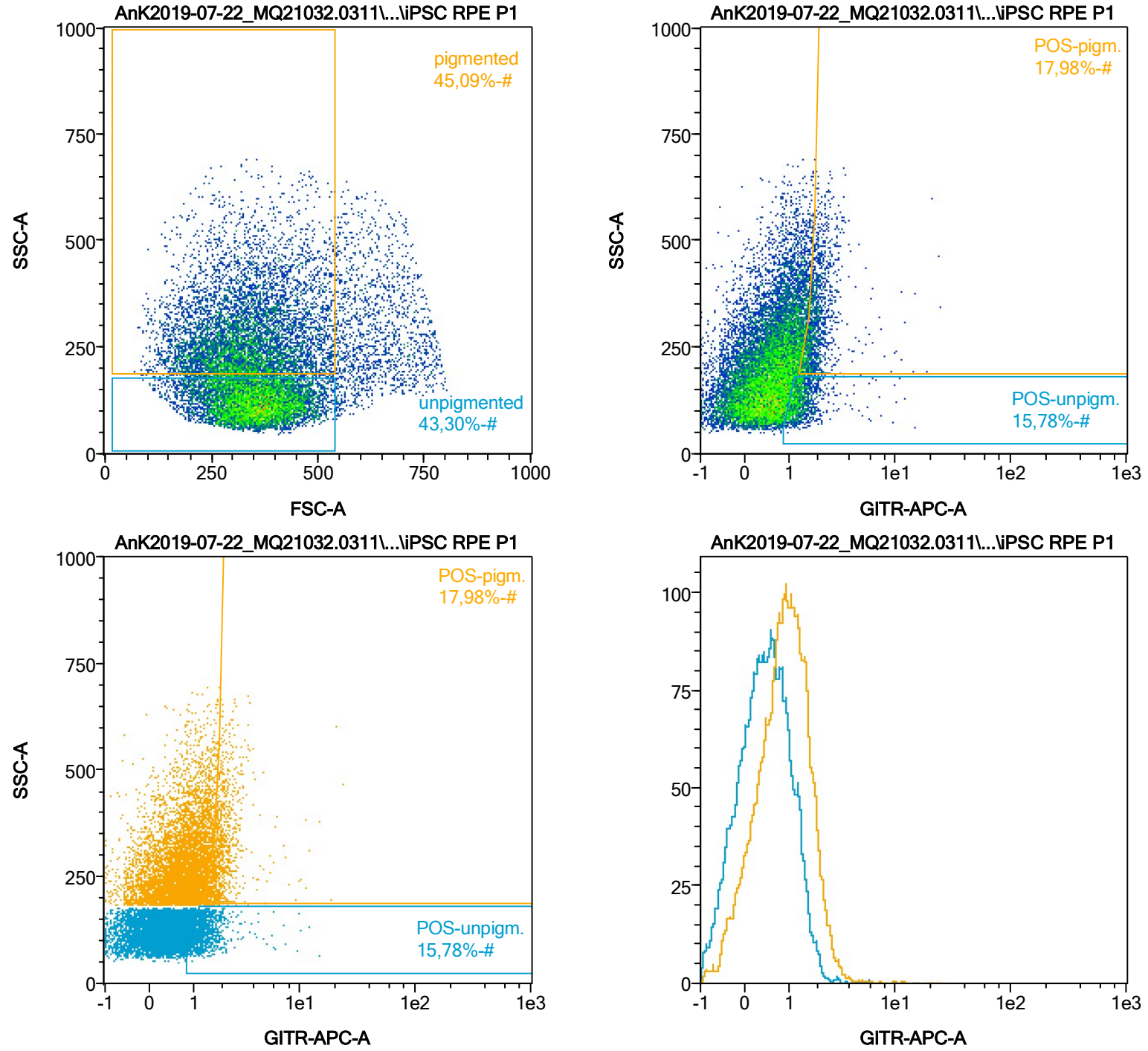

Name	%-#	GITR-APC-A Mean	GITR-APC-A Median
POS-unpigm.	15,78	1,47	1,29
POS-pigm.	17,98	2,11	1,87

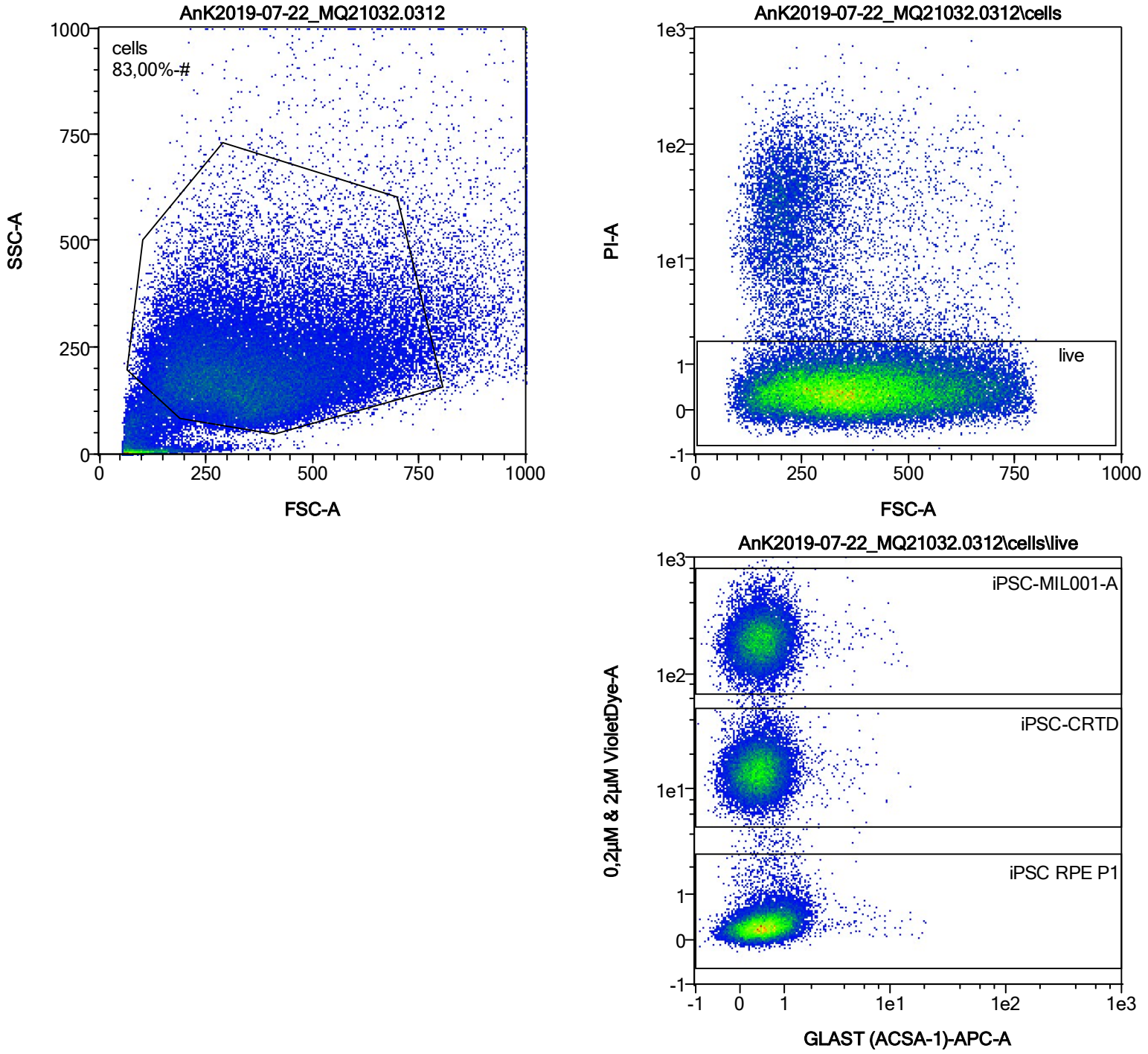

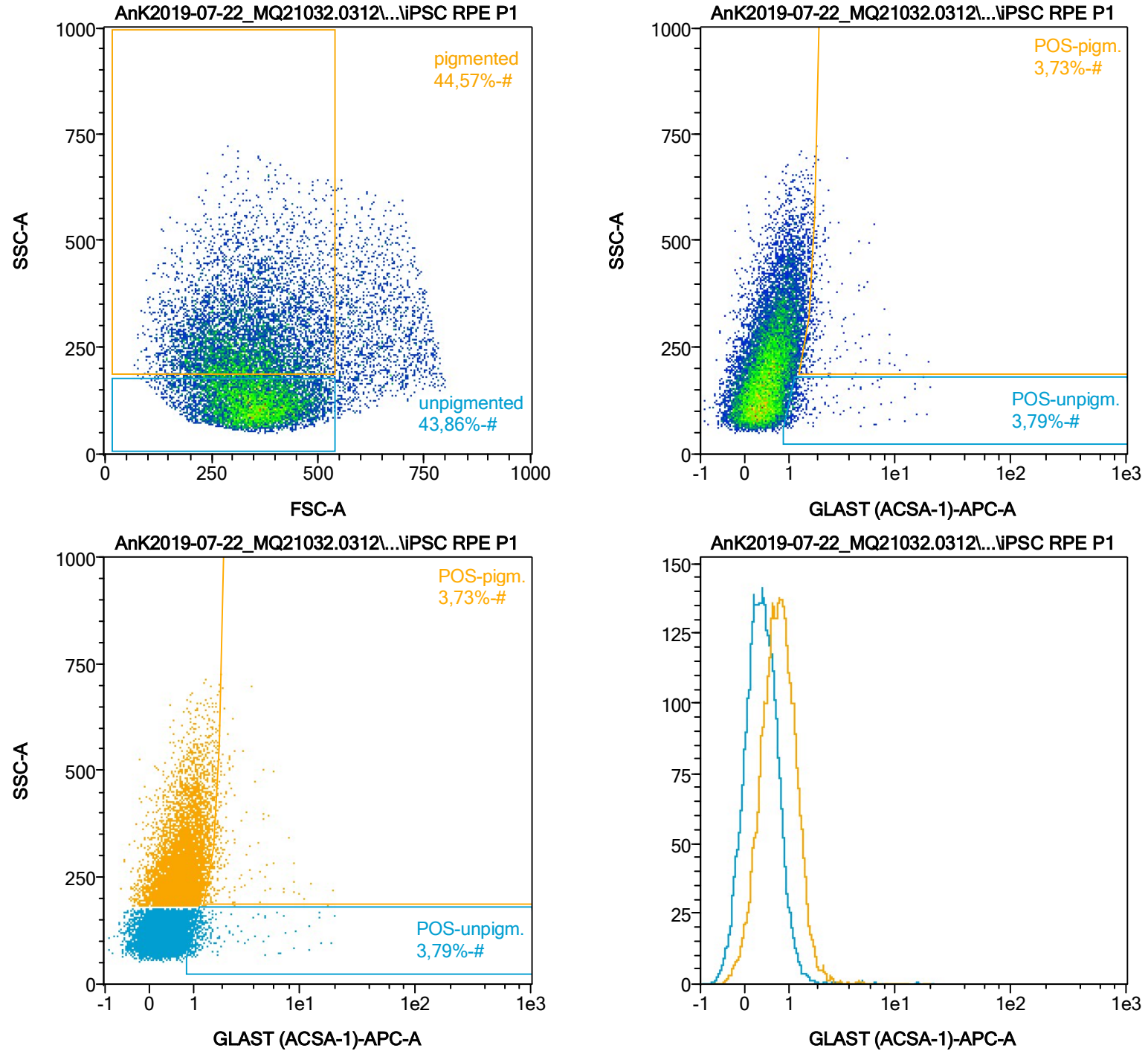

Name	%-#	GLAST (ACSA-1)-APC-A Mean	GLAST (ACSA-1)-APC-A Median
POS-unpigm.	3,79	1,96	1,21
POS-pigm.	3,73	2,47	1,86

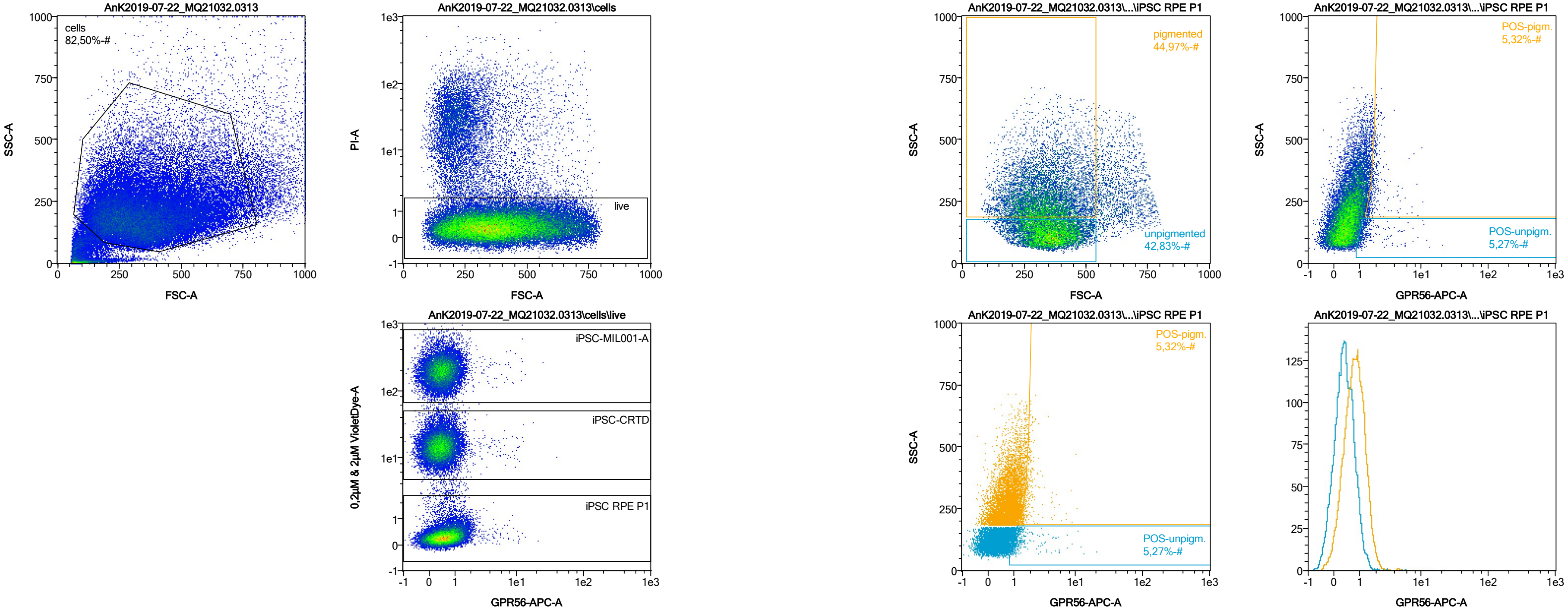

Name	%-#	GPR56-APC-A Mean	GPR56-APC-A Median
POS-unpigm.	5,27	1,65	1,19
POS-pigm.	5,32	2,05	1,80

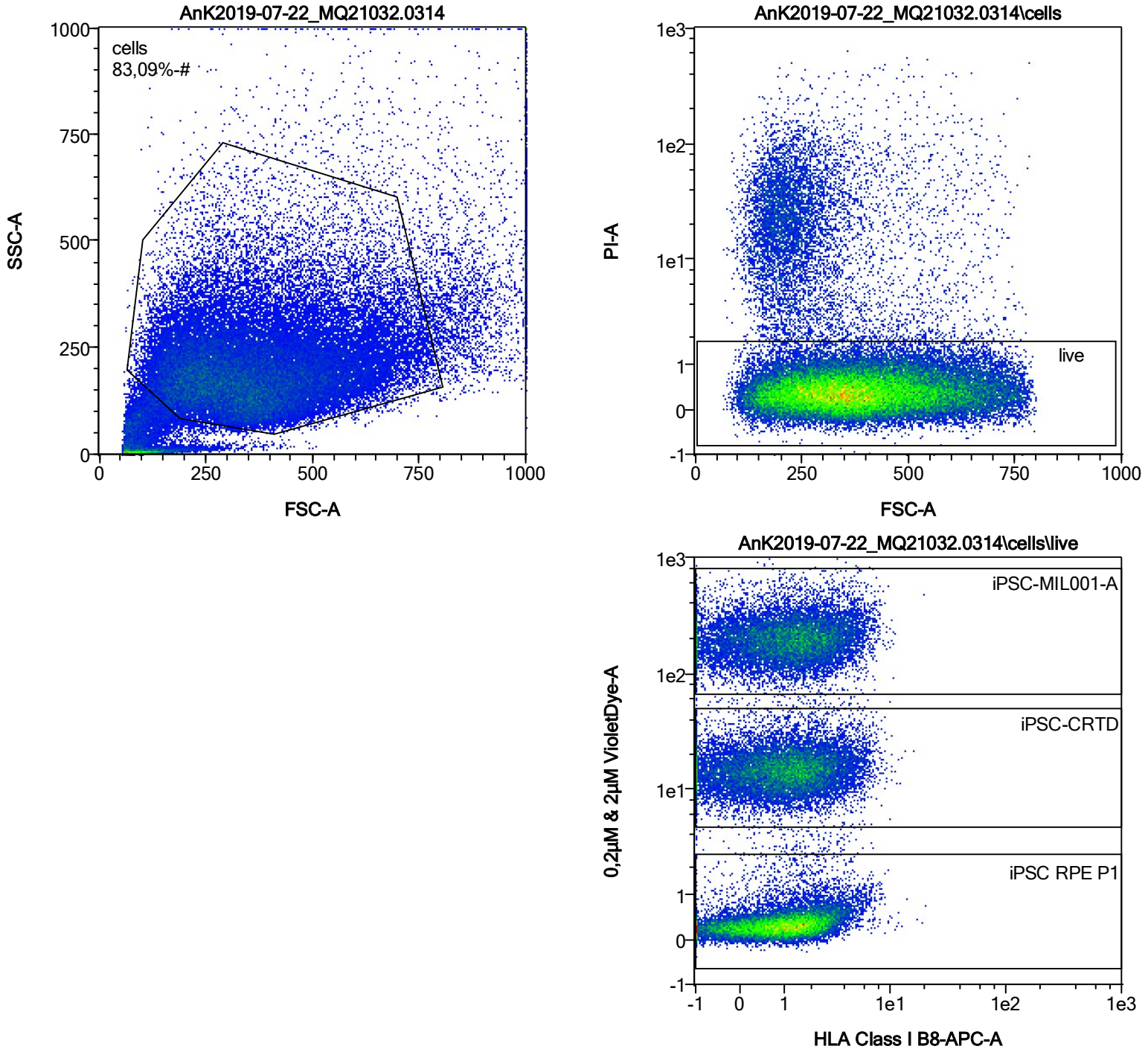

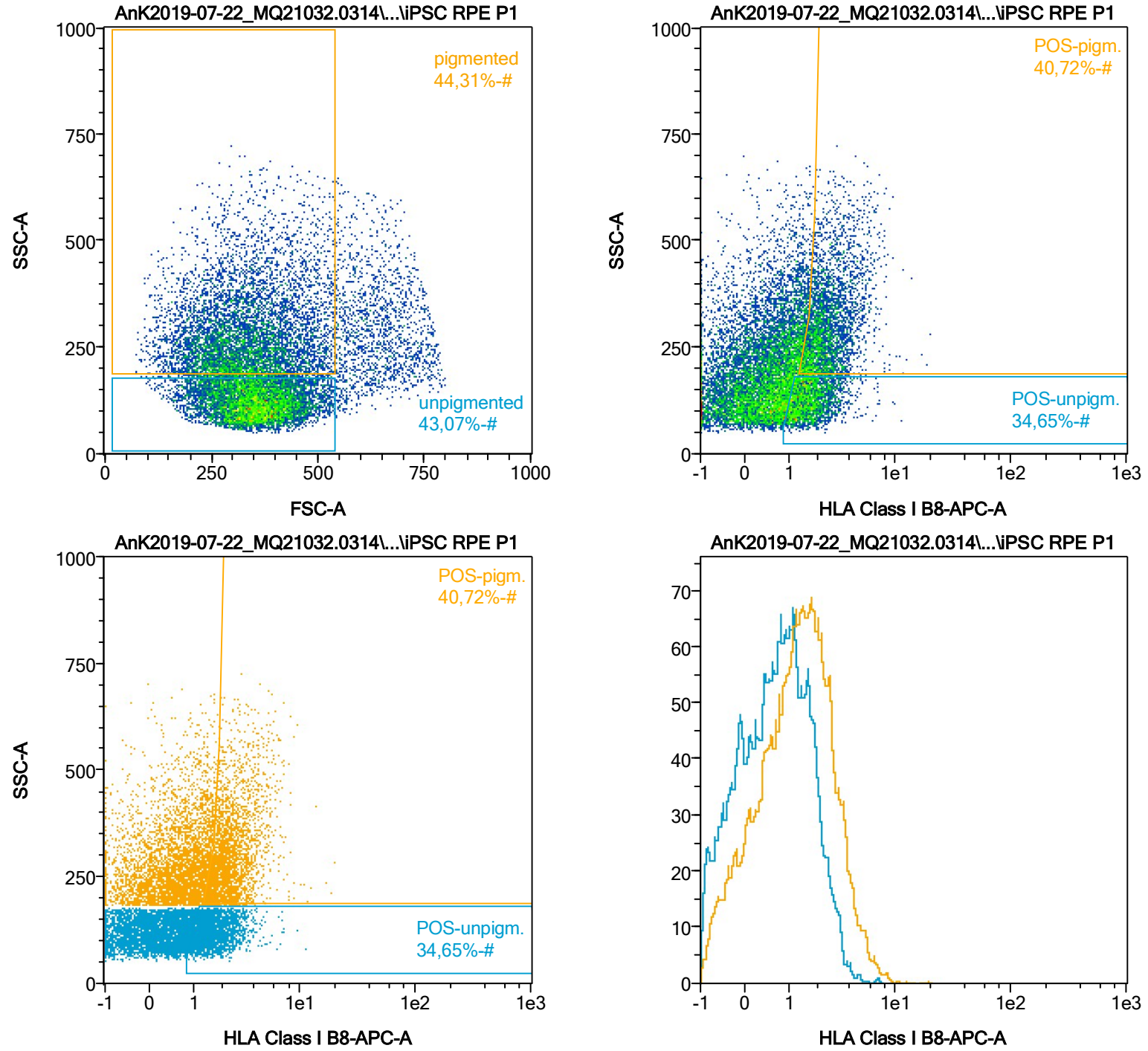

Name	%-#	HLA Class I B8-APC-A Mean	HLA Class I B8-APC-A Median
POS-unpigm.	34,65	1,78	1,60
POS-pigm.	40,72	2,65	2,29

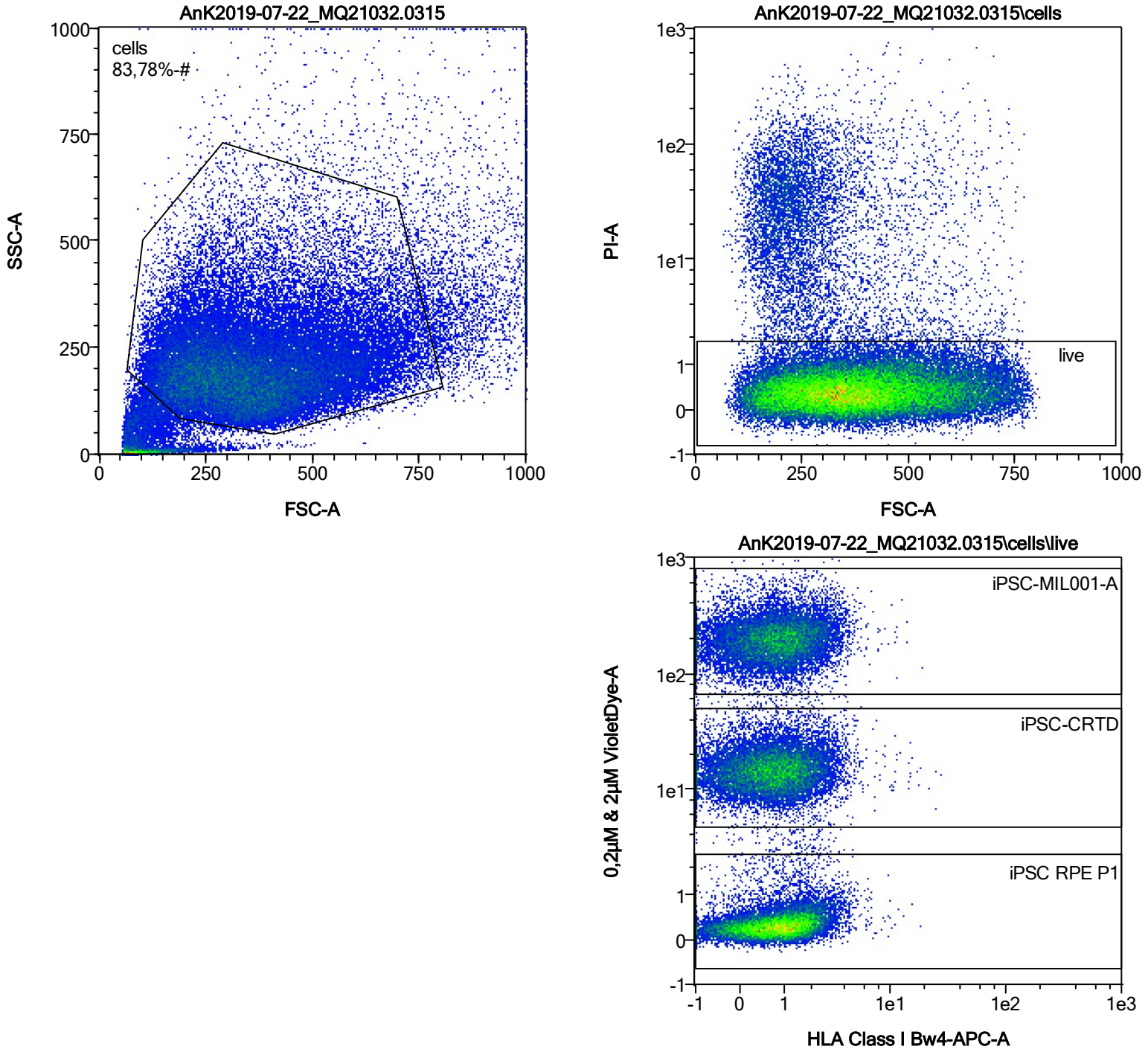

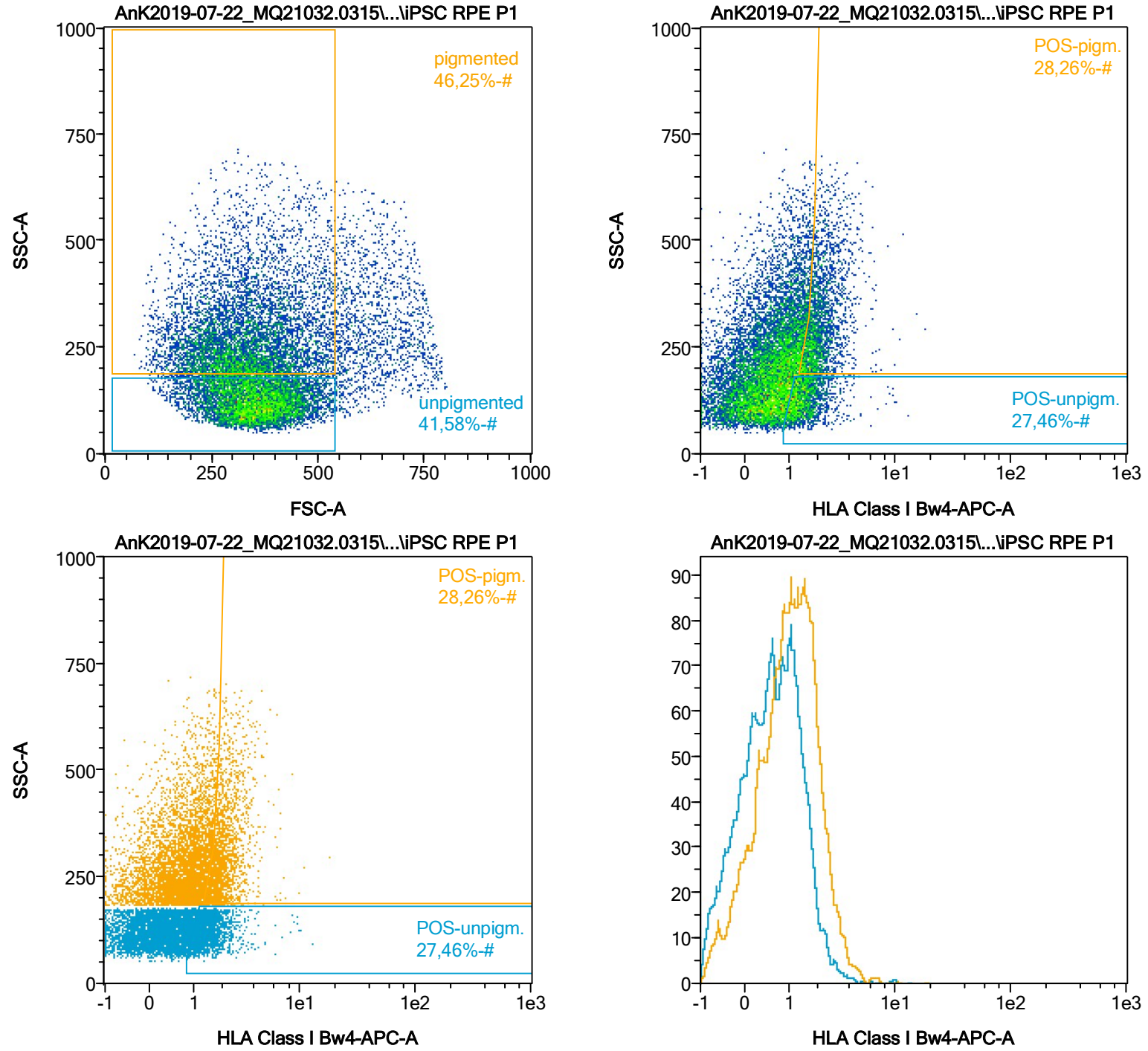

Name	%-#	HLA Class I Bw4-APC-A Mean	HLA Class I Bw4-APC-A Median
POS-unpigm.	27,46	1,57	1,39
POS-pigm.	28,26	2,20	1,99

Date:2024-06-14
Operator: Sebastian Knoebel
Version:2.13.0+54.g95abf0f4

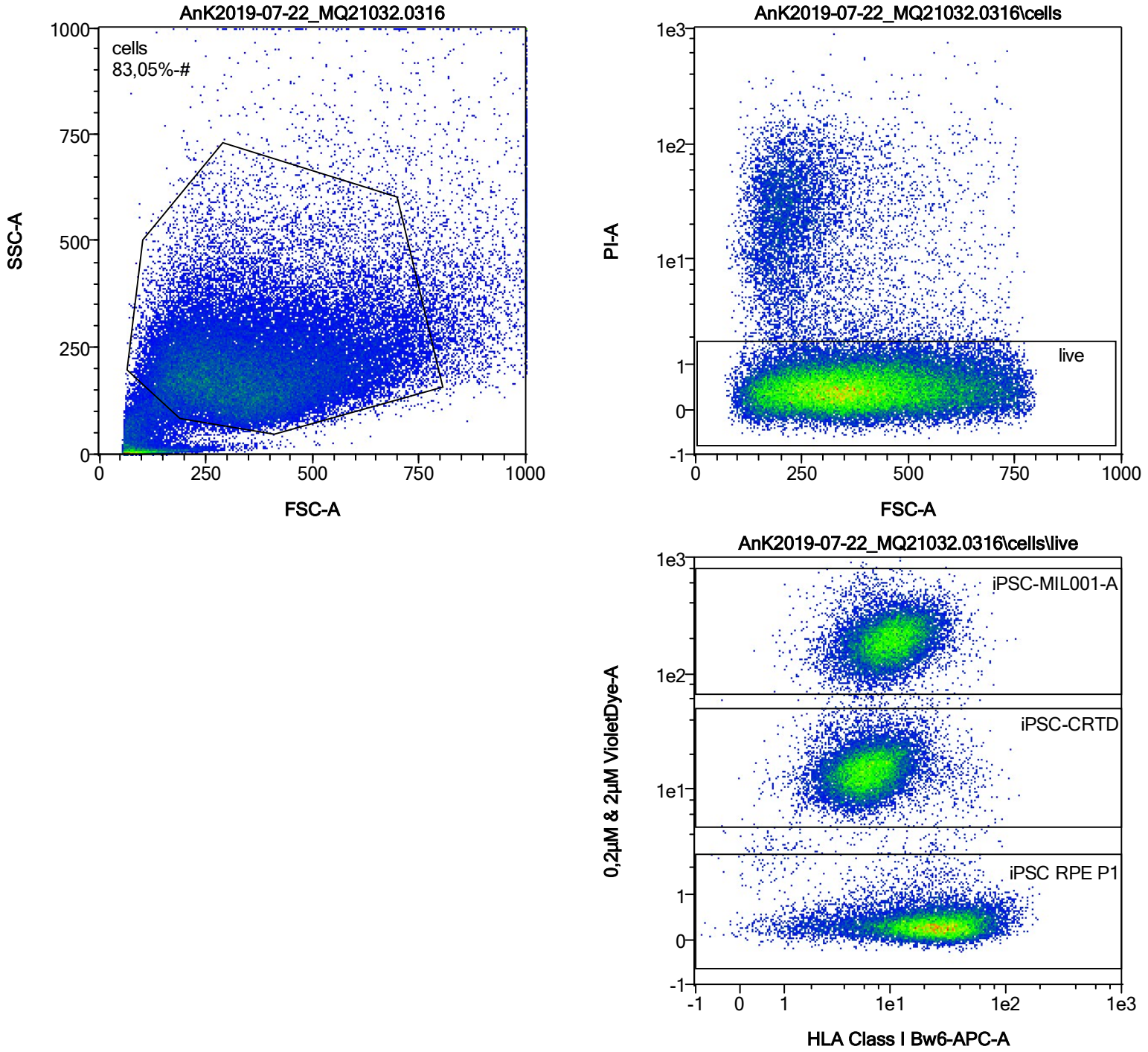

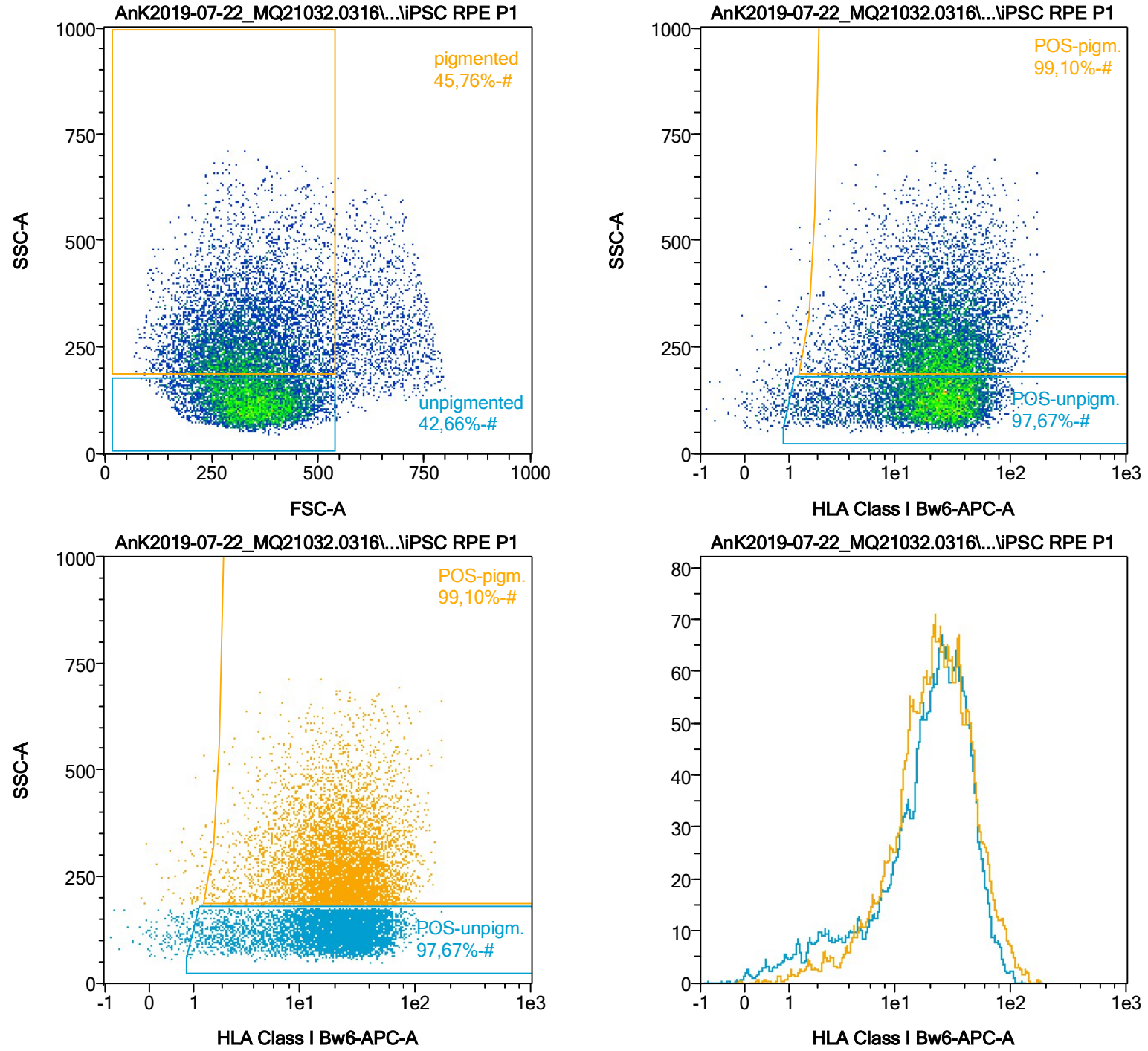

Name	%-#	HLA Class I Bw6-APC-A Mean	HLA Class I Bw6-APC-A Median
POS-unpigm.	97,67	24,84	22,25
POS-pigm.	99,10	27,52	22,66

Date:2024-06-14

Operator: Sebastian Knoebel

Version:2.13.0+54.g95abf0f4

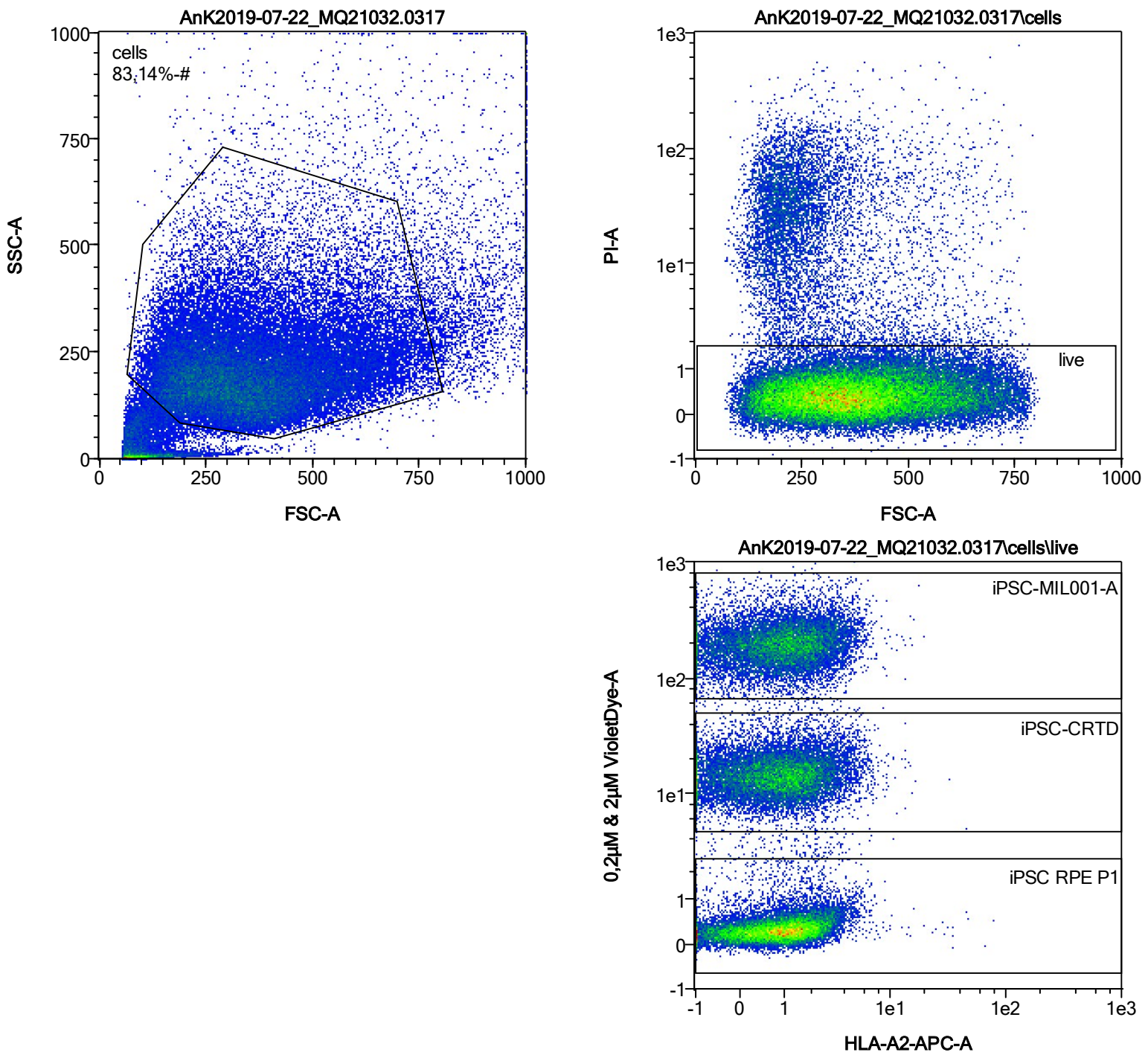

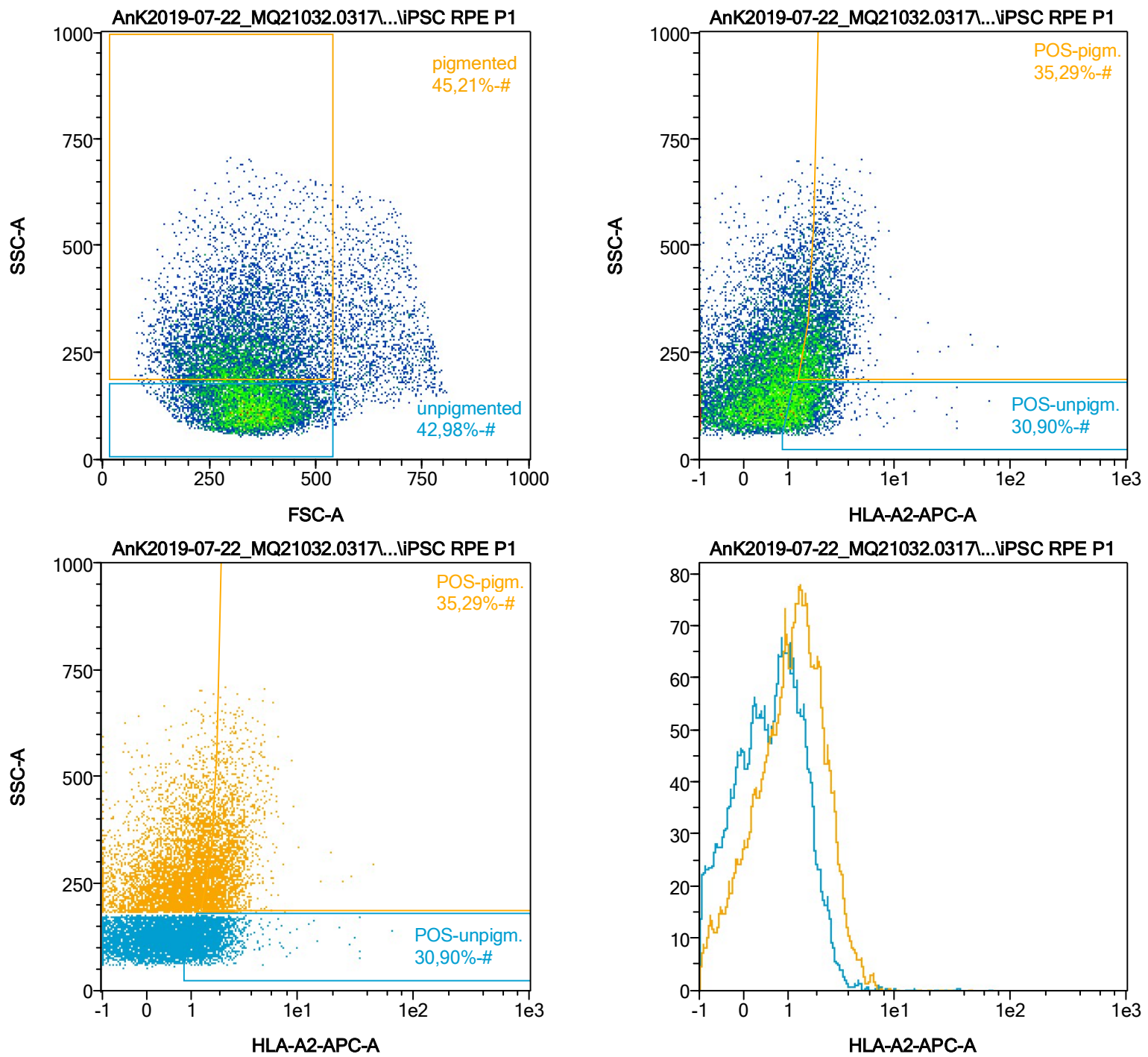

Name	%-#	HLA-A2-APC-A Mean	HLA-A2-APC-A Median
POS-unpigm.	30,90	1,82	1,49
POS-pigm.	35,29	2,48	2,19

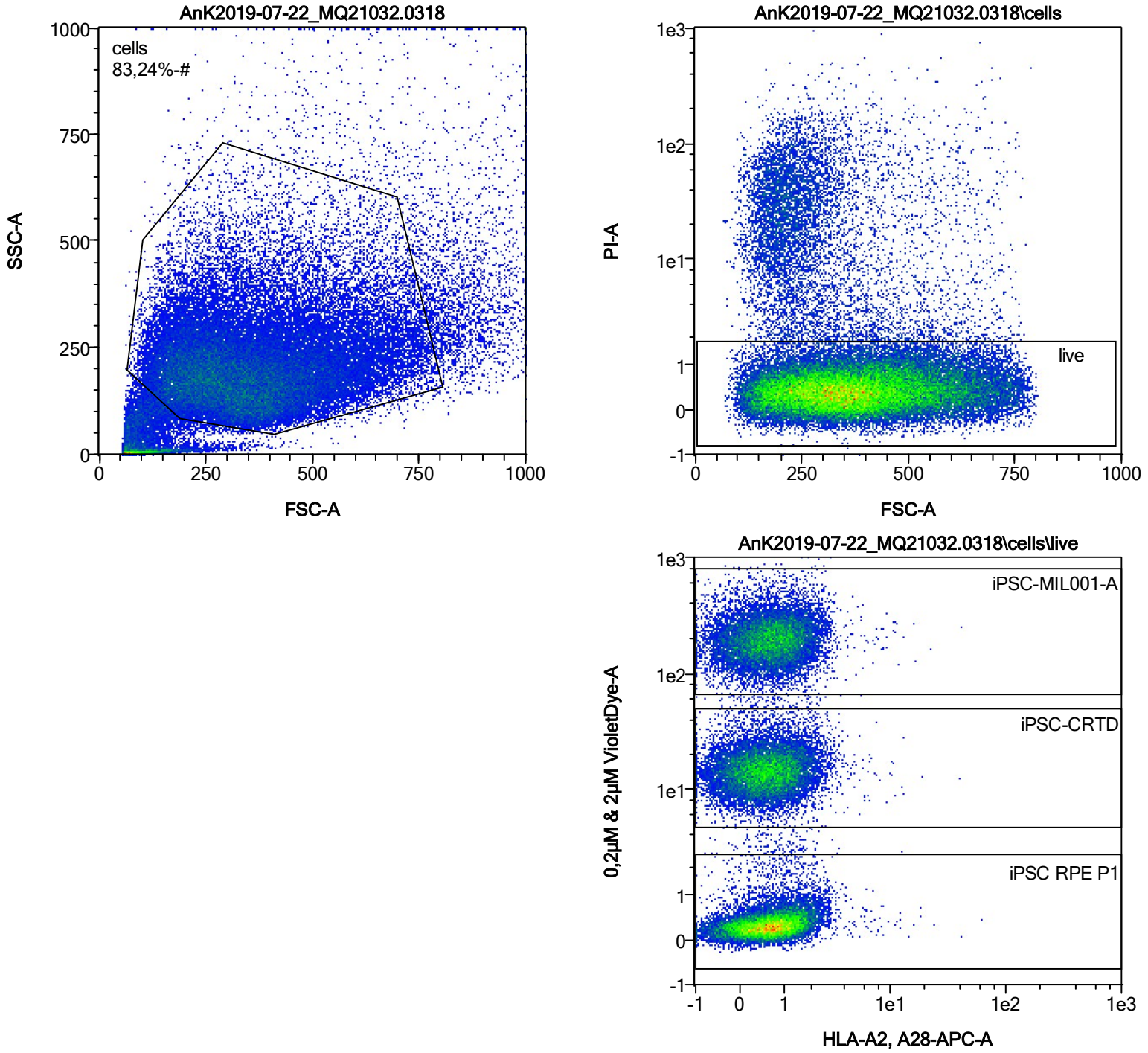

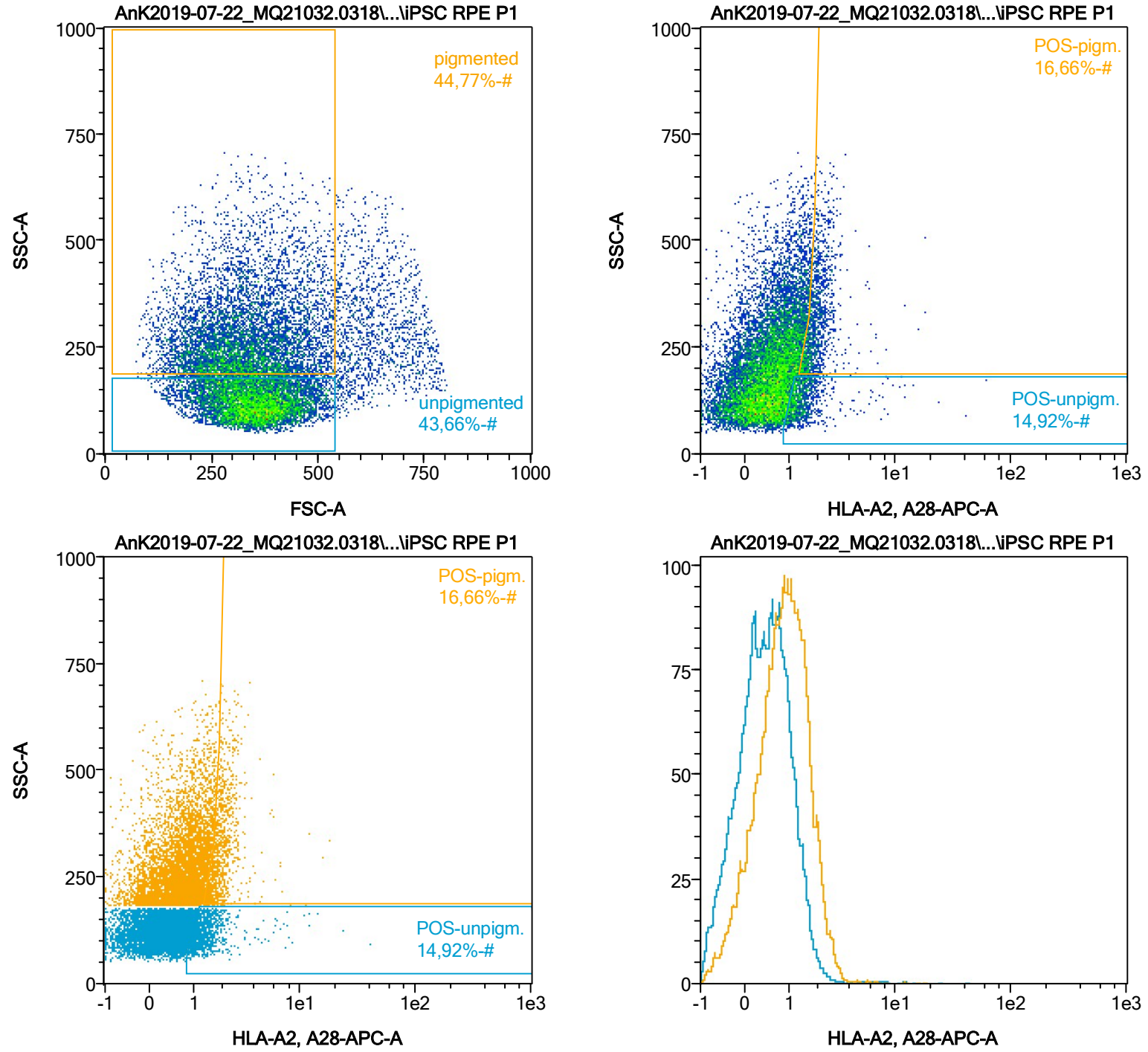

Name	%-#	HLA-A2, A28-APC-A Mean	HLA-A2, A28-APC-A Median
POS-unpigm.	14,92	1,57	1,29
POS-pigm.	16,66	2,05	1,88

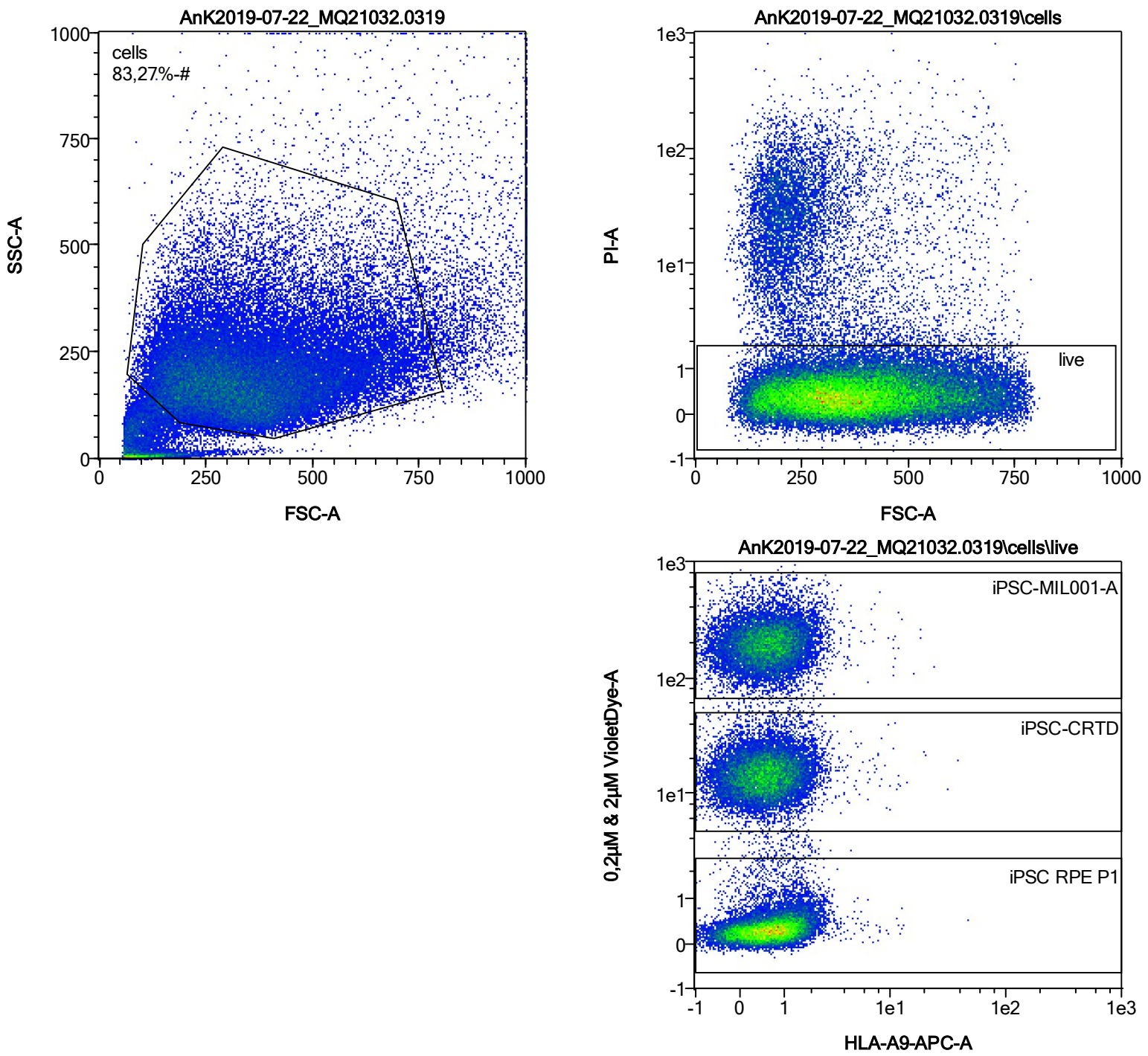

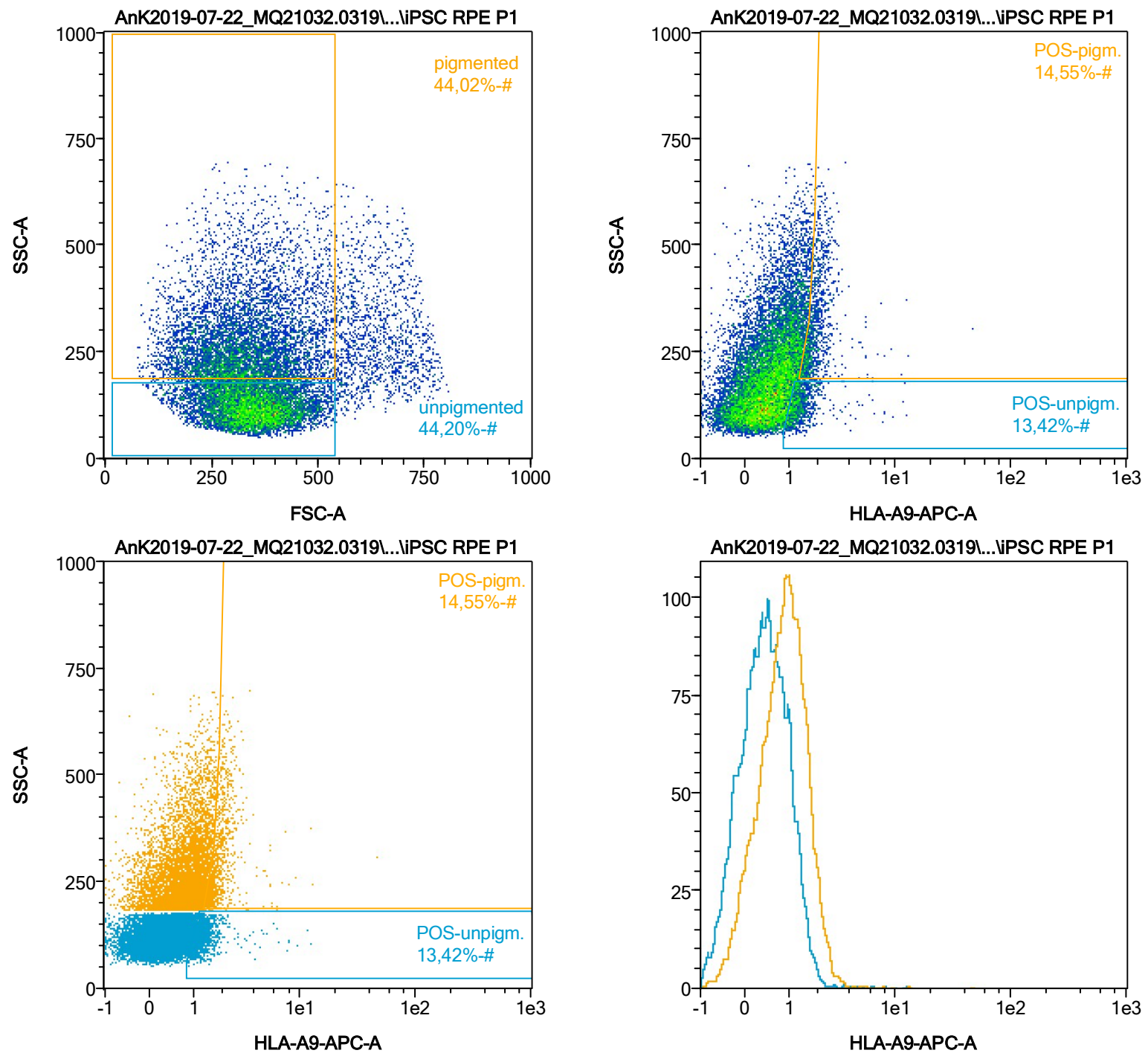

Name	%-#	HLA-A9-APC-A Mean	HLA-A9-APC-A Median
POS-unpigm.	13,42	1,42	1,25
POS-pigm.	14,55	2,06	1,84

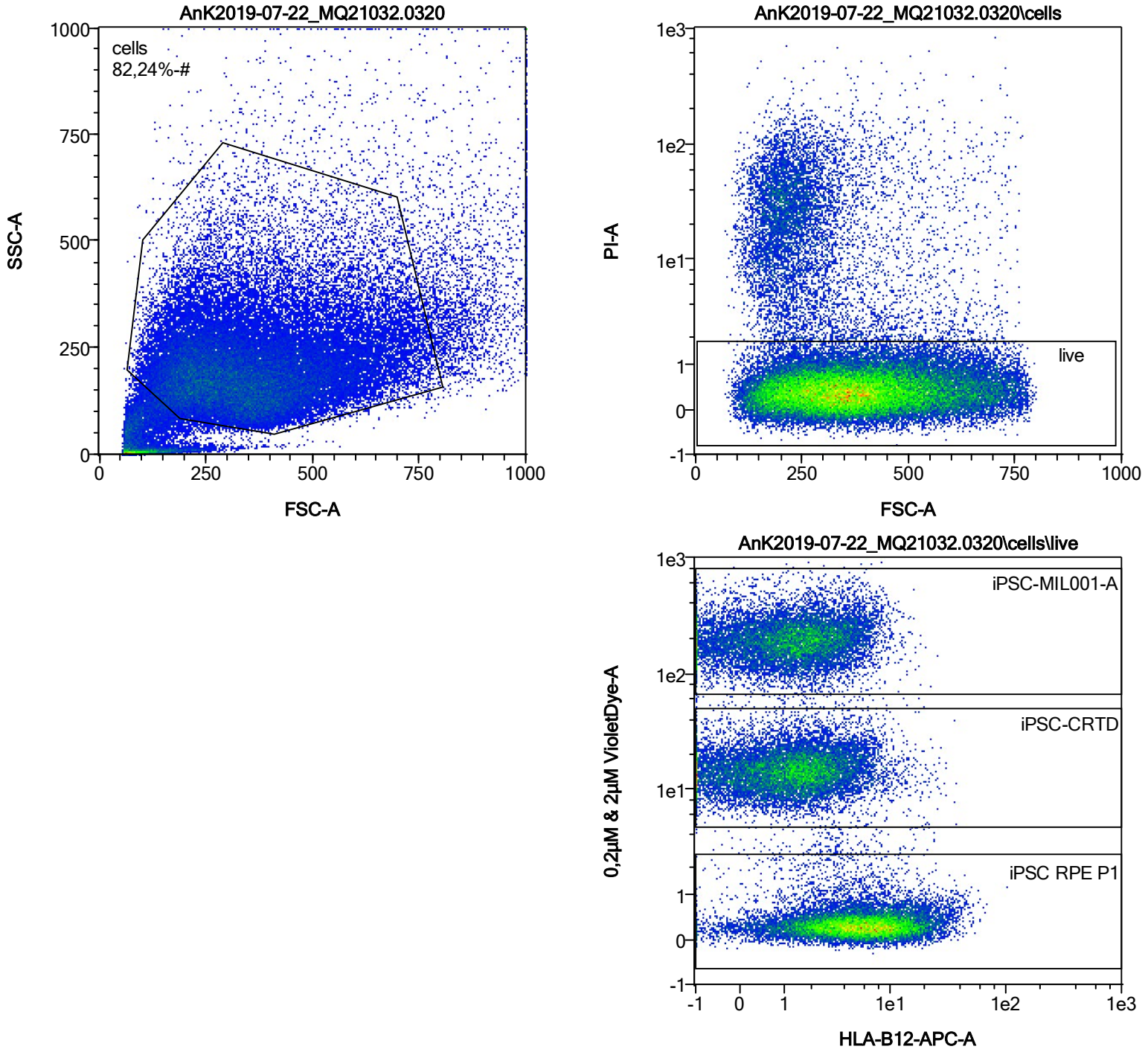

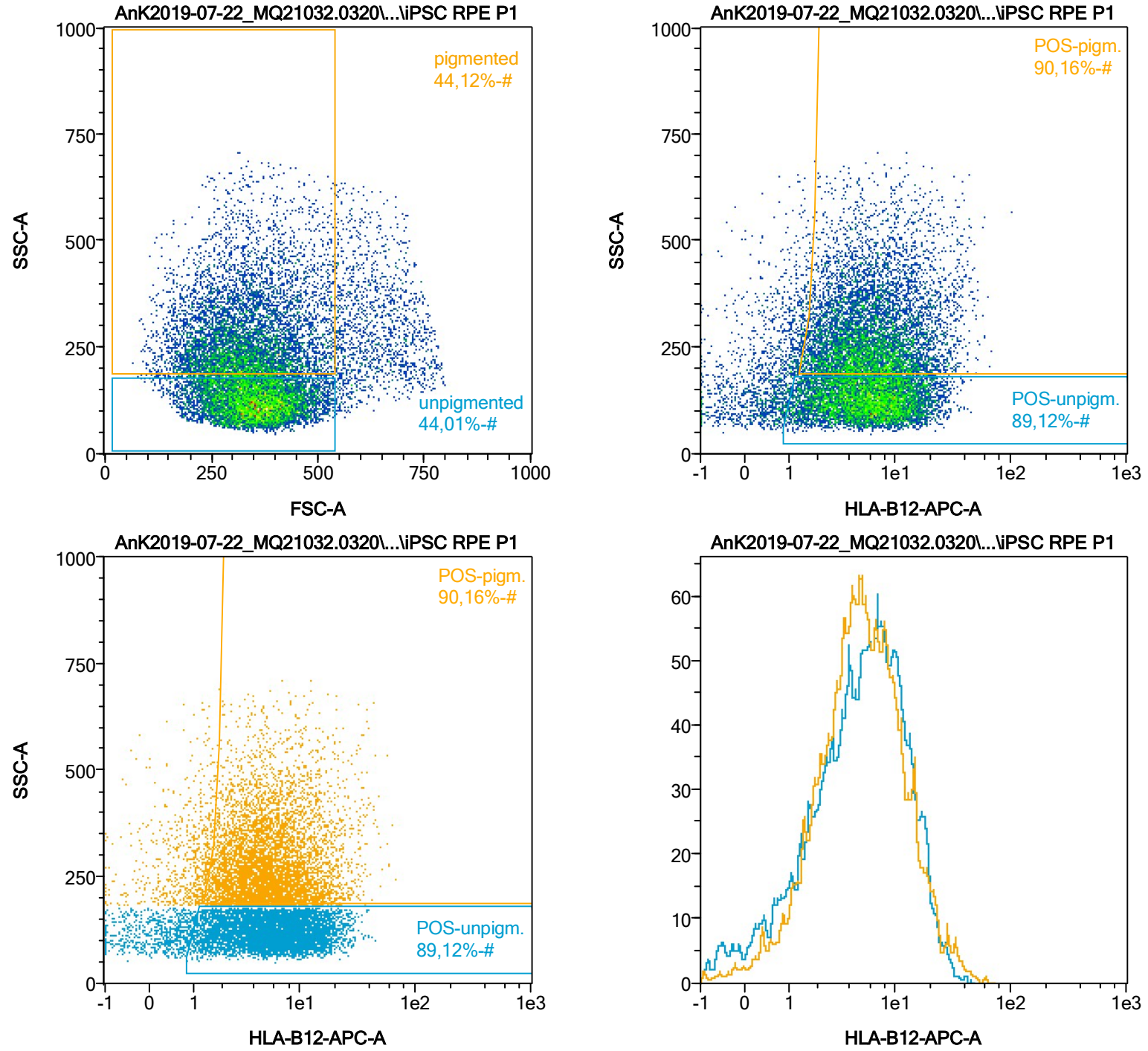

Name	%-#	HLA-B12-APC-A Mean	HLA-B12-APC-A Median
POS-unpigm.	89,12	7,36	5,96
POS-pigm.	90,16	7,41	5,46

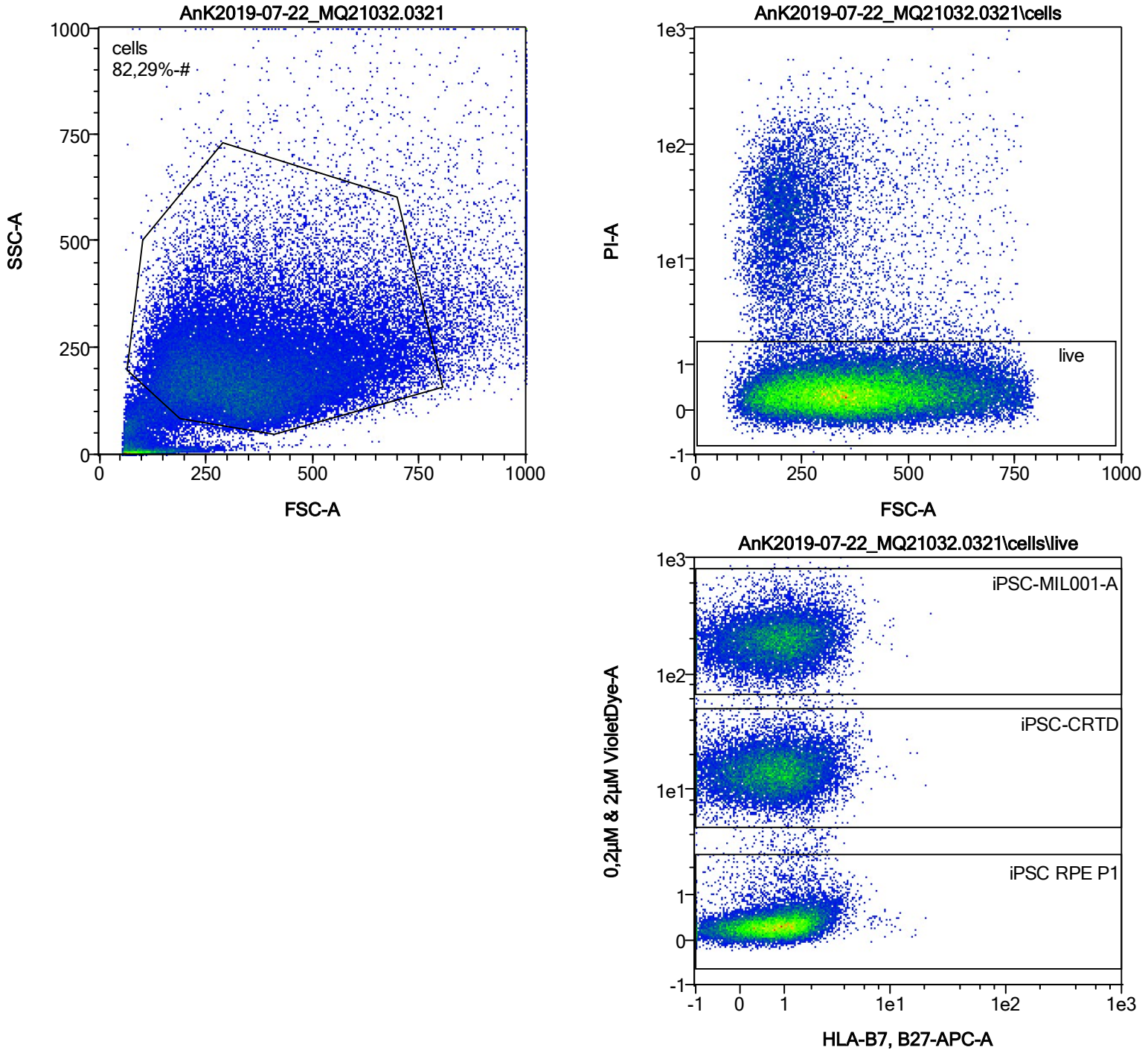

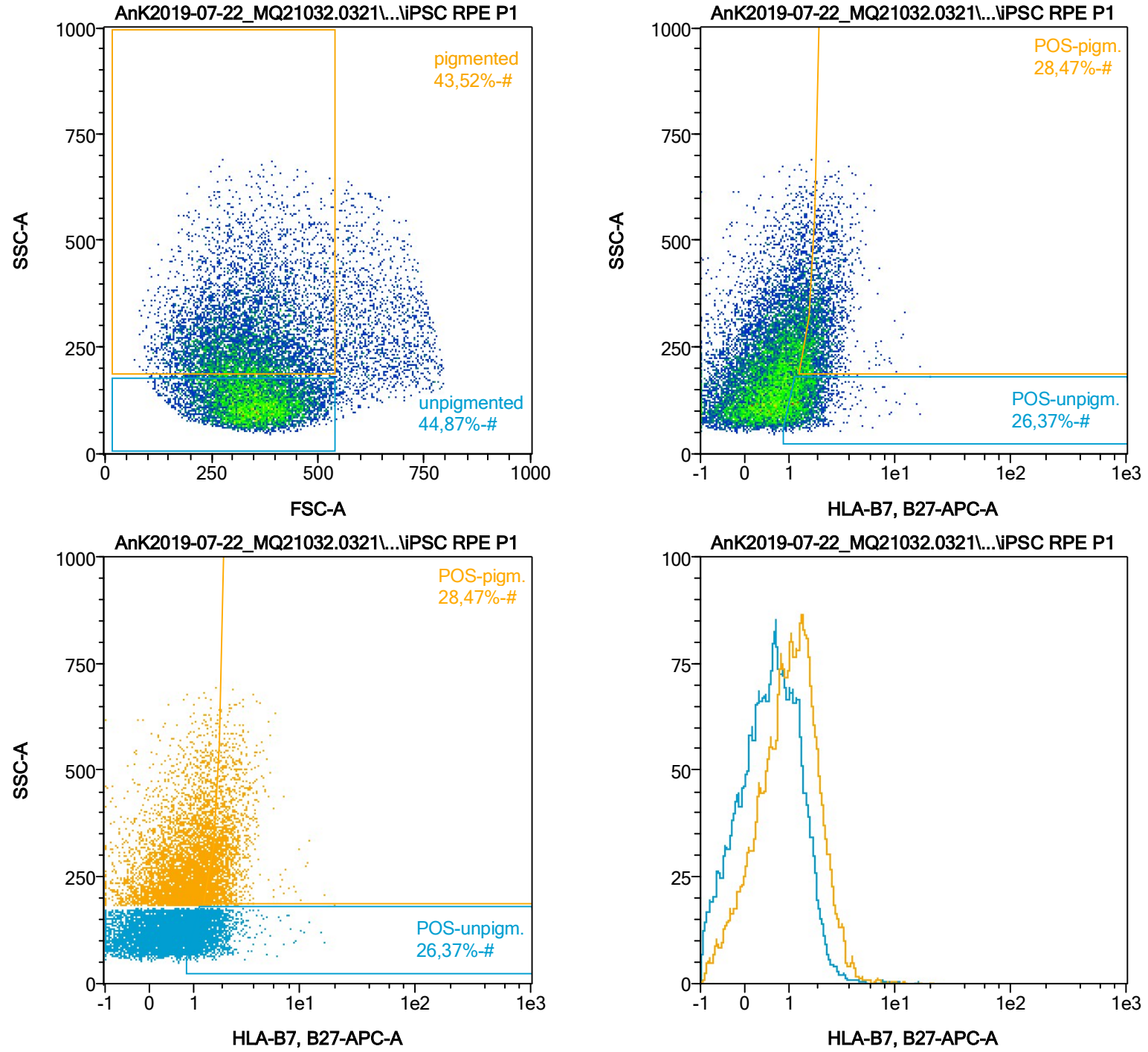

Name	%-#	HLA-B7, B27-APC-A Mean	HLA-B7, B27-APC-A Median
POS-unpigm.	26,37	1,60	1,40
POS-pigm.	28,47	2,25	2,01

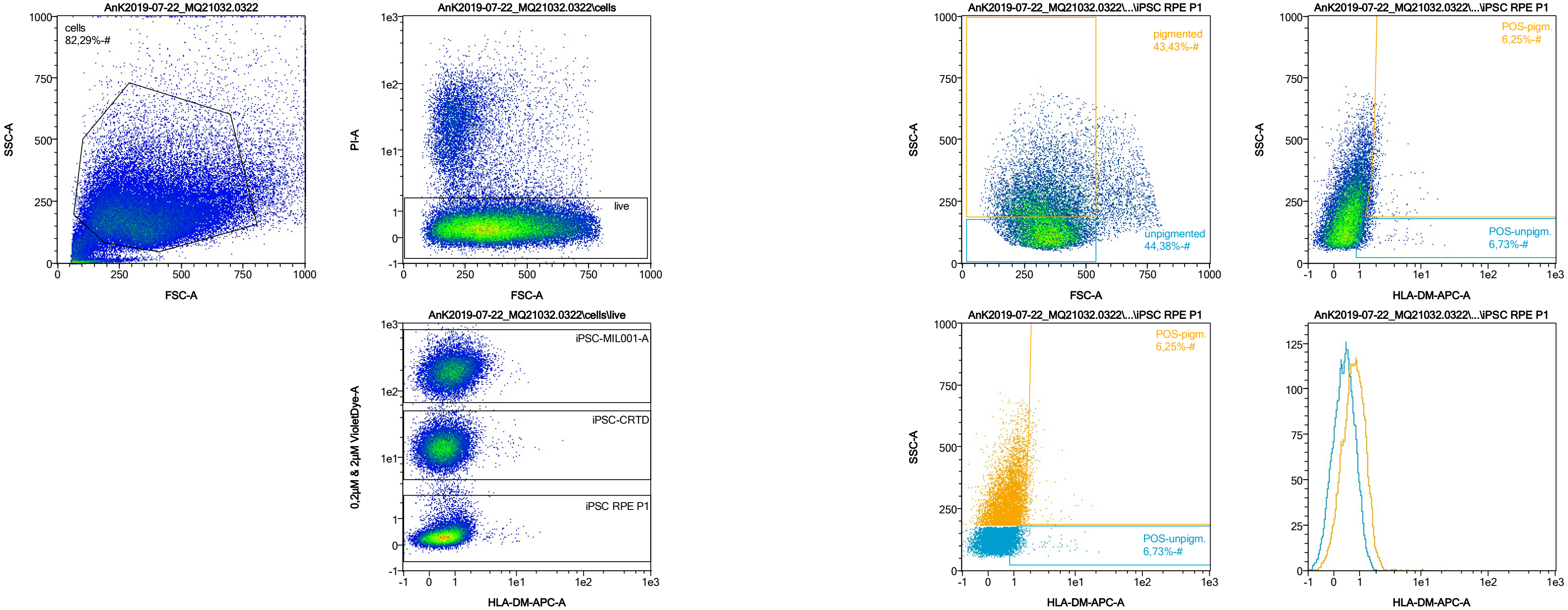

Name	%-#	HLA-DM-APC-A Mean	HLA-DM-APC-A Median
POS-unpigm.	6,73	1,60	1,19
POS-pigm.	6,25	2,13	1,79

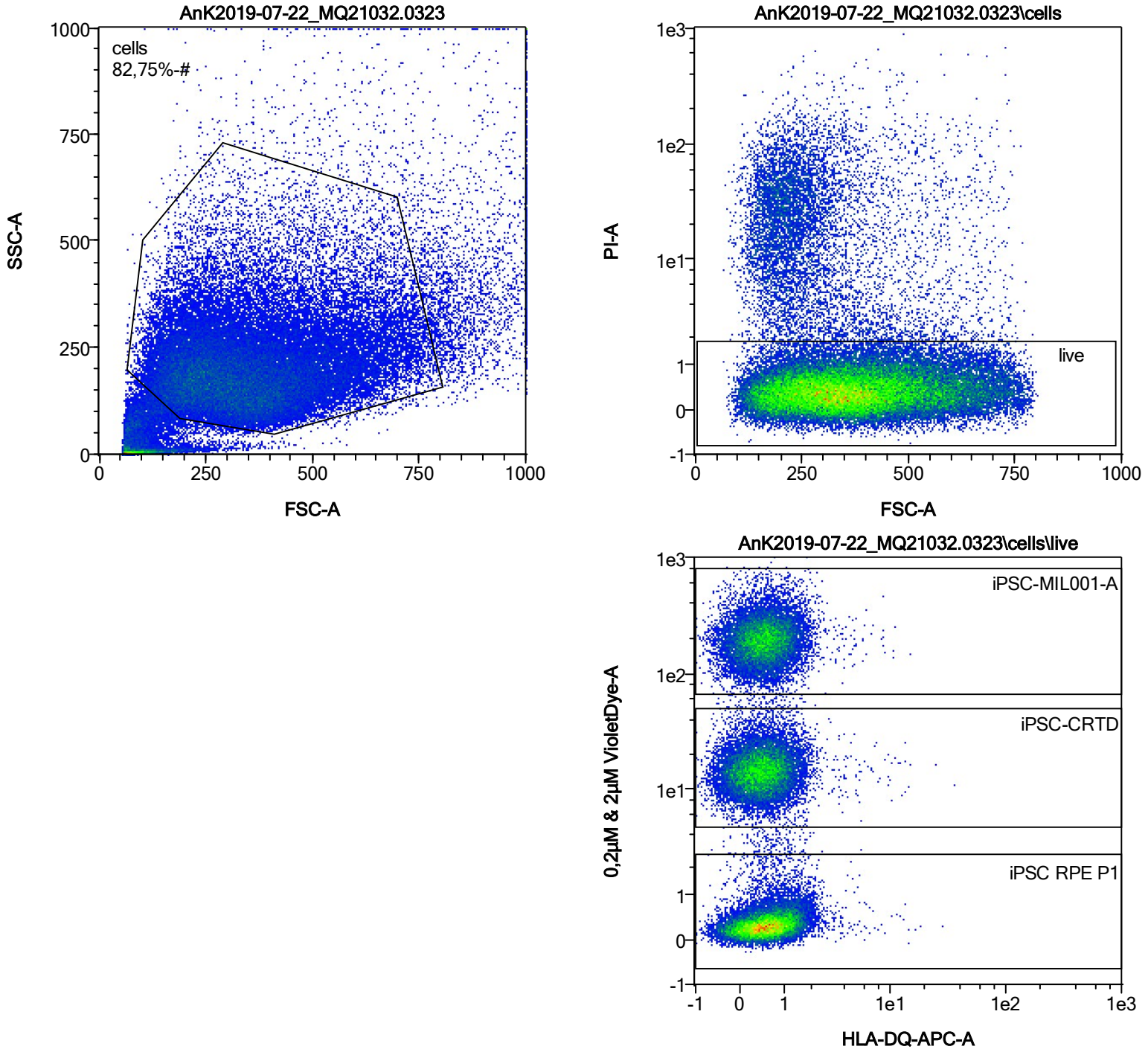

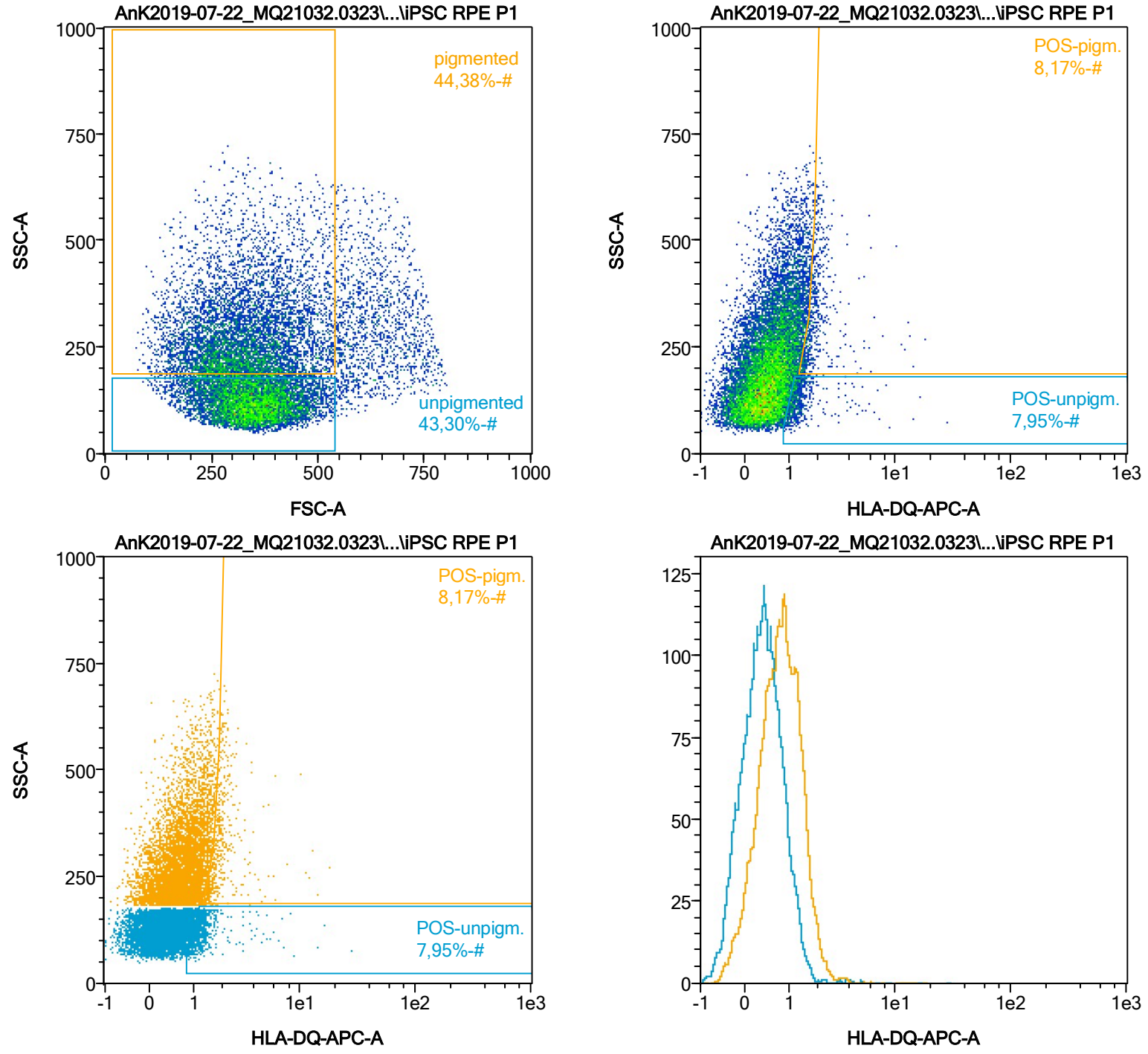

Name	%-#	HLA-DQ-APC-A Mean	HLA-DQ-APC-A Median
POS-unpigm.	7,95	1,54	1,22
POS-pigm.	8,17	2,09	1,82

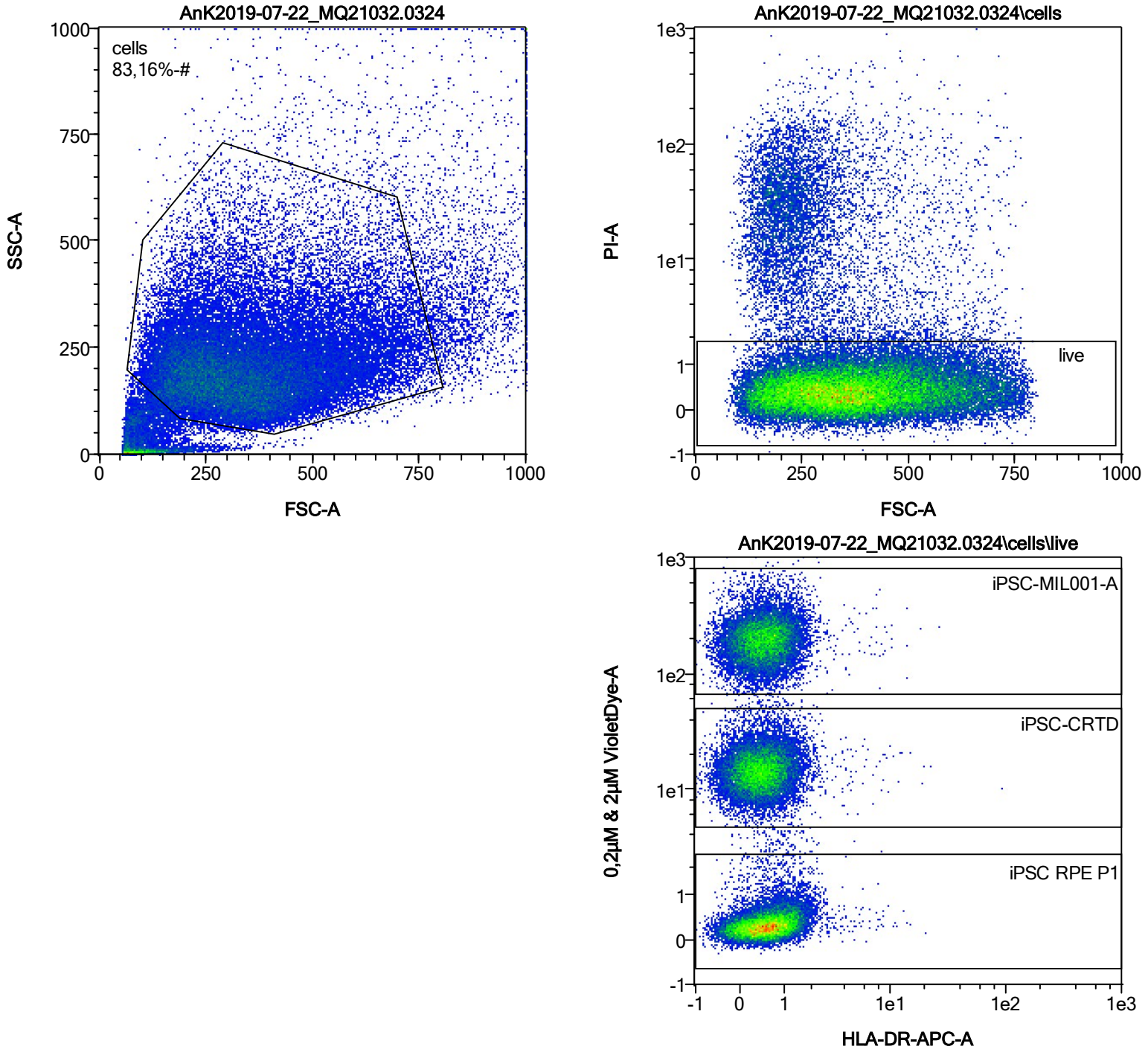

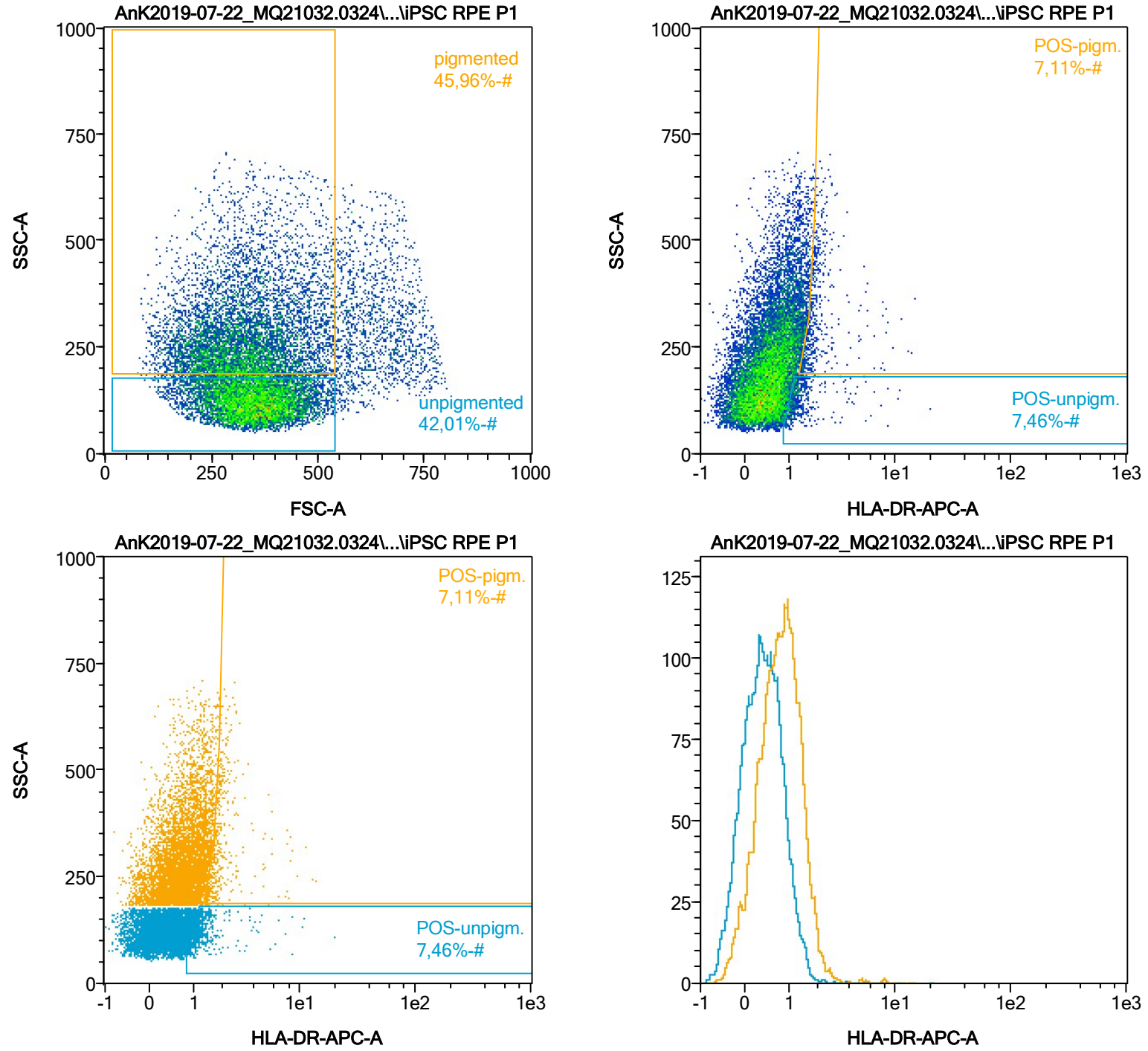

Name	%-#	HLA-DR-APC-A Mean	HLA-DR-APC-A Median
POS-unpigm.	7,46	1,51	1,22
POS-pigm.	7,11	2,16	1,84

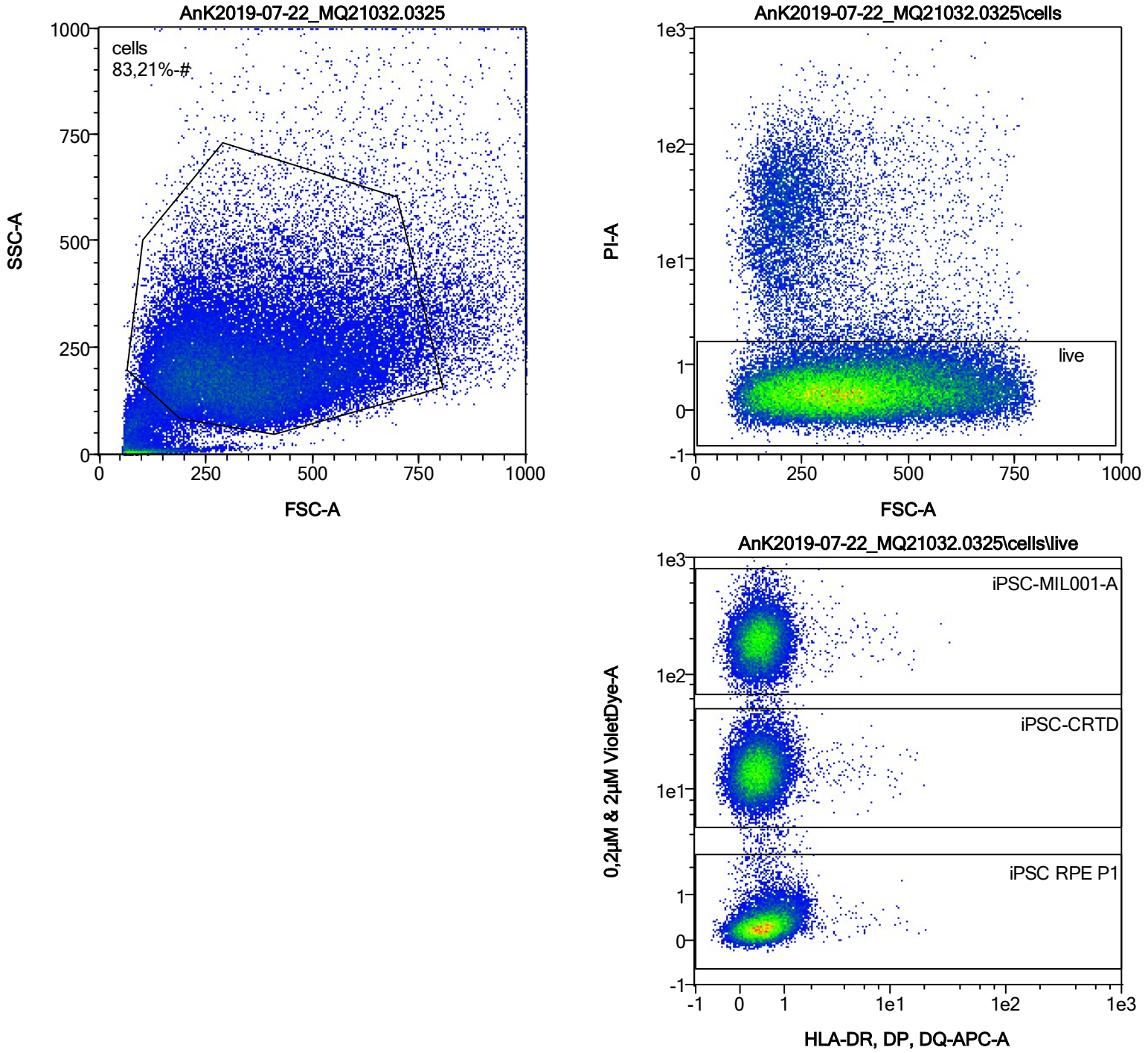

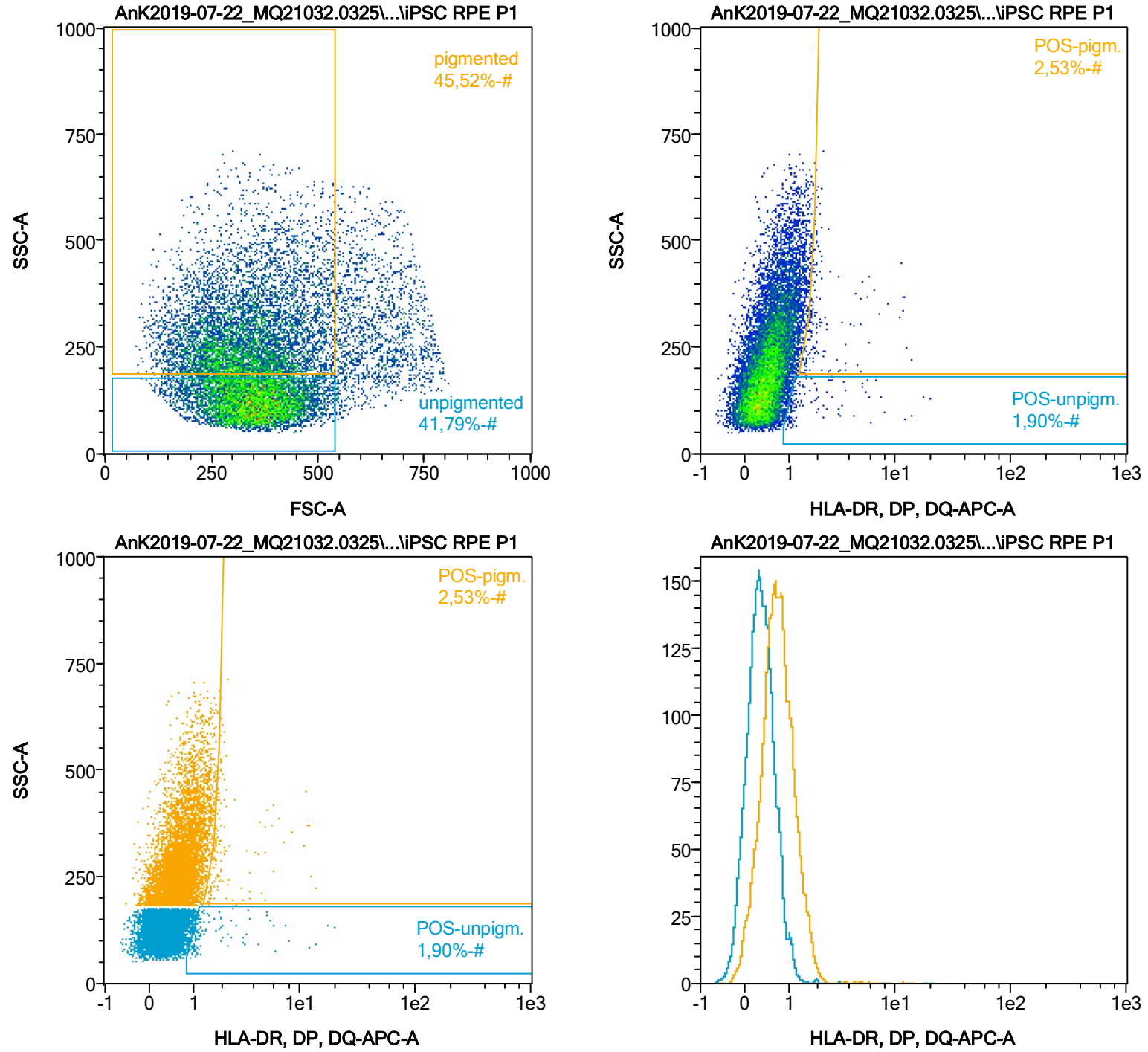

Name	%-#	HLA-DR, DP, DQ-APC-A Mean	HLA-DR, DP, DQ-APC-A Median
POS-unpigm.	1,90	2,26	1,18
POS-pigm.	2,53	2,57	1,84

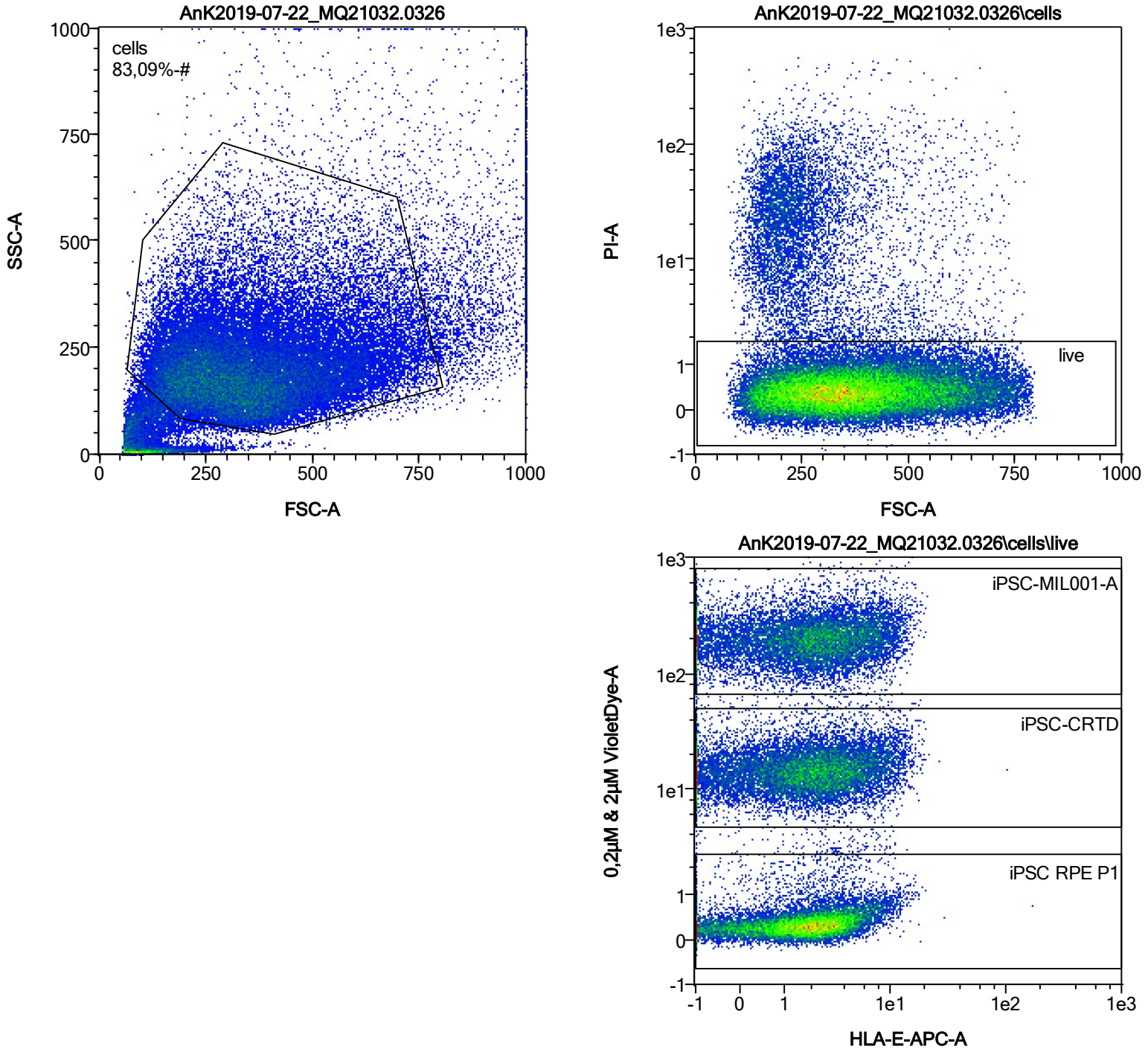

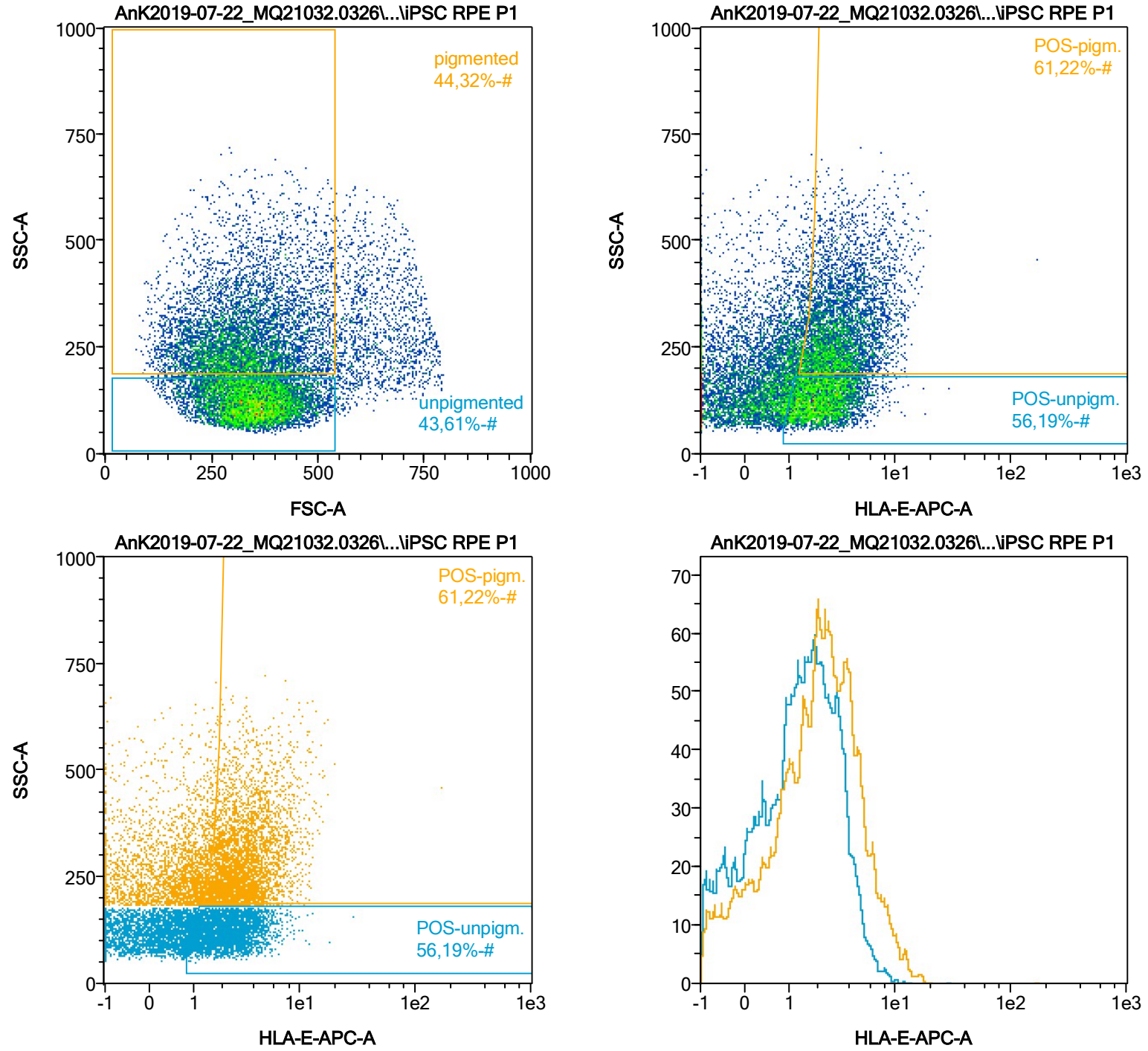

Name	%-#	HLA-E-APC-A Mean	HLA-E-APC-A Median
POS-unpigm.	56,19	2,48	2,15
POS-pigm.	61,22	3,73	3,07

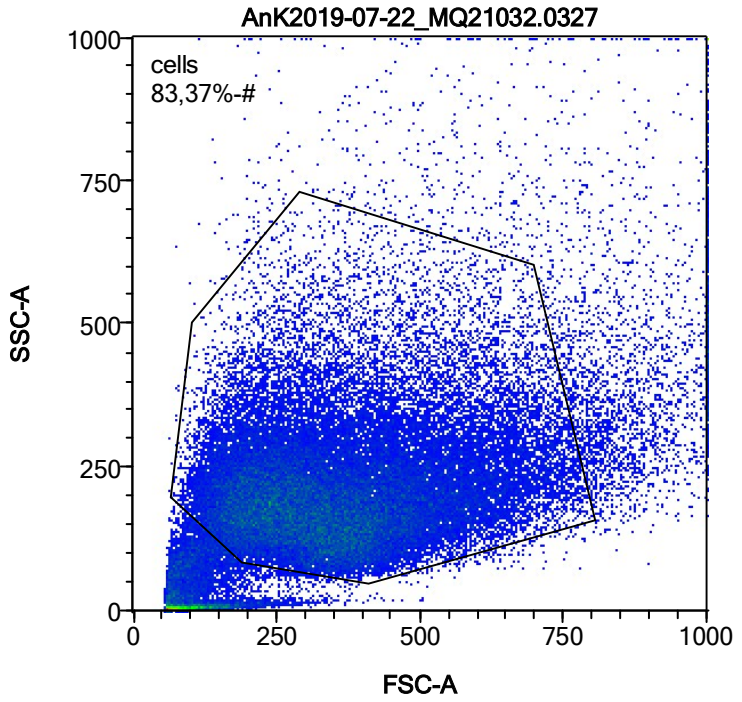

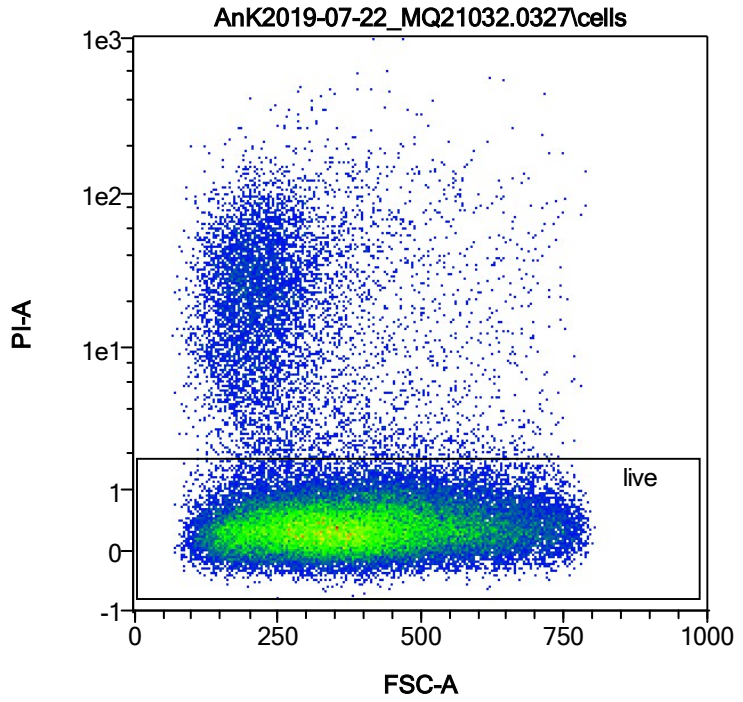

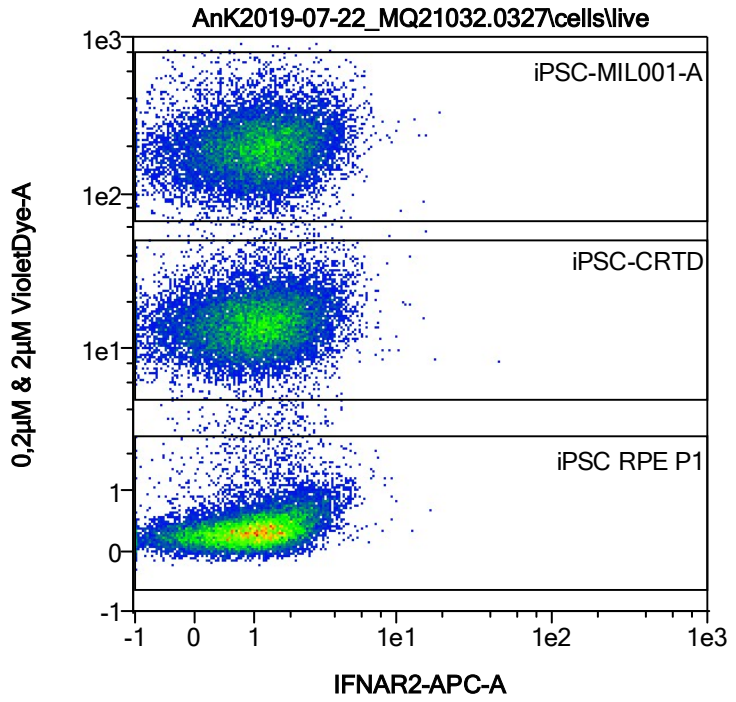

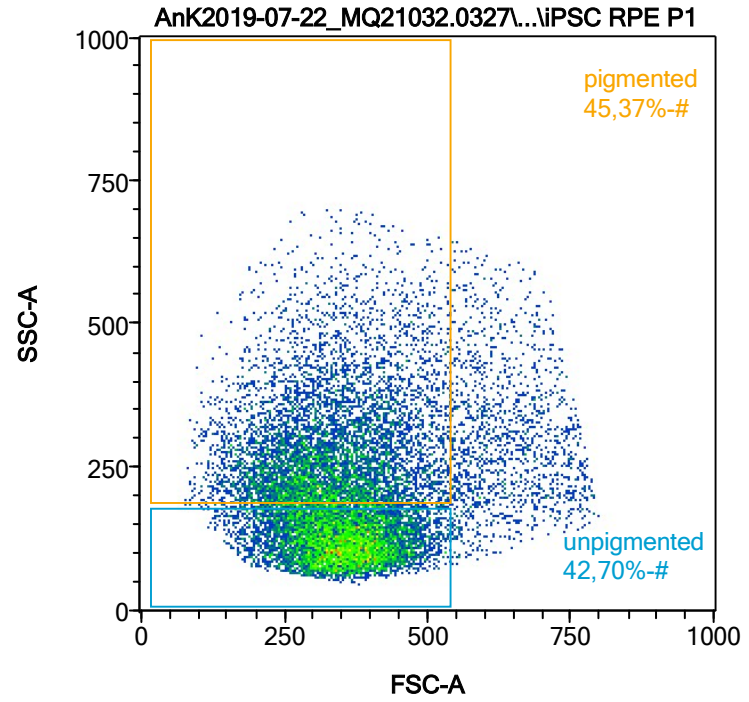

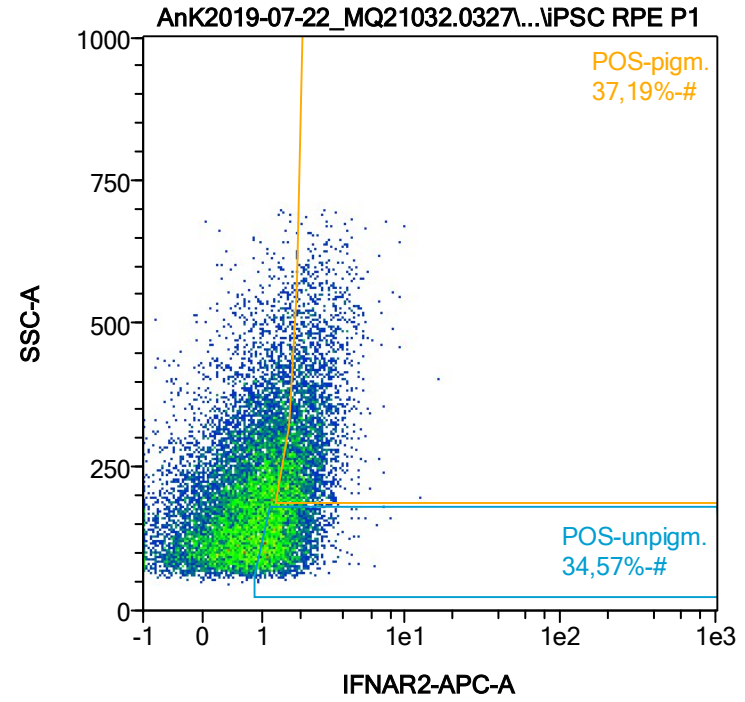

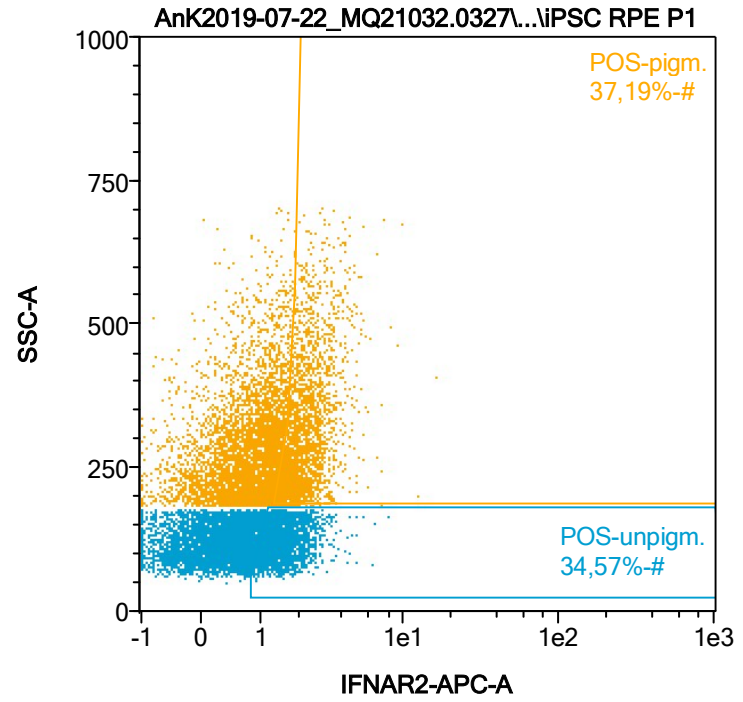

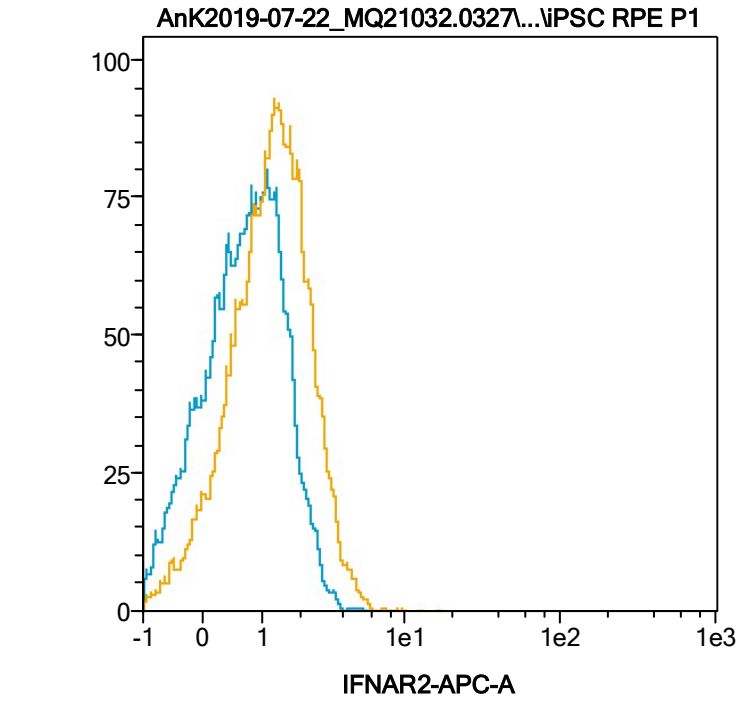

Name	%-#	IFNAR2-APC-A Mean	IFNAR2-APC-A Median
POS-unpigm.	34,57	1,57	1,42
POS-pigm.	37,19	2,30	2,08

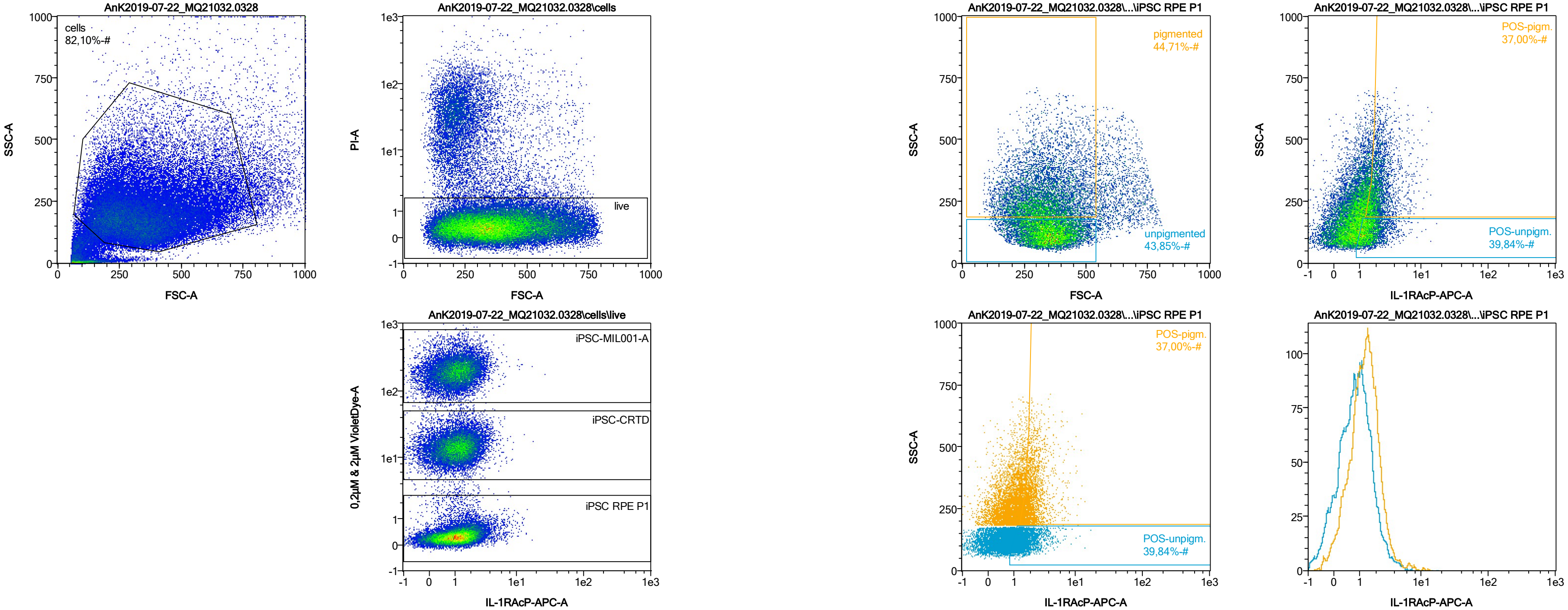

Name	%-#	IL-1RAcP-APC-A Mean	IL-1RAcP-APC-A Median
POS-unpigm.	39,84	1,70	1,47
POS-pigm.	37,00	2,32	2,02

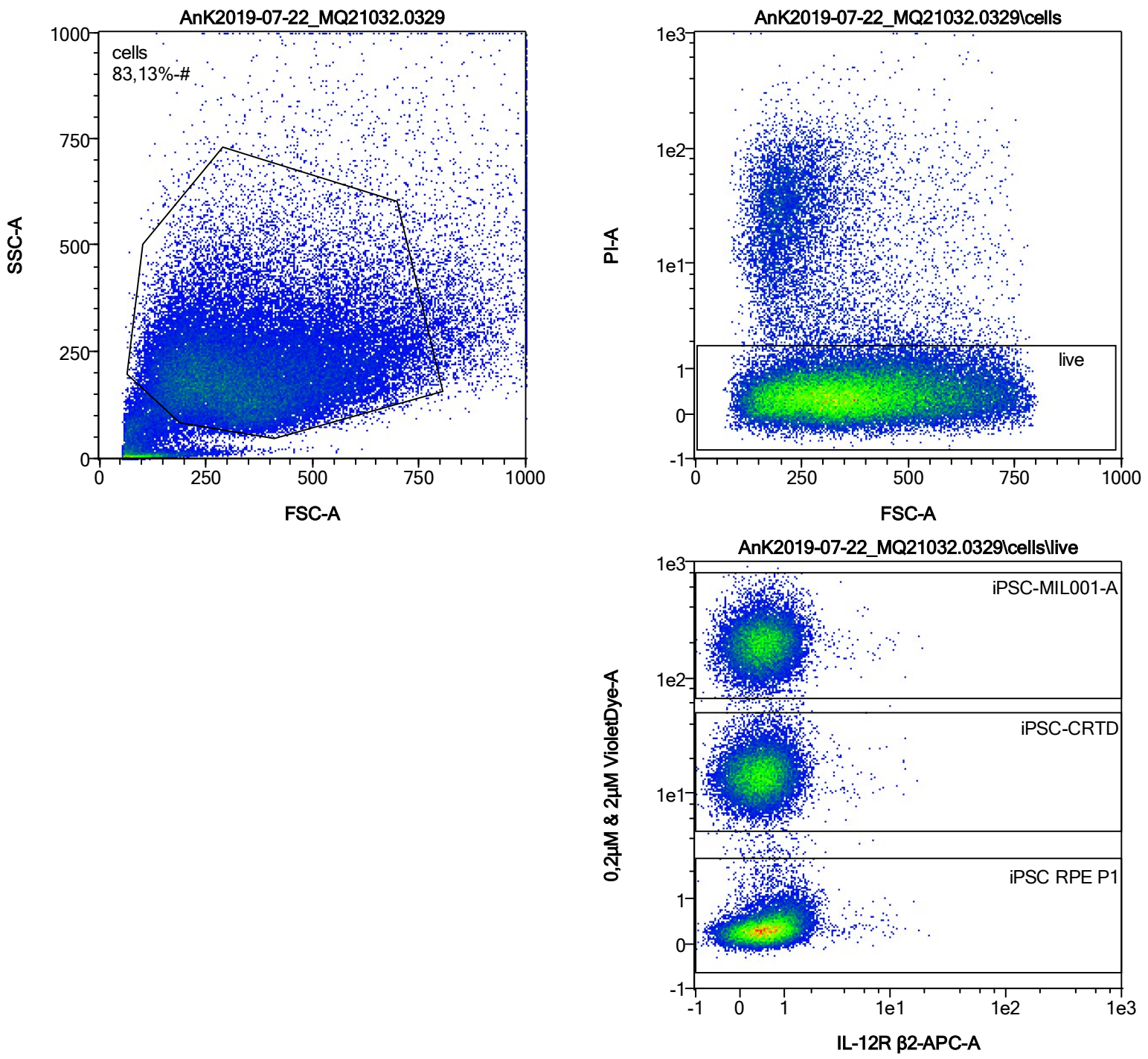

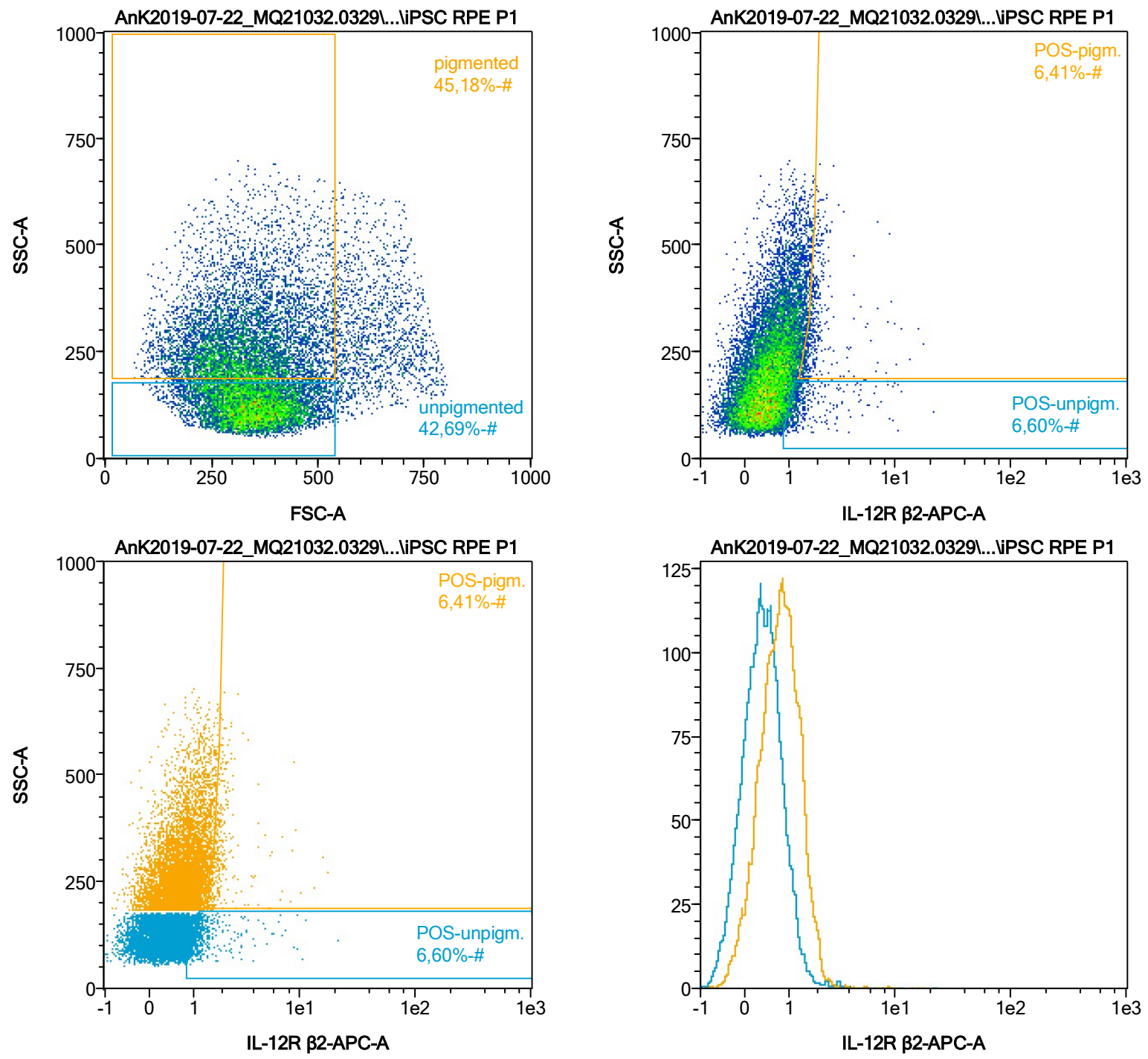

Name	%-#	IL-12R β2-APC-A Mean	IL-12R β2-APC-A Median
POS-unpigm.	6,60	1,63	1,21
POS-pigm.	6,41	2,13	1,81

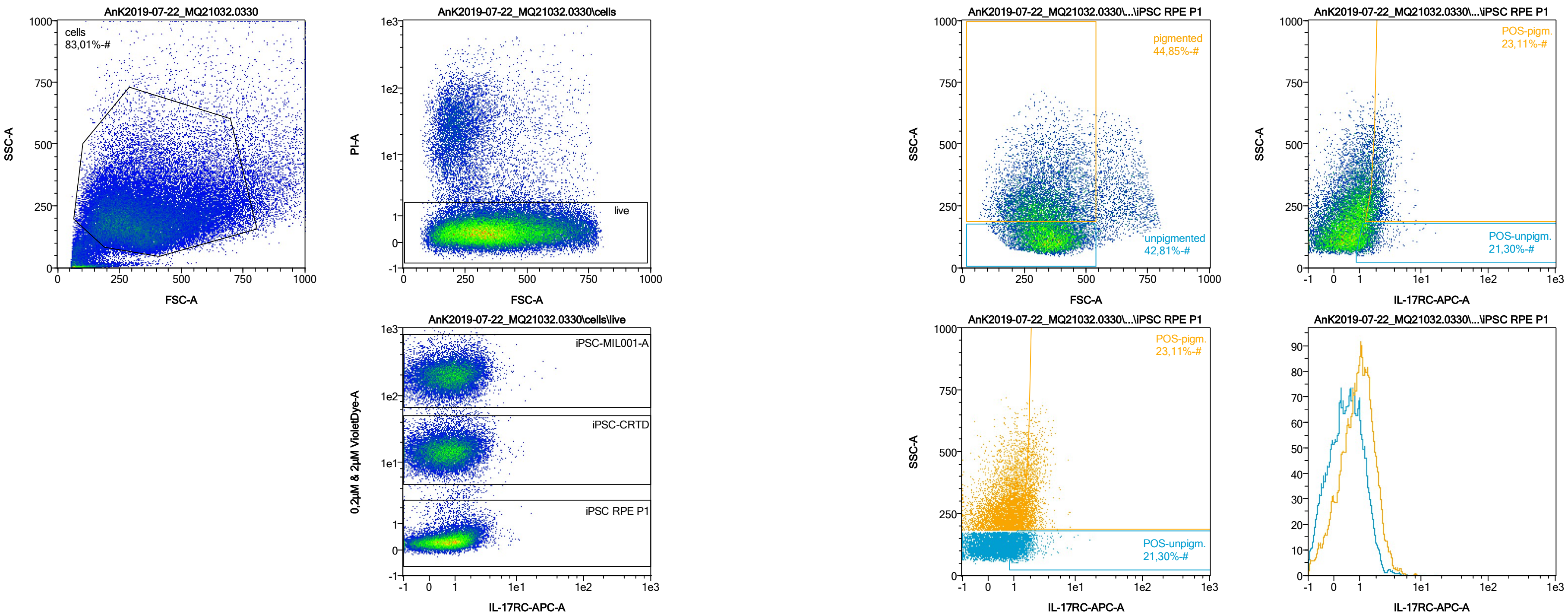

Name	%-#	IL-17RC-APC-A Mean	IL-17RC-APC-A Median
POS-unpigm.	21,30	1,53	1,35
POS-pigm.	23,11	2,16	1,99

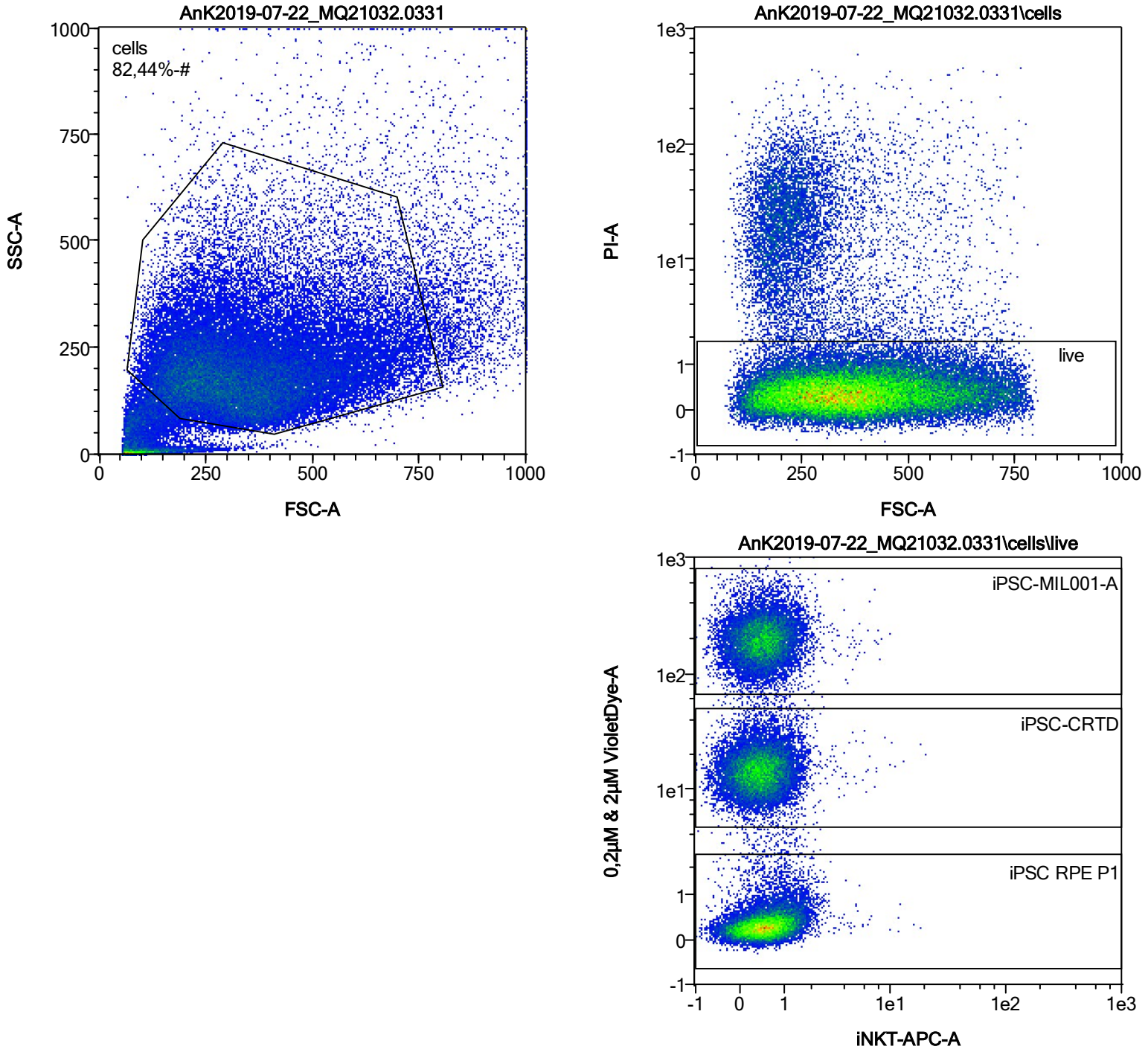

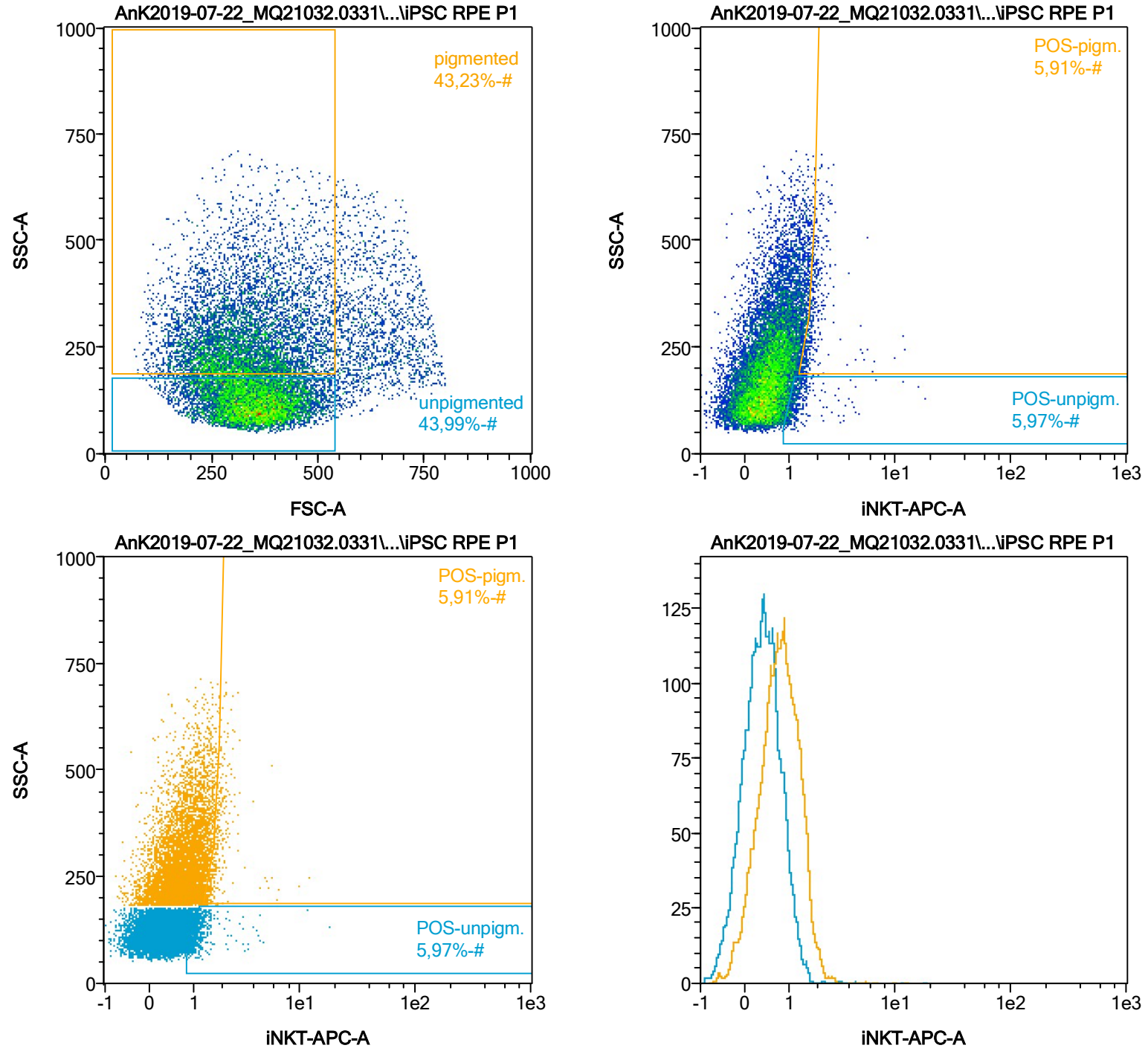

Name	%-#	iNKT-APC-A Mean	iNKT-APC-A Median
POS-unpigm.	5,97	1,36	1,17
POS-pigm.	5,91	1,94	1,77

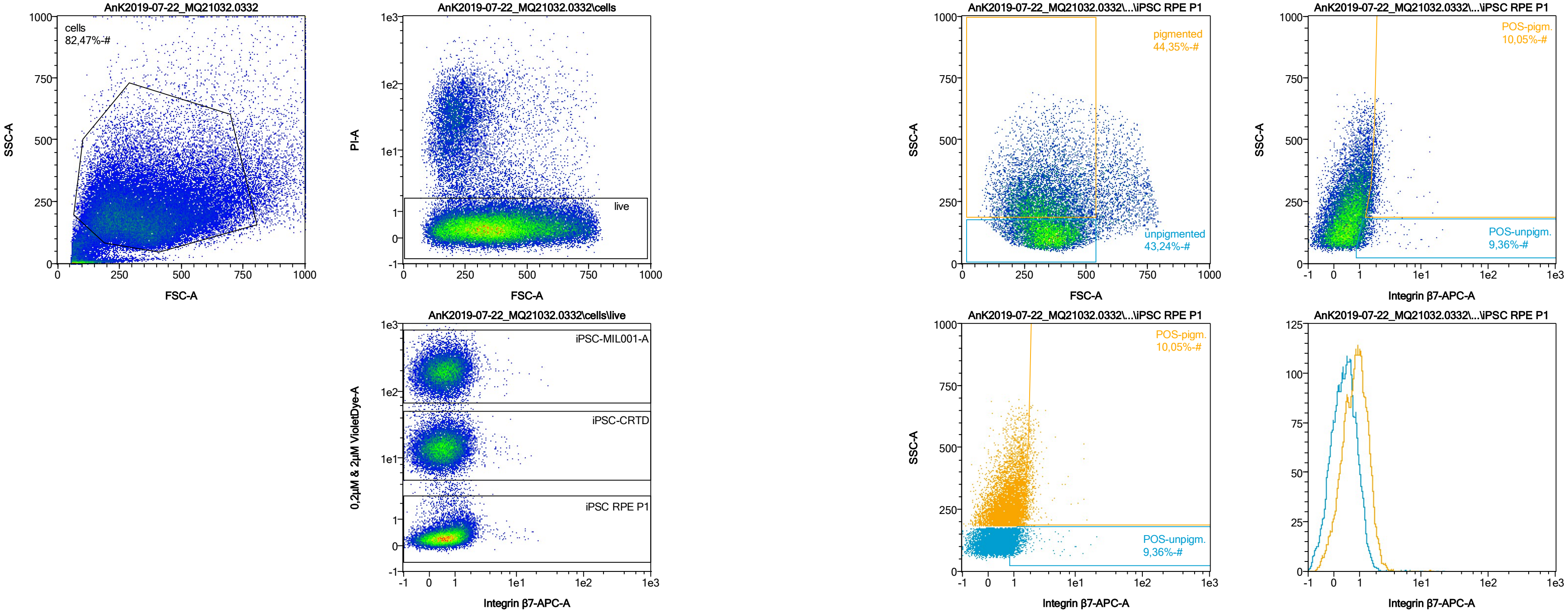

Name	%-#	Integrin β7-APC-A Mean	Integrin β7-APC-A Median
POS-unpigm.	9,36	1,49	1,21
POS-pigm.	10,05	1,99	1,79

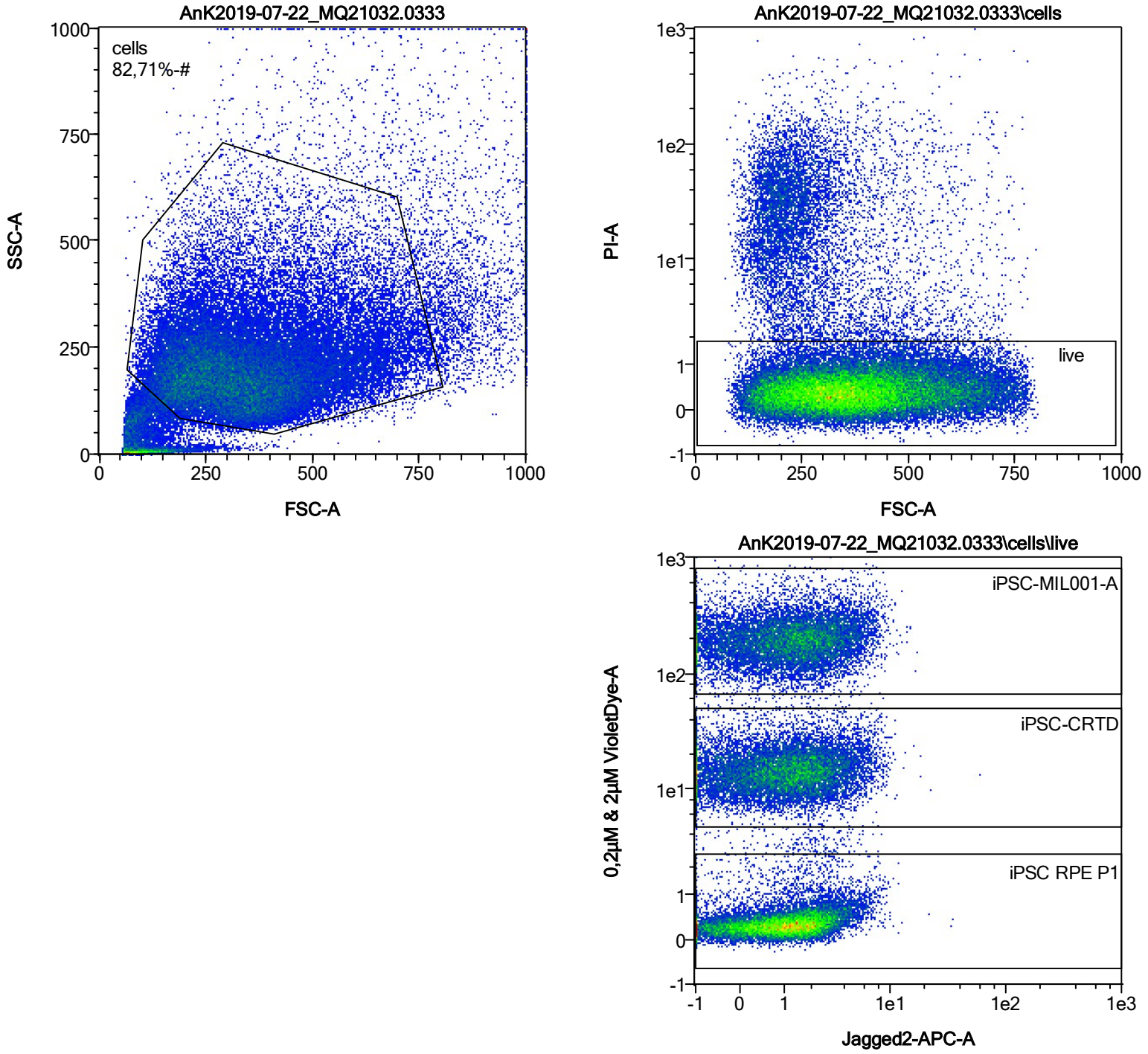

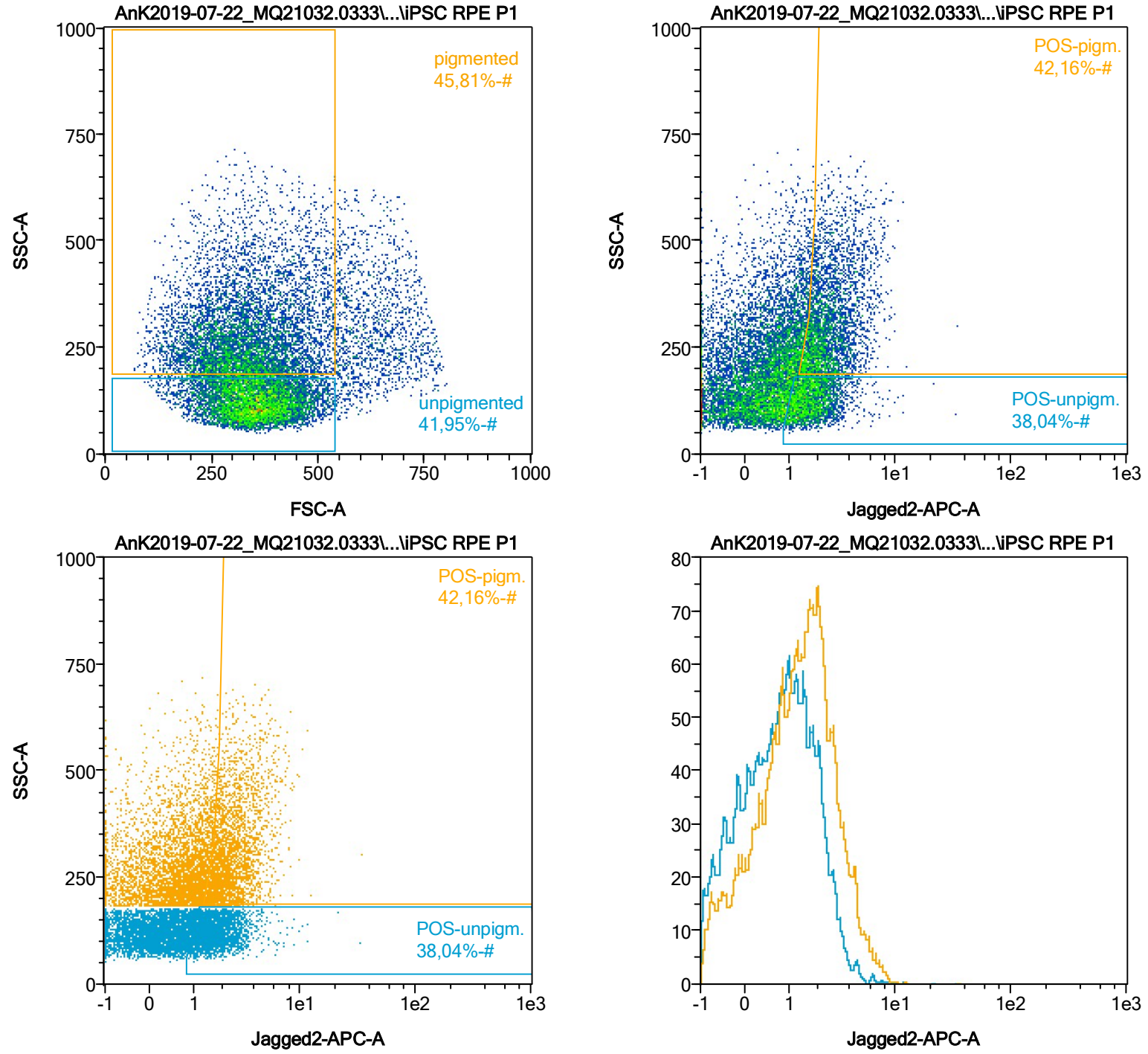

Name	%-#	Jagged2-APC-A Mean	Jagged2-APC-A Median
POS-unpigm.	38,04	1,91	1,67
POS-pigm.	42,16	2,71	2,34

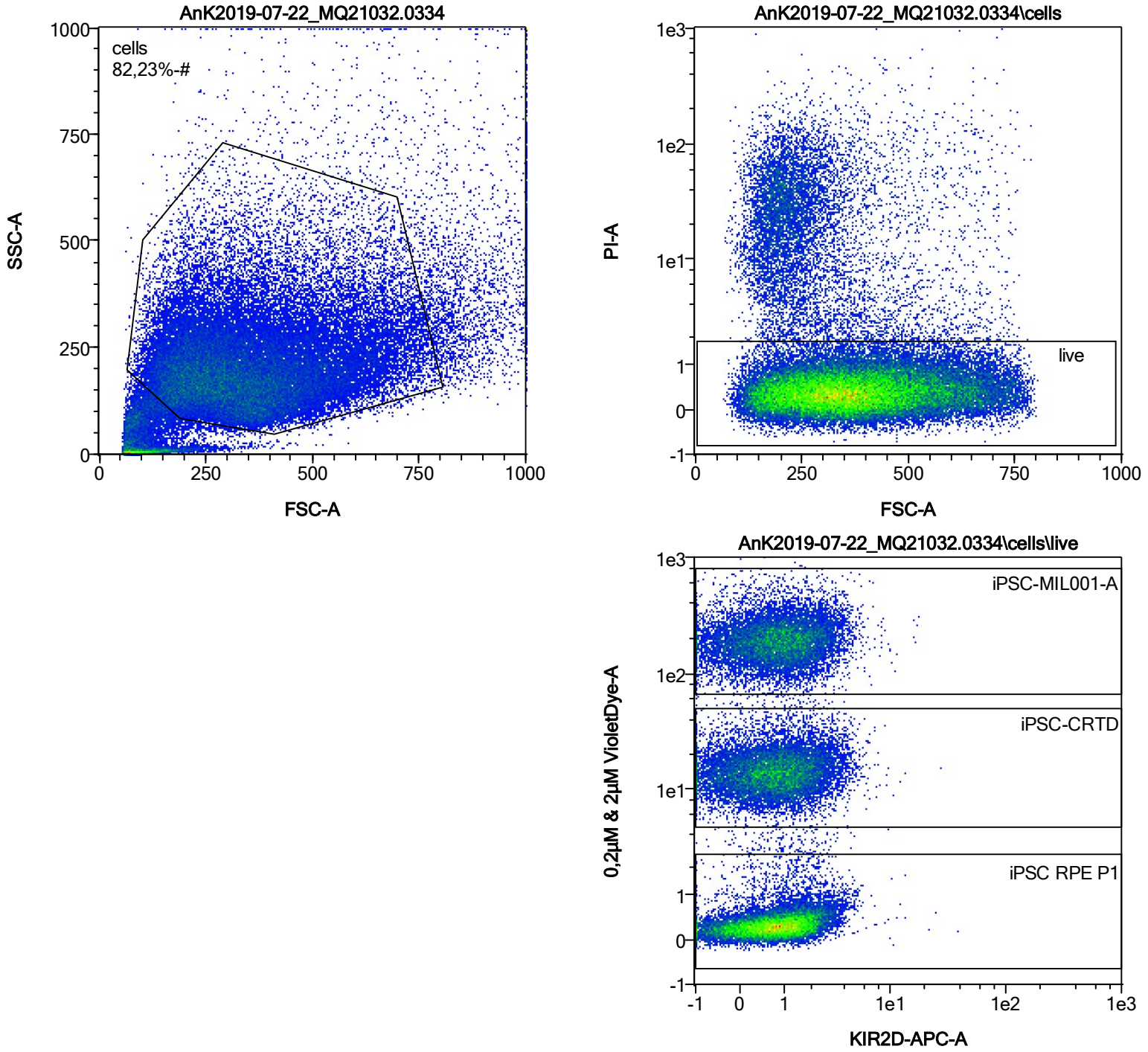

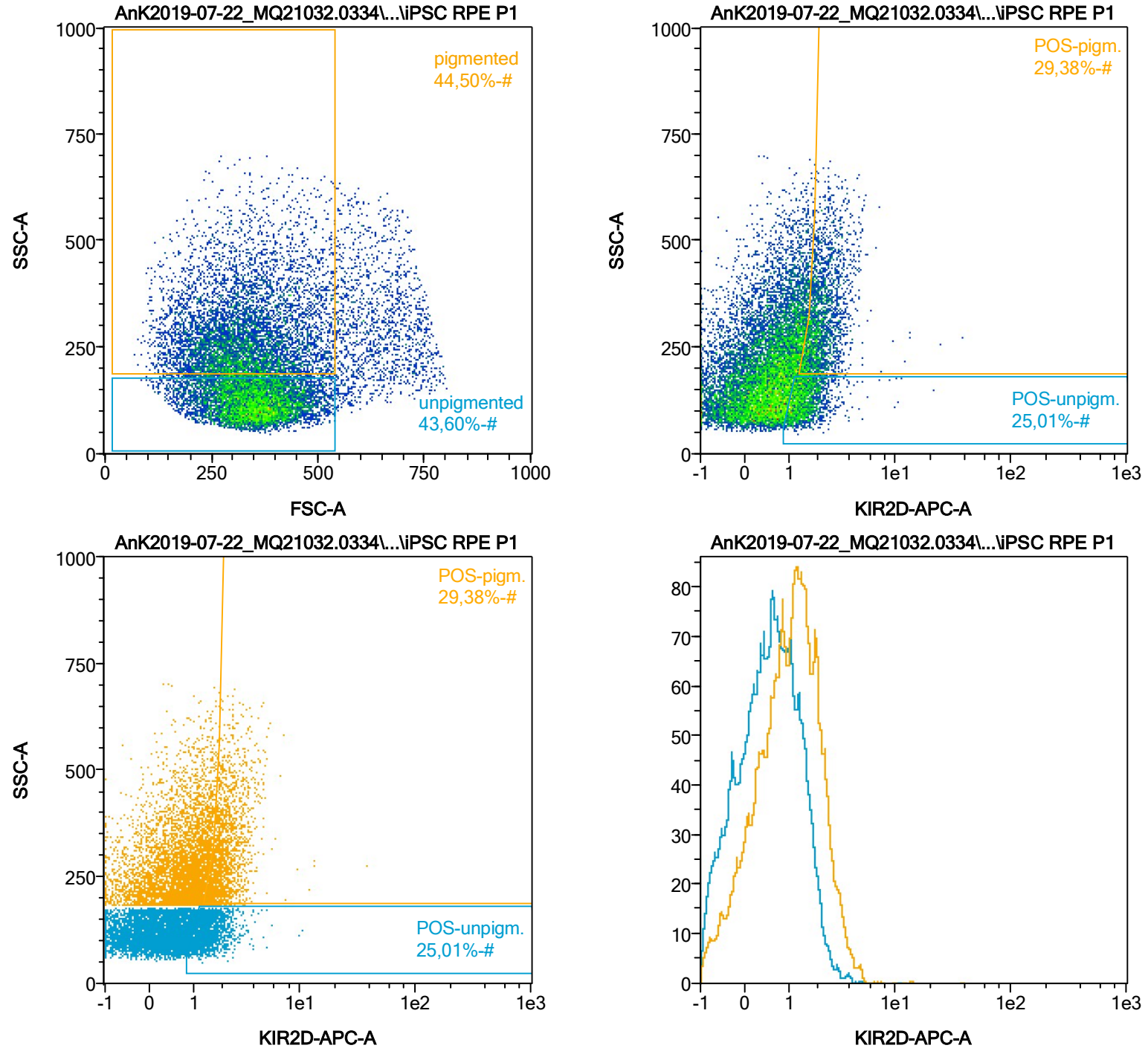

Name	%-#	KIR2D-APC-A Mean	KIR2D-APC-A Median
POS-unpigm.	25,01	1,55	1,42
POS-pigm.	29,38	2,27	2,05

Date:2024-06-14

Operator: Sebastian Knoebel

Version:2.13.0+54.g95abf0f4

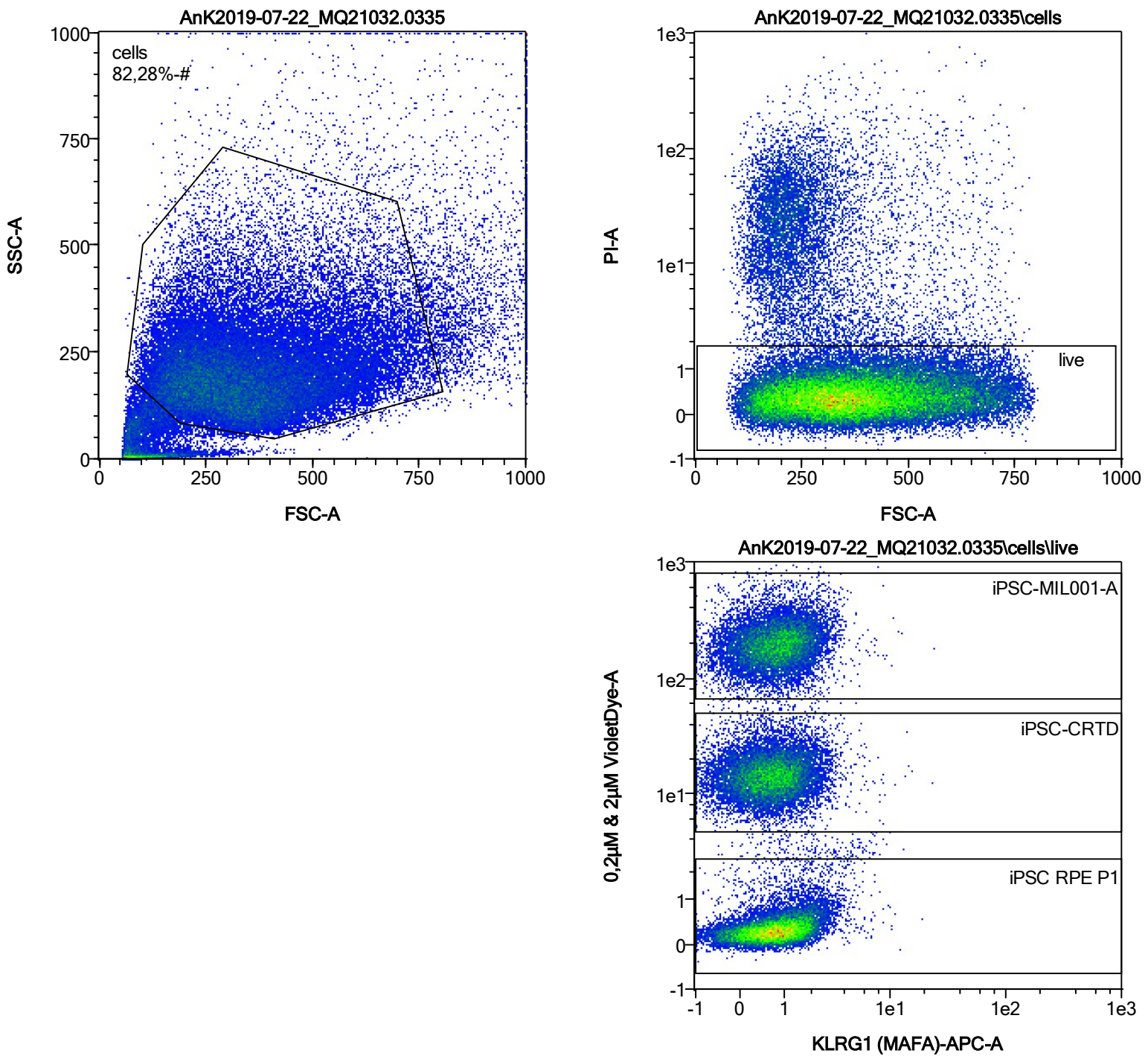

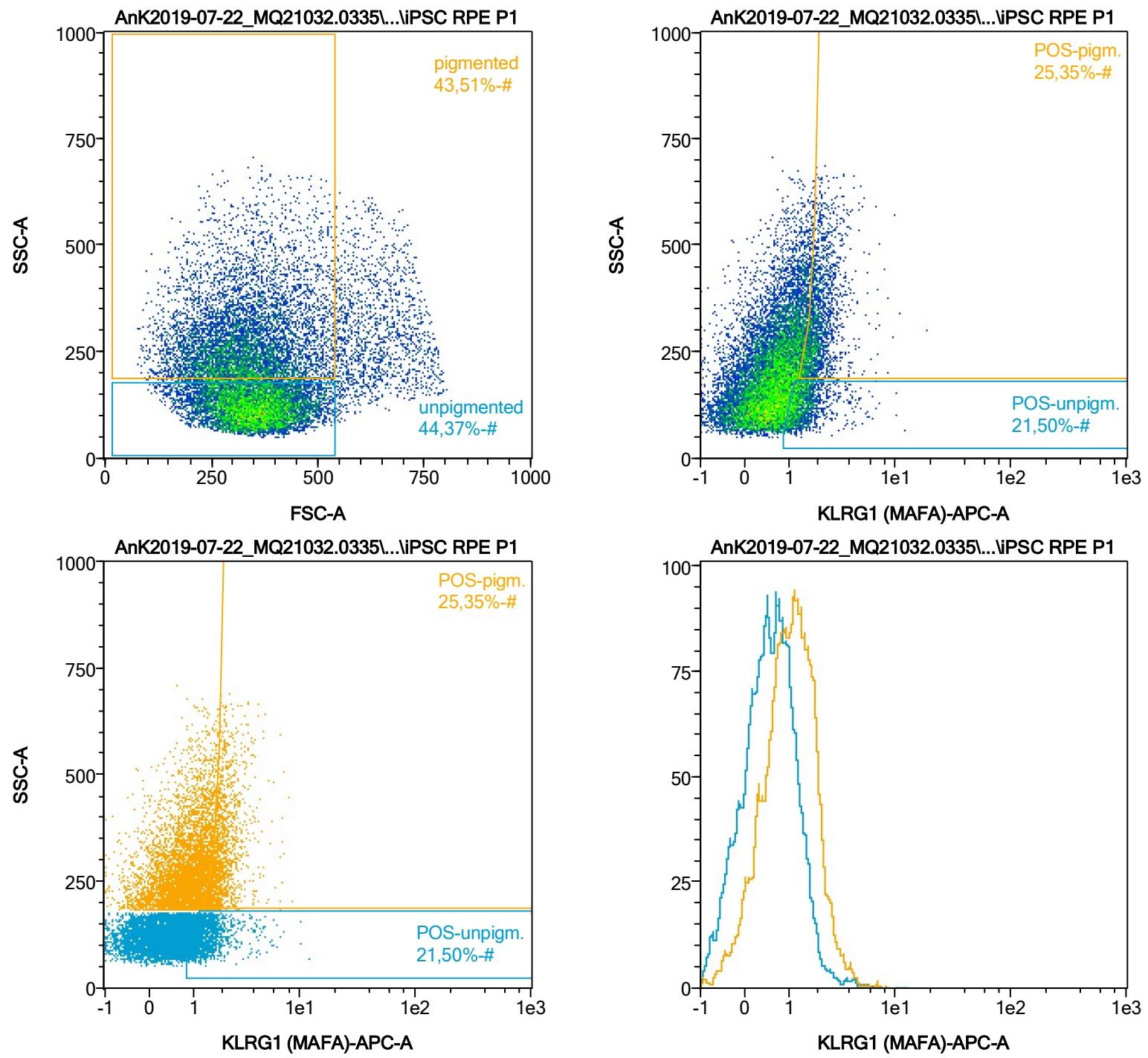

Name	%-#	KLRG1 (MAFA)-APC-A Mean	KLRG1 (MAFA)-APC-A Median
POS-unpigm.	21,50	1,54	1,33
POS-pigm.	25,35	2,12	1,94

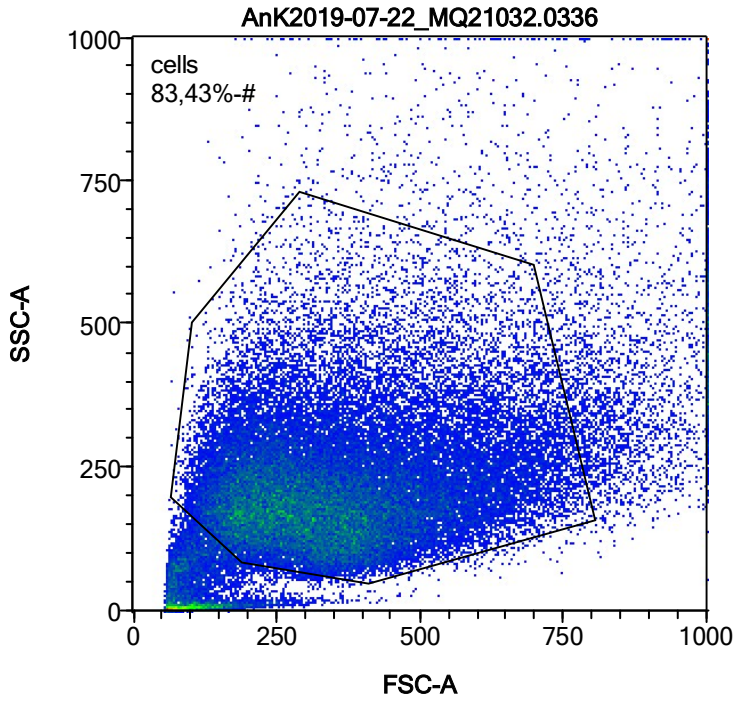

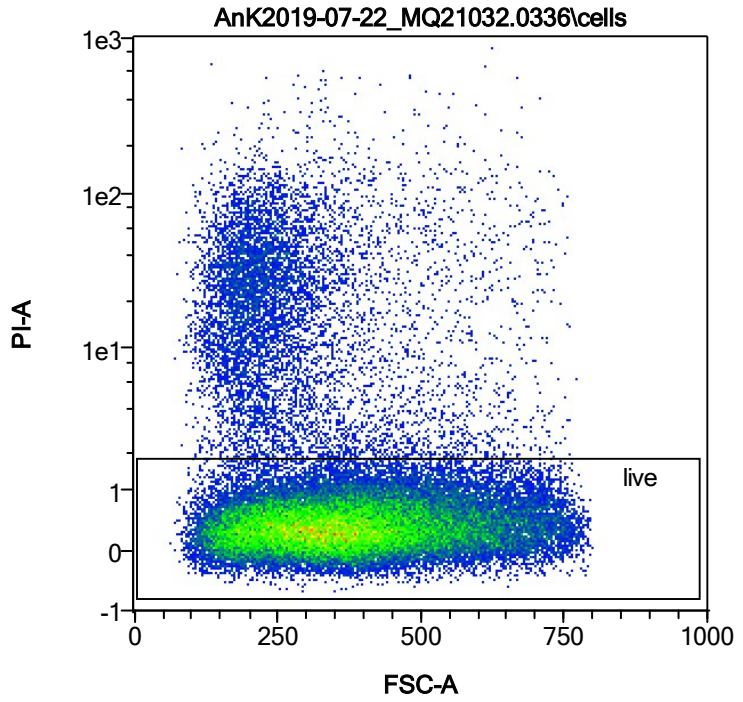

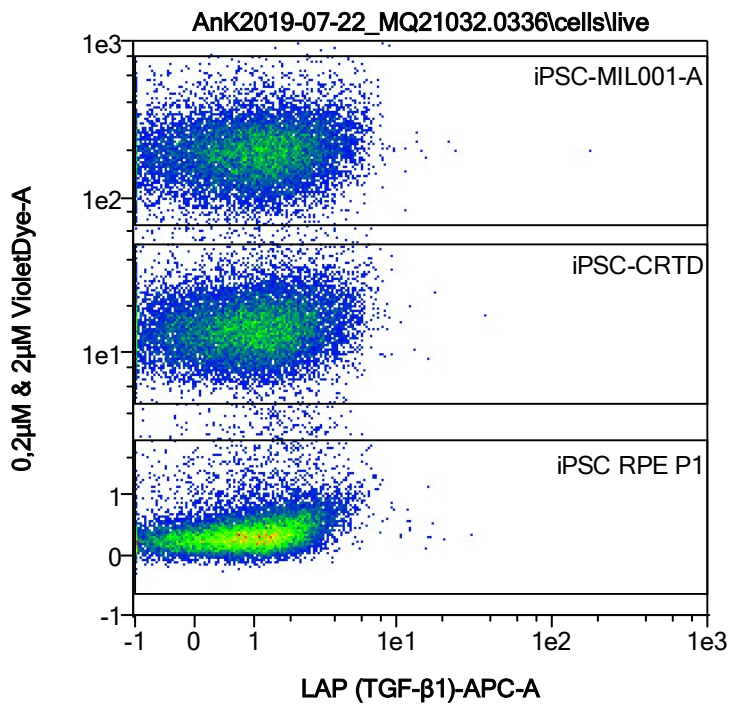

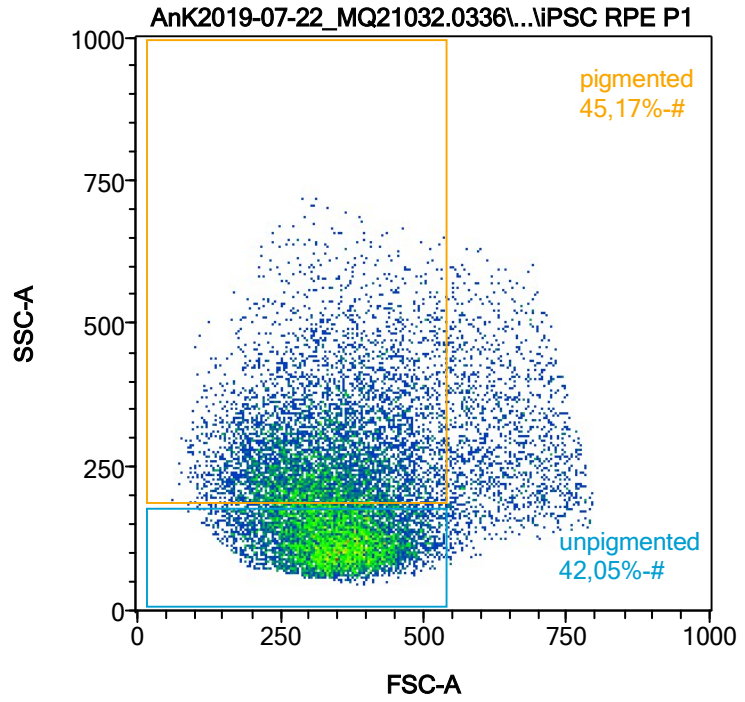

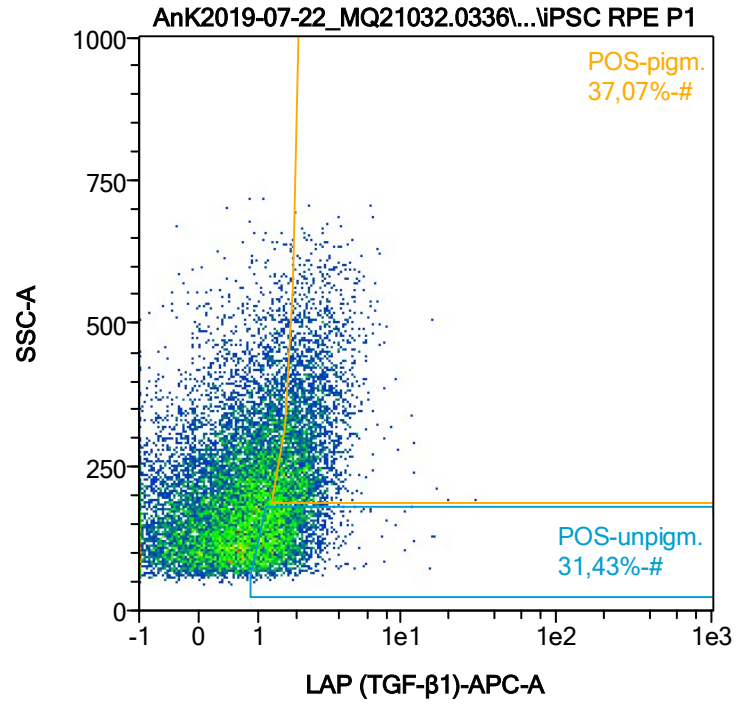

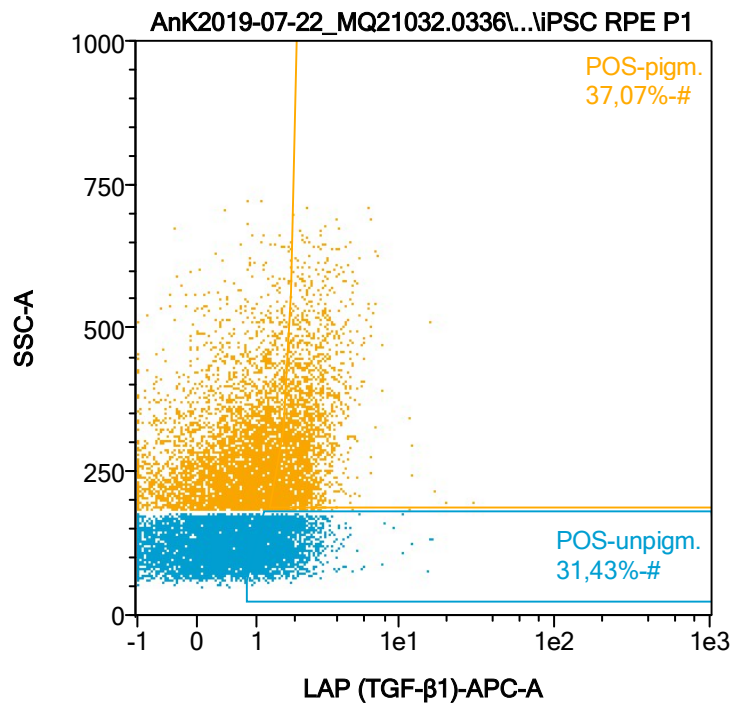

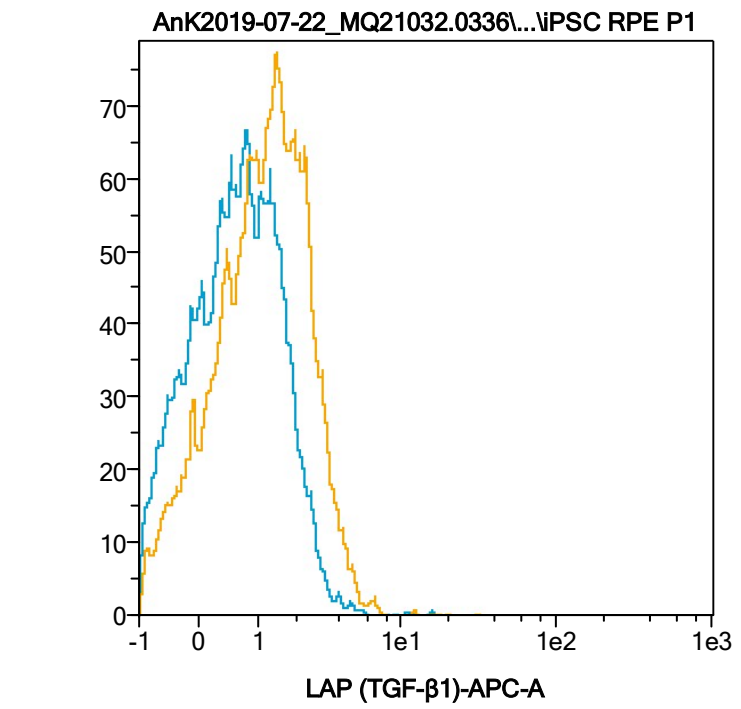

Name	%-#	LAP (TGF-β1)-APC-A Mean	LAP (TGF-β1)-APC-A Median
POS-unpigm.	31,43	1,70	1,50
POS-pigm.	37,07	2,45	2,21

Date:2024-06-14
Operator: Sebastian Knoebel
Version:2.13.0+54.g95abf0f4

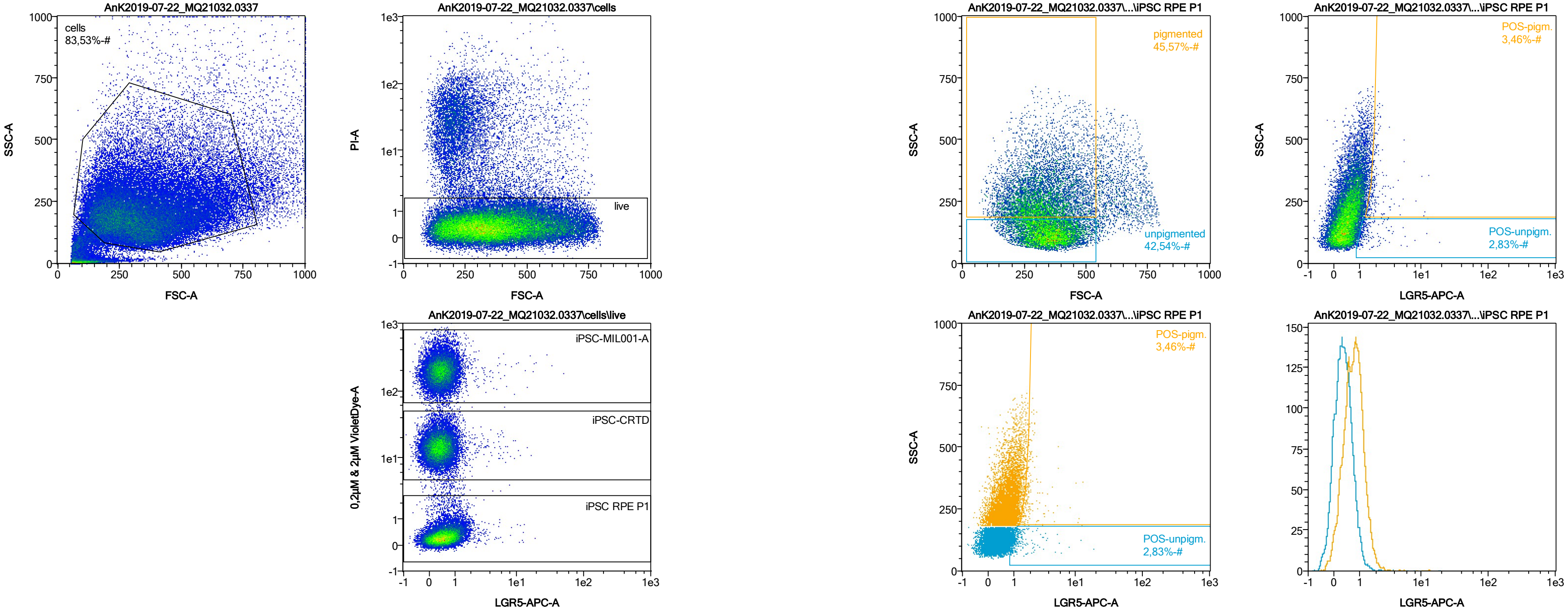

Name	%-#	LGR5-APC-A Mean	LGR5-APC-A Median
POS-unpigm.	2,83	1,68	1,21
POS-pigm.	3,46	2,25	1,82

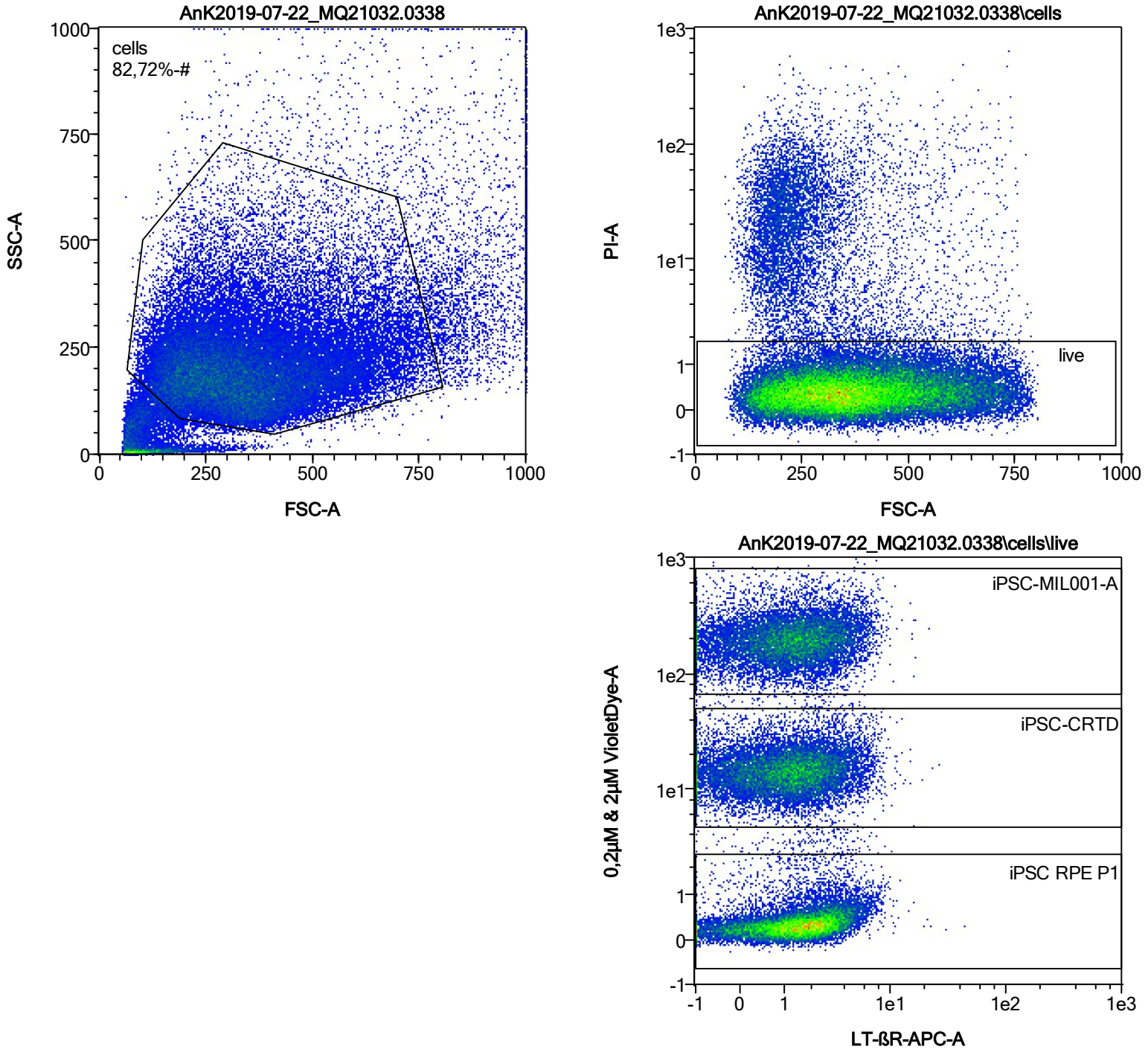

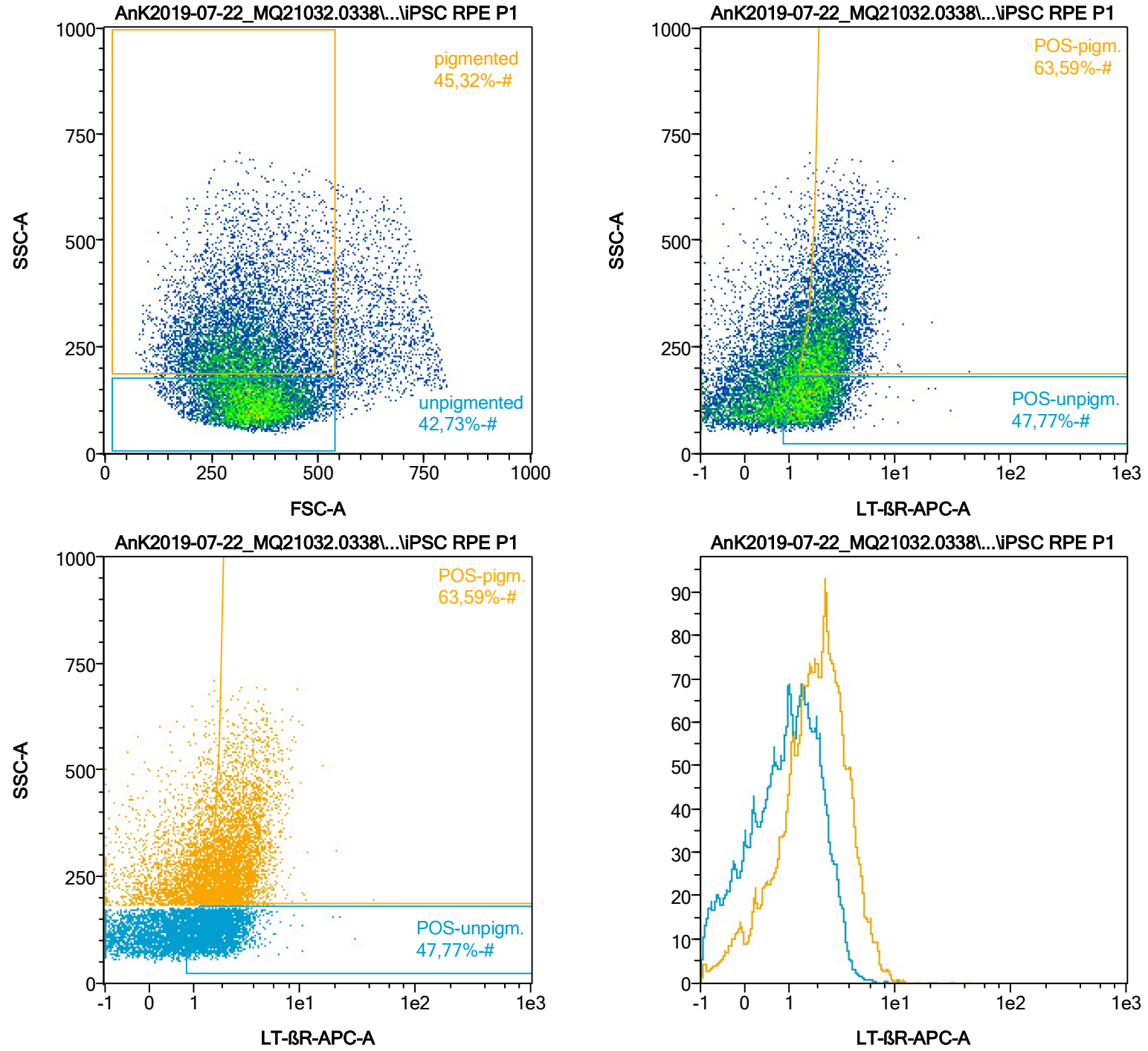

Name	%-#	LT-ßR-APC-A Mean	LT-ßR-APC-A Median
POS-unpigm.	47,77	1,95	1,73
POS-pigm.	63,59	2,97	2,63

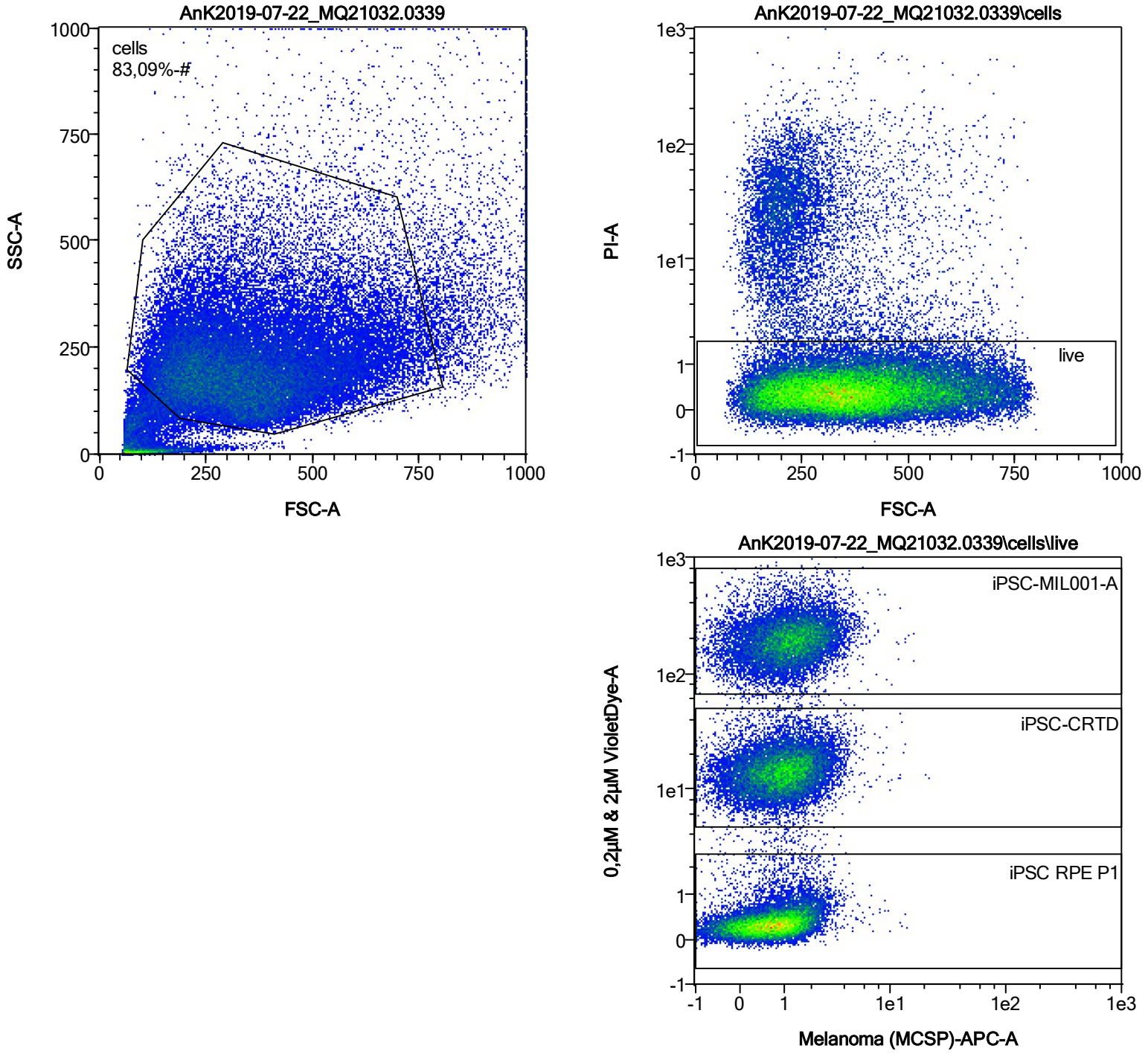

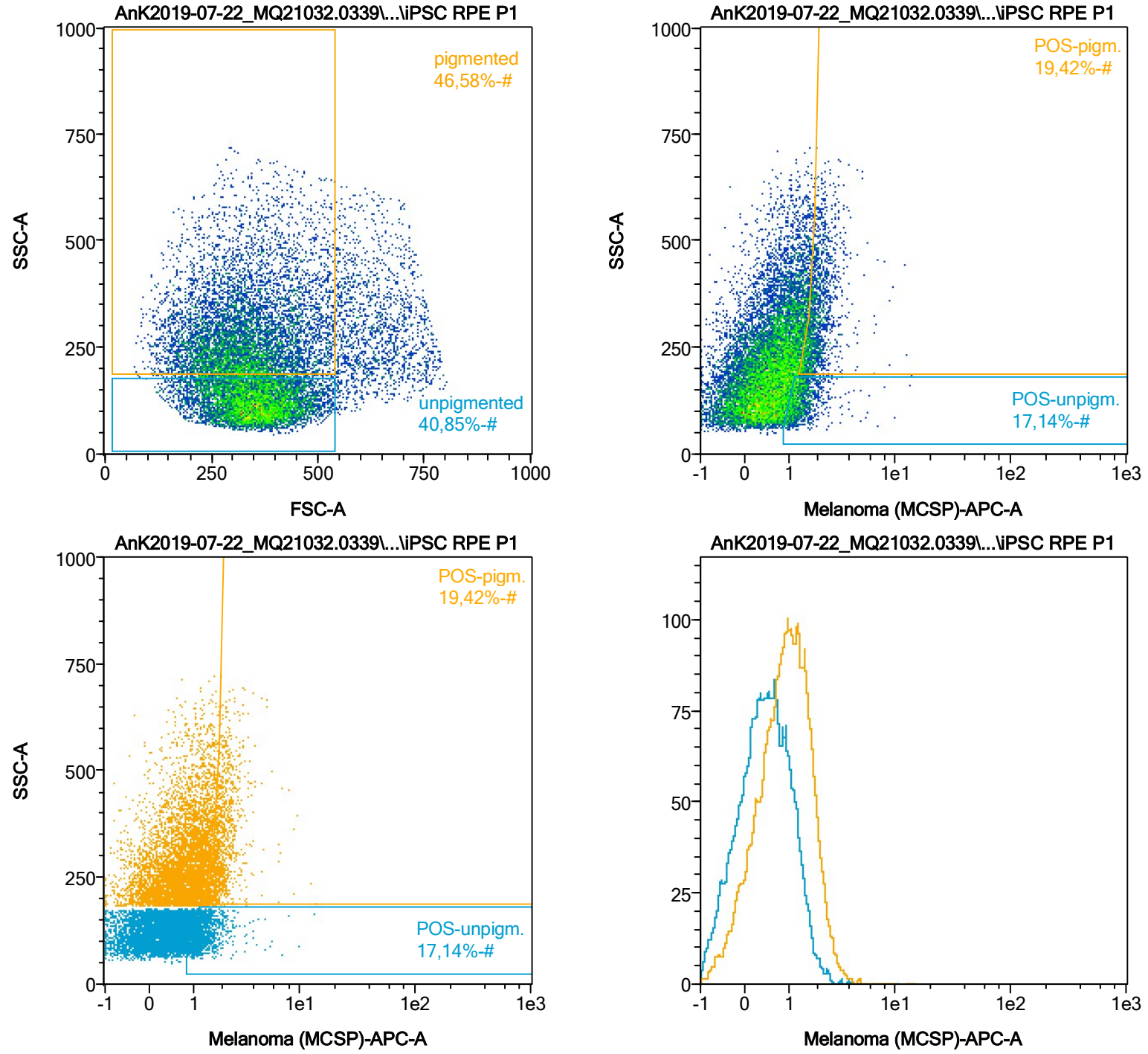

Name	%-#	Melanoma (MCSP)-APC-A Mean	Melanoma (MCSP)-APC-A Median
POS-unpigm.	17,14	1,44	1,29
POS-pigm.	19,42	2,07	1,91

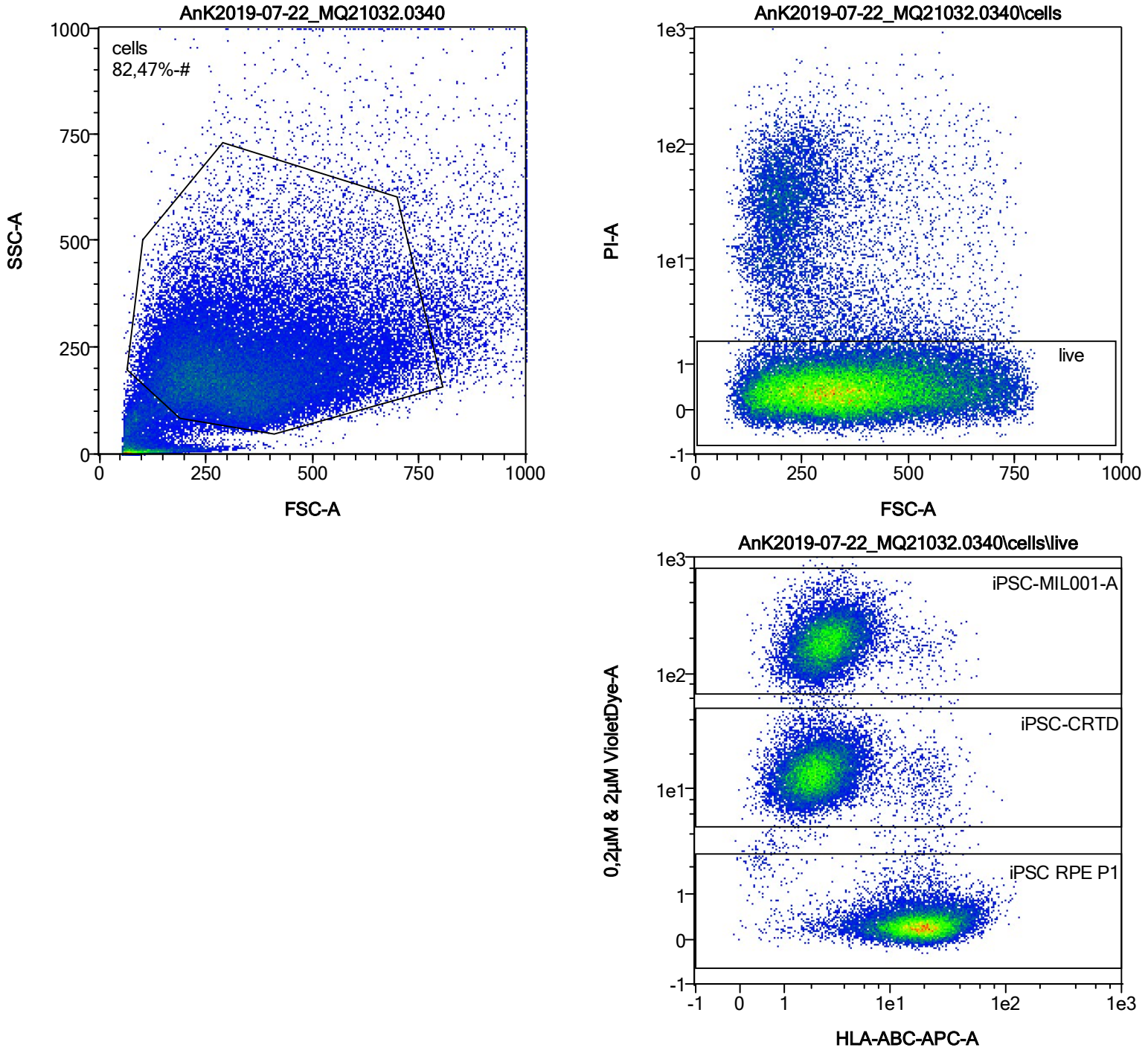

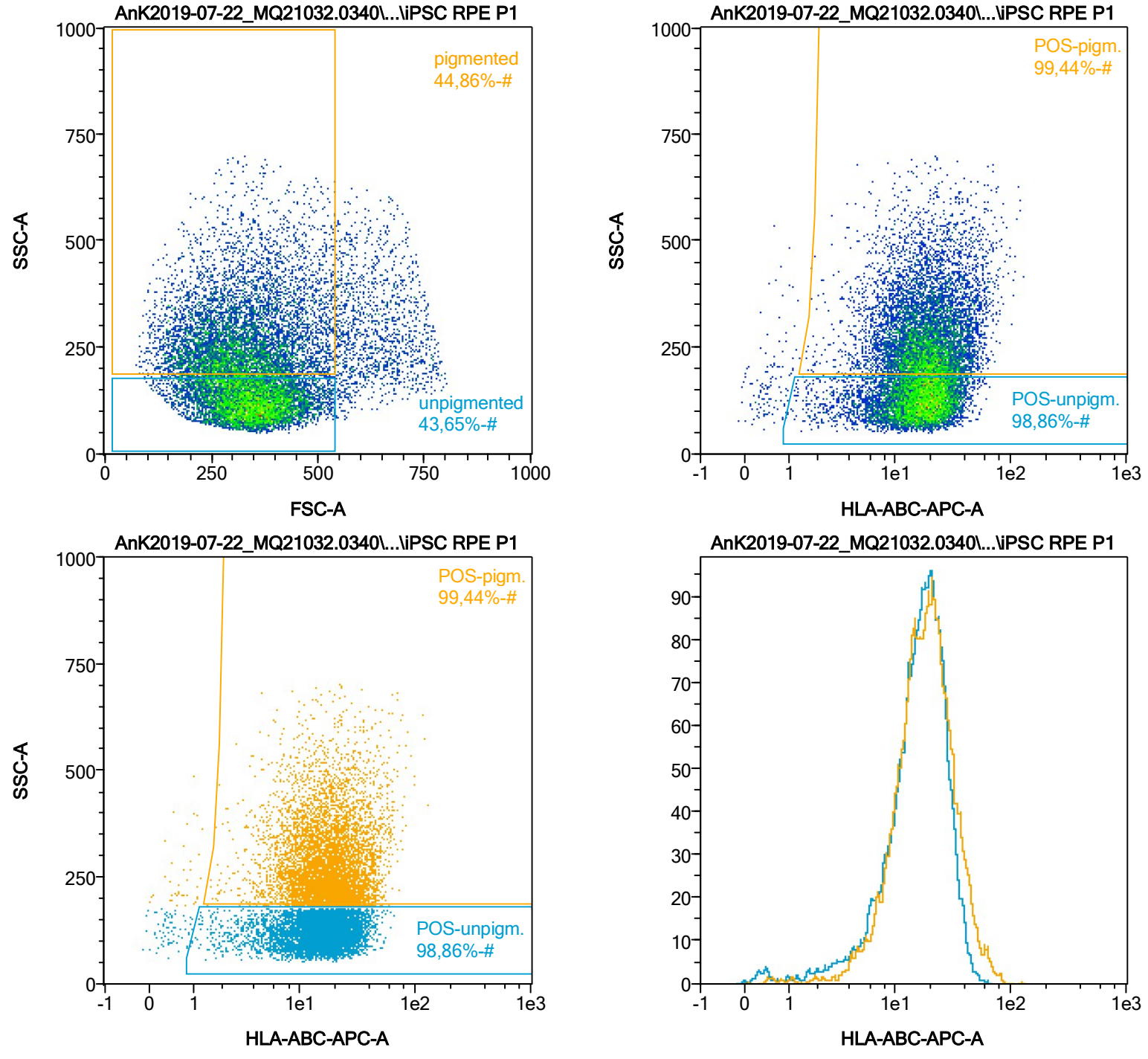

Name	%-#	HLA-ABC-APC-A Mean	HLA-ABC-APC-A Median
POS-unpigm.	98,86	17,64	16,85
POS-pigm.	99,44	20,34	18,02

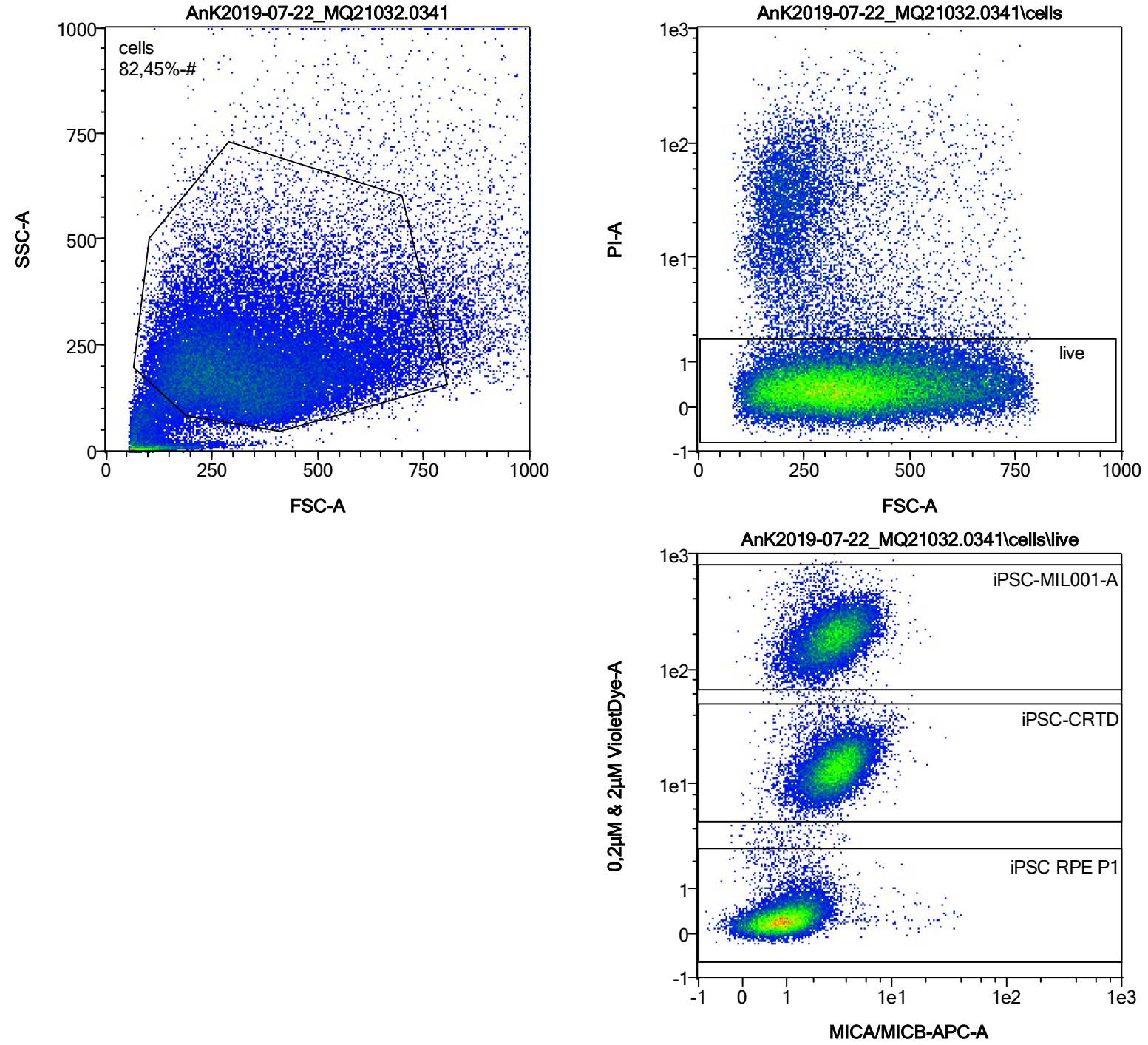

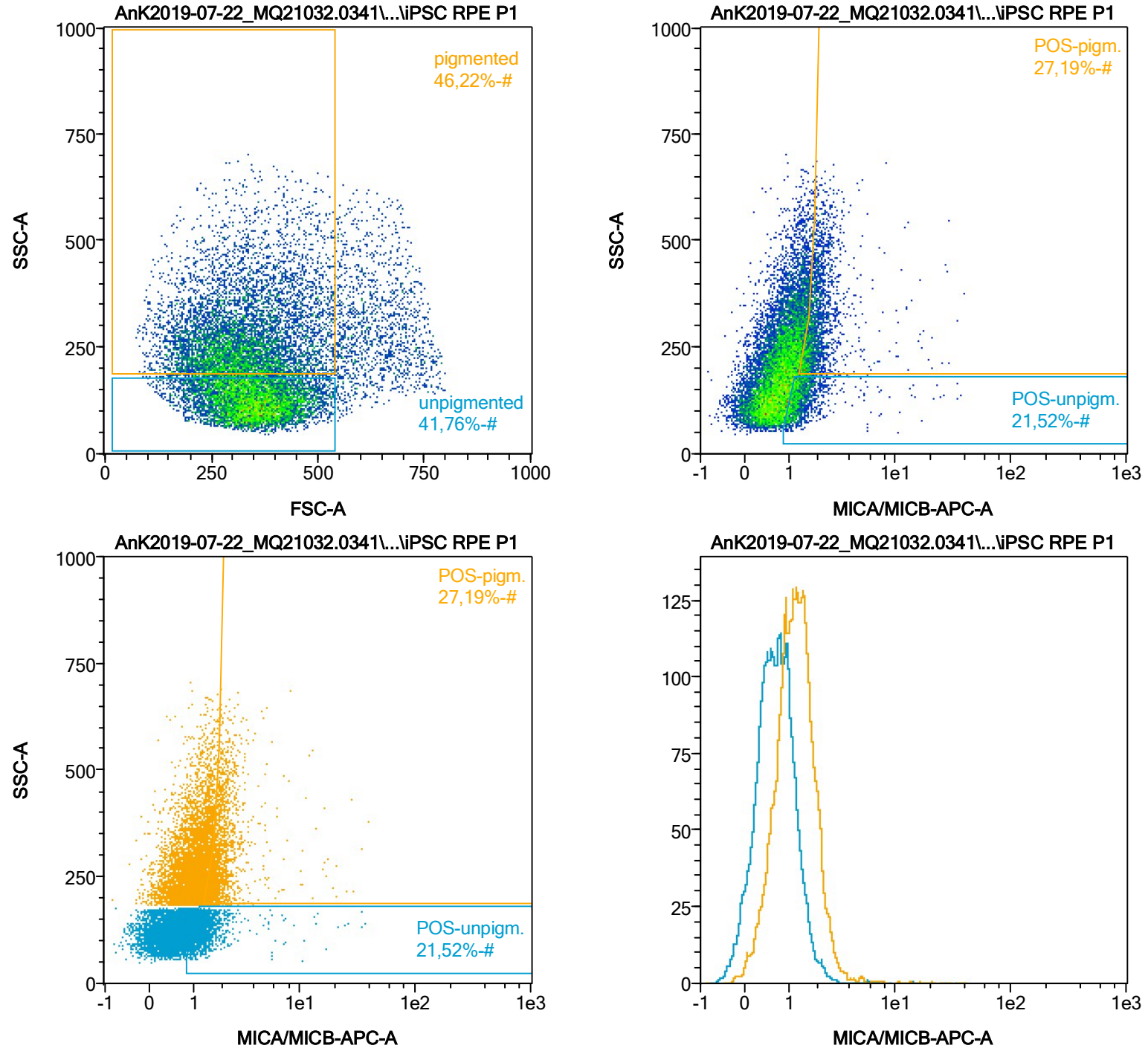

Name	%-#	MICA/MICB-APC-A Mean	MICA/MICB-APC-A Median
POS-unpigm.	21,52	1,60	1,26
POS-pigm.	27,19	2,22	1,88

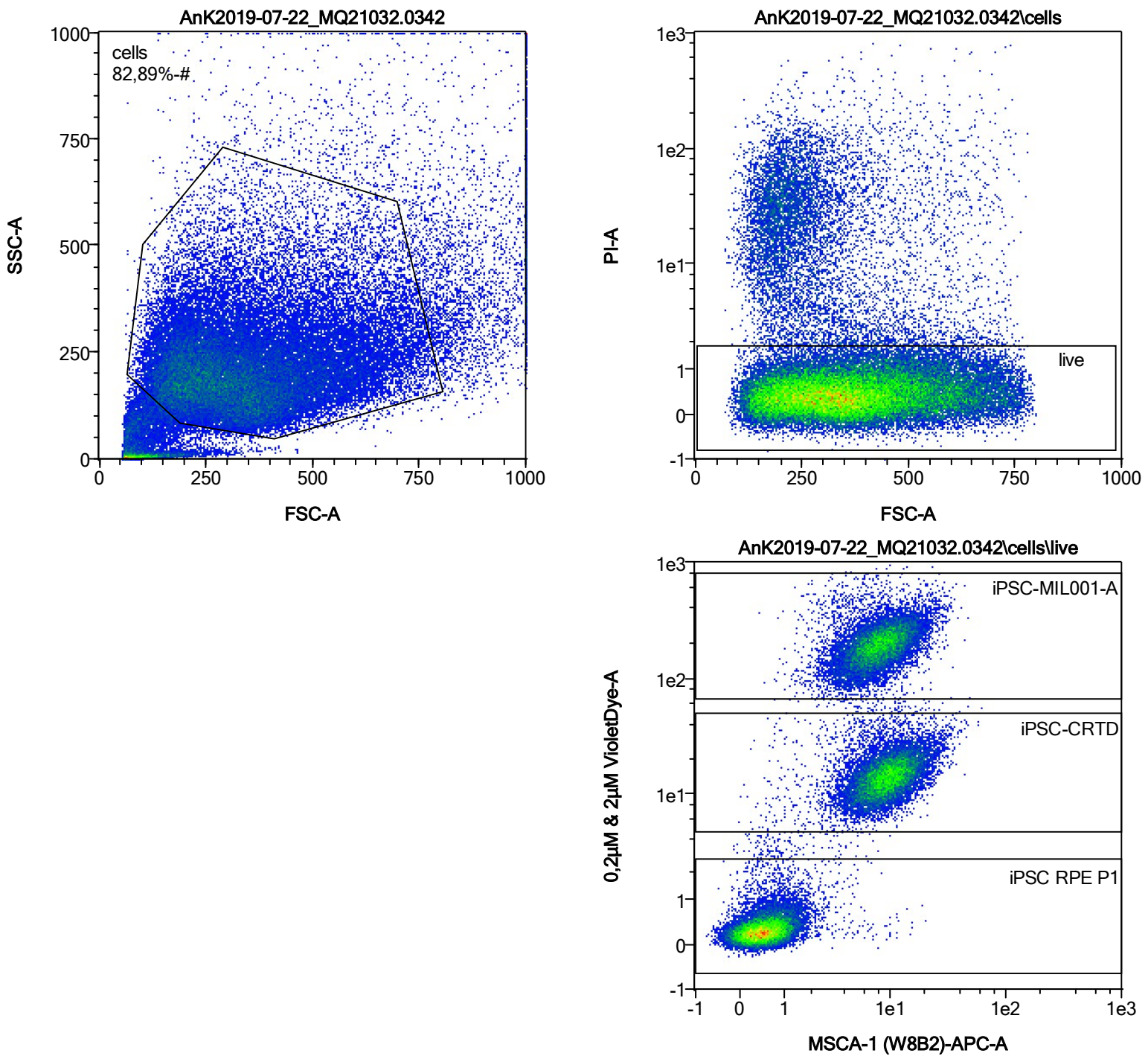

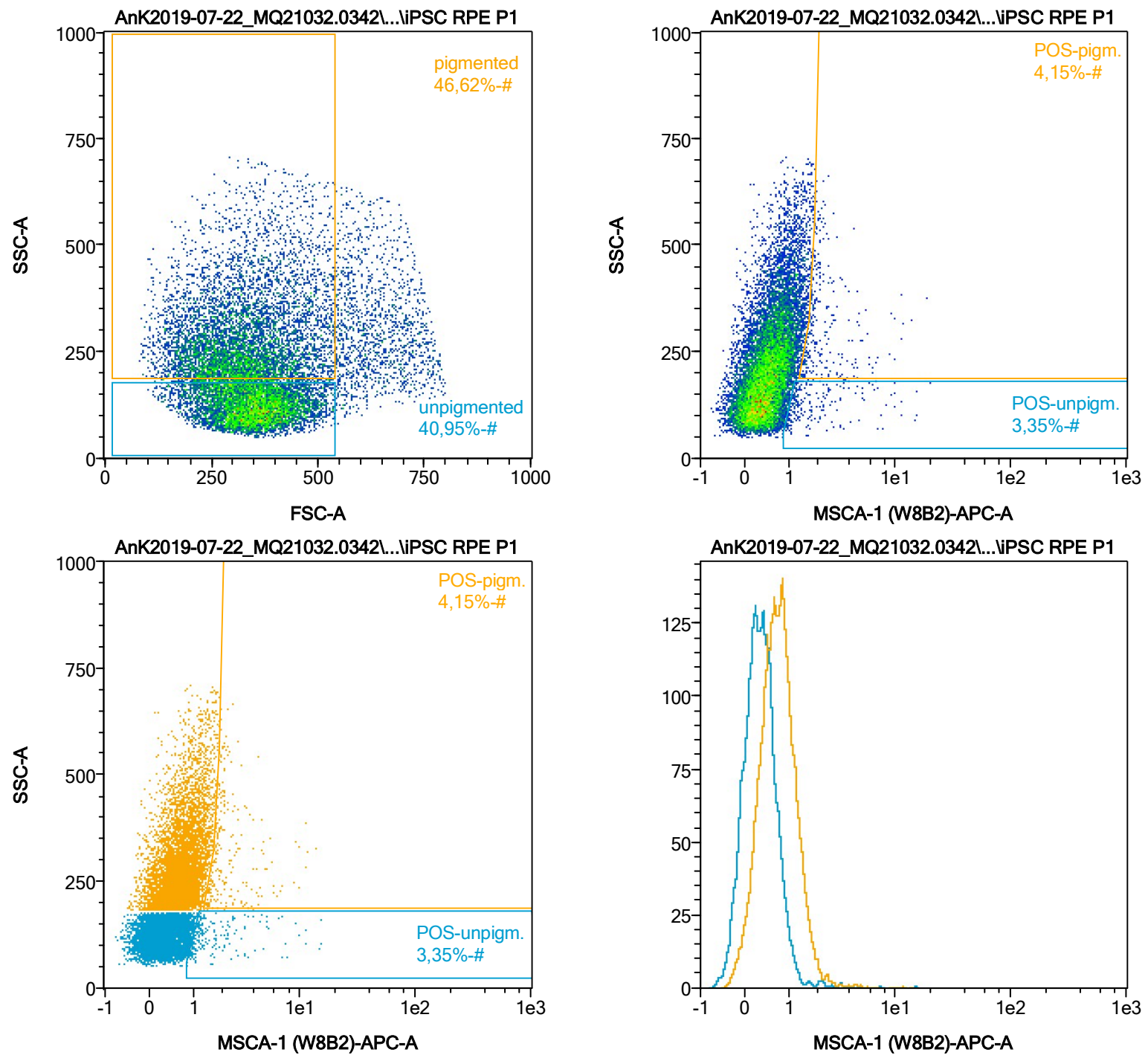

Name	%-#	MSCA-1 (W8B2)-APC-A Mean	MSCA-1 (W8B2)-APC-A Median
POS-unpigm.	3,35	2,20	1,30
POS-pigm.	4,15	2,51	1,93

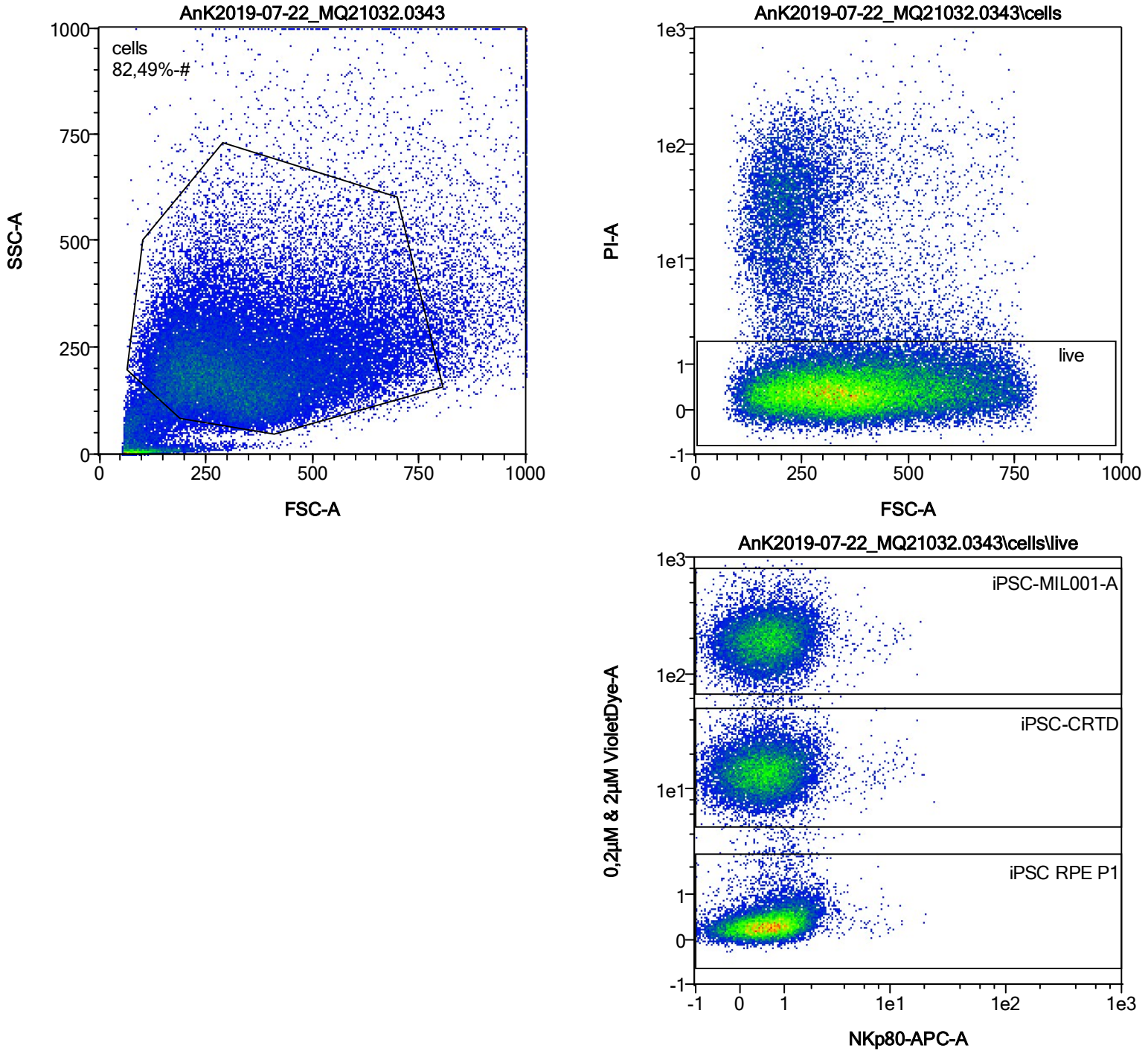

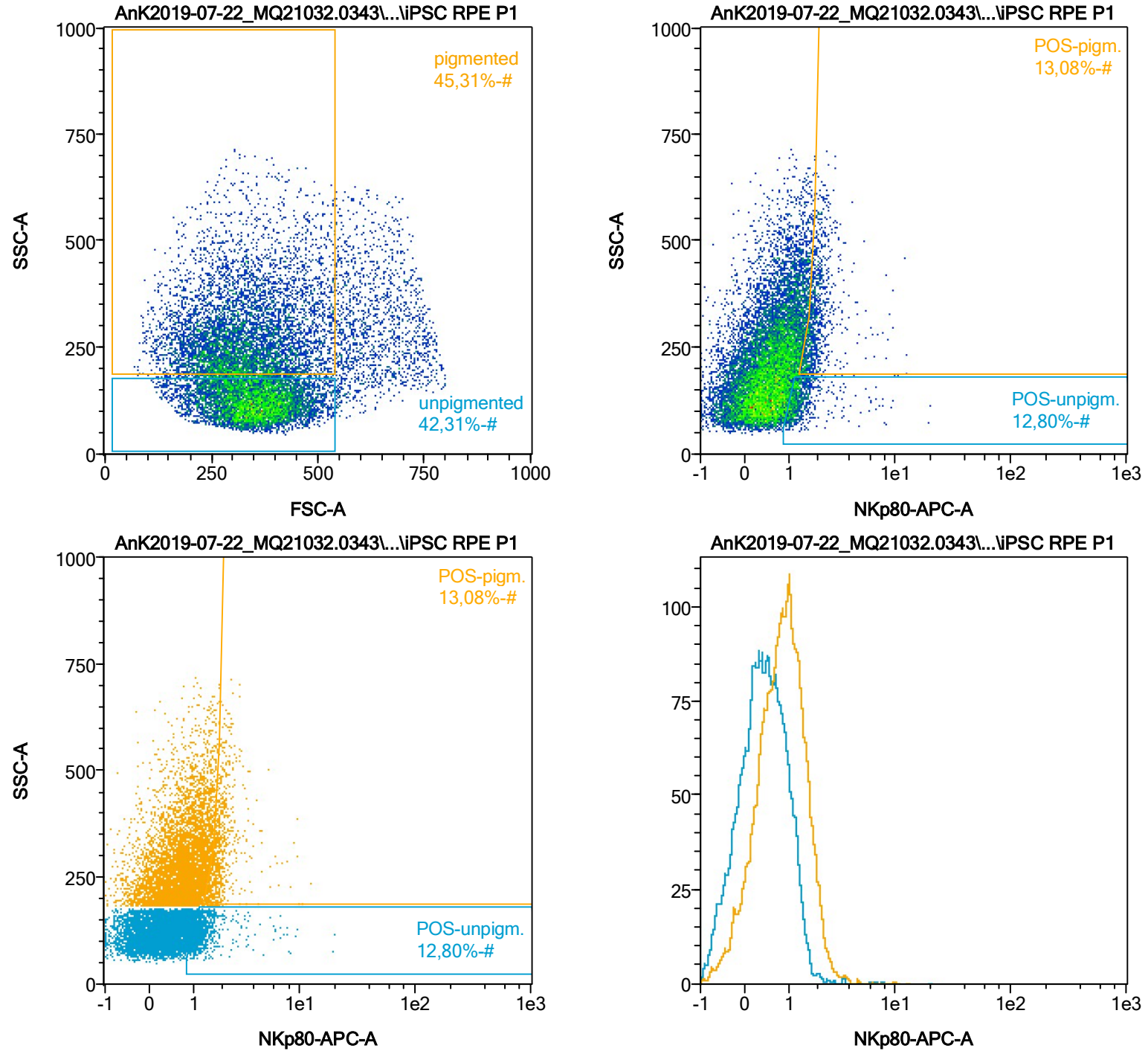

Name	%-#	NKp80-APC-A Mean	NKp80-APC-A Median
POS-unpigm.	12,80	1,50	1,23
POS-pigm.	13,08	2,05	1,86

Date:2024-06-14

Operator: Sebastian Knoebel

Version:2.13.0+54.g95abf0f4

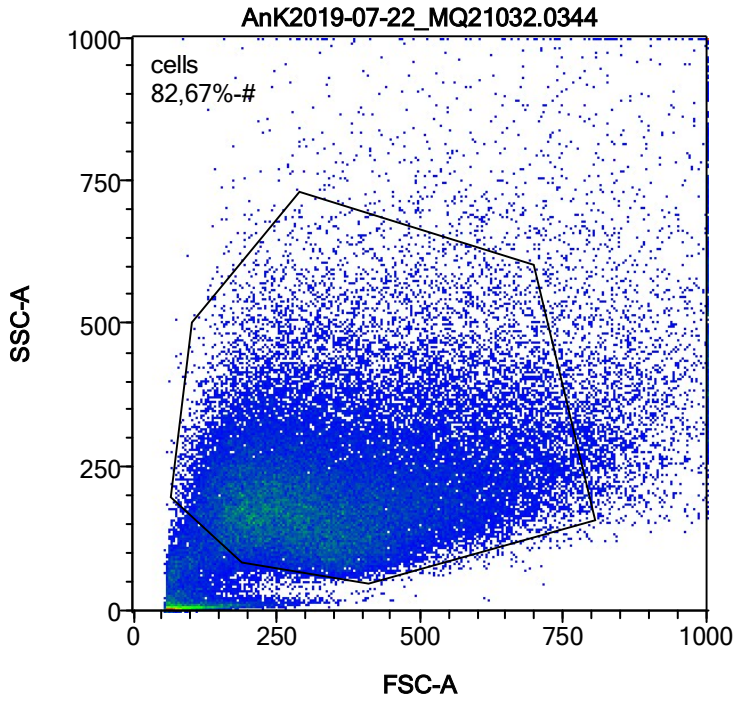

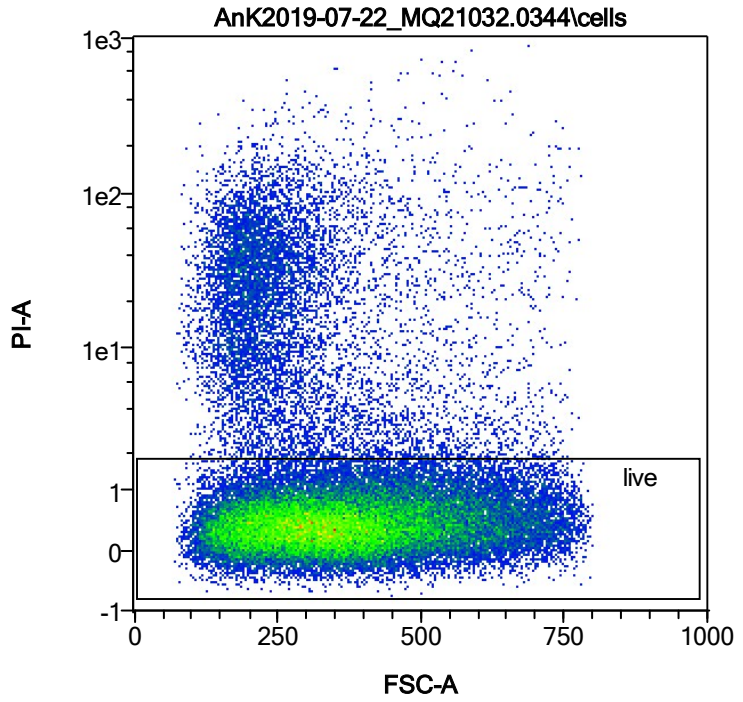

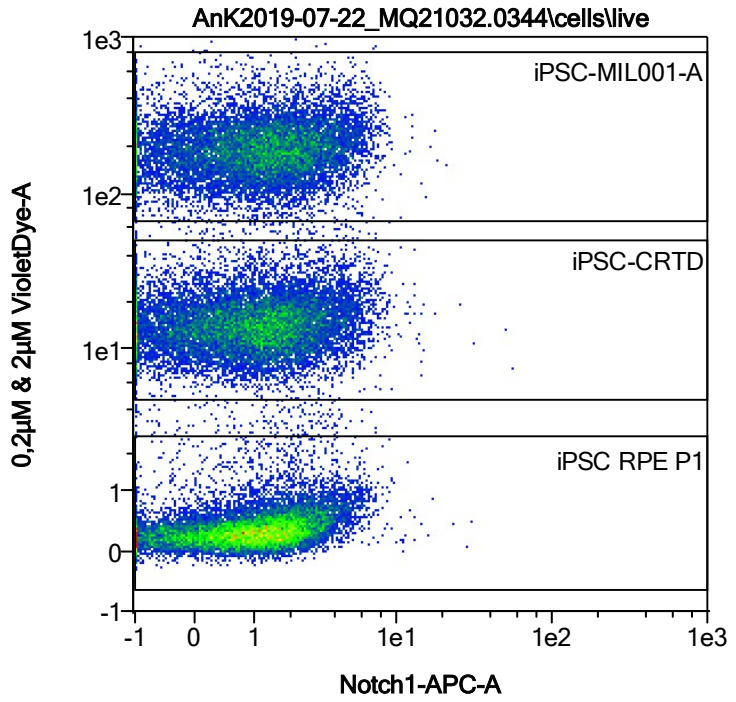

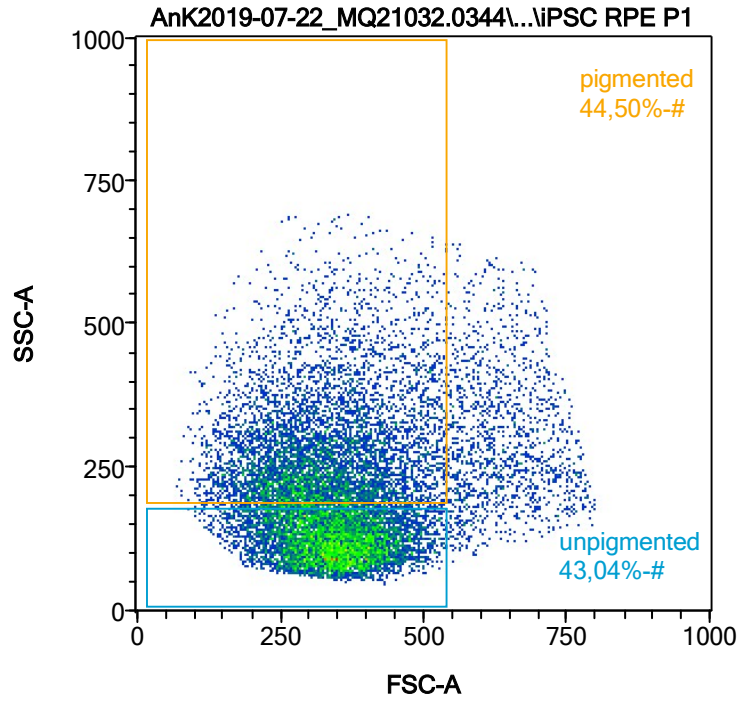

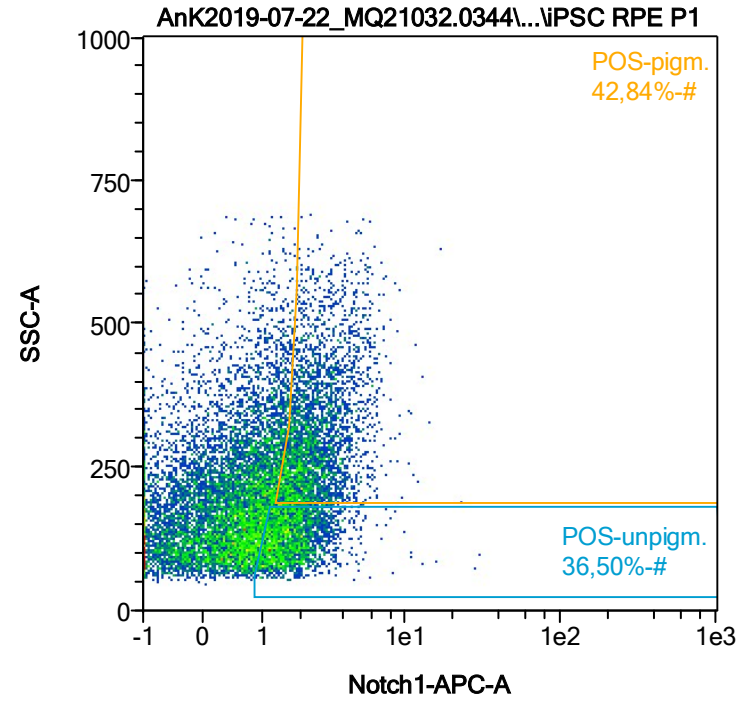

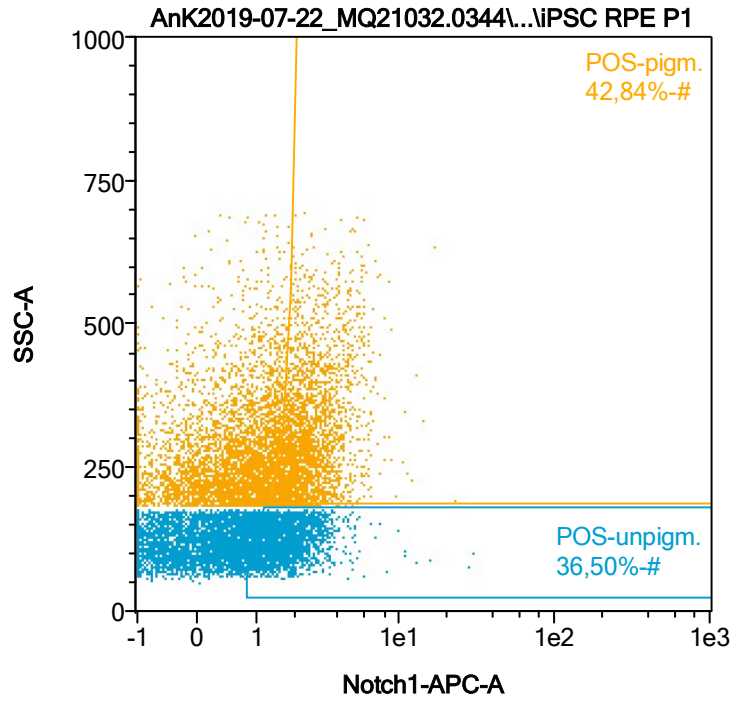

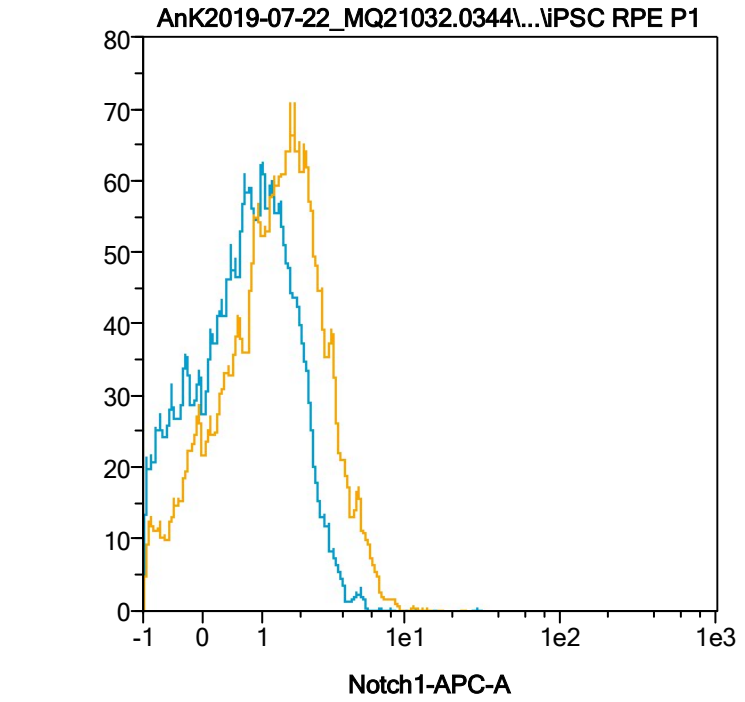

Name	%-#	Notch1-APC-A Mean	Notch1-APC-A Median
POS-unpigm.	36,50	1,85	1,62
POS-pigm.	42,84	2,72	2,35

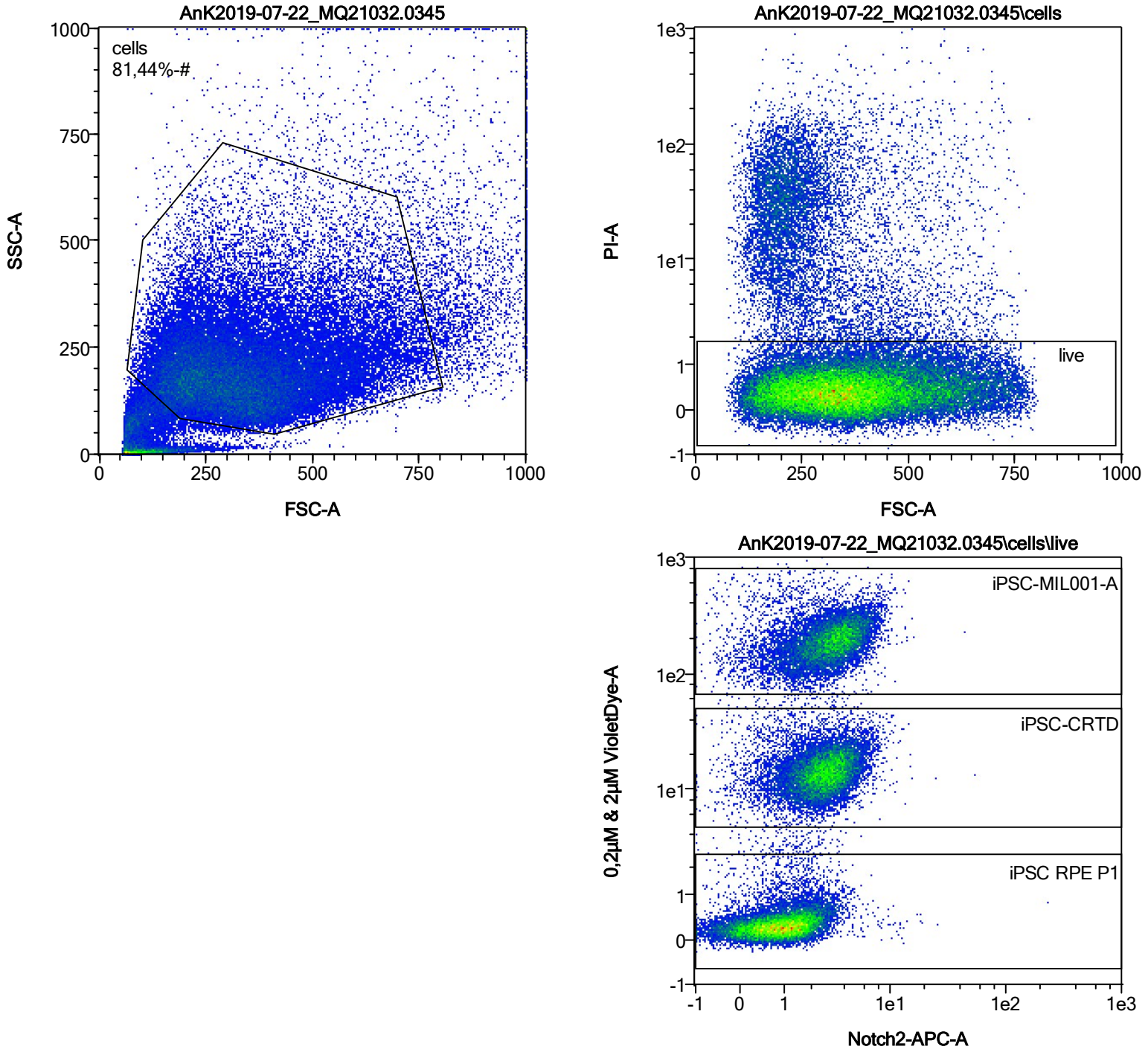

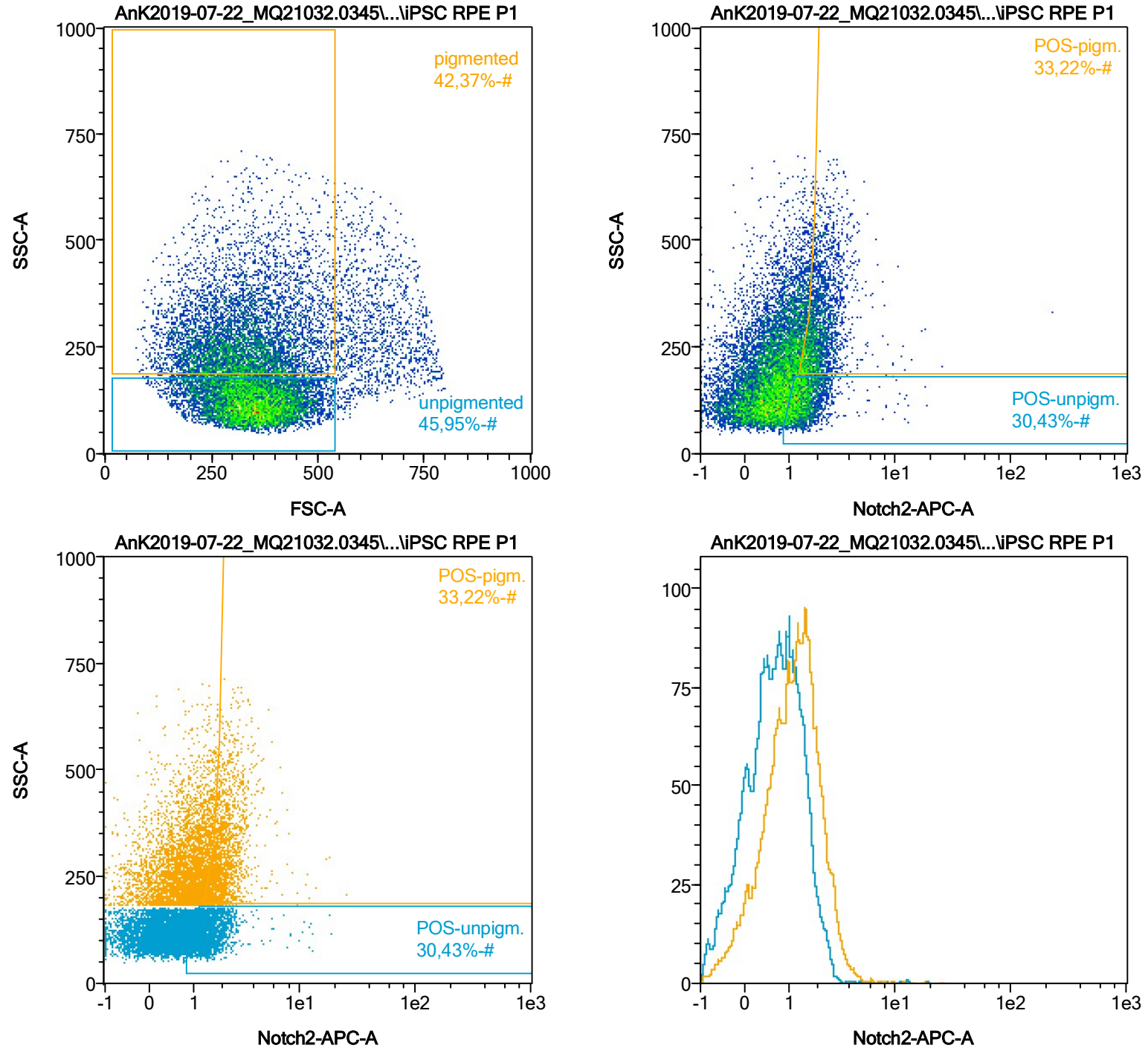

Name	%-#	Notch2-APC-A Mean	Notch2-APC-A Median
POS-unpigm.	30,43	1,56	1,39
POS-pigm.	33,22	2,20	1,97

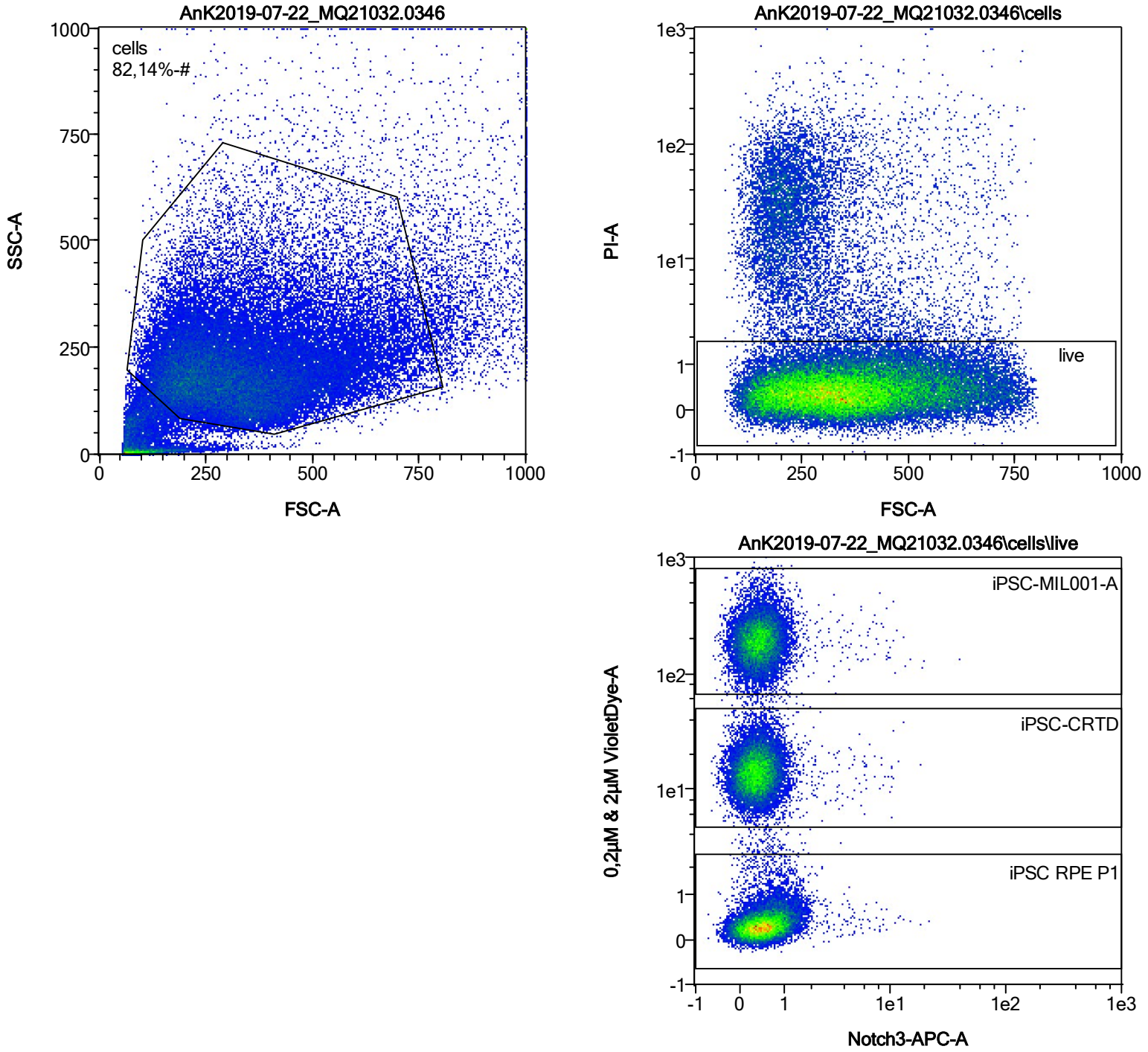

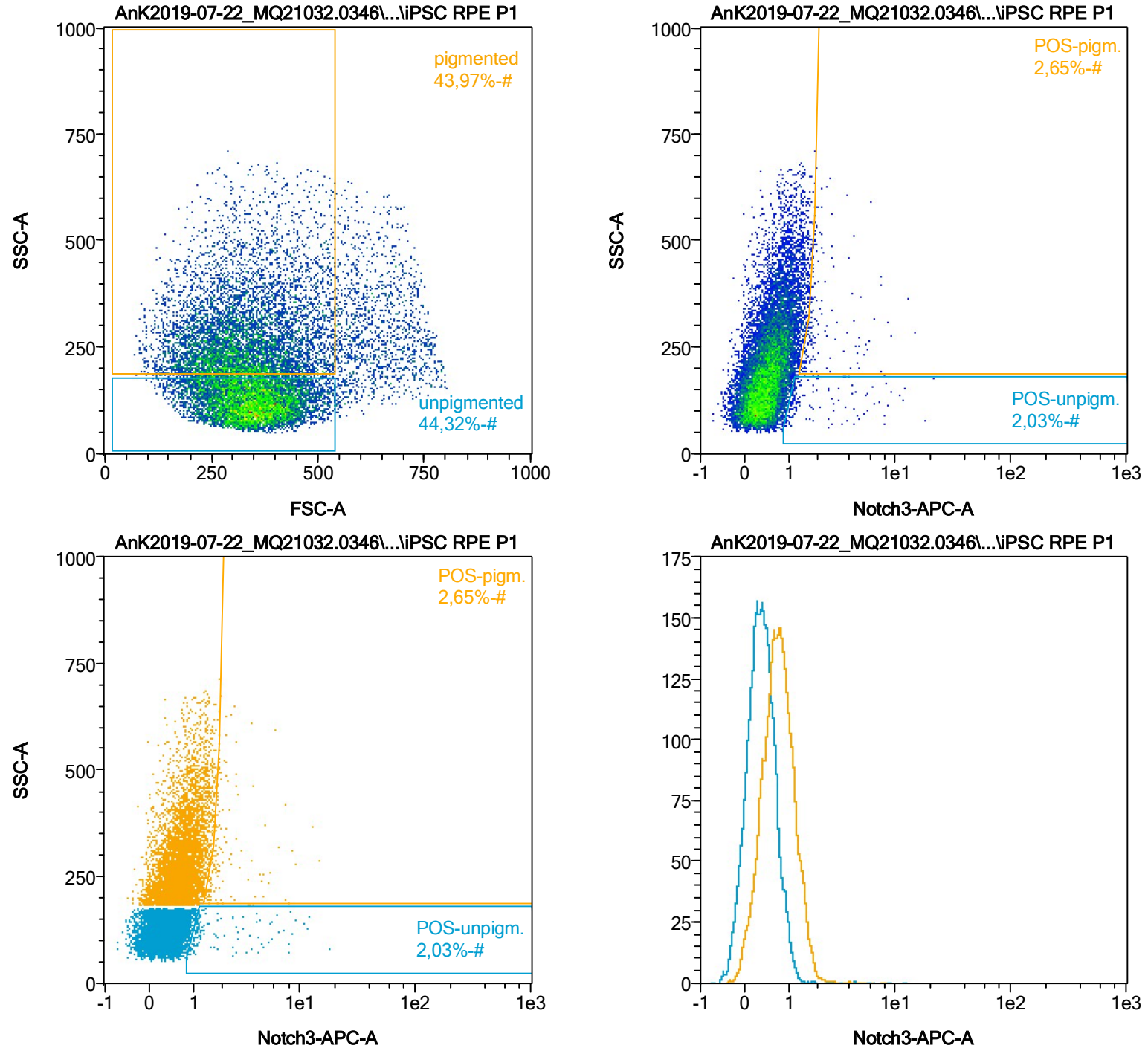

Name	%-#	Notch3-APC-A Mean	Notch3-APC-A Median
POS-unpigm.	2,03	2,44	1,20
POS-pigm.	2,65	2,43	1,81

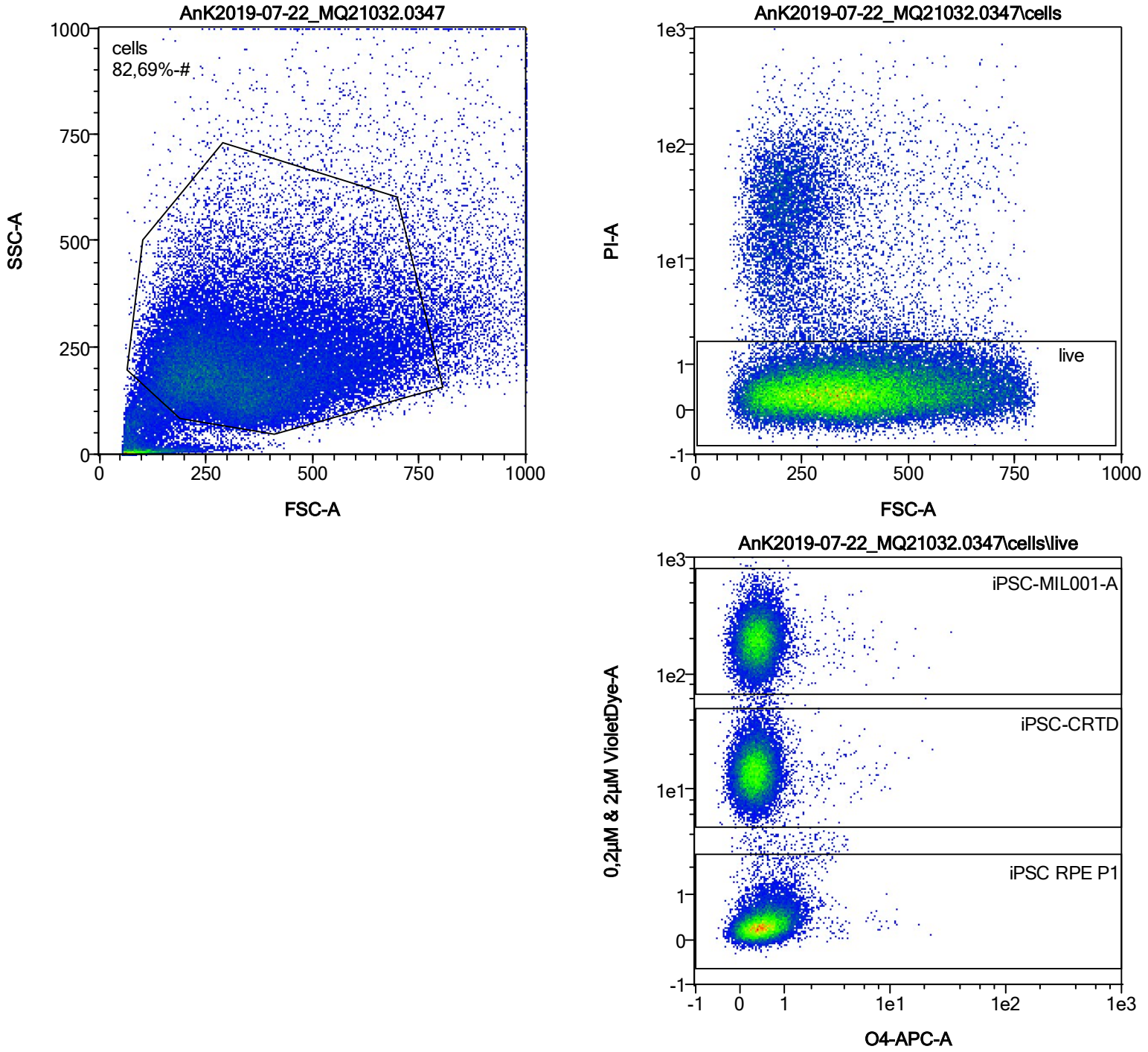

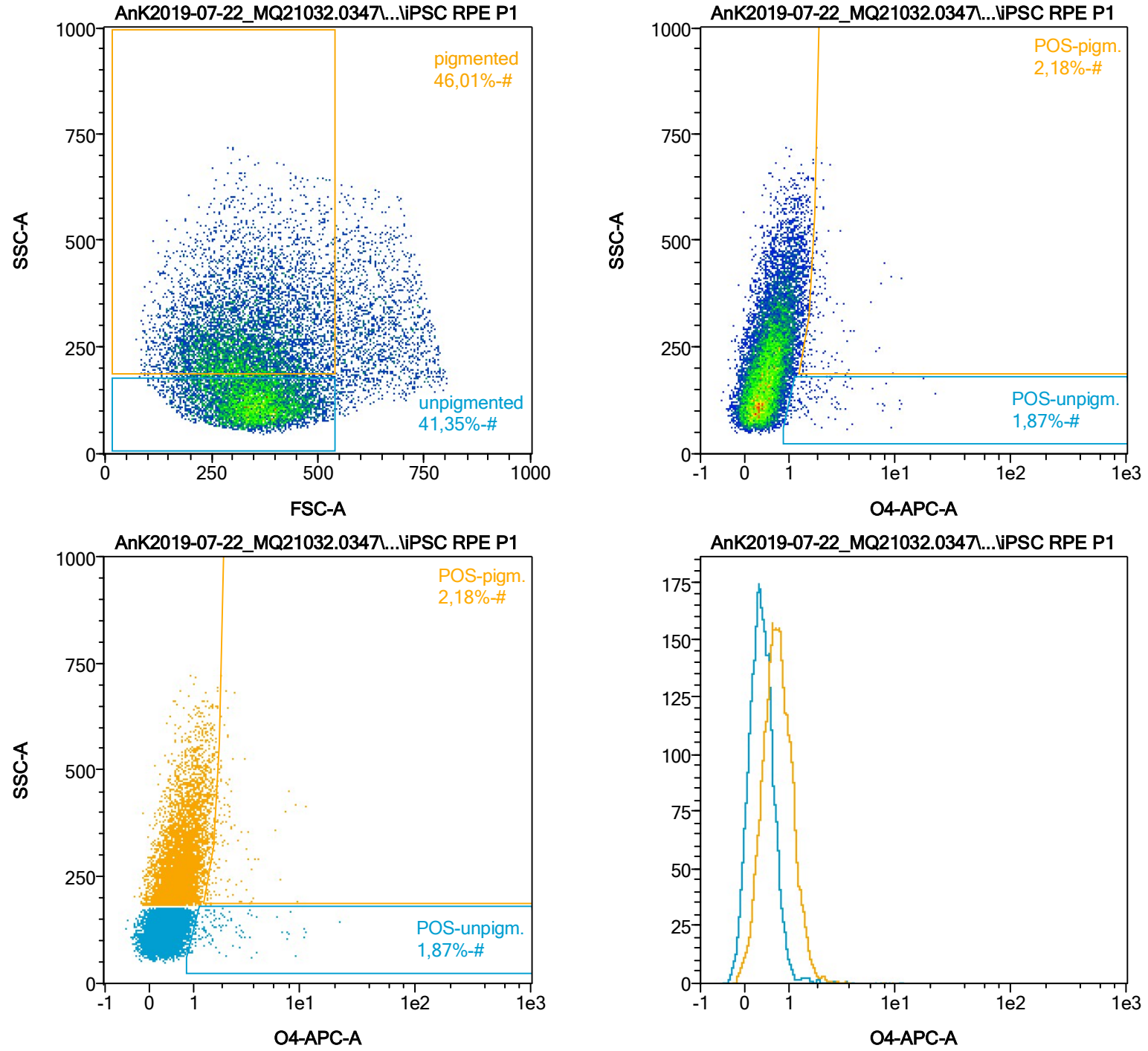

Name	%-#	O4-APC-A Mean	O4-APC-A Median
POS-unpigm.	1,87	2,64	1,61
POS-pigm.	2,18	2,38	1,92

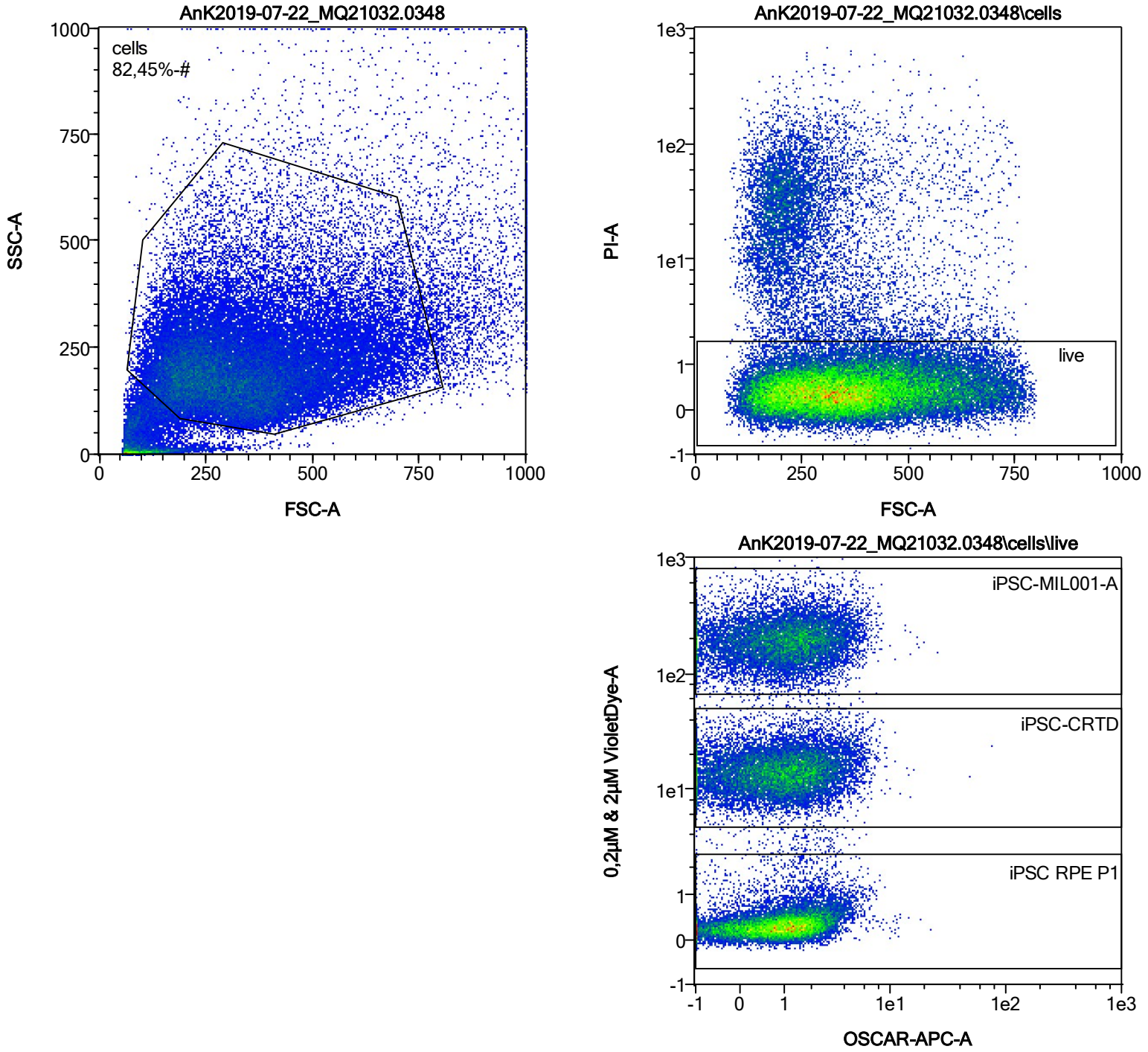

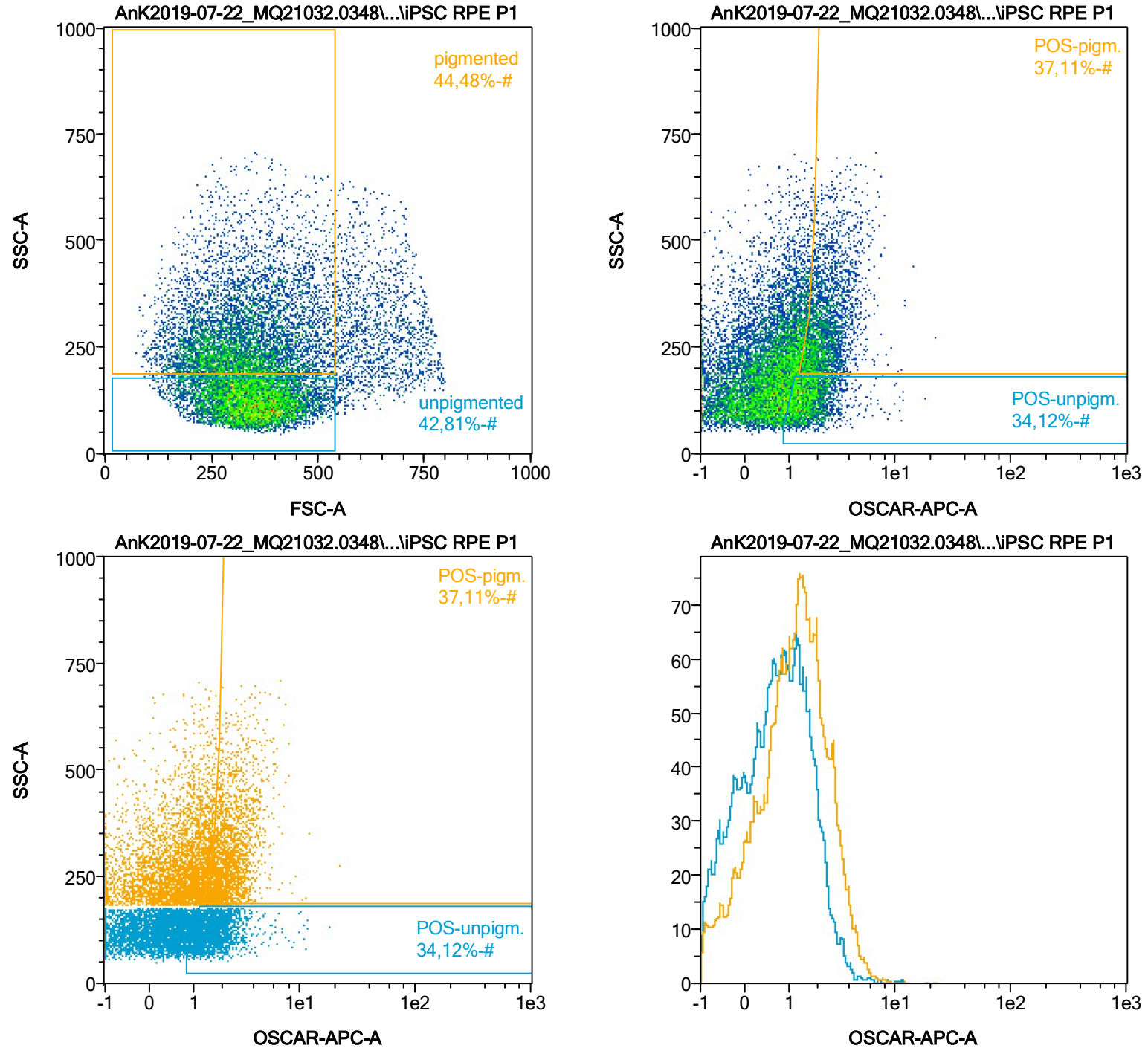

Name	%-#	OSCAR-APC-A Mean	OSCAR-APC-A Median
POS-unpigm.	34,12	1,80	1,57
POS-pigm.	37,11	2,48	2,20

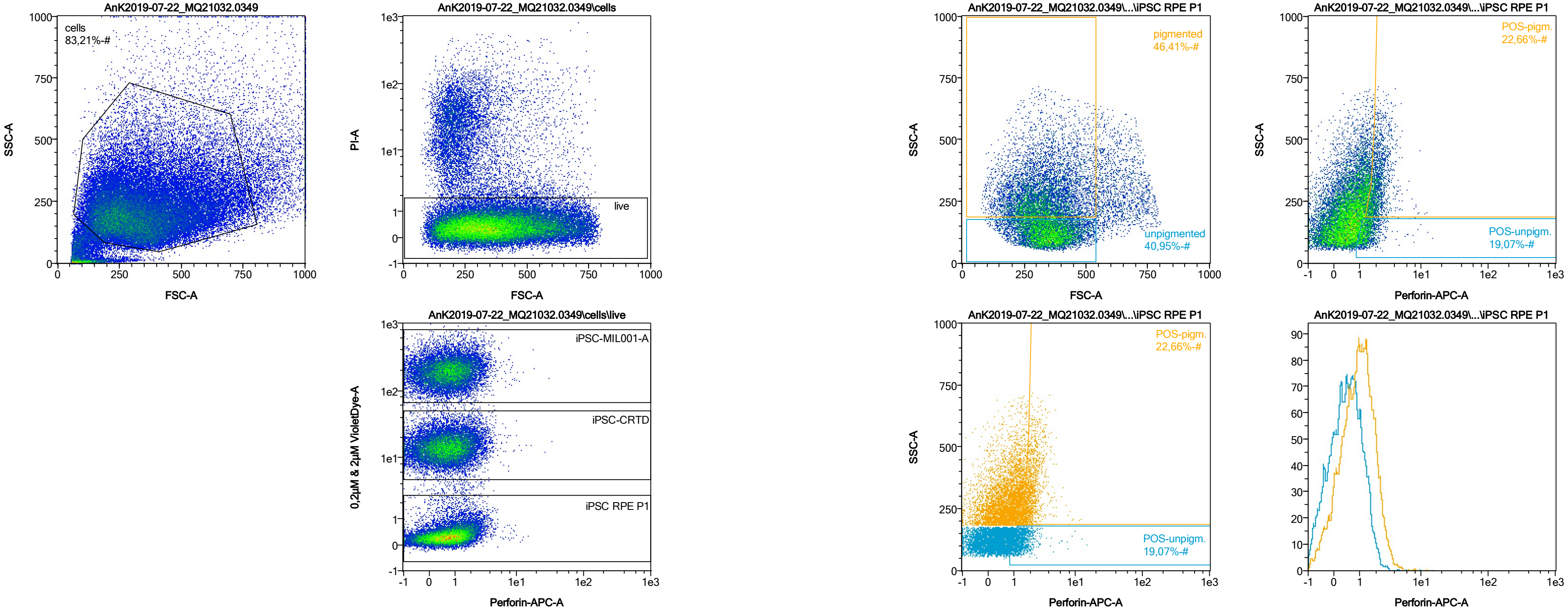

Name	%-#	Perforin-APC-A Mean	Perforin-APC-A Median
POS-unpigm.	19,07	1,49	1,34
POS-pigm.	22,66	2,19	2,00

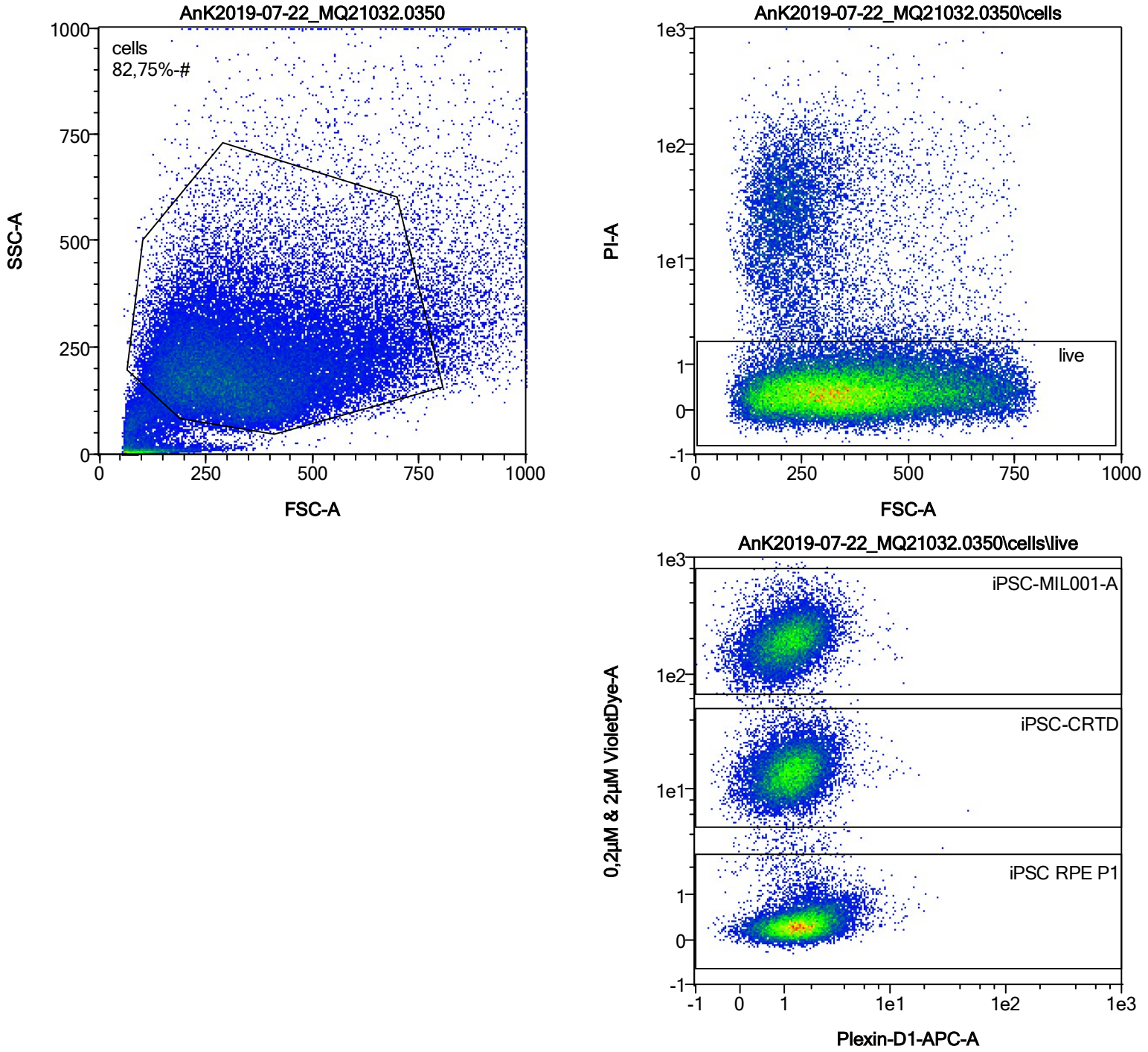

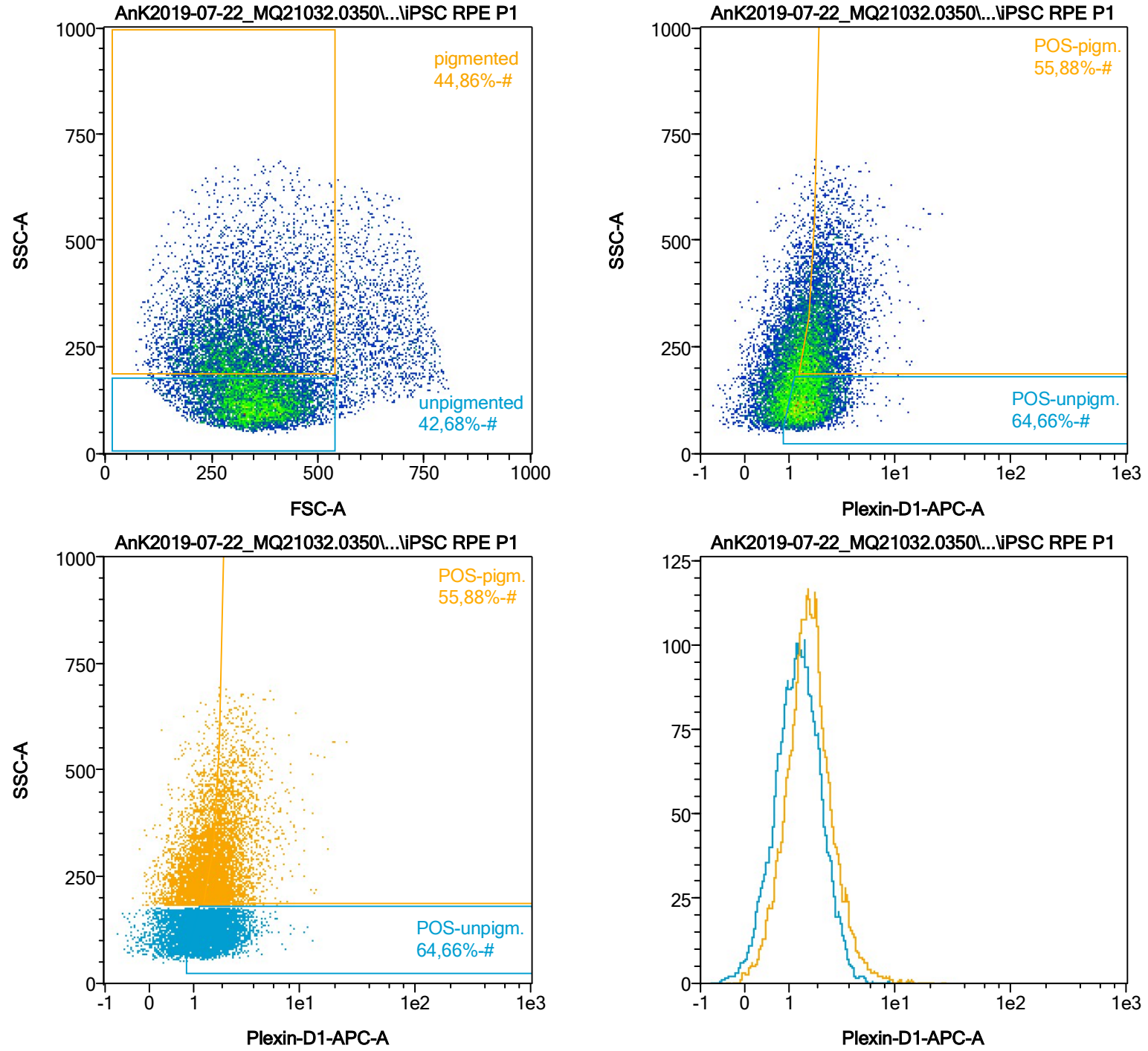

Name	%-#	Plexin-D1-APC-A Mean	Plexin-D1-APC-A Median
POS-unpigm.	64,66	1,81	1,62
POS-pigm.	55,88	2,46	2,12

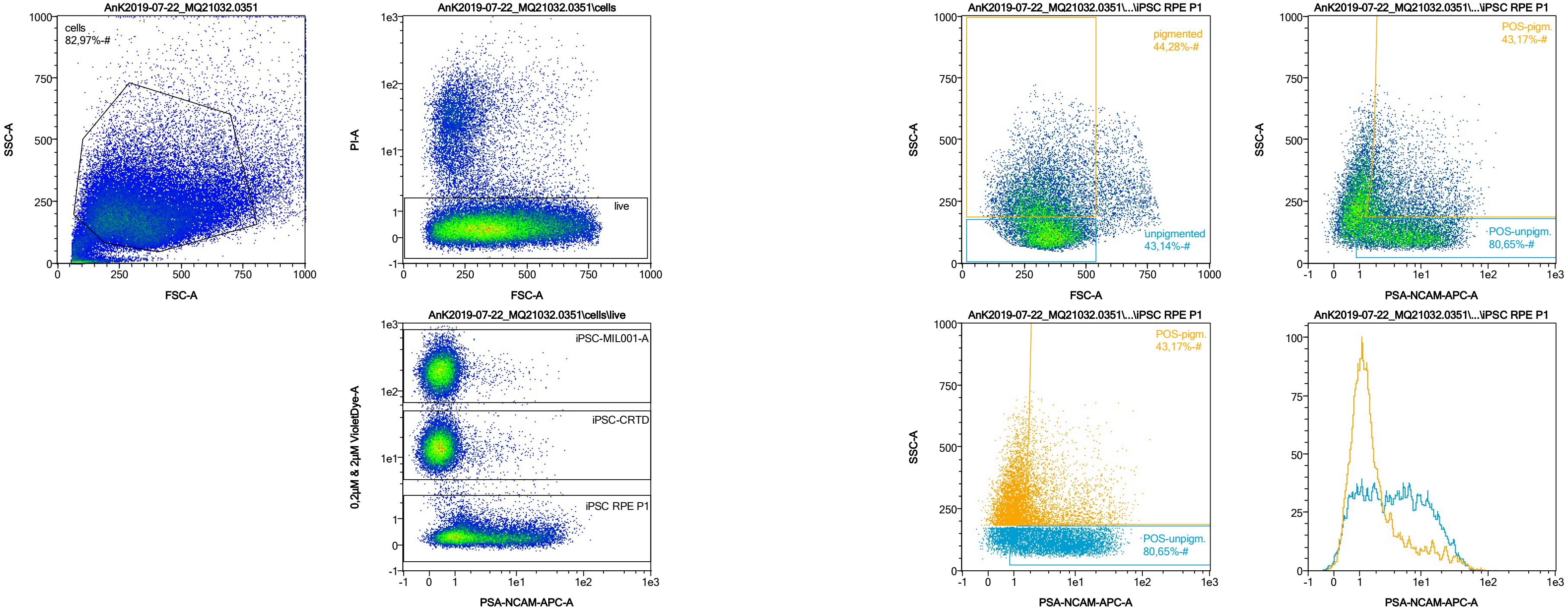

Name	%-#	PSA-NCAM-APC-A Mean	PSA-NCAM-APC-A Median
POS-unpigm.	80,65	8,35	5,26
POS-pigm.	43,17	7,43	3,13

Date:2024-06-14

Operator: Sebastian Knoebel

Version:2.13.0+54.g95abf0f4

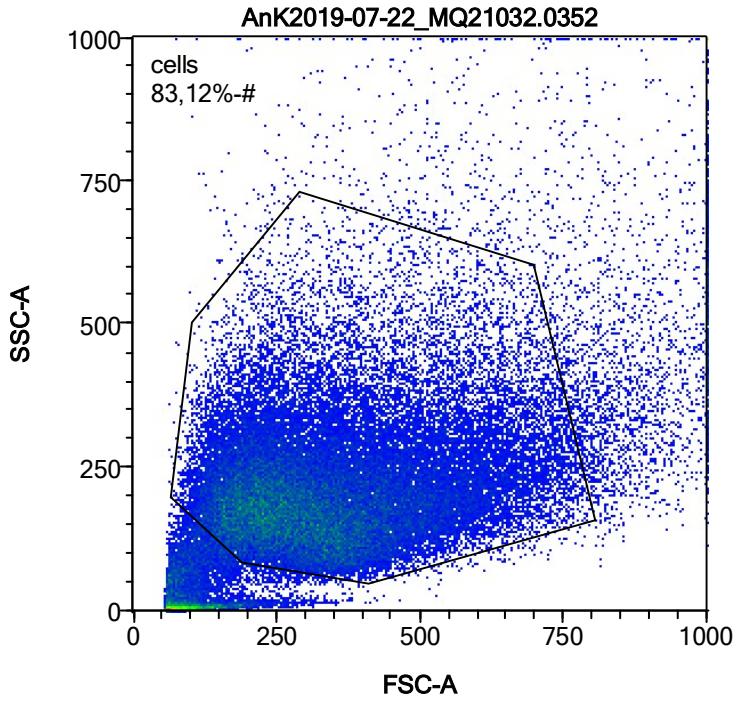

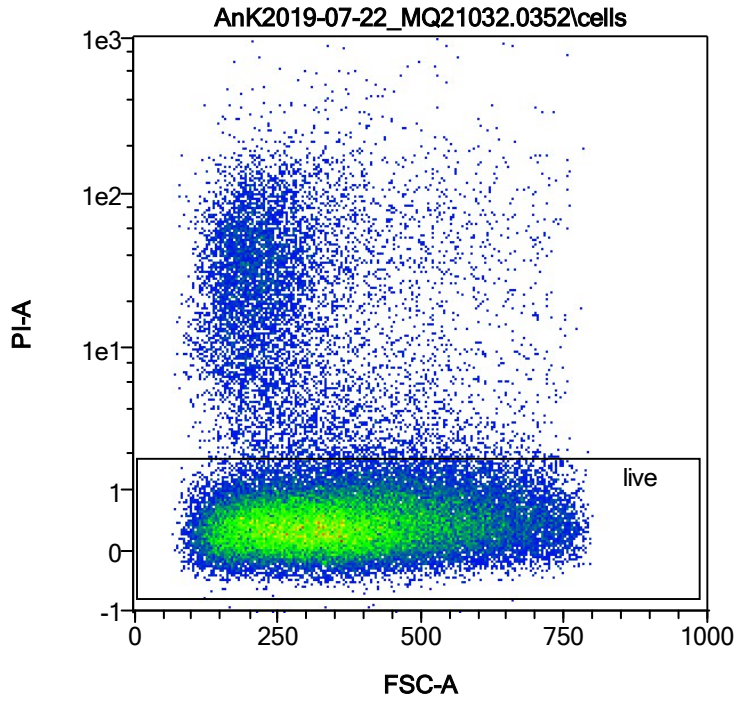

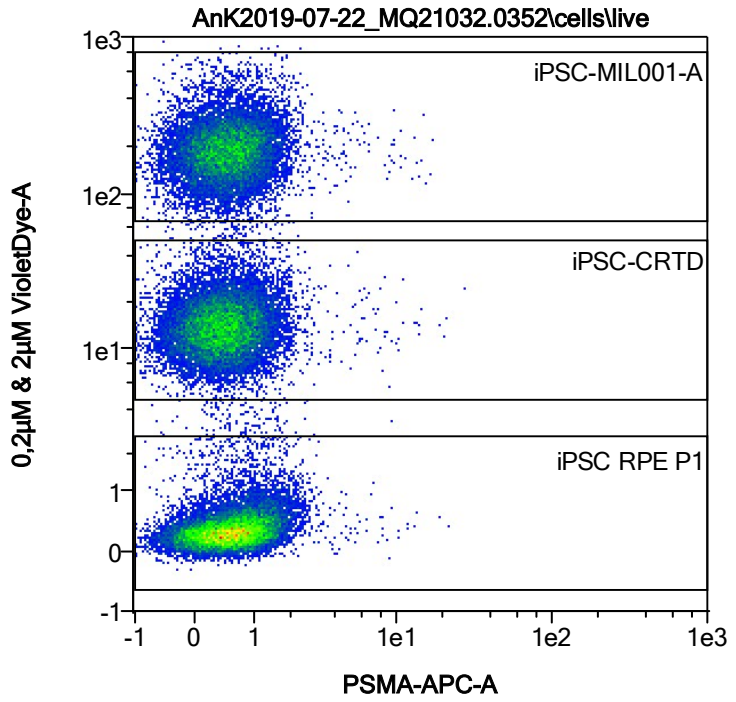

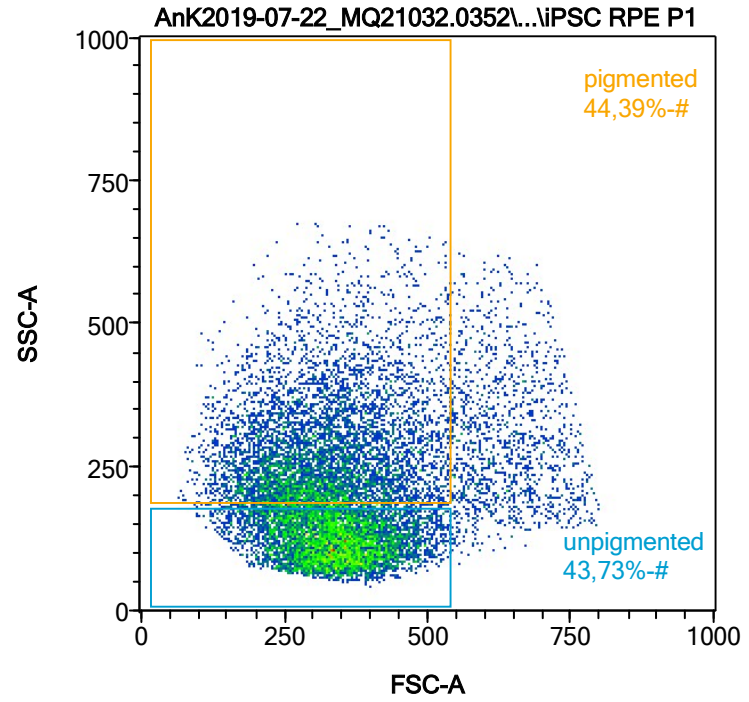

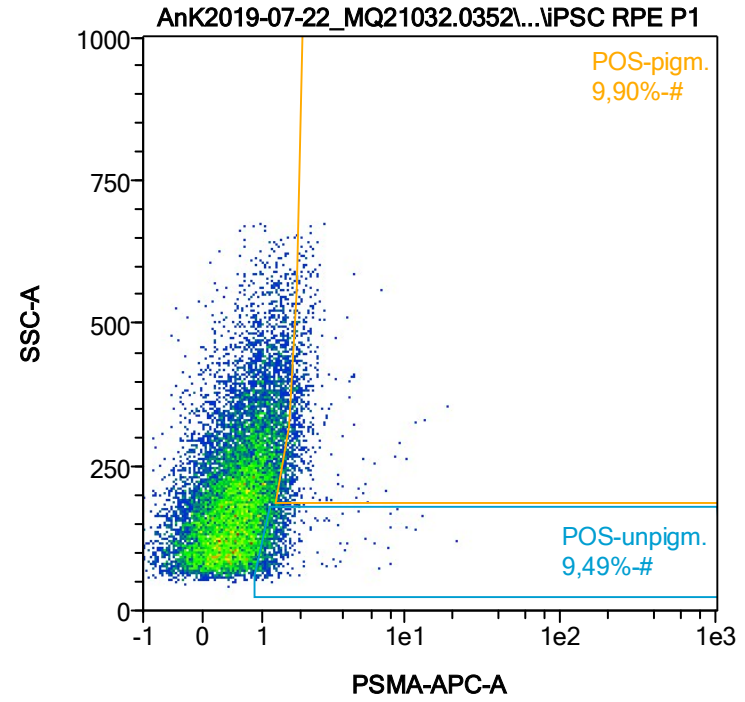

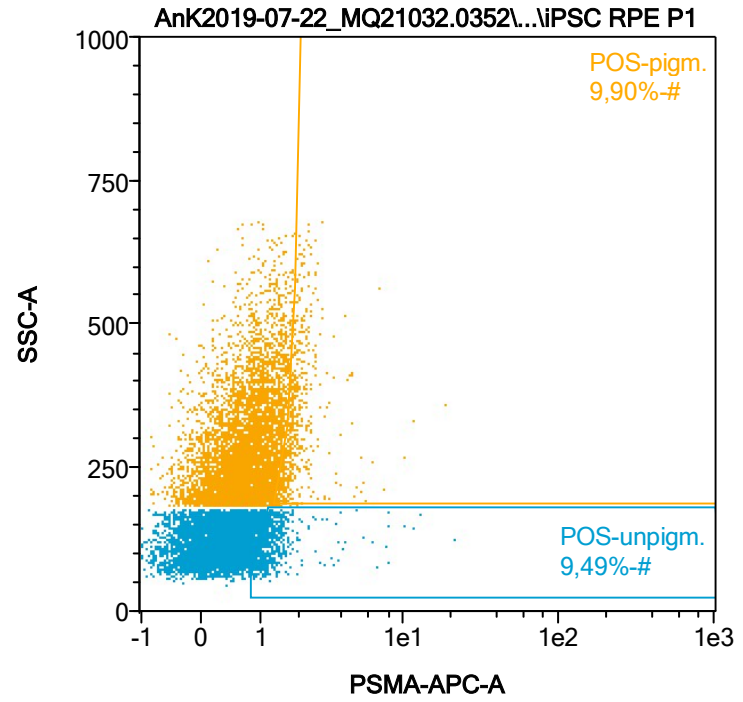

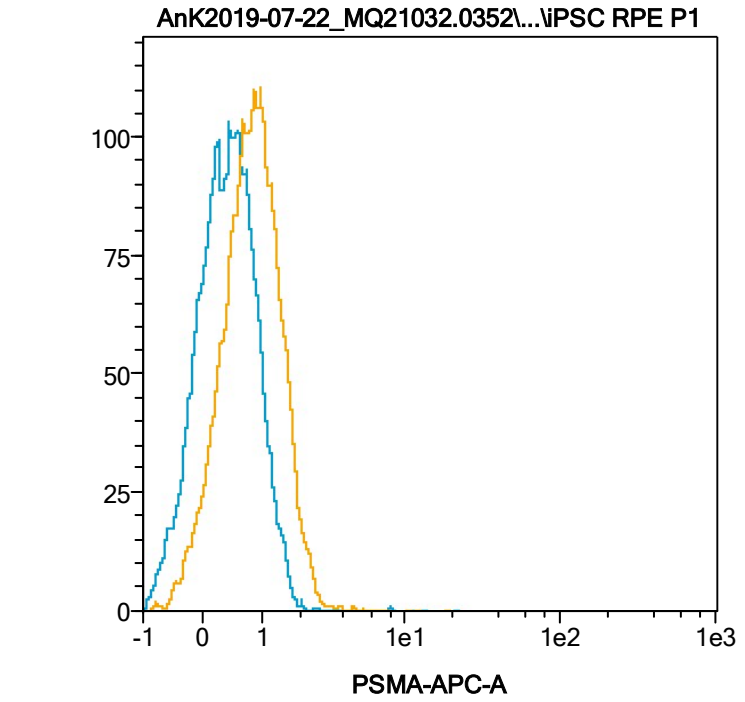

Name	%-#	PSMA-APC-A Mean	PSMA-APC-A Median
POS-unpigm.	9,49	1,47	1,22
POS-pigm.	9,90	2,03	1,83

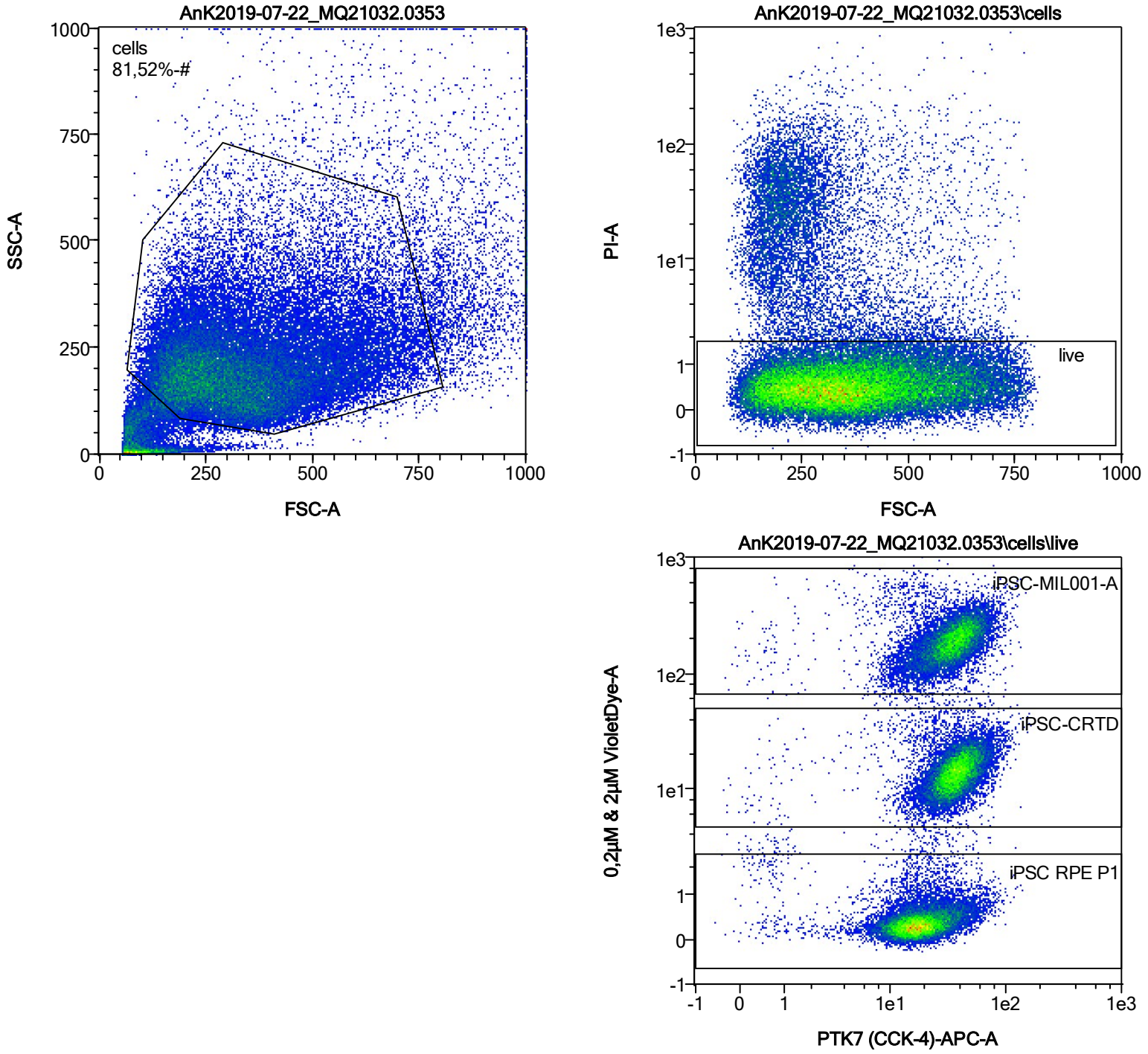

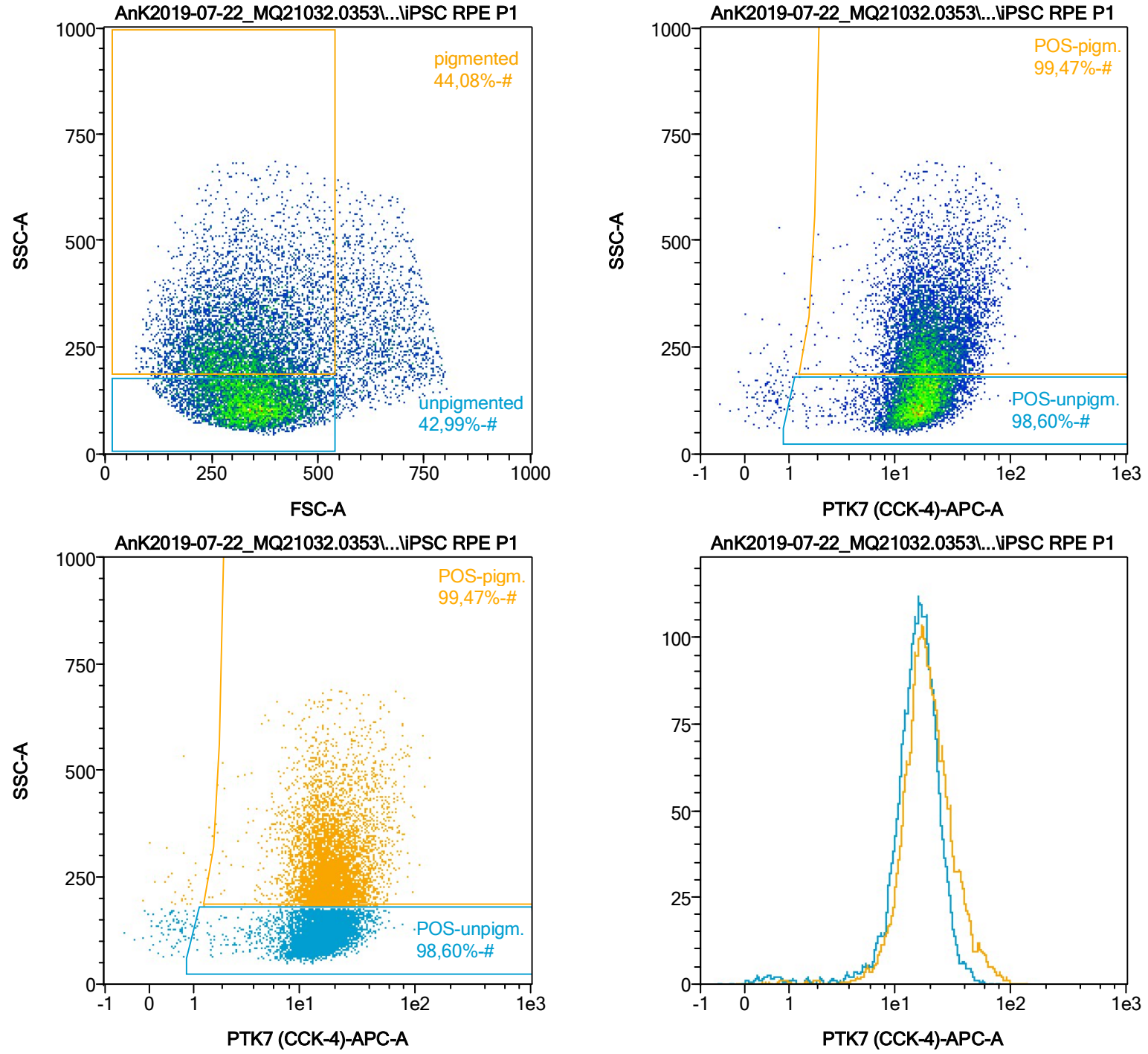

Name	%-#	PTK7 (CCK-4)-APC-A Mean	PTK7 (CCK-4)-APC-A Median
POS-unpigm.	98,60	16,73	15,86
POS-pigm.	99,47	21,27	18,25

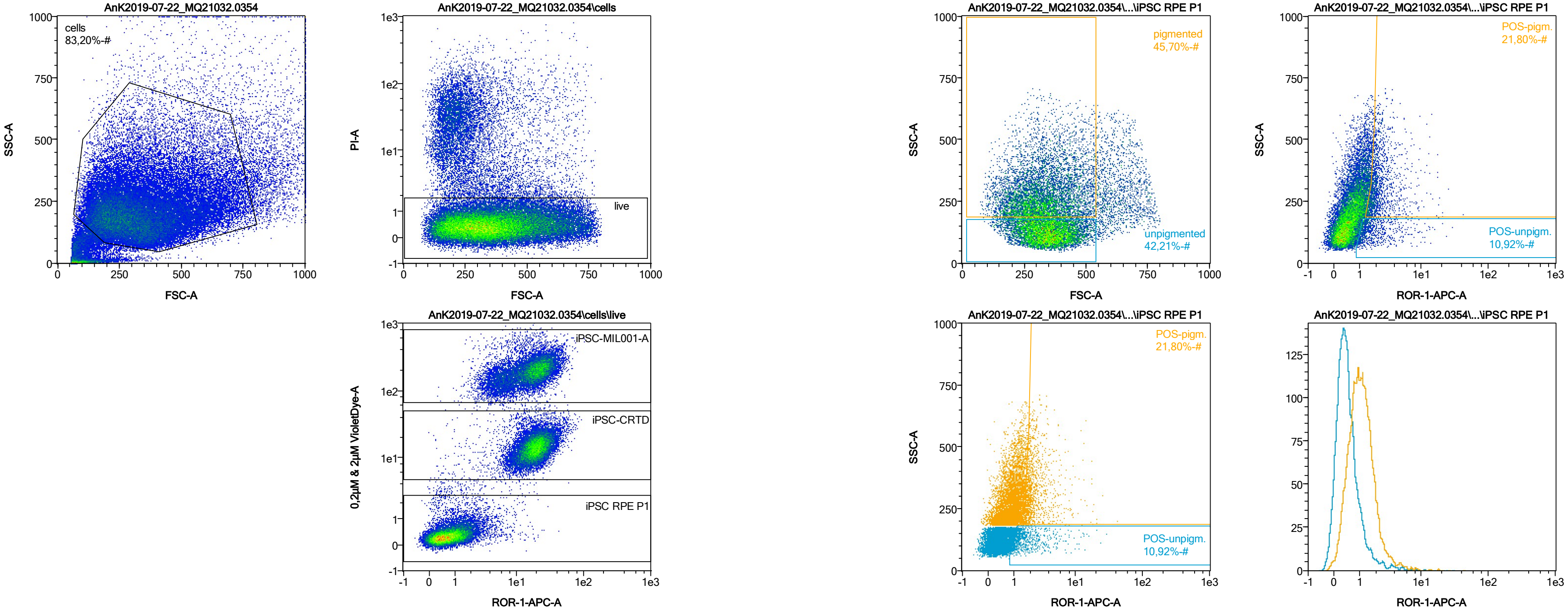

Name	%-#	ROR-1-APC-A Mean	ROR-1-APC-A Median
POS-unpigm.	10,92	2,20	1,45
POS-pigm.	21,80	2,58	1,99

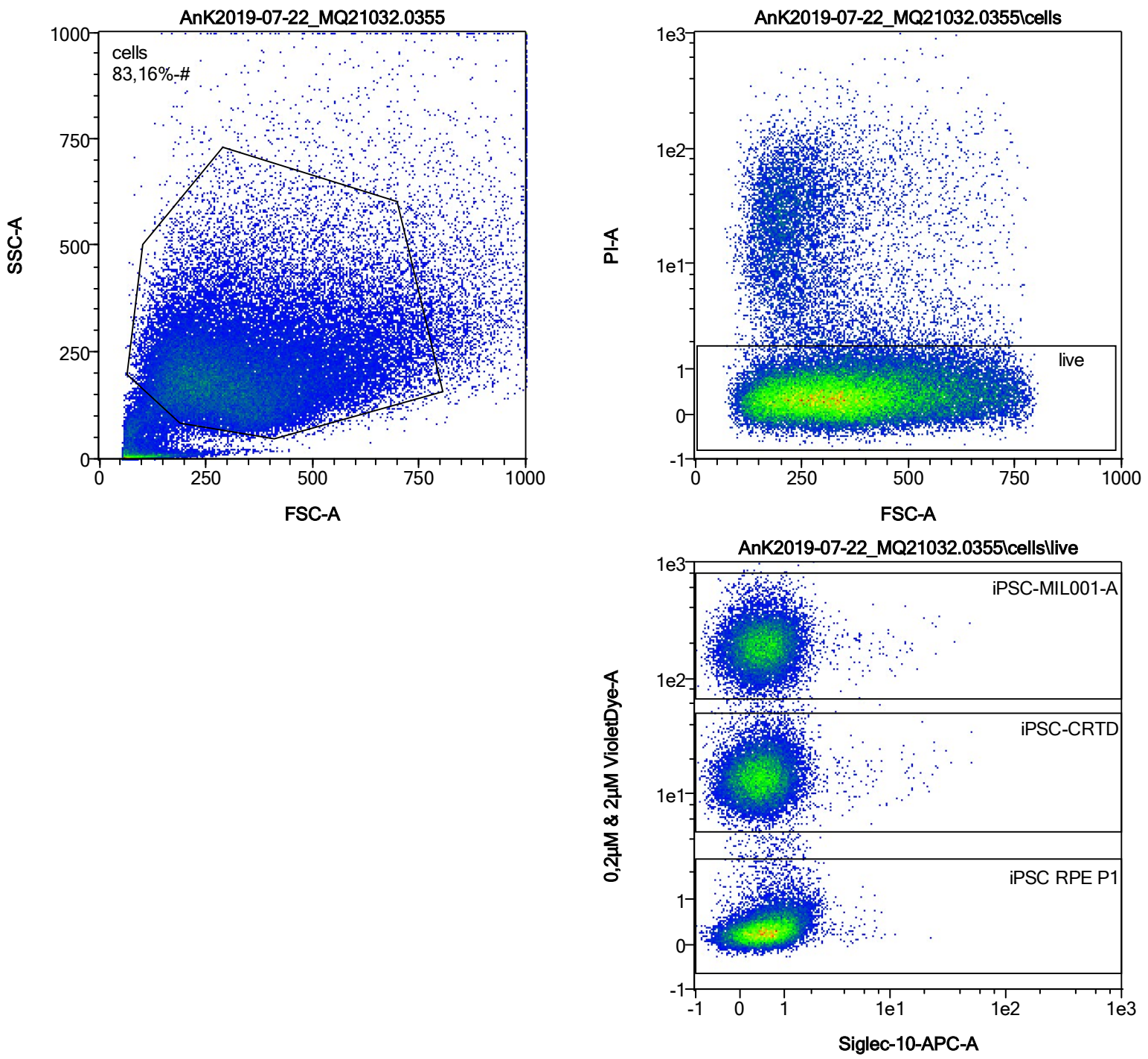

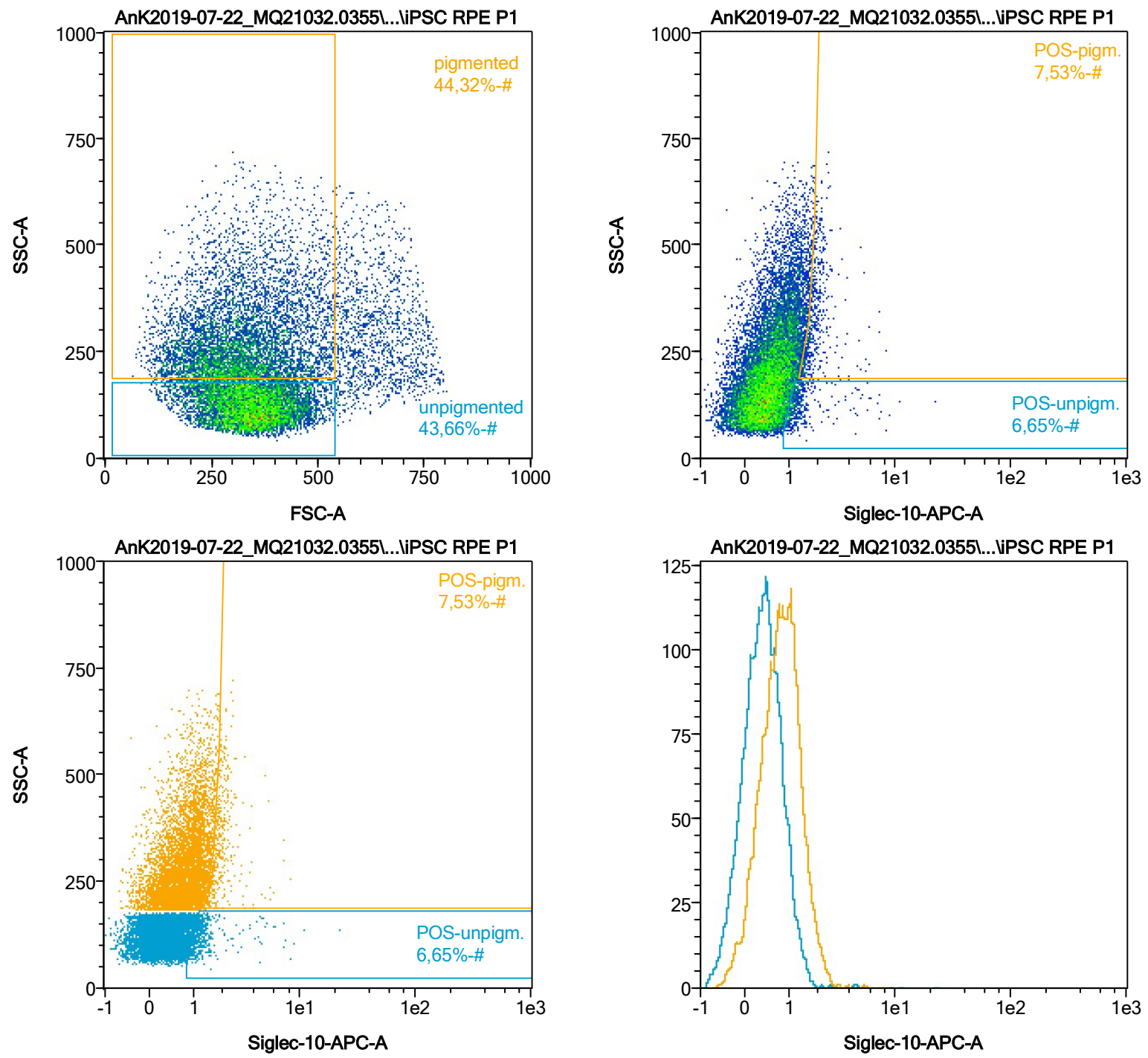

Name	%-#	Siglec-10-APC-A Mean	Siglec-10-APC-A Median
POS-unpigm.	6,65	1,55	1,21
POS-pigm.	7,53	1,99	1,84

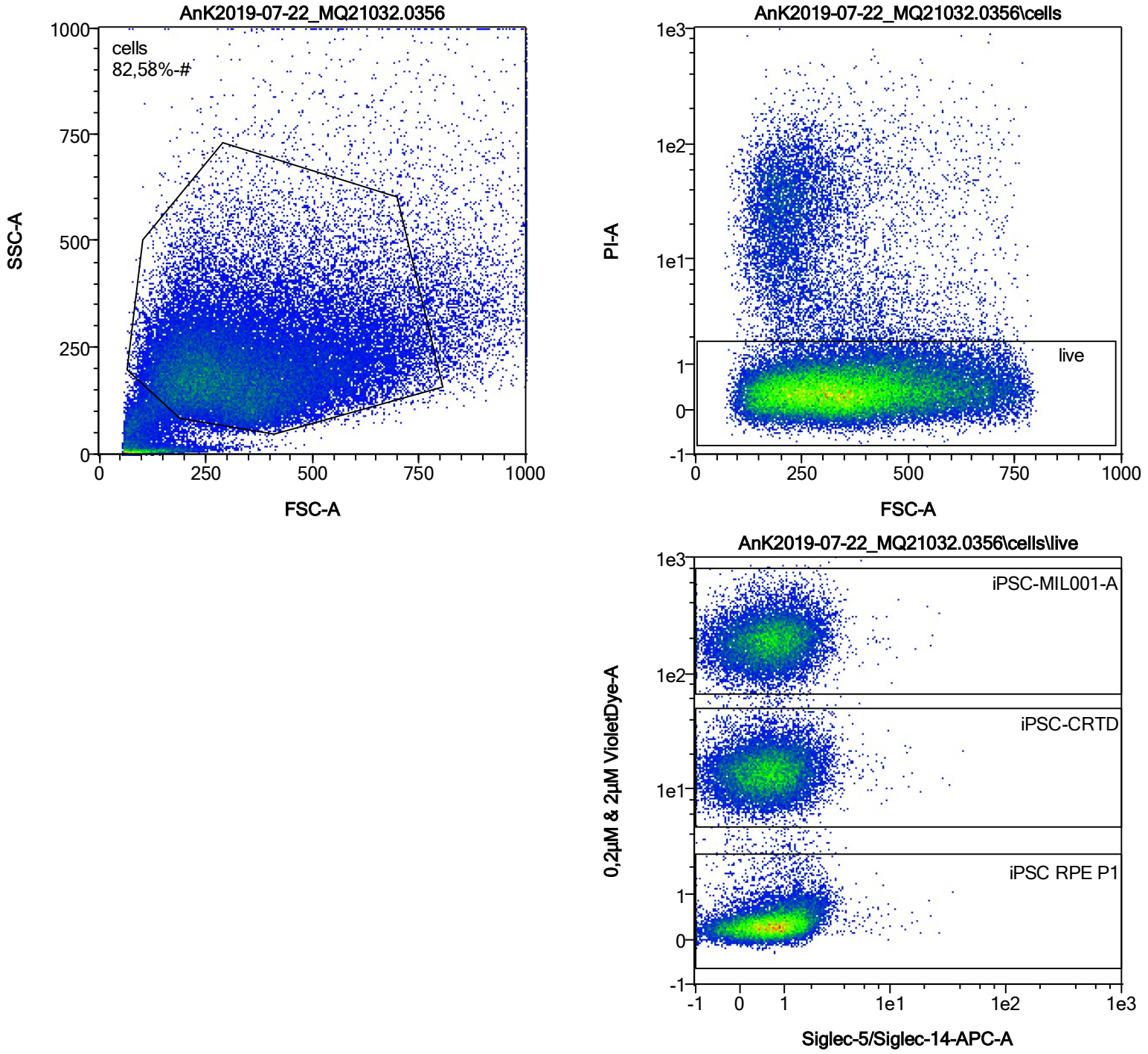

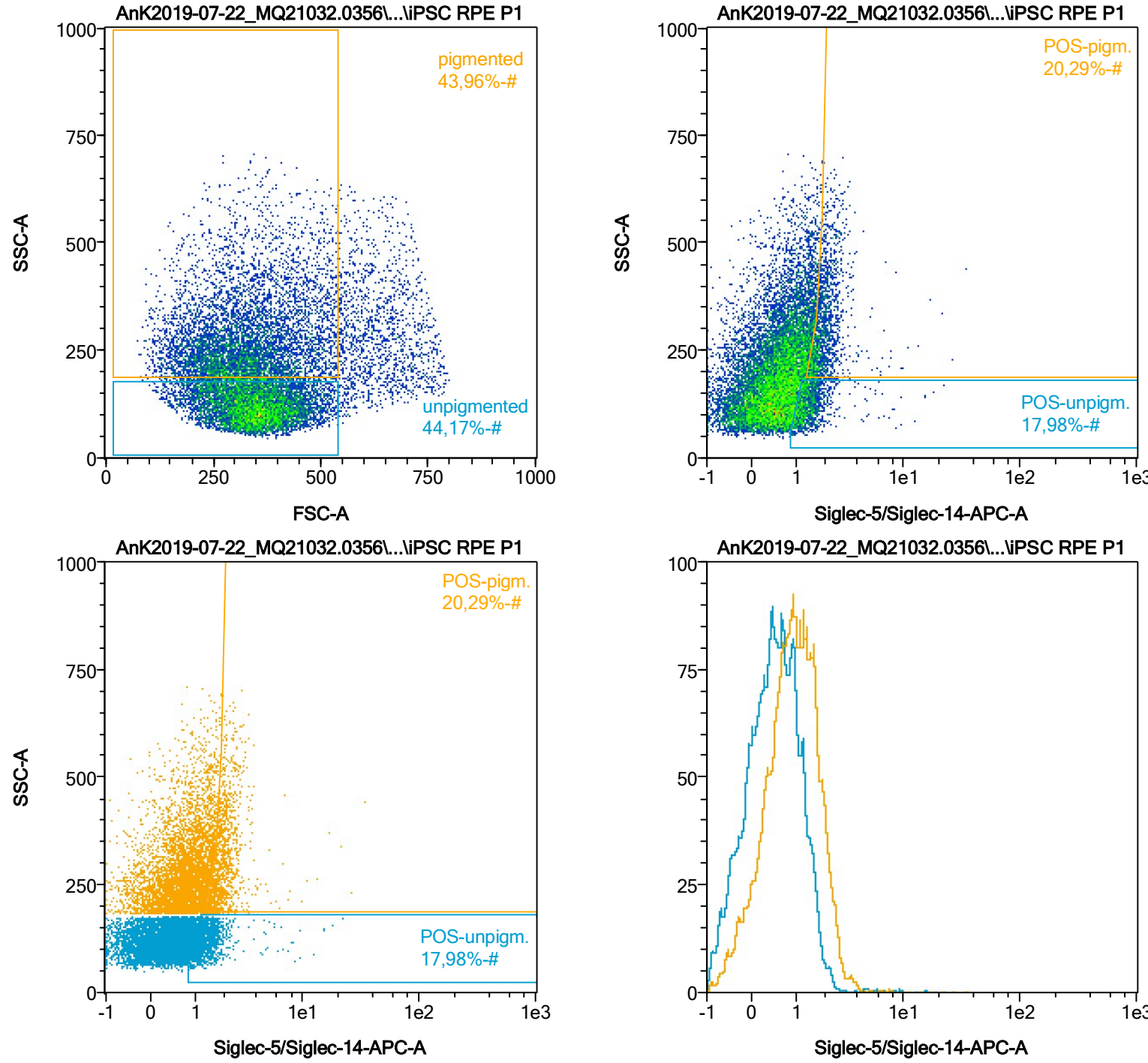

Name	%-#	Siglec-5/Siglec-14-APC-A Mean	Siglec-5/Siglec-14-APC-A Median
POS-unpigm.	17,98	1,51	1,28
POS-pigm.	20,29	2,11	1,90

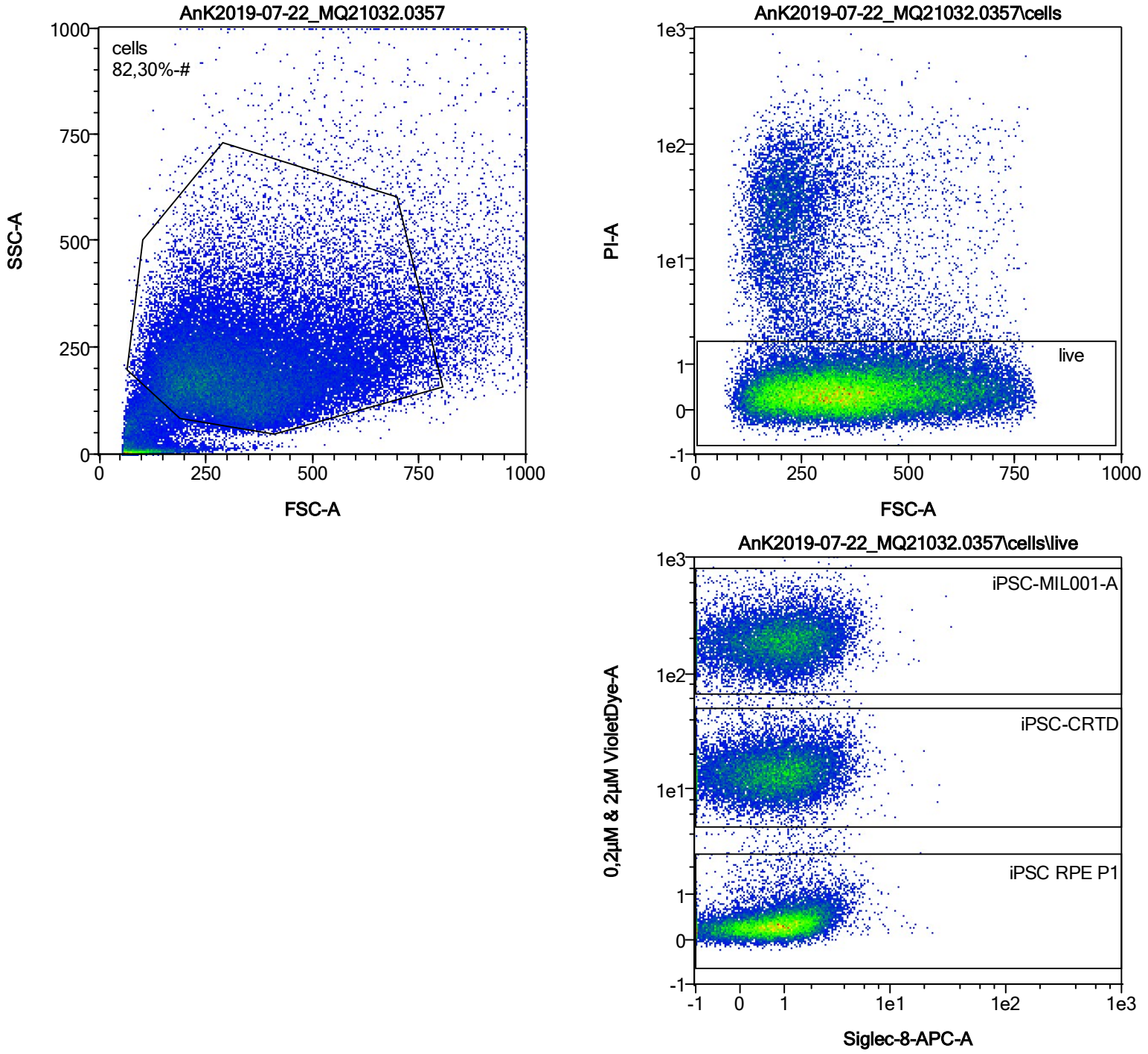

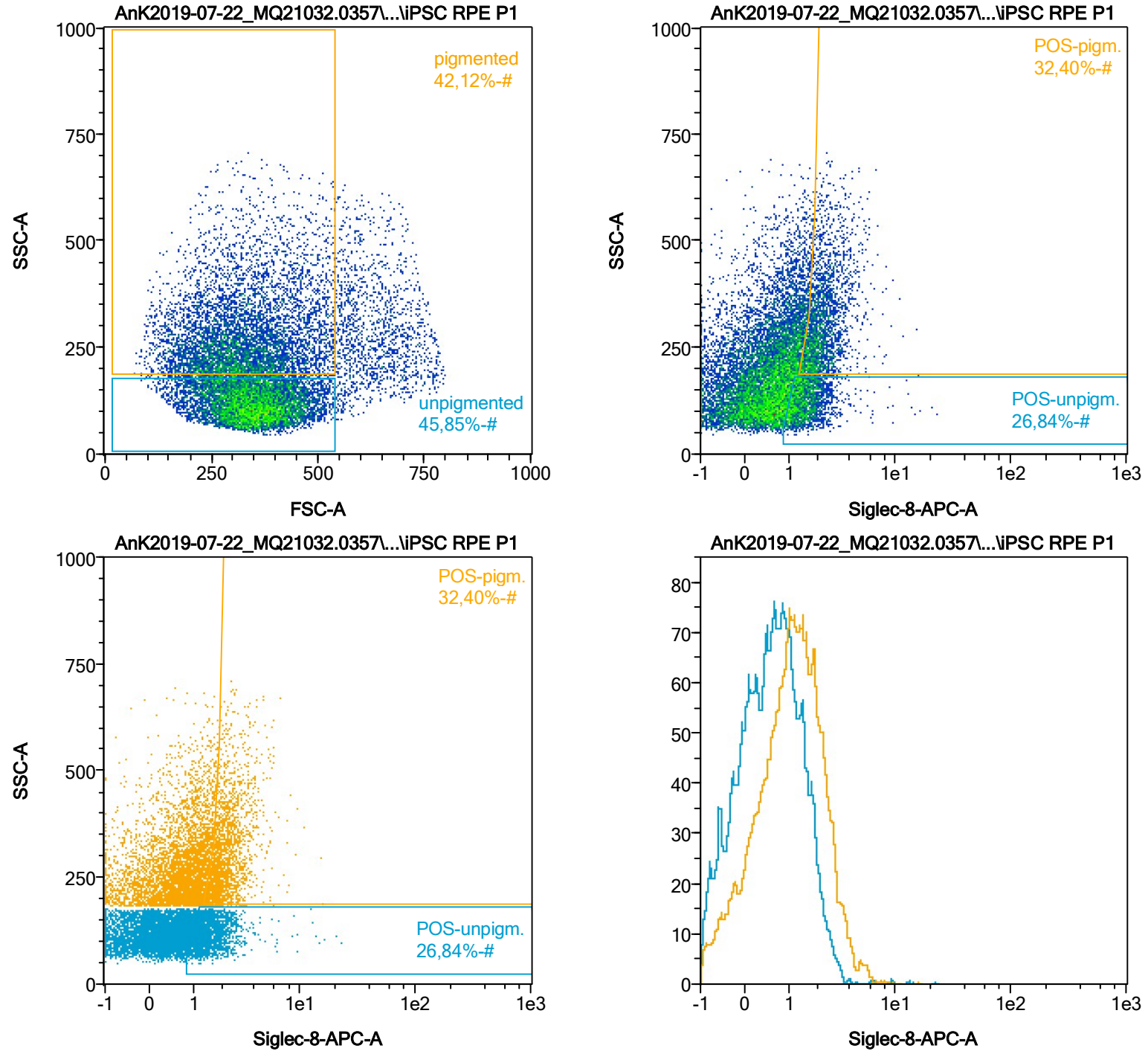

Name	%-#	Siglec-8-APC-A Mean	Siglec-8-APC-A Median
POS-unpigm.	26,84	1,66	1,44
POS-pigm.	32,40	2,35	2,10

Date:2024-06-14
Operator: Sebastian Knoebel
Version:2.13.0+54.g95abf0f4

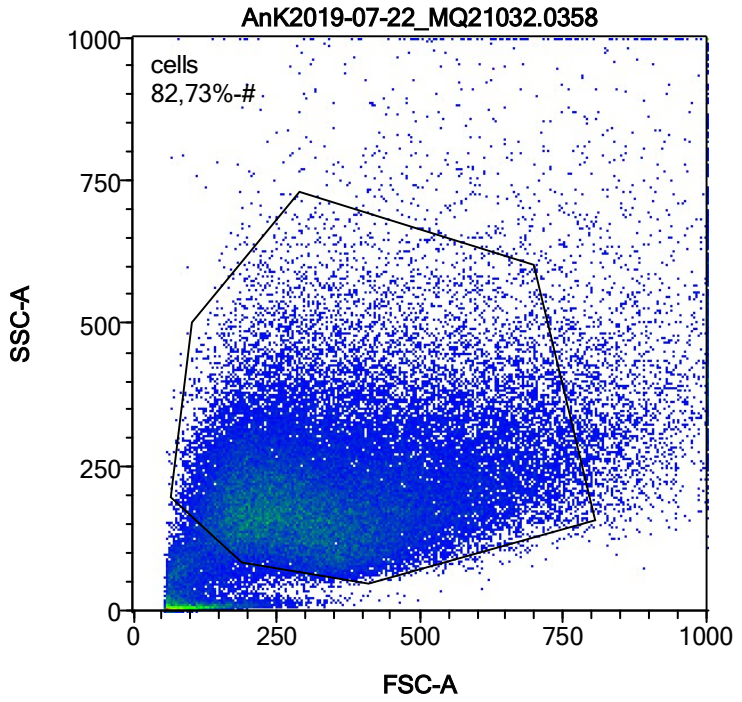

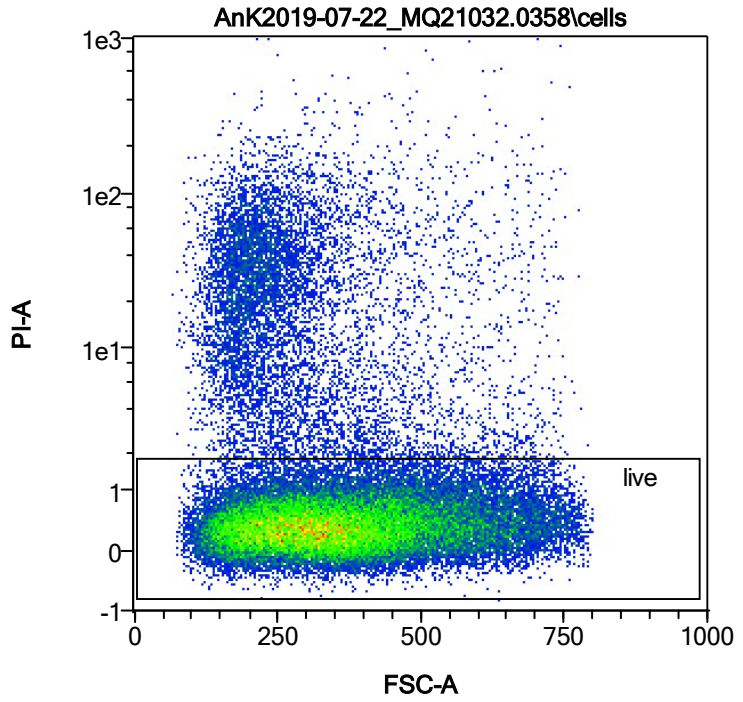

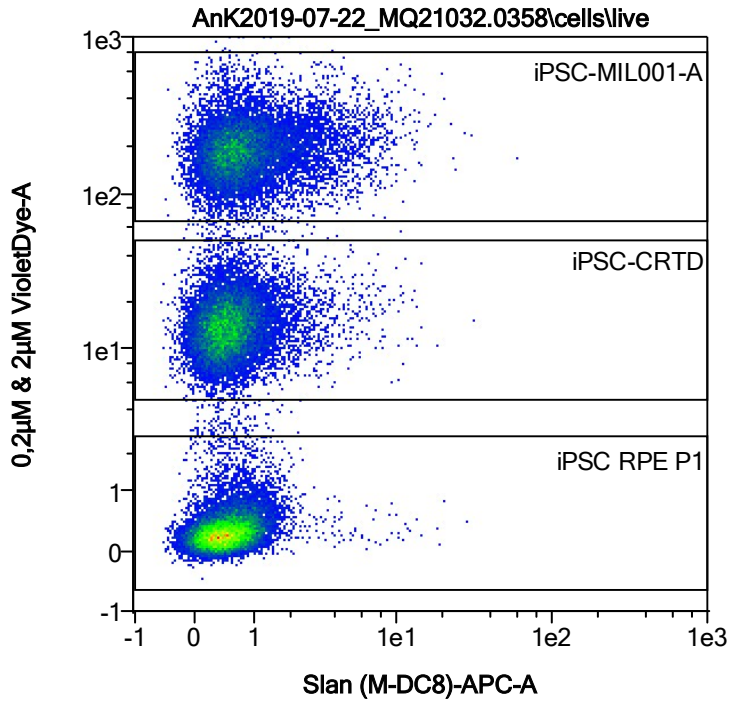

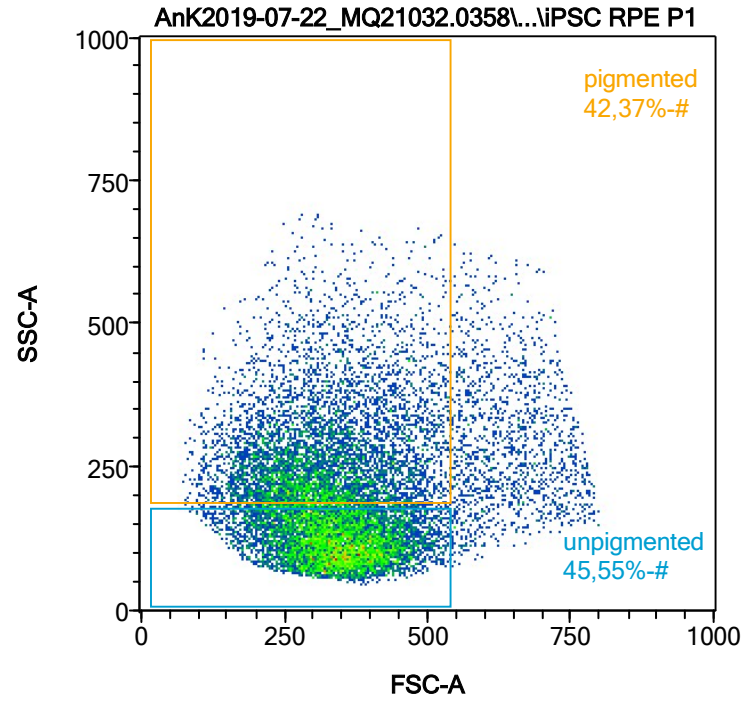

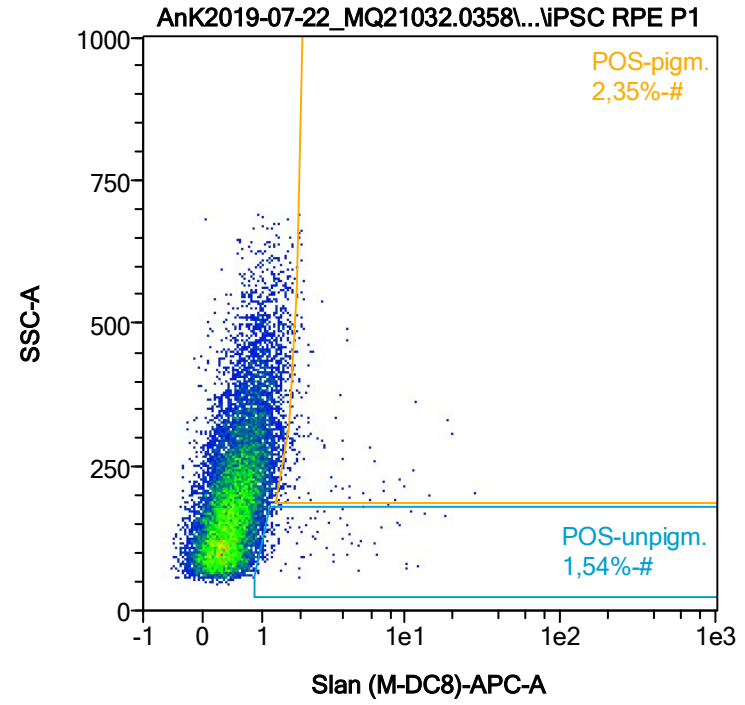

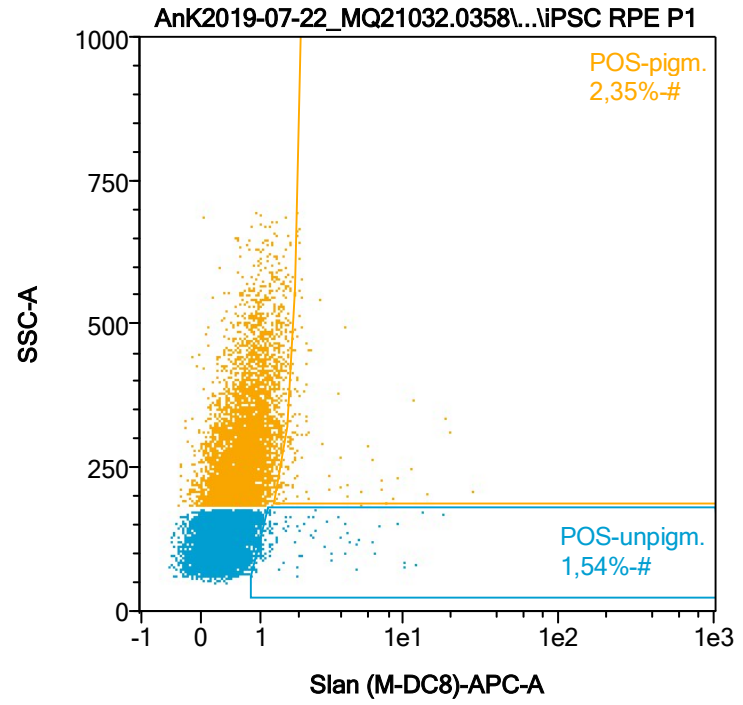

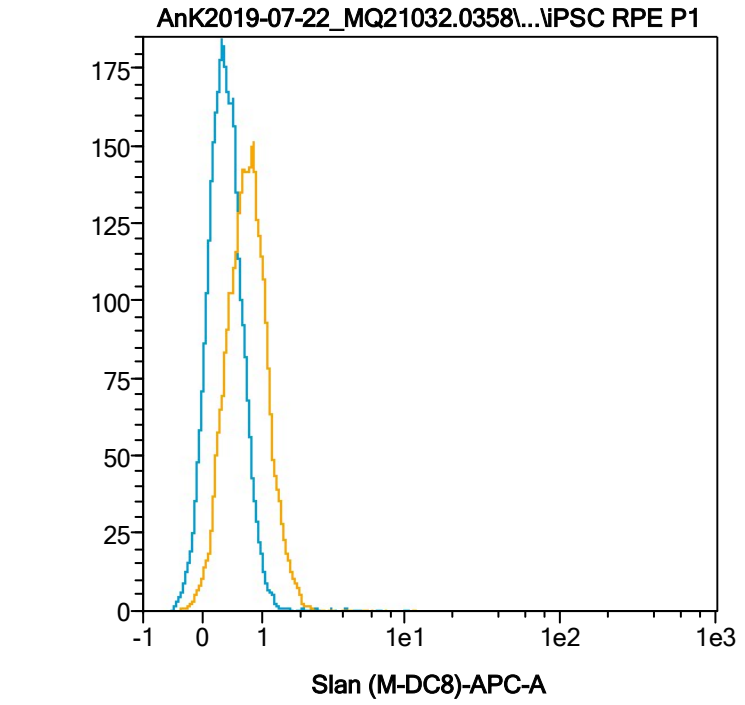

Name	%-#	Slan (M-DC8)-APC-A Mean	Slan (M-DC8)-APC-A Median
POS-unpigm.	1,54	2,53	1,30
POS-pigm.	2,35	2,91	1,87

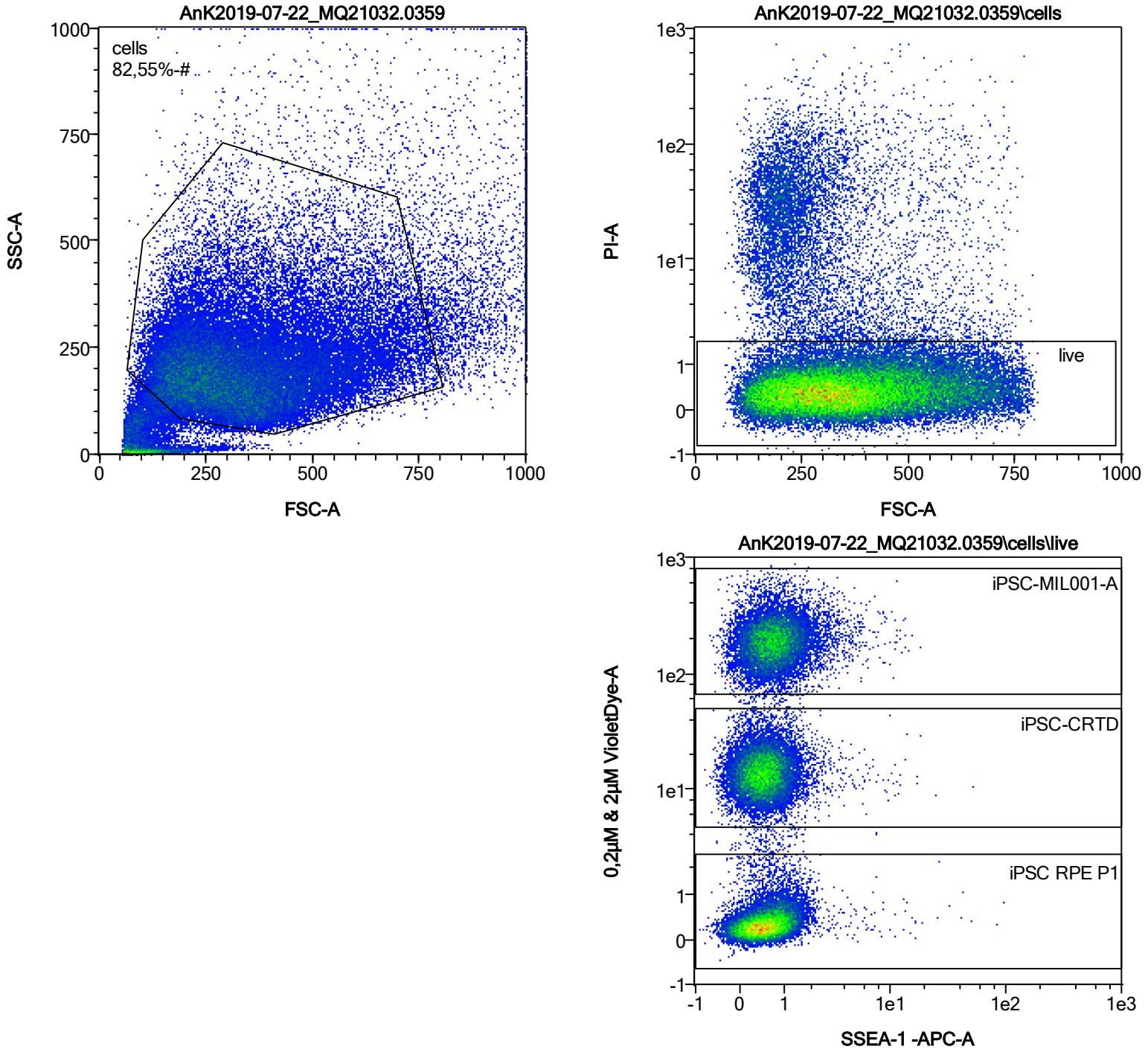

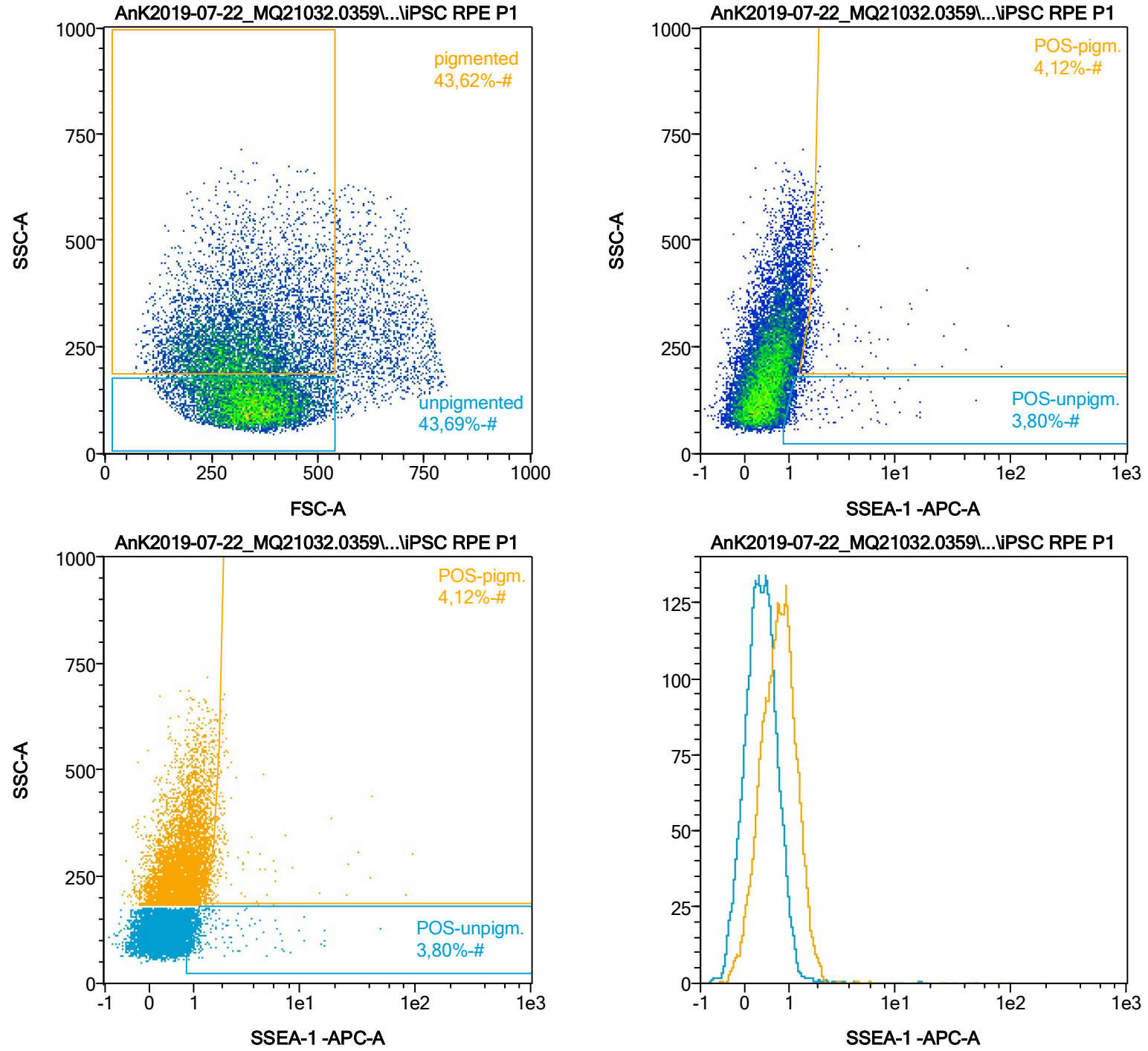

Name	%-#	SSEA-1 -APC-A Mean	SSEA-1 -APC-A Median
POS-unpigm.	3,80	2,16	1,17
POS-pigm.	4,12	3,43	1,81

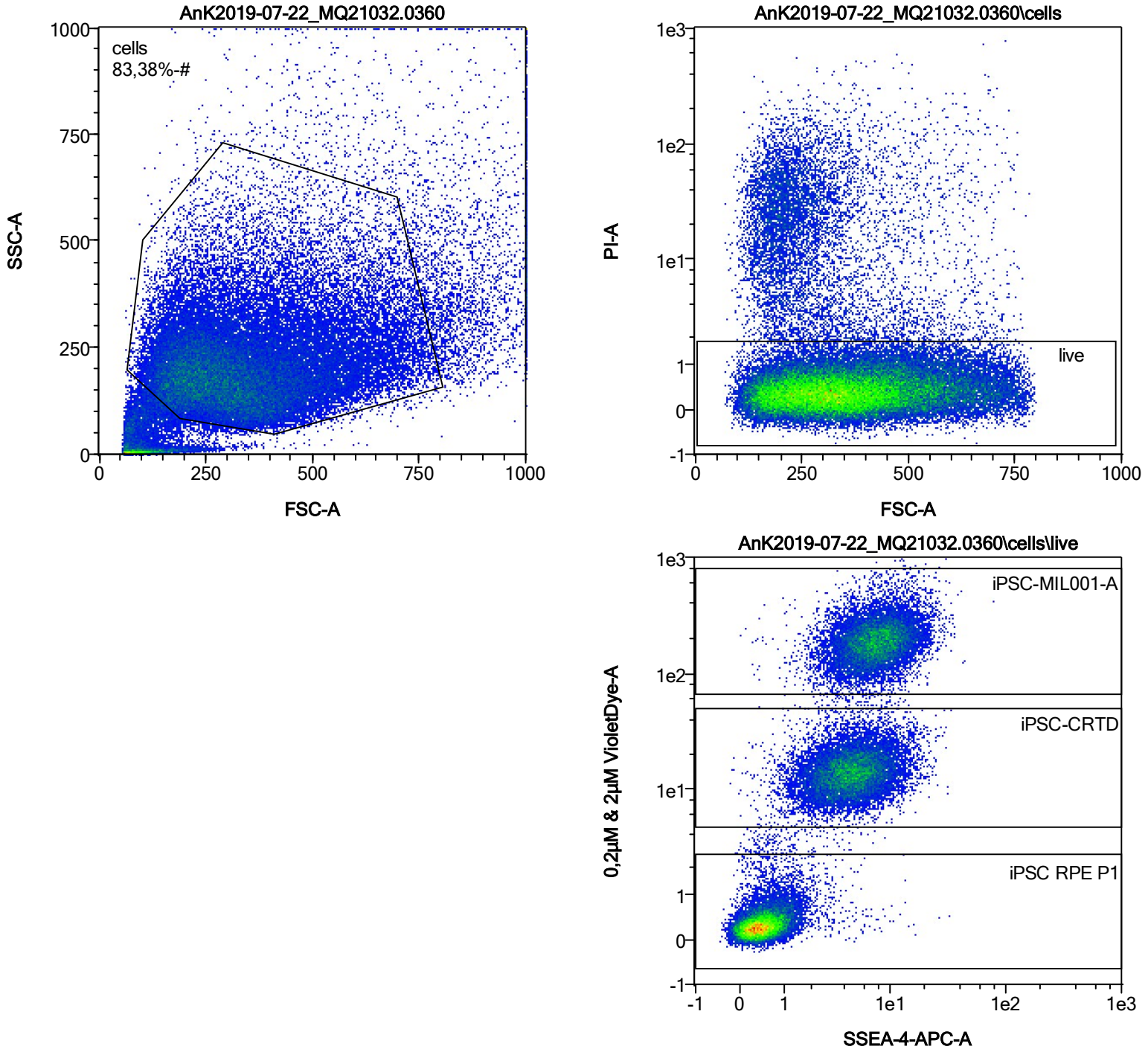

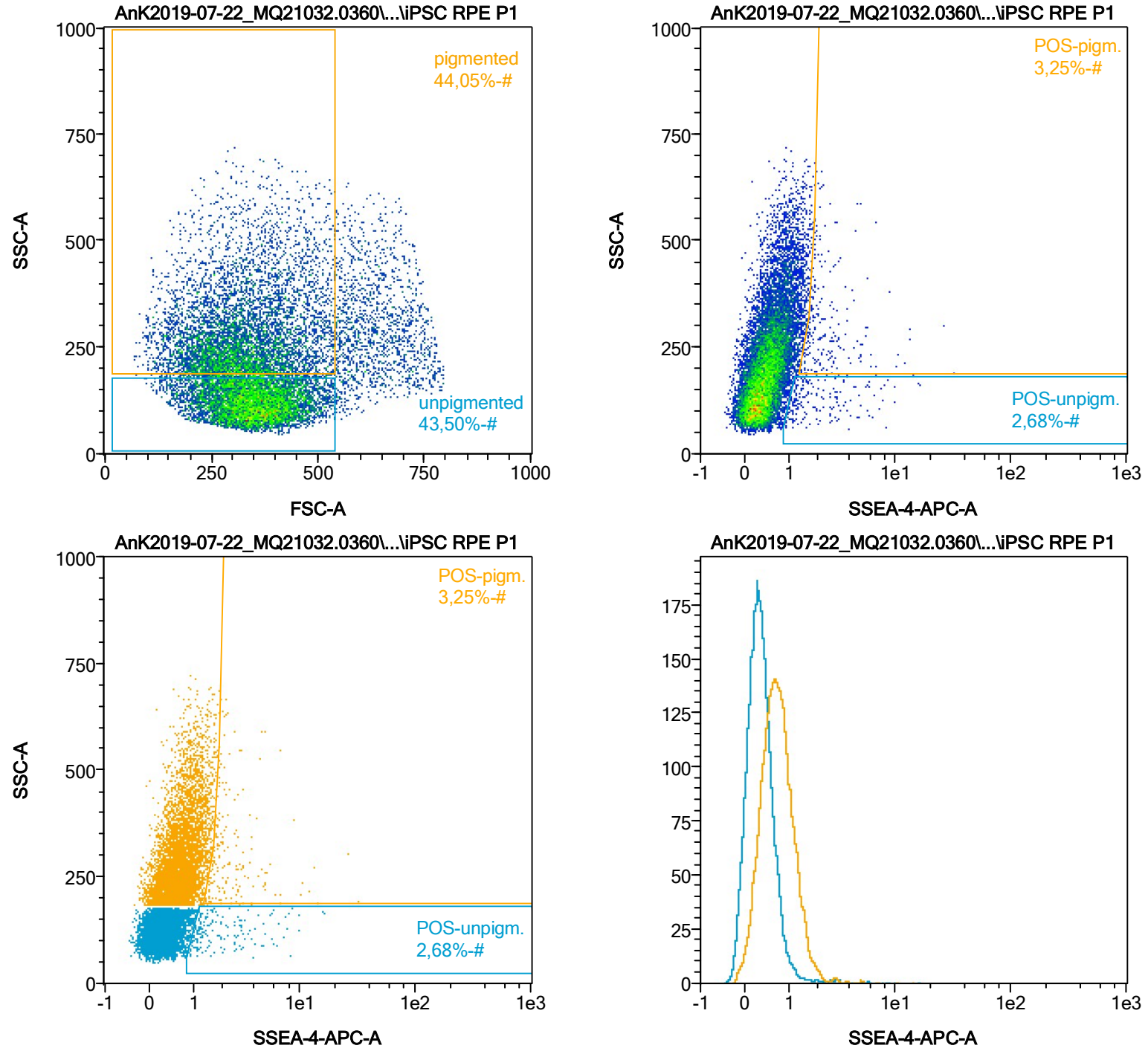

Name	%-#	SSEA-4-APC-A Mean	SSEA-4-APC-A Median
POS-unpigm.	2,68	2,46	1,48
POS-pigm.	3,25	2,89	2,01

Date:2024-06-14

Operator: Sebastian Knoebel

Version:2.13.0+54.g95abf0f4

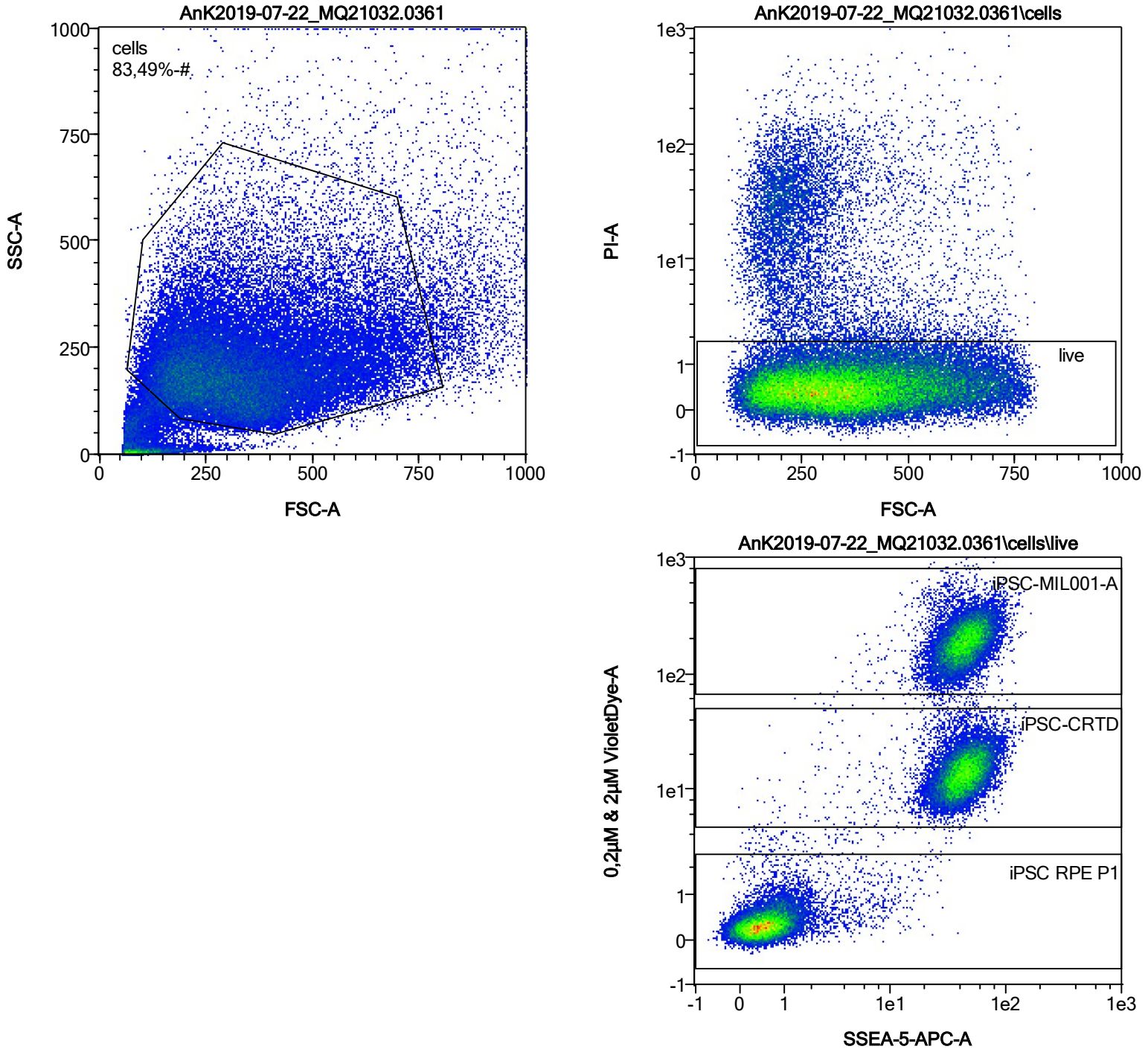

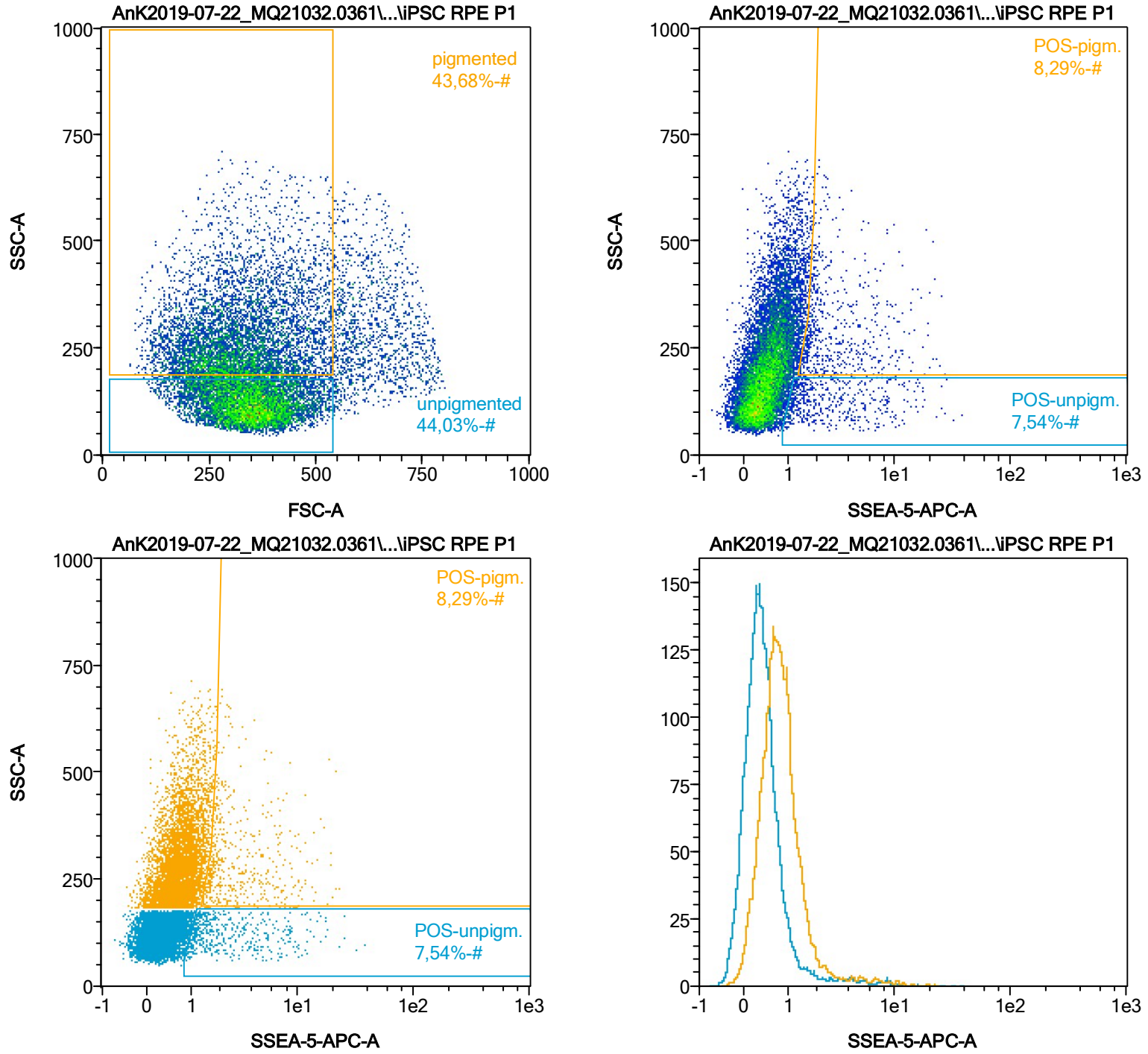

Name	%-#	SSEA-5-APC-A Mean	SSEA-5-APC-A Median
POS-unpigm.	7,54	3,43	1,78
POS-pigm.	8,29	3,75	2,29

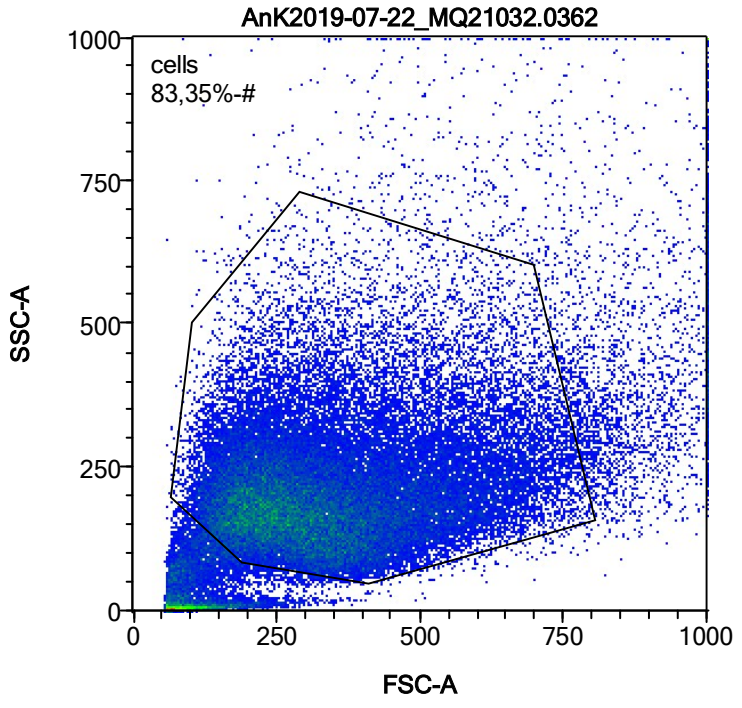

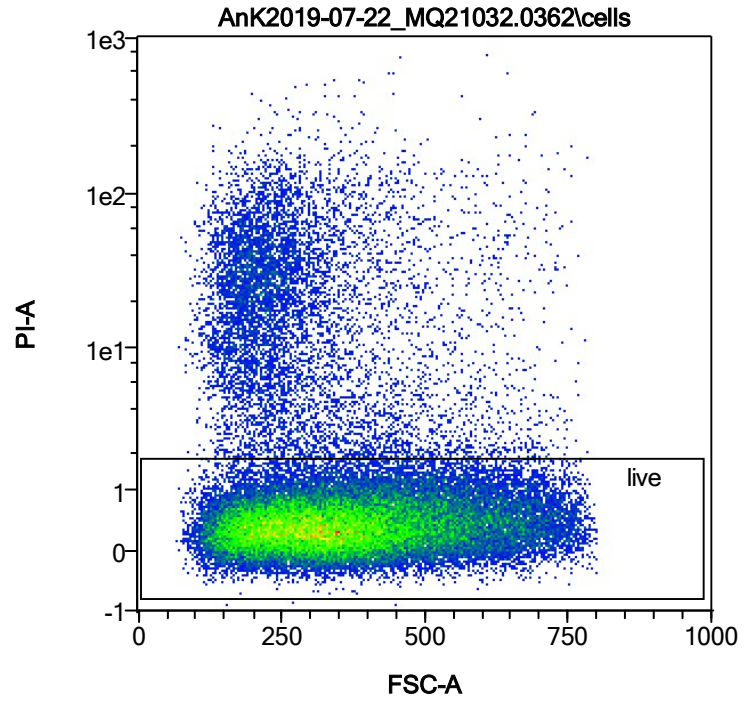

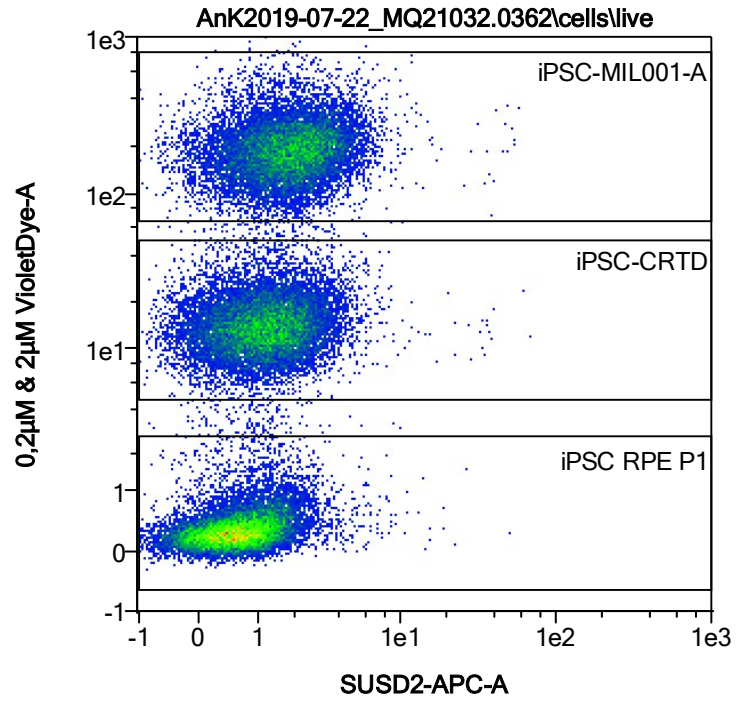

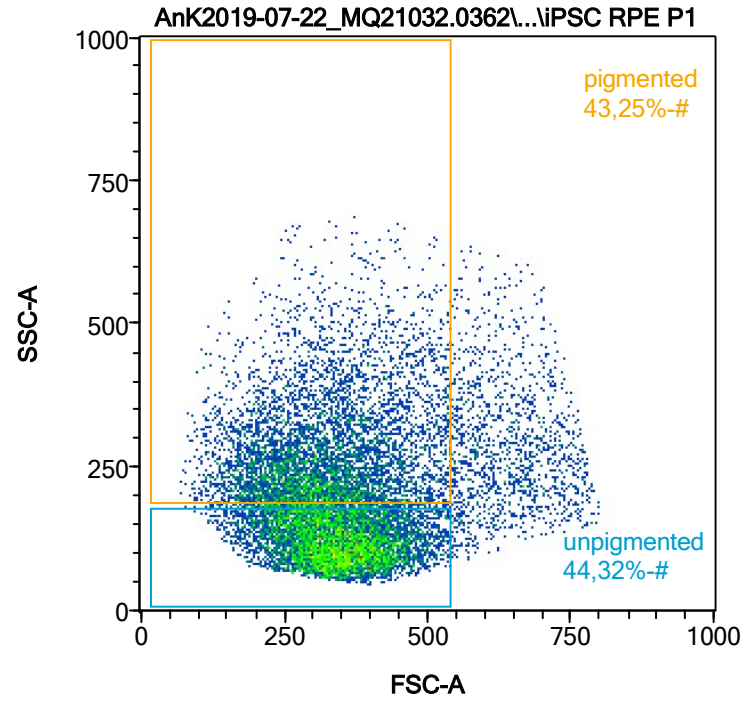

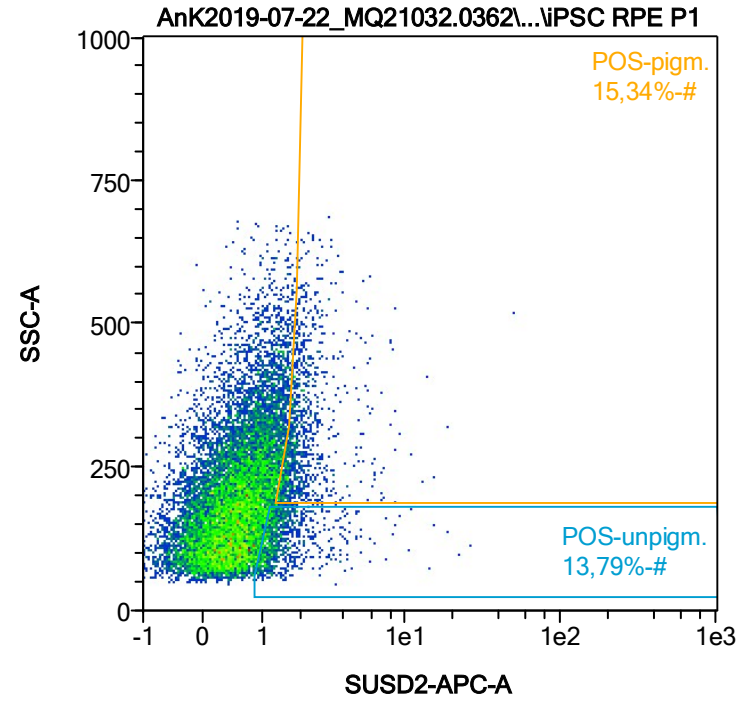

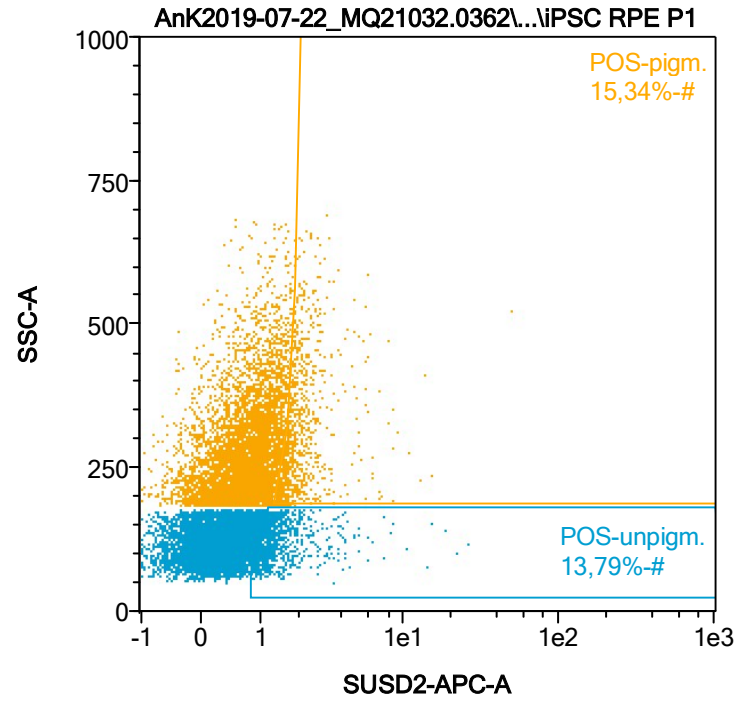

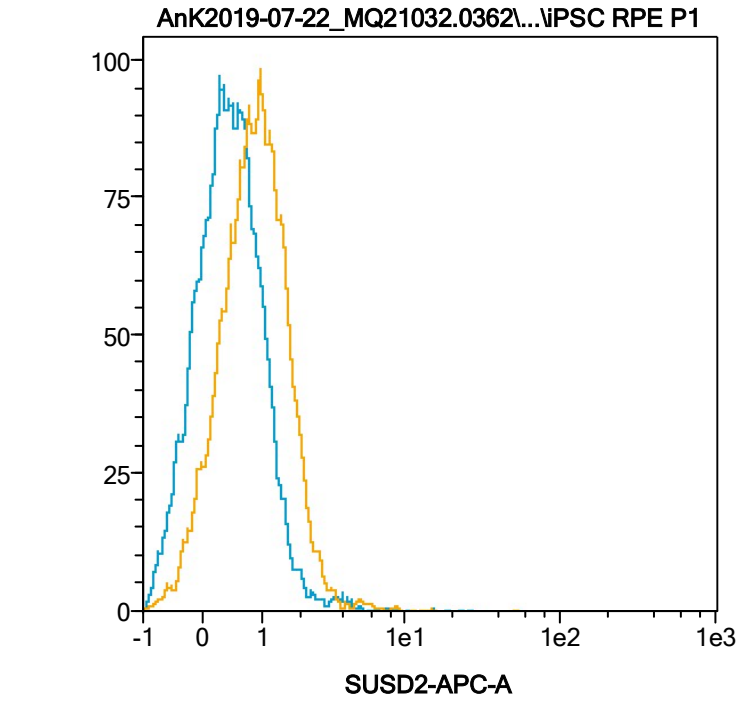

Name	%-#	SUSD2-APC-A Mean	SUSD2-APC-A Median
POS-unpigm.	13,79	1,65	1,28
POS-pigm.	15,34	2,24	1,87

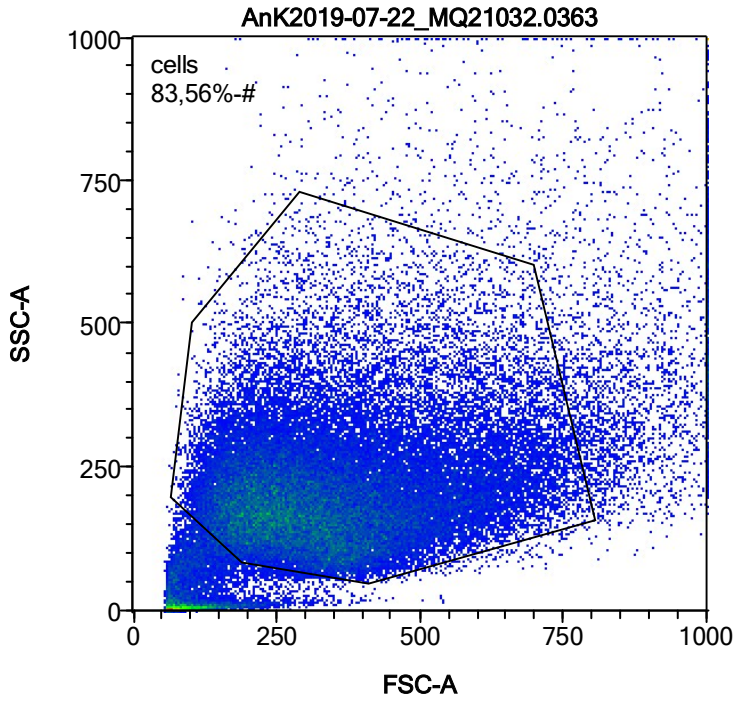

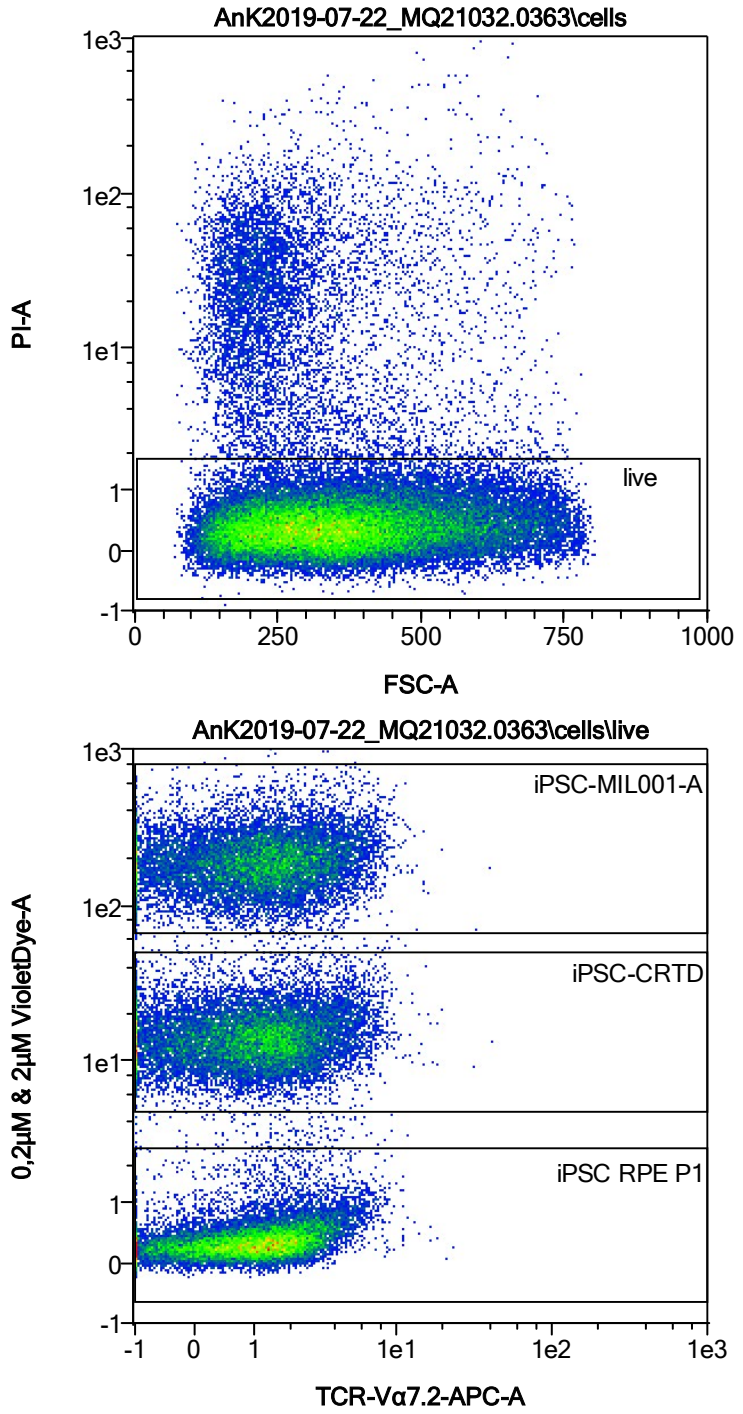

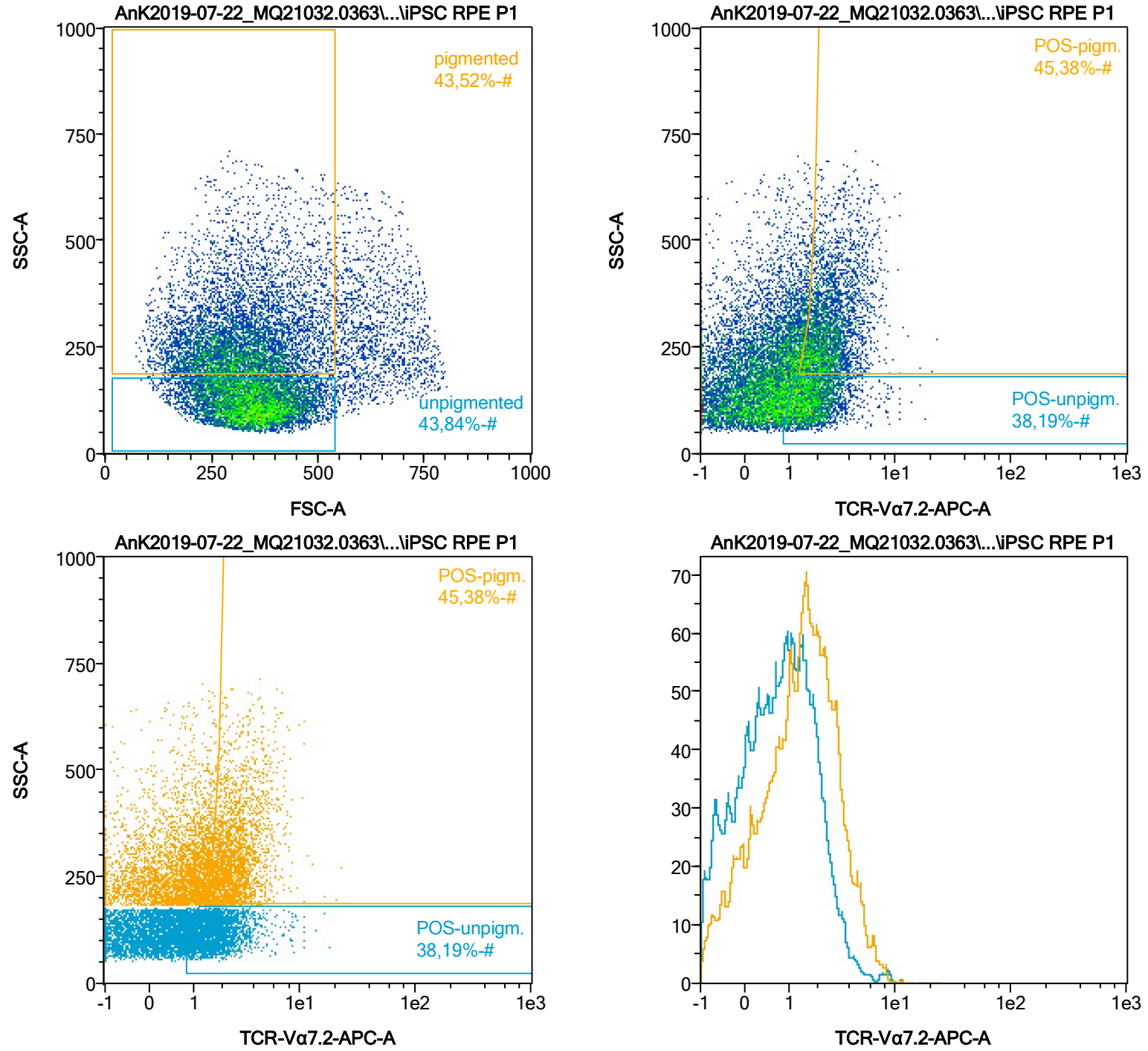

Name	%-#	TCR-Vα7.2-APC-A Mean	TCR-Vα7.2-APC-A Median
POS-unpigm.	38,19	1,95	1,66
POS-pigm.	45,38	2,79	2,41

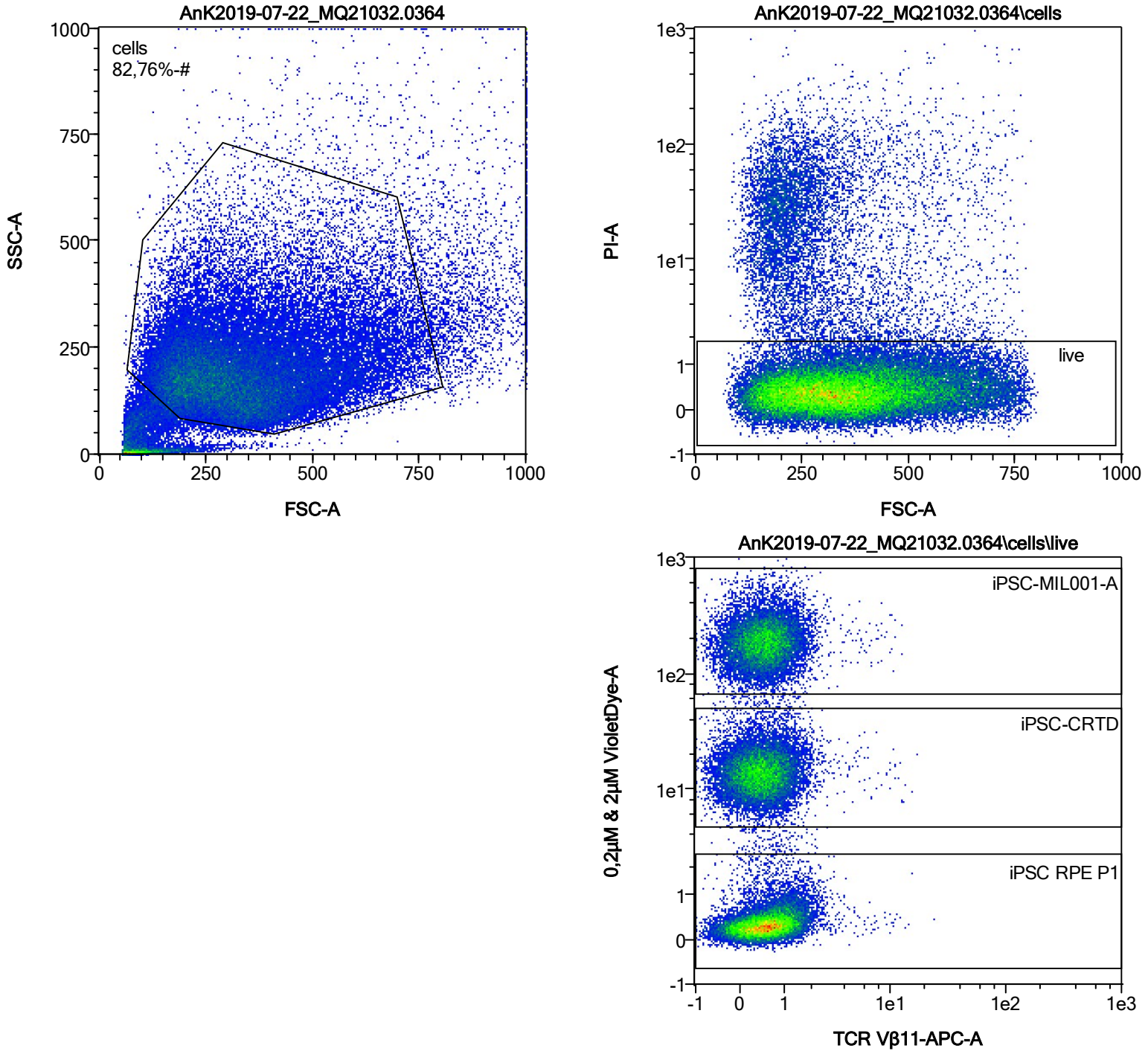

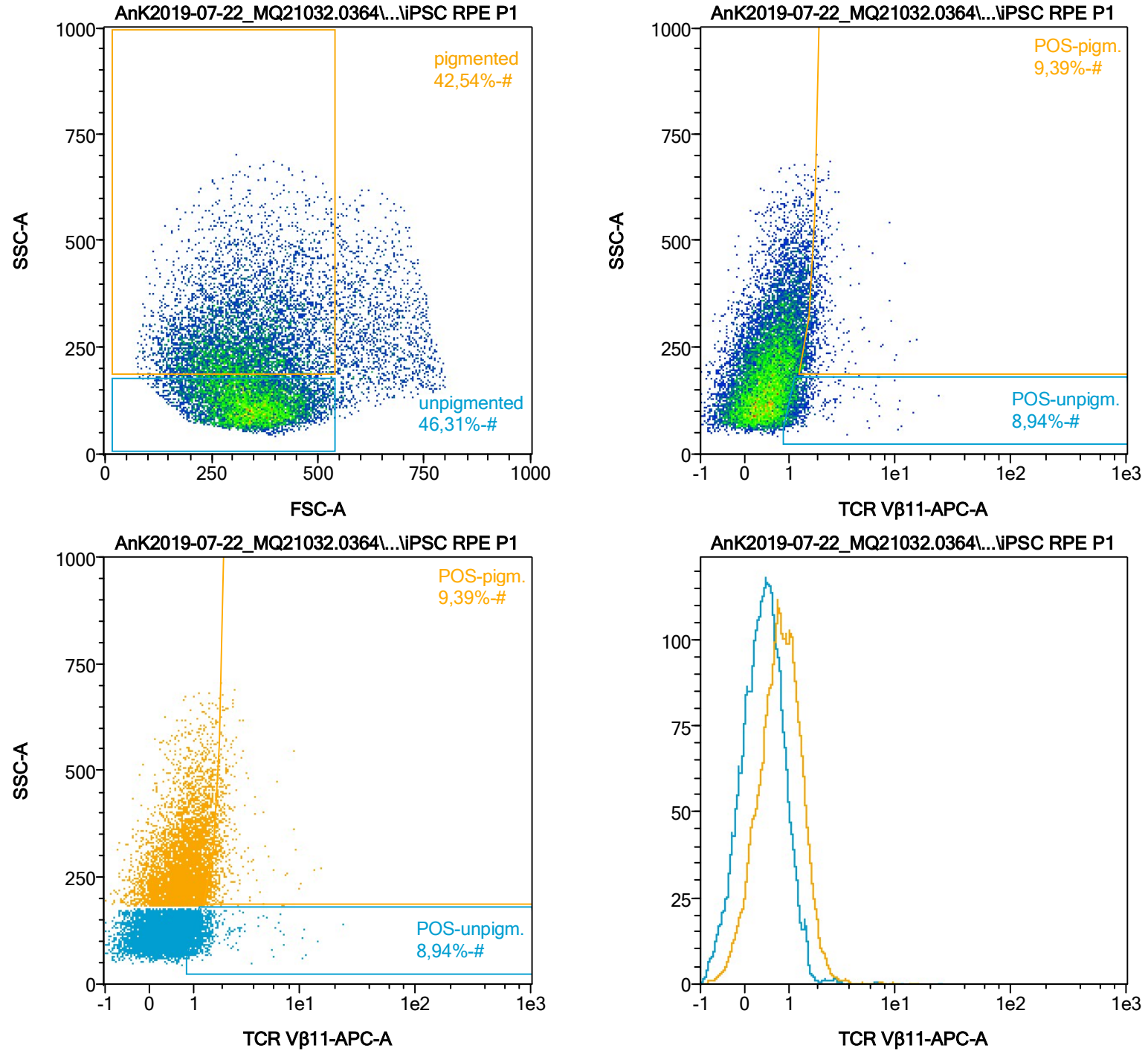

Name	%-#	TCR Vβ11-APC-A Mean	TCR Vβ11-APC-A Median
POS-unpigm.	8,94	1,53	1,23
POS-pigm.	9,39	2,09	1,82

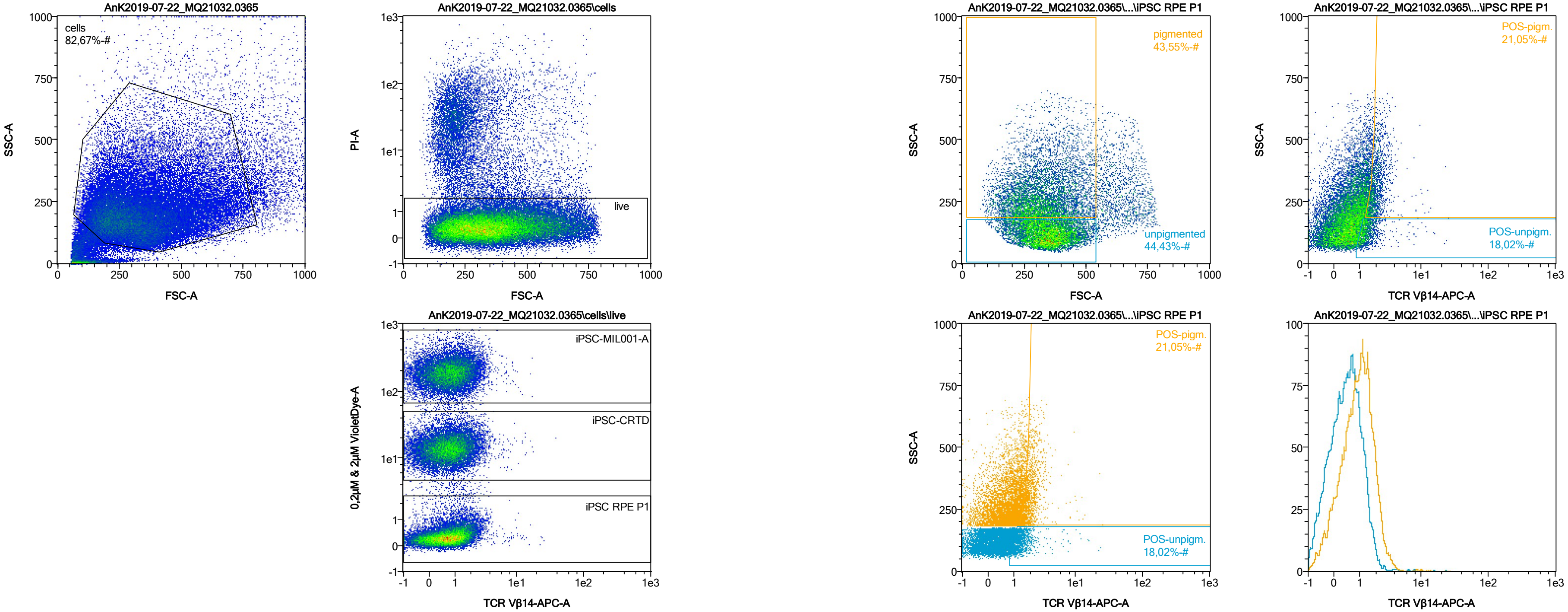

Name	%-#	TCR Vβ14-APC-A Mean	TCR Vβ14-APC-A Median
POS-unpigm.	18,02	1,54	1,29
POS-pigm.	21,05	2,15	1,92

Date:2024-06-14

Operator: Sebastian Knoebel

Version:2.13.0+54.g95abf0f4

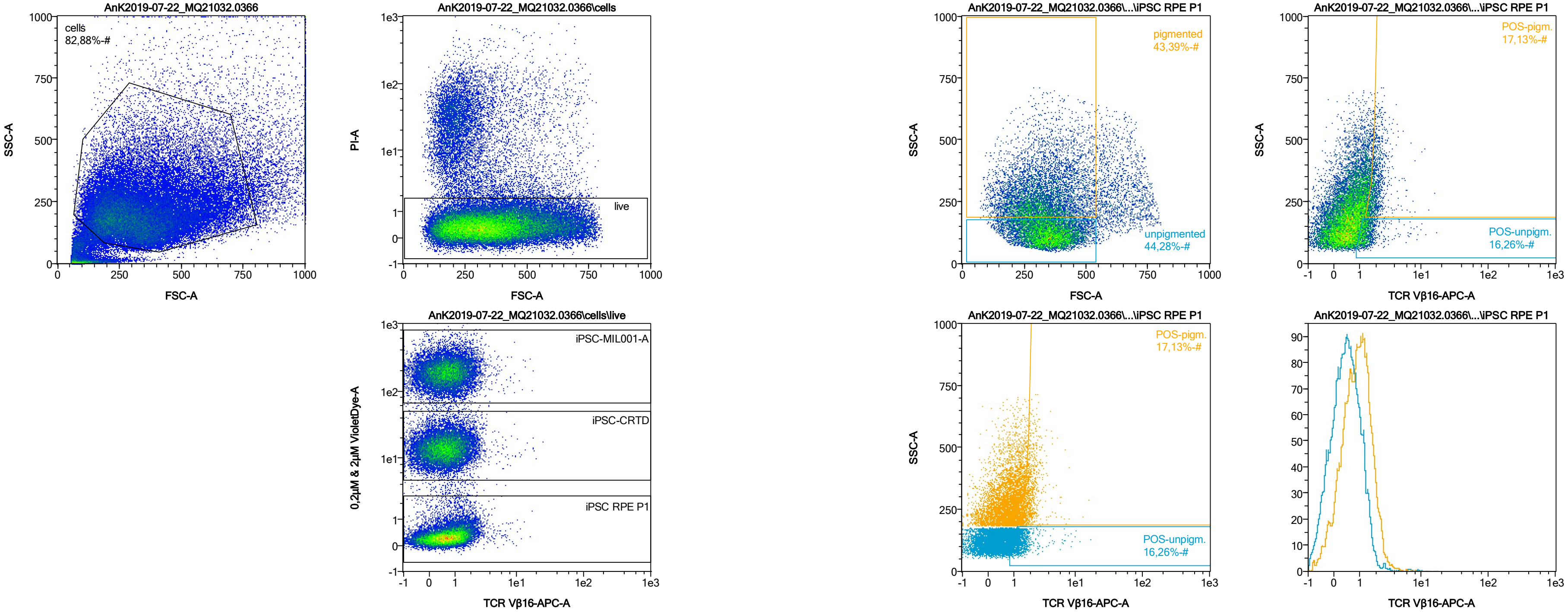

Name	%-#	TCR Vβ16-APC-A Mean	TCR Vβ16-APC-A Median
POS-unpigm.	16,26	1,50	1,27
POS-pigm.	17,13	2,06	1,89

Date:2024-06-14

Operator: Sebastian Knoebel

Version:2.13.0+54.g95abf0f4

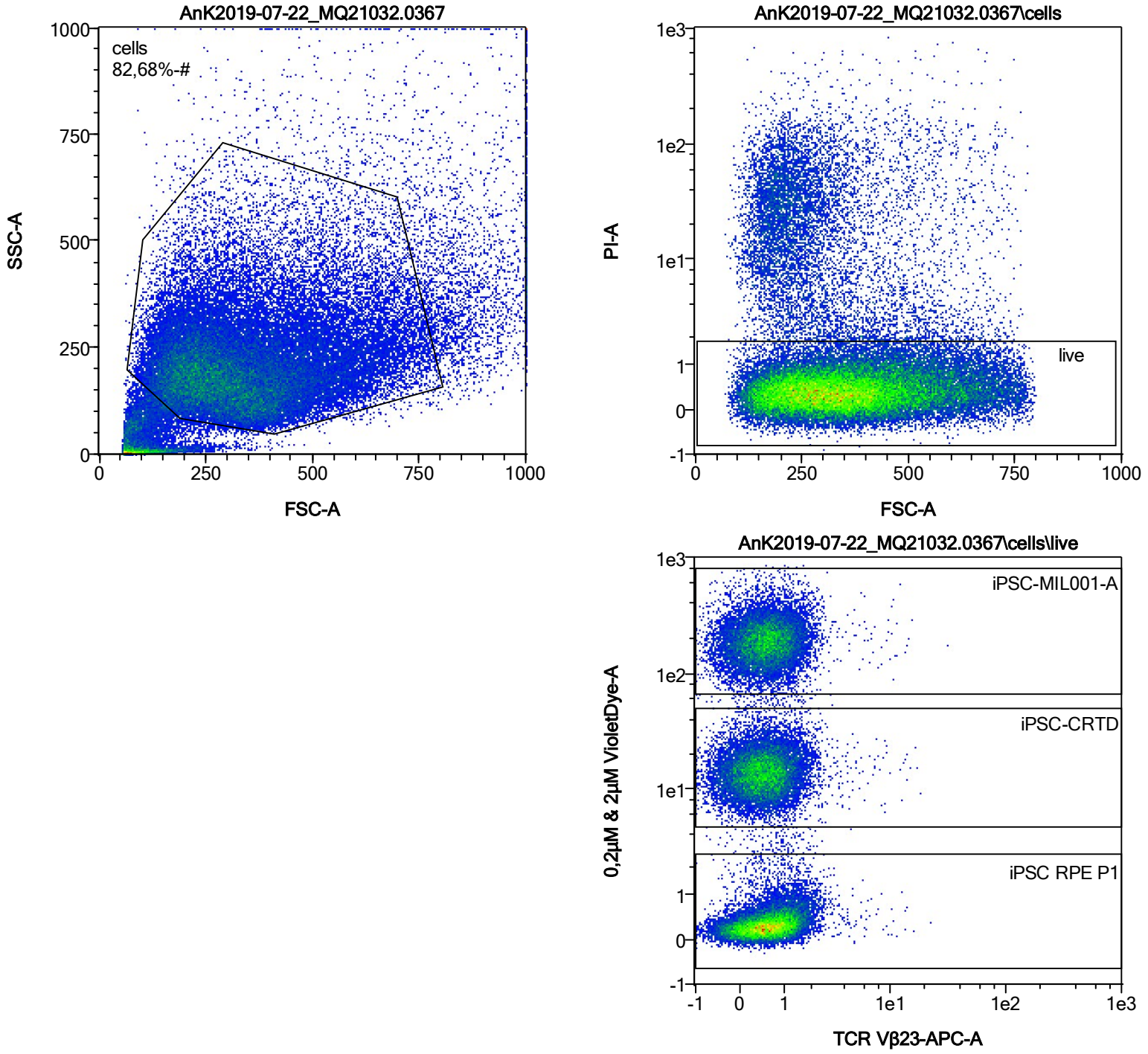

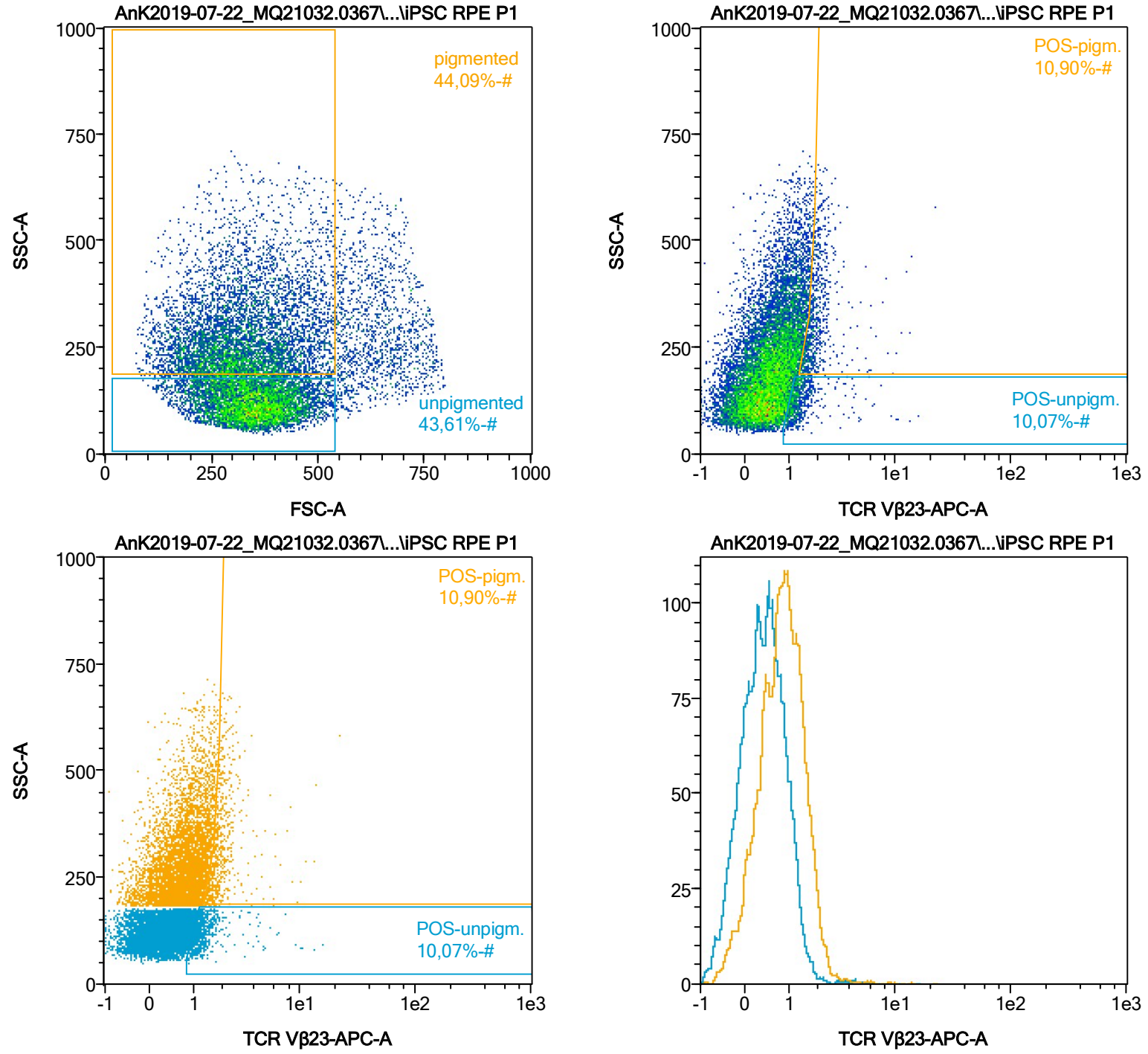

Name	%-#	TCR Vβ23-APC-A Mean	TCR Vβ23-APC-A Median
POS-unpigm.	10,07	1,47	1,22
POS-pigm.	10,90	2,12	1,86

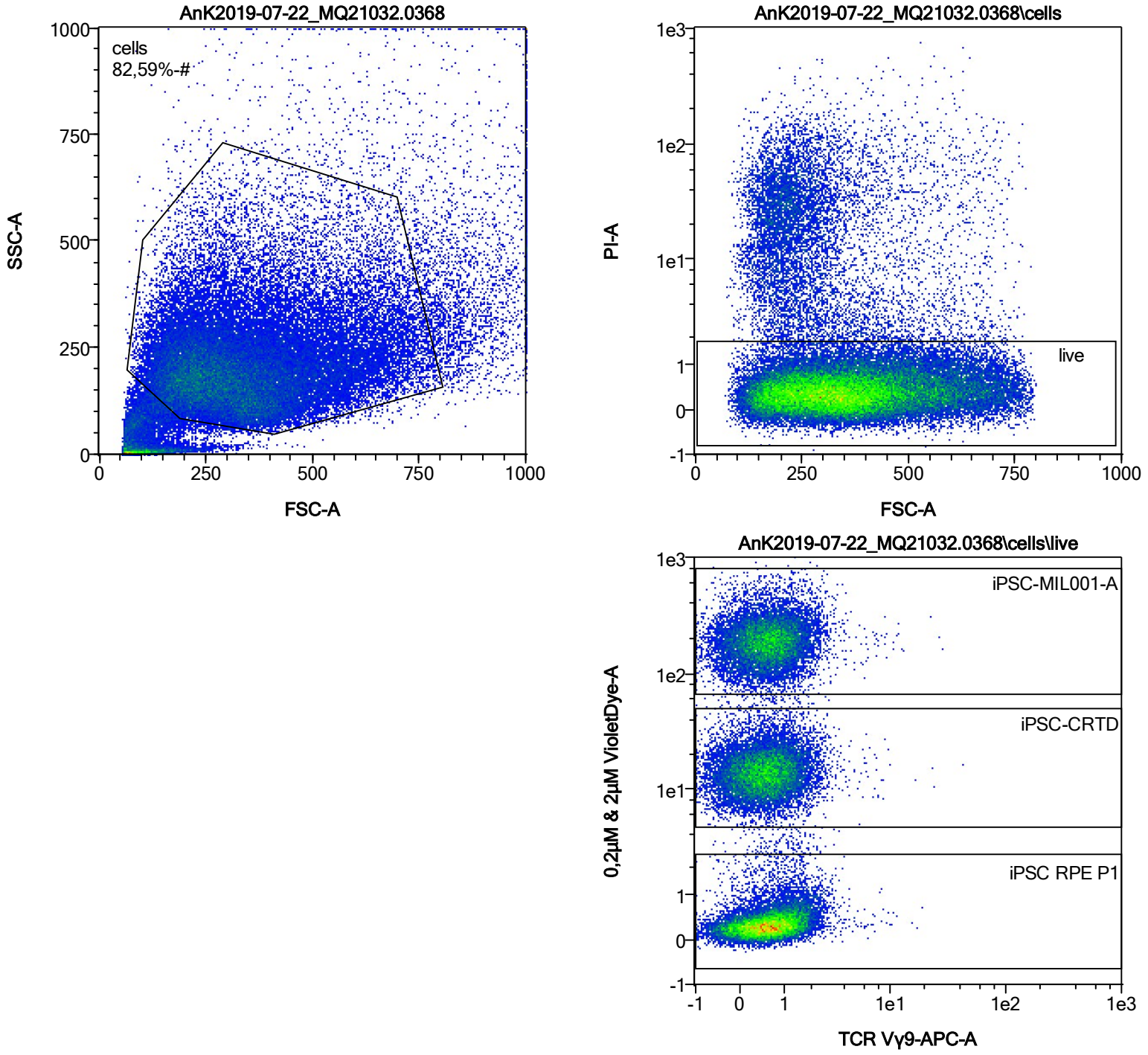

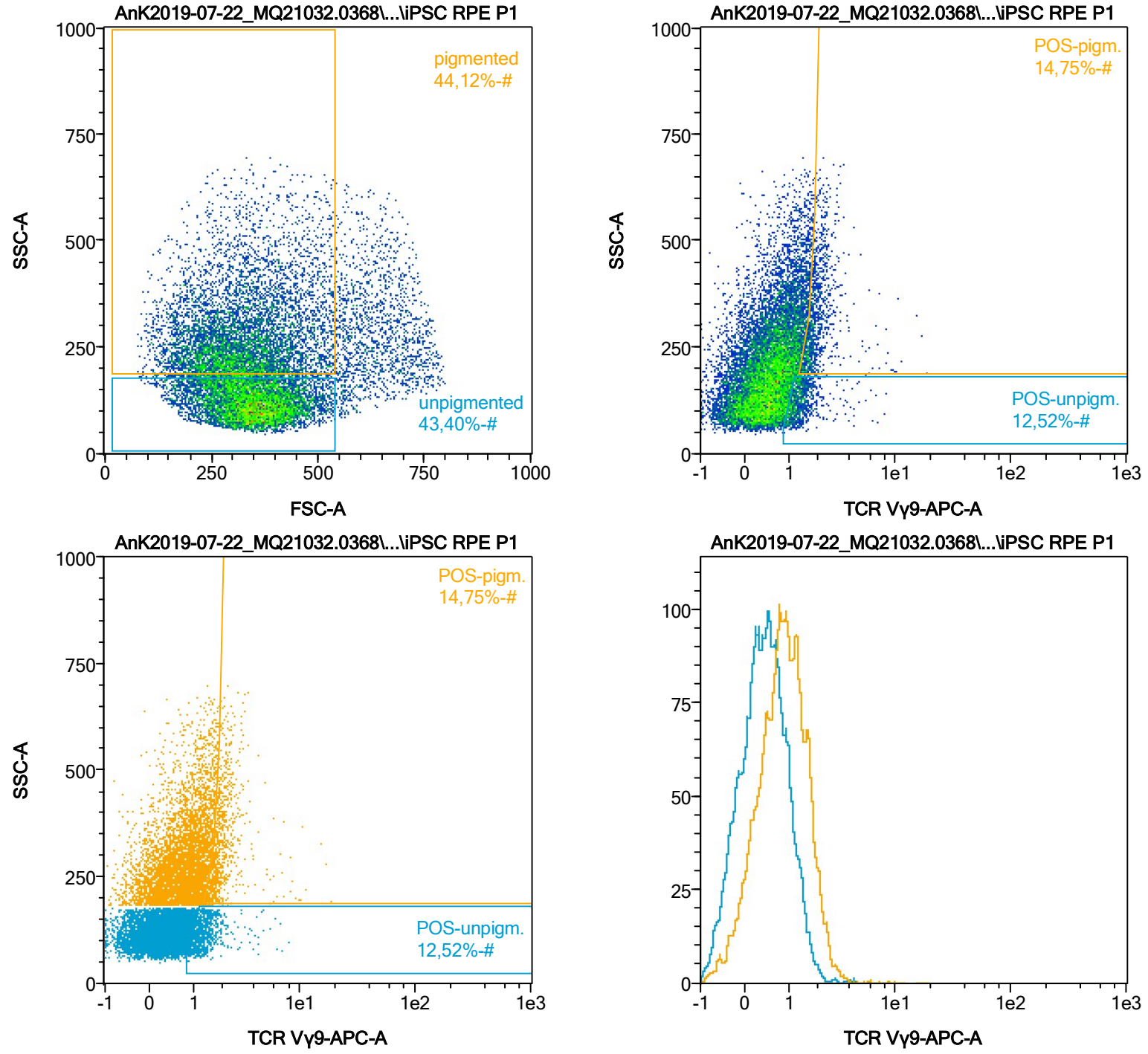

Name	%-#	TCR Vγ9-APC-A Mean	TCR Vγ9-APC-A Median
POS-unpigm.	12,52	1,42	1,27
POS-pigm.	14,75	2,13	1,86

Date:2024-06-14

Operator: Sebastian Knoebel

Version:2.13.0+54.g95abf0f4

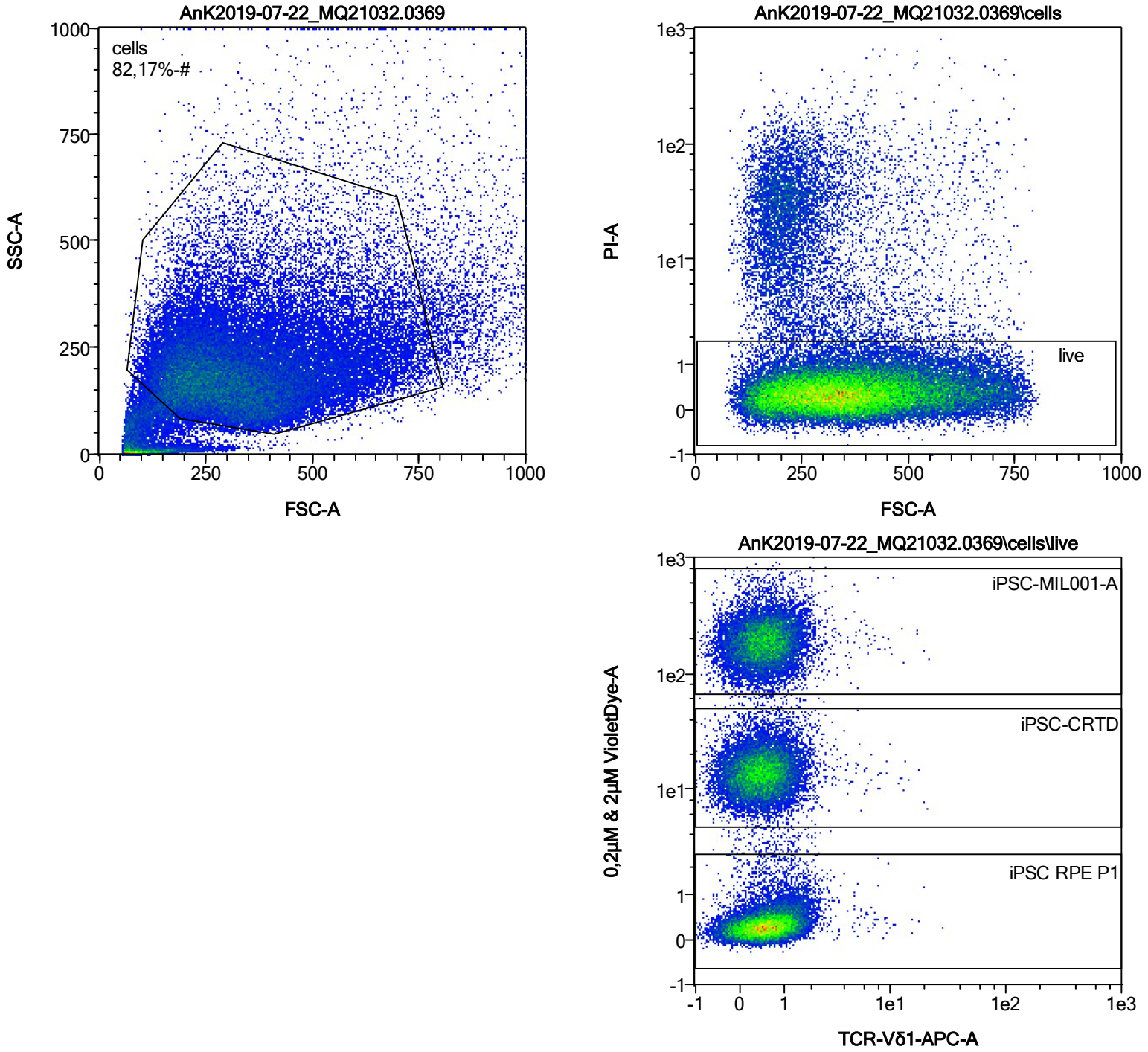

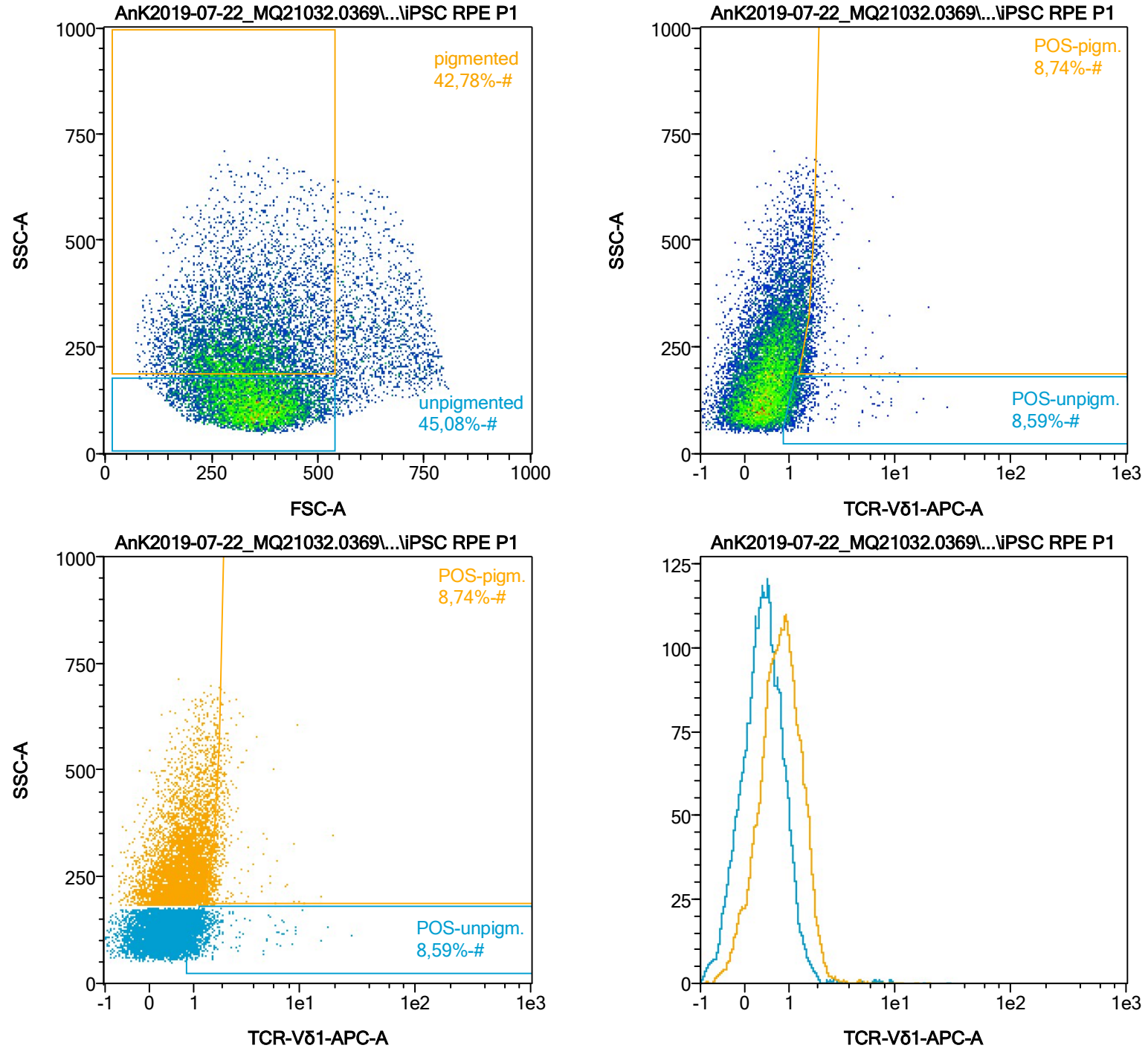

Name	%-#	TCR-Vδ1-APC-A Mean	TCR-Vδ1-APC-A Median
POS-unpigm.	8,59	1,60	1,20
POS-pigm.	8,74	2,06	1,77

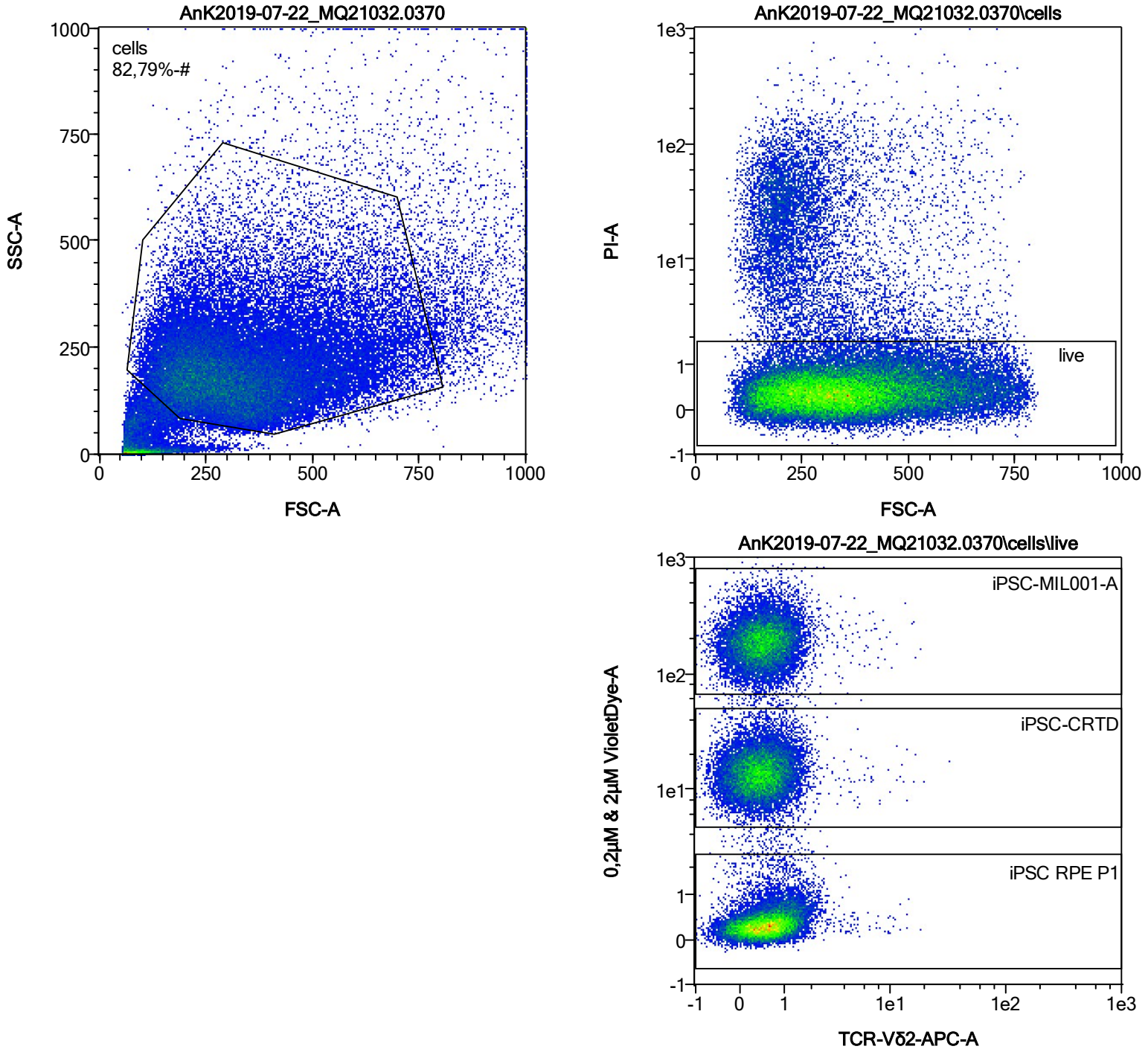

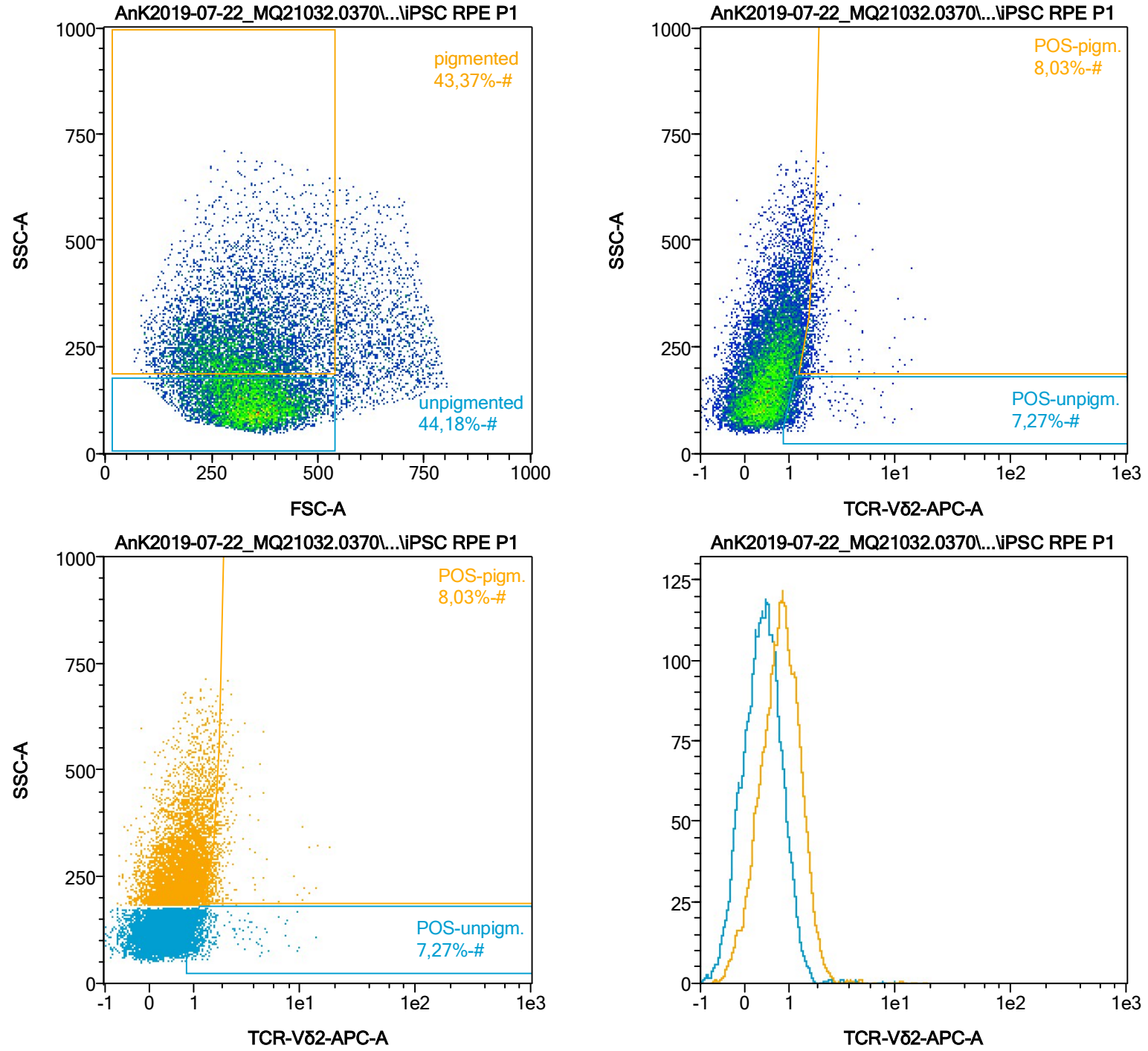

Name	%-#	TCR-Vδ2-APC-A Mean	TCR-Vδ2-APC-A Median
POS-unpigm.	7,27	1,52	1,20
POS-pigm.	8,03	2,10	1,78

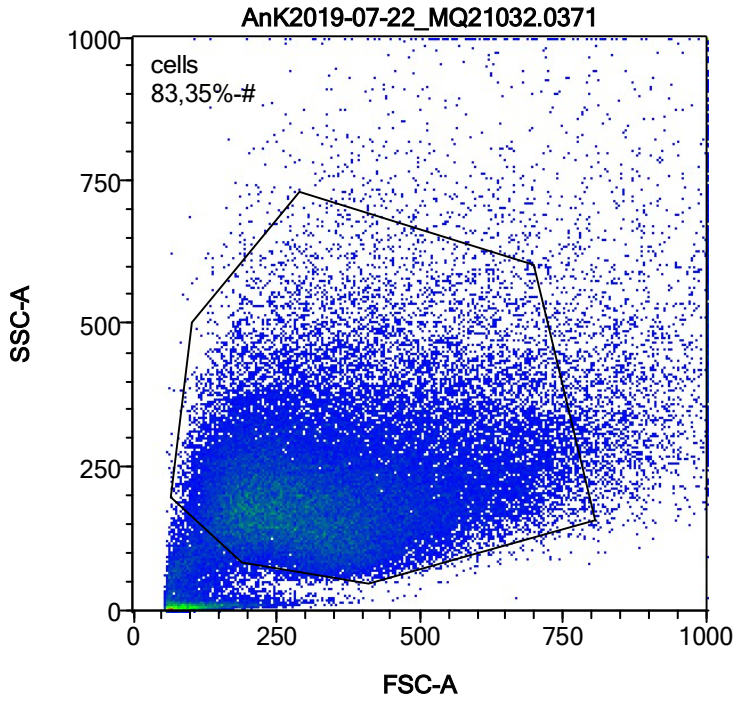

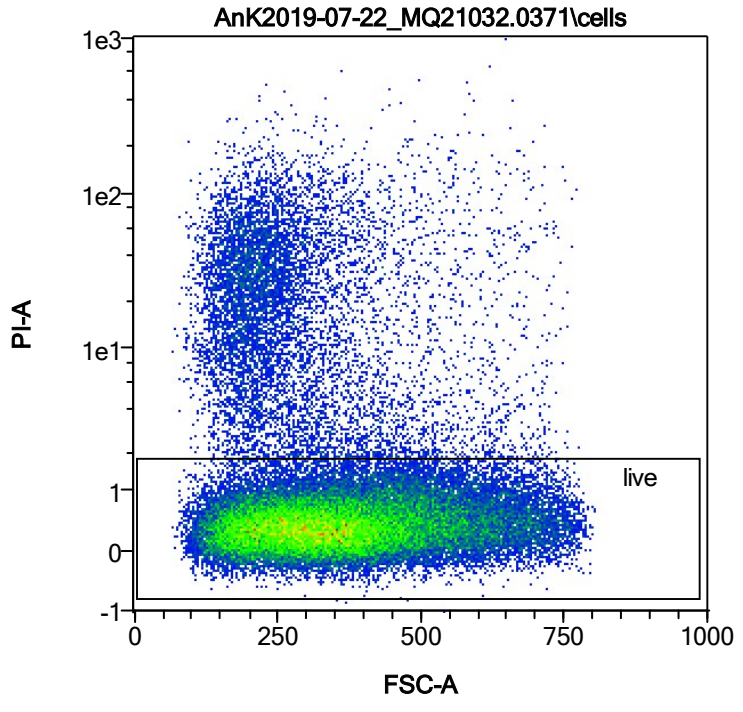

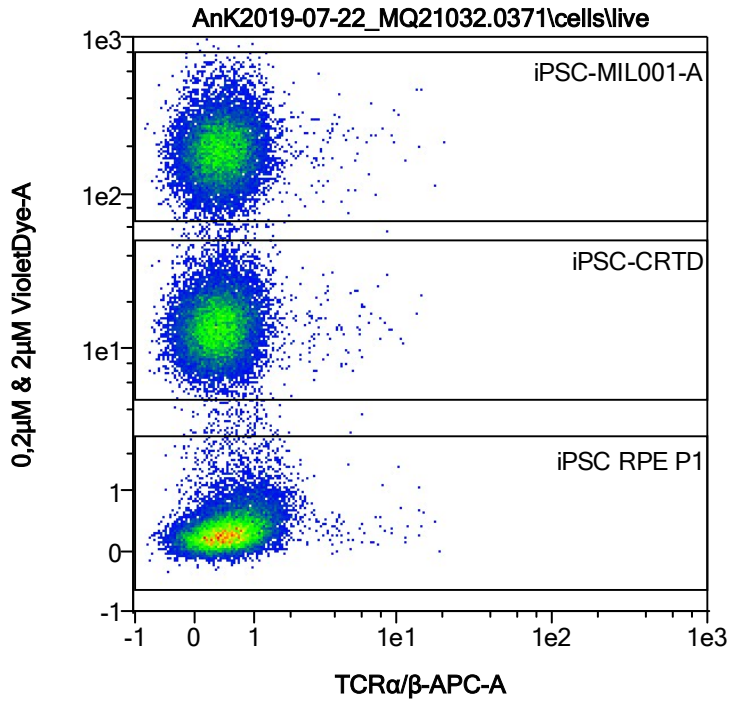

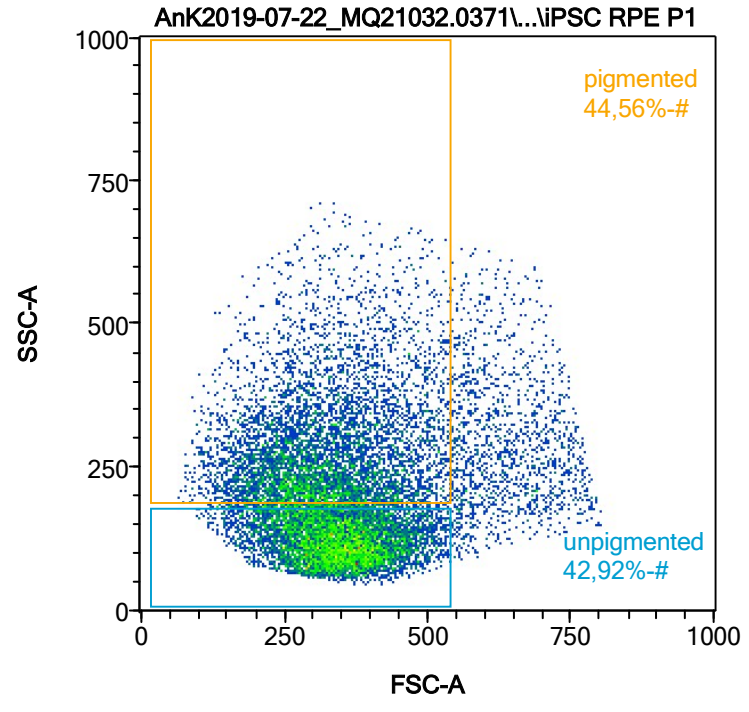

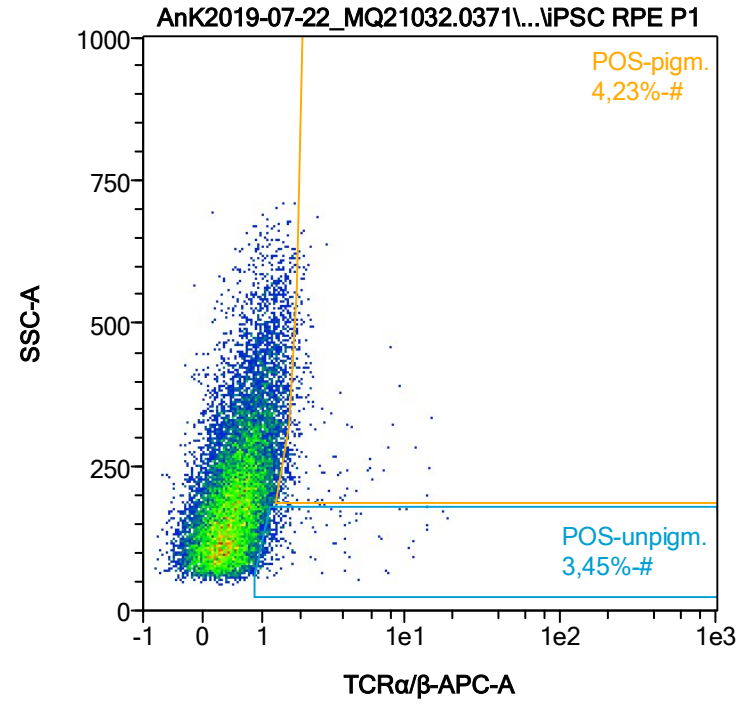

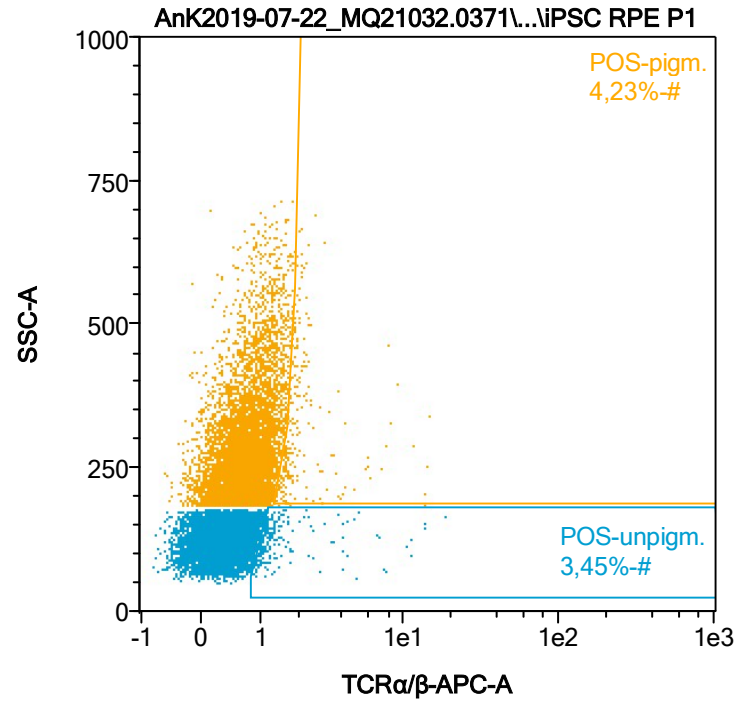

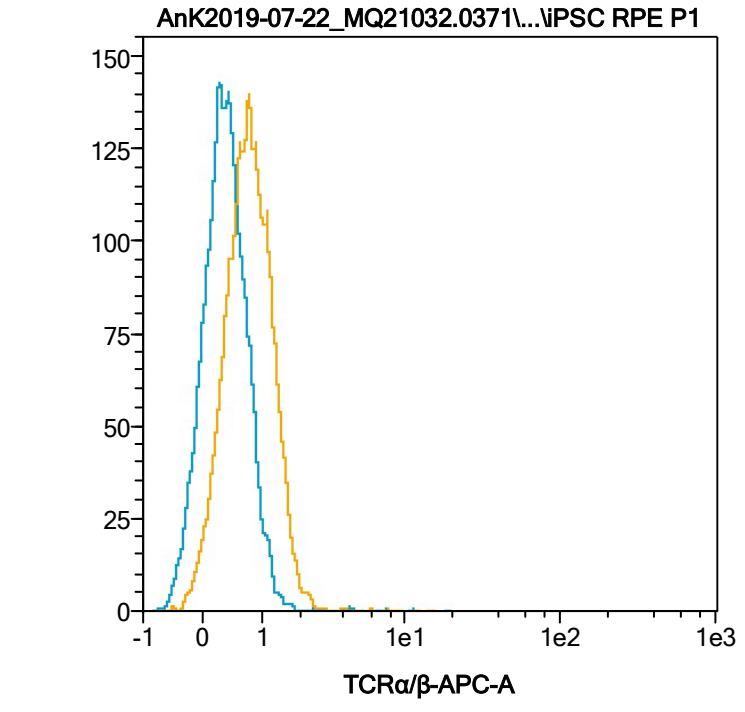

Name	%-#	TCRα/β-APC-A Mean	TCRα/β-APC-A Median
POS-unpigm.	3,45	1,88	1,18
POS-pigm.	4,23	2,29	1,80

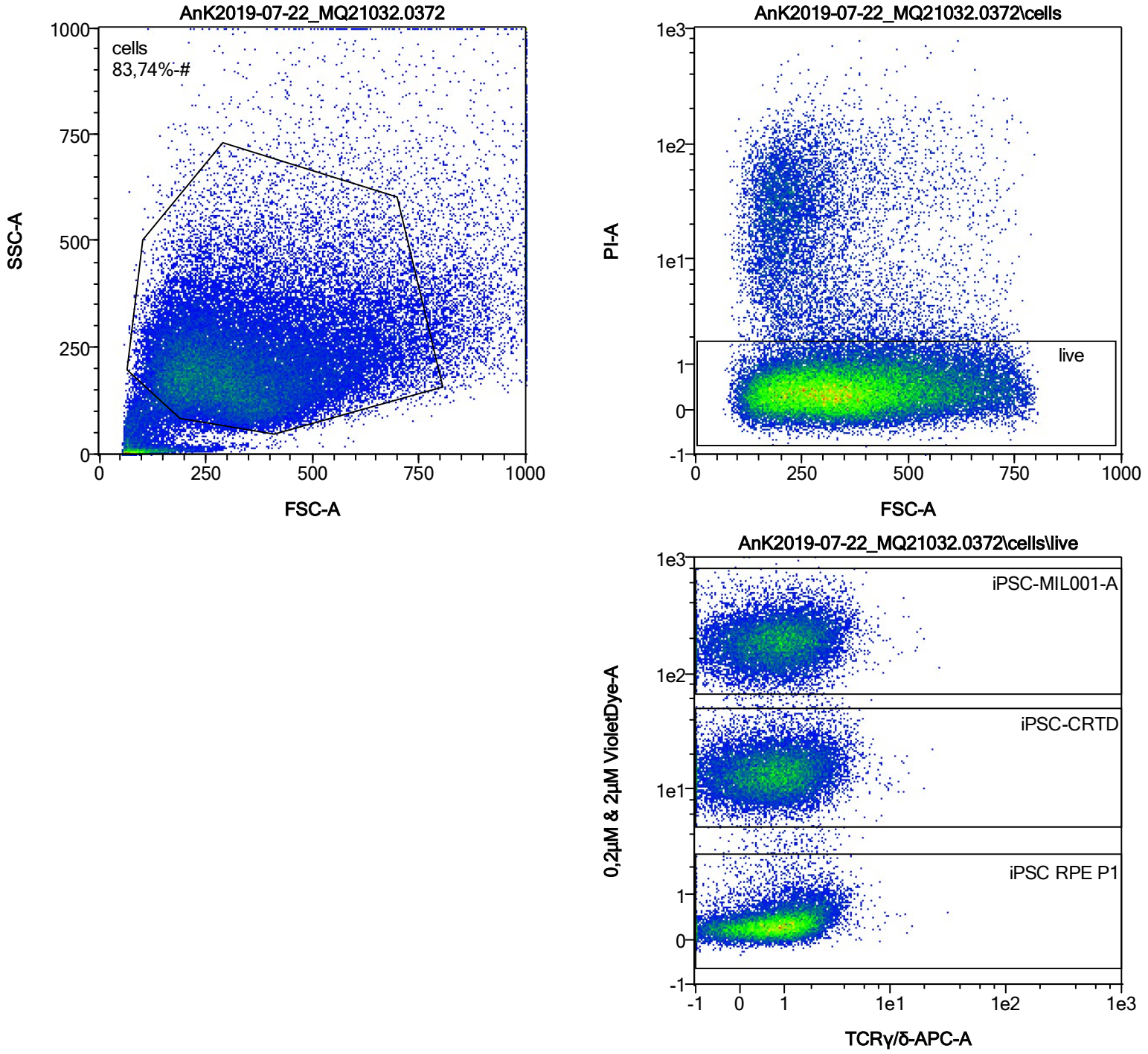

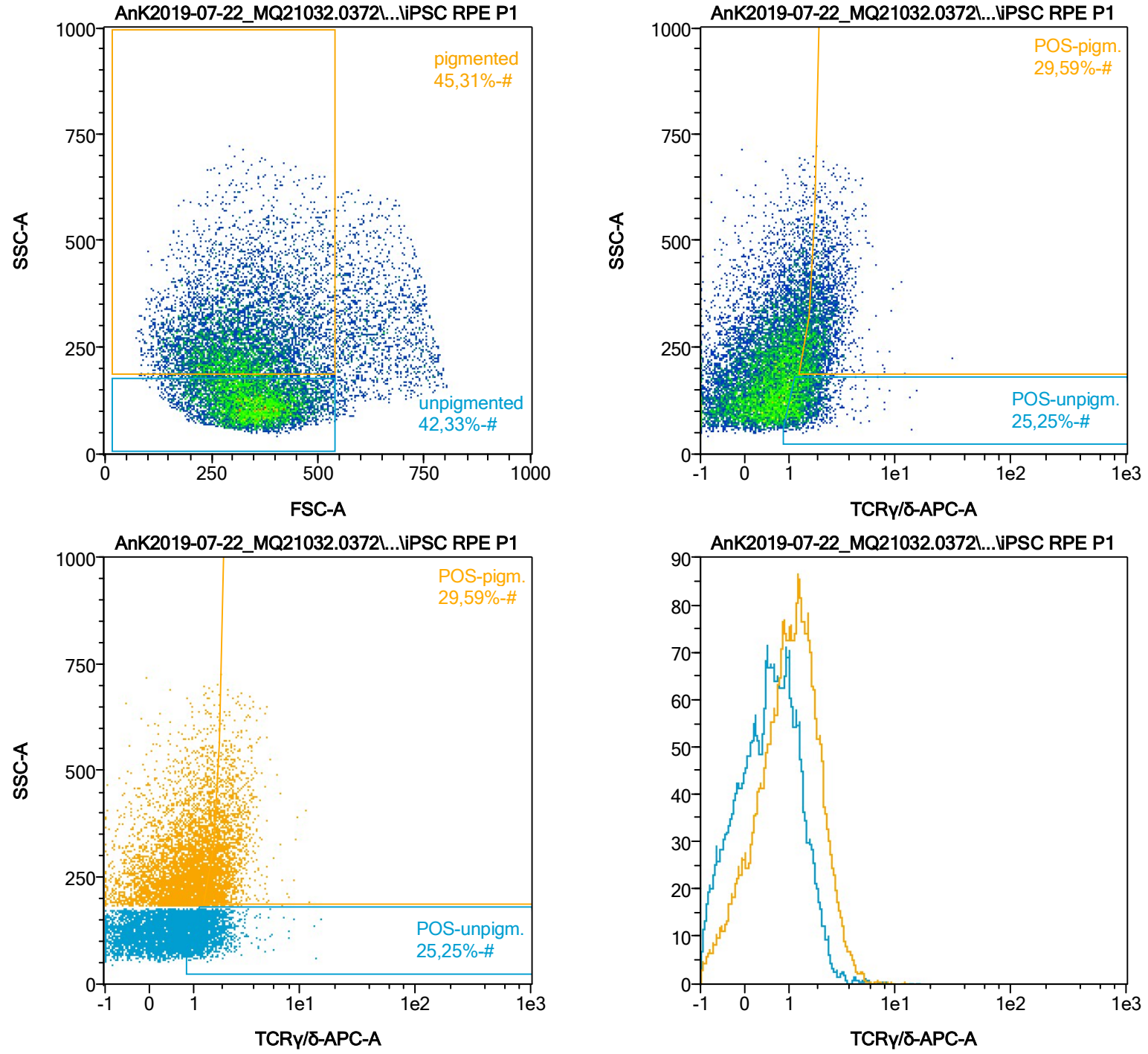

Name	%-#	TCRγ/δ-APC-A Mean	TCRγ/δ-APC-A Median
POS-unpigm.	25,25	1,59	1,39
POS-pigm.	29,59	2,25	2,04

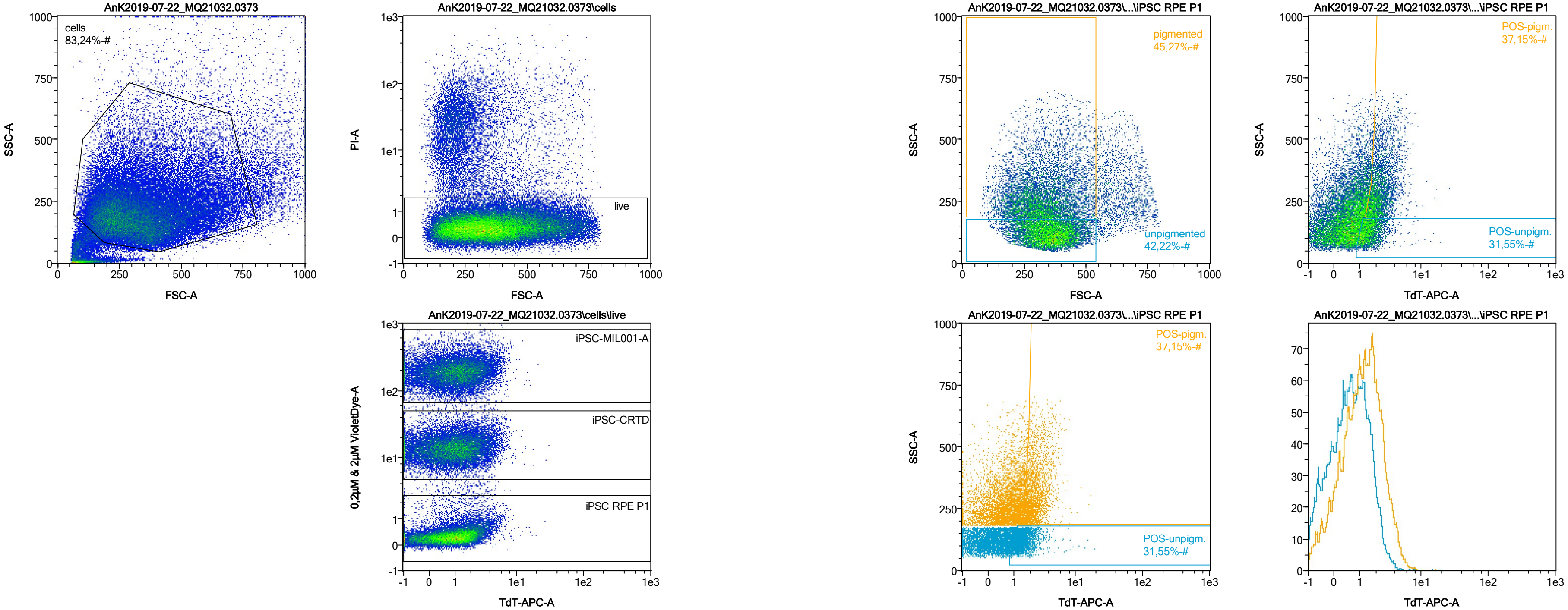

Name	%-#	TdT-APC-A Mean	TdT-APC-A Median
POS-unpigm.	31,55	1,68	1,49
POS-pigm.	37,15	2,49	2,20

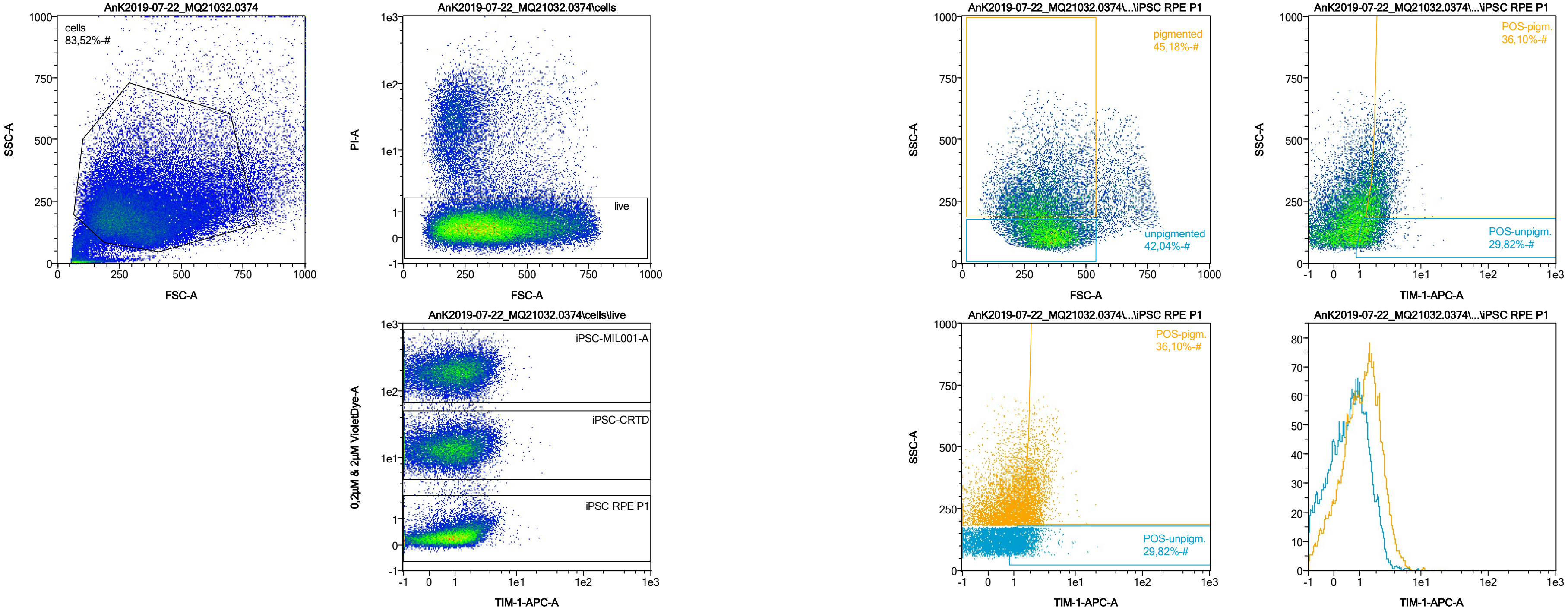

Name	%-#	TIM-1-APC-A Mean	TIM-1-APC-A Median
POS-unpigm.	29,82	1,68	1,47
POS-pigm.	36,10	2,40	2,16

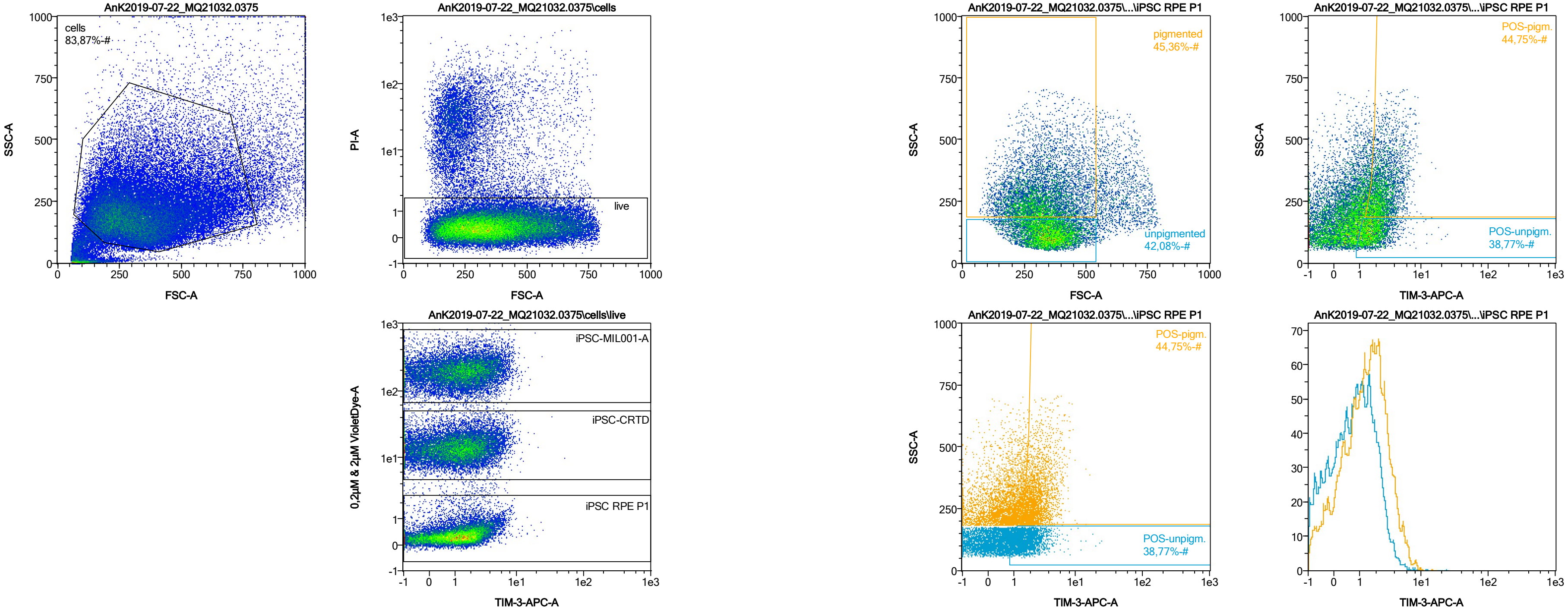

Name	%-#	TIM-3-APC-A Mean	TIM-3-APC-A Median
POS-unpigm.	38,77	1,93	1,67
POS-pigm.	44,75	2,76	2,39

Date:2024-06-14
Operator: Sebastian Knoebel
Version:2.13.0+54.g95abf0f4

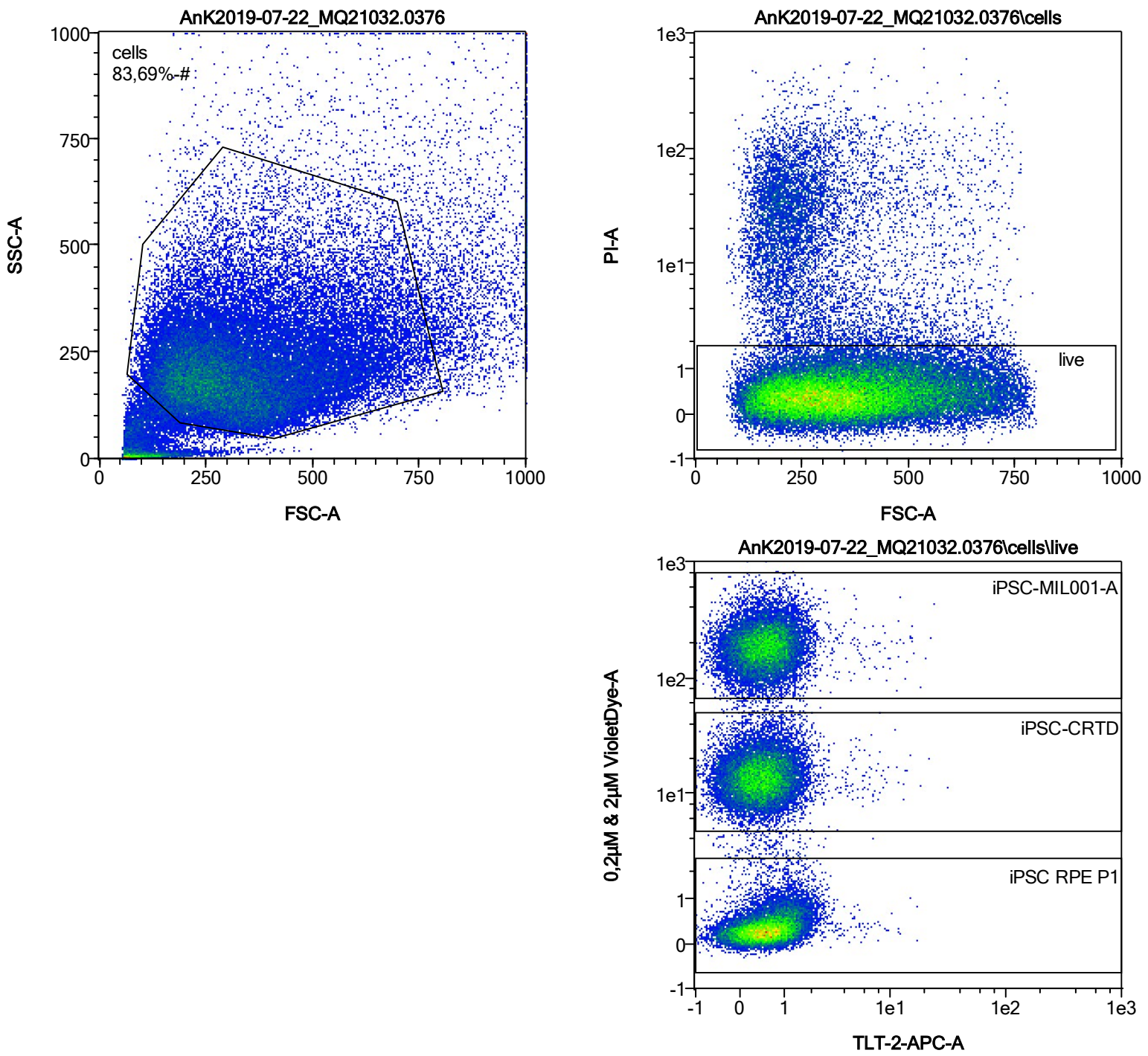

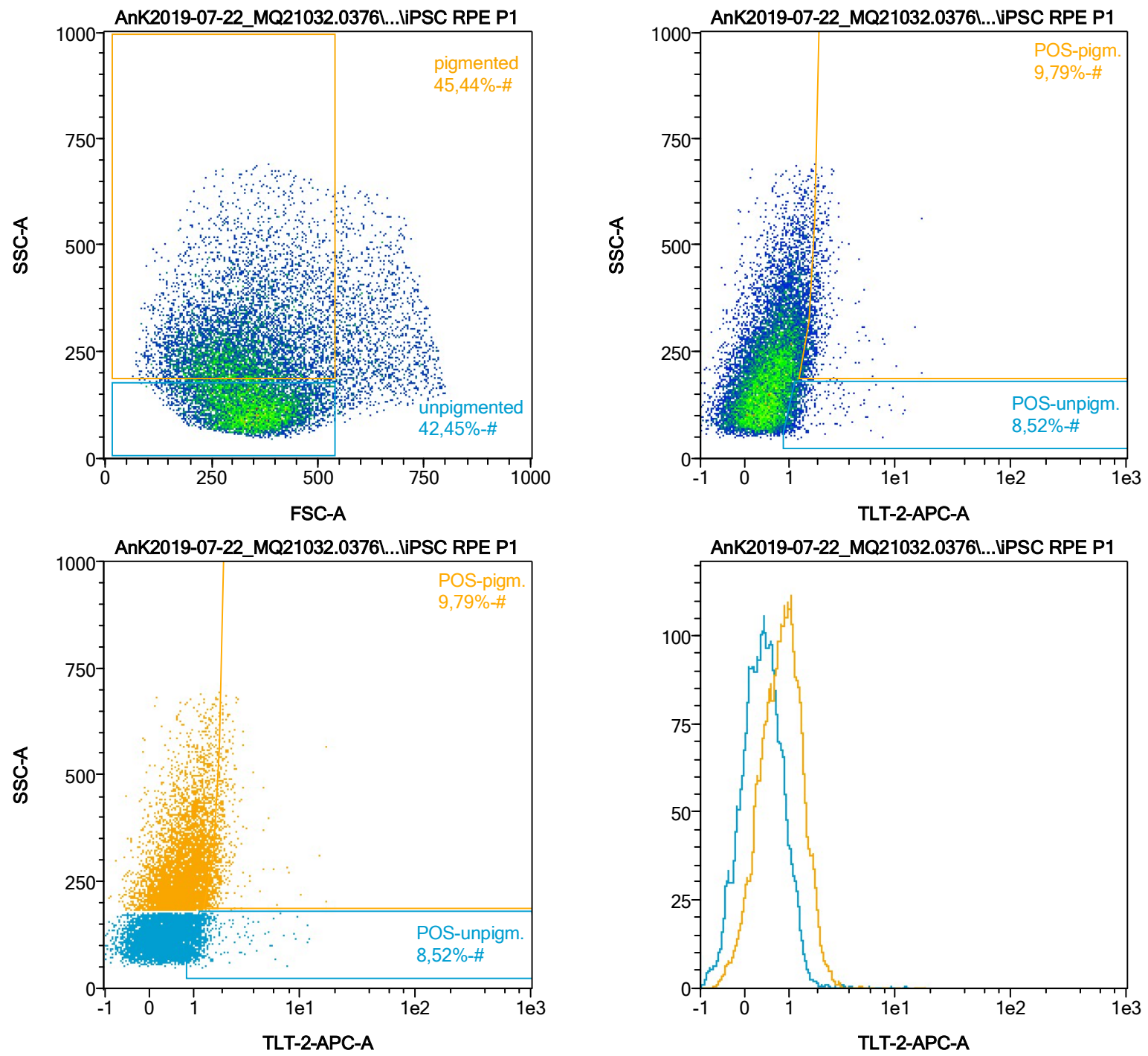

Name	%-#	TLT-2-APC-A Mean	TLT-2-APC-A Median
POS-unpigm.	8,52	1,53	1,21
POS-pigm.	9,79	2,04	1,84

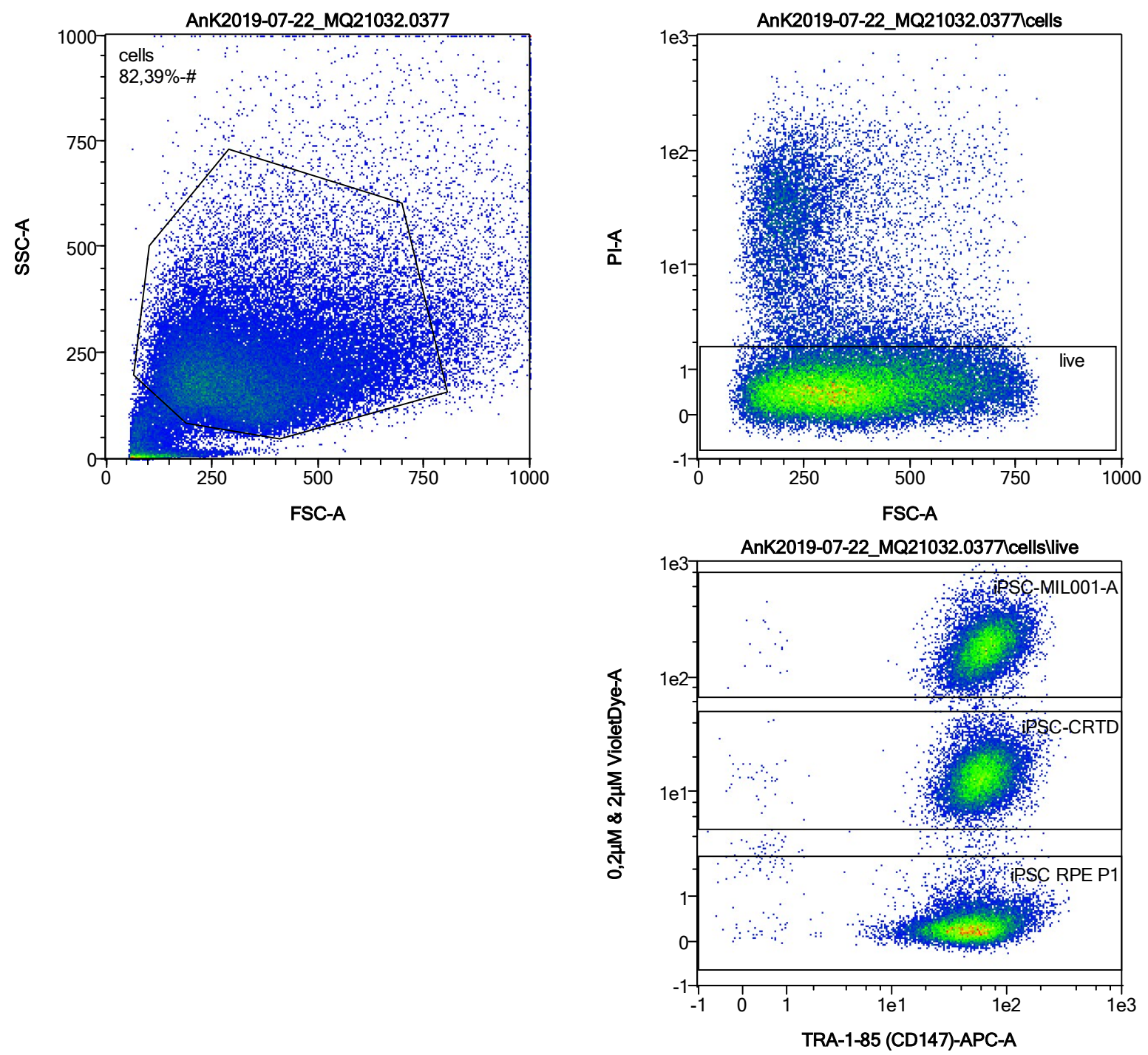

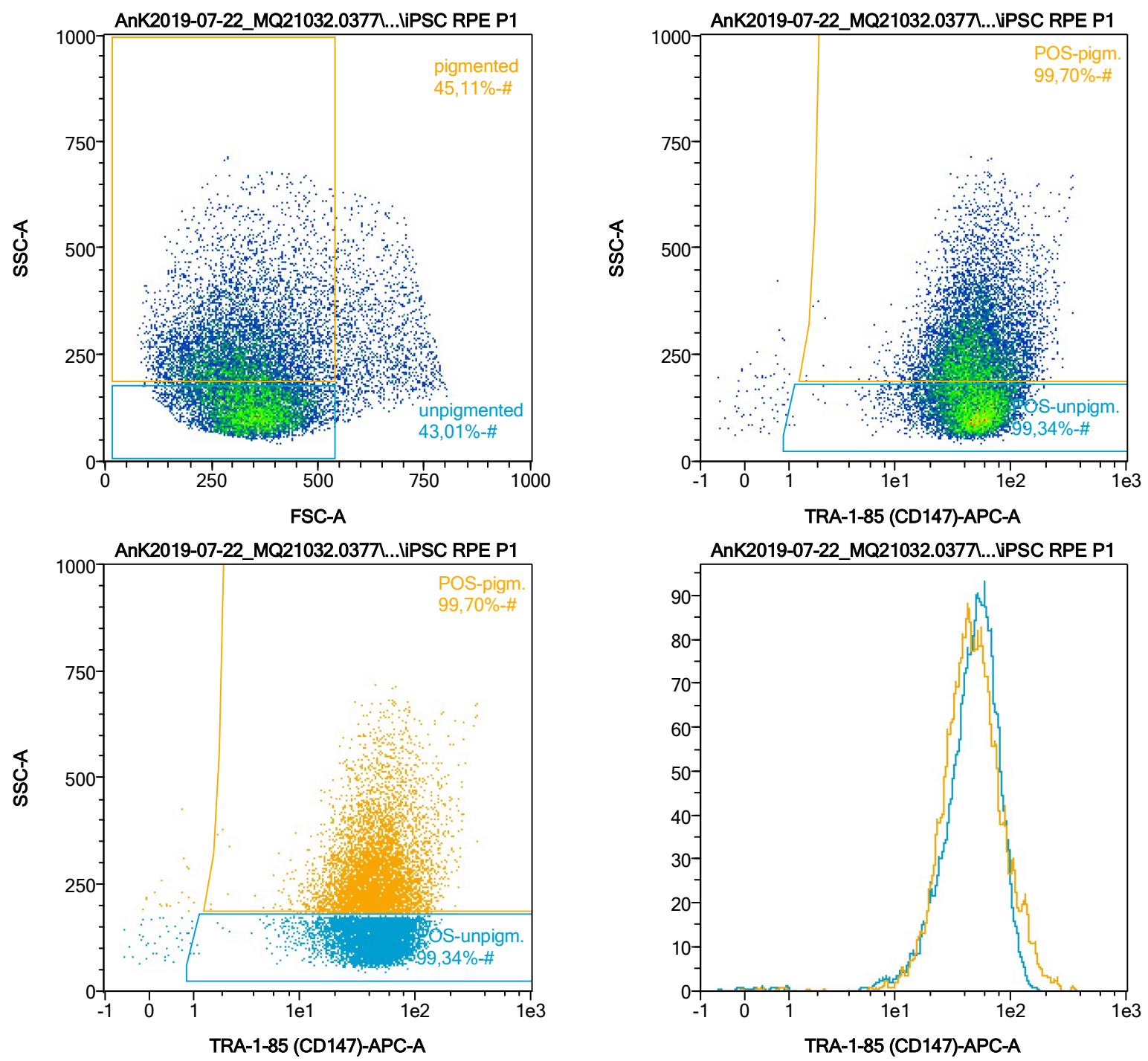

Name	%-#	TRA-1-85 (CD147)-APC-A Mean	TRA-1-85 (CD147)-APC-A Median
POS-unpigm.	99,34	50,97	48,44
POS-pigm.	99,70	54,33	45,37

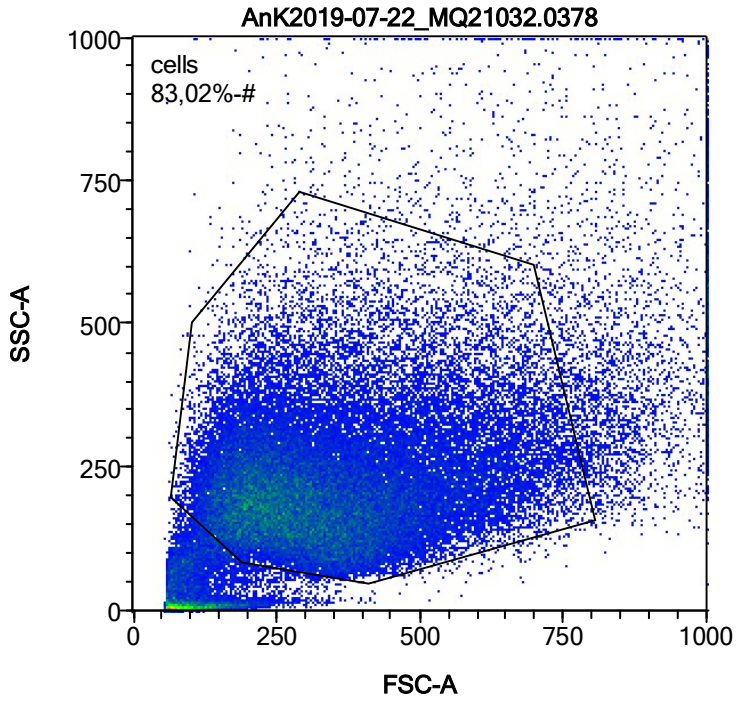

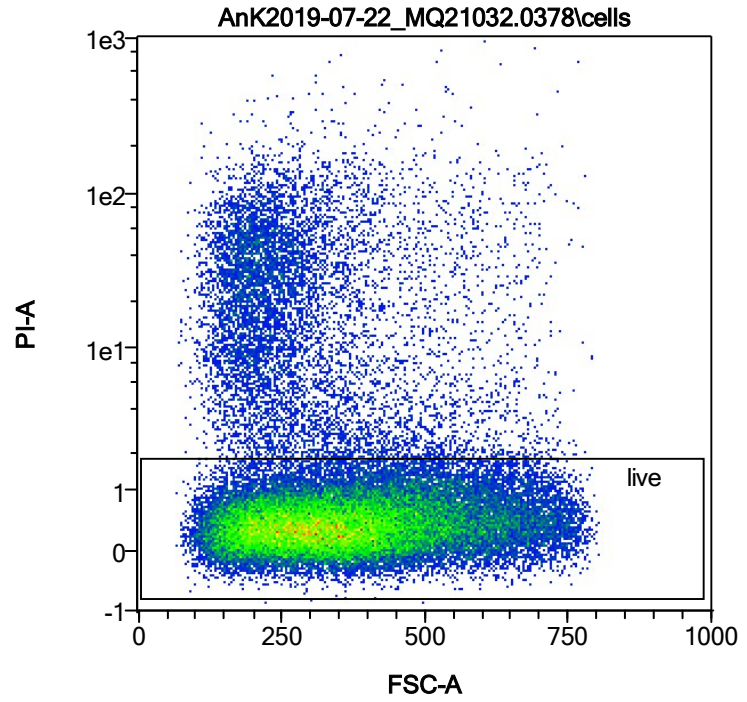

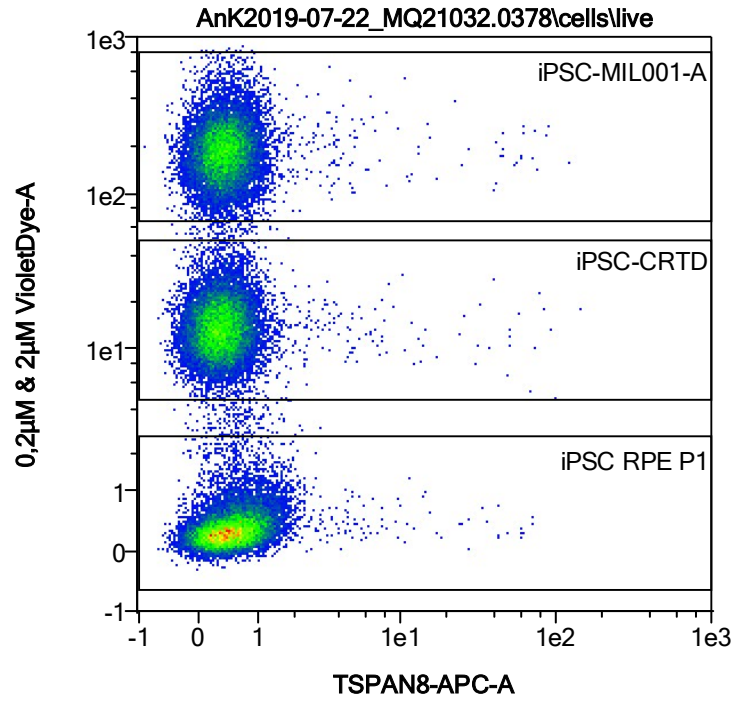

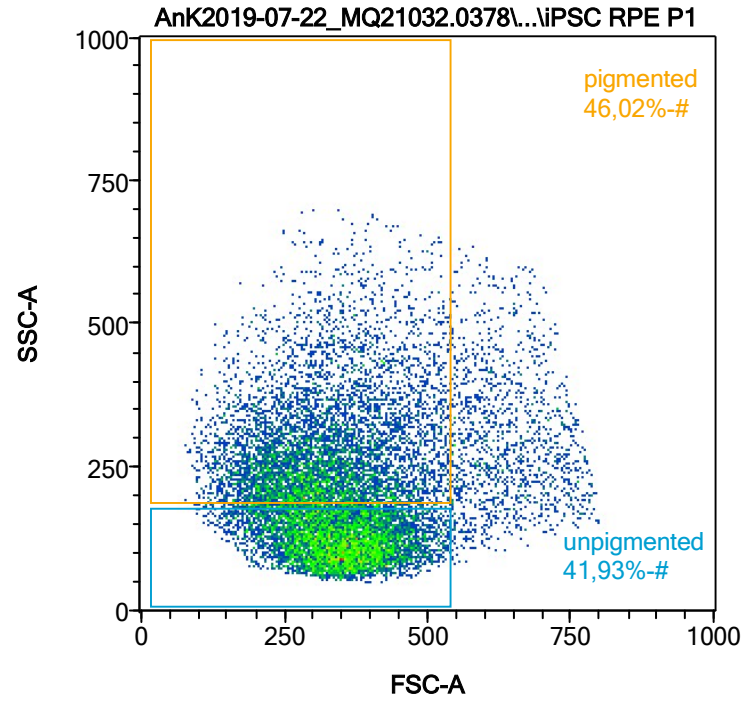

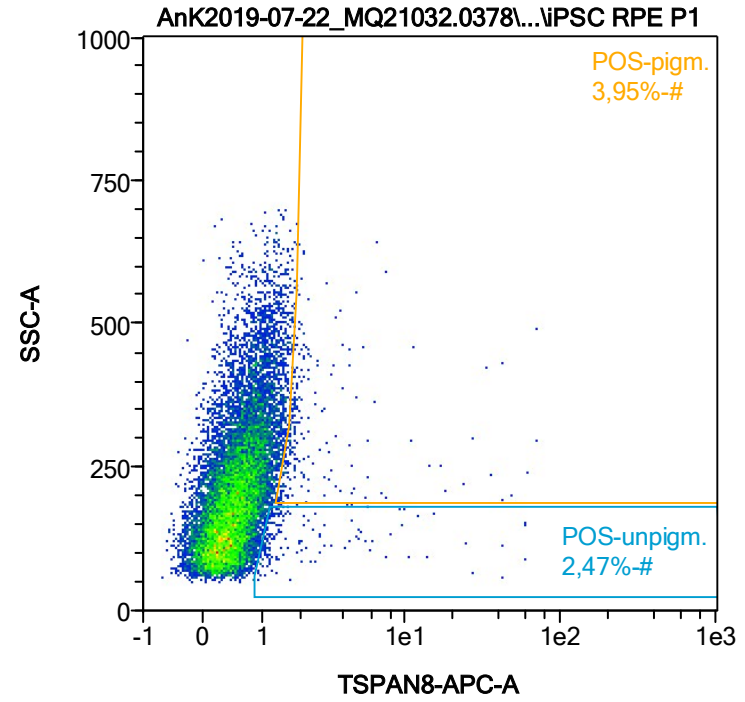

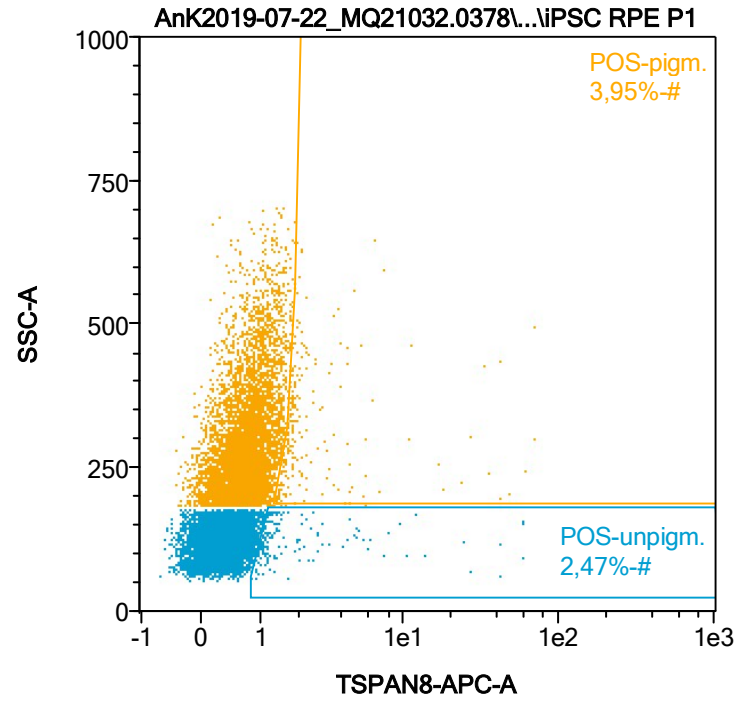

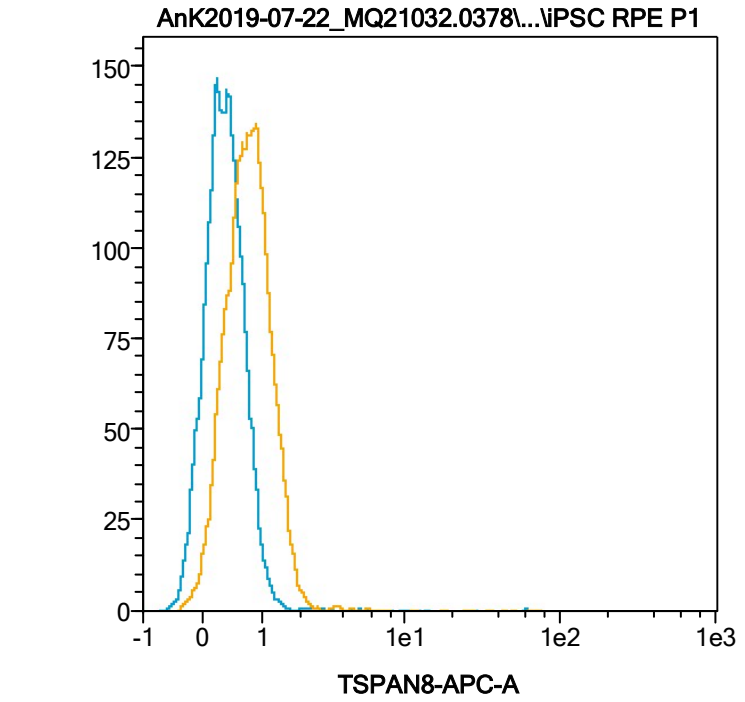

Name	%-#	TSPAN8-APC-A Mean	TSPAN8-APC-A Median
POS-unpigm.	2,47	4,11	1,25
POS-pigm.	3,95	4,02	1,83

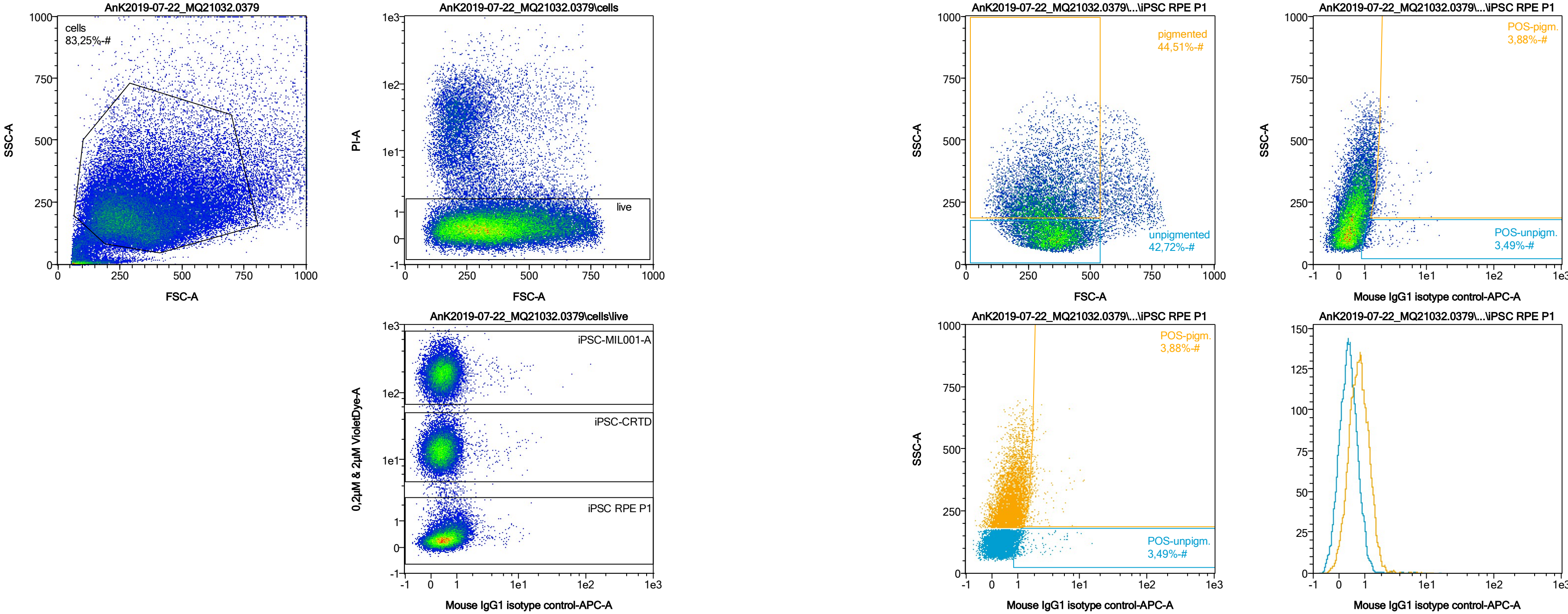

Name	%-#	Mouse IgG1 isotype control-APC-A Mean	Mouse IgG1 isotype control-APC-A Median
POS-unpigm.	3,49	1,82	1,21
POS-pigm.	3,88	2,31	1,86

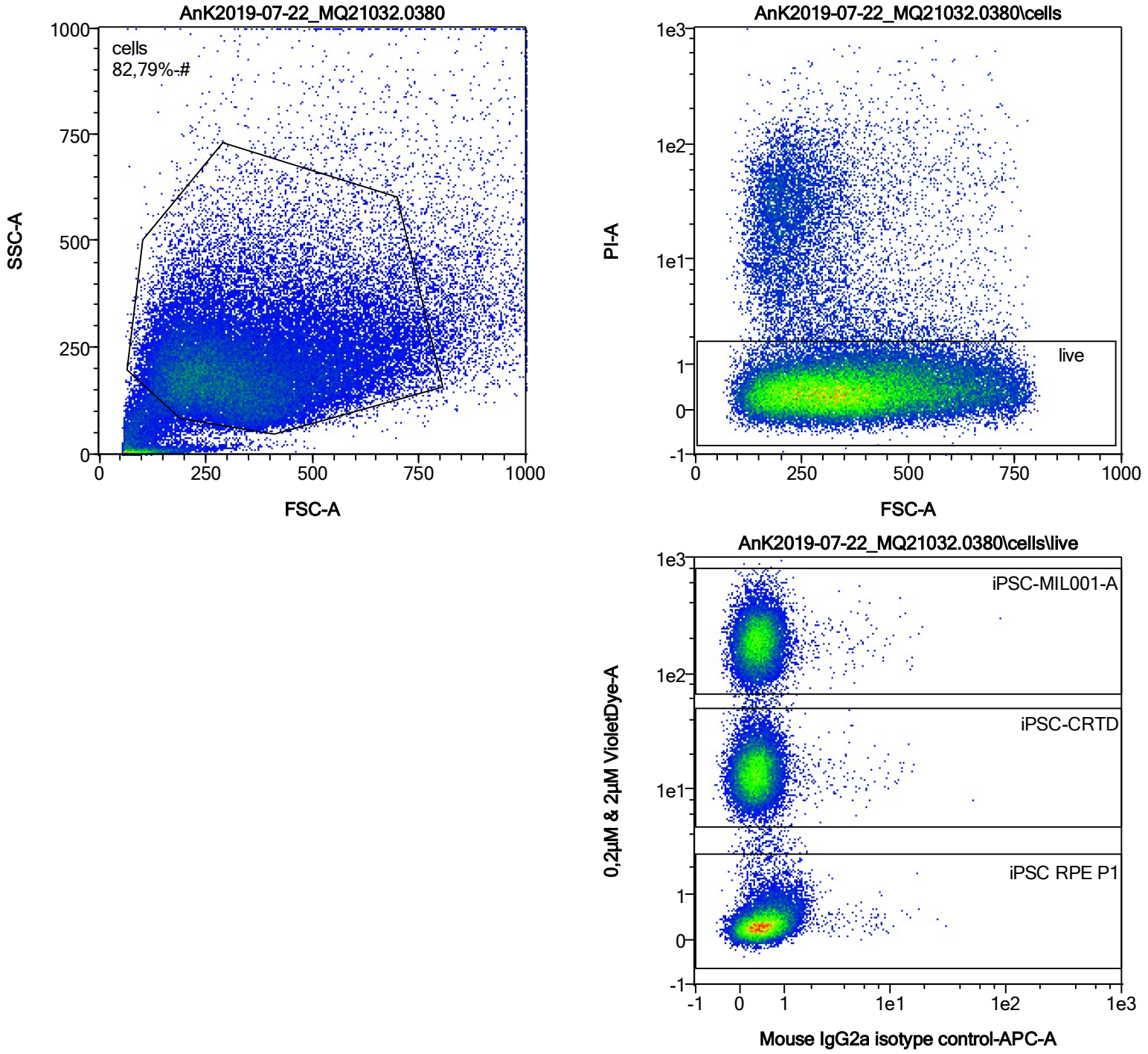

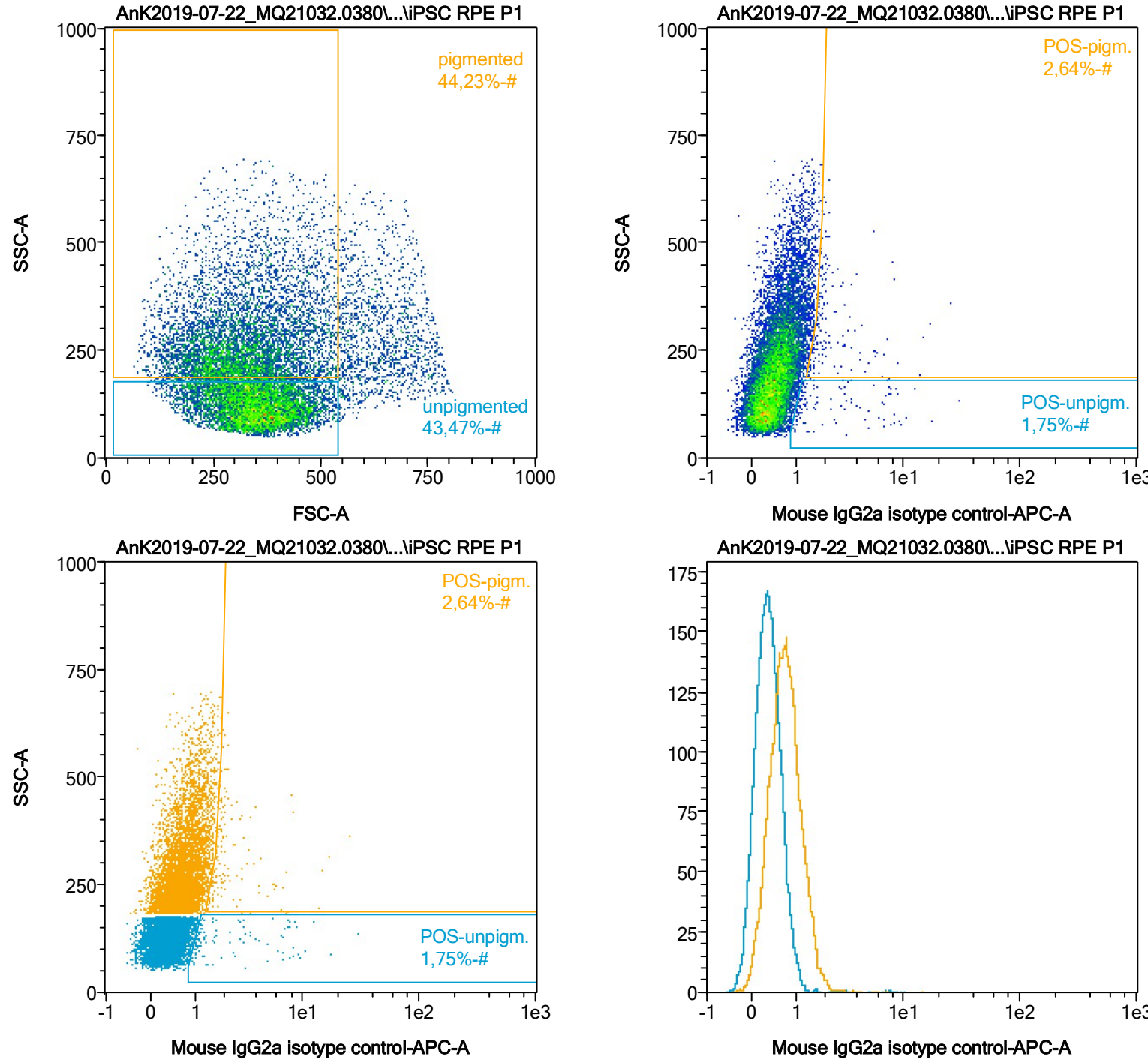

Name	%-#	Mouse IgG2a isotype control-APC-A Mean	Mouse IgG2a isotype control-APC-A Median
POS-unpigm.	1,75	3,22	1,31
POS-pigm.	2,64	2,71	1,89

Date:2024-06-14
Operator: Sebastian Knoebel
Version:2.13.0+54.g95abf0f4

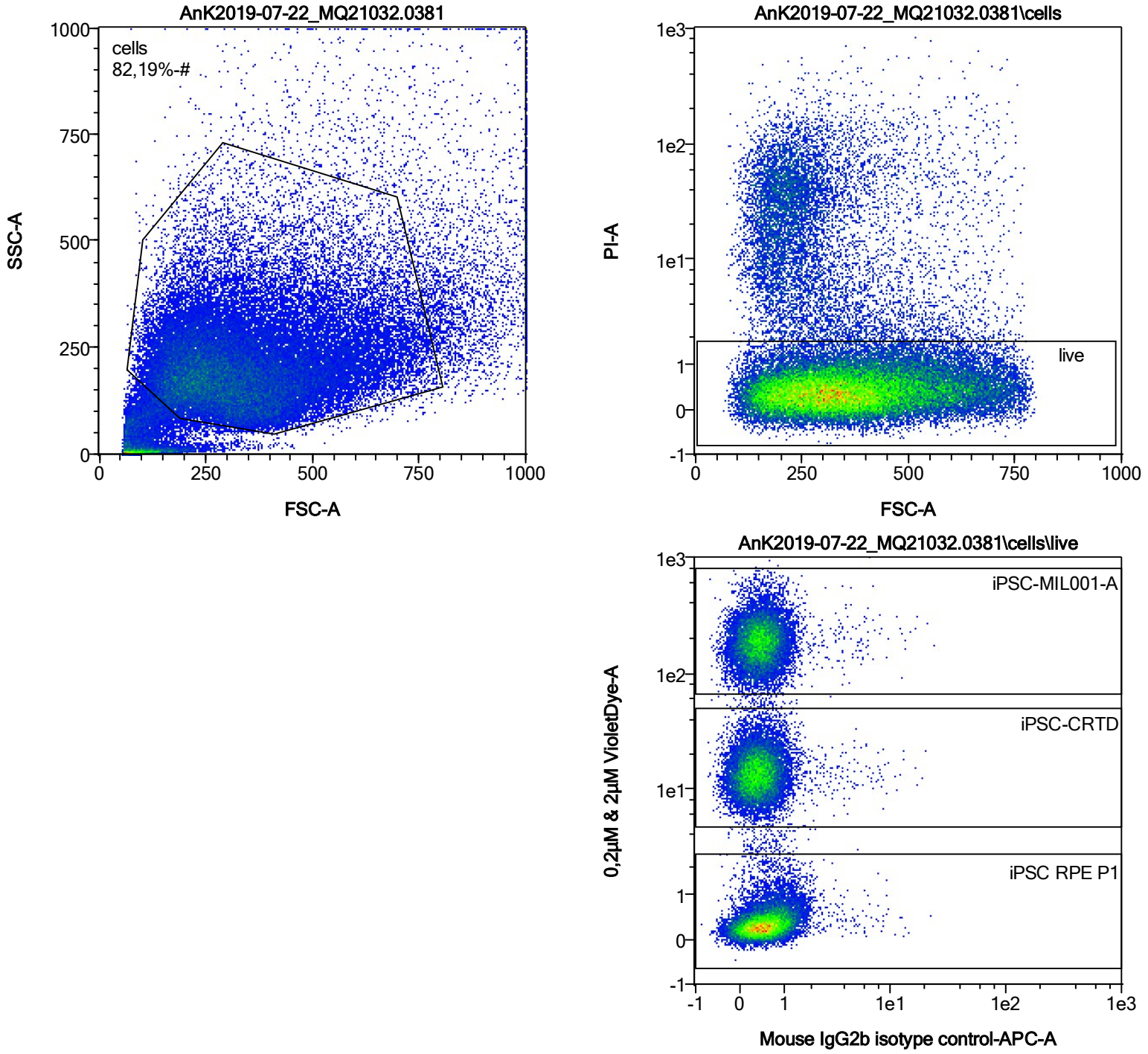

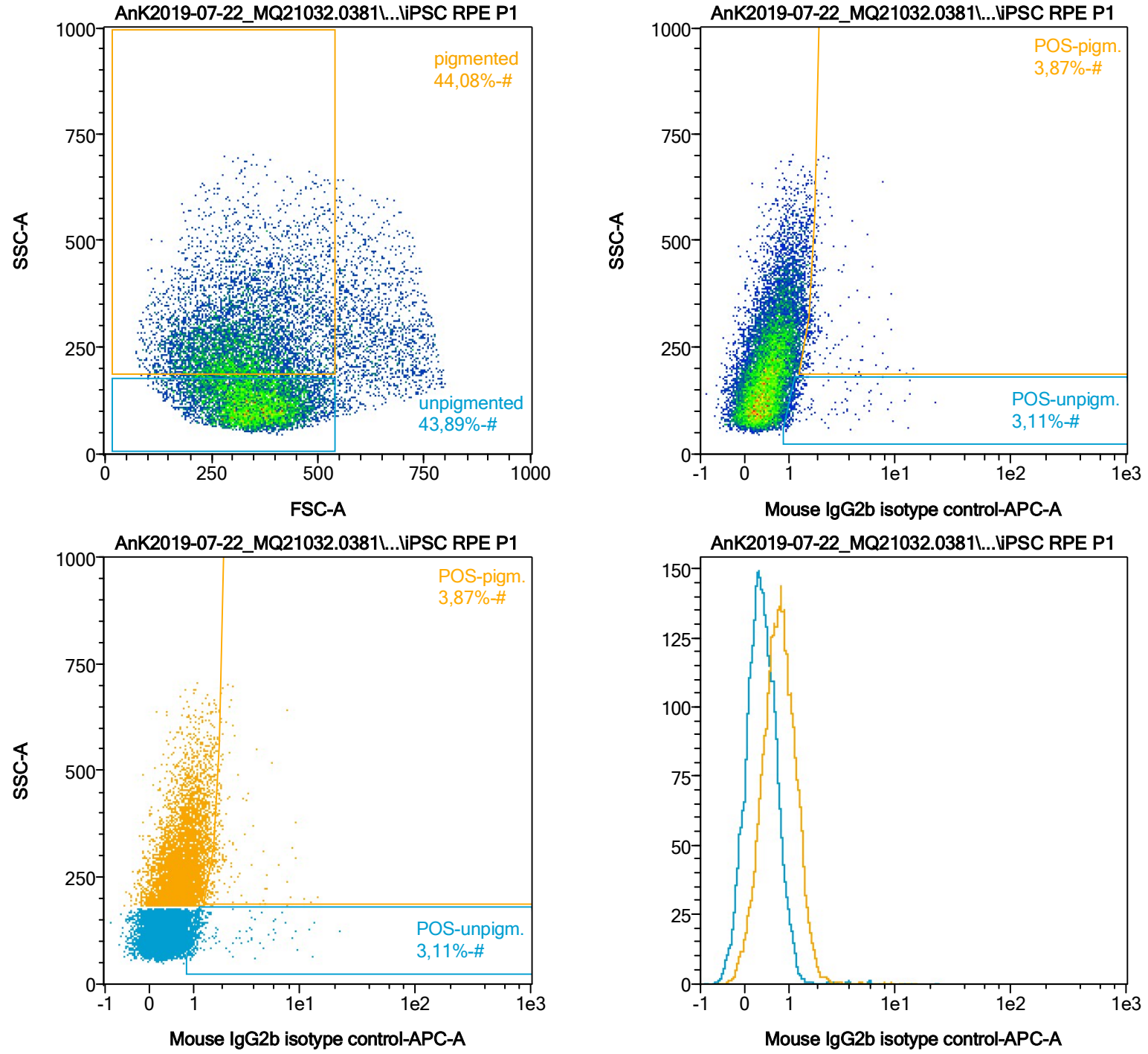

Name	%-#	Mouse IgG2b isotype control-APC-A Mean	Mouse IgG2b isotype control-APC-A Median
POS-unpigm.	3,11	2,02	1,17
POS-pigm.	3,87	2,33	1,81

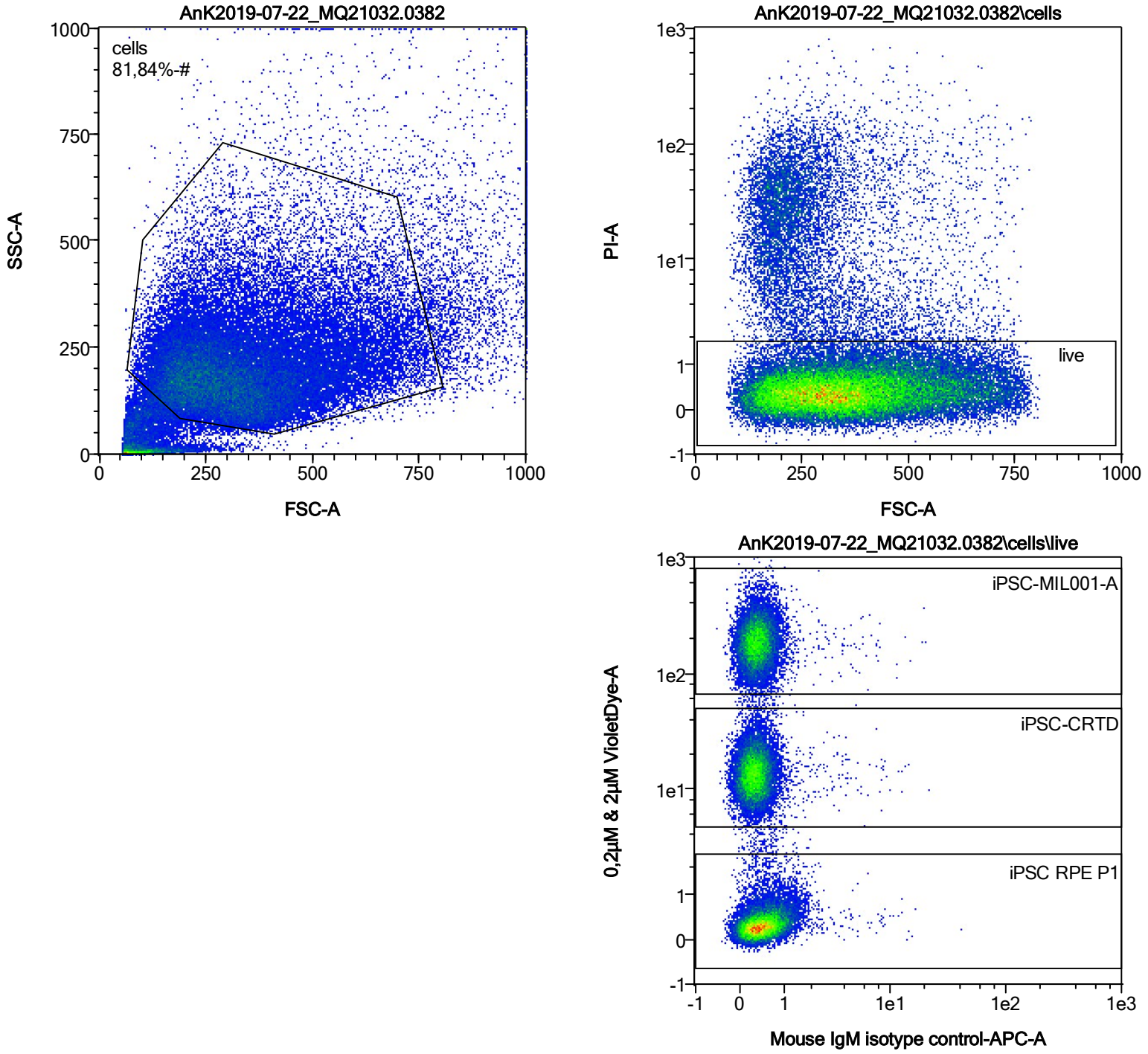

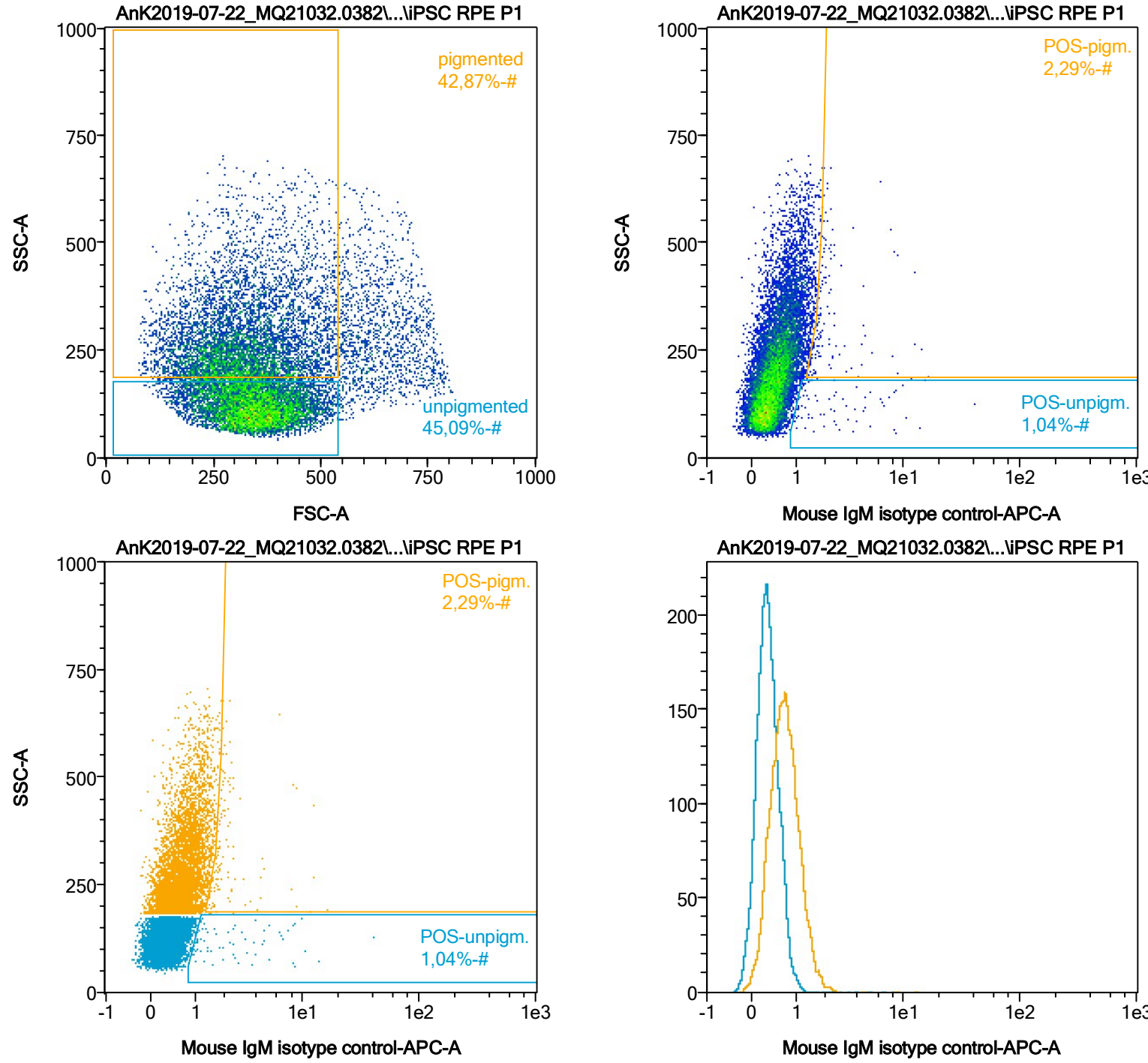

Name	%-#	Mouse IgM isotype control-APC-A Mean	Mouse IgM isotype control-APC-A Median
POS-unpigm.	1,04	3,67	1,94
POS-pigm.	2,29	2,48	1,81

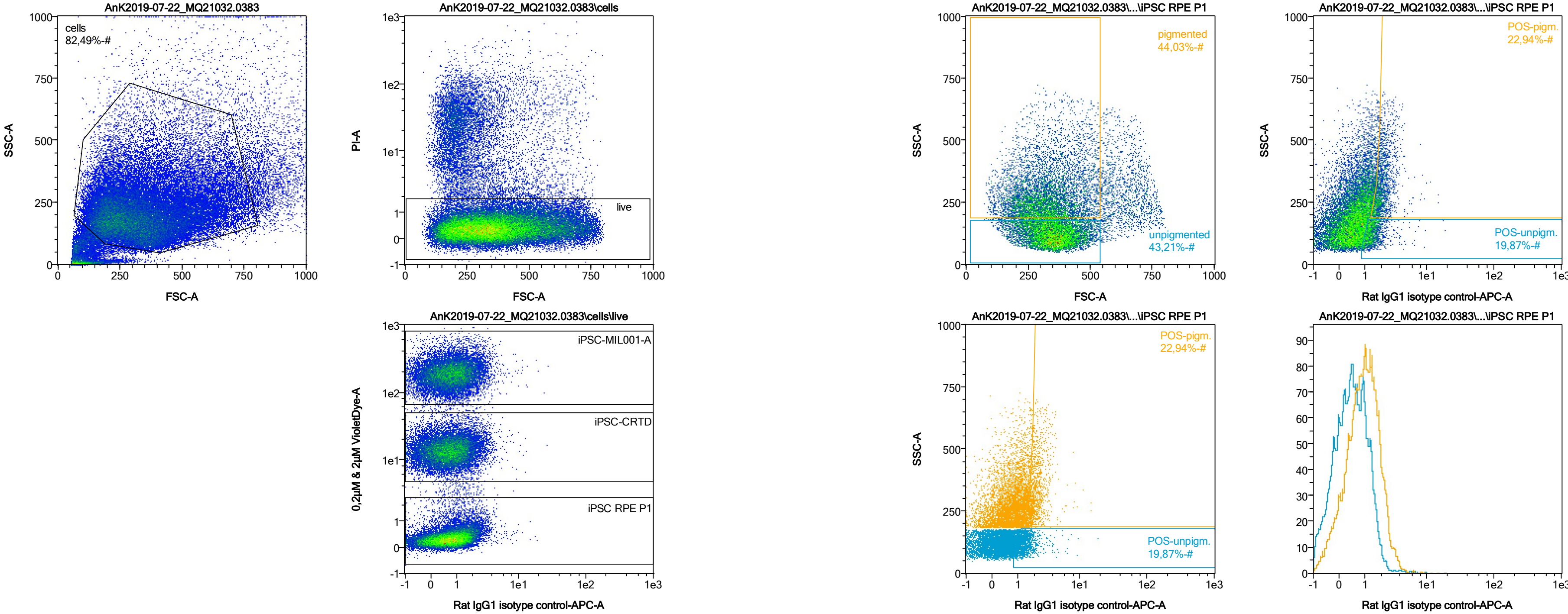

Name	%-#	Rat IgG1 isotype control-APC-A Mean	Rat IgG1 isotype control-APC-A Median
POS-unpigm.	19,87	1,49	1,33
POS-pigm.	22,94	2,15	1,95

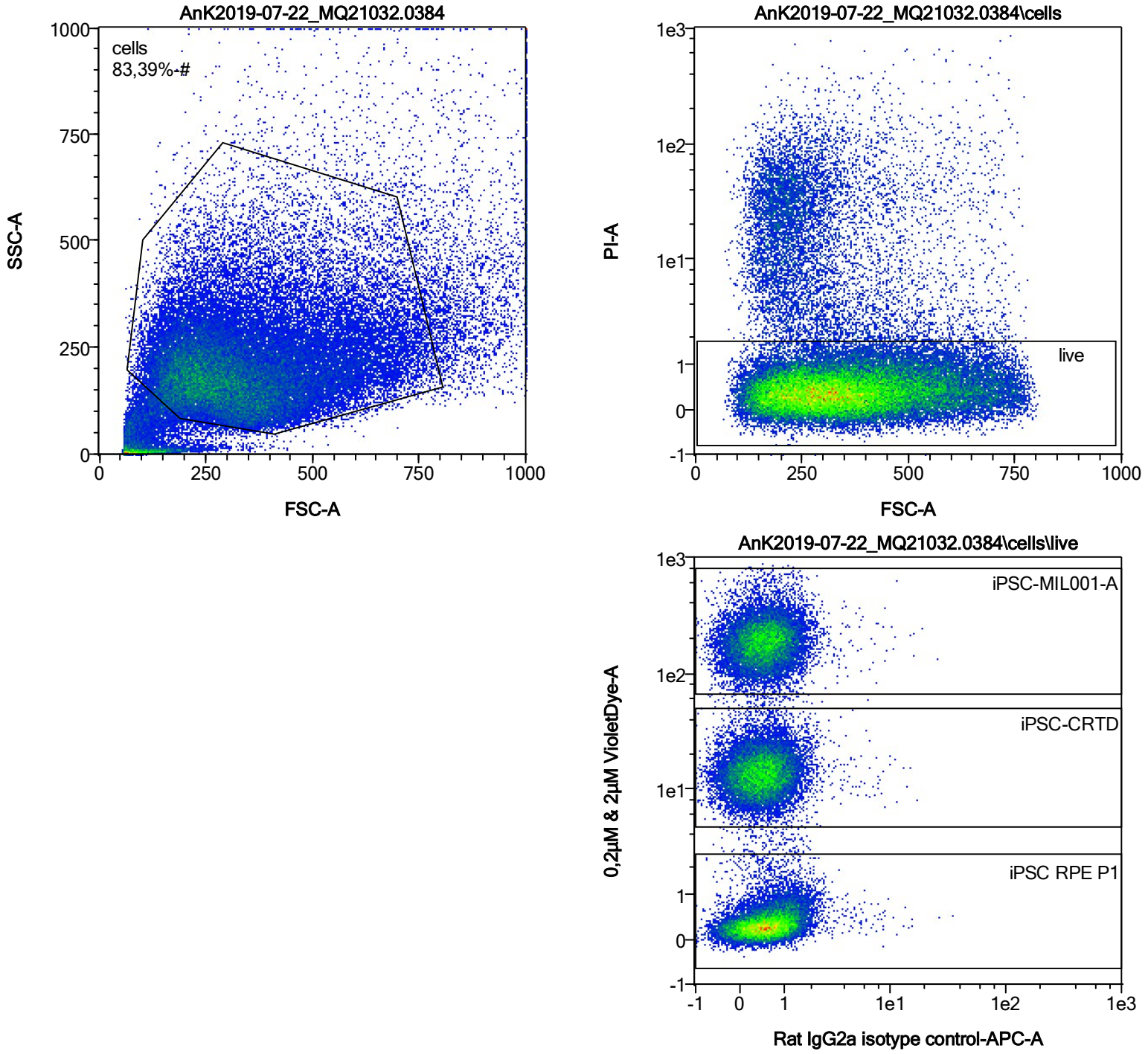

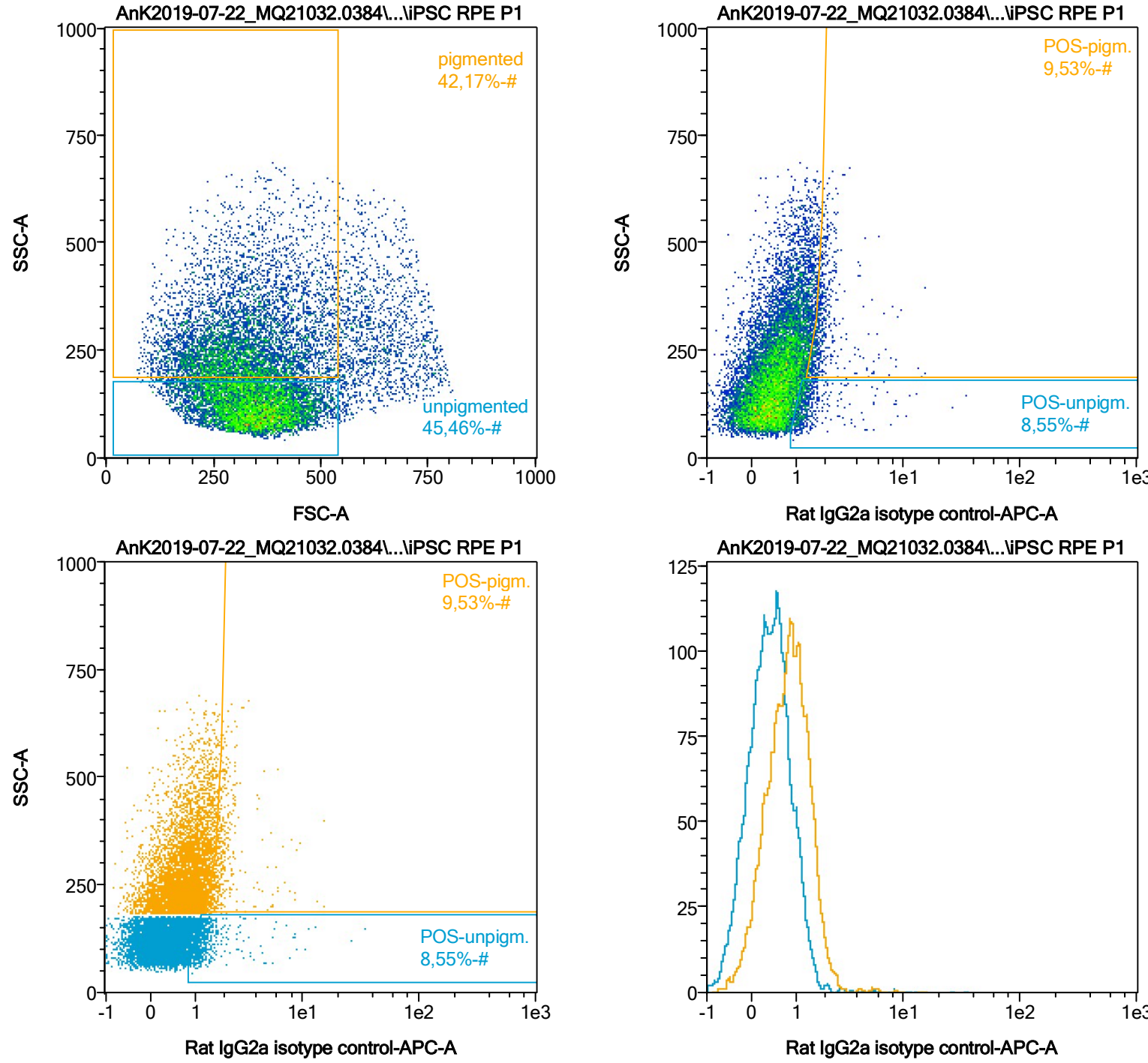

Name	%-#	Rat IgG2a isotype control-APC-A Mean	Rat IgG2a isotype control-APC-A Median
POS-unpigm.	8,55	1,60	1,21
POS-pigm.	9,53	2,11	1,80

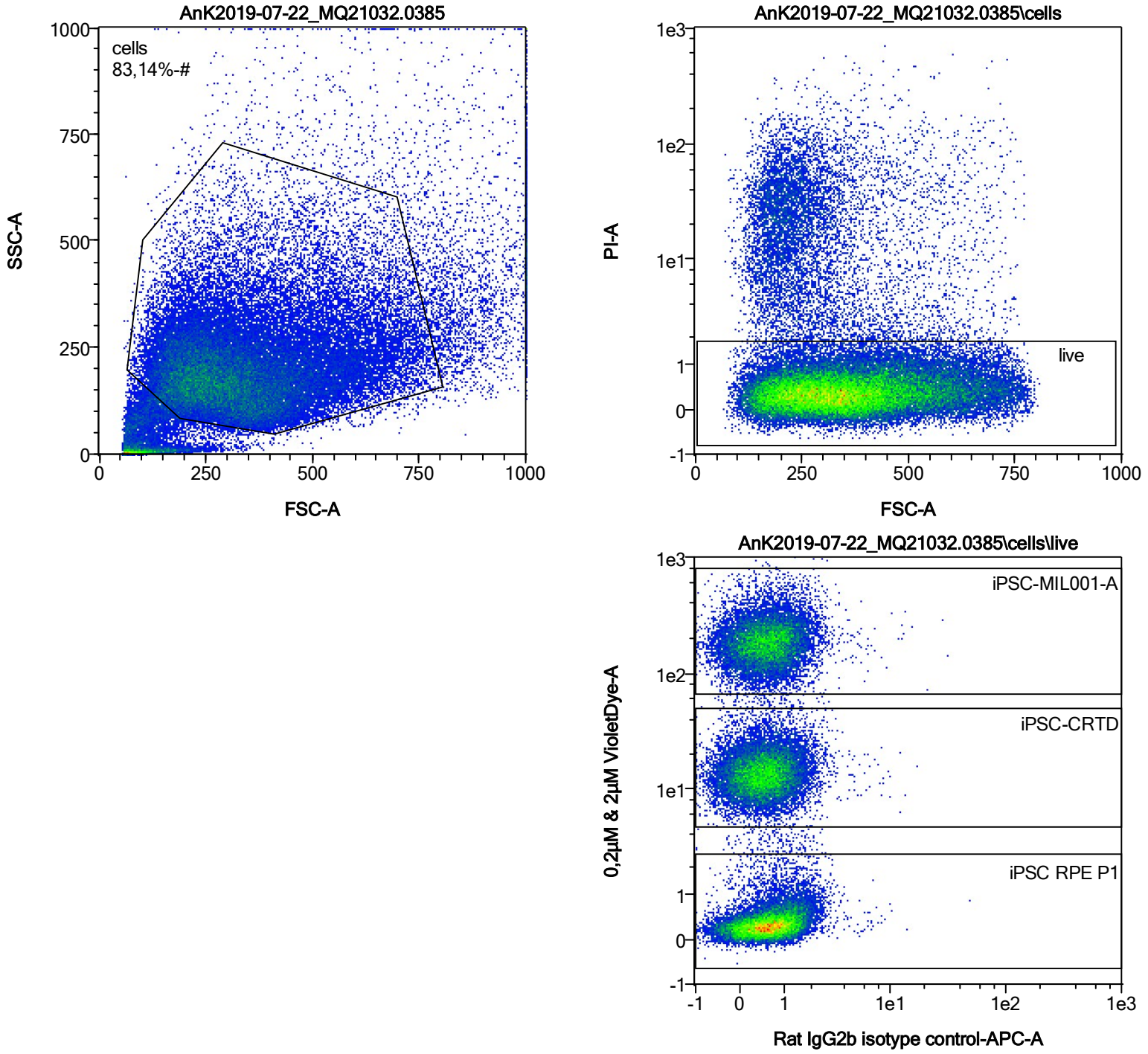

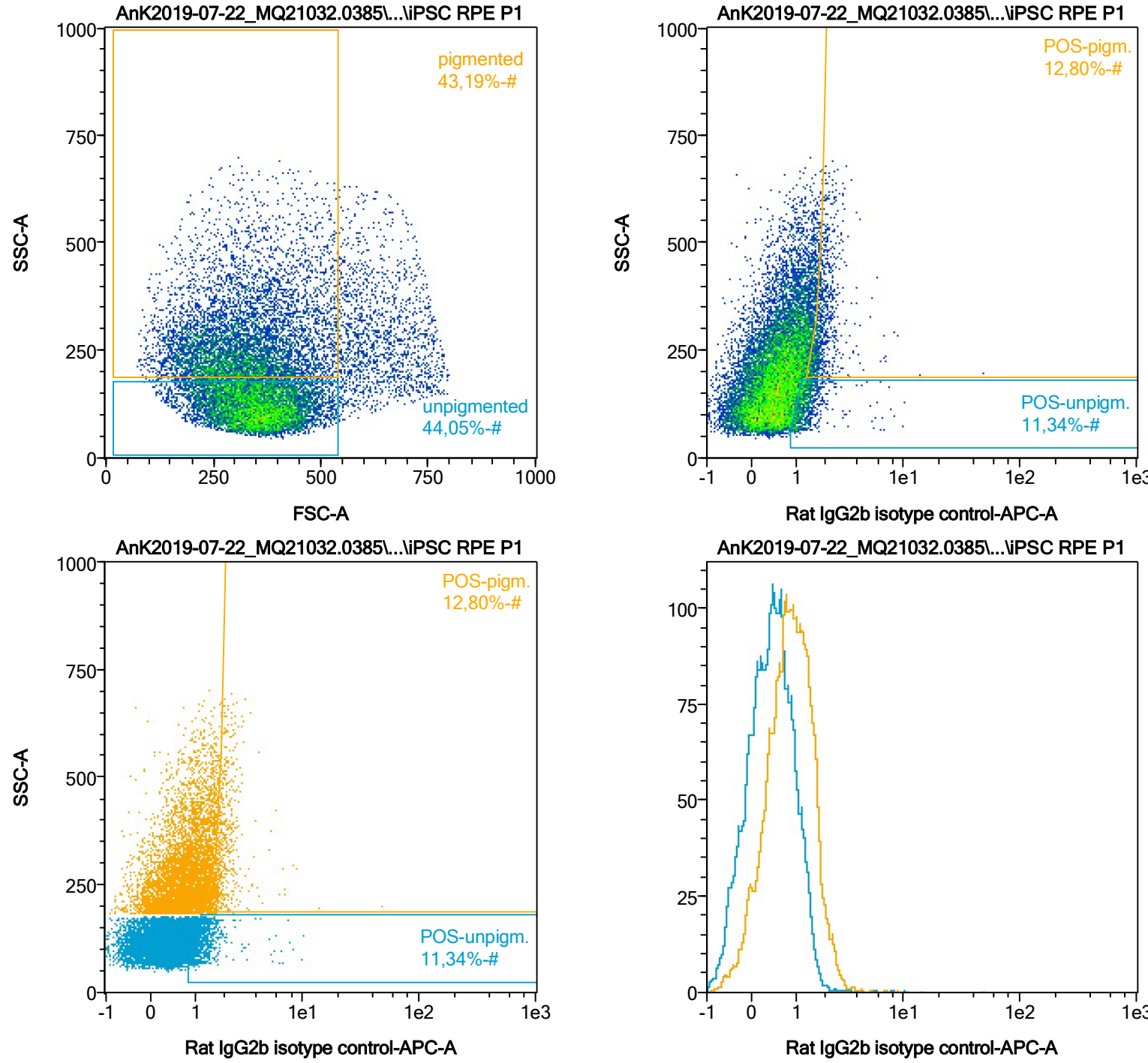

Name	%-#	Rat IgG2b isotype control-APC-A Mean	Rat IgG2b isotype control-APC-A Median
POS-unpigm.	11,34	1,39	1,22
POS-pigm.	12,80	2,03	1,81

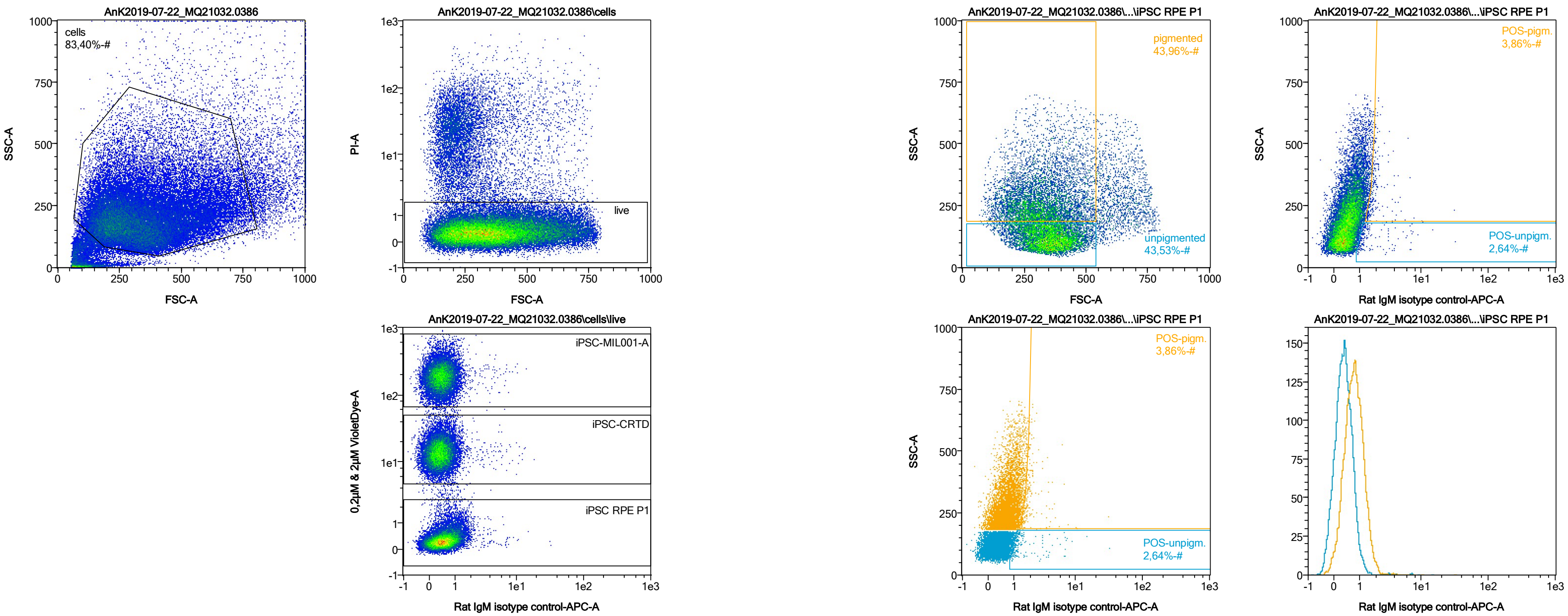

Name	%-#	Rat IgM isotype control-APC-A Mean	Rat IgM isotype control-APC-A Median
POS-unpigm.	2,64	2,04	1,19
POS-pigm.	3,86	2,21	1,79

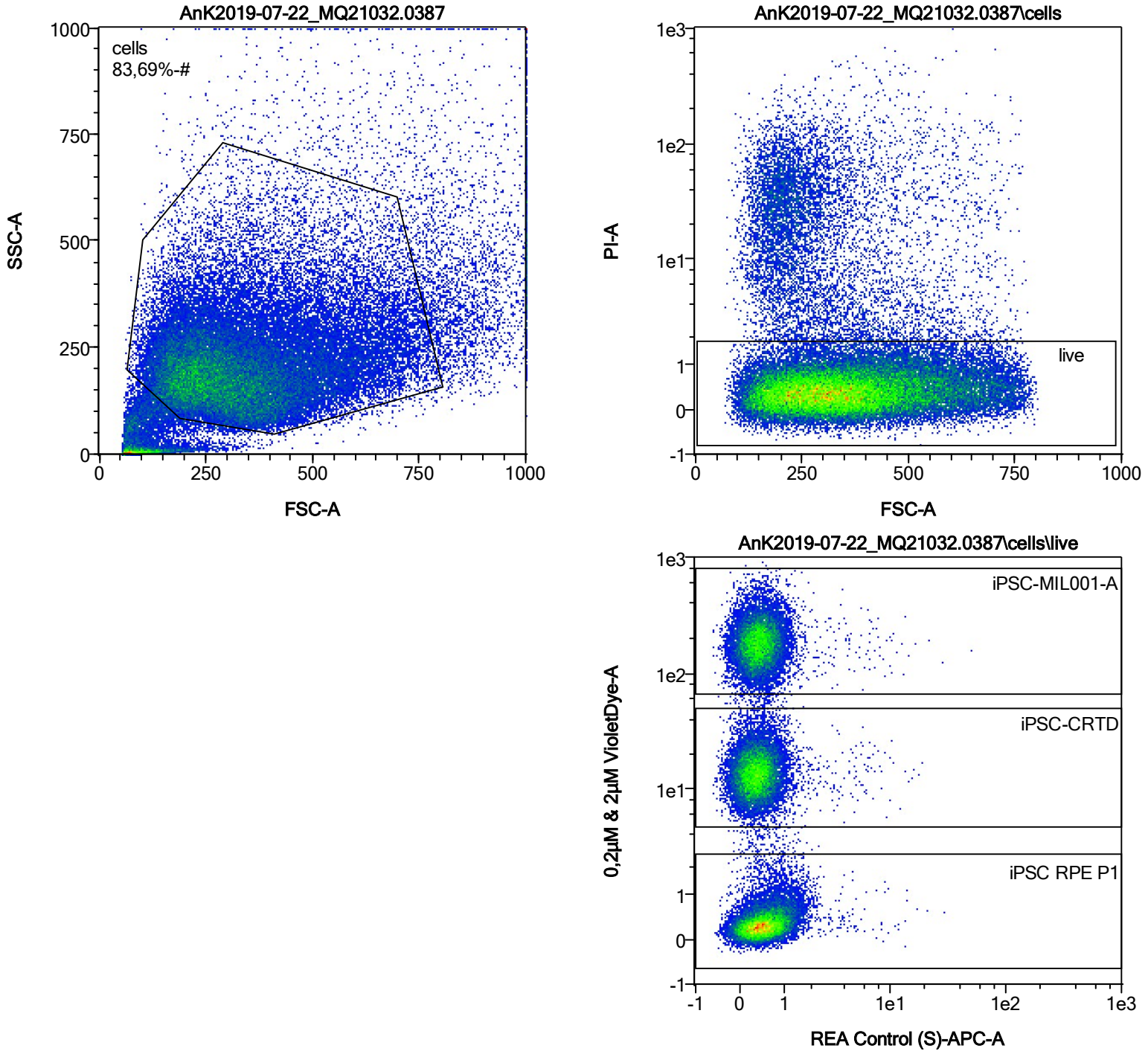

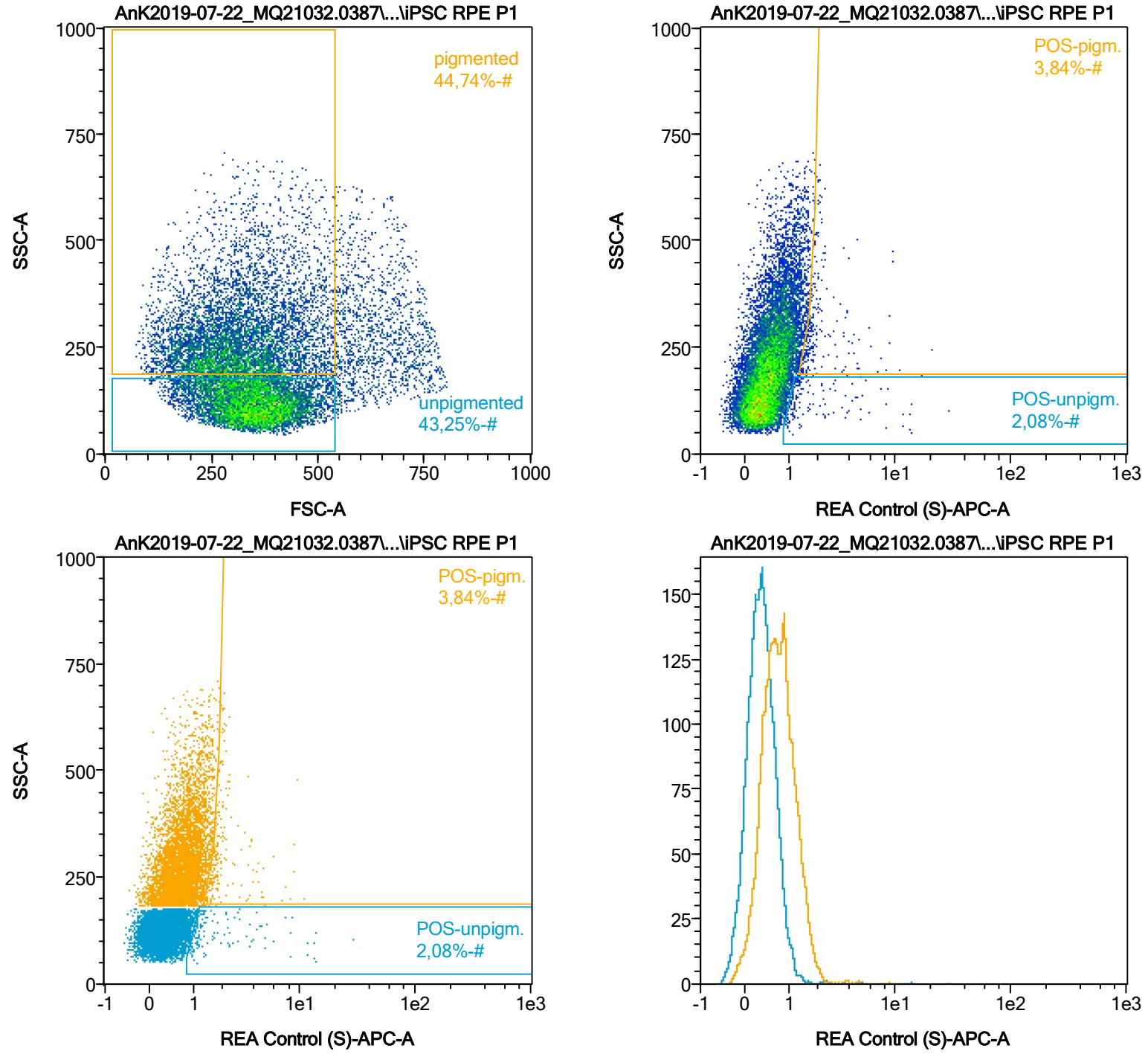

Name	%-#	REA Control (S)-APC-A Mean	REA Control (S)-APC-A Median
POS-unpigm.	2,08	2,65	1,29
POS-pigm.	3,84	2,19	1,79
